# Diversified local CRISPR-Cas immunity to viruses of *Sulfolobus islandicus*

**DOI:** 10.1098/rstb.2018.0093

**Published:** 2019-03-25

**Authors:** Matthew D. Pauly, Maria A. Bautista, Jesse A. Black, Rachel J. Whitaker

**Affiliations:** 1 Department of Microbiology, University of Illinois at Urbana-Champaign, 601 South Goodwin Avenue, Urbana, IL 61801, USA; 2 Carl R. Woese Institute for Genomic Biology, University of Illinois at Urbana-Champaign, 1206 West Gregory Drive, Urbana, IL 61801, USA

**Keywords:** CRISPR immunity, population diversity, microbial evolution, virus–host interactions

## Abstract

The population diversity and structure of CRISPR-Cas immunity provides key insights into virus–host interactions. Here, we examined two geographically and genetically distinct natural populations of the thermophilic crenarchaeon *Sulfolobus islandicus* and their interactions with *Sulfolobus* spindle-shaped viruses (SSVs) and *S. islandicus* rod-shaped viruses (SIRVs). We found that both virus families can be targeted with high population distributed immunity, whereby most immune strains target a virus using unique unshared CRISPR spacers. In Kamchatka, Russia, we observed high immunity to chronic SSVs that increases over time. In this context, we found that some SSVs had shortened genomes lacking genes that are highly targeted by the *S. islandicus* population, indicating a potential mechanism of immune evasion. By contrast, in Yellowstone National Park, we found high inter- and intra-strain immune diversity targeting lytic SIRVs and low immunity to chronic SSVs. In this population, we observed evidence of SIRVs evolving immunity through mutations concentrated in the first five bases of protospacers. These results indicate that diversity and structure of antiviral CRISPR-Cas immunity for a single microbial species can differ by both the population and virus type, and suggest that different virus families use different mechanisms to evade CRISPR-Cas immunity.

This article is part of a discussion meeting issue ‘The ecology and evolution of prokaryotic CRISPR-Cas adaptive immune systems’.

## 1. Introduction

Infection by viruses is a common feature among the three domains of life. Genetic diversity within genes associated with antiviral immunity is often high compared with the rest of an organism's genome and is maintained by diversifying selection [1,2]. Maintenance of diversity at immunity-associated genes may result from negative frequency-dependent selection or arms race dynamics [3–5]. The structure of this diversity, which describes how immunity is similar or different among members of a population, can be used to infer the nature of interactions between virus and host populations at a given point in time and ultimately to predict viral epidemics within microbial populations. In microbial populations, the most common dynamic described is a Lotka–Volterra model in which recurring selective sweeps of resistant types are fixed to create low diversity at any single time point within a population but high diversity among populations through time [6]. Some single-celled microbes exhibit high genetic diversity in their CRISPR-Cas systems both within and among populations [7–9].

Clustered regularly interspaced short palindromic repeats (CRISPRs) are genetic loci that function with CRISPR-associated system (*Cas*) genes to provide bacteria and archaea with adaptive immunity against viruses and foreign nucleic acids [10]. In these systems, short genome-encoded sequences called spacers match viral sequences called protospacers to confer immunity. Spacers are generally incorporated unidirectionally at the leader end of CRISPR spacer arrays [11–13]. The entire CRISPR spacer array is expressed and processed into individual spacer containing CRISPR RNAs that associate with *Cas* genes, which they direct to complementary protospacers [14,15]. The effector proteins of type I CRISPR-Cas systems cleave viral DNA containing protospacers with protospacer adjacent motifs (PAMs) [16,17]. Most type III CRISPR-Cas systems target and degrade RNA or actively transcribed DNA containing protospacers without a PAM requirement [18,19]. Viruses can evade CRISPR-Cas-mediated immunity by mutating their targeted protospacer or PAM sequences, mutating the promotor of genes targeted by type III CRISPR systems, or by encoding anti-CRISPR proteins [20–23]. CRISPR spacers are heritable and maintain a chronology of ancestral interactions with viruses. Characterizing the diversity and structure of CRISPR-Cas immune spacers at the population level is important for understanding microbial evolution in the context of virus–host interactions.

The population structure and dynamics of CRISPR-Cas immunity have been studied using laboratory evolved, artificially constructed or simulated microbial populations [22,24–30]. These studies show that host populations diversify their CRISPR spacers in response to virus challenge. This immune diversity, characterized by many evenly distributed CRISPR spacer genotypes leading to the same immunity phenotype, is called population distributed immunity (PDI) [26]. Simulations suggest that certain conditions (low virus mutation rate, high rate of spacer addition, large numbers of spacers and protospacers) promote high PDI, which, in turn, maintains stability and diversity in microbial populations by limiting genetic sweeps of immune host genotypes [26]. Like epidemiology in human populations, the number of susceptible individuals in a population is a key factor to determining whether a virus can establish and spread as an epidemic in a single population. High PDI limits the ability of a virus to adapt and evade immunity, keeping viral titres low and prone to extinction [26,27,29,31,32]. Studies of CRISPR-Cas immunity using natural populations show substantial diversity in CRISPR-Cas immunity, but their application to population structure is limited by the use of small numbers of individual virus and host isolates, incomplete identification of all spacer sequences or difficulty linking spacers from metagenomic sequences to individual cells [8,33–38].

To expand our understanding of the population structure of CRISPR diversity and its impact on virus–host interactions, we present findings from our study of *Sulfolobus islandicus* populations isolated from hot springs in Yellowstone National Park in the United States and near Mutnovsky volcano in Kamchatka, Russia. We examined their interactions with contemporary chronic, non-lytic *Sulfolobus* spindle-shaped viruses (SSVs) from the *Fuselloviridae* family and lytic *S. islandicus* rod-shaped viruses (SIRVs) from the *Rudiviridae* family. We suggest that differences in immunity structure and virus escape from immunity reveal distinct virus–host interactions and coevolution in each local population.

## 2. Material and methods

### (a) Cell and virus isolation and sequencing

The isolation of *S. islandicus* strains from hot springs near Mutnovsky volcano in Kamchatka, Russia, in 2000 (21 strains used in this study) and 2010 (29 strains used in this study) were described previously [8,25,39,40]. Water and sediment were collected from hot springs near Nymph Lake in Yellowstone National Park, USA, in 2012. Individual *S. islandicus* strains (clones) were isolated by sequential colony purifications [8,25,40]. Thirteen of the strains from Mutnovsky were previously sequenced with complete genome assemblies [39,40]. The DNA of the remaining eight Mutnovsky strains from 2000, the 29 Mutnovsky strains from 2010 and the 40 Yellowstone strains from 2012 was purified using either DNeasy Blood and Tissue kit (Qiagen, Hilden, Germany) or phenol, chloroform and isoamyl alcohol precipitation of cell lysates [41,42]. Genomic libraries were prepared using the Nextera XT library preparation kit (Illumina, San Diego, CA, USA) and sequenced at the W.M. Keck Center for Comparative and Functional Genomics at the University of Illinois at Urbana-Champaign. Strains from Yellowstone were sequenced on an Illumina HiSeq 2000 with 2 × 100 bp paired-end reads. Strains from Mutnovsky were sequenced on an Illumina MiSeq with 2 × 250 bp paired-end reads. Downstream analysis did not appear biased by the different sequencing methods as both were sufficient for spacer identification and assembly of integrated viruses and CRISPR loci (see below).

Isolation and sequencing of cell-free SSVs from Mutnovsky volcano in 2010 and Yellowstone National Park in 2012 was done as previously described [43]. Briefly, filtered environmental samples or enrichment culture supernatants were spotted on *S. islandicus* overlays. Samples that produced zones of clearance were selected for further purification. Individual plaques were picked and used to inoculate mid-log cultures of *S. islandicus* growing in dextrin-tryptone media [44] followed by two more rounds of plaque purification and screening by transmission electron microscopy for the presence of a single virus morphotype. The genomes of SSVs were isolated from concentrated samples using phenol/chloroform extractions as described previously [43,45]. The resulting nucleic acids were desalted using QIAEX II beads (Qiagen), prepared for sequencing using the Nextera XT kit (Illumina) and sequenced using an Illumina MiSeq with 2 × 250 bp paired-end reads. Reads were quality filtered using the FASTX-Toolkit and Cutadapt [46]. Viral genomes were assembled using Geneious v. 9.1.2 [47].

### (b) Identification of spindle-shaped viruses integrated into *S. islandicus* genomes

Quality filtered reads from each Mutnovsky and Yellowstone strain were used as queries in BLASTn searches against all known SSV genomes [48–52]. Reads similar to SSV sequences were pooled along with their paired-end mates, assembled into contigs using SPAdes genome assembler, and manually verified [53]. The ends of the viral genomes were determined by the locations of two identical *attP*-like sequences [54]. Viral integration sites were determined by the cellular sequences adjacent to these *attP*-like sequences.

### (c) Comparison of spindle-shaped viruses

All putative open reading frames (ORFs) from all SSVs were determined using glimmer3.02, allowing for alternative start codons, ORFs overlapping up to 250 bp, and ignoring the in-frame score for ORFs longer than 200 bp [55]. Putative ORFs were translated and homologous protein sequences were determined using CD-HIT with a sequence identity cut-off of 0.4 [56,57]. Ten ORFs (homologues of SSV1 VP1, VP3, C166, B251, A154, B277, A82, B115, B129 and C84/A92) identified in all 47 SSV genomes were aligned using MAFFT v. 7, concatenated into a single nucleotide sequence retaining alignment positions, and used to generate a phylogenic tree with RAxML using options -f a –x 100 -p 100 -N autoMR -m GTRGAMMA [58–60].

### (d) Identification of CRISPR spacers and *Cas* genes in *S. islandicus* genomes

The CRISPR spacer sequences from all *S. islandicus* strains were identified from quality filtered sequencing reads by extracting the sequences between CRISPR repeats (Mutnovsky A1 and A2 repeat = GATAATCTACTATAGAATTGAAAG; Mutnovsky C repeat = GATTAATCCTAAAAGGAATTGAAAG; Yellowstone A1 and A2 repeat = GCTAATCTACTATAGAATTGAAAG; or the previously unreported Yellowstone NL repeat = ATTTGTAGAAATCCTTAGAGGACTTGAAAC) [8]. The novel Yellowstone NL repeat sequence was identified from repetitive sequences within reads. Redundant spacers resulting from sequencing errors were removed by aligning spacers with high nucleotide identity and keeping only the consensus for each spacer from each strain. CRISPR loci were assembled by identifying reads containing CRISPR repeat sequences or *Cas* genes and assembling using SPAdes [53]. The presence of type I and type III CRISPR-Cas systems was determined using *Cas3* and *Cas10*/*Cmr2* from diverse *S. islandicus* strains (M.16.4, M.16.27, HVE10/4, REY15A, LAL 14/1, Y.G.57.14, Y.N.15.51 and L.S.2.15) as queries for tBLASTn analysis against reads and partly assembled CRISPR loci for each sequenced strain [39]. The location of spacers within Yellowstone CRISPR loci relative to the leader sequence was determined by sequentially matching spacers to the regions between repeats in assembled CRISPR loci.

### (e) Identification of protospacers in viral genomes

All CRISPR spacers were used with BLASTn and CLdb (https://github.com/nick-youngblut/CLdb) [43] to identify putative protospacer sequences in all Mutnovsky and Yellowstone SSVs and SIRVs along with the genetic locations, protospacer flanking sequences, PAM sequences and the locations of spacer:protospacer mismatches. Scripts written in R were used to perform subsequent analyses. Except where noted, our analyses included spacers with up to four mismatches to viral protospacers. All spacers containing a CC PAM dinucleotide in the -3 and -2 positions of the protospacer (same strand as crRNA) were classified as providing immunity [16,61]. Except where noted, spacers targeting protospacers lacking PAM sequences were only considered to provide immunity if the host strain had a type III CRISPR-Cas system. The relative locations of spacers within CRISPR loci were determined by their spacer order relative to the CRISPR leader sequence divided by the total number of spacers in the loci. The locations of all mismatches between spacers and protospacers containing PAMs were oriented by the spacer sequence in the 5′ to 3′ direction. Mismatches from all unique spacer:protospacer pairs were combined and binned by 5 bp windows. The expected distribution of mismatches, assuming they were located randomly across the protospacer, was determined by simulating mutations in a 40 bp nucleotide sequence (average spacer size). The number of mutations simulated was equal to the number of mismatches observed in the unique spacer:protospacers matches from the dataset.

### (f) Immunity metrics

Four complementary metrics were used to describe a population's immunity structure. Population immunity (PI), which describes how immune a population is, was calculated by dividing the number of immune strains by the total number of strains in a population. The inverse of PI describes how susceptible a population is to a virus. Distributed immunity (DI), which describes the diversity and distribution of virus-targeting spacers, was calculated by comparing the immunity providing spacers for every pair of immune strains in a population. Every unique pairwise comparison of immune strains with non-identical spacers targeting a virus added 1. Pairwise comparisons of immune strains targeting a virus with identical spacers added 0. The sum of these comparisons was divided by the total number of unique pairwise comparisons between immune strains to obtain DI. Individual distributed immunity (IDI), which describes how many virus-targeting spacers each strain has, was calculated as the total number of spacers in a population targeting a virus divided by the number of strains in the population. PDI, which compares how all strains in a population target a virus, was calculated according to Childs *et al*. [26]. The equation is

$$\mathrm{PDI}=\;\underset{i}{\sum }\;\underset{j}{\sum }\;\underset{k}{\sum }\;\left(1-\frac{\left|N_{i}-N_{j}\right|}{\max\;(N)}\right)\;\sigma_{ijk}\;N_{i\;}N_{j}\;V_{k}$$

and

$$\sigma_{ijk}=\left\{ \begin{array}{l} \begin{array}{ll} 1, & \mathrm{if}\;R(G_{i},H_{k})\;R(G_{j},H_{k})>R^{2}(G_{i},G_{j},H_{k})\\ 0 & \mathrm{otherwise}\; \end{array}, \end{array}\right.$$

where *N_i_* and *V_k_* are the population proportions of the *i*th strain and *k*th virus, *G_i_* is the spacers encoded by the *i*th strain, *H_k_* is the protospacers in the genome of the *k*th virus and *R*(*G,H*) determined the number of overlapping spacers and protospacers between *G* and *H*. This equation is simplified to

$$\mathrm{PDI}=\;\underset{i}{\sum }\;\underset{j}{\sum }\sigma_{ijk}\;N_{i\;}N_{j}$$

because we looked at each unique virus independently (*V_k_* = 1) and *N* is equivalent for each strain in the population (1/number of strains). Only comparisons in which both strains are immune to the virus through a distinct set of spacers add to the result, which ranges from 0 to 1 – (1/number of strains).

### (g) Statistical analyses

Statistical analysis of data was performed using either Graphpad Prism for Windows (v. 7.02) or R (v. 3.3.2).

## 3. Results

### (a) *S. islandicus* populations have high CRISPR spacer diversity

To investigate the spatio-temporal structure and dynamics of antiviral CRISPR-Cas immunity in nature, we used two geographically separated populations of the thermophilic crenarchaeon, *S. islandicus*. We sequenced individual strains (colonies) isolated from hot springs near Mutnovsky volcano in Russia in 2000 (21 strains) and in 2010 (29 strains), and near Nymph Lake in Yellowstone National Park in 2012 (40 strains) [25,39,42,62,63]. Many of the strains from Mutnovsky have been described previously, including 13 with complete genome assemblies [8,25,39,40]. All Yellowstone strains are previously unreported. For each of these strains, we identified the sequences of all the CRISPR spacers from sequencing reads containing *Sulfolobus* repeat sequences.

*S. islandicus* can encode both type I-A DNA-targeting CRISPR-Cas systems (characterized by Cas3) and type III-B systems (characterized by Cas10 (Cmr2)) which target transcribed DNA and RNA [10,64]. All strains encode a *Cas3* gene and were classified as possessing type I systems. Type III-B CRISPR were identified by the presence of a *Cas10*/*Cmr2* gene in 45% of Yellowstone strains and 66% of Mutnovsky strains (electronic supplementary material, tables S1 and S2). All strains possess spacers with a minimum of 31 spacers found one of the Yellowstone strains (NL03.C02.08).

**Figure 1.**
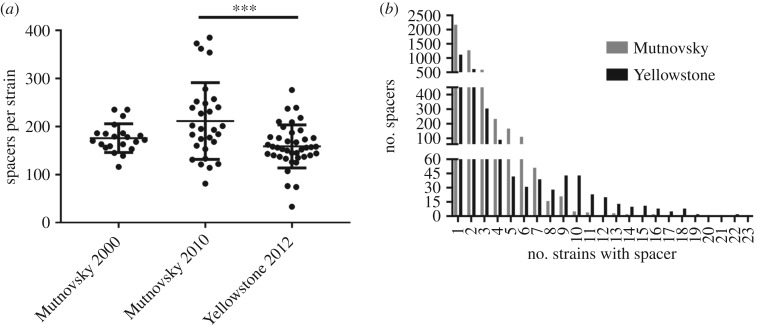
*S. islandicus* strains have diverse spacer repertoires. (*a*) The total number of spacers encoded by each strain from each *S. islandicus* population. The average and standard deviation are shown. Populations were compared using a one-way ANOVA with Tukey's multiple comparison test (***p < 0.001). (*b*) The number of strains from the combined Mutnovsky population (2000 and 2010) (grey) or the Yellowstone population (black) that have each unique CRISPR spacer.

The Mutnovsky population (combined time points) had 9832 total spacers with 4659 unique sequences. The Yellowstone population had 6348 total spacers with 2455 unique sequences. All strains isolated from Mutnovsky and Yellowstone possess both A1 and A2 CRISPR arrays [8]. Additionally, 58–72% of strains from each population possess a third CRISPR array (C or NL repeat sequence, electronic supplementary material, tables S1 and S2) [8]. The presence of this array is not correlated with the presence or absence of type III systems. We found that Mutnovsky strains from 2000 and 2010 have similar numbers of CRISPR spacers, while Yellowstone strains have, on average, fewer CRISPR spacers than strains from the 2010 Mutnovsky population (figure 1*a*). The average length of spacers in each population is 40 bases.

*S. islandicus* populations possess high diversity in their CRISPR spacers [8,25]. To quantify this diversity, we determined the number of strains that shared each unique spacer sequence. We found that most spacers are unique to only one or a few strains (figure 1*b*). We also found no overlap between spacers identified from Mutnovsky and Yellowstone, suggesting that all spacers were acquired in response to geographically specific nucleic acids [43,50,65].

### (b) Local virus diversity

Hot springs are home to diverse virus types, some of which are targeted by *S. islandicus* CRISPR immunity [50,66,67]. In this study, we first focused on SSVs because they are prominent and ubiquitous [49,50]. The SSVs have circular genomes around 15 kb in length that encode a highly diverse set of variable genes [48,49]. These viruses have non-lytic replication cycles and can be maintained for extended periods as an integrated virus or in an episomal form [68–70]. To determine the contemporary viruses of our *S. islandicus* populations, we identified SSVs integrated into host cell genomes. Each of the three populations had a similar percentage of strains carrying an integrated SSV (29% of 2000 Mutnovsky strains, 24% of 2010 Mutnovsky strains and 40% of 2012 Yellowstone strains), suggesting that each population interacts with this family of virus to a similar extent. We identified a range of SSV genome sizes and four unique tRNA integration sites within these populations (table 1). Three SSVs are integrated into two *S. islandicus* strains and strain M.06.0.8 has two unique viruses integrated into its genome (table 1). Many integrated viruses appear to be actively replicating, because sequencing reads indicating circularized, non-integrated viral genomes were observed for 3 of 11 integrated Mutnovsky SSVs and 12 of 16 integrated Yellowstone SSVs (table 1).

**Table 1.** Local *S. islandicus* viruses.

group name	virus type	location	year	genomes from	virus	accession	genome size (bp)	integration site	other strains containing
Mut. int. SSVs 2000	*Sulfolobus* spindle-shaped virus	Mutnovsky volcano area, Kamchatka, Russia	2000	*S. islandicus* genomes	M.12.04.SSV	MK054209	11 682	tRNA-Arg-GCG	M.14.34
					M.14.25.SSV [50]	MK054210	14 967	tRNA-Arg-TCG	M.14.17
					M.16.12.SSV	MK054211	11 323	tRNA-Arg-GCG	M.16.22
Mut. int. SSVs 2010	*Sulfolobus* spindle-shaped virus	Mutnovsky volcano area, Kamchatka, Russia	2010	*S. islandicus* genomes	M.03.0.27.SSV	MK054212	11 433	tRNA-Arg-GCG	
					M.03.0.42.SSV	MK054213	15 176	tRNA-Arg-GCG	
					M.03.2.5.SSV	MK054214	11 661	tRNA-Arg-GCG	
					M.04.0.13.SSV	MK054215	17 148	tRNA-Arg-GCG	
					M.04.0.29.SSV	MK054216	11 887	tRNA-Arg-GCG	
					M.04.0.37.SSV	MK054217	11 593	tRNA-Arg-GCG	
					M.06.0.8v1.SSV	MK054218	11 658	tRNA-Arg-GCG	
					M.06.0.8v2.SSV	MK054219	14 972	tRNA-Asp-GTC	
Mut. free SSVs 2010	*Sulfolobus* spindle-shaped virus	Mutnovsky volcano area, Kamchatka, Russia	2010	cell-free virus preps	SSV12	MK054204	17 195		
					SSV13	MK054205	16 184		
					SSV14	MK054206	18 208		
					SSV15	MK054207	15 088		
					SSV17	MK054208	15 529		
Yel. SSVs	*Sulfolobus* spindle-shaped virus	Nymph Lake area, Yellowstone National Park, USA	2012	*S. islandicus* genomes	NL01B.C01.01.SSV	MK054220	16 319	tRNA-Asp-GTC	
					NL01B.C01.03.SSV	MK054221	15 029	tRNA-Leu-GAG	
					NL01B.C01.05.SSV	MK054222	15 750	tRNA-Asp-GTC	
					NL01B.C01.06.SSV	MK054223	15 766	tRNA-Asp-GTC	
					NL01B.C01.07.SSV	MK054224	16 044	tRNA-Asp-GTC	
					NL01B.C01.09.SSV	MK054225	15 815	tRNA-Asp-GTC	
					NL01B.C01.13.SSV	MK054226	16 026	tRNA-Asp-GTC	
					NL01B.C01.14.SSV	MK054227	14 609	tRNA-Leu-GAG	
					NL01B.C01.18.SSV	MK054228	16 451	tRNA-Asp-GTC	
					NL01B.C01.20.SSV	MK054229	16 213	tRNA-Asp-GTC	
					NL01B.C01.22.SSV	MK054230	15 881	tRNA-Asp-GTC	
					NL01B.C01.24.SSV	MK054231	15 412	tRNA-Asp-GTC	
					NL03.C02.01.SSV	MK054232	15 575	tRNA-Asp-GTC	
					NL03.C02.05.SSV	MK054233	15 548	tRNA-Asp-GTC	
					NL13.C01.02.SSV	MK054234	15 911	tRNA-Asp-GTC	
					NL13.C01.04.SSV	MK054235	15 789	tRNA-Asp-GTC	
Yel. SIRVs	*S. islandicus* rod-shaped virus	Nymph Lake area, Yellowstone National Park, USA	2010	cell-free virus preps	SIRV4 [43]	NC_034 628	35 035		
					SIRV5 [43]	NC_034621	36 306		
					SIRV6 [43]	KY744235	35 439		
					SIRV7 [43]	NC_034619	34 190		
					SIRV11 [43]	NC_034624	33 356		

**Figure 2.**
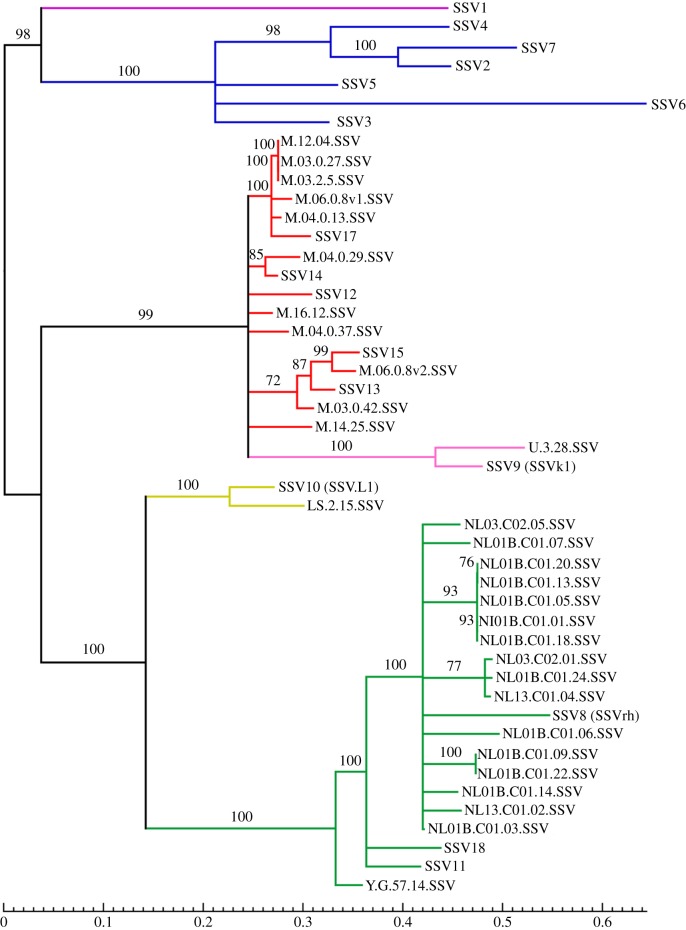
Phylogeny of *Sulfolobus* SSVs. Phylogenetic tree of all currently known SSVs. Tree is built with RAxML v. 8 using concatenated, aligned nucleotide sequences from 10 core genes shared by all SSVs. Numbers indicate bootstrap values for the upstream node. Nodes with bootstrap values below 70% are collapsed. Branches are coloured by the geographical location where each SSV was identified. Red, Mutnovsky volcano, Russia; pink, Uzon volcano, Russia; purple, Beppu, Japan; blue, Hveragerdi, Iceland; yellow, Lassen National Park, USA; green, Yellowstone National Park, USA. Integrated viruses begin with the name of an *S. islandicus* strain, while free viruses begin with SSV.

In addition to the integrated SSVs, we identified five freely circulating viruses from filtered hot-spring samples from Mutnovsky collected in 2010 (table 1). We constructed a phylogenetic tree using the nucleotide sequences of 10 core genes shared by all known SSVs (figure 2) [48–50]. All the integrated and free SSVs from Mutnovsky are monophyletic, but their relationships do not correspond to time point or whether they are free or integrated. Some closely related SSVs are integrated into closely related *S. islandicus* strains, suggesting either viral evolution occurring while integrated as a provirus or susceptible host strains being grouped phylogenetically (electronic supplementary material, figure S1). Additionally, we found that Yellowstone SSVs cluster together and are distant from the Mutnovsky strains, showing that the SSV populations are geographically distinct (figure 2).

### (c) CRISPR immunity differs with geographical location and time

*Sulfolobus* CRISPR-Cas systems have a functional tolerance for mismatches between spacers and protospacers [71]. To accommodate this tolerance for mismatches, while maintaining high confidence that a spacer provides effective immunity, we conservatively limited our analysis to spacers that target viral protospacers with four or fewer mismatches. Within this limit, all spacers targeting a PAM-containing protospacer were classified as providing immunity [61]. Spacers targeting protospacers lacking PAM sequences were only counted if the host strain possessed a type III CRISPR system [18,64]. From Mutnovsky, 92 spacers (2% of the unique spacer sequences) target SSVs and none target SIRVs. In the Yellowstone population, only seven spacers (0.3% of unique) target SSVs, while 211 spacers (8.6% of unique) target SIRVs. We characterized the immunity of populations to individual viruses using four metrics. PI is the proportion of strains in a population with at least one virus-targeting spacer and describes how immune the population is to a virus. DI is the proportion of immune strain pairs that target a virus using distinct spacers and describes how diverse the ways that the population targets a virus are. PDI accounts for both the PI and the DI of the population [26]. Low PDI values indicate that viruses can easily find susceptible hosts or evolve to find susceptible hosts within a population because of either low PI or low DI. IDI is the average number of virus-targeting spacers possessed by each strain in the population and describes whether strains target a virus using few or many spacers. Each metric describes a different aspect of a population's immunity structure, which individually would not adequately describe this structure. The use of these metrics improves with the depth of population sampling. Low sampling could bias the observable immunity and decrease the ability to resolve differences between populations.

**Figure 3.**
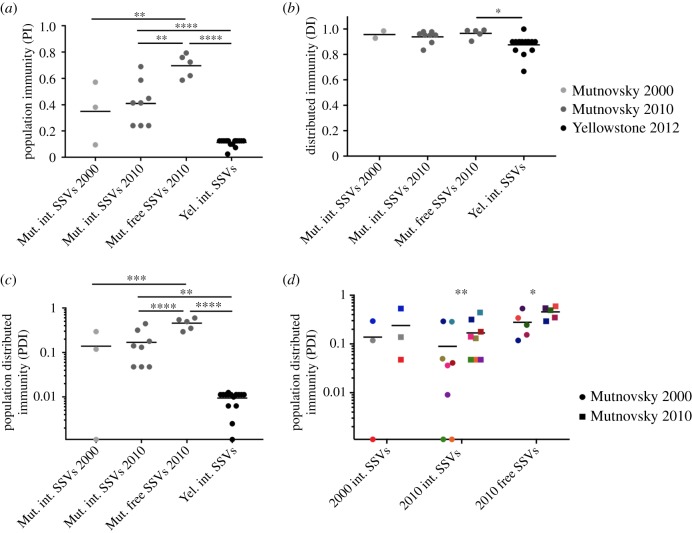
CRISPR immunity differs by geographical location and with time. (*a*) The PI to contemporary viruses from the same geographical location and time point (light grey, Mutnovsky 2000; dark grey, Mutnovsky 2010; black, Yellowstone 2012). Spacers with fewer than four mismatches to viral protospacers are considered to provide a strain with immunity if they target a PAM-containing protospacer or come from a strain with a type III CRISPR-Cas system. The average and values for individual viruses are shown. The DI (*b*) and PDI (*c*) for the same contemporary virus—*S. islandicus* interactions shown in (*a*). Values were compared among virus classes using a one-way ANOVA with Tukey's multiple comparison test. *p < 0.05, **p < 0.01, ***p < 0.001, ****p < 0.0001. The PDI (*d*) for the Mutnovsky *S. islandicus* populations from 2000 (filled circle) and 2010 (filled square) for the three groups of SSVs identified from Mutnovsky. Matching colours identify a single virus targeted by the two population time points. The immunity to each group of viruses by the two population time points was compared using a paired *t*-test. (Online version in colour.)

We first tested how each population targets contemporary SSVs, because both Yellowstone and Mutnovsky populations interact with this virus type. We define contemporary to mean from the same hot spring and time point. We found very low PI to contemporary SSVs in the Yellowstone population (figure 3*a*). By contrast, the Mutnovsky population had high PI to contemporary SSVs, especially freely circulating viruses (figure 3*a*), suggesting that this population has high antiviral immunity to SSVs. Most SSV-specific spacers target multiple viruses (cross-immunity) (electronic supplementary material, figure S2A) and are not widely shared among *S. islandicus* strains (electronic supplementary material, figure S2B). A small proportion (5%) of SSV-specific Mutnovsky spacers cross-react with SSVs from Yellowstone. Only 2 of 50 Mutnovsky strains from the two time points are not immune to at least one contemporary SSV, suggesting that most strains from this population have encountered this virus type. There is still a large proportion of this population that is susceptible to each SSV (the inverse of the PI value) and individual strains are immune to on average 7 of the 16 SSVs (electronic supplementary material, table S1), suggesting that this population is unlikely to prevent virus spread or future -epidemics through herd immunity [30]. The Mutnovsky population is more susceptible to viruses we identified in an integrated state than to freely circulating viruses. We also note that three Mutnovsky strains (M.03.0.42, M.04.0.37 and M.06.0.8) have spacers that target their own integrated SSVs.

High PI can result from either selective sweeps of CRISPR spacers or the independent acquisition of unique CRISPR spacers by many strains. To differentiate between these possibilities and to determine the structure of CRISPR immunity, we assessed the diversity of spacers (DI). There was high DI to SSVs with most pairs of immune strains targeting a virus through different spacers (figure 3*b*). The PDI, which takes into account both PI and DI, was highest for freely circulating Mutnovsky SSVs and very low for Yellowstone SSVs (figure 3*c*). Populations with high PDI targeting a virus have a diversity of virus-targeting CRISPR spacers that are distributed among the strains that comprise the population (electronic supplementary material, figure S3). The higher this value, the more difficult it is for a virus to find a susceptible host to infect or evolve to evade CRIPSPR immunity within its local population.

We next investigated whether CRISPR-Cas immunity changes over time by comparing the targeting of SSVs from 2000 and 2010 by the Mutnovsky *S. islandicus* population at each time point. We found that the population in 2010 had a higher PDI to all SSVs from both time points than the population in 2000 (figure 3*d*). This finding, along with the genetic similarity of the viruses from each time point (electronic supplementary material, figure S1), indicates that the *S. islandicus* population in hot springs near Mutnovsky volcano evolved to better target their local virus population over 10 years.

### (d) Spindle-shaped viruses with short genomes are rarely targeted in accessory genes

**Figure 4.**
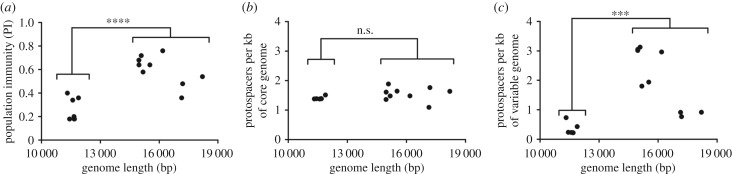
SSVs limit CRISPR targeting through shortened genomes. (*a*) The PI of the combined 2000 and 2010 Mutnovsky population targeting each Mutnovsky SSV plotted against the length of the SSV genome. The number of targeted protospacers located in the core genes (*b*) or variable genes (*c*) per kilobase of core or variable genome for each Mutnovsky SSV plotted against its genome length. The values for SSVs with short genomes (smaller than 12 000 bp) and SSVs with long genomes (larger than 14 500 bp) were compared using a two-tailed *t*-test. n.s., not significant, ***p < 0.001, ****p < 0.0001.

Among the SSVs found in the Mutnovsky population, there is a large range of genome lengths. We investigated how genome length affects how a virus is targeted by CRISPR spacers. We found that the PI is significantly higher for long genome SSVs (greater than 14 500 bp) than short genome SSVs (less than 12 000 bp) (figure 4*a*). Each of the SSVs from Mutnovsky contains a core set of 13 genes, which comprise 6800–7500 bp of the genome, with the remainder of the genomic content containing accessory or variable genes that are not shared by all viruses. Short genome SSVs encode only 9–10 variable genes, while long genome SSVs have 22–28 variable genes. SSVs of all sizes are equally targeted at protospacers in core genes (figure 4*b*), but only long genome SSVs are highly targeted in accessory genes (figure 4*c*). Even though variable genes comprise greater than one-third of the genome of short SSVs, they are rarely targeted. This finding may suggest that SSVs can evade CRISPR-Cas immunity and increase the size of the host population that is susceptible to infection by losing or rearranging accessory genes. In support of this hypothesis, passage of SSV9 (SSV Kamchatka-1) with *S. islandicus* strain M.16.04 evolved a viral variant that deleted bases 2823–9693 of its genome, which include the only targeted viral protospacer. While the parental virus (17 382 bp genome) forms no zones of clearance in a plaque assay on strain M.16.04, the variant virus (10 513 bp genome) does. All of the integrated SSVs from the Yellowstone population have long genomes, possibly indicating that the lower level of PI to these viruses provides less pressure to lose targeted protospacers.

### (e) *S. islandicus* rod-shaped viruses are highly targeted in Yellowstone

Having investigated population-level CRISPR-Cas immunity to the non-lytic SSVs, we next compared these results with a lytic family of viruses. The SIRVs have large linear genomes that can use a lytic replication cycle or establish a carrier (non-integrated) infection state that does not include integration into the host genome [72,73]. For this study, we used five previously reported SIRVs identified from the same Nymph Lake hot springs as our Yellowstone *S. islandicus* strains, but from 2 years earlier (table 1) [43]. We did not identify any SIRVs from filtered hot-spring samples collected from Mutnovsky in 2010 and no Mutnovsky strains have spacers targeting known SIRVs with four or fewer mismatches.

**Figure 5.**
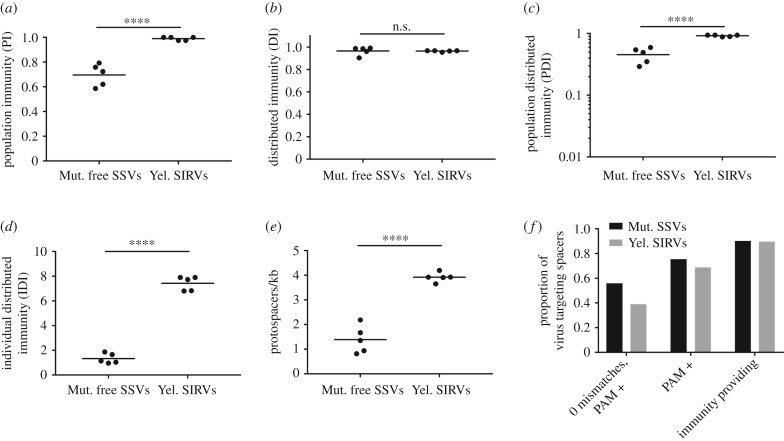
CRISPR immunity differs by virus type. The PI (*a*), DI (*b*) and PDI (*c*) for the 2010 Mutnovsky population targeting free 2010 Mutnovsky SSVs and the Yellowstone population targeting SIRVs identified from the same Nymph Lake hot springs. Spacers with fewer than four mismatches to viral protospacers are considered to provide a strain with immunity of they target a PAM-containing protospacer or come from a strain with a type III CRISPR-Cas system. (*d*) The IDI (number of spacers each strain uses to target virus) for the same population–virus interactions as above. (*e*) For each virus, the number of targeted protospacers per basepair of viral genome. For each measurement, the average and values for individual viruses are shown. The two groups were compared by a two-tailed *t*-test. n.s., no significance, ***p < 0.001, ****p < 0.0001. (*f*) The proportion of unique spacers targeting Mutnovsky SSVs or Yellowstone SIRVs with four or fewer mismatches that perfectly match PAM-containing protospacers, match PAM-containing protospacers, or are classified as providing immunity based upon our criteria for strains to possess type III CRISPR-Cas systems to target protospacers lacking PAMs.

We measured the CRISPR-Cas immune structure of the 2012 Yellowstone *S. islandicus* population targeting Yellowstone SIRVs from 2010 using the same CRISPR spacer targeting criteria as above for SSVs. We compared these results with those obtained from the 2010 Mutnovsky population that highly targets freely circulating contemporary SSVs, because the Yellowstone population has low immunity to its contemporary SSVs (figure 3). We found that the Yellowstone population, where nearly all strains possess spacers targeting each SIRV, has a higher PI to SIRVs than the Mutnovsky population has to SSVs (figure 5*a*). The DI was very high for local targeting of SIRVs in Yellowstone and SSVs in Mutnovsky (figure 5*b*), indicating that most immune strains target the virus through unique spacers. PDI was significantly higher for Yellowstone targeting of SIRVs than for Mutnovsky targeting of SSVs, probably driven by their differences in PI (figure 5*c*). As with Mutnovsky SSV-specific spacers, Yellowstone SIRV-specific spacers target multiple viruses (electronic supplementary material, figure S2*c*) and are generally shared with only a few other strains (electronic supplementary material, figure S2D). We also assessed immunity to three SIRVs [43] and two novel freely circulating SSVs (SSV11 and SSV18, accession numbers MK054237 and MK054236, SSV18 has been previously called SSV10 [74]) isolated from a distinct region of Yellowstone National Park (Norris Geyser Basin) (electronic supplementary material, figure S4). As with the local Nymph Lake viruses, we found high PDI to the SIRVs, but very low immunity to freely circulating SSVs from a different location.

PDI does not describe the number of virus-targeting spacers each strain has. To test whether viruses with different lifestyles are equally targeted on an individual strain level, we measured IDI, which is the average number of spacers that a strain in the population targets a virus with. We found that Yellowstone SIRVs are targeted by an average of seven spacers per strain, while Mutnovsky SSVs are targeted by an average of only one spacer per strain (figure 5*d*). The longer genomes of SIRVs do not account for this difference as SIRVs have significantly more targeted protospacers per kilobase of genome than SSVs (figure 5*e*). Protospacers targeted by individual strains are distributed across the genome without local clustering [75]. The majority of SSV- and SIRV-specific spacers target PAM-containing protospacers (figure 5*f*). Interestingly, we found that SIRVs had a much lower PAM density (31 PAMs per kilobase of genome) than either SSVs or the genomes of *S. islandicus* (74 and 64 PAM per kilobase, respectively). Together, these finding may suggest that low PAM density in SIRV genomes is selected for by high type I CRISPR-Cas targeting (figure 5*f*).

Requiring strains to have type III CRISPR-Cas systems to include spacers targeting protospacers without PAM sequences means that only 90% of the unique virus-specific spacers were included in our analyses (figure 5*f*). To acquire a historical, rather than a contemporary, picture of the PI, we used all spacers regardless of the presence of PAM sequences or type III CRISPR-Cas systems (electronic supplementary material, figure S5). The only major change using these conditions is the increased immunity of the Yellowstone population to SSVs provided by a shared spacer that cross-targets several viruses (low DI values, electronic supplementary material, figure S5B). This finding suggests that PAM loss from targeted protospacers has little effect on the overall PI structure within these two *S. islandicus* populations.

### (f) *S. islandicus* rod-shaped viruses evolve in response to CRISPR immunity

**Figure 6.**
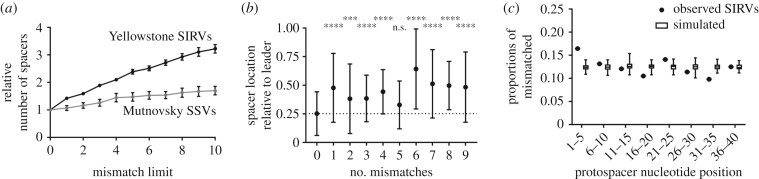
Viruses evolve in response to targeting by spacers. (*a*) The relative number of spacers that target PAM-containing protospacers in Yellowstone SIRVs (black) or Mutnovsky SSVs (grey) when the allowable number of mismatches between spacer and protospacer is relaxed. (*b*) The spacer location relative to the leader end of its CRISPR locus grouped by the number of mismatches to PAM-containing protospacers in Yellowstone SIRVs. The spacer immediately following the CRISPR leader sequence is at 0 and the spacer farthest from the leader sequence is at 1. Error bars indicate standard deviations. The relative location of mismatched spacers was compared with the location of perfectly matching spacers (0 mismatches, dashed line) by one-way ANOVA with Dunnett's multiple comparison test (n.s., no significance, ***p < 0.001, ****p < 0.0001). (*c*) The locations of all mismatches between spacers and PAM-containing protospacers in Yellowstone SIRVs. Observed data show the proportion of mismatches found within 5 bp windows of the protospacer oriented so the PAM is located upstream of nucleotide position 1. The simulated data represent 2037 mismatches randomly located across protospacers. Whiskers represent the minimum and maximum proportions from 100 simulations.

We investigated whether there were signatures of CRISPR-Cas-driven viral evolution in the mismatch mutations between the PAM, spacer and protospacer in our populations. While we cannot directly follow viral evolution with sequences representing a single point in time, we can assume that some CRISPR spacers targeted ancestral versions of the virus. By analysing spacers that target mismatched protospacers, we can infer how viruses have evolved. To increase the number of putative viral mutations and our power to detect directed evolution, we expanded our mismatch limit to 10 bases in spacers targeting PAM-containing protospacers. We observed that a higher proportion of SIRV-specific spacers have mismatches than do SSV-specific spacers (figure 6*a*). To determine whether these mismatches between spacers and protospacers indicate directed evolution in response to CRISPR targeting, we identified the location of SIRV-specific spacers within CRISPR arrays. The addition of new spacers generally occurs at the leader end of a CRISPR array [11,76]. Therefore, we would expect newer spacers to have fewer mismatches than older spacers if viruses are evolving after being targeting by CRISPR immunity. We found that Yellowstone spacers perfectly targeting SIRV protospacers are on average nearer the leader end of their CRISPR locus than spacers that target PAM-containing protospacers with mismatches (figure 6*b*). This finding suggests that mutated protospacers arise in SIRVs after spacers are acquired by the host population. Owing to fewer mismatched spacers targeting SSVs, we did not have sufficient power to test this hypothesis in these viruses.

Finally, we used the location of mismatches within a protospacer to investigate whether there is directed virus evolution to evade CRISPR immunity. Mutations in the PAM and in the seed region, which are the seven nucleotides nearest to PAM (for *S. islandicus*), have the greatest effect on the efficiency of CRISPR interference [71,77,78]. Bacteriophage CRISPR escape mutants accumulate mutations in the seed region of protospacers [21]. Using PAM-containing protospacers with no more than 10 mismatches to a spacer, we determined the location of all mismatches in all unique SIRV protospacers and found that they were not randomly distributed (*χ*^2^ (7, *N* = 2037) = 24.23, *p* = 0.0010) (figure 6*c*). We found that mutations in the five bases nearest to the PAM occur more frequently than in even the most extreme value obtained in 100 simulations of 2037 random mutations in a 40 nucleotide protospacer (figure 6*c*). Additionally, we found a relatively high number of mismatches between positions 21 and 25 of the protospacer, which may be important for spacer targeting specificity in *S. islandicus* [78]. We also performed this analysis on the 169 mismatches between spacers and Mutnovsky SSVs, but the distribution of mismatches was no different than random (*χ*^2^ (7, *N* = 169) = 4.804, *p* = 0.6839). Together, our results suggest that SIRVs evolve in a directed way in response to CRISPR-Cas immunity in the Yellowstone population.

## 4. Discussion

We investigated whether *in silico* and *in vitro* predictions about the distributed population structure of CRISPR-Cas immunity are applicable in nature using two geographically distinct populations of *S. islandicus* [26,27]. Consistent with these predictions, we found that both populations evolve DI, whereby individual strains are immune to local viruses through unique, rather than shared, CRISPR spacers. Each population develops this immunity structure targeting a different family of viruses. The Mutnovsky volcano population from Kamchatka, Russia, exhibits high, increasing PDI to chronic SSVs circulating at the time point of sampling, but does not have immunity to lytic SIRVs, which have never been identified at this location. By contrast, the Nymph Lake population from Yellowstone National Park, USA, exhibits very low immunity to SSVs, despite their prevalence in the population, and nearly complete immunity to circulating lytic SIRVs. Using a conservative spacer mismatch tolerance, the average PDI of the 2010 Mutnovsky population targeting free SSVs (0.45) is lower than that of the Yellowstone population targeting SIRVs (0.92) and higher than Yellowstone targeting SSVs (0.009), suggesting that the immune structure of one population does not always indicate how others are structured, even in targeting similar viruses. Despite our study being limited to only two virus types, our results may suggest that lytic viruses and freely circulating viruses are more likely to elicit broad diversified immune responses in *Sulfolobus* communities than are non-lytic or integrated viruses. These different interactions may also be related to SSVs following a different trajectory of symbiosis from antagonisms to mutualism [79].

High PDI can promote increased genetic diversity, a stable population structure and constrained viral evolution [26,27]. We have previously shown that the Mutnovsky population has high genetic diversity that is maintained over time [8,25]. The high virus-targeting PDI we present here, with different immunity providing spacers in different genetic backgrounds, may be a key contributor to the maintenance of genetic diversity within this natural microbial population. The general lack of dominant virus-targeting CRISPR spacers in both of our populations suggests that there has been relatively stable population without selective sweeps of immunity. While there are a few SSV- or SIRV-specific spacers that are shared by more than 10% of a population, these spacers are dwarfed in number by those that are unshared or shared sparingly. Spacers that target multiple viruses are common within these *S. islandicus* populations. A cross-reactive spacer would provide a strain with immunity to multiple viruses at once, increasing its utility and allowing it to retain function if some of the targeted viruses evolve to evade immunity. Immune cross-reactivity may also lead to viruses being structured into genetically divergent groups that can only infect subsets of the host population [80].

High PDI combined with cross-reactive spacers would make it difficult for viruses to efficiently evade CRISPR-Cas immunity. We found that different types of viruses use different mechanisms to evade immunity. Mutnovsky SSVs may increase the size of the susceptible host population by shortening their genomes through the loss or shuffling of variable genes containing targeted protospacers. Recombination has been suggested as a strategy to evade CRISPR-Cas immunity [21,28]. This strategy is plausible for SSVs because much of their genetic content is unnecessary for replication and recombination has been suggested to promote the genetic diversity observed among fuselloviruses [49,81]. Indeed, we observe that some SSVs appear to be chimeras of multiple SSVs suggesting recombination events (electronic supplementary material, figure S6). While SSVs have relatively few mismatches in protospacers, most targeted SIRV protospacers contain mismatches. These mismatches are enriched in the seed region that is important for protospacer recognition by spacers. Additionally, we observe a much lower density of PAM sequences in SIRVs than SSVs. Therefore, we conclude that point mutation in protospacers and the preemptive loss of PAM targets are common CRISPR-Cas immune evasion mechanisms of SIRVs.

Even with the possibility of evading CRISPR-Cas immunity through point mutations, SIRVs are still targeted with very high PDI and IDI, raising the question of how these viruses persist in the Yellowstone population. With nearly all strains immune to SIRVs, herd immunity would probably make it difficult for CRISPR-escape mutants to spread within the *S. islandicus* population. In addition to *S. islandicus,* SIRVs may have other host organisms that they infect. *Sulfolobus acidocaldarius* and *Acidianus hospitalis* populations from Yellowstone National Park encode CRISPR spacers that target SIRVs, suggesting that they encounter this type of virus [42,82]. Alternatively, these viruses may have ways of inactivating CRISPR-Cas systems. Anti-CRISPR proteins that inhibit type I-D CRISPR-Cas systems have been recently identified in SIRVs isolated from elsewhere in the world [83]. While SIRVs from Yellowstone do not possess close homologues of the reported *Sulfolobus* virus anti-CRISPRs, these types of proteins are structurally diverse even among closely related bacteriophage [84].

The potential for anti-CRISPR genes in *Sulfolobus* viruses may help to explain the auto-immunity we observe in the Mutnovsky population. Three of the 11 integrated SSVs are targeted by their own host strain with one or more spacers that perfectly match PAM-containing protospacers. Self-targeting CRISPR spacers provide a strong selective pressure to avoid auto-immunity by losing or modifying either the targeted protospacer or the CRISPR system [61,85]. The type I CRISPR system is intact for each of these strains. While type III CRISPR targeting can prevent replication of proviruses, this is unlikely here owing to the presence of PAMs in the protospacers and that only one of the four auto-immune spacers is antisense to a predicted mRNA strand [23,86]. While we did not identify any known *Sulfolobus* anti-CRISPR genes in any of the Mutnovsky SSVs or strains, their presence could allow for the observed tolerance of auto-immunity [87]. Of note, the three strains that self-target their integrated SSV (M.03.0.42, M.04.0.37 and M.06.0.8) have the second, fourth and sixth most spacers out of the 50 Mutnovsky strains (figure 1*b*), suggesting that high spacer number may be correlated with autoimmunity and/or tolerance to autoimmunity. Strains M.03.0.42 and M.04.0.37 are both immune to most contemporary SSVs, many of which they target with multiple spacers (electronic supplementary material, table S1). It is also worth noting that strain M.06.0.8 has two integrated SSVs. Additionally, different SSVs may elicit different CRISPR-Cas responses in their host cell [88].

Our data from natural populations paint a richer picture of how antiviral CRISPR immunity is structured in *S. islandicus* than would have been possible if we had looked at only a few individual strains or viruses. Our results show that natural populations do indeed evolve immunity that is distributed among many individuals that each possess unique ways of targeting a single virus. Both sides of this coevolutionary equation promote the maintenance of genetic diversity within the host and virus populations, which our dataset probably underestimates owing to sampling depth and culture biases [89,90]. While our approach using the CRISPR spacer repertoires of 21–40 individual cells from a population is a step towards more completely defining immunity in microbial populations, this level of sampling has probably not exhausted the diversity or fully captured the nuances of the population structure. To further refine this picture of CRISPR-Cas immunity, and to apply it to other microbial systems, we will need future studies focusing on natural isolates that sample populations more frequently, more deeply or over longer periods of time. Our study shows the utility of using natural populations as a tool to test hypotheses that stem from *in silico* and *in vitro* work, and as a tool to generate new hypotheses related to the mechanisms of CRISPR immunity and virus–host coevolution.

## Supplementary Material

Supplemental data and figures

## Supplementary Material

CRISPR.Spacers - List of all spacers used for this analysis from Kamchatka

## Supplementary Material

CRISPR.Spacers - List of all spacers used for this analysis from Yellowstone

## Data accessibility

This article has no additional data.
