# Supplementary material for: Diversified local CRISPR-Cas immunity to viruses of Sulfolobus islandicus
Source: Philos Trans R Soc Lond B Biol Sci. 2019 Mar 25;374(1772):20180093. doi: 10.1098/rstb.2018.0093 (PMC6452263; doi:10.1098/rstb.2018.0093)
Supplement: Supplemental data and figures [file rstb20180093supp1.pdf]

Diversified local CRISPR-Cas immunity to viruses of *Sulfolobus islandicus*

Authors

Matthew D. Pauly[1], Maria A. Bautista[1,2], Jesse A. Black[1], and Rachel J. Whitaker[1,3,] *

[1] Department of Microbiology, University of Illinois at Urbana-Champaign, 601 S. Goodwin Ave., Urbana, IL 61801, USA.

[2] Current address: Department of Biological Sciences, University of Calgary, 2500 University Dr. NW., Calgary, AB T2R 1K8, Canada.

[3] Carl R. Woese Institute for Genomic Biology, University of Illinois at Urbana-Champaign, 1206 W. Gregory Dr., Urbana, IL 61801, USA.

* Correspondence: rwhitaker@life.illinois.edu ; (217) 244-8420

Supplementary Information

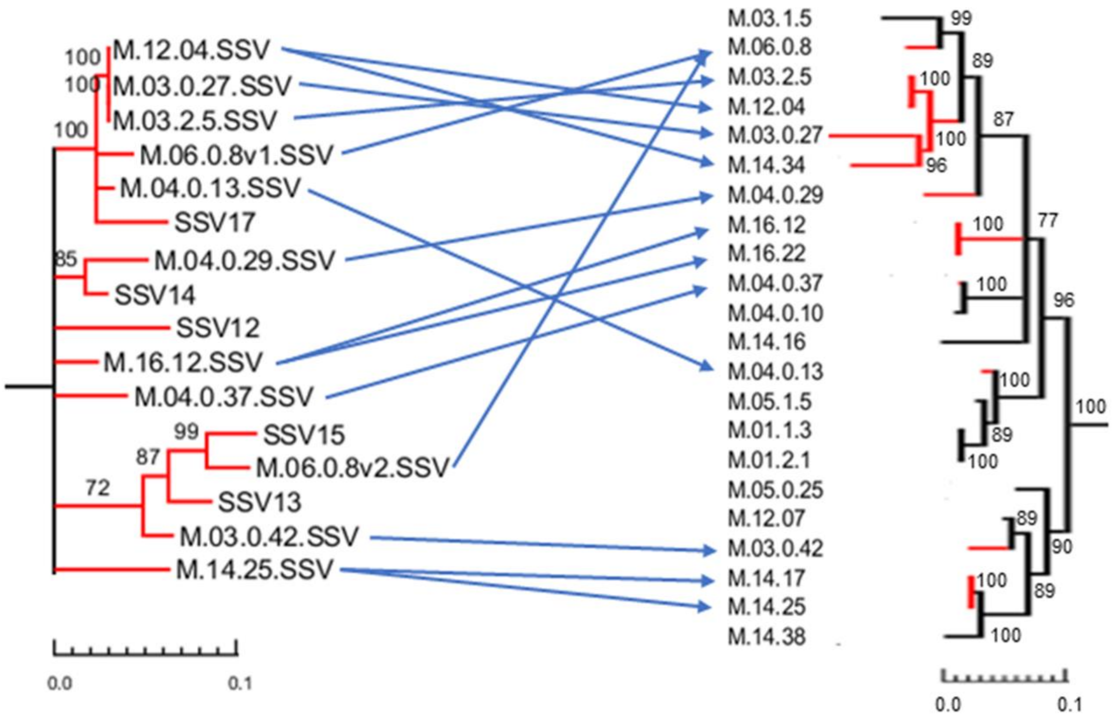

Supplementary Figure 1. Phylogenetic comparison of Mutnovsky SSVs and Mutnovsky *S. islandicus* strains.

Phylogenetic tree of Mutnovsky SSVs built with RAxML version 8 using concatenated, aligned nucleotide sequences from 10 shared core genes (left). Nodes with bootstrap values less than 70% are collapsed. Numbers indicate bootstrap values for the upstream node. Phylogenetic tree of all Mutnovsky *S. islandicus* strains created using ClonalFrame v1.2 using alignments to regions of the M.16.27 genome present in all strains (right). Only the portion of the phylogenetic tree containing strains with integrated SSVs (red branches) is shown. Blue arrows indicate which SSVs are integrated into which strains.

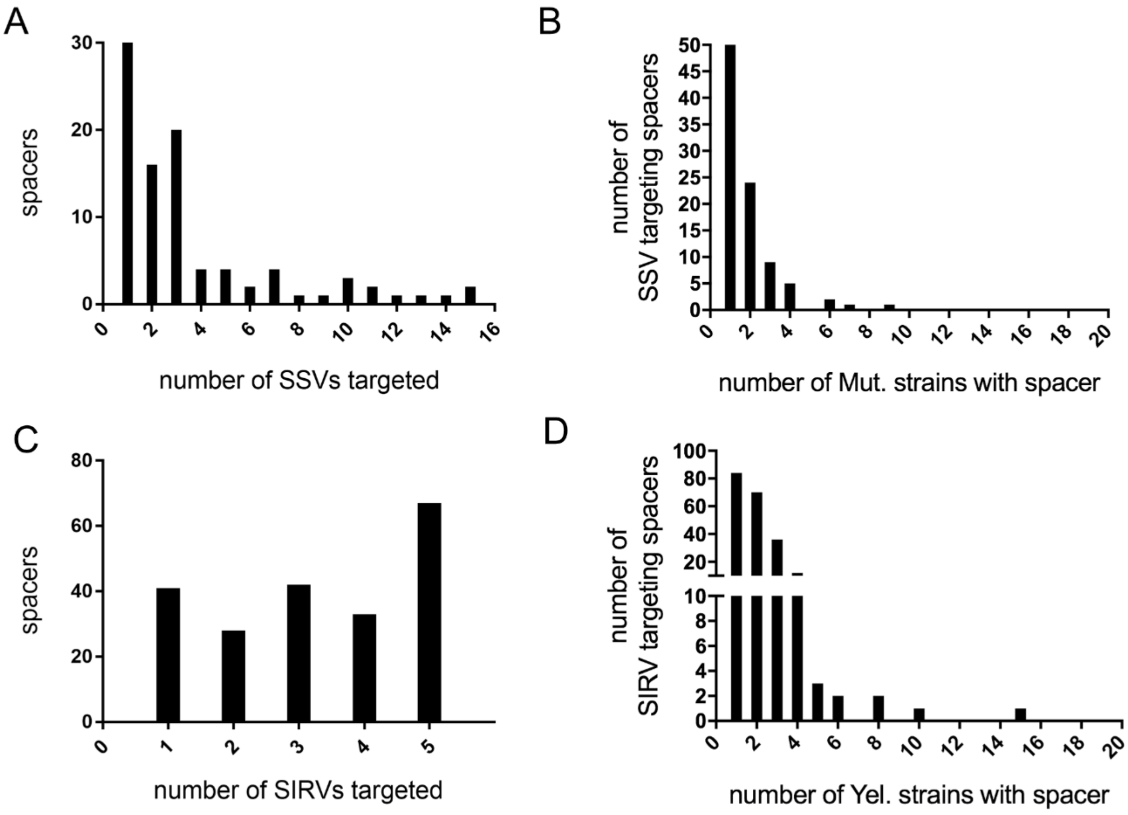

Supplementary Figure 2. Most spacers target multiple viruses and are shared sparingly.

A. The number of SSVs targeted by each SSV-targeting Mutnovsky spacer. **B.** The number of Mutnovsky strains that share each SSV-targeting spacer. **C.** The number of SIRVs targeted by each SIRV-targeting Yellowstone spacer. **D.** The number of Yellowstone strains that share each SIRV-targeting spacer.

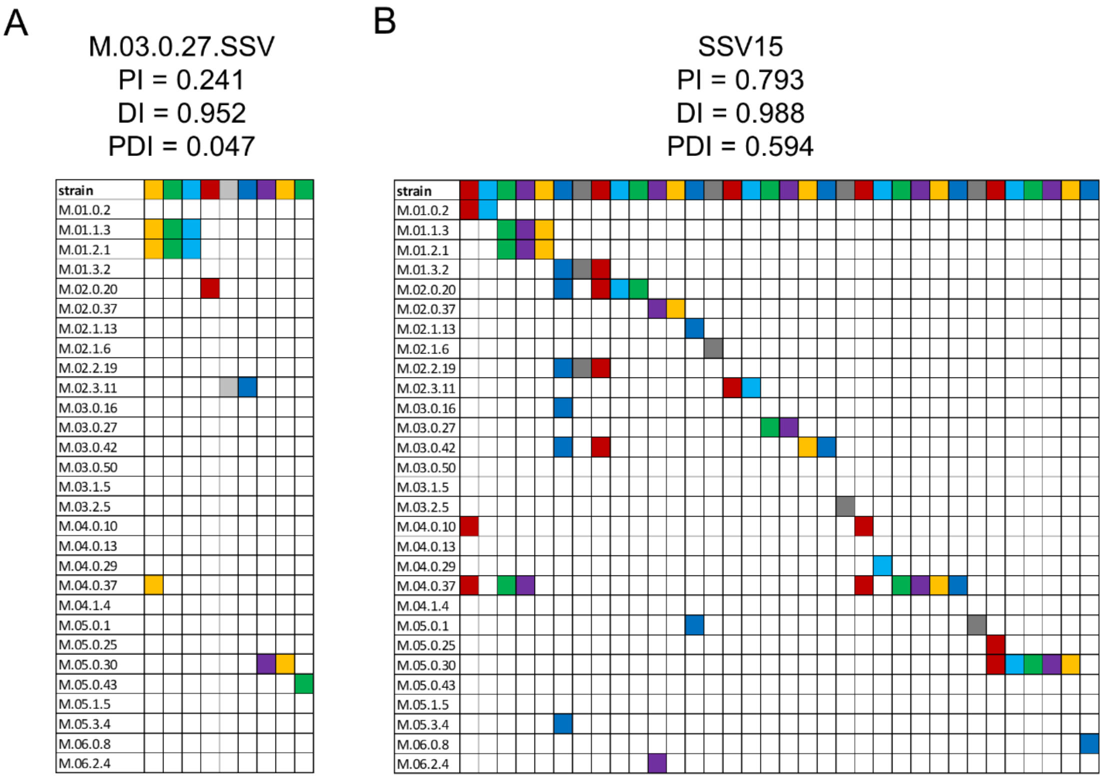

Supplementary Figure 3. Virus-targeting spacers within a population.

Diagrams representing immunity structure of the 2010 Mutnovsky population targeting M.03.0.27.SSV (**A**) and SSV15 (**B**). Each column indicates a unique spacer targeting protospacers in the virus. Colored boxes indicate that a strain from the population (first column) possesses that spacer. Strains with no colored boxes in their row do not target the virus. Repeated colors do not indicate relationships among spacers.

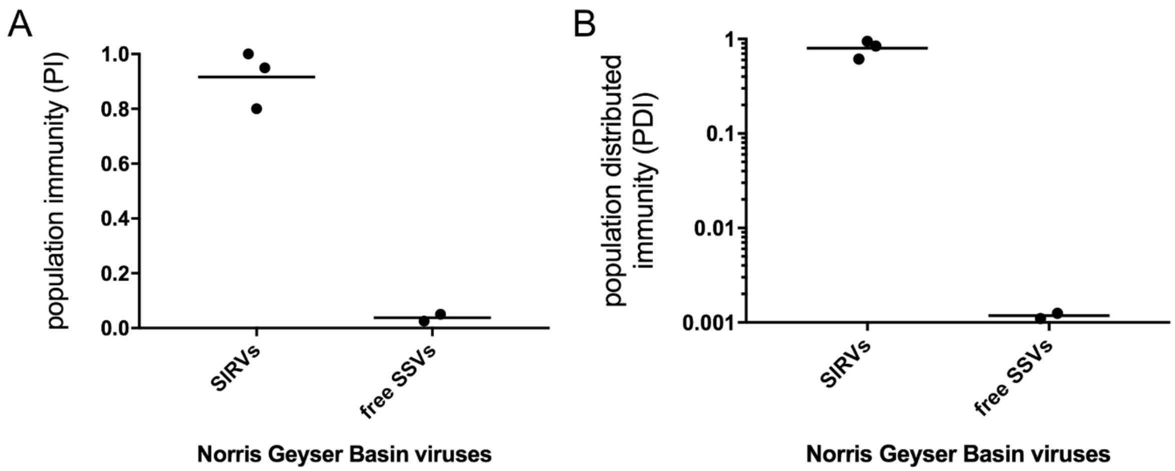

Supplementary Figure 4. Targeting of viruses from Norris Geyser Basin.

The PI (**A**) and PDI (**B**) for the Yellowstone *S. islandicus* population from the Nymph Lake area targeting viruses isolated the Norris Geyser Basin area of Yellowstone National Park. Norris Geyser Basin SSVs include two newly identified cell-free viruses (SSV11 and SSV18) and the SIRVs include SIRV8, SIRV9, and SIRV10 (Bautista *et al.* 2017).

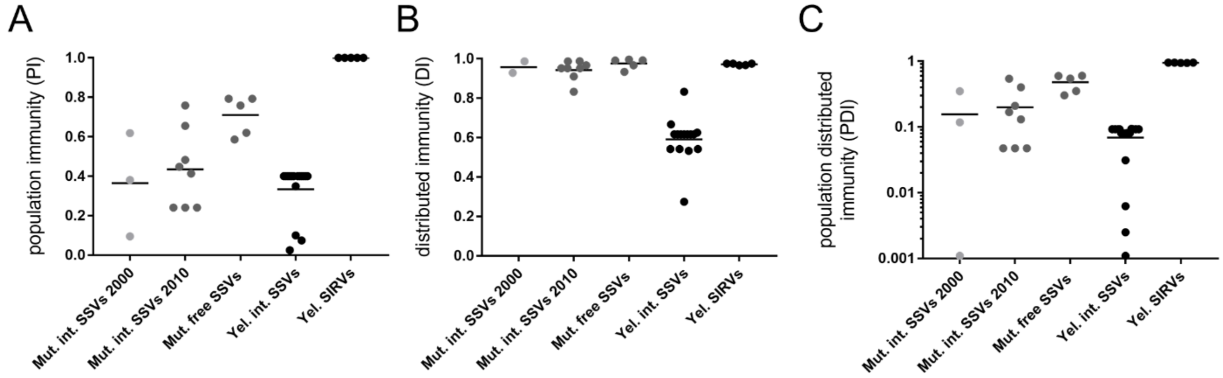

Supplementary Figure 5. CRISPR-Cas immunity without criteria for Type III system.

The PI (**A**), DI (**B**), and PDI (**C**) for contemporary interactions between *S. islandicus* populations (light gray = Mutnovsky 2000, dark gray = Mutnovsky 2010, black = Yellowstone 2012) and virus groups (indicated on x-axis). The average and values for individual viruses are shown. Immune metrics were calculated using all spacers with 4 or fewer mismatches to a viral protospacer with no criteria for the presence of a PAM sequence or type III CRISPR system.

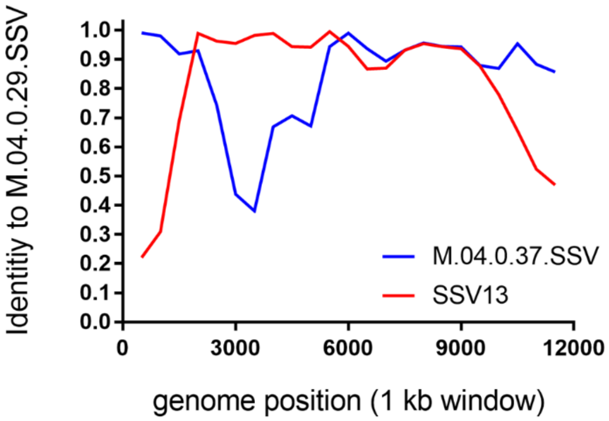

Supplementary Figure 6. SSVs share genetic material with multiple viruses.

The nucleotide identity of M.04.0.37.SSV and bases 4500-16184 of SSV13 aligned to the genome of M.04.0.29.SSV. Multisequence alignment was generated using MAFFT. Percent identities are shown for 1000 bp windows with 500 bp overlaps.

Supplementary Table 1. Mutnovsky Population CRISPR-Cas targeting

						spacers targeting																
strain	A1,A2 spacers	C spacers	NL spacers	Type I	Type III	M.03.0.27.SSV	M.03.0.42.SSV	M.03.2.5.SSV	M.04.0.13.SSV	M.04.0.29.SSV	M.04.0.37.SSV	M.06.0.8v1.SSV	M.06.0.8v2.SSV	M.12.04.SSV	M.14.25.SSV	M.16.12.SSV	SSV12	SSV13	SSV14	SSV15	SSV17	SSV immunity
**2010 strains**																						
M.01.0.2	162	77		X	X	0	1	0	1	0	0	0	1	0	2	0	1	2	2	2	0	0.50
M.01.1.3	117	114		X		3	3	3	2	3	2	1	1	3	3	2	3	3	2	3	2	1.00
M.01.2.1	216	146		X		3	3	3	2	3	2	1	1	3	3	2	3	3	2	3	2	1.00
M.01.3.2	118	75		X		0	3	0	0	1	1	0	2	0	2	1	1	3	2	3	1	0.69
M.02.0.20	172	76		X	X	1	5	1	0	2	3	1	4	1	4	2	1	2	2	4	2	0.94
M.02.0.37	104	97		X	X	0	2	0	0	0	0	0	0	0	2	0	0	2	0	2	2	0.31
M.02.1.13	158	69		X	X	0	1	0	1	0	0	0	2	0	1	0	1	3	1	1	1	0.56
M.02.1.6	122			X	X	0	0	0	1	0	0	0	2	0	0	0	1	2	1	1	1	0.44
M.02.2.19	117	85		X		0	3	0	0	1	1	0	2	0	1	1	1	2	2	3	1	0.69
M.02.3.11	121			X	X	2	1	2	1	2	2	2	2	2	0	2	1	1	1	2	1	0.94
M.03.0.16	107	76		X		0	2	0	0	1	1	0	1	0	1	1	1	2	1	1	0	0.63
M.03.0.27	81			X		0	0	0	1	0	0	0	0	0	0	0	0	0	0	2	0	0.13
M.03.0.42	302	71		X	X	0	3	0	2	1	2	0	4	0	3	2	3	2	5	4	1	0.75
M.03.0.50	146	132		X	X	0	0	0	0	0	0	0	0	0	2	0	0	0	0	0	1	0.13
M.03.1.5	102	82		X		0	0	0	0	0	0	0	0	0	2	0	0	2	0	0	0	0.13
M.03.2.5	168			X	X	0	0	0	0	0	0	0	1	0	2	0	0	1	0	1	1	0.31
M.04.0.10	101	76		X	X	0	0	0	1	0	0	0	1	0	2	0	1	0	1	2	1	0.44
M.04.0.13	99	96		X		0	0	0	0	0	0	0	0	0	0	0	0	0	0	0	1	0.06
M.04.0.29	114			X	X	0	1	0	0	0	0	0	1	0	2	0	0	1	0	1	1	0.38
M.04.0.37	279	75		X	X	1	5	1	4	2	1	1	6	1	6	1	5	5	4	8	3	1.00
M.04.1.4	214	26		X	X	0	1	0	0	0	0	0	2	0	1	0	0	2	0	0	0	0.25
M.05.0.1	133			X	X	0	1	0	1	0	0	0	2	0	1	0	1	2	1	2	1	0.56
M.05.0.25	153			X	X	0	0	0	0	0	0	0	0	0	0	0	0	0	0	1	0	0.06
M.05.0.30	344	41		X	X	2	0	2	1	1	1	2	4	2	4	2	2	5	2	5	3	0.94
M.05.0.43	131			X	X	1	0	1	1	1	1	1	0	1	1	1	1	1	1	0	2	0.81
M.05.1.5	93	67		X		0	0	0	0	0	0	0	0	0	0	0	0	0	0	0	0	0.00
M.05.3.4	96	78		X		0	1	0	0	1	1	0	1	0	1	1	1	1	1	1	0	0.63
M.06.0.8	219	38		X	X	0	0	0	0	0	0	0	1	0	3	0	0	1	2	1	1	0.38
M.06.2.4	154	98		X	X	0	1	0	0	0	0	0	0	0	0	0	0	0	0	1	1	0.19
susceptible						0.76	0.41	0.76	0.55	0.59	0.59	0.76	0.31	0.76	0.24	0.59	0.41	0.24	0.38	0.21	0.28	
**2000 strains**																						
M.12.04	168			X	X	0	0	0	0	0	0	0	1	0	2	0	0	1	0	1	1	0.31
M.12.07	85	85		X		0	1	0	0	0	0	0	0	0	0	1	0	1	0	0	0	0.19
M.12.37	116			X		0	3	0	0	1	1	0	2	0	1	1	1	2	2	3	1	0.69
M.12.46	153			X	X	0	0	0	0	0	0	0	2	0	0	0	0	1	0	2	1	0.25
M.14.16	147	57		X		0	1	0	0	0	0	0	0	0	2	0	0	3	0	0	1	0.25
M.14.17	146	39		X	X	2	1	2	2	2	2	2	3	2	0	2	1	2	1	3	2	0.94
M.14.25	113	109		X	X	2	1	2	2	2	2	2	3	2	0	2	1	2	1	3	2	0.94
M.14.34	143	38		X		0	0	0	0	0	0	0	1	0	2	0	0	1	1	1	1	0.38
M.14.38	138	40		X	X	0	1	0	0	0	0	0	0	0	1	0	0	0	0	1	1	0.25
M.16.02	186			X		0	0	0	2	0	0	0	0	0	1	0	2	0	2	0	0	0.25
M.16.04	139			X	X	0	1	0	0	0	0	0	1	0	1	0	0	1	0	0	0	0.25
M.16.12	111	34		X	X	0	0	0	0	0	0	0	2	0	0	0	0	2	0	1	0	0.19
M.16.13	110	53		X	X	0	1	0	0	0	0	0	0	0	1	0	0	1	0	1	1	0.31
M.16.22	111	52		X	X	0	0	0	0	0	0	0	2	0	0	0	0	2	0	1	0	0.19
M.16.23	113	59		X	X	0	0	0	3	0	0	0	0	0	0	0	3	1	3	0	0	0.25
M.16.27	164	71		X	X	0	3	0	1	1	2	0	2	0	2	2	2	2	3	2	0	0.69
M.16.30	137	19		X	X	0	0	0	0	0	0	0	0	0	0	0	0	0	0	0	0	0.00
M.16.40	159			X	X	0	0	0	0	0	0	0	0	0	0	0	0	0	0	0	2	0.06
M.16.43	179			X		0	2	0	0	1	0	1	4	0	1	1	0	3	1	2	1	0.63
M.16.46	161	25		X	X	0	3	0	0	1	2	0	2	0	2	2	1	1	2	2	0	0.63
M.16.47	153	82		X		0	1	0	0	0	0	0	0	0	1	1	0	0	0	0	0	0.19
susceptible						0.90	0.43	0.90	0.76	0.71	0.76	0.86	0.43	0.90	0.43	0.62	0.67	0.24	0.57	0.38	0.48	

Supplementary Table 2. Yellowstone population CRISPR-Cas targeting

						spacers targeting																				
strain	A1,A2 spacers	C spacers	NL spacers	Type I	Type III	SIRV4	SIRV5	SIRV6	SIRV7	SIRV11	NL01B.C01.01.SSV	NL01B.C01.03.SSV	NL01B.C01.05.SSV	NL01B.C01.06.SSV	NL01B.C01.07.SSV	NL01B.C01.09.SSV	NL01B.C01.13.SSV	NL01B.C01.14.SSV	NL01B.C01.18.SSV	NL01B.C01.20.SSV	NL01B.C01.22.SSV	NL01B.C01.24.SSV	NL03.C02.01.SSV	NL03.C02.05.SSV	NL13.C01.02.SSV	NL13.C01.04.SSV
NL01B.C01.01	148		61	X	X	11	14	13	14	9	0	0	0	0	0	0	0	0	0	0	0	0	0	0	0	0
NL01B.C01.03	199			X	X	19	16	16	17	18	0	0	0	0	0	0	0	0	0	0	0	0	0	0	0	0
NL01B.C01.04	117		70	X		4	4	3	5	3	0	0	0	0	0	0	0	0	0	0	0	0	0	0	0	0
NL01B.C01.05	148		62	X	X	11	14	13	14	9	0	0	0	0	0	0	0	0	0	0	0	0	0	0	0	0
NL01B.C01.06	150			X	X	12	14	14	14	13	0	0	0	0	0	0	0	0	0	0	0	0	0	0	0	0
NL01B.C01.07	192			X		5	6	6	6	6	0	0	0	0	0	0	0	0	0	0	0	1	0	0	0	0
NL01B.C01.08	80		73	X		2	2	2	2	1	0	0	0	0	0	0	0	0	0	0	0	0	0	0	0	0
NL01B.C01.09	137			X	X	4	5	4	6	4	0	0	0	0	0	0	0	0	0	0	0	0	0	0	0	0
NL01B.C01.10	77		58	X		2	2	2	2	1	0	0	0	0	0	0	0	0	0	0	0	0	0	0	0	0
NL01B.C01.11	78		66	X		2	2	2	2	1	0	0	0	0	0	0	0	0	0	0	0	0	0	0	0	0
NL01B.C01.12	80		53	X		2	2	2	2	1	0	0	0	0	0	0	0	0	0	0	0	0	0	0	0	0
NL01B.C01.13	107		62	X	X	4	8	7	9	6	0	0	0	0	0	0	0	0	0	0	0	0	0	0	0	0
NL01B.C01.14	125			X	X	10	14	14	15	10	0	0	0	0	0	0	0	0	0	0	0	0	0	0	0	0
NL01B.C01.15	173			X		11	14	13	15	13	0	0	0	0	0	0	0	0	0	0	0	0	0	0	0	0
NL01B.C01.16	137			X	X	10	11	10	12	8	0	0	0	0	0	0	0	0	0	0	0	0	0	0	0	0
NL01B.C01.17	76			X		2	3	3	3	3	0	0	0	0	0	0	0	0	0	0	0	0	0	0	0	0
NL01B.C01.18	184		55	X	X	11	16	15	16	10	1	0	1	1	1	1	1	0	1	1	1	0	1	1	1	1
NL01B.C01.19	81		76	X		2	2	2	2	1	0	0	0	0	0	0	0	0	0	0	0	0	0	0	0	0
NL01B.C01.20	108		50	X	X	4	8	7	9	6	0	0	0	0	0	0	0	0	0	0	0	0	0	0	0	0
NL01B.C01.21	176			X		7	9	9	9	8	1	0	1	1	1	1	1	0	1	1	1	1	1	1	1	0
NL01B.C01.22	156			X	X	5	5	4	6	4	0	0	0	0	0	0	0	0	0	0	0	0	0	0	0	0
NL01B.C01.23	106		56	X	X	17	15	14	19	14	0	0	0	0	0	0	0	1	0	0	0	0	0	0	0	0
NL01B.C01.24	139		79	X	X	19	27	27	27	20	2	0	2	1	2	1	2	0	2	2	1	0	2	2	1	1
NL01B.C01.25	111		56	X	X	17	15	14	20	12	0	0	0	0	0	0	0	1	0	0	0	0	0	0	0	0
NL03.C02.01	143			X		3	5	5	5	6	0	0	0	0	0	0	0	0	0	0	0	0	0	0	0	0
NL03.C02.02	176			X	X	5	12	10	12	7	1	1	1	1	1	1	1	1	1	1	1	1	1	1	1	1
NL03.C02.03	81		64	X		2	2	2	2	1	0	0	0	0	0	0	0	0	0	0	0	0	0	0	0	0
NL03.C02.04	178			X		7	8	8	8	8	1	0	1	2	1	2	1	0	1	1	2	2	2	1	1	1
NL03.C02.05	260		16	X		10	11	11	9	10	0	0	0	0	0	0	0	0	0	0	0	0	0	0	0	0
NL03.C02.06	215		22	X	X	10	14	12	13	14	0	0	0	0	0	0	0	0	0	0	0	0	0	0	0	0
NL03.C02.07	68		39	X		4	6	6	6	3	0	0	0	0	0	0	0	0	0	0	0	0	0	0	0	0
NL03.C02.08	31			X		0	2	2	2	1	0	0	0	0	0	0	0	0	0	0	0	0	0	0	0	0
NL03.C02.09	158			X	X	2	5	4	5	4	0	0	0	0	0	0	0	0	0	0	0	0	0	0	0	0
NL03.C02.10	45		29	X		1	1	1	1	0	0	0	0	0	0	0	0	0	0	0	0	0	0	0	0	0
NL03.C02.11	140			X		5	6	5	8	7	0	0	0	0	0	0	0	0	0	0	0	0	0	0	0	0
NL03.C02.12	140			X		5	6	5	8	8	0	0	0	0	0	0	0	0	0	0	0	0	0	0	0	0
NL13.C01.01	80		63	X		2	2	2	2	1	0	0	0	0	0	0	0	0	0	0	0	0	0	0	0	0
NL13.C01.02	114		38	X	X	18	17	16	15	18	0	0	0	0	0	0	0	0	0	0	0	0	0	0	0	0
NL13.C01.03	75		50	X		3	5	5	5	3	0	0	0	0	0	0	0	0	0	0	0	0	0	0	0	0
NL13.C01.04	147		9	X		7	8	8	7	6	0	0	0	0	0	0	0	0	0	0	0	0	0	0	0	0
susceptible						0	0	0	0	0	0.88	0.98	0.88	0.88	0.88	0.88	0.88	0.93	0.88	0.88	0.88	0.90	0.88	0.88	0.88	0.90
