# Supplementary material for: Diversified local CRISPR-Cas immunity to viruses of Sulfolobus islandicus
Source: Philos Trans R Soc Lond B Biol Sci. 2019 Mar 25;374(1772):20180093. doi: 10.1098/rstb.2018.0093 (PMC6452263; doi:10.1098/rstb.2018.0093)
Supplement: CRISPR.Spacers - List of all spacers used for this analysis from Kamchatka [file rstb20180093supp2.docx]

All CRISPR spacers from the Mutnovsky *Sulfolobus islandicus* population from 2000 and 2010. Names indicate the strain name, CRISPR array locus, and an identifying number.

>M.01.0.2_C_1

ATAGATAGAAATTACTCTCTACTGGATCCGCAACTTC

>M.01.0.2_C_2

TTCTACACTTTCAGAGAACATAGTTTCTTTATTTACA

>M.01.0.2_C_3

TTAGCTAGATCAGCTGCTTCAGCTTCTCTCAGCGCTCTC

>M.01.0.2_C_4

TCTTTTAGTGTTGAAAGATTTGCTTTATCATTTAAAAAT

>M.01.0.2_C_5

TTAAGTCCTTTGATGTGACAGATTATATCAAAATTGAT

>M.01.0.2_C_6

TCTTGTAGAAACTTTATCCACTTTACTTTTTCTTCGC

>M.01.0.2_C_7

GTTAAGCTCTATGTCCTGCAGGATAATACTTTTG

>M.01.0.2_C_8

TATTACTCTTAGCAGCTTGCTCATAATTTATACTAA

>M.01.0.2_C_9

TTTCAATTCCTTTTAGGATTAATCAAAGAAGGATATCTT

>M.01.0.2_C_10

TCGTATATCTGTACAATCTTCAATTCGTTAAGTAGTTTC

>M.01.0.2_C_11

ATATTTTCAACTCTATAGCCGTTTTTCATGCATTCTTCTGAT

>M.01.0.2_C_12

CAATAATGTTTATGTCTTTTGTTTGCCTAAATTCTT

>M.01.0.2_C_13

TCCAATGCGTCTTGCAATTCTTCATCGTAAACCGCTTCTA

>M.01.0.2_C_14

CACTACTTGTTGTATATGCGTAACTATTAGAATTGATATT

>M.01.0.2_C_15

ATAACTCTACTACATTTTCTCATGTAAATTCTAGTGCAT

>M.01.0.2_C_16

TATTTTGCCCAATAGGTCTTTCGGAGTTATACCTTTCT

>M.01.0.2_C_17

GACTACAATTGGCAAAGATCATTCAGTTGGTAGTATAC

>M.01.0.2_C_18

TTCATGAAAACATCTCTGTCAAGTCTTTTTAATCCTAAA

>M.01.0.2_C_19

CTTGAATAAGCACTTATAGAATTTTTATTAATAGCTTCAG

>M.01.0.2_C_20

ATAGTCACGTATATTGGATACCCAAGCTTCTCATAATTA

>M.01.0.2_C_21

ATAAGTTTAACGCCTAGGGTACTCCCTGCGTAAATTC

>M.01.0.2_C_22

TCTCATCTTTAGCAATAAATCATGAGTTACTGGAA

>M.01.0.2_C_23

TAGACAGGTCAGTATAGGTCTTACCATCTAATAAAGCAAG

>M.01.0.2_C_24

GTTACAAAATTAGAATACTATTTAACAAACTTATCAAA

>M.01.0.2_C_25

TTTTTCTTTCACCTCGATGTAAATATTTACTTCTTCAA

>M.01.0.2_C_26

TTGCTAATGATTCAACGTTAGTCTCTACTATTTCTT

>M.01.0.2_C_27

TTATAGTCTTTATATTGTTCTTCTGTTAAATTGTTAAGATC

>M.01.0.2_C_28

TTTAATCTTATTTCTTATGTCTATGCCTTCTTCACGT

>M.01.0.2_C_29

TTAGAACAAATCGTAAATGAGTATTATGATATGAAGTGCC

>M.01.0.2_C_30

CCGCCCTACTTGTTTTTATATTAAATGCTATTCCC

>M.01.0.2_C_31

TAAGCTTATCAGATATTCTACAGCTTTGTCAAATTCTTT

>M.01.0.2_C_32

AAATCAATATATAGTGCTTCAGATAGTGGCACATAT

>M.01.0.2_C_33

AGTTTAATATACTCAACCCTAGCCTTTATCATTTCTT

>M.01.0.2_C_34

ATTTTTATCGCCCCGTCAATCATCAGATCTTTAACG

>M.01.0.2_C_35

TTAGGAAGAAGGGGGACTACTGTAGTGTGTGTTACAATA

>M.01.0.2_C_36

TTCATGGGCGGGTCACCTCATCCTTTCCCTTTATTTCA

>M.01.0.2_C_37

AAATATTAAAGTTTTGCATTCTTTGTACGCGTCACCAAA

>M.01.0.2_C_38

ATTACAGTTTGACGAGAATCATGGACTTGTAAAACT

>M.01.0.2_C_39

ATTTCGTACATCAAGTATTTTGATGACAGTAAGAAATATTG

>M.01.0.2_C_40

TTAGTATATCATATGCTTCCTTCATCATGTCAATATC

>M.01.0.2_C_41

GTATAATAACAAAGAGTGAAACTATGGACGCTATAAA

>M.01.0.2_C_42

AGGATCTCAAGACATATGGTAACAAGTTTGTTTTCTT

>M.01.0.2_C_43

TCGTGAGGGCAGAGATAGCAAAGAAGTCAAAGTATAATGC

>M.01.0.2_C_44

TTTTCAGATCCTCCCCTACCTTTACGCCCAGCAATCT

>M.01.0.2_C_45

TCTTTCACATACGTTTCTATACGCTTCTTCTTTCCATC

>M.01.0.2_C_46

ATATTCTTGCAATTGCTTCTAAAGTTCCAATGCTTATTG

>M.01.0.2_C_47

ACCATATCTTTCCATGCATCAGTTTCTTGACTTCCATAC

>M.01.0.2_C_48

CTAAACCTATCAGAGTTAGCAGAATATAGTAATATTGATATT

>M.01.0.2_C_49

TGATAATCTTCAACCTTAATAAATTCGAAATAAATATC

>M.01.0.2_C_50

ATATTTTTAAGCTTCTTAGTAAAAGAAGGATATCTT

>M.01.0.2_C_51

TCAAAATGAGTAATTTTTCTTCATGCAACATAATTCAC

>M.01.0.2_C_52

TATTCATATCTGCCAATGAGTAATGTATACTTCTAT

>M.01.0.2_C_53

ATATATTTCTTTACTAGTTGCTGTGACGATTTAACGCTGCT

>M.01.0.2_C_54

ATTTAAATTATATTTTAGGGCGTATCTGTCTAGCAGTTGTA

>M.01.0.2_C_55

AATCATATACTGTATCGTTCGTTACAATCACGTTATAT

>M.01.0.2_C_56

TCTTGTCTAGTTCATCTAACAATTTTAATAGCGTCTCGA

>M.01.0.2_C_57

ATTACACTTAAGATTGTTGCTTTTTTACTATTCTG

>M.01.0.2_C_58

ATTGTTAAATCAGTTATTCTCGGTCTCATTATAAAATTCA

>M.01.0.2_C_59

CTACATAAGCTATCTGGTTCCAAGGGAACGGCGTATT

>M.01.0.2_C_60

GAAAGTTCTTTTTGCAGACGTTCGCCTAATCGAATAATAT

>M.01.0.2_C_61

TTATTATTTACAGTTATTCTTATCTTTGCCATCCTCACCAC

>M.01.0.2_C_62

ATCAGTATTGGGAAGTAGATTGCTTTGCCTTTATTCTCT

>M.01.0.2_C_63

ATATATAGGTTCCATGTCATTGCTCCCCAACCATAATC

>M.01.0.2_C_64

GTAAAATACCAGGTTCCCTACTATAATCGTTACTGCC

>M.01.0.2_C_65

ACATTATTCACGAAATCTTCAACGCCACCGTAAGGTGCT

>M.01.0.2_C_66

ATGCTTTCGTTTTGTGTTTTTTCATTTACTTGTTCTT

>M.01.0.2_C_67

ACGTTCAACTTTAGCGATTCATCTACTTTCTCTTCATT

>M.01.0.2_C_68

ACTTTATACTGTAGATATTTACCGGGTAGGACAAAGTAT

>M.01.0.2_C_69

AACTCTAGTTTTATTGGTTTCTTTTCCCCCTTATTCTC

>M.01.0.2_C_70

AGGTAACTTCTAAGATAGTAAGGTAATGCTAAACGAACCAT

>M.01.0.2_C_71

TTTAATTCTTTAATATAAGTAAGCTGTTCTACATAGAA

>M.01.0.2_C_72

CTAGTTATCCCTGCCCTACCGGTAAGTCTAACGAATATA

>M.01.0.2_C_73

TAGATTTGTAATTTGAATTATATAACTATGAGTAAATGTT

>M.01.0.2_C_74

TTGTTACTCAAATATATTCACCTCCATCTTTTGT

>M.01.0.2_C_75

TTCAAATACCCTAATTGTTCTCTCTACCTCATTTAGATC

>M.01.0.2_C_76

AATTCCTTTATCTTTGCTTCCAGCTCTTCTATCTTCTTT

>M.01.0.2_C_77

CATATATAGTGAATTTCTTTTGAGATAATAGATTAT

>M.01.1.3_C_1

CATCATTATTTACTATGATGACGCTTATAGATATGTAG

>M.01.1.3_C_2

ATTGCCAACCAAGTGGGAGATTAAAGATAGTAGAAAGA

>M.01.1.3_C_3

TGAACTCTTCAGATACCTCGTGTTCGTTTTTTACTTCGT

>M.01.1.3_C_4

TGATATAGATTATAGTACATCACAGTTACAATTATTACAAA

>M.01.1.3_C_5

ACTTGTATATCAGATATCATGTCTACTAACGGCAAATTAT

>M.01.1.3_C_6

TTTGTGATAACTTCTTTCAATTCATCTAAATTAGCATCGG

>M.01.1.3_C_7

ATTTATTGTCTTCTTACCCTACAGCGAATTCCACACTCT

>M.01.1.3_C_8

AACAGTTACGCATATACAACAAGTAGTGCAATAAATTC

>M.01.1.3_C_9

TGTTCTTCTAATAGCCCATCTTCAATTAGTTCTTGTTTTG

>M.01.1.3_C_10

TTCAAAAACTGTTTAAAATTTATTTTATTTGTTCCCGT

>M.01.1.3_C_11

TTTTTTAATATTATCTCTATCTCTTTATCCTTCTTCTT

>M.01.1.3_C_12

ATACAGCTGGTTAACGGTTCAAGGATCGGCGAAGC

>M.01.1.3_C_13

AATGATTTTGAGATTATGCTAAATCCATCGAGTACTG

>M.01.1.3_C_14

ACTACATAAGAAGATTGTAATTGATAAGATGGCGGTG

>M.01.1.3_C_15

GTTATCTTATGGGGCGATATAAAGACGTTAAAATCAATT

>M.01.1.3_C_16

AACATACCTTGAAATTCACCTCTAAAAACCATTGAACTA

>M.01.1.3_C_17

TCCCAAAATACCTTTGGTAATCTTCACTCTGCTTCTTTG

>M.01.1.3_C_18

CACATATAATCAGTTTCATATCTACTCTTTAACTCATCAA

>M.01.1.3_C_19

CTAGAAATACAAATAACAATGCGAAGAATAAGCTGT

>M.01.1.3_C_20

ATAATTTCTTTCACCATCTTTTCTTCATTCTCAGTCAT

>M.01.1.3_C_21

TTTATACTTGTTCTAGTCGCTTTCTCTATACATTTTACG

>M.01.1.3_C_22

TTACCCTCCCTAATGCGGGGATATCAACACCATATCTT

>M.01.1.3_C_23

TACTTTACCTCAAGAAATAGTAGTAGATAGTCAATCCT

>M.01.1.3_C_24

CTTGGTAACCCAAATTCGTTTATCCCATTGTCTCTA

>M.01.1.3_C_25

TTGAAGTTTCAACTATCTCTTGCAGATTTGCTATTTCGT

>M.01.1.3_C_26

GAATATCCTTCTAATGATAGAGAATTTGCTAATTCGTTG

>M.01.1.3_C_27

TTTAAAAATGTATATAGTGTACTCATTTTAACTAACCTCCACC

>M.01.1.3_C_28

ACATTAGAAGAGTGGCAGATGTACAAAGGGAAAACTGTA

>M.01.1.3_C_29

TTTTGATCACAAACTTTTTAGCATACACTCTAGTTGTC

>M.01.1.3_C_30

CGTATTCTCTTGTCAATTGCCGTTACGCTCTGAGCAC

>M.01.1.3_C_31

TTCCCCATCACCATTATGTCACCTATTCCAACTGCTA

>M.01.1.3_C_32

TTTACCTCATTCCTTGCGTAAAGCTGGTAAGATATAGC

>M.01.1.3_C_33

ACTACTTTTCTATCCTGTATTTTAAAGTAACAAATGCT

>M.01.1.3_C_34

AGGAATCTCCTATCTTCTTTAAATAGATATAGGAAATC

>M.01.1.3_C_35

TCACTAATATCTTTCTCGTTTCTTATCTCGTATATA

>M.01.1.3_C_36

ATAATACTACGTGCACTATTTTAAAAGATATAGAA

>M.01.1.3_C_37

CAGAAGTATATGTAAATAAAGACTATGACGTAGATGTGA

>M.01.1.3_C_38

CCTTCTGCTTCACCGTAGTCAAACGGGCTATCGTCAT

>M.01.1.3_C_39

CTATCGCCTTTATATGAAAGTAGGGGTACGTGAGGA

>M.01.1.3_C_40

ACAACGCCCCTCCTAATATTAGCAAAAACGTGATATCGC

>M.01.1.3_C_41

TCTATCTCAATATTATCAATATCTAGCCTTAACTCATT

>M.01.1.3_C_42

GAACAGCTAATCAGATATCATTTTTCACGCAAAAAATTCAA

>M.01.1.3_C_43

TCATCCTCATCATAAAGATATAGCGGATCATCGCATTC

>M.01.1.3_C_44

TTAATTTGCAGAGCAGGGCATAGGGTGTGGCCTCCTGGCC

>M.01.1.3_C_45

AAGTATTCCAAGATGCTTGAGGAGTGGAGAAATCAAGTT

>M.01.1.3_C_46

AAGACCATTACCTTTACGGATATGAAATTGATGATATCGCT

>M.01.1.3_C_47

ATTTAGAAATAAGTTATGAAGCTTATCTTAGCTCAAATT

>M.01.1.3_C_48

CTTTATACTACAAAAATAGTATTTATAATGGTCATTAT

>M.01.1.3_C_49

GTGTACTGTCCATATGTAAAAATTAGAAAATAAAATAAA

>M.01.1.3_C_50

TTTAAATTGGATATAGGGAGTTTGTGTAACTAAAAG

>M.01.1.3_C_51

TCTTATAGCTACTGAACCCAATGGTACTTTTACAATAAT

>M.01.1.3_C_52

TGAATAAATGTAAATTACTGGAGTATAATTCACCACTTCG

>M.01.1.3_C_53

ATATTCTATCATAACTCAGCAGAATTAGTTAATAGTAAT

>M.01.1.3_C_54

TATATGTTCATTGAAATCACACCAGAAAGTTAAACCGA

>M.01.1.3_C_55

TGTGATAGAGGGGTATAGTGCAAATCAGATACAACAGT

>M.01.1.3_C_56

CACATTTCTCAAGCGCCGCAGCAGGTTATCCATTAAT

>M.01.1.3_C_57

TTCTTTTCTTTCCTTTACAATATAACCTCTTGCTATT

>M.01.1.3_C_58

AGGTTTTTAGCACTTCATCAATTCTTATATTGTCTGCA

>M.01.1.3_C_59

AAATAAAAGAAAAAATCAATCTAACTTTGAATACTCAA

>M.01.1.3_C_60

TGTGATAATTAAAAAGGAGACTTTACGTTGAGGGAATG

>M.01.1.3_C_61

CACCACATTAGGCTATTTGCTCTTAGCAGCATAAACTA

>M.01.1.3_C_62

AAAAAATAATTGAAAGTATAGTTATAGACCATATAAGAGAG

>M.01.1.3_C_63

CGTTAAACTTTTACTAACTTACAAACTTAC

>M.01.1.3_C_64

ACTAATTTCTTTTCCTCCTCTAACCTTTTCTTAACGTCA

>M.01.1.3_C_65

AGTGTTAATCAAGGGCTAATTTCACTATTAACTGATTT

>M.01.1.3_C_66

TCAAATAAAGTAAATTTACCATCGTTTGTATACAATCTT

>M.01.1.3_C_67

AACTTTTATCACGACTAACGTGACCGCAAATGTTGACCA

>M.01.1.3_C_68

ATTATTTGCTTAGCTTCTTCTTTACTGGAGAATTTGAC

>M.01.1.3_C_69

TTATCTTCGTGGGTTTGGGACATTTTTGATTCACCTAT

>M.01.1.3_C_70

TCTATGCTGTAGTGAGTGTAGCCCTCCACCAATATCT

>M.01.1.3_C_71

AGTAATATGCATTACTTATAATTAGAATAGGAAATCTTG

>M.01.1.3_C_72

ATCCCCCAAAAATATCTATAAAACATCTATTATATGACC

>M.01.1.3_C_73

TTATACAAATTATATTCTTCAGACTGAAACAATGCATAAGCC

>M.01.1.3_C_74

CCTCTTTCCGCTATCTTCTCATAAATTTTGTTTACATC

>M.01.1.3_C_75

AAAAGAACTAATAGTTCTGATTTTGCACACCCCATG

>M.01.1.3_C_76

TGAGACCGCGAATAACTGACCTAGCGATTGAAACTAGAGCG

>M.01.1.3_C_77

ATATTTGTCAATTGCATTACTTTTAAGAACATTACATAC

>M.01.1.3_C_78

TATTGTGAATAGATTAGGTTCAGTAGTCGTCTGTGTCTCGTT

>M.01.1.3_C_79

TTTTGTAACTCAGATAATATTTTTGCATCTGCACTATC

>M.01.1.3_C_80

TGAGTTTTTACAACAAACATGTCGATTAATTGTTTCTCT

>M.01.1.3_C_81

TACTGTGTATTATAAATGTAAGCATCAGAAAGACGTTTAA

>M.01.1.3_C_82

TATTATCCGCTGTTATGGATTGATATTACGGGGAGC

>M.01.1.3_C_83

GATTTGCTCATCGTATTTATTATAACACAAAGCACTAT

>M.01.1.3_C_84

TTGCAGATGTTGTAACGTGGGATAACAAATTATTTTCCCC

>M.01.1.3_C_85

TATTTTTTTAACTTTGCTGGAATATATTGAATGTAGTCT

>M.01.1.3_C_86

GTATTATCCGGATAAAGATAGAATACTTTTCTTAATAG

>M.01.1.3_C_87

TTGTTATCTAATATAAGTTGTTTGACATTCGGTGGTAAC

>M.01.1.3_C_88

AGTAACTCTGGTGTTTGCCTTATCCAAACGTTTGTTACG

>M.01.1.3_C_89

CGCCCTATGCTCAATTTAATTTATCTGCTGGTTACTAC

>M.01.1.3_C_90

ACTAATATCGCTTCAACGTCACCATATATAACTTTATAC

>M.01.1.3_C_91

TTCAAGATGTAAACGCATCTTTATTGCATTCGGAGAAATAGCA

>M.01.1.3_C_92

ATATTCCTCTAACGCAAACGGCGTTAAAACACTTGCTTTC

>M.01.1.3_C_93

TCTAAAATATATGCAATAAGGGGGGATCAAATATATCCCC

>M.01.1.3_C_94

GATAGAAACCCCTTGATGCTATTCTTAATTCCGTCAGA

>M.01.1.3_C_95

ACTTACGCTCTGAAAGAAGTAGTTTTTTTGCTTCTTCAC

>M.01.1.3_C_96

ATAGAAATAAAAGGATATGCTTCCCGTTACTGTTAGA

>M.01.1.3_C_97

GATTGATACTCTATGAAGTTATTCTTTGTTATATATA

>M.01.1.3_C_98

TTATAGTCTTTATATTGTTCTTCTGTTAAATTGTTAAGATC

>M.01.1.3_C_99

CTGTGCTTTATTGGTCTCACTCTTTCTACGTGAATCTTCACG

>M.01.1.3_C_100

TATAAATTGGCAAGGGTGGAATGTAACGATATACC

>M.01.1.3_C_101

TCTTTCACATACGTTTCTATACGCTTCTTCTTTCCATC

>M.01.1.3_C_102

AAATAAATATCATATAGATAATTATATTCAGCCTCGT

>M.01.1.3_C_103

TCAAATAGTGCATCAGATATTAAGAGACTTCTAACTTTAC

>M.01.1.3_C_104

GGGAATGATGTTACGTGAATCGTAACGTTAATAATGTTTG

>M.01.1.3_C_105

ATTATATAATGCGTAAGCAGATGCAGATATAGTTATAACAT

>M.01.1.3_C_106

CAGAACCTCCTCCTAGCTTCACCGTGCAGGTTCTCAAG

>M.01.1.3_C_107

TTTTTTTCCAGCTCTTTAATGATCTCATTAACACT

>M.01.1.3_C_108

ACATTTATAGCCACAATTACTGCAGTTTCTGTTTACCGTC

>M.01.1.3_C_109

TATATGTACTGTTTTGCCCAAATTGTATGGTGAACGCT

>M.01.1.3_C_110

ATATAGATTTAAGAATGAGAATGAAAAACTTGAGGCAG

>M.01.1.3_C_111

TGTCATAATTATCAATAACCCATTCAGTTATTGCATAAT

>M.01.1.3_C_112

TCGGTGATGATTATCCCTATGTTACTCACCTTACTATCA

>M.01.1.3_C_113

GTACACTAGATGCCGATATCGGTATTGACATGTTCACGCC

>M.01.1.3_C_114

ATAATCCATTGAGTTATTGCATAATATCCTTGTCCGCT

>M.01.2.1_C_1

CATCATTATTTACTATGATGACGCTTATAGATATGTAG

>M.01.2.1_C_2

ATGTATAGCTAGGTGAATTAGTAGTTCCAGTATTAATTAT

>M.01.2.1_C_3

CAGATCTATTTACTGGAGCAGGAGCTGCAGGGAGAGAGCTA

>M.01.2.1_C_4

ATTGCCAACCAAGTGGGAGATTAAAGATAGTAGAAAGA

>M.01.2.1_C_5

TGAACTCTTCAGATACCTCGTGTTCGTTTTTTACTTCGT

>M.01.2.1_C_6

TGATATAGATTATAGTACATCACAGTTACAATTATTACAAA

>M.01.2.1_C_7

ACTTGTATATCAGATATCATGTCTACTAACGGCAAATTAT

>M.01.2.1_C_8

TTTGTGATAACTTCTTTCAATTCATCTAAATTAGCATCGG

>M.01.2.1_C_9

ATTTATTGTCTTCTTACCCTACAGCGAATTCCACACTCT

>M.01.2.1_C_10

AACAGTTACGCATATACAACAAGTAGTGCAATAAATTC

>M.01.2.1_C_11

TGTTCTTCTAATAGCCCATCTTCAATTAGTTCTTGTTTTG

>M.01.2.1_C_12

TTCAAAAACTGTTTAAAATTTATTTTATTTGTTCCCGT

>M.01.2.1_C_13

TTTTTTAATATTATCTCTATCTCTTTATCCTTCTTCTT

>M.01.2.1_C_14

AGAGGGAGCCTTCGGGGTGTGGCTTCCCCCCTCCCATTTTCC

>M.01.2.1_C_15

TCTTTATCGTCTTCATTTATAAAATACTTAATATATTC

>M.01.2.1_C_16

ATACAGCTGGTTAACGGTTCAAGGATCGGCGAAGC

>M.01.2.1_C_17

ATATCTAAATCTAAGGGAATATCTGTCATGTTTTTGTC

>M.01.2.1_C_18

TATACCTTATGCTGATGTCTCTGAGCTTCCTGATATCA

>M.01.2.1_C_19

TATTATCCGCTGTTATGGATTGATATTACGGGGAGC

>M.01.2.1_C_20

ACTACATAAGAAGATTGTAATTGATAAGATGGCGGTG

>M.01.2.1_C_21

GTTATCTTATGGGGCGATATAAAGACGTTAAAATCAATT

>M.01.2.1_C_22

AACATACCTTGAAATTCACCTCTAAAAACCATTGAACTA

>M.01.2.1_C_23

TCCCAAAATACCTTTGGTAATCTTCACTCTGCTTCTTTG

>M.01.2.1_C_24

CTAGAAATACAAATAACAATGCGAAGAATAAGCTGT

>M.01.2.1_C_25

ATAATTTCTTTCACCATCTTTTCTTCATTCTCAGTCAT

>M.01.2.1_C_26

TTTATACTTGTTCTAGTCGCTTTCTCTATACATTTTACG

>M.01.2.1_C_27

TTACCCTCCCTAATGCGGGGATATCAACACCATATCTT

>M.01.2.1_C_28

ACAAACCATATCATTATCTCGGTTTGGGATATCATGAAC

>M.01.2.1_C_29

TACTTTACCTCAAGAAATAGTAGTAGATAGTCAATCCT

>M.01.2.1_C_30

TATTGTGAATAGATTAGGTTCAGTAGTCGTCTGTGTCTCGTT

>M.01.2.1_C_31

CTTGGTAACCCAAATTCGTTTATCCCATTGTCTCTA

>M.01.2.1_C_32

TTGAAGTTTCAACTATCTCTTGCAGATTTGCTATTTCGT

>M.01.2.1_C_33

ATGCTTTCGTTTTGTGTTTTTTCATTTACTTGTTCTT

>M.01.2.1_C_34

GAATATCCTTCTAATGATAGAGAATTTGCTAATTCGTTG

>M.01.2.1_C_35

TTTAAAAATGTATATAGTGTACTCATTTTAACTAACCTCCACC

>M.01.2.1_C_36

TTGTTATCTAATATAAGTTGTTTGACATTCGGTGGTAAC

>M.01.2.1_C_37

TTTTGATCACAAACTTTTTAGCATACACTCTAGTTGTC

>M.01.2.1_C_38

CGTATTCTCTTGTCAATTGCCGTTACGCTCTGAGCAC

>M.01.2.1_C_39

TTCCCCATCACCATTATGTCACCTATTCCAACTGCTA

>M.01.2.1_C_40

TTTATGAATATAAAAGATCTAGAAAAGATGGACAT

>M.01.2.1_C_41

ACTACTTTTCTATCCTGTATTTTAAAGTAACAAATGCT

>M.01.2.1_C_42

AGGAAGTCGACTCAACATCATTAACGAACTGTTCTACA

>M.01.2.1_C_43

AGGAATCTCCTATCTTCTTTAAATAGATATAGGAAATC

>M.01.2.1_C_44

TCCATGACGATCATATTACCCTCAATAGTTACTTTC

>M.01.2.1_C_45

TCACTAATATCTTTCTCGTTTCTTATCTCGTATATA

>M.01.2.1_C_46

ATAATACTACGTGCACTATTTTAAAAGATATAGAA

>M.01.2.1_C_47

CAGAAGTATATGTAAATAAAGACTATGACGTAGATGTGA

>M.01.2.1_C_48

CCTTCTGCTTCACCGTAGTCAAACGGGCTATCGTCAT

>M.01.2.1_C_49

CTATCGCCTTTATATGAAAGTAGGGGTACGTGAGGA

>M.01.2.1_C_50

ACAACGCCCCTCCTAATATTAGCAAAAACGTGATATCGC

>M.01.2.1_C_51

TCTATCTCAATATTATCAATATCTAGCCTTAACTCATT

>M.01.2.1_C_52

GAACAGCTAATCAGATATCATTTTTCACGCAAAAAATTCAA

>M.01.2.1_C_53

AAATGCCCAAACTGGCAACCTTCCGCTAATAACAAGT

>M.01.2.1_C_54

TCATCCTCATCATAAAGATATAGCGGATCATCGCATTC

>M.01.2.1_C_55

TTAATTTGCAGAGCAGGGCATAGGGTGTGGCCTCCTGGCC

>M.01.2.1_C_56

TGGACCCACTAGAATTATAATAAAACCAGAAGAAAGCG

>M.01.2.1_C_57

AAGTATTCCAAGATGCTTGAGGAGTGGAGAAATCAAGTT

>M.01.2.1_C_58

AAGACCATTACCTTTACGGATATGAAATTGATGATATCGCT

>M.01.2.1_C_59

ATATTAAAATTTATTTGCTTAAGTATTGAGACTATTTC

>M.01.2.1_C_60

ATTTAGAAATAAGTTATGAAGCTTATCTTAGCTCAAATT

>M.01.2.1_C_61

CTTTATACTACAAAAATAGTATTTATAATGGTCATTAT

>M.01.2.1_C_62

GTGTACTGTCCATATGTAAAAATTAGAAAATAAAATAAA

>M.01.2.1_C_63

TTTAAATTGGATATAGGGAGTTTGTGTAACTAAAAG

>M.01.2.1_C_64

GCAAGGCAGTGGACAATATGCTCTAGGGACAGAATATACGC

>M.01.2.1_C_65

TCTTATAGCTACTGAACCCAATGGTACTTTTACAATAAT

>M.01.2.1_C_66

ATTTGAAAGTGTAATTCTTCTATAACGTCTTGTTCCAT

>M.01.2.1_C_67

TCTTCATTTATCTCAATTTTGACATGTAGATATGTTCCG

>M.01.2.1_C_68

TGAATAAATGTAAATTACTGGAGTATAATTCACCACTTCG

>M.01.2.1_C_69

ATATTCTATCATAACTCAGCAGAATTAGTTAATAGTAAT

>M.01.2.1_C_70

TATATGTTCATTGAAATCACACCAGAAAGTTAAACCGA

>M.01.2.1_C_71

TGTGATAGAGGGGTATAGTGCAAATCAGATACAACAGT

>M.01.2.1_C_72

CACATTTCTCAAGCGCCGCAGCAGGTTATCCATTAAT

>M.01.2.1_C_73

TTCTTTTCTTTCCTTTACAATATAACCTCTTGCTATT

>M.01.2.1_C_74

TTTACCTCATTCCTTGCGTAAAGCTGGTAAGATATAGC

>M.01.2.1_C_75

AGGTTTTTAGCACTTCATCAATTCTTATATTGTCTGCA

>M.01.2.1_C_76

TTTTACAAAAAATAAATTCTAATGATGCATGGAAAGATAT

>M.01.2.1_C_77

TCCTAAAGAGGCACCTCTAGTACTTTTATATGATCGC

>M.01.2.1_C_78

AAATAAAAGAAAAAATCAATCTAACTTTGAATACTCAA

>M.01.2.1_C_79

TGTGATAATTAAAAAGGAGACTTTACGTTGAGGGAATG

>M.01.2.1_C_80

CACCACATTAGGCTATTTGCTCTTAGCAGCATAAACTA

>M.01.2.1_C_81

AAAAAATAATTGAAAGTATAGTTATAGACCATATAAGAGAG

>M.01.2.1_C_82

CGTTAAACTTTTACTAACTTACAAACTTAC

>M.01.2.1_C_83

ACTAATTTCTTTTCCTCCTCTAACCTTTTCTTAACGTCA

>M.01.2.1_C_84

AGTGTTAATCAAGGGCTAATTTCACTATTAACTGATTT

>M.01.2.1_C_85

TCAAATAAAGTAAATTTACCATCGTTTGTATACAATCTT

>M.01.2.1_C_86

AACTTTTATCACGACTAACGTGACCGCAAATGTTGACCA

>M.01.2.1_C_87

TTATCAGCTGGTGAGGTGACCCACCCGTGAAAGCCT

>M.01.2.1_C_88

ATTATTTGCTTAGCTTCTTCTTTACTGGAGAATTTGAC

>M.01.2.1_C_89

TTATCTTCGTGGGTTTGGGACATTTTTGATTCACCTAT

>M.01.2.1_C_90

CTAATTTACCGAATTCTTTCACATACGTTTCTACACGC

>M.01.2.1_C_91

TCTATGCTGTAGTGAGTGTAGCCCTCCACCAATATCT

>M.01.2.1_C_92

ATAAGGTAAGATTACATAAGATATTGAACTAGAATTAGCATT

>M.01.2.1_C_93

AAATTACAAAATATCAGTTTCACTCGTTCCACTACT

>M.01.2.1_C_94

AGTAATATGCATTACTTATAATTAGAATAGGAAATCTTG

>M.01.2.1_C_95

ATCCCCCAAAAATATCTATAAAACATCTATTATATGACC

>M.01.2.1_C_96

TTATACAAATTATATTCTTCAGACTGAAACAATGCATAAGCC

>M.01.2.1_C_97

CCTCTTTCCGCTATCTTCTCATAAATTTTGTTTACATC

>M.01.2.1_C_98

ATGTCTAATGTCGAGCAAAATTCTAGCTGATCGATC

>M.01.2.1_C_99

AAAAGAACTAATAGTTCTGATTTTGCACACCCCATG

>M.01.2.1_C_100

TGAGACCGCGAATAACTGACCTAGCGATTGAAACTAGAGCG

>M.01.2.1_C_101

ATATTTGTCAATTGCATTACTTTTAAGAACATTACATAC

>M.01.2.1_C_102

CTGTGCTTTATTGGTCTCACTCTTTCTACGTGAATCTTCACG

>M.01.2.1_C_103

TTTTGTAACTCAGATAATATTTTTGCATCTGCACTATC

>M.01.2.1_C_104

TATATGTACTGTTTTGCCCAAATTGTATGGTGAACGCT

>M.01.2.1_C_105

TACTGTGTATTATAAATGTAAGCATCAGAAAGACGTTTAA

>M.01.2.1_C_106

AATGATTTTGAGATTATGCTAAATCCATCGAGTACTG

>M.01.2.1_C_107

GATTTGCTCATCGTATTTATTATAACACAAAGCACTAT

>M.01.2.1_C_108

TTGCAGATGTTGTAACGTGGGATAACAAATTATTTTCCCC

>M.01.2.1_C_109

TATTTTTTTAACTTTGCTGGAATATATTGAATGTAGTCT

>M.01.2.1_C_110

GTATTATCCGGATAAAGATAGAATACTTTTCTTAATAG

>M.01.2.1_C_111

ACATTAGAAGAGTGGCAGATGTACAAAGGGAAAACTGTA

>M.01.2.1_C_112

ACAATACAAAACTCTTCTATCTTTCTACCACTGACTCTC

>M.01.2.1_C_113

AGTAACTCTGGTGTTTGCCTTATCCAAACGTTTGTTACG

>M.01.2.1_C_114

CGCCCTATGCTCAATTTAATTTATCTGCTGGTTACTAC

>M.01.2.1_C_115

GTTTATTGTTTCATGGCATTACAATCAATAACAAAT

>M.01.2.1_C_116

ACTAATATCGCTTCAACGTCACCATATATAACTTTATAC

>M.01.2.1_C_117

TTCAAGATGTAAACGCATCTTTATTGCATTCGGAGAAATAGCA

>M.01.2.1_C_118

TTCGTTAATAAGGAGACCGCTGAAAGCATTTCTTTCCCCG

>M.01.2.1_C_119

CGAATGTTACGCTTACATATGATATTGTAGATATA

>M.01.2.1_C_120

ATATTCCTCTAACGCAAACGGCGTTAAAACACTTGCTTTC

>M.01.2.1_C_121

TATTTAGAGGTGAATTGCAAGGTGTTGTTGATGGCATTG

>M.01.2.1_C_122

CCATCTACTTCTAGACCAATCTCATCACTATTAAAATA

>M.01.2.1_C_123

TCTAAAATATATGCAATAAGGGGGGATCAAATATATCCCC

>M.01.2.1_C_124

GATAGAAACCCCTTGATGCTATTCTTAATTCCGTCAGA

>M.01.2.1_C_125

ACTTACGCTCTGAAAGAAGTAGTTTTTTTGCTTCTTCAC

>M.01.2.1_C_126

ATAGAAATAAAAGGATATGCTTCCCGTTACTGTTAGA

>M.01.2.1_C_127

GATTGATACTCTATGAAGTTATTCTTTGTTATATATA

>M.01.2.1_C_128

ATGATGGGTCACCTCGTGATATTAAGGTAAATATCAACT

>M.01.2.1_C_129

TTATAGTCTTTATATTGTTCTTCTGTTAAATTGTTAAGATC

>M.01.2.1_C_130

TATAAATTGGCAAGGGTGGAATGTAACGATATACC

>M.01.2.1_C_131

TCTTTCACATACGTTTCTATACGCTTCTTCTTTCCATC

>M.01.2.1_C_132

AAATAAATATCATATAGATAATTATATTCAGCCTCGT

>M.01.2.1_C_133

TCAAATAGTGCATCAGATATTAAGAGACTTCTAACTTTAC

>M.01.2.1_C_134

GGGAATGATGTTACGTGAATCGTAACGTTAATAATGTTTG

>M.01.2.1_C_135

ATTATATAATGCGTAAGCAGATGCAGATATAGTTATAACAT

>M.01.2.1_C_136

ACAGAGGTAATGTATTTAGGCTTACGCCCTAGCTTCCAGCGT

>M.01.2.1_C_137

CAGAACCTCCTCCTAGCTTCACCGTGCAGGTTCTCAAG

>M.01.2.1_C_138

TTTTTTTCCAGCTCTTTAATGATCTCATTAACACT

>M.01.2.1_C_139

ACATTTATAGCCACAATTACTGCAGTTTCTGTTTACCGTC

>M.01.2.1_C_140

TGAGTTTTTACAACAAACATGTCGATTAATTGTTTCTCT

>M.01.2.1_C_141

ATATAGATTTAAGAATGAGAATGAAAAACTTGAGGCAG

>M.01.2.1_C_142

TGTCATAATTATCAATAACCCATTCAGTTATTGCATAAT

>M.01.2.1_C_143

TCGGTGATGATTATCCCTATGTTACTCACCTTACTATCA

>M.01.2.1_C_144

GTACACTAGATGCCGATATCGGTATTGACATGTTCACGCC

>M.01.2.1_C_145

ACATTAACATTATTGCTTTTTATCCAAGGATATTTCCA

>M.01.2.1_C_146

ATAATCCATTGAGTTATTGCATAATATCCTTGTCCGCT

>M.01.3.2_C_1

TACTTTAGTCTTATGAGATTTTTAATGGCTCTTATACT

>M.01.3.2_C_2

ATGGATAACCTGCTGCGGCGCTTGAGAAATGTGAGAA

>M.01.3.2_C_3

AAATATTAAAGTTTTGCATTCTTTGTACGCGTCACCAA

>M.01.3.2_C_4

GTTATTCGCGGTCTCATTATAAAATTCAAAGCATATTCTAA

>M.01.3.2_C_5

AAATATGAGAGATTCACATCTTTTATAATCTTCACC

>M.01.3.2_C_6

CAGATCTATTTACTGGAGCAGGAGCTGCAGGGAGAGAGCTA

>M.01.3.2_C_7

ATAGGATAGAAGAACTTCCTAACGAAATCTTCATGCCTTT

>M.01.3.2_C_8

ATGAAAGGTTTTACGTTAAAATCACTTCCGCCCGGCA

>M.01.3.2_C_9

TTATAATCTTTATATTGTTCTTCTGTTAAATTGTTAAGATC

>M.01.3.2_C_10

TTAAAACATTAATTCTTTTGCTAGAGTTAAATGTTATTT

>M.01.3.2_C_11

CTAACTAAAACACAACTAAATATAACACTATATAACACAC

>M.01.3.2_C_12

TATTTAGCAATCAACTTCCCACTATCATATTTAAATAT

>M.01.3.2_C_13

ATTTGTTGTACCAAATCCGATGGTGCGCCTTGTTGTTCTA

>M.01.3.2_C_14

TTGATGGGTCACCTCGTGATATTAAGGTAAATATCAACT

>M.01.3.2_C_15

CTTTAGATTAGCTAGTTTTAATACGATGTAGGACAT

>M.01.3.2_C_16

ATATATACAGAATAACGGTATTAAAGATGCTGACTATACGC

>M.01.3.2_C_17

ATTACTATTTGACGTTACACCTTCCTTCCAAAACGTA

>M.01.3.2_C_18

CTTTATTTAGGTACTCCCCTTGCTTTAAATTGTCCTTT

>M.01.3.2_C_19

AAATTAATCTCTAGAATCTCAGATAGTGGCACATATCTT

>M.01.3.2_C_20

AAAGTTTCAACGATACAATTTAAACTTTGAGCAATTAAT

>M.01.3.2_C_21

AATAGTGCATCAGATATTAAGAGACTTCTAACTTTAC

>M.01.3.2_C_22

TATACCTTATGCTGATGTCTCTGAGCTTCCTGATATCA

>M.01.3.2_C_23

TAGACAGGTCAGTATAGGTCTTACCATCTAATAAAGCAAG

>M.01.3.2_C_24

ATAAGGTAAGATTACATAAGATATTGAACTAGAATTAGCATT

>M.01.3.2_C_25

TTTGCAATATAGTATGGTTGAATACCCCAACCGTACAAA

>M.01.3.2_C_26

TTAATGAAAACATCTCTGTCAACTCTAATTAAATTCAG

>M.01.3.2_C_27

GTTAAAAGCGTAACTAGATGATTGGGTTGGAACGTATAT

>M.01.3.2_C_28

AGAGGGAGCCTTCGGGGTGTGGCTTCCCCCCTCCCATTTTCC

>M.01.3.2_C_29

TGTTCAATCACTTGTATTCTTTTGTGTAAATTTCGC

>M.01.3.2_C_30

TTTTGCCTGCTGGGTTGTAAAAAACAACAGGTGTAAAGAC

>M.01.3.2_C_31

ACAACAGGTGGGACGTATTCCTTTCCCTCCCTTTCAGC

>M.01.3.2_C_32

ATGCTATTCCAAGGTGACTAGATATGATAGAGATGAAG

>M.01.3.2_C_33

TACGATAGACGGATAGAAGTATTCCCTTAATATCTTCTC

>M.01.3.2_C_34

ATACCAGTATCAGAATATGACGCGCCAGAACCAGAAC

>M.01.3.2_C_35

ACTTATCAACCAATTGAATATTTGAATCATTACTAGG

>M.01.3.2_C_36

GTATTGACATGTTCACGCCTTTTATGAAGCCTGACAAA

>M.01.3.2_C_37

ATATTAAAAGGATGACGTTTTGTCATTCTCCCTGATTCTA

>M.01.3.2_C_38

AATTCATGATCAACGTAGGTTTCTACAGCGAGTTCAAGAA

>M.01.3.2_C_39

ATTATCTTCTCTGCATATAACATAAAGGCAAAGACTATG

>M.01.3.2_C_40

TAATTAAATCTAGATAACGAAGGTATTTAACATTTTGT

>M.01.3.2_C_41

ATAAACGATAGGCTTCAGTCCCTCAAAGCGAGAAACGA

>M.01.3.2_C_42

AGCTAGAATTAACCATTTATAGCCCCAGGGAATATCTTC

>M.01.3.2_C_43

CACACACACACCCCCATATTAAATCCGAAAGTACGTTAGT

>M.01.3.2_C_44

AGTATTACTTTGTACGAAAACGGGGCTGCAAACATTAG

>M.01.3.2_C_45

TTTCCAGTGCGGAAACTTATCAAGACCGTGTCACTG

>M.01.3.2_C_46

CAAAATCATTAGATAACTTTGCTCTTCAAATTGAAAACTT

>M.01.3.2_C_47

TTTTTTCCACCTCGCTAGAAAACTCCCTACCCTGATTTGC

>M.01.3.2_C_48

AAATTACTATCTTGAATAGAGATATTAGCGTTGTCTATTGCT

>M.01.3.2_C_49

ATTTGTTTAAAAACTTGTGGTTCAATTGCGAAAACAAC

>M.01.3.2_C_50

TTGCTATAACAAACCTAGGATTTTTGACTACACTTTCAG

>M.01.3.2_C_51

GCAAGGCAGTGGACAATATGCTCTAGGGACAGAATATACGC

>M.01.3.2_C_52

AGATTTAACAGAATAGTAGATATTACCAGGCTGTATTATT

>M.01.3.2_C_53

TAGATGACCCTGCGAGTTGGATATCGAAATGGCAAAAT

>M.01.3.2_C_54

TTCTGTTAGCCTGCCTCCTTCATAGGCTAAAATAACAG

>M.01.3.2_C_55

TGACCTACACTCAAATCCGAATTGTTTACATTCCTCAA

>M.01.3.2_C_56

ACTATCATTCCTTGTTTATAAAGTCCAAAAGCATATAT

>M.01.3.2_C_57

GTAATTAAATATCGTGAATTTTCAGTAATTATTAACTT

>M.01.3.2_C_58

TTTAATCTTATTTCTTATGTCTATGCCTTCTTCACGT

>M.01.3.2_C_59

TCAACAGATGCAACCTTATCCCCAACCTAGTAATGATT

>M.01.3.2_C_60

ATAATTTGTATCGATGCTTTTCCATTATCATCATATGAAAT

>M.01.3.2_C_61

TATTTACTAGCATAGTTTCCAAATACAGGCTCGA

>M.01.3.2_C_62

CTTTCACTCTTCTTCTTTATCTCATCAAGTTTATTGT

>M.01.3.2_C_63

TATTTCACTTCTTAGCAACTGCAATGACTATCACAAGT

>M.01.3.2_C_64

ATTCTTCTATGTACTTGTGCTAGCATGCGAAACAAACG

>M.01.3.2_C_65

AATAAATTCATTACTTTCCTTAGATCTCCATCTGATAAAT

>M.01.3.2_C_66

GGTGTGAATATGAACGAATTAGAAACATATGTTAACAACA

>M.01.3.2_C_67

TTTATGAATATAAAAGATCTAGAAAAGATGGACAT

>M.01.3.2_C_68

CAAGGTGTCGCTGCTGCGCAACCCAAGATGGCTGCAGTA

>M.01.3.2_C_69

TTTCACTTCATTTGTAAGATATGCTACAGTTTGTAAATTG

>M.01.3.2_C_70

AAGTAAAGAATAGCAAATATAATCCTACCCATTCCCGCTCA

>M.01.3.2_C_71

ACATTAACATTATTGCTTTTTATCCAAGGATATTTCCA

>M.01.3.2_C_72

TAGAAAACGAACCACCGTCCGCAATGTGGGCATTGAT

>M.01.3.2_C_73

TTGTAATACTTCTATTTTCATATTTATACACCCGAAAA

>M.01.3.2_C_74

TATTAATGATCTTGAATTCAGTAATTGCAATGAGATCAT

>M.01.3.2_C_75

TTGTTACTCAAATATATTCACCTCCATCTTTTGT

>M.02.0.20_C_1

TTCTACACTTTCAGAGAACATAGTTTCTTTATTTACA

>M.02.0.20_C_2

GACTACAATTGGCAAAGATCATTCAGTTGGTAGTATAC

>M.02.0.20_C_3

TCTTTTAGTGTTGAAAGATTTGCTTTATCATTTAAAAAT

>M.02.0.20_C_4

TAAGCTTATCAGATATTCTACAGCTTTGTCAAATTCTTT

>M.02.0.20_C_5

TCTTGTAGAAACTTTATCCACTTTACTTTTTCTTCGC

>M.02.0.20_C_6

GTTAAGCTCTATGTCCTGCAGGATAATACTTTTG

>M.02.0.20_C_7

TATTACTCTTAGCAGCTTGCTCATAATTTATACTAA

>M.02.0.20_C_8

TCGTATATCTGTACAATCTTCAATTCGTTAAGTAGTTTC

>M.02.0.20_C_9

ATATTTTCAACTCTATAGCCGTTTTTCATGCATTCTTCTGAT

>M.02.0.20_C_10

CAATAATGTTTATGTCTTTTGTTTGCCTAAATTCTT

>M.02.0.20_C_11

CACTACTTGTTGTATATGCGTAACTATTAGAATTGATATT

>M.02.0.20_C_12

ATAACTCTACTACATTTTCTCATGTAAATTCTAGTGCAT

>M.02.0.20_C_13

TATTTTGCCCAATAGGTCTTTCGGAGTTATACCTTTCT

>M.02.0.20_C_14

TTACTATTTTCTTAAGATCTGGCTTATGTACGTAGT

>M.02.0.20_C_15

TTCATGAAAACATCTCTGTCAAGTCTTTTTAATCCTAAA

>M.02.0.20_C_16

CTTGAATAAGCACTTATAGAATTTTTATTAATAGCTTCAG

>M.02.0.20_C_17

ATAGTCACGTATATTGGATACCCAAGCTTCTCATAATTA

>M.02.0.20_C_18

ATAAGTTTAACGCCTAGGGTACTCCCTGCGTAAATTC

>M.02.0.20_C_19

TTAAGTCCTTTGATGTGACAGATTATATCAAAATTGAT

>M.02.0.20_C_20

TCTTGTCTAGTTCATCTAACAATTTTAATAGCGTCTCGA

>M.02.0.20_C_21

TAGACAGGTCAGTATAGGTCTTACCATCTAATAAAGCAAG

>M.02.0.20_C_22

TTTTTCTTTCACCTCGATGTAAATATTTACTTCTTCAA

>M.02.0.20_C_23

TTGCTAATGATTCAACGTTAGTCTCTACTATTTCTT

>M.02.0.20_C_24

TTATAGTCTTTATATTGTTCTTCTGTTAAATTGTTAAGATC

>M.02.0.20_C_25

TTTAATCTTATTTCTTATGTCTATGCCTTCTTCACGT

>M.02.0.20_C_26

TTAGAACAAATCGTAAATGAGTATTATGATATGAAGTGCC

>M.02.0.20_C_27

ACTTTATACTGTAGATATTTACCGGGTAGGACAAAGTAT

>M.02.0.20_C_28

ATATATAGGTTCCATGTCATTGCTCCCCAACCATAATC

>M.02.0.20_C_29

AAATCAATATATAGTGCTTCAGATAGTGGCACATAT

>M.02.0.20_C_30

ATAGATAGAAATTACTCTCTACTGGATCCGCAACTTC

>M.02.0.20_C_31

ACATCAGCCATTTTACTCACCTAGTTTGTACAATCCT

>M.02.0.20_C_32

TTAGGAAGAAGGGGGACTACTGTAGTGTGTGTTACAATA

>M.02.0.20_C_33

TATTCATATCTGCCAATGAGTAATGTATACTTCTAT

>M.02.0.20_C_34

CCGCCCTACTTGTTTTTATATTAAATGCTATTCCC

>M.02.0.20_C_35

ATTAAGATAATAATATCCTACAGAAATATTTACTGGGGCGT

>M.02.0.20_C_36

TTATTATTTACAGTTATTCTTATCTTTGCCATCCTCACCAC

>M.02.0.20_C_37

TTAGTATATCATATGCTTCCTTCATCATGTCAATATC

>M.02.0.20_C_38

ATTGTTAAATCAGTTATTCTCGGTCTCATTATAAAATTCA

>M.02.0.20_C_39

CCCTTGTATTTTAAAATCCCACAGCCTATTTACCTCAC

>M.02.0.20_C_40

AGGATCTCAAGACATATGGTAACAAGTTTGTTTTCTT

>M.02.0.20_C_41

TCGTGAGGGCAGAGATAGCAAAGAAGTCAAAGTATAATGC

>M.02.0.20_C_42

AGCTACATTTAATGCTCTATTATGCATATGCCTCATAAC

>M.02.0.20_C_43

TCTTTCACATACGTTTCTATACGCTTCTTCTTTCCATC

>M.02.0.20_C_44

ATATTCTTGCAATTGCTTCTAAAGTTCCAATGCTTATTG

>M.02.0.20_C_45

ACCATATCTTTCCATGCATCAGTTTCTTGACTTCCATAC

>M.02.0.20_C_46

CTAAACCTATCAGAGTTAGCAGAATATAGTAATATTGATATT

>M.02.0.20_C_47

TGATAATCTTCAACCTTAATAAATTCGAAATAAATATC

>M.02.0.20_C_48

ATATTTTTAAGCTTCTTAGTAAAAGAAGGATATCTT

>M.02.0.20_C_49

TCAAAATGAGTAATTTTTCTTCATGCAACATAATTCAC

>M.02.0.20_C_50

GTTACAAAATTAGAATACTATTTAACAAACTTATCAAA

>M.02.0.20_C_51

ATGCTTTCGTTTTGTGTTTTTTCATTTACTTGTTCTT

>M.02.0.20_C_52

AATAATAGTTATAGTCGCCTTATCTGGAGGGAAGAAAC

>M.02.0.20_C_53

TTTAGATTAGCTAGTTTTAATACGATGTAGGACATGAG

>M.02.0.20_C_54

TTAATTAACTGCTTTATCAACCTCCTTATTGCCTCACTCCTCG

>M.02.0.20_C_55

ATATATTTCTTTACTAGTTGCTGTGACGATTTAACGCTGCT

>M.02.0.20_C_56

ATTTCGTACATCAAGTATTTTGATGACAGTAAGAAATATTG

>M.02.0.20_C_57

ATTTAAATTATATTTTAGGGCGTATCTGTCTAGCAGTTGTA

>M.02.0.20_C_58

AATCATATACTGTATCGTTCGTTACAATCACGTTATAT

>M.02.0.20_C_59

TCTCATCTTTAGCAATAAATCATGAGTTACTGGAA

>M.02.0.20_C_60

TTAGCTAGATCAGCTGCTTCAGCTTCTCTCAGCGCTCTC

>M.02.0.20_C_61

ATTACACTTAAGATTGTTGCTTTTTTACTATTCTG

>M.02.0.20_C_62

ATTTTTATCGCCCCGTCAATCATCAGATCTTTAACG

>M.02.0.20_C_63

GAAGTTAGAATAGCTAGTACTCCATGAAACTAAATC

>M.02.0.20_C_64

GAAAGTTCTTTTTGCAGACGTTCGCCTAATCGAATAATAT

>M.02.0.20_C_65

ATCAGTATTGGGAAGTAGATTGCTTTGCCTTTATTCTCT

>M.02.0.20_C_66

GTAAAATACCAGGTTCCCTACTATAATCGTTACTGCC

>M.02.0.20_C_67

ATAATTTTTACTGTTATGTATACGTTTCCGCTATTACTT

>M.02.0.20_C_68

ACGTTCAACTTTAGCGATTCATCTACTTTCTCTTCATT

>M.02.0.20_C_69

AGGTAACTTCTAAGATAGTAAGGTAATGCTAAACGAACCAT

>M.02.0.20_C_70

TTTAATTCTTTAATATAAGTAAGCTGTTCTACATAGAA

>M.02.0.20_C_71

CTAGTTATCCCTGCCCTACCGGTAAGTCTAACGAATATA

>M.02.0.20_C_72

TTGTTACTCAAATATATTCACCTCCATCTTTTGT

>M.02.0.20_C_73

TTCAAATACCCTAATTGTTCTCTCTACCTCATTTAGATC

>M.02.0.20_C_74

AATTCCTTTATCTTTGCTTCCAGCTCTTCTATCTTCTTT

>M.02.0.20_C_75

CATATATAGTGAATTTCTTTTGAGATAATAGATTAT

>M.02.0.20_C_76

GCCTTGCGGAATAAGCTGATATGCGATAGCCCCACCAG

>M.02.0.37_C_1

TTATAGATCCAGCCCTAGATCTAGTTTCTTTTATCTCT

>M.02.0.37_C_2

ATATAATAGATATCCTATTATTGCTATGATGATGAT

>M.02.0.37_C_3

AGAAGAATATGTAGAACCTATTCTTTCTCTCTATGATCAC

>M.02.0.37_C_4

ATTTTTGATTATTTCCTTGACATCCTCCTTGATATCTTG

>M.02.0.37_C_5

GTAGGTGTCAGTACTGGAAACAGATCGTCGAACCTTCCTG

>M.02.0.37_C_6

ATAAATCTACATTATTATACAAAACTTGTAAAATTGTATTT

>M.02.0.37_C_7

CTAATACTAATGTGTATGGCTCTTTTTTCGCTATATTCG

>M.02.0.37_C_8

TCAGTATGAGGGTCTTCAGTAACGTCGTCTTTCCCG

>M.02.0.37_C_9

CGCACTTGCAGCAATTAGAGAATTCTACGATGCCCCT

>M.02.0.37_C_10

TTGAGAACCTTTCCTTCTTCAAGCCTCACTGCTATATTA

>M.02.0.37_C_11

TTATACTTTTTCTAATTGCTTTCTGAATCTTTCTTTTCT

>M.02.0.37_C_12

ATATAATGAAGTATACGGATATACTGCATCAAATTTAT

>M.02.0.37_C_13

TATTAAAGCATATTTTTCTATCCGTATTAGTAT

>M.02.0.37_C_14

GTTCACAAAGAGGCAAAGCAGTTCTAGGTTACACGGA

>M.02.0.37_C_15

TAGGTGCAGGCGCAGTAGCGATATATTTAATATCTA

>M.02.0.37_C_16

TGAATTATAAAACTCACAATTGCACCTCTGTACCTGATAATT

>M.02.0.37_C_17

ATAGAGTTGCAAGCCCGAAGGCTCCCTCTCGACCTCCCTCG

>M.02.0.37_C_18

CTACTTTATACTGTAGATATTTACTGGGTAGGACAAAGTAT

>M.02.0.37_C_19

ATTCAATATTATGCATTTCCCCTTACGTCCCCTCAAGT

>M.02.0.37_C_20

GTGGAAACTCAGTTTACGGCGAATACGAAATGGAC

>M.02.0.37_C_21

ATATAGAGACATTTATAGATCTGCTAGATATTATCTATCGT

>M.02.0.37_C_22

GTGTAATAAAATATCTATATAGTAAATTAAAACATCAT

>M.02.0.37_C_23

GATAAGCTACCACAAAACATTCTAAATGAACTGAAAAA

>M.02.0.37_C_24

TTATATACATTAGTTGTAGTATTGACGATTAGTTGTACTGAG

>M.02.0.37_C_25

CTGGTGTGATGTTAGTTATTAAATATGATAGCGGT

>M.02.0.37_C_26

TACTGCTGTATGGAAAAGGTGGGGGGACATAAACCCTAGT

>M.02.0.37_C_27

TTATAATCTTTATATTGTTCTTCTGTTAAATTGTTAAGATC

>M.02.0.37_C_28

TACAGGGGTCATACTTGCTAGATTTATTACAACATCTA

>M.02.0.37_C_29

TATATCATACTCTTTAGCTTTGCCATTAATATCTCAA

>M.02.0.37_C_30

GTGAATTACGATACTGATGCACCGGAAGATTTCAGAGCTGAGG

>M.02.0.37_C_31

ATAACATCTCTAAAAATTGTATATGTTTCTTCCGTG

>M.02.0.37_C_32

TTTTTATCCTTCCTTCTTGTTCTTCTATTTGCTTTTC

>M.02.0.37_C_33

ATTTTGCTCGCTACTTTTCCTGCGATACCGGGTCCTAAA

>M.02.0.37_C_34

ATTTTACTAGATGGTATTTTTATAGATGATTCACTTTT

>M.02.0.37_C_35

CTGCATATTTTAGCTGCTTCCGCAAACTCTTTCCTCCACT

>M.02.0.37_C_36

TTAGATAATCTACTTAATTGGATATATAGTAAATTAGTAT

>M.02.0.37_C_37

CACTTGTCTAAATAATGTAACAAACCTTAGACTACAAT

>M.02.0.37_C_38

ATACTATATTGCCAAAATATGTCATTAGACTTTTTGGC

>M.02.0.37_C_39

CCATCACAGAACCATTGCCATACCCAATCACCGGACATA

>M.02.0.37_C_40

TTATTTACCGCCTACCCCCTGTGATGTCAATTCAT

>M.02.0.37_C_41

AGCTCTACTGGACTTAGTGGCTCAATGCTATTCTGGG

>M.02.0.37_C_42

ATTTCATTAATTCTTTCTGTACGTTCTCTTTTTCATCATAC

>M.02.0.37_C_43

AACTTTTCCAATTAACCCCGCATAATCGATTCCTAATTC

>M.02.0.37_C_44

ATAATTAATGAACTTGTAATTTTTAAACTTGCCGTACCTCCCG

>M.02.0.37_C_45

GGGGAGAAGATGGATAAAGTAGTTAAAAAACAATTGGAATTAT

>M.02.0.37_C_46

AGACGATCCTTACTATAACGCGTTACAAATGCTAGTAA

>M.02.0.37_C_47

ATACCATAATAATTATCAACTATATTCTGCCATAACTTAAT

>M.02.0.37_C_48

TCAGTATCTTTATAACCACCACTTACCAATCTTCTT

>M.02.0.37_C_49

CTACATTTATAATATGTCTCACTTTTAAATACATCATA

>M.02.0.37_C_50

TCATATTATTACTACTTGCGGTAACAGGGCTAAACAA

>M.02.0.37_C_51

TTATTTATATAATCATTGAAACATTCGATATAATAATCTT

>M.02.0.37_C_52

TTGAGCAGACCCCCGAGGGTGGGGAGGTTTTATGTTGTC

>M.02.0.37_C_53

ATAGTAACGACATCAATACTTATCATCGTCCACATT

>M.02.0.37_C_54

AGTTAGGCTATCTGCGTGAAACTGCTACTATAACTATT

>M.02.0.37_C_55

CCTAACACCGCAGCACTTAATTTAGGGTCTGCAATTGCT

>M.02.0.37_C_56

TTTCAACTCTGCTATCCTATTTTCATATTCGTTT

>M.02.0.37_C_57

CTGCTATTAGTAATATCAAATTATAGACCTCTTTTC

>M.02.0.37_C_58

GATAACGGAATAACGGGTGTTTATGTTCTTTTAGTCACATT

>M.02.0.37_C_59

CTTAACATATCAATTACTTCCCCATCCTGTGGACACTT

>M.02.0.37_C_60

AAAAGATAACTATAAGCATTTTTTACATCTGAAAATAACGCA

>M.02.0.37_C_61

TGAAGTAACTGAATAGTTCTAAGCACTGCATAAGTAGTT

>M.02.0.37_C_62

AATCTATCACCACCATTTACTGTCACAATTGATTGGG

>M.02.0.37_C_63

ATAACTTATACTTCATACCGTATTACACACATATAAT

>M.02.0.37_C_64

ATATAGAGATAATCTGAAATGGAACATACCTACATATGGTCAAG

>M.02.0.37_C_65

TAATAGAATTAGGATTACCGATGTCCTTTGTTATGC

>M.02.0.37_C_66

ACAAGAACTATAGTAGTTCAGCAAAATCCGAAGCTT

>M.02.0.37_C_67

TCTTTATCTAGTAAACTAATTAAAAAAAGAAGATCTTCC

>M.02.0.37_C_68

AGAATACTATATAATTCTGCGGGTATTACTGCATTATTCC

>M.02.0.37_C_69

AACATAGCGAACTATCAAGTGTTGAATTATATTGTATTCC

>M.02.0.37_C_70

AGTACCTCATGTAACTCTTTCGGCGTAGCTTTTCCTTC

>M.02.0.37_C_71

AGTTTTTTATTAGGTAAAATTTTTACACTCATTTT

>M.02.0.37_C_72

CTTAAATTATTAAAGCTTGAATATAATATTCTTGCAATAG

>M.02.0.37_C_73

TTATTTCCTTCACTTTCCTCTCCCTCATTTTCATTACT

>M.02.0.37_C_74

ATAATGACTTTCCTCTGTACATTTCAAACTCTTCAACA

>M.02.0.37_C_75

AAAGAAGCATCATGGAAGTTGCTGGCGTTAGTAGCTC

>M.02.0.37_C_76

TTGATATAATACGTCACATCAATCGTTCTTCTTCTCGAT

>M.02.0.37_C_77

CTGTACTGATTTCACAACTTTCCAACGGCTCTTATGC

>M.02.0.37_C_78

AAGAAAAAATGAAAGACAGTGTACTCGAGGAAGTCC

>M.02.0.37_C_79

CTAATATAAACGTAATAATCGTCTGGTTTTTCATTAGTTTG

>M.02.0.37_C_80

GCAATGTTATCAAGATGATAGAACTTATTATTTATTTCAT

>M.02.0.37_C_81

AACTTATTATGTATTGTTACCATGTGTACATTTCTAGC

>M.02.0.37_C_82

GATGAAGTTTGGAAGCAGGGTGTAGCTAGTCTTGG

>M.02.0.37_C_83

AGAGAAGAAATGTACGTCTTAAATAATATATGCAAAT

>M.02.0.37_C_84

GTAATCGCTGGGATTCCTCATTATCAGATAATCGT

>M.02.0.37_C_85

CATTCTTAAAAATATATGCCAATATCTTATATAATTCT

>M.02.0.37_C_86

TTATTGTTTTCCTTATGCTCTTCTGAACTTTCCTCT

>M.02.0.37_C_87

ATAAACTCGTTCTAGGAAAAGAACCACCCTCAAACCTAA

>M.02.0.37_C_88

TATTTACCTAAAAAAAGGGTTGAAAGAAAGACTGCACT

>M.02.0.37_C_89

GCGGATTAACACCGGCACTTACGTTCACCCCTTGGCTT

>M.02.0.37_C_90

ACATTTATTTCACCTCCATTCTGTGTGATTGAATTTTC

>M.02.0.37_C_91

AATAGTCATTGATATACTTCTTTAAAAACCTCATTCCA

>M.02.0.37_C_92

TTATTTATAATTTTTTCGGGTGCTATAGAATGAAATTTAGAG

>M.02.0.37_C_93

ATGTATACCATGTCAATATAGCCGTTCTGGAACGGGTAG

>M.02.0.37_C_94

AGTTTTTTACATTATCATGAACAAGTAAACCGGAAAC

>M.02.0.37_C_95

AGTTTCTTAAAAGACTCAATATCGTTACATGCACTAATGT

>M.02.0.37_C_96

ATGAACTTTATCTTACCTTGGGATAGCAATTGCTTTATCTC

>M.02.0.37_C_97

AAACAAGAAGAAAAAGAAGTAGATGAGTTTTTTTTATTT

>M.02.1.6_C_1

ATTAATTCTTTTACTGTATAGAAATATCTCATTTT

>M.02.1.6_C_2

ACCTTACTTTCTCTTCTCCCTTTAGATTAAGTTTT

>M.02.1.6_C_3

TCAAAATGAGTAATTTTTCTTCATGCAACATAATTCAC

>M.02.1.6_C_4

GCTTGTTTCTGATTGATGATATACCTTATTATTACTGGT

>M.02.1.6_C_5

AAGTACACTTTATTAGCATTTTCTGGCAAACGATTA

>M.02.1.6_C_6

TATAAATAAGCGGTAAAAATGAAGAAAAAAACGTTTCTAT

>M.02.1.6_C_7

TTCCAAATACACGTTTATTCAATATGATATCCAGCAAC

>M.02.1.6_C_8

ACTTGTATATCAGATATCATGTCTACTAACGGCAAATT

>M.02.1.6_C_9

TCTATTAATATTGTATGCAACATTATTTTAACTTTGC

>M.02.1.6_C_10

TAGAATATCAAGCAGATAAATTAGTAGTACAAGATGTGA

>M.02.1.6_C_11

TCAAAATATATATCGATGATTTCTTCAAGATCACTT

>M.02.1.6_C_12

CTGACTGGTATCAAAGATAACGCTTTCTTAGGGTTCGTA

>M.02.1.6_C_13

AGTTTAATATACTCAACCCTAGCCTTTATCATTTCTTAAT

>M.02.1.6_C_14

GATGAATATGAAGAATTTGCTACTATAATAGTGAAACTCC

>M.02.1.6_C_15

TAATACTAAATGATACATGCAACTTACATGTATTATATCT

>M.02.1.6_C_16

TGGACACCCAACCACGCTTGAGGACTTGGATCATTTTC

>M.02.1.6_C_17

ACAAGATTCGTAACACTATTGACGAAGGTAAGAACGTTATTCTTG

>M.02.1.6_C_18

ACAATACCATGTGCAGAATCAATAAACAATATGCTGT

>M.02.1.6_C_19

TAGACAGGTCAGTATAGGTCTTACCATCTAATAAAGCAAG

>M.02.1.6_C_20

CTAAATATTTCGTGTTTATATCAAATGCATATACTTT

>M.02.1.6_C_21

TTATATGTAGTGACGGTAAGTAGTAGCATCTATAACTCT

>M.02.1.6_C_22

TCCGTTTCTAACTTATTAAAGTATTCATATAATGC

>M.02.1.6_C_23

TTATAGTCTTTATATTGTTCTTCTGTTAAATTGTTAAGATC

>M.02.1.6_C_24

CTATATATTCGAGAAACAGGAATTTTCCCTGACTTATTT

>M.02.1.6_C_25

TATAAGAACCTAAAAGAATTAATCTTTCAATTCCTTTTAGGATTAAT

>M.02.1.6_C_26

TTTTACAATCAGATATATTGATAGTTGATTTCTGCTA

>M.02.1.6_C_27

TTATTAATAATTTTTCGGGTGTTTTCATGTTTGTGCC

>M.02.1.6_C_28

GATTACTCTTCACAGATAAACATTCTTTAGGTAATTCT

>M.02.1.6_C_29

TCTTTCACATACGTTTCTATACGCTTCTTCTTTCCATC

>M.02.1.6_C_30

ATAGATATGATGAGATGGCAAACAATGTATACTAGGAAA

>M.02.1.6_C_31

TTTAAATTCCTTTTAACATCACATAATCTTTATTTCATATC

>M.02.1.6_C_32

TTATTATTTACAGTTATTCTTATCTTTGCCATCCTCACCAC

>M.02.1.6_C_33

TAGAATCTAGCAGTACTATGACCCATATAACCATATACA

>M.02.1.6_C_34

ATAATATTCTCTCTACTATTTCTTCTTTCAACTATTCA

>M.02.1.6_C_35

TTCATTTCCGGCTCACCCTAGCCAAATACTCTTTGAAT

>M.02.1.6_C_36

TGATTCTCATCTATCTCCTCCTCCAATTCCGCGAGGAC

>M.02.1.6_C_37

TTCAGTAAGTAAGTTCGATTTTACTTATCGCCCATC

>M.02.1.6_C_38

AAATTACTATCTTGAATAGAGATATTAGCGTTGTCTATTGCT

>M.02.1.6_C_39

CTAACTAAAACACAACTAAATATAACACTATATAACACA

>M.02.1.6_C_40

GCAGTTAGGAAAACCACCAGATTTGGCTAGTTATATAGCCCAG

>M.02.1.6_C_41

AAAACTAATTGTATATTAGCAGGGTCACTTTGCACTCT

>M.02.1.6_C_42

ATGTTTGATAATATGGGGGTTTTAGGGGGTGTTCCC

>M.02.1.6_C_43

AGATATTATCCTTATATTCACCTACTTTTTGCAGATATTTC

>M.02.1.6_C_44

ATCATCTGCTGGGGGTTATTCTGTTGTGAAGTGTTTGTT

>M.02.1.6_C_45

GCTATTAACATTATTAAGTTATAGACATTTTGTCCATATTCTACG

>M.02.1.6_C_46

ATGACAATGTGCGAAATAGCGACATCCCAATCAGC

>M.02.1.6_C_47

CTTGTATGCGCACTTACGAAAAGCCAATAATACTCAAA

>M.02.1.6_C_48

CTAATGAAACATAAGAGCGAAAAGAAGTTTAGTAGAGATTC

>M.02.1.6_C_49

GTATGCTATGCAACAGTATTTTAATCTTATTTCTCGGATCCT

>M.02.1.6_C_50

AGTTATTTCGACGTGATTTAACGCACAGTAAGATTTATAA

>M.02.1.6_C_51

CTTGTTAGTATATCTATTTTCATATTTATACACCCGAA

>M.02.1.6_C_52

AATTTGATCTATCGCGGGTATTAATTTGTTCATAACTG

>M.02.1.6_C_53

TATAAGAACCTAAAAGAATTAACTAAGAGTGTTGACGG

>M.02.1.6_C_54

ATTATCAAACGGATGGGTAAACCTATATAAAACATCGCCT

>M.02.1.6_C_55

CTCATCACCCCATGTTTCTTCAAATCCACCCAGATTCTC

>M.02.1.6_C_56

ATACTCTGTTCCTTGCTCCATCTTTAGTAATTTGCTAA

>M.02.1.6_C_57

TATCATCATTCTCCTTGATAAACCAGTCTCACTTTCT

>M.02.1.6_C_58

GTATTAAGTCATCAGAATATCCTTCTAATGATAGAG

>M.02.1.6_C_59

ATTAATACAATTAAGCTCCGTCTTTGCTAGACTCTTTTA

>M.02.1.6_C_60

TATTAAAATACTTATTAGCAATCATTGCTGCACGTAC

>M.02.1.6_C_61

GATATCCATTCCTCAGCACTCTTAAATCCGCTGAAATT

>M.02.1.6_C_62

GATGTACTAAGGGTAAGTCATTTATGTTATCTAGTATAA

>M.02.1.6_C_63

CTATATATGGGAAGGCTTATGATCACCTAGTAGAA

>M.02.1.6_C_64

ATTAATTCTTTTAGGTTCTTATACTTTCAATTCCTTTTAGGATTAAT

>M.02.1.6_C_65

ATTGAAAAGACTTTACGAAGTATATACAAGTGCTCA

>M.02.1.6_C_66

AAGAATCCCCAAGGATAAACACTAGCATATCGACC

>M.02.1.6_C_67

TAATTTCATTTTCATTATTTCTCAACTCCCTTAATTAC

>M.02.1.6_C_68

ACAATAAGATAGAAGGGTCTCTCGCGATAACTCTGTAGT

>M.02.1.6_C_69

TTTCGTTATCGTTCACGGAATTTATCAATGCAGTTAATT

>M.02.2.19_C_1

CAAGAAATCAGCAAATCCCCCTAAATCTCTTTTGATAG

>M.02.2.19_C_2

ACTGATTTTGACAATAATAGCGATTGTTATAGGCTCATT

>M.02.2.19_C_3

AAGAATTCTTTTAATTTGACATCTGTCATTTTTTCTCAC

>M.02.2.19_C_4

TTATACTTCTGTAAACTTCATGCTCGCCCTCACTTCTCTTAG

>M.02.2.19_C_5

CTCTTTGTTGTTATACTGATTCACAAAATTTTGCTTAG

>M.02.2.19_C_6

GGGTCACAAGTACCTAATACCCCACAACCACTTTCTAAT

>M.02.2.19_C_7

TTGTCAAACTTCTCTTTATCAGTGACCTGAAACACAC

>M.02.2.19_C_8

AAACTATATTATCTTTTCAAGATACCTACGATCATGAGCCTGT

>M.02.2.19_C_9

ATAGACAAATGATTTGCTTCCATCATAACTACTACTTCC

>M.02.2.19_C_10

ACTTAGTCCGTATAAAAGTTTATAACACAAACTCTAGTTC

>M.02.2.19_C_11

TCTTTCTCTTTTTTATTCTTTTGAATGTAATATCATTT

>M.02.2.19_C_12

TAGTAATTCAAAACCATCCTTTTTTAATATTATCTCTATC

>M.02.2.19_C_13

TTATCCTTTCGTAAGAACGATTCTAAAGTTTGTTATCGTAA

>M.02.2.19_C_14

AATTCATATTTTTGCTGTCGTAGTTGCTGTCATAGCAATT

>M.02.2.19_C_15

TTATTAAATACTGTGAAACACTTATGACAATATAGTTTAG

>M.02.2.19_C_16

TTAGTTATCTGAAATGTTAACACCCATAAGAATGCTAT

>M.02.2.19_C_17

CTGGTAGTCCATAGCTCTTCTCATAACTTTCTTTCGCTT

>M.02.2.19_C_18

ATTATTAAAATGGCAACTACATGTACTGACCTACTTCAAT

>M.02.2.19_C_19

TTTCTAGGTCAGATATCGTATATCTCATAGTCTTTGCCT

>M.02.2.19_C_20

TTATAGTCTTTATATTGTTCTTCTGTTAAATTGTTAAGATC

>M.02.2.19_C_21

TAGATTAGCTAAACAGAAAGGTGTTGTATTACCTACTT

>M.02.2.19_C_22

CCGTCAAGGTGCTGTTCTTACAGGATCCGGAAATAGTGGC

>M.02.2.19_C_23

TATATATATCTTTTGTTATTATCGTGGTTTTCATATGGATTA

>M.02.2.19_C_24

ACGCTGCAACTATGCCGTTTTTCTATGCTCTACCATTCCC

>M.02.2.19_C_25

ATAATAAATTTTCTGTTGATGTAGGTTGTTGATTTAGTGT

>M.02.2.19_C_26

AAGTCTACTTGTACATGTCAAAAGACGCTAGAATGAGA

>M.02.2.19_C_27

TCACAAATCTTCGTAAACTACGATACATTTCCTTAC

>M.02.2.19_C_28

TTTAATACTAATGTTGCTTGTATATGTTTACTTAGT

>M.02.2.19_C_29

AAGGATATATTGACATAGATGACGAAGATCTTTACTT

>M.02.2.19_C_30

CCCTGCCTCTTCATGGCAGAGGACCTGGGTCGGGCCGC

>M.02.2.19_C_31

ATACTAGCCGGCTGGTTTCTACTGGCGGATATACCCCCA

>M.02.2.19_C_32

TCTTCTAATAAGTCCATGCCCTCTGCATCAGAGGGCAT

>M.02.2.19_C_33

TCACATTCTTGGGGGAATTCGTTGCCATGATCAAACAC

>M.02.2.19_C_34

ATTCTGAAAATTGCCTATTATACCAAAGCCAAAAACTC

>M.02.2.19_C_35

AATTTATTAAGGTTGAAGATTATCAAGACAAGACAATAA

>M.02.2.19_C_36

GTCCTTTCCATATACTTTTTGAATTTTTTGCATTCCTG

>M.02.2.19_C_37

TTAATTATATACCTATGCATTCCTTCTATGTTAAGATG

>M.02.2.19_C_38

AACTTTTCAAATAGTTTGCAAGCATATAATCTATACTTAT

>M.02.2.19_C_39

GCGTAAGCAAATACACCGTTGATATTTAGTGTTTCACT

>M.02.2.19_C_40

GTAATATCTGCCATGTCATCAGAGCAAATACC

>M.02.2.19_C_41

CACACACACACCCCTATTAAATTCGAAAGCACGTT

>M.02.2.19_C_42

GTATTTGTTCAATCTGTGAGGGCGATGGGAATGAGCGAAG

>M.02.2.19_C_43

TTTACTTCTCTTTTAAGGTACTTTTTATGGCCTTATCCC

>M.02.2.19_C_44

TTATTTGAATTGCATCTTGTTCGCTTATTAAACCTAT

>M.02.2.19_C_45

ATGAGTTTATAGGGAAAGAGGACAAAGCCAAAGAGTTGA

>M.02.2.19_C_46

ATATTTATTAAGAAAGTTCGTGATAGCGTCCTTTGGCGT

>M.02.2.19_C_47

ATTATTTATAAGCTTTAAACTCGCTGATCACAGCTTATT

>M.02.2.19_C_48

AGAATACTATATAATTCTGCGGGTATTACTGCATTATTCC

>M.02.2.19_C_49

AATAGTGCATCACTAATTATTAAACTTTTTAACTTACATGT

>M.02.2.19_C_50

TTTTCAGCTCTTGCGAGAGCTAAAAAAAGACGTGTGTT

>M.02.2.19_C_51

ACTTAAAGATATAGATGAACTAGCACGGCAATATAA

>M.02.2.19_C_52

AAAGTTATCAGTTGGGTACAAAAACAATCCACAAGG

>M.02.2.19_C_53

ACAGTTGGGGATACCACAATATAATCCATAGGTTGTAC

>M.02.2.19_C_54

ATCGTATCGGTGAGGAAGAAAAAATTGAGGGCGATAAGG

>M.02.2.19_C_55

TGAAACCAACAATATCCTTTGTATTAATACTTTTAC

>M.02.2.19_C_56

GTTACGGTTAGTTTTTCCTTTTTCGCCAAAAAATCAA

>M.02.2.19_C_57

TAGTATATCTATTTTCATATTTATACACCCGAAAAAAAG

>M.02.2.19_C_58

CAAACGGGCGAGGTTATCAGTCCTGCGCCAGTGCCT

>M.02.2.19_C_59

AATTTTAGCGGGTCTTCGACCAATTTACTTGAATGTGTTT

>M.02.2.19_C_60

GTATGGTTTCTTCAGGATAGTTAAGTTTCAACATCTTCTCA

>M.02.2.19_C_61

TATGTATTAATAAAAAAACAATACCGTCATTACTTAGT

>M.02.2.19_C_62

CTATTCCTGACTGCCCATAAAATACGTATAGTAGAT

>M.02.2.19_C_63

TAGAAGATAGCGAATTAAGTCGCTTTCGTCTGCACCCA

>M.02.2.19_C_64

GACATGTACAACAGAGTTTTGCCCATTACATTTGTATTT

>M.02.2.19_C_65

CTCCTCAATATCTTATTCTTTATACAATAACTATCTA

>M.02.2.19_C_66

ATCGATTCTAACACCTTGACCATATGTAGGTATGTTCC

>M.02.2.19_C_67

TTGTCATTACTCAGACCGCTATATATTCCGACATCATAT

>M.02.2.19_C_68

TGCCTTACAGTCCTTCCTGACGTTTTATGTTAACGTC

>M.02.2.19_C_69

GTCCTAAACATTCTTGTAAACATTCATTTCTATTTTTC

>M.02.2.19_C_70

TTTATATTAAGTAACCTCAATAAGTCATTACTGTTTATTAACT

>M.02.2.19_C_71

AGAAAGTGGGATACAGAAGCCTCAAGACGATCCTTACT

>M.02.2.19_C_72

TTAATTTCGCCGAAGTCTTAGGCCCGTTTGTAGGTCAAA

>M.02.2.19_C_73

AGATATAATTTTGCATTTTTCTTGTTCCACTCTTCTATTA

>M.02.2.19_C_74

AAGTAAATATACTAATATATATATAACTAAGAATAACTT

>M.02.2.19_C_75

TCAATTGCTTTGGCGACGTCAAAGGCTAAATCGCCT

>M.02.2.19_C_76

TCAAAAATACGAGAAAACTTATGAAGCTGATATACCTTACGTTAGAAG

>M.02.2.19_C_77

TAAACGCTAGCAAAGTATCGCTTTACATTATCAATAACGATTC

>M.02.2.19_C_78

TACTTCTCACCATGATGTGGGTAATGTCCACCATTTTGCA

>M.02.2.19_C_79

AGGATTAGGTTTTGGGTTGTCTATATCAATAGAAATAAGC

>M.02.2.19_C_80

CCCATGGGCTTTTGCCCATGGTTTCATTGAGCAGACCC

>M.02.2.19_C_81

TTATCATACCTAGAAAGTAGTAAACATAAGAGAAAATCCT

>M.02.2.19_C_82

CTTTGTGTCTCCCCGTTTCTCCCGGTCAGTAAAACTACT

>M.02.2.19_C_83

TCCACCCTACCATCTGCATGCACAACGACTAAGTACCCG

>M.02.2.19_C_84

GCATATTATTACTACTTGCGGTAACAGGGCTAAACAA

>M.02.2.19_C_85

TTTATAAGAAGTTCATAAAACATCGTAAAAAATATT

>M.03.0.16_C_1

TTGTCATTACTCAGACCGCTATATATTCCGACATCATAT

>M.03.0.16_C_2

ACTGATTTTGACAATAATAGCGATTGTTATAGGCTCATT

>M.03.0.16_C_3

TTTAATACTAATGTTGCTTGTATATGTTTACTTAGT

>M.03.0.16_C_4

TCCACCCTACCATCTGCATGCACAACGACTAAGTACCCG

>M.03.0.16_C_5

ATAATAAATTTTCTGTTGATGTAGGTTGTTGATTTAGTGT

>M.03.0.16_C_6

AAACTATATTATCTTTTCAAGATACCTACGATCATGAGCCTGT

>M.03.0.16_C_7

ATATTTATTAAGAAAGTTCGTGATAGCGTCCTTTGGCGT

>M.03.0.16_C_8

AATAGTGCATCACTAATTATTAAACTTTTTAACTTACATGT

>M.03.0.16_C_9

CAAACGGGCGAGGTTATCAGTCCTGCGCCAGTGCCT

>M.03.0.16_C_10

GGGTCACAAGTACCTAATACCCCACAACCACTTTCTAAT

>M.03.0.16_C_11

TTTATATTAAGTAACCTCAATAAGTCATTACTGTTTATTAACT

>M.03.0.16_C_12

TTATCCTTTCGTAAGAACGATTCTAAAGTTTGTTATCGTAA

>M.03.0.16_C_13

TAGTAATTCAAAACCATCCTTTTTTAATATTATCTCTATC

>M.03.0.16_C_14

AGGAAGAGTAATGATGATATTTATCACCAATAATATA

>M.03.0.16_C_15

AGAATTTACATATCTACTGACGTCAGCGATGTATCTA

>M.03.0.16_C_16

TCACATTCTTGGGGGAATTCGTTGCCATGATCAAACAC

>M.03.0.16_C_17

ATTATTAAAATGGCAACTACATGTACTGACCTACTTCAAT

>M.03.0.16_C_18

ATAATTTCTTTAACGTTAGGGGCGTCTCTTAATAACTC

>M.03.0.16_C_19

TTATAGTCTTTATATTGTTCTTCTGTTAAATTGTTAAGATC

>M.03.0.16_C_20

TTATACTTCTGTAAACTTCATGCTCGCCCTCACTTCTCTTAG

>M.03.0.16_C_21

GTTACGGTTAGTTTTTCCTTTTTCGCCAAAAAATCAA

>M.03.0.16_C_22

AAGTCTACTTGTACATGTCAAAAGACGCTAGAATGAGA

>M.03.0.16_C_23

TCACAAATCTTCGTAAACTACGATACATTTCCTTAC

>M.03.0.16_C_24

AGAAAGTGGGATACAGAAGCCTCAAGACGATCCTTACT

>M.03.0.16_C_25

AAGGATATATTGACATAGATGACGAAGATCTTTACTT

>M.03.0.16_C_26

CCCTGCCTCTTCATGGCAGAGGACCTGGGTCGGGCCGC

>M.03.0.16_C_27

ATAATATACGTATAAAATTATATTATTCGCTCTGTAGTAAT

>M.03.0.16_C_28

CTATTCCTGACTGCCCATAAAATACGTATAGTAGAT

>M.03.0.16_C_29

ATCGATTCTAACACCTTGACCATATGTAGGTATGTTCC

>M.03.0.16_C_30

TTATCAACTACCTTAAACAGACATGTGCCTACACCATGAT

>M.03.0.16_C_31

TTTACTTCTCTTTTAAGGTACTTTTTATGGCCTTATCCC

>M.03.0.16_C_32

ATAAAAAGGATTGATGGGAGATCTTATACACATACATAA

>M.03.0.16_C_33

CAAGAAATCAGCAAATCCCCCTAAATCTCTTTTGATAG

>M.03.0.16_C_34

GCGTAAGCAAATACACCGTTGATATTTAGTGTTTCACT

>M.03.0.16_C_35

CACACACACACCCCTATTAAATTCGAAAGCACGTT

>M.03.0.16_C_36

TTTTCAGCTCTTGCGAGAGCTAAAAAAAGACGTGTGTT

>M.03.0.16_C_37

AAGATAATCGCTGAAAGGGAGGGAAAGGAATACGTCCCA

>M.03.0.16_C_38

GTCCTTTCCATATACTTTTTGAATTTTTTGCATTCCTG

>M.03.0.16_C_39

TTATTTGAATTGCATCTTGTTCGCTTATTAAACCTAT

>M.03.0.16_C_40

ATGAGTTTATAGGGAAAGAGGACAAAGCCAAAGAGTTGA

>M.03.0.16_C_41

ACAAAAATACGAGAAAACTTATGAAGCTGATATACCTTACGTTAGAAG

>M.03.0.16_C_42

ATTATTTATAAGCTTTAAACTCGCTGATCACAGCTTATT

>M.03.0.16_C_43

TATATATATCTTTTGTTATTATCGTGGTTTTCATATGGATTA

>M.03.0.16_C_44

TATTACTTCTAAGAATTTTGCCAACTGTTCTTTATTCGC

>M.03.0.16_C_45

TTAGTTATCTGAAATGTTAACACCCATAAGAATGCTAT

>M.03.0.16_C_46

ACTTAAAGATATAGATGAACTAGCACGGCAATATAA

>M.03.0.16_C_47

ACAGTTGGGGATACCACAATATAATCCATAGGTTGTAC

>M.03.0.16_C_48

ATTATAAAAAATGTTTTATTTGAACTATTAAAATAGAA

>M.03.0.16_C_49

TGAAACCAACAATATCCTTTGTATTAATACTTTTAC

>M.03.0.16_C_50

TATTTATCTCTAGTAGCTAACTTTACTATGTGTATGCCCG

>M.03.0.16_C_51

TAGTATATCTATTTTCATATTTATACACCCGAAAAAAAG

>M.03.0.16_C_52

CCCATGGGCTTTTGCCCATGGTTTCATTGAGCAGACCC

>M.03.0.16_C_53

GTATGGTTTCTTCAGGATAGTTAAGTTTCAACATCTTCTCA

>M.03.0.16_C_54

TGTGATGAGTGTCCAGAAGAACTCTGCGGAGATGATT

>M.03.0.16_C_55

AATTTATTAAGGTTGAAGATTATCAAGACAAGACAATAA

>M.03.0.16_C_56

TAGAAGATAGCGAATTAAGTCGCTTTCGTCTGCACCCA

>M.03.0.16_C_57

GACATGTACAACAGAGTTTTGCCCATTACATTTGTATTT

>M.03.0.16_C_58

CTCCTCAATATCTTATTCTTTATACAATAACTATCTA

>M.03.0.16_C_59

ATAGACAAATGATTTGCTTCCATCATAACTACTACTTCC

>M.03.0.16_C_60

TAAACGTTACTGGTGCTTGGATCACATATGGAATTAA

>M.03.0.16_C_61

ATACTAGCCGGCTGGTTTCTACTGGCGGATATACCCCCA

>M.03.0.16_C_62

TTTCTAGGTCAGATATCGTATATCTCATAGTCTTTGCCT

>M.03.0.16_C_63

ACTTAGTCCGTATAAAAGTTTATAACACAAACTCTAGTTC

>M.03.0.16_C_64

AGAATACTATATAATTCTGCGGGTATTACTGCATTATTCC

>M.03.0.16_C_65

AGATATAATTTTGCATTTTTCTTGTTCCACTCTTCTATTA

>M.03.0.16_C_66

TCAAGAATTTACATATACATCTTTACATTAATGTTCTAT

>M.03.0.16_C_67

CCGTCAAGGTGCTGTTCTTACAGGATCCGGAAATAGTGGC

>M.03.0.16_C_68

TAAACGCTAGCAAAGTATCGCTTTACATTATCAATAACGATTC

>M.03.0.16_C_69

GCATATTATTACTACTTGCGGTAACAGGGCTAAACAA

>M.03.0.16_C_70

AGGATTAGGTTTTGGGTTGTCTATATCAATAGAAATAAGC

>M.03.0.16_C_71

TAAGAAACTATTAAGACTAAATGATGAAGAGAAGAAAA

>M.03.0.16_C_72

AACTTTTCAAATAGTTTGCAAGCATATAATCTATACTTAT

>M.03.0.16_C_73

TTATCATACCTAGAAAGTAGTAAACATAAGAGAAAATCCT

>M.03.0.16_C_74

CTGGACAGTCACGGTCGTATAGGGCCCTCCGGGATGATAATA

>M.03.0.16_C_75

GTCCTAAACATTCTTGTAAACATTCATTTCTATTTTTC

>M.03.0.16_C_76

TACTTCTCACCATGATGTGGGTAATGTCCACCATTTTGCA

>M.03.0.42_C_1

ACCTTACTTTCTCTTCTCCCTTTAGATTAAGTTTT

>M.03.0.42_C_2

ATTAATTCTTTTACTGTATAGAAATATCTCATTTT

>M.03.0.42_C_3

GCTTGTTTCTGATTGATGATATACCTTATTATTACTGGT

>M.03.0.42_C_4

AAGTACACTTTATTAGCATTTTCTGGCAAACGATTA

>M.03.0.42_C_5

TATAAATAAGCGGTAAAAATGAAGAAAAAAACGTTTCTAT

>M.03.0.42_C_6

TTCCAAATACACGTTTATTCAATATGATATCCAGCAAC

>M.03.0.42_C_7

TTTTACAATCAGATATATTGATAGTTGATTTCTGCTA

>M.03.0.42_C_8

TCAAAATGAGTAATTTTTCTTCATGCAACATAATTCAC

>M.03.0.42_C_9

TAGAATATCAAGCAGATAAATTAGTAGTACAAGATGTGA

>M.03.0.42_C_10

ATTAATACAATTAAGCTCCGTCTTTGCTAGACTCTTTTA

>M.03.0.42_C_11

TAAATTTCTATGTTTCATTTCAAGCATTTTTCTCTACCCT

>M.03.0.42_C_12

TTACCAACCCGTTTTTATTGACAATATAACGGTTGTA

>M.03.0.42_C_13

ACAAGATTCGTAACACTATTGACGAAGGTAAGAACGTTATTCTTG

>M.03.0.42_C_14

ATATTATTCAAAAAACTGATTACGTGAATGGATTTAT

>M.03.0.42_C_15

TTTAAATTCCTTTTAACATCACATAATCTTTATTTCATATC

>M.03.0.42_C_16

TAGACAGGTCAGTATAGGTCTTACCATCTAATAAAGCAAG

>M.03.0.42_C_17

CTAAATATTTCGTGTTTATATCAAATGCATATACTTT

>M.03.0.42_C_18

TTATAGTCTTTATATTGTTCTTCTGTTAAATTGTTAAGATC

>M.03.0.42_C_19

CTATATATTCGAGAAACAGGAATTTTCCCTGACTTATTT

>M.03.0.42_C_20

TTAAACTTTTACTAACTTACTAACTTACAAACTTAC

>M.03.0.42_C_21

AAGAATGATAGAATACACTACCAGTAAACGTCACTAAAA

>M.03.0.42_C_22

ACAATACCATGTGCAGAATCAATAAACAATATGCTGT

>M.03.0.42_C_23

TTATTAATAATTTTTCGGGTGTTTTCATGTTTGTGCC

>M.03.0.42_C_24

TTAATTTCTTTGACTGGTAATTTCTGTTCTATTTCTTT

>M.03.0.42_C_25

TATCATCATTCTCCTTGATAAACCAGTCTCACTTTCT

>M.03.0.42_C_26

TCTTTCACATACGTTTCTATACGCTTCTTCTTTCCATC

>M.03.0.42_C_27

CTGACTGGTATCAAAGATAACGCTTTCTTAGGGTTCGTA

>M.03.0.42_C_28

ACTTTTGCGTTTTCATTTGTCTCTTCTTCTTCAAGTAG

>M.03.0.42_C_29

TTATTATTTACAGTTATTCTTATCTTTGCCATCCTCACCAC

>M.03.0.42_C_30

TAGAATCTAGCAGTACTATGACCCATATAACCATATACA

>M.03.0.42_C_31

ATAATATTCTCTCTACTATTTCTTCTTTCAACTATTCA

>M.03.0.42_C_32

GCAGTTAGGAAAACCACCAGATTTGGCTAGTTATATAGCCCAG

>M.03.0.42_C_33

TTCATTTCCGGCTCACCCTAGCCAAATACTCTTTGAAT

>M.03.0.42_C_34

TGATCAATCACTTGTATTCTTTTGTGTAAATTTCGTC

>M.03.0.42_C_35

TTTGTTGGGTGTTCGTCGTTGTCCCGGCATTGAGGTTTA

>M.03.0.42_C_36

TGATTCTCATCTATCTCCTCCTCCAATTCCGCGAGGAC

>M.03.0.42_C_37

TCAAAATATATATCGATGATTTCTTCAAGATCACTT

>M.03.0.42_C_38

TTCAGTAAGTAAGTTCGATTTTACTTATCGCCCATC

>M.03.0.42_C_39

AAATTACTATCTTGAATAGAGATATTAGCGTTGTCTATTGCT

>M.03.0.42_C_40

ATAGATATGATGAGATGGCAAACAATGTATACTAGGAAA

>M.03.0.42_C_41

TAATAATGATATATTGTAGATATTATCGTTGCCCGGGGC

>M.03.0.42_C_42

CTAACTAAAACACAACTAAATATAACACTATATAACACA

>M.03.0.42_C_43

ACTTGTATATCAGATATCATGTCTACTAACGGCAAATT

>M.03.0.42_C_44

AAAACTAATTGTATATTAGCAGGGTCACTTTGCACTCT

>M.03.0.42_C_45

AGATATTATCCTTATATTCACCTACTTTTTGCAGATATTTC

>M.03.0.42_C_46

CTTGTATGCGCACTTACGAAAAGCCAATAATACTCAAA

>M.03.0.42_C_47

CTAATGAAACATAAGAGCGAAAAGAAGTTTAGTAGAGATTC

>M.03.0.42_C_48

GTATGCTATGCAACAGTATTTTAATCTTATTTCTCGGATCCT

>M.03.0.42_C_49

AGTTATTTCGACGTGATTTAACGCACAGTAAGATTTATAA

>M.03.0.42_C_50

CTTGTTAGTATATCTATTTTCATATTTATACACCCGAA

>M.03.0.42_C_51

GATGAATATGAAGAATTTGCTACTATAATAGTGAAACTCC

>M.03.0.42_C_52

GCTATTAACATTATTAAGTTATAGACATTTTGTCCATATTCTACG

>M.03.0.42_C_53

TATTTATTATAACACAAAGCACTATACACTTCAAAATT

>M.03.0.42_C_54

CTCATCACCCCATGTTTCTTCAAATCCACCCAGATTCTC

>M.03.0.42_C_55

ATACTCTGTTCCTTGCTCCATCTTTAGTAATTTGCTAA

>M.03.0.42_C_56

TTATATGTAGTGACGGTAAGTAGTAGCATCTATAACTCT

>M.03.0.42_C_57

AATTTGATCTATCGCGGGTATTAATTTGTTCATAACTG

>M.03.0.42_C_58

GTATTAAGTCATCAGAATATCCTTCTAATGATAGAG

>M.03.0.42_C_59

TCTATTAATATTGTATGCAACATTATTTTAACTTTGC

>M.03.0.42_C_60

TTTCGTTATCGTTCACGGAATTTATCAATGCAGTTAATT

>M.03.0.42_C_61

GATATCCATTCCTCAGCACTCTTAAATCCGCTGAAATT

>M.03.0.42_C_62

GATTACTCTTCACAGATAAACATTCTTTAGGTAATTCT

>M.03.0.42_C_63

CTATATATGGGAAGGCTTATGATCACCTAGTAGAA

>M.03.0.42_C_64

TCCGTTTCTAACTTATTAAAGTATTCATATAATGC

>M.03.0.42_C_65

ATTGAAAAGACTTTACGAAGTATATACAAGTGCTCA

>M.03.0.42_C_66

AAGAATCCCCAAGGATAAACACTAGCATATCGACC

>M.03.0.42_C_67

TAATTTCATTTTCATTATTTCTCAACTCCCTTAATTAC

>M.03.0.42_C_68

TATAAGAACCTAAAAGAATTAACTAAGAGTGTTGACGG

>M.03.0.42_C_69

ACATAGAGGGCGTTCCTTGTCACTATTAACCATGT

>M.03.0.42_C_70

ACAATAAGATAGAAGGGTCTCTCGCGATAACTCTGTAGT

>M.03.0.42_C_71

TATTAAAATACTTATTAGCAATCATTGCTGCACGTAC

>M.03.0.50_C_1

ACGTACAACTTTATGTTCCCCTTTCCGTTTCTTTCAAA

>M.03.0.50_C_2

TATAATAGTACTAGAGGTGCTTCTTTAGGAAGA

>M.03.0.50_C_3

ACAGTTAATATCTTATTGAGATATTTTTTATGAGCCTGTCTC

>M.03.0.50_C_4

GTTCCTTGCTCCATCTTTAGTAATTTGCTAAGTAAATTAT

>M.03.0.50_C_5

AAGACCTATACGCTTCAGTGAACCCCTTCATTATCTCTCC

>M.03.0.50_C_6

GATATTCTGAATGATGCACTTGAGGCATACATCAAGAACCAT

>M.03.0.50_C_7

AAGGATATATTGACATAGATGACGAAGATCTTTACTT

>M.03.0.50_C_8

CTTCTTATGTGTTCCTTGATGAAGTCTGCTATCTCCTTGT

>M.03.0.50_C_9

TTAAGTCCTTTGATGTGACAGATTATATCAAAATTGATG

>M.03.0.50_C_10

TGGCTTGGTAGACCCCCTAAAGATATTAAGACACCA

>M.03.0.50_C_11

ATTATCTCATATATTGCATTTATTCAGATATATATGC

>M.03.0.50_C_12

GTTTCCTCATCATAGATATGCAAACGCGCGTATTCTGT

>M.03.0.50_C_13

TTAAATAACAAATAGGACATCTTTCTACAATATCTCTCA

>M.03.0.50_C_14

TACTTTAGTCTTATGAGATTTTTAATGGCTCTTATACTAT

>M.03.0.50_C_15

TCATCCTCTAGCTCATTGAAGATTTCCTTCCACTTTT

>M.03.0.50_C_16

ACTAAGAAGTAAATAATTTTCACTAATTTACCGAATTCT

>M.03.0.50_C_17

TCCTATTGAGTTTAAGCAACATGAGGAACCAACGC

>M.03.0.50_C_18

CATTTATATTATATAAAGCATGTAATTTAATCAATTTATCTA

>M.03.0.50_C_19

TTTGTTATTATCGTGGTTTTCATATGGATTAGCATTAT

>M.03.0.50_C_20

TTTCTTTATCGAATTCATATTTAGCAATCAACTTCCCAC

>M.03.0.50_C_21

TAGATTTGTTTTATCAACTCATCTTTTCTTCCCATACAA

>M.03.0.50_C_22

ATAGATAGAAATTACTCTCTACTGGATCCGCAACTTCTCG

>M.03.0.50_C_23

GTTTCATATCTACTCTTTAACTCATCAAATTTCTTTCTT

>M.03.0.50_C_24

TCCTCTTCTTTGCATTTATCGGGATGACTTCTACACCA

>M.03.0.50_C_25

AACAGATCCACTAGCGATATAGAAAGCATACAGTATATA

>M.03.0.50_C_26

GCCTTATCCTGTGGCCCTCTCACTGCCCTACCGAT

>M.03.0.50_C_27

ATAGAAAACTCCCTACCCTAATTTTTTCGTTGTTTTT

>M.03.0.50_C_28

CGATATACGTTTCAAGTCCTTATTTCATAGACTGGAGTTG

>M.03.0.50_C_29

TATTAAATTTAGGGATGTAGAAATCAAATTTCTTCTTA

>M.03.0.50_C_30

TAAAATACAAACCATCGCCCACAACGCGGGCATTGATAT

>M.03.0.50_C_31

TAATATCTGATGCACTATTTGATGAAAAATTTAA

>M.03.0.50_C_32

TATAGCTGAATGACCTTAAGGTTATTGCCATAGTCGAAAAC

>M.03.0.50_C_33

TGATTTAAAATGTACAGTCACATATTTATATTTTTTCCGCT

>M.03.0.50_C_34

TCATATAGTGTAAGCTTTTCCTCAGCGTCTTCATAGATAT

>M.03.0.50_C_35

TCAAATCCTCCTCTGGATTTACGATAACAAACTTTAGA

>M.03.0.50_C_36

CTCTTCTTTGTTGCTTTCTTGCCGTTTCCCTGTCTAATT

>M.03.0.50_C_37

TTTAATACTAATGTTGCTTGTATATGTTTACTTAGT

>M.03.0.50_C_38

TCTACGATGAACTAAAACAGCACGCGGAAGCCTTAACC

>M.03.0.50_C_39

TTCTTCTCCCTTTAGATTAGCTAGTTTTAATACGATGTA

>M.03.0.50_C_40

TCACTTAGGGGAACGACCCAAGAGACATGTAACC

>M.03.0.50_C_41

AAATAAGATGTACCAACCCCATTACGATAAAAACTAATATA

>M.03.0.50_C_42

TAGTTTATTCTCTACTACAATAGCTGTAACTGCATTTGTAT

>M.03.0.50_C_43

ATGGTGGTAAAGCCTTTACGAAAAGAGGGACTATTAAGAAA

>M.03.0.50_C_44

ACTAAGAAGTGGTATGTAACGTGCTTTCAATTCCTTTTAGGATTAAT

>M.03.0.50_C_45

TCGCTTCGATTAGGTCATCGAGTTGTAGTGGATCAA

>M.03.0.50_C_46

GCATATAGTGCTAACTTTTCCTCAACATCCTCGTATA

>M.03.0.50_C_47

ATATTCAACTTTAAACATCTCGTGTACATGCTAGCTGGA

>M.03.0.50_C_48

CCCTAATACTATAGTCAATGATTTCCTTAAAATTTAACTG

>M.03.0.50_C_49

AGGACCAATAGTAACATCTAATGCTTGACCATAGCTTAG

>M.03.0.50_C_50

TCATATTATTACTACTTGCGGTAACAGGGCTAAACAA

>M.03.0.50_C_51

TAAGTACCGTAATGTAATGCTACAATACCTAATTGT

>M.03.0.50_C_52

ACTATAAATAGATGTGAAAAAGTTTACAATATCAGAA

>M.03.0.50_C_53

ATTACAATCCACCCCCACAACCTCACAAGAACAATTATT

>M.03.0.50_C_54

TCATTCTCAGTCATAGACGAGACACCTCTTCATCTTCTT

>M.03.0.50_C_55

TTTATTTTCTTCTTCAGGTCTTCAACACTTATTTTTAATC

>M.03.0.50_C_56

AAGCATTTCTGAACAGCTACTCAGATATATTTTTTGC

>M.03.0.50_C_57

GGATTTGCTTCCCACCGGTATAACCGGTGGGAAAACTG

>M.03.0.50_C_58

ATTTTGCGGTTTTACTGCAAAAAATCAAAAACACGGT

>M.03.0.50_C_59

TTATATATCTCCATAATAAGTCTGTATCACTTCTCCAAC

>M.03.0.50_C_60

TGGAAGTCAAAGATTTCCTCAGGGTGTTGCTGCGG

>M.03.0.50_C_61

ATAGTGACAGTCATTTGTTCTTGTTTCTCTTGTTTTT

>M.03.0.50_C_62

TGACATGTAGTAGAGCAGAACTTCAAATCCTACTAGA

>M.03.0.50_C_63

TAATACTCTAGATTTGAATAATCTTGATACATATAATTC

>M.03.0.50_C_64

TTTATACCTAATCTAACTTTAGAGTTTGTTTGCATTTTGT

>M.03.0.50_C_65

TTGTTATATCCTATGTAACGAAATATTGGCGAGTCTACA

>M.03.0.50_C_66

GGGAACATAAAGTTGTACGTCTTTCAATTCCTTTTAGGATTAAT

>M.03.0.50_C_67

AAGATCAACATATGCTAGATTAGGATTAATGATACCACCA

>M.03.0.50_C_68

TTGATGTCTTGTACGCCGTACCAAACCGCCCTTGTTTCT

>M.03.0.50_C_69

TCTAAATGTTCTTCTGGGTTATAACGATTCTTAATGTG

>M.03.0.50_C_70

AGATAAAATAGCAAATATTATAGATGAAGAACTAAA

>M.03.0.50_C_71

TAAGTTACGCTTTTTTGCGAAACAAAGTAAATAAGCCGT

>M.03.0.50_C_72

AATATGTCAGCAATTATTTCTAATCCATTTCTTTTCAT

>M.03.0.50_C_73

GGTATAGCTATCAGTAGTATAGCAGTGAAAAGAACAGA

>M.03.0.50_C_74

TTATGTTGATTTATAATTTCCTCAGCTTTACTGGCCTT

>M.03.0.50_C_75

CACTCTTCCATTATCTCACAACCTTATCTTTTCAACTCT

>M.03.0.50_C_76

AAAATTAATTGTAAGTTTATGGGGTCACTTTGCACCCT

>M.03.0.50_C_77

AAAGGATCATATAGGTATATGTCAGTAAAAGGTGTTCTATG

>M.03.0.50_C_78

AGAATACTATATAATTCTGCGGGTATTACTGCATTATTCC

>M.03.0.50_C_79

GTTGTGAAACTGATTGTACATTAATTCTATTAAATGTAC

>M.03.0.50_C_80

ACTTAAAGATATAGATGAACTAGCACGGCAATATAA

>M.03.0.50_C_81

TTGCTTGTATTCCAAATACTCCTAATACCCCCAAGG

>M.03.0.50_C_82

CAATCAATATAATTACAATAATCGCAGATTACCCTAA

>M.03.0.50_C_83

TTTATATCCCCGAAAAAAGAAGTTACGCTACGCCAAAC

>M.03.0.50_C_84

TGATAGACTGTTTTGCTTGGAGAAACTGAAGGAAGAAA

>M.03.0.50_C_85

AATATCCATATATAGGTATAAATCCCCTGTTGACATCCAATT

>M.03.0.50_C_86

TTTCAATTCCTTTTAGGATTAATCTTGATTTTTTGGCGA

>M.03.0.50_C_87

TATCTAATCTTGATTTTTTAAATGAAACAACAAATAGTA

>M.03.0.50_C_88

CCTTGTATGAATCCGTGATCCCTAAGCCACCTCAATAGCTG

>M.03.0.50_C_89

TTTATTAAGAAATTAAAATCATTTAAATGCTTCACAATT

>M.03.0.50_C_90

TCCACCCTACCATCTGCATGCACAACGACTAAGTACCCG

>M.03.0.50_C_91

CCTTTATTTTTATGAGGTTCTGCAAGTACGCCTTGATT

>M.03.0.50_C_92

ATATTTTTCTATTTGAGATTTTATTAATTCATCGTTAAATATC

>M.03.0.50_C_93

GTATCGGTGTGTAGGGCAATGATAGTGTTTTCAAGGA

>M.03.0.50_C_94

TTCACCAGTGCCGAAAGCAAATATAACTTCAGTAATACCT

>M.03.0.50_C_95

AAAAGCGCCCCGTAAGCTAGGGCTACTACTTCCTCA

>M.03.0.50_C_96

CTAGATATAATTTATGGTGTAGAACCTCAAGATATTCCCG

>M.03.0.50_C_97

ATATAATAACTAGTTAGATCATACTCATATTCATCATCTAC

>M.03.0.50_C_98

GAAAATCATTTGCTCGGTGATGTCACACTGTTTTCCAT

>M.03.0.50_C_99

ACTTTCTTCTCCTTTATTGCGTTATTGAAATCAGCCC

>M.03.0.50_C_100

TGATCTTAACTACAAATTATATCAAAATTAATATAAACGT

>M.03.0.50_C_101

ATAGCATTTGTTAATAAGATTCTCAATTTAGTTTCTATAT

>M.03.0.50_C_102

TATATAATTAATAAACATGCTAAAAACAGTGATGA

>M.03.0.50_C_103

TAAATAAAATTTACTTCCTTTTGTAACATAAAAATTGAA

>M.03.0.50_C_104

TTATAGTCTTTATATTGTTCTTCTGTTAAATTGTTAAGATC

>M.03.0.50_C_105

GTTTTTCTTTTCACGAGTGAGTCACCTCATTCTTCCTCTTC

>M.03.0.50_C_106

ATTTTCTTTTTTATTGTCGTCTTTCCCGTTTTTTTCAA

>M.03.0.50_C_107

ATTTCATGCGGTGGTTCTTCTTCTTCTTTTTTCTTCT

>M.03.0.50_C_108

CACTAAAACAAAAAGTGTAGACAAAGTGTCTACACTTTTCAC

>M.03.0.50_C_109

ATAAGGGCTGTATCTGGGCCGAACTCGCCTTTTCTGTC

>M.03.0.50_C_110

CACGTTACATACCACTTCTTAGGTAACATATTCTCTTTA

>M.03.0.50_C_111

TCAGTCCATGAGCTTCCAGTGATATCAATCCATAACAGCGG

>M.03.0.50_C_112

AATTTATTAAGGTTGAAGATTATCAAGACAAGACAATAA

>M.03.0.50_C_113

AAACAGATATTTCTGTACATTACCCTTGTCATTCTCATATG

>M.03.0.50_C_114

TCATAGTTATTATAAGATGTAAATAATATTACTACCGTCCT

>M.03.0.50_C_115

TTTACTTCTCTTTTAAGGTACTTTTTATGGCCTTATCCC

>M.03.0.50_C_116

TAAACTATTTAATAGTTTTATCCACCTTACTTTTTCTT

>M.03.0.50_C_117

CACATCTTTTAATTTTATACACAACTCATCAGCTAAATC

>M.03.0.50_C_118

CTAGGAAATATAATGTTTTTAAGAGCAATTATTCCAAGC

>M.03.0.50_C_119

TTTTAACTAACCTCCACCGCCCGAAGTTGTACTTAACGTT

>M.03.0.50_C_120

ATATTGATAAGAATTATATGTGAGTATTCCTCGGCTGTTTCT

>M.03.0.50_C_121

CTAACTAAAACACAACTAACCATAACACTATATCAC

>M.03.0.50_C_122

TCTTATATATTGGCTCCACCTATCATATCGAGTGCAT

>M.03.0.50_C_123

GTTACGGTTAGTTTTTCCTTTTTCGCCAAAAAATCAA

>M.03.0.50_C_124

TTTCAATTCCTTTTAGGATTAATCAGAAGAAATTGCGTTACTT

>M.03.0.50_C_125

ATGATTATATTATCGTTAATCATTATTCCTATAACGA

>M.03.0.50_C_126

GTATGGTTTCTTCAGGATAGTTAAGTTTCAACATCTTCTCA

>M.03.0.50_C_127

AATAAAATAAGTTATAGTTTTGTATTTTGCTTCTGGCT

>M.03.0.50_C_128

TGGTGTACGACAATATTTCCTATTACCTAGCAAATACCTA

>M.03.0.50_C_129

TATTCATATTTTTGCTGTCGTAGTTGCTGTCATAGCAATT

>M.03.0.50_C_130

TATGTTGTTCTAGCCATTCGTTTATACATTCTTCTACTGT

>M.03.0.50_C_131

GCATCATTCATCTTTGAAGGATCAAGTAACGCAATTTCTTCT

>M.03.0.50_C_132

TACCTATCCAGATAGCAGAGTTATAGTTGAGCATAA

>M.03.1.5_C_1

TACATTAACGTTAGGAGTATTGACTACATTTACATTT

>M.03.1.5_C_2

ATAATGACTTTCCTCTGTACATTTCAAACTCTTCAACA

>M.03.1.5_C_3

TTAGCATATCTCTCTACAAAGCTTTCAACATCGCTCTC

>M.03.1.5_C_4

CTCATTTAGAATGACGGATGCTTCTTTGTGTCTTTCTT

>M.03.1.5_C_5

AGTGTTAATCAAGGGCTAATTTCACTATTAACTGATTT

>M.03.1.5_C_6

TAAACACTAACCCCAATGCACCGTTTCTAGCATCCAT

>M.03.1.5_C_7

TATGGATTTACTGGACTTAGTTGATAGTTATAATTTAC

>M.03.1.5_C_8

TTCTTCTTCTTCATAGAATATTTCATCTCAGTTCACCAA

>M.03.1.5_C_9

GCGGACTTACCTGATAATTATAATTAACTTGCTCCCACTT

>M.03.1.5_C_10

TAGGTGCAGGCGCAGTAGCGATATATTTAATATCTA

>M.03.1.5_C_11

TTATATACATTAGTTGTAGTATTGACGATTAGTTGTACTGAG

>M.03.1.5_C_12

CTGGTGTGATGTTAGTTATTAAATATGATAGCGGT

>M.03.1.5_C_13

CCTAATCAGTTCAACCCCACAGAGTCCATCCCCGAC

>M.03.1.5_C_14

GTGGAAACTCAGTTTACGGCGAATACGAAATGGAC

>M.03.1.5_C_15

ATATAATAGATATCCTATTATTGCTATGATGATGAT

>M.03.1.5_C_16

CCTTTAGCGATTGGATCAGTTTCTCTTTCGCGTTCCA

>M.03.1.5_C_17

GTATAACACTACAAATTATAGTGAAGCGATACGTAGG

>M.03.1.5_C_18

AAAAGATAACTATAAGCATTTTTTACATCTGAAAATAACGCA

>M.03.1.5_C_19

GAACCGGTAAGGTTATCAAATCTCACGTACATACATCAC

>M.03.1.5_C_20

ACTTCTTCTTCCTCATCATTAGCCTTCTTACTAATTTTC

>M.03.1.5_C_21

GTACTCGAATTCTAGCTTTACGACTTTTTGTCTTTCCCCC

>M.03.1.5_C_22

TATATCATACTCTTTAGCTTTGCCATTAATATCTCAA

>M.03.1.5_C_23

ATACTCTGTTCTCTGTATATTCATTTTTCATCATCATC

>M.03.1.5_C_24

GCAATGTTATCAAGATGATAGAACTTATTATTTATTTCAT

>M.03.1.5_C_25

AAGAAAAAATGAAAGACAGTGTACTCGAGGAAGTCC

>M.03.1.5_C_26

TTACCGCTTATCCATCAACGGGAACCAGTACTACAAATACTAC

>M.03.1.5_C_27

CATCATTGACCCCCCTAGAACTTTATCTTTTCGACCCTCG

>M.03.1.5_C_28

AAAGTCAAACGGCAATTCACCGTTTACAATCATAT

>M.03.1.5_C_29

TTATAGTCTTTATATTGTTCTTCTGTTAAATTGTTAAGATC

>M.03.1.5_C_30

TCAGTATGAGGGTCTTCAGTAACGTCGTCTTTCCCG

>M.03.1.5_C_31

TTTCTCTCTCAGTTTTTCTTTCCATCTTAGTACTGTT

>M.03.1.5_C_32

ATATAATACTTTAGATCACGATCTCCTAAAATCAT

>M.03.1.5_C_33

CTGCATATTTTAGCTGCTTCCGCAAACTCTTTCCTCCACT

>M.03.1.5_C_34

TCTTTTGATTGCTCTGATGCTAAATGGTGTCTCAACTTA

>M.03.1.5_C_35

CTAACCACTTTAACGATTGCAAATACAGGCGGTGCTGTACTT

>M.03.1.5_C_36

TTAGATAATCTACTTAATTGGATATATAGTAAATTAGTAT

>M.03.1.5_C_37

GAGAAAAGCTCTAAATGGTAATAAAAAATGAGTAGT

>M.03.1.5_C_38

AAATTGTACAAAATGGAAATACTGTCGCGCAAATTCAACC

>M.03.1.5_C_39

ATAAATCATCCATTTTTACGTATGCTTTTCGATGCATATT

>M.03.1.5_C_40

AATATAACTTCAGTAATACCTAAATTATTAGAATTAATA

>M.03.1.5_C_41

AGTTTTTTATTAGGTAAAATTTTTACACTCATTTT

>M.03.1.5_C_42

TTTATCCGCCATCTCGAATTTGATAACTACACCACCCA

>M.03.1.5_C_43

TGTGGGCTATTACGCTACTGAGGGGCTTGGAGGAGTAGGTG

>M.03.1.5_C_44

ACAAGAACTATAGTAGTTCAGCAAAATCCGAAGCTT

>M.03.1.5_C_45

TCCTCGAATATGAGAGGATCATAAAGAGTATAAACACAA

>M.03.1.5_C_46

AGTTTAATATACTCAACCCTAGCCTTTATCATTTCTT

>M.03.1.5_C_47

TAGGTATGTAGATATGGATATTTTTGTTTTGTAGTTTGT

>M.03.1.5_C_48

TTGAGAACCTTTCCTTCTTCAAGCCTCACTGCTATATTA

>M.03.1.5_C_49

ATGCCATCAATGCCAAGAGTTTAACGTCTGTATCCCCA

>M.03.1.5_C_50

ATAAACTCGTTCTAGGAAAAGAACCACCCTCAAACCTAA

>M.03.1.5_C_51

CCATCACAGAACCATTGCCATACCCAATCACCGGACATA

>M.03.1.5_C_52

TCTTTATCTAGTAAACTAATTAAAAAAAGAAGATCTTCC

>M.03.1.5_C_53

ATATTACTTCTCTTCCTCTTGTTTCAGTATCAATATAAC

>M.03.1.5_C_54

CCACTTCTTTACCAGTTCAAAATAATCATATTCTGTTCTA

>M.03.1.5_C_55

AATAAACTCTTTCTTTGATTTCATCAGAAAAAGCGTCAT

>M.03.1.5_C_56

AGTACCTCATGTAACTCTTTCGGCGTAGCTTTTCCTTC

>M.03.1.5_C_57

GTGTAATAAAATATCTATATAGTAAATTAAAACATCAT

>M.03.1.5_C_58

TAATACGTATTTGTCAGAAGCGTTGTTAGAGCTGACGAAATTG

>M.03.1.5_C_59

GGGGAGAAGATGGATAAAGTAGTTAAAAAACAATTGGAATTAT

>M.03.1.5_C_60

AAATAGAGACATTTATAGATCTGCTAGATATTATCTATCGT

>M.03.1.5_C_61

GTTTATTCTAATGCAATATATCATCTTTCTACTTGT

>M.03.1.5_C_62

CTAACTGCCCTATATGATGTTCTACGGTATTCGTATTCT

>M.03.1.5_C_63

ATAATAATTGTGAAAGTCGTCTAATCTCATCGTATCCAC

>M.03.1.5_C_64

ACGTTTTCATAGCTTTTATTTGTTTCTTGTATTTCTT

>M.03.1.5_C_65

GCGGATTAACACCGGCACTTACGTTCACCCCTTGGCTT

>M.03.1.5_C_66

ATATTTCCTCCCTTATCCCTTCTACCACTTTTTCATTAC

>M.03.1.5_C_67

GTTACATCATACACCTGATGACATTGCTAGAATGAAGAT

>M.03.1.5_C_68

ATAAATCTACATTATTATACAAAACTTGTAAAATTGTATTT

>M.03.1.5_C_69

CTAATATTGTTTCAACGTTTGACGCCAAGGCCCCA

>M.03.1.5_C_70

GCCAAGGGACTTTCAATCTTACCACAGGCGTTTATTATT

>M.03.1.5_C_71

CTACATTTATAATATGTCTCACTTTTAAATACATCATA

>M.03.1.5_C_72

TCATATTATTACTACTTGCGGTAACAGGGCTAAACAA

>M.03.1.5_C_73

ATGAACTTTATCTTACCTTGGGATAGCAATTGCTTTATCTC

>M.03.1.5_C_74

ATGTATACCATGTCAATATAGCCGTTCTGGAACGGGTAG

>M.03.1.5_C_75

ACTACAATAGCTGTAACTGCGTTTGTATGGTGGCAAGCT

>M.03.1.5_C_76

AGTTTTTTACATTATCATGAACAAGTAAACCGGAAAC

>M.03.1.5_C_77

TAATCCCTTGGTAACCTTAACCCCCCTTCGTTTAAG

>M.03.1.5_C_78

TTGTATAAAGGAGCTAGTATTCGTATCCTATCATTCATG

>M.03.1.5_C_79

ATTCAATATTATGCATTTCCCCTTACGTCCCCTCAAGT

>M.03.1.5_C_80

ATAGTAACGACATCAATACTTATCATCGTCCACATT

>M.03.1.5_C_81

CGAGCTACTCGCATAATCTTCCACTTTTTTTAGTAAT

>M.03.1.5_C_82

AAACAAGAAGAAAAAGAAGTAGATGAGTTTTTTTTATTT

>M.04.0.10_C_1

CAAGAAATCAGCAAATCCCCCTAAATCTCTTTTGATAG

>M.04.0.10_C_2

TTTATATTAAGTAACCTCAATAAGTCATTACTGTTTATTAACT

>M.04.0.10_C_3

TTATACTTCTGTAAACTTCATGCTCGCCCTCACTTCTCTTAG

>M.04.0.10_C_4

TCCACCCTACCATCTGCATGCACAACGACTAAGTACCCG

>M.04.0.10_C_5

ATAATAAATTTTCTGTTGATGTAGGTTGTTGATTTAGTGT

>M.04.0.10_C_6

AAACTATATTATCTTTTCAAGATACCTACGATCATGAGCCTGT

>M.04.0.10_C_7

ATATTTATTAAGAAAGTTCGTGATAGCGTCCTTTGGCGT

>M.04.0.10_C_8

TGCTAAGTCTAAACGTACTTCTAGCAGATTTCGGCATTT

>M.04.0.10_C_9

ACTTAGTCCGTATAAAAGTTTATAACACAAACTCTAGTTC

>M.04.0.10_C_10

ACGTAGTAAGAATATCTCAGAGGACACAATTAAACACT

>M.04.0.10_C_11

GGGTCACAAGTACCTAATACCCCACAACCACTTTCTAAT

>M.04.0.10_C_12

ACTGATTTTGACAATAATAGCGATTGTTATAGGCTCATT

>M.04.0.10_C_13

TTATCCTTTCGTAAGAACGATTCTAAAGTTTGTTATCGTAA

>M.04.0.10_C_14

ATAGACAAATGATTTGCTTCCATCATAACTACTACTTCC

>M.04.0.10_C_15

TAGTAATTCAAAACCATCCTTTTTTAATATTATCTCTATC

>M.04.0.10_C_16

TAGTACGTTCAGCGTAAAGCAGCTAGCTCCTTACTTGCC

>M.04.0.10_C_17

ACAGTTGGGGATACCACAATATAATCCATAGGTTGTAC

>M.04.0.10_C_18

TCACATTCTTGGGGGAATTCGTTGCCATGATCAAACAC

>M.04.0.10_C_19

AGATATAATTTTGCATTTTTCTTGTTCCACTCTTCTATTA

>M.04.0.10_C_20

TTAGTTATCTGAAATGTTAACACCCATAAGAATGCTAT

>M.04.0.10_C_21

TTCTATTAATTTCAATTCCTTTAGGCATAGGATTTTTTC

>M.04.0.10_C_22

CAAACGGGCGAGGTTATCAGTCCTGCGCCAGTGCCT

>M.04.0.10_C_23

TTTCTAGGTCAGATATCGTATATCTCATAGTCTTTGCCT

>M.04.0.10_C_24

TTATAGTCTTTATATTGTTCTTCTGTTAAATTGTTAAGATC

>M.04.0.10_C_25

AAGTCTACTTGTACATGTCAAAAGACGCTAGAATGAGA

>M.04.0.10_C_26

TCACAAATCTTCGTAAACTACGATACATTTCCTTAC

>M.04.0.10_C_27

AGAAAGTGGGATACAGAAGCCTCAAGACGATCCTTACT

>M.04.0.10_C_28

AAGGATATATTGACATAGATGACGAAGATCTTTACTT

>M.04.0.10_C_29

CCCTGCCTCTTCATGGCAGAGGACCTGGGTCGGGCCGC

>M.04.0.10_C_30

AATTCATATTTTTGCTGTCGTAGTTGCTGTCATAGCAATT

>M.04.0.10_C_31

ATCGATTCTAACACCTTGACCATATGTAGGTATGTTCC

>M.04.0.10_C_32

CTATTCCTGACTGCCCATAAAATACGTATAGTAGAT

>M.04.0.10_C_33

TAAAGAAGATCTTCCCTAGTTCTCTTTCTTTTTCCAAGATT

>M.04.0.10_C_34

TTTACTTCTCTTTTAAGGTACTTTTTATGGCCTTATCCC

>M.04.0.10_C_35

TTATTCTCTTCTTCTTCAAGTAGATCCACATCCCC

>M.04.0.10_C_36

TTGTCATTACTCAGACCGCTATATATTCCGACATCATAT

>M.04.0.10_C_37

GCGTAAGCAAATACACCGTTGATATTTAGTGTTTCACT

>M.04.0.10_C_38

CACACACACACCCCTATTAAATTCGAAAGCACGTT

>M.04.0.10_C_39

TTACTCCGTTTCCTTCCTCATCCCAGAGGTCGATAG

>M.04.0.10_C_40

GTCCTTTCCATATACTTTTTGAATTTTTTGCATTCCTG

>M.04.0.10_C_41

TTATTTGAATTGCATCTTGTTCGCTTATTAAACCTAT

>M.04.0.10_C_42

ATGAGTTTATAGGGAAAGAGGACAAAGCCAAAGAGTTGA

>M.04.0.10_C_43

ACAAAAATACGAGAAAACTTATGAAGCTGATATACCTTACGTTAGAAG

>M.04.0.10_C_44

ATTATTTATAAGCTTTAAACTCGCTGATCACAGCTTATT

>M.04.0.10_C_45

TATATATATCTTTTGTTATTATCGTGGTTTTCATATGGATTA

>M.04.0.10_C_46

TTTAATACTAATGTTGCTTGTATATGTTTACTTAGT

>M.04.0.10_C_47

TACACTATGTCCTTTTGATGCTAAACTTACTTTTTGT

>M.04.0.10_C_48

TTTCTTAGTTTTCTATTCTCATTCTTTAACTTTCTTAT

>M.04.0.10_C_49

GCATATTATTACTACTTGCGGTAACAGGGCTAAACAA

>M.04.0.10_C_50

CCCATGGGCTTTTGCCCATGGTTTCATTGAGCAGACCC

>M.04.0.10_C_51

TGAAACCAACAATATCCTTTGTATTAATACTTTTAC

>M.04.0.10_C_52

GTTACGGTTAGTTTTTCCTTTTTCGCCAAAAAATCAA

>M.04.0.10_C_53

ATTATTAAAATGGCAACTACATGTACTGACCTACTTCAAT

>M.04.0.10_C_54

TTTTAGTGTGGGACTGATAAATCAAGAACCTATCTGT

>M.04.0.10_C_55

GTATGGTTTCTTCAGGATAGTTAAGTTTCAACATCTTCTCA

>M.04.0.10_C_56

AATTTATTAAGGTTGAAGATTATCAAGACAAGACAATAA

>M.04.0.10_C_57

GACATGTACAACAGAGTTTTGCCCATTACATTTGTATTT

>M.04.0.10_C_58

CTCCTCAATATCTTATTCTTTATACAATAACTATCTA

>M.04.0.10_C_59

TCTTCTAATAAGTCCATGCCCTCTGCATCAGAGGGCAT

>M.04.0.10_C_60

CTATATATCTCACATCTTTTAATTTTATACATAACTCAT

>M.04.0.10_C_61

ATACTAGCCGGCTGGTTTCTACTGGCGGATATACCCCCA

>M.04.0.10_C_62

ATTAGGTACAGGGCTTTATGCTATAGTAATAAGCAG

>M.04.0.10_C_63

TAGTATATCTATTTTCATATTTATACACCCGAAAAAAAG

>M.04.0.10_C_64

AATAGTGCATCACTAATTATTAAACTTTTTAACTTACATGT

>M.04.0.10_C_65

AGAATACTATATAATTCTGCGGGTATTACTGCATTATTCC

>M.04.0.10_C_66

ATTTCTTTAGAAATAAGTGATATTTTATCTCTATTAGT

>M.04.0.10_C_67

CACAATAGCTCAACTTCGGTTACATGTCTCTTGGGTCGT

>M.04.0.10_C_68

CCGTCAAGGTGCTGTTCTTACAGGATCCGGAAATAGTGGC

>M.04.0.10_C_69

TAAACGCTAGCAAAGTATCGCTTTACATTATCAATAACGATTC

>M.04.0.10_C_70

GTCCTAAACATTCTTGTAAACATTCATTTCTATTTTTC

>M.04.0.10_C_71

AGGATTAGGTTTTGGGTTGTCTATATCAATAGAAATAAGC

>M.04.0.10_C_72

AACTTTTCAAATAGTTTGCAAGCATATAATCTATACTTAT

>M.04.0.10_C_73

AATCTTCCATTCACATACTATACTACATCATGCCCACA

>M.04.0.10_C_74

TTATCATACCTAGAAAGTAGTAAACATAAGAGAAAATCCT

>M.04.0.10_C_75

TTTTCAGCTCTTGCGAGAGCTAAAAAAAGACGTGTGTT

>M.04.0.10_C_76

TACTTCTCACCATGATGTGGGTAATGTCCACCATTTTGCA

>M.04.0.13_C_1

CCTATCAATTCGGCAGGGAGCAGTTCTGGCGCTAC

>M.04.0.13_C_2

TGGTTGTGGATGAAAGGAGCGGCCTGACTACGAAAG

>M.04.0.13_C_3

TTCTTCTTCTTCATAGAATATTTCATCTCAGTTCACCAA

>M.04.0.13_C_4

CCAATAGATACTATGAAGAAAAGACAGATAGAGTTC

>M.04.0.13_C_5

ACGGATCAATATTTATAAACTTCTTAGTAAAAGAAGGATATCT

>M.04.0.13_C_6

ATTCCTTTATCAAGAAATTAAAATAATCTAAATACTTTGCT

>M.04.0.13_C_7

ATAAATCTACATTATTATACAAAACTTGTAAAATTGTATTT

>M.04.0.13_C_8

TCTCTGCATATAACATAAAGGCAAAGACTATGAGATAT

>M.04.0.13_C_9

AAATAGAGACATTTATAGATCTGCTAGATATTATCTATCGT

>M.04.0.13_C_10

CAGAAGTCCCAATGGATATCCCTCCAGGCCCTATGCT

>M.04.0.13_C_11

CCTAAGTTTTTAATTCTGTCTGCGAGTGACTGAACTTCGC

>M.04.0.13_C_12

TCAGTATGAGGGTCTTCAGTAACGTCGTCTTTCCCG

>M.04.0.13_C_13

TTGAGAACCTTTCCTTCTTCAAGCCTCACTGCTATATTA

>M.04.0.13_C_14

TCGATTTTGCTCGCTACTTTTCCTGCGATACCGGGTCCT

>M.04.0.13_C_15

ACCCCATGTATCATGAGAAAAAACTAAAAATTCCTTTTC

>M.04.0.13_C_16

TAAGTAGACATGTTATAGACTTGGACGAAGACCCTCGT

>M.04.0.13_C_17

ATGAAGTTATTCTTAGTTATATATATATTAGTATATTTA

>M.04.0.13_C_18

CTTAATTTCATTCTACAATCACCACATCATCTTTACTT

>M.04.0.13_C_19

TAGGTGCAGGCGCAGTAGCGATATATTTAATATCTA

>M.04.0.13_C_20

ATATAATATTGAAGATGCCGAAATCAGAAAGAGTGGCATA

>M.04.0.13_C_21

AATATAACTTCAGTAATACCTAAATTATTAGAATTAATA

>M.04.0.13_C_22

ACCACCTAATGACGATCCAATATACGATGACATTAGAG

>M.04.0.13_C_23

GGCGTAACAACTATATAGTTACCTAACTGTTCTATTTTT

>M.04.0.13_C_24

CTTATTTGGTCATCGGTTAAGCAATTAAACGTCTTTAAC

>M.04.0.13_C_25

GTTCTACATAGAATTCTACATCAGCCATTTTACTCAC

>M.04.0.13_C_26

AACGTTGATGCGTCTAGTGTTAAATCTGGAGAGGGTGTT

>M.04.0.13_C_27

ATAACGGTTGGGTGGGATATGTTGGGCAGGGAAATAT

>M.04.0.13_C_28

GTGTAATAAAATATCTATATAGTAAATTAAAACATCAT

>M.04.0.13_C_29

CCTTGATCGAGGCGAGCTTGCCGTCTAAAACTTCAAT

>M.04.0.13_C_30

TTATATACATTAGTTGTAGTATTGACGATTAGTTGTACTGAG

>M.04.0.13_C_31

CTGGTGTGATGTTAGTTATTAAATATGATAGCGGT

>M.04.0.13_C_32

ATAATTATAACGTTTGTGCCTACCTTAGCGTTTCCTATT

>M.04.0.13_C_33

TTATAATCTTTATATTGTTCTTCTGTTAAATTGTTAAGATC

>M.04.0.13_C_34

TATATCATACTCTTTAGCTTTGCCATTAATATCTCAA

>M.04.0.13_C_35

GGTGTGATTCTATTTCCTGTCAGAAGGCTCTCAGCT

>M.04.0.13_C_36

TATAAAAGAAGTACAAGATGCACTACAAGATAATGAAT

>M.04.0.13_C_37

ACTACTAATGTCATAAATAATAATTATGAAATAGGTGCGTT

>M.04.0.13_C_38

AGGCTATCTCCGTTTCTCTTTACCACGTTCAATGATCCGA

>M.04.0.13_C_39

CTGCATATTTTAGCTGCTTCCGCAAACTCTTTCCTCCACT

>M.04.0.13_C_40

TCATTTATCTCAATTTTTAGATGCAAATACGACCCATATT

>M.04.0.13_C_41

TTAGATAATCTACTTAATTGGATATATAGTAAATTAGTAT

>M.04.0.13_C_42

TTGAAAGTGTTGTTTTTCCAGTACCGGGCGGGCCCGTGA

>M.04.0.13_C_43

GGAAAATACTATTATTATCCCGGAGGGCCCTATACGACC

>M.04.0.13_C_44

CCATCACAGAACCATTGCCATACCCAATCACCGGACATA

>M.04.0.13_C_45

GGGGAGAAGATGGATAAAGTAGTTAAAAAACAATTGGAATTAT

>M.04.0.13_C_46

ATTCAATATTATGCATTTCCCCTTACGTCCCCTCAAGT

>M.04.0.13_C_47

CTAACTGCCCTATATGATGTTCTACGGTATTCGTATTCT

>M.04.0.13_C_48

AGATAAATATACCAATTGCCTGCAAAAAGGGCATTTGAAT

>M.04.0.13_C_49

TACAAGACCGCGTCCAGCCAACTCCATTAAAAGTTCTTT

>M.04.0.13_C_50

TCATTTTCGAGCTGTACGGAGAGTTTTGCGATTTTTGT

>M.04.0.13_C_51

AAACTTCTCGCATGTATTACTATCATAATCTAGTGTTATC

>M.04.0.13_C_52

TCATATTATTACTACTTGCGGTAACAGGGCTAAACAA

>M.04.0.13_C_53

ACAAACTCGTAGACCTTACACGTGCCGTCCTTGAGCTT

>M.04.0.13_C_54

GTGGAAACTCAGTTTACGGCGAATACGAAATGGAC

>M.04.0.13_C_55

CCTTTCATTCTTTGGCTCGTAGTTAATTAGAATCTTAAGAA

>M.04.0.13_C_56

TAATTTTCGATGAAGCTCACAACTTGGAACTCCAAG

>M.04.0.13_C_57

GTACTCGAATTCTAGCTTTACGACTTTTTGTCTTTCCCCC

>M.04.0.13_C_58

AGTTTTATTGGTTTCTTTTCCCCCTTATTCTCACCCGTGGG

>M.04.0.13_C_59

AAAAGATAACTATAAGCATTTTTTACATCTGAAAATAACGCA

>M.04.0.13_C_60

TCTCTTGTGCGCTTCTCATGTTCCAGGTTGATGAC

>M.04.0.13_C_61

AGGTTCTCTTAGTGATATTGCTAACGCTTTTGCAACAT

>M.04.0.13_C_62

GACATAGTGTAGCGTCATTATCACGTAGTTAAACAGTAAA

>M.04.0.13_C_63

CATAGTGAGCATACATATGGTGTTTGACAAACATATAT

>M.04.0.13_C_64

TTACATATAAACTTAACGTGTTTCTCGTCTGCTTCAAT

>M.04.0.13_C_65

ACAAGAACTATAGTAGTTCAGCAAAATCCGAAGCTT

>M.04.0.13_C_66

TCTTTATCTAGTAAACTAATTAAAAAAAGAAGATCTTCC

>M.04.0.13_C_67

ATTTTCTCGTTCATAGGATATTAGGCTATAATCCCCAC

>M.04.0.13_C_68

AGTACCTCATGTAACTCTTTCGGCGTAGCTTTTCCTTC

>M.04.0.13_C_69

GTATAACACTACAAATTATAGTGAAGCGATACGTAGG

>M.04.0.13_C_70

AGTTTTTTATTAGGTAAAATTTTTACACTCATTTT

>M.04.0.13_C_71

CTAATATTGTTTCAACGTTTGACGCCAAGGCCCCA

>M.04.0.13_C_72

TGGACTGGAGAACTTCTCTCAAGCAGTCCAGCAATTTAA

>M.04.0.13_C_73

ATGTTTGATAATATGGGGGTTTTAGGGGGTGTTCCCCCTAATG

>M.04.0.13_C_74

ATATAATAGATATCCTATTATTGCTATGATGATGAT

>M.04.0.13_C_75

ATAATGACTTTCCTCTGTACATTTCAAACTCTTCAACA

>M.04.0.13_C_76

CTACATTTATAATATGTCTCACTTTTAAATACATCATA

>M.04.0.13_C_77

GAAACTACACAATAAACGATAACTACACTGTAGTAACCGTGC

>M.04.0.13_C_78

ATTTATGACATTCAGGAAAACAGACAACGGTGTAGA

>M.04.0.13_C_79

AAGAAAAAATGAAAGACAGTGTACTCGAGGAAGTCC

>M.04.0.13_C_80

ATATGTGATCCAAGCACCAGTAACGTTTATGAAACCAGCA

>M.04.0.13_C_81

GCAATGTTATCAAGATGATAGAACTTATTATTTATTTCAT

>M.04.0.13_C_82

TGATAATCAGACTTATGTACGGGAGGAGAGTTAAAAT

>M.04.0.13_C_83

GTCATAATAAACACACTCCTCTACGTTAATGTACCCCCTT

>M.04.0.13_C_84

TAGGTATGTAGATATGGATATTTTTGTTTTGTAGTTTGT

>M.04.0.13_C_85

TCTTCTTCAAGTAGATCCACATCCCCCGAAATAGGGGA

>M.04.0.13_C_86

ATAAACTCGTTCTAGGAAAAGAACCACCCTCAAACCTAA

>M.04.0.13_C_87

TTATTCTCTTCTTCTAATAAGTCCATGCCCTCTGCATCAGA

>M.04.0.13_C_88

ATAAAAACCGGTACGCTGGAATACTGCCGGCCGGACTCC

>M.04.0.13_C_89

TCAATAGATAAAATAGATGAATGGGAGGTATCTGGTAA

>M.04.0.13_C_90

ATAGTAACGACATCAATACTTATCATCGTCCACATT

>M.04.0.13_C_91

GCGGATTAACACCGGCACTTACGTTCACCCCTTGGCTT

>M.04.0.13_C_92

GTTGATGTAGGTTGTTGATTTAGTGTTGATATGCT

>M.04.0.13_C_93

ATGTATACCATGTCAATATAGCCGTTCTGGAACGGGTAG

>M.04.0.13_C_94

AGTTTTTTACATTATCATGAACAAGTAAACCGGAAAC

>M.04.0.13_C_95

ATGAACTTTATCTTACCTTGGGATAGCAATTGCTTTATCTC

>M.04.0.13_C_96

AAACAAGAAGAAAAAGAAGTAGATGAGTTTTTTTTATTT

>M.04.0.37_C_1

AACTTTTCAAATAGTTTGCAAGCATATAATCTATACTTAT

>M.04.0.37_C_2

ACTGATTTTGACAATAATAGCGATTGTTATAGGCTCATT

>M.04.0.37_C_3

TTATACTTCTGTAAACTTCATGCTCGCCCTCACTTCTCTTAG

>M.04.0.37_C_4

GGGTCACAAGTACCTAATACCCCACAACCACTTTCTAAT

>M.04.0.37_C_5

TTTCTTAGTTTTCTATTCTCATTCTTTAACTTTCTTAT

>M.04.0.37_C_6

AAACTATATTATCTTTTCAAGATACCTACGATCATGAGCCTGT

>M.04.0.37_C_7

ATATTTATTAAGAAAGTTCGTGATAGCGTCCTTTGGCGT

>M.04.0.37_C_8

TGCTAAGTCTAAACGTACTTCTAGCAGATTTCGGCATTT

>M.04.0.37_C_9

AGAAAGTGGGATACAGAAGCCTCAAGACGATCCTTACT

>M.04.0.37_C_10

ACGTAGTAAGAATATCTCAGAGGACACAATTAAACACT

>M.04.0.37_C_11

TAGTAATTCAAAACCATCCTTTTTTAATATTATCTCTATC

>M.04.0.37_C_12

TTATCCTTTCGTAAGAACGATTCTAAAGTTTGTTATCGTAA

>M.04.0.37_C_13

AATTCATATTTTTGCTGTCGTAGTTGCTGTCATAGCAATT

>M.04.0.37_C_14

TAGTACGTTCAGCGTAAAGCAGCTAGCTCCTTACTTGCC

>M.04.0.37_C_15

TTAGTTATCTGAAATGTTAACACCCATAAGAATGCTAT

>M.04.0.37_C_16

ATTATTAAAATGGCAACTACATGTACTGACCTACTTCAAT

>M.04.0.37_C_17

TTTCTAGGTCAGATATCGTATATCTCATAGTCTTTGCCT

>M.04.0.37_C_18

ATAGACAAATGATTTGCTTCCATCATAACTACTACTTCC

>M.04.0.37_C_19

ATTTCTTTAGAAATAAGTGATATTTTATCTCTATTAGT

>M.04.0.37_C_20

TCTTCTAATAAGTCCATGCCCTCTGCATCAGAGGGCAT

>M.04.0.37_C_21

ATAATAAATTTTCTGTTGATGTAGGTTGTTGATTTAGTGT

>M.04.0.37_C_22

AAGTCTACTTGTACATGTCAAAAGACGCTAGAATGAGA

>M.04.0.37_C_23

TCACAAATCTTCGTAAACTACGATACATTTCCTTAC

>M.04.0.37_C_24

TTTAATACTAATGTTGCTTGTATATGTTTACTTAGT

>M.04.0.37_C_25

AAGGATATATTGACATAGATGACGAAGATCTTTACTT

>M.04.0.37_C_26

CCCTGCCTCTTCATGGCAGAGGACCTGGGTCGGGCCGC

>M.04.0.37_C_27

ATACTAGCCGGCTGGTTTCTACTGGCGGATATACCCCCA

>M.04.0.37_C_28

TCACATTCTTGGGGGAATTCGTTGCCATGATCAAACAC

>M.04.0.37_C_29

ATCGATTCTAACACCTTGACCATATGTAGGTATGTTCC

>M.04.0.37_C_30

AATTTATTAAGGTTGAAGATTATCAAGACAAGACAATAA

>M.04.0.37_C_31

CTATATATCTCACATCTTTTAATTTTATACATAACTCAT

>M.04.0.37_C_32

GTCCTTTCCATATACTTTTTGAATTTTTTGCATTCCTG

>M.04.0.37_C_33

TTATTCTCTTCTTCTTCAAGTAGATCCACATCCCC

>M.04.0.37_C_34

CAAGAAATCAGCAAATCCCCCTAAATCTCTTTTGATAG

>M.04.0.37_C_35

GCGTAAGCAAATACACCGTTGATATTTAGTGTTTCACT

>M.04.0.37_C_36

CACACACACACCCCTATTAAATTCGAAAGCACGTT

>M.04.0.37_C_37

TTACTCCGTTTCCTTCCTCATCCCAGAGGTCGATAG

>M.04.0.37_C_38

TTTACTTCTCTTTTAAGGTACTTTTTATGGCCTTATCCC

>M.04.0.37_C_39

TTATTTGAATTGCATCTTGTTCGCTTATTAAACCTAT

>M.04.0.37_C_40

ACAAAAATACGAGAAAACTTATGAAGCTGATATACCTTACGTTAGAAG

>M.04.0.37_C_41

ATGAGTTTATAGGGAAAGAGGACAAAGCCAAAGAGTTGA

>M.04.0.37_C_42

ATTATTTATAAGCTTTAAACTCGCTGATCACAGCTTATT

>M.04.0.37_C_43

AGAATACTATATAATTCTGCGGGTATTACTGCATTATTCC

>M.04.0.37_C_44

AATAGTGCATCACTAATTATTAAACTTTTTAACTTACATGT

>M.04.0.37_C_45

TACACTATGTCCTTTTGATGCTAAACTTACTTTTTGT

>M.04.0.37_C_46

TTTTCAGCTCTTGCGAGAGCTAAAAAAAGACGTGTGTT

>M.04.0.37_C_47

TTTTAGTGTGGGACTGATAAATCAAGAACCTATCTGT

>M.04.0.37_C_48

TTCTATTAATTTCAATTCCTTTAGGCATAGGATTTTTTC

>M.04.0.37_C_49

ACAGTTGGGGATACCACAATATAATCCATAGGTTGTAC

>M.04.0.37_C_50

ATTAGGTACAGGGCTTTATGCTATAGTAATAAGCAG

>M.04.0.37_C_51

TGAAACCAACAATATCCTTTGTATTAATACTTTTAC

>M.04.0.37_C_52

GTTACGGTTAGTTTTTCCTTTTTCGCCAAAAAATCAA

>M.04.0.37_C_53

TAGTATATCTATTTTCATATTTATACACCCGAAAAAAAG

>M.04.0.37_C_54

CAAACGGGCGAGGTTATCAGTCCTGCGCCAGTGCCT

>M.04.0.37_C_55

GTATGGTTTCTTCAGGATAGTTAAGTTTCAACATCTTCTCA

>M.04.0.37_C_56

CTATTCCTGACTGCCCATAAAATACGTATAGTAGAT

>M.04.0.37_C_57

GACATGTACAACAGAGTTTTGCCCATTACATTTGTATTT

>M.04.0.37_C_58

CTCCTCAATATCTTATTCTTTATACAATAACTATCTA

>M.04.0.37_C_59

TATATATATCTTTTGTTATTATCGTGGTTTTCATATGGATTA

>M.04.0.37_C_60

TTGTCATTACTCAGACCGCTATATATTCCGACATCATAT

>M.04.0.37_C_61

TAAAGAAGATCTTCCCTAGTTCTCTTTCTTTTTCCAAGATT

>M.04.0.37_C_62

GTCCTAAACATTCTTGTAAACATTCATTTCTATTTTTC

>M.04.0.37_C_63

TTTATATTAAGTAACCTCAATAAGTCATTACTGTTTATTAACT

>M.04.0.37_C_64

ACTTAGTCCGTATAAAAGTTTATAACACAAACTCTAGTTC

>M.04.0.37_C_65

AGATATAATTTTGCATTTTTCTTGTTCCACTCTTCTATTA

>M.04.0.37_C_66

CACAATAGCTCAACTTCGGTTACATGTCTCTTGGGTCGT

>M.04.0.37_C_67

CCGTCAAGGTGCTGTTCTTACAGGATCCGGAAATAGTGGC

>M.04.0.37_C_68

TAAACGCTAGCAAAGTATCGCTTTACATTATCAATAACGATTC

>M.04.0.37_C_69

TACTTCTCACCATGATGTGGGTAATGTCCACCATTTTGCA

>M.04.0.37_C_70

AGGATTAGGTTTTGGGTTGTCTATATCAATAGAAATAAGC

>M.04.0.37_C_71

CCCATGGGCTTTTGCCCATGGTTTCATTGAGCAGACCC

>M.04.0.37_C_72

AATCTTCCATTCACATACTATACTACATCATGCCCACA

>M.04.0.37_C_73

TTATCATACCTAGAAAGTAGTAAACATAAGAGAAAATCCT

>M.04.0.37_C_74

TCCACCCTACCATCTGCATGCACAACGACTAAGTACCCG

>M.04.0.37_C_75

GCATATTATTACTACTTGCGGTAACAGGGCTAAACAA

>M.04.1.4_C_1

CTGCTCATCTCCCCCACCCTAACTCCCTTCGGGACGGCTC

>M.04.1.4_C_2

TTCCAAATACACGTTTATTCAATATGATATCCAGCAAC

>M.04.1.4_C_3

ACAATTAACAAAACTTCAGCAACTCATTCATCAAAAA

>M.04.1.4_C_4

ATTTTTGCTATAGTAGTTGTAATATATTCACCACTATT

>M.04.1.4_C_5

TCGTCCTTTTTTACTAAAAAAATAAAATGATTATATTGT

>M.04.1.4_C_6

TTTATCCTCATGCCGTTTACTAACTGTATTAATAAGATAG

>M.04.1.4_C_7

ATTAGTTTCTTGCCGTTTTCACTTATCAAAAAGTCTTTTATT

>M.04.1.4_C_8

AGAGGGAGCCTTCGGGGTGTGGCTTCCCCCCTCCCATTTTCC

>M.04.1.4_C_9

ATAAGGAATAAATATGATAGAACTTTCATATCAGAT

>M.04.1.4_C_10

AGTATTATGTCAAGTATTTTGCTAGGTCGTACGATATTG

>M.04.1.4_C_11

ATATTAAAATTTATTTGCTTAAGTATTGAGACTATTTC

>M.04.1.4_C_12

TTCATGAGTGGGTCACCTCATTCCTCCTCTTCACAATA

>M.04.1.4_C_13

TTCATATTTGAACCTCTTCCGTAATTCATTCACTTCTTC

>M.04.1.4_C_14

GCAAGGCAGTGGACAATATGCTCTAGGGACAGAATATACGC

>M.04.1.4_C_15

CTTCTTCCCCATCTTTCCCCCCACTATATTCTGTTTTAC

>M.04.1.4_C_16

ATTTAAATTATATTTTAGGGCGTATCTGTCTAGCAGTTGTA

>M.04.1.4_C_17

CTCCATATCGCTATCCAGTAATCGTTAATGTATTTCT

>M.04.1.4_C_18

ATGCTTTCGTTTTGTGTTTTTTCATTTACTTGTTCTT

>M.04.1.4_C_19

TCAAATAATGTGCTAACCATGCCGAAGGCACTTCAGCAT

>M.04.1.4_C_20

TTCATCACCCCATGTTTCTTCAAATCCACCCAGATTCTC

>M.04.1.4_C_21

TTTATATCTGTGATATCAACACCAGTAATTTTCTGTAT

>M.04.1.4_C_22

ACATTAACATTATTGCTTTTTATCCAAGGATATTTCCA

>M.04.1.4_C_23

AAGAATCCCCAAGGATAAACACTAGCATATCGACC

>M.04.1.4_C_24

AATTTGAATGTTACAACCCTGTTGTTCATAAGTATTACTC

>M.04.1.4_C_25

ATATCTTTTGATCGTTTGTAGAATTCTATCAACCTCC

>M.04.1.4_C_26

AGTTGCAAATTCATTATTATTATCAAGTCCAAGTACGAAA

>M.05.0.30_C_1

TATTTTATCACCTGCTTCTTAATATAGTTCAAACACCTC

>M.05.0.30_C_2

ACAATAAACCTGGAGTATATACTGTAAAAATACTG

>M.05.0.30_C_3

AGAAGATGATCTACGTGAATTGTGCAGAAAAACTAATGAT

>M.05.0.30_C_4

TTCTACACTTTCAGAGAACATAGTTTCTTTATTTACA

>M.05.0.30_C_5

CAATAATGTTTATGTCTTTTGTTTGCCTAAATTCTT

>M.05.0.30_C_6

TCAAAATGAGTAATTTTTCTTCATGCAACATAATTCAC

>M.05.0.30_C_7

ATAACTCTACTACATTTTCTCATGTAAATTCTAGTGCAT

>M.05.0.30_C_8

ATAATAATAATAATGCATTATACTACACAATTAATGCAA

>M.05.0.30_C_9

CTTGAATAAGCACTTATAGAATTTTTATTAATAGCTTCAG

>M.05.0.30_C_10

ATAGTCACGTATATTGGATACCCAAGCTTCTCATAATTA

>M.05.0.30_C_11

TATACTATAATCTATAACGTCTCTGAAATTGATGTCTT

>M.05.0.30_C_12

TAGACAGGTCAGTATAGGTCTTACCATCTAATAAAGCAAG

>M.05.0.30_C_13

TTTTTCTTTCACCTCGATGTAAATATTTACTTCTTCAA

>M.05.0.30_C_14

TTATAGTCTTTATATTGTTCTTCTGTTAAATTGTTAAGATC

>M.05.0.30_C_15

TTTAATCTTATTTCTTATGTCTATGCCTTCTTCACGT

>M.05.0.30_C_16

TTAGAACAAATCGTAAATGAGTATTATGATATGAAGTGCC

>M.05.0.30_C_17

ACTTTATACTGTAGATATTTACCGGGTAGGACAAAGTAT

>M.05.0.30_C_18

ATAGATAGAAATTACTCTCTACTGGATCCGCAACTTC

>M.05.0.30_C_19

TTAGGAAGAAGGGGGACTACTGTAGTGTGTGTTACAATA

>M.05.0.30_C_20

AAATTAGTCGATACTATAAATCAGTTACAAAAACTATTGTT

>M.05.0.30_C_21

TAAAAAGGTAAACGAGAAATACGGTCATATCTCTTTT

>M.05.0.30_C_22

AGGATCTCAAGACATATGGTAACAAGTTTGTTTTCTT

>M.05.0.30_C_23

TCTTTCACATACGTTTCTATACGCTTCTTCTTTCCATC

>M.05.0.30_C_24

ACCATATCTTTCCATGCATCAGTTTCTTGACTTCCATAC

>M.05.0.30_C_25

CTAAACCTATCAGAGTTAGCAGAATATAGTAATATTGATATT

>M.05.0.30_C_26

ATTTAAATTATATTTTAGGGCGTATCTGTCTAGCAGTTGTA

>M.05.0.30_C_27

TCTTGTCTAGTTCATCTAACAATTTTAATAGCGTCTCGA

>M.05.0.30_C_28

ATTACACTTAAGATTGTTGCTTTTTTACTATTCTG

>M.05.0.30_C_29

ATTTTTATCGCCCCGTCAATCATCAGATCTTTAACG

>M.05.0.30_C_30

TGATTTCGTCTAGTAACTTCATCTTTACGCTTTCTGG

>M.05.0.30_C_31

TTGCAACCTAGGTTGATAACATAGAAATTTTCCAATTAATT

>M.05.0.30_C_32

TTATTATTTACAGTTATTCTTATCTTTGCCATCCTCACCAC

>M.05.0.30_C_33

ATCAGTATTGGGAAGTAGATTGCTTTGCCTTTATTCTCT

>M.05.0.30_C_34

ATTTCGTACATCAAGTATTTTGATGACAGTAAGAAATATTG

>M.05.0.30_C_35

TCTCATCTTTAGCAATAAATCATGAGTTACTGGAA

>M.05.0.30_C_36

ATGCTTTCGTTTTGTGTTTTTTCATTTACTTGTTCTT

>M.05.0.30_C_37

TACTTCTCACCATGATGTGGGTAATGTCCACCATTTTGCAG

>M.05.0.30_C_38

ATTTTTGCTATAGTAGTTGTAATATATTCACCACTATT

>M.05.0.30_C_39

TTGTTACTCAAATATATTCACCTCCATCTTTTGT

>M.05.0.30_C_40

GAATGATATTAAGATGGTATCACTAACTGCCCTATA

>M.05.0.30_C_41

CATATATAGTGAATTTCTTTTGAGATAATAGATTAT

>M.05.1.5_C_1

TTATAATCTTTATATTGTTCTTCTGTTAAATTGTTAAGATC

>M.05.1.5_C_2

TGATGAACTTATCACTTGGCATTTTCATTTCACCTT

>M.05.1.5_C_3

GTGTAATAAAATATCTATATAGTAAATTAAAACATCAT

>M.05.1.5_C_4

TTCTTCTTCTTCATAGAATATTTCATCTCAGTTCACCAA

>M.05.1.5_C_5

CTAACAGGTATCTAAAAACAAACACACACTCGCTTCGTTAT

>M.05.1.5_C_6

TAGGTGCAGGCGCAGTAGCGATATATTTAATATCTA

>M.05.1.5_C_7

GTCATTCATCATCACTCTCCTTTTTCGGATAATACCA

>M.05.1.5_C_8

AATCGCTTCCTGAGTCTCATCTAATTCTGCATTTTTGT

>M.05.1.5_C_9

TTAATAATATCATAAGGATTATTATGAGAAAAAGAGGTAC

>M.05.1.5_C_10

TAAACACTAACCCCAATGCACCGTTTCTAGCATCCAT

>M.05.1.5_C_11

GTTTATAAACGGGCGTTATGAGGACCGTGTAGTTGTTT

>M.05.1.5_C_12

CTGCATATTTTAGCTGCTTCCGCAAACTCTTTCCTCCACT

>M.05.1.5_C_13

GAACCGGTAAGGTTATCAAATCTCACGTACATACATCAC

>M.05.1.5_C_14

ATAATTGATTTCCAAGTCCCGTCAAATTTGATTTCCT

>M.05.1.5_C_15

TTCTATGATTTAGAAAAGCGTCCAAAGGACTGAAATAA

>M.05.1.5_C_16

ATAAATCTACATTATTATACAAAACTTGTAAAATTGTATTT

>M.05.1.5_C_17

GCAATGTTATCAAGATGATAGAACTTATTATTTATTTCAT

>M.05.1.5_C_18

TATAAATTGGCAAGGGTGGAATGTAACGATATACCA

>M.05.1.5_C_19

ATAACAATATTGAGGACGGGCAATGGAATTGAAAAATTCTT

>M.05.1.5_C_20

CCCATGCGTTAGCGTTTCTGTTTGCGTAAATGCGGTCT

>M.05.1.5_C_21

TAAACTTCTTCATGTTCATCCCTTTTTCCTTCTCCTTTC

>M.05.1.5_C_22

AAAGTTTCAACGATACAATTTAAACTTTGAGCAATTAATTTC

>M.05.1.5_C_23

ATAAGTTGTTTCTCCCTTTCATTCTTTGGCTCGTAGCT

>M.05.1.5_C_24

TTCTTAAGAAAGCCAAACTTTTCTCATTTATCTCCATGATT

>M.05.1.5_C_25

TAGGTATGTAGATATGGATATTTTTGTTTTGTAGTTTGT

>M.05.1.5_C_26

TTGAGAACCTTTCCTTCTTCAAGCCTCACTGCTATATTA

>M.05.1.5_C_27

TAACTACAGCTTATACATATGCTAGCTTAAGCAGCACAG

>M.05.1.5_C_28

TTAGATAATCTACTTAATTGGATATATAGTAAATTAGTAT

>M.05.1.5_C_29

CGGGTCGGTATCACTGACTTGTATTCCACATGTGTTTATAT

>M.05.1.5_C_30

AAAAAATTTTCTATATCTTGAATTATAAAACTCAC

>M.05.1.5_C_31

TTTTTACATTATCATGAACAAGTAAACCGGAAACCTT

>M.05.1.5_C_32

GGGTTATCGTCAATTATAACAACGTCTCCTAATCCCTTTAA

>M.05.1.5_C_33

ATAAACTCGTTCTAGGAAAAGAACCACCCTCAAACCTAA

>M.05.1.5_C_34

ATACGCTTTAAATCGTCAAAGTCCCAGATAAATGGC

>M.05.1.5_C_35

TGTATATACTTCGTAAAGTCTTTTCAATTGAATTAATTC

>M.05.1.5_C_36

ATAATATACGTATAAAATTATATTATTCGCTCTGTAGTA

>M.05.1.5_C_37

TCTATCACTGTACTGATTTCACAACTTTCCAACGGCTC

>M.05.1.5_C_38

TTATATACATTAGTTGTAGTATTGACGATTAGTTGTACTGAG

>M.05.1.5_C_39

TTAGCAGATTCACGTTCTGGTCTTGAATTTTTACTCA

>M.05.1.5_C_40

TATATTGAGGATCGTTAGAATATTGTGTATAAAGC

>M.05.1.5_C_41

TTTATAACATCTGGTATGTCTTCAGTTTCTGTGTATCTTGT

>M.05.1.5_C_42

GTATAACACTACAAATTATAGTGAAGCGATACGTAGG

>M.05.1.5_C_43

CTTTTCTCTCAAACTTCTCACAATAGCTTACGTCTTCTTT

>M.05.1.5_C_44

TATTATAAGTAACGCATATTACTGGAAGTTTTCTGACAT

>M.05.1.5_C_45

TTAGTACTCGTCATTGTTCAACAACCTCCACGTTTAAAAT

>M.05.1.5_C_46

CTAACTGCCCTATATGATGTTCTACGGTATTCGTATTCT

>M.05.1.5_C_47

AGCCTAGAATTATAATCATAAACTCTATCAGTCTTCCT

>M.05.1.5_C_48

CTTCGTTGTTTTCATTTTTTCCACCATTTTGCAAGGT

>M.05.1.5_C_49

CTAATATTGTTTCAACGTTTGACGCCAAGGCCCCA

>M.05.1.5_C_50

CTCTCCATCAGGTATAACTGAAGGCCCAGAGCTAGCAC

>M.05.1.5_C_51

TCTTTATCTAGTAAACTAATTAAAAAAAGAAGATCTTCC

>M.05.1.5_C_52

ATTAATTGCTCCAGTGGTATAAAATGTAAATAGTCA

>M.05.1.5_C_53

AACTTATACACTATTGTTTCACAATTATTAGAGCTT

>M.05.1.5_C_54

TCATATTATTACTACTTGCGGTAACAGGGCTAAACAA

>M.05.1.5_C_55

AATCATTCTTCAATTGATTAAAGTCGATGAAAGATACTT

>M.05.1.5_C_56

GTATTGCTTCTTTGGTATTACTCTGAGCGAAGTAATAC

>M.05.1.5_C_57

AAATAGAGACATTTATAGATCTGCTAGATATTATCTATCGT

>M.05.1.5_C_58

AGTTTAAATGTGACAGTTCTCATTTTTTCCACCTCA

>M.05.1.5_C_59

TTTTCCCATTGATTTGTAAAATATCGTAAAACTTTCC

>M.05.1.5_C_60

TTTATTATCTTTTCGTCTTCATTTATCTCAATTTTTAG

>M.05.1.5_C_61

TTATAGGCTCTGTACTGATAATCATATCAACGGCAT

>M.05.1.5_C_62

TTAACTATCTTGCGGAGAAGAAGGGTCTTGACGGCA

>M.05.1.5_C_63

TATATATTTTCTTTTTACTCCCTGCTTCTGTTTTAGTTTCT

>M.05.1.5_C_64

ATTCAATATTATGCATTTCCCCTTACGTCCCCTCAAGT

>M.05.1.5_C_65

GTGGAAACTCAGTTTACGGCGAATACGAAATGGAC

>M.05.1.5_C_66

ATGAACTTTATCTTACCTTGGGATAGCAATTGCTTTATCTC

>M.05.1.5_C_67

AAACAAGAAGAAAAAGAAGTAGATGAGTTTTTTTTATTT

>M.05.3.4_C_1

AACTTTTCAAATAGTTTGCAAGCATATAATCTATACTTAT

>M.05.3.4_C_2

ACTGATTTTGACAATAATAGCGATTGTTATAGGCTCATT

>M.05.3.4_C_3

TTATACTTCTGTAAACTTCATGCTCGCCCTCACTTCTCTTAG

>M.05.3.4_C_4

TCCACCCTACCATCTGCATGCACAACGACTAAGTACCCG

>M.05.3.4_C_5

GGGTCACAAGTACCTAATACCCCACAACCACTTTCTAAT

>M.05.3.4_C_6

AAACTATATTATCTTTTCAAGATACCTACGATCATGAGCCTGT

>M.05.3.4_C_7

ATATTTATTAAGAAAGTTCGTGATAGCGTCCTTTGGCGT

>M.05.3.4_C_8

AGTAACGCCTTTGTTATATCAGTCCCTCCGCTTGG

>M.05.3.4_C_9

ATTATAAAAAATGTTTTATTTGAACTATTAAAATAGAA

>M.05.3.4_C_10

CAAACGGGCGAGGTTATCAGTCCTGCGCCAGTGCCT

>M.05.3.4_C_11

ATAGACAAATGATTTGCTTCCATCATAACTACTACTTCC

>M.05.3.4_C_12

TTATCCTTTCGTAAGAACGATTCTAAAGTTTGTTATCGTAA

>M.05.3.4_C_13

TAGTAATTCAAAACCATCCTTTTTTAATATTATCTCTATC

>M.05.3.4_C_14

CAATATTACTGTAATATATATTTATATGAATAATCAACA

>M.05.3.4_C_15

AGGAAGAGTAATGATGATATTTATCACCAATAATATA

>M.05.3.4_C_16

TTTAATACTAATGTTGCTTGTATATGTTTACTTAGT

>M.05.3.4_C_17

TCACATTCTTGGGGGAATTCGTTGCCATGATCAAACAC

>M.05.3.4_C_18

TTAGTTATCTGAAATGTTAACACCCATAAGAATGCTAT

>M.05.3.4_C_19

TTTCTAGGTCAGATATCGTATATCTCATAGTCTTTGCCT

>M.05.3.4_C_20

TTATAGTCTTTATATTGTTCTTCTGTTAAATTGTTAAGATC

>M.05.3.4_C_21

TATATATATCTTTTGTTATTATCGTGGTTTTCATATGGATTA

>M.05.3.4_C_22

GTTACGGTTAGTTTTTCCTTTTTCGCCAAAAAATCAA

>M.05.3.4_C_23

ATAATAAATTTTCTGTTGATGTAGGTTGTTGATTTAGTGT

>M.05.3.4_C_24

TCACAAATCTTCGTAAACTACGATACATTTCCTTAC

>M.05.3.4_C_25

AGAAAGTGGGATACAGAAGCCTCAAGACGATCCTTACT

>M.05.3.4_C_26

AAGGATATATTGACATAGATGACGAAGATCTTTACTT

>M.05.3.4_C_27

CCCTGCCTCTTCATGGCAGAGGACCTGGGTCGGGCCGC

>M.05.3.4_C_28

AATTCATATTTTTGCTGTCGTAGTTGCTGTCATAGCAATT

>M.05.3.4_C_29

CTATTCCTGACTGCCCATAAAATACGTATAGTAGAT

>M.05.3.4_C_30

AAGTCTACTTGTACATGTCAAAAGACGCTAGAATGAGA

>M.05.3.4_C_31

TTTACTTCTCTTTTAAGGTACTTTTTATGGCCTTATCCC

>M.05.3.4_C_32

ATAAAAAGGATTGATGGGAGATCTTATACACATACATAA

>M.05.3.4_C_33

TTGTCATTACTCAGACCGCTATATATTCCGACATCATAT

>M.05.3.4_C_34

GCGTAAGCAAATACACCGTTGATATTTAGTGTTTCACT

>M.05.3.4_C_35

TTTAAAAATGTATATAGTGTACTCATTTTAACTAACCTCCTC

>M.05.3.4_C_36

CACACACACACCCCTATTAAATTCGAAAGCACGTT

>M.05.3.4_C_37

AAGATAATCGCTGAAAGGGAGGGAAAGGAATACGTCCCA

>M.05.3.4_C_38

GTCCTTTCCATATACTTTTTGAATTTTTTGCATTCCTG

>M.05.3.4_C_39

TTATTTGAATTGCATCTTGTTCGCTTATTAAACCTAT

>M.05.3.4_C_40

ATGAGTTTATAGGGAAAGAGGACAAAGCCAAAGAGTTGA

>M.05.3.4_C_41

ACAAAAATACGAGAAAACTTATGAAGCTGATATACCTTACGTTAGAAG

>M.05.3.4_C_42

ATTATTTATAAGCTTTAAACTCGCTGATCACAGCTTATT

>M.05.3.4_C_43

ATCGATTCTAACACCTTGACCATATGTAGGTATGTTCC

>M.05.3.4_C_44

TATTACTTCTAAGAATTTTGCCAACTGTTCTTTATTCGC

>M.05.3.4_C_45

AATAGTGCATCACTAATTATTAAACTTTTTAACTTACATGT

>M.05.3.4_C_46

TTTTCAGCTCTTGCGAGAGCTAAAAAAAGACGTGTGTT

>M.05.3.4_C_47

ACTTAAAGATATAGATGAACTAGCACGGCAATATAA

>M.05.3.4_C_48

GTGTTATAGTTGATCCGCCACTTCCACCTGAACCACC

>M.05.3.4_C_49

GCATATTATTACTACTTGCGGTAACAGGGCTAAACAA

>M.05.3.4_C_50

ACAGTTGGGGATACCACAATATAATCCATAGGTTGTAC

>M.05.3.4_C_51

TGAAACCAACAATATCCTTTGTATTAATACTTTTAC

>M.05.3.4_C_52

TATTTATCTCTAGTAGCTAACTTTACTATGTGTATGCCCG

>M.05.3.4_C_53

ATTATTAAAATGGCAACTACATGTACTGACCTACTTCAAT

>M.05.3.4_C_54

CCCATGGGCTTTTGCCCATGGTTTCATTGAGCAGACCC

>M.05.3.4_C_55

GTATGGTTTCTTCAGGATAGTTAAGTTTCAACATCTTCTCA

>M.05.3.4_C_56

TGTGATGAGTGTCCAGAAGAACTCTGCGGAGATGATT

>M.05.3.4_C_57

AATTTATTAAGGTTGAAGATTATCAAGACAAGACAATAA

>M.05.3.4_C_58

TAGAAGATAGCGAATTAAGTCGCTTTCGTCTGCACCCA

>M.05.3.4_C_59

GACATGTACAACAGAGTTTTGCCCATTACATTTGTATTT

>M.05.3.4_C_60

CTCCTCAATATCTTATTCTTTATACAATAACTATCTA

>M.05.3.4_C_61

ATAATATACGTATAAAATTATATTATTCGCTCTGTAGTAAT

>M.05.3.4_C_62

TAAACGTTACTGGTGCTTGGATCACATATGGAATTAA

>M.05.3.4_C_63

ATACTAGCCGGCTGGTTTCTACTGGCGGATATACCCCCA

>M.05.3.4_C_64

TTTATATTAAGTAACCTCAATAAGTCATTACTGTTTATTAACT

>M.05.3.4_C_65

TAGTATATCTATTTTCATATTTATACACCCGAAAAAAAG

>M.05.3.4_C_66

ACTTAGTCCGTATAAAAGTTTATAACACAAACTCTAGTTC

>M.05.3.4_C_67

AGAATACTATATAATTCTGCGGGTATTACTGCATTATTCC

>M.05.3.4_C_68

AGATATAATTTTGCATTTTTCTTGTTCCACTCTTCTATTA

>M.05.3.4_C_69

TCAAGAATTTACATATACATCTTTACATTAATGTTCTAT

>M.05.3.4_C_70

CCGTCAAGGTGCTGTTCTTACAGGATCCGGAAATAGTGGC

>M.05.3.4_C_71

TAAACGCTAGCAAAGTATCGCTTTACATTATCAATAACGATTC

>M.05.3.4_C_72

AAATCAAGCCCTATATCCGCTAACTTCTTCTTCATTTCC

>M.05.3.4_C_73

AGGATTAGGTTTTGGGTTGTCTATATCAATAGAAATAAGC

>M.05.3.4_C_74

CAAGAAATCAGCAAATCCCCCTAAATCTCTTTTGATAG

>M.05.3.4_C_75

TTATCATACCTAGAAAGTAGTAAACATAAGAGAAAATCCT

>M.05.3.4_C_76

CTGGACAGTCACGGTCGTATAGGGCCCTCCGGGATGATAATA

>M.05.3.4_C_77

GTCCTAAACATTCTTGTAAACATTCATTTCTATTTTTC

>M.05.3.4_C_78

TACTTCTCACCATGATGTGGGTAATGTCCACCATTTTGCA

>M.06.0.8_C_1

TTCTACACTTTCAGAGAACATAGTTTCTTTATTTACA

>M.06.0.8_C_2

CCGCCCTACTTGTTTTTATATTAAATGCTATTCCC

>M.06.0.8_C_3

TTAAGTCCTTTGATGTGACAGATTATATCAAAATTGAT

>M.06.0.8_C_4

GTATAATCTTCAACAGACATATTTTACTTTTATTATTACT

>M.06.0.8_C_5

TCGTATATCTGTACAATCTTCAATTCGTTAAGTAGTTTC

>M.06.0.8_C_6

ATATTTTCAACTCTATAGCCGTTTTTCATGCATTCTTCTGAT

>M.06.0.8_C_7

TCAAAATGAGTAATTTTTCTTCATGCAACATAATTCAC

>M.06.0.8_C_8

ATAACTCTACTACATTTTCTCATGTAAATTCTAGTGCAT

>M.06.0.8_C_9

TTAGCTAGATCAGCTGCTTCAGCTTCTCTCAGCGCTCTC

>M.06.0.8_C_10

CTTGAATAAGCACTTATAGAATTTTTATTAATAGCTTCAG

>M.06.0.8_C_11

ATAGTCACGTATATTGGATACCCAAGCTTCTCATAATTA

>M.06.0.8_C_12

TAGACAGGTCAGTATAGGTCTTACCATCTAATAAAGCAAG

>M.06.0.8_C_13

ACAACAAGAAGTACGATACATAACTGTGCCGTCGTTGA

>M.06.0.8_C_14

TTATAGTCTTTATATTGTTCTTCTGTTAAATTGTTAAGATC

>M.06.0.8_C_15

TTTAATCTTATTTCTTATGTCTATGCCTTCTTCACGT

>M.06.0.8_C_16

TTAGAACAAATCGTAAATGAGTATTATGATATGAAGTGCC

>M.06.0.8_C_17

ACTTTATACTGTAGATATTTACCGGGTAGGACAAAGTAT

>M.06.0.8_C_18

TGATAATCTTCAACCTTAATAAATTCGAAATAAATATC

>M.06.0.8_C_19

ATAGATAGAAATTACTCTCTACTGGATCCGCAACTTC

>M.06.0.8_C_20

TTAGGAAGAAGGGGGACTACTGTAGTGTGTGTTACAATA

>M.06.0.8_C_21

TCTTTCACATACGTTTCTATACGCTTCTTCTTTCCATC

>M.06.0.8_C_22

TTATTATTTACAGTTATTCTTATCTTTGCCATCCTCACCAC

>M.06.0.8_C_23

TTAGTATATCATATGCTTCCTTCATCATGTCAATATC

>M.06.0.8_C_24

AGGATCTCAAGACATATGGTAACAAGTTTGTTTTCTT

>M.06.0.8_C_25

CTAAACCTATCAGAGTTAGCAGAATATAGTAATATTGATATT

>M.06.0.8_C_26

TTAATAAAACCAAACTTATTGCATTTAATAACGGCGTT

>M.06.0.8_C_27

ATTTAAATTATATTTTAGGGCGTATCTGTCTAGCAGTTGTA

>M.06.0.8_C_28

TCTTGTCTAGTTCATCTAACAATTTTAATAGCGTCTCGA

>M.06.0.8_C_29

ATTACACTTAAGATTGTTGCTTTTTTACTATTCTG

>M.06.0.8_C_30

ATAGCTTCTTTAATGTATTTCTGTGTCACTTTGTCTTT

>M.06.0.8_C_31

ATCAGTATTGGGAAGTAGATTGCTTTGCCTTTATTCTCT

>M.06.0.8_C_32

ATTTCGTACATCAAGTATTTTGATGACAGTAAGAAATATTG

>M.06.0.8_C_33

ATGCTTTCGTTTTGTGTTTTTTCATTTACTTGTTCTT

>M.06.0.8_C_34

ATTTCTGTCTTATGTAGCTTCTTAGCTTCGTTTAACCAT

>M.06.0.8_C_35

TTGTTACTCAAATATATTCACCTCCATCTTTTGT

>M.06.0.8_C_36

TTCAAATACCCTAATTGTTCTCTCTACCTCATTTAGATC

>M.06.0.8_C_37

AATTCCTTTATCTTTGCTTCCAGCTCTTCTATCTTCTTT

>M.06.0.8_C_38

CATATATAGTGAATTTCTTTTGAGATAATAGATTAT

>M.06.2.4_C_1

TTATAGATCCAGCCCTAGATCTAGTTTCTTTTATCTCT

>M.06.2.4_C_2

TCAAAAAATTATTTTCTCTATTTTGTGAAAAAGTTAA

>M.06.2.4_C_3

ATATAATAGATATCCTATTATTGCTATGATGATGAT

>M.06.2.4_C_4

AGAAGAATATGTAGAACCTATTCTTTCTCTCTATGATCAC

>M.06.2.4_C_5

GTTCACAAAGAGGCAAAGCAGTTCTAGGTTACACGGA

>M.06.2.4_C_6

TACAGGGGTCATACTTGCTAGATTTATTACAACATCTA

>M.06.2.4_C_7

GTAGGTGTCAGTACTGGAAACAGATCGTCGAACCTTCCTG

>M.06.2.4_C_8

ATAAATCTACATTATTATACAAAACTTGTAAAATTGTATTT

>M.06.2.4_C_9

CTAATACTAATGTGTATGGCTCTTTTTTCGCTATATTCG

>M.06.2.4_C_10

TCAGTATGAGGGTCTTCAGTAACGTCGTCTTTCCCG

>M.06.2.4_C_11

CGCACTTGCAGCAATTAGAGAATTCTACGATGCCCCT

>M.06.2.4_C_12

TTGAGAACCTTTCCTTCTTCAAGCCTCACTGCTATATTA

>M.06.2.4_C_13

TTATACTTTTTCTAATTGCTTTCTGAATCTTTCTTTTCT

>M.06.2.4_C_14

CCAAACCTGATTCCAGTGCAAGACCTCCTGCTTCTCCTAT

>M.06.2.4_C_15

ATATAATGAAGTATACGGATATACTGCATCAAATTTAT

>M.06.2.4_C_16

AGTAATCGGGTTCGGGTTTGCTATCGGTCTTTACGTTC

>M.06.2.4_C_17

TGAATTATAAAACTCACAATTGCACCTCTGTACCTGATAATT

>M.06.2.4_C_18

ATAGAGTTGCAAGCCCGAAGGCTCCCTCTCGACCTCCCTCG

>M.06.2.4_C_19

CTACTTTATACTGTAGATATTTACTGGGTAGGACAAAGTAT

>M.06.2.4_C_20

ATTCAATATTATGCATTTCCCCTTACGTCCCCTCAAGT

>M.06.2.4_C_21

GTGGAAACTCAGTTTACGGCGAATACGAAATGGAC

>M.06.2.4_C_22

ATATAGAGACATTTATAGATCTGCTAGATATTATCTATCGT

>M.06.2.4_C_23

GTGTAATAAAATATCTATATAGTAAATTAAAACATCAT

>M.06.2.4_C_24

GATAAGCTACCACAAAACATTCTAAATGAACTGAAAAA

>M.06.2.4_C_25

TTATATACATTAGTTGTAGTATTGACGATTAGTTGTACTGAG

>M.06.2.4_C_26

CTGGTGTGATGTTAGTTATTAAATATGATAGCGGT

>M.06.2.4_C_27

TACTGCTGTATGGAAAAGGTGGGGGGACATAAACCCTAGT

>M.06.2.4_C_28

TTATAATCTTTATATTGTTCTTCTGTTAAATTGTTAAGATC

>M.06.2.4_C_29

AGAGAAGAAATGTACGTCTTAAATAATATATGCAAAT

>M.06.2.4_C_30

GTGAATTACGATACTGATGCACCGGAAGATTTCAGAGCTGAGG

>M.06.2.4_C_31

ATAACATCTCTAAAAATTGTATATGTTTCTTCCGTG

>M.06.2.4_C_32

TTTTTATCCTTCCTTCTTGTTCTTCTATTTGCTTTTC

>M.06.2.4_C_33

ATTTTACTAGATGGTATTTTTATAGATGATTCACTTTT

>M.06.2.4_C_34

CTGCATATTTTAGCTGCTTCCGCAAACTCTTTCCTCCACT

>M.06.2.4_C_35

TTAGATAATCTACTTAATTGGATATATAGTAAATTAGTAT

>M.06.2.4_C_36

CACTTGTCTAAATAATGTAACAAACCTTAGACTACAAT

>M.06.2.4_C_37

ATACTATATTGCCAAAATATGTCATTAGACTTTTTGGC

>M.06.2.4_C_38

CCATCACAGAACCATTGCCATACCCAATCACCGGACATA

>M.06.2.4_C_39

TTATTTACCGCCTACCCCCTGTGATGTCAATTCAT

>M.06.2.4_C_40

AGCTCTACTGGACTTAGTGGCTCAATGCTATTCTGGG

>M.06.2.4_C_41

ATTTCATTAATTCTTTCTGTACGTTCTCTTTTTCATCATAC

>M.06.2.4_C_42

AACTTTTCCAATTAACCCCGCATAATCGATTCCTAATTC

>M.06.2.4_C_43

ATAATTAATGAACTTGTAATTTTTAAACTTGCCGTACCTCCCG

>M.06.2.4_C_44

GGGGAGAAGATGGATAAAGTAGTTAAAAAACAATTGGAATTAT

>M.06.2.4_C_45

AGACGATCCTTACTATAACGCGTTACAAATGCTAGTAA

>M.06.2.4_C_46

ATACCATAATAATTATCAACTATATTCTGCCATAACTTAAT

>M.06.2.4_C_47

TCAGTATCTTTATAACCACCACTTACCAATCTTCTT

>M.06.2.4_C_48

CTACATTTATAATATGTCTCACTTTTAAATACATCATA

>M.06.2.4_C_49

TCATATTATTACTACTTGCGGTAACAGGGCTAAACAA

>M.06.2.4_C_50

TTGAGCAGACCCCCGAGGGTGGGGAGGTTTTATGTTGTC

>M.06.2.4_C_51

ATAGTAACGACATCAATACTTATCATCGTCCACATT

>M.06.2.4_C_52

AGTTAGGCTATCTGCGTGAAACTGCTACTATAACTATT

>M.06.2.4_C_53

CCTAACACCGCAGCACTTAATTTAGGGTCTGCAATTGCT

>M.06.2.4_C_54

ATTTTTGATTATTTCCTTGACATCCTCCTTGATATCTTG

>M.06.2.4_C_55

CTGCTATTAGTAATATCAAATTATAGACCTCTTTTC

>M.06.2.4_C_56

CTTAACATATCAATTACTTCCCCATCCTGTGGACACTT

>M.06.2.4_C_57

AAAAGATAACTATAAGCATTTTTTACATCTGAAAATAACGCA

>M.06.2.4_C_58

TGAAGTAACTGAATAGTTCTAAGCACTGCATAAGTAGTT

>M.06.2.4_C_59

CTCTTCTCCTTTGACTTGTAATACTAATCAATGCCCTAT

>M.06.2.4_C_60

AATCTATCACCACCATTTACTGTCACAATTGATTGGG

>M.06.2.4_C_61

ATAACTTATACTTCATACCGTATTACACACATATAAT

>M.06.2.4_C_62

ATATAGAGATAATCTGAAATGGAACATACCTACATATGGTCAAG

>M.06.2.4_C_63

TAATAGAATTAGGATTACCGATGTCCTTTGTTATGC

>M.06.2.4_C_64

ACAAGAACTATAGTAGTTCAGCAAAATCCGAAGCTT

>M.06.2.4_C_65

TCTTTATCTAGTAAACTAATTAAAAAAAGAAGATCTTCC

>M.06.2.4_C_66

AGAATACTATATAATTCTGCGGGTATTACTGCATTATTCC

>M.06.2.4_C_67

AACATAGCGAACTATCAAGTGTTGAATTATATTGTATTCC

>M.06.2.4_C_68

AGTACCTCATGTAACTCTTTCGGCGTAGCTTTTCCTTC

>M.06.2.4_C_69

AGTTTTTTATTAGGTAAAATTTTTACACTCATTTT

>M.06.2.4_C_70

TTATTTCTTAGTATGATTTCTTTTAATTCATTTCCAT

>M.06.2.4_C_71

TTTTATCAATTCACTCTTGCTTATCCCTAATCTTTTTG

>M.06.2.4_C_72

CTTAAATTATTAAAGCTTGAATATAATATTCTTGCAATAG

>M.06.2.4_C_73

TTATTTCCTTCACTTTCCTCTCCCTCATTTTCATTACT

>M.06.2.4_C_74

ATAATGACTTTCCTCTGTACATTTCAAACTCTTCAACA

>M.06.2.4_C_75

GCATAATAATTTAATAGATCTTCTAAGCCGTCTAAGATC

>M.06.2.4_C_76

AACTTATTATGTATTGTTACCATGTGTACATTTCTAGC

>M.06.2.4_C_77

CTAATATAAACGTAATAATCGTCTGGTTTTTCATTAGTTTG

>M.06.2.4_C_78

GCAATGTTATCAAGATGATAGAACTTATTATTTATTTCAT

>M.06.2.4_C_79

AAGAAAAAATGAAAGACAGTGTACTCGAGGAAGTCC

>M.06.2.4_C_80

GATGAAGTTTGGAAGCAGGGTGTAGCTAGTCTTGG

>M.06.2.4_C_81

CTAGTAAACGCGTTAATCGCGTCACACCTTAACACGCTG

>M.06.2.4_C_82

AATTAAAGCATATTTTTCTATCCGTATTAGTAT

>M.06.2.4_C_83

TATATCATACTCTTTAGCTTTGCCATTAATATCTCAA

>M.06.2.4_C_84

TAAACATATCTAAGACAATACTCATGTAGCAGTCTATTAG

>M.06.2.4_C_85

TTATTGTTTTCCTTATGCTCTTCTGAACTTTCCTCT

>M.06.2.4_C_86

ATTTTAATTGAAGTAAACCCTTCTTCAACTTTATCTTC

>M.06.2.4_C_87

ATAAACTCGTTCTAGGAAAAGAACCACCCTCAAACCTAA

>M.06.2.4_C_88

GCTTTTTAATATAAAATCAGGGGAAAGCGGAATGAATG

>M.06.2.4_C_89

TTTCATCTATACTTACTTCCACAATATTCCCTTTTGGCACAT

>M.06.2.4_C_90

GCGGATTAACACCGGCACTTACGTTCACCCCTTGGCTT

>M.06.2.4_C_91

AATAGTCATTGATATACTTCTTTAAAAACCTCATTCCA

>M.06.2.4_C_92

TTATTTATAATTTTTTCGGGTGCTATAGAATGAAATTTAGAG

>M.06.2.4_C_93

ATGTATACCATGTCAATATAGCCGTTCTGGAACGGGTAG

>M.06.2.4_C_94

AGTTTTTTACATTATCATGAACAAGTAAACCGGAAAC

>M.06.2.4_C_95

AGTTTCTTAAAAGACTCAATATCGTTACATGCACTAATGT

>M.06.2.4_C_96

ATAACTAATCTCTCACATATTTCCCTGCCCAACATATCCCAC

>M.06.2.4_C_97

ATGAACTTTATCTTACCTTGGGATAGCAATTGCTTTATCTC

>M.06.2.4_C_98

AAACAAGAAGAAAAAGAAGTAGATGAGTTTTTTTTATTT

>M.12.37_C_1

CAAGAAATCAGCAAATCCCCCTAAATCTCTTTTGATAG

>M.12.37_C_2

ACTGATTTTGACAATAATAGCGATTGTTATAGGCTCATT

>M.12.37_C_3

TTATACTTCTGTAAACTTCATGCTCGCCCTCACTTCTCTTAG

>M.12.37_C_4

TCCACCCTACCATCTGCATGCACAACGACTAAGTACCCG

>M.12.37_C_5

ATAATAAATTTTCTGTTGATGTAGGTTGTTGATTTAGTGT

>M.12.37_C_6

TTGTCAAACTTCTCTTTATCAGTGACCTGAAACACAC

>M.12.37_C_7

AAACTATATTATCTTTTCAAGATACCTACGATCATGAGCCTGT

>M.12.37_C_8

ATATTTATTAAGAAAGTTCGTGATAGCGTCCTTTGGCGT

>M.12.37_C_9

ACTTAGTCCGTATAAAAGTTTATAACACAAACTCTAGTTC

>M.12.37_C_10

TCTTTCTCTTTTTTATTCTTTTGAATGTAATATCATTT

>M.12.37_C_11

GGGTCACAAGTACCTAATACCCCACAACCACTTTCTAAT

>M.12.37_C_12

GCGTAAGCAAATACACCGTTGATATTTAGTGTTTCACT

>M.12.37_C_13

ACAAGTGAATATACCAGGAATTGCCACACATTCCAACAA

>M.12.37_C_14

TTATCCTTTCGTAAGAACGATTCTAAAGTTTGTTATCGTAA

>M.12.37_C_15

ATAGACAAATGATTTGCTTCCATCATAACTACTACTTCC

>M.12.37_C_16

TAGTAATTCAAAACCATCCTTTTTTAATATTATCTCTATC

>M.12.37_C_17

TTATTAAATACTGTGAAACACTTATGACAATATAGTTTAG

>M.12.37_C_18

TCACATTCTTGGGGGAATTCGTTGCCATGATCAAACAC

>M.12.37_C_19

CTGGTAGTCCATAGCTCTTCTCATAACTTTCTTTCGCTT

>M.12.37_C_20

GTATTTGTTCAATCTGTGAGGGCGATGGGAATGAGCGAAG

>M.12.37_C_21

CAAACGGGCGAGGTTATCAGTCCTGCGCCAGTGCCT

>M.12.37_C_22

TTTCTAGGTCAGATATCGTATATCTCATAGTCTTTGCCT

>M.12.37_C_23

TTTACTTCTCTTTTAAGGTACTTTTTATGGCCTTATCCC

>M.12.37_C_24

CTTAGCTTTTAGCTAAGCTAAATAATAGTGTCTAGAATTT

>M.12.37_C_25

TCAAAAATACGAGAAAACTTATGAAGCTGATATACCTTACGTTAGAAG

>M.12.37_C_26

TATGTATTAATAAAAAAACAATACCGTCATTACTTAGT

>M.12.37_C_27

AACAGTAGATGTTTGCACGGAAGAAACATATATAATTT

>M.12.37_C_28

AAGTCTACTTGTACATGTCAAAAGACGCTAGAATGAGA

>M.12.37_C_29

TCACAAATCTTCGTAAACTACGATACATTTCCTTAC

>M.12.37_C_30

AGAAAGTGGGATACAGAAGCCTCAAGACGATCCTTACT

>M.12.37_C_31

ATTCTGAAAATTGCCTATTATACCAAAGCCAAAAACTC

>M.12.37_C_32

TTAATTATATACCTATGCATTCCTTCTATGTTAAGATG

>M.12.37_C_33

AATTCATATTTTTGCTGTCGTAGTTGCTGTCATAGCAATT

>M.12.37_C_34

AAGGATATATTGACATAGATGACGAAGATCTTTACTT

>M.12.37_C_35

ATCGATTCTAACACCTTGACCATATGTAGGTATGTTCC

>M.12.37_C_36

CTATTCCTGACTGCCCATAAAATACGTATAGTAGAT

>M.12.37_C_37

GTCCTTTCCATATACTTTTTGAATTTTTTGCATTCCTG

>M.12.37_C_38

CCCTGCCTCTTCATGGCAGAGGACCTGGGTCGGGCCGC

>M.12.37_C_39

TTGTCATTACTCAGACCGCTATATATTCCGACATCATAT

>M.12.37_C_40

AATTTTAGCGGGTCTTCGACCAATTTACTTGAATGTGTTT

>M.12.37_C_41

GTAATATCTGCCATGTCATCAGAGCAAATACC

>M.12.37_C_42

CACACACACACCCCTATTAAATTCGAAAGCACGTT

>M.12.37_C_43

AATGTAGTAACTGTATTTAGAACCCGTTAATTTTACGCT

>M.12.37_C_44

CTCTTTGTTGTTATACTGATTCACAAAATTTTGCTTAG

>M.12.37_C_45

TTATTTGAATTGCATCTTGTTCGCTTATTAAACCTAT

>M.12.37_C_46

ATGAGTTTATAGGGAAAGAGGACAAAGCCAAAGAGTTGA

>M.12.37_C_47

GTGTTAAATTCTCAAAGATAATATCTAAAAACGTAGGT

>M.12.37_C_48

ATTATTTATAAGCTTTAAACTCGCTGATCACAGCTTATT

>M.12.37_C_49

TATATATATCTTTTGTTATTATCGTGGTTTTCATATGGATTA

>M.12.37_C_50

TTTAATACTAATGTTGCTTGTATATGTTTACTTAGT

>M.12.37_C_51

TAACGCAGCTGAAGATGAACCAATCGTGATTAACTTA

>M.12.37_C_52

TTAGTTATCTGAAATGTTAACACCCATAAGAATGCTAT

>M.12.37_C_53

ACTTAAAGATATAGATGAACTAGCACGGCAATATAA

>M.12.37_C_54

AAAGTTATCAGTTGGGTACAAAAACAATCCACAAGG

>M.12.37_C_55

ACAGTTGGGGATACCACAATATAATCCATAGGTTGTAC

>M.12.37_C_56

CTGGGAGGGACATGGGGGCGTAACCGTTTCGTTTCCCAGA

>M.12.37_C_57

TGAAACCAACAATATCCTTTGTATTAATACTTTTAC

>M.12.37_C_58

GTTACGGTTAGTTTTTCCTTTTTCGCCAAAAAATCAA

>M.12.37_C_59

ATTATTAAAATGGCAACTACATGTACTGACCTACTTCAAT

>M.12.37_C_60

CCCATGGGCTTTTGCCCATGGTTTCATTGAGCAGACCC

>M.12.37_C_61

ATCGTATCGGTGAGGAAGAAAAAATTGAGGGCGATAAGG

>M.12.37_C_62

GTATGGTTTCTTCAGGATAGTTAAGTTTCAACATCTTCTCA

>M.12.37_C_63

AATTTATTAAGGTTGAAGATTATCAAGACAAGACAATAA

>M.12.37_C_64

TAGAAGATAGCGAATTAAGTCGCTTTCGTCTGCACCCA

>M.12.37_C_65

GACATGTACAACAGAGTTTTGCCCATTACATTTGTATTT

>M.12.37_C_66

CTCCTCAATATCTTATTCTTTATACAATAACTATCTA

>M.12.37_C_67

TCTTCTAATAAGTCCATGCCCTCTGCATCAGAGGGCAT

>M.12.37_C_68

TCAATTGCTTTGGCGACGTCAAAGGCTAAATCGCCT

>M.12.37_C_69

ATACTAGCCGGCTGGTTTCTACTGGCGGATATACCCCCA

>M.12.37_C_70

TTTATATTAAGTAACCTCAATAAGTCATTACTGTTTATTAACT

>M.12.37_C_71

TAGTATATCTATTTTCATATTTATACACCCGAAAAAAAG

>M.12.37_C_72

AATAGTGCATCACTAATTATTAAACTTTTTAACTTACATGT

>M.12.37_C_73

AGAATACTATATAATTCTGCGGGTATTACTGCATTATTCC

>M.12.37_C_74

AGATATAATTTTGCATTTTTCTTGTTCCACTCTTCTATTA

>M.12.37_C_75

TTATAGTCTTTATATTGTTCTTCTGTTAAATTGTTAAGATC

>M.12.37_C_76

CCGTCAAGGTGCTGTTCTTACAGGATCCGGAAATAGTGGC

>M.12.37_C_77

TAAACGCTAGCAAAGTATCGCTTTACATTATCAATAACGATTC

>M.12.37_C_78

ACGCTGCAACTATGCCGTTTTTCTATGCTCTACCATTCCC

>M.12.37_C_79

GCATATTATTACTACTTGCGGTAACAGGGCTAAACAA

>M.12.37_C_80

AGGATTAGGTTTTGGGTTGTCTATATCAATAGAAATAAGC

>M.12.37_C_81

AACTTTTCAAATAGTTTGCAAGCATATAATCTATACTTAT

>M.12.37_C_82

TTATCATACCTAGAAAGTAGTAAACATAAGAGAAAATCCT

>M.12.37_C_83

CTTTGTGTCTCCCCGTTTCTCCCGGTCAGTAAAACTACT

>M.12.37_C_84

TTTTCAGCTCTTGCGAGAGCTAAAAAAAGACGTGTGTT

>M.12.37_C_85

TACTTCTCACCATGATGTGGGTAATGTCCACCATTTTGCA

>M.14.16_C_1

TAGTAGAAGGTATAGCACCTAACGCAAACGCAATAGAA

>M.14.16_C_2

ATGGATAACCTGCTGCGGCGCTTGAGAAATGTGAGAA

>M.14.16_C_3

CTTTATTTAGGTACTCCCCTTGCTTTAAATTGTCCTTT

>M.14.16_C_4

AAGTAAAGAATAGCAAATATAATCCTACCCATTCCCGCTCA

>M.14.16_C_5

CAGATCTATTTACTGGAGCAGGAGCTGCAGGGAGAGAGCTA

>M.14.16_C_6

TACTTTAGTCTTATGAGATTTTTAATGGCTCTTATACT

>M.14.16_C_7

ATGAAAGGTTTTACGTTAAAATCACTTCCGCCCGGCA

>M.14.16_C_8

TTCCAAATACACGTTTATTCAATATGATATCCAGCAAC

>M.14.16_C_9

TTAAAACATTAATTCTTTTGCTAGAGTTAAATGTTATTT

>M.14.16_C_10

ACAATTAACAAAACTTCAGCAACTCATTCATCAAAAA

>M.14.16_C_11

TTATAATCTTTATATTGTTCTTCTGTTAAATTGTTAAGATC

>M.14.16_C_12

ATATATACAGAATAACGGTATTAAAGATGCTGACTATACGC

>M.14.16_C_13

GATGAATATGAAGAATTTGCTACTATAATAGTGAAACTCC

>M.14.16_C_14

GTATTGACATGTTCACGCCTTTTATGAAGCCTGACAAA

>M.14.16_C_15

ATTACTATTTGACGTTACACCTTCCTTCCAAAACGTA

>M.14.16_C_16

GTTATTCGCGGTCTCATTATAAAATTCAAAGCATATTCTAA

>M.14.16_C_17

AAATTAATCTCTAGAATCTCAGATAGTGGCACATATCTT

>M.14.16_C_18

AATAGTGCATCAGATATTAAGAGACTTCTAACTTTAC

>M.14.16_C_19

ATTGTAAATACTATTTTTGTAGTACAAAGTGAAATCTAAT

>M.14.16_C_20

TAGACAGGTCAGTATAGGTCTTACCATCTAATAAAGCAAG

>M.14.16_C_21

TTTGCAATATAGTATGGTTGAATACCCCAACCGTACAAA

>M.14.16_C_22

TTAATGAAAACATCTCTGTCAACTCTAATTAAATTCAG

>M.14.16_C_23

GTTAAAAGCGTAACTAGATGATTGGGTTGGAACGTATAT

>M.14.16_C_24

AGAGGGAGCCTTCGGGGTGTGGCTTCCCCCCTCCCATTTTCC

>M.14.16_C_25

TGTTCAATCACTTGTATTCTTTTGTGTAAATTTCGC

>M.14.16_C_26

ATGCTATTCCAAGGTGACTAGATATGATAGAGATGAAG

>M.14.16_C_27

TATACCTTATGCTGATGTCTCTGAGCTTCCTGATATCA

>M.14.16_C_28

GTTCACTTCATTTGTAAGATATGCTACAGTTTGTAAATTG

>M.14.16_C_29

CTATATATGGGAAGGCTTATGATCACCTAGTAGAA

>M.14.16_C_30

AATTCATGATCAACGTAGGTTTCTACAGCGAGTTCAAGAA

>M.14.16_C_31

TAATTAAATCTAGATAACGAAGGTATTTAACATTTTGT

>M.14.16_C_32

ATAAACGATAGGCTTCAGTCCCTCAAAGCGAGAAACGA

>M.14.16_C_33

ACTTATCAACCAATTGAATATTTGAATCATTACTAGG

>M.14.16_C_34

AGTATTACTTTGTACGAAAACGGGGCTGCAAACATTAG

>M.14.16_C_35

TTTTTTCCACCTCGCTAGAAAACTCCCTACCCTGATTTGC

>M.14.16_C_36

AAATTACTATCTTGAATAGAGATATTAGCGTTGTCTATTGCT

>M.14.16_C_37

GCAAGGCAGTGGACAATATGCTCTAGGGACAGAATATACGC

>M.14.16_C_38

AGATTTAACAGAATAGTAGATATTACCAGGCTGTATTATT

>M.14.16_C_39

TAGATGACCCTGCGAGTTGGATATCGAAATGGCAAAAT

>M.14.16_C_40

TTCTGTTAGCCTGCCTCCTTCATAGGCTAAAATAACAG

>M.14.16_C_41

TGACCTACACTCAAATCCGAATTGTTTACATTCCTCAA

>M.14.16_C_42

ACTATCATTCCTTGTTTATAAAGTCCAAAAGCATATAT

>M.14.16_C_43

TCTATAAATATTAGATACTTACCATTACACTCTTCTAC

>M.14.16_C_44

ATAATTTGTATCGATGCTTTTCCATTATCATCATATGAAAT

>M.14.16_C_45

TATTTACTAGCATAGTTTCCAAATACAGGCTCGA

>M.14.16_C_46

CTTTCACTCTTCTTCTTTATCTCATCAAGTTTATTGT

>M.14.16_C_47

TATTTCACTTCTTAGCAACTGCAATGACTATCACAAGT

>M.14.16_C_48

AATAAATTCATTACTTTCCTTAGATCTCCATCTGATAAAT

>M.14.16_C_49

TATTAATGATCTTGAATTCAGTAATTGCAATGAGATCAT

>M.14.16_C_50

TTCATCACCCCATGTTTCTTCAAATCCACCCAGATTCTC

>M.14.16_C_51

CAAGGTGTCGCTGCTGCGCAACCCAAGATGGCTGCAGTA

>M.14.16_C_52

AAATATGAGAGATTCACATCTTTTATAATCTTCACC

>M.14.16_C_53

TTGATGGGTCACCTCGTGATATTAAGGTAAATATCAACT

>M.14.16_C_54

ACATTAACATTATTGCTTTTTATCCAAGGATATTTCCA

>M.14.16_C_55

TTGTAATACTTCTATTTTCATATTTATACACCCGAAAA

>M.14.16_C_56

AAGAATCCCCAAGGATAAACACTAGCATATCGACC

>M.14.16_C_57

ATAGGATAGAAGAACTTCCTAACGAAATCTTCATGCCTTT

>M.14.17_C_1

CCAATAAACCCCTACTGCTTACGTGGCTTAATACGTTAT

>M.14.17_C_2

CTTACTTTTTCATGGCACATTCTTGACGCTTGACCGAAC

>M.14.17_C_3

TATTCTAATAATGGAAAGCTACTATGATTTTGTGCAT

>M.14.17_C_4

ACGAGTTTGTAGAGTTCAAACGTCTGGTTGAGTATGTT

>M.14.17_C_5

TTCAAAGCTCTAAGCAGGGAGTTAGCCAACGCCTTGTT

>M.14.17_C_6

TAGTATTTATAGCTTCTTTTATCGTAAAAACACCTTTC

>M.14.17_C_7

AAGTTAGCCGAGGTAGTGATTAGCCATGGATAGATT

>M.14.17_C_8

GAGACTTCTACATCTTGTCCGCTCATCTGTGTTCTTTT

>M.14.17_C_9

TTGTCTAAGCTAAAAGCTAAGCAAAAAGCTTATATACT

>M.14.17_C_10

TTATATCAATAATGATGGTCTTATCACTGCTGTTCTTCTTCC

>M.14.17_C_11

TTTGTATAATAGAAACTTGCCTTCCTAAATCTCAGTTT

>M.14.17_C_12

AATAATTTCTTTGCTTTTTCTTCCTCTTGTTGTAATAATT

>M.14.17_C_13

AATACATTAGCTATACTTGGATATCCAAGATTTATACCT

>M.14.17_C_14

TTATAGTCTTTATATTGTTCTTCTGTTAAATTGTTAAGATC

>M.14.17_C_15

GATTTATCAAAAAGCGGAATAACGAAGACGCCAAATTC

>M.14.17_C_16

AAAAAGTCCACGCTATAATCCCATGAATGTTCGAT

>M.14.17_C_17

GAGGTTGGACGGCTAACGAATATCCCGCATTAGGCGGAT

>M.14.17_C_18

TTGATATCTAGGGCGGGCGTTAAATTCGTAGAGGCGG

>M.14.17_C_19

CGTAATCGTTCCAACGGGCAATAATTCTATATATCGT

>M.14.17_C_20

TTCAAACTCCGGGTCTGTCTCTTCGTAATTAATTTTCTTT

>M.14.17_C_21

ATATCTTGACTCATATTTACACCCGAAAAAATCAAT

>M.14.17_C_22

TAATTATCAATAATCCATTGAGTTATTGCATAATATCCTT

>M.14.17_C_23

TTTTTCGCTAAAGTGAGTAAATCTAGGTAATGCTTCGA

>M.14.17_C_24

ATTTAAATTTCCAGTACCATAAGCAACTGCTTGTCTATT

>M.14.17_C_25

ATCCGTATATAGTAAATAATGCCACCTAAATCTATTTT

>M.14.17_C_26

TTTTTTAATATTTTTCTAATTTCATCACTGATTTC

>M.14.17_C_27

GTAATTTTACTAACTCGTTACAAGCTTACAATCTATATCTG

>M.14.17_C_28

TCTAATTTGAATGTTATAACCCTGTTCATAAGTATT

>M.14.17_C_29

TTTATTATATATAACAATACTATATCCTGCGTCCTTTAGT

>M.14.17_C_30

CTTTCGTCTGCACCCATTTTTTTGCTTAACTCTTCTAGCAT

>M.14.17_C_31

ACGTGTTATATCTATATCTGTCTTCAGCATGTAACGATTT

>M.14.17_C_32

GTATAGAACAACCACTGCCTAGCACTGTTTGGGTGTCCAT

>M.14.17_C_33

ATAACAACTTCGCCTCCCCGTCGTCCTTAACGAACTTCA

>M.14.17_C_34

TTTCGCTTCAGAAAAGTCACTTTAGATCACTTTATATA

>M.14.17_C_35

AACACTATTCTCTTCCTTACCTCTTTTACGTCCTC

>M.14.17_C_36

AAATTATATGGCCTGAAGTGGGTGTTAAGTACGACAAT

>M.14.17_C_37

GAACTATTGCACCTTTATAAGTATCCTCTTCTTCAGTTTTCTTA

>M.14.17_C_38

CTTTTGCGTAATAAATTTATACGTCCAATCGGGCATTCTT

>M.14.17_C_39

TTAAAATTAGATGATTTGTTAGGCATGTTTGGCTATCCT

>M.14.25_C_1

CCAATAAACCCCTACTGCTTACGTGGCTTAATACGTTAT

>M.14.25_C_2

CTTACTTTTTCATGGCACATTCTTGACGCTTGACCGAAC

>M.14.25_C_3

TATTCTAATAATGGAAAGCTACTATGATTTTGTGCAT

>M.14.25_C_4

ACGAGTTTGTAGAGTTCAAACGTCTGGTTGAGTATGTT

>M.14.25_C_5

TTCAAAGCTCTAAGCAGGGAGTTAGCCAACGCCTTGTT

>M.14.25_C_6

TAGTATTTATAGCTTCTTTTATCGTAAAAACACCTTTC

>M.14.25_C_7

AAGTTAGCCGAGGTAGTGATTAGCCATGGATAGATT

>M.14.25_C_8

TTAAAATTAGATGATTTGTTAGGCATGTTTGGCTATCCT

>M.14.25_C_9

AATAATTTCTTTGCTTTTTCTTCCTCTTGTTGTAATAATT

>M.14.25_C_10

TTGTCTAAGCTAAAAGCTAAGCAAAAAGCTTATATACT

>M.14.25_C_11

GAGGTTGGACGGCTAACGAATATCCCGCATTAGGCGGAT

>M.14.25_C_12

CTTTCGTCTGCACCCATTTTTTTGCTTAACTCTTCTAGCAT

>M.14.25_C_13

AATACATTAGCTATACTTGGATATCCAAGATTTATACCT

>M.14.25_C_14

GATTTATCAAAAAGCGGAATAACGAAGACGCCAAATTC

>M.14.25_C_15

TTATATCAATAATGATGGTCTTATCACTGCTGTTCTTCTTCC

>M.14.25_C_16

TTTGTATAATAGAAACTTGCCTTCCTAAATCTCAGTTT

>M.14.25_C_17

TTGATATCTAGGGCGGGCGTTAAATTCGTAGAGGCGG

>M.14.25_C_18

CGTAATCGTTCCAACGGGCAATAATTCTATATATCGT

>M.14.25_C_19

TTCAAACTCCGGGTCTGTCTCTTCGTAATTAATTTTCTTT

>M.14.25_C_20

ATATCTTGACTCATATTTACACCCGAAAAAATCAAT

>M.14.25_C_21

TAATTATCAATAATCCATTGAGTTATTGCATAATATCCTT

>M.14.25_C_22

TTTTTCGCTAAAGTGAGTAAATCTAGGTAATGCTTCGA

>M.14.25_C_23

ATTTAAATTTCCAGTACCATAAGCAACTGCTTGTCTATT

>M.14.25_C_24

ATCCGTATATAGTAAATAATGCCACCTAAATCTATTTT

>M.14.25_C_25

TTTTTTAATATTTTTCTAATTTCATCACTGATTTC

>M.14.25_C_26

GTAATTTTACTAACTCGTTACAAGCTTACAATCTATATCTG

>M.14.25_C_27

TCTAATTTGAATGTTATAACCCTGTTCATAAGTATT

>M.14.25_C_28

TTTATTATATATAACAATACTATATCCTGCGTCCTTTAGT

>M.14.25_C_29

AAAAAGTCCACGCTATAATCCCATGAATGTTCGAT

>M.14.25_C_30

ACGTGTTATATCTATATCTGTCTTCAGCATGTAACGATTT

>M.14.25_C_31

GTATAGAACAACCACTGCCTAGCACTGTTTGGGTGTCCAT

>M.14.25_C_32

ATAACAACTTCGCCTCCCCGTCGTCCTTAACGAACTTCA

>M.14.25_C_33

TTTCGCTTCAGAAAAGTCACTTTAGATCACTTTATATA

>M.14.25_C_34

AACACTATTCTCTTCCTTACCTCTTTTACGTCCTC

>M.14.25_C_35

AAATTATATGGCCTGAAGTGGGTGTTAAGTACGACAAT

>M.14.25_C_36

GAACTATTGCACCTTTATAAGTATCCTCTTCTTCAGTTTTCTTA

>M.14.25_C_37

CTTTTGCGTAATAAATTTATACGTCCAATCGGGCATTCTT

>M.14.25_C_38

GAGACTTCTACATCTTGTCCGCTCATCTGTGTTCTTTT

>M.14.34_C_1

TTCTACACTTTCAGAGAACATAGTTTCTTTATTTACA

>M.14.34_C_2

TTAGCTAGATCAGCTGCTTCAGCTTCTCTCAGCGCTCTC

>M.14.34_C_3

ATTTAAATTATATTTTAGGGCGTATCTGTCTAGCAGTTGTA

>M.14.34_C_4

TTAAGTCCTTTGATGTGACAGATTATATCAAAATTGAT

>M.14.34_C_5

GTATAATCTTCAACAGACATATTTTACTTTTATTATTACT

>M.14.34_C_6

TCGTATATCTGTACAATCTTCAATTCGTTAAGTAGTTTC

>M.14.34_C_7

ATATTTTCAACTCTATAGCCGTTTTTCATGCATTCTTCTGAT

>M.14.34_C_8

TCAAAATGAGTAATTTTTCTTCATGCAACATAATTCAC

>M.14.34_C_9

ATAACTCTACTACATTTTCTCATGTAAATTCTAGTGCAT

>M.14.34_C_10

CTTGAATAAGCACTTATAGAATTTTTATTAATAGCTTCAG

>M.14.34_C_11

ATAGTCACGTATATTGGATACCCAAGCTTCTCATAATTA

>M.14.34_C_12

TAGACAGGTCAGTATAGGTCTTACCATCTAATAAAGCAAG

>M.14.34_C_13

TTATAGTCTTTATATTGTTCTTCTGTTAAATTGTTAAGATC

>M.14.34_C_14

ATTACACTTAAGATTGTTGCTTTTTTACTATTCTG

>M.14.34_C_15

TTAGAACAAATCGTAAATGAGTATTATGATATGAAGTGCC

>M.14.34_C_16

ACTTTATACTGTAGATATTTACCGGGTAGGACAAAGTAT

>M.14.34_C_17

TGATAATCTTCAACCTTAATAAATTCGAAATAAATATC

>M.14.34_C_18

ATAGATAGAAATTACTCTCTACTGGATCCGCAACTTC

>M.14.34_C_19

TTATTATTTACAGTTATTCTTATCTTTGCCATCCTCACCAC

>M.14.34_C_20

TTAGGAAGAAGGGGGACTACTGTAGTGTGTGTTACAATA

>M.14.34_C_21

TCTTTCACATACGTTTCTATACGCTTCTTCTTTCCATC

>M.14.34_C_22

ATAGCTTCTTTAATGTATTTCTGTGTCACTTTGTCTTT

>M.14.34_C_23

TTAGTATATCATATGCTTCCTTCATCATGTCAATATC

>M.14.34_C_24

AGGATCTCAAGACATATGGTAACAAGTTTGTTTTCTT

>M.14.34_C_25

CTAAACCTATCAGAGTTAGCAGAATATAGTAATATTGATATT

>M.14.34_C_26

ATGCTTTCGTTTTGTGTTTTTTCAATTCCTTTTAGGATTAAT

>M.14.34_C_27

CCGCCCTACTTGTTTTTATATTAAATGCTATTCCC

>M.14.34_C_28

ACAACAAGAAGTACGATACATAACTGTGCCGTCGTTGA

>M.14.34_C_29

TCTTGTCTAGTTCATCTAACAATTTTAATAGCGTCTCGA

>M.14.34_C_30

TTTAATCTTATTTCTTATGTCTATGCCTTCTTCACGT

>M.14.34_C_31

TTAATAAAACCAAACTTATTGCATTTAATAACGGCGTT

>M.14.34_C_32

ATCAGTATTGGGAAGTAGATTGCTTTGCCTTTATTCTCT

>M.14.34_C_33

ATTTCGTACATCAAGTATTTTGATGACAGTAAGAAATATTG

>M.14.34_C_34

ATGCTTTCGTTTTGTGTTTTTTCATTTACTTGTTCTT

>M.14.34_C_35

AATTCCTTTATCTTTGCTTCCAGCTCTTCTATCTTCTTT

>M.14.34_C_36

ATTTAAATTCCTTTTAGGATTAAT

>M.14.34_C_37

TTGTTACTCAAATATATTCACCTCCATCTTTTGT

>M.14.34_C_38

TTCAAATACCCTAATTGTTCTCTCTACCTCATTTAGATC

>M.14.34_C_39

ATTTCTGTCTTATGTAGCTTCTTAGCTTCGTTTAACCAT

>M.14.34_C_40

CATATATAGTGAATTTCTTTTGAGATAATAGATTAT

>M.14.38_C_1

TTATAGATCCAGCCCTAGATCTAGTTTCTTTTATCTCT

>M.14.38_C_2

ATATAATAGATATCCTATTATTGCTATGATGATGAT

>M.14.38_C_3

AGAAGAATATGTAGAACCTATTCTTTCTCTCTATGATCAC

>M.14.38_C_4

TACAGGGGTCATACTTGCTAGATTTATTACAACATCTA

>M.14.38_C_5

GTAGGTGTCAGTACTGGAAACAGATCGTCGAACCTTCCTG

>M.14.38_C_6

ATAAATCTACATTATTATACAAAACTTGTAAAATTGTATTT

>M.14.38_C_7

CTAATACTAATGTGTATGGCTCTTTTTTCGCTATATTCG

>M.14.38_C_8

TCATTATCTTTCAATTCCTTTTAGGATTAAT

>M.14.38_C_9

TCAGTATGAGGGTCTTCAGTAACGTCGTCTTTCCCG

>M.14.38_C_10

CGCACTTGCAGCAATTAGAGAATTCTACGATGCCCCT

>M.14.38_C_11

TTGAGAACCTTTCCTTCTTCAAGCCTCACTGCTATATTA

>M.14.38_C_12

TTATACTTTTTCTAATTGCTTTCTGAATCTTTCTTTTCT

>M.14.38_C_13

ATATAATGAAGTATACGGATATACTGCATCAAATTTAT

>M.14.38_C_14

TTTTTGTTCTTCTTGTTCTTCTAATAGCCCATCTTC

>M.14.38_C_15

TATTAAAGCATATTTTTCTATCCGTATTAGTAT

>M.14.38_C_16

GTTCACAAAGAGGCAAAGCAGTTCTAGGTTACACGGA

>M.14.38_C_17

AAACAAGAAGAAAAAGTATAACTTTCAATTCCTTTTAGGATTAAT

>M.14.38_C_18

TTATTTATAATTTTTTCTTCTTTCAATTCCTTTTAGGATTAAT

>M.14.38_C_19

TAGGTGCAGGCGCAGTAGCGATATATTTAATATCTA

>M.14.38_C_20

TGAATTATAAAACTCACAATTGCACCTCTGTACCTGATAATT

>M.14.38_C_21

AGACGATTATTACGTTTATATTAGCTTTCAATTCCTTTTAGGATTAAT

>M.14.38_C_22

CTACTTTATACTGTAGATATTTACTGGGTAGGACAAAGTAT

>M.14.38_C_23

ATTCAATATTATGCATTTCCCCTTACGTCCCCTCAAGT

>M.14.38_C_24

GTGGAAACTCAGTTTACGGCGAATACGAAATGGAC

>M.14.38_C_25

ATATAGAGACATTTATAGATCTGCTAGATATTATCTATCGT

>M.14.38_C_26

TTTCAACTCTGCTATCCTATTTTCATATTCGTTT

>M.14.38_C_27

GTGTAATAAAATATCTATATAGTAAATTAAAACATCAT

>M.14.38_C_28

CTAACAGGTATCTAAAAACAAACACACACTCGCTTCGTTAT

>M.14.38_C_29

GATAAGCTACCACAAAACATTCTAAATGAACTGAAAAA

>M.14.38_C_30

TTATATACATTAGTTGTAGTATTGACGATTAGTTGTACTGAG

>M.14.38_C_31

CTGGTGTGATGTTAGTTATTAAATATGATAGCGGT

>M.14.38_C_32

TACTGCTGTATGGAAAAGGTGGGGGGACATAAACCCTAGT

>M.14.38_C_33

TTATAATCTTTATATTGTTCTTCTGTTAAATTGTTAAGATC

>M.14.38_C_34

TCATATTATTACACCTTTCAATTCCTTTTAGGATT

>M.14.38_C_35

AGAGAAGAAATGTACGTCTTAAATAATATATGCAAAT

>M.14.38_C_36

GTGAATTACGATACTGATGCACCGGAAGATTTCAGAGCTGAGG

>M.14.38_C_37

ATAACATCTCTAAAAATTGTATATGTTTCTTCCGTG

>M.14.38_C_38

TTTTTATCCTTCCTTCTTGTTCTTCTATTTGCTTTTC

>M.14.38_C_39

ATTTTACTAGATGGTATTTTTATAGATGATTCACTTTT

>M.14.38_C_40

ATCTATAAATGTCTCTATATCTTTCAATTCCTTTTAGGATTAAT

>M.14.38_C_41

CTGCATATTTTAGCTGCTTCCGCAAACTCTTTCCTCCACT

>M.14.38_C_42

TTAGATAATCTACTTAATTGGATATATAGTAAATTAGTAT

>M.14.38_C_43

ATAGAGTTGCAAGCCCGAAGGCTCCCTCTCGACCTCCCTCG

>M.14.38_C_44

CACTTGTCTAAATAATGTAACAAACCTTAGACTACAAT

>M.14.38_C_45

ATACTATATTGCCAAAATATGTCATTAGACTTTTTGGC

>M.14.38_C_46

CCATCACAGAACCATTGCCATACCCAATCACCGGACATA

>M.14.38_C_47

TTTACGTAGGAGCTTCCGTTCAGCTGTATTACTCCTCCT

>M.14.38_C_48

AGCTCTACTGGACTTAGTGGCTCAATGCTATTCTGGG

>M.14.38_C_49

ATTTCATTAATTCTTTCTGTACGTTCTCTTTTTCATCATAC

>M.14.38_C_50

AACTTTTCCAATTAACCCCGCATAATCGATTCCTAATTC

>M.14.38_C_51

ATAATTAATGAACTTGTAATTTTTAAACTTGCCGTACCTCCCG

>M.14.38_C_52

GGGGAGAAGATGGATAAAGTAGTTAAAAAACAATTGGAATTAT

>M.14.38_C_53

TTATTTACCGCCTACCCCCTGTGATGTCAATTCAT

>M.14.38_C_54

GACAGCCGCTTTCCAAGATTAACCTGCTTGAGGAACTCAA

>M.14.38_C_55

AGACGATCCTTACTATAACGCGTTACAAATGCTAGTAA

>M.14.38_C_56

ATACCATAATAATTATCAACTATATTCTGCCATAACTTAAT

>M.14.38_C_57

TCAGTATCTTTATAACCACCACTTACCAATCTTCTT

>M.14.38_C_58

CTACATTTATAATATGTCTCACTTTTAAATACATCATA

>M.14.38_C_59

TCATATTATTACTACTTGCGGTAACAGGGCTAAACAA

>M.14.38_C_60

ATTTTTTCTTTATTTCTTAGTATGATTTCTTTTAA

>M.14.38_C_61

TTATTTATATAATCATTGAAACATTCGATATAATAATCTT

>M.14.38_C_62

TTGAGCAGACCCCCGAGGGTGGGGAGGTTTTATGTTGTC

>M.14.38_C_63

ATAGTAACGACATCAATACTTATCATCGTCCACATT

>M.14.38_C_64

AGTTAGGCTATCTGCGTGAAACTGCTACTATAACTATT

>M.14.38_C_65

CCTAACACCGCAGCACTTAATTTAGGGTCTGCAATTGCT

>M.14.38_C_66

ATTTTTGATTATTTCCTTGACATCCTCCTTGATATCTTG

>M.14.38_C_67

CTGCTATTAGTAATATCAAATTATAGACCTCTTTTC

>M.14.38_C_68

CTTAACATATCAATTACTTCCCCATCCTGTGGACACTT

>M.14.38_C_69

AAAAGATAACTATAAGCATTTTTTACATCTGAAAATAACGCA

>M.14.38_C_70

TGAAGTAACTGAATAGTTCTAAGCACTGCATAAGTAGTT

>M.14.38_C_71

TAAAACAGGGAGACATTCTTCTACTCTGTGAACAACGT

>M.14.38_C_72

AATCTATCACCACCATTTACTGTCACAATTGATTGGG

>M.14.38_C_73

ATAACTTATACTTCATACCGTATTACACACATATAAT

>M.14.38_C_74

ATATAGAGATAATCTGAAATGGAACATACCTACATATGGTCAAG

>M.14.38_C_75

CTAATATAAACGTAAGGATCGTCTCTTTCAATTCCTTTTAGGATTAAT

>M.14.38_C_76

TAATAGAATTAGGATTACCGATGTCCTTTGTTATGC

>M.14.38_C_77

CCTTTTAGGATTAATCTATGTCCGGT

>M.14.38_C_78

TTTCAATTCCTTTTAGGATTAATCACCGGACATA

>M.14.38_C_79

ACAAGAACTATAGTAGTTCAGCAAAATCCGAAGCTT

>M.14.38_C_80

GTAGCCAAATATAAACAACTGCTGGCCGTGAGATGGATAAT

>M.14.38_C_81

TCTTTATCTAGTAAACTAATTAAAAAAAGAAGATCTTCC

>M.14.38_C_82

AGAATACTATATAATTCTGCGGGTATTACTGCATTATTCC

>M.14.38_C_83

AACATAGCGAACTATCAAGTGTTGAATTATATTGTATTCC

>M.14.38_C_84

AGTACCTCATGTAACTCTTTCGGCGTAGCTTTTCCTTC

>M.14.38_C_85

AGTTTTTTATTAGGTAAAATTTTTACACTCATTTT

>M.14.38_C_86

CTTAAATTATTAAAGCTTGAATATAATATTCTTGCAATAG

>M.14.38_C_87

ATAATGACTTTCCTCTGTACTTTCAATTCCTTTTAGGATTAAT

>M.14.38_C_88

TTATTTCCTTCACTTTCCTCTCCCTCATTTTCATTACT

>M.14.38_C_89

ATAATGACTTTCCTCTGTACATTTCAAACTCTTCAACA

>M.14.38_C_90

AAAGAAGCATCATGGAAGTTGCTGGCGTTAGTAGCTC

>M.14.38_C_91

ATAATGACTTTCAATTCCTTTTAGGATTAAT

>M.14.38_C_92

TTGATATAATACGTCACATCAATCGTTCTTCTTCTCGAT

>M.14.38_C_93

AAGAAAAAATGAAAGACAGTGTACTCGAGGAAGTCC

>M.14.38_C_94

CTAATATAAACGTAATAATCGTCTGGTTTTTCATTAGTTTG

>M.14.38_C_95

GCAATGTTATCAAGATGATAGAACTTATTATTTATTTCAT

>M.14.38_C_96

AACTTATTATGTATTGTTACCATGTGTACATTTCTAGC

>M.14.38_C_97

GATGAAGTTTGGAAGCAGGGTGTAGCTAGTCTTGG

>M.14.38_C_98

TATATCATACTCTTTAGCTTTGCCATTAATATCTCAA

>M.14.38_C_99

GTAATCGCTGGGATTCCTCATTATCAGATAATCGT

>M.14.38_C_100

CATTCTTAAAAATATATGCCAATATCTTATATAATTCT

>M.14.38_C_101

ATAAACTCGTTCTAGGAAAAGAACCACCCTCAAACCTAA

>M.14.38_C_102

TATTTACCTAAAAAAAGGGTTGAAAGAAAGACTGCACT

>M.14.38_C_103

GCGGATTAACACCGGCACTTACGTTCACCCCTTGGCTT

>M.14.38_C_104

AATAGTCATTGATATACTTCTTTAAAAACCTCATTCCA

>M.14.38_C_105

TTATTTATAATTTTTTCGGGTGCTATAGAATGAAATTTAGAG

>M.14.38_C_106

ATGTATACCATGTCAATATAGCCGTTCTGGAACGGGTAG

>M.14.38_C_107

AGTTTTTTACATTATCATGAACAAGTAAACCGGAAAC

>M.14.38_C_108

AGTTTCTTAAAAGACTCAATATCGTTACATGCACTAATGT

>M.14.38_C_109

AAACAAGAAGAAAAAGAAGTAGATGAGTTTTTTTTATTT

>M.16.12_C_1

CCAATAAACCCCTACTGCTTACGTGGCTTAATACGTTAT

>M.16.12_C_2

TTTTCTATGAGAGAACCATTTAAGAACCTAGATTACGT

>M.16.12_C_3

TTCAAACTCCGGGTCTGTCTCTTCGTAATTAATTTTCTTT

>M.16.12_C_4

ACGAGTTTGTAGAGTTCAAACGTCTGGTTGAGTATGTT

>M.16.12_C_5

TAAAAATATATGCCAATATCTTATATAATTCTACAATT

>M.16.12_C_6

TTATTCATTTCCGCCTTATTAGTAACGCCTACGCT

>M.16.12_C_7

CTAATCTAGCTTATTTGAACGCACTGGAAATATTCC

>M.16.12_C_8

TTAAAATTAGATGATTTGTTAGGCATGTTTGGCTATCCT

>M.16.12_C_9

ACCTCATTATCATAAGTATTCTGAGCTAAGCTATTCAT

>M.16.12_C_10

TTATATCAATAATGATGGTCTTATCACTGCTGTTCTTCTTCC

>M.16.12_C_11

AATACATTAGCTATACTTGGATATCCAAGATTTATACCT

>M.16.12_C_12

GATTTATCAAAAAGCGGAATAACGAAGACGCCAAATTC

>M.16.12_C_13

ATTATTCTAACTCCTTTCAATTCATTTTGAGATAATG

>M.16.12_C_14

GAGGTTGGACGGCTAACGAATATCCCGCATTAGGCGGAT

>M.16.12_C_15

TCATTTATCTCAATTTTTAGATGCAAATACGACCCATATT

>M.16.12_C_16

TTCTCATATTGGACTAGGCTAATCTTCTGGGTTCTCTGCT

>M.16.12_C_17

TACCCTACAGCGAATTCCACACTCTTTACTTTATGCCT

>M.16.12_C_18

ACCTCGGTGCGACATTGAAAACATAATCTTTGTTACTC

>M.16.12_C_19

TTTAAGAAGTCATAGTTACTTTGCCTTTGCAAGTCTAG

>M.16.12_C_20

TGTGACCATTTTTTTATCCCTATATTATACTATGTTTAAGTA

>M.16.12_C_21

GGAATATATATGTGGGTATCGATATGAACCAGCCTG

>M.16.12_C_22

ACAACTCTTTATTTATACCGTTAAACGCGAATTTGG

>M.16.12_C_23

TCTAATTTGAATGTTATAACCCTGTTCATAAGTATT

>M.16.12_C_24

AAGACTTCTTCAGTCATTATTTTTCACCTTAGCAAATTC

>M.16.12_C_25

AAATATCCTGAATCGGCATATTCGAAATTACACTGTT

>M.16.12_C_26

TGCAGTTCTTCTAATTCTTTACATATTTTTTCCTCAG

>M.16.12_C_27

AAAGAGTCTCTTCTCATCATTGATCCCCCTAGAACTTT

>M.16.12_C_28

AAATTATATGGCCTGAAGTGGGTGTTAAGTACGACAAT

>M.16.12_C_29

TTAACTATGATACATTCCCGTACCAGACCGCAACACTT

>M.16.12_C_30

TTTAGGTGCTTTCTCAGATGGTTGAGGTATGGATTCCTG

>M.16.12_C_31

CATATCTCTCATTCACTTTACGCAAAGTGAAAATTAAAT

>M.16.12_C_32

GAACTATTGCACCTTTATAAGTATCCTCTTCTTCAGTTTTCTTA

>M.16.12_C_33

CTTTTGCGTAATAAATTTATACGTCCAATCGGGCATTCTT

>M.16.12_C_34

GAGACTTCTACATCTTGTCCGCTCATCTGTGTTCTTTT

>M.16.13_C_1

ACTGATTTTGACAATAATAGCGATTGTTATAGGCTCATT

>M.16.13_C_2

TTTTTCCACCTCAATATAAAACTCCCTACCCTGATTTGTT

>M.16.13_C_3

TTTCCTGCTTCTACCCACTGGTGTTTATGGTTTGA

>M.16.13_C_4

CTAACGTCCTCATCTAACATTCTGAAGAGTTCTTTCTCT

>M.16.13_C_5

ATTTGAACATTATCTACATCTATCTTAACTTCATTTATATAT

>M.16.13_C_6

AATAATTCAGCTTGTTTCTGATTGTTGATATACCTAGC

>M.16.13_C_7

ACGTACAACTTTATGTTCCCCTTTCCGTTTCTTTCAA

>M.16.13_C_8

TTATCCTTTCGTAAGAACGATTCTAAAGTTTGTTATCGTAA

>M.16.13_C_9

TAGTAATTCAAAACCATCCTTTTTTAATATTATCTCTATC

>M.16.13_C_10

ATCATTCCTGCTATAATTAATAGACTCAACATTAAGAGC

>M.16.13_C_11

ATACATTATCTAAGATGATAAACGCCAGCTACTACTAC

>M.16.13_C_12

GTTGTCTGGTCTCCAATATTAGGAGAATAAATTACAAT

>M.16.13_C_13

TATAATGTAATGTTAACAAACATTTCTATCACTATAG

>M.16.13_C_14

TTGGTATTTGCCATGATAAGGATTCTCAACTGGAGTTAAT

>M.16.13_C_15

AGTACAGAAGAACTTCATACGGTGCCGTCAGTGACACCAT

>M.16.13_C_16

CTAGTGGATCTGTTAGAACTAATAAAGCTGCCTTAATA

>M.16.13_C_17

AGATATTTTTGATGCAGTTTCCTCGCTTCCGGTAAGGAA

>M.16.13_C_18

GTTACGGTTAGTTTTTCCTTTTTCGCCAAAAAATCAA

>M.16.13_C_19

TTTCCATATTCTACGTTATCCGGGTACATATAGAATATT

>M.16.13_C_20

TTTAATACTAATGTTGCTTGTATATGTTTACTTAGT

>M.16.13_C_21

TTTCCATATTCTACGTTATCTGGATAGAGATAATATAT

>M.16.13_C_22

AAGGATATATTGACATAGATGACGAAGATCTTTACTT

>M.16.13_C_23

AGATGGCGGATAAATGATGCCATTCAGTCATTTGTC

>M.16.13_C_24

AAACAGATATTTCTGTACATTACCCTTGTCATTCTCATATG

>M.16.13_C_25

AACTTTTCAAATAGTTTGCAAGCATATAATCTATACTTAT

>M.16.13_C_26

ATATCCAAATCTTTTACTTTCTATTACTTTATCTATCAA

>M.16.13_C_27

AACATCTTTCTCTTCTCTTGTGCGCTTCTCATGTTCCAG

>M.16.13_C_28

TTTACTTCTCTTTTAAGGTACTTTTTATGGCCTTATCCC

>M.16.13_C_29

AGTAGTCCTTTTAGCGACCTCCCTCACACCTAAAACT

>M.16.13_C_30

ACTTCAGAGTGAGAAACATGGGAATACCAGGTTTGCCATA

>M.16.13_C_31

GGATAATCGCTTCCACCAGGTACATTATGACTCGTTAC

>M.16.13_C_32

ACTTAAAGATATAGATGAACTAGCACGGCAATATAA

>M.16.13_C_33

ACAGTTGGGGATACCACAATATAATCCATAGGTTGTAC

>M.16.13_C_34

AATTTATTAAGGTTGAAGATTATCAAGACAAGACAATAA

>M.16.13_C_35

TAGTATATCTATTTTCATATTTATACACCCGAAAAAAAG

>M.16.13_C_36

CAAACGGGCGAGGTTATCAGTCCTGCGCCAGTGCCT

>M.16.13_C_37

TTTACATAGAATAACTGTTTTACATCATTTTGTGACGC

>M.16.13_C_38

GTATGGTTTCTTCAGGATAGTTAAGTTTCAACATCTTCTCA

>M.16.13_C_39

ACAGAAACATACCCGCCCCCACTAACTGCCTTTAATAA

>M.16.13_C_40

GTATTTTTTCGATAACGTCTTTAAAAAGCATTTTCTTCGCCT

>M.16.13_C_41

GTCCTAAACATTCTTGTAAACATTCATTTCTATTTTTC

>M.16.13_C_42

TATTCATATTTTTGCTGTCGTAGTTGCTGTCATAGCAATT

>M.16.13_C_43

AGAAAGTGGGATACAGAAGCCTCAAGACGATCCTTACT

>M.16.13_C_44

AGAATACTATATAATTCTGCGGGTATTACTGCATTATTCC

>M.16.13_C_45

AGATATAATTTTGCATTTTTCTTGTTCCACTCTTCTATTA

>M.16.13_C_46

AACAAACTTCGGACGAGGTATGGAAGCAGGGTGTTGCAACC

>M.16.13_C_47

TCAAAAATACGAGAAAACTTATGAAGCTGATATACCTTACGTTAGAAG

>M.16.13_C_48

ATAGTATACTTTTGTCTCTACATTTATAATACGTCTCA

>M.16.13_C_49

CGTTTTCAGCGGGCTTTCTAGATTTGGTATTAAAACAG

>M.16.13_C_50

AAAAACAGTGTGGACCCAGCAATAGTAGCAAAGATAACG

>M.16.13_C_51

ACGATATATCTCTTCTATTACCTCATCGGGCATGAATACTT

>M.16.13_C_52

TTTTTATTCTTTTGAATGTAATATCATTTAAAGTCGGGATACC

>M.16.13_C_53

GCATATTATTACTACTTGCGGTAACAGGGCTAAACAA

>M.16.22_C_1

AAACCATCATTGTATGAATGACCAAATAGAATGTATAA

>M.16.22_C_2

CCAATAAACCCCTACTGCTTACGTGGCTTAATACGTTAT

>M.16.22_C_3

TTTTCTATGAGAGAACCATTTAAGAACCTAGATTACGT

>M.16.22_C_4

TTCAAACTCCGGGTCTGTCTCTTCGTAATTAATTTTCTTT

>M.16.22_C_5

ACGAGTTTGTAGAGTTCAAACGTCTGGTTGAGTATGTT

>M.16.22_C_6

TAAAAATATATGCCAATATCTTATATAATTCTACAATT

>M.16.22_C_7

TTCAAAGCTCTAAGCAGGGAGTTAGCCAACGCCTTGTT

>M.16.22_C_8

TAGTATTTATAGCTTCTTTTATCGTAAAAACACCTTTC

>M.16.22_C_9

AAGTTAGCCGAGGTAGTGATTAGCCATGGATAGATT

>M.16.22_C_10

CTAATCTAGCTTATTTGAACGCACTGGAAATATTCC

>M.16.22_C_11

TTAAAATTAGATGATTTGTTAGGCATGTTTGGCTATCCT

>M.16.22_C_12

ACCTCATTATCATAAGTATTCTGAGCTAAGCTATTCAT

>M.16.22_C_13

TTATATCAATAATGATGGTCTTATCACTGCTGTTCTTCTTCC

>M.16.22_C_14

ATAACAAATACAACTGTGCCTCCTACGCATACAGTTCAT

>M.16.22_C_15

AATACATTAGCTATACTTGGATATCCAAGATTTATACCT

>M.16.22_C_16

GATTTATCAAAAAGCGGAATAACGAAGACGCCAAATTC

>M.16.22_C_17

ATTATTCTAACTCCTTTCAATTCATTTTGAGATAATG

>M.16.22_C_18

GTTACATCGTTAGTATCTGAAAATTTCTTCTTTAACTTC

>M.16.22_C_19

GAGGTTGGACGGCTAACGAATATCCCGCATTAGGCGGAT

>M.16.22_C_20

TCATTTATCTCAATTTTTAGATGCAAATACGACCCATATT

>M.16.22_C_21

ATTTATTATATATAACAATACTATATCCTGCGTCCTTTAGT

>M.16.22_C_22

TTCTCATATTGGACTAGGCTAATCTTCTGGGTTCTCTGCT

>M.16.22_C_23

TACCCTACAGCGAATTCCACACTCTTTACTTTATGCCT

>M.16.22_C_24

AAATTATATGGCCTGAAGTGGGTGTTAAGTACGACAAT

>M.16.22_C_25

GAGTAGTAGCTATAAGCACTAAATTTTTTATTATATCTT

>M.16.22_C_26

ACCTCGGTGCGACATTGAAAACATAATCTTTGTTACTC

>M.16.22_C_27

AAGACTTCTTCAGTCATTATTTTTCACCTTAGCAAATTC

>M.16.22_C_28

ATTTAAATTTCCAGTACCATAAGCAACTGCTTGTCTATT

>M.16.22_C_29

TTTAAGAAGTCATAGTTACTTTGCCTTTGCAAGTCTAG

>M.16.22_C_30

TCATCCTCAAGAATCTTTAACTTCCCTTGCAGCAATCTT

>M.16.22_C_31

TGTGACCATTTTTTTATCCCTATATTATACTATGTTTAAGTA

>M.16.22_C_32

TTATTCATTTCCGCCTTATTAGTAACGCCTACGCT

>M.16.22_C_33

ACAACTCTTTATTTATACCGTTAAACGCGAATTTGG

>M.16.22_C_34

TCTAATTTGAATGTTATAACCCTGTTCATAAGTATT

>M.16.22_C_35

AAACTGTAGATGAGAATGTTGCTAAAGATGTTAATATCTC

>M.16.22_C_36

GAACTATTGCACCTTTATAAGTATCCTCTTCTTCAGTTTTCTTA

>M.16.22_C_37

AAATATCCTGAATCGGCATATTCGAAATTACACTGTT

>M.16.22_C_38

TGCAGTTCTTCTAATTCTTTACATATTTTTTCCTCAG

>M.16.22_C_39

AAAGAGTCTCTTCTCATCATTGATCCCCCTAGAACTTT

>M.16.22_C_40

GTATAGAACAACCACTGCCTAGCACTGTTTGGGTGTCCAT

>M.16.22_C_41

TTAACTATGATACATTCCCGTACCAGACCGCAACACTT

>M.16.22_C_42

TCTTTTTTCTCTAGTAATTCTATGATGTTTACGTCTCCA

>M.16.22_C_43

ATACCAGGATAACCGTAATATTGTGGGAACGATGT

>M.16.22_C_44

TTTTAAGTTACTTAACTCCTGGCTTATTTCACTCTCTTTC

>M.16.22_C_45

TTTAGGTGCTTTCTCAGATGGTTGAGGTATGGATTCCTG

>M.16.22_C_46

AACACTATTCTCTTCCTTACCTCTTTTACGTCCTC

>M.16.22_C_47

CATATCTCTCATTCACTTTACGCAAAGTGAAAATTAAAT

>M.16.22_C_48

AACACCAAAGTATTTAATTATATGGTTCTCTATCACGTTT

>M.16.22_C_49

GGAATATATATGTGGGTATCGATATGAACCAGCCTG

>M.16.22_C_50

TCTTCATCATAAAGATATAGCGGGTCATCACATTCT

>M.16.22_C_51

CTTTTGCGTAATAAATTTATACGTCCAATCGGGCATTCTT

>M.16.22_C_52

GAGACTTCTACATCTTGTCCGCTCATCTGTGTTCTTTT

>M.16.23_C_1

ATTAATTCTTTTACTGTATAGAAATATCTCATTTT

>M.16.23_C_2

ACCTTACTTTCTCTTCTCCCTTTAGATTAAGTTTT

>M.16.23_C_3

TCAAAATGAGTAATTTTTCTTCATGCAACATAATTCAC

>M.16.23_C_4

TTTTACAATCAGATATATTGATAGTTGATTTCTGCTA

>M.16.23_C_5

AAGTACACTTTATTAGCATTTTCTGGCAAACGATTA

>M.16.23_C_6

TATAAATAAGCGGTAAAAATGAAGAAAAAAACGTTTCTAT

>M.16.23_C_7

TTCCAAATACACGTTTATTCAATATGATATCCAGCAAC

>M.16.23_C_8

ACTTGTATATCAGATATCATGTCTACTAACGGCAAATT

>M.16.23_C_9

TCTATTAATATTGTATGCAACATTATTTTAACTTTGC

>M.16.23_C_10

GTATTAAGTCATCAGAATATCCTTCTAATGATAGAG

>M.16.23_C_11

ATTAATACAATTAAGCTCCGTCTTTGCTAGACTCTTTTA

>M.16.23_C_12

TTACTTTCTTCTCCACTATCTCCCATATGTCACGCTTC

>M.16.23_C_13

ATTAAATTCCTTTTAACATCACATAATCTTTATTTCATATC

>M.16.23_C_14

ACAATACCATGTGCAGAATCAATAAACAATATGCTGT

>M.16.23_C_15

TAGACAGGTCAGTATAGGTCTTACCATCTAATAAAGCAAG

>M.16.23_C_16

CTAAATATTTCGTGTTTATATCAAATGCATATACTTT

>M.16.23_C_17

TCCGTTTCTAACTTATTAAAGTATTCATATAATGC

>M.16.23_C_18

CTATATATTCGAGAAACAGGAATTTTCCCTGACTTATTT

>M.16.23_C_19

TTATTAATAATTTTTCGGGTGTTTTCATGTTTGTGCC

>M.16.23_C_20

TTATTATTTACAGTTATTCTTATCTTTGCCATCCTCACCAC

>M.16.23_C_21

ATTGAAAAGACTTTACGAAGTATATACAAGTGCTCA

>M.16.23_C_22

ACAAGATTCGTAACACTATTGACGAAGGTAAGAACGTTATTCTTG

>M.16.23_C_23

TCTTTCACATACGTTTCTATACGCTTCTTCTTTCCATC

>M.16.23_C_24

ATAGATATGATGAGATGGCAAACAATGTATACTAGGAAA

>M.16.23_C_25

TATAAGAACCTAAAAGAATTAACTAAGAGTGTTGACGG

>M.16.23_C_26

TTATATGTAGTGACGGTAAGTAGTAGCATCTATAACTCT

>M.16.23_C_27

TTCATTTCCGGCTCACCCTAGCCAAATACTCTTTGAAT

>M.16.23_C_28

TGATTCTCATCTATCTCCTCCTCCAATTCCGCGAGGAC

>M.16.23_C_29

TTCAGTAAGTAAGTTCGATTTTACTTATCGCCCATC

>M.16.23_C_30

CTTGTTAGTATATCTATTTTCATATTTATACACCCGAA

>M.16.23_C_31

GATATAATGATCTTGGATGAAAAAGCAAGAAAGGTGTTGG

>M.16.23_C_32

AAGAATCCCCAAGGATAAACACTAGCATATCGACC

>M.16.23_C_33

CTAACTAAAACACAACTAAATATAACACTATATAACACA

>M.16.23_C_34

GCAGTTAGGAAAACCACCAGATTTGGCTAGTTATATAGCCCAG

>M.16.23_C_35

AAAACTAATTGTATATTAGCAGGGTCACTTTGCACTCT

>M.16.23_C_36

AAATTACTATCTTGAATAGAGATATTAGCGTTGTCTATTGCT

>M.16.23_C_37

GCTTGTTTCTGATTGATGATATACCTTATTATTACTGGT

>M.16.23_C_38

AGATATTATCCTTATATTCACCTACTTTTTGCAGATATTTC

>M.16.23_C_39

CTTGTATGCGCACTTACGAAAAGCCAATAATACTCAAA

>M.16.23_C_40

CTAATGAAACATAAGAGCGAAAAGAAGTTTAGTAGAGATTC

>M.16.23_C_41

GTATGCTATGCAACAGTATTTTAATCTTATTTCTCGGATCCT

>M.16.23_C_42

AGTTATTTCGACGTGATTTAACGCACAGTAAGATTTATAA

>M.16.23_C_43

CTGACTGGTATCAAAGATAACGCTTTCTTAGGGTTCGTA

>M.16.23_C_44

GATGAATATGAAGAATTTGCTACTATAATAGTGAAACTCC

>M.16.23_C_45

GCTATTAACATTATTAAGTTATAGACATTTTGTCCATATTCTACG

>M.16.23_C_46

CTCATCACCCCATGTTTCTTCAAATCCACCCAGATTCTC

>M.16.23_C_47

ATACTCTGTTCCTTGCTCCATCTTTAGTAATTTGCTAA

>M.16.23_C_48

TATCATCATTCTCCTTGATAAACCAGTCTCACTTTCT

>M.16.23_C_49

TAGAATATCAAGCAGATAAATTAGTAGTACAAGATGTGA

>M.16.23_C_50

TCAAAATATATATCGATGATTTCTTCAAGATCACTT

>M.16.23_C_51

TATTAAAATACTTATTAGCAATCATTGCTGCACGTAC

>M.16.23_C_52

GATATCCATTCCTCAGCACTCTTAAATCCGCTGAAATT

>M.16.23_C_53

GATTACTCTTCACAGATAAACATTCTTTAGGTAATTCT

>M.16.23_C_54

CTATATATGGGAAGGCTTATGATCACCTAGTAGAA

>M.16.23_C_55

ATAATATTCTCTCTACTATTTCTTCTTTCAACTATTCA

>M.16.23_C_56

TAGAATCTAGCAGTACTATGACCCATATAACCATATACA

>M.16.23_C_57

TAATTTCATTTTCATTATTTCTCAACTCCCTTAATTAC

>M.16.23_C_58

ACAATAAGATAGAAGGGTCTCTCGCGATAACTCTGTAGT

>M.16.23_C_59

TTTCGTTATCGTTCACGGAATTTATCAATGCAGTTAATT

>M.16.27_C_1

ATTAATTCTTTTACTGTATAGAAATATCTCATTTT

>M.16.27_C_2

ACCTTACTTTCTCTTCTCCCTTTAGATTAAGTTTT

>M.16.27_C_3

TCAAAATGAGTAATTTTTCTTCATGCAACATAATTCAC

>M.16.27_C_4

TTTTACAATCAGATATATTGATAGTTGATTTCTGCTA

>M.16.27_C_5

AAGTACACTTTATTAGCATTTTCTGGCAAACGATTA

>M.16.27_C_6

TATAAATAAGCGGTAAAAATGAAGAAAAAAACGTTTCTAT

>M.16.27_C_7

TTCCAAATACACGTTTATTCAATATGATATCCAGCAAC

>M.16.27_C_8

ACTTGTATATCAGATATCATGTCTACTAACGGCAAATT

>M.16.27_C_9

TCTATTAATATTGTATGCAACATTATTTTAACTTTGC

>M.16.27_C_10

GTATTAAGTCATCAGAATATCCTTCTAATGATAGAG

>M.16.27_C_11

ATTAATACAATTAAGCTCCGTCTTTGCTAGACTCTTTTA

>M.16.27_C_12

TTACCAACCCGTTTTTATTGACAATATAACGGTTGTA

>M.16.27_C_13

TAAATTTCTATGTTTCATTTCAAGCATTTTTCTCTACCCT

>M.16.27_C_14

ATATTATTCAAAAAACTGATTACGTGAATGGATTTAT

>M.16.27_C_15

ACAAGATTCGTAACACTATTGACGAAGGTAAGAACGTTATTCTTG

>M.16.27_C_16

ACAATACCATGTGCAGAATCAATAAACAATATGCTGT

>M.16.27_C_17

TAATAATGATATATTGTAGATATTATCGTTGCCCGGGGC

>M.16.27_C_18

CTAAATATTTCGTGTTTATATCAAATGCATATACTTT

>M.16.27_C_19

TCCGTTTCTAACTTATTAAAGTATTCATATAATGC

>M.16.27_C_20

CTATATATTCGAGAAACAGGAATTTTCCCTGACTTATTT

>M.16.27_C_21

TTAAACTTTTACTAACTTACTAACTTACAAACTTAC

>M.16.27_C_22

AAGAATGATAGAATACACTACCAGTAAACGTCACTAAAA

>M.16.27_C_23

CTAATGAAACATAAGAGCGAAAAGAAGTTTAGTAGAGATTC

>M.16.27_C_24

TTATTATTTACAGTTATTCTTATCTTTGCCATCCTCACCAC

>M.16.27_C_25

ATTGAAAAGACTTTACGAAGTATATACAAGTGCTCA

>M.16.27_C_26

TTAATTTCTTTGACTGGTAATTTCTGTTCTATTTCTTT

>M.16.27_C_27

TCTTTCACATACGTTTCTATACGCTTCTTCTTTCCATC

>M.16.27_C_28

ATAGATATGATGAGATGGCAAACAATGTATACTAGGAAA

>M.16.27_C_29

ACTTTTGCGTTTTCATTTGTCTCTTCTTCTTCAAGTAG

>M.16.27_C_30

TATAAGAACCTAAAAGAATTAACTAAGAGTGTTGACGG

>M.16.27_C_31

TTATATGTAGTGACGGTAAGTAGTAGCATCTATAACTCT

>M.16.27_C_32

TATTTATTATAACACAAAGCACTATACACTTCAAAATT

>M.16.27_C_33

TTCATTTCCGGCTCACCCTAGCCAAATACTCTTTGAAT

>M.16.27_C_34

TGATCAATCACTTGTATTCTTTTGTGTAAATTTCGTC

>M.16.27_C_35

TTTGTTGGGTGTTCGTCGTTGTCCCGGCATTGAGGTTTA

>M.16.27_C_36

TGATTCTCATCTATCTCCTCCTCCAATTCCGCGAGGAC

>M.16.27_C_37

TTCAGTAAGTAAGTTCGATTTTACTTATCGCCCATC

>M.16.27_C_38

CTTGTTAGTATATCTATTTTCATATTTATACACCCGAA

>M.16.27_C_39

GATGAATATGAAGAATTTGCTACTATAATAGTGAAACTCC

>M.16.27_C_40

AAGAATCCCCAAGGATAAACACTAGCATATCGACC

>M.16.27_C_41

TAGACAGGTCAGTATAGGTCTTACCATCTAATAAAGCAAG

>M.16.27_C_42

CTAACTAAAACACAACTAAATATAACACTATATAACACA

>M.16.27_C_43

GCAGTTAGGAAAACCACCAGATTTGGCTAGTTATATAGCCCAG

>M.16.27_C_44

AAAACTAATTGTATATTAGCAGGGTCACTTTGCACTCT

>M.16.27_C_45

AAATTACTATCTTGAATAGAGATATTAGCGTTGTCTATTGCT

>M.16.27_C_46

GCTTGTTTCTGATTGATGATATACCTTATTATTACTGGT

>M.16.27_C_47

AGATATTATCCTTATATTCACCTACTTTTTGCAGATATTTC

>M.16.27_C_48

AACTATATCTAGGTGTTTCATGGACGGGTCACCTCATTTC

>M.16.27_C_49

CTTGTATGCGCACTTACGAAAAGCCAATAATACTCAAA

>M.16.27_C_50

TTTAAATTCCTTTTAACATCACATAATCTTTATTTCATATC

>M.16.27_C_51

GTATGCTATGCAACAGTATTTTAATCTTATTTCTCGGATCCT

>M.16.27_C_52

AGTTATTTCGACGTGATTTAACGCACAGTAAGATTTATAA

>M.16.27_C_53

CTGACTGGTATCAAAGATAACGCTTTCTTAGGGTTCGTA

>M.16.27_C_54

AATTTGATCTATCGCGGGTATTAATTTGTTCATAACTG

>M.16.27_C_55

GCTATTAACATTATTAAGTTATAGACATTTTGTCCATATTCTACG

>M.16.27_C_56

CTCATCACCCCATGTTTCTTCAAATCCACCCAGATTCTC

>M.16.27_C_57

ATACTCTGTTCCTTGCTCCATCTTTAGTAATTTGCTAA

>M.16.27_C_58

TATCATCATTCTCCTTGATAAACCAGTCTCACTTTCT

>M.16.27_C_59

TTATTAATAATTTTTCGGGTGTTTTCATGTTTGTGCC

>M.16.27_C_60

TAGAATATCAAGCAGATAAATTAGTAGTACAAGATGTGA

>M.16.27_C_61

TCAAAATATATATCGATGATTTCTTCAAGATCACTT

>M.16.27_C_62

TATTAAAATACTTATTAGCAATCATTGCTGCACGTAC

>M.16.27_C_63

GATATCCATTCCTCAGCACTCTTAAATCCGCTGAAATT

>M.16.27_C_64

GATTACTCTTCACAGATAAACATTCTTTAGGTAATTCT

>M.16.27_C_65

CTATATATGGGAAGGCTTATGATCACCTAGTAGAA

>M.16.27_C_66

ATAATATTCTCTCTACTATTTCTTCTTTCAACTATTCA

>M.16.27_C_67

TAGAATCTAGCAGTACTATGACCCATATAACCATATACA

>M.16.27_C_68

TAATTTCATTTTCATTATTTCTCAACTCCCTTAATTAC

>M.16.27_C_69

ACATAGAGGGCGTTCCTTGTCACTATTAACCATGT

>M.16.27_C_70

ACAATAAGATAGAAGGGTCTCTCGCGATAACTCTGTAGT

>M.16.27_C_71

TTTCGTTATCGTTCACGGAATTTATCAATGCAGTTAATT

>M.16.30_C_1

TTTCATCTATACTTACTTCCACAATATTCCCTTTTGGCACAT

>M.16.30_C_2

TTATTTCTTAGTATGATTTCTTTTAATTCATTTCCAT

>M.16.30_C_3

TAAACATATCTAAGACAATNACTCATGTAGCAGTCTATTAG

>M.16.30_C_4

TTATACTTTTTCTAATTGCTTTCTGAATCTTTCTTTTCT

>M.16.30_C_5

GCATAATAATTTAATAGATCTTCTAAGCCGTCTAAGATC

>M.16.30_C_6

TTTTATCAATTCACTCTTGCTTATCCCTAATCTTTTTG

>M.16.30_C_7

ATTTTAATTGAAGTAAACCCTTCTTCAACTTTATCTTC

>M.16.30_C_8

GTGTAATAAAATATCTATATAGTAAATTAAAACATCAT

>M.16.30_C_9

CCAAACCTGATTCCAGTGCAAGACCTCCTGCTTCTCCTAT

>M.16.30_C_10

ATAAACTCGTTCTAGGAAAAGAACCACCCTCAAACCTAA

>M.16.30_C_11

TCAAAAAATTATTTTCTCTATTTTGTGAAAAAGTTAA

>M.16.30_C_12

TCATATTATTACTACTTGCGGTAACAGGGCTAAACAA

>M.16.30_C_13

TTATATACATTAGTTGTAGTATTGACGATTAGTTGTACTGAG

>M.16.30_C_14

ATTCAATATTATGCATTTCCCCTTACGTCCCCTCAAGT

>M.16.30_C_15

GCAATGTTATCAAGATGATAGAACTTATTATTTATTTCAT

>M.16.30_C_16

AGTAATCGGGTTCGGGTTTGCTATCGGTCTTTACGTTC

>M.16.30_C_17

ATAACTAATCTCTCACATATTTCCCTGCCCAACATATCCCAC

>M.16.30_C_18

GCTTTTTAATATAAAATCAGGGGAAAGCGGAATGAATG

>M.16.30_C_19

AAACAAGAAGAAAAAGAAGTAGATGAGTTTTTTTTATTT

>M.16.46_C_1

TCAAAATGAGTAATTTTTCTTCATGCAACATAATTCAC

>M.16.46_C_2

TTCCAAATACACGTTTATTCAATATGATATCCAGCAAC

>M.16.46_C_3

AAGAATGATAGAATACACTACCAGTAAACGTCACTAAAA

>M.16.46_C_4

GATGAATATGAAGAATTTGCTACTATAATAGTGAAACTCC

>M.16.46_C_5

TAGAATATCAAGCAGATAAATTAGTAGTACAAGATGTGA

>M.16.46_C_6

TCTATTAATATTGTATGCAACATTATTTTAACTTTGC

>M.16.46_C_7

TTACCAACCCGTTTTTATTGACAATATAACGGTTGTA

>M.16.46_C_8

TAAATTTCTATGTTTCATTTCAAGCATTTTTCTCTACCCT

>M.16.46_C_9

TAGACAGGTCAGTATAGGTCTTACCATCTAATAAAGCAAG

>M.16.46_C_10

TCATCTTCTATTTGTTGGACCACCTGGGACTGGTAAAA

>M.16.46_C_11

TCTTTCACATACGTTTCTATACGCTTCTTCTTTCCATC

>M.16.46_C_12

ACTTTTGCGTTTTCATTTGTCTCTTCTTCTTCAAGTAG

>M.16.46_C_13

ACTAGATGTTTGAACTCTACAAACTCGTAGACCTTACAA

>M.16.46_C_14

TATTTATTATAACACAAAGCACTATACACTTCAAAATT

>M.16.46_C_15

TTAACATAAGAGCTTCCGTTTACTTCTATTACTCCTCC

>M.16.46_C_16

TGATCAATCACTTGTATTCTTTTGTGTAAATTTCGTC

>M.16.46_C_17

TTTGTTGGGTGTTCGTCGTTGTCCCGGCATTGAGGTTTA

>M.16.46_C_18

AAATTACTATCTTGAATAGAGATATTAGCGTTGTCTATTGCT

>M.16.46_C_19

TAATAATGATATATTGTAGATATTATCGTTGCCCGGGGC

>M.16.46_C_20

AGATATTATCCTTATATTCACCTACTTTTTGCAGATATTTC

>M.16.46_C_21

TTATTATTTACAGTTATTCTTATCTTTGCCATCCTCACCAC

>M.16.46_C_22

CTCATCACCCCATGTTTCTTCAAATCCACCCAGATTCTC

>M.16.46_C_23

CTATATATGGGAAGGCTTATGATCACCTAGTAGAA

>M.16.46_C_24

AAGAATCCCCAAGGATAAACACTAGCATATCGACC

>M.16.46_C_25

ACATAGAGGGCGTTCCTTGTCACTATTAACCATGT

>M.16.47_C_1

ATAGATAGAAATTACTCTCTACTGGATCCGCAACTTC

>M.16.47_C_2

TTCTACACTTTCAGAGAACATAGTTTCTTTATTTACA

>M.16.47_C_3

TTAGCTAGATCAGCTGCTTCAGCTTCTCTCAGCGCTCTC

>M.16.47_C_4

TCTTTTAGTGTTGAAAGATTTGCTTTATCATTTAAAAAT

>M.16.47_C_5

TTAAGTCCTTTGATGTGACAGATTATATCAAAATTGAT

>M.16.47_C_6

TCTTGTAGAAACTTTATCCACTTTACTTTTTCTTCGC

>M.16.47_C_7

GTTAAGCTCTATGTCCTGCAGGATAATACTTTTG

>M.16.47_C_8

TTCATGAAAACATCTCTGTCAAGTCTTTTTAATCCTAAA

>M.16.47_C_9

TTGTTACTCAAATATATTCACCTCCATCTTTTGT

>M.16.47_C_10

TCGTATATCTGTACAATCTTCAATTCGTTAAGTAGTTTC

>M.16.47_C_11

ATATTTTCAACTCTATAGCCGTTTTTCATGCATTCTTCTGAT

>M.16.47_C_12

CAATAATGTTTATGTCTTTTGTTTGCCTAAATTCTT

>M.16.47_C_13

CACTACTTGTTGTATATGCGTAACTATTAGAATTGATATT

>M.16.47_C_14

ATAACTCTACTACATTTTCTCATGTAAATTCTAGTGCAT

>M.16.47_C_15

GACTACAATTGGCAAAGATCATTCAGTTGGTAGTATAC

>M.16.47_C_16

ACTTTAATTCTAGACCAGTCCGATAGGATATCTCTT

>M.16.47_C_17

ATTTTTATCGCCCCGTCAATCATCAGATCTTTAACG

>M.16.47_C_18

AATCATATACTGTATCGTTCGTTACAATCACGTTATAT

>M.16.47_C_19

ATAGTCCAGTTTTAGCAGTACTTTATTCTTATGTATT

>M.16.47_C_20

ATAGTCACGTATATTGGATACCCAAGCTTCTCATAATTA

>M.16.47_C_21

ATAAGTTTAACGCCTAGGGTACTCCCTGCGTAAATTC

>M.16.47_C_22

TCTCATCTTTAGCAATAAATCATGAGTTACTGGAA

>M.16.47_C_23

ATAATTATGAATAGATCAAATAATAAGAGAAACGATT

>M.16.47_C_24

ATAGTCTATTTATCGTCTTCTTACCCTACAGCGAATTCCA

>M.16.47_C_25

GTTACAAAATTAGAATACTATTTAACAAACTTATCAAA

>M.16.47_C_26

ATTTAAATTATATTTTAGGGCGTATCTGTCTAGCAGTTGTA

>M.16.47_C_27

TTTTTCTTTCACCTCGATGTAAATATTTACTTCTTCAA

>M.16.47_C_28

ACGTTCAACTTTAGCGATTCATCTACTTTCTCTTCATT

>M.16.47_C_29

TTGCTAATGATTCAACGTTAGTCTCTACTATTTCTT

>M.16.47_C_30

TTTAATCTTATTTCTTATGTCTATGCCTTCTTCACGT

>M.16.47_C_31

TTAGAACAAATCGTAAATGAGTATTATGATATGAAGTGCC

>M.16.47_C_32

CCGCCCTACTTGTTTTTATATTAAATGCTATTCCC

>M.16.47_C_33

ATATATAGGTTCCATGTCATTGCTCCCCAACCATAATC

>M.16.47_C_34

TGATAATCTTCAACCTTAATAAATTCGAAATAAATATC

>M.16.47_C_35

AAGAATTTTGCTAACTGTTCTTTATTCGCAAGAGTTAC

>M.16.47_C_36

CTAAACCTATCAGAGTTAGCAGAATATAGTAATATTGATATT

>M.16.47_C_37

TTAGGAAGAAGGGGGACTACTGTAGTGTGTGTTACAATA

>M.16.47_C_38

AAATATTAAAGTTTTGCATTCTTTGTACGCGTCACCAAA

>M.16.47_C_39

TCGTGAGGGCAGAGATAGCAAAGAAGTCAAAGTATAATGC

>M.16.47_C_40

GTACCAGCAAACGGTTATGGTCTGATTTATGCTTATTTTT

>M.16.47_C_41

TTAGTATATCATATGCTTCCTTCATCATGTCAATATC

>M.16.47_C_42

TAGATTAAACGAAGAGGAAAAGAGAATGCTAGAAGA

>M.16.47_C_43

AGGATCTCAAGACATATGGTAACAAGTTTGTTTTCTT

>M.16.47_C_44

ATATTCTTGCAATTGCTTCTAAAGTTCCAATGCTTATTG

>M.16.47_C_45

TCTTTCACATACGTTTCTATACGCTTCTTCTTTCCATC

>M.16.47_C_46

ACCATATCTTTCCATGCATCAGTTTCTTGACTTCCATAC

>M.16.47_C_47

ACATAGATGATCTTATAACCCCTTAACGTCTCTTCCGGCAAC

>M.16.47_C_48

AACGTACCGCATGTTCATATCCCTGTAAAGTATTTAGA

>M.16.47_C_49

ATATTTTTAAGCTTCTTAGTAAAAGAAGGATATCTT

>M.16.47_C_50

TCAAAATGAGTAATTTTTCTTCATGCAACATAATTCAC

>M.16.47_C_51

TAGACAGGTCAGTATAGGTCTTACCATCTAATAAAGCAAG

>M.16.47_C_52

GTAAAATACCAGGTTCCCTACTATAATCGTTACTGCC

>M.16.47_C_53

AAATCAATATATAGTGCTTCAGATAGTGGCACATAT

>M.16.47_C_54

ATATATTTCTTTACTAGTTGCTGTGACGATTTAACGCTGCT

>M.16.47_C_55

CTTGAATAAGCACTTATAGAATTTTTATTAATAGCTTCAG

>M.16.47_C_56

TCTTCGGAGTAGTAAGGAACTCTTATTTACCTAAAAAAA

>M.16.47_C_57

TATTCATATCTGCCAATGAGTAATGTATACTTCTAT

>M.16.47_C_58

TCTTGTCTAGTTCATCTAACAATTTTAATAGCGTCTCGA

>M.16.47_C_59

ATTACACTTAAGATTGTTGCTTTTTTACTATTCTG

>M.16.47_C_60

ATTGTTAAATCAGTTATTCTCGGTCTCATTATAAAATTCA

>M.16.47_C_61

CTATCTAGTTCCTCTCTAATATTTTTATTAATCTTAAGATA

>M.16.47_C_62

GAAAGTTCTTTTTGCAGACGTTCGCCTAATCGAATAATAT

>M.16.47_C_63

TTATTATTTACAGTTATTCTTATCTTTGCCATCCTCACCAC

>M.16.47_C_64

ACAATTCCTACGCCTGGGACTTTTACTTTCTCCTTCTCCT

>M.16.47_C_65

ATCAGTATTGGGAAGTAGATTGCTTTGCCTTTATTCTCT

>M.16.47_C_66

ATTATCAAACGGATGGGTAAACCTATATAAAACATCGCCT

>M.16.47_C_67

TTCATGGGCGGGTCACCTCATCCTTTCCCTTTATTTCATT

>M.16.47_C_68

TATTACTCTTAGCAGCTTGCTCATAATTTATACTAA

>M.16.47_C_69

TCACTTATTTTCTTCATTATCTGAGTGTTGACAACGTCTTG

>M.16.47_C_70

ATTTCGTACATCAAGTATTTTGATGACAGTAAGAAATATTG

>M.16.47_C_71

ACTTTATACTGTAGATATTTACCGGGTAGGACAAAGTAT

>M.16.47_C_72

CCTTGTAACTTTACAGTAAGAAGCTGGATTAGGATTTC

>M.16.47_C_73

AACTCTAGTTTTATTGGTTTCTTTTCCCCCTTATTCTC

>M.16.47_C_74

TATTTTGCCCAATAGGTCTTTCGGAGTTATACCTTTCT

>M.16.47_C_75

TAAGCTTATCAGATATTCTACAGCTTTGTCAAATTCTTT

>M.16.47_C_76

CATATATAGTGAATTTCTTTTGAGATAATAGATTAT

>M.16.47_C_77

TTTAATTCTTTAATATAAGTAAGCTGTTCTACATAGAA

>M.16.47_C_78

CTAGTTATCCCTGCCCTACCGGTAAGTCTAACGAATATA

>M.16.47_C_79

TTTATACAATGAACATAATTCTTCATCGTTGTATTGTT

>M.16.47_C_80

TTCAAATACCCTAATTGTTCTCTCTACCTCATTTAGATC

>M.16.47_C_81

AATTCCTTTATCTTTGCTTCCAGCTCTTCTATCTTCTTT

>M.16.47_C_82

AGGTAACTTCTAAGATAGTAAGGTAATGCTAAACGAACCAT

>M.01.0.2_A_1

TCAGTCGTTTCAACGGCTGTAAACATTGACTATTCAAATG

>M.01.0.2_A_2

ATTACCAAAATCTACTGATTCTTTTACATCCTTAAAAAA

>M.01.0.2_A_3

CGACTCTGCGGGAGAGAATAAGGGGGCTCAGTCCTCCTCGG

>M.01.0.2_A_4

ATAGCAATATGATGTACTATATTTAAATGAAAAACTGT

>M.01.0.2_A_5

ATTTTGATTGTGACAATCCTCATCCTCGCACCTCTTTAA

>M.01.0.2_A_6

AAATGCGTATCAAGTTGCAGAGCAGAACAATATAAGTAGT

>M.01.0.2_A_7

CCACCACCACAGTATATAATACATTTGATGGATTATTAA

>M.01.0.2_A_8

TATTGGAGTACCCTAATTTAGATAGTACAATCTACGGATC

>M.01.0.2_A_9

CGTGATAATTATAAATGGGCTGAGGCTATGGGTAATAAAGTT

>M.01.0.2_A_10

TCAAGGGGCTATTATAGCAATATTAGGAGTAAAAGTAAGT

>M.01.0.2_A_11

TAAATTATATTGCCACCAATTACTATCATCGCAAGAGGTGT

>M.01.0.2_A_12

TACTTACACCCTTGTTTTAAGTATCTATTTTTGGGTTT

>M.01.0.2_A_13

GTTGCTCTAGCTTCGGTATTGTTTTTCACAATCACGATG

>M.01.0.2_A_14

AGAAACAGTGTATGGGATAAATTAGGTATAAGTGGCG

>M.01.0.2_A_15

CTTCCCCCATGGGCTTTTGCCCATGGTTTCATTGAGCAG

>M.01.0.2_A_16

ACCATCTCCTTAAACTTTCAATTCTATAGTAGATTAT

>M.01.0.2_A_17

TTTAGATTAAGTTTTACATAAATTATAGACACGATAACA

>M.01.0.2_A_18

ATTTTAGATCAAGTGTGGCAGCATATACAAAGTTGCCCAG

>M.01.0.2_A_19

AACCTAAGGTTAAGGCTTCTACGTGCTGTTTTAGGTCAT

>M.01.0.2_A_20

TCCTGGTCTTAAGAACGCTTCATCAATGTCAGTAGCTCTG

>M.01.0.2_A_21

GTTATTCTATTAAATGTGCATACTTCTTGATTTGCCGCCAT

>M.01.0.2_A_22

CATGTGTTTACTGGTACAAGTAAAAGAAGGAAAGTGGT

>M.01.0.2_A_23

GGCGTGTCGCCTCATCTTTATCAATTCGTAAAGCTCCATC

>M.01.0.2_A_24

ACACTAGCTGCAGCTTGCCACCATACAAACGCAGTTACA

>M.01.0.2_A_25

TATATCATGCGGTATAAATGCGGGGTCGGCTTCCTCGT

>M.01.0.2_A_26

TGGATCTGAGTTTTTAACGATTTTTCTTAGCAAACTGGGGAGT

>M.01.0.2_A_27

TAGAAAAATTTATCAATAGCCTAGTAACACTGAAGTTTCCTT

>M.01.0.2_A_28

CAAAAAACGGGATAGACCCGGCAATTATCGCAAAGATCAC

>M.01.0.2_A_29

GTGGTAAAGGCCGGGGGCAAGGGGGCTCAGTCCTCCTCGG

>M.01.0.2_A_30

AAATAAATATTCGTCTAAACGGGTTAAAAAGCTTTTTCCGCCTCCT

>M.01.0.2_A_31

ATAAAATTGAAGGGCATGGAACAAGACGTTATAGAGGAAT

>M.01.0.2_A_32

TTAAGGATTCTTATTATCCGCTTATCTGGTTAGATATA

>M.01.0.2_A_33

TCATAAGGTTTTACTGTTGCAGTAGGTGTTAAATTAGA

>M.01.0.2_A_34

ATATTTTTACTTCAAACATAAAGACCCCGACGTCACTACGGTAAA

>M.01.0.2_A_35

ATCTTCAAAATATGCCTTAATTCGTCTTCGGACGTCGCA

>M.01.0.2_A_36

TACTTTATGCTAATGTCCCTGAGTTTCCTAATATCGA

>M.01.0.2_A_37

GGGACTTTCGGTTCAGTGACTGCATACTTTTACGGCTTTT

>M.01.0.2_A_38

AAGTAGGTACGTATAAGTCCGCATTCACACGTTTAACC

>M.01.0.2_A_39

ATTTTATCGAGATATCATAATTACCATATGTTATAG

>M.01.0.2_A_40

CTGGTGTGATGTCAGTTATTAAATATGATAGCGGTAAGT

>M.01.0.2_A_41

AGTCTTCTACGGAGAGGCTGAGGACGAACGCCATCGACG

>M.01.0.2_A_42

CTCAAGGCAATTATAAATGAGGTGTCGACCAAGGTACCT

>M.01.0.2_A_43

CAGTCATAGCAAACATAGCAGATAATGAGGGTGAAATA

>M.01.0.2_A_44

CTGGACTTCAGCGACACGCTCGTGACATCGGTCATTTTA

>M.01.0.2_A_45

TCTAGTATAACTTTATACAAAAGCGGAACTGCAAATAT

>M.01.0.2_A_46

CTAATTCCAATATATCCTATTTGTGACCAAGGGAATGG

>M.01.0.2_A_47

TACTTTTTCTTCACCTTTGATATTTAATTTTACTATGAT

>M.01.0.2_A_48

TTCGATTTTGGGAATCCCACCGCCCAGCAATTCGCAGAC

>M.01.0.2_A_49

CTGTCATCAACCAATAAATAGACAAAAGATATCTTTTTTT

>M.01.0.2_A_50

TTTCTATTAAGAGAAAGTTACACTTTATCAAAAGACACT

>M.01.0.2_A_51

CTATCGGCTTATATCTAACATCCTCTGGGAGGCTTTCCAT

>M.01.0.2_A_52

TCAATGCTTTCAGTAGACGGTGCAATAATAATACATAAGAAG

>M.01.0.2_A_53

CAACTAATGCACCATATATTATTGCATGTAAATTCTATAA

>M.01.0.2_A_54

AATCCAAACTACATATAACTACATATAAGTAAAAAATTA

>M.01.0.2_A_55

CTAGTGCCAGGGTTCAGGGGTGTACCCTCACTGCCACA

>M.01.0.2_A_56

ATGAGTGCGAAATATACCTCGTTGCTATTTATGCTAGAAG

>M.01.0.2_A_57

AGAGTTTGAAAAGCGTTTACTATAGCTTGCCCCACGCTAC

>M.01.0.2_A_58

TGATTTTATTGTTTGTCCAGTGCGAAAACTTATTAGTAT

>M.01.0.2_A_59

AAACAAACTTCTCAACCATCAACAACACCACCTACATCG

>M.01.0.2_A_60

AAGAGTCTCTTCTCATCATTGACCCCCCTAGAACTTTAT

>M.01.0.2_A_61

AGAAATATTCCCAATAACTTGAGAAATCCGCGTAATGATT

>M.01.0.2_A_62

TATTTTGACCATATAATGTATAGTAATTTTGTTCAAT

>M.01.0.2_A_63

AAAGAAATAACGGCATCTATTGGGAGACTTCGTGCTA

>M.01.0.2_A_64

TATCGCTTAAGTAGGGTAAGAATTGATTAAGATAAGAA

>M.01.0.2_A_65

CAGCCCCAAGCTACTTTGCCAACCGTCGCTGAACAAGTGGC

>M.01.0.2_A_66

GTGTACATCCATCATTGCTTTTATTGAACAGATTATGTCATTA

>M.01.0.2_A_67

ATGTCGTTAAATGCATTTGATAGTAAATTTACCGCATTTT

>M.01.0.2_A_68

TATTTACACCGCCTTTCCCATTACCCTATCGCGGTTT

>M.01.0.2_A_69

CTTAACGTTCTGAAGTATATTCCACCACCACCAGGGTT

>M.01.0.2_A_70

ATACCGTTCACTACTATATCCTCTTCCTTCAGATTAAG

>M.01.0.2_A_71

CATAATAAATTTTCGGTTTGTGAGTTTTGTGTACTTAG

>M.01.0.2_A_72

ACTCCCACCTCGTCTCCACTACCACCCAACTGTATTCAAG

>M.01.0.2_A_73

GAATAAGCGGGGCGAAGCCGACGTGTACGCCGTGTCTTTC

>M.01.0.2_A_74

GTGGGGGTGGGGGAACCCCCCGCGGGGTTTACTGAACT

>M.01.0.2_A_75

TCTTATATAAAATCAAAATTGTATGAAATCCAAGCTGAAGTTTTT

>M.01.0.2_A_76

TAGAGACGGGAAAGAGGTTCAACACCCCATTACGGAATC

>M.01.0.2_A_77

TTTGAATTTTTCTTGCGAAAAAGTATATCTGAACAGCTA

>M.01.0.2_A_78

ATTTTTTCCCAAACCGATTTGAGAAACGAAAAAAACTCATC

>M.01.0.2_A_79

ATATTTTTACTTCAAACATGAAGACCCATTATTACAACTT

>M.01.0.2_A_80

TTCATATTTCTTAATGTATTTGGCCTTAACGTCCTCGTAC

>M.01.0.2_A_81

ATGTTACACTATATGCAATCTTTAAGAATAATTGCTATA

>M.01.0.2_A_82

ATAAAATGCGTCCTCTAGCACCGAATACGTTAGAGTGT

>M.01.0.2_A_83

TTATTTTGCATCACCTCCTTCTAAGCCCTAAGACTAG

>M.01.0.2_A_84

AAAATATTGTTTGGTGTTGTATTTATTTGTGCCAAATTAG

>M.01.0.2_A_85

ATTCCAAAGTTACGTCATCGATGACCACGTAATGGAAA

>M.01.0.2_A_86

ATAGAAGGGGCGAAAGGAAGAGCAATAAGTGCTATCATT

>M.01.0.2_A_87

GAAGTCTGCCGCTTAGTACTATGAGCTTAGAATCTATA

>M.01.0.2_A_88

TTTTTGGGGAACCTCTATCAATCAATCTATCAATCCGGG

>M.01.0.2_A_89

AACGGTATAAAAGGTGCTTTCAATTCTATAGTAGATTAT

>M.01.0.2_A_90

TATTTTAATCCACGTAATGAAGATATATTCAAATTTATTA

>M.01.0.2_A_91

GCACAAGCGGGTGCAAGTTAAATAACTTTTATTTTTTTAT

>M.01.0.2_A_92

CTATCAGTTATTTGTACCGGTGGTAGATTAGTACTTACTGT

>M.01.0.2_A_93

AGCAATTCTTGCACAGGACTCGTATATATATTTCCGC

>M.01.0.2_A_94

GTTAATGACTAATTCTATAGATTTGGAGGAATATTGTAG

>M.01.0.2_A_95

AATCCACTTCTATGACTTAATCTTTTTCTTTCTATATGTT

>M.01.0.2_A_96

CCATATCCAGGATTTCCCTGTGGAGGCCACCAGAATG

>M.01.0.2_A_97

ATGACTTTGTCCCTTTAGGTGAATACATTTTGACAGTGA

>M.01.0.2_A_98

TATCTAATGATAATTCTTTAATGCTAAAGAACTCACTAGG

>M.01.0.2_A_99

CTTGATTCTTTCTTCTCCCTTTAGATTAGCTAGTTTTA

>M.01.0.2_A_100

GCTTTTGATGTTTTTATTGTATATATAATGCCTAATGCTT

>M.01.0.2_A_101

TAATTCAACCCTAGCACCGATTTGCACAAGTTTTCTAACA

>M.01.0.2_A_102

AGATTTTGTAGTGCTTCTTGCAGCAGTAAAGCTCTAG

>M.01.0.2_A_103

ATTTAAGCAAAACGCGATTCTATAGTAGATTAT

>M.01.0.2_A_104

AGGACGTAATTCTACAATTGTTCTAGTAACTCCTTGGGGCGGA

>M.01.0.2_A_105

TATGTATCAGTAAAGTATAACGGATATGATGAAGGAAC

>M.01.0.2_A_106

TTTAAGGAGATGGTTTTATGAAAATTGCAGATAAGGTCAA

>M.01.0.2_A_107

CTCAGATTTCCATAATTGATCGAATGTTTGAAGGATGGG

>M.01.0.2_A_108

AACAACTGGATTTTTTGAATGATTGACACTTTTATGACTAT

>M.01.0.2_A_109

TAATTCAACCCTAGCACCGAATACGTTAGAGTGT

>M.01.0.2_A_110

TTCTAATAGCTGAACCCCTGCCTGATAGTCTCTATTAA

>M.01.0.2_A_111

TTGTAATAAATATTGGCTACCATTATGATACTCTACTA

>M.01.0.2_A_112

GTTTTTGGAGCAACAAATGTTTTTTCGCATAGGTGGTTA

>M.01.0.2_A_113

AACTTATACTTGTACAAGTATATGATTGATAATATTGCAC

>M.01.0.2_A_114

TAATTTAACTAACTCATTCTGAGTTTAAAAATTTAACGG

>M.01.0.2_A_115

GATATTCAAAGCTAACATCAAATCTATTTAGATTCTCTT

>M.01.0.2_A_116

CCATCAAGGCAATCGAGGCGAGTATCGTTTGCCCTTTG

>M.01.0.2_A_117

ATAAACTAAATATTGACGTGAAACCGCAACTAATAGATAA

>M.01.0.2_A_118

ACAATTTACCATCTCCTAGGGTGAGACAAACTTTTCTA

>M.01.0.2_A_119

CCCATACCTTTAGCACGTAAACGTCCTATTGATGTTGT

>M.01.0.2_A_120

CGTGCTTTAAGGGAAGATCGAGCTGGATAAGCAAACGGGC

>M.01.0.2_A_121

TGGCAATCGTAATAATGCCCGTAACCGTAAGCTCTGAGAG

>M.01.0.2_A_122

ATTAATTCTTTCTTCTTTTGCTCATAATATTTTATTTCATCTT

>M.01.0.2_A_123

TAAGTCTTTATATATCTCCATAATAAGTCTGTATCACTTCT

>M.01.0.2_A_124

TTAAATGATCCAATAGGATTTGTAATTGTTGTAGAAC

>M.01.0.2_A_125

TACTTCATCACGTCCACGTTTGTTATTTTTGTTGATTTAA

>M.01.0.2_A_126

AATAGAATTTGTCCATTTACTGCGGAAATATTTGCTACTA

>M.01.0.2_A_127

TAGTATAATCCGCGTATTCACCGTTTATCACGTTTTCAAT

>M.01.0.2_A_128

TGATTTCCACTTCTTGAAGTGTGTAGTTTGTTTGATATCA

>M.01.0.2_A_129

GATTGCATTAAATTCTCTAAACATGCAGCGAAACCAGA

>M.01.0.2_A_130

ATTGTTTTCTTATTTTCTTATCCATCATACTTTCTTCCCC

>M.01.0.2_A_131

TAATTCTTCTAGCTTTCTTAAGCGTTTTTACTCTATCTTTT

>M.01.0.2_A_132

AATCTTTTTTCTCATATGCAAAGGGTTATTTGTAAATCAAG

>M.01.0.2_A_133

ATCATCAGGCCCATGAGCGTTTTGCCGGTGCCGGTCGG

>M.01.0.2_A_134

TCTCCTACTCTCCCTACCATACCTACAATTCCTACGACT

>M.01.0.2_A_135

TTCTTGTAATGTCGTTATGTTACCATCATCTTTAATCTC

>M.01.0.2_A_136

ATCGACATGTTGGGGACCTTTAGTATGGGCATATTTGAG

>M.01.0.2_A_137

AATATAATATGAATTTCGTAGAAGAGTTCTTTCTATTAATT

>M.01.0.2_A_138

TGATGTGGGTAATGTCCACCATTTTGCAGTAAGTTGCTT

>M.01.0.2_A_139

CGACTCTGCGGGAGAGAATAAGGGGGAAAAGAAACCAAT

>M.01.0.2_A_140

AGATAATTGCGGAATCATTATATTGAGGGAACCACAAG

>M.01.0.2_A_141

TTGTTATCTTTTTCCATACTATACTTAAACTTAGTCTTTACAAT

>M.01.0.2_A_142

ATTTAAGCAAAACGCGATTCTTATGTATAGTCATCGA

>M.01.0.2_A_143

TGTTTTGTATACAGACTCACGAAGTTGTAGCCGTACC

>M.01.0.2_A_144

AACTCCAGTACTGCTTCGCCGTAGGATCCGTCCTCAGACG

>M.01.0.2_A_145

GTTTATGCACCGTCACTATCACCTATTGACATCATGAAA

>M.01.0.2_A_146

GAAAATGTATTAAAAAAATATGTAATTAAGGGGGTTGA

>M.01.0.2_A_147

CACCTTTTATACCTAACTTGATAACTATGTAGGACAC

>M.01.0.2_A_148

AACGGTATAAAAGTGTATTTTTCTCTTAGTTTCGGGGGTA

>M.01.0.2_A_149

GTATAGAGAAAATAACTTAACACTCATATGTAAAAAAA

>M.01.0.2_A_150

AATAAATATATTATAGATCCTATCAGCATCGCCTTAGGAGT

>M.01.0.2_A_151

AATTTCTCATGGCTAGGAGGCACAAGTCACATCATAAA

>M.01.0.2_A_152

TAGCGTGAAGTTACTACTTGTTGAAGGTGCTTGGCTA

>M.01.0.2_A_153

TGATAGGAATGATAGATAATGAAGAAACAATGATGTTAGT

>M.01.0.2_A_154

ATACCGTTCTTTCAATTCTATAGTAGATTAT

>M.01.0.2_A_155

GACGTAGATGAAACCTCTTTTGTTAACTCTTTTGCTCAC

>M.01.0.2_A_156

ATGTGGTACCATAAAGACCCCGACGTCACTACGGTAAA

>M.01.0.2_A_157

TATTTATACAGTAAAGTTTTGGCAAATTCCAATTGTAA

>M.01.0.2_A_158

GTATGCTTATCGTATAAAGTAATTTGCCAAAGTTGTACG

>M.01.0.2_A_159

TATATGCCATCACTCACACCAGCATTAGCAGTAGCGGT

>M.01.0.2_A_160

GTAATAGATAATTCCTCGTTTCTATCGTTGAAAGGATCG

>M.01.0.2_A_161

TATATTATACGAAATACCATCATCTTTCGTGAATGTACCT

>M.01.0.2_A_162

ACGTTAGCGTTAGAAGTATATTCTTCGCAACCTTCTTTCT

>M.01.1.3_A_1

TCTATTATAATCGATGAAACAGATAGAGCTGAATTGTGTAC

>M.01.1.3_A_2

TTCCCTTGTTGAATTAGTAACTGTTGTATCTGATTTG

>M.01.1.3_A_3

CCGAGAAAGGCTAAGCCTGATAGTGAATGTATGGAATGCCCC

>M.01.1.3_A_4

GGACTTCCACCTGTAGCTAGAGTTCCATTGAATGCTTGCCAA

>M.01.1.3_A_5

TAACTAGTTGCGGTATGTCACCGATTATTTGAGGTAGA

>M.01.1.3_A_6

AGACTGCAGAGAATATGTATTCAAATGCCTGAAGATGAT

>M.01.1.3_A_7

TATAAGATATCGATGACAATACATAAGAATAAAGTACTGC

>M.01.1.3_A_8

GTTGAGGAAACATATGCAGACCATAACTCGATTGATGCCGT

>M.01.1.3_A_9

ATTTTAGATCAAGTGTGGCAGCATATACAAAGTTGCCCAG

>M.01.1.3_A_10

GTGCGGAAACTTATCAAGACCGTGTCACTGACTGCACCG

>M.01.1.3_A_11

TCCTCACCCCGTAAGGGGGTCATCGCCATCCGCTTT

>M.01.1.3_A_12

ACTTCTTCAAATCGTTCAATTTCTCAGATAGCTCTTTCCATCT

>M.01.1.3_A_13

ATAACCTTCCTGCCAGGGTAGTAAATATCATCCGGG

>M.01.1.3_A_14

TTTTCTATCAGTCCACCGCAAGGAGTTACTAGAACAAT

>M.01.1.3_A_15

GGTTGATATGCATATGTTTGTTGAAGTGTACGCGAAAATA

>M.01.1.3_A_16

TCTAGAACAGATATGGGGTGTCGGTTTTACTGTAGAACA

>M.01.1.3_A_17

AAAATAACGGTGACAATAAAAGTAGATGAAAATGATCTAGT

>M.01.1.3_A_18

GAGGTGGCATAATGTTAGTCAATCTCGGTGCATTATTACA

>M.01.1.3_A_19

TCTTCGGTTCTTACTAACACCCTACCAGCTTTTACACCTT

>M.01.1.3_A_20

AGACCTACGGTTACATAGGTTTCTGACGGAGGGGTATTCT

>M.01.1.3_A_21

CTCAAGGCAATTATAAATGAGGTGTCGACCAGGGTACCT

>M.01.1.3_A_22

GGTATATCCTTACATGATCCCTCTCCCTCATAAAGTAATTTCAT

>M.01.1.3_A_23

CGGGGGGTTCCCCCACCCCCACGGGTGAGAATAAGGGGG

>M.01.1.3_A_24

GTCCTTAGTTTTTCTTTTCCTCTTCTTCTAAATTTCATTC

>M.01.1.3_A_25

ACATATGCACCAACATCAACGACAACAGTCGGTGTTATCT

>M.01.1.3_A_26

AATATAACGCTGACAATAAGCTTTTGCGTTCTTTCGAACCC

>M.01.1.3_A_27

TTTTATGTTGCACAGAACGGGGGGAACTACGCTCCTCCTC

>M.01.1.3_A_28

ATAGCTGCTGCGATTGCCAACTGCTCGGGATTCTGTGTA

>M.01.1.3_A_29

TGAAATGCAACTTGCTAACAAAATAAGTACCGCTAAGG

>M.01.1.3_A_30

TTTGAAAGTATGTATCAGTTCGAGATCATAGAGAAAGCTGT

>M.01.1.3_A_31

TTACCACTAACTGCAATAGGTGTAAACTTACGTGTTGCA

>M.01.1.3_A_32

TTACGATACATTGAGCTTCTTCCAGTAGGGACTATTACGG

>M.01.1.3_A_33

GTATGGAATTTGATGAACGAAATGAAGTACAAAATAGACGT

>M.01.1.3_A_34

AATTGCAAACTTATTGGTACTTAAAGCCCTTCATAAACAT

>M.01.1.3_A_35

ACCAATTCGCAAAATCTTTATGAATTGCTTTAACTATAT

>M.01.1.3_A_36

TAGCTTTACTTATAGGTGGAGGAGGGCCTAATAATAGCGG

>M.01.1.3_A_37

TCTGGGACTACTACTATTTCAATCCCAATATTATCAA

>M.01.1.3_A_38

GAATATTGCCCGATGCCGATGTTACCGTACTCTTTAAGGA

>M.01.1.3_A_39

CAAAACGGGAATGGGAGGAAGGTCAGACACCATCTTA

>M.01.1.3_A_40

CAAATCTACAAGATACGTGGGTTACTATCTCACCGCG

>M.01.1.3_A_41

GATCCAGGCGTAATAACTCTATATATTTTTGGATTTTGT

>M.01.1.3_A_42

TCTCCAAAGTCTAACGGGTGTTTTTATGATCGCCCGCG

>M.01.1.3_A_43

TCTTATATAAAATCAAAATTGTATGAAATCCAAGCTGAAGTTTTT

>M.01.1.3_A_44

GTGATTGGGTATGGCAATGGTTCTGTGATGGTGACATGT

>M.01.1.3_A_45

CTTTTCATACAACATATAAATCACCTCGAAAAATAAAAA

>M.01.1.3_A_46

TTCGAAACTAACCTCATAATTACTGCATAGGCGTATCTCTG

>M.01.1.3_A_47

AAATTGATGTTTAATGTTGGTAGTGTAAAGTTTGAAGGAA

>M.01.1.3_A_48

ATTTTTCCTAGATAGCGTTGAAAATCTTCTGATTGTTTC

>M.01.1.3_A_49

GTGACTGAGGCAACGGATAGTGTTACATTGCCTCCAAT

>M.01.1.3_A_50

AGATACCTGCGTATTGATAATATCTATCCAATACCATCGC

>M.01.1.3_A_51

ATTCCAAAGTTACGTCATCGATGACCACGTAATGGAAA

>M.01.1.3_A_52

GAAGTCTGCCGCTTAGTACTATGAGCTTAGAATCTATA

>M.01.1.3_A_53

AACGGGATTCTGAAGAACTATCAAAACGTGAACAATATTT

>M.01.1.3_A_54

TAGTTATCGTACCGAAACCAGGGGTGATGTAATGCCCATA

>M.01.1.3_A_55

TTAACATACAATCCGGGGCTACGGTCACTGTAGCCACTAA

>M.01.1.3_A_56

ATTTATAACAAACTCCAAGAATGGGAGAGAGTAAACATA

>M.01.1.3_A_57

TTACTATTCACTAAGTCAATAGGGATAGTAGAGAGAC

>M.01.1.3_A_58

TCAAATGTACCCCGCAATATCCTCTTTCCTACATTCACTT

>M.01.1.3_A_59

GAAAATGTATTAAAAAAATATGTAATTAATGGGGTTGAG

>M.01.1.3_A_60

ACTCTGTAGTTTGTAGTGCAGATAAAATACTACTCAGAGAT

>M.01.1.3_A_61

GAATAAGCGGGGCGAAGCCGACGTGTACGCCGTGTCTTTC

>M.01.1.3_A_62

TTTATCACTATTGCTATCTTGTTATACAGTGCATTCTTTGC

>M.01.1.3_A_63

TTGGTAACCTCATCACTCACCGTTGTTAAAAATACGT

>M.01.1.3_A_64

TAATAGATTTCGTAATAGTAGTGGCGGGAGTAATAGT

>M.01.1.3_A_65

TTTTCTTTATCCTCTTTATTATTTCTTCTAGTTGTTTAT

>M.01.1.3_A_66

GTTTTGTTCGCTTCGTTTAACTTAAGAGAAGATTCAACT

>M.01.1.3_A_67

CTATTACTTCTTTCTACTTCGTACATTAAGTATCGAGAGGGCAAT

>M.01.1.3_A_68

TATTCATTGCCGTTCCACGCTGTAACTACTCAAAAGG

>M.01.1.3_A_69

TTCGTAATGAGACCTCCGATTCTCAACCCCGATTTTTCTGTCC

>M.01.1.3_A_70

TATCATAAAATACAAATTAGTTACTATCATTACATATAAG

>M.01.1.3_A_71

GCATTCAACCCCGCGGGGGAATCCCCCGCCCCCATGGGGG

>M.01.1.3_A_72

AATCCTAACGTCGCTGATGTAATAAAAAGAGCGAAAGAG

>M.01.1.3_A_73

ATGCATTATAGATGATGCTACCGAATTGTTTAAGGTCAT

>M.01.1.3_A_74

CTTTCGTCACTGAATATCTCAAATCCATCCAGACCTCATT

>M.01.1.3_A_75

ATTTCAGTGTTTACCCACCTCTTCGCATAGAAACGAAT

>M.01.1.3_A_76

CAACCCCGTAACTGCCTCCTAGCTTATCATCTGCACTTTT

>M.01.1.3_A_77

GTATCATATCAGTACCCAATATTTGCACTATATATGCT

>M.01.1.3_A_78

CGTCATAGTAATGATATTGTATTGTCTTAACTTCACTTTCAC

>M.01.1.3_A_79

CTATGGTCGAGTGGCGTAAAAAGTTCCGTTTCCTCGTCAT

>M.01.1.3_A_80

ATCCTTAAAGTTAGTCTGTATCAATAGATTGTTAATATCTT

>M.01.1.3_A_81

ATAAGTATAAAGATGAATAAACCTAGAATGGCACCTATG

>M.01.1.3_A_82

TTTTAAACAAAATAAACTGTCCATCCTTCCTCACATTTCTT

>M.01.1.3_A_83

TGCTTGTTCCGCCATTGACAACGCTTGAGAATTTCCAT

>M.01.1.3_A_84

CCTCACTATATATGCTTTACAAAACTACCGACACGTATTATT

>M.01.1.3_A_85

TTTTGCTACCCGCACATCTATAGTACTTTAAGATCGG

>M.01.1.3_A_86

CAATATAAGATAATAATAAAACTAAAGGAAAGTAAGTGCG

>M.01.1.3_A_87

TTCTATAATAAATTTTCTGTTGATGTAGGTTGTTGATTTAG

>M.01.1.3_A_88

AAAGATCAATTAAAGGAGTTCTTTGAGTGGATAGAGAAAG

>M.01.1.3_A_89

TACTTCATCACGTCCACGTTTGTTATTTTTGTTGATTTAA

>M.01.1.3_A_90

ACGTTAAGACCATTTACAATTGCGAATAATGTAATTGT

>M.01.1.3_A_91

TAAGAAACGCTATCGATACTTCAGAGGCCGGCGCTAGG

>M.01.1.3_A_92

TATCTTCCGCCATTCATACAAAAAATCCTAAAAGCAGAG

>M.01.1.3_A_93

TGTACCGGGGACATTTACTTGTAGTGTATTTAATGCTA

>M.01.1.3_A_94

AACCACCATCCTGTGATTAATGCAACAACTACAAATACTAT

>M.01.1.3_A_95

GTATTGAACGCAGATATTAAAGCAGCATCTTCATATTCCG

>M.01.1.3_A_96

CTAATCAGTTCAACCCCGCGGGGGAATCCCCCGCCCCCA

>M.01.1.3_A_97

TACATGGGCTACAACCCATACGCTTACGGATTAGGACAAT

>M.01.1.3_A_98

TTGAAAATTCTGAAAAAAGATCTTAAAGGTCCCGAGAGG

>M.01.1.3_A_99

TTCTGCATACTTCTTATACTCTTCTTTAGCGATTGCAAA

>M.01.1.3_A_100

TGCAGTAAAACCGCAAAATGGACAAATTCCACCTGGTTT

>M.01.1.3_A_101

TAGAGATTCCGGCAAAACAACATTTCTGCAATTGGTTAAG

>M.01.1.3_A_102

ATTAAATTCAAGAAGTGCGTCTATAATAATTGTGTGCCGT

>M.01.1.3_A_103

TTAAAAATTAGATTAAATTATGAGTCAGGTAATCGGTTTT

>M.01.1.3_A_104

AATCCACATGCATTTTATAAACCATAGCAACTGCATT

>M.01.1.3_A_105

AGTATGTCTCTATCAATTACATCTGGTATATCCTCAACTTCAG

>M.01.1.3_A_106

ATGTTAATACACGAGGAGAGAGGTTCCGTGGCACTTGTGCA

>M.01.1.3_A_107

TAATCAGTTCAACCCCACAGAGTCCATCCCCGACTCTG

>M.01.1.3_A_108

CTTCTAATTCTTCTTTTAGCCTAGATGCAATCAGTATCACAT

>M.01.1.3_A_109

AAGCTTAGAACTTGGGTCATTTTCGGCCCAATGGAAAG

>M.01.1.3_A_110

TGTGCTGTTAATTCATATAGTACTACATAGCTACCATAGA

>M.01.1.3_A_111

TTCTCATCTAATATTATAGTACCTATATACATAGGTATTA

>M.01.1.3_A_112

ACGAGAGTGTTAGGATCTTCCCATCCTACCCCTAACGGG

>M.01.1.3_A_113

AGAGTACTCCCTGCGTAAATTCCGCCAACAATAGATATT

>M.01.1.3_A_114

ATTGTTAGTTATGTCACTCTACTAACCACACCATCTTTCA

>M.01.1.3_A_115

GATACAACACCGGGATTTAGATATGGACGGCAAAGTG

>M.01.1.3_A_116

TCTCTTAGTATTGCTCTCCTTACTTTTTTGTTACTACA

>M.01.1.3_A_117

AAATGGACTTTGGTTTTTCCTATTACTAAAACGTTAGCA

>M.01.2.1_A_1

TCTATTATAATCGATGAAACAGATAGAGCTGAATTGTGTAC

>M.01.2.1_A_2

AAGATTATGCCGATCAAGAATCTGACGGGAAATATTATGTT

>M.01.2.1_A_3

CCTACACCGACAATAATTATGTTTACCTTTTCCCATT

>M.01.2.1_A_4

TTCCCTTGTTGAATTAGTAACTGTTGTATCTGATTTG

>M.01.2.1_A_5

CAAATTCATATATAGCACCCGGATTTAAAGAATTTCCTC

>M.01.2.1_A_6

TACCCTGTACGTCTGTACTCGAATTCTAGCTTTACTACT

>M.01.2.1_A_7

TTACGGGGGTTCACCAGGTCAACATCCCCACTGAAGTTGA

>M.01.2.1_A_8

TAGTAACATCCTTGGTTCTAAGAACCAACCTTTCCCCA

>M.01.2.1_A_9

CCGAGAAAGGCTAAGCCTGATAGTGAATGTATGGAATGCCCC

>M.01.2.1_A_10

GGACTTCCACCTGTAGCTAGAGTTCCATTGAATGCTTGCCAA

>M.01.2.1_A_11

TAACTAGTTGCGGTATGTCACCGATTATTTGAGGTAGA

>M.01.2.1_A_12

CGTCCAACTTAACCTTGAACGTCGACGGGTCAATCGG

>M.01.2.1_A_13

TTTGTTCATACTATCACCCAGCTACAAATTCAATCA

>M.01.2.1_A_14

AGAAACAGTGTATGGGATAAATTAGGTATAAGTGGCG

>M.01.2.1_A_15

AGACTGCAGAGAATATGTATTCAAATGCCTGAAGATGAT

>M.01.2.1_A_16

TATAAGATATCGATGACAATACATAAGAATAAAGTACTGC

>M.01.2.1_A_17

GTTGAGGAAACATATGCAGACCATAACTCGATTGATGCCGT

>M.01.2.1_A_18

ATTTTAGATCAAGTGTGGCAGCATATACAAAGTTGCCCAG

>M.01.2.1_A_19

CGTTGTCGTGGAGCTGTTCGACGGTTACGACGAAGCAATCGT

>M.01.2.1_A_20

GTGCGGAAACTTATCAAGACCGTGTCACTGACTGCACCG

>M.01.2.1_A_21

TCCTCACCCCGTAAGGGGGTCATCGCCATCCGCTTT

>M.01.2.1_A_22

GTAGGTATAGGCGACATAATGGTGATGGGTAAAGAATTCT

>M.01.2.1_A_23

ACTTCTTCAAATCGTTCAATTTCTCAGATAGCTCTTTCCATCT

>M.01.2.1_A_24

TCTCTTAGTATTGCTCTTTTAACTTTTTTATTACTACATAT

>M.01.2.1_A_25

ATAACCTTCCTGCCAGGGTAGTAAATATCATCCGGG

>M.01.2.1_A_26

ACTCCCATCTACCCGATACACCACTTTCTGAGATAAACAA

>M.01.2.1_A_27

TGTACCGGGGACATTTACTTGTAGTGTATTTAATGCTA

>M.01.2.1_A_28

TTTTCTATCAGTCCACCGCAAGGAGTTACTAGAACAAT

>M.01.2.1_A_29

GGTTGATATGCATATGTTTGTTGAAGTGTACGCGAAAATA

>M.01.2.1_A_30

TCTAGAACAGATATGGGGTGTCGGTTTTACTGTAGAACA

>M.01.2.1_A_31

AATCCACATGCATTTTATAAACCATAGCAACTGCATT

>M.01.2.1_A_32

TGTTTACTTAATGCTAATGTATATCTGTCATTAGTTGATATA

>M.01.2.1_A_33

TACACATTAGTATTAGTGATAACAAGTACGAGTATAGTGCT

>M.01.2.1_A_34

GTCCTTAGTTTTTCTTTTCCTCTTCTTCTAAATTTCATTC

>M.01.2.1_A_35

ATTGACATCATGAAACAACGACAAATAGAACTATTACTAA

>M.01.2.1_A_36

AGACCTACGGTTACATAGGTTTCTGACGGAGGGGTATTCT

>M.01.2.1_A_37

TCATAAGGTTTTACTGTTGCAGTAGGTGTTAAATTAGA

>M.01.2.1_A_38

GGTATATCCTTACATGATCCCTCTCCCTCATAAAGTAATTTCAT

>M.01.2.1_A_39

CGGGGGGTTCCCCCACCCCCACGGGTGAGAATAAGGGGG

>M.01.2.1_A_40

TTTACAAGACCGACACTTAGATCCTTCGGTGGAAAGA

>M.01.2.1_A_41

GGCGGTAATGCGAAATCAACATAACCCACTACTGAACCTCC

>M.01.2.1_A_42

ATTCCAAAGTTACGTCATCGATGACCACGTAATGGAAA

>M.01.2.1_A_43

AATATAACGCTGACAATAAGCTTTTGCGTTCTTTCGAACCC

>M.01.2.1_A_44

TTTATTTAATGTAATTTTTTATTTCTTCTCTCACTATTC

>M.01.2.1_A_45

ATAGCTGCTGCGATTGCCAACTGCTCGGGATTCTGTGTA

>M.01.2.1_A_46

AAGTCAGCTACGAACGGTAACCCTGTAACGAGGAGG

>M.01.2.1_A_47

GATGAGAAGGAGATGTTAAGGCAAAAGCTTGAGGAGATG

>M.01.2.1_A_48

TAAGTCCTCGTCATAGTAATGATATTGTATTGTCTTAAC

>M.01.2.1_A_49

TGAAATGCAACTTGCTAACAAAATAAGTACCGCTAAGG

>M.01.2.1_A_50

CTGGAGTGAATAGAATACCAGTTATCGCTGGAAGCACAA

>M.01.2.1_A_51

TTTGAAAGTATGTATCAGTTCGAGATCATAGAGAAAGCTGT

>M.01.2.1_A_52

TTACCACTAACTGCAATAGGTGTAAACTTACGTGTTGCA

>M.01.2.1_A_53

AAATCGCCGTCAATATCATATTACTTACGCTCTGAAAGA

>M.01.2.1_A_54

TTACGATACATTGAGCTTCTTCCAGTAGGGACTATTACGG

>M.01.2.1_A_55

ACTGACAAAGAGAAGTTTGACAAAGAGATTTTGCTA

>M.01.2.1_A_56

GTATGGAATTTGATGAACGAAATGAAGTACAAAATAGACGT

>M.01.2.1_A_57

TTTCTATTAAGAGAAAGTTACACTTTATCAAAAGACACT

>M.01.2.1_A_58

CTATCGGCTTATATCTAACATCCTCTGGGAGGCTTTCCAT

>M.01.2.1_A_59

GAAAGTAAGGTTTTCTTAAAAGAGTGTGAGTTCTCAC

>M.01.2.1_A_60

AATTGCAAACTTATTGGTACTTAAAGCCCTTCATAAACAT

>M.01.2.1_A_61

CAGCCTATTTACCTCACCCCCGGTAGCATACCTTTTAAGAT

>M.01.2.1_A_62

GTTCTGGTGATAGCCTTTTCCAGTGTCTCAGCGACTTCGG

>M.01.2.1_A_63

TTCGCCTCTTTTTCAGCATTTATTGCTGTATTTCTACT

>M.01.2.1_A_64

TTTTATGTTGCACAGAACGGGGGGAACTACGCTCCTCCTC

>M.01.2.1_A_65

ATGTAGGTATGTTCCATTTCAGATTATCTCTATATAGA

>M.01.2.1_A_66

TAGCTTTACTTATAGGTGGAGGAGGGCCTAATAATAGCGG

>M.01.2.1_A_67

AACCTATACTTATACAAGTACATGATTGATAATATTATG

>M.01.2.1_A_68

TCATCGTAGCCGTTGTACTTCACGGCGGTATAGGGTATAT

>M.01.2.1_A_69

TTTTTAGCAAGCAGCGGTTTACATGATGTCTTTTCAATCA

>M.01.2.1_A_70

ATTTAGTATACTTCTATAACGGTGTAGGTGAAGAAAAGA

>M.01.2.1_A_71

AACCCCTTACGCCTCTTTGCACCTATCAATGTGATGATGT

>M.01.2.1_A_72

TCTGGGACTACTACTATTTCAATCCCAATATTATCAA

>M.01.2.1_A_73

GAATATTGCCCGATGCCGATGTTACCGTACTCTTTAAGGA

>M.01.2.1_A_74

CAAAACGGGAATGGGAGGAAGGTCAGACACCATCTTA

>M.01.2.1_A_75

ACATCAGCATCAATAAATATTACATCATCATTATCTACT

>M.01.2.1_A_76

ATGACTTTGTGTCTTTAGGTGCATACATTCTGACGGTAAG

>M.01.2.1_A_77

ATTCGCTACTCCCGAAGCTTGGGTTCCTGTTGCACCTGCACC

>M.01.2.1_A_78

ATGTATTAGCGGGTGGTACACCATATTGTGATATATCT

>M.01.2.1_A_79

TCATTAGTTTGACCTGAATTTAAATTTACAATAAATCTG

>M.01.2.1_A_80

CAAATCTACAAGATACGTGGGTTACTATCTCACCGCG

>M.01.2.1_A_81

TAACACAGAAAGCTGAGTTATTGCCTTTCACCTAATAA

>M.01.2.1_A_82

AAGCAAAAGCGGAAATCACGGTGTACGACAATATTTCCT

>M.01.2.1_A_83

GAATAAGCGGGGCGAAGCCGACGTGTACGCCGTGTCTTTC

>M.01.2.1_A_84

GATCCAGGCGTAATAACTCTATATATTTTTGGATTTTGT

>M.01.2.1_A_85

ATTTTACTCTTCCAACTTTAAACCTCAATCTCGGGAACTT

>M.01.2.1_A_86

TCTCCAAAGTCTAACGGGTGTTTTTATGATCGCCCGCG

>M.01.2.1_A_87

TATACGTAGCGTACCGCCGTTATCGTCAATTGGTAAG

>M.01.2.1_A_88

CTATTACTTCTTTCTACTTCGTACATTAAGTATCGAGAGGGCAAT

>M.01.2.1_A_89

TTTGAATTTTTCTTGCGAAAAAGTATATCTGAACAGCTA

>M.01.2.1_A_90

GTGATTGGGTATGGCAATGGTTCTGTGATGGTGACATGT

>M.01.2.1_A_91

GCATATGTTGCTGCTCTTCCGGAAATGTTTGCGATAGG

>M.01.2.1_A_92

CTTTTCATACAACATATAAATCACCTCGAAAAATAAAAA

>M.01.2.1_A_93

TTCGAAACTAACCTCATAATTACTGCATAGGCGTATCTCTG

>M.01.2.1_A_94

TCTTATATAAAATCAAAATTGTATGAAATCCAAGCTGAAGTTTTT

>M.01.2.1_A_95

AAATTGATGTTTAATGTTGGTAGTGTAAAGTTTGAAGGAA

>M.01.2.1_A_96

AACTCCAGTACTGCTTCGCCGTAGGATCCGTCCTCAGACG

>M.01.2.1_A_97

ACATCAAAATGGAATGCAGAACAATTAGATAGGTATGAT

>M.01.2.1_A_98

ATTTTTCCTAGATAGCGTTGAAAATCTTCTGATTGTTTC

>M.01.2.1_A_99

GTGACTGAGGCAACGGATAGTGTTACATTGCCTCCAAT

>M.01.2.1_A_100

TTATTTTCAATTGTAATATTATTAAGTTGATTCATATTTA

>M.01.2.1_A_101

AGATACCTGCGTATTGATAATATCTATCCAATACCATCGC

>M.01.2.1_A_102

ATTTTTGAAAATACTTACAATATTATAATGACTTATATAGA

>M.01.2.1_A_103

ACTGCTATTATTATCAGCCACACGATATACTCCATTCCCTT

>M.01.2.1_A_104

GACGAGAAGAGACTAATCGATCACGCAATTGAAGCCGG

>M.01.2.1_A_105

GAAGTCTGCCGCTTAGTACTATGAGCTTAGAATCTATA

>M.01.2.1_A_106

TTTACTATTATTTCTTTTCCATTGATCGTAATTTTCTCCGGT

>M.01.2.1_A_107

TAATTACAGCTTCTACCAAGTACTTGACTAACCTTTTT

>M.01.2.1_A_108

TTTGTCTCCTCTACTGCTTTCTTCCTTTCCTCATCTGA

>M.01.2.1_A_109

AACGGGATTCTGAAGAACTATCAAAACGTGAACAATATTT

>M.01.2.1_A_110

TAGTTATCGTACCGAAACCAGGGGTGATGTAATGCCCATA

>M.01.2.1_A_111

AAGCTTAGAACTTGGGTCATTTTCGGCCCAATGGAAAG

>M.01.2.1_A_112

TTAACATACAATCCGGGGCTACGGTCACTGTAGCCACTAA

>M.01.2.1_A_113

ATTTATAACAAACTCCAAGAATGGGAGAGAGTAAACATA

>M.01.2.1_A_114

TACACAGTACTACCTGGGATGACAAAAATCTTAAAGGTT

>M.01.2.1_A_115

TTACTATTCACTAAGTCAATAGGGATAGTAGAGAGAC

>M.01.2.1_A_116

TCAAATGTACCCCGCAATATCCTCTTTCCTACATTCACTT

>M.01.2.1_A_117

GAAAATGTATTAAAAAAATATGTAATTAATGGGGTTGAG

>M.01.2.1_A_118

ACTCTGTAGTTTGTAGTGCAGATAAAATACTACTCAGAGAT

>M.01.2.1_A_119

TAAATAAACTTACTCATAAATTTTGCTATAATTATGACA

>M.01.2.1_A_120

TTTATCACTATTGCTATCTTGTTATACAGTGCATTCTTTGC

>M.01.2.1_A_121

AAAGATCAATTAAAGGAGTTCTTTGAGTGGATAGAGAAAG

>M.01.2.1_A_122

TAATAGATTTCGTAATAGTAGTGGCGGGAGTAATAGT

>M.01.2.1_A_123

TTTTCTTTATCCTCTTTATTATTTCTTCTAGTTGTTTAT

>M.01.2.1_A_124

GTTTTGTTCGCTTCGTTTAACTTAAGAGAAGATTCAACT

>M.01.2.1_A_125

ACAAGCCATATACGTTCCAACCCAATCATCTAGTTACGTTACT

>M.01.2.1_A_126

CCAAATTTCGGGCTCTCGCCCGCTTTTACTTTCTAACCGC

>M.01.2.1_A_127

TTTGGTTTCGTACTTGGACTTGATAATAATAATGAATTTGC

>M.01.2.1_A_128

TATTCATTGCCGTTCCACGCTGTAACTACTCAAAAGG

>M.01.2.1_A_129

TTCGTAATGAGACCTCCGATTCTCAACCCCGATTTTTCTGTCC

>M.01.2.1_A_130

ACTCCATGTTACGTCTACGTTTTGGCTTAGGTTTAGG

>M.01.2.1_A_131

TATCATAAAATACAAATTAGTTACTATCATTACATATAAG

>M.01.2.1_A_132

CCACCACCACAGTATATAATACATTTGATGGATTATTAA

>M.01.2.1_A_133

AGAGCCGAGATGATAATTAAACAAGCAAAACAATATCTA

>M.01.2.1_A_134

AATTTAACTAACAACGCGGGGGTTTAAAAATTTAACGG

>M.01.2.1_A_135

GCATTCAACCCCGCGGGGGAATCCCCCGCCCCCATGGGGG

>M.01.2.1_A_136

AATCCTAACGTCGCTGATGTAATAAAAAGAGCGAAAGAG

>M.01.2.1_A_137

ATTTATATTATCTTGTGATCCCGCTACTCCTCTAGGTGG

>M.01.2.1_A_138

TTCTAATAGCTGAACCCCTGCCTGATAGTCTCTATTAA

>M.01.2.1_A_139

ATGCATTATAGATGATGCTACCGAATTGTTTAAGGTCAT

>M.01.2.1_A_140

TTCGATTTTGGGAATCCCACCGCCCAGCAATTCGCAGAC

>M.01.2.1_A_141

ATTATTGCCTTTTGGGCTTGAAGTTTTAATTTATCCGCTT

>M.01.2.1_A_142

CCTTTCTATAACAAATTTTCGCTAGTGCTATTTGTT

>M.01.2.1_A_143

CTTTCGTCACTGAATATCTCAAATCCATCCAGACCTCATT

>M.01.2.1_A_144

ATTTCAGTGTTTACCCACCTCTTCGCATAGAAACGAAT

>M.01.2.1_A_145

AGAACGGGGATATCAACACCATATCTTATACTTGCCTGA

>M.01.2.1_A_146

GGCGTGTCGCCTCATCTTTATCAATTCGTAAAGCTCCATC

>M.01.2.1_A_147

TTACTTTTTGCAATTCTTTAATGCTTTCTAATAGTTTCATC

>M.01.2.1_A_148

CAACCCCGTAACTGCCTCCTAGCTTATCATCTGCACTTTT

>M.01.2.1_A_149

AAAAATAGCCCTGCTGCACCGTAGAGTAGATGTTTGAA

>M.01.2.1_A_150

AAATCTACAATTGCATTTACAAAATTATCAATACCCTTTTTCA

>M.01.2.1_A_151

GTATCATATCAGTACCCAATATTTGCACTATATATGCT

>M.01.2.1_A_152

CGTCATAGTAATGATATTGTATTGTCTTAACTTCACTTTCAC

>M.01.2.1_A_153

CTATGGTCGAGTGGCGTAAAAAGTTCCGTTTCCTCGTCAT

>M.01.2.1_A_154

ATCCTTAAAGTTAGTCTGTATCAATAGATTGTTAATATCTT

>M.01.2.1_A_155

ATAAGTATAAAGATGAATAAACCTAGAATGGCACCTATG

>M.01.2.1_A_156

GCGGATAGTTAGGAAGACCAGGCATGTTCGCTGGATTCCT

>M.01.2.1_A_157

ATTGTTAGTTATGTCACTCTACTAACCACACCATCTTTCA

>M.01.2.1_A_158

TGAAAACGTGGAGCAAAAACTGCGACAACTAGGAATAGT

>M.01.2.1_A_159

TTTTAAACAAAATAAACTGTCCATCCTTCCTCACATTTCTT

>M.01.2.1_A_160

TGCTTGTTCCGCCATTGACAACGCTTGAGAATTTCCAT

>M.01.2.1_A_161

CCTCACTATATATGCTTTACAAAACTACCGACACGTATTATT

>M.01.2.1_A_162

TTTTGCTACCCGCACATCTATAGTACTTTAAGATCGG

>M.01.2.1_A_163

CAATATAAGATAATAATAAAACTAAAGGAAAGTAAGTGCG

>M.01.2.1_A_164

TTCTATAATAAATTTTCTGTTGATGTAGGTTGTTGATTTAG

>M.01.2.1_A_165

TTGGTAACCTCATCACTCACCGTTGTTAAAAATACGT

>M.01.2.1_A_166

ACTGATTGACCGTATGGGCCGAATACTGTGAAATAGCAT

>M.01.2.1_A_167

ACGTTAAGACCATTTACAATTGCGAATAATGTAATTGT

>M.01.2.1_A_168

ACATATGCACCAACATCAACGACAACAGTCGGTGTTATCT

>M.01.2.1_A_169

TACTTCATCACGTCCACGTTTGTTATTTTTGTTGATTTAA

>M.01.2.1_A_170

TCATCCTTCTCGTGGCTATATATAAATCCACCGAAACCG

>M.01.2.1_A_171

ACCAATTCGCAAAATCTTTATGAATTGCTTTAACTATAT

>M.01.2.1_A_172

TAAGAAACGCTATCGATACTTCAGAGGCCGGCGCTAGG

>M.01.2.1_A_173

TATCTTCCGCCATTCATACAAAAAATCCTAAAAGCAGAG

>M.01.2.1_A_174

CGCCATCGCCGACACGGGGGTATGTAGTAAGTATTGCT

>M.01.2.1_A_175

TATACGTAGTTTGCACCTATAAGTACTTCGGTTGATATA

>M.01.2.1_A_176

CAACAAAATCCAATTACTTCTCCTCAAATTCAATCAACAA

>M.01.2.1_A_177

GTATTGAACGCAGATATTAAAGCAGCATCTTCATATTCCG

>M.01.2.1_A_178

CTAATCAGTTCAACCCCGCGGGGGAATCCCCCGCCCCCA

>M.01.2.1_A_179

TCACCGATTGCGGTACGTCACACCTTCGACATTTCATACC

>M.01.2.1_A_180

TACATGGGCTACAACCCATACGCTTACGGATTAGGACAAT

>M.01.2.1_A_181

TTGAAAATTCTGAAAAAAGATCTTAAAGGTCCCGAGAGG

>M.01.2.1_A_182

TGACGGAGGGAGGTGGATAATCACTGCCGTCCTATACG

>M.01.2.1_A_183

TTCTGCATACTTCTTATACTCTTCTTTAGCGATTGCAAA

>M.01.2.1_A_184

TGTTATTGTAAGTACATCGTCTGGAGTTTTTGTAACCGG

>M.01.2.1_A_185

TGCAGTAAAACCGCAAAATGGACAAATTCCACCTGGTTT

>M.01.2.1_A_186

GTAATCGTTAATGTATTTCTTAAGGAATTTCATCCCCCCAA

>M.01.2.1_A_187

TAGAGATTCCGGCAAAACAACATTTCTGCAATTGGTTAAG

>M.01.2.1_A_188

ATTAAATTCAAGAAGTGCGTCTATAATAATTGTGTGCCGT

>M.01.2.1_A_189

GATGTACACCGGTTTCAGTAATTAGCAAACTGATATCGC

>M.01.2.1_A_190

TTAAAAATTAGATTAAATTATGAGTCAGGTAATCGGTTTT

>M.01.2.1_A_191

AATCCTAACGCTAAGGCTTCTGCGTGCTGTTTCAATTCAT

>M.01.2.1_A_192

AAAATAACGGTGACAATAAAAGTAGATGAAAATGATCTAGT

>M.01.2.1_A_193

AGTATGTCTCTATCAATTACATCTGGTATATCCTCAACTTCAG

>M.01.2.1_A_194

GCTGCAGGTGCTGCAGCCACTGGAACCCAAATTTCAGGA

>M.01.2.1_A_195

ATGTTAATACACGAGGAGAGAGGTTCCGTGGCACTTGTGCA

>M.01.2.1_A_196

ATATACTCGAATCCTACATCAAATCGATTTAAGTTTTCT

>M.01.2.1_A_197

TAATCAGTTCAACCCCACAGAGTCCATCCCCGACTCTG

>M.01.2.1_A_198

AACCACCATCCTGTGATTAATGCAACAACTACAAATACTAT

>M.01.2.1_A_199

CACTGGGAGGGGCATAGGGGTGTTGAATTCCCAGGCCTTGG

>M.01.2.1_A_200

CTTCTAATTCTTCTTTTAGCCTAGATGCAATCAGTATCACAT

>M.01.2.1_A_201

AGTGATAAGTTCATCACGATATTAGGATACGCATGTCAT

>M.01.2.1_A_202

GAGGTGGCATAATGTTAGTCAATCTCGGTGCATTATTACA

>M.01.2.1_A_203

TGTGCTGTTAATTCATATAGTACTACATAGCTACCATAGA

>M.01.2.1_A_204

TGGCAATCGTAATAATGCCCGTAACCGTAAGCTCTGAGAG

>M.01.2.1_A_205

CTATTTGTATACAATCTTATACATATTTCCTCATTATTCT

>M.01.2.1_A_206

TTCTCATCTAATATTATAGTACCTATATACATAGGTATTA

>M.01.2.1_A_207

TCCTCCCATTCCCGTTTTGCGGTGTTCGAACTCTAACTTG

>M.01.2.1_A_208

GATACAACACCGGGATTTAGATATGGACGGCAAAGTG

>M.01.2.1_A_209

TATATTATTGCATGTAAATTCTATAACTTATACTTCATACCG

>M.01.2.1_A_210

GTATGCTTATCGTATAAAGTAATTTGCCAAAGTTGTACG

>M.01.2.1_A_211

AAACCATCATTAATAGTTATACTCACTATTTCTGCTTGTT

>M.01.2.1_A_212

GTTAACATTGCTTGCACGATATCTTTCATATTAGTGT

>M.01.2.1_A_213

AGAGTACTCCCTGCGTAAATTCCGCCAACAATAGATATT

>M.01.2.1_A_214

AAATGGACTTTGGTTTTTCCTATTACTAAAACGTTAGCA

>M.01.2.1_A_215

ACGAGAGTGTTAGGATCTTCCCATCCTACCCCTAACGGG

>M.01.2.1_A_216

TCTTCGGTTCTTACTAACACCCTACCAGCTTTTACACCTT

>M.01.3.2_A_1

ATACATTTCCAGAAAACGTTAGCTGTCATTGTGGTATA

>M.01.3.2_A_2

CGTATAAGGTGCAATAGAACTGGAATTGTGAAACTTCTT

>M.01.3.2_A_3

TTTCTAACCTCATAGATTCTCATAGGCAATATAGCTACTT

>M.01.3.2_A_4

TAACCTATAAACCCGGGCAAAAACCCCCACCAAAAAATTTT

>M.01.3.2_A_5

ACCGCTAGCTCCGGAAGGAACTATTATGTTTATCGTTTTGC

>M.01.3.2_A_6

TGTTCTTTCTTTTCTTTGTTCAGATTTATATATTTGTTGT

>M.01.3.2_A_7

TTTCATATGGCACGTCCTTCGGGTCTATGTAGACGAGGTA

>M.01.3.2_A_8

TACGAAAGTAAGACGGAAAAGACATGGGAAGGAATAGAT

>M.01.3.2_A_9

TACCACCGTTGCATTTGTTATCTCTCGGTAGTATACTTT

>M.01.3.2_A_10

TAGTTGTAGCTGGGAATCTTGCTAAGTCGAATTCCCACATCACG

>M.01.3.2_A_11

AGCAAACTATATTTGTTTTGTAAAGATTTAATATTAAATTCCT

>M.01.3.2_A_12

GTCACGCCATTTCGTTATAATGTAAGTCCTTTCACCCTT

>M.01.3.2_A_13

AAGCCCTATTTAGGGGGTAAACCCCCTAAAACCCCACGT

>M.01.3.2_A_14

ACACTTTAAGAATTATGTAGTAGTTTATCCGCATGTACTT

>M.01.3.2_A_15

CCGAAAACCGTGATGTATACCTCATCAAAGAACTCTCCGT

>M.01.3.2_A_16

CTATGGTCGAGTGGCGTAAAAAGTTCCGTTTCCTCGTCA

>M.01.3.2_A_17

CCGTTTACGACAATAAAGACCCTCCTGAACCCGTCGCT

>M.01.3.2_A_18

CAATTTCCGCAAATTTGTTTTCGTAACGTGCGATACGTTT

>M.01.3.2_A_19

AAACTATTTAATAGTTTTATCCACCTTACTTTTTCTTCC

>M.01.3.2_A_20

CCCGTCGATGAAGGCGATTCAGGAGGGCCGTTAATTAG

>M.01.3.2_A_21

GTCAAAATTATACCATTCCGTGATATTTCTATTCCATTG

>M.01.3.2_A_22

TTTATTTTAAGATATCTTATGGAAACTGGTCTTGTGAC

>M.01.3.2_A_23

ACAACAGAACCAGCTGGAGCACTTGCCGCAATATTAAAG

>M.01.3.2_A_24

TTTAGTTTTCCGCCTTGATTCTGTGCTTGCTGTTCTGCTT

>M.01.3.2_A_25

CATATGATCATAAGCAAGAACATGTAGTTGCGTATAAATCT

>M.01.3.2_A_26

ACCAATTGCGCTCTGATTTTTCTAATGAAGTTATTTCTA

>M.01.3.2_A_27

TCAAAAAACAAATTTTTAGTAAGAGAAAAAATAGTTAAA

>M.01.3.2_A_28

TTGAAAATGATGATGAGCCTCATCTATCACAAATAAAGTA

>M.01.3.2_A_29

TTTCTTTTTGCCATTTGGGGATTTGCCTTAAATCCAAATT

>M.01.3.2_A_30

ATCGTCTGTCTCACCTTGATTATTGCCTTCTCCTTCATAA

>M.01.3.2_A_31

AAACTATCGTAAAAACGAAGCCTATCATATAAAGCGGT

>M.01.3.2_A_32

ATTGTAGTCAGAAAGTCTATTATATCTTGAGTAGTAATT

>M.01.3.2_A_33

AATAAATATCCTAATCCTAATCCACCGCCCGCTATTCCT

>M.01.3.2_A_34

CATATCCTCTCTTCCCTCACCCAATTGAAGAAGAACCCTA

>M.01.3.2_A_35

AAATAATATTTTCAGCATCATACTCTCCATCATCAACAACA

>M.01.3.2_A_36

AAAAGAGGACAGAACTAACGGTATTAAATAATGTAA

>M.01.3.2_A_37

AGTCTTTATAGCTATCCTTCTAGTTTCACTCAAGTAAGTA

>M.01.3.2_A_38

TGACGAATTTGTATAGAGTTAGACCTGCCCACACACCTGC

>M.01.3.2_A_39

GCATTATTCCAGGCTTAGGGACAATATTTGGGGCTGGGAT

>M.01.3.2_A_40

TACCTCAACCATCTGAGAAAGCACCTAAAAAGCGAC

>M.01.3.2_A_41

AGCCTTTATCTCGCCCGCCTTTTTTGCCTGGAGCATATTCT

>M.01.3.2_A_42

GTTCTAATAGCTTCTGTTTCAGAGATGGGTTAGCTAA

>M.01.3.2_A_43

TATACCGCCGTGAAGTACAACGGCTACGATGAAGGTAC

>M.01.3.2_A_44

AGTGAAATATTTGAATTATATCCAGTTATTTCATACGCCTG

>M.01.3.2_A_45

ACTGCACAACTTATATATGATGAATTTCCGGTACCA

>M.01.3.2_A_46

GTTTTATTAGATAAAAATGAAGTTGAAGAAAAAATTATTA

>M.01.3.2_A_47

CTCTGCGGATTAACACCGGCACTTATGTTCACCCCTTGG

>M.01.3.2_A_48

ACTATATAAATCCGAAAGCACGTTAGTGAGAGAGAAAGATT

>M.01.3.2_A_49

TCATCTCACCCTAGCGTTATTATATCATTATATAAGGAT

>M.01.3.2_A_50

GATTGATCTATTGCTGGTACTGGATAAAACTCATCAA

>M.01.3.2_A_51

CCGTTTATAAAGCCTCTTTCTTTTTTCCCACTTAATGAA

>M.01.3.2_A_52

ACAACGGATGGTCTCTTCGGTTCCGTCGCCTCTTTTAGAACTT

>M.01.3.2_A_53

GCATTCATTTGTCCTAATGTAGTCTGAGTTTGTTGATTAT

>M.01.3.2_A_54

TCATTATACTTAATTATGGATATTCAAACAGTTGAGG

>M.01.3.2_A_55

GAGAAAAAAGCCTCACCTCGGGGATTTTCGTGTTAAATGAA

>M.01.3.2_A_56

ATTCCAAGCACATTTAGGACAATAACCAATGAAACCTAT

>M.01.3.2_A_57

GTATTATTCGTATCGTTGCACTCACTGAGGTATTAAAT

>M.01.3.2_A_58

TGTCATTACTGTCTCTTATGCAGCTAAGGAAGAATAT

>M.01.3.2_A_59

TTATAACTATGTTTTTCGGGTGTAATTATGTCTAATATCTTA

>M.01.3.2_A_60

GTTTCATCGAGACCACTTAGGAAGAAGTCCTCTAGTTC

>M.01.3.2_A_61

ATAATCGTTCAGGGTGGCAACCCACAGTTCGTGATACAA

>M.01.3.2_A_62

ATGGAAGCTAGATTTTATATAGTAGATGACATTCTTAT

>M.01.3.2_A_63

CTCTCGTCTAAGCGTCCTGCATTCTCTACTAGCTCGTTAAG

>M.01.3.2_A_64

CACGATAAACATCTTTCTCGACAATCTAGCGTTTGCTCTAT

>M.01.3.2_A_65

ATCTTTATCAACCTCTGTCCGACATTTGAACCATAATCTT

>M.01.3.2_A_66

GGAAGGAATTGTAACTGTTCTTCATTGAACTTTATTACT

>M.01.3.2_A_67

TCTACGGTTTGTTCGCTCCAACGCTCGTGCAGAAGGCG

>M.01.3.2_A_68

AAGTTTGACTAGGAGTTACTGTTACGTTGATTGACGGT

>M.01.3.2_A_69

TGATGAGATTTCTTCATTAGAAGAATATCTATTACAGCCT

>M.01.3.2_A_70

GAAGATGATTTAGCTGATGAAATATGCGTTAAGTTGAAA

>M.01.3.2_A_71

ATTACTATAACTACGACATTACCATATCCAGGATTTCCCTG

>M.01.3.2_A_72

GTACTATAGTGCCGTTAATTCCATATGTGATCCAAGCACC

>M.01.3.2_A_73

TATCCCCCTAGAAGAATGTTAATATTAGCTAAATCTGATG

>M.01.3.2_A_74

GTGATAATAATCCAGTTGAAGTTGAAGTAGTCATAGATGT

>M.01.3.2_A_75

CAATATATATAAAATTGGCACAAAATCTAAATGCGAATA

>M.01.3.2_A_76

ACCTTTCCCCAAATTTACTATTTTCAACAGTAACATCC

>M.01.3.2_A_77

TTGTAGCTGGGAATCTTGCTAAGTCGAATTCCCACATCACG

>M.01.3.2_A_78

TATTATTCAAGTGTCGGGCTATATAACATTAGCGAGTGGA

>M.01.3.2_A_79

TATACTTTTCCTTATGCTCTTCTGAATCTTTCTTTTCT

>M.01.3.2_A_80

CCATTGGTAAAATGATATCATCACTAGAATGTTTATTATT

>M.01.3.2_A_81

TCACTTTTACTTAGTCAAATAGAACTCGCTTCTATGATTG

>M.01.3.2_A_82

TCAAAAATTTGTTCTTGACAGGTCAGAAAAGGACTGGTAAG

>M.01.3.2_A_83

TCTTCAGAGTGTTGGGGGCCGATAATATGGGGTTATTTA

>M.01.3.2_A_84

CTTGACATTATTCTTATACAATCTAAACAATAACTATGTG

>M.01.3.2_A_85

TACTTTATGCTAATGTCCCTGAGTTTCCTAATATCGATCG

>M.01.3.2_A_86

GTTAGTGTACCTCGATATAACTCAAGAAATTGAGTAGTT

>M.01.3.2_A_87

ACATCTTTGTGGCCGTGAACTTCCTCAGATATTTCGGGGC

>M.01.3.2_A_88

AAGCCCTGGCAAACGTTTACGCTTGAAGTGGGAGTTTCC

>M.01.3.2_A_89

AATTCATGTAGAACTACCGCATATGATAACAACACCG

>M.01.3.2_A_90

ATGCAAGTTTACTAAATTCAGATTTTCCATTTGCATCGG

>M.01.3.2_A_91

ATTAAAATACATATTATAACCCCAACACTTGCAAATTTTGT

>M.01.3.2_A_92

TAAATAATTATATCTTGTTGTTAATAATAAATTTCATGA

>M.01.3.2_A_93

TCAACTACTACAACTCCTGCATATGTATTTCCTACAACTCTT

>M.01.3.2_A_94

TTAAAAGCAAAAAAGGATCCTATAACTATCAATGTTATT

>M.01.3.2_A_95

ACTATGCCGGTTCGCAAGGGGCTACTATAGCGTCGTTAG

>M.01.3.2_A_96

AATGTACTCCCTGCGTAAATTCCGCCAACAATAGATATT

>M.01.3.2_A_97

CTACTTGTTTGTTTAATTCCTATCATATATATATATAA

>M.01.3.2_A_98

ACGAGAACGGCAAAGATCAAGCCCATCGTAAATAATTTC

>M.01.3.2_A_99

AATTTTATATATAGATGAGTAGTAGTCAGTGGAACTATA

>M.01.3.2_A_100

AAACGAAATATCGAAGGCGTCAGATGAGGAAAGGAAGAA

>M.01.3.2_A_101

TGTACTCTGGATAGGGGAACCCGACAATGAAGATAGCCTT

>M.01.3.2_A_102

TATTAACACATATTCGTAAAATTTTTGTTGAGATAGTGTT

>M.01.3.2_A_103

GAAGTTTCATTTATGATGTACCCGTAAGTCTGGAAGCCGT

>M.01.3.2_A_104

CAAATATCGTAGTTACTATCCAGATTAACCAGTCAACAA

>M.01.3.2_A_105

CCATTATGGGACGGGGATAATTCTAGCTAGTCAGAGAATT

>M.01.3.2_A_106

CAAAGTACTTATCAGCCGTAAAACATAAACTTAAGCTGAAA

>M.01.3.2_A_107

GATAAAATGTCTTGAGCTACTATTTTTAGATCTTTGAAT

>M.01.3.2_A_108

AAAATGACTGCAGTTATGAACAAATTAATACCCGCCATAG

>M.01.3.2_A_109

TCAGGTGGCTTTAGCCTCTTTACAACCTCTACCGTTTTCT

>M.01.3.2_A_110

TGCGAATCGTGGGCTAAAATATGAACTGGATCTTCATCG

>M.01.3.2_A_111

GTTTTAATGTCGATGACAGTTTTAGTTCTTAACTCAAGT

>M.01.3.2_A_112

TGAACTTTTCGTAGACTGTCTTTGGTACAATGTATGTGTC

>M.01.3.2_A_113

TTGTAACGTACCTGTAACCTATTTAGTATACTTCTATAA

>M.01.3.2_A_114

TGCGATTTCGCCGTTTGGCTTTAATAGCTCGGGGTGATTCT

>M.01.3.2_A_115

TACGTATATAAATCCTTTTTTGTTAACTCTCTTATGAGTA

>M.01.3.2_A_116

AAATAAGGAAAAACTGCCGAAAGAACTTCAACCAAAAAT

>M.01.3.2_A_117

ATGTTTAAGACAATAGCAAGAGAGATATTTAACGATGA

>M.01.3.2_A_118

AGGTATAGTGAAAACGGCTACATCATATCTTTTTATT

>M.02.0.20_A_1

TTGTTAGAAGACGAGGGGATATGGTATGGTGGTGTGATA

>M.02.0.20_A_2

AAATTATTGAAATAAAAATCAGATTCGATATAAAATT

>M.02.0.20_A_3

TTTTCTTCAGTCCCACCCTGTCGAGTGTGACCTCATAGG

>M.02.0.20_A_4

AAACCGCATCACAGTGGTTTTGAAGAAGTAAATATTTA

>M.02.0.20_A_5

TAACCTTAGATACATCGCTGACGTCAGTAGATATGCAAA

>M.02.0.20_A_6

TTACTAGGTCTGTAAGGATGACCGAAGCAGACCACGAA

>M.02.0.20_A_7

TCACAAGTTTCTTATCTTTATTTATAGATGGTATTATAT

>M.02.0.20_A_8

TTAATTAATTATTTAGTCCTTATTATTCAAGTGTCGGG

>M.02.0.20_A_9

TTATTTCTCAGAATATCAGAGAAATGGGATCGACAAACGGG

>M.02.0.20_A_10

TACCACCGTTGCATTTGTTATCTCTCGGTAGTATACTTT

>M.02.0.20_A_11

CTAGTCTCCAACCACTTAACTGGGGAGTTAAGTGGTTA

>M.02.0.20_A_12

ATAAAGAACACGTTCGTATCCCGACTTTACTAGGGAAACC

>M.02.0.20_A_13

TGTAACGGTTGAATATATTAATCACGTACACACGTATGT

>M.02.0.20_A_14

AAGCCCTATTTAGGGGGTAAACCCCCTAAAACCCCACGT

>M.02.0.20_A_15

GTTGTTTCACCGTAATCGGCGTTGAAATATGTTTTTA

>M.02.0.20_A_16

GAACTACTACTTGATGCCATTGATTGTTGGCATCAAAA

>M.02.0.20_A_17

TTCCCTGTTGGTGTACTGGCATTTTCTAGCACATCGTCAC

>M.02.0.20_A_18

TTTATGTTACCGCTTCTATAATCTAGCTTTAGCAGTACTT

>M.02.0.20_A_19

ACCTAGTGGTAATGGGGGATTCGCGTTAAACGTCACCTT

>M.02.0.20_A_20

AATACTTCATCAATAAGTAGACCACTGTGGTCAATTGGCGT

>M.02.0.20_A_21

AGTAATGTAACGAGATGATTAGGTTTGAACGTGTAAGG

>M.02.0.20_A_22

TAGGGTTTGGGATAGCTAATGCCAATCTAAGTCATCTAT

>M.02.0.20_A_23

AGAAAGAAACTGGGCAGAACTAAGAAACTTTATATCAA

>M.02.0.20_A_24

CTTCAAATTGAATGCTTAATCGTTTATCTTTTTTATAAT

>M.02.0.20_A_25

CGCTAATAAGACTGGAGTCTCCACATACTGACCTTGAATAA

>M.02.0.20_A_26

TAAATAACGAAAACCTCCTTACCAGACTCCATCTCTTCCTC

>M.02.0.20_A_27

CTTTTTAACATTAATATAAATAACATAATCGATATCAA

>M.02.0.20_A_28

ATTTTAGTCAAGAAATCTATTATATCTTGAGTATTAAT

>M.02.0.20_A_29

GTCGTCTTGGTACCGAGTTTCCTAATTGCGACGTATACG

>M.02.0.20_A_30

ACAACAGAACCAGCTGGAGCACTTGCCGCAATATTAAAG

>M.02.0.20_A_31

TTTACAGTGATGCGTATCGTTTTCAGGATCAAGAAGTGG

>M.02.0.20_A_32

CTTCGGCATAACGACAGGTCAAGCGACGTATAACGCAAATT

>M.02.0.20_A_33

GTTAAAAAACCCTACTTTTTCCTAAACTTTTAGGGGAA

>M.02.0.20_A_34

GTGACTGCAGCACCTGCAACAGGTGCCGTTCCAGTGCCGT

>M.02.0.20_A_35

TCTTGTAGCCGACCTCATACGTCAAATTCTGCCTCAGT

>M.02.0.20_A_36

TTGAAAATGATGATGAGCCTCATCTATCACAAATAAAGTA

>M.02.0.20_A_37

AAAATATATTCTTAGCGGTGCTAAAGTAGTGACTACAAT

>M.02.0.20_A_38

AGATGGCTGCAGTTATGAACAAATTAATACCCGCCATAG

>M.02.0.20_A_39

TTCCTATTAATTCATCAGTATCTTTATAACCACTGTTTACC

>M.02.0.20_A_40

TTATCAAATAATGTAATCCTACCAGTACGCCTCTCGTAT

>M.02.0.20_A_41

TCGAATTCGTTGAAAATGGGAAGTCGCTGATTAAGAGGG

>M.02.0.20_A_42

TTTCTATTAAGAGAAAGTTACACTTTATCAAAAGACACT

>M.02.0.20_A_43

ATTACTAAAAGGTCGTTCTTGGGATCGTATCTATATC

>M.02.0.20_A_44

GTCCTTCATCTCATGACGAAAGAACTTCATTCCATCCGA

>M.02.0.20_A_45

ATTGTAGTCAGAAAGTCTATTATATCTTGAGTAGTAATT

>M.02.0.20_A_46

GTGGGTTTACAATAGTGATGAAGTAAGGTATTTGCGG

>M.02.0.20_A_47

TCATTTGTATACAATCTTATACATATTTCTTCATTATTCT

>M.02.0.20_A_48

GAACAGGTCGAAAGAGCTGAGCTTTGGCATGCAATTTA

>M.02.0.20_A_49

TATTTTCTATGGCTTTTCGTATGGCTTCAGATCTGTTTA

>M.02.0.20_A_50

AGAAAGGCTAAGCCAGATAGCGAATGTATGGAATGCCCCT

>M.02.0.20_A_51

GTTCTCTGTTCTGCCATATCGCTATCCAGTAATCACT

>M.02.0.20_A_52

CTTGTTCTAACGTCGCATATGCAGAATCTATTAATCCGCGT

>M.02.0.20_A_53

TTTGTGAGTTGGAGACCGTTACGGCCACGCATTCCACGCGT

>M.02.0.20_A_54

CCAAAAATCTCTTTACGTTCTCTAATGATGCTAAAAGTTC

>M.02.0.20_A_55

GTAATCATATCAGGAAACGAAGATGTTTACTTTCCTAATA

>M.02.0.20_A_56

AGAAGAGGAAAAACTTGTAAGTCAAAATTCAATTTATTT

>M.02.0.20_A_57

GTTCTAATAGCTTCTGTTTCAGAGATGGGTTAGCTAA

>M.02.0.20_A_58

TTCCCATGTGATTGTCCCTATCGTTAGGTTCCACGTA

>M.02.0.20_A_59

TGGGAAATACTCATCCGCCATGTTTTCACTAAAACATA

>M.02.0.20_A_60

ACCGCTAGCTCCGGAAGGAACTATTATGTTTATCGTTTTGC

>M.02.0.20_A_61

TAATTTTTTCGTTGTTTTTATCAAAACACAAAGCACTA

>M.02.0.20_A_62

CTGTAGAACTCGTCCTCGTCGCCCCAAAGCGGAGCGAAGT

>M.02.0.20_A_63

ATGATACCAACATCTTGAAACAACTCAATAATAAACAAG

>M.02.0.20_A_64

GTTTTATTAGATAAAAATGAAGTTGAAGAAAAAATTATTA

>M.02.0.20_A_65

AAGATCGTGGACGCGAGCACCGTAGACGAAGAGATAATCCT

>M.02.0.20_A_66

GAAGAAGAGTTAGCAATATTAGATCTATATTGCGCAAATATG

>M.02.0.20_A_67

TCATCTCACCCTAGCGTTATTATATCATTATATAAGGAT

>M.02.0.20_A_68

TCTATATTGAATTCTATGCATATTCAATATTTCTCAACACCTT

>M.02.0.20_A_69

TTTTTTTACGCGTCTCGTCACGTAATCGGAAAGGGGTGAT

>M.02.0.20_A_70

ATCGCATTCTTTTTTCTTGAAATATGAAAAAAATCCGGC

>M.02.0.20_A_71

GTAACTACTTCACAGTCCCCTCTTCCAGTATATACAC

>M.02.0.20_A_72

TCATTATACTTAATTATGGATATTCAAACAGTTGAGG

>M.02.0.20_A_73

AACTCCAGTACTGCTTCGCCGTAGGATCCGTCCTCAGACG

>M.02.0.20_A_74

TATACCTTGACTTAAATCTACTCCTTCTTCCTTACAA

>M.02.0.20_A_75

GGAAACTATAATGAACTTATGAGAATTGGGAATGTTGAA

>M.02.0.20_A_76

TGATATTGTAATGCTATCTTATTCGCATTTTCTGGTATT

>M.02.0.20_A_77

CCTTACTTTTTCTTCCCCTTTGATACTTAATATACTTAA

>M.02.0.20_A_78

GAAGTCTGCCGCTTAGTACTATGAGCTTAGAATCTATA

>M.02.0.20_A_79

ATCTTTGGGAGGGCGAGCGGGTTTATCCCGAAGTTCGGG

>M.02.0.20_A_80

ATAATACGGTACCTACGTCCATTTGCGACGTCTCATTAAT

>M.02.0.20_A_81

CTCTTCGTTGAAGAACGACGAAACTGTTTCAACCACATATT

>M.02.0.20_A_82

TTAAGTTATTGGGGACAATACGGGGGATTTTTATTCAAT

>M.02.0.20_A_83

CAAATAAAGGCAAGCCCGCAGGCTAATACACAACCTACATTAAA

>M.02.0.20_A_84

GTTTCATCGAGACCACTTAGGAAGAAGTCCTCTAGTTC

>M.02.0.20_A_85

AATCTACGGTGCAGCAGGTCTATTTTTTGGAGTATTGA

>M.02.0.20_A_86

ATAATCGTTCAGGGTGGCAACCCACAGTTCGTGATACAA

>M.02.0.20_A_87

TTATCTTTCAACAATACAGTATGCATGTTTCAGGGATGCCG

>M.02.0.20_A_88

TTTGTAACCACCTTTTCGGTTACGCTCTCAAACTTACCACTAAT

>M.02.0.20_A_89

CGTATTCAATATCATTTTTAATAAGAAAATTAAGAAGGGG

>M.02.0.20_A_90

TCTATATTATTAACTTCTATATCTATGTAAAGTACCTTAT

>M.02.0.20_A_91

CACGATAAACATCTTTCTCGACAATCTAGCGTTTGCTCTAT

>M.02.0.20_A_92

TAATAAATTATCTTTATTGTTCCCTCATAATCATATTTAA

>M.02.0.20_A_93

CCTGAATCCCAGATAAGATATGCACCAGAAGACAGCGT

>M.02.0.20_A_94

GGATTAAATATAGAAGTGAAACAGAAACGAAGAAAAAAGA

>M.02.0.20_A_95

TCCAGGACGGAACAAAGCTAGGGGAATTATTTGCAACGTCC

>M.02.0.20_A_96

ATAAGAAACACTTCAAAAAGTGTTATCATTCTTCCTCA

>M.02.0.20_A_97

TCCTTAAGGTCTACAGGTACACCGAGATCATCAATAAAA

>M.02.0.20_A_98

GTGTTATATTAGGGTATCTACTAGGAACAACATTATATG

>M.02.0.20_A_99

TCTACGGTTTGTTCGCTCCAACGCTCGTGCAGAAGGCG

>M.02.0.20_A_100

TAATCCATTTTTTTCGACGTGTTCTTTAATCTTTTTCAAC

>M.02.0.20_A_101

AGCAATGTAATTTATAGTGTAGTAGTGGAGGTCGTGTTTAC

>M.02.0.20_A_102

GTTAGCAGAAATAATAGCTACATATGACCCTAACAATGT

>M.02.0.20_A_103

TACTCTGTATTATCCGGATAAAGATAGAATACTTTTCT

>M.02.0.20_A_104

TATCCCCCTAGAAGAATGTTAATATTAGCTAAATCTGATG

>M.02.0.20_A_105

CCGAACTCATCCCTACTGTATCACTTAATAATATATTAAT

>M.02.0.20_A_106

TTTGCTATTATCTCTTCTTCTTTTAAGTTGAGTGTAAG

>M.02.0.20_A_107

AAACGTAAGTTACTCTTTACGCTTACATTGAACGTAACAGT

>M.02.0.20_A_108

AAATGATTTGTTTAGCGCTTTTGCAACTTTATATGCTATA

>M.02.0.20_A_109

ATTTTAGTGTCTGTTTTACTACTCATTTTTTATTACCATTT

>M.02.0.20_A_110

ATTCTTCTAAAATCATTCGCAAATCCTCCTCCTACATCCCCT

>M.02.0.20_A_111

CCCTATTAGTTCATCAGTATCTTTATAACCACTGCTTACT

>M.02.0.20_A_112

CCATTGGTAAAATGATATCATCACTAGAATGTTTATTATT

>M.02.0.20_A_113

TCTCGTGGGTCGAGTCTCCCAGACAACTTTGCGTTACAT

>M.02.0.20_A_114

TCAAAAATTTGTTCTTGACAGGTCAGAAAAGGACTGGTAAG

>M.02.0.20_A_115

CTTGACATTATTCTTATACAATCTAAACAATAACTATGTG

>M.02.0.20_A_116

ATCCTCTTCCTGGACTCACGGGACGTCGAGGTCTTGTT

>M.02.0.20_A_117

CCAACGTGACTGTTGTATATTCAAACTCTACGTCTACTAC

>M.02.0.20_A_118

AATGAATTCTATTTCTCTCGGCTCTCTTGTGTAGAAAAG

>M.02.0.20_A_119

AAGCCCTGGCAAACGTTTACGCTTGAAGTGGGAGTTTCC

>M.02.0.20_A_120

ACGTACATAAAAATTGCTATAGCATTTGGAGCTGCGGCA

>M.02.0.20_A_121

AAGAAGTGGTACGTGACGTGGGATTGAAAATGTACAAG

>M.02.0.20_A_122

ATACTAAGAAGTAACGTAACAGCTTCTGAGAGAACAAAG

>M.02.0.20_A_123

TATTGTAATGTACACATTTCCTAGGTTACTTCTTTCCA

>M.02.0.20_A_124

TCCTTTGGAACTCCCTCAATACATCTTTGTGATATAAA

>M.02.0.20_A_125

ATCTTCATCTCCAAACTACTGCTATTAACTTATCTCACTC

>M.02.0.20_A_126

TAAATAATTATATCTTGTTGTTAATAATAAATTTCATGA

>M.02.0.20_A_127

TCAACTACTACAACTCCTGCATATGTATTTCCTACAACTCTT

>M.02.0.20_A_128

ATCTTTGCTGTACCGCTAACCTGTAGGACTTGATGGTT

>M.02.0.20_A_129

ACAAATAGAATTGCGACCGGGTACAATTTTGACAACAAAACTA

>M.02.0.20_A_130

TCATCCTTCTCGTGGCTATATATAAATCCACCGAAACCG

>M.02.0.20_A_131

GTGATAATAATCCAGTTGAAGTTGAAGTAGTCATAGATGT

>M.02.0.20_A_132

TGTTTTTGATTTTTTGCAGTAAAACCGCAAAATGGACAA

>M.02.0.20_A_133

CTCGTACTTCTTACATGATGGATAATGAGTTCTGCAGAT

>M.02.0.20_A_134

CTTACTTTATGGTCCCTTTCTAATTATGCTGACTTCAG

>M.02.0.20_A_135

AGTACAGGGACGTTCAATGAGTAAGGCTCCGCAAGGTAG

>M.02.0.20_A_136

TTGCGGAGAGGGACAGGTCGCTCAAACCGACCAGCAAA

>M.02.0.20_A_137

AAACCTATAAGCCCTAAACTGAATTCCTCTTCTTTCTTTTGT

>M.02.0.20_A_138

TTAGTTTTATATCAGATAATAGGTTTACTAAAATTATTG

>M.02.0.20_A_139

AAACGAAATATCGAAGGCGTCAGATGAGGAAAGGAAGAA

>M.02.0.20_A_140

ATCTTCTCCATGATGTTCTTGTAGGTAAACCGCTTGT

>M.02.0.20_A_141

ACACGTATTATTGATCCGTTATGATCATAACATAACGCAC

>M.02.0.20_A_142

TAGAAATAGTATTTCCGTCGGTGGTAGTTAAGTGCTCAAAAAA

>M.02.0.20_A_143

TTTATCGGGGTCATCCCATGTCCGCAACCCAAAATCAA

>M.02.0.20_A_144

TATATTATGAATAAGTGTTAGGAGTTGGCGTATGTCCT

>M.02.0.20_A_145

ATATTATCATCATTGAATATCTCTACTGTATATGTTTG

>M.02.0.20_A_146

AATTCTTTCAATCTTTCTCTTATTGCCTCGTTAACAAATTC

>M.02.0.20_A_147

CTAGCATATGTTAGATTTAGCGATTGAATTACAGTTCCCGCT

>M.02.0.20_A_148

TCTGAAGTTGTGCCCCCCTCACTTTCACTTTCCTCCTC

>M.02.0.20_A_149

TGTACTCTGGATAGGGGAACCCGACAATGAAGATAGCCTT

>M.02.0.20_A_150

ATTACTTGCAAGACTTGGTGGTTTGCCCCATAGTTAGTAT

>M.02.0.20_A_151

TCTACAATTTTGGAGGAATTTGGATTAGATTTAACTTTAT

>M.02.0.20_A_152

GTAAATCCATATGAGTTCTATCAACTACTTCAGCAAACTGGA

>M.02.0.20_A_153

ACTTAACTCTGATAATGCCTCTTTTAATGCTGTAAA

>M.02.0.20_A_154

ACTGTACTTCTCTATGTTTTTTGTTCCTTATAAATATTT

>M.02.0.20_A_155

ATCCCTAAGCCCGCTAATGCACTCCCCGCCCCAATTCCCCC

>M.02.0.20_A_156

GAAGAACTCGGCTTTCTCCTCGTCGTCCATTACGGACAT

>M.02.0.20_A_157

CTACGAATTTAACGCCCGCCCTAGATATCAATGATGCCTCGG

>M.02.0.20_A_158

ACAGTGTGGCATTAACATCAGAAGTTTATGAAGAAGACCC

>M.02.0.20_A_159

ACTGATACTCTATCCCATATATTGCAAGATCCCTATC

>M.02.0.20_A_160

CAGATAATCCACTCAATCATCAAAACGAAATTCTTTGAA

>M.02.0.20_A_161

ATTAGATTAATTTTTCTTATATTCTGAGGAAGTATGAATCA

>M.02.0.20_A_162

CATATCCTCTCTTCCCTCACCCAATTGAAGAAGAACCCTA

>M.02.0.20_A_163

TATTGTCCTGCAGTCTCGTAATATACTTTAGTCCCATACA

>M.02.0.20_A_164

AGGGCATTTCGGCTCAACAATAGAGCATGATATATCGCATG

>M.02.0.20_A_165

TATAATAATTAAATACTTTGGTGTTAGACTGAAATCTCTT

>M.02.0.20_A_166

CTCAGTTGCGGAAGAAAAGTTTATTAAAATGGAAAGCG

>M.02.0.20_A_167

TGCGATTTCGCCGTTTGGCTTTAATAGCTCGGGGTGATTCT

>M.02.0.20_A_168

ATAGAACCCGAAAAAGTCACATACGGCGGTCAAGAAATT

>M.02.0.20_A_169

ATTTATAACAAACTCCAAGAATGGGAGAGAGTAAACAT

>M.02.0.20_A_170

TAGACCCTTCTTATCTACTTAGGAAGTTAGACGGTTACC

>M.02.0.20_A_171

CTGTGGATGCTGACTATTACACTTATAAGGCTGAACTCG

>M.02.0.20_A_172

TTAGTTTGAACAAGTTGGACATAACGTCCGTAGAACC

>M.02.0.37_A_1

TTCCTTTATCTGTGAGTTGCACAATTTTCTTTATAGATC

>M.02.0.37_A_2

CCACCACCACAGTATATAATACATTTGATGGATTATTAA

>M.02.0.37_A_3

TCAACATCGACATAATATACTATAACTTCCGCATTATCAA

>M.02.0.37_A_4

GTTACAACAAACTATTAAGCGGCATTGCTCCTTCTGATG

>M.02.0.37_A_5

ATATTGGTAGACAAACAAAGTATTACTGAAAATTCAT

>M.02.0.37_A_6

ACCGAGGAAAGTGGCGAGGGACGGTCTGCGGACATAG

>M.02.0.37_A_7

TATCCCGTACGTCTGTACTCAAATTTTATTACCTTTCT

>M.02.0.37_A_8

TTACCTTTACGGATATGAAATTGATGATATCGCTGA

>M.02.0.37_A_9

AGAAACAGTGTATGGGATAAATTAGGTATAAGTGGCG

>M.02.0.37_A_10

TTATTTTCTTCTTCTGCACCCCCCTCTAAATTTTGTTG

>M.02.0.37_A_11

AATGAAACTTTCATAATAACTGAGATCGACCCATTACCAG

>M.02.0.37_A_12

TATTTTTCTAGCTTCTTCTTGAAGTCCTCAAACTCTAT

>M.02.0.37_A_13

AAAAAGGAATGGAAAGAGCAGGAAATGAAACTATATG

>M.02.0.37_A_14

AATGGCAGCCATATATAATCAAAAATCGCTTCTACTAAAC

>M.02.0.37_A_15

TATACGTCAGCTATAACTATAGCATATGGTTATGCACCAG

>M.02.0.37_A_16

TTCGAAACTAACCTCATAATTACGGCATACGCGTATTTTT

>M.02.0.37_A_17

GGCGTGTCGCCTCATCTTTATCAATTCGTAAAGCTCCATC

>M.02.0.37_A_18

TTAGTAATGATTCTCTATACTCACTTTTATCTAATTTACTTC

>M.02.0.37_A_19

AAATGAGCGTACGCCCACACCGGAAGTCTTCCGCTTAGT

>M.02.0.37_A_20

TTCTAAATTCTTGAAATTCTGCAGACTAGTAAGGCCCT

>M.02.0.37_A_21

TTGTTTTGACAACTTCATTTCCGCTCACGGAAACCTT

>M.02.0.37_A_22

TTCGCGTTTCATTACCGTTGCCATATTCCGTGAGAAAA

>M.02.0.37_A_23

CCGCGGAATCCGCGGGTTCAAAGTCGTCTCAAGTTGGA

>M.02.0.37_A_24

GTTATATGGCCTGAAGTGGGTGTTAAGTACGACGATGTAAC

>M.02.0.37_A_25

GCAGGTTATGGCAATAATGGTATTGTTTCTATCACAGTAAA

>M.02.0.37_A_26

TTTATAGCTAGAGCAATAAGCAGCAATCGGATGGGTAG

>M.02.0.37_A_27

AATAACAAACTTAGGGAAAGCGTCGCAAAACTTAGAAA

>M.02.0.37_A_28

ATAACTGATGGCGTTGCTGAAGCTATCGGATGGGCATCTAT

>M.02.0.37_A_29

TAATGCCTATTCTCATGAAGTACTTCTTTCTGTGCTAC

>M.02.0.37_A_30

TTTCCATCGTTTGTTTGCCCTGCAGCTAAAGTCACAACA

>M.02.0.37_A_31

TTCGATTTTGGGAATCCCACCGCCCAGCAATTCGCAGAC

>M.02.0.37_A_32

TCCATGAACTGCCTGAGCTGACGAATTGCTACTATCAAAG

>M.02.0.37_A_33

TTTCTATTAAGAGAAAGTTACACTTTATCAAAAGACACT

>M.02.0.37_A_34

GGATGTTTGATCGCTTTTTGAATCCATTTGCCCTTCTTAGCCAT

>M.02.0.37_A_35

AATATTAATTTAAGTATACTGCAATGTATCCATTCTGAAGA

>M.02.0.37_A_36

TCGTAACCCTGATTTGGTACCAACCCTTTTCCCTCAG

>M.02.0.37_A_37

TATATATGATCCTTTGTATTTATGTCCTCCCCACTCATATAG

>M.02.0.37_A_38

TCTAAGTTCAAGAATTGGCTTAAGGCTTTGGTGAGCCGGA

>M.02.0.37_A_39

AAAATATCTTACTTGAGGTTTAGAAGAGAGAATGGTGAAG

>M.02.0.37_A_40

ATTAGATAATTATAAACTTCTTTTTGTTTATATTGAA

>M.02.0.37_A_41

ATCTAACTATGATAAACTGGATGTAGAAATATTATTTAGA

>M.02.0.37_A_42

GAATTGTATAACTTTGTGTAGATGTCGTTGGGCCACTTCC

>M.02.0.37_A_43

AGATAAAAAAGCTGAGGTACGTATGCCCACACCCTGGCTT

>M.02.0.37_A_44

TTCTCTTGTTCTTTCGCGATCATATAAAAGTACTAAAGG

>M.02.0.37_A_45

AATTTACTATTCTCTACTAAAGTGTCTTGTACTACTAA

>M.02.0.37_A_46

ACTAAGTATCAGCAATGCTCAGCTGAGAATTTATTACCA

>M.02.0.37_A_47

ATACTCTGTTCCTTGCTCCATCTTTAGTAATTTGCTAAT

>M.02.0.37_A_48

TCTTTTACTAAGAAGCTTAAAAATATCGATCCATATATT

>M.02.0.37_A_49

TGCCTTCCGCTTCTACTTGTGTGTAATCTTGATTTGATA

>M.02.0.37_A_50

AACTCCAGTACTGCTTCGCCGTAGGATCCGTCCTCAGACG

>M.02.0.37_A_51

TATTTAATACAATACCCTTTTCACGCATTTGCCCACCACT

>M.02.0.37_A_52

GGGTCTTTCAGAATTTTTAGTATTTCCGAAAGTCTAGCCCC

>M.02.0.37_A_53

GCAATAATACCGCAACAAATTCCAGACCCACCTACTTATAC

>M.02.0.37_A_54

TCTTATTTTCATGCAACTATACATCTGCTTTATCATACTTA

>M.02.0.37_A_55

ATTCCAAAGTTACGTCATCGATGACCACGTAATGGAAA

>M.02.0.37_A_56

ATGAAATGTAATACGCTAAAAAGTACGTGTTATACTT

>M.02.0.37_A_57

CCACCACCGCCGCCACTACTACCACCACTGCCAGCACTGC

>M.02.0.37_A_58

GAGGGAGTATAGTTGAATCGAAGTATGACGTACCAATCCCAT

>M.02.0.37_A_59

TAATCCACATAAGCCCTCTTTCACTCCCGTACTTTGTCG

>M.02.0.37_A_60

CTGAAAATGGCATTGTTATAACCTTTACTTCGTAATGTAC

>M.02.0.37_A_61

CATACTGTGGCATATGTTTCATTCGTTTTAAGAGTTTA

>M.02.0.37_A_62

CTCTCGTCTAAGCGTCCTGCATTCTCTACTAGCTCGTTAAG

>M.02.0.37_A_63

AAAGTAACGGTGACAATAAAAGTAGATGAAAATGATCT

>M.02.0.37_A_64

ACCGGCGAAGTACTTAGTATATCAAATATATAGAAGTAACTT

>M.02.0.37_A_65

AGAGGAGTGTCGGGATCACAAGACAACATAAACAGGATGGT

>M.02.0.37_A_66

GAACTGAACCATACTGTACCGTTTTCGGTGTTTTGTAT

>M.02.0.37_A_67

TAGGTTTTTCGATTTTAAGAGGCTCTTCTACTTGCTCTA

>M.02.0.37_A_68

CAACTAAATAAATAGAAATTTCACTAACCAACACATAACT

>M.02.0.37_A_69

CCAACACTAGCACCAGCACCTCCATATGTCCATGCTATC

>M.02.0.37_A_70

CTTGTATCGACAATTGATGCACTAAATGATAATGACTGTC

>M.02.0.37_A_71

TGTCCGCTTGTGTGTGTCATTTGCAATTCCTCATAATA

>M.02.0.37_A_72

TCTTTCTCTGAGGGATACATCGGCGATATCTTCAATAA

>M.02.0.37_A_73

AATAATATATTTATATATCTTTTCTGCATCAACTATCATTCCT

>M.02.0.37_A_74

CCAAAAAAGTAAAAGTTAGAACATTAAAATATGAAGCA

>M.02.0.37_A_75

TCGGCGGATCCTACCCGCCTAGGCAAAATGGACACTT

>M.02.0.37_A_76

GAAAAAGTAATAGAGGGCATCTTTGAATTAGGCTTATTT

>M.02.0.37_A_77

TACGTATCGCTTCACTATAGTTTGTAGTGTTATACAGTT

>M.02.0.37_A_78

TACTTCCTCTATTCTTCGTGATCCTTATCTTTTTATATT

>M.02.0.37_A_79

TGCCTTAAATTGTCCTTTTTGTTTGTGCAATGCAGTTAC

>M.02.0.37_A_80

TCATCGAAAACGATTATTGATGGTTTTTGTGAAAACAGC

>M.02.0.37_A_81

TACTTCATCACGTCCACGTTTGTTATTTTTGTTGATTTAA

>M.02.0.37_A_82

TTAATTACTATTGCTATCTTGCTATATAATGCTTTTTTCGC

>M.02.0.37_A_83

TAATTATCCTTGAAAGTGTTGTTTTTCCAGTACCGGGCG

>M.02.0.37_A_84

CTTTTGACTATAAGGTGCCTGTTAAATATTAGCTTAACT

>M.02.0.37_A_85

TAATATCATACATGGACCTTCGCACGAGAAGTATTCAA

>M.02.0.37_A_86

ATTATCCTCATATCATTAATTAATTTTCTATACTGGAATA

>M.02.0.37_A_87

GAAATAGAGCTAAAGGAAATCGAAGTTGAACCACTCGA

>M.02.0.37_A_88

TTAGTTTGAACAAGTTGGACATAACGTCCGTAGAACCCTA

>M.02.0.37_A_89

TCGCCAAAGTTTCCTAATGAGGAAGAACCCGGTAAGACCTA

>M.02.0.37_A_90

TTCATCACTTTCCATTATGTTTCTTATAGGATATCATCTT

>M.02.0.37_A_91

ATGTTTGGTAAGTTTAGCAAGGCAAGTTTGGTTCAG

>M.02.0.37_A_92

GTATCAGTAGAGCATATATTAGCTAACATTTCCACATTAT

>M.02.0.37_A_93

ATATATGCTGTAATCTATAGACTAGGATATACTTTTGTT

>M.02.0.37_A_94

TTGTAGTTGCATCTCCTTATTGCCGTTTCTGAATTTCTTTT

>M.02.0.37_A_95

TACTAGAATTAGTTCCTCATTACTCTTAGAAGACTTCTC

>M.02.0.37_A_96

TGTCCTCTTCTATGGGCATGGACTTAGGATTAGGGTTTTTA

>M.02.0.37_A_97

TAATTTAACTAATAACGCGGGGGTTTAAAAATTTAACGGT

>M.02.0.37_A_98

TCTAAATTTTGTTCTATGTCGGAAGAAGTATAAATGCTAT

>M.02.0.37_A_99

AACAAAACGCTAGATTGTAGAGTTATATTAGTAATAGCG

>M.02.0.37_A_100

TTGTAACGGTATGTGCGTTTGATGGGATTGTAGTCGTAAT

>M.02.0.37_A_101

GTATGCTTATCGTATAAAGTAATTTGCCAAAGTTGTACG

>M.02.0.37_A_102

TTATATATCTTATTAAACTGATACTCGTTGACTACTATCA

>M.02.0.37_A_103

TATCTTCTAATACCTTATTCTTCTCTTCCAGTTGCTGTAT

>M.02.0.37_A_104

TCTTGTATGATAATCCATTCCTCAGTTCTATCTTTAGTAT

>M.02.1.13_A_1

TCACTCTTTCCTCCTTTTTCATTTTGTTCTGTGCCCAA

>M.02.1.13_A_2

CAATCAATCGTAAACGCTAAATCAAACTCCTAACTTTATC

>M.02.1.13_A_3

GCTGCTTGTAACATTCTTAATAAATTTATATTTGTTATC

>M.02.1.13_A_4

CCCGTCGCCAACGTTCCGTTGAACGCTTGCCAATTTGCCA

>M.02.1.13_A_5

ACTCCGCTTACGATTGATACAGTAACGGCACCGACGA

>M.02.1.13_A_6

CTAGTGAGACAGACCCAATAAACGAGACGAAAACAGCCCT

>M.02.1.13_A_7

AAGAGGTGGTATGTCACATGGGATTAGCTATGTATAAAT

>M.02.1.13_A_8

CCACCACCACAGTATATAATACATTTGATGGATTATTAA

>M.02.1.13_A_9

TTCTTATTGTATTTCATGATGCCTTGGTTTGCAATATCT

>M.02.1.13_A_10

CTTGAATTTTGTATAGCCTTTAATGAACCTAATAGAATT

>M.02.1.13_A_11

TACAATTAATGCAACAACTATAAATACTATTACTAAGGG

>M.02.1.13_A_12

ATTCACACCAATAGTTACCACGATGAGTCGGTACGTGAT

>M.02.1.13_A_13

TTCCAAGTGCAGCGACGTTCAATGAGTAAGGCTCCGC

>M.02.1.13_A_14

CAAAACAAGGTGATACATGCAACTTACATGTATTATATCTA

>M.02.1.13_A_15

TAAAAGGGTTAATGACAAACTGGAAAGCTACCGTGCTCAATTTC

>M.02.1.13_A_16

ACGTATGATTTGCCGGTTTTCTCTATCAATTCCATTCAA

>M.02.1.13_A_17

AGAAACAGTGTATGGGATAAATTAGGTATAAGTGGCG

>M.02.1.13_A_18

ATTTATTTTAATATTATGTATAATGCTTAATAACTCTCTA

>M.02.1.13_A_19

ACCGACCATCAGTTTTAACATTAACCCAAATCAGGGCA

>M.02.1.13_A_20

AAACTAATTCTTTTAAGCTTAAGTATTGGGGCAACCAGA

>M.02.1.13_A_21

CCATCTATTATGGTACCGGGTTTGATGTACTCTTCAACTA

>M.02.1.13_A_22

CGAAAGGATTCTTTCTATTCCCGTTATCAGTAAATACACT

>M.02.1.13_A_23

TGATTAAAATAGTTAGCTATCTCGGTAGCCGTTGTTAG

>M.02.1.13_A_24

AAATAAAGTGAGCTAAATGGCGGCAAATCAAGAAGTATG

>M.02.1.13_A_25

TAGCAACTGCATTTATTAACATGTTTAAAAATTCTAAA

>M.02.1.13_A_26

ATTTATTGTGAGTGCGGACTTTCAAATAATCTAGACGT

>M.02.1.13_A_27

TTTTGTTTTCCCCATTTTTCACTATCTTCGTTGTTCCTT

>M.02.1.13_A_28

ATATTTCCGCTATTTGGATTTATAACGTCTATAATGAG

>M.02.1.13_A_29

AAATTTAATATGTATATCGATATAACCGGTACTCAGTT

>M.02.1.13_A_30

TTTTCTATCAGTCCACCGCAAGGAGTTACTAGAACAAT

>M.02.1.13_A_31

CTAAATATGAGAGATTCACATCTTTTATAATCTTCACCAA

>M.02.1.13_A_32

TATCTTAATCCCGGTGTTGTATCTGGTGCATTGCGGAACAA

>M.02.1.13_A_33

ACGCTTAATGAACTCAAAGCGAAAACGGGAAAAACGAT

>M.02.1.13_A_34

TTAACACTTAAACTTGAGTTATCATCATCAATAGATAAAA

>M.02.1.13_A_35

TTAATAAATCCACTTAGGTACGACACTTTTAGCACTATACT

>M.02.1.13_A_36

GTACTTTCATCTCTTTGTCGTAATACTAAATGATACAT

>M.02.1.13_A_37

CTAATCAGTTCAACCCCACAGAGTCCATCCCCGACTCTGC

>M.02.1.13_A_38

AATTCGTTAGAAAACGGGTTTTCACAAGCACTTGAAGCGTT

>M.02.1.13_A_39

AGTTCATAATACCCTTGACCAGTGCCTGGTGCTAAAGCGT

>M.02.1.13_A_40

TTATTGTTGATGCTAGAAGTGTAAATGTTGAAGGTAA

>M.02.1.13_A_41

ATAAACTTCGGTTATAGTTTTACTGGTATAGATGGGTTAT

>M.02.1.13_A_42

CTATTTCGTGGTCTCAGACATCGAGTTACAACAATATTAT

>M.02.1.13_A_43

ACGCCACCGCTTCTAACTAGCGGAACTTTCGGGCAAAG

>M.02.1.13_A_44

TTTCAAACGCTATTACTTGTTCATTAGACGTGTTAGTTAT

>M.02.1.13_A_45

TAAGTCCTCGTCATAGTAATGATATTGTATTGTCTTAAC

>M.02.1.13_A_46

TTAGACCGTAGTAATTCGCAATATTCTTCGCAAATGTAGA

>M.02.1.13_A_47

AAACATTCATGCAGTAGATGGCAAAGAAGTAAAACAACT

>M.02.1.13_A_48

CTGTGTCTTCAAATATTATAGTAAATATCTCATATTTTTTC

>M.02.1.13_A_49

TTAACTGAGGACTTGAGCGATGAAGCCGAGGAAATTTAT

>M.02.1.13_A_50

AGTATTATTGTTACATTACTCTTAAAGTTGATATACGTCTT

>M.02.1.13_A_51

AAATTCTTTGTACATAGTGATGCAGACATTAAGGTAGCAAT

>M.02.1.13_A_52

TTTTTGATTCACTTTTCAAATAGTGATAAGTGAAATCT

>M.02.1.13_A_53

GCGTTGTGGGCGATGGTTTGTATTTTACGAATATAAACC

>M.02.1.13_A_54

AATGCAGCAAAAGAACTCATATAATATACAGTAGAACTAT

>M.02.1.13_A_55

TAAAAATTCCCGCTAACATATATATTAGATGTTTAAAGT

>M.02.1.13_A_56

TCGGATTAGTAACACTAGTGCTAGGATAAACTGAGGCTGGGATAGAA

>M.02.1.13_A_57

TTGATATTTTCGTTAGGCTTTGGAGTCGCAGGTGGAGGG

>M.02.1.13_A_58

CAATATAAAACTCCCTACCCTGATTTGTTCATTATATTTAT

>M.02.1.13_A_59

TATCCTTATCAAGAGGAGGTTATCAACAAGATTCGTAACGCT

>M.02.1.13_A_60

GGTTTTCGTCATCCCGTTAGTCATTGTCGGGAAGCTCGT

>M.02.1.13_A_61

GTACTTGGTCATTGGGTGTTTTTGTTATTGATGTGTTT

>M.02.1.13_A_62

CTTCCTGTTATACTTTCAGTAGCTTCTGCCCCTTGTAATTGC

>M.02.1.13_A_63

GAGGAATAGCCTAATTCAACATGAGGCATAGGAGGCACAA

>M.02.1.13_A_64

TATCTATTCCCCGAACTTTCCGAGATCGAGGAAGCGATTT

>M.02.1.13_A_65

ATCGCATATCGATTTAAATTTCCAGTACCATACGCAACTG

>M.02.1.13_A_66

ACAAAGTCATTAAAAGCCGATTACACCTAAGCAAAAGCG

>M.02.1.13_A_67

TTAGCACGTAAACGTCCTATTGATGTTGTTATCTCTTT

>M.02.1.13_A_68

CCTAGTTTCAATTAACTACTCAGCTATTCACACTCTGACT

>M.02.1.13_A_69

GAATAAGCGGGGCGAAGCCGACGTGTACGCCGTGTCTTTC

>M.02.1.13_A_70

TCTTATATAAAATCAAAATTGTATGAAATCCAAGCTGAAGTTTTT

>M.02.1.13_A_71

ACCTCAGTCTATGAACCGACTAGCATCAACGAAACTAGCG

>M.02.1.13_A_72

TGTGTTTACTGGTACAAGTAAAAGAAGGAAAGTGGTAAA

>M.02.1.13_A_73

TAAAAGTACAAATCTACGTATTAACACCAGTTTATGCAC

>M.02.1.13_A_74

TACATGGGCGGAAACCCATATGGATTCGGCTTAGCCCA

>M.02.1.13_A_75

ACTCAATTATATCAATCACTACAATATGTAATTTATTTT

>M.02.1.13_A_76

GATATCTTAACAATCCGACAGCTTTAGGTGCTCTAAGAT

>M.02.1.13_A_77

TCCTGCGATTAATGCAACAACTACAAATACTATTACTAA

>M.02.1.13_A_78

TTATACTCAAACGGTTGCGTACCCCAAGGACGTAATTCTAC

>M.02.1.13_A_79

ATTCCAAAGTTACGTCATCGATGACCACGTAATGGAAA

>M.02.1.13_A_80

CTGTAAACACTAATTCTTTTTACGTACTACGTTATGT

>M.02.1.13_A_81

CTAACAGCCTTTCTATTAACAGCTGTTTTTGAATGTTTA

>M.02.1.13_A_82

GCATCTGTTATTAGTATTATAGTCTTTATTCTTCTCAAGT

>M.02.1.13_A_83

AGGAAATGTGTACATTACAATAAGGATTGTAGAAGTCA

>M.02.1.13_A_84

TTAAGTGCTAACTCGTTAGTGAGTATTAAAAAGATAT

>M.02.1.13_A_85

ATAAAGAACGATACAAAGAATAATAACAATACAGCCGAAG

>M.02.1.13_A_86

GATACAATTGCGTTAGCTAACGCTTCTAAACCGCTAG

>M.02.1.13_A_87

AATGATAAAGTTATAACAATCCTAGGATATCCATGCTATAGT

>M.02.1.13_A_88

TTTAAATATAGCATTTATTTGGGGGTTTATTGCTTCTTTA

>M.02.1.13_A_89

TACGACAGAAGCAGACATCAACCAACGCCATGATAATCCG

>M.02.1.13_A_90

TTATTGATGGCTACGTTGTGAATGCATTTTATAACGGATCTAA

>M.02.1.13_A_91

TTCTATAATAAATTTTCTGTTGATGTAGGTTGTTGATTTAG

>M.02.1.13_A_92

TCGGCTATGATTACACCGAAATAACCGTCGTCATTAAGT

>M.02.1.13_A_93

TATATAATAAGCATAGCTCTTCGTCACTCAGATATTTATA

>M.02.1.13_A_94

GAAGTCTTTCCCCATCACCATTATGTCACCTATTCCAACT

>M.02.1.13_A_95

TATTAATAAGTTTCCGCACTGGAAAAGTGATATCAAAC

>M.02.1.13_A_96

TTTGGTGACGCGTACAAAGAATGCAAAACTTTAATATTT

>M.02.1.13_A_97

GAACCATAGGCAACCGCTTGTCTATTATTCTTTAGCTTCAT

>M.02.1.13_A_98

CGTTCAAAGCGTTTACATGAATTATTGTATAAATGTTGAA

>M.02.1.13_A_99

CTAACTAACGGCCCACCACTGTCCCCTTCATCAACTGGA

>M.02.1.13_A_100

CTATCAGTTTCATCTATTATAATTGCTGGTAATGCAAAAC

>M.02.1.13_A_101

ATGATAGAGAATTTGCTAACTCATTCACATCTTGTACGC

>M.02.1.13_A_102

AGCTTAATCTGTCTCTGACATTGTACACATTTCGCCCC

>M.02.1.13_A_103

TAAAAGTGTTGATTAAGCCTTATGTATGTATTATTGCAA

>M.02.1.13_A_104

GAATTAATTATGAGTTCTACCCTATCAAGCCAAAGTAAAT

>M.02.1.13_A_105

AACAAATAGCGAAAATTTGTTATAGAAAGGTGGAAGGA

>M.02.1.13_A_106

ACCCTAAGATAAAAAAGTAGAAGTGAAAAAGCTAGGAGA

>M.02.1.13_A_107

TTAAGTGATGCGGTAAATAAAGCATTAGATAATATAAGGG

>M.02.1.13_A_108

TACAACAAGGCAATTATCAGACCGCATTACAATATCTAA

>M.02.1.13_A_109

CTATTAATAGCTCTTGTTTAGTGACAGGTATTGTTACTTCGG

>M.02.1.13_A_110

TGATTTCCGCTTTTGCTTAGGTGTAATCGGCTTTTAAT

>M.02.1.13_A_111

CCCTCGCCTCCTTCTTTTTCCTTTTTTAAGTTGT

>M.02.1.13_A_112

GTTTATTTTATTAATTCTTCTCTTCCCTTTAACCTATA

>M.02.1.13_A_113

TTTGTGGCACCCAAGATTATAGTCGCCGAGGTGGTATT

>M.02.1.13_A_114

ATTGTACTTATTGCTGATAATGTATTAGTAAAGATTGAC

>M.02.1.13_A_115

TTTCAGCGTTTAAAAAGCCCATTCAGAAAGGAATTGAA

>M.02.1.13_A_116

AAAGCAGAAAACGGTAGAGGTTGTAAAGAGGCTAAAGTCA

>M.02.1.13_A_117

CCGAGACGCCCTGGCGAGGGTGATACACCTCTGCCCCACCCTTCG

>M.02.1.13_A_118

TCAGCCATGCCAACAAGAGATGTCAGTCCACCACTTTG

>M.02.1.13_A_119

GTGGCTGCAGCACCTGCTGCAGGTGCCGTTCCAGTGCCG

>M.02.1.13_A_120

TTCCTTAAGTGACTCTGGAAATGCGGTAAGTTCCTGT

>M.02.1.13_A_121

AATTCTCTTAATTCTACTATGACCTCATATTTTTTCTTCATCGT

>M.02.1.13_A_122

ATTCCCTTTGCTCTTAATCTTCCAACAGATGCCGTTATTTC

>M.02.1.13_A_123

ACTAAGGTCCTGTCTGCTGCTACTGCCTACCGGTCGCT

>M.02.1.13_A_124

AGGTGTTTAAGGAACTGCTCGAATTCATTGGAGAAAGG

>M.02.1.13_A_125

GTTCTGAGACTTATTAGTATTGTATCGCTTCTTGCACCATA

>M.02.1.13_A_126

TACTTCATCACGTCCACGTTTGTTATTTTTGTTGATTTAA

>M.02.1.13_A_127

ACTAGAATTCCCATTATAAAAGCAATCAAAAAGAATGAAA

>M.02.1.13_A_128

AATTTTGTTAATAATTCTATAGTAAATGTACCAACACCAC

>M.02.1.13_A_129

CCTAAATCATTATCAAATTACGTAATTCCACAAAATCA

>M.02.1.13_A_130

TCACTAGAAAACTTCCTACTCTCATCTTTTCATTGTTTT

>M.02.1.13_A_131

TCTTTGCTTTGCTCTTCAGTCCATGAGCTTCCAGTTAT

>M.02.1.13_A_132

TTCTATCTTCTTTTTGTACTTTCGCCGGGGTTGTTGT

>M.02.1.13_A_133

GTCTCCATTAAATATCTTAAAATGAATGGCGGTAATGGTC

>M.02.1.13_A_134

TTTCCGCAACGCAAAGCTTCATTCCTAGTAATTGTGAG

>M.02.1.13_A_135

GTTAAAATATCAGTCCAAATTGAGGGCATCTATTCTTATAT

>M.02.1.13_A_136

AGCGGAGTACAAAATGCTATATTGATAGCTAGCAAAAACT

>M.02.1.13_A_137

AAAGTACGTTAGTGAGAGAGAAATATTTATAAGGAACAAA

>M.02.1.13_A_138

AAAATTTCTCCTATTGCCATATATTACTTTCACTCATCTCTC

>M.02.1.13_A_139

CAAGATATTGACTTAGAGTATGCTAAAGCCATTGAGATATGT

>M.02.1.13_A_140

ATGCAGTAATTATGAGGTTAGTTTCGAAAGGTGTTTTTA

>M.02.1.13_A_141

GCAGGTTGGTACTATGAATTCACTGGTTCTAACGTT

>M.02.1.13_A_142

TACGACTGAAGCAGACATCAACCACCTCCAGCTAAGCCCG

>M.02.1.13_A_143

AAACTGCGGTGAACAATTGGGTTCAGAGAACACAAAGTG

>M.02.1.13_A_144

CCGCATAAGCAAATCTTGCTACTACCATCCCGCCTGCCCCA

>M.02.1.13_A_145

AAATAAGTGGTAAAAGATGGCGGCAAATCAAGAAGTATG

>M.02.1.13_A_146

CTTGCCTTAAATTGTCCTTTCTGTTTGTGCAATGCAGT

>M.02.1.13_A_147

CCAACACCAATAATAATTATATTTACTTTTTCCCATTGT

>M.02.1.13_A_148

CTTGAGACCATTGTAAGGGTCACGAACGCAAAGAGCGGT

>M.02.1.13_A_149

ATTCCCATCGCCCTCACAGATCGAATAAGTACATTAGTTTT

>M.02.1.13_A_150

TATTTCTGGCTCATCCATTAATATAAAATCGTAATTCTCT

>M.02.1.13_A_151

TGTACTGTATGCAGCTTACCTGTTCCGACATGGGATAAA

>M.02.1.13_A_152

TAATCTCTGCAGGCAATTACCAAAAAGTGGTAATAGACG

>M.02.1.13_A_153

GTAGAAATAATACTAACGAAACCTCTATTTCAACTGGAA

>M.02.1.13_A_154

ATTAGTTCATCAGTATCTTTATATCCTTTTGATACCAAT

>M.02.1.13_A_155

GTATCGAAAAGAATAGATGCCCATTCTGCAATGTAACGT

>M.02.1.13_A_156

AAGTAGGTGTACTTATCCCTAAGTTAAGATTTATATTAGC

>M.02.1.13_A_157

TCTAACTCATTTACATTTATGGGCATTTTTTCACCCTTATT

>M.02.1.13_A_158

ATTAATTGCGATCTCGTTATATAAACTATTACTAAATCGCCCT

>M.02.1.6_A_1

AGATAAAGGAGAATCTTCTAATCACGATAAATGATATT

>M.02.1.6_A_2

CCTAACTTAATATGGTTAGGAGGGATTATATTGCATG

>M.02.1.6_A_3

GTAACAAATAAGTTATCCCATTCAGAATAAACTGCACTCCA

>M.02.1.6_A_4

AAACGGATCACCTTCCCACGTCTCATCTGTTTTCTCTGTA

>M.02.1.6_A_5

GATAGCTAATTCCACCGCAGTGGTGGTTAATCATGTCAC

>M.02.1.6_A_6

AATAGAAACACTGCTTAAGCTGTTAGATGAACTAGACA

>M.02.1.6_A_7

AAAATTGATTTTGATTGTTCGGCAACTTGTGCAGGTTCCT

>M.02.1.6_A_8

CTGAATATGCCGGTGATACCGCCTAACACATTACCTAATC

>M.02.1.6_A_9

TTTCTGACTGTTTCAAAAACTCATAAACTTGTTTTATCAATA

>M.02.1.6_A_10

GAGGTTTCCTCAGGGTGTAGCTGCAGCACAACCTAAGA

>M.02.1.6_A_11

GAAGCTCTGTCTCCTCTACTTGCGCTTCTTCCTCTGTCTCT

>M.02.1.6_A_12

AATAAGATATACAGTGTTGGGTAGAAGCCAAAGAAGCTGGT

>M.02.1.6_A_13

TCATGAAATAGTCCTTCGTGTTCCATTCACTAACGTTTATCGT

>M.02.1.6_A_14

CCGTGTCGGCGACGGCGGGGTAGAAAAATATAAGTCTCC

>M.02.1.6_A_15

ATTGAAACTTTACCTTCGTATACTGTACCTAAACTAAA

>M.02.1.6_A_16

ACATCCTTTGATACAGCTAGTGGTGCCACTCTTCCCGGT

>M.02.1.6_A_17

GGGCGGGTACCCGCGCCCGGGGGGCGAGCACACCCGGGTACAA

>M.02.1.6_A_18

GGTTAGATATCACTAAGTTATCATCAGTTGAAATTAACAT

>M.02.1.6_A_19

TCTGGGTAAACCTTTATTTCTCCCCGCCACTACTTTTTTA

>M.02.1.6_A_20

ATCTATCATCTGGATAGAATACTGCAGAGAACGTATCACT

>M.02.1.6_A_21

GTTTCTTTCTGGGCCTTTTAGGTCTTTTTTCAAAACTCGT

>M.02.1.6_A_22

GCTCTCGTCGTATTTCTTGTAATATTCTATGATTTTTCTG

>M.02.1.6_A_23

GTTTCCTCATCTTTCGTCTCAGCAATGACTCTCACGAA

>M.02.1.6_A_24

TTTACCCTTACCTCGGTGGCACTACCGAAAGTCTCTACCCCA

>M.02.1.6_A_25

ACATACCCTTCTGCATAAGAGGGCTGTTCCGAATACACTTCT

>M.02.1.6_A_26

TACAAGAAGTTCGAGGAGGTGTACAAACGATCGTTCCCGAG

>M.02.1.6_A_27

TAATAAGTCTGTATCACTTCTCCAACCAAATCCTTTATA

>M.02.1.6_A_28

CATTTAGTTGATATGCGTAACTTGTTGTCCCACTAAT

>M.02.1.6_A_29

ACTGGGACTTTATACATACCTGTACAGAGTTTCAATCCT

>M.02.1.6_A_30

CTGATAGCTCATATAATAAAACATTCTGATGATAGAATCC

>M.02.1.6_A_31

ACTATCAATCGTCAGCTTCTGTTCTTTCTGAATTTCTTC

>M.02.1.6_A_32

CTTCATAAATTATAATTCCGCTAGCATATGTTAGATTTAGCG

>M.02.1.6_A_33

TAGAAGACCTGTATTTTTACGGGTTCGTAAGGAGATAT

>M.02.1.6_A_34

TTCTTCTTCATTATCTTTTTCTTTATTATACAATCCCTTA

>M.02.1.6_A_35

CACATTTTCTGAAAAGTTTTATCACATAAAGGACATTTA

>M.02.1.6_A_36

CGATTGCAACGTATTTGTATACAGATGCCTGAAGATGAT

>M.02.1.6_A_37

AATTCAAAGATGCCCTCGATTATCTTCTCGGGAATTTTA

>M.02.1.6_A_38

TTTGGATTAAGCTGGAGGTGGTTGATGTCTGCTTCTGT

>M.02.1.6_A_39

AGTCTTACTAATAAGGAGAGAGATAAAGGAGAACGGAG

>M.02.1.6_A_40

GATCTTTGGATGCCAAGTAATAAAAGGGCTGGTATCGA

>M.02.1.6_A_41

AAATAATAATTCTATCATAAGTTTGAAAATTTTCATCTT

>M.02.1.6_A_42

TACGAAGAAAACAGCCTTCTTGTTTAACATTTTGGCAAG

>M.02.1.6_A_43

TGCTAGTTGGATATCGAAATGGCAAAATGTACAAGCTAGGA

>M.02.1.6_A_44

GGTACTCCTAACTCTAGCATTTTTGTGGCCGTGAATTT

>M.02.1.6_A_45

TCATTTATAACATCTGGTATGTCTTCAGTTTCTGTGTATCT

>M.02.1.6_A_46

AATGGCGAAACTGACAATGAAGCAGAATGCTGTACTGTAT

>M.02.1.6_A_47

AGGCTGGCCAGAGATCTTTATGACTCGTTCAAGCTCCCT

>M.02.1.6_A_48

ACGTTACATACCATTTCTTAGGAAGCATATTCTCTCTAA

>M.02.1.6_A_49

AGAACATCTCGAAAATATAACAGATCTCAACATAACTGA

>M.02.1.6_A_50

CCTGCTCCTCCACTACCTGCTGCATTTGAAGTAGCGTTTG

>M.02.1.6_A_51

GCGCGTAAAAAGTACTCCAGGCTTCAGTTGGTTATCGAA

>M.02.1.6_A_52

ACCTTCGTTCAGGTTCCAGCATCAGTAGTAGGTTATTC

>M.02.1.6_A_53

GTCCCTACAATTTACCGGGTACAGGTCTACCATCAT

>M.02.1.6_A_54

ATCTACTCCGCTCAGGGTACTGTTGAAGATCTACGCCCT

>M.02.1.6_A_55

TATTATAACTTATTACCATCTGAGAAGCACTAAATATTA

>M.02.1.6_A_56

GTAACGCTGACGTTAGTTATTACATATCCGCCCTGTATCGT

>M.02.1.6_A_57

AAAAGGTGGTATGTAACGTGGGATTGAAAATGTACAAGTGT

>M.02.1.6_A_58

AATGATGCTACCGCATTTGTAAGAAGATTTCCTAGATTT

>M.02.1.6_A_59

TCTCAGCCATTCCCATATAACTTAGTCTCAGAAATCAGACTAG

>M.02.1.6_A_60

GGTACTACGGTCGAAACAGTCCTCCAAGCTAACATCGA

>M.02.1.6_A_61

GTATTGAACGCGGATATTAAAGCAGCATCTTCATATTCCG

>M.02.1.6_A_62

TCTCTTCTTAAAATTTGAAATTCTCTCTGTGCAACAAA

>M.02.1.6_A_63

AGCCAAATCAGTGTCGAGAACTTTCAGCCTAACGAAAAC

>M.02.1.6_A_64

TTAAGCCCCTGTTTCTTTAATAGTTCATAAACTCTCAAA

>M.02.1.6_A_65

TTTGCTGTATTCTGTATAACAATCCCGTTTCCGCCGTTTAT

>M.02.1.6_A_66

TTAATTTCATCCTCTTTAGCTCTAATAGCCCATAAAATA

>M.02.1.6_A_67

CTGACATTGTTAGGGTCATACGTGGCTATTATTTCCGCT

>M.02.1.6_A_68

CGTAAAGATTACCTTACCCTCCTTCTTTACCTGTACTCTCA

>M.02.1.6_A_69

ATCCACTTGGCTGTATGCACGACTCTCGACGCTGTGA

>M.02.1.6_A_70

CTTGACAGAGGCACTGATTGCCCTACTTATACGGTTCCT

>M.02.1.6_A_71

CCAGAGACTGAGACAGAGCCTGAAGTAGTCCAGATATTT

>M.02.1.6_A_72

GAGTTTGTTTAGCTGGGGCTACGTAGTAGCCCTGTT

>M.02.1.6_A_73

CGTTATAAAGGTCTGTATAATATTTCACGTTTATACTACT

>M.02.1.6_A_74

TCTTCAATTTATGTACCTTTGAAATACATTCCCACAAATT

>M.02.1.6_A_75

TTTCGTTTAGCTTTCCCAAAAGTTGCTTTACGTAATCT

>M.02.1.6_A_76

TTGAAAATTTTGAAAAAAGACTTGAGGGGGCCTGAGAGG

>M.02.1.6_A_77

CAACCGAACCAAAAGAAGCAAAGGAAGTGGATAACGTACAG

>M.02.1.6_A_78

TAAGGAGGGCTTTCGCTAGTAGTTTGAACGATTCGGCCAT

>M.02.1.6_A_79

CCCGTTACCGCCTCGTATGATAGATCTAACCAATATG

>M.02.1.6_A_80

ATAATACTCAAAGTGTTTATATAATACGTGAGACGCAATACCG

>M.02.1.6_A_81

ATCAGACGGCAGTATCGATAGGGTTGCATGTGATAGATAT

>M.02.1.6_A_82

TTCAATGTCGTAACAGGTACTGTATCATCTTCGCTAATCCC

>M.02.1.6_A_83

TAAGCTCTAAGACGATGAAAACCACCAATTATTCCAATTACAT

>M.02.1.6_A_84

AGATTTTGTAGTGCTTCTTGCAACAGTAAAGCTCTTGTGC

>M.02.1.6_A_85

ATACTAATTATGTTGGCCCATTAACGCCTATCGTATATA

>M.02.1.6_A_86

GTTCTATTGGTTGTTTGTTAGTGCACACACAAGGGACAG

>M.02.1.6_A_87

ATGTCAAAACAAAGTAATAATACTTGGGTAGCGACCAT

>M.02.1.6_A_88

GCACAATACACGTTCACTATTAATAGCAACTATAAAA

>M.02.1.6_A_89

TGCTATATAACTCTGTACAATCGCAGCTACTTGTGCGGGTA

>M.02.1.6_A_90

AGAAAGATATTTTCATCTACACTGTCATATCAACGATAT

>M.02.1.6_A_91

CTAAGGATGACATCCTACCTCCTAACGTGATAAGGATTA

>M.02.1.6_A_92

AGTAATATAGTACGTGAGGAAAATTAAAGGTGTTGAGAT

>M.02.1.6_A_93

TTCTCAAATATCCTTTCCATGCTGAACTCCAAAATCG

>M.02.1.6_A_94

CCCCGTGAAGAGTGGTTGCCCCGAGTTCATGAATCCCT

>M.02.1.6_A_95

CTAAGCTTTATCGTTGCGACGTCGGGGACGACGAGTTCTT

>M.02.1.6_A_96

TGCTAATTTCTGGTAGGTTTGAAGCGAAGTGCGGAAAG

>M.02.1.6_A_97

GAACCCGCATTGGTGTGTTACTGGGACGACAGGTGCCGG

>M.02.1.6_A_98

TAAGACCATCGTCGCTTACAGCGTTCGGGACGAGCTTCAC

>M.02.1.6_A_99

ATCATTATTATAGCCCCGCTATCGAAAGTAGCATTAGGT

>M.02.1.6_A_100

GTAGATATTCCCGGGCCATACAGTCGCTACGCCAACGATCG

>M.02.1.6_A_101

AAACGAAATATCGAAGGCGTCAGATGAGGAAAGGAAGAA

>M.02.1.6_A_102

ATATACGGAGCAATTGCAGGTCTATCACACCTCCTACTG

>M.02.1.6_A_103

ATGCTTACGTCTAAGGCCTGTCCATAGCTTAACGTGG

>M.02.1.6_A_104

TATACGCATCTCTAACTCCAGTTATTTTTACAATTTTAT

>M.02.1.6_A_105

AAGGTGAATAAAGAGGGCTTAGATCTTTGGATGCCAAGTAA

>M.02.1.6_A_106

TTCGAAACTAACCTCATAATTACGACATACGCGTATTTTTGA

>M.02.1.6_A_107

ATACGGTTATAGACGAACGCTTGCCACTGGACGCTACCAGT

>M.02.1.6_A_108

GTAACTACTTCACAGTCACCCCTTCCTATGTACGCACCGT

>M.02.1.6_A_109

AAAGTACGCCGACCCGAACCTCAGGCTGGACGTAGAG

>M.02.1.6_A_110

ATGATTTCCATATGTTTATAATTGTGAATTTTTTCACG

>M.02.1.6_A_111

TTTTATTTTGATAGTTTGAATAAAATATTAGCAAATGA

>M.02.1.6_A_112

GTGCATCACCCCAAAGCCACGTGTGGTAGTCGAGGAACG

>M.02.1.6_A_113

ACAGAAGTTCTACCTCAGTGATATGTCTCTTTGGCCTTTCCCC

>M.02.1.6_A_114

GTTATACCTCAGCACGCTGGAAGCTTGCAACTCCAT

>M.02.1.6_A_115

CAACACACTACAGAGGCAGAAACAATATTTCAACCAAT

>M.02.1.6_A_116

GTGTGGAATTTTTGCCGTCTTCTTTCAACTCTACTTTCGC

>M.02.1.6_A_117

CAATGTCAACGGAGATATACTGAAGGAAGCTAGTCATGA

>M.02.1.6_A_118

AGAGTTAGTCAGCGAGGAGGCGATCAAAGCGGAACTCAAG

>M.02.1.6_A_119

CTTAACTGTGCCACCCCTACGATTATTCGCTAGATATTG

>M.02.1.6_A_120

AATACAATAATCCTCCCTCGCCTTTTCATCCTTTTTCCA

>M.02.1.6_A_121

AATTCGTTAGAAAATGGCTTTTCACAAGCACTTGAAGCGTT

>M.02.1.6_A_122

GAAGGCATTAAGCGGGGTAACGTCCTCGCAGGGAGGG

>M.02.2.19_A_1

ATACATTTCCAGAAAACGTTAGCTGTCATTGTGGTATA

>M.02.2.19_A_2

CGTATAAGGTGCAATAGAACTGGAATTGTGAAACTTCTT

>M.02.2.19_A_3

TTTCTAACCTCATAGATTCTCATAGGCAATATAGCTACTT

>M.02.2.19_A_4

TAACCTATAAACCCGGGCAAAAACCCCCACCAAAAAATTTT

>M.02.2.19_A_5

ACCGCTAGCTCCGGAAGGAACTATTATGTTTATCGTTTTGC

>M.02.2.19_A_6

TGTTCTTTCTTTTCTTTGTTCAGATTTATATATTTGTTGT

>M.02.2.19_A_7

ACTATATAAATCCGAAAGCACGTTAGTGAGAGAGAAAGATT

>M.02.2.19_A_8

TACGAAAGTAAGACGGAAAAGACATGGGAAGGAATAGAT

>M.02.2.19_A_9

TACCACCGTTGCATTTGTTATCTCTCGGTAGTATACTTT

>M.02.2.19_A_10

AGCAAACTATATTTGTTTTGTAAAGATTTAATATTAAATTCCT

>M.02.2.19_A_11

GTCACGCCATTTCGTTATAATGTAAGTCCTTTCACCCTT

>M.02.2.19_A_12

AAGCCCTATTTAGGGGGTAAACCCCCTAAAACCCCACGT

>M.02.2.19_A_13

ACACTTTAAGAATTATGTAGTAGTTTATCCGCATGTACTT

>M.02.2.19_A_14

CCGAAAACCGTGATGTATACCTCATCAAAGAACTCTCCGT

>M.02.2.19_A_15

CTATGGTCGAGTGGCGTAAAAAGTTCCGTTTCCTCGTCA

>M.02.2.19_A_16

CCGTTTACGACAATAAAGACCCTCCTGAACCCGTCGCT

>M.02.2.19_A_17

CAATTTCCGCAAATTTGTTTTCGTAACGTGCGATACGTTT

>M.02.2.19_A_18

AAACTATTTAATAGTTTTATCCACCTTACTTTTTCTTCC

>M.02.2.19_A_19

CCCGTCGATGAAGGCGATTCAGGAGGGCCGTTAATTAG

>M.02.2.19_A_20

GTCAAAATTATACCATTCCGTGATATTTCTATTCCATTG

>M.02.2.19_A_21

ACAACAGAACCAGCTGGAGCACTTGCCGCAATATTAAAG

>M.02.2.19_A_22

TTTAGTTTTCCGCCTTGATTCTGTGCTTGCTGTTCTGCTT

>M.02.2.19_A_23

TTAAAAGCAAAAAAGGATCCTATAACTATCAATGTTATT

>M.02.2.19_A_24

ACCAATTGCGCTCTGATTTTTCTAATGAAGTTATTTCTA

>M.02.2.19_A_25

TCAAAAAACAAATTTTTAGTAAGAGAAAAAATAGTTAAA

>M.02.2.19_A_26

TTGAAAATGATGATGAGCCTCATCTATCACAAATAAAGTA

>M.02.2.19_A_27

TTTAGTTATAGCCTATGCGTTAAACAGGACCAGATAACCT

>M.02.2.19_A_28

TTTCTTTTTGCCATTTGGGGATTTGCCTTAAATCCAAATT

>M.02.2.19_A_29

ATCGTCTGTCTCACCTTGATTATTGCCTTCTCCTTCATAA

>M.02.2.19_A_30

AAACTATCGTAAAAACGAAGCCTATCATATAAAGCGGT

>M.02.2.19_A_31

ATTGTAGTCAGAAAGTCTATTATATCTTGAGTAGTAATT

>M.02.2.19_A_32

AATAAATATCCTAATCCTAATCCACCGCCCGCTATTCCT

>M.02.2.19_A_33

CATATCCTCTCTTCCCTCACCCAATTGAAGAAGAACCCTA

>M.02.2.19_A_34

AAATAATATTTTCAGCATCATACTCTCCATCATCAACAACA

>M.02.2.19_A_35

AAAAGAGGACAGAACTAACGGTATTAAATAATGTAA

>M.02.2.19_A_36

TCATCTCACCCTAGCGTTATTATATCATTATATAAGGAT

>M.02.2.19_A_37

AGTCTTTATAGCTATCCTTCTAGTTTCACTCAAGTAAGTA

>M.02.2.19_A_38

TGACGAATTTGTATAGAGTTAGACCTGCCCACACACCTGC

>M.02.2.19_A_39

GCATTATTCCAGGCTTAGGGACAATATTTGGGGCTGGGAT

>M.02.2.19_A_40

TACCTCAACCATCTGAGAAAGCACCTAAAAAGCGAC

>M.02.2.19_A_41

AGCCTTTATCTCGCCCGCCTTTTTTGCCTGGAGCATATTCT

>M.02.2.19_A_42

GTTCTAATAGCTTCTGTTTCAGAGATGGGTTAGCTAA

>M.02.2.19_A_43

AGTGAAATATTTGAATTATATCCAGTTATTTCATACGCCTG

>M.02.2.19_A_44

ACTGCACAACTTATATATGATGAATTTCCGGTACCA

>M.02.2.19_A_45

GTTTTATTAGATAAAAATGAAGTTGAAGAAAAAATTATTA

>M.02.2.19_A_46

CTCTGCGGATTAACACCGGCACTTATGTTCACCCCTTGG

>M.02.2.19_A_47

TTTCATATGGCACGTCCTTCGGGTCTATGTAGACGAGGTA

>M.02.2.19_A_48

CATATGATCATAAGCAAGAACATGTAGTTGCGTATAAATCT

>M.02.2.19_A_49

AATGTACTCCCTGCGTAAATTCCGCCAACAATAGATATT

>M.02.2.19_A_50

ATCTTTATCAACCTCTGTCCGACATTTGAACCATAATCTT

>M.02.2.19_A_51

GATTGATCTATTGCTGGTACTGGATAAAACTCATCAA

>M.02.2.19_A_52

ATGCAAGTTTACTAAATTCAGATTTTCCATTTGCATCGG

>M.02.2.19_A_53

ACAACGGATGGTCTCTTCGGTTCCGTCGCCTCTTTTAGAACTT

>M.02.2.19_A_54

GCATTCATTTGTCCTAATGTAGTCTGAGTTTGTTGATTAT

>M.02.2.19_A_55

TCATTATACTTAATTATGGATATTCAAACAGTTGAGG

>M.02.2.19_A_56

GAGAAAAAAGCCTCACCTCGGGGATTTTCGTGTTAAATGAA

>M.02.2.19_A_57

ATTCCAAGCACATTTAGGACAATAACCAATGAAACCTAT

>M.02.2.19_A_58

TACGTATATAAATCCTTTTTTGTTAACTCTCTTATGAGTA

>M.02.2.19_A_59

TGTCATTACTGTCTCTTATGCAGCTAAGGAAGAATAT

>M.02.2.19_A_60

TTATAACTATGTTTTTCGGGTGTAATTATGTCTAATATCTTA

>M.02.2.19_A_61

GTTTCATCGAGACCACTTAGGAAGAAGTCCTCTAGTTC

>M.02.2.19_A_62

ATAATCGTTCAGGGTGGCAACCCACAGTTCGTGATACAA

>M.02.2.19_A_63

ATGGAAGCTAGATTTTATATAGTAGATGACATTCTTAT

>M.02.2.19_A_64

CACGATAAACATCTTTCTCGACAATCTAGCGTTTGCTCTAT

>M.02.2.19_A_65

TCAACCTGTCACGGGTGGGTCACCTCACCAGCTGGTAT

>M.02.2.19_A_66

GGAAGGAATTGTAACTGTTCTTCATTGAACTTTATTACT

>M.02.2.19_A_67

TCTACGGTTTGTTCGCTCCAACGCTCGTGCAGAAGGCG

>M.02.2.19_A_68

AAGTTTGACTAGGAGTTACTGTTACGTTGATTGACGGT

>M.02.2.19_A_69

TGATGAGATTTCTTCATTAGAAGAATATCTATTACAGCCT

>M.02.2.19_A_70

GAAGATGATTTAGCTGATGAAATATGCGTTAAGTTGAAA

>M.02.2.19_A_71

ATTACTATAACTACGACATTACCATATCCAGGATTTCCCTG

>M.02.2.19_A_72

GTACTATAGTGCCGTTAATTCCATATGTGATCCAAGCACC

>M.02.2.19_A_73

TATCCCCCTAGAAGAATGTTAATATTAGCTAAATCTGATG

>M.02.2.19_A_74

GTGATAATAATCCAGTTGAAGTTGAAGTAGTCATAGATGT

>M.02.2.19_A_75

CAATATATATAAAATTGGCACAAAATCTAAATGCGAATA

>M.02.2.19_A_76

TTGTAGCTGGGAATCTTGCTAAGTCGAATTCCCACATCACG

>M.02.2.19_A_77

TATTATTCAAGTGTCGGGCTATATAACATTAGCGAGTGGA

>M.02.2.19_A_78

TATACTTTTCCTTATGCTCTTCTGAATCTTTCTTTTCT

>M.02.2.19_A_79

CCATTGGTAAAATGATATCATCACTAGAATGTTTATTATT

>M.02.2.19_A_80

TCACTTTTACTTAGTCAAATAGAACTCGCTTCTATGATTG

>M.02.2.19_A_81

TCAAAAATTTGTTCTTGACAGGTCAGAAAAGGACTGGTAAG

>M.02.2.19_A_82

TCTTCAGAGTGTTGGGGGCCGATAATATGGGGTTATTTA

>M.02.2.19_A_83

CTTGACATTATTCTTATACAATCTAAACAATAACTATGTG

>M.02.2.19_A_84

TACTTTATGCTAATGTCCCTGAGTTTCCTAATATCGATCG

>M.02.2.19_A_85

GTTAGTGTACCTCGATATAACTCAAGAAATTGAGTAGTT

>M.02.2.19_A_86

TATACCGCCGTGAAGTACAACGGCTACGATGAAGGTAC

>M.02.2.19_A_87

AAGCCCTGGCAAACGTTTACGCTTGAAGTGGGAGTTTCC

>M.02.2.19_A_88

AATTCATGTAGAACTACCGCATATGATAACAACACCG

>M.02.2.19_A_89

CCGTTTATAAAGCCTCTTTCTTTTTTCCCACTTAATGAA

>M.02.2.19_A_90

ATTAAAATACATATTATAACCCCAACACTTGCAAATTTTGT

>M.02.2.19_A_91

TAAATAATTATATCTTGTTGTTAATAATAAATTTCATGA

>M.02.2.19_A_92

TCAACTACTACAACTCCTGCATATGTATTTCCTACAACTCTT

>M.02.2.19_A_93

ACTATGCCGGTTCGCAAGGGGCTACTATAGCGTCGTTAG

>M.02.2.19_A_94

CTACTTGTTTGTTTAATTCCTATCATATATATATATAA

>M.02.2.19_A_95

ACGAGAACGGCAAAGATCAAGCCCATCGTAAATAATTTC

>M.02.2.19_A_96

AATTTTATATATAGATGAGTAGTAGTCAGTGGAACTATA

>M.02.2.19_A_97

ACATCTTTGTGGCCGTGAACTTCCTCAGATATTTCGGGGC

>M.02.2.19_A_98

AAACGAAATATCGAAGGCGTCAGATGAGGAAAGGAAGAA

>M.02.2.19_A_99

TGTACTCTGGATAGGGGAACCCGACAATGAAGATAGCCTT

>M.02.2.19_A_100

TATTAACACATATTCGTAAAATTTTTGTTGAGATAGTGTT

>M.02.2.19_A_101

GAAGTTTCATTTATGATGTACCCGTAAGTCTGGAAGCCGT

>M.02.2.19_A_102

CAAATATCGTAGTTACTATCCAGATTAACCAGTCAACAA

>M.02.2.19_A_103

CCATTATGGGACGGGGATAATTCTAGCTAGTCAGAGAATT

>M.02.2.19_A_104

CAAAGTACTTATCAGCCGTAAAACATAAACTTAAGCTGAAA

>M.02.2.19_A_105

AGGCTTTATAAACGGCTTTCAATTCTATAGTAGATTAT

>M.02.2.19_A_106

GATAAAATGTCTTGAGCTACTATTTTTAGATCTTTGAAT

>M.02.2.19_A_107

AAAATGACTGCAGTTATGAACAAATTAATACCCGCCATAG

>M.02.2.19_A_108

TCAGGTGGCTTTAGCCTCTTTACAACCTCTACCGTTTTCT

>M.02.2.19_A_109

TGCGAATCGTGGGCTAAAATATGAACTGGATCTTCATCG

>M.02.2.19_A_110

AAATAAGGAAAAACTGCCGAAAGAACTTCAACCAAAAAT

>M.02.2.19_A_111

TGAACTTTTCGTAGACTGTCTTTGGTACAATGTATGTGTC

>M.02.2.19_A_112

TTGTAACGTACCTGTAACCTATTTAGTATACTTCTATAA

>M.02.2.19_A_113

TGCGATTTCGCCGTTTGGCTTTAATAGCTCGGGGTGATTCT

>M.02.2.19_A_114

GTATTATTCGTATCGTTGCACTCACTGAGGTATTAAAT

>M.02.2.19_A_115

GTTTTAATGTCGATGACAGTTTTAGTTCTTAACTCAAGT

>M.02.2.19_A_116

ATGTTTAAGACAATAGCAAGAGAGATATTTAACGATGA

>M.02.2.19_A_117

AGGTATAGTGAAAACGGCTACATCATATCTTTTTATT

>M.02.3.11_A_1

CCCGTTACCGCCTCGTATGCAATATCCACCCAATACGT

>M.02.3.11_A_2

TGAACAACTCTAGTAAACGCAGGGACATTTATAGCTTATTCAA

>M.02.3.11_A_3

TGCTGCAGGTTCTGCAGCCACTGGAACTCAAGTAAGTGGTAT

>M.02.3.11_A_4

TCTACCGAGTGGGGCAGCTGCTGTACCCTCAAGTTCAT

>M.02.3.11_A_5

TCTTTATGATTTTAATACAGCTTTGGCATTAACTGTCTCTGG

>M.02.3.11_A_6

TCACAATGTCTTTATAAGCGTCCTCAGGCTTTATTAAATCT

>M.02.3.11_A_7

TAAGGTAATCCGTCTCCAGTGGGGTCTCCCACTGGATAATAA

>M.02.3.11_A_8

TAGAATTATGCAATTCTTCTAAGTCAGTGGAAACTCTA

>M.02.3.11_A_9

TGTACTGTTTATCACCAGCTCACCGTTTTGCCAATATGGC

>M.02.3.11_A_10

TATGCAATACCAACCTTACGGAGAACAGAATATCCAT

>M.02.3.11_A_11

TTTATTGATATATTTACTTATTCGGCTAGTGCTGCAGGGG

>M.02.3.11_A_12

TTATGATATATGTGTACCTAAAGACCAAAGGAAAGAATTCT

>M.02.3.11_A_13

GAGAATTTTTATCATAGTTAGAACCATTATTTGAATTTTCC

>M.02.3.11_A_14

TTCTCTTATGCGGTCTAATAGGATACTTGTTATGGCTTTG

>M.02.3.11_A_15

TAAACTAATATTTGTAAAGGACTTCCAAAAGACGTAGATTGT

>M.02.3.11_A_16

CAAATACTATTAGCACCTTTTACGCTGATTTTTCTCATA

>M.02.3.11_A_17

TATGTAAAACACTACAAAAAAGTTTAAATATCAGTTTAAT

>M.02.3.11_A_18

AGGAATAGGAGGACAAGAGACAGTATAAGTCCTATGCCGTCA

>M.02.3.11_A_19

TATTCACAATTCCTGAAGCTTGGGTTCCAGTGGCTGCA

>M.02.3.11_A_20

TACAAACGCAGTTACAGCTATTGTAGTAGAGAATAAAC

>M.02.3.11_A_21

TACGACTATTACTATTACATTATCAAAAGCACCACTCGGTGGC

>M.02.3.11_A_22

TTCAATTACTTCCTTACTTCCCTCATCTTGCTTAATTTTT

>M.02.3.11_A_23

TATACTCTGTTCCTTGCTCCATCTTTAGTAATTTGCTAAG

>M.02.3.11_A_24

TTGTTCAACCTCCTCCTTGTGTAGTAGTTTGCTTAATTCC

>M.02.3.11_A_25

TAATCCTAATGCCGTTGGTACGATAAAACGAGCAAAAGAG

>M.02.3.11_A_26

TTATTATATAGATAAATCGCTAAGTGTGCGTAAGCCCATACT

>M.02.3.11_A_27

AGTTGATAACTTTCGATACTTATCTCTTCACTCATTTTCAAC

>M.02.3.11_A_28

ATACTAAGAAGTAACGTAACAGCTTCTGAGAGAACAAA

>M.02.3.11_A_29

TCAGTATCGACATAAACGATATATAACCCTATATATGAT

>M.02.3.11_A_30

TCTCATCATCTATTTTCTTTTTTATTGTCGTCTTTCCCGT

>M.02.3.11_A_31

TTAGATCGATCTGGATTTACGCCTAACTTATTCAGTGAGTT

>M.02.3.11_A_32

TTAATCTTTTCGCTTAAGAATAATGCTCTTTCTACTTTCTC

>M.02.3.11_A_33

ATAAAGAACGATTCTTGTGTAAACGGTTGTATTTGTGCT

>M.02.3.11_A_34

TTATTTCTAGAAGATTTAGAATTTTTACACATGTTAAT

>M.02.3.11_A_35

TTCCAAACATCAAAACCCCTAACTCCTTCTCAGCTTAAGT

>M.02.3.11_A_36

TGCGAAACCCGCTGCTGCTAAGATAGCCAATAACAAAAC

>M.02.3.11_A_37

TAATTCAGTTAATAGTGAAATTAGCCCTTGATTAACACTA

>M.02.3.11_A_38

TTCGCTAGCTAATTTTTCTAATATGTCTTTTACCTCTGGA

>M.02.3.11_A_39

TAACTTTTACGTAGGTTCTAAATTCTACGTAAAAGAGATTA

>M.02.3.11_A_40

TAGACTTTGAACTGCATTTGTTAGCAAGTTTCCTAAATTT

>M.02.3.11_A_41

TCCTGTACCTCAGCAACCCATACCTACACAGTCTTCTAGA

>M.02.3.11_A_42

TTTCTCTTCATAAGATCTCTTCATCAGTTTAATCAGGT

>M.02.3.11_A_43

TCTAGGTAGCAAGTTAGCGGGGAGTTGTAACGTTACTGT

>M.02.3.11_A_44

TCATAAAACGCCGAATCTACTGCATTCCATATTTCCGCC

>M.02.3.11_A_45

TACTTACATTTTTGTCTTCTCCAACGGAACACTTCTATTT

>M.02.3.11_A_46

TCTCATCTACAACCTTAATTTCCCTTATTACCTTTAACCCAT

>M.02.3.11_A_47

TCCGGCAGGCGCGGTGCCTCCTCCCTGTACCACTTATCGAA

>M.02.3.11_A_48

TTATCTCAACCCCGGTGTTGTTAGCGGTGCTTTGAGAAAT

>M.02.3.11_A_49

TTTCAGCTGGACTGAAGGAATAATGCATATGCATATACTGTT

>M.02.3.11_A_50

ATAACCCCACCTGCAAATTCATCAGTTAAAAGCATAACAA

>M.02.3.11_A_51

TGTTGATGGAGAGGTAGTAGAGAAAAAGAAGGTACCAAAA

>M.02.3.11_A_52

TTTGTTTCGCAGTGTACCGCTTATAACACCGGGATTTAGATA

>M.02.3.11_A_53

TTCATTAGTTTGACCAGCACTGAGATTGACCACAAATCTG

>M.02.3.11_A_54

AAATACATTACCTCTGTAGAGAACACGTTCGTATCCCG

>M.02.3.11_A_55

ATAACAGTTAATGTGAGTGCTGCACCTGTGGAAATTGACGCT

>M.02.3.11_A_56

TTATACCATACCTGCCTATGCCCCGTTGAACATAGACG

>M.02.3.11_A_57

TCTATTATTTTTAGAAAAGCCTATACTCCCGCTGTCTG

>M.02.3.11_A_58

TGTGTATCTATGGTGACGTTCTACTATTTCAGTTTTAGGGC

>M.02.3.11_A_59

TAGCAATGTAATTTATAGTGTAGTAGTCGAGGTTGTGTT

>M.02.3.11_A_60

TGCTAGTTGGATATCGAAATGGCAAAATGTACAAGCTAG

>M.02.3.11_A_61

TTATTGATGATGGGGTTTGCTACATTATTCGCTAAAAAG

>M.02.3.11_A_62

TCTTAGGAAGGAACGCATTTACAGAAGTGTTGGCGGGTA

>M.02.3.11_A_63

ATATATGTTTCCAGGCCATACAGTCGCAACTCCGATAAC

>M.02.3.11_A_64

TAAATCAACAACTACAAATTGGGCTAAATAACCTCGTTAT

>M.02.3.11_A_65

TATTGTCACATCAGAAGATTGCTGTGACATTAAGTTTC

>M.02.3.11_A_66

TGCCAACGCCACGATAATCCGAATGGGGAAGGAATTCCTAT

>M.02.3.11_A_67

TTTCAAAACCAGATAAAAAGCATAATATATAACTATTAT

>M.02.3.11_A_68

TTAAAAGAGAAGTAAAACTTTTCACATGTGTGTAAGTTCG

>M.02.3.11_A_69

TTCAGACTCATTGCGAAATTTGCTATCAATCGCCCCTATCAG

>M.02.3.11_A_70

TAATTATGAGGTTAGTTTCGAAAGGTGTTTTTACGATAAAA

>M.02.3.11_A_71

TCTTTCACAACTGTTTCAGGTACTGTGACGCAAACTAAT

>M.02.3.11_A_72

TGGTGGTGTACCAGAACTATATGCGATGTATGCGTTTATTCC

>M.02.3.11_A_73

TCACGACAAGCAGCGGAACTAGCGGTTATGCGATCGTT

>M.02.3.11_A_74

TTGAAAGTTATTACTCGTACCATGTTAACATAGTATGTT

>M.02.3.11_A_75

TGGGGATTTCTTCGCTGCTCATCTTTTTTCACCTCCTG

>M.02.3.11_A_76

TTAAATACGGCGTATTACTAAATAATACCGGCATCTACG

>M.02.3.11_A_77

CACTGGAATAGGAGATGTAGCACAAGCATTTAAGAAACCA

>M.02.3.11_A_78

TCTTTAGGTTTACATGTAAATTCTATACCGGGATAAACTA

>M.02.3.11_A_79

TGGATAAACTTCAGCGAGTTTAAGTACATCACTTGTGACAT

>M.02.3.11_A_80

TAAATCATTTGTTTTTGGTAACCCTAAGGCAGACATCATTT

>M.02.3.11_A_81

TATATAGGCTTTCAAATTATCATGATCTATAACGATATT

>M.02.3.11_A_82

TACAAGAACGTGATGGAGAAGATGGGCTTGGAGGTGA

>M.02.3.11_A_83

TAACTTGATATGAGAGTAAAAAATAGTGTAGTTATTTCAA

>M.02.3.11_A_84

TTTTAATCAACAGCTCCAAATACAAACTGCACCAACGTCCT

>M.02.3.11_A_85

ATTAAATCCGAAAGCACGTTAGTGAGAGAGAAAGATTTATA

>M.02.3.11_A_86

TTTCATCTTCGGTGAGCTGGAATTCGGTCGGGATCTTCC

>M.02.3.11_A_87

CCGAGACGCCCTGGCGAGGGTGATACACCTCTGCCCCACCCTTCG

>M.02.3.11_A_88

ACGATGCAAAACGGATTATTGAAGAAGTTATAAACAAATTGG

>M.02.3.11_A_89

TTCTATTTTGCGGGGAAGACATATGCATAGTTTATACATTAC

>M.02.3.11_A_90

TACTGGAGTTGGAACACTTACTAATACTATTAATTATGAA

>M.02.3.11_A_91

TTTTGTATCGTTTCCAAAATATCTGAAATTATCTCTAAA

>M.02.3.11_A_92

TTAAAGAATTTGCGAATTGGTACGGCTACAACTTCGTGAG

>M.02.3.11_A_93

TAAATCTTTCTCTAAGTCCTTTCGTAACCTCATCTGAGAC

>M.02.3.11_A_94

TAAAAAGTGGTATGTAACGTGGGATTGAAAATGTACAAGTGT

>M.02.3.11_A_95

TCTTCACTCCTTTAACGACATGGTCACGAATACTTTTTGG

>M.02.3.11_A_96

TTTCGAAACTAACCTCATAATGACAACATATGCATACTTCTGA

>M.02.3.11_A_97

AAAGCTTAACGTCCTCGTCACCAACGTGGTACTCCTCACTCC

>M.02.3.11_A_98

TAATTCCTCAATCTTTTCTTTTACCTTCATTAATTTTCACC

>M.02.3.11_A_99

CTAAATATGAGAGATTCACATCTTTTATAATCTTCACCAA

>M.02.3.11_A_100

TTGTAGAATTTGCTGTAGTTGTAATTGTGTGTTTTGCTG

>M.02.3.11_A_101

TCCAGATAAGGCGACTATAACTATTATTAGTATAATGAATA

>M.02.3.11_A_102

TGTGTCAGTTTGAGAGAATGCAACGTTACTTAGTAATAATGT

>M.02.3.11_A_103

TTTTATGTTCAACTTGTGTAAAGTGGTTTTCCCATAAATAC

>M.02.3.11_A_104

TATTACAATTAACATGAGTACTGCACCCGTGGAAATCG

>M.02.3.11_A_105

TTAATTGTAATAGATCCAGGGGTTGAAAATATAAATGCAGG

>M.02.3.11_A_106

TTAAAAAATCAGAGGGAAACATATACGTTTTCTCATGCGTT

>M.02.3.11_A_107

TATTTTTCTGATATTCGTAAAAACAATGGGATATCCCA

>M.02.3.11_A_108

TGGGGTACGCAACAGTTTGAGTATAAAGGGTATTATGAT

>M.02.3.11_A_109

CCTACTTGTCCGTTAGTCCCGACGTATGCGAGCGGTTCGT

>M.02.3.11_A_110

TCTAATGATAGAGAATTTGCTAATTCGTTGATGTCTTGTA

>M.02.3.11_A_111

TTGCAATAAGTTTTTCCCTAGTATAGAAAAAACCCCTGTATT

>M.02.3.11_A_112

TTCGATAAATGTTACCGTAACGCCTAGTCAAACTTCCGGAACTAC

>M.02.3.11_A_113

GACTGCTCGCTCATATTTCATAAATAATTAACAACTAA

>M.02.3.11_A_114

TCTTTTGCTTCATGCCGAGGAAAAAAACCTGAAAATTATA

>M.02.3.11_A_115

TCTATATCTATTATATATACAAGAAATTCAATAAGTGATTT

>M.02.3.11_A_116

TAAGATGTTATCGGCAAATTGCTGCGCTTCTGGATTTC

>M.02.3.11_A_117

TGATAATAACGAAATGATAACATCTAAATAATTTTTATACG

>M.02.3.11_A_118

TTTAATGTTCTAATGTCCATTACTAATCATCTTCGCTA

>M.02.3.11_A_119

TTTTTGATATGCTAGTATGTCAATAGGCCCTATGATGTCAT

>M.02.3.11_A_120

TCTTCTCCAAGATTTGAATAAGCAAGTCTTGTACTTCTA

>M.02.3.11_A_121

TTTTAAGACCCAACTCTTTCTTAGTTTTCTATTTTCCAACAT

>M.03.0.16_A_1

TCTGTTATTCTTCCCTTTTCGTATATTTTTATTAAAAT

>M.03.0.16_A_2

CTTCAGTCTCTACCGTTAGGGTAAATGGTAAATAACC

>M.03.0.16_A_3

AATACTTCATCAATAAGTAGACCACTGTGGTCAATTGGCGT

>M.03.0.16_A_4

ATTGGAATCTCGTTTATTCCAGCACTTGTAAACGAACGAA

>M.03.0.16_A_5

TAACCTTAGATACATCGCTGACGTCAGTAGATATGCAAA

>M.03.0.16_A_6

TCACAAGTTTCTTATCTTTATTTATAGATGGTATTATAT

>M.03.0.16_A_7

ATGATACCAACATCTTGAAACAACTCAATAATAAACAAG

>M.03.0.16_A_8

CTAGTCTCCAACCACTTAACTGGGGAGTTAAGTGGTTA

>M.03.0.16_A_9

CACTGAACTTAATCGTGAATTATACAGAGGCCAAAGCCG

>M.03.0.16_A_10

AACATAAAAGCAATTATAGTTATCACAGACATCTTAGTAC

>M.03.0.16_A_11

ACCAAGATTGAGAACATGGGTCATCTTTGGACCGATGGAA

>M.03.0.16_A_12

CTACCATCAATAATTGCACCGGGTCTGATGTACTCTTCTAC

>M.03.0.16_A_13

CACAAGAAGGCGAGACTCAACTAGAAAACAAAATTAA

>M.03.0.16_A_14

AGTAATGTAACGAGATGATTAGGTTTGAACGTGTAAGG

>M.03.0.16_A_15

CTTCAAATTGAATGCTTAATCGTTTATCTTTTTTATAAT

>M.03.0.16_A_16

TAAATAACGAAAACCTCCTTACCAGACTCCATCTCTTCCTC

>M.03.0.16_A_17

CTTTTTAACATTAATATAAATAACATAATCGATATCAA

>M.03.0.16_A_18

CTATATTTAAACATTTGTCTGGCAGACTTTTAAAGTTTC

>M.03.0.16_A_19

ACAACAGAACCAGCTGGAGCACTTGCCGCAATATTAAAG

>M.03.0.16_A_20

TTTACAGTGATGCGTATCGTTTTCAGGATCAAGAAGTGG

>M.03.0.16_A_21

TTGAAAATGATGATGAGCCTCATCTATCACAAATAAAGTA

>M.03.0.16_A_22

AAAATATATTCTTAGCGGTGCTAAAGTAGTGACTACAAT

>M.03.0.16_A_23

AGATGGCTGCAGTTATGAACAAATTAATACCCGCCATAG

>M.03.0.16_A_24

TTTCTATTAAGAGAAAGTTACACTTTATCAAAAGACACT

>M.03.0.16_A_25

CCCCTATACCCATTCAGAAAAAACTGTCGTACAGCCGGCCG

>M.03.0.16_A_26

ATTGTAGTCAGAAAGTCTATTATATCTTGAGTAGTAATT

>M.03.0.16_A_27

TATCTTTTAGCTACTGGTACTACTAAATTATTGACAA

>M.03.0.16_A_28

TTACGCCGCAAAGAACGGGAAGAGTACTTATAATTATTAA

>M.03.0.16_A_29

ATAAAAAGACCATCTAGTAAAATTCAATAGAAGTTTT

>M.03.0.16_A_30

GTGGGTTTACAATAGTGATGAAGTAAGGTATTTGCGG

>M.03.0.16_A_31

TTAGCACGAAGTCTCCCAATAGATGCCGTTATTTCT

>M.03.0.16_A_32

CTGAAGTCAGCATAATTAGAAAGGGACCATAAAGTA

>M.03.0.16_A_33

CATATCCTCTCTTCCCTCACCCAATTGAAGAAGAACCCTA

>M.03.0.16_A_34

TATTTTATACTCGCTTCATGTACACCATTTTCTGTAACTAG

>M.03.0.16_A_35

AGAAGAGGAAAAACTTGTAAGTCAAAATTCAATTTATTT

>M.03.0.16_A_36

ACCGCTAGCTCCGGAAGGAACTATTATGTTTATCGTTTTGC

>M.03.0.16_A_37

CTGTAGAACTCGTCCTCGTCGCCCCAAAGCGGAGCGAAGT

>M.03.0.16_A_38

TTAATTAATTATTTAGTCCTTATTATTCAAGTGTCGGG

>M.03.0.16_A_39

AAGATCGTGGACGCGAGCACCGTAGACGAAGAGATAATCCT

>M.03.0.16_A_40

ATACTAAGAAGTAACGTAACAGCTTCTGAGAGAACAAAG

>M.03.0.16_A_41

TTGCTTTGCGGAATAAGCTGATATGCGATAGCCCCACCAG

>M.03.0.16_A_42

GCGTTATGTTTTAGCTTATGAGTTCCCCTAATCAGTTC

>M.03.0.16_A_43

TCATCTCACCCTAGCGTTATTATATCATTATATAAGGAT

>M.03.0.16_A_44

CTCGGCTCGTGGGCTCAGATCGGGAACACGGTGTACGTG

>M.03.0.16_A_45

AATCCTTATTCTTTGACCTGCTGAATAATAATTTATTAATT

>M.03.0.16_A_46

ATCGCATTCTTTTTTCTTGAAATATGAAAAAAATCCGGC

>M.03.0.16_A_47

GTAACTACTTCACAGTCCCCTCTTCCAGTATATACAC

>M.03.0.16_A_48

TAGCGTCGTTATTTAGCCAAGCTCCTTCAACAAGTAGT

>M.03.0.16_A_49

TGTTCTTTCGCGATCATATAATAGTACTAGAGGTGCTTCTT

>M.03.0.16_A_50

TTAAACTATTATAAGTCTATGGAAGTGACATTTGTCTCTAA

>M.03.0.16_A_51

AACTCCAGTACTGCTTCGCCGTAGGATCCGTCCTCAGACG

>M.03.0.16_A_52

TATACCTTGACTTAAATCTACTCCTTCTTCCTTACAA

>M.03.0.16_A_53

GGAAACTATAATGAACTTATGAGAATTGGGAATGTTGAA

>M.03.0.16_A_54

CCTTACTTTTTCTTCCCCTTTGATACTTAATATACTTAA

>M.03.0.16_A_55

TCATTTACTATGATGATGCTTATAGATATGTAGACATTGCCT

>M.03.0.16_A_56

GAAGTCTGCCGCTTAGTACTATGAGCTTAGAATCTATA

>M.03.0.16_A_57

TTTACTTTTACAGTGATTCTAACAGAAAACAGTGCT

>M.03.0.16_A_58

CTCTTCGTTGAAGAACGACGAAACTGTTTCAACCACATATT

>M.03.0.16_A_59

CAAATAAAGGCAAGCCCGCAGGCTAATACACAACCTACATTAAA

>M.03.0.16_A_60

GTTTCATCGAGACCACTTAGGAAGAAGTCCTCTAGTTC

>M.03.0.16_A_61

ATAATCGTTCAGGGTGGCAACCCACAGTTCGTGATACAA

>M.03.0.16_A_62

TTATCTTTCAACAATACAGTATGCATGTTTCAGGGATGCCG

>M.03.0.16_A_63

GATTCGTAGTTCTGTCTGTTACTTGTATCGTTATTGATTGT

>M.03.0.16_A_64

AAATTACTCACAAAAGGTTCAAATTCATCATAGAAATAT

>M.03.0.16_A_65

GTAATCATATCAGGAAACGAAGATGTTTACTTTCCTAATA

>M.03.0.16_A_66

TCTATATTATTAACTTCTATATCTATGTAAAGTACCTTAT

>M.03.0.16_A_67

CACGATAAACATCTTTCTCGACAATCTAGCGTTTGCTCTAT

>M.03.0.16_A_68

TAATAAATTATCTTTATTGTTCCCTCATAATCATATTTAA

>M.03.0.16_A_69

GGATTAAATATAGAAGTGAAACAGAAACGAAGAAAAAAGA

>M.03.0.16_A_70

AAATTATTGAAATAAAAATCAGATTCGATATAAAATT

>M.03.0.16_A_71

ATAAGAAACACTTCAAAAAGTGTTATCATTCTTCCTCA

>M.03.0.16_A_72

GTGTTATATTAGGGTATCTACTAGGAACAACATTATATG

>M.03.0.16_A_73

TCTACGGTTTGTTCGCTCCAACGCTCGTGCAGAAGGCG

>M.03.0.16_A_74

AGCAATGTAATTTATAGTGTAGTAGTGGAGGTCGTGTTTAC

>M.03.0.16_A_75

TACTCTGTATTATCCGGATAAAGATAGAATACTTTTCT

>M.03.0.16_A_76

TATCCCCCTAGAAGAATGTTAATATTAGCTAAATCTGATG

>M.03.0.16_A_77

TTTGCTATTATCTCTTCTTCTTTTAAGTTGAGTGTAAG

>M.03.0.16_A_78

ATTCTTCTAAAATCATTCGCAAATCCTCCTCCTACATCCCCT

>M.03.0.16_A_79

TTTTTATATAATAAATTATATTCTTCAGACTGAAACAAT

>M.03.0.16_A_80

CCCTATTAGTTCATCAGTATCTTTATAACCACTGCTTACT

>M.03.0.16_A_81

CCATTGGTAAAATGATATCATCACTAGAATGTTTATTATT

>M.03.0.16_A_82

CCATTCTGTGTAATTGAATTTTCTTGACTTTTTCTTCTCTTT

>M.03.0.16_A_83

TACTATCTTCCTTGCCAAATGATATCTCATTTTGGACG

>M.03.0.16_A_84

ATCCTCTTCCTGGACTCACGGGACGTCGAGGTCTTGTT

>M.03.0.16_A_85

AATGAATTCTATTTCTCTCGGCTCTCTTGTGTAGAAAAG

>M.03.0.16_A_86

GTTAGCGTTACCGCAGGTCGGCGAAAGTATAAACGTTTT

>M.03.0.16_A_87

TCTAAGAACTCAGCTAAATCCCTATCCTTTACCGTTTGCCT

>M.03.0.16_A_88

ATTTTAGTCAAGAAATCTATTATATCTTGAGTATTAAT

>M.03.0.16_A_89

ATCTTCATCTCCAAACTACTGCTATTAACTTATCTCACTC

>M.03.0.16_A_90

TAAATAATTATATCTTGTTGTTAATAATAAATTTCATGA

>M.03.0.16_A_91

TCATCCTTCTCGTGGCTATATATAAATCCACCGAAACCG

>M.03.0.16_A_92

CTCGTACTTCTTACATGATGGATAATGAGTTCTGCAGAT

>M.03.0.16_A_93

AAACCTATAAGCCCTAAACTGAATTCCTCTTCTTTCTTTTGT

>M.03.0.16_A_94

AAACGAAATATCGAAGGCGTCAGATGAGGAAAGGAAGAA

>M.03.0.16_A_95

GAAGCTTGGGTTCCAGTGGCTGCAGCACCTGCTGCAGGT

>M.03.0.16_A_96

ATTTAGATATTGTAATGCGGTCTGATAATTGCCTTGTTGTA

>M.03.0.16_A_97

TTTATCGGGGTCATCCCATGTCCGCAACCCAAAATCAA

>M.03.0.16_A_98

ATTACTTGCAAGACTTGGTGGTTTGCCCCATAGTTAGTAT

>M.03.0.16_A_99

TCATTATACTTAATTATGGATATTCAAACAGTTGAGG

>M.03.0.16_A_100

ACTGTACTTCTCTATGTTTTTTGTTCCTTATAAATATTT

>M.03.0.16_A_101

CTACGAATTTAACGCCCGCCCTAGATATCAATGATGCCTCGG

>M.03.0.16_A_102

ATTAGATTAATTTTTCTTATATTCTGAGGAAGTATGAATCA

>M.03.0.16_A_103

CAATTACAAACACAATTACAACAAACACAACCTCAACAA

>M.03.0.16_A_104

TGCGATTTCGCCGTTTGGCTTTAATAGCTCGGGGTGATTCT

>M.03.0.16_A_105

ATAGAACCCGAAAAAGTCACATACGGCGGTCAAGAAATT

>M.03.0.16_A_106

GCTAAAATGTCTTGAGCTACTATTTTTAGATCTTTGAAT

>M.03.0.16_A_107

CTTGTTCTAACGTCGCATATGCAGAATCTATTAATCCGCGT

>M.03.0.27_A_1

AGCTTTCTTCTCGAACGGTCAGCTAGTAATAAATACCGT

>M.03.0.27_A_2

ATGTTGTGAAAGTATATAAAGTTGTTGAAGATTAATACTT

>M.03.0.27_A_3

TTCGATTTCGGAAATCCACAAGCACAACAATTCGCCGAT

>M.03.0.27_A_4

ATCTTCATCTCCAAACTACTGCTATTAACTTATCTCACTC

>M.03.0.27_A_5

TTCCGTGTAACCTAGAACTTTCAATTCTATAGTAGATTAT

>M.03.0.27_A_6

CAGTAAAGTAAGTAATAGTTTACGTATAAACCAATGTA

>M.03.0.27_A_7

GATGGTATGACTTTCAATTCTATAGTAGATTAT

>M.03.0.27_A_8

CCGCCTCCGCCACCACCTCCAAGTCCACCACCGCCA

>M.03.0.27_A_9

ATGCATAAACTCACTGAATAAATTAGGCGTAAATCCTGA

>M.03.0.27_A_10

ACAAAACAATTTGATTTGTATACTGCAGTTGCAGATTATGT

>M.03.0.27_A_11

ATATTAACTGTCTTCGTAAAAAAAGTTATAACTGGTATA

>M.03.0.27_A_12

AGACCTACGAGATGCCCAAATACTACATAACCTTTTTT

>M.03.0.27_A_13

TGTAATGGGCAAAACTCTGTTGTACATGTCAGAATATTC

>M.03.0.27_A_14

TTATATTCAAAAGGTTGCGTACCCCAAGGACGCAACTCC

>M.03.0.27_A_15

ATTTACACTAAGTGATGATGACAAGTATAGTATATTGT

>M.03.0.27_A_16

CGTTCACCGCCTATTAGCTCGATGCATCAACCATCACAA

>M.03.0.27_A_17

CGTAAAGATTACCTTACCCTCCTTCTTTACCTGTACTCTC

>M.03.0.27_A_18

ATTTGGCTGGCGAAGAAGCAAGATAAATCACCATTTCCATC

>M.03.0.27_A_19

GTAGATATTCCCGGGCCATACAGTCGCTACGCCAACGA

>M.03.0.27_A_20

CAAGACATGCGTTATATCTAGCTTGTGTGTTAAACAGAG

>M.03.0.27_A_21

TTTCTAAAGATGATAAAAGGTCAAACATTCTTTGACACTCG

>M.03.0.27_A_22

TACAGCTCGAAAATGATGATGAATAAAAACTTTTTCCAT

>M.03.0.27_A_23

AAACGAAATATCGAAGGCGTCAGATGAGGAAAGGAAGAA

>M.03.0.27_A_24

GTCTTATTAGCGTGGCAAAGAGGAAACAATTTTGCCACA

>M.03.0.27_A_25

ATTGTGCACTCCAAAACACGTTGATGAGCCTTGTTTGTTT

>M.03.0.27_A_26

ACTGAACGGGAGAGAGCTTACAAGCCTGTCAAACACATCC

>M.03.0.27_A_27

TTTACCCTTACCTCGGTGGTACTACCGAAAGTCTCTACCCCA

>M.03.0.27_A_28

CTCGGTGGCCACCAGAATGACGTGTTAGGATTTGTAT

>M.03.0.27_A_29

TTGCAATCAGCTGTTTTAAGCCTTACAACGTCTCCAG

>M.03.0.27_A_30

TATTTCTCATGAAAAACGACCTGATTAAACTGATGAAG

>M.03.0.27_A_31

AAGCTCTAAGACGATGAAAACCACCAATTATTCCAATTACAT

>M.03.0.27_A_32

AGTTCTTTCATAATCGGTTCAACGACCTCAAACTTCAG

>M.03.0.27_A_33

GAAGTTGCATAATAAGTTTTTGTTCCGTATACTCCCACTAT

>M.03.0.27_A_34

GCACCTGCCGAAGGTGCCGTTCCAGTGCCGTACGTAAC

>M.03.0.27_A_35

TTAACCCGGTAAATAACATTCCTACCTTTCTGGCATTTTC

>M.03.0.27_A_36

TTCCGCCGTATGTCGTAGTTTACGCTAATTATATACAGC

>M.03.0.27_A_37

TTATATCTGTGATATCAACACCAGTAATTTTCTGTATA

>M.03.0.27_A_38

TAAAACGACTGAAAACGGGTTCATTAGGCCTCGCCTT

>M.03.0.27_A_39

GAAATAATTCACTGCTTCTCTCTATCTATAAGAACGCAA

>M.03.0.27_A_40

AGCTTCCAGCGTGTTAAGGCGTGACGCACTTAACGGGTTT

>M.03.0.27_A_41

GGCATGACTGTAGGTAGCATTGGTGGTGGCCCCATCGG

>M.03.0.27_A_42

TTCTTATTTCCAGTCTTTATATCAGTACTTGTATAATA

>M.03.0.27_A_43

CTTATACTTAGACGTGACGGGGACACAGTTTACTAAAAG

>M.03.0.27_A_44

GCTGGTGAGGTGACATAATGAAAGCATATGTTTGCAAATTCTG

>M.03.0.27_A_45

CCACCCTTCGAACCCGTTGAACCTTGAGCTATAATATTAC

>M.03.0.27_A_46

ACCCGTTTGCTTTCGTACTTGGACTTGATAATAATAAT

>M.03.0.27_A_47

AGTACACTTGGAACATTTTGTAATGCATTAAATATATTTTCA

>M.03.0.27_A_48

TAATTTTATACGGTAATATACATTCGCTTAAAGCTTTCGC

>M.03.0.27_A_49

AGAGAGAGATGATTTGCTTCAACAAATCCTAAATCATAA

>M.03.0.27_A_50

AACCTTATCCAGTGATTGTTGATGCGTATACTTCAACTCGT

>M.03.0.27_A_51

AAAAATATACAACCCCTCGCGCTTGAGGTGTTTGAACTATATTA

>M.03.0.27_A_52

CGTTATCGCAAGTAGTAATAATATGAAGAATGCCGCAG

>M.03.0.27_A_53

CTTGTCGCACTGAATTCTCTAACTTGATTTCTCCACTCCT

>M.03.0.27_A_54

ATATATGTTTCCAGGCCATACAGTCGCAACTCCGATAAC

>M.03.0.27_A_55

TATATTGTGTATAAGCGATAACAATTCCCTAACGTCCT

>M.03.0.27_A_56

CAACAATCGATAACAATACAAGTAACAGACAGAACTACA

>M.03.0.27_A_57

TTCAAAAAATTGTTCTAAGTTGTACTCTCGGTTGTTTT

>M.03.0.27_A_58

AATTTTTTCAACTCTCGCAACTGGAATCTCTTCTTTTG

>M.03.0.27_A_59

TTAATTTCGACGTAAAGAACCCAATTATTGTTTTCAAGA

>M.03.0.27_A_60

AAGCTGTATTAAAATCATAAAGTGGTGCAGAATTTGT

>M.03.0.27_A_61

ATATTAACTGTCTTCGTAAAAAAAGTTGTGCAGAATTTGT

>M.03.0.27_A_62

GATCCTGTGACACTCTCGCTCGCTTCTGCTCCGCTAATCTC

>M.03.0.27_A_63

ATATCGAAGGAATAGATATATTGGCTAAGGGCAAAATT

>M.03.0.27_A_64

ACGATAGCACCGTAAATAACATCCGAGTACTCACCTAG

>M.03.0.27_A_65

TCATACCATCATTCTATAGTAGTGCGGTATACTTTGCAAA

>M.03.0.27_A_66

TTTGATCCAACTACACATTGGTTTAAGCAGCTTTATGC

>M.03.0.27_A_67

CCTAAATATCAACGTTACGTATTACGAACTTGCACTACATTT

>M.03.0.27_A_68

CAACATTAGTCGTCGTTTGGCTTAAGTTTAGTAAAAACCC

>M.03.0.27_A_69

AGTACACTTGGAACAATTTTTTGAACTTTCAATTCTATAGTAGATTAT

>M.03.0.27_A_70

ACCTGCGGTAACGCTAACGGGAATGCATAATATTGAATCG

>M.03.0.27_A_71

TTTCAATTCTATAGTAGATTATCTTATGATTTAGGATTTGTAT

>M.03.0.27_A_72

TTCAAAAAATTGTTCCAAGTGTACTCTTTCAATTCTATAGTAGATTAT

>M.03.0.27_A_73

TTCCGTGTAACCTAGAACTGCTTTGCCTCTTTGTGAACTG

>M.03.0.27_A_74

CTAATAAATATTAAAGTTTTGCATTCTTTGTACGCGTCACCA

>M.03.0.27_A_75

GGTAACGGTGTGCTATTAGAGGCACAGAGCGCTATACCTC

>M.03.0.27_A_76

TCATACCATCATTCTATAGTAGATTAT

>M.03.0.27_A_77

GTGACTTTATCAATAACTAGGTGACCCTGAGCTTCAAACC

>M.03.0.27_A_78

TCCACTTTCAGTGCAATATTGACAGAAAATTCCGCCGGAAA

>M.03.0.27_A_79

CACGTAGCTGATGTGTTCAGACTTTATTGGACAAAAGTCC

>M.03.0.27_A_80

CAATGTAAGTGAAATGAGGCCTGTAGATCATCTCATCAC

>M.03.0.27_A_81

ACGGTGCAACAAAAATGGATATTGCACACGGCAAAAG

>M.03.0.42_A_1

TACTGTTCCGTTTGCTGTATTCTGTATAACAATCCCGTTT

>M.03.0.42_A_2

GTTTTATTAGATAAAAATGAAGTTGAAGAAAAAATTATTA

>M.03.0.42_A_3

AAATTATTGAAATAAAAATCAGATTCGATATAAAATT

>M.03.0.42_A_4

CCCGTCGCCAACGTTCCGTTGAACGCTTGCCAATTTGCCA

>M.03.0.42_A_5

TATCCCCCTAGAAGAATGTTAATATTAGCTAAATCTGATG

>M.03.0.42_A_6

TTCTCTATATATTCAACGTGGAGGATTTTGGCGATAAATTTA

>M.03.0.42_A_7

CTTCAAATTGAATGCTTAATCGTTTATCTTTTTTATAAT

>M.03.0.42_A_8

GTCCACGTGTTTAACGCTACTCTCATTTACGATGAAT

>M.03.0.42_A_9

ACTACCATCCAGTCCCTACAATTTACCGGGTACAGGTCTAC

>M.03.0.42_A_10

AATGGCAAAAGGAACTAGTTGAATCTTCTCTTAAGTTAAA

>M.03.0.42_A_11

TGTAGAAATTTATCTTCCCCGCTTCGAGATATTTCATG

>M.03.0.42_A_12

TAACCTTAGATACATCGCTGACGTCAGTAGATATGCAAA

>M.03.0.42_A_13

AAACGAAATATCGAAGGCGTCAGATGAGGAAAGGAAGAA

>M.03.0.42_A_14

GATATCATAAGCAAAGGTGTAGTAACATATAATACAAATA

>M.03.0.42_A_15

ATAAATGTAAACGAACTTGAGGGGGTAACCCCAAAAAAGGT

>M.03.0.42_A_16

ATTTTTTATCCCTATATTATACTATGTTTAAGTAGTATAT

>M.03.0.42_A_17

ACGAAAAGTGGAGATGGAGGTTATGTTACTATAACCTAC

>M.03.0.42_A_18

TCTATATTATTAACTTCTATATCTATGTAAAGTACCTTAT

>M.03.0.42_A_19

TTTTCATATAGTCAAACAGTAGATGTTTGCA

>M.03.0.42_A_20

AGCCATTCGGCGTATGGCGGAAAAACTTTATTATTAAT

>M.03.0.42_A_21

ATGATACCAACATCTTGAAACAACTCAATAATAAACAAG

>M.03.0.42_A_22

CCTATTGGGACTTCATTGATACCCGCTGACGTAAAAGAT

>M.03.0.42_A_23

ACCGACCATCAGTTTTAACATTAACCCAAATCAGGGCA

>M.03.0.42_A_24

TACCACCGTTGCATTTGTTATCTCTCGGTAGTATACTTT

>M.03.0.42_A_25

AGTCATGAATAGCTTTTCTTAACTCAGGAAAAGCATCGCT

>M.03.0.42_A_26

ATGTTGGAGAAAGAAATCCTGCCAGTTGTACCACACATG

>M.03.0.42_A_27

CCTTACACGTTCAAACCTAACCATTTGGTCACATTACTT

>M.03.0.42_A_28

GCTATATGCCCCCACCCCCTGGAAATCCTGCCTTCTTT

>M.03.0.42_A_29

TAAGTTCTAAGAGCGCCCCGTAAGCTAGGGCTACTACTTCTT

>M.03.0.42_A_30

CTAAAGGTTGGAAATTCTCAATAGAGATCTGTGAGGGA

>M.03.0.42_A_31

TTTCAATACGGTGCAGTAAAGTACAGTATAAACTCCGTTT

>M.03.0.42_A_32

AAATCATTATCAAATTACGTAATTTCACAAAATCAATCT

>M.03.0.42_A_33

CTTACTTTATGGTCCCTTTCTAATTATGCTGACTTCAGTGGAT

>M.03.0.42_A_34

AAGCCCTATTTAGGGGGTAAACCCCCTAAAACCCCACGT

>M.03.0.42_A_35

TTTTGTTTTCCCCATTTTTCACTATCTTCGTTGTTCCTT

>M.03.0.42_A_36

TTACTATGTAGCAATTTTTGTATATCTCTAATGCTTTCT

>M.03.0.42_A_37

TCATCCTTCTCGTGGCTATATATAAATCCACCGAAACCG

>M.03.0.42_A_38

GATACAATTGCGTTAGCTAACGCTTCTAAACCGCTAG

>M.03.0.42_A_39

AAACTGCGGTGAACAATTGGGTTCAGAGAACACAAAGTG

>M.03.0.42_A_40

TGAATTAACCCACGAATTATTACATTACTAGACGCAAATGG

>M.03.0.42_A_41

CGGCGGTTACAACAACAAGTACTGCATACGTGATGGTGGGTTA

>M.03.0.42_A_42

CATCTACATGCATTTTATAAACCATAGCAACTGCATTTA

>M.03.0.42_A_43

CGTATTCAATATCATTTTTAATAAGAAAATTAAGAAGGGG

>M.03.0.42_A_44

ACCTAGTGGTAATGGGGGATTCGCGTTAAACGTCACCTT

>M.03.0.42_A_45

AATACTTCATCAATAAGTAGACCACTGTGGTCAATTGGCGT

>M.03.0.42_A_46

ATGTGGGGACACCCCACTCCCCAATCCTATTTATCACGTG

>M.03.0.42_A_47

ATAATTTTTTAATAGAAGATGCTGAACTTATTTTCACAATT

>M.03.0.42_A_48

TAGGGTTTGGGATAGCTAATGCCAATCTAAGTCATCTAT

>M.03.0.42_A_49

AGAAAGAAACTGGGCAGAACTAAGAAACTTTATATCAA

>M.03.0.42_A_50

TAACACGGTGAATGGTAAGGGTTACTATGCTACAATAA

>M.03.0.42_A_51

TCATAAACAAACTCAATGTATATATCTTTATCACCTCGT

>M.03.0.42_A_52

TTACAATTATTCGTTCGTGATTTGTCCTTAATTGTAGAT

>M.03.0.42_A_53

TGGCTTGTTAAGCCAGGGAACTTACACACGTAACGTTT

>M.03.0.42_A_54

ATTTATACCTAAATGCTAGATAGAACACTTGTAATTGTTT

>M.03.0.42_A_55

TAAATAACGAAAACCTCCTTACCAGACTCCATCTCTTCCTC

>M.03.0.42_A_56

CTTTTTAACATTAATATAAATAACATAATCGATATCAA

>M.03.0.42_A_57

ATTTTAGTCAAGAAATCTATTATATCTTGAGTATTAAT

>M.03.0.42_A_58

TTAATATTCTCGCTTGGATTTGGGGTTGCAGGTGGCGGA

>M.03.0.42_A_59

GATGATTATTACATATATGTGAGCGAAACTACTGATGA

>M.03.0.42_A_60

ACAACAGAACCAGCTGGAGCACTTGCCGCAATATTAAAG

>M.03.0.42_A_61

TAATATACTAAACTTGTTTGTTGAAGTTCAAAGAAGTTGC

>M.03.0.42_A_62

TCATAGTATACAAAATGATGAAATGATGAGGCGATATG

>M.03.0.42_A_63

TTTCTTTTCATATAATCCCCCTAGAATCTAATTTTTTCGA

>M.03.0.42_A_64

CTAGCATATGTTAGATTTAGCGATTGAATTACAGTTCCCGCT

>M.03.0.42_A_65

TCATTATCATTAACGCTAACAAACGCCGTATGATAGCCT

>M.03.0.42_A_66

CATTATAGCTATTTCAAGTAAAGATAGATGCCCTTTTAT

>M.03.0.42_A_67

ATCCTCTTCCTGGACTCACGGGACGTCGAGGTCTTGTT

>M.03.0.42_A_68

TAAGTTTCATTGTATAGATAATTTTGTCCTGGTGAT

>M.03.0.42_A_69

TCTGATGCAGAGGGCATGGACTTATTAGAAGAAGAGACAA

>M.03.0.42_A_70

CTTTCCTTCTTTTACTTGTACCAGTAAACACATGGCGG

>M.03.0.42_A_71

TAGAAAGGGTAGGGAGAGTAGTAGGAATGCCTCCCAGTAT

>M.03.0.42_A_72

GAAAGCATAAAAAAGCTTATACTAGATAACATAAATGACT

>M.03.0.42_A_73

AATATGAAGATGTAGAAGATGTACCAAGGTATTAAACCTA

>M.03.0.42_A_74

TTTCTATTAAGAGAAAGTTACACTTTATCAAAAGACACT

>M.03.0.42_A_75

ATTACTTGCAAGACTTGGTGGTTTGCCCCATAGTTAGTAT

>M.03.0.42_A_76

ACGTACATAAAAATTGCTATAGCATTTGGAGCTGCGGCA

>M.03.0.42_A_77

TTTATCGGGGTCATCCCATGTCCGCAACCCAAAATCAA

>M.03.0.42_A_78

ATAATCGTTCAGGGTGGCAACCCACAGTTCGTGATACAA

>M.03.0.42_A_79

ATTGTAGTCAGAAAGTCTATTATATCTTGAGTAGTAATT

>M.03.0.42_A_80

GTTCTAATAGCTTCTGTTTCAGAGATGGGTTAGCTAA

>M.03.0.42_A_81

TCCTATCTCCTCAAAACCTAGCGATGTTAGCTACAACAGC

>M.03.0.42_A_82

TATCTTTTAGCTACTGGTACTACTAAATTATTGACAA

>M.03.0.42_A_83

ATAAAAAGACCATCTAGTAAAATTCAATAGAAGTTTT

>M.03.0.42_A_84

GTGGGTTTACAATAGTGATGAAGTAAGGTATTTGCGG

>M.03.0.42_A_85

AAACCTTTATTAAATTACGTAATTCCTCAAAATCAATCAT

>M.03.0.42_A_86

CATTAAATATTGCCTTAAACGAATATTTAATATTGTCTT

>M.03.0.42_A_87

GAACAGGTCGAAAGAGCTGAGCTTTGGCATGCAATTTA

>M.03.0.42_A_88

AGGTATTGAACAGGTGGCTCGTTCTCACCGGAAGGTAGT

>M.03.0.42_A_89

TAAAAATTCCCGCTAACATATATATTAGATGTTTAAAGT

>M.03.0.42_A_90

CTTGTTCTAACGTCGCATATGCAGAATCTATTAATCCGCGT

>M.03.0.42_A_91

GTTCTCTGTTCTGCCATATCGCTATCCAGTAATCACT

>M.03.0.42_A_92

CTCGTACTTCTTACATGATGGATAATGAGTTCTGCAGGT

>M.03.0.42_A_93

TTAGTTTGAACAAGTTGGACATAACGTCCGTAGAACC

>M.03.0.42_A_94

TCATCTCACCCTAGCGTTATTATATCATTATATAAGGAT

>M.03.0.42_A_95

TAAATTCCAAAATCAAAAAAATGATTATAAAAGTAATCA

>M.03.0.42_A_96

TTTGTGAGTTGGAGACCGTTACGGCCACGCATTCCACGCGT

>M.03.0.42_A_97

TTGAAAATGATGATGAGCCTCATCTATCACAAATAAAGTA

>M.03.0.42_A_98

GATGAATTGCCCTACGTATAAATCCCCTAAGAACTTAAA

>M.03.0.42_A_99

CTTTTCTGTAATCGCTGGGATTCCTCATTATCAGATAAT

>M.03.0.42_A_100

TGGGAAATACTCATCCGCCATGTTTTCACTAAAACATA

>M.03.0.42_A_101

TTATAATTATAACTTAAGTCTAGCTCATTTGCTAACGCAAT

>M.03.0.42_A_102

CATATCCTCTCTTCCCTCACCCAATTGAAGAAGAACCCTA

>M.03.0.42_A_103

CCGAGACGCCCTGGCGAGGGTGATACACCTCTGCCCCACCCTTCG

>M.03.0.42_A_104

GTAGGTAGATAATCTACTCTGAGAGGAACGCTAGATTGA

>M.03.0.42_A_105

TGATTTTTCCGTTTTCATTAACTAGGGCTTGGTTATCTTT

>M.03.0.42_A_106

CATTAGGCCATATAAGTTGAAATTCGCTTATTTGATGCCCC

>M.03.0.42_A_107

TATAATTATAAAAAGGTGACCACACTTAGCCCCGCAAT

>M.03.0.42_A_108

ATCTATAAAGAATATAGCCTCAAACATCTTATTTTGACCA

>M.03.0.42_A_109

CCTTCTTCCTAAAGAGGCACCTCTAGTACTATTATAC

>M.03.0.42_A_110

AAAACAGCTGTTTGAACTCTAAAATTTCTATTTTATGGCGA

>M.03.0.42_A_111

CCAGCAGCAGCAAACCCTAGCGTGAATGTCAATGGA

>M.03.0.42_A_112

TCACAAGTTTCTTATCTTTATTTATAGATGGTATTATAT

>M.03.0.42_A_113

TAATTTTTTCGTTGTTTTTATCAAAACACAAAGCACTA

>M.03.0.42_A_114

TTTACAGTGATGCGTATCGTTTTCAGGATCAAGAAGTGG

>M.03.0.42_A_115

AATTTAATTTCTTCAACTGTGGTAACTGCACCATCACCAGC

>M.03.0.42_A_116

CCTTACACGTTCAAACCTAATCATCTCGTTACATTACTCA

>M.03.0.42_A_117

AAAATTTCTCCTACCATATATTACTTTCACTCATCTCTC

>M.03.0.42_A_118

TTTTTGGTTTCACTTAATACTGTTAACGGGAAAGCATAT

>M.03.0.42_A_119

TTTATTTCGGATTGTTCTGTGATCCATAAAGCTTGTGACCCG

>M.03.0.42_A_120

ACTTTTACACTGTTGCAAATTTAACCTTTACTGTTGCATTCA

>M.03.0.42_A_121

AAGATCGTGGACGCGAGCACCGTAGACGAAGAGATAATCCT

>M.03.0.42_A_122

TTCGATTTCGCAAACATAGTGAGCACTTTCGATGCGAAAG

>M.03.0.42_A_123

AATTTACTATTTTCAGTAATCACATCTTGTACTACTAATT

>M.03.0.42_A_124

ACGCCACCATTATTGACATCAGGCACATTTGGGCAAAGTT

>M.03.0.42_A_125

CCTAGCTTCACCGTGCAGGTTCTCAAGTGACGCTACAC

>M.03.0.42_A_126

CCTATGATGCCTTCTATGTTTCTTTTTATGATGTGACT

>M.03.0.42_A_127

TTAGATATTTCGCTAAGGCCGGAGTAAATACCTCCCCCTT

>M.03.0.42_A_128

TCTATATTGAATTCTATGCATATTCAATATTTCTCAACACCTT

>M.03.0.42_A_129

ACACGTATTATTGATCCGTTATGATCATAACATAACGCACTG

>M.03.0.42_A_130

ATCAAAACTGCTACCAAAACCCCTGCCGCTAGATACGGCC

>M.03.0.42_A_131

CTTGTGTGTGTCATTTGCAATTCCTCATAATAAACA

>M.03.0.42_A_132

AAATTAGTCGATACTATAAATCAGTTACAAAAACTATTGT

>M.03.0.42_A_133

ATCGCATTCTTTTTTCTTGAAATATGAAAAAAATCCGGC

>M.03.0.42_A_134

CTAAATATTAAAGTTTTGCATTCTTTGTACGCGTCACCAG

>M.03.0.42_A_135

TTAAACTATTATAAGTCTATGGAAGTGACATTTGTCTCTAA

>M.03.0.42_A_136

TTGATTAACACTAGAAATCGCATTTGAAATAATCGTTACGG

>M.03.0.42_A_137

TAAATTCCGCCAACAATAGATATTAATTCTAATAATTT

>M.03.0.42_A_138

ATCAGAGTTGATTATTTGCCAACTGGTGCAGTTCTTATAGG

>M.03.0.42_A_139

AATTGATTCAGTGCTTGTAAATATTGCTGAGGCGTTGATAACACA

>M.03.0.42_A_140

AAACCGCATCACAGTGGTTTTGAAGAAGTAAATATTTA

>M.03.0.42_A_141

TTATTTCTCAGAATATCAGAGAAATGGGATCGACAAACGGG

>M.03.0.42_A_142

GAATATTCATCGACATATAAAATAACTTCCTTATTTCTGT

>M.03.0.42_A_143

CTGTAGAACTCGTCCTCGTCGCCCCAAAGCGGAGCGAAGT

>M.03.0.42_A_144

AGGATATCCATGCTATAGTGATGTCGAATTTAGTCCCC

>M.03.0.42_A_145

TAATAATATATTTATATATCTTTTCTGCATCAACTATCA

>M.03.0.42_A_146

GAAGTCTGCCGCTTAGTACTATGAGCTTAGAATCTATA

>M.03.0.42_A_147

CCACCTCGACGACGTTTTTGCCCTTCTCGACCTTCACGAC

>M.03.0.42_A_148

ATCTTTGGGAGGGCGAGCGGGTTTATCCCGAAGTTCGGG

>M.03.0.42_A_149

ACCACTCCAGTTAAACTTTTACTAACTTACTAACTTACA

>M.03.0.42_A_150

CACGACATTAGAAGGAGTGGGGCGTATTGGCGTATAAA

>M.03.0.42_A_151

ATAATACGGTACCTACGTCCATTTGCGACGTCTCATTAAT

>M.03.0.42_A_152

CTCTTCGTTGAAGAACGACGAAACTGTTTCAACCACATATT

>M.03.0.42_A_153

TTAAGTTATTGGGGACAATACGGGGGATTTTTATTCAAT

>M.03.0.42_A_154

AATAGAAATTATAGAAACTAAAACAGAAGCAGGGAGTAAAAA

>M.03.0.42_A_155

GTTTCATCGAGACCACTTAGGAAGAAGTCCTCTAGTTC

>M.03.0.42_A_156

AATCTACGGTGCAGCAGGTCTATTTTTTGGAGTATTGA

>M.03.0.42_A_157

AGACTAGCTACACCCTGTTTCCAAACTTCATCTGATGAC

>M.03.0.42_A_158

TCATTTTAGACATTGTGTCGAGGAGCATGGACTTGATGTTG

>M.03.0.42_A_159

GTGTATACAATCTTTGAATAAGATAAGTGTAAACCCAGAT

>M.03.0.42_A_160

TTATCTTTCAACAATACAGTATGCATGTTTCAGGGATGCCG

>M.03.0.42_A_161

TAACGCTTTGCCGATTCTTCTTCAGTCCAACTTGAACCA

>M.03.0.42_A_162

TCATTATACTTAATTATGGATATTCAAACAGTTGAGG

>M.03.0.42_A_163

ATATAAAGATTTTGCGATTCAAGTGTCTTTCATTGACTTT

>M.03.0.42_A_164

TTTTTCCGCCATACGCCGAATGGCTCTTTCAATTCTATAGTAGATTAT

>M.03.0.42_A_165

TTATTTCTCAAAGCACCGCTAACAACACCGGGGTTGAGA

>M.03.0.42_A_166

TTATCTGCTAATAATTTCGTATGCTTAAGCACAGTTCCAG

>M.03.0.42_A_167

TTTGTAACCACCTTTTCGGTTACGCTCTCAAACTTACCACTAAT

>M.03.0.42_A_168

GTAATCATATCAGGAAACGAAGATGTTTACTTTCCTAATA

>M.03.0.42_A_169

AAATTTTGTAGTGCTTCTTGCAACAGTAAAGCTCTAGT

>M.03.0.42_A_170

TTTGCTGTGTTTTGAATTATCACACCGCTTCCGCCAT

>M.03.0.42_A_171

CACGATAAACATCTTTCTCGACAATCTAGCGTTTGCTCTAT

>M.03.0.42_A_172

TAATAAATTATCTTTATTGTTCCCTCATAATCATATTTAA

>M.03.0.42_A_173

GGATTAAATATAGAAGTGAAACAGAAACGAAGAAAAAAGA

>M.03.0.42_A_174

GTAAAAATTACAGAATCTCTTTATAAAATTTTAGAGCAGAAG

>M.03.0.42_A_175

CCATTGGTAAAATGATATCATCACTAGAATGTTTATTATT

>M.03.0.42_A_176

GAAGTTGCATAATAAGTTTTTGTTCCGTATACTCCCACTAT

>M.03.0.42_A_177

TCATTTGTATACAATCTTATACATATTTCTTCATTATTCT

>M.03.0.42_A_178

ATAAGAAACACTTCAAAAAGTGTTATCATTCTTCCTCA

>M.03.0.42_A_179

AATATAAGTTGCAAGATATTTTTAAAGAGTAACGCCTT

>M.03.0.42_A_180

CCAACACTAGCACCAGCACCTCCATATGTCCATGCTATCTG

>M.03.0.42_A_181

TAACACAGAAGACTGGACTAAGAAGGAACGTAAAACGGC

>M.03.0.42_A_182

TGTAACGGTTGAATATATTAATCACGTACACACGTATGT

>M.03.0.42_A_183

GTGTTATATTAGGGTATCTACTAGGAACAACATTATATG

>M.03.0.42_A_184

AAGATGTTATTGTTTCGCCTAAAAGTAGTTGGGCAAGT

>M.03.0.42_A_185

TTTATCATCGCCCATCTCTACCCCCTCACTCGTTTACATACTTTT

>M.03.0.42_A_186

AACAACCCCGCCATAAGGGTCAAGATAACTATAGTGAAA

>M.03.0.42_A_187

ACGTCAGTCTATGAACCGACGAACATTAACGAAACTAGCG

>M.03.0.42_A_188

CTGAGCTTACTTAAAAGAATATTCTTGTTATTATCTTGTTT

>M.03.0.42_A_189

AACTGATAGCGAAATAATAGATATGCTTAGAGATGGAAG

>M.03.0.42_A_190

ATATACTCGAATCCTACATCAAATCGATTTAAGTTTTCTT

>M.03.0.42_A_191

GTTAGCAGAAATAATAGCTACATATGACCCTAACAATGT

>M.03.0.42_A_192

ATTTCAATATTCGGCATGGTCTCCATTACGCCTTCTACTA

>M.03.0.42_A_193

GATAAATGGCACATTCGCAGATACACCTTCTTCTACT

>M.03.0.42_A_194

AATACATCGCTCATATCACTAACCCTGTCACGATTTGTTC

>M.03.0.42_A_195

GTGATAATAATCCAGTTGAAGTTGAAGTAGTCATAGATGT

>M.03.0.42_A_196

TTTGCTATTATCTCTTCTTCTTTTAAGTTGAGTGTAAG

>M.03.0.42_A_197

TGACGGAACTTTTTGGGCGACAACGAAATATGGCTCAGA

>M.03.0.42_A_198

GATATAATATATGACATATTGTACGGATGTGAACAGAATGCA

>M.03.0.42_A_199

TCTACGGTTTGTTCGCTCCAACGCTCGTGCAGAAGGCG

>M.03.0.42_A_200

TAAATATATTCTTAGCGGTGCTAAAGTAGTGACTACAAT

>M.03.0.42_A_201

GTAAATCCATATGAGTTCTATCAACTACTTCAGCAAACTGGA

>M.03.0.42_A_202

CTTAACGTCTCTTCCGGCAACCTGTCTATCCCGTGGC

>M.03.0.42_A_203

GGAAACTATAATGAACTTATGAGAATTGGGAATGTTGAA

>M.03.0.42_A_204

ATTCTTCTAAAATCATTCGCAAATCCTCCTCCTACATCCCCT

>M.03.0.42_A_205

CTTCTAATTCTTCTTTTAGCCTTGATGCAATCAGTATCA

>M.03.0.42_A_206

CCCTATTAGTTCATCAGTATCTTTATAACCACTGCTTACT

>M.03.0.42_A_207

CCTGGATCATGATATGTAGGTGCATTTTTAATGAAT

>M.03.0.42_A_208

GAATTTTGACCAAACGTGCCTGATGTTAATAATGGTGG

>M.03.0.42_A_209

GAAGAAGAGTTAGCAATATTAGATCTATATTGCGCAAATATG

>M.03.0.42_A_210

TAATTCATTCACTTCTTCTAACAATTGGTTATATCTCTC

>M.03.0.42_A_211

GTACCATAAGCAACTGCTTGTCTATTATTCTTTAGCTT

>M.03.0.42_A_212

TCACTTCTTAGCAACTGCAATGACTATCACAAGTAAGAA

>M.03.0.42_A_213

AAAAACTTTTAAACTCACAAGACTATAATAAAAATAAGGGT

>M.03.0.42_A_214

GTTTTGCGTTCAACTCAGGCGGTAGTTTAAATGATATTA

>M.03.0.42_A_215

TCAAAAATTTGTTCTTGACAGGTCAGAAAAGGACTGGTAAG

>M.03.0.42_A_216

CGATAATAGTGCAATGTATACTTATCAACATTAGAAGAT

>M.03.0.42_A_217

CTTGACATTATTCTTATACAATCTAAACAATAACTATGTG

>M.03.0.42_A_218

ATTACTAAAAGGTCGTTCTTGGGATCGTATCTATATC

>M.03.0.42_A_219

ACAAATAGAATTGCGACCGGGTACAATTTTGACAACAAAACTA

>M.03.0.42_A_220

ATCATTATTCCCACCACCTTATAGATACTTGTTTCATCATC

>M.03.0.42_A_221

AAACGTTTATTTTATCTTCAAAGATTTGTATTTAGGGGG

>M.03.0.42_A_222

AATGAATTCTATTTCTCTCGGCTCTCTTGTGTAGAAAAG

>M.03.0.42_A_223

AAGCCCTGGCAAACGTTTACGCTTGAAGTGGGAGTTTCC

>M.03.0.42_A_224

ATACTAAGAAGTAACGTAACAGCTTCTGAGAGAACAAAG

>M.03.0.42_A_225

TTAAATACACAAGGATTATAATTTTCATTAGCATATTCA

>M.03.0.42_A_226

ATTTCGGGGGATGTGGTTTTTGAGGTTTTACTTTTTACCA

>M.03.0.42_A_227

AATCCTTATTCTTTGACCTGCTGAATAATAATTTATTAATT

>M.03.0.42_A_228

CAGCACGCTGGAAGCTATGGCGTCAGCCTAAATACATTA

>M.03.0.42_A_229

ATCTTCATCTCCAAACTACTGCTATTAACTTATCTCACTC

>M.03.0.42_A_230

TCAACAGATATTCAGAAATATCTCGAGGAAATTACAATAA

>M.03.0.42_A_231

ATAGAACCCGAAAAAGTCACATACGGCGGTCAAGAAATT

>M.03.0.42_A_232

TCCTTTGGAACTCCCTCAATACATCTTTGTGATATAAA

>M.03.0.42_A_233

TAAATAATTATATCTTGTTGTTAATAATAAATTTCATGA

>M.03.0.42_A_234

TCAACTACTACAACTCCTGCATATGTATTTCCTACAACTCTT

>M.03.0.42_A_235

AGTTATGAGGAGTTAGAAGCCTATAAGAGTTTGTTCTTCGG

>M.03.0.42_A_236

GTTATTTCATACGCCTGTGCGAAATTGTTTGCATATTCTA

>M.03.0.42_A_237

GCTATCAAAACCACCAGAGATAGGATTAACCCCACTCCAC

>M.03.0.42_A_238

AGCAATGTAATTTATAGTGTAGTAGTGGAGGTCGTGTTTAC

>M.03.0.42_A_239

TATTGTAATGTACACATTTCCTAGGTTACTTCTTTCCA

>M.03.0.42_A_240

TCCAGGACGGAACAAAGCTAGGGGAATTATTTGCAACGTCC

>M.03.0.42_A_241

ACTCTGCTAGTCCTTTTACCATAAGCAGAAATTTAGCAGTGT

>M.03.0.42_A_242

AGTACAGGGACGTTCAATGAGTAAGGCTCCGCAAGGTAG

>M.03.0.42_A_243

TTAATTAATTATTTAGTCCTTATTATTCAAGTGTCGGG

>M.03.0.42_A_244

CTAGTCTCCAACCACTTAACTGGGGAGTTAAGTGGTTA

>M.03.0.42_A_245

ATTTAGATGGACAGACACCAGTTTAAGCACTTGATACACGT

>M.03.0.42_A_246

CAACAAAATCCAATTACTTCTCCTCAAATTCAATCAACAA

>M.03.0.42_A_247

TCTGCAGTTAGCTCCGGAAGTTCTCCTTCTTCTTCTACTTGCG

>M.03.0.42_A_248

TTCCTCAGCACTATTAAATCCACTGAAGTCAGCATAATTA

>M.03.0.42_A_249

AAACCTATAAGCCCTAAACTGAATTCCTCTTCTTTCTTTTGT

>M.03.0.42_A_250

TGTTTATATACACATATTTTCCAGTGAGGTCTCTTAGT

>M.03.0.42_A_251

AGAAGAGGAAAAACTTGTAAGTCAAAATTCAATTTATTT

>M.03.0.42_A_252

AATGATGACCCGATATATGACGATGTCAGGATAACGTAC

>M.03.0.42_A_253

AAAAATATACAACCCCTCGCGCTTGAGGTGTTTGAACT

>M.03.0.42_A_254

TTCCCTCCCTTTCAGCGATTATCTTTGAGATTGTAAGCT

>M.03.0.42_A_255

AGAAATACGGTGATTTTACTTAGGACCGGGGGTGGTCTG

>M.03.0.42_A_256

GCGATAATTGAACTTGCTAACGCTTCAAGTGCTTGTGAA

>M.03.0.42_A_257

TAGAAATAGTATTTCCGTCGGTGGTAGTTAAGTGCTCAAAAAA

>M.03.0.42_A_258

TACTCTGTATTATCCGGATAAAGATAGAATACTTTTCT

>M.03.0.42_A_259

AACTACGTCCACGTATTCTACTTGTGAATTAATGACGGG

>M.03.0.42_A_260

AGAACGGGTAAGCATTCCTCTACCTTATGCACCACATCAATCT

>M.03.0.42_A_261

GTTTCGGTAATTAATAAACTGATATCGCTATTTGGCAC

>M.03.0.42_A_262

ATATTATCATCATTGAATATCTCTACTGTATATGTTTG

>M.03.0.42_A_263

AATTCTTTCAATCTTTCTCTTATTGCCTCGTTAACAAATTC

>M.03.0.42_A_264

TCTGAAGTTGTGCCCCCCTCACTTTCACTTTCCTCCTC

>M.03.0.42_A_265

TGTACTCTGGATAGGGGAACCCGACAATGAAGATAGCCTT

>M.03.0.42_A_266

TTAAAAGTTCTTTGGCCGTAGTGGTGTCTAACCCGTT

>M.03.0.42_A_267

ACGCTTAATGAACTCAAAGCGAAAACGGGAAAAACGAT

>M.03.0.42_A_268

TTTGGTAACTCAACTTGTATACTTCTCCTTTCACCTTTA

>M.03.0.42_A_269

TTTTCTATAAGTCCGCCTCAAGGAGTTACTAGAACAAT

>M.03.0.42_A_270

TCTACAATTTTGGAGGAATTTGGATTAGATTTAACTTTAT

>M.03.0.42_A_271

TTTCCTCCGTTTATTGCTATTGCACTGCTGCTAGAATTCA

>M.03.0.42_A_272

CCGCATAAGCAAATCTTGCTACTACCATCCCGCCTGCCCCA

>M.03.0.42_A_273

CCAGTTGTCGCTTCATATGCAACATCTATCCAATACGTT

>M.03.0.42_A_274

AACTCCAGTACTGCTTCGCCGTAGGATCCGTCCTCAGACG

>M.03.0.42_A_275

ACTGTACTTCTCTATGTTTTTTGTTCCTTATAAATATTT

>M.03.0.42_A_276

TATCTTCCGCCATTCATACAAAAAATCCTAAAAGCAGA

>M.03.0.42_A_277

CTACGAATTTAACGCCCGCCCTAGATATCAATGATGCCTCGG

>M.03.0.42_A_278

ACTGATACTCTATCCCATATATTGCAAGATCCCTATC

>M.03.0.42_A_279

TTTTGTTGACTTATCACCACTAGCCCGCTCTTATAAGGAT

>M.03.0.42_A_280

AGTACAACCACCGCAATCTGCATCTCATTCACCGTTAT

>M.03.0.42_A_281

GTATTGAACGCAGATATTAAAGCAGCATCTTCATATTCCGT

>M.03.0.42_A_282

ATGGTTGAAAGTACGTCATGGAAAGTACTACCGTAT

>M.03.0.42_A_283

TATTGTCCTGCAGTCTCGTAATATACTTTAGTCCCATACA

>M.03.0.42_A_284

TATAATAATTAAATACTTTGGTGTTAGACTGAAATCTCTT

>M.03.0.42_A_285

TTCCTATTAATTCATCAGTATCTTTATAACCACTGTTTACC

>M.03.0.42_A_286

AAATCTTTCGCTAACGGAGAGGTTAGATCTGCGGCT

>M.03.0.42_A_287

TGTATTAGTAAAAATTGACTGTGAGGGATGTGAGTATA

>M.03.0.42_A_288

TCGATATTTACTTTCACTTCGCTCAAACCATCCAGATAT

>M.03.0.42_A_289

TTTTGACGACCGTGTCTATGAATTCAACGGAATCCTCAT

>M.03.0.42_A_290

TGCGATTTCGCCGTTTGGCTTTAATAGCTCGGGGTGATTCT

>M.03.0.42_A_291

ACCTTCTCTTCCTAGAGCTATAGGATCTAAGCCCATCTC

>M.03.0.42_A_292

TATTTAGGTGGCAAATTGTTGGCGAAAACAAATGTTACAA

>M.03.0.42_A_293

GTTACGTGCATTGTATTTAGCAAAGAGGCTTAAGGAAATG

>M.03.0.42_A_294

ACCGCTAGCTCCGGAAGGAACTATTATGTTTATCGTTTTGC

>M.03.0.42_A_295

TATTTTTTCAGGTTCTCAATTTTGGCCTTATTTAAGT

>M.03.0.42_A_296

ATTTATAACAAACTCCAAGAATGGGAGAGAGTAAACAT

>M.03.0.42_A_297

TAGACCCTTCTTATCTACTTAGGAAGTTAGACGGTTACC

>M.03.0.42_A_298

ACGCAACCCCCAAATTTCATAGTATATGAATATCTAAA

>M.03.0.42_A_299

CCTTACTTTTTCTTCCCCTTTGATACTTAATATACTTAA

>M.03.0.42_A_300

AACCTGCATTAACAAGCGTATTTCCGCTTCCTGTTACACTTTCGGT

>M.03.0.42_A_301

ACATTATAGGCAGTCTATCATCTTTAGATGCAAGCATT

>M.03.0.42_A_302

CCTTTATCTTCGGTAAGCAAGTGTTATTGGGGACACCA

>M.03.0.50_A_1

ATTGAGAACGAAGAAGTGATATATACCAGGCCAAGCGGAGG

>M.03.0.50_A_2

TTTTCTATAAGTCCACCGCAAGGAGTTACTAGAACAAT

>M.03.0.50_A_3

TACTCGTGTTTGATGTTGCAAATACACTACCTACACCAAT

>M.03.0.50_A_4

GTACCCCTTTAGCACTAGATATAAATACGATAGAATTTCCG

>M.03.0.50_A_5

TCATATTATTTTATCTATGAAACAGAAAATGAGAATTTTTA

>M.03.0.50_A_6

CATAAAGTATATGTGCTGAAGTCTTCTTTGATCCGATA

>M.03.0.50_A_7

TTCCTACTCTAATTTTAAACTAGAGAAAATCCTTATTTA

>M.03.0.50_A_8

TTGGCTGGAACCAGATATTGCTGAGGTAAGCTAGTTCC

>M.03.0.50_A_9

TCATCTAATGAAATTATATAAAAATTCTGCAAAAATGAAT

>M.03.0.50_A_10

GTAATGATGAACTTTACAAAATTCCTCATTGTATTAGCC

>M.03.0.50_A_11

TAAGCTAAAGAATGAGTAGCTAATACTATAAATTGCTTAT

>M.03.0.50_A_12

TTCAATATACCTTTACCTTTAGATCTTCAGAAAATAGTA

>M.03.0.50_A_13

TCGGCTTATATCTAACATCCTCTGGGAGGCTTTCCATGT

>M.03.0.50_A_14

TTGCATAATCTTTTCTGAATCCTAATGCCATCTTTTCCTT

>M.03.0.50_A_15

ACATCTTTGTGGCCGTGAACTTCCTCAGATATTTCGGGGCTA

>M.03.0.50_A_16

ATTTTAGATCAAGTGTGGCAGCATATACAAAGTTGCCCAG

>M.03.0.50_A_17

TTTCAATTCTATAGTAGATTATCTTAGAGGGGGGCCTCATTCTCA

>M.03.0.50_A_18

CTGTTCCTGGAGTTTATCAATCAAGTTTATGACTTCATT

>M.03.0.50_A_19

TTTCAAATCATTGTAGTCCCAAATAAAGGGTATTTTTG

>M.03.0.50_A_20

ACTGGTTTATTTTCCTGTTTCAAAAGCGTTATAATATCAT

>M.03.0.50_A_21

GCATTACTAATAGGCGACCCGGGTACCGGTAAAACGGAA

>M.03.0.50_A_22

AATATTGACAATATTAAACCGCTTAAGCCTATGACAACTCC

>M.03.0.50_A_23

GGATATAAAATATTACAATTGAAAGCAGAAGTACGTCAA

>M.03.0.50_A_24

AGAATTGATCCCATGAAAATTCCAGCTAACATATATATTA

>M.03.0.50_A_25

ATACCCCTACACTGGTATATATTCAGCCGTATATAG

>M.03.0.50_A_26

ACATTTAACTTGGTATAAAGGACTAGGTCGTCCTCTTTGTA

>M.03.0.50_A_27

AATTTAATTTCTTCAGTAACTGCACCATCACCAGCACCAT

>M.03.0.50_A_28

CCCATGATTTTCAATTCACCAATTGTACAAAAATATATTCTT

>M.03.0.50_A_29

GAAAGCGTAAAGATGAAGTTACTAGACGAAATCAAAAA

>M.03.0.50_A_30

CATTATCCATAAGCGGTAGCGGTACATTGGAGATAGCCT

>M.03.0.50_A_31

AAGACTTAGATCATGGTGTGATACATCAATATTTAGGA

>M.03.0.50_A_32

TTCAAGTAACTCTTTTTCTTGAGTTTGGGCCATTTTGGGG

>M.03.0.50_A_33

CAAACGACGCCTATCGTAGATATCATTGAGTCCTCAC

>M.03.0.50_A_34

AAATAACAATATAAGCCACCCCGATGGATGTGAGGGG

>M.03.0.50_A_35

ATACCAAGGCTGAATGGAGGCCTAGAGTAAACCCCCCA

>M.03.0.50_A_36

TCTGATCTGTTATCAGGTCTAATGAGTATTTGGCCTTA

>M.03.0.50_A_37

TATCTCTTATCTTATGTCTTCCAAACGCCAGAAATCTT

>M.03.0.50_A_38

AGATTTGTCATTTGAATTATATAACTATGAGTAAATGTTA

>M.03.0.50_A_39

GTAGACGCTACTGCATTAGGCATAGGTGTTATAAACGT

>M.03.0.50_A_40

AGATAATTAAATTGAGCGAAAGTGAAAAAGATATGTTAAA

>M.03.0.50_A_41

CTCAAGGCAATTATAAATGAGGTGTCGACCAAGGTACCT

>M.03.0.50_A_42

GTAGTGCTGTTAGGGGCCACTGTATTAGGCACTAAGAT

>M.03.0.50_A_43

TTTGAAAGTATGTATCAGTTCGAGATCATAGAGAAAGCTGT

>M.03.0.50_A_44

CTCTTTTGTTAAACATGTTTTTCCTAAGCGTATTATAAT

>M.03.0.50_A_45

TACTAGAAATCCCGCCACTATTCCTATGAAATTAGTTAGCAA

>M.03.0.50_A_46

AATTGCAAACTTATTGGTACTTAAAGCCCTTCATAAACAT

>M.03.0.50_A_47

ATTAGTTTTATTAAATGATCCATCAATTCAAACTTTGACATGT

>M.03.0.50_A_48

GGTTTTATCGGAACAGAAAAATTAACTTGATTATAAGT

>M.03.0.50_A_49

AGGTGATTAGTGACGTCAGTACCTTATAGTGTAGATCA

>M.03.0.50_A_50

ACTAAAACACAACTAAATATAACACTATATAACACACACA

>M.03.0.50_A_51

GATTGAGACACCGCCACCAGCAACAACTCCTACAACACCT

>M.03.0.50_A_52

ATAAACTATCAACTTGTACAATCCATATATTTTAAGGTAGC

>M.03.0.50_A_53

TAGTTCTTACAGGATTTGCAGGGGTTATGAAAATAGCCT

>M.03.0.50_A_54

AGTAGTCCATTACCTTCTCCATCATCTCCATTAATGGGTGC

>M.03.0.50_A_55

AATCATCTCGTTACATTACTCACAGACGTTAAGTCAATCA

>M.03.0.50_A_56

GAAAAATAAAAAAGCTTTTTAGAAATATACTTCTAAATA

>M.03.0.50_A_57

ACAATCCGCAATTAATTCTTTACATTCCTCCAAATATTTT

>M.03.0.50_A_58

ATTTTATATATATTGTATTAGTCGTTGAATTAACTACA

>M.03.0.50_A_59

TTCCAATACGGCATGGTGAAGTACAGCATAAATTCCGTTT

>M.03.0.50_A_60

TTAAGTCCCCACATCGTTCCAAATTGTTTTTCACATAA

>M.03.0.50_A_61

CGATACTCGAACGGTTGCGTACCCCAAGGACGTAATTCTA

>M.03.0.50_A_62

GAGGCATTACTAATAGGCGACCCGGGTACCGGTAAAACGGAA

>M.03.0.50_A_63

CCTTCTCAGGTGCATTTTTCTCTTTCCGCAGTCTCTCTAT

>M.03.0.50_A_64

GAAGAAGTGTTACCCTAAGGAGACACATGACACGATAGACCA

>M.03.0.50_A_65

CCTCGTCATGGCTTACCACGCCTTTCCCCTTTGGTGG

>M.03.0.50_A_66

GAACCCGTTTTTCAATATTTATCAAACTGTTTCAAACTT

>M.03.0.50_A_67

ACCACTCCCGTTAAACTTTTACTAACTTACTAACTTACA

>M.03.0.50_A_68

GAAGTCTGCCGCTTAGTACTATGAGCTTAGAATCTATA

>M.03.0.50_A_69

AACGGTGAAAGTGCTACTTTATCATACGGGCAAGCATTAGA

>M.03.0.50_A_70

CAGACCAGTTATATACGGTAATTCAGAAAAGCAATATA

>M.03.0.50_A_71

GTTTCCATAAGATATCGCAGAATAAATGGCGGTAATGGTCT

>M.03.0.50_A_72

GTAACAGGGACTTTTGGACATTTAGCAGCGCATCCGAA

>M.03.0.50_A_73

AGGTGTATAGGTGACATGTGATATCTGTTCCTCCTCCTGAT

>M.03.0.50_A_74

TTTCATGTTTTTGTTATCATTCAATCCTTGGCAATTGAGTGT

>M.03.0.50_A_75

CATTTATGTGGATCTGGTTGTTTCTCTTTAAGAAGATAA

>M.03.0.50_A_76

GTCTAGTTTTTTGTGCCCCGTTATTTTTGCTATGAT

>M.03.0.50_A_77

TAATGTTCTAATCCCCTATGCCTCACTGGGAGGGGCATA

>M.03.0.50_A_78

AGTTTAGATGAAATTAAGCAAACGTTAGAAAAAGCGAA

>M.03.0.50_A_79

AATCAGGGGATGATACTAGTTTTCACATACAACGGACAGTCGGT

>M.03.0.50_A_80

GCTTTCTGCCGAACTTTTCAGCAGTCATTATAGCTCCGT

>M.03.0.50_A_81

GACTGGGGAGGATTATCAAGTATGAGGATGAAAGTCTTAT

>M.03.0.50_A_82

TCTAATAATACTAATAATAATCAACAAACTCAGACTACAT

>M.03.0.50_A_83

TAATATTGTCCCTATAAAAATTCCCGCTAACATGTATACT

>M.03.0.50_A_84

CCTTGGCTTGGGTTAGGATAATACCCGCCGTAGGGCAAAG

>M.03.0.50_A_85

TGTTTCTAAGTTTTGCGACGCTTTCCCTAAGTTTGT

>M.03.0.50_A_86

ATTAGAATCTATGAAGTTGAAGACGGAATAAGGGTCAAAA

>M.03.0.50_A_87

TGGAAACCGCTCACGTAAACACTTATAAATCCATTCTCTTGT

>M.03.0.50_A_88

TGTGGCAACACAAATATAGCTTCACAGATAACATCCCTTTGT

>M.03.0.50_A_89

ACAGATGTAATAGATGTTTCATGGCAATCCCTCTTAGCAG

>M.03.0.50_A_90

GAAGGCAGTGCAATCTTATATGTGTTCCCTTTTTCTTTTGGT

>M.03.0.50_A_91

GACTCACTTATGATTCATGCATGAGTAAAGTTTAAATATTT

>M.03.0.50_A_92

AACGATGCTACCGCATTTGTTAATAAATTTCCTAAGTTTTGT

>M.03.0.50_A_93

ATCTTTGAAAGATAATTAATAAGTAGCTCTAGATCCTTT

>M.03.0.50_A_94

TTCAATGTTAAAACTGGAATAAATGGTGTTGAATATCCT

>M.03.0.50_A_95

ATACCTTTTTAACGTCGGATACCATAATCATTAATCCCGC

>M.03.0.50_A_96

TTTATTATTGAGTTGTTTCAAGATGTTGGTATCATAG

>M.03.0.50_A_97

AATGATTGAACTACTAAAGTCAGTGGCGGAACTGCAGGCT

>M.03.0.50_A_98

GTAATACTCAAAGTGTTTATATAACACGTGAGACGCAA

>M.03.0.50_A_99

ACTAATGCACATTTCTACTTTCTATTTTATGGAGATAA

>M.03.0.50_A_100

TTGCATTGGAACAACAAACTTTATAGTTTTTACTTGCCCA

>M.03.0.50_A_101

AAGACTGCTACGTTAGTATATCTCCCTACGGGGGGATC

>M.03.0.50_A_102

AGTGTTAGTATAAATCGCGAACAAAATAAGCGGATAAAGAGT

>M.03.0.50_A_103

ATCCTTAAAGTTAGTCTGTATCAATAGATTGTTAATATCTT

>M.03.0.50_A_104

TAATAATCTGTAACGCTTACAGTTATCTTAACTGGA

>M.03.0.50_A_105

AATTTTTTAGCCTCTTCATCCAACTTTTCTACCTCATCTTTT

>M.03.0.50_A_106

TTTTAAACAAAATAAACTGTCCATCCTTCCTCACATTTCTT

>M.03.0.50_A_107

TAACGATATAGATCCAGTAGCTTTGGCTGCAGTCCTCTTTTT

>M.03.0.50_A_108

AAGATAACACATTTATTTTCTACGTAGTACTTACATACT

>M.03.0.50_A_109

AAAGATCAATTAAAGGAGTTCTTTGAGTGGATAGAGAAAG

>M.03.0.50_A_110

TGCCCGATAGGGGCCCTCTATCGAGCGGGGCCTCATTCTCA

>M.03.0.50_A_111

AAAGTTGAAGAGCAAAAAGAAAAACCGAAAGAGGAAGAAG

>M.03.0.50_A_112

AAAATAACTGACGTATAACTTAGTGTCGCAGTATAAGTCTT

>M.03.0.50_A_113

GTGTATAAACTCACTAAATAAATTAGGTGTAAACCCAGAT

>M.03.0.50_A_114

AAATTACTAACCTTACTATCAATTTTTTTAAGTAAATCTT

>M.03.0.50_A_115

TTTGAATCGATAACCATAGGTTGTAAATTACCGTCCTCATC

>M.03.0.50_A_116

TACCAATTCGCAAAATCTTTATGAATACTTTTTATAA

>M.03.0.50_A_117

ATTGACATATCAGTTGCATAGCTTGGATCACTATATAA

>M.03.0.50_A_118

TACATGGGCTACAACCCATACGCTTACGGATTAGGACAAT

>M.03.0.50_A_119

TGACGGAGGGAGGTGGATAATCACTGCCGTCCTATACG

>M.03.0.50_A_120

CTCGATGTTGACTTCAGCGAAAGTGATCTTGAAGAAAT

>M.03.0.50_A_121

ATACCATCAGCTAAATATAATTCGTTGCAGTAATCAAG

>M.03.0.50_A_122

GTTACGGGTACTGTTTGTGTATAATATGAGGGGTAACTCATGT

>M.03.0.50_A_123

ACGATAGGTGCACCTATTCCTACTCCTAATAGTCCTCCTA

>M.03.0.50_A_124

CAGCACGCTGGAAGCTAGGGCGTCAGCCTAAATACA

>M.03.0.50_A_125

AAAGTATAACTAAGTTTTATCCACTTTACTTTCTCTTCTCCC

>M.03.0.50_A_126

ATTTTTACATTATTTTCTTCTTCTGCACCCCCCTCTAA

>M.03.0.50_A_127

CTAACGTCAAGAGGTCTAAGTAGTGTTTCGTTAGGATAT

>M.03.0.50_A_128

CCTACCGTGACTGCGACATAATGGGGTATTATTGCATAAT

>M.03.0.50_A_129

ATTATATTATCAAGACCTTTAGGTGTTGTGTATGAGATA

>M.03.0.50_A_130

ATTGACTACATTCAAGCCACCAACGGTAACGATAAATTTACGT

>M.03.0.50_A_131

TTGCGTAATTCCCTAACGTCGTGTCACTGAGTAACATTT

>M.03.0.50_A_132

CTTATTCCCGAATGATTTCTTCCTCCTATAGATTTCCTCAA

>M.03.0.50_A_133

CTTATTGCATAAATCTTTGATGAGTTTGAAACGACATGGT

>M.03.0.50_A_134

CTAATAGGCAATGTTTCGGGAAGAAGATTCGCACATATAG

>M.03.0.50_A_135

ATAGTCTGTCTCACCTTGATTATTGCTTTCTCCTTCAT

>M.03.0.50_A_136

CTTTATTGAGCGTTTTTTAAGGTTGAATAAAAGATGAAAA

>M.03.0.50_A_137

GAAAGTTTGAAAGTTAAGAAGACTAAAGCAGATTTGAAAAT

>M.03.0.50_A_138

CGTTGAGACTATGGTGGTAATAACACCTCTTGTACACTC

>M.03.0.50_A_139

TTAAATGATCCAATAGGATTTGTAATTGTTGTAGAACCAT

>M.03.0.50_A_140

CTTTCCAGTAACAGTTTAAATAACAGCTCTACCCTAGTCCC

>M.03.0.50_A_141

CTACCTAATAGTTCTACTGTATATTATATGAGTTCTTTTGC

>M.03.0.50_A_142

CTCTATTTCTTCTTCGAATTTCCTTAGTGTGTAATCTTCA

>M.03.0.50_A_143

GCAAGGTCACTGACCCCAACCCTGATAAAAATTATTGACTT

>M.03.0.50_A_144

GAAGAGATTATAGCAAAATTCGGAAGGCTAAAGGCTAAC

>M.03.0.50_A_145

ACGAGAGTGTTAGGATCTTCCCATCCTACCCCTAACGGG

>M.03.0.50_A_146

CGCCGTCGCCGACGCGGGGGTATGTAGTCAGTATTACT

>M.03.1.5_A_1

TCATATTCTTCTTTGTCATCAGATACCATGTCACCGCAAA

>M.03.1.5_A_2

TATCTTGTCACCGTAACATTTGTTATCTCTCGATAGTAT

>M.03.1.5_A_3

AAGAGGTGGTATGTCACATGGGATTAGCTATGTATAAAT

>M.03.1.5_A_4

TGAAAGTGTGTATCACACCACACTACTACATTCTCATCTA

>M.03.1.5_A_5

TCAACATCGACATAATATACTATAACTTCCGCATTATCAA

>M.03.1.5_A_6

GTTACAACAAACTATTAAGCGGCATTGCTCCTTCTGATG

>M.03.1.5_A_7

ATATTGGTAGACAAACAAAGTATTACTGAAAATTCAT

>M.03.1.5_A_8

ACAGATGTAGGAGTCGTAGGAATTGTAGGTATGCTAGGG

>M.03.1.5_A_9

TATCCCGTACGTCTGTACTCAAATTTTATTACCTTTCT

>M.03.1.5_A_10

TTACCTTTACGGATATGAAATTGATGATATCGCTGA

>M.03.1.5_A_11

TCTACTACTAGGACGTCTTTCTCATCGTAACCAATGACT

>M.03.1.5_A_12

AATGAAACTTTCATAATAACTGAGATCGACCCATTACCAG

>M.03.1.5_A_13

AAAAAGGAATGGAAAGAGCAGGAAATGAAACTATATG

>M.03.1.5_A_14

GAAACCGCAGTAATATTAACTGCTGCAGGATAGGGAAGC

>M.03.1.5_A_15

TATACGTCAGCTATAACTATAGCATATGGTTATGCACCAG

>M.03.1.5_A_16

GGTATGCTATACGTCATGCTTAACCTACCTTCTGGTGTTACC

>M.03.1.5_A_17

TTAGTAATGATTCTCTATACTCACTTTTATCTAATTTACTTC

>M.03.1.5_A_18

GGTTTTCACAAGCACTTGAAGCGTTAGCAAGTTCAATTGT

>M.03.1.5_A_19

AAATGAGCGTACGCCCACACCGGAAGTCTTCCGCTTAGT

>M.03.1.5_A_20

TTGTTTTGACAACTTCATTTCCGCTCACGGAAACCTT

>M.03.1.5_A_21

ATCACAGTTTTTCCGCTTGTCGCACTGAATTCTCTAACTTGA

>M.03.1.5_A_22

CTAATGCTCCCGCTAGTCCCGGGGCTATACGATATACTTAGCC

>M.03.1.5_A_23

GTTATATGGCCTGAAGTGGGTGTTAAGTACGACGATGTAAC

>M.03.1.5_A_24

TTAAAAGAAGCAGTAAAGGAAATAGCAAGATCAGCTAAT

>M.03.1.5_A_25

GCAGGTTATGGCAATAATGGTATTGTTTCTATCACAGTAAA

>M.03.1.5_A_26

TTTATAGCTAGAGCAATAAGCAGCAATCGGATGGGTAG

>M.03.1.5_A_27

AAAATATCTTACTTGAGGTTTAGAAGAGAGAATGGTGAAG

>M.03.1.5_A_28

TAATGCCTATTCTCATGAAGTACTTCTTTCTGTGCTAC

>M.03.1.5_A_29

TTTCCATCGTTTGTTTGCCCTGCAGCTAAAGTCACAACA

>M.03.1.5_A_30

CTTACTGACGCTAATGCCTCTAAAGCCTCAGGTGTTACT

>M.03.1.5_A_31

TCCATGAACTGCCTGAGCTGACGAATTGCTACTATCAAAG

>M.03.1.5_A_32

AATATTAATTTAAGTATACTGCAATGTATCCATTCTGAAGA

>M.03.1.5_A_33

AATTCGTTAGAAAACGGGTTTTCACAAGCACTTGAAGCA

>M.03.1.5_A_34

TCGTAACCCTGATTTGGTACCAACCCTTTTCCCTCAG

>M.03.1.5_A_35

TTCAACAATGCATGGGTTATGGAAGGTACAGTAACCA

>M.03.1.5_A_36

TGATATCCATCTTTTCACTCTTAATTTAACTAACTCATT

>M.03.1.5_A_37

AATAATTACGGTGGACTTTTACCAAGTGGAAGGGAAAAC

>M.03.1.5_A_38

TCTAAGTTCAAGAATTGGCTTAAGGCTTTGGTGAGCCGGA

>M.03.1.5_A_39

ATTAGATAATTATAAACTTCTTTTTGTTTATATTGAA

>M.03.1.5_A_40

ATCATTTAGTTACGCTTTTAACTGACGTTAAGGCGATT

>M.03.1.5_A_41

ATCTAACTATGATAAACTGGATGTAGAAATATTATTTAGA

>M.03.1.5_A_42

AGATAAAAAAGCTGAGGTACGTATGCCCACACCCTGGCTT

>M.03.1.5_A_43

CAACTACTACACTATAAATTACATTAGATGGATTATTTA

>M.03.1.5_A_44

TCTTTCTCTGAGGGATACATCGGCGATATCTTCAATAA

>M.03.1.5_A_45

AATTTACTATTCTCTACTAAAGTGTCTTGTACTACTAA

>M.03.1.5_A_46

GCCACTGTGCTTGTGTTTGACGTTGCAAATGCACTACCTA

>M.03.1.5_A_47

ACTAAGTATCAGCAATGCTCAGCTGAGAATTTATTACCA

>M.03.1.5_A_48

TCTTTTACTAAGAAGCTTAAAAATATCGATCCATATATT

>M.03.1.5_A_49

TGCCTTCCGCTTCTACTTGTGTGTAATCTTGATTTGATA

>M.03.1.5_A_50

TATTTAATACAATACCCTTTTCACGCATTTGCCCACCACT

>M.03.1.5_A_51

GCAATAATACCGCAACAAATTCCAGACCCACCTACTTATAC

>M.03.1.5_A_52

CCACCACCGCCGCCACTACTACCACCACTGCCAGCACTGC

>M.03.1.5_A_53

TCATATTTCACGCTAGGACTTGATAATAATAATGAATTTGC

>M.03.1.5_A_54

GAGGGAGTATAGTTGAATCGAAGTATGACGTACCAATCCCAT

>M.03.1.5_A_55

TAATCCACATAAGCCCTCTTTCACTCCCGTACTTTGTCG

>M.03.1.5_A_56

ATATCGGTATTGACATGTTCACGCCTTTTATGAAGCCTGA

>M.03.1.5_A_57

CTGAAAATGGCATTGTTATAACCTTTACTTCGTAATGTAC

>M.03.1.5_A_58

CTCTCGTCTAAGCGTCCTGCATTCTCTACTAGCTCGTTAAG

>M.03.1.5_A_59

ATCGAATTTCCATCTCATCAGGAACCTGCACAAGTTGCCG

>M.03.1.5_A_60

ACCGGCGAAGTACTTAGTATATCAAATATATAGAAGTAACTT

>M.03.1.5_A_61

ACGCTCTCATCTTGCTCAAATTTATTCTCTTCTGGTAGT

>M.03.1.5_A_62

TCATGAAATTTATTATTAACAACAAGATATAATTATTTA

>M.03.1.5_A_63

AATAACAAACTTAGGGAAAGCGTCGCAAAACTTAGAAA

>M.03.1.5_A_64

AGAGGAGTGTCGGGATCACAAGACAACATAAACAGGATGGT

>M.03.1.5_A_65

TTTATAAAGCCACTTAAATAACTTATCTTTAGTATGATGTT

>M.03.1.5_A_66

GAACTGAACCATACTGTACCGTTTTCGGTGTTTTGTAT

>M.03.1.5_A_67

TAGGTTTTTCGATTTTAAGAGGCTCTTCTACTTGCTCTA

>M.03.1.5_A_68

CAACTAAATAAATAGAAATTTCACTAACCAACACATAACT

>M.03.1.5_A_69

CCAACACTAGCACCAGCACCTCCATATGTCCATGCTATC

>M.03.1.5_A_70

CCAGAATGACGTGTTAGGATTTACATTGTAAGATATACT

>M.03.1.5_A_71

CGATAGGGTAATGGGAAAGGCGGTGTAAATATGGTTTGATAG

>M.03.1.5_A_72

ATTATAGCTATGCCTATTTCAAAATTTTTTGCATCAA

>M.03.1.5_A_73

TGTCCGCTTGTGTGTGTCATTTGCAATTCCTCATAATA

>M.03.1.5_A_74

TTCTCTTGTTCTTTCGCGATCATATAAAAGTACTAAAGG

>M.03.1.5_A_75

TTTATCAGATCAATGCTTGGCAATTTCACTGGTAGTCCA

>M.03.1.5_A_76

CCAAAAAAGTAAAAGTTAGAACATTAAAATATGAAGCA

>M.03.1.5_A_77

GAAAAAGTAATAGAGGGCATCTTTGAATTAGGCTTATTT

>M.03.1.5_A_78

TGCCTTAAATTGTCCTTTTTGTTTGTGCAATGCAGTTAC

>M.03.1.5_A_79

ATCTTTATCAACCTCGGTGCGACATTAGAAAAATAATAA

>M.03.1.5_A_80

TTAGCTATACCTGTTAGTTCATGATGGGATTTTCTACCA

>M.03.1.5_A_81

AATTTCTCATGATGAAGTCATTTCTATCTATAATATTCTCTC

>M.03.1.5_A_82

AATATTGAAATCGATGACCAAAAGCAGTGTATCAAACTTG

>M.03.1.5_A_83

TTAATTACTATTGCTATCTTGCTATATAATGCTTTTTTCGC

>M.03.1.5_A_84

CTGTCCCCCTCATCGACGGGGGCTGTGAATTGTATT

>M.03.1.5_A_85

ACAACGTGGACATTGATATAATAACGCTCCAGTGCTTTCAT

>M.03.1.5_A_86

TCGCCAAAGTTTCCTAATGAGGAAGAACCCGGTAAGACCTA

>M.03.1.5_A_87

GTATCAGTAGAGCATATATTAGCTAACATTTCCACATTAT

>M.03.1.5_A_88

ATATATGCTGTAATCTATAGACTAGGATATACTTTTGTT

>M.03.1.5_A_89

TACTAGAATTAGTTCCTCATTACTCTTAGAAGACTTCTC

>M.03.1.5_A_90

TTGCTTATGATATCAGGATAGAATGATGCCATTATAGCT

>M.03.1.5_A_91

TGTCCTCTTCTATGGGCATGGACTTAGGATTAGGGTTTTTA

>M.03.1.5_A_92

TAATTTAACTAATAACGCGGGGGTTTAAAAATTTAACGGT

>M.03.1.5_A_93

TCTAAATTTTGTTCTATGTCGGAAGAAGTATAAATGCTAT

>M.03.1.5_A_94

AACAAAACGCTAGATTGTAGAGTTATATTAGTAATAGCG

>M.03.1.5_A_95

AGAAATAAATTGATAGATCTTTTTATAACGGGAGCTCCGAG

>M.03.1.5_A_96

TCGAAGATATTGCACGTCACATTCATTATCCCGGCAAGTTCT

>M.03.1.5_A_97

TTGTAACGGTATGTGCGTTTGATGGGATTGTAGTCGTAAT

>M.03.1.5_A_98

CAAAACGGGAATGGGAGGAAGGTCAGACACCATCTTAATA

>M.03.1.5_A_99

TAATATCATACATGGACCTTCGCACGAGAAGTATTCAA

>M.03.1.5_A_100

TTAGTTTGAACAAGTTGGACATAACGTCCGTAGAACCCTA

>M.03.1.5_A_101

TATCTTCTAATACCTTATTCTTCTCTTCCAGTTGCTGTAT

>M.03.1.5_A_102

TCTTGTATGATAATCCATTCCTCAGTTCTATCTTTAGTAT

>M.03.2.5_A_1

TAAAGATAAGGGAAGATATTTTTTTGAAAGGTAAACTT

>M.03.2.5_A_2

CTGGAACTTTAACAGTTGTCCCGTTAGGCAATATATAGC

>M.03.2.5_A_3

AAGTCCTTTCTTCCCTTATTATTATTAATCCATTCTCTT

>M.03.2.5_A_4

CATTATCCGCTGCATTTGTCAATATTGCTGCAAACGG

>M.03.2.5_A_5

TTATGGTCATAACATAACGCACTGTATATCTCATCGTTATCC

>M.03.2.5_A_6

AGTACTTGACTAACCTTTTTTGCCTTGCATTGAATTTTTTG

>M.03.2.5_A_7

CTGGTTGTTTCAATCTGAATGTAACTACTTTGTTCCCCT

>M.03.2.5_A_8

GTTTATCGCTCTGTTTTCTAGTCATTTCTCTAAAACTTCT

>M.03.2.5_A_9

CAAACACAGCGATAGCAGGTGATAATAATACGACTACTG

>M.03.2.5_A_10

TGTATATAGTTTGGTGTATAGTTATTACCACTGTTGTCC

>M.03.2.5_A_11

CCTGCAGTTCCAGTTGCAAGAGTCTCTTCCACTACAACA

>M.03.2.5_A_12

AAGAAGTGGTACGTGACGTGGGATTGAAAATGTACAAGTGT

>M.03.2.5_A_13

CTACAATTAAAATCTCTTTTCCATTATATTTATCTATAA

>M.03.2.5_A_14

ACTTCTCGGCGAAGACAGCAGCAGATTCTTCGACTTTTAT

>M.03.2.5_A_15

CAGGGTGTTGCTGCGGCGCAGCCCAAGATGGCTGCAGTTATG

>M.03.2.5_A_16

ATTCGCTACTCCCGAAGCTTGGGTTCCTGTTGCACCTGCACCT

>M.03.2.5_A_17

TATAGCCACTGTCCCTAGAGGAATCTTAACAATTATATCCTTA

>M.03.2.5_A_18

AAATACTTTAGGAAGTTTGTCGCTAGTAAACTAGCGGA

>M.03.2.5_A_19

ACGATTATCATTGACAGTGGAAACGGTATTCATGTTTAT

>M.03.2.5_A_20

ACATCAAAGAAAAGATATCCTAAACCTATCGCATAT

>M.03.2.5_A_21

ATGCATAAACTCACTGAATAAATTAGGCGTAAATCCTGA

>M.03.2.5_A_22

TTGAATGCTTGCCAATTCGCAACGCCGCTAATCTGTGT

>M.03.2.5_A_23

GAACTTCACTACAATAGGCCATGCTAAATTCGGAAATATT

>M.03.2.5_A_24

AGTTTTAAAGTCCAAGGTTTATCACTATTTAGTATGTAA

>M.03.2.5_A_25

ACTATATAAATTTGAAAACGGGAAAGAGAGAGAAAGATTTAT

>M.03.2.5_A_26

AACGAAATAACATTCACACAAACTTCCGCAGAAGTGC

>M.03.2.5_A_27

AGAGGGAGTTGAAGAAGCTCCTGGAGAGCCTGGAGGACGA

>M.03.2.5_A_28

ACACTAGCACCAGCACCTCCATATGTCCATGCTATCTGTCC

>M.03.2.5_A_29

CTAATCCGTGTATCACCTGCAGGGTTTGGTGTCTCCCTGC

>M.03.2.5_A_30

ATATACGAAGAAATATACATAACTATCTTGATACTCATGG

>M.03.2.5_A_31

TCTACCGGAAATAATTCACTGCTTCTCTCTATCTATAAG

>M.03.2.5_A_32

GAGCTGGGCATAAGCGGATATAGGATTAACGTAAAC

>M.03.2.5_A_33

GATACACAAATACATGATATCGTTGACGTTTTATTAGTTTC

>M.03.2.5_A_34

CTTCTTTCGAATAACTGCAGAATTCATAACCCTTCTCTG

>M.03.2.5_A_35

GACGTCGTTAATTAGTTTATGAGTTACCTTAATTCGTTA

>M.03.2.5_A_36

CCTTCTGCGTTAATTGAAGGACTATTAACCGTTTGGCCT

>M.03.2.5_A_37

TAGAAATACGCTGTGACGCTTCCAAATGTTCCAGGCGGTAA

>M.03.2.5_A_38

TTTTCTACAAACAAACCACTTCCGTTTACGACTACATGTA

>M.03.2.5_A_39

AATGTAGATGAGGACGGTAATTTACAACCTATGGTAATA

>M.03.2.5_A_40

ATTGACATATCAGTTGCATAGCTTGGATCACTGTATAAT

>M.03.2.5_A_41

AACCCAAGAAAAGGAATTTTTAGTTTTTTCTCATGATACA

>M.03.2.5_A_42

TTATAAAGACCGCACAACTCCTCATCGTCATACTGCTT

>M.03.2.5_A_43

AATCCACATGCATTTTATAAACCATAGCAACTGCATTTA

>M.03.2.5_A_44

GCTACCATTAACCCTACTGACCATTGATAACCGTAGTCATAT

>M.03.2.5_A_45

AGTCTTGCGTCAATACTGATTTCATGAATCAAACTCA

>M.03.2.5_A_46

TTTGCTATTAGAATTAATGAAAAATACTACAAACTGTACA

>M.03.2.5_A_47

TAGCAACTGCATTTATTAACATATTCAAGAATTCTAAAT

>M.03.2.5_A_48

TAAAGTAAAGTTTGCAGATGTCGAAGGTGCTTGGCTGAA

>M.03.2.5_A_49

CTCTATGTGAGATATAGATACGATCCCAAGAACGACCT

>M.03.2.5_A_50

AATGAATGAGAAGCATTTAGCATAACATATTCCGCTCTAAT

>M.03.2.5_A_51

TGCTATAATTTACGGCTCAACTGGGTCAGGGAAATCAAA

>M.03.2.5_A_52

TTTTTTTAATATAATTCAAACACCTCAAGTGCAAGAGGT

>M.03.2.5_A_53

CCCAAAGAAGCACCTTTAGTACTTTTATATGATCGCGAAA

>M.03.2.5_A_54

TTGATTTCCGTGTATCGTTATTATTCTGAATTCCTCGCC

>M.03.2.5_A_55

AGTTTGTTGAGTGAGTTTATGCACTGATCTACTGTAAGT

>M.03.2.5_A_56

ATCTTCTTATTGCAACCGATCCCAATGGAATTTTTACAAT

>M.03.2.5_A_57

TTCCTTGGGCGATTTAGAAATAATGGATCAGTTAAGAG

>M.03.2.5_A_58

TTTTTCATGCATTCTTCTATTTTATCAGATTGCAATAA

>M.03.2.5_A_59

TTGTCGCTATGTTTGATAAGTACAACACATATAGTATA

>M.03.2.5_A_60

AATAATAGTTATAGTCGCCTTATCTGGAGGGAAGAAACA

>M.03.2.5_A_61

GAAGACGTATATACAATGCTTACCTCCAGATATTTCTTTGT

>M.03.2.5_A_62

TTAAAACTATACGTCCTTTTAATTCCCTTCTCATACTCAT

>M.03.2.5_A_63

ACACTCCTAGATTTTGTAGTGCTTCTTGCAGCAGTAGAG

>M.03.2.5_A_64

TATAATTAAGCATTGATTTATACATGAGCCTAGTTTTAG

>M.03.2.5_A_65

ATTTAAAGAAAAATAACTAGCATTGAGATTCAATCCGTCCGT

>M.03.2.5_A_66

ATTGTAATTGCCAGTTGCTAAATTACAACACGAACCAG

>M.03.2.5_A_67

TATTCTGTATTAGCCACATAACCACCGCTTCTTCTAT

>M.03.2.5_A_68

CTTATTCCTACATAACCTATTTGAGACCATGGAAACGG

>M.03.2.5_A_69

AATTCTTTCAATCTTTCTCTAATAGCCTCATTTACAA

>M.03.2.5_A_70

TTTGTTAATTTCCTCAATTTTTGGTTGAAGTTCTTTCGGC

>M.03.2.5_A_71

AATAGTGTGATGAGAAATGAATAAGCTAAAAGCACTGAA

>M.03.2.5_A_72

ACTACCTCAATGGCGTTGTTTATACTGTAGGTGAAAAC

>M.03.2.5_A_73

GAATATTCACTCATATATAATAGAGTTTTGCCCATTACA

>M.03.2.5_A_74

ACAGAAATTGAACCTAGCTGCGATTCAAGTTCAATAGTACC

>M.03.2.5_A_75

GTTAATGTGCCTCGATATAATTCAAGAAATTGAGTAGT

>M.03.2.5_A_76

AGTAATCGGTTTTAAAGTAGACGATAAATTAAAAAAGTT

>M.03.2.5_A_77

AAGATATTTTTTTGAAAGGAAAACTTCTCGCATCTACGCA

>M.03.2.5_A_78

CCCAAACCCACGGCGCCAGCGCCTAATCCCGCTAATGCCG

>M.03.2.5_A_79

TGCGGTGGACTGATAGAAAATGGAAGGGGATCAGTTCCA

>M.03.2.5_A_80

GATATTGCAATATCTAAACGCTGAGAAAAACGCTCTACA

>M.03.2.5_A_81

CATAAGAAAGTAGGCTTAAAAAATCATCTCTTTTATTAAGAA

>M.03.2.5_A_82

TATATTGTGTATAAGCGATAACAATTCCCTAACGTC

>M.03.2.5_A_83

TTACTTTCGGATACGTCGGCCCAGTAAACGTCACCGTCTC

>M.03.2.5_A_84

TGCATTCTACGAACTTATTTTTTCTCCTCTCTCCTGTG

>M.03.2.5_A_85

GCGACGCTATACTTAAACGCCACATCAACCTTAGTCTTAAG

>M.03.2.5_A_86

ATAATTAAATTTCATAGATTTTAAACCATTAACCTTAAACTC

>M.03.2.5_A_87

AAGCAGCCCAAGATTATTAGTTAGAGGATTAACAGCGAGT

>M.03.2.5_A_88

TAACTCGATTGATGCCGTCCAGTAGATATTCCCGGGCC

>M.03.2.5_A_89

GTAACGGAAAGGAGGGAGAGGAATCTACTCAACAAAACGAGA

>M.03.2.5_A_90

TTCTGTTTATATTATCTTGTGATCCCGCTACTCCCCTAG

>M.03.2.5_A_91

AATCTATTCTGACGCCTCTATCTCGCATCTGAAATAGTTT

>M.03.2.5_A_92

AAATCATGTTCTGATGGTATAAAACGGTTTTCACGAA

>M.03.2.5_A_93

AACTTCTCCCTCTTCTCCTTTGACTTGTAATACTAAT

>M.03.2.5_A_94

TTATTAAGGAGCCTTGTTTAACTGCTTCAATAAAAGAATA

>M.03.2.5_A_95

ATTACGACTGGTTTAGGGTTTTTCCCCTCAAGATGCCCT

>M.03.2.5_A_96

TAACAAACAATAGAGCTAAAACTTGTTCTTTAAAGCT

>M.03.2.5_A_97

TCTTATCTCCTCAAAACCTAGCGATGTTAGCTACAACAGC

>M.03.2.5_A_98

ACGTCATCGCCTAGAGCATTATAGACCATTATCACTCCA

>M.03.2.5_A_99

CTAACTAACGGCCCTCCTGAATCCCCCTCATCGACGGGGGC

>M.03.2.5_A_100

GGATCTATTACAATTAATGATAACTCAAATGTAATTTCT

>M.03.2.5_A_101

TGGATTCGCAGAGGTTCCCGCAGGGTGTGAGCGCTGCGC

>M.03.2.5_A_102

CTTTCTGGTCTGTTATCAGGTCTAATGAGTATTTGGCCG

>M.03.2.5_A_103

TTTATTTTGCGATATCTTATGGAGACTGGTCTTGTGACT

>M.03.2.5_A_104

ACTTATCTTTCTACACTTTCAGAGAACATAGTTTCTTTAT

>M.03.2.5_A_105

GTCGAATTTCCATCTCATCAGGTACCAACGCAAACTGCA

>M.03.2.5_A_106

TCGTTTGTTTGACCAGCACTGAGATTAACAACAAATCTG

>M.03.2.5_A_107

TTATAAAAGCAATCAAAAAGAATGAAACTACGTCATTCA

>M.03.2.5_A_108

AAGCTACTATGAGAGAAGAAATATTTAATTCAGTAA

>M.03.2.5_A_109

TGGCATTAGTTTATATTGTAACATGTCCTTAACGTTGA

>M.03.2.5_A_110

TACGGGCTATATATTATTCAACCATATGGCGGGCCCCT

>M.03.2.5_A_111

CCGTTCAATTTTTATGTTACAAAAGGAAGTAAATTTTATTT

>M.03.2.5_A_112

TTACAAACTTTGTGATAAAGCCCTTAGTTTACGCACTTTT

>M.03.2.5_A_113

AAACCATTAACAAAGTTTGAGGAGTATGGGCTAAAACCCG

>M.03.2.5_A_114

GTAGTGGGTATACTTTTAGGGAATGAGTAGTACCATT

>M.03.2.5_A_115

AGGTTTGTAAGTAGAGAAATCAGCCCTTGATTAACACT

>M.03.2.5_A_116

TTTTGTAAGAGGGAATTTGAAACATTTGTAGGTTTAAGGA

>M.03.2.5_A_117

TAGACGTAGTATTAGTTATATGAGCGGGGCTAACGCTT

>M.03.2.5_A_118

TTTCTCTATAGCATCCTCCAAAGCACTTCTCAAAGATGAC

>M.03.2.5_A_119

GCGATTATTACACACCTTTACAACAGAGGGTATACTATAT

>M.03.2.5_A_120

TTCTATAACAAATTTTCGCTTTGATTCTGCGTGTTGCT

>M.03.2.5_A_121

ATATCTTTTCATCCTGCTCATCGAAGACATCTTAACGA

>M.03.2.5_A_122

TCTAGTATTACTTTGTACGAAAACGGGGCTGCAAACATTAG

>M.03.2.5_A_123

TTTCAATTCTATAGTAGATTATCTCTGAATATATCTACTAG

>M.03.2.5_A_124

AGCTTCCAGCGTGTTAAGGCGTGACGCACTTAACGGGTTT

>M.03.2.5_A_125

TTTTTAACATATCTTTTTCACTTTCGCTCAATTTAATT

>M.03.2.5_A_126

ATCGCATTAGCTGCAGTCCTCTTCTTTACCATAACGAT

>M.03.2.5_A_127

AACACTTAGCAGTCCTTCTATTAGATCAGAAGGCGGTAAT

>M.03.2.5_A_128

GTTTTCGACGGGGTAGATGTCTTTCCATAACCTGCCAT

>M.03.2.5_A_129

TCCACTTTCAGTGCAATATTGACAGAAAATTCCGCCGGAAA

>M.03.2.5_A_130

ATCACGTCAGCTTCACAGCTCGTGTCCGTTTACAACG

>M.03.2.5_A_131

ATAATGAAATAATACATTTGTTTGTAAATTTAGCGGATTATT

>M.03.2.5_A_132

GCTTTTGATGTTTTTATATTAAATACTATTCCTAGTTGT

>M.03.2.5_A_133

ATGTTTAAATTTAGTGTTGGTAGTGTAAAGTTTGAAGG

>M.03.2.5_A_134

ACAAGCTTAGCTGCATACGATAGAAATCCAGTTTGTTGCAG

>M.03.2.5_A_135

ATAATACGTGCCGATAGTTTTGTAAAGCATATATAGTGAG

>M.03.2.5_A_136

GCGACTCCTCTAGGTGGCAAGGTCTTTCTAATTTGATCTAT

>M.03.2.5_A_137

CTTGCTATTGCTTCTGCTACTAGTTCCCGTGTACCTTGA

>M.03.2.5_A_138

TTATTGTCATAACCTGCCACTGTGCTTGTGTTTGACGT

>M.03.2.5_A_139

CTTTATTCTGTTTGCCTTATCTCTGAATATATCTACTAG

>M.03.2.5_A_140

TGTCCACGTGTTTAACGCTACTCTCATCTACGATGAACT

>M.03.2.5_A_141

TCTGATTTTTTAAGACGAAATTTACACAAAAGAATACAAG

>M.03.2.5_A_142

AATCCTATCGCTAGCTTATGCCTTCTCAAGAAATCAGCA

>M.03.2.5_A_143

ACTATACTCTGACATGTACAACAGAGTTTTACCCATTA

>M.03.2.5_A_144

ATACTTGAACGAGGCTATGAATGATGCGAATAACGGT

>M.03.2.5_A_145

TAATTCCAGTTGGCGTTACGGTGAGTTCTAGTACACC

>M.03.2.5_A_146

GGTTTTCTACAAACAAACCACTTCCGTTTACGACTACATGTA

>M.03.2.5_A_147

ATATTCCTTTTCCTTCATCATAATAATAAATTATGAGATCAT

>M.03.2.5_A_148

CCTCCAGCTAAGACCGAAAGGACTAGGAATACCGATT

>M.03.2.5_A_149

AATTATATAAAAATAGCGATCGCGTTCGGAGCTGCGGCAT

>M.03.2.5_A_150

ATACATATGCATATTGTTTCAATGATGATTCTTTATCC

>M.03.2.5_A_151

ACGGCACAGGTAATCTTAATAGATATACGATAGGATTAGGG

>M.03.2.5_A_152

TATTAACATAACGCCACCTAAAGAGATAACAACATCTAT

>M.03.2.5_A_153

GCTACGCTAACACTATCCGTTATACTGTACATGATTCAGT

>M.03.2.5_A_154

AATATAGACAAGAAGGCATTTTTTAACGCTGAGGTGGT

>M.03.2.5_A_155

GGTATAACACTGCCAACGCCAGCACCGACTATTGCACCAG

>M.03.2.5_A_156

ATAACTGCGTATCCACTATCGCCATTCGTAGTCAGAAAT

>M.03.2.5_A_157

TTTACTTTCAAAAATTTGTTCTTGACAGGTCAGAAAA

>M.03.2.5_A_158

CCTACCGGCGACGGCGGGGTAGAAAAATATAAGTCTCCCC

>M.03.2.5_A_159

TATACGTTCCAACCCAACCATTTAGTTACGCTTTTAACTGACGT

>M.03.2.5_A_160

TGAAAACGCAGTTACAGCTATTGTAGTAGAGAATAAACTA

>M.03.2.5_A_161

TTTATCTCAAGAACTATAAAGTAGTAACATATAAAGA

>M.03.2.5_A_162

AATCATTCATAAAAGGACTTATGCAAAATTGGAAAAGCAC

>M.03.2.5_A_163

CTGCAATCTTAGATTACATTAATAATCTCTTAAAAGAAAA

>M.03.2.5_A_164

ATTAGATATATATATATAATGTACTTCCAGTGCCCGTGATCG

>M.03.2.5_A_165

TTAGATATTAATCTTCTTATAGCTACTGAACCCAATGGTACT

>M.03.2.5_A_166

GTAGGGACTATTACGGGAAGGAATGCTATAGAAATTGAT

>M.03.2.5_A_167

TAATTTCCAAAAAAAGTCTAATAAAATAAGCTTTAATT

>M.03.2.5_A_168

GACTGGGCCTATGATTTCATAACGCAAAAGAGAGAGATTACA

>M.04.0.10_A_1

TCAGTCGTTTCAACGGCTGTAAACATTGACTATTCAAATG

>M.04.0.10_A_2

AGTGGTGGTGAATTGTTTTCTACACCGTTATCTTGAAG

>M.04.0.10_A_3

CGTGATAATTATAAATGGGCTGAGGCTATGGGTAATAAAGTT

>M.04.0.10_A_4

GACATTTGAATTAAAGCCAGAATTGCCAACAGATTCGC

>M.04.0.10_A_5

TACTTACACCCTTGTTTTAAGTATCTATTTTTGGGTTT

>M.04.0.10_A_6

AGGTATTGAACAGGTGGCTCGTTCTCACCGGAAGGTAGTC

>M.04.0.10_A_7

AGTTTCTTGACTTCCATACGAATTAATTATCAGAGTCTA

>M.04.0.10_A_8

CTACAATAATTGCGTTGTGATGCTTTCTGCATTCAATCATGA

>M.04.0.10_A_9

ATTTTAGATCAAGTGTGGCAGCATATACAAAGTTGCCCAG

>M.04.0.10_A_10

AACCTAAGGTTAAGGCTTCTACGTGCTGTTTTAGGTCAT

>M.04.0.10_A_11

GTTATTCTATTAAATGTGCATACTTCTTGATTTGCCGCCAT

>M.04.0.10_A_12

TTTGATTCCAGAATTTCCCCCACACTTACTTTTACTCCT

>M.04.0.10_A_13

GTCAGTTGCAATTATCACAGCCGTCGTTACCGACCCCG

>M.04.0.10_A_14

GCGTCCTTTAGTATCTTTTCTTGAGCTGGTAATAGTTTCTC

>M.04.0.10_A_15

TTGTTATCTTTTTCCATACTATACTTAAACTTAGTCTTTACAAT

>M.04.0.10_A_16

TAGCAATGCAACAGCTTCATTCTCACCCTCACCGGGCGA

>M.04.0.10_A_17

TAGAAGAATGTTAATATTAGCTAAATCTGATGAAGAGAATTA

>M.04.0.10_A_18

TCAAGGGGCTATTATAGCAATATTAGGAGTAAAAGTAAGT

>M.04.0.10_A_19

ATAGCAATATGATGTACTATATTTAAATGAAAAACTGT

>M.04.0.10_A_20

TTACGAAAAGAGGGACTATTAAGAAAGAATATTTAAAGA

>M.04.0.10_A_21

AGTTTTTTTATGAATATTACATCAAAAACGCCATTTATA

>M.04.0.10_A_22

TTGAACGTTATAGTTAAATTAGTTAAAGATAAATGTATTG

>M.04.0.10_A_23

AAGTAGGTACGTATAAGTCCGCATTCACACGTTTAACC

>M.04.0.10_A_24

GTGTACATCCATCATTGCTTTTATTGAACAGATTATGTCATTA

>M.04.0.10_A_25

TTCTTAAAGAATCTCTCATAATAACATTATTCTTAACAT

>M.04.0.10_A_26

CAAAATAGAAGGCAGTTACAACAACCACAGCAAACAGAT

>M.04.0.10_A_27

GCACCCTCACTAGCCTTTATAAGATCAGTAGAAATTTTC

>M.04.0.10_A_28

CTGGACTTCAGCGACACGCTCGTGACATCGGTCATTTTA

>M.04.0.10_A_29

TCTAGTATAACTTTATACAAAAGCGGAACTGCAAATAT

>M.04.0.10_A_30

TACTTTTTCTTCACCTTTGATATTTAATTTTACTATGAT

>M.04.0.10_A_31

CTGTCATCAACCAATAAATAGACAAAAGATATCTTTTTTT

>M.04.0.10_A_32

GGGTACAGTATACACGAGCCAGGGGTTGCCGGTTCCCGGT

>M.04.0.10_A_33

CTAGTGCCAGGGTTCAGGGGTGTACCCTCACTGCCACA

>M.04.0.10_A_34

CTGATGAGACTTGAAAAACGAGGTTTACTGAAAAAAGTCGA

>M.04.0.10_A_35

TATTTTGACCATATAATGTATAGTAATTTTGTTCAAT

>M.04.0.10_A_36

GCGATAAACCGCTTAGTTTTTGCAAAACACTATTATACG

>M.04.0.10_A_37

GACTATGATATAGCAATAGTAGATTCGTATTCTAGTGATGG

>M.04.0.10_A_38

TATTTACACCGCCTTTCCCATTACCCTATCGCGGTTT

>M.04.0.10_A_39

AACTTTAACGAAGGAAGAATAGAATTCAATCAACAAGACT

>M.04.0.10_A_40

GATATTTTTTTGAAAGGAAAACTTCTCGCATCTACGC

>M.04.0.10_A_41

AAGACACTGCTTCAAACAGTTTCAACTGAGGTCTTCAAAACACCA

>M.04.0.10_A_42

AATTTTATGACTACGCCACCCATACCTTTAGCACGTAA

>M.04.0.10_A_43

GGTATTATGGCAAACCTGGTATTCCCATGTTTCTCACTCT

>M.04.0.10_A_44

TCTTATATAAAATCAAAATTGTATGAAATCCAAGCTGAAGTTTTT

>M.04.0.10_A_45

TAGAGACGGGAAAGAGGTTCAACACCCCATTACGGAATC

>M.04.0.10_A_46

TATGTAGTTTCTTAGCTTCGTTTAACCATTCCTCAG

>M.04.0.10_A_47

AAGTCGTAGCACCGTATGTACTCGAGGTACCTATAGTA

>M.04.0.10_A_48

AATGGGCTGTCGTAGACTATTCTTGCACCTGTACCAGATA

>M.04.0.10_A_49

GATTGCATTAAATTCTCTAAACATGCAGCGAAACCAGA

>M.04.0.10_A_50

ATGTTACACTATATGCAATCTTTAAGAATAATTGCTATA

>M.04.0.10_A_51

GTCCCCTGGGGTGTTGTTCGCGACAACTGCAGCAACGAG

>M.04.0.10_A_52

ATAATCACCCTAATATACCAAATGATAAAGAAGTATGCT

>M.04.0.10_A_53

GAAGTCTGCCGCTTAGTACTATGAGCTTAGAATCTATA

>M.04.0.10_A_54

TTTTTGGGGAACCTCTATCAATCAATCTATCAATCCGGG

>M.04.0.10_A_55

TGGAGTTTTAAAGACCTCTATGGAAACGTTTTGAAGGA

>M.04.0.10_A_56

TGTCTTCCGGTTCGTGATGTATGTACGTGAGATTTGATAAT

>M.04.0.10_A_57

ACTCCCACCTCGTCTCCACTACCACCCAACTGTATTCAAG

>M.04.0.10_A_58

GACTATTTCTTCTTTAAGAACTAATGTAGTATCATTTATC

>M.04.0.10_A_59

CCATATCCAGGATTTCCCTGTGGAGGCCACCAGAATG

>M.04.0.10_A_60

ACGTCAACTTGCAAAAAGTACAACTGATGAATTTGCCTTT

>M.04.0.10_A_61

GTTGGTTCTACTGTAAGCGTCCAATATTCAAATGGAACTTCT

>M.04.0.10_A_62

TTAGTACTTTTATATGATCGCGAAAGAACAAGGGAAAGA

>M.04.0.10_A_63

GTGGGGGTGGGGGAACCCCCCGCGGGGTTTACTGAACT

>M.04.0.10_A_64

TTTATTATACAATATTCTATGGTACTCATGTTTTTCATTT

>M.04.0.10_A_65

AGACCGGTTCCGCTGACACTGTATAGCGTCTTTGAGCCTT

>M.04.0.10_A_66

GCAGATAATGATATATTTAACCCGGTGACTTGTGATATAA

>M.04.0.10_A_67

TTTAAGGAGATGGTTTTATGAAAATTGCAGATAAGGTCAA

>M.04.0.10_A_68

CTCAGATTTCCATAATTGATCGAATGTTTGAAGGATGGG

>M.04.0.10_A_69

TAGAAATACTATAAATTAGACTTTCTAAATAGAGAATATATT

>M.04.0.10_A_70

TTGTAATAAATATTGGCTACCATTATGATACTCTACTA

>M.04.0.10_A_71

CATTTTAATCCACGTAATGAAGATATATTCAAATTTATTA

>M.04.0.10_A_72

TAATTTAACTAACTCATTCTGAGTTTAAAAATTTAACGG

>M.04.0.10_A_73

GAATAGAACTATAGCAGAAATAACAGCTTGCACTCCGAA

>M.04.0.10_A_74

CATAATAAATTTTCGGTTTGTGAGTTTTGTGTACTTAG

>M.04.0.10_A_75

CAGTCAATGCGTCGAACGACAGCTCGACACCGACACGGA

>M.04.0.10_A_76

CGTGCTTTAAGGGAAGATCGAGCTGGATAAGCAAACGGGC

>M.04.0.10_A_77

AATTTCTCATGGCCAGGAGGCATAAGCACAAGTCACATC

>M.04.0.10_A_78

AAATGGTGTAACAATGACTATATATCAATTACTATTTGA

>M.04.0.10_A_79

TTCTTTTCTCTGCTGACCAATCAGGAATTTATTACAAAA

>M.04.0.10_A_80

TTAAATGATCCAATAGGATTTGTAATTGTTGTAGAAC

>M.04.0.10_A_81

AATAGAATTTGTCCATTTACTGCGGAAATATTTGCTACTA

>M.04.0.10_A_82

TGATTTCCACTTCTTGAAGTGTGTAGTTTGTTTGATATCA

>M.04.0.10_A_83

ATATTTTTACTTCAAACATGAAGACCCATTATTACAACTT

>M.04.0.10_A_84

ATTGTTTTCTTATTTTCTTATCCATCATACTTTCTTCCCC

>M.04.0.10_A_85

GTTCAACTACTAATACAATAACAAAATCTGATTTTTCCTT

>M.04.0.10_A_86

AATCTTTTTTCTCATATGCAAAGGGTTATTTGTAAATCAAG

>M.04.0.10_A_87

TTCTTGTAATGTCGTTATGTTACCATCATCTTTAATCTC

>M.04.0.10_A_88

CAAAAAACGGGATAGACCCGGCAATTATCGCAAAGATCAC

>M.04.0.10_A_89

CGTTAGCATGCAATACGGGCCTCAAGGCGCTTATATCTA

>M.04.0.10_A_90

ATTTAAGCAAAACGCGATTCTTATGTATAGTCATCGA

>M.04.0.10_A_91

CTGGTGTGATGTCAGTTATTAAATATGATAGCGGTAAGT

>M.04.0.10_A_92

TTTATCGGGGTCATCCCATGTCCGCACAAGTTAATTCAAA

>M.04.0.10_A_93

ATATATGTTTCCAGGCCATACAATCGCTACGCCAACGAT

>M.04.0.10_A_94

AATTTCTCATGGCTAGGAGGCACAAGTCACATCATAAA

>M.04.0.10_A_95

TGAATTCTTGATATAAAGTTTCTTAGTTCTGCCCAGTT

>M.04.0.10_A_96

AAAGTTAGATTAGGTATAAAAGAAAAGAAAGCTATATAT

>M.04.0.10_A_97

GAGGAAGTTTATATAGCTATCTTATTAATACAGTTAGT

>M.04.0.10_A_98

GGCTGCACGCCCTACAGCCCTCGCTTGTGCTCTCACTGT

>M.04.0.10_A_99

GTAATAGATAATTCCTCGTTTCTATCGTTGAAAGGATCG

>M.04.0.10_A_100

TGTTTTGTATACAGACTCACGAAGTTGTAGCCGTACC

>M.04.0.10_A_101

TATATTGGATCGTCATTAGGTGGTAGTGTAACATTAACATC

>M.04.0.13_A_1

TCTATTATAATCGATGAAACAGATAGAGCTGAATTGTGTAC

>M.04.0.13_A_2

ATTCCTCCACCTACACTAATGAATAGTATCAACATCTGA

>M.04.0.13_A_3

GCGCACGGTTCACAGTTCGTATTCGACGACAGTAACATAC

>M.04.0.13_A_4

TTCCCTTGTTGAATTAGTAACTGTTGTATCTGATTTG

>M.04.0.13_A_5

GGACTTCCACCTGTAGCTAGAGTTCCATTGAATGCTTGCCAA

>M.04.0.13_A_6

TTAACACTTAAACTTGAGTTATCATCATCAATAGATA

>M.04.0.13_A_7

AGACTGCAGAGAATATGTATTCAAATGCCTGAAGATGAT

>M.04.0.13_A_8

TATAAGATATCGATGACAATACATAAGAATAAAGTACTGC

>M.04.0.13_A_9

GTTGAGGAAACATATGCAGACCATAACTCGATTGATGCCGT

>M.04.0.13_A_10

ATTTTAGATCAAGTGTGGCAGCATATACAAAGTTGCCCAG

>M.04.0.13_A_11

GTGCGGAAACTTATCAAGACCGTGTCACTGACTGCACCG

>M.04.0.13_A_12

TCCTCACCCCGTAAGGGGGTCATCGCCATCCGCTTT

>M.04.0.13_A_13

TGCTTGTTCCGCCATTGACAACGCTTGAGAATTTCCAT

>M.04.0.13_A_14

TTTTCTATCAGTCCACCGCAAGGAGTTACTAGAACAAT

>M.04.0.13_A_15

GGTTGATATGCATATGTTTGTTGAAGTGTACGCGAAAATA

>M.04.0.13_A_16

TCTAGAACAGATATGGGGTGTCGGTTTTACTGTAGAACA

>M.04.0.13_A_17

AATTTAGCTACATATATCGTTATGCCGGCTCTCTTTCTTATA

>M.04.0.13_A_18

AAAATAACGGTGACAATAAAAGTAGATGAAAATGATCTAGT

>M.04.0.13_A_19

TCTCTTAGTATTGCTCTTTTAACTTTTTTATTACTACATAT

>M.04.0.13_A_20

TCGTAAAAGGCGTATTCAGGTTTCCGCGCGATGTAGTGCTT

>M.04.0.13_A_21

CGGGGGGTTCCCCCACCCCCACGGGTGAGAATAAGGGGG

>M.04.0.13_A_22

ACATATGCACCAACATCAACGACAACAGTCGGTGTTATCT

>M.04.0.13_A_23

AATATAACGCTGACAATAAGCTTTTGCGTTCTTTCGAACCC

>M.04.0.13_A_24

TTGTGTATCACTTATGTTACCCACAAATAAGCCCGGAAT

>M.04.0.13_A_25

CTCAAGGCAATTATAAATGAGGTGTCGACCAAGGTACCT

>M.04.0.13_A_26

TTTGAAAGTATGTATCAGTTCGAGATCATAGAGAAAGCTGT

>M.04.0.13_A_27

TTACCACTAACTGCAATAGGTGTAAACTTACGTGTTGCA

>M.04.0.13_A_28

CTTCACAATATTCTTCCTCCTCCTCTTCATCATAAAGATAT

>M.04.0.13_A_29

TTTTTTGTTGCACATAATATTTTAACATTTCTGTTAGTCTT

>M.04.0.13_A_30

GTATGGAATTTGATGAACGAAATGAAGTACAAAATAGACGT

>M.04.0.13_A_31

GATTATACTAACACCTAGAATAGAATCTAATATATATTTTC

>M.04.0.13_A_32

CTCCCAACGTTCCTGGGAAGCGTAGGTCTAGCACTA

>M.04.0.13_A_33

AATTGCAAACTTATTGGTACTTAAAGCCCTTCATAAACAT

>M.04.0.13_A_34

CGACGGGAATGCTCTGTTGGGTTTGGTCTTCAGACTCGG

>M.04.0.13_A_35

CTAATTTGACACTGACCTTCTCTGTTACCGGTGCCGG

>M.04.0.13_A_36

GGTATTGTATTAGGTGGTATTTGGATAACTTTAGCTTG

>M.04.0.13_A_37

TTTTCTGTAACTAGTAATGATATATCACTATTTGTCACTA

>M.04.0.13_A_38

CATCTTACCCCACCACATTAGGCTATTTGCTCTTAGCA

>M.04.0.13_A_39

CAAAACGGGAATGGGAGGAAGGTCAGACACCATCTTA

>M.04.0.13_A_40

AAAATCACGAATTGATCACCTCATTCTTCCTCTTCTT

>M.04.0.13_A_41

TAATTTTTTCGTTGTTTTTATCAAAACACAAAGCACTA

>M.04.0.13_A_42

CAAATCTACAAGATACGTGGGTTACTATCTCACCGCG

>M.04.0.13_A_43

AAATCGAAAGGCAGCTCACCTGAGACAATCATATCGTAGT

>M.04.0.13_A_44

TTAGCCCAAATGCTGACAAAAGACCCCGAGAAAGTAAA

>M.04.0.13_A_45

TATTTAGCTACGTATACAATACTGTATAACATATTCGCT

>M.04.0.13_A_46

GAATAAGCGGGGCGAAGCCGACGTGTACGCCGTGTCTTTC

>M.04.0.13_A_47

TCTTATATAAAATCAAAATTGTATGAAATCCAAGCTGAAGTTTTT

>M.04.0.13_A_48

TCTCACCCACTGCTACTGCTTCCGCAACGTTTACAGCATCT

>M.04.0.13_A_49

AGTGATACTATGGCATGGAAGCTAGCTATACAAAATGCT

>M.04.0.13_A_50

AAATTGATGTTTAATGTTGGTAGTGTAAAGTTTGAAGGAA

>M.04.0.13_A_51

ATTTTTCCTAGATAGCGTTGAAAATCTTCTGATTGTTTC

>M.04.0.13_A_52

GTGACTGAGGCAACGGATAGTGTTACATTGCCTCCAAT

>M.04.0.13_A_53

TGTATAACTTGTAGAACCGGATGATAAGTTAATGGT

>M.04.0.13_A_54

ATTCCAAAGTTACGTCATCGATGACCACGTAATGGAAA

>M.04.0.13_A_55

ACAATGACTACTGGCATGTTCGCTACACGGAATAACTCT

>M.04.0.13_A_56

GAAGTCTGCCGCTTAGTACTATGAGCTTAGAATCTATA

>M.04.0.13_A_57

AAAGATCAATTAAAGGAGTTCTTTGAGTGGATAGAGAAAG

>M.04.0.13_A_58

AACGGGATTCTGAAGAACTATCAAAACGTGAACAATATTT

>M.04.0.13_A_59

GGTATATCCTTACATGATCCCTCTCCCTCATAAAGTAATTTCAT

>M.04.0.13_A_60

ATTTATAACAAACTCCAAGAATGGGAGAGAGTAAACATA

>M.04.0.13_A_61

TTTCTTCAAGTGATAGCCCCCACAGGAACGCCCATGACC

>M.04.0.13_A_62

TCAAATGTACCCCGCAATATCCTCTTTCCTACATTCACTT

>M.04.0.13_A_63

GAAAATGTATTAAAAAAATATGTAATTAATGGGGTTGAG

>M.04.0.13_A_64

TTTATCACTATTGCTATCTTGTTATACAGTGCATTCTTTGC

>M.04.0.13_A_65

GTGATACTAGATAACGGCACATGGAATGATGAAGCGACAT

>M.04.0.13_A_66

ATTTCAAAAAACGCTTGATTCTGAGTAGAACTCACTGGA

>M.04.0.13_A_67

TATACAAAGTTGAAGTCATTTGTTGTTACATTTATGATGC

>M.04.0.13_A_68

TTTTCTTTATCCTCTTTATTATTTCTTCTAGTTGTTTAT

>M.04.0.13_A_69

ACTTTTTGTAATTGTATTTGTTGTTGTGTTGTTGATTG

>M.04.0.13_A_70

TATCATAAAATACAAATTAGTTACTATCATTACATATAAG

>M.04.0.13_A_71

TTACGATACATTGAGCTTCTTCCAGTAGGGACTATTACGG

>M.04.0.13_A_72

AGTTATTTACCGCAGTCTCGGGGTTCCTAATTGTTTTTGC

>M.04.0.13_A_73

GCATTCAACCCCGCGGGGGAATCCCCCGCCCCCATGGGGG

>M.04.0.13_A_74

CTTTCGTCACTGAATATCTCAAATCCATCCAGACCTCATT

>M.04.0.13_A_75

ATTTCAGTGTTTACCCACCTCTTCGCATAGAAACGAAT

>M.04.0.13_A_76

CAACCCCGTAACTGCCTCCTAGCTTATCATCTGCACTTTT

>M.04.0.13_A_77

GCCTCTGTTTCAAGTGCATAGACAGTCACTAAACCCCCGC

>M.04.0.13_A_78

TATTGATAACAAAGCGGTAAACGAAACGCTGAACCGTTAC

>M.04.0.13_A_79

CTATGGTCGAGTGGCGTAAAAAGTTCCGTTTCCTCGTCAT

>M.04.0.13_A_80

ATCCTTAAAGTTAGTCTGTATCAATAGATTGTTAATATCTT

>M.04.0.13_A_81

TTTTAAACAAAATAAACTGTCCATCCTTCCTCACATTTCTT

>M.04.0.13_A_82

CCTCACTATATATGCTTTACAAAACTACCGACACGTATTATT

>M.04.0.13_A_83

TTTTGCTACCCGCACATCTATAGTACTTTAAGATCGG

>M.04.0.13_A_84

TTGGTAACCTCATCACTCACCGTTGTTAAAAATACGT

>M.04.0.13_A_85

TACTTCATCACGTCCACGTTTGTTATTTTTGTTGATTTAA

>M.04.0.13_A_86

CATAATAAATTTTCGGTTTGTGAGTTTTGTGTACTTAGTGTA

>M.04.0.13_A_87

GGGCGGGTTCAACTTCAGTGGGCTGTCTCGTCTCGTTA

>M.04.0.13_A_88

AGACCTATGCAAGATGAGATCCTGCAGTTCGTATGCATG

>M.04.0.13_A_89

TACATGGGCTACAACCCATACGCTTACGGATTAGGACAAT

>M.04.0.13_A_90

TTCTGCATACTTCTTATACTCTTCTTTAGCGATTGCAAA

>M.04.0.13_A_91

TGCAGTAAAACCGCAAAATGGACAAATTCCACCTGGTTT

>M.04.0.13_A_92

TTAAAAATTAGATTAAATTATGAGTCAGGTAATCGGTTTT

>M.04.0.13_A_93

ATTCTATATTCATTCAAATTAGAATTATATGCAATTTCT

>M.04.0.13_A_94

AGTATGTCTCTATCAATTACATCTGGTATATCCTCAACTTCAG

>M.04.0.13_A_95

TACACCCCATTCATCACTTTCCATTATGTTTCTTATATGA

>M.04.0.13_A_96

AAGCTTAGAACTTGGGTCATTTTCGGCCCAATGGAAAG

>M.04.0.13_A_97

TCTGGTTACTACCCGTTCCAAAACGGCTATATCGACAT

>M.04.0.13_A_98

AGAGTACTCCCTGCGTAAATTCCGCCAACAATAGATATT

>M.04.0.13_A_99

ACGAGAGTGTTAGGATCTTCCCATCCTACCCCTAACGGG

>M.04.0.29_A_1

TCTGATCTAATAGAAGGACTGCTAAGTGTTTGGCCCTAC

>M.04.0.29_A_2

CCATTCTGTGTAATTGAATTTTCTTGACTTTTTCTTCT

>M.04.0.29_A_3

CTACAATTAAAATCTCTTTTCCGTTATACTTGTCAATTCCT

>M.04.0.29_A_4

ATCGAATTTCCATCTCATCAGGAACCTGCACAAGTTGC

>M.04.0.29_A_5

CCCGTACATTGTAAATAGTAAGCGTCAATTGCCACAGGTGC

>M.04.0.29_A_6

CAAAACAAGGTGATACATGCAACTTACATGTATTATATCTA

>M.04.0.29_A_7

TATTGGCGTTAGGCGGTAGTGCTATAACAATCCCTTACGC

>M.04.0.29_A_8

TACGAAAGTAAGACGGAAAAGACATGGGAAGGAATAGAT

>M.04.0.29_A_9

TCTAATGTAATTTATAGTGTAGTAGTTGAGGTTGTGTTTA

>M.04.0.29_A_10

AAACTAATTCTTTTAAGCTTAAGTATTGGGGCAACCAGA

>M.04.0.29_A_11

CGAAAGGATTCTTTCTATTCCCGTTATCAGTAAATACACT

>M.04.0.29_A_12

GCTTTACGGGATATAGTCCTTCAACACCGCCTAATTATTG

>M.04.0.29_A_13

ATACACTTTTGCCTAATACTATCCTCACGGGGCATGTGCAA

>M.04.0.29_A_14

TTAAGTGATGCGGTAAATAAAGCATTAGATAATATAAGGG

>M.04.0.29_A_15

AGAATATCATCTAACTTCATACTGCTGTATGGAAAAGGT

>M.04.0.29_A_16

ATTTATTGTGAGTGCGGACTTTCAAATAATCTAGACGT

>M.04.0.29_A_17

AGAATATGTAGAACCAGATGTTTTAAAAATTGATTGTG

>M.04.0.29_A_18

AGAATAGCATTGAGCCACTAAGTCCAGTAGAGCTAGAG

>M.04.0.29_A_19

ACAAGTTGGTAGGTCTGGTTCGTGAACGGGTTAATATAAA

>M.04.0.29_A_20

TAGGGTTTGGGATAGCTAATGCCAATCTAAGTCATCTAT

>M.04.0.29_A_21

AGTTCATAATACCCTTGACCAGTGCCTGGTGCTAAAGCGT

>M.04.0.29_A_22

TTTATGTTCCCTTCTCTCTTTTTTACTTTACTTCATTCT

>M.04.0.29_A_23

GGAATAGTTATAGCAAATGCCGAAACTACCATTGCCAGTGTT

>M.04.0.29_A_24

TCAGACTCATTGCGAAATTTGCTATCAATCGCCCCTATCAG

>M.04.0.29_A_25

AATAGATACCCTAATATTGTCCCTATAAAAATTCCCGC

>M.04.0.29_A_26

CTTTTACAGTTTTACTACTTGGTTTATTGACTAACGAGT

>M.04.0.29_A_27

TTCCTGAACCGGTCTGATTTTTAATAATTTTTTTCTCCA

>M.04.0.29_A_28

ACAGCGGATATAGTAAGTATAAATAATAGGGATAATAGT

>M.04.0.29_A_29

CGTCGCAGAAATACTGAAACCCCCACTTGATTAGGGCT

>M.04.0.29_A_30

AGAATTTGAGAAAGAACGTCCGTCAATTGTGCTTTGCTTTC

>M.04.0.29_A_31

ATTAGTATCGTCCTTACAGACCTTTGTAGGCGGTTTCCA

>M.04.0.29_A_32

TTAAACGTAATGTGGGAATTCGATTTAGCTAGGTTCC

>M.04.0.29_A_33

TATAAAATTTTTGCTAAAATTTTATTTGAAAGATGAAGATAT

>M.04.0.29_A_34

TTAACTGAGGACTTGAGCGATGAAGCCGAGGAAATTTAT

>M.04.0.29_A_35

CTTAATAACTTATCTATCGTCTTCCCCACATTGGTAAAGAT

>M.04.0.29_A_36

ATATTAACTGTCTTCGTAAAAAAAGTTATAACTGGTATA

>M.04.0.29_A_37

TAGCAACTGCATTTATTAACATATTCAAGAATTCTAAAT

>M.04.0.29_A_38

CCACCCTTCGAACCCGTTGAACCTTGAGCTATAATATTAC

>M.04.0.29_A_39

CGTAAAGATTACCTTACCCTCCTTCTTTACCTGTACTCTC

>M.04.0.29_A_40

CCTATATTGAATTCTATGCATATTCAATATTTCTCAACACCTT

>M.04.0.29_A_41

TTAAATATTTTTTGTCCAAAACTTTTTATATTATCATAAA

>M.04.0.29_A_42

GAACAGGTCGAAAGAGCTGAGCTTTGGCATGCAATTTA

>M.04.0.29_A_43

ACGTATACTATATTGTAGAAAGTGCCACATCATCACGAAC

>M.04.0.29_A_44

TTTTCTCATTCTCTTCGTCTTGTTGAGATTTGAAATACGT

>M.04.0.29_A_45

CCTTCGCCACCGCAGGTCAATGTGCCACCGCAGAATAAT

>M.04.0.29_A_46

GTAATTTACATTTATTCAAGAATACTTTTTAATAAGGCA

>M.04.0.29_A_47

TTTGTGAGTTGGAGACCGTTACGGCCACGCATTCCACGCGT

>M.04.0.29_A_48

AACGAACAAATTGTTGATCATGAATTTATTTATTATAATAA

>M.04.0.29_A_49

ATATCTGTCTCTATTGGTACGATTTGGTAACCTCCAGAT

>M.04.0.29_A_50

CATCAAGTTGTAAGAAGAGTTCTTTATCTGTAATAGTTTCA

>M.04.0.29_A_51

TTTGGCTTTAACAACTCAGGATGATTCTCTTTAAGCCAT

>M.04.0.29_A_52

GCTGGTGAGGTGACATAATGAAAGCATATGTTTGCAAATTCTG

>M.04.0.29_A_53

CTTCCTGTTATACTTTCAGTAGCTTCTGCCCCTTGTAATTGC

>M.04.0.29_A_54

CCTGTAGCTAGAGTTCCATTGAATGCTTGCCAATTAAC

>M.04.0.29_A_55

ACATATAAGAGAAAAAGGTTTTTTTCTTGTCTTGATTAAA

>M.04.0.29_A_56

GTCTCTGCTCTAATGAAGATTATTTTACCTTGCTCTAGG

>M.04.0.29_A_57

GTATGGTTAGCGAAGAAGCAAGATAAATCACCATTTCCATC

>M.04.0.29_A_58

GATAAAAAGCTTATTCTTCAGAACGATAACGTTGTTAGA

>M.04.0.29_A_59

AAAATTTCTCCTATTGCCATATATTACTTTCACTCATCTCTC

>M.04.0.29_A_60

CTTATAATAGGATATAATGAAAGGGTAATTGGAGGTAGA

>M.04.0.29_A_61

TTCTCAGTTAAATAGTACACATTTTCATTTCTCTTCAT

>M.04.0.29_A_62

GTTATTTCATACGCTTGGGCATAATTATTACTGTATACTAC

>M.04.0.29_A_63

AAAGAATCGAAGTATTCATACGGCGTGCCTATCATACCGT

>M.04.0.29_A_64

ACGCAAACTGCCGAACAATCAAAATCAATTTTTGGTCA

>M.04.0.29_A_65

ATAATACGGTACCTACGTCCATTTGCGACGTCTCATTAAT

>M.04.0.29_A_66

AACGTTATAGCCACATATATATAGCCTATGTTTATCCCT

>M.04.0.29_A_67

TACGACAGAAGCAGACATCAACCAACGCCATGATAATCCG

>M.04.0.29_A_68

TTATTGATGGCTACGTTGTGAATGCATTTTATAACGGATCTAA

>M.04.0.29_A_69

TCGGCTATGATTACACCGAAATAACCGTCGTCATTAAGT

>M.04.0.29_A_70

GCTATTACTTCGTAAAGTAATAGTAACGTTATTGTTACGCT

>M.04.0.29_A_71

GAAGTTGCATAATAAGTTTTTGTTCCGTATACTCCCACTAT

>M.04.0.29_A_72

ATTTATGATAAAATATTAATCCCTCAGACCAGGGGCGAGG

>M.04.0.29_A_73

TTCAAAAAATTGTTCTAAGTTGTACTCTCGGTTGTTTT

>M.04.0.29_A_74

GCAAATAATTGCCCTCAGCGATCTTGCAACTCTATAGAAG

>M.04.0.29_A_75

CGTTCAAAGCGTTTACATGAATTATTGTATAAATGTTGAA

>M.04.0.29_A_76

TTACACTCTGAGCCGACCAAAAGACGTTAATAAGCCTAG

>M.04.0.29_A_77

TCTCCTCGCCCCACGCCCAAGCTTGGCGTGCTTGGGGTG

>M.04.0.29_A_78

CTTCCCGAAGCTCTCGACGACCTTCTTTACCATCACAGT

>M.04.0.29_A_79

TGCAAATGCTAATCTCTTGAAAGTTATAGCAATCACTTGT

>M.04.0.29_A_80

TTGAGTACTGTATTACCTAAATCAGTAAGTAGAGAAATT

>M.04.0.29_A_81

TTCCGCCGTATGTCGTAGTTTACGCTAATTATATACAGC

>M.04.0.29_A_82

ACGGGATGACTGAAATAGAAGTAACAAAGGAATACCA

>M.04.0.29_A_83

GTACTCAGTTCACAAAGAGGCAAAGCAGTTCTAGGT

>M.04.0.29_A_84

CCGAGACGCCCTGGCGAGGGTGATACACCTCTGCCCCACCCTTCG

>M.04.0.29_A_85

TAAATAGTTTTAACTAGATCATGTAAAGTGTTTTCATCA

>M.04.0.29_A_86

TAACGTCCTCGTCCAACATCTTAAATATCTCTCTTTCT

>M.04.0.29_A_87

CAATGTAAGTGAAATGAGGCCTGTAGATCATCTCATCAC

>M.04.0.29_A_88

GTTATTTCATACGCCTGTGCGAAATTGTTTGAATATTCTAC

>M.04.0.29_A_89

ATCTTTGCTACTATTGCTGGGTCCACACTGTTTTTCGA

>M.04.0.29_A_90

ATCTTCATCTCCAAACTACTGCTATTAACTTATCTCACTC

>M.04.0.29_A_91

ACTAAGGTCCTGTCTGCTGCTACTGCCTACCGGTCGCT

>M.04.0.29_A_92

GAGAAAGTCAAGACGCTTAAACAATACTATTTTCTTATC

>M.04.0.29_A_93

ATTTATACCAATACACGAAATTGAAAATATAGAAACAT

>M.04.0.29_A_94

ATTAAGAGAAGGTTACACTTTAACAAAGGATAGTATAAT

>M.04.0.29_A_95

ACGAGAACGGCAAAGATCAAGCCCATCGTAAATAATTTC

>M.04.0.29_A_96

GAGTATGAGTACGATGTTATGAAGCTAAATGAGGAAA

>M.04.0.29_A_97

GATTTAAACACGCTTCTAATATCAATATTAGCGGGCAT

>M.04.0.29_A_98

AGAACGGGTAAGCATTCCTCTACCTTATGCACCACATCAATCT

>M.04.0.29_A_99

AAACGAAATATCGAAGGTGTCAGATGAGGAAAGGAAGAA

>M.04.0.29_A_100

AAGTAGAAGTGGCAAAGAAGTATGACGTATTAGGGCG

>M.04.0.29_A_101

AACTCTTTCATAATCGGTTCAACGACCTCAAACTTCAG

>M.04.0.29_A_102

TCAAAAAAACAAATTTTTAGTAAGAGAAAAAATAGTTAAA

>M.04.0.29_A_103

TCTACAATTTTGGAGGAATTTGGATTAGATTTAACTTTAT

>M.04.0.29_A_104

TTATACGTTGAGGATGCCGTAAATGCGGAAATAAAAACGC

>M.04.0.29_A_105

ACTGATACTCTATCCCATATATTGCAAGATCCCTATC

>M.04.0.29_A_106

TATTGTCCTGCAGTCTCGTAATATACTTTAGTCCCATACA

>M.04.0.29_A_107

CAAGACATGCGTTATATCTAGCTTGTGTGTTAAACAGAG

>M.04.0.29_A_108

CTATAAACCCCCGTGAAAAAGTTTACAATATCAGAACCT

>M.04.0.29_A_109

AAGAGAGTTCCCAGCCCTGGTATAACACTGCCAACGCCAT

>M.04.0.29_A_110

CCTACTCATTCGCATATTTTTCTAGAAACACTTTCACTTT

>M.04.0.29_A_111

GTATCGAAAAGAATAGATGCCCATTCTGCAATGTAACGT

>M.04.0.29_A_112

AAACCACATCACAGTGGTTTTGAAGAAGTAAATATTTA

>M.04.0.29_A_113

AGAGTACTCCCTGCGTAAATTCCGCCAACAATAGATATT

>M.04.0.29_A_114

TTAGTTTGAACAAGTTGGACATAACGTCCGTAGAACC

>M.04.0.37_A_1

TCAGTCGTTTCAACGGCTGTAAACATTGACTATTCAAATG

>M.04.0.37_A_2

CCCGTCGCCAACGTTCCGTTGAACGCTTGCCAATTTGCCA

>M.04.0.37_A_3

AAATAAAGTGAGCTAAATGGCGGCAAATCAAGAAGTATG

>M.04.0.37_A_4

TTCCCTTGTTGAATTAGTAACTGTTGTATCTGATTTG

>M.04.0.37_A_5

CGTCGCAAATATTCATTAACTATGATACATTCCCGTACCAG

>M.04.0.37_A_6

GAAGTCTGCCGCTTAGTACTATGAGCTTAGAATCTATA

>M.04.0.37_A_7

AGACCTACGAGATGCCCAAATACTACATAACCTTTTTT

>M.04.0.37_A_8

CGTGATAATTATAAATGGGCTGAGGCTATGGGTAATAAAGTT

>M.04.0.37_A_9

GACATTTGAATTAAAGCCAGAATTGCCAACAGATTCGC

>M.04.0.37_A_10

TTTCTAAAGATGATAAAAGGTCAAACATTCTTTGACACTCG

>M.04.0.37_A_11

GTATGCTTATCGTAAAGCGAAATGACCCAGAGCGGGACAT

>M.04.0.37_A_12

TACTTACACCCTTGTTTTAAGTATCTATTTTTGGGTTT

>M.04.0.37_A_13

ATGTAAGTATGCCTTTCCTTATTACCATCACCCTTCTCTA

>M.04.0.37_A_14

AACCCACCTGCTGCAGCTAATATCACTAACAATAGAAT

>M.04.0.37_A_15

AGTTTCTTGACTTCCATACGAATTAATTATCAGAGTCTA

>M.04.0.37_A_16

CTACAATAATTGCGTTGTGATGCTTTCTGCATTCAATCATGA

>M.04.0.37_A_17

ATAACCTTCCTGCCAGGGTAGTAAATATCATCCGGG

>M.04.0.37_A_18

GTCCCCTGGGGTGTTGTTCGCGACAACTGCAGCAACGAG

>M.04.0.37_A_19

CTTGGTTTATGGAATGAGAAGAAGAGATTATATCAGTT

>M.04.0.37_A_20

TACGACAGAAGCAGACATCAACCAACGCCATGATAATCCG

>M.04.0.37_A_21

TATCCTTATCAAGAGGAGGTTATCAACAAGATTCGTAACGCT

>M.04.0.37_A_22

AAACTAATTCTTTTAAGCTTAAGTATTGGGGCAACCAGA

>M.04.0.37_A_23

TGAATTCTTGATATAAAGTTTCTTAGTTCTGCCCAGTT

>M.04.0.37_A_24

ATTTTAGATCAAGTGTGGCAGCATATACAAAGTTGCCCAG

>M.04.0.37_A_25

AACCTAAGGTTAAGGCTTCTACGTGCTGTTTTAGGTCAT

>M.04.0.37_A_26

TACGTCCCCTCAAGTAGGGGAAAGTGTTTCAGTATTAGG

>M.04.0.37_A_27

TTCCGCCGTATGTCGTAGTTTACGCTAATTATATACAGC

>M.04.0.37_A_28

GTTATTCTATTAAATGTGCATACTTCTTGATTTGCCGCCAT

>M.04.0.37_A_29

CCTCGTATGCGATATCTACCCAATACGTTGAACCAA

>M.04.0.37_A_30

TAGCAACTGCATTTATTAACATGTTTAAAAATTCTAAA

>M.04.0.37_A_31

TTTGATTCCAGAATTTCCCCCACACTTACTTTTACTCCT

>M.04.0.37_A_32

TCTCTTAGTATTGCTCTTTTAACTTTTTTATTACTACATAT

>M.04.0.37_A_33

ATAAGAGCCTAAAAAGCAGTTCAACCCTAGAGCCTTT

>M.04.0.37_A_34

TGCTTGTTCCGCCATTGACAACGCTTGAGAATTTCCAT

>M.04.0.37_A_35

GCAGCTTGTAGCATCCTTAATAAATTTATATTTGTTATT

>M.04.0.37_A_36

ATAAAGAACGATACAAAGAATAATAACAATACAGCCGAAG

>M.04.0.37_A_37

TTTTCTATCAGTCCACCGCAAGGAGTTACTAGAACAAT

>M.04.0.37_A_38

CCACTAACTGCCTTTAATAAACTAAGGAATTCTTGGG

>M.04.0.37_A_39

AAAATAACGGTGACAATAAAAGTAGATGAAAATGATCTAGT

>M.04.0.37_A_40

TATTAACACATATTCGTAAAATTTTTGTTGAGATAGTGT

>M.04.0.37_A_41

AGTGGTGGTGAATTGTTTTCTACACCGTTATCTTGAAG

>M.04.0.37_A_42

TTGTTATCTTTTTCCATACTATACTTAAACTTAGTCTTTACAAT

>M.04.0.37_A_43

TAGCAATGCAACAGCTTCATTCTCACCCTCACCGGGCGA

>M.04.0.37_A_44

AGTTCATAATACCCTTGACCAGTGCCTGGTGCTAAAGCGT

>M.04.0.37_A_45

TCTTCGGTTCTTACTAACACCCTACCAGCTTTTACACCTT

>M.04.0.37_A_46

ATCCCTCCAACAGTAGTTGTAAAACGCACACTCTAGGCA

>M.04.0.37_A_47

TAATTTAACTAACTCATTCTGAGTTTAAAAATTTAACGG

>M.04.0.37_A_48

TCAAGGGGCTATTATAGCAATATTAGGAGTAAAAGTAAGT

>M.04.0.37_A_49

AGACCTACGGTTACATAGGTTTCTGACGGAGGGGTATTCT

>M.04.0.37_A_50

TTTTTCCCTGCTTTTTAGAAACGTTATCCGTCCATGGGT

>M.04.0.37_A_51

ATAGCAATATGATGTACTATATTTAAATGAAAAACTGT

>M.04.0.37_A_52

TGGAGTTTTAAAGACCTCTATGGAAACGTTTTGAAGGA

>M.04.0.37_A_53

AGCTCTTTGAATCTTTGCACCAAATATTGAACTCCATT

>M.04.0.37_A_54

GTCCTTAGTTTTTCTTTTCCTCTTCTTCTAAATTTCATTC

>M.04.0.37_A_55

ATCTTTTTCAGATCCTCCCCTACCTTTACGCCCAGCAAT

>M.04.0.37_A_56

TTACGAAAAGAGGGACTATTAAGAAAGAATATTTAAAGA

>M.04.0.37_A_57

AGTTTTTTTATGAATATTACATCAAAAACGCCATTTATA

>M.04.0.37_A_58

TTGAACGTTATAGTTAAATTAGTTAAAGATAAATGTATTG

>M.04.0.37_A_59

AAGTAGGTACGTATAAGTCCGCATTCACACGTTTAACC

>M.04.0.37_A_60

TTTTATGTTGCACAGAACGGGGGGAACTACGCTCCTCCTC

>M.04.0.37_A_61

ATAGCTGCTGCGATTGCCAACTGCTCGGGATTCTGTGTA

>M.04.0.37_A_62

GTGTACATCCATCATTGCTTTTATTGAACAGATTATGTCATTA

>M.04.0.37_A_63

TTCTTAAAGAATCTCTCATAATAACATTATTCTTAACAT

>M.04.0.37_A_64

TCTAACAAATTTACCTTCACCAACCTTCTTATAATAATGTG

>M.04.0.37_A_65

ACATGTAGTCAGTATCGTAATTGTTTTTCAATTCATCAA

>M.04.0.37_A_66

CAAAATAGAAGGCAGTTACAACAACCACAGCAAACAGAT

>M.04.0.37_A_67

CTCAAGGCAATTATAAATGAGGTGTCGACCAAGGTACCT

>M.04.0.37_A_68

TGAAATGCAACTTGCTAACAAAATAAGTACCGCTAAGG

>M.04.0.37_A_69

TCAACAATATATTTCTTTTCCACTGACCCCAACCCTGAT

>M.04.0.37_A_70

TTTGAAAGTATGTATCAGTTCGAGATCATAGAGAAAGCTGT

>M.04.0.37_A_71

GCACCCTCACTAGCCTTTATAAGATCAGTAGAAATTTTC

>M.04.0.37_A_72

TCAGTTGAGGAAATACGTGCGGTGCTGAGACATGAA

>M.04.0.37_A_73

CTGGACTTCAGCGACACGCTCGTGACATCGGTCATTTTA

>M.04.0.37_A_74

AAGCTCTAAGACGATGAAAACCACCAATTATTCCAATTACAT

>M.04.0.37_A_75

TCTAGTATAACTTTATACAAAAGCGGAACTGCAAATAT

>M.04.0.37_A_76

GTCAGTTGCAATTATCACAGCCGTCGTTACCGACCCCG

>M.04.0.37_A_77

TTTAATCACCTGAGTTAAAGTGTGTAAATACATATTTATAT

>M.04.0.37_A_78

TTAACTGAGGACTTGAGCGATGAAGCCGAGGAAATTTAT

>M.04.0.37_A_79

TACTTTTTCTTCACCTTTGATATTTAATTTTACTATGAT

>M.04.0.37_A_80

CTGTCATCAACCAATAAATAGACAAAAGATATCTTTTTTT

>M.04.0.37_A_81

TACGTTACGCTTAACATGATTTCTTCGTAGTCTCCCGT

>M.04.0.37_A_82

CCACCCTTCGAACCCGTTGAACCTTGAGCTATAATATTAC

>M.04.0.37_A_83

CGTAAAGATTACCTTACCCTCCTTCTTTACCTGTACTCTC

>M.04.0.37_A_84

GGGTACAGTATACACGAGCCAGGGGTTGCCGGTTCCCGGT

>M.04.0.37_A_85

AATTGCAAACTTATTGGTACTTAAAGCCCTTCATAAACAT

>M.04.0.37_A_86

ACCAATTCGCAAAATCTTTATGAATTGCTTTAACTATAT

>M.04.0.37_A_87

AATTTATATTTATTATATCGGCTTTTACTGGAAAGCGGTGCT

>M.04.0.37_A_88

CTAGTGCCAGGGTTCAGGGGTGTACCCTCACTGCCACA

>M.04.0.37_A_89

TACAATTAATGCAACAACTATAAATACTATTACTAAGGG

>M.04.0.37_A_90

GAAAGAAGAGGATAGTTAAGCTAGCCTTAGAGTCACTA

>M.04.0.37_A_91

GTCTGTCCGTACACTCTTTTATCATGTCATAGATTTTTCTC

>M.04.0.37_A_92

TCGAAATCTTCCCTTTTAGCCATAAGGAGTAAAGTTTAGG

>M.04.0.37_A_93

ATTCACACCAATAGTTACCACGATGAGTCGGTACGTGAT

>M.04.0.37_A_94

TTTTAAACAAAATAAACTGTCCATCCTTCCTCACATTTCTT

>M.04.0.37_A_95

CAATATAAAACTCCCTACCCTGATTTGTTCATTATATTTAT

>M.04.0.37_A_96

TATTTTGACCATATAATGTATAGTAATTTTGTTCAAT

>M.04.0.37_A_97

GGACTTCCACCTGTAGCTAGAGTTCCATTGAATGCTTGCCAA

>M.04.0.37_A_98

GCGATAAACCGCTTAGTTTTTGCAAAACACTATTATACG

>M.04.0.37_A_99

TACCTTTTAGAACTACTACAGTAGCAGTATCACGGATACG

>M.04.0.37_A_100

GACTATGATATAGCAATAGTAGATTCGTATTCTAGTGATGG

>M.04.0.37_A_101

TCTCTTCATGAGTCCGAGAAAAAAAGGTGACAGATATGTC

>M.04.0.37_A_102

GCTGGTGAGGTGACATAATGAAAGCATATGTTTGCAAATTCTG

>M.04.0.37_A_103

CTGTGCTTGTGTTTGACGTTGCAAATGCACTACCTACTCCTA

>M.04.0.37_A_104

TATTTACACCGCCTTTCCCATTACCCTATCGCGGTTT

>M.04.0.37_A_105

AAAATTATAGATGCAAAAACTATAAAAGAGGAGATAATTCT

>M.04.0.37_A_106

AATCCTAACGTCGCTGATGTAATAAAAAGAGCGAAAGAG

>M.04.0.37_A_107

TATGGACTATACCTCATCCAGCCGTACGGAGGACCTTTAA

>M.04.0.37_A_108

AACTTTAACGAAGGAAGAATAGAATTCAATCAACAAGACT

>M.04.0.37_A_109

GATATTTTTTTGAAAGGAAAACTTCTCGCATCTACGC

>M.04.0.37_A_110

CAAATCTACAAGATACGTGGGTTACTATCTCACCGCG

>M.04.0.37_A_111

AAGACACTGCTTCAAACAGTTTCAACTGAGGTCTTCAAAACACCA

>M.04.0.37_A_112

CTTTCGTCACTGAATATCTCAAATCCATCCAGACCTCATT

>M.04.0.37_A_113

TTATATCTGTGATATCAACACCAGTAATTTTCTGTATA

>M.04.0.37_A_114

TTTGAAATAACGCGAAAGCGAGGGGTTATCATCCAGTCTG

>M.04.0.37_A_115

AATTTTATGACTACGCCACCCATACCTTTAGCACGTAA

>M.04.0.37_A_116

TTTACTGTGATAGAAACAATACCATTATTGTCATAACCTGC

>M.04.0.37_A_117

AACATCTACTGTTTGACTGTATGAAAATCTTTTACA

>M.04.0.37_A_118

TCTCCAAAGTCTAACGGGTGTTTTTATGATCGCCCGCG

>M.04.0.37_A_119

GTGGGGGTGGGGGAACCCCCCGCGGGGTTTACTGAACT

>M.04.0.37_A_120

CAGTTCCAGATAATTTAGCTCTTTTATTGTAATCATTTTT

>M.04.0.37_A_121

TCTTATATAAAATCAAAATTGTATGAAATCCAAGCTGAAGTTTTT

>M.04.0.37_A_122

AGACCGGTTCCGCTGACACTGTATAGCGTCTTTGAGCCTT

>M.04.0.37_A_123

ATTAATATAAATATGAATAATGCTATGAGCAACCCGACGTT

>M.04.0.37_A_124

CTTTTCATACAACATATAAATCACCTCGAAAAATAAAAA

>M.04.0.37_A_125

TTCGAAACTAACCTCATAATTACTGCATAGGCGTATCTCTG

>M.04.0.37_A_126

CCTCACTATATATGCTTTACAAAACTACCGACACGTATTATT

>M.04.0.37_A_127

TATGTAGTTTCTTAGCTTCGTTTAACCATTCCTCAG

>M.04.0.37_A_128

AAGTCGTAGCACCGTATGTACTCGAGGTACCTATAGTA

>M.04.0.37_A_129

AATGGGCTGTCGTAGACTATTCTTGCACCTGTACCAGATA

>M.04.0.37_A_130

ATATTTTTACTTCAAACATGAAGACCCATTATTACAACTT

>M.04.0.37_A_131

GATATCTTAACAATCCGACAGCTTTAGGTGCTCTAAGAT

>M.04.0.37_A_132

TTTTGTTTTCCCCATTTTTCACTATCTTCGTTGTTCCTT

>M.04.0.37_A_133

ATGTTACACTATATGCAATCTTTAAGAATAATTGCTATA

>M.04.0.37_A_134

TTATACTCAAACGGTTGCGTACCCCAAGGACGTAATTCTAC

>M.04.0.37_A_135

GTGACTGAGGCAACGGATAGTGTTACATTGCCTCCAAT

>M.04.0.37_A_136

ACCGACCATCAGTTTTAACATTAACCCAAATCAGGGCA

>M.04.0.37_A_137

CTGGTAATCATAATTGCGTTGGCAACGTTAGGGATTGGA

>M.04.0.37_A_138

TACCATATCCCCTCGTCTTCTAACAAAGGTAATACTTGGA

>M.04.0.37_A_139

TTCCTTGATAATGTAACCTCTTGACACCGCCTCAGTGATT

>M.04.0.37_A_140

TTTTTGGGGAACCTCTATCAATCAATCTATCAATCCGGG

>M.04.0.37_A_141

ATTACTATAGCATAAAGCCCTGTACCTAGTAAATACCCT

>M.04.0.37_A_142

TATTGGCGTTAGGCGGTAGTGCTATAACAATCCCTTACG

>M.04.0.37_A_143

TTTATTGCTTCATTTATCTCATTACAGAATTCTTCCT

>M.04.0.37_A_144

TAGTTATCGTACCGAAACCAGGGGTGATGTAATGCCCATA

>M.04.0.37_A_145

TTATAATTGAAATGAGGATAAATTCTTTCCTTAGATGTTTCA

>M.04.0.37_A_146

AGTCATGAATAGCTTTTCTTAACTCAGGAAAAGCATCGCT

>M.04.0.37_A_147

TTACCACTAACTGCAATAGGTGTAAACTTACGTGTTGCA

>M.04.0.37_A_148

GTCTTATTAGCGTGGCAAAGAGGAAACAATTTTGCCACA

>M.04.0.37_A_149

TGTCTTCCGGTTCGTGATGTATGTACGTGAGATTTGATAAT

>M.04.0.37_A_150

TCGGCTATGATTACACCGAAATAACCGTCGTCATTAAGT

>M.04.0.37_A_151

TTTACCCTTACCTCGGTGGTACTACCGAAAGTCTCTACCCCA

>M.04.0.37_A_152

ACTCCCACCTCGTCTCCACTACCACCCAACTGTATTCAAG

>M.04.0.37_A_153

CCTAAATCATTATCAAATTACGTAATTCCACAAAATCA

>M.04.0.37_A_154

GACTATTTCTTCTTTAAGAACTAATGTAGTATCATTTATC

>M.04.0.37_A_155

CCATATCCAGGATTTCCCTGTGGAGGCCACCAGAATG

>M.04.0.37_A_156

ACGTCAACTTGCAAAAAGTACAACTGATGAATTTGCCTTT

>M.04.0.37_A_157

GTTGGTTCTACTGTAAGCGTCCAATATTCAAATGGAACTTCT

>M.04.0.37_A_158

TTAGTACTTTTATATGATCGCGAAAGAACAAGGGAAAGA

>M.04.0.37_A_159

GAAGTTGCATAATAAGTTTTTGTTCCGTATACTCCCACTAT

>M.04.0.37_A_160

GGTATTATGGCAAACCTGGTATTCCCATGTTTCTCACTCT

>M.04.0.37_A_161

ATTTGATTTTGAACGTTCTCTTTCTGATGACAGAGCTA

>M.04.0.37_A_162

TTTATTATACAATATTCTATGGTACTCATGTTTTTCATTT

>M.04.0.37_A_163

CTCAGAATATAAGAAAATCTAATCTAATGGGCGGAGCTCT

>M.04.0.37_A_164

CGTTCAAAGCGTTTACATGAATTATTGTATAAATGTTGAA

>M.04.0.37_A_165

GTTTTGTTCGCTTCGTTTAACTTAAGAGAAGATTCAACT

>M.04.0.37_A_166

AGGTATTGAACAGGTGGCTCGTTCTCACCGGAAGGTAGTC

>M.04.0.37_A_167

TAATCCATGACGTTAACATAAAACGTCAGGAAGGACTGT

>M.04.0.37_A_168

CCTTATATAATTCGAGAGCTTTGCGAATTAATCTGTTTTG

>M.04.0.37_A_169

ATGATAGAGAATTTGCTAACTCATTCACATCTTGTACGC

>M.04.0.37_A_170

TTTGTAGGCCCAGCAGGAGCACCACTACCACTGGCACCTG

>M.04.0.37_A_171

AAAGAGATCGCGATGAAGTTCAAAGAGGTAATAACGGAGT

>M.04.0.37_A_172

AATTTGAATGCTAACAAGGCATTAGGAACTTGTTGCTGTCTTC

>M.04.0.37_A_173

TATCATAAAATACAAATTAGTTACTATCATTACATATAAG

>M.04.0.37_A_174

CCATACTACGTTCTTTTCCGTGTAAACTGGTTTTTTCTT

>M.04.0.37_A_175

GAATTAATTATGAGTTCTACCCTATCAAGCCAAAGTAAAT

>M.04.0.37_A_176

GATGTTTGGACGCTATGCCAGAGCAGTATTATTGCAAAT

>M.04.0.37_A_177

TTCAAAAAATTGTTCTAAGTTGTACTCTCGGTTGTTTT

>M.04.0.37_A_178

TGTTAGATGAATTTGCTAAGGTGAAAAATAATGACTGA

>M.04.0.37_A_179

GCAGATAATGATATATTTAACCCGGTGACTTGTGATATAA

>M.04.0.37_A_180

ATCGGATTAGTAACACTAGTGCTAGGATAAACTGAGGCT

>M.04.0.37_A_181

TTTAAGGAGATGGTTTTATGAAAATTGCAGATAAGGTCAA

>M.04.0.37_A_182

TACAACAAGGCAATTATCAGACCGCATTACAATATCTAA

>M.04.0.37_A_183

CTTGTTGGTGTCTTATATTGATATGCTCCGCTCCATTGT

>M.04.0.37_A_184

CTCAGATTTCCATAATTGATCGAATGTTTGAAGGATGGG

>M.04.0.37_A_185

GCATTCAACCCCGCGGGGGAATCCCCCGCCCCCATGGGGG

>M.04.0.37_A_186

ATATTAACTGTCTTCGTAAAAAAAGTTATAACTGGTATA

>M.04.0.37_A_187

TTAACATACAATCCGGGGCTACGGTCACTGTAGCCACTAA

>M.04.0.37_A_188

TAGAAATACTATAAATTAGACTTTCTAAATAGAGAATATATT

>M.04.0.37_A_189

GTGATTGGGTATGGCAATGGTTCTGTGATGGTGACATGT

>M.04.0.37_A_190

TTGTAATAAATATTGGCTACCATTATGATACTCTACTA

>M.04.0.37_A_191

ACTTCTTCAAATCGTTCAATTTCTCAGATAGCTCTTTCCATCT

>M.04.0.37_A_192

CATTTTAATCCACGTAATGAAGATATATTCAAATTTATTA

>M.04.0.37_A_193

TTAGGATCGGTTGCAATAAGAAGATTGGTAAGCAGTGGTTAT

>M.04.0.37_A_194

AAACTGCGGTGAACAATTGGGTTCAGAGAACACAAAGTG

>M.04.0.37_A_195

TAGAAGAATGTTAATATTAGCTAAATCTGATGAAGAGAATTA

>M.04.0.37_A_196

ACTTTCAGCCTATAACGGATGGACTTACACATCTTCTCT

>M.04.0.37_A_197

TTTGATAAAGCGATGACATTACGTATATACTTTTATGAT

>M.04.0.37_A_198

GAATAGAACTATAGCAGAAATAACAGCTTGCACTCCGAA

>M.04.0.37_A_199

GCATAACCTAGACCAATAACTGATGGCGTTGCTGAAGC

>M.04.0.37_A_200

CAATGTAAGTGAAATGAGGCCTGTAGATCATCTCATCAC

>M.04.0.37_A_201

AAAGCAGAAAACGGTAGAGGTTGTAAAGAGGCTAAAGTCA

>M.04.0.37_A_202

CCGAGACGCCCTGGCGAGGGTGATACACCTCTGCCCCACCCTTCG

>M.04.0.37_A_203

CATAATAAATTTTCGGTTTGTGAGTTTTGTGTACTTAG

>M.04.0.37_A_204

TAGCTTTACTTATAGGTGGAGGAGGGCCTAATAATAGCGG

>M.04.0.37_A_205

CAGTCAATGCGTCGAACGACAGCTCGACACCGACACGGA

>M.04.0.37_A_206

GCGTCCTTTAGTATCTTTTCTTGAGCTGGTAATAGTTTCTC

>M.04.0.37_A_207

TTAACTCATATATTGATAGAAAAGGAACAGTTACAACATAT

>M.04.0.37_A_208

TAGCCAAACAAGGCACTAATGACTACAGACCGGACGGTAT

>M.04.0.37_A_209

CGTGCTTTAAGGGAAGATCGAGCTGGATAAGCAAACGGGC

>M.04.0.37_A_210

AATTTCTCATGGCCAGGAGGCATAAGCACAAGTCACATC

>M.04.0.37_A_211

TCGAATGAAGGTTATCAGCTACAATTTACACCTTGGTT

>M.04.0.37_A_212

AAATGGTGTAACAATGACTATATATCAATTACTATTTGA

>M.04.0.37_A_213

ACCCGTTTGCTTTCGTACTTGGACTTGATAATAATAAT

>M.04.0.37_A_214

TTCTTTTCTCTGCTGACCAATCAGGAATTTATTACAAAA

>M.04.0.37_A_215

TTGGTAACCTCATCACTCACCGTTGTTAAAAATACGT

>M.04.0.37_A_216

ACGTTAAGACCATTTACAATTGCGAATAATGTAATTGT

>M.04.0.37_A_217

TTAAATGATCCAATAGGATTTGTAATTGTTGTAGAAC

>M.04.0.37_A_218

AATAGAATTTGTCCATTTACTGCGGAAATATTTGCTACTA

>M.04.0.37_A_219

TGATTTCCACTTCTTGAAGTGTGTAGTTTGTTTGATATCA

>M.04.0.37_A_220

AATATTCTATGAACGAGAAAATAGAGTTCCAAATAAAT

>M.04.0.37_A_221

TAAGAAACGCTATCGATACTTCAGAGGCCGGCGCTAGG

>M.04.0.37_A_222

GATTGCATTAAATTCTCTAAACATGCAGCGAAACCAGA

>M.04.0.37_A_223

ATTGTTTTCTTATTTTCTTATCCATCATACTTTCTTCCCC

>M.04.0.37_A_224

GTTCAACTACTAATACAATAACAAAATCTGATTTTTCCTT

>M.04.0.37_A_225

CAACGGGATGAACGTTAGTATGCGAACAGGAGCACCTTTGT

>M.04.0.37_A_226

ACGCTTAATGAACTCAAAGCGAAAACGGGAAAAACGAT

>M.04.0.37_A_227

AATCTTTTTTCTCATATGCAAAGGGTTATTTGTAAATCAAG

>M.04.0.37_A_228

ATCTTCATCTCCAAACTACTGCTATTAACTTATCTCACTC

>M.04.0.37_A_229

GTTAAAATATCAGTCCAAATTGAGGGCATCTATTCTTATAT

>M.04.0.37_A_230

CAACAATATTTACAAGCACTGAATCAATTAGGGAGTTCAAGG

>M.04.0.37_A_231

TAGAGACGGGAAAGAGGTTCAACACCCCATTACGGAATC

>M.04.0.37_A_232

AAACGAAATATCGAAGGCGTCAGATGAGGAAAGGAAGAA

>M.04.0.37_A_233

TTCTTGTAATGTCGTTATGTTACCATCATCTTTAATCTC

>M.04.0.37_A_234

GAATTAAATCAAGAAGAAAAAGAAGTAGATGAGTTTTTTT

>M.04.0.37_A_235

CAAAAAACGGGATAGACCCGGCAATTATCGCAAAGATCAC

>M.04.0.37_A_236

CGTTAGCATGCAATACGGGCCTCAAGGCGCTTATATCTA

>M.04.0.37_A_237

TGCAGTAAAACCGCAAAATGGACAAATTCCACCTGGTTT

>M.04.0.37_A_238

TTTGTTAATTTCCTCAATTTTTGGTTGAAGTTCTTTCGGCAA

>M.04.0.37_A_239

ATTTAAGCAAAACGCGATTCTTATGTATAGTCATCGA

>M.04.0.37_A_240

CTGGTGTGATGTCAGTTATTAAATATGATAGCGGTAAGT

>M.04.0.37_A_241

TTATTACTACGTCACCGCCAACTATGTGAATTTCGAAT

>M.04.0.37_A_242

TTTATCGGGGTCATCCCATGTCCGCACAAGTTAATTCAAA

>M.04.0.37_A_243

ATGTTTGCAAATTCTGTGAGTTTTCTACAAATAACGAAT

>M.04.0.37_A_244

TTCTTCGAACCTATATGCCCCCTTTATTATATTTTTT

>M.04.0.37_A_245

GATACAATTGCGTTAGCTAACGCTTCTAAACCGCTAG

>M.04.0.37_A_246

CCGCATAAGCAAATCTTGCTACTACCATCCCGCCTGCCCCA

>M.04.0.37_A_247

ATGGGACAGTTAATAGACGAGGCAGTGAAACTATTGGAGGT

>M.04.0.37_A_248

AAAATTGATTTTGATTTTTCTGCAGTTTGCGTTGGTTCCT

>M.04.0.37_A_249

GATAAAATGTCTTGAGCTACTATTTTTAGATCTTTGAAT

>M.04.0.37_A_250

ATATATGTTTCCAGGCCATACAATCGCTACGCCAACGAT

>M.04.0.37_A_251

AGAATTTTGGACTCTTGTAGATATGATTTGCGATGAATTT

>M.04.0.37_A_252

AATTTCTCATGGCTAGGAGGCACAAGTCACATCATAAA

>M.04.0.37_A_253

TTAGTTCCGCCAGTCCAGCCCCGAGAGCCGGTATTATT

>M.04.0.37_A_254

CTTCTAATTCTTCTTTTAGCCTAGATGCAATCAGTATCACAT

>M.04.0.37_A_255

AGCATTAGCGGGACTAGCGGAACAAGCACAGCGATAGC

>M.04.0.37_A_256

CAAGACATGCGTTATATCTAGCTTGTGTGTTAAACAGAG

>M.04.0.37_A_257

CTGATGAGACTTGAAAAACGAGGTTTACTGAAAAAAGTCGA

>M.04.0.37_A_258

ATCATTCTCAGATTAAATTCCCCTAATGTGCATAATAAT

>M.04.0.37_A_259

AATTCATAACTTTCGATACTTATCTCTTCACCCATGTT

>M.04.0.37_A_260

TGCTATATTTTTTACTCAGATCTTGTAACATTCTCTTCTCT

>M.04.0.37_A_261

GAAGCTGTTATCGCTTTTTTGCTTTTCTTTCGTTTCAGA

>M.04.0.37_A_262

AGTAGGATATCTTCTAGATTTAACCCATAAGGTGTTGA

>M.04.0.37_A_263

AAAGTTAGATTAGGTATAAAAGAAAAGAAAGCTATATAT

>M.04.0.37_A_264

GATCCTGTGACACTCTCGCTCGCTTCTGCTCCGCTAATCTC

>M.04.0.37_A_265

GTAGAAATAATACTAACGAAACCTCTATTTCAACTGGAA

>M.04.0.37_A_266

TAATAGATTTCGTAATAGTAGTGGCGGGAGTAATAGT

>M.04.0.37_A_267

TAGTATCTTAGCCCTAATCTCATTAGCGTTATAAGTCATGGC

>M.04.0.37_A_268

GTATCGAAAAGAATAGATGCCCATTCTGCAATGTAACGT

>M.04.0.37_A_269

GAGGAAGTTTATATAGCTATCTTATTAATACAGTTAGT

>M.04.0.37_A_270

GGCTGCACGCCCTACAGCCCTCGCTTGTGCTCTCACTGT

>M.04.0.37_A_271

ATAATCACCCTAATATACCAAATGATAAAGAAGTATGCT

>M.04.0.37_A_272

GTAATAGATAATTCCTCGTTTCTATCGTTGAAAGGATCG

>M.04.0.37_A_273

TGTTTTGTATACAGACTCACGAAGTTGTAGCCGTACC

>M.04.0.37_A_274

TATATTGGATCGTCATTAGGTGGTAGTGTAACATTAACATC

>M.04.0.37_A_275

AGAGTACTCCCTGCGTAAATTCCGCCAACAATAGATATT

>M.04.0.37_A_276

AAATTCAGATTAAATGCTAATTTAGTTTGTCCCGCCAC

>M.04.0.37_A_277

ACGAGAGTGTTAGGATCTTCCCATCCTACCCCTAACGGG

>M.04.0.37_A_278

TTTCCCATCCTTCATCAGCTTCTTCTTCCCTGCATAATAAA

>M.04.0.37_A_279

ATTGTTAGTTATGTCACTCTACTAACCACACCATCTTTCA

>M.04.1.4_A_1

AGGTGGTGGTGGCGGAGGCGGTGGCGGTGAATATTTATC

>M.04.1.4_A_2

CATGAACATTAATGTAGTTGAGGCCGACCTAACATACAAG

>M.04.1.4_A_3

GGTTGTAAGTACGCTACATGTATTGGAGCGCTAGATGCGGG

>M.04.1.4_A_4

CTGGAACTTTAACAGTTGTCCCGTTAGGCAATATATAGC

>M.04.1.4_A_5

CGGGTAGTAAAAAGTACGGAACATATCTACATGTCAAAAT

>M.04.1.4_A_6

ACTATATAAGTGAATTAAGGCAGAATGGAAAGACCCAC

>M.04.1.4_A_7

ATGCATAAACTCACTGAATAAATTAGGCGTAAATCCTGA

>M.04.1.4_A_8

TAATACTAAGATAACACCGACTGTTGTCGTTGATGTTGGT

>M.04.1.4_A_9

ATTCCCGACTAAACTCGGGATTTTATCGTGTTCTCTATG

>M.04.1.4_A_10

AGTACTTGACTAACCTTTTTTGCCTTGCATTGAATTTTTTG

>M.04.1.4_A_11

ACTCTTTGCCATAACTAATAAAATAGTCAACAACTTTCTT

>M.04.1.4_A_12

ACAAAAACATGAGCCAAATTCCACTCGATTTGGATATA

>M.04.1.4_A_13

TTTGCCTGCTTTTGGTGGTCCAGATAAGATTATTAACAT

>M.04.1.4_A_14

ATAGGAAAGTGAAAAGAGCGATACTAAGAGACGGCACAAA

>M.04.1.4_A_15

TAAACAGGTTCATTAATTGCTCTTCTTTTACCTCATTCCT

>M.04.1.4_A_16

AACTTATTATGTATTGTTACCATGTGTACATTTCTAGCG

>M.04.1.4_A_17

AAGACTAAAGCTATTTCATCAGCCGGAGGATTTGAAGCAT

>M.04.1.4_A_18

GTTTATCGCTCTGTTTTCTAGTCATTTCTCTAAAACTTCT

>M.04.1.4_A_19

TTTAGTTATAGCCTATGCGTTAAACAGGACCAGATAACCTT

>M.04.1.4_A_20

AATATCTGGATCATATATGCCTTAAGTTTTGGATTATATCT

>M.04.1.4_A_21

TACAATTTCGCTAGTTGTATCTACTACGACATCAACTCCA

>M.04.1.4_A_22

CAAACACAGCGATAGCAGGTGATAATAATACGACTACTG

>M.04.1.4_A_23

ACCGCTAGCTCCGGAAGGAACTATTATGTTTATCGTTTTGC

>M.04.1.4_A_24

AATTTCTCATGGCCAGGAGGCATAAGCATAAAAAGAAAC

>M.04.1.4_A_25

ATGATACCAACATCTTGAAACAACTCAATAATAAACAAG

>M.04.1.4_A_26

AGGGTACTACCAGCGTAAATTCCGCCTACAATTGATATT

>M.04.1.4_A_27

TCTTTTAATTCCTCTAATGATTGGACAATTTTACTATCGC

>M.04.1.4_A_28

ATTTCGCTCTCTAACTCTATTGTTCCAGTTGAGGAAACA

>M.04.1.4_A_29

TTCAACTTCTTTGTCTCTTCCTATTGTAGTTGATTGTTT

>M.04.1.4_A_30

ACTTTTCTGATATTCTGAGAAATAATGGAATGTCCCACAT

>M.04.1.4_A_31

TACACCCCTCGCGGGGGTCATCGCCATCCGCTTTTA

>M.04.1.4_A_32

TAAACCTAAACCTTTCCCTCAATCCCTTAGTGACCTCAT

>M.04.1.4_A_33

GTAGATCCAGATCTAAATACTGTATTAATATCAATAGTCGC

>M.04.1.4_A_34

CTTCCTCCAAAAGAAGCACCCTTGGTATTATTGTACGAT

>M.04.1.4_A_35

TTCATAGACATATTTACTTTCAATGCAACAAGTTCTGGAT

>M.04.1.4_A_36

TCATCCTTCTCGTGGCTATATATAAATCCACCGAAACCG

>M.04.1.4_A_37

TAACCATTCCCACGTAATCGTCCCCACGCTTAAATTCCAT

>M.04.1.4_A_38

ATTTCAATAAACTTAAAAGTTCTTTATCCAGAATCGATAA

>M.04.1.4_A_39

CCTCATCTTCATCAATATTATATTCTTCCTTAGCTGCATA

>M.04.1.4_A_40

TTTATCTCAAGAACTATAAAGTAGTAACATATAAAGA

>M.04.1.4_A_41

GTTTTCGACGGGGTAAATGTCTTTCCATAACCTGCCAT

>M.04.1.4_A_42

TTTTCAGAGGTATGTTATACATACCAAAAGATGCCATTATT

>M.04.1.4_A_43

CTTCAAATTGAATGCTTAATCGTTTATCTTTTTTATAAT

>M.04.1.4_A_44

TTGAATGCTTGCCAATTCGCAACGCCGCTAATCTGTGT

>M.04.1.4_A_45

GAGCTGGGCATAAGCGGATATAGGATTAACGTAAAC

>M.04.1.4_A_46

TGAACTGCTTGTGCAGAAGAATTGCTACTATCGAAACTG

>M.04.1.4_A_47

GTTAATTCATGGTGGGATTTTCTACCATGCCTTACTAGT

>M.04.1.4_A_48

AATTATATAAAAATAGCGATCGCGTTCGGAGCTGCGGCAT

>M.04.1.4_A_49

CTTGCTATTGCTTCTGCTACTAGTTCCCGTGTACCTTGA

>M.04.1.4_A_50

ATCAGAGTTGGATCGTTCTTTAGGTATTGGTGATTAAAA

>M.04.1.4_A_51

ACGCGTATAATAGTGTTTTGCAAAAACTAAGCGGTTTAT

>M.04.1.4_A_52

CTTTTTAACATTAATATAAATAACATAATCGATATCAA

>M.04.1.4_A_53

TTTGGTGGTCTCACGCCGTACTCTCTCACTAACTCTTTCT

>M.04.1.4_A_54

AACCCTATCAGAGTTACAACAATATTTACAATCTTTACA

>M.04.1.4_A_55

TAACCTTAGATACATCGCTGACGTCAGTAGATATGCAAA

>M.04.1.4_A_56

CAACATCCGCAATCAATAGAAAAAGTTCCGTGGCATGCTCCGT

>M.04.1.4_A_57

CATAAGAAAGTAGGCTTAAAAAATCATCTCTTTTATTAAGAA

>M.04.1.4_A_58

GCTACCATTAACCCTACTGACCATTGATAACCGTAGTCATAT

>M.04.1.4_A_59

ATTTAGTTACTGTAAACTCCCCAGTTTACGAAATAATTAAT

>M.04.1.4_A_60

TTGAAAATGATGATGAGCCTCATCTATCACAAATAAAGTA

>M.04.1.4_A_61

GTAGTGGGTATACTTTTAGGGAATGAGTAGTACCATT

>M.04.1.4_A_62

CCTGCAGGTAATTCTGCAGCCACTGGAACACAGATTAG

>M.04.1.4_A_63

TAAAGTAAAGTTTGCAGATGTCGAAGGTGCTTGGCTGAA

>M.04.1.4_A_64

ATGACATATTTACAGCACTAAAAGAGGCATTATCAGAGTTAA

>M.04.1.4_A_65

AAGCAACTAACGGATTGGTTTTTAAATACTTTTCAATTCCAT

>M.04.1.4_A_66

TAGTATCGTGGCTAAATAAAGCTAGGTATGAGGAGTGT

>M.04.1.4_A_67

TTTTTCACTTCCTCCAGCTTGGCTTCTAATTCTCTGACAC

>M.04.1.4_A_68

TTTACTAACCCTAACGGTAAGTTCACTATCACTTTCGGG

>M.04.1.4_A_69

TTTCCATCTTCAATGTATTCTCTTTTAAATGTAAGTAGC

>M.04.1.4_A_70

TTTTTAACATATCTTTTTCACTTTCGCTCAATTTAATT

>M.04.1.4_A_71

ACCAAAAGACGTTAATAAGCCTAGTCTGTTTGCTGTAAT

>M.04.1.4_A_72

TCTAAGATTTCCCCTATAGTATCTTGCAGTTCTTCTAATTC

>M.04.1.4_A_73

GAGTCTATAAATAATATTGTAAACGGTTTTATTTCTTTAGA

>M.04.1.4_A_74

TGCTGAACTGTTATTGACGTTGTACGATATACTGACAC

>M.04.1.4_A_75

TCCCATCCCCCACGCTAACTCGATATTACCATCATCTAA

>M.04.1.4_A_76

TACACAAAAATAGCTATCTGACTCCAGCTATATCCTAATT

>M.04.1.4_A_77

TCTTTTCATTCATTCTGCTTTCCCCTGATTTTATAGTAG

>M.04.1.4_A_78

TAAAATTCCTAAATTTTGCAATGCTTCTTGCAATAACAAA

>M.04.1.4_A_79

GAAAGATATTCTATTTGTTAGCCATCATAGCTGTTCCCGCGT

>M.04.1.4_A_80

CATGTAATAGATGCACAATATTGGTTTCCTGTAAATACGC

>M.04.1.4_A_81

TTTTTCATGCATTCTTCTATTTTATCAGATTGCAATAA

>M.04.1.4_A_82

AGTATGTCCCTATCAATTACATCTGGTATATCCTCAACCT

>M.04.1.4_A_83

AGAAGAGGAAAAACTTGTAAGTCAAAATTCAATTTATTT

>M.04.1.4_A_84

TAGAAATACGCTGTGACGCTTCCAAATGTTCCAGGCGGTAA

>M.04.1.4_A_85

CTTTATTCTGTTTGCCTTATCTCTGAATATATCTACTAG

>M.04.1.4_A_86

TGTATCGTGAAGGTCAGGATTATTTCGTTAGGCTCACAT

>M.04.1.4_A_87

CTGACCCAACCCTAAAATAACGTATAAGTTCGCTCTCAT

>M.04.1.4_A_88

GACTTTATATTTGAACAAGTCATAGACGATTTAACGTT

>M.04.1.4_A_89

CTTATTCCTACATAACCTATTTGAGACCATGGAAACGG

>M.04.1.4_A_90

GCTACGCTAACACTATCCGTTATACTGTACATGATTCAGT

>M.04.1.4_A_91

AAGCCCTGGCAAACGTTTACGCTTGAAGTGGGAGTTTCC

>M.04.1.4_A_92

TTTCAATTCTATAGTAGATTATCACGAGGCTTGTTGCAATAACAAA

>M.04.1.4_A_93

TTAACACTTAAACTTGAGTTATCATCATCAATTGATAAA

>M.04.1.4_A_94

TGGGATCCCAGATACCGTGTAATTTTTGCTGATATAATC

>M.04.1.4_A_95

AAGATCGTGGACGCGAGCACCGTAGACGAAGAGATAATCCT

>M.04.1.4_A_96

TTGAACGCTTGCCAATTTGCGATACCACTTGCTTGAGTTCCA

>M.04.1.4_A_97

AATGTTGTCTTTCCCGTGTTTGGTAGTGATAATTCGAA

>M.04.1.4_A_98

ATCATCAAGCCCATCAGTGTCTTTCCCGTCCCCGTAGGG

>M.04.1.4_A_99

GATATTGCAATATCTAAACGCTGAGAAAAACGCTCTACA

>M.04.1.4_A_100

CACTTCCTCAATCTCCTTTACATTATAAGTGACCTTG

>M.04.1.4_A_101

TAGGAACACTAATAGGTGAGTAAGTATCGAACCCTACAT

>M.04.1.4_A_102

AACTCCAGTACTGCTTCGCCGTAGGATCCGTCCTCAGACG

>M.04.1.4_A_103

ACTCTCAATAGAAAACTCCCTACCCTGATTTGTTCATCA

>M.04.1.4_A_104

AACAGAACGGCTAGTAACATTATAAATATCATTGCGATT

>M.04.1.4_A_105

CAACGAAACATTTTATACAGATATAATAGAAAAAGAATTAG

>M.04.1.4_A_106

GGCGGTTCTCCATCATCACCCCTACCATCCTCACCAA

>M.04.1.4_A_107

AGCCATCCAGGTCTGAAAATGACTAAGGGGATTATTTG

>M.04.1.4_A_108

TTTGCTAGCACATTAATGAGATTGAATATTGCTATATGTG

>M.04.1.4_A_109

TACTGCCTGGAAAATATATATGACATTCAAGAAAACATT

>M.04.1.4_A_110

TATACCTGAAAGTAATGAATAACGAGTTAGCATATAAT

>M.04.1.4_A_111

CCTATACTAACAATTATTGACAACGACACATTACAAGAAGA

>M.04.1.4_A_112

TCGTAAGAACGATCCTAAAGTTTGTTATCGTAAATCCA

>M.04.1.4_A_113

TAGGTAATTCTGGAGGATCTGGCAGATTGGGCAAGGGTTTCT

>M.04.1.4_A_114

ATCCCAATAGCTCGAAAAACCCCTCACTTTCGTTAAACTT

>M.04.1.4_A_115

TATCTATGAACTCTCTCATGTTGTTTATATACTCCACA

>M.04.1.4_A_116

ATAATCGTTCAGGGTGGCAACCCACAGTTCGTGATACAA

>M.04.1.4_A_117

CTTGTTCTAACGTCGCATATGCAGAATCTATTAATCCGCGT

>M.04.1.4_A_118

ATTAAACCAAAATGTGCCTTGAGCCAACTTTATTACAAT

>M.04.1.4_A_119

ATAAATGCTTTTGTTGAAAATTTTCTTGAAAATTCGCTACATCG

>M.04.1.4_A_120

TTTACGACAAAGTTACCTAGACTTTGAACTGCATTTGTTAAT

>M.04.1.4_A_121

ACAATTATTGTGCCGGGTTTAGCATATTCTTCAACTAT

>M.04.1.4_A_122

TCTTATCTCCTCAAAACCTAGCGATGTTAGCTACAACAGC

>M.04.1.4_A_123

TCATTTGCATAAGTCCCTAGGGATTTCATGCTAAATGTA

>M.04.1.4_A_124

ATTCCAGTATTCTTTGCACATCTCTCGTAACTTTGCTTA

>M.04.1.4_A_125

CAAACAGCTGTTCAACAACAAACAGTAACGCAACAAGCCTCGT

>M.04.1.4_A_126

ATAAGAAACACTTCAAAAAGTGTTATCATTCTTCCTCA

>M.04.1.4_A_127

CTCGTTTCTCTTTTTTCTCTTTTTATGCTCAAAAGGGAAC

>M.04.1.4_A_128

TTTTAATATAGTCCTCGACCATAAGTAGAATCTCAGCAAT

>M.04.1.4_A_129

GGATCTATTACAATTAATGATAACTCAAATGTAATTTCT

>M.04.1.4_A_130

TCAAATAAAGATATAACGTTGATCCGCTTCCAGTGATT

>M.04.1.4_A_131

AAGAATTCCAGTTCCCTACCGGTGTAAGGATTAATCTTT

>M.04.1.4_A_132

GTCGAATTTCCATCTCATCAGGAACCAACGCAAACTGCA

>M.04.1.4_A_133

ACTTTCGTTTAGATTTACCTAGGGAGATTCAAGAAAAGT

>M.04.1.4_A_134

AACCGGGCCTCTCTCATTTAGAATGACGGATGCTTCTTT

>M.04.1.4_A_135

TTCTGTTCTGTGTTGTTCTGTTCTATACTCTGTTCTCTG

>M.04.1.4_A_136

TCGTTTGTTTGACCAGCACTGAGATTAACAACAAATCTG

>M.04.1.4_A_137

TTATAAAAGCAATCAAAAAGAATGAAACTACGTCATTCA

>M.04.1.4_A_138

CTTGATTCTTTCTTCTCCCTTTAGATTATCTAGTTTT

>M.04.1.4_A_139

TATTCTGTCTCTCAAGATTCAAGATTTGCAAAACACTTA

>M.04.1.4_A_140

TACGGGCTATATATTATTCAACCATATGGCGGGCCCCT

>M.04.1.4_A_141

TACAATTTCGCTAGTTGTATCTACGTTGTTAAACACTAC

>M.04.1.4_A_142

TATTGGAGTACCCTAATTTAGATAGCACAATTTATGGATC

>M.04.1.4_A_143

AATCACCCCTTGTAACATCCTCATTACGTCTTCTGCGTTAT

>M.04.1.4_A_144

CAGCACGCTGGAAGCTATGGCGTCAGCCTAAATACATTCC

>M.04.1.4_A_145

CACCCCGTAAGGGGGTCATCGCCATCTGCTTTTATGG

>M.04.1.4_A_146

CAATATAAAATAATAATAAAATTGAAAGGAAGTAAGTGCG

>M.04.1.4_A_147

TTTGCTATTATCTCTTCTTCTTTTAAGTTGAGTGTAAG

>M.04.1.4_A_148

TCTTTATTGCATTCGGAGAAATAGCAAAGTATATTTCACT

>M.04.1.4_A_149

TCTACGGTTTGTTCGCTCCAACGCTCGTGCAGAAGGCG

>M.04.1.4_A_150

AAAAAATGGTATGTAACGTGGGATTAACTATGTACAAATGT

>M.04.1.4_A_151

AAATACCTGCAGAGATGCTAGAATGGGAAACTATGGAG

>M.04.1.4_A_152

ATGAGTAAAAATTCAAGACCAGAACGTGAATCTGCTAA

>M.04.1.4_A_153

ACGATTGTTGAATAGATGTCATATATTGTAATAAAGGTTTT

>M.04.1.4_A_154

ATAACAATTCCTAGCTCTTCATCCTATATACCGTCTGTT

>M.04.1.4_A_155

CCCTATTAGTTCATCAGTATCTTTATAACCACTGCTTACT

>M.04.1.4_A_156

CTCAGGCAGAGCAAAACTTCGCTACCGCTATGCAACAAGT

>M.04.1.4_A_157

TTTTCACAGAATCCCGAGCAGTTGGCAATCGCAGCAGCT

>M.04.1.4_A_158

ACAAGTGTTATTGATATCGGTGCATATGTTGGCGATACTGC

>M.04.1.4_A_159

TAAAGTATTTGAATTGGAGTATAAGGGTGACTATGAAT

>M.04.1.4_A_160

TGTAACTTTACAGTAAGAAGCTGGATTAGGATTTCTTCTACAT

>M.04.1.4_A_161

TTGAGTTATTGCGTAATATCCTTGTCCGCTTGTGTGTGT

>M.04.1.4_A_162

ATACTAAGGGAAGAAGAGAGTAATGAGGGGAAAGTTCT

>M.04.1.4_A_163

ATACTAAGAAGTAACGTAACAGCTTCTGAGAGAACAAAG

>M.04.1.4_A_164

CATGTAGTTGACGCGTTTAAGCAATTCTGGACTAAAGTCCC

>M.04.1.4_A_165

TCTTCAATGCATTTATTATTTCATGTAATACATTATAT

>M.04.1.4_A_166

CATGCAGAAAGTGGCTTAGGTCTGTCATCATTTGCTTCGACGT

>M.04.1.4_A_167

AAAACAGCTGTTTAGGACAGCTTTTTTTACACCTTTTTT

>M.04.1.4_A_168

ATAGAACCCGAAAAAGTCACATACGGCGGTCAAGAAATT

>M.04.1.4_A_169

TACACGGTATTTATTACTGCCTTTATTGCAGCAACTAACCTT

>M.04.1.4_A_170

ATTACATGTCTACTTAAATTTCCAGTTCCATACGCCACCGCT

>M.04.1.4_A_171

CAAGGGGCTAGCGGGTGGTATATTAAATATGAAGTATTAT

>M.04.1.4_A_172

ACTAGCTCTAGCTCTGCTGGAGGATTTAGTGGCTCAACGCTAT

>M.04.1.4_A_173

GCGACTCCTCTAGGTGGCAAGGTCTTTCTAATTTGATCTAT

>M.04.1.4_A_174

AGCAATGTAATTTATAGTGTAGTAGTGGAGGTCGTGTTTAC

>M.04.1.4_A_175

AGCCCTAAGGCCAGGGCTTCCGCGTGCTGTTTTAGTTCAT

>M.04.1.4_A_176

GTACCTTATTTCAACCCTAGAAATGAGGATATATTTAAA

>M.04.1.4_A_177

GTAGTTCCAGAAAGCGTAAAAATGAAGCTACTAGACGAA

>M.04.1.4_A_178

CTAAACACCGCGTTACTGTATCCTTTGTTCCTAAGT

>M.04.1.4_A_179

ATTCTTATATTGTCTGCATCTAGCACTTTCGTGAATTCATCAT

>M.04.1.4_A_180

TATCTCTCTCAACAAACACAGTCCCCAACTTCCTAGT

>M.04.1.4_A_181

TGAGAATAGACACTTTAATTTAGGCATTATTTTAGCATCTC

>M.04.1.4_A_182

ATATATTGATGAGATTGAATATTGCTATATGTGTGTAATACGG

>M.04.1.4_A_183

TTTTTGCTGCACTGTTGGAATTTGAAATACATTCATTATTT

>M.04.1.4_A_184

GAGACTTGGACTAAAGAAGATTTTGACGCTTTGATATTA

>M.04.1.4_A_185

TTAATGTCAGCGTTTATTTTCTTTCCCGTTTTCTCGC

>M.04.1.4_A_186

CTTTTAGGCTATAAGACTCATGCAAATATACAGAGAAGA

>M.04.1.4_A_187

TTTCTCTCTATGTCATTTGTTCCTTATAAATGTTTCTCTCT

>M.04.1.4_A_188

TGTTAAGATTGTACGTTCCTTCATCCTTCTTCATATAAATA

>M.04.1.4_A_189

TTTGGCTTCAATAGCTCGGGATGATTCTCTTTAAGCCAT

>M.04.1.4_A_190

AGTCTTGCGTCAATACTGATTTCATGAATCAAACTCA

>M.04.1.4_A_191

TAAAGATAAGGGAAGATATTTTTTGAAAGGTAAACTT

>M.04.1.4_A_192

GTGAACGCGACAACTGGCGCACTTATGCTGGTATTCAT

>M.04.1.4_A_193

TGCACATGTCTCAAGACTATTGTACTTTTGCTTGCAAAGAT

>M.04.1.4_A_194

CTACGAATTTAACGCCCGCCCTAGATATCAATGATGCCTCGG

>M.04.1.4_A_195

AATATAGACAAGAAGGCATTTTTTAACGCTGAGGTGGT

>M.04.1.4_A_196

GGTTCTCGTTAAGATAGGAGACATTCCAAACAATGACGG

>M.04.1.4_A_197

AACCCGCCTAATCCGTTTGTCTTTAAACTCGGACTTGAT

>M.04.1.4_A_198

CCGAGGTGTGGCTTCCCCTCCCGCATCGCTCTCCTTCGG

>M.04.1.4_A_199

TATCGGTGCCTTAGCGTCAAACGTTGAAACAATATTAGC

>M.04.1.4_A_200

ATAACTGCGTATCCACTATCGCCATTCGTAGTCAGAAAT

>M.04.1.4_A_201

TTTACTTTCAAAAATTTGTTCTTGACAGGTCAGAAAA

>M.04.1.4_A_202

CCTACCGGCGACGGCGGGGTAGAAAAATATAAGTCTCCCC

>M.04.1.4_A_203

CATTCTTTCCCGCCTTCTCATCTTCTATCTTTTTCCTTACTG

>M.04.1.4_A_204

TCATGGGCTTATCACCTCATTCTTCCTCCTCTTCGTCTT

>M.04.1.4_A_205

TTGAATATGAGAGATTCACATTCTTTGTACGTGTCACC

>M.04.1.4_A_206

TGCGATTTCGCCGTTTGGCTTTAATAGCTCGGGGTGATTCT

>M.04.1.4_A_207

TCTTACCTAAACGGGATGGATCCGCCGATGGTATTTCAAT

>M.04.1.4_A_208

ATCATACTTCTCACCATGATGTGGGTAATGTCCACCATTT

>M.04.1.4_A_209

ATAGAATGAATATTGCACCGTCTTTACAACATTACCAAA

>M.04.1.4_A_210

TACTCTCGCAAGCTCCTCCATTGCTTCCTTTGTTTTCCCTT

>M.04.1.4_A_211

GTTGCCGCAAGAGTCTCTTCTACTACGACATCAACTCCA

>M.04.1.4_A_212

ATATCTCTCTATGAAATTGTACTACTACATCTTCCTCACT

>M.04.1.4_A_213

GCTGCCTGCAGCATCCTCAGTAAGTTTATATTCGTTATCT

>M.04.1.4_A_214

TTATGTTTGATTGCCTTTTGAATCCACCTCTTCCCTCTAGCCAT

>M.05.0.1_A_1

TTTTCAGAAAATGACATGCGAAAGACGATTAAAAACGCC

>M.05.0.1_A_2

CCTCCTCCAAGACCACCCAAACCACCGCCTAAACCACC

>M.05.0.1_A_3

TCACTCTTTCCTCCTTTTTCATTTTGTTCTGTGCCCAA

>M.05.0.1_A_4

CAATCAATCGTAAACGCTAAATCAAACTCCTAACTTTATC

>M.05.0.1_A_5

TTAACACTTAAACTTGAGTTATCATCATCAATAGATAAAA

>M.05.0.1_A_6

CCCGTCGCCAACGTTCCGTTGAACGCTTGCCAATTTGCCA

>M.05.0.1_A_7

AAATAAAGTGAGCTAAATGGCGGCAAATCAAGAAGTATG

>M.05.0.1_A_8

TTCTTATTGTATTTCATGATGCCTTGGTTTGCAATATCT

>M.05.0.1_A_9

ATCCCATTACGATAAAAACTAATATAATATGCAAAATAGT

>M.05.0.1_A_10

AATTAACTACGGACGTTATAACCGACGGCGAATAGTC

>M.05.0.1_A_11

TACAATTAATGCAACAACTATAAATACTATTACTAAGGG

>M.05.0.1_A_12

ATTCACACCAATAGTTACCACGATGAGTCGGTACGTGAT

>M.05.0.1_A_13

TATTTTAGTGCGTATCTATCTAGGAGTTGTAATAAGTCG

>M.05.0.1_A_14

CCTAAAACCCCCAATGCTAATTATGATGAATTGAAAGGA

>M.05.0.1_A_15

TAAAAGGGTTAATGACAAACTGGAAAGCTACCGTGCTCAATTTC

>M.05.0.1_A_16

ACGTATGATTTGCCGGTTTTCTCTATCAATTCCATTCAA

>M.05.0.1_A_17

ACCGACCATCAGTTTTAACATTAACCCAAATCAGGGCA

>M.05.0.1_A_18

TCTATTAATTAATCTGGGATTATCTCCCTTATATAGGC

>M.05.0.1_A_19

TGATTAAAATAGTTAGCTATCTCGGTAGCCGTTGTTAG

>M.05.0.1_A_20

TAGCAACTGCATTTATTAACATGTTTAAAAATTCTAAA

>M.05.0.1_A_21

TTTTGTTTTCCCCATTTTTCACTATCTTCGTTGTTCCTT

>M.05.0.1_A_22

TTTTCTATCAGTCCACCGCAAGGAGTTACTAGAACAAT

>M.05.0.1_A_23

ACAACGTGGACATTGATATAATAACGCTCCAGTGCTTTCAT

>M.05.0.1_A_24

ACGCTTAATGAACTCAAAGCGAAAACGGGAAAAACGAT

>M.05.0.1_A_25

AATGCAGAAAATGTACTTTCATAGTAGACAGTAGAACTA

>M.05.0.1_A_26

GTACTTTCATCTCTTTGTCGTAATACTAAATGATACAT

>M.05.0.1_A_27

TTAGTGACCTCATCGCTTACAGTCGTCAAAAACACATT

>M.05.0.1_A_28

CTAATCAGTTCAACCCCACAGAGTCCATCCCCGACTCTGC

>M.05.0.1_A_29

CGTGGAGTGAGATGCTGTCAGACAATGGGGATAATGGGCC

>M.05.0.1_A_30

TTTTATTTATACTTGAGTATGGTAATAGTCCTTTTTCGA

>M.05.0.1_A_31

TCCATATTACTTCTCTTCCTCTTGTTTCAGTATCAATAT

>M.05.0.1_A_32

CCATTAGATTTGTCAGCTCCACTATTTCGTGGTCTCAGA

>M.05.0.1_A_33

TTATTGTTGATGCTAGAAGTGTAAATGTTGAAGGTAA

>M.05.0.1_A_34

GCATACATATTCGCTCATTTTAGTAACCTCCTTTTTATTTCA

>M.05.0.1_A_35

CTATTTCGTGGTCTCAGACATCGAGTTACAACAATATTAT

>M.05.0.1_A_36

AATTAATAGGAACGCGGTGAGACTGTTTGAGCCAAC

>M.05.0.1_A_37

ATGAGTGTTGATGAATTCGAAGAATATCAGAAAGAACTTG

>M.05.0.1_A_38

TTGAAACTTCCTATCGGATTAGTAACACTAGTGCTAGG

>M.05.0.1_A_39

TTTAGTTATAGCCTATGCGTTAAACAGGACCAGATAACCT

>M.05.0.1_A_40

ATTAGTGTAAAAATTATTTTACTAGTAATTTATGAGATAC

>M.05.0.1_A_41

AAACCGAAGCCGGCGGCACCCATGCCAAGTGCAAAAACT

>M.05.0.1_A_42

TATCCCCTTCGCGATAATAACTCTTGCTATTAACAAGTCGT

>M.05.0.1_A_43

GCACTATTCTCAGTTAGTATTACACTGAATGTAAATGGG

>M.05.0.1_A_44

AAATTCTTTGTACATAGTGATGCAGACATTAAGGTAGCAAT

>M.05.0.1_A_45

TTTTTGATTCACTTTTCAAATAGTGATAAGTGAAATCT

>M.05.0.1_A_46

TCATCTAAATGTTCTTCTGGGTTATAACGATTCTTAA

>M.05.0.1_A_47

TACATGAACATGAACAGAATGAATAAAGAAGATTTCAAAAT

>M.05.0.1_A_48

TAAATTCCAAAATCAAAAAAATGATTATAAAAGTAATCA

>M.05.0.1_A_49

TTCAACAATGCATGGGTTATGGAAGGTACAGTAACCA

>M.05.0.1_A_50

CAATATAAAACTCCCTACCCTGATTTGTTCATTATATTTAT

>M.05.0.1_A_51

TATCCTTATCAAGAGGAGGTTATCAACAAGATTCGTAACGCT

>M.05.0.1_A_52

ATAAACTTCGGTTATAGTTTTACTGGTATAGATGGGTTAT

>M.05.0.1_A_53

GGTTTTCGTCATCCCGTTAGTCATTGTCGGGAAGCTCGT

>M.05.0.1_A_54

GTACTTGGTCATTGGGTGTTTTTGTTATTGATGTGTTT

>M.05.0.1_A_55

CCTCAGGTACGGCAAGGACGTTGATGATAAGTTTGTC

>M.05.0.1_A_56

GAGGAATAGCCTAATTCAACATGAGGCATAGGAGGCACAA

>M.05.0.1_A_57

ATCATTTAGTTACGCTTTTAACTGACGTTAAGGCGATT

>M.05.0.1_A_58

TTTCTCGTTTCTCGCTTTGAGGGACTGAAGCCTATCGTT

>M.05.0.1_A_59

AGAGCTAAAAGCAATGACTTATTTTACGCGCTTAGT

>M.05.0.1_A_60

GATTAAACTGATGAAGAGATCTTATGAAGAGAACGGCTT

>M.05.0.1_A_61

TTTACTTTCACTTTATAGCTCAGTTCTCTGAATCTT

>M.05.0.1_A_62

TTAGCACGTAAACGTCCTATTGATGTTGTTATCTCTTT

>M.05.0.1_A_63

TTATCTGTACATGTGCAGATGCAACTTTTTGTTTGGGTAT

>M.05.0.1_A_64

CCTAGTTTCAATTAACTACTCAGCTATTCACACTCTGACT

>M.05.0.1_A_65

CTATCAGTTTCATCTATTATAATTGCTGGTAATGCAAAAC

>M.05.0.1_A_66

TATCTATTCCCCGAACTTTCCGAGATCGAGGAAGCGATTT

>M.05.0.1_A_67

ACCTCAGTCTATGAACCGACTAGCATCAACGAAACTAGCG

>M.05.0.1_A_68

TACATGGGCGGAAACCCATATGGATTCGGCTTAGCCCA

>M.05.0.1_A_69

ACTCAATTATATCAATCACTACAATATGTAATTTATTTT

>M.05.0.1_A_70

GATATCTTAACAATCCGACAGCTTTAGGTGCTCTAAGAT

>M.05.0.1_A_71

TCCTGCGATTAATGCAACAACTACAAATACTATTACTAA

>M.05.0.1_A_72

TTATACTCAAACGGTTGCGTACCCCAAGGACGTAATTCTAC

>M.05.0.1_A_73

ACATATTTAAGACTATTAATTAAATTAGTGCCAAAATCTA

>M.05.0.1_A_74

CTGTAAACACTAATTCTTTTTACGTACTACGTTATGT

>M.05.0.1_A_75

GCTGCTTGTAACATTCTTAATAAATTTATATTTGTTATC

>M.05.0.1_A_76

GCATCTGTTATTAGTATTATAGTCTTTATTCTTCTCAAGT

>M.05.0.1_A_77

TTGCACCTACGAGTAAAGCACCTATTATCAGCCCAGGGTT

>M.05.0.1_A_78

GATACAATTGCGTTAGCTAACGCTTCTAAACCGCTAG

>M.05.0.1_A_79

GTGAAATTGTACTACTAAATCCTCCTCACTCAACCGGCTCCA

>M.05.0.1_A_80

AATAACGTCATGCCTACAATAAGTAGCATCGGTCTTTA

>M.05.0.1_A_81

CAAATAAATGCTATATTTAAACGGCTTATTTACTTTGTTT

>M.05.0.1_A_82

ACGCTCTCATCTTGCTCAAATTTATTCTCTTCTGGTAGT

>M.05.0.1_A_83

GAAGTCTTTCCCCATCACCATTATGTCACCTATTCCAACT

>M.05.0.1_A_84

TATTAATAAGTTTCCGCACTGGAAAAGTGATATCAAAC

>M.05.0.1_A_85

CTGCATCAACTACAGTAGCGGTGCCATTCTTTACACGCT

>M.05.0.1_A_86

GAACCATAGGCAACCGCTTGTCTATTATTCTTTAGCTTCAT

>M.05.0.1_A_87

GCATTCAACCCCGCGGGGGAATCCCCCGGCCCCACGGGTG

>M.05.0.1_A_88

TTTCTGGGATTTTATCTATCGCGAACATTTCACTTAG

>M.05.0.1_A_89

ATGATAGAGAATTTGCTAACTCATTCACATCTTGTACGC

>M.05.0.1_A_90

TTCGGGCTTAGCTGGAGGTGGTTGATGTCTGCTTCAGTCG

>M.05.0.1_A_91

TAAAAGTGTTGATTAAGCCTTATGTATGTATTATTGCAA

>M.05.0.1_A_92

AATTTAGATAGTACAATATATGGATCGATATTTTTAAGC

>M.05.0.1_A_93

GAATTAATTATGAGTTCTACCCTATCAAGCCAAAGTAAAT

>M.05.0.1_A_94

TTAAGTTTTTGTTCTATACCGTCTACTTGTTGTTCTACG

>M.05.0.1_A_95

ACCCTAAGATAAAAAAGTAGAAGTGAAAAAGCTAGGAGA

>M.05.0.1_A_96

TACAACAAGGCAATTATCAGACCGCATTACAATATCTAA

>M.05.0.1_A_97

TTTTCGTACAAAGTAATACTAGACGGCGTATTATAAGACC

>M.05.0.1_A_98

CAGGGTGTCGCTGCTGCGCAACCCAAGATGGCTGCAGTA

>M.05.0.1_A_99

TTTATCAGATCAATGCTTGGCAATTTCACTGGTAGTCCA

>M.05.0.1_A_100

TTCACTGGTGTGGCAATTAAAGCTGATAATGTGTCTAGTT

>M.05.0.1_A_101

AATCTAATTCCAGGGTTTTTTTGCATTTTGTTTTCCCCATA

>M.05.0.1_A_102

CCCTCGCCTCCTTCTTTTTCCTTTTTTAAGTTGT

>M.05.0.1_A_103

GTTTATTTTATTAATTCTTCTCTTCCCTTTAACCTATA

>M.05.0.1_A_104

TTTGTGGCACCCAAGATTATAGTCGCCGAGGTGGTATT

>M.05.0.1_A_105

CAATACATATGTCGGCAACACCTTATCGGTGAAAAATAA

>M.05.0.1_A_106

TCTCATCAGGAACCAACGCAAACTGCAGAAAAATCAAAAT

>M.05.0.1_A_107

GTCGGCACAACCTCGTTAGTTATTAATTGCCCTGCCC

>M.05.0.1_A_108

AAAGCAGAAAACGGTAGAGGTTGTAAAGAGGCTAAAGTCA

>M.05.0.1_A_109

TCAGCCATGCCAACAAGAGATGTCAGTCCACCACTTTG

>M.05.0.1_A_110

GTGGCTGCAGCACCTGCTGCAGGTGCCGTTCCAGTGCCG

>M.05.0.1_A_111

AATGATAAAGTTATAACAATCCTAGGATATCCATGCTATAGT

>M.05.0.1_A_112

AATTCTCTTAATTCTACTATGACCTCATATTTTTTCTTCATCGT

>M.05.0.1_A_113

ATTCCCTTTGCTCTTAATCTTCCAACAGATGCCGTTATTTC

>M.05.0.1_A_114

TGATTTCCGCTTTTGCTTAGGTGTAATCGGCTTTTAAT

>M.05.0.1_A_115

CCTAAATCATTATCAAATTACGTAATTCCACAAAATCA

>M.05.0.1_A_116

ACTAGAATTCCCATTATAAAAGCAATCAAAAAGAATGAAA

>M.05.0.1_A_117

GTCTCCATTAAATATCTTAAAATGAATGGCGGTAATGGTC

>M.05.0.1_A_118

CAACAAAATCCAATTACTTCTCCTCAAATTCAATCAACAA

>M.05.0.1_A_119

TTTCCGCAACGCAAAGCTTCATTCCTAGTAATTGTGAG

>M.05.0.1_A_120

GTTAAAATATCAGTCCAAATTGAGGGCATCTATTCTTATAT

>M.05.0.1_A_121

ACTGGACCTCATTTCCCGCTCACCCAAACCTTAAG

>M.05.0.1_A_122

CAAGATATTGACTTAGAGTATGCTAAAGCCATTGAGATATGT

>M.05.0.1_A_123

ATGCAGTAATTATGAGGTTAGTTTCGAAAGGTGTTTTTA

>M.05.0.1_A_124

GCAGGTTGGTACTATGAATTCACTGGTTCTAACGTT

>M.05.0.1_A_125

AAACATTCATGCAGTAGATGGCAAAGAAGTAAAACAACT

>M.05.0.1_A_126

AGTATTATTGTTACATTACTCTTAAAGTTGATATACGTCTT

>M.05.0.1_A_127

AAATAAGTGGTAAAAGATGGCGGCAAATCAAGAAGTATG

>M.05.0.1_A_128

TATATAATAAGCATAGCTCTTCGTCACTCAGATATTTATA

>M.05.0.1_A_129

TGTACTGTATGCAGCTTACCTGTTCCGACATGGGATAAA

>M.05.0.1_A_130

TAATCTCTGCAGGCAATTACCAAAAAGTGGTAATAGACG

>M.05.0.1_A_131

GTAGAAATAATACTAACGAAACCTCTATTTCAACTGGAA

>M.05.0.1_A_132

AAACTGATGGAACTGATATATTAGCTAAGGGCAAGT

>M.05.0.1_A_133

TCTAACTCATTTACATTTATGGGCATTTTTTCACCCTTATT

>M.05.0.25_A_1

ATAAAGAACACGTTTGTATCCCGACTTTACTAGGGAACAA

>M.05.0.25_A_2

CACAAATTCCCAGAAGCTTAACAATAGTTTTGAAAGCGT

>M.05.0.25_A_3

AAACAGTGCTAGGCAGTGGTTGTTCTATACAGATCATATA

>M.05.0.25_A_4

TACTGTTCCGTTTGCTGTATTCTGTATAACAATCCCGT

>M.05.0.25_A_5

TTTAAATAAGGTTTAATATGAACAATTTAAAACACATAACAT

>M.05.0.25_A_6

TTAAGTACTGTATTACCTAGATCAGTTAATAGTGAAATT

>M.05.0.25_A_7

CCTGCTCCTCCACTACCTGCTGCACCTGAAGTAGCGTT

>M.05.0.25_A_8

CACATATATTGTATTTGCATTCCATTTTTTGCCTTGAGA

>M.05.0.25_A_9

CCCCGTGAAGGGTGGTTGCCCCGAGTTCATGAATCCCT

>M.05.0.25_A_10

ATATGGCTAGCAGGGCTATTAGGGACTCGAAAATAGTG

>M.05.0.25_A_11

AGTTAGATCAAACTCGGGAAGCAGTAAGAAAAGAAGTGTT

>M.05.0.25_A_12

GGCTATTTGGACTTTGAAGCGTAGACAATTATGAG

>M.05.0.25_A_13

AATAGAAACACTGCTTAAGCTGTTAGATGAACTAGACA

>M.05.0.25_A_14

GTAACGCTGACGTTAGTTATTACATATCCGCCCTGTATCGT

>M.05.0.25_A_15

TCAATAGAGTTCTTAAATAATGCAGGGTAATCAACAGC

>M.05.0.25_A_16

AAACTAATTCTTTTAAGCTTAAGTATTGGGGCAACCAGA

>M.05.0.25_A_17

TAGATTTAGATATAGATATTATTCACGTTTCATGCATGTA

>M.05.0.25_A_18

CGAAAGGATTCTTTCTATTCCCGTTATCAGTAAATACACT

>M.05.0.25_A_19

TTCAGTGGACAGGTCAGTGGACAGGCGCAAGAGACTGCT

>M.05.0.25_A_20

AAATAAAGTGAGCTAAATGGCGGCAAATCAAGAAGTATG

>M.05.0.25_A_21

ATTTATTGTGAGTGCGGACTTTCAAATAATCTAGACGT

>M.05.0.25_A_22

TAAAATTAGTTACAGCGTTCTCTACATCGTTAAATAGTCCT

>M.05.0.25_A_23

ATATTTCCGCTATTTGGATTTATAACGTCTATAATGAG

>M.05.0.25_A_24

GCTATCACTGGTATCTTGTTCTCTCCAGATTGAAGATTA

>M.05.0.25_A_25

GTCTGTCCGTACACTCTTTTATCATGTCATAGATTTTTCTC

>M.05.0.25_A_26

GGGCGGGTACCCGCGCCCGGGGGGCGAGCACACCCGGGTACAA

>M.05.0.25_A_27

TTTATAGAATATATATATGTACCATTTGGTATTATACTAC

>M.05.0.25_A_28

ATCTATCATCTGGATAGAATACTGCAGAGAACGTATCACT

>M.05.0.25_A_29

TTTTGATTGATTTTGCCATTGCAAAAGCCAATTTGCT

>M.05.0.25_A_30

CCTCTTCACAATATTCTTCCTCTTCATCCTCATCATAAA

>M.05.0.25_A_31

GTTTCCTCATCTTTCGTCTCAGCAATGACTCTCACGAA

>M.05.0.25_A_32

AGTTCATAATACCCTTGACCAGTGCCTGGTGCTAAAGCGT

>M.05.0.25_A_33

CTAGTATAGTATTGATAAGTAAGGGATAAATCTGGAGCAGG

>M.05.0.25_A_34

TCAAACTGGACGAAGAGGACGGGTTCGTTACGGTCGTCAC

>M.05.0.25_A_35

AGAAAACTTCCGATTGCTTTGATAGCATCCCCTATCGT

>M.05.0.25_A_36

AATAACTTCCTCTCTCGGCTTATCAAGGGACATGAACTT

>M.05.0.25_A_37

AAATTCCGCTTATACTTAGACGTGACGGGGACACAGTTT

>M.05.0.25_A_38

TCTAAGAAGTCCGTATGTTTCATTTTCATGAAAAATATAG

>M.05.0.25_A_39

TTTCTGGGATTTTATCTATCGCGAACATTTCACTTAGTTT

>M.05.0.25_A_40

TTCAATTTTTGTTGGATTTCGGTGAAAACGCTGAAATA

>M.05.0.25_A_41

TGTTAGATGAATTTGCTAAGGTGAAAAATAATGACTGA

>M.05.0.25_A_42

AGTAAAAACGTGGAAAACCTTACTCCTTACCACGTTCG

>M.05.0.25_A_43

TAGAAGACCTGTATTTTTACGGGTTCGTAAGGAGATAT

>M.05.0.25_A_44

TTTGTAGGCCCAGCAGGAGCACCACTACCACTGGCACCTG

>M.05.0.25_A_45

ATAAAAATTCCCGCTAACATGTATACTAGATGTTTAAAGT

>M.05.0.25_A_46

AAGTTCCAGTACTTAGGATAGACTGATGAAGGCACCGGA

>M.05.0.25_A_47

GCACTGAGATTAACAACAAATCTGAAAATTCTAGTTGCCAT

>M.05.0.25_A_48

GTTCTACAAAGCGGTTCTCAGATCATTATAGATGATGA

>M.05.0.25_A_49

CAATATAAAACTCCCTACCCTGATTTGTTCATTATATTTAT

>M.05.0.25_A_50

TCGTTCTGTTCTGTTCTATACTGTTCTCTGTATATTCATTT

>M.05.0.25_A_51

TATATTTCATCATTATAATCAGACAAATTAACAACTTCATC

>M.05.0.25_A_52

CCATTTTCAATATATTTTAGCGTTTTCACCGAAATCCAGG

>M.05.0.25_A_53

TGCTAGTTGGATATCGAAATGGCAAAATGTACAAGCTAGGA

>M.05.0.25_A_54

TTATCACTTTAACTAACACTAAACTAGAGAATTAAGTCTT

>M.05.0.25_A_55

CTTCCTGTTATACTTTCAGTAGCTTCTGCCCCTTGTAATTGC

>M.05.0.25_A_56

TCCCTGCCAGCCTATCAGGACGAAAGAGTAGGCAGTGTAA

>M.05.0.25_A_57

CTGTGCTTGTGTTTGACGTTGCAAATGCACTACCTACTCCTA

>M.05.0.25_A_58

TCAGAGTTCTCCGGCATATCCAAACGGTATAAGGCGT

>M.05.0.25_A_59

AAAATCTTCTTTGACCTCCTCAAACTGAATACTTAATCGTT

>M.05.0.25_A_60

AACACTCCTAAATTTTGTAGTGCTTCTTGCAACAGTAAAG

>M.05.0.25_A_61

TTAGGACTTTCAATATTTGGAGGAAAAGGAAATAATCA

>M.05.0.25_A_62

ATTTGTGGTTTCACAACGATTAGATTATTCGCGTTAAAAC

>M.05.0.25_A_63

GATTTTGTCGATCATAAAATGCAGAGAATCGTTGAAAT

>M.05.0.25_A_64

GCGCGTAAAAAGTACTCCAGGCTTCAGTTGGTTATCGAA

>M.05.0.25_A_65

GTCCCTACAATTTACCGGGTACAGGTCTACCATCAT

>M.05.0.25_A_66

AGTGTTGTCAGCATAATAAATAGGAGAGAGAAAAGATTA

>M.05.0.25_A_67

TTCAATGTCGTAACAGGTACTGTATCATCTTCGCTAATCCC

>M.05.0.25_A_68

TTCTCGCTAGCGAATTTAAGTAGTTTTTCATAGTTTTTAG

>M.05.0.25_A_69

TCTCATCAGGAACCTGCGCAAGTTGCCGAACAGAGTAAA

>M.05.0.25_A_70

TCATCTTCTACATCTTCTAGCGGATCATCTTCTAGTAG

>M.05.0.25_A_71

AAAATTTCTCCTATTGCCATATATTACTTTCACTCATCTCTC

>M.05.0.25_A_72

TTATACTCAAACGGTTGCGTACCCCAAGGACGTAATTCTAC

>M.05.0.25_A_73

CTGGTAATCATAATTGCGTTGGCAACGTTAGGGATTGGA

>M.05.0.25_A_74

GTTAACTTGGCAAAATCTTCATACGGGGTTCCTGATGTAG

>M.05.0.25_A_75

CTAGGTAATAGATTGGCGGGGAGTTGTAACGTTACTGT

>M.05.0.25_A_76

CCCTTATTGCATAAATCTTTGATGAGTTTGAAACGACAT

>M.05.0.25_A_77

TCTCTTAATACTCTAGCTTGTATTTGTAATTTTTCTTTT

>M.05.0.25_A_78

TGCTTCTTGAGCTTGTAATCCGTTACTGAATGGATTATAT

>M.05.0.25_A_79

GCTCTGATGTTGCTGCCTCAATGTAAGTGAAATGAGGCC

>M.05.0.25_A_80

CTGTCATTGACTCCCACCCGTACTAGGTATAAGTGTGGT

>M.05.0.25_A_81

TTAAGCCCCTGTTTCTTTAATAGTTCATAAACTCTCAAA

>M.05.0.25_A_82

GAAACTGACAAAGATTGTCATTTTTTAAGCCAGTTGATTA

>M.05.0.25_A_83

TACGACAGAAGCAGACATCAACCAACGCCATGATAATCCG

>M.05.0.25_A_84

TTATTGATGGCTACGTTGTGAATGCATTTTATAACGGATCTAA

>M.05.0.25_A_85

CTCTACCAGGGCTGAGCTACGCCCGGTATTATATTTATAT

>M.05.0.25_A_86

GTAAAGCCTCCAGGCATGATTGTAGGTAGCATTGGTGGT

>M.05.0.25_A_87

AGGAATTAAACAAACAAGTAGTACTGGAGGTGGTGGCTAA

>M.05.0.25_A_88

GCTGTATTAATAGTATAGCCACGTAAGCCTCATAGGGCAT

>M.05.0.25_A_89

ATGTATATGTAGCCTGGTTGGATAAATTGTACATGGAT

>M.05.0.25_A_90

AGACCCCCAACGTACACGTGGGGGTCAGGATGTACAGGG

>M.05.0.25_A_91

TTTACATATACAGTAATACTTACTGAGAACGGTGCTGGTA

>M.05.0.25_A_92

TAATTTTTCAATTTCAGTTCTTTCAGTAGAGATGAGTAACT

>M.05.0.25_A_93

TTTCTGACTGTTTCAAAAACTCATAAACCTGTTTTATCAATA

>M.05.0.25_A_94

CCTTATATAATTCGAGAGCTTTGCGAATTAATCTGTTTTG

>M.05.0.25_A_95

CGTTCAAAGCGTTTACATGAATTATTGTATAAATGTTGAA

>M.05.0.25_A_96

AATTATATAAAAATTGCGATCGCGTTCGGAGCTGCGGCATT

>M.05.0.25_A_97

GAATTAATTATGAGTTCTACCCTATCAAGCCAAAGTAAAT

>M.05.0.25_A_98

TTAACTGAGGACTTGAGCGATGAAGCCGAGGAAATTTAT

>M.05.0.25_A_99

ATCACAGAACTAAACATTGGTATTACTATCAGTGTTACTAGT

>M.05.0.25_A_100

ACACTTAGGTACGCATCTAACAACAACTCTTCTAACTTATT

>M.05.0.25_A_101

TTATTGAATTTCTTAACAAATCTCCAAATGCTTCAAAAC

>M.05.0.25_A_102

TTGAAAATTTTGAAAAAAGACTTGAGGGGGCCTGAGAGG

>M.05.0.25_A_103

CTTTAAGCCTGAACAAACCCCTGACGTATTTTACCAAC

>M.05.0.25_A_104

GTAAGAGAGCGTATGCGCTCAATCTCAGCTACTAGCTTTT

>M.05.0.25_A_105

TAAGGAGGGCTTTCGCTAGTAGTTTGAACGATTCGGCCAT

>M.05.0.25_A_106

AGCAATGTACTGACCGCCATTAGACGCTCCAGTTGA

>M.05.0.25_A_107

TTTTATTATACCTTTCATGAACTTCCATTATAACATAAT

>M.05.0.25_A_108

TTTGATAAAGCGATGACATTACGTATATACTTTTATGAT

>M.05.0.25_A_109

TCTAACAAATTTACCTTCACCAACCTTCTTATAATAATGTG

>M.05.0.25_A_110

CAACAATATTTACAAGCACTGAATCAATTAGGGAGTTCAAGG

>M.05.0.25_A_111

TCTCACTCGACGCTTGTGCATCAGAAGGGGTCGACTGCA

>M.05.0.25_A_112

AAAGCAGAAAACGGTAGAGGTTGTAAAGAGGCTAAAGTCA

>M.05.0.25_A_113

CCGAGACGCCCTGGCGAGGGTGATACACCTCTGCCCCACCCTTCG

>M.05.0.25_A_114

ATCAGACGGCAGTATCGATAGGGTTGCATGTGATAGATAT

>M.05.0.25_A_115

TATACTTGGGATTCAGCTTCTTGGCGCAATGCAGCGG

>M.05.0.25_A_116

TTAACTCATATATTGATAGAAAAGGAACAGTTACAACATAT

>M.05.0.25_A_117

GTAGAAATAATACTAACGAAACCTCTATTTCAACTGGAA

>M.05.0.25_A_118

ATAATGAAATAATACATTTGTTTGTAAATTTAGCGGATTATT

>M.05.0.25_A_119

TTACACTCGCTACATATTGAAGAATTTACTGATAAACT

>M.05.0.25_A_120

ACTAAGGTCCTGTCTGCTGCTACTGCCTACCGGTCGCT

>M.05.0.25_A_121

AAGTCGGTGTTTGCTAATGCACTTAAGATTTGTTGTA

>M.05.0.25_A_122

TTCTCAAATATCCTTTCCATGCTGAACTCCAAAATCG

>M.05.0.25_A_123

CCTAAATCATTATCAAATTACGTAATTCCACAAAATCA

>M.05.0.25_A_124

GAAAGGTGGCGAAAAAATGGAAGATGAAAGAAAAATCCAA

>M.05.0.25_A_125

ATCAATATAGCTCCTATTGATGCCCATTTTTATTTCACTTT

>M.05.0.25_A_126

ATATACGGAGCAATTGCAGGTCTATCACACCTCCTACTG

>M.05.0.25_A_127

CAACGGGATGAACGTTAGTATGCGAACAGGAGCACCTTTGT

>M.05.0.25_A_128

TAAGACCATCGTCGCTTACAGCGTTCGGGACGAGCTTCAC

>M.05.0.25_A_129

TGTGTGTTTTATTGAAGTATATATCGCTTTTTAAGCAAACGA

>M.05.0.25_A_130

GTTAAAATATCAGTCCAAATTGAGGGCATCTATTCTTATAT

>M.05.0.25_A_131

TCTCTAGCCATTGTAGTGAGATGAAGCTCAAGAAGTAA

>M.05.0.25_A_132

AAACGAAATATCGAAGGCGTCAGATGAGGAAAGGAAGAA

>M.05.0.25_A_133

GGTTTCTCTGCATTACTTTTAAATCCTAATCAGGGGAT

>M.05.0.25_A_134

GCTTGAGGGCGACCGGCAGCGTTAGGACGAAGTAGTTCC

>M.05.0.25_A_135

CGCAAACGTTCTAAATATAATTTTCAATTTAATGAATA

>M.05.0.25_A_136

CTTCATAAATTATAATTCCGCTAGCATATGTTAGATTTAGCG

>M.05.0.25_A_137

AAGAAAGCATATTTCTATAATAATGGACAGAGAGAAGAT

>M.05.0.25_A_138

TTTATTGAAAGGTCGTTTCTGACAGCTTTATTAGGATC

>M.05.0.25_A_139

GTAACTACTTCACAGTCACCCCTTCCTATGTACGCACCGT

>M.05.0.25_A_140

TCACTAGAAAACTTCCTACTCTCATCTTTTCATTGTTTT

>M.05.0.25_A_141

ATATAAATCCGAAAGCACGTTAGTGAGAGAGAAAGATTT

>M.05.0.25_A_142

CATTCCTCTTCTTTGGCATTTTCAAAGTGTATGCTTAAT

>M.05.0.25_A_143

CTTAGGAAGAAGTCCTCTAGTTCGTTGTACATTGGCGCTA

>M.05.0.25_A_144

CCAGAGACTGAGACAGAGCCTGAAGTAGTCCAGATATTT

>M.05.0.25_A_145

AGAGTTAGTCAGCGAGGAGGCGATCAAAGCGGAACTCAAG

>M.05.0.25_A_146

ACGTCGCAACGGTAAAGCTCAGCGGTATTGTTGAGGCTG

>M.05.0.25_A_147

TCTATGTTTCTCTTCTTTCTATAAGCTAAGAAGGATCCATT

>M.05.0.25_A_148

ACTTTTCTCTCGACTGATATATAGCTATATCGAAGACAGA

>M.05.0.25_A_149

GTATTGAGGTACGTCGAGCCAGACGAGACTTACGAAATAAT

>M.05.0.25_A_150

AGGCTGGCCAGAGATCTTTATGACTCGTTCAAGCTCCCT

>M.05.0.25_A_151

GCGATAGATCAAATTAGAAAGACCTTGCCACCTAGAGGA

>M.05.0.25_A_152

CTTTCAGCGCTTCCCTGATTAAGGCGCTTACCGACATT

>M.05.0.25_A_153

CGAATAAATAAATCGTTTATATTATTACTGTAAGGATAA

>M.05.0.30_A_1

TTTAAATAAGGTTTAATATGAACAATTTAAAACACATAACAT

>M.05.0.30_A_2

AAGAGGTGGTATGTCACATGGGATTAGCTATGTATAAAT

>M.05.0.30_A_3

ATAAAGAACACGTTTGTATCCCGACTTTACTAGGGAACAA

>M.05.0.30_A_4

GGCTAAGAATAATATAGACCCCGCAATCATAGCAAAAATAA

>M.05.0.30_A_5

ATATTGGTAGACAAACAAAGTATTACTGAAAATTCAT

>M.05.0.30_A_6

CACATATATTGTATTTGCATTCCATTTTTTGCCTTGAGA

>M.05.0.30_A_7

AGTTTAGAAGAAATAAAGAAAGTGCTGAACCATGAAGATG

>M.05.0.30_A_8

TAATTTATATTTATTATATCGGCTTTTACTGGAAAGCGGT

>M.05.0.30_A_9

AGTTAGATCAAACTCGGGAAGCAGTAAGAAAAGAAGTGTT

>M.05.0.30_A_10

TCTCCCACATCTAGGGCATTGATATAATAACGCCCCA

>M.05.0.30_A_11

AGAAACAGTGTATGGGATAAATTAGGTATAAGTGGCG

>M.05.0.30_A_12

ATTTATTGTGAGTGCGGACTTTCAAATAATCTAGACGT

>M.05.0.30_A_13

ATTATCAGACCAATAGAAATAATAAGGCGTGCGGAGGAAA

>M.05.0.30_A_14

GCTATCACTGGTATCTTGTTCTCTCCAGATTGAAGATTA

>M.05.0.30_A_15

CCCAACAAGGGGTGGTTGCCCCGGGTTCATCTATCCCCCGGATCA

>M.05.0.30_A_16

TACTGTTCCGTTTGCTGTATTCTGTATAACAATCCCGT

>M.05.0.30_A_17

CATTCCTCTTCTTTGGCATTTTCAAAGTGTATGCTTAAT

>M.05.0.30_A_18

CCTCTTCACAATATTCTTCCTCTTCATCCTCATCATAAA

>M.05.0.30_A_19

TCATAAGGTTTTACTGTTGCAGTAGGTGTTAAATTAGA

>M.05.0.30_A_20

ATCTTCAAAATATGCCTTAATTCGTCTTCGGACGTCGCA

>M.05.0.30_A_21

TTGCCAATTTAATGTATATATGCATATCCCGTTTTCACAAA

>M.05.0.30_A_22

ACGGATAAACTGAGGATTCAATTCTAAACTGAAATAAGG

>M.05.0.30_A_23

ACTGCTATAGTTTCATAACGTGACACTACTCTATCAAAG

>M.05.0.30_A_24

CTAACGTAGGTACCTTGAGGTCTGGTTTCGTTTTCTTAACTTT

>M.05.0.30_A_25

TTTCTGGGATTTTATCTATCGCGAACATTTCACTTAGTTT

>M.05.0.30_A_26

TAAAGTTGATGAAACGTGATGAAAGACTATTTAAGCTA

>M.05.0.30_A_27

TTAACTGAGGACTTGAGCGATGAAGCCGAGGAAATTTAT

>M.05.0.30_A_28

ACCCGTACACCATCATACCATCATTCTATAGTAGCGCTG

>M.05.0.30_A_29

GAAAAAGTAATAGAGGGCATCTTTGAATTAGGCTTATTT

>M.05.0.30_A_30

TACAAATGCGGTAACATCGTTAGGGAATTTTATCGTAAA

>M.05.0.30_A_31

GCACTGAGATTAACAACAAATCTGAAAATTCTAGTTGCCAT

>M.05.0.30_A_32

TCGTTCTGTTCTGTTCTATACTGTTCTCTGTATATTCATTT

>M.05.0.30_A_33

AGTGTTGTCAGCATAATAAATAGGAGAGAGAAAAGATTA

>M.05.0.30_A_34

AAAATCTTCTTTGACCTCCTCAAACTGAATACTTAATCGTT

>M.05.0.30_A_35

AGATAAAAAAGCTGAGGTACGTATGCCCACACCCTGGCTT

>M.05.0.30_A_36

AATAGAAACACTGCTTAAGCTGTTAGATGAACTAGACA

>M.05.0.30_A_37

TCTTTCTCTGAGGGATACATCGGCGATATCTTCAATAA

>M.05.0.30_A_38

TCAGAGTTCTCCGGCATATCCAAACGGTATAAGGCGT

>M.05.0.30_A_39

TTTGCATCTTAAAAACGTATCACCTACGCAAAATAATTCAAT

>M.05.0.30_A_40

AAAATTTCTCCTATTGCCATATATTACTTTCACTCATCTCTC

>M.05.0.30_A_41

AACTCCAGTACTGCTTCGCCGTAGGATCCGTCCTCAGACG

>M.05.0.30_A_42

TCCACTTTTTCAGTTGTTCTTCCTTTACCTTTTCTATATCTT

>M.05.0.30_A_43

CCCCTATACCCATTCAGAAAAAACTGTTAAACAAACGTCA

>M.05.0.30_A_44

TCTCTTAATACTCTAGCTTGTATTTGTAATTTTTCTTTT

>M.05.0.30_A_45

TTTACGGGCACTTTCGGTCACCTTTTCGCTCACCCTCAT

>M.05.0.30_A_46

ATAAACACTACGGGGATCAGATGAATGATAACCCCGACAACCG

>M.05.0.30_A_47

TAATCCACATAAGCCCTCTTTCACTCCCGTACTTTGTCG

>M.05.0.30_A_48

TTAAGCCCCTGTTTCTTTAATAGTTCATAAACTCTCAAA

>M.05.0.30_A_49

CTCTCGTCTAAGCGTCCTGCATTCTCTACTAGCTCGTTAAG

>M.05.0.30_A_50

TTATTGATGGCTACGTTGTGAATGCATTTTATAACGGATCTAA

>M.05.0.30_A_51

ACCGGCGAAGTACTTAGTATATCAAATATATAGAAGTAACTT

>M.05.0.30_A_52

ACTGTCACAATGGTTATCACACAGAATTGCTAATAATGGT

>M.05.0.30_A_53

TCATGAAATTTATTATTAACAACAAGATATAATTATTTA

>M.05.0.30_A_54

AAAACTTTAAGTGCTTCATCAATGTCTTTGCTGAGCCTA

>M.05.0.30_A_55

TAGGTTTTTCGATTTTAAGAGGCTCTTCTACTTGCTCTA

>M.05.0.30_A_56

CCTTATATAATTCGAGAGCTTTGCGAATTAATCTGTTTTG

>M.05.0.30_A_57

TCTCACTCGACGCTTGTGCATCAGAAGGGGTCGACTGCA

>M.05.0.30_A_58

GTAAGAGAGCGTATGCGCTCAATCTCAGCTACTAGCTTTT

>M.05.0.30_A_59

TAGGCAACCGCTTGTCTATTATTCTTTAGCTTCATTTTG

>M.05.0.30_A_60

AAGTCGGTGTTTGCTAATGCACTTAAGATTTGTTGTA

>M.05.0.30_A_61

ATATACGGAGCAATTGCAGGTCTATCACACCTCCTACTG

>M.05.0.30_A_62

TAAGACCATCGTCGCTTACAGCGTTCGGGACGAGCTTCAC

>M.05.0.30_A_63

TCAGACATTGCGAAAAATTTAGACGGGGGTATAGAAG

>M.05.0.30_A_64

TCTCTAGCCATTGTAGTGAGATGAAGCTCAAGAAGTAA

>M.05.0.30_A_65

TTTACAAGACCGACACTTAGATCCTTCGGTGGAAAGA

>M.05.0.30_A_66

TAGAAAAATTTATCAATAGCCTAGTAACACTGAAGTTTCCTT

>M.05.0.30_A_67

CTTAGGAAGAAGTCCTCTAGTTCGTTGTACATTGGCGCTA

>M.05.0.30_A_68

TTTATCTACCATTTAGCTCACCCCTTGGTAATTAGTTTGT

>M.05.0.30_A_69

ACTTTTCTCTCGACTGATATATAGCTATATCGAAGACAGA

>M.05.0.30_A_70

TATCTTCTAATACCTTATTCTTCTCTTCCAGTTGCTGTAT

>M.05.0.30_A_71

CTTTCAGCGCTTCCCTGATTAAGGCGCTTACCGACATT

>M.05.0.30_A_72

CGAATAAATAAATCGTTTATATTATTACTGTAAGGATAA

>M.05.0.30_A_73

ATAACAATAATAAATGTTACTATTGAGGGCGTATTATTCCCAG

>M.05.0.30_A_74

CACAAATTCCCAGAAGCTTAACAATAGTTTTGAAAGCGT

>M.05.0.30_A_75

AAACAGTGCTAGGCAGTGGTTGTTCTATACAGATCATATA

>M.05.0.30_A_76

TCAACATCGACATAATATACTATAACTTCCGCATTATCAA

>M.05.0.30_A_77

TTACCTTTACGGATATGAAATTGATGATATCGCTGA

>M.05.0.30_A_78

GGCTATTTGGACTTTGAAGCGTAGACAATTATGAG

>M.05.0.30_A_79

TCTAACTTCCTAAGTAGATAAGAAGGGTCTACGGGTTTAAT

>M.05.0.30_A_80

TAGGATTATTTATACTAGCCAAGTTTTTTAGTTTAACTT

>M.05.0.30_A_81

CGAAAGGATTCTTTCTATTCCCGTTATCAGTAAATACACT

>M.05.0.30_A_82

TATACGTCAGCTATAACTATAGCATATGGTTATGCACCAG

>M.05.0.30_A_83

CATTTTGTAGGCTTACAAATAACATTTACTCATAGTTAT

>M.05.0.30_A_84

GGCGTGTCGCCTCATCTTTATCAATTCGTAAAGCTCCATC

>M.05.0.30_A_85

ATAAAAATTCCCGCTAACATGTATACTAGATGTTTAAAGT

>M.05.0.30_A_86

GGGCGGGTACCCGCGCCCGGGGGGCGAGCACACCCGGGTACAA

>M.05.0.30_A_87

AGAAGAGGAAAAACTTGTAAAACAAAGTTCAGTTTTTTTA

>M.05.0.30_A_88

ATCTATCATCTGGATAGAATACTGCAGAGAACGTATCACT

>M.05.0.30_A_89

TTTTGATTGATTTTGCCATTGCAAAAGCCAATTTGCT

>M.05.0.30_A_90

ACATATATACCCCAAGTTATTATGTTGCTAATGCTG

>M.05.0.30_A_91

GGCGGTAATGCGAAATCAACATAACCCACTACTGAACCTCC

>M.05.0.30_A_92

AGCATCGATGAACGACTTGGATAGAGTACTAGCGGTTCTG

>M.05.0.30_A_93

AGAAAACTTCCGATTGCTTTGATAGCATCCCCTATCGT

>M.05.0.30_A_94

AATAACTTCCTCTCTCGGCTTATCAAGGGACATGAACTT

>M.05.0.30_A_95

ATTTTATCGAGATATCATAATTACCATATGTTATAG

>M.05.0.30_A_96

ATAGGGATTCCCAGCCCGTTCGGGCTTAGCTGGAGGTGGT

>M.05.0.30_A_97

AGTAAAAACGTGGAAAACCTTACTCCTTACCACGTTCG

>M.05.0.30_A_98

TCCATGAACTGCCTGAGCTGACGAATTGCTACTATCAAAG

>M.05.0.30_A_99

TAGAAGACCTGTATTTTTACGGGTTCGTAAGGAGATAT

>M.05.0.30_A_100

TACGACAGAAGCAGACATCAACCAACGCCATGATAATCCG

>M.05.0.30_A_101

AGTACGTTTAGCGTAAAGCAGCTAGCTCCTTACTTGCCCT

>M.05.0.30_A_102

TGTAACGATTGAATATATTAATCACGTACACACGTATG

>M.05.0.30_A_103

TCGTAACCCTGATTTGGTACCAACCCTTTTCCCTCAG

>M.05.0.30_A_104

AACACTTCCCGTCGTTTGCACCGTAACGTTTGTAATT

>M.05.0.30_A_105

AAAATATCTTACTTGAGGTTTAGAAGAGAGGAGAATGGT

>M.05.0.30_A_106

TATGCCGGTCTATTTCGTCGTGTTCACATCGATATTTG

>M.05.0.30_A_107

CCATTTTCAATATATTTTAGCGTTTTCACCGAAATCCAGG

>M.05.0.30_A_108

ATTAGATAATTATAAACTTCTTTTTGTTTATATTGAA

>M.05.0.30_A_109

TTATCACTTTAACTAACACTAAACTAGAGAATTAAGTCTT

>M.05.0.30_A_110

CTAATTCCAATATATCCTATTTGTGACCAAGGGAATGG

>M.05.0.30_A_111

TTAGGTAAGCCGTATGTTATCCCGTATTCATTATAGTTAT

>M.05.0.30_A_112

TCCCTGCCAGCCTATCAGGACGAAAGAGTAGGCAGTGTAA

>M.05.0.30_A_113

AACACTCCTAAATTTTGTAGTGCTTCTTGCAACAGTAAAG

>M.05.0.30_A_114

TTAGGACTTTCAATATTTGGAGGAAAAGGAAATAATCA

>M.05.0.30_A_115

GTCCCTACAATTTACCGGGTACAGGTCTACCATCAT

>M.05.0.30_A_116

TTCTCGCTAGCGAATTTAAGTAGTTTTTCATAGTTTTTAG

>M.05.0.30_A_117

GATAATGACGATAAGGACAGCATTATCGTGTTTGATG

>M.05.0.30_A_118

TTAATTACTATTGCTATCTTGCTATATAATGCTTTTTTCGC

>M.05.0.30_A_119

TCATCTTCTACATCTTCTAGCGGATCATCTTCTAGTAG

>M.05.0.30_A_120

GACGAGAAGAGACTAATCGATCACGCAATTGAAGCCGG

>M.05.0.30_A_121

CCCTTATTGCATAAATCTTTGATGAGTTTGAAACGACAT

>M.05.0.30_A_122

GCTCTGATGTTGCTGCCTCAATGTAAGTGAAATGAGGCC

>M.05.0.30_A_123

GAAACTGACAAAGATTGTCATTTTTTAAGCCAGTTGATTA

>M.05.0.30_A_124

TAAAATTTTTTAACTCAGTCTCATTTTTTAAAATATGGG

>M.05.0.30_A_125

GCTGTATTAATAGTATAGCCACGTAAGCCTCATAGGGCAT

>M.05.0.30_A_126

ATGTATATGTAGCCTGGTTGGATAAATTGTACATGGAT

>M.05.0.30_A_127

CGTTCAAAGCGTTTACATGAATTATTGTATAAATGTTGAA

>M.05.0.30_A_128

AAATCGCCGTCAATATCATATTACTTACGCTCTGAAAGA

>M.05.0.30_A_129

TAATTTTTCAATTTCAGTTCTTTCAGTAGAGATGAGTAACT

>M.05.0.30_A_130

AATTTTTCCCAAACCGATTTGAGAAACGAAAAAAACTCATC

>M.05.0.30_A_131

TTAACCATCCCAAAATTCGTAAGCCAATTACCATTCTCAT

>M.05.0.30_A_132

AGAGCCGAGATGATAATTAAACAAGCAAAACAATATCTA

>M.05.0.30_A_133

AACATTATATATTAGAAGGCTATAAGGGTGAGTCATAATCGA

>M.05.0.30_A_134

TTTAATCACCTGAGTTAAAGTGTGTAAATACATATTTAT

>M.05.0.30_A_135

TCTTGGGCTGCGCCGCAGCAACACCCTGAGGAAATCTTTGAC

>M.05.0.30_A_136

CTTTAAGCCTGAACAAACCCCTGACGTATTTTACCAAC

>M.05.0.30_A_137

CCCCGTGAAGGGTGGTTGCCCCGAGTTCATGAATCCCT

>M.05.0.30_A_138

AGAATTATAATCATAAACTCTATCAGTCTTCCTAAACCTA

>M.05.0.30_A_139

CTATCAGTAATTGACAAAGTCACAGAAGCACCTTGTGAAA

>M.05.0.30_A_140

CCGAGACGCCCTGGCGAGGGTGATACACCTCTGCCCCACCCTTCG

>M.05.0.30_A_141

ACGTCGCAACGGTAAAGCTCAGCGGTATTGTTGAGGCTG

>M.05.0.30_A_142

ACAGACGTATTAAGATATGAACCTACTGATGAGTTAGT

>M.05.0.30_A_143

CTTTTACCAGACTTTGGTGGCCCTGCAAATAATATAATCA

>M.05.0.30_A_144

GAATTCCAAACAGCCGAATATAAGTCAAAGAGACAGTACT

>M.05.0.30_A_145

TAGTATAATCCGCGTATTCACCGTTTATCACGTTTTCAAT

>M.05.0.30_A_146

GAAAGGTGGCGAAAAAATGGAAGATGAAAGAAAAATCCAA

>M.05.0.30_A_147

TAAAATTAGTTACAGCGTTCTCTACATCGTTAAATAGTCCT

>M.05.0.30_A_148

GGTTTCTCTGCATTACTTTTAAATCCTAATCAGGGGAT

>M.05.0.30_A_149

AATCCTAACGCTAAGGCTTCTGCGTGCTGTTTCAATTCAT

>M.05.0.30_A_150

AAGAAAGCATATTTCTATAATAATGGACAGAGAGAAGAT

>M.05.0.30_A_151

AGTAACCGCTTAGGTTCTGTAGGGTAACATTTCGGT

>M.05.0.30_A_152

AATAAATATATTATAGATCCTATCAGCATCGCCTTAGGAGT

>M.05.0.30_A_153

TTGTAGTTGCATCTCCTTATTGCCGTTTCTGAACTTCTTTT

>M.05.0.30_A_154

GTAGAAATAATACTAACGAAACCTCTATTTCAACTGGAA

>M.05.0.30_A_155

TCCAGAACGTGTATCTAATAATAATCGCCTTATACTTGAGT

>M.05.0.30_A_156

TTAGTTTGAACAAGTTGGACATAACGTCCGTAGAACCCTA

>M.05.0.30_A_157

GTATTGAGGTACGTCGAGCCAGACGAGACTTACGAAATAAT

>M.05.0.30_A_158

TTTAGCATACTCTAAGTCAATATCTTGGGGACTAAATTCGA

>M.05.0.30_A_159

ACGGTGGAGCGATAATAAGAAAGATTCTAAAGGCCGAGG

>M.05.0.30_A_160

CTGAATCCTTACGAACTCTATGAGATGTTACAACAAACTGGAT

>M.05.0.30_A_161

GCCTATGTTCGTGGGTGCGAGTACATAAGTTGTTTGACCGTT

>M.05.0.30_A_162

AAATAAAGTGAGCTAAATGGCGGCAAATCAAGAAGTATG

>M.05.0.30_A_163

AATAGCCTAATGTGGTGAGAGGGAATGTTTGAAAAGCTGAAAC

>M.05.0.30_A_164

CCTGCTCCTCCACTACCTGCTGCACCTGAAGTAGCGTT

>M.05.0.30_A_165

CAGACATCTAACGTAGGCAGTTCTAGCGGTGGTACTAGT

>M.05.0.30_A_166

TTCTAATAGCTGAACCCCTGCCTGATAGTCTCTATTAA

>M.05.0.30_A_167

TCTAAGAAGTCCGTATGTTTCATTTTCATGAAAAATATAG

>M.05.0.30_A_168

TCAATAGAGTTCTTAAATAATGCAGGGTAATCAACAGC

>M.05.0.30_A_169

TCTAATGTAATTTATAGTGTAGTAGTTGAGGTTGTGTTTA

>M.05.0.30_A_170

AAAAAGGAATGGAAAGAGCAGGAAATGAAACTATATG

>M.05.0.30_A_171

TAGATTTAGATATAGATATTATTCACGTTTCATGCATGTA

>M.05.0.30_A_172

TATATCATGCGGTATAAATGCGGGGTCGGCTTCCTCGT

>M.05.0.30_A_173

AAATGAGCGTACGCCCACACCGGAAGTCTTCCGCTTAGT

>M.05.0.30_A_174

ATACGTTGAACCAATACTAAGACCAGTTACAATTGCGAA

>M.05.0.30_A_175

TTTATAGAATATATATATGTACCATTTGGTATTATACTAC

>M.05.0.30_A_176

CTAGTATAGTATTGATAAGTAAGGGATAAATCTGGAGCAGG

>M.05.0.30_A_177

TTAAAGTGTATACGCCCTTCTTCGTTACACTTACAGACA

>M.05.0.30_A_178

AAGTCAGCTACGAACGGTAACCCTGTAACGAGGAGG

>M.05.0.30_A_179

AAATTCCGCTTATACTTAGACGTGACGGGGACACAGTTT

>M.05.0.30_A_180

CTAAAAATCATTGAACTACACATTTGATCGTCTTCAT

>M.05.0.30_A_181

CTCGAAAAACCAACCACTCCCGTTAAACTTTTACTAACT

>M.05.0.30_A_182

TTCAATTTTTGTTGGATTTCGGTGAAAACGCTGAAATA

>M.05.0.30_A_183

TGTTAGATGAATTTGCTAAGGTGAAAAATAATGACTGA

>M.05.0.30_A_184

TTCGATTTTGGGAATCCCACCGCCCAGCAATTCGCAGAC

>M.05.0.30_A_185

TTTCTATTAAGAGAAAGTTACACTTTATCAAAAGACACT

>M.05.0.30_A_186

CTATCGGCTTATATCTAACATCCTCTGGGAGGCTTTCCAT

>M.05.0.30_A_187

TTTTATTATACCTTTCATGAACTTCCATTATAACATAAT

>M.05.0.30_A_188

TATATTTCATCATTATAATCAGACAAATTAACAACTTCATC

>M.05.0.30_A_189

CGGTTCACCGCTCGACAGTAAGGCGGTTTTCGATGTCAT

>M.05.0.30_A_190

TAAGCCCTCACCATCCGTATTTGCTTCCCACCGGTATA

>M.05.0.30_A_191

ATCTAACTATGATAAACTGGATGTAGAAATATTATTTAGT

>M.05.0.30_A_192

CTTCCTGTTATACTTTCAGTAGCTTCTGCCCCTTGTAATTGC

>M.05.0.30_A_193

CTGTGCTTGTGTTTGACGTTGCAAATGCACTACCTACTCCTA

>M.05.0.30_A_194

AATGATGACCCGATATATGACGATGTCAAGATAACGTA

>M.05.0.30_A_195

GCGCGTAAAAAGTACTCCAGGCTTCAGTTGGTTATCGAA

>M.05.0.30_A_196

AGTAAATTTGAGGACGTACTACCCAATGCTCACCGACA

>M.05.0.30_A_197

TATACTTGGGATTCAGCTTCTTGGCGCAATGCAGCGG

>M.05.0.30_A_198

TTACACTCGCTACATATTGAAGAATTTACTGATAAACT

>M.05.0.30_A_199

ACTAAGTATCAGCAATGCTCAGCTGAGAATTTATTACCA

>M.05.0.30_A_200

TGCCTTCCGCTTCTACTTGTGTGTAATCTTGATTTGATA

>M.05.0.30_A_201

GTATAACTATCATGTTCCTCACTACATTCCTGATACT

>M.05.0.30_A_202

CTGGTAATCATAATTGCGTTGGCAACGTTAGGGATTGGA

>M.05.0.30_A_203

ATTCCAAAGTTACGTCATCGATGACCACGTAATGGAAA

>M.05.0.30_A_204

CTAGGTAATAGATTGGCGGGGAGTTGTAACGTTACTGT

>M.05.0.30_A_205

CCACCACCGCCGCCACTACTACCACCACTGCCAGCACTGC

>M.05.0.30_A_206

CTGTCATTGACTCCCACCCGTACTAGGTATAAGTGTGGT

>M.05.0.30_A_207

GTCGTTTTTCTCCTTAATTTTGCATTCTCATTCCCAGCTT

>M.05.0.30_A_208

TTATGCCCGTCACCTATTACGTCCCTACGAATGATACC

>M.05.0.30_A_209

ACTACCTCGGGGTTGTTTAAGGTTATTTTTTGTGCTTC

>M.05.0.30_A_210

CATATGTCGGATCACTGTATAATATCCAATAATGAAAT

>M.05.0.30_A_211

GTAAAGCCTCCAGGCATGATTGTAGGTAGCATTGGTGGT

>M.05.0.30_A_212

AGGAATTAAACAAACAAGTAGTACTGGAGGTGGTGGCTAA

>M.05.0.30_A_213

TTTCTGACTGTTTCAAAAACTCATAAACCTGTTTTATCAATA

>M.05.0.30_A_214

CAACATCTACCTAATATCTAGACCGACAAACACACAGACAGG

>M.05.0.30_A_215

TTAGTGATATTGCTACAATACTAGAATACCCTAATTTAGATAG

>M.05.0.30_A_216

ATCACAGAACTAAACATTGGTATTACTATCAGTGTTACTAGT

>M.05.0.30_A_217

AGATTTTGTAGTGCTTCTTGCAGCAGTAAAGCTCTAG

>M.05.0.30_A_218

TTATTGAATTTCTTAACAAATCTCCAAATGCTTCAAAAC

>M.05.0.30_A_219

TAAGGAGGGCTTTCGCTAGTAGTTTGAACGATTCGGCCAT

>M.05.0.30_A_220

GTATGCTTATCGTATAAAGTAATTTGCCAAAGTTGTACGGATG

>M.05.0.30_A_221

TCTAACAAATTTACCTTCACCAACCTTCTTATAATAATGTG

>M.05.0.30_A_222

GCGGATAGTTAGGAAGACCAGGCATGTTCGCTGGATTCCT

>M.05.0.30_A_223

TTAACTCATATATTGATAGAAAAGGAACAGTTACAACATAT

>M.05.0.30_A_224

CCGTGAAGTACAATGGATATGATGAAGGAACTGTCTATAT

>M.05.0.30_A_225

ATTAATTCTTTCTTCTTTTGCTCATAATATTTTATTTCATCTT

>M.05.0.30_A_226

TACTTCATCACGTCCACGTTTGTTATTTTTGTTGATTTAA

>M.05.0.30_A_227

TTCTCAAATATCCTTTCCATGCTGAACTCCAAAATCG

>M.05.0.30_A_228

CCTAAATCATTATCAAATTACGTAATTCCACAAAATCA

>M.05.0.30_A_229

GTAATCGTTAATGTATTTCTTAAGGAATTTCATCCCCCCAA

>M.05.0.30_A_230

TGGCAATCGTAATAATGCCCGTAACCGTAAGCTCTGAGAG

>M.05.0.30_A_231

TGTGTGTTTTATTGAAGTATATATCGCTTTTTAAGCAAACGA

>M.05.0.30_A_232

CATGCAGCTGGGTTCTTAACCTTAATGCAACAGATATA

>M.05.0.30_A_233

TTACAGTATTCGTATGCGTCACAGTATGGGCCATAGTTGT

>M.05.0.30_A_234

GTCTGTCCGTACACTCTTTTATCATGTCATAGATTTTTCTC

>M.05.0.30_A_235

CGCAAACGTTCTAAATATAATTTTCAATTTAATGAATA

>M.05.0.30_A_236

ATATAATAAATATAATGAACTATATTGCTTAGTTTTCTCA

>M.05.0.30_A_237

CTTCATAAATTATAATTCCGCTAGCATATGTTAGATTTAGCG

>M.05.0.30_A_238

AAATAGTCCAATGAAGCGTTAGTAGTAGCTACTATTATTAC

>M.05.0.30_A_239

GTATCAGTAGAGCATATATTAGCTAACATTTCCACATTAT

>M.05.0.30_A_240

ATATATGCTGTAATCTATAGACTAGGATATACTTTTGTT

>M.05.0.30_A_241

CCAAATTTCGGGCTCTCGCCCGCTTTTACTTTCTAACCGC

>M.05.0.30_A_242

ATTATAAGAACAACGGTCTCTTTTACGTCAATGTTTA

>M.05.0.30_A_243

AGAGTTAGTCAGCGAGGAGGCGATCAAAGCGGAACTCAAG

>M.05.0.30_A_244

TCTAAATTTTGTTCTATGTCGGAAGAAGTATAAATGCTAT

>M.05.0.30_A_245

AACAAAACGCTAGATTGTAGAGTTATATTAGTAATAGCG

>M.05.0.30_A_246

CGACTTCACTATACCTTGTACCATTTATCACTATTGCTAT

>M.05.0.30_A_247

GCATATTTCACGCTAGGACTTGATAATAATAATGAATTTGT

>M.05.0.30_A_248

AAATGCGTATCAAGTTGCAGAGCAGAACAATATAAGTAGT

>M.05.0.30_A_249

TTAAGTACTGTATTACCTAGATCAGTTAATAGTGAAATT

>M.05.0.30_A_250

TCAAACTGGACGAAGAGGACGGGTTCGTTACGGTCGTCAC

>M.05.0.30_A_251

CGGAAGTAGGGCTTCCCTATCCTGCAGCAGTTAATATTAC

>M.05.0.30_A_252

ATATGGCTAGCAGGGCTATTAGGGACTCGAAAATAGTG

>M.05.0.30_A_253

ATTTTCTTCACGCGAATATAACCACCTATGCGAAAAAACA

>M.05.0.30_A_254

GTAACGCTGACGTTAGTTATTACATATCCGCCCTGTATCGT

>M.05.0.30_A_255

AAACTAATTCTTTTAAGCTTAAGTATTGGGGCAACCAGA

>M.05.0.30_A_256

TTCAGTGGACAGGTCAGTGGACAGGCGCAAGAGACTGCT

>M.05.0.30_A_257

ATCATTATTCCCACCATTTTGCCGTTAGCTGCTTCATCA

>M.05.0.30_A_258

TTCTCAGGGTGGTTGCCCCGGGTTATGTATTCCCCGA

>M.05.0.30_A_259

ATATTTCCGCTATTTGGATTTATAACGTCTATAATGAG

>M.05.0.30_A_260

AGGACACCTGCCGTTAGTAACTGCTCCGGGTTTCTGGT

>M.05.0.30_A_261

GTTTCCTCATCTTTCGTCTCAGCAATGACTCTCACGAA

>M.05.0.30_A_262

AGTTCATAATACCCTTGACCAGTGCCTGGTGCTAAAGCGT

>M.05.0.30_A_263

ACTATTCTAGACTTAGATAGTATATTCACTAAAACACCGTT

>M.05.0.30_A_264

AAATAACTAGGAAGTTTAGCAAACTCAAACGCCGTAAGTAT

>M.05.0.30_A_265

GATAGATAGCTAAAGTCCATGCTACTTGAGGAGATGGGG

>M.05.0.30_A_266

AATGCTATAGCAATTTTTATGTACGTCGGGCTAAGCA

>M.05.0.30_A_267

AGCCTATAACGACGACTGTACCTTCTTTACCACCTACGACAA

>M.05.0.30_A_268

GTACTGGATCAGGTGTGGCAACATATACAAAGTTGCCCAG

>M.05.0.30_A_269

ACTGCCCCTCTCTTTGCCGTGGGGATCGCCATCCCCCAC

>M.05.0.30_A_270

GTAGAAGTTCCTCTAACTTATTATGTATCGTTACCATA

>M.05.0.30_A_271

AAGTTCCAGTACTTAGGATAGACTGATGAAGGCACCGGA

>M.05.0.30_A_272

TCACTGAATAATCGATATGCTTTCTGTGCCTCATTAACA

>M.05.0.30_A_273

GCTGCAGGTGCTGCAGCCACTGGAACCCAAATTTCAGGA

>M.05.0.30_A_274

CAATATAAAACTCCCTACCCTGATTTGTTCATTATATTTAT

>M.05.0.30_A_275

TTAGCTCAGTACTAGCTAACTGCTTCAAAGCTTCTATAT

>M.05.0.30_A_276

AGATTGTAGTAGAACCCGGACAAGAAGGTGCAAGTTAAA

>M.05.0.30_A_277

TGCTAGTTGGATATCGAAATGGCAAAATGTACAAGCTAGGA

>M.05.0.30_A_278

CATCGCTTAAGTAGGGTAAGAATTGATTAAGATAAGAA

>M.05.0.30_A_279

TTTTATAAATTGTATCTCAATTGGTAGTATAAATTCA

>M.05.0.30_A_280

AGGCTGGCCAGAGATCTTTATGACTCGTTCAAGCTCCCT

>M.05.0.30_A_281

ATTTGTGGTTTCACAACGATTAGATTATTCGCGTTAAAAC

>M.05.0.30_A_282

GATTTTGTCGATCATAAAATGCAGAGAATCGTTGAAAT

>M.05.0.30_A_283

TTTGAATTTTTCTTGCGAAAAAGTATATCTGAACAGCTA

>M.05.0.30_A_284

AATTTACTATTCTCTACTAAAGTGTCTTGTACTACTAA

>M.05.0.30_A_285

GTCTTCTGTGTTAGGGTCTTGTAATTTAACAACTAATATTC

>M.05.0.30_A_286

TCTCATCAGGAACCTGCGCAAGTTGCCGAACAGAGTAAA

>M.05.0.30_A_287

TGCTTCTTGAGCTTGTAATCCGTTACTGAATGGATTATAT

>M.05.0.30_A_288

TGCGATTTCGCCGTTACTTTTGAGCAATTCGGGGTGTTCT

>M.05.0.30_A_289

TTTGTAGGCCCAGCAGGAGCACCACTACCACTGGCACCTG

>M.05.0.30_A_290

CTCTACCAGGGCTGAGCTACGCCCGGTATTATATTTATAT

>M.05.0.30_A_291

GAAAGACGCAAACTGCCGAACAATCAAAATCAATTTTTGGTCAAG

>M.05.0.30_A_292

AGACCCCCAACGTACACGTGGGGGTCAGGATGTACAGGG

>M.05.0.30_A_293

TTTACATATACAGTAATACTTACTGAGAACGGTGCTGGTA

>M.05.0.30_A_294

CAACTAAATAAATAGAAATTTCACTAACCAACACATAACT

>M.05.0.30_A_295

AATTATATAAAAATTGCGATCGCGTTCGGAGCTGCGGCATT

>M.05.0.30_A_296

CTTACTAACCCCGCCGCCAAGAGAAACAGTACTCCTAATTC

>M.05.0.30_A_297

GAATTAATTATGAGTTCTACCCTATCAAGCCAAAGTAAAT

>M.05.0.30_A_298

ACACTTAGGTACGCATCTAACAACAACTCTTCTAACTTATT

>M.05.0.30_A_299

TAATGAAAACATCTCTGTCAACTCTAATTAAATTCAGAT

>M.05.0.30_A_300

CTACTCTTAACTACAACTACACTAAGCCGTTTAGTAAT

>M.05.0.30_A_301

TTGAAAATTTTGAAAAAAGACTTGAGGGGGCCTGAGAGG

>M.05.0.30_A_302

ATTACTTGTTTTTGAACAGATGAAAGTGCGTTTACAAT

>M.05.0.30_A_303

TTCCCTTTGCTCTTAATCTTCCAACAGATGCCGTTATTTC

>M.05.0.30_A_304

AGCAATGTACTGACCGCCATTAGACGCTCCAGTTGA

>M.05.0.30_A_305

TTTGATAAAGCGATGACATTACGTATATACTTTTATGAT

>M.05.0.30_A_306

TAACATGTCGCTTCATGGCCTACAATAGGGCTTTTATA

>M.05.0.30_A_307

TCTCATCAGGAACCAACGCAAACTGCAGAAAAATCAAAAT

>M.05.0.30_A_308

ATAAACTAAATATTGACGTGAAACCGCAACTAATAGATAA

>M.05.0.30_A_309

AAAGCAGAAAACGGTAGAGGTTGTAAAGAGGCTAAAGTCA

>M.05.0.30_A_310

ATCAGACGGCAGTATCGATAGGGTTGCATGTGATAGATAT

>M.05.0.30_A_311

TCGGCAAGCATAGTATCTTTGTTTAACACTTCACCAAGGT

>M.05.0.30_A_312

TTCAATGTCGTAACAGGTACTGTATCATCTTCGCTAATCCC

>M.05.0.30_A_313

TCTATGTTTCTCTTCTTTCTATAAGCTAAGAAGGATCCATT

>M.05.0.30_A_314

ATCTACCTTACCTACCTGCTTTCCGAACCCTATACCAGCGT

>M.05.0.30_A_315

GTTTCAGTAATTAGCAAACTGATATCGCTATTTGGCACT

>M.05.0.30_A_316

ATAATGAAATAATACATTTGTTTGTAAATTTAGCGGATTATT

>M.05.0.30_A_317

ACTAAGGTCCTGTCTGCTGCTACTGCCTACCGGTCGCT

>M.05.0.30_A_318

TCACTAGAAAACTTCCTACTCTCATCTTTTCATTGTTTT

>M.05.0.30_A_319

ATCAATATAGCTCCTATTGATGCCCATTTTTATTTCACTTT

>M.05.0.30_A_320

CAACGGGATGAACGTTAGTATGCGAACAGGAGCACCTTTGT

>M.05.0.30_A_321

ATTTAAGCAAAACGCGATTCTTATGTATAGTCATCGATAT

>M.05.0.30_A_322

AAACTACGTCCACGTATTCTACTTGTGAATTAATTACCG

>M.05.0.30_A_323

GTTAAAATATCAGTCCAAATTGAGGGCATCTATTCTTATAT

>M.05.0.30_A_324

CAACAATATTTACAAGCACTGAATCAATTAGGGAGTTCAAGG

>M.05.0.30_A_325

AAACGAAATATCGAAGGCGTCAGATGAGGAAAGGAAGAA

>M.05.0.30_A_326

GCTTGAGGGCGACCGGCAGCGTTAGGACGAAGTAGTTCC

>M.05.0.30_A_327

TTTATTGAAAGGTCGTTTCTGACAGCTTTATTAGGATC

>M.05.0.30_A_328

GTAACTACTTCACAGTCACCCCTTCCTATGTACGCACCGT

>M.05.0.30_A_329

TAGCCCACACTTTGTACAAAGTAGCAAACCACAACCTGA

>M.05.0.30_A_330

ATATAAATCCGAAAGCACGTTAGTGAGAGAGAAAGATTT

>M.05.0.30_A_331

GTTAACTTGGCAAAATCTTCATACGGGGTTCCTGATGTAG

>M.05.0.30_A_332

AATTTAGGGTCTGCAACCGCCACATTGAAGCCCTTACTAT

>M.05.0.30_A_333

TACTAGAATTAGTTCCTCATTACTCTTAGAAGACTTCTC

>M.05.0.30_A_334

CCAGAGACTGAGACAGAGCCTGAAGTAGTCCAGATATTT

>M.05.0.30_A_335

TGTCCTCTTCTATGGGCATGGACTTAGGATTAGGGTTTTTA

>M.05.0.30_A_336

TTTCCTGTTCATCAGTCCTTATGTATTTACTCTTTATTT

>M.05.0.30_A_337

GTTCTACAAAGCGGTTCTCAGATCATTATAGATGATGA

>M.05.0.30_A_338

TAATTTAACTAATAACGCGGGGGTTTAAAAATTTAACGGT

>M.05.0.30_A_339

GACATGGAATTATATATTAGTTACATTAACAATATCTGTAGT

>M.05.0.30_A_340

TATTTATACAGTAAAGTTTTGGCAAATTCCAATTGTAA

>M.05.0.30_A_341

TCATTATACTCAAGTGCAGTATACTTTGCAAATGCATT

>M.05.0.30_A_342

TAACTTCATTTGAAGCTGAAAAGAATGCTAACCAGGGC

>M.05.0.30_A_343

GCGATAGATCAAATTAGAAAGACCTTGCCACCTAGAGGA

>M.05.0.30_A_344

TCTTGTATGATAATCCATTCCTCAGTTCTATCTTTAGTAT

>M.05.0.43_A_1

AATTTACTATTTTCAGTAATCACATCTTGCACTACTAA

>M.05.0.43_A_2

CATAAAGTATATGTGCTGAAGTCTTCTTTGATCCGATA

>M.05.0.43_A_3

ATTATATATCCCGAGAAAAGAAATATCATAAGGATCCGAC

>M.05.0.43_A_4

CTATTACACACATATAGTGTTGTTTAATCTCATCAATGT

>M.05.0.43_A_5

TTGGCTGGAACCAGATATTGCTGAGGTAAGCTAGTTCC

>M.05.0.43_A_6

GTAATGATGAACTTTACAAAATTCCTCATTGTATTAGCC

>M.05.0.43_A_7

AACGCTTTATGTGAAAGTTAAGGATTCTTATTATCCGCT

>M.05.0.43_A_8

TTGCATAATCTTTTCTGAATCCTAATGCCATCTTTTCCTT

>M.05.0.43_A_9

ACATCTTTGTGGCCGTGAACTTCCTCAGATATTTCGGGGCTA

>M.05.0.43_A_10

AAGACACAAAGTCATGGGTAATTGTAGAATGGAGTA

>M.05.0.43_A_11

TTTCAAATCATTGTAGTCCCAAATAAAGGGTATTTTTG

>M.05.0.43_A_12

TTAACCCTACTGACCATTGATAACCGTAGTCATATGATG

>M.05.0.43_A_13

ACTGGTTTATTTTCCTGTTTCAAAAGCGTTATAATATCAT

>M.05.0.43_A_14

GCATTACTAATAGGCGACCCGGGTACCGGTAAAACGGAA

>M.05.0.43_A_15

TTTATTATTGAGTTGTTTCAAGATGTTGGTATCATAG

>M.05.0.43_A_16

TGATGTGGGTAATGTCCACCATTTTGCAGTAAGTTGCTTTAT

>M.05.0.43_A_17

CTCGGTAATCCTACCAGAATTGTATAACTTTGTGTAGATG

>M.05.0.43_A_18

AGTTAAATAAAAAGAAGGAGGAGGAGGCACGAAATGAGT

>M.05.0.43_A_19

AAGACTTAGATCATGGTGTGATACATCAATATTTAGGA

>M.05.0.43_A_20

TGGAAGGAATTTTGGAAGCCTTCTAAGTTTAAGAATTGGCTT

>M.05.0.43_A_21

CTTTCCAGTAACAGTTTAAATAACAGCTCTACCCTAGTCCC

>M.05.0.43_A_22

TAAATCTAACTCTTGCGAATAAAGAACAGTTGGCAAAAT

>M.05.0.43_A_23

AGCAGTTAGAGATATTTCAGCTGAAGGAAACGCTGAGAAAGATAT

>M.05.0.43_A_24

CCCATGATTTTCAATTCACCAATTGTACAAAAATATATTCTT

>M.05.0.43_A_25

GTAATACTCAAAGTGTTTATATAACACGTGAGACGCAA

>M.05.0.43_A_26

GAAAGCGTAAAGATGAAGTTACTAGACGAAATCAAAAA

>M.05.0.43_A_27

TTTTGTTTAAACTCCTCCAGTAATTTTTCTATCTCATCTT

>M.05.0.43_A_28

CATTATCCATAAGCGGTAGCGGTACATTGGAGATAGCCT

>M.05.0.43_A_29

ACAAATCCGTATCGGATCTAACACATCAGATCGTTCCAT

>M.05.0.43_A_30

TTCAAGTAACTCTTTTTCTTGAGTTTGGGCCATTTTGGGG

>M.05.0.43_A_31

ACTAAAACACAACTAAATATAACACTATATAACACACACA

>M.05.0.43_A_32

CAAACGACGCCTATCGTAGATATCATTGAGTCCTCAC

>M.05.0.43_A_33

ATACCAAGGCTGAATGGAGGCCTAGAGTAAACCCCCCA

>M.05.0.43_A_34

TCTGATCTGTTATCAGGTCTAATGAGTATTTGGCCTTA

>M.05.0.43_A_35

GTGTATAACCTCACTAAATAAATTAGGTGTAAACCCAGAT

>M.05.0.43_A_36

GTAGACGCTACTGCATTAGGCATAGGTGTTATAAACGT

>M.05.0.43_A_37

AGATAATTAAATTGAGCGAAAGTGAAAAAGATATGTTAAA

>M.05.0.43_A_38

AGATTTGTCATTTGAATTATATAACTATGAGTAAATGTTA

>M.05.0.43_A_39

ATTTATAACAAACTCCAAGAATGGGAGAGAGTAAACATA

>M.05.0.43_A_40

TTTCATGTTTTTGTTATCATTCAATCCTTGGCAATTGAGTGT

>M.05.0.43_A_41

TACTAGAAATCCCGCCACTATTCCTATGAAATTAGTTAGCAA

>M.05.0.43_A_42

ATTAGTTTTATTAAATGATCCATCAATTCAAACTTTGACATGT

>M.05.0.43_A_43

TCTTTTCTTCATTCTCAGTCATAGACGAGACACCTC

>M.05.0.43_A_44

GTTTGCTGAAGTAGTTGATAGAACTCATATGGATTTAC

>M.05.0.43_A_45

AGGATAGCCAAACATGCCTAACAAATCATCTAATTTT

>M.05.0.43_A_46

AGTAGTCCATTACCTTCTCCATCATCTCCATTAATGGGTGC

>M.05.0.43_A_47

AATCATCTCGTTACATTACTCACAGACGTTAAGTCAATCA

>M.05.0.43_A_48

CAATGCTGCAGCAGTGTTTTTTAGCTAAAACTAGTAAT

>M.05.0.43_A_49

GAAAAATAAAAAAGCTTTTTAGAAATATACTTCTAAATA

>M.05.0.43_A_50

CTGTATCTCCCCAATACGTCAATACACTTCCAGAAGACGT

>M.05.0.43_A_51

CTATATATTCCAACATCATATGTAGGTAAAACGTCT

>M.05.0.43_A_52

ACAATCCGCAATTAATTCTTTACATTCCTCCAAATATTTT

>M.05.0.43_A_53

ATGTATCGCACATCTTTCAACTTAACGCATATTTCAT

>M.05.0.43_A_54

ATTAAGTTCATCTAGATGATATATAGTTTGTGCTTTT

>M.05.0.43_A_55

CGATACTCGAACGGTTGCGTACCCCAAGGACGTAATTCTA

>M.05.0.43_A_56

CTTATTCCCGAATGATTTCTTCCTCCTATAGATTTCCTCAA

>M.05.0.43_A_57

TTACTTTTTGCAATTCTTTAATGCTTTCTAATAGTTTCAT

>M.05.0.43_A_58

GATAATGCTTTAATTTGTGGGCAAACTCGGCAAGTTCCGT

>M.05.0.43_A_59

ACCACTCCCGTTAAACTTTTACTAACTTACTAACTTACA

>M.05.0.43_A_60

TGAGACTCCATAGATCTGATTCCCGCTTCTCTTTCCCTCT

>M.05.0.43_A_61

AACGGTGAAAGTGCTACTTTATCATACGGGCAAGCATTAGA

>M.05.0.43_A_62

ATTTCCGTCTCTATCTCTTCTATATTCGTAGTCTCA

>M.05.0.43_A_63

TTCTCTGATCTTTAGGTACACATATATCATATGATTCCTC

>M.05.0.43_A_64

GTTTCCATAAGATATCGCAGAATAAATGGCGGTAATGGTCT

>M.05.0.43_A_65

TTTCGCATATTCTTCCACATATTCTCTTGTTTGCTTTAAG

>M.05.0.43_A_66

AGGTGTATAGGTGACATGTGATATCTGTTCCTCCTCCTGAT

>M.05.0.43_A_67

AGAGAATGATATTAAAATTAAAAGCGTACAAACGCAGA

>M.05.0.43_A_68

CCTTATAGATACTTGTTTAATCATCTTCATTTTTGTAAT

>M.05.0.43_A_69

CATTTATGTGGATCTGGTTGTTTCTCTTTAAGAAGATAA

>M.05.0.43_A_70

GTTGTAATATACGCTGACGGACAAAAACTAACACAGGGATTCT

>M.05.0.43_A_71

TAATGTTCTAATCCCCTATGCCTCACTGGGAGGGGCATA

>M.05.0.43_A_72

AGTTTAGATGAAATTAAGCAAACGTTAGAAAAAGCGAA

>M.05.0.43_A_73

AATCAGGGGATGATACTAGTTTTCACATACAACGGACAGTCGGT

>M.05.0.43_A_74

GATTTGTAGTTCTATCAGTTACTTGTATCGTTATTGAT

>M.05.0.43_A_75

GCTTTCTGCCGAACTTTTCAGCAGTCATTATAGCTCCGT

>M.05.0.43_A_76

TCTAATAATACTAATAATAATCAACAAACTCAGACTACAT

>M.05.0.43_A_77

TTTGAGTAGTTACAGCGTGAAAAGGAAAGGAATAAGGTAT

>M.05.0.43_A_78

GGTTGTAAATACGCCACGTGAATTGGAGCGCTAGACGCGGG

>M.05.0.43_A_79

TGTTTCTAAGTTTTGCGACGCTTTCCCTAAGTTTGT

>M.05.0.43_A_80

ATTAGAATCTATGAAGTTGAAGACGGAATAAGGGTCAAAA

>M.05.0.43_A_81

TCATATTATTTTATCTATGAAACAGAAAATGAGAATTTTTA

>M.05.0.43_A_82

TGGAAACCGCTCACGTAAACACTTATAAATCCATTCTCTTGT

>M.05.0.43_A_83

TGTGGCAACACAAATATAGCTTCACAGATAACATCCCTTTGT

>M.05.0.43_A_84

CTCATCTGTGTTCTTTTCGTTAAAGTTACAACATATATAAT

>M.05.0.43_A_85

GAAGGCAGTGCAATCTTATATGTGTTCCCTTTTTCTTTTGGT

>M.05.0.43_A_86

AACGATGCTACCGCATTTGTTAATAAATTTCCTAAGTTTTGT

>M.05.0.43_A_87

ATCTTTGAAAGATAATTAATAAGTAGCTCTAGATCCTTT

>M.05.0.43_A_88

TTCAATGTTAAAACTGGAATAAATGGTGTTGAATATCCT

>M.05.0.43_A_89

ATACCTTTTTAACGTCGGATACCATAATCATTAATCCCGC

>M.05.0.43_A_90

GATCCTGTGACACTCTCGCTCGCTTCTGCTCCGCTAATC

>M.05.0.43_A_91

AATGATTGAACTACTAAAGTCAGTGGCGGAACTGCAGGCT

>M.05.0.43_A_92

GTTGTAGTTTTAATATGACAGCATCTCTTACATATTCACT

>M.05.0.43_A_93

CCCGCATTTGGGGGGAATTCCCCCACTACTAATATAAGT

>M.05.0.43_A_94

TTAAGTCCCCACATCGTTCCAAATTGTTTTTCACATAA

>M.05.0.43_A_95

AAGACTGCTACGTTAGTATATCTCCCTACGGGGGGATC

>M.05.0.43_A_96

TTATAATTATAACTTAAGTCTAGCTCATTTGCTAACGCA

>M.05.0.43_A_97

AGTGTTAGTATAAATCGCGAACAAAATAAGCGGATAAAGAGT

>M.05.0.43_A_98

CCGAGACGCCCTGGCGAGGGTGATACACCTCTGCCCCACCCTTCG

>M.05.0.43_A_99

TAATAATCTGTAACGCTTACAGTTATCTTAACTGGA

>M.05.0.43_A_100

ACTATTAATAGCTCTTGTTTAGTGACAGGTATTGTTACTT

>M.05.0.43_A_101

CATACGCTTTTCTCACCTCTTCCTCAGCTCTTTTCTCCGCA

>M.05.0.43_A_102

TAACGATATAGATCCAGTAGCTTTGGCTGCAGTCCTCTTTTT

>M.05.0.43_A_103

AAGATAACACATTTATTTTCTACGTAGTACTTACATACT

>M.05.0.43_A_104

AAAGTTGAAGAGCAAAAAGAAAAACCGAAAGAGGAAGAAG

>M.05.0.43_A_105

TTCTGTTTATGCAGCGCAGTAACGAAGTTCACCATTCTG

>M.05.0.43_A_106

AAAATAACTGACGTATAACTTAGTGTCGCAGTATAAGTCTT

>M.05.0.43_A_107

AGTCTGATATTGACGATGCCATGTCCGTGATCAGTAGCCTA

>M.05.0.43_A_108

AAATTACTAACCTTACTATCAATTTTTTTAAGTAAATCTT

>M.05.0.43_A_109

GCTTTCCGAACCCTATACCAGCGTCATCGATTATTATTAA

>M.05.0.43_A_110

CTCGATGTTGACTTCAGCGAAAGTGATCTTGAAGAAAT

>M.05.0.43_A_111

ATACCATCAGCTAAATATAATTCGTTGCAGTAATCAAG

>M.05.0.43_A_112

CGATGATTTCCCCTACGTTCATGGTAAACATGTTCAAGC

>M.05.0.43_A_113

GTTACGGGTACTGTTTGTGTATAATATGAGGGGTAACTCATGT

>M.05.0.43_A_114

ACGATAGGTGCACCTATTCCTACTCCTAATAGTCCTCCTA

>M.05.0.43_A_115

CAGCACGCTGGAAGCTAGGGCGTCAGCCTAAATACA

>M.05.0.43_A_116

TCTCTTAGTATTGCTCTCCTTACTTTTTTGTTACTACATAT

>M.05.0.43_A_117

ATTAATTCATCAGTATCTTTATATCCTTTTGATACCAAT

>M.05.0.43_A_118

CCTACCGTGACTGCGACATAATGGGGTATTATTGCATAAT

>M.05.0.43_A_119

ATACCCCTACACTGGTATATATTCAGCCGTATATAG

>M.05.0.43_A_120

ATTATATTATCAAGACCTTTAGGTGTTGTGTATGAGATA

>M.05.0.43_A_121

ATTGACTACATTCAAGCCACCAACGGTAACGATAAATTTACGT

>M.05.0.43_A_122

CTATATCTCCCTAAAACATCGATACATTTCCAGAAAAC

>M.05.0.43_A_123

GTCTAGTTTTTTGTGCCCCGTTATTTTTGCTATGAT

>M.05.0.43_A_124

CTTATTGCATAAATCTTTGATGAGTTTGAAACGACATGGT

>M.05.0.43_A_125

CTTTATTGAGCGTTTTTTAAGGTTGAATAAAAGATGAAAA

>M.05.0.43_A_126

GAAAGTTTGAAAGTTAAGAAGACTAAAGCAGATTTGAAAAT

>M.05.0.43_A_127

CTCTATTTCTTCTTCGAATTTCCTTAGTGTGTAATCTTCA

>M.05.0.43_A_128

GCAAGGTCACTGACCCCAACCCTGATAAAAATTATTGACTT

>M.05.0.43_A_129

GAAGAGATTATAGCAAAATTCGGAAGGCTAAAGGCTAAC

>M.05.0.43_A_130

ATTTTATATGATATATATTGTCACCATTATGTTCTTCA

>M.05.0.43_A_131

CGCCGTCGCCGACGCGGGGGTATGTAGTCAGTATTACT

>M.05.1.5_A_1

TCTATTATAATCGATGAAACAGATAGAGCTGAATTGTGTAC

>M.05.1.5_A_2

GTCTGTGTAAAGGGTGTCTCCGTCGACCTCGGCACGC

>M.05.1.5_A_3

GCGCACGGTTCACAGTTCGTATTCGACGACAGTAACATAC

>M.05.1.5_A_4

TTCCCTTGTTGAATTAGTAACTGTTGTATCTGATTTG

>M.05.1.5_A_5

AAATTCTTTGTACATAGTGATGCAGATATCAAAATAGCGAT

>M.05.1.5_A_6

GAGTTAAAAATTAGATTAAATTATGAGTCAGGTAATCGGTTTT

>M.05.1.5_A_7

GGACTTCCACCTGTAGCTAGAGTTCCATTGAATGCTTGCCAA

>M.05.1.5_A_8

TTAACACTTAAACTTGAGTTATCATCATCAATAGATA

>M.05.1.5_A_9

TTGACACAAACGGCGTTAAAACACTTGCTTTCAAAGAAT

>M.05.1.5_A_10

AGACTGCAGAGAATATGTATTCAAATGCCTGAAGATGAT

>M.05.1.5_A_11

TATAAGATATCGATGACAATACATAAGAATAAAGTACTGC

>M.05.1.5_A_12

GTTGAGGAAACATATGCAGACCATAACTCGATTGATGCCGT

>M.05.1.5_A_13

ATTTTAGATCAAGTGTGGCAGCATATACAAAGTTGCCCAG

>M.05.1.5_A_14

GTGCGGAAACTTATCAAGACCGTGTCACTGACTGCACCG

>M.05.1.5_A_15

TCCTCACCCCGTAAGGGGGTCATCGCCATCCGCTTT

>M.05.1.5_A_16

TGCTTGTTCCGCCATTGACAACGCTTGAGAATTTCCAT

>M.05.1.5_A_17

TTTTCTATCAGTCCACCGCAAGGAGTTACTAGAACAAT

>M.05.1.5_A_18

GGTTGATATGCATATGTTTGTTGAAGTGTACGCGAAAATA

>M.05.1.5_A_19

AAAATAACGGTGACAATAAAAGTAGATGAAAATGATCTAGT

>M.05.1.5_A_20

TCTCTTAGTATTGCTCTTTTAACTTTTTTATTACTACATAT

>M.05.1.5_A_21

CGGGGGGTTCCCCCACCCCCACGGGTGAGAATAAGGGGG

>M.05.1.5_A_22

GTCCTTAGTTTTTCTTTTCCTCTTCTTCTAAATTTCATTC

>M.05.1.5_A_23

GCATTTAGTTGATATGTGTAACTTGTTGTCCCGCTAATGA

>M.05.1.5_A_24

AATATAACGCTGACAATAAGCTTTTGCGTTCTTTCGAACCC

>M.05.1.5_A_25

TAAACGAATATTTAATATTGTCTTGATTAATCATTTTAAT

>M.05.1.5_A_26

TATACAAAGTTGAAGTCATTTGTTGTTACATTTATGATGC

>M.05.1.5_A_27

TTACCACTAACTGCAATAGGTGTAAACTTACGTGTTGCA

>M.05.1.5_A_28

ATTTATAACAAACTCCAAGAATGGGAGAGAGTAAACATA

>M.05.1.5_A_29

CTTCACAATATTCTTCCTCCTCCTCTTCATCATAAAGATAT

>M.05.1.5_A_30

TTTTTTGTTGCACATAATATTTTAACATTTCTGTTAGTCTT

>M.05.1.5_A_31

GTATGGAATTTGATGAACGAAATGAAGTACAAAATAGACGT

>M.05.1.5_A_32

GATTATACTAACACCTAGAATAGAATCTAATATATATTTTC

>M.05.1.5_A_33

CTCCCAACGTTCCTGGGAAGCGTAGGTCTAGCACTA

>M.05.1.5_A_34

ACCAATTCGCAAAATCTTTATGAATTGCTTTAACTATAT

>M.05.1.5_A_35

GGTATTGTATTAGGTGGTATTTGGATAACTTTAGCTTG

>M.05.1.5_A_36

CAAAACGGGAATGGGAGGAAGGTCAGACACCATCTTA

>M.05.1.5_A_37

CTAATCTATTACCTGCTTACAAAATGTTTCCCGTTGCGG

>M.05.1.5_A_38

TAATTTTTTCGTTGTTTTTATCAAAACACAAAGCACTA

>M.05.1.5_A_39

CAAATCTACAAGATACGTGGGTTACTATCTCACCGCG

>M.05.1.5_A_40

TTACCATATCCGATAACGGGACATAAACATCCGGCAA

>M.05.1.5_A_41

TTAGCCCAAATGCTGACAAAAGACCCCGAGAAAGTAAA

>M.05.1.5_A_42

GAATAAGCGGGGCGAAGCCGACGTGTACGCCGTGTCTTTC

>M.05.1.5_A_43

TCTTATATAAAATCAAAATTGTATGAAATCCAAGCTGAAGTTTTT

>M.05.1.5_A_44

TTCGAAACTAACCTCATAATTACTGCATAGGCGTATCTCTG

>M.05.1.5_A_45

AAATTGATGTTTAATGTTGGTAGTGTAAAGTTTGAAGGAA

>M.05.1.5_A_46

ATTTTTCCTAGATAGCGTTGAAAATCTTCTGATTGTTTC

>M.05.1.5_A_47

CTCAAGGCAATTATAAATGAGGTGTCGACCAAGGTACCT

>M.05.1.5_A_48

GTGACTGAGGCAACGGATAGTGTTACATTGCCTCCAAT

>M.05.1.5_A_49

ATTCCAAAGTTACGTCATCGATGACCACGTAATGGAAA

>M.05.1.5_A_50

GAAGTCTGCCGCTTAGTACTATGAGCTTAGAATCTATA

>M.05.1.5_A_51

TCACTACGACATTTAGGACATGGAATAGCTTCCACAAT

>M.05.1.5_A_52

TTATATTTTAATAATTCTTTTATTGCCCTACGTATCGC

>M.05.1.5_A_53

AACGGGATTCTGAAGAACTATCAAAACGTGAACAATATTT

>M.05.1.5_A_54

TGTTTTGTATACAGACTCACGAAGTTGTAGCCGTACCAA

>M.05.1.5_A_55

GCGCACCCACTGCTACTGCTTCCGCAACGTTTACAGCATCT

>M.05.1.5_A_56

TCGAAAACCGGAAGGGTACTTATAATTATTAGTGCAGT

>M.05.1.5_A_57

TCAAATGTACCCCGCAATATCCTCTTTCCTACATTCACTT

>M.05.1.5_A_58

ATTTCAAAAAACGCTTGATTCTGAGTAGAACTCACTGGA

>M.05.1.5_A_59

TTTTCTTTATCCTCTTTATTATTTCTTCTAGTTGTTTAT

>M.05.1.5_A_60

GTTTTGTTCGCTTCGTTTAACTTAAGAGAAGATTCAACT

>M.05.1.5_A_61

CAAGTTCTGATATTCGTGCTTGCAATAGCGATAGTAATAG

>M.05.1.5_A_62

TTACGATACATTGAGCTTCTTCCAGTAGGGACTATTACGG

>M.05.1.5_A_63

TCTGGTTACTACCCGTTCCAAAACGGCTATATCGACAT

>M.05.1.5_A_64

GCATTCAACCCCGCGGGGGAATCCCCCGCCCCCATGGGGG

>M.05.1.5_A_65

CTTTCGTCACTGAATATCTCAAATCCATCCAGACCTCATT

>M.05.1.5_A_66

ATTTCAGTGTTTACCCACCTCTTCGCATAGAAACGAAT

>M.05.1.5_A_67

TCTACAACATCAACTTTAGGCTTTTCCAGATATTCAAA

>M.05.1.5_A_68

TATTGATAACAAAGCGGTAAACGAAACGCTGAACCGTTAC

>M.05.1.5_A_69

CTATGGTCGAGTGGCGTAAAAAGTTCCGTTTCCTCGTCAT

>M.05.1.5_A_70

TTTTAAACAAAATAAACTGTCCATCCTTCCTCACATTTCTT

>M.05.1.5_A_71

CCTCACTATATATGCTTTACAAAACTACCGACACGTATTATT

>M.05.1.5_A_72

ACAGACCAGACAGCTCCTATTACAGAAGGGAAAAGGGTAGCG

>M.05.1.5_A_73

TTGGTAACCTCATCACTCACCGTTGTTAAAAATACGT

>M.05.1.5_A_74

TACTTCATCACGTCCACGTTTGTTATTTTTGTTGATTTAA

>M.05.1.5_A_75

GGGCGGGTTCAACTTCAGTGGGCTGTCTCGTCTCGTTA

>M.05.1.5_A_76

ACGTTAAGACCATTTACAATTGCGAATAATGTAATTGT

>M.05.1.5_A_77

TAAGAAACGCTATCGATACTTCAGAGGCCGGCGCTAGG

>M.05.1.5_A_78

CAACTCACGAATGAAGGGTTGATAAAGCGGCAGCTGCCC

>M.05.1.5_A_79

TTTTACTCACCTAGTTTGTACAATTGTACAAAATCGAAT

>M.05.1.5_A_80

TTCTGCATACTTCTTATACTCTTCTTTAGCGATTGCAAA

>M.05.1.5_A_81

TGCAGTAAAACCGCAAAATGGACAAATTCCACCTGGTTT

>M.05.1.5_A_82

TTAAAAATTAGATTAAATTATGAGTCAGGTAATCGGTTTT

>M.05.1.5_A_83

ATTCTATATTCATTCAAATTAGAATTATATGCAATTTCT

>M.05.1.5_A_84

AATCCACATGCATTTTATAAACCATAGCAACTGCATT

>M.05.1.5_A_85

CTAATTTGACACTGACCTTCTCTGTTACCGGTGCCGG

>M.05.1.5_A_86

TATTTAGCTACGTATACAATACTGTATAACATATTCGCT

>M.05.1.5_A_87

AGAGTACTCCCTGCGTAAATTCCGCCAACAATAGATATT

>M.05.1.5_A_88

TTCTCATCTAATATTATAGTACCTATATACATAGGTATTA

>M.05.1.5_A_89

TTAGCTGGTACTTATGCCGACGTCGAGACTAATAACGT

>M.05.1.5_A_90

AGTTATTTACCGCAGTCTCGGGGTTCCTAATTGTTTTTGC

>M.05.1.5_A_91

ATAAAACTAGAGTTCGAATACCGCAAAACGGGAATGGGTG

>M.05.1.5_A_92

TTATCCTCACTCATATCCAGTTGTAATTTCAGCATGAC

>M.05.1.5_A_93

ACGAGAGTGTTAGGATCTTCCCATCCTACCCCTAACGGG

>M.05.3.4_A_1

TCTGTTATTCTTCCCTTTTCGTATATTTTTATTAAAAT

>M.05.3.4_A_2

CTTCAGTCTCTACCGTTAGGGTAAATGGTAAATAACC

>M.05.3.4_A_3

AAATTATTGAAATAAAAATCAGATTCGATATAAAATT

>M.05.3.4_A_4

ATTGGAATCTCGTTTATTCCAGCACTTGTAAACGAACGAA

>M.05.3.4_A_5

TAACCTTAGATACATCGCTGACGTCAGTAGATATGCAAA

>M.05.3.4_A_6

TCACAAGTTTCTTATCTTTATTTATAGATGGTATTATAT

>M.05.3.4_A_7

ATGATACCAACATCTTGAAACAACTCAATAATAAACAAG

>M.05.3.4_A_8

CTAGTCTCCAACCACTTAACTGGGGAGTTAAGTGGTTA

>M.05.3.4_A_9

GGTTTGATGTACTCTTCAACTATCAATTTTGGTTTACCTT

>M.05.3.4_A_10

CACTGAACTTAATCGTGAATTATACAGAGGCCAAAGCCG

>M.05.3.4_A_11

AACATAAAAGCAATTATAGTTATCACAGACATCTTAGTAC

>M.05.3.4_A_12

CTACCATCAATAATTGCACCGGGTCTGATGTACTCTTCTAC

>M.05.3.4_A_13

AATACTTCATCAATAAGTAGACCACTGTGGTCAATTGGCGT

>M.05.3.4_A_14

AGTAATGTAACGAGATGATTAGGTTTGAACGTGTAAGG

>M.05.3.4_A_15

CTTCAAATTGAATGCTTAATCGTTTATCTTTTTTATAAT

>M.05.3.4_A_16

TAAATAACGAAAACCTCCTTACCAGACTCCATCTCTTCCTC

>M.05.3.4_A_17

CTTTTTAACATTAATATAAATAACATAATCGATATCAA

>M.05.3.4_A_18

CTATATTTAAACATTTGTCTGGCAGACTTTTAAAGTTTC

>M.05.3.4_A_19

ACAACAGAACCAGCTGGAGCACTTGCCGCAATATTAAAG

>M.05.3.4_A_20

TTTACAGTGATGCGTATCGTTTTCAGGATCAAGAAGTGG

>M.05.3.4_A_21

TTGAAAATGATGATGAGCCTCATCTATCACAAATAAAGTA

>M.05.3.4_A_22

AAAATATATTCTTAGCGGTGCTAAAGTAGTGACTACAAT

>M.05.3.4_A_23

AGATGGCTGCAGTTATGAACAAATTAATACCCGCCATAG

>M.05.3.4_A_24

TTTCTATTAAGAGAAAGTTACACTTTATCAAAAGACACT

>M.05.3.4_A_25

ATTTTAGTCAAGAAATCTATTATATCTTGAGTATTAAT

>M.05.3.4_A_26

ATTGTAGTCAGAAAGTCTATTATATCTTGAGTAGTAATT

>M.05.3.4_A_27

TATCTTTTAGCTACTGGTACTACTAAATTATTGACAA

>M.05.3.4_A_28

TTACGCCGCAAAGAACGGGAAGAGTACTTATAATTATTAA

>M.05.3.4_A_29

ATAAAAAGACCATCTAGTAAAATTCAATAGAAGTTTT

>M.05.3.4_A_30

GTGGGTTTACAATAGTGATGAAGTAAGGTATTTGCGG

>M.05.3.4_A_31

CTGAAGTCAGCATAATTAGAAAGGGACCATAAAGTA

>M.05.3.4_A_32

CATATCCTCTCTTCCCTCACCCAATTGAAGAAGAACCCTA

>M.05.3.4_A_33

TATTTTATACTCGCTTCATGTACACCATTTTCTGTAACTAG

>M.05.3.4_A_34

AGAAGAGGAAAAACTTGTAAGTCAAAATTCAATTTATTT

>M.05.3.4_A_35

ACCGCTAGCTCCGGAAGGAACTATTATGTTTATCGTTTTGC

>M.05.3.4_A_36

CTGTAGAACTCGTCCTCGTCGCCCCAAAGCGGAGCGAAGT

>M.05.3.4_A_37

TTAATTAATTATTTAGTCCTTATTATTCAAGTGTCGGG

>M.05.3.4_A_38

AAGATCGTGGACGCGAGCACCGTAGACGAAGAGATAATCCT

>M.05.3.4_A_39

CCATTCTGTGTAATTGAATTTTCTTGACTTTTTCTTCTCTTT

>M.05.3.4_A_40

TCATCTCACCCTAGCGTTATTATATCATTATATAAGGAT

>M.05.3.4_A_41

CTCGGCTCGTGGGCTCAGATCGGGAACACGGTGTACGTG

>M.05.3.4_A_42

AATCCTTATTCTTTGACCTGCTGAATAATAATTTATTAATT

>M.05.3.4_A_43

ATCGCATTCTTTTTTCTTGAAATATGAAAAAAATCCGGC

>M.05.3.4_A_44

GTAACTACTTCACAGTCCCCTCTTCCAGTATATACAC

>M.05.3.4_A_45

TAGCGTCGTTATTTAGCCAAGCTCCTTCAACAAGTAGT

>M.05.3.4_A_46

TGTTCTTTCGCGATCATATAATAGTACTAGAGGTGCTTCTT

>M.05.3.4_A_47

TTAAACTATTATAAGTCTATGGAAGTGACATTTGTCTCTAA

>M.05.3.4_A_48

AACTCCAGTACTGCTTCGCCGTAGGATCCGTCCTCAGACG

>M.05.3.4_A_49

TATACCTTGACTTAAATCTACTCCTTCTTCCTTACAA

>M.05.3.4_A_50

GGAAACTATAATGAACTTATGAGAATTGGGAATGTTGAA

>M.05.3.4_A_51

CCTTACTTTTTCTTCCCCTTTGATACTTAATATACTTAA

>M.05.3.4_A_52

TTTACTTTTACAGTGATTCTAACAGAAAACAGTGCT

>M.05.3.4_A_53

CTCTTCGTTGAAGAACGACGAAACTGTTTCAACCACATATT

>M.05.3.4_A_54

CAAATAAAGGCAAGCCCGCAGGCTAATACACAACCTACATTAAA

>M.05.3.4_A_55

GTTTCATCGAGACCACTTAGGAAGAAGTCCTCTAGTTC

>M.05.3.4_A_56

TTATCTTTCAACAATACAGTATGCATGTTTCAGGGATGCCG

>M.05.3.4_A_57

GATTCGTAGTTCTGTCTGTTACTTGTATCGTTATTGATTGT

>M.05.3.4_A_58

AAATTACTCACAAAAGGTTCAAATTCATCATAGAAATAT

>M.05.3.4_A_59

GTAATCATATCAGGAAACGAAGATGTTTACTTTCCTAATA

>M.05.3.4_A_60

TCTATATTATTAACTTCTATATCTATGTAAAGTACCTTAT

>M.05.3.4_A_61

CACGATAAACATCTTTCTCGACAATCTAGCGTTTGCTCTAT

>M.05.3.4_A_62

TAATAAATTATCTTTATTGTTCCCTCATAATCATATTTAA

>M.05.3.4_A_63

GGATTAAATATAGAAGTGAAACAGAAACGAAGAAAAAAGA

>M.05.3.4_A_64

ATAAGAAACACTTCAAAAAGTGTTATCATTCTTCCTCA

>M.05.3.4_A_65

GTGTTATATTAGGGTATCTACTAGGAACAACATTATATG

>M.05.3.4_A_66

AGCAATGTAATTTATAGTGTAGTAGTGGAGGTCGTGTTTAC

>M.05.3.4_A_67

TACTCTGTATTATCCGGATAAAGATAGAATACTTTTCT

>M.05.3.4_A_68

TATCCCCCTAGAAGAATGTTAATATTAGCTAAATCTGATG

>M.05.3.4_A_69

TTTGCTATTATCTCTTCTTCTTTTAAGTTGAGTGTAAG

>M.05.3.4_A_70

ATTCTTCTAAAATCATTCGCAAATCCTCCTCCTACATCCCCT

>M.05.3.4_A_71

TTTTTATATAATAAATTATATTCTTCAGACTGAAACAAT

>M.05.3.4_A_72

CCCTATTAGTTCATCAGTATCTTTATAACCACTGCTTACT

>M.05.3.4_A_73

CCATTGGTAAAATGATATCATCACTAGAATGTTTATTATT

>M.05.3.4_A_74

TACTATCTTCCTTGCCAAATGATATCTCATTTTGGACG

>M.05.3.4_A_75

ATCCTCTTCCTGGACTCACGGGACGTCGAGGTCTTGTT

>M.05.3.4_A_76

AATGAATTCTATTTCTCTCGGCTCTCTTGTGTAGAAAAG

>M.05.3.4_A_77

AAGCCCTGGCAAACGTTTACGCTTGAAGTGGGAGTTTCC

>M.05.3.4_A_78

ATACTAAGAAGTAACGTAACAGCTTCTGAGAGAACAAAG

>M.05.3.4_A_79

TGCGATTTCGCCGTTTGGCTTTAATAGCTCGGGGTGATTCT

>M.05.3.4_A_80

ATCTTCATCTCCAAACTACTGCTATTAACTTATCTCACTC

>M.05.3.4_A_81

TAAATAATTATATCTTGTTGTTAATAATAAATTTCATGA

>M.05.3.4_A_82

TCATCCTTCTCGTGGCTATATATAAATCCACCGAAACCG

>M.05.3.4_A_83

CTCGTACTTCTTACATGATGGATAATGAGTTCTGCAGAT

>M.05.3.4_A_84

AAACCTATAAGCCCTAAACTGAATTCCTCTTCTTTCTTTTGT

>M.05.3.4_A_85

AAACGAAATATCGAAGGCGTCAGATGAGGAAAGGAAGAA

>M.05.3.4_A_86

GAAGCTTGGGTTCCAGTGGCTGCAGCACCTGCTGCAGGT

>M.05.3.4_A_87

ATTTAGATATTGTAATGCGGTCTGATAATTGCCTTGTTGTA

>M.05.3.4_A_88

TTTATCGGGGTCATCCCATGTCCGCAACCCAAAATCAA

>M.05.3.4_A_89

ATTACTTGCAAGACTTGGTGGTTTGCCCCATAGTTAGTAT

>M.05.3.4_A_90

TCATTATACTTAATTATGGATATTCAAACAGTTGAGG

>M.05.3.4_A_91

ACTGTACTTCTCTATGTTTTTTGTTCCTTATAAATATTT

>M.05.3.4_A_92

CTACGAATTTAACGCCCGCCCTAGATATCAATGATGCCTCGG

>M.05.3.4_A_93

ATTAGATTAATTTTTCTTATATTCTGAGGAAGTATGAATCA

>M.05.3.4_A_94

TCTAAGAACTCAGCTAAATCCCTATCCTTTACCGTTTGCCT

>M.05.3.4_A_95

ATAGAACCCGAAAAAGTCACATACGGCGGTCAAGAAATT

>M.05.3.4_A_96

CTTGTTCTAACGTCGCATATGCAGAATCTATTAATCCGCGT

>M.06.0.8_A_1

ATCAAAACTGCTACCAAAACCCCTGCCGCTAGATACGGCC

>M.06.0.8_A_2

ATGCAAAAACTTATAGAAAGATGGAAGTAACGAAGAG

>M.06.0.8_A_3

TCAACGATCGTTCCAGCGACAAAATATGTACATGTAGT

>M.06.0.8_A_4

AGATAAAGGAGAATCTTCTAATCACGATAAATGATATT

>M.06.0.8_A_5

CTTAATATACTGCCCAGGAATCAGGCTTTGCTAACGCC

>M.06.0.8_A_6

TTACCAGAAGATAGACCTGATTTTAGAAAGCAAGAGCT

>M.06.0.8_A_7

CATCTACATGCATTTTATAAACCATAGCAACTGCATTTA

>M.06.0.8_A_8

TCTACAATAACCCCGCCTAAGTGAATTCGGAATACTCCAT

>M.06.0.8_A_9

CTCTCGTCTAAGCGTCCTGCATTCTCTACTAGCTCGTTA

>M.06.0.8_A_10

CTCACTCCATAATTTAGTTATTTCTTCATACTCGCCGTAT

>M.06.0.8_A_11

AAACGAAATATCGAAGGCGTCAGATGAGGAAAGGAAGAA

>M.06.0.8_A_12

AGGATTGAAGACTTAGGAATGGAACCGACTGAATTAAC

>M.06.0.8_A_13

AGCCAAATCAGTGTCGAGAACTTTCAGCCTAACGAAAAC

>M.06.0.8_A_14

TGCGATATCTTCCCTTATGCTAGTAAGAGTCCAAAGCTTAG

>M.06.0.8_A_15

CTAGAATTAACGACATTCAAGAAAAGTTAGACAGTTTG

>M.06.0.8_A_16

TGCAACATCTTCATAACTTCATCTGCGCTTTTTGCATCCTT

>M.06.0.8_A_17

TTGTTGGAGAAAGAAATCCTGCCAGTTGTACCACACATG

>M.06.0.8_A_18

TAGCGATCTTTTACTATCCTCATTGCACGAAATCTCTCG

>M.06.0.8_A_19

AAACTAAAATACAATCTTGTGGGATACGGTAAAATATTATT

>M.06.0.8_A_20

AAAACGACTGAAAACGGGTTCATTAGGCCTCACCTTT

>M.06.0.8_A_21

ATGAAATGTGGACGATGATAAGTATTGATGTCGTTACTAT

>M.06.0.8_A_22

TCGGGAAAATACTACTATTACCCTGGCGGGCCTTATTCG

>M.06.0.8_A_23

CCTTACACGTTCAAACCTAACCATTTGGTCACATTACTT

>M.06.0.8_A_24

TAAGTTCTAAGAGCGCCCCGTAAGCTAGGGCTACTACTTCTT

>M.06.0.8_A_25

GAAAAAGAAGTTGTGTCAGGTTACTACAGTCCAGGTC

>M.06.0.8_A_26

TAATATTCAACTCAATATATCTAATTTTTCATATCTG

>M.06.0.8_A_27

AAATCATTATCAAATTACGTAATTTCACAAAATCAATCT

>M.06.0.8_A_28

GTTTATGAATTGCTAAGGCATGTGTCCTTAAACCTACAAA

>M.06.0.8_A_29

ATTGTGTTAATTGAACCGTCACCTAGTAAATCGAGACTGT

>M.06.0.8_A_30

TTAAGTTATTGGGGGCAATACGGAGGGTTTCTATTCAA

>M.06.0.8_A_31

CTTTTCAGAGCGATATGCTGAAAATGGGTTTCGGTTAT

>M.06.0.8_A_32

TGGTCGCCGTCATGCGTGGGAGGACGAAAGCTACTCTT

>M.06.0.8_A_33

GACTCCGACGGCTGTGTATGCGGATGGACAAAAACTAACG

>M.06.0.8_A_34

ACGAATAATGAATAGACAGTATCAGCATTTTGTATAGCTAGC

>M.06.0.8_A_35

TTCTTGTATTTTCTTAGTTTCACCGTTTTCATTTACTTCTAT

>M.06.0.8_A_36

GAGCTTTGTTTACGTATTTTACAAACTTATCGTCAACATC

>M.06.0.8_A_37

TATAGTAAATCATCATGTTTTTGTAATGTATCAAGTCTTG

>M.06.0.8_A_38

ATATATGGAAGGAGTAGGAATAGGTAAAATTATCGTGAGA

>M.06.0.8_A_39

TTAATCCACATGAGACCCCTCTCTGAGCCATATTTCGT

>M.06.0.8_A_40

ACCTATCATAATGATGGCTGACGATGAAGATAACTACAA

>M.06.0.8_A_41

ACGTAGATATCCGCATAACCACCACTTGGAGCACCGGTAAT

>M.06.0.8_A_42

ATAATTTTTTAATAGAAGATGCTGAACTTATTTTCACAATT

>M.06.0.8_A_43

ACCATCTTTAAGTATGCCCTGATCCTTGGCTTCCTGTACAC

>M.06.0.8_A_44

TCCAATATGTCATATATTATATCTAACTCATCTCTTCTC

>M.06.0.8_A_45

ATCATGTTCGAATACCAGACGTCAAATGATATAATAAAA

>M.06.0.8_A_46

GGCTTAGTTCACTCTCTTTCATAAGTATATATCTCTGCC

>M.06.0.8_A_47

ATCACATTTGTAAATTTTATCCCCATTGTAATCAAAACTCT

>M.06.0.8_A_48

TTACGTGTGTTACATCGATTGTTACATCGAATGTCATAG

>M.06.0.8_A_49

TTTAGAGAAGACGTCTTAAGAGCCGAAATTAAAGAGGCAA

>M.06.0.8_A_50

TTTACCTTAGCCTGTTCAGGCACGTTCTCTTCCTTAAGTA

>M.06.0.8_A_51

TATATCTTGTCAATCGGATATCCTTTAATATATATATTT

>M.06.0.8_A_52

TATAGGGTTCTTTTTTTGCTATCGTTACTGTGATGTAAGTT

>M.06.0.8_A_53

AATCCATATGGGGTTCCGCCCATGTACGGAGAATATGGA

>M.06.0.8_A_54

CTCAAGAAGACTTACATTACCGAAAGCATTATAGACCAT

>M.06.0.8_A_55

AGGAGGAGCGAAAGCAGGAGGAAAAAGAAAGAGAAGAGA

>M.06.0.8_A_56

TAGATTAATACTACCTACTGGTACAGTATCTATATGCGCAAT

>M.06.0.8_A_57

GTATACCTGTTCACGAGGGAGATATTGCTGAACAGTGCCT

>M.06.0.8_A_58

TTATCTATTGCAAACTATAATATACGTGATGTATGGAGAAT

>M.06.0.8_A_59

CAGCCGTGCCGTTCGCTATAAATAGCCCAGAGCCGTT

>M.06.0.8_A_60

TCTCTGTTAATTCTCTTATTTTAGTTATTATCCCTTTCAG

>M.06.0.8_A_61

ACTGAGTTTCCGCCTAAAATTACTGATGGTGCACCCGCACC

>M.06.0.8_A_62

CTAAAAATCATTGAACTACACATTTGATCGTCTTCAT

>M.06.0.8_A_63

AGTGTCCAACGTTATTAGTCTTTCAGCCATTTCCCTACCT

>M.06.0.8_A_64

TTTCCGCCAAATAGACTCAAACCTAGCGGAATGCCTATT

>M.06.0.8_A_65

GAAAGCATAAAAAAGCTTATACTAGATAACATAAATGACT

>M.06.0.8_A_66

AATATGAAGATGTAGAAGATGTACCAAGGTATTAAACCTA

>M.06.0.8_A_67

CTTTGCTGAATTGAAAGTTATTTTTATCTTTTTACCATTA

>M.06.0.8_A_68

TTTACATATACCGTAATATTAACTGAAAATTCCGCTGGT

>M.06.0.8_A_69

GCCACTCGCAGCTCCCGTTACAAAAAGATCTATCAGTTTAT

>M.06.0.8_A_70

TTCGTTTGTATGTTTTGTGGAATGTTGATCCTATTCCCTTT

>M.06.0.8_A_71

TAGCTATGGTTCTTGCAATTGTTATAGCACTCGTCAGG

>M.06.0.8_A_72

CAAGCATATTACTTCTAAAACTATCTAATCTTCTTATAGCA

>M.06.0.8_A_73

AAAATGTCCATATCTGCACACATATCTAACACTATAT

>M.06.0.8_A_74

TTAATTCTGAGGTTTCTGAAAATCATTTCTTCTGCGGCGT

>M.06.0.8_A_75

AATAGAAACACTGCTTAAGCTGTTAGATGAACTAGACAA

>M.06.0.8_A_76

TACTCTTATTCTAATTCTTCTTCATCATCATATACTAATTTC

>M.06.0.8_A_77

GCACTCAGAAAGCAGTCAAACATATCAAAATAATTAAGC

>M.06.0.8_A_78

TATTTCTCATGAAAAATGACCTAATACGGCTTATGAA

>M.06.0.8_A_79

TTGGTTCTAGTCTTCTGTTATTGTAGTAAAAATAATCTTC

>M.06.0.8_A_80

CGGAAAATTACCATCTATATTATCATTTATTGCGACAACT

>M.06.0.8_A_81

TAGTGATTTTTAATGAGTCAAACACTTGTAACTTTCAA

>M.06.0.8_A_82

CTTTTTCAATTCATTTTCTATTCTTTCCTTATAGTACTT

>M.06.0.8_A_83

GTAGGTAGATAATCTACTCTGAGAGGAACGCTAGATTGA

>M.06.0.8_A_84

CATTAGGCCATATAAGTTGAAATTCGCTTATTTGATGCCCC

>M.06.0.8_A_85

TTAGATAAAGATATAGCGTACTTCCTGATCCCGTGATTGAC

>M.06.0.8_A_86

AAAAAGAGTTACTAACACAGCTGGCATTGTTGGAGGCCA

>M.06.0.8_A_87

CCAGCAGCAGCAAACCCTAGCGTGAATGTCAATGGA

>M.06.0.8_A_88

TCGAACCCTTCAACAATATATTTCTTTTCCACTCCTA

>M.06.0.8_A_89

AAAATTTCTCCTACCATATATTACTTTCACTCATCTCTC

>M.06.0.8_A_90

TATTTACCTGCACTCCGCTTGATCCTAGATTTAAGTTAAA

>M.06.0.8_A_91

ATCTAACTATGATAAACTGGATGTAGAAATATTATTTAGA

>M.06.0.8_A_92

AAGTCAGTATTTACTAATGCGTTTAAAATTTGTTGTAACGAT

>M.06.0.8_A_93

TTCATAATTGTAAAAAGTGCTAAATTTACGTATCTCGCTTA

>M.06.0.8_A_94

TTCTGGTATGGTTATGTCATATATTGATCCTTCGAAA

>M.06.0.8_A_95

AGCGTGAATAGTGCTATTATCATAGAGATGATATTAA

>M.06.0.8_A_96

AGAACATCTCGAAAATATAACAGATCTCAACATAACTGA

>M.06.0.8_A_97

TATAAATATTTCTCTCTCACTAACGTGCTTTCGGATTTAA

>M.06.0.8_A_98

TTCGATTTCGCAAACATAGTGAGCACTTTCGATGCGAAAG

>M.06.0.8_A_99

AATTTACTATTTTCAGTAATCACATCTTGTACTACTAATT

>M.06.0.8_A_100

GTTTAAGGATGAGAACGACGTTGTTCACAGAGTAGAAG

>M.06.0.8_A_101

TGCGATAATAACGTAGATGTGGTCTATAAAGAGCACGGCT

>M.06.0.8_A_102

ATTTAGTTATGATTCCGTCAATTCTATCCTTACTTATTTT

>M.06.0.8_A_103

GTAGCATCTTTCCAGTATACTACCTCTGCATCAACTACA

>M.06.0.8_A_104

ACTTTAGCTCCATAAAATATATATAATAAACTTCCTTCT

>M.06.0.8_A_105

TCAATAATTCATATAGATTTAAAGCATTTTTTATCTCT

>M.06.0.8_A_106

AAGTAGGTACGTATAAGTCCGCATTCACACGTTTAACCTT

>M.06.0.8_A_107

TAAATTCCGCCAACAATAGATATTAATTCTAATAATTT

>M.06.0.8_A_108

TTGAACTTAATGAAACATAGTAATGTACGTGTGAATATAA

>M.06.0.8_A_109

CTATATCTTTATGATGAAGAGGAGGAGGAATGAGGTGACT

>M.06.0.8_A_110

TTTGCTTTGATAAAAATCTCTTTTGTTTCGAGATCTTCA

>M.06.0.8_A_111

GAATATTCATCGACATATAAAATAACTTCCTTATTTCTGT

>M.06.0.8_A_112

CATAAGCCCTCTTTCACTTCCGTATTTCGTTGTCGCC

>M.06.0.8_A_113

AGTATACCTGAAGAACTTTCCTTGGATCATAATAGTGGA

>M.06.0.8_A_114

AAAGTAAAGCTTATTCCTAATCCTGATCCATACGTTACGCT

>M.06.0.8_A_115

ATTTTATAATAGTTATATATTATGCTTTTTATCTGGTTTT

>M.06.0.8_A_116

TTCAACTCTATTGCATTTCTTCCTGTGATACTTCCAACT

>M.06.0.8_A_117

GAAAATAGAATCTCGTTCATAATAACATGGGAATATGGA

>M.06.0.8_A_118

ACAGCCACAGCGAGGACAGCCGAACCCTTGTTCTCCTGT

>M.06.0.8_A_119

GTGTATACAATCTTTGAATAAGATAAGTGTAAACCCAGAT

>M.06.0.8_A_120

AGATAAAAAAGCTGAGGTACGTATGCCCACACCCTGGCTT

>M.06.0.8_A_121

GTATTATTAGCGTTGTTAAAACATTTTCTTGAAAAAAAGC

>M.06.0.8_A_122

GAAGTTTGGCTAGGAGTTACGGTTACATTTATTGAAGGT

>M.06.0.8_A_123

AGTGTATCTATAGTACGCAATTACTTTATCTACTTTAA

>M.06.0.8_A_124

TAATAGAATTTGTCCATTAACCGCAGTAACATTGATAACA

>M.06.0.8_A_125

ATTACACTATTTATGTATACGTCTATTACTACTTTTTGGT

>M.06.0.8_A_126

TCATAAATTGCTTAATTAGAAAGTCTTCAGTCACAAGA

>M.06.0.8_A_127

GATGACGATTTGGCCGATGAGTTGTGTATTAAATTGA

>M.06.0.8_A_128

CCTTTCTGGAAACTTTAACCTACTCATGGGCGGATCACCTCG

>M.06.0.8_A_129

TTCACTAGGAAGTTGGGGACTGTGTTTGTTGAGAGAGAT

>M.06.0.8_A_130

TACAGTCGCAACTCCGATAACTGCGTATCCACTATCGC

>M.06.0.8_A_131

ATAATTTCACCGTCCTCTAGTGCCTTCATAACGTCCTCA

>M.06.0.8_A_132

TTTCTTTTCATATAATCCCCCTAGAATCTAATTTTTTCGA

>M.06.0.8_A_133

ATAAAACCAGTTCGTGATGATGTGGCACTTTCTACAATATAGTA

>M.06.0.8_A_134

ATATGATAATACTAATAGTTTCTTGTCTTAATTTTGAAC

>M.06.0.8_A_135

CAATGTCAACGGAGATATACTGAAGGAAGCTAGTCATGA

>M.06.0.8_A_136

ATTATAGCTATGCCTATTTCAAAATTTTTTGCATCAA

>M.06.0.8_A_137

TTTATCATCGCCCATCTCTACCCCCTCACTCGTTTACATACTTTT

>M.06.0.8_A_138

CAACTAAATAAATAGAAATTTCACTAACCAACACATAACT

>M.06.0.8_A_139

TAAATTATGCCTAAAAGCCCAAAAAGTTCTTGGAAAAGT

>M.06.0.8_A_140

CTATCACCAATTCAAGTAAGTTTTGCTCAGACAAATGTAACT

>M.06.0.8_A_141

ACGTCAGTCTATGAACCGACGAACATTAACGAAACTAGCG

>M.06.0.8_A_142

CCCGAAGTTGTACTTAACGTTTCTTCCACTACAACATCAA

>M.06.0.8_A_143

ATAATACTTTTTTATTTTGACGCTGTTATCACTTTTTGA

>M.06.0.8_A_144

TTTACTGTGATAGAAACAATACCATTATTGTCATAACCTGC

>M.06.0.8_A_145

CCACTATCAACTTTGGCTTGCCTTCAACAGTAATACTTCT

>M.06.0.8_A_146

ATTTTTATATGATTTTCTTCTTCTATACCCCCGTCTAA

>M.06.0.8_A_147

AATTCACTGGAAAATGGCTTTTCACAAGCACTTGAAGCGT

>M.06.0.8_A_148

ACGCAACCCCCAAATTTCATAGTATATGAATATCTAAA

>M.06.0.8_A_149

CAACTGATGGCACGCGGTAAACGGGGATATTTACTTTCT

>M.06.0.8_A_150

AACTGAGAAGCGTAAGTCTGAGCTTGCTGTAAGTACT

>M.06.0.8_A_151

AGGAAAACAGCTACTCTCTCAATTTCTTTATGCATTCCTCAC

>M.06.0.8_A_152

TTAGGATCGGTTGCAATAAGAAGATTGGTAAGCAGTGGT

>M.06.0.8_A_153

AAACATTCTTGTAAACATTCATTTCTATTTTTCTTATT

>M.06.0.8_A_154

CTATGGTCTAACGGTGTATATAGTTCTGTTTCCTCATCAT

>M.06.0.8_A_155

GAATTTTGACCAAACGTGCCTGATGTTAATAATGGTGG

>M.06.0.8_A_156

TTATATCGTTAGGTAATTTCACATATTCCTCCGTAGTAAG

>M.06.0.8_A_157

CCTAATAACGACTCGCAATTGATACAAGAACATATATCATC

>M.06.0.8_A_158

TCACTTCTTAGCAACTGCAATGACTATCACAAGTAAGAA

>M.06.0.8_A_159

GTAGTGCTGTTAGGGGCCACTGTATTAGGCACTAAGATAAT

>M.06.0.8_A_160

TTCTGTCCCTATTTCCGTCTCTATCTCTTCTATGTCCGT

>M.06.0.8_A_161

CAAAATCAATCTTGGGGAATACAATATAATTCAACACT

>M.06.0.8_A_162

TCTTGTAGCGTGGTATGCAGCAGCTAGCCCGCTTTGTAT

>M.06.0.8_A_163

AATAACCATGAGGGAGTTGCACCATTAACTATGTAAAGTCG

>M.06.0.8_A_164

TTAAATACACAAGGATTATAATTTTCATTAGCATATTCA

>M.06.0.8_A_165

GCACTTTAAGAATTATGTAATAATTGATTCGCATATACTT

>M.06.0.8_A_166

TAAAGATAATGGTCTTCAAGCAAATGCGATATTATACGT

>M.06.0.8_A_167

TAACGCTTTGCCGATTCTTCTTCAGTCCAACTTGAACCA

>M.06.0.8_A_168

CAGCACGCTGGAAGCTATGGCGTCAGCCTAAATACATTA

>M.06.0.8_A_169

TTAGACCTAGTCGTTGGAGGATACCAGGAAGAAAGGGATGGAGA

>M.06.0.8_A_170

TATTCTTTGACCTGCTGAATAATAATTTATTAATTAATT

>M.06.0.8_A_171

TACATCTTTCTTGCTCTCTTCTTTATAGTTTTGAAAA

>M.06.0.8_A_172

GATGTTGTAGCGTTTTTCTCGCGGAAAAGCTTTTATAGT

>M.06.0.8_A_173

GCTAGATGGGAAAACAGTGACAGCAGAAAGAATATGCATC

>M.06.0.8_A_174

TTCTACAATTGCCCATGACTTTGTACCTTTTGGTGCATAC

>M.06.0.8_A_175

ACGCCCGCACCTACTATGGCCCCAGCCCCAGCACTAGCTG

>M.06.0.8_A_176

CTCTGGGTCATTTCGCTTTACGATAAAAAAACGAGTG

>M.06.0.8_A_177

TTTTCCTCGAACGCTAGGTAACTGAGTGCTTTCTTCGTCAA

>M.06.0.8_A_178

TTAAGATCTTTTTTCAGAATTTTCAACGGATGTTGAGAAG

>M.06.0.8_A_179

TCTTTCCATCTGACAATGTACCAGGGTTCTGATACTCCAT

>M.06.0.8_A_180

ATTTAGATGGACAGACACCAGTTTAAGCACTTGATACACGT

>M.06.0.8_A_181

TCTGCAGTTAGCTCCGGAAGTTCTCCTTCTTCTTCTACTTGCG

>M.06.0.8_A_182

CTGATAACACCGGGATTTAGATATGGACGGCAAAGTGCAT

>M.06.0.8_A_183

GTGACACTCTCGCTCGCTTCTGCTCCGCTAATCTCT

>M.06.0.8_A_184

AAGTTGGTGCTAAGCTGAATATCTGTAATTGTTCCTGTT

>M.06.0.8_A_185

TCCATTTTTTTGCTTTTTTCCCGCGAGAAATGATATC

>M.06.0.8_A_186

TTGAGTTATTGCATAATATCCTTGTCCGCTCGCGTGTGT

>M.06.0.8_A_187

GTACCATAAGCAACTGCTTGTCTATTATTCTTTAGCTT

>M.06.0.8_A_188

AGTATACATTGTTTGCCATCTCATCATATCTATAGAGTCTTA

>M.06.0.8_A_189

TGAAGAAGAGATTAAGCAAAAACTCGCTGAGGTGATGG

>M.06.0.8_A_190

CTTGTGTGTGTCATTTGCAATTCCTCATAATAAACA

>M.06.0.8_A_191

TGACTTAAATCATACCAACTCCATGTTGCGTCTACGTTTT

>M.06.0.8_A_192

TTCTTGTAAAAATGACCTTGCCCTCCTTCTTCACTTGT

>M.06.0.8_A_193

ATTGAGATCAATGAGGAAGTTGTGGCGTTAGCGTATGG

>M.06.0.8_A_194

CTGGAGTGAATAGAATACCAGTTATCGCTGGAAGCACAA

>M.06.0.8_A_195

ATATAAATCCGAAAGCACGTTAGTGAGAGAGAAAGGTGA

>M.06.0.8_A_196

TTAAAAGTTCTTTGGCCGTAGTGGTGTCTAACCCGTT

>M.06.0.8_A_197

ATACGGTTATAGACGAACGCTTGCCACTGGACGCTACCAGT

>M.06.0.8_A_198

TTTCCTCCGTTTATTGCTATTGCACTGCTGCTAGAATTCA

>M.06.0.8_A_199

GTATCGATATGAATTAACTTGAATGATTTGTTTAGCGCTTT

>M.06.0.8_A_200

GGATTAGTACTACCAGTTTGTTTCGGAAACGATGTTACA

>M.06.0.8_A_201

GTATCAGTAGAGCATATATTAGCTAACATTTCCACATTAT

>M.06.0.8_A_202

TTTTTTCTTTCTTCTCCTTTTTCTCTGTCTTTTCTTCAG

>M.06.0.8_A_203

TTCACACTTTTCTTATCCTTTTTTAGAGTCTTTAAAATG

>M.06.0.8_A_204

TTAATTTTCTTATACCATATCCTAAAGGAAATAGCCAATT

>M.06.0.8_A_205

TTCGAGTACAAAGGATACTACGATACAGAACTTTATCAC

>M.06.0.8_A_206

AATTCTCTTAATTCTACTATGACCTCATATTTTTTCTTCAT

>M.06.0.8_A_207

TTAAAATCCACAAATAATATATAATCGCAACAAAAACCCT

>M.06.0.8_A_208

TTGAAACTTCCTATCGGATTAGTAACACTAGTGCTAGG

>M.06.0.8_A_209

TTTTCTATAAGTCCACCCCAAGGAGTTACTAGAACAAT

>M.06.0.8_A_210

TTATTGTCATAACCTGCCACTGTACTCGTGTTTGATGT

>M.06.0.8_A_211

AATTCTTCTAAATAATTCCAATAACTTCAGATTCTTTTGTGAT

>M.06.0.8_A_212

TTTTGACGACCGTGTCTATGAATTCAACGGAATCCTCAT

>M.06.0.8_A_213

TATTTAGGTGGCAAATTGTTGGCGAAAACAAATGTTACAA

>M.06.0.8_A_214

GTTACGTGCATTGTATTTAGCAAAGAGGCTTAAGGAAATG

>M.06.0.8_A_215

AGCGCTCCGTTCCCCTACAATTTAGTACAGACCTTTAACCTTA

>M.06.0.8_A_216

TCATCATTTCTAAGTAAGTTTCGATAATCTTACTTTTTA

>M.06.0.8_A_217

TAATGAAAACATCTCTGTCAACTCTAATTAAATTCAGAT

>M.06.0.8_A_218

TAAAGCTTGTGACCCGCCTACAAGCTTAGCTGCATACG

>M.06.0.8_A_219

CCGTCCGCAATGTGGGCATTGATAAAGTAACGCTCCA

>M.06.2.4_A_1

CATTCCACCAACATCGTCCATTGTTATCGATGGTGT

>M.06.2.4_A_2

TCATCTAATGAAATTATATAAAAATTCTGCAAAAATGAAT

>M.06.2.4_A_3

GTACCCCTTTAGCACTAGATATAAATACGATAGAATTTCCG

>M.06.2.4_A_4

CATAAAGTATATGTGCTGAAGTCTTCTTTGATCCGATA

>M.06.2.4_A_5

ATCATACTTCATTCTTCCTCATCCTCCTCTTTACATTTA

>M.06.2.4_A_6

CTACTTACCAATTTCCTTATCGCAACCGATCCTAATGGAA

>M.06.2.4_A_7

TTGGCTGGAACCAGATATTGCTGAGGTAAGCTAGTTCC

>M.06.2.4_A_8

TAATTAATGGTCTTAATTATGACGTAAAAATAGGCCCAG

>M.06.2.4_A_9

GTAATGATGAACTTTACAAAATTCCTCATTGTATTAGCC

>M.06.2.4_A_10

TAAGCTAAAGAATGAGTAGCTAATACTATAAATTGCTTAT

>M.06.2.4_A_11

TGATATGAGGAGAAGATAGGAGAGTATATAGAGTATG

>M.06.2.4_A_12

TTCAATATACCTTTACCTTTAGATCTTCAGAAAATAGTA

>M.06.2.4_A_13

CAAACTCAGCGATAAGCAAAAGTTTAAAACAGCTGTTT

>M.06.2.4_A_14

TTGCATAATCTTTTCTGAATCCTAATGCCATCTTTTCCTT

>M.06.2.4_A_15

ACATCTTTGTGGCCGTGAACTTCCTCAGATATTTCGGGGCTA

>M.06.2.4_A_16

ATTCTCAAGGAAACCAAAAGTCTTACCTGCGGAAACTAT

>M.06.2.4_A_17

TTTCAAATCATTGTAGTCCCAAATAAAGGGTATTTTTG

>M.06.2.4_A_18

ACGCACTGGAAATATTCCGAACAGTTGTGCGGAATCGACC

>M.06.2.4_A_19

GAATTGTTTCGCGACAAGGACATATGCTAACCTATCTAT

>M.06.2.4_A_20

CATAGATTCTCATAGGCAATATAGCTACTTTGTCATCAT

>M.06.2.4_A_21

TATATGAATCTGATTGCATGTTCTGACATGTCGGCCAC

>M.06.2.4_A_22

AGAATTGATCCCATGAAAATTCCAGCTAACATATATATTA

>M.06.2.4_A_23

ATACCCCTACACTGGTATATATTCAGCCGTATATAG

>M.06.2.4_A_24

TCAAATGTACCCCGCAATATCCTCTTTCCTACATTCACTTC

>M.06.2.4_A_25

ATATACTCGAATCCTACATCAAATCGATTTAAGTTTTCT

>M.06.2.4_A_26

TATTGTAGGTCATGAAGCGACATGTTACGGAATTTCTAAAG

>M.06.2.4_A_27

AGCAGTTAGAGATATTTCAGCTGAAGGAAACGCTGAGAAAGATAT

>M.06.2.4_A_28

TTAGTTCAGTAAACCCCGCGGGGGGTTCCCCCACCCCCA

>M.06.2.4_A_29

TCAACCCTACTGACCATTGATAGCCGTAGTCATATGATGT

>M.06.2.4_A_30

AGGACTTTAGTCACGCTTTCTCTTATTTTATAACGTAAACTCTC

>M.06.2.4_A_31

ATACCAAGGCTGAATGGAGGCCTAGAGTAAACCCCCCA

>M.06.2.4_A_32

ACAAATCCGTATCGGATCTAACACATCAGATCGTTCCAT

>M.06.2.4_A_33

AACGATGCTACCGCATTTGTTAATAAATTTCCTAAGTTTTGT

>M.06.2.4_A_34

AGCTGGAGGAAGTGAAAAATGGACAACGACAGTAAGCTA

>M.06.2.4_A_35

AGATTTGTCATTTGAATTATATAACTATGAGTAAATGTTA

>M.06.2.4_A_36

TCTGATCTGTTATCAGGTCTAATGAGTATTTGGCCTTA

>M.06.2.4_A_37

TATCTCTTATCTTATGTCTTCCAAACGCCAGAAATCTT

>M.06.2.4_A_38

GTAGACGCTACTGCATTAGGCATAGGTGTTATAAACGT

>M.06.2.4_A_39

CTCAAGGCAATTATAAATGAGGTGTCGACCAAGGTACCT

>M.06.2.4_A_40

AAAACCGGGATACGAACGTGTTCTCTACAGAGGTAATGTA

>M.06.2.4_A_41

TTTGAAAGTATGTATCAGTTCGAGATCATAGAGAAAGCTGT

>M.06.2.4_A_42

CAGCACGCTGGAAGCTAGGGCGTCAGCCTAAATACA

>M.06.2.4_A_43

ATCAGTCGTGACTATTGTACCTCCTTGGTAGTAGTTTA

>M.06.2.4_A_44

TCGGTTATATCTTCAGTAGGGGGTTTTTACTAAACTTA

>M.06.2.4_A_45

GCACATTCAGCAATCACTTGAAACGCTTTAGTATTTAT

>M.06.2.4_A_46

TACTAGAAATCCCGCCACTATTCCTATGAAATTAGTTAGCAA

>M.06.2.4_A_47

AATTGCAAACTTATTGGTACTTAAAGCCCTTCATAAACAT

>M.06.2.4_A_48

GAAAAATAAAAAAGCTTTTTAGAAATATACTTCTAAATA

>M.06.2.4_A_49

AGTGAAATATACTTTGCTATTTCTCCGAATGCAATAAA

>M.06.2.4_A_50

AATGCAGCAAAAGAACTCATGTAATAAACAGTAGAACT

>M.06.2.4_A_51

TTTACAGTCCTTTCTTCCACCTTATTTAACCATTTTCCT

>M.06.2.4_A_52

AGAGCAGTTAGACCAATTGTACCACTCCATTCAAAAAAT

>M.06.2.4_A_53

TGTTTTAATTAATGAATCTACCCATTTGATTATCATCATAA

>M.06.2.4_A_54

ATAAACTATCAACTTGTACAATCCATATATTTTAAGGTAGC

>M.06.2.4_A_55

AAAAACTTTGCTGATGAGGAAATAACAGATATTAAACTAT

>M.06.2.4_A_56

TTATAGTTAACATAAGGTACTATACCGCTACTCGCTACA

>M.06.2.4_A_57

TCCGTAGTAGTCAATAGCAATGTTACTGTTAATTACTTGTA

>M.06.2.4_A_58

TCTAATAATACTAATAATAATCAACAAACTCAGACTACAT

>M.06.2.4_A_59

TGTACCGTTACCTTGATTTGTTAATTGTATCGACTGTCCAAA

>M.06.2.4_A_60

ATTAGTTTTATTAAATGATCCATCAATTCAAACTTTGACATGT

>M.06.2.4_A_61

AAGCATATAGCTCCAGAGAAATACACATTGAAAATTGATTT

>M.06.2.4_A_62

CTGTATCTCCCCAATACGTCAATACACTTCCAGAAGACGT

>M.06.2.4_A_63

ACAATCCGCAATTAATTCTTTACATTCCTCCAAATATTTT

>M.06.2.4_A_64

TTTTGTTTGAACTCCTCCAGTAATTTTTCTATCTCATCTT

>M.06.2.4_A_65

GCCATGACATACGCAGTACTTGTTGTTGTAACCGCCGAA

>M.06.2.4_A_66

GAAAGCGTAAAGATGAAGTTACTAGACGAAATCAAAAA

>M.06.2.4_A_67

TTCTTGAGCTCATAAATCGGGCTGAGGTCCTCATAAT

>M.06.2.4_A_68

ACAGCTTCTCCAGCAGGGCAAATACCAGCAGGCATTAC

>M.06.2.4_A_69

ATTAAGTTCATCTAGATGATATATAGTTTGTGCTTTT

>M.06.2.4_A_70

CGATACTCGAACGGTTGCGTACCCCAAGGACGTAATTCTA

>M.06.2.4_A_71

CCCATGATTTTCAATTCACCAATTGTACAAAAATATATTCTT

>M.06.2.4_A_72

TATTGCTGATGAATTAAACGTCATTATACTATTTTTGCCG

>M.06.2.4_A_73

TTTACTGACATATACCTATATGATCCTTTAGAAGTGAT

>M.06.2.4_A_74

ATTTTTTGGTTTGAAAAAACACTTTTGTTACCTTTAACA

>M.06.2.4_A_75

AAAGTATAACTAAGTTTTATCCACTTTACTTTCTCTTCTCCC

>M.06.2.4_A_76

TGGAAGTGTAACCGTCTGCCCACTTATACCTGTATAGTCA

>M.06.2.4_A_77

AACGGTGAAAGTGCTACTTTATCATACGGGCAAGCATTAGA

>M.06.2.4_A_78

ATTTCCGTCTCTATCTCTTCTATATTCGTAGTCTCA

>M.06.2.4_A_79

ATTAACTGGTGGTTGGAAGAAAAACTATCGGCACTTACA

>M.06.2.4_A_80

GTTTCCATAAGATATCGCAGAATAAATGGCGGTAATGGTCT

>M.06.2.4_A_81

ATTGTAGGTTTAAGTGTCCTCTTGAACTGCTGGCTTTCT

>M.06.2.4_A_82

GTAACAGGGACTTTTGGACATTTAGCAGCGCATCCGAA

>M.06.2.4_A_83

AGGTGTATAGGTGACATGTGATATCTGTTCCTCCTCCTGAT

>M.06.2.4_A_84

TACTTCCATTATCATATTGAGCATAATTGGATGTCAA

>M.06.2.4_A_85

TTTCATGTTTTTGTTATCATTCAATCCTTGGCAATTGAGTGT

>M.06.2.4_A_86

GTCTAGTTTTTTGTGCCCCGTTATTTTTGCTATGAT

>M.06.2.4_A_87

TTTTTTACATTTTTTATAGTCCATCTTAACTTAAGTTCTGT

>M.06.2.4_A_88

AATCAGGGGATGATACTAGTTTTCACATACAACGGACAGTCGGT

>M.06.2.4_A_89

GCTTTCTGCCGAACTTTTCAGCAGTCATTATAGCTCCGT

>M.06.2.4_A_90

AAAGATCAATTAAAGGAGTTCTTTGAGTGGATAGAGAAAG

>M.06.2.4_A_91

GGTTGTAAATACGCCACGTGAATTGGAGCGCTAGACGCGGG

>M.06.2.4_A_92

TGGAAACCGCTCACGTAAACACTTATAAATCCATTCTCTTGT

>M.06.2.4_A_93

TGTGGCAACACAAATATAGCTTCACAGATAACATCCCTTTGT

>M.06.2.4_A_94

ACAGATGTAATAGATGTTTCATGGCAATCCCTCTTAGCAG

>M.06.2.4_A_95

GAAGGCAGTGCAATCTTATATGTGTTCCCTTTTTCTTTTGGT

>M.06.2.4_A_96

TTTATTATTGAGTTGTTTCAAGATGTTGGTATCATAG

>M.06.2.4_A_97

TTTATATCACCTCCTAGCATATAATGATACTAAATAA

>M.06.2.4_A_98

ATCTTTGAAAGATAATTAATAAGTAGCTCTAGATCCTTT

>M.06.2.4_A_99

TTCAATGTTAAAACTGGAATAAATGGTGTTGAATATCCT

>M.06.2.4_A_100

ATACCTTTTTAACGTCGGATACCATAATCATTAATCCCGC

>M.06.2.4_A_101

TACGTAACGCGTATAGTGACGTCTTCATAGTCGCCCAT

>M.06.2.4_A_102

TTAGTAGTTAGACAGGCAGAATCGGGTTAAGGGCGTTAGC

>M.06.2.4_A_103

AATGATTGAACTACTAAAGTCAGTGGCGGAACTGCAGGCT

>M.06.2.4_A_104

CATGCATTTTATAAACCATAGCAACTGCATTTATTAACAT

>M.06.2.4_A_105

GTAATACTCAAAGTGTTTATATAACACGTGAGACGCAA

>M.06.2.4_A_106

AATTTCTCATAATGAAGTCATTTCTATCTATAATATTCTCTC

>M.06.2.4_A_107

TAATAGAATATTTCAATATTGCACTCAGTGCGAATACGC

>M.06.2.4_A_108

AGGTTCTGTAGTTCATAATGAAGAAGCTTATAATATG

>M.06.2.4_A_109

AAGTCACAACAAATCTAAAAATTCTAGTTGCCATTAGCC

>M.06.2.4_A_110

AGTGTTAGTATAAATCGCGAACAAAATAAGCGGATAAAGAGT

>M.06.2.4_A_111

ATCCTTAAAGTTAGTCTGTATCAATAGATTGTTAATATCTT

>M.06.2.4_A_112

AAAACATCGTCATACACAGTATTAGTCCAACCTAATGTA

>M.06.2.4_A_113

TAATAATCTGTAACGCTTACAGTTATCTTAACTGGA

>M.06.2.4_A_114

TTTTAAACAAAATAAACTGTCCATCCTTCCTCACATTTCTT

>M.06.2.4_A_115

TTGTATTTATGTCCTCCCCACTCATATAGTATATATTCTCC

>M.06.2.4_A_116

AAGATAACACATTTATTTTCTACGTAGTACTTACATACT

>M.06.2.4_A_117

CCTTCTCAGGTGCATTTTTCTCTTTCCGCAGTCTCTCTAT

>M.06.2.4_A_118

TTCTGTTTATGCAGCGCAGTAACGAAGTTCACCATTCTG

>M.06.2.4_A_119

CTCTTTTGTTAAACATGTTTTTCCTAAGCGTATTATAAT

>M.06.2.4_A_120

AAATTACTAACCTTACTATCAATTTTTTTAAGTAAATCTT

>M.06.2.4_A_121

AAGTAGATCGTCTTGTCTGGTTCGGTCGTCATGTTCAAC

>M.06.2.4_A_122

TACATGGGCTACAACCCATACGCTTACGGATTAGGACAAT

>M.06.2.4_A_123

TGATTATTAATATTCTGTCCTCCTCTTTGTCATCGGCCCT

>M.06.2.4_A_124

TCTTTCACAACGTGGACATTGATATAATAACGCTCCA

>M.06.2.4_A_125

AAAGCTTCTTGAAAAGTTAAGCCATGCGTAATTTTTAAT

>M.06.2.4_A_126

TGACGGAGGGAGGTGGATAATCACTGCCGTCCTATACG

>M.06.2.4_A_127

CGATGATTTCCCCTACGTTCATGGTAAACATGTTCAAGC

>M.06.2.4_A_128

GTTACGGGTACTGTTTGTGTATAATATGAGGGGTAACTCATGT

>M.06.2.4_A_129

TTTTACTCACCTAGTTTGTACAAAATCGAATCCTGA

>M.06.2.4_A_130

AAAAGTTCTTGGAAAAGTAACGTAAACAGATATATTCCGT

>M.06.2.4_A_131

TTGGTAACCTCATCACTCACCGTTGTTAAAAATACGT

>M.06.2.4_A_132

GTTGGTTTTCATCAAGACTAAGTTTAAATAAATCTAT

>M.06.2.4_A_133

ATCATAGTTTTTGCAACTTCTAAGTAATTTATGTTCTCTCTG

>M.06.2.4_A_134

ATGTTTGGTAAGTTTAGCAAGGCAAGTTTGGTTCAGCAAG

>M.06.2.4_A_135

CCTACCGTGACTGCGACATAATGGGGTATTATTGCATAAT

>M.06.2.4_A_136

CCACATCAGCCTTTCAAGCCTTGACTTAGGCTGGTCTAC

>M.06.2.4_A_137

TTAACACTTAAACTAGAATTATCATCATCAATAGATAA

>M.06.2.4_A_138

ATTATATTATCAAGACCTTTAGGTGTTGTGTATGAGATA

>M.06.2.4_A_139

ATTGACTACATTCAAGCCACCAACGGTAACGATAAATTTACGT

>M.06.2.4_A_140

TCGTAGAAGTGTTAGTTATATGAGCTGGGCTAACGC

>M.06.2.4_A_141

CTATATCTCCCTAAAACATCGATACATTTCCAGAAAAC

>M.06.2.4_A_142

CTTATTCCCGAATGATTTCTTCCTCCTATAGATTTCCTCAA

>M.06.2.4_A_143

GACTGGGGAGGATTATCAAGTATGAGGATGAAAGTCTTAT

>M.06.2.4_A_144

CTAATAGGCAATGTTTCGGGAAGAAGATTCGCACATATAG

>M.06.2.4_A_145

TCATCTATAGCTTTTTGTATTAGACTTTTAATTTCCTCCT

>M.06.2.4_A_146

ATAGTCTGTCTCACCTTGATTATTGCTTTCTCCTTCAT

>M.06.2.4_A_147

AAGACTTAGATCATGGTGTGATACATCAATATTTAGGA

>M.06.2.4_A_148

GCAGTTAAAACGATACCAGTCCCTTCGCCCGGAATTGGA

>M.06.2.4_A_149

TTAAATGATCCAATAGGATTTGTAATTGTTGTAGAACCAT

>M.06.2.4_A_150

CTCTATTTCTTCTTCGAATTTCCTTAGTGTGTAATCTTCA

>M.06.2.4_A_151

GTTTACATTATTGCTTTTACACTTCCTAGTCAATTTATTT

>M.06.2.4_A_152

AAGAAGCAATACTGATTACGGTAGGAGTGCTATTCGTTATATT

>M.06.2.4_A_153

ACGAGAGTGTTAGGATCTTCCCATCCTACCCCTAACGGG

>M.06.2.4_A_154

CGCCGTCGCCGACGCGGGGGTATGTAGTCAGTATTACT

>M.12.04_A_1

TAAAGATAAGGGAAGATATTTTTTTGAAAGGTAAACTT

>M.12.04_A_2

GTTTATCGCTCTGTTTTCTAGTCATTTCTCTAAAACTTCT

>M.12.04_A_3

AAGTCCTTTCTTCCCTTATTATTATTAATCCATTCTCTT

>M.12.04_A_4

ATGCATAAACTCACTGAATAAATTAGGCGTAAATCCTGA

>M.12.04_A_5

TTATGGTCATAACATAACGCACTGTATATCTCATCGTTATCC

>M.12.04_A_6

AGTACTTGACTAACCTTTTTTGCCTTGCATTGAATTTTTTG

>M.12.04_A_7

CTGGTTGTTTCAATCTGAATGTAACTACTTTGTTCCCCT

>M.12.04_A_8

CTGGAACTTTAACAGTTGTCCCGTTAGGCAATATATAGC

>M.12.04_A_9

CAAACACAGCGATAGCAGGTGATAATAATACGACTACTG

>M.12.04_A_10

TGTATATAGTTTGGTGTATAGTTATTACCACTGTTGTCC

>M.12.04_A_11

AAGAAGTGGTACGTGACGTGGGATTGAAAATGTACAAGTGT

>M.12.04_A_12

TCTACCGGAAATAATTCACTGCTTCTCTCTATCTATAAG

>M.12.04_A_13

CTACAATTAAAATCTCTTTTCCATTATATTTATCTATAA

>M.12.04_A_14

ACTTCTCGGCGAAGACAGCAGCAGATTCTTCGACTTTTAT

>M.12.04_A_15

CAGGGTGTTGCTGCGGCGCAGCCCAAGATGGCTGCAGTTATG

>M.12.04_A_16

ATTCGCTACTCCCGAAGCTTGGGTTCCTGTTGCACCTGCACCT

>M.12.04_A_17

TATAGCCACTGTCCCTAGAGGAATCTTAACAATTATATCCTTA

>M.12.04_A_18

AAATACTTTAGGAAGTTTGTCGCTAGTAAACTAGCGGA

>M.12.04_A_19

ACGATTATCATTGACAGTGGAAACGGTATTCATGTTTAT

>M.12.04_A_20

GATACACAAATACATGATATCGTTGACGTTTTATTAGTTTC

>M.12.04_A_21

ACATCAAAGAAAAGATATCCTAAACCTATCGCATAT

>M.12.04_A_22

CCTCCAGCTAAGACCGAAAGGACTAGGAATACCGATT

>M.12.04_A_23

GCGATTATTACACACCTTTACAACAGAGGGTATACTATAT

>M.12.04_A_24

GAACTTCACTACAATAGGCCATGCTAAATTCGGAAATATT

>M.12.04_A_25

ACTATATAAATTTGAAAACGGGAAAGAGAGAGAAAGATTTAT

>M.12.04_A_26

AACGAAATAACATTCACACAAACTTCCGCAGAAGTGC

>M.12.04_A_27

AGTTTTAAAGTCCAAGGTTTATCACTATTTAGTATGTAA

>M.12.04_A_28

GAGCTGGGCATAAGCGGATATAGGATTAACGTAAAC

>M.12.04_A_29

ACACTAGCACCAGCACCTCCATATGTCCATGCTATCTGTCC

>M.12.04_A_30

TTTTTCATGCATTCTTCTATTTTATCAGATTGCAATAA

>M.12.04_A_31

ATATACGAAGAAATATACATAACTATCTTGATACTCATGG

>M.12.04_A_32

TTGAATGCTTGCCAATTCGCAACGCCGCTAATCTGTGT

>M.12.04_A_33
[truncated: 164,190 more chars]
