# Supplementary material for: Diversified local CRISPR-Cas immunity to viruses of Sulfolobus islandicus
Source: Philos Trans R Soc Lond B Biol Sci. 2019 Mar 25;374(1772):20180093. doi: 10.1098/rstb.2018.0093 (PMC6452263; doi:10.1098/rstb.2018.0093)
Supplement: CRISPR.Spacers - List of all spacers used for this analysis from Yellowstone [file rstb20180093supp3.docx]

All CRISPR spacers from the Yellowstone *Sulfolobus islandicus* population from 2012. Names indicate the strain name, CRISPR array locus, and an identifying number.

>NL01B.C01.01_A_1

ACCTTCTTACCCATAATTATTATATTCATAAAATGTTAA

>NL01B.C01.01_A_2

AAAGAGTATTCCCTTCAACATATGCTTTTTTTCAGTTAAACTTT

>NL01B.C01.01_A_3

CATTATTCTCTCTACTCTTGCTTTCGGCACCGTATTTTT

>NL01B.C01.01_A_4

CGAAGTGACGGTGAACGTTATGACCTGGACGACGATTATG

>NL01B.C01.01_A_5

TCACGTTTTACCACCTTTTACAGCTTTCGTTACTTCT

>NL01B.C01.01_A_6

ATCCAAGCTCTCCCTCCAACACCGGCATAACAGGGCTGTAT

>NL01B.C01.01_A_7

TTTGATACCGTTATGATAGACTGAACCAGTTAGTATGCCA

>NL01B.C01.01_A_8

GCTGAATTCTCCGCCAGGATGACGCTGAAGGTAAATGGGA

>NL01B.C01.01_A_9

TTCTCATCGACTTTGCCCAAGAACTTTGAGAATTGTTTC

>NL01B.C01.01_A_10

AAACAAGGGCGAAAAGCAACAAGAAGGGCAGAAGCA

>NL01B.C01.01_A_11

TAGAGATGCAGAAAGTGAAAGTGATGATAGGAGGGATGTA

>NL01B.C01.01_A_12

TTCTTTTTATGTGAAATTTGACATAATTATCAAGCTTTAGA

>NL01B.C01.01_A_13

TTTGGTATATGACAGGAGGCATCCTCTATTAGAGATGGCG

>NL01B.C01.01_A_14

TAGGACTTCATCTTCAGCAGTATATTGTGTGCTTCCAGG

>NL01B.C01.01_A_15

GCTACTGTGGTATATAGCATGACAACTGGGTTACCATA

>NL01B.C01.01_A_16

GAGGGGAAGGTCTTGAGTACACAAGAAATCAGGGAGATGTATAA

>NL01B.C01.01_A_17

AACTTTTTTGCTAATTCCTCCAATTTGATACTTTCTTCCTTA

>NL01B.C01.01_A_18

ATCATTATCGTCACAATTATATTTCTTGTCCAACATTTTCT

>NL01B.C01.01_A_19

AATTAAAATCAGTGTCGAGATAAACCCAAAAACGGGCAGG

>NL01B.C01.01_A_20

TTTATACTGCCGTTAAATATGACGGTGCGTTTGAAGG

>NL01B.C01.01_A_21

TTTTTGGTCAGTATATTTTTATGCCAACCCGGTATTTA

>NL01B.C01.01_A_22

GATAATGAATTAAATCTTAATTTCAATCATGTAAACAGTAT

>NL01B.C01.01_A_23

TCGCCGCCCCCGTGAATTTTTGCAAACCGAGAAAAGTGAGT

>NL01B.C01.01_A_24

TATTATAATTCCCCATACGATTTTCCAGACCTTTTCCCTTCT

>NL01B.C01.01_A_25

ACTAACATTATTAAATGCTTTCTTCTGCCTTATAAACTTTTCTTTC

>NL01B.C01.01_A_26

ATCAGTTAGATTCAAGTTGTTGCTTGATCTTGATTACGATTA

>NL01B.C01.01_A_27

TGCTGTTAGGTATTGTCCTGAACACGAAGCCGATGAAGAA

>NL01B.C01.01_A_28

AGTATACCTGAAGTTAGATTGGGACAATCGGATATAAA

>NL01B.C01.01_A_29

GATATAGATGAAATATTCAAGTGATACTATGCTAAAAGATTA

>NL01B.C01.01_A_30

GCAATAATAGATAAATTGGCTGAAATTCTTCCTGAGAT

>NL01B.C01.01_A_31

TTATTATTATTTACGATTGCTAATGAGTAAAGTGGAATATTG

>NL01B.C01.01_A_32

ATAATTAAAAGCCATATTGGATCTATTGATGTATTTAAATT

>NL01B.C01.01_A_33

TTGAAAAGGTGTCATTAAACTTCATATATAGGAATGAGACGA

>NL01B.C01.01_A_34

TGCTATTTTACGCTCCAGCGCTGCTATTGTATTAAGCGCTT

>NL01B.C01.01_A_35

TGAACTTGTAAAAAAAGGCTATTCAATAGCTCAAATTGCAAA

>NL01B.C01.01_A_36

GAGTATATAGTTCATAGCATCTTTCATTTCTTTCTCCTCAA

>NL01B.C01.01_A_37

ACAGCTTGCACAAGTTTTGCCAAAAATAGTTAGTTCTCAGT

>NL01B.C01.01_A_38

ATAATGAGTATTTGAATGCGTTAGCTGAATTTGATAGAACTGG

>NL01B.C01.01_A_39

GTCCTGTTTGTTTCACCAACTTGTTGAAAACCTGCTTTTT

>NL01B.C01.01_A_40

CTTCTTCGGACGAGTTGAAAAGATAATAGTATGTATCTGG

>NL01B.C01.01_A_41

ATACATGTAGCATATCTACAACCGGGGCAAATCTATA

>NL01B.C01.01_A_42

GTTACGTTTACGTTTCTGTGCAGCAAGGTAATTCGTTTAG

>NL01B.C01.01_A_43

TAGTATTTGCTTAGGCCCCGGCGCTGTAGAATGTAAGGCA

>NL01B.C01.01_A_44

TATAAATCATGAGTTTTTCCACTAAAAAAATAATGAAACA

>NL01B.C01.01_A_45

ATCTCCTACAACCTATCGGTGCTTATAGGAGTAATACCTA

>NL01B.C01.01_A_46

TATTAATTTCCGTAGTAATCAATTCTGTAAACTTATTAA

>NL01B.C01.01_A_47

TTGAGTATATGGACTTTAACGGTAACTGGCATACAAATAG

>NL01B.C01.01_A_48

GAGACCTTGATAAGATTATTACTACGCGACGATTTG

>NL01B.C01.01_A_49

ATATAACATATGTAAAAACTAGTATTTAAAGTTTTCTTT

>NL01B.C01.01_A_50

ACTTTCTGAAAGATTACGGCGATTATTTTGTAATGATAAT

>NL01B.C01.01_A_51

TCTGCAAGTTCAAAGAGTTCTTTTGCAATTTCGTAATTATT

>NL01B.C01.01_A_52

ACATCACGTATCTCTGCTAATAAAATATGGTCTATTGAACA

>NL01B.C01.01_A_53

TTTCTGTCCATGCGTTTTATCGTAATATTCTTCAACTTCTG

>NL01B.C01.01_A_54

TTGTCAAGTTGTATTGGTTCAAATCCGACCTTTGTGG

>NL01B.C01.01_A_55

GCCCCTCCTCCTAAAGGCATTCCTTGACTATGATAATAA

>NL01B.C01.01_A_56

CTATTAGAAATTTCGATATAATTTATCTGTCTTTTCGTTAC

>NL01B.C01.01_A_57

ATTTCTCTTGCTTCTTTCAAATACTCCAAAAGTTTAGGTG

>NL01B.C01.01_A_58

AATTCTCTGGTATTATCATCAATATCATTAGCACTCATT

>NL01B.C01.01_A_59

TGGAAAACTGGCAATTCAACATTTAGACCTACTTCTTGAA

>NL01B.C01.01_A_60

CATTTCATAATGAAGTACAAGACGTTTGACCACATCAGCGG

>NL01B.C01.01_A_61

ATATACATTTCTCTTTCCCCCTTTATAAATTTTTCTCT

>NL01B.C01.01_A_62

CCTGCGATGTCGACCAGAAAGACCTGCGTATAGAAAAAAG

>NL01B.C01.01_A_63

CTTCAGGACTCAAATAGGTAAAGTTAATTTCATTTCCATCC

>NL01B.C01.01_A_64

TTATAGCTTCTTTTAAATACTCTATAATTTCACTATACTG

>NL01B.C01.01_A_65

CCATATAGTGTATATTGGGTTGATACTGCATGGAGTAAATACAAT

>NL01B.C01.01_A_66

AAACTGTATAGCCTTGCTTTACTAATTCATGAGCCAAATTTA

>NL01B.C01.01_A_67

CAGTTAATAACCCAATTACCTATCTTAACGTACACTTCTT

>NL01B.C01.01_A_68

GTTATTGATTCACCTTTTGCATAACATGTTGAAAAAACTT

>NL01B.C01.01_A_69

TGCTTATCTTGAAAATACGGATATTTCTCATCTTTGTAAT

>NL01B.C01.01_A_70

TATTTTTGAGCAAATTCATGTGCTAACTTAATTATTTGTT

>NL01B.C01.01_A_71

AGCACTGATATCATAGATATTAACATACCAAGTGCTACT

>NL01B.C01.01_A_72

GTTTCATCTATGATGACAGCACCACTTATTGATAAGC

>NL01B.C01.01_A_73

TTTCAATTCTATAGTAGATTAGCATACGGGTCAGACGACGATTATG

>NL01B.C01.01_A_74

AGATATAATCGAGAAGAACTAGAAAATGGATTATTATCAA

>NL01B.C01.01_A_75

GGTCTTATGGAGTTGACGCAAAATACATCAAAAAGCATGAAG

>NL01B.C01.01_A_76

ACAGTTGTGCTTGCAGTCGTTGCTTTGATTTATTCTGTCTG

>NL01B.C01.01_A_77

AGCTATTAATATCAATAGAAGACTTATTCGATGTATTTGTGTTG

>NL01B.C01.01_A_78

GTATTGGCAGTGGTACAATACCGATGATTGCGTCGTA

>NL01B.C01.01_A_79

TCATTCATCTTAATTTCAACTCTGTTTTTTTGCAGTTGAG

>NL01B.C01.01_A_80

GATTAACATTTATTACAAATTTCCAAAGTTTGAATTTGC

>NL01B.C01.01_A_81

GTAGGAAAATTAAAGACGAAAGTGTATTTCTGTGTTGCA

>NL01B.C01.01_A_82

AAACCACCAACACCTATAATCATAAAAAGTTTATCTTCCC

>NL01B.C01.01_A_83

ATATTACCTATGCGTACATGTATTCGCTGTCCTTCATTG

>NL01B.C01.01_A_84

TTCTTTTGCATTACATATAGTCTCTTCATCATTACTGCAT

>NL01B.C01.01_A_85

CCTAACCCACCGATACATAAACCAGAACCAATCGTTAA

>NL01B.C01.01_A_86

TCACCGCTTTACTCAGGTCCATCCATAGAAACTGTAAGAATAA

>NL01B.C01.01_A_87

TCTGTATTGTAGAAACTACATTCTGGTAATATTTCGCAT

>NL01B.C01.01_A_88

AATAAACGCCAAGAATTTAGTAAATTCAGGGAATTTC

>NL01B.C01.01_A_89

AAATTCTTTATTCCATCAATTTCTTCTTTTAGTGCGTTTCGT

>NL01B.C01.01_A_90

GGATAGACATGTTTGTATTCTTCACAAAACCACTTAAGTAAG

>NL01B.C01.01_A_91

TTTTTAGCTAGACTTTCAATTTGTGAAATTTGATATTTCACT

>NL01B.C01.01_A_92

AATTCCTTGTTCAACTGCTCTTTGTAAAAGTCTAGCAGTTT

>NL01B.C01.01_A_93

TTCTTTTGCAAATTCTGTAACCTTTTCGCCATGACAGAA

>NL01B.C01.01_A_94

AAGGGCTAGTAGTTGACTTGGGTGTACTAGTGTTACCAT

>NL01B.C01.01_A_95

GTAAATTATAAAATAATAACGCGAAAGAATTAATTTTCCCC

>NL01B.C01.01_A_96

AGGCATATCTGCTCGTTGATGATATTGGGATGGTCATGTATAT

>NL01B.C01.01_A_97

TCCCAAGGCTGGGAGGGTTTTCGTCTCGTCATCAATCAGG

>NL01B.C01.01_A_98

TTTCAATTCTATAGTAGATTAGCCAGACT

>NL01B.C01.01_A_99

TATTGGATTTAATGGAGCATTTAAATTCATATTTCCACACCA

>NL01B.C01.01_A_100

ATATTGTACGTTAAAACAGTGCTAAACTATGATGAATCTA

>NL01B.C01.01_A_101

TGGTCATATACACTTCCAAAAGAGATGCTTCCAATTTTAGTC

>NL01B.C01.01_A_102

TTTTGCTTTAATTCAGTTAAAAGATGTTTCTTAAGTTCACAAG

>NL01B.C01.01_A_103

GGAACTAACGGTATCATAGGAGCGTCTTTCGCATTTTAT

>NL01B.C01.01_A_104

TTAGCCTCCCCGCTAATAATCGTCGTCTGACCCGTAT

>NL01B.C01.01_A_105

TTTTTTCACCTCTAACTAAGAGTCTGTGTTAGACTCTA

>NL01B.C01.01_A_106

GGGGATTAGGTTATGCTCCTGAAGATGCATTGGAAGATGCAGCT

>NL01B.C01.01_A_107

ACTTTACGCGAGGAATGAGGTGAATGAGGAACAGCTGATG

>NL01B.C01.01_A_108

TATGACTTTCCGGACCTTTTTCCTTCTTACGAAGATTATT

>NL01B.C01.01_A_109

TCCCTACAACGAGTTTCATTTACTTTACTTTATGCCTCCAT

>NL01B.C01.01_A_110

TGAGGAGGTTCTTGGTATGGTGGCGGAGCGTATGGAGAATA

>NL01B.C01.01_A_111

GACATTTTTTTATCACCGTGTGCTTAGTTCAATAATTTG

>NL01B.C01.01_A_112

TCTTGTTCTACTTTTTGACTTTTACCCATAGATTTTACACC

>NL01B.C01.01_A_113

GAAATTTTTAGACCTTCAATCTCACCTTTTATCTCAACTTCA

>NL01B.C01.01_A_114

TTATCATATTGTGCATAACTCGCGGATAAATCTGGTCTCATTC

>NL01B.C01.01_A_115

TCGTAATCATCACCGTATTCGTATAACAGTATAACGGTTCTT

>NL01B.C01.01_A_116

CGAGGGGAAAGCTAAGGAGGTAGACTTCAAAAAAGAAGAGAA

>NL01B.C01.01_A_117

CCGTCACCTCCACCACCTGTATTCTCTTCAACACAGAG

>NL01B.C01.01_A_118

CATAAGATCCGCTATCTCGTTTAATAGTAGTGCTAATTCTA

>NL01B.C01.01_A_119

TTAAATTCGCAATTTTAGCATAAATAATTATTTGCTCCTGG

>NL01B.C01.01_A_120

TATTTCTAACTCTGTTGCTCACATGACTGAATTTGTCCATTTA

>NL01B.C01.01_A_121

TTTCTTGAATTTAGCATTGGGGGTATGGGGGTATCCCCCACT

>NL01B.C01.01_A_122

AGACAAAGAAGATGTAATAAGCCTAAGCCATGAGCTTGCGAAAAA

>NL01B.C01.01_A_123

TTTAATTCAAAACTTCCGCCACGGGAAGTCAGAAGATAT

>NL01B.C01.01_A_124

AGATGACGTTACTTTATATATGATCACGAGACCACCTTTGG

>NL01B.C01.01_A_125

ATTTATATCGCTGGATTTATAGCTACTGCAAATCAGCAA

>NL01B.C01.01_A_126

GGGTTTTATAACGTAGTCGTTGTACCGTCTGAAAATAACA

>NL01B.C01.01_A_127

GTAGGGTAGCACAGATAGCCACACTCAAAGCTACTGACGT

>NL01B.C01.01_A_128

TATAAATCGTGAGTTTTTCCGTGAATAAAATAATAAAACAGC

>NL01B.C01.01_A_129

TTTTTTCGTAAAACTTGTGCAATATCCTCTGAAACATTTCG

>NL01B.C01.01_A_130

TTAATGTAAGATTTTAAAAAATGACAAATATAAATCATTC

>NL01B.C01.01_A_131

ATAAACGGTGGCGATTATGCATATATTCGCTCAACATTTTCTC

>NL01B.C01.01_A_132

AGTTTGAAGTTGATTAAAAAAAGGAAGACGACTTATACG

>NL01B.C01.01_A_133

ATATAGACTATTACCCTGAAACTTGTGCTGATAATATAAAGGGACT

>NL01B.C01.01_A_134

TCCATGTAGTATTTTCTAGATAGTTCGACAAACTGTTTCTC

>NL01B.C01.01_A_135

AATTGCCTAATTACATAATTTCCTAATTTCTCCTCCAAT

>NL01B.C01.01_A_136

CCTGTCAGGAGCGTCGTAGTCATAGCAGACGGGGTTTATGT

>NL01B.C01.01_A_137

ACTTTATCACAAATATTGAAATCTTTGCAAACTTTCTCTC

>NL01B.C01.01_A_138

ATTCCTAGAGTCGGAAGATCTATAGAGATATTTGTAGTCGT

>NL01B.C01.01_A_139

ATTTATTAATTATAAAAATTATAACTTGCAAATTAATTATTTA

>NL01B.C01.01_A_140

AAAAACATAGTAGAAATGTTAATGACCAACAAAAGTGAAT

>NL01B.C01.01_A_141

GTCAGCTGTCTAATTCTCTCAATTTCTGAAACTAGCTTTT

>NL01B.C01.01_A_142

ACATAAATTAACTTAATGATATCGTCTTCTCCGACTACGT

>NL01B.C01.01_A_143

CAAACAAGAGAATATGTAAAAGAGTATGCAAATTGGTTA

>NL01B.C01.01_A_144

ATTCCTACAGCAAGGATATGACAAAAAGATTTCACATTATT

>NL01B.C01.01_A_145

ATTTCTTGCTATCTCAGTACCAACAAAATTATGGCAACGT

>NL01B.C01.01_A_146

TTGTCTTACATACACATTTATGTTTGCATTTACGCGTACAAT

>NL01B.C01.01_A_147

ATAATTTCTAATCTTAAGTCGCTGATTACGTCCATCCAGACT

>NL01B.C01.01_A_148

CCAAAATAACTACATGTACTTGGTGTTATAAATCCTACAGT

>NL01B.C01.03_A_1

TAATTTTGAAGATATTATAGAATATTTAGCTGGCGGAGA

>NL01B.C01.03_A_2

CTAGCCCTAAACAGAATTCTTCTTTATCTATCTTATCTA

>NL01B.C01.03_A_3

CATTTGGCATGTACACTTTTTTTTGCATTTTCTTGTACA

>NL01B.C01.03_A_4

TTCTGTTCAGGAACAAATTACATTTTCAGGAAAAATCAAA

>NL01B.C01.03_A_5

TCACCTATGTTCTTTACTCAAGACGCTGACATAATTAACAT

>NL01B.C01.03_A_6

TATGTATAAATTACATTACGAAACCTAAAGGAAACTTTTAC

>NL01B.C01.03_A_7

GTCCTTGCAGTGTCCTCATCTCCTATATCCGTAACATATAACGA

>NL01B.C01.03_A_8

CGAAGTTATTCGCAAAGATATGCAAAATGTTCAGCTAAATT

>NL01B.C01.03_A_9

GGCGAACCTCTTAACCTGTTTGAAGAAGGGAGTCGTGAGACAGA

>NL01B.C01.03_A_10

TCACGTTTTCGTTATCTAGTGGTAGCTCTGTAACAGG

>NL01B.C01.03_A_11

GGATTTCTATGATGAAAGACTAAAGGAGGCAGTGAGAAAA

>NL01B.C01.03_A_12

GTTCTTTTCTAGTTCAGTCTCACAAAGTTTGTTGATTAAA

>NL01B.C01.03_A_13

GTGTACCTACCGACATCGCTTCAACAGGTGGTACACCAA

>NL01B.C01.03_A_14

ATAAATTGTCACTGACAATAAATAAGACAAAGATAGTACTAA

>NL01B.C01.03_A_15

TAAAACGTCTGGAAATCTACAACTATCAACTAACAAATCTT

>NL01B.C01.03_A_16

ATGATAAGAAATAACCTATCTTCCCAAGTTTGAAGTTGA

>NL01B.C01.03_A_17

ATCAATATGAAGCCGGAATACTACAGACAGCTTAGCCTTTACGT

>NL01B.C01.03_A_18

AAGAGAAACACTAAATATGCCACCGAGATTTAAAATAACAA

>NL01B.C01.03_A_19

CTCCGAGGGCAAATATCAGCAAGCCTTACAGCTAGCACAA

>NL01B.C01.03_A_20

TATCTCTTAGCCAATTCTCTTAAATTCCTACTTCTTTCATCTC

>NL01B.C01.03_A_21

TTTCAATTCTATAGTAGATTAGCTTTTCTCTT

>NL01B.C01.03_A_22

ATATAAACAATATAATTACTTAAGTTCTGAAGGATTATTCTT

>NL01B.C01.03_A_23

AGGGCATGGTATGCGATTTTAATGAAGTAGTTATAAAACCGA

>NL01B.C01.03_A_24

ACTTGTACTAAGATATGATCGGATGGCGATGTTATCAT

>NL01B.C01.03_A_25

CAAGCGTATGCAATAAAAAGGGCAAAAGTACTAAAAATGA

>NL01B.C01.03_A_26

TTAATTTGGCGTGTACGTTTATTAAAGCCGGTGAGACG

>NL01B.C01.03_A_27

TCAAACGCACCGTCATATTTAACGGCAGTATAAATGTTGT

>NL01B.C01.03_A_28

TTCCATCCCGCCTTTATACTTCTTCGTAAAAGTCACAGAT

>NL01B.C01.03_A_29

CTACCCGTTGCATGATAAGATTATAATAAACAAAATGGCGGT

>NL01B.C01.03_A_30

GTATATTTTCTAGTCTGGTTATCTTGAGGTCATATAT

>NL01B.C01.03_A_31

CGTATAATTTACAAATATACGAATAAGTACAAGATAATA

>NL01B.C01.03_A_32

TATTTTCTAGTAGCAATTAGAAACACTATCACTATAATCA

>NL01B.C01.03_A_33

ACGTCTGTGCATATTTATGTATCTTTTCTAGCCTCAATCTGTC

>NL01B.C01.03_A_34

TGTTAATATCTTATAACAGAAGAGTTTTAAAAGAAGAAGA

>NL01B.C01.03_A_35

ATAGTGCCGAAACTGGGAAACCCGTAATTGATCCCGAG

>NL01B.C01.03_A_36

ATGCTTATAAACGTGAATTGGCAACTATAATACGGGGCG

>NL01B.C01.03_A_37

AAAGTAATTGAAGGCAATAAAGAGGAAATTTTAAAGTTAATTG

>NL01B.C01.03_A_38

AAACATAATAAAAATGCCTACCAAAAACTCCTTTATTTATTT

>NL01B.C01.03_A_39

ATCGACGTCATCGAAAGCAATGAAAACACCATAAAACTGCA

>NL01B.C01.03_A_40

ATAATTTATTAATTATAACTACTAATTAACTAATTAGCT

>NL01B.C01.03_A_41

AAGTTATACTCTGGGCGGTATCTATACAACTCTATTGCTT

>NL01B.C01.03_A_42

AGTCCTGCAAAAAACCATGATATACTGTCTTTCAATTTT

>NL01B.C01.03_A_43

TTTGATGAATTTACAAGTTGGCAATGGAATTTATTGATTAAATCT

>NL01B.C01.03_A_44

TCTTCAACTGCTCTTTGCAAAAGTCTAGCAGTTTGATTAT

>NL01B.C01.03_A_45

ACGTTATGCTCTAGTGTTATTCGCTGCTGCTATGGCAT

>NL01B.C01.03_A_46

CCACTATCGACCCAAGTTTCATTTCTCCACTTGATTTGC

>NL01B.C01.03_A_47

GTTCCTCTATTAGAAATTTCAATATAATTAATTTGTCTTTT

>NL01B.C01.03_A_48

CACTCGATAACATTGTGACTAAGATGCCTGCACTACCA

>NL01B.C01.03_A_49

GATTCAAAAAGATTTAAATCAAAGTAATTTTTTTGAAGCG

>NL01B.C01.03_A_50

TTATATCCAATTGATTTTTTAACGTTTGTGAGGTCTCTTA

>NL01B.C01.03_A_51

TATTTCAACACGGGATTTCAACACGGGGCGGGGGCA

>NL01B.C01.03_A_52

GAGGTTACGAGGCGAGGAAGAGGGAGGTGAGTTACGAGGTGAGGA

>NL01B.C01.03_A_53

ATACATGATCTGTCTGATGTAGAAGTTGCGGATAAGCTGAG

>NL01B.C01.03_A_54

AAATAGTAATCTGTTAGTACTTGCTGATAACCACCGG

>NL01B.C01.03_A_55

TTGAATAAGTTTGTAAAGAATTCCACTATTGGGTGTAAGTAATT

>NL01B.C01.03_A_56

TCTGGAGGACTCCAAGTTTTTCTTATTGGAACCCATCTTA

>NL01B.C01.03_A_57

ACAAATGTTAATGTAAACGTCACATTTACTGCATCTAACGG

>NL01B.C01.03_A_58

TTAGGAACTGAAATGTTCTCAAGTGGTGTTGGAAGAAC

>NL01B.C01.03_A_59

GCATTTTGGACGACGCCATCTGAATATATGCGTTTATCTGA

>NL01B.C01.03_A_60

TCAATTTTCCATTTTTCAGGTTCCATAATTCCATTTTTGG

>NL01B.C01.03_A_61

TTCTACCTTTTTAACTCTTAACGCTAACTCTCTCTATAGAG

>NL01B.C01.03_A_62

ACAGCCATGGCTTCAATAGGAGGTAGTCCAAAGCCTT

>NL01B.C01.03_A_63

TCAATACAAAGCTATCGGGTGTAAGTCCACTAAGTGTAAC

>NL01B.C01.03_A_64

TAATTTTTAAAGCAGAAACATATAAATATTTTGCAATAACTT

>NL01B.C01.03_A_65

GTTAGCAAAACAAGCGATGTACACTAAAATTACAATAACAAT

>NL01B.C01.03_A_66

AAATAGGTTTTTTTACGCGATTTTACCGTTCGTACTGAA

>NL01B.C01.03_A_67

TTAATATCATTAGTACTCATTAATATCAATTCATATTCACCGT

>NL01B.C01.03_A_68

ACCACATTCCGCCCATAAATTTCCAGATTAACTAAACTCA

>NL01B.C01.03_A_69

TTGAGATTTTTATGATGACTTCTCCCATTCCCTTTTCTCTT

>NL01B.C01.03_A_70

TTATACGTGCAGTGGACAATTAACTTTAGTGTCATCGA

>NL01B.C01.03_A_71

CCTTATTTAATTGTGCGTGTAAAAGCAGATGATGACACAC

>NL01B.C01.03_A_72

TAAATGAAATAATAAGTTTGTGATGAAGGAGCTGGCTCGT

>NL01B.C01.03_A_73

CTCCTCAGCGTAACGTTTTCAACAGCTTCCAACGCCTTCCC

>NL01B.C01.03_A_74

ATGGTAGCGAAGGGGATAGAATATGTGGAGGAAAAATT

>NL01B.C01.03_A_75

TATTGTAATATGAAGACTAAAAATTTATCAATTCATTTAT

>NL01B.C01.03_A_76

TTTATGCAGTTCTGCTAAATCAAAAGGATTATCAACATCT

>NL01B.C01.03_A_77

CAATTTAGCGTATTTGCATGATTTATTAATTTCGCAGA

>NL01B.C01.03_A_78

ACTCTTTTATAACTCCTAGTGCATGTAATTGTTTTTTAATTTCAT

>NL01B.C01.03_A_79

AAGTCTCCTATTTCTTGCATATTCAAGATAAGTATTTTGC

>NL01B.C01.03_A_80

TATTGGCATTAACATAAATAATATATTTAAATTTGAAT

>NL01B.C01.03_A_81

GTTGGAACGCCAGTACGGCTACCGCGTGACATTCATATAGT

>NL01B.C01.03_A_82

TATATTTAAATCTTTCTTTATCTTTTCATGTTTGTATCTTT

>NL01B.C01.03_A_83

TCACAACTGCATGTAAACATAGGACGACTAAGAGAAAA

>NL01B.C01.03_A_84

CTATTGAAAGATATATCAGCTGAAGGAACGTCTCTCACA

>NL01B.C01.03_A_85

ATAAGAGCTTTGCCGGCGTTTCGGTTTTGCTATCGAAAGGA

>NL01B.C01.03_A_86

AACATTAAAATCCACTAATCCTACAATCCGAGAAAATTT

>NL01B.C01.03_A_87

ACTCTAACTATACCTATACCTAGTACATTATCCAATGAATACTT

>NL01B.C01.03_A_88

TCCTTAAGCTTCACCACGACTCTGCGGTCTGTGTTCTC

>NL01B.C01.03_A_89

TATTATGTCATCTAAACTATCTATTGAAGGTGCCACATTACC

>NL01B.C01.03_A_90

CCATATAGTGTATATTGGGTTGATACTGCATGGAGTAAA

>NL01B.C01.03_A_91

TATAAACTTTTCTTTCTAAACTTCCAATTTCTCCAACACTA

>NL01B.C01.03_A_92

TTATAAAAATGTCAGTATTTGGATATTGATTTCCAGTCCAT

>NL01B.C01.03_A_93

TAAACTTTTCTGCCCAACTGGTTAGGAGGAAACTGAGGAGG

>NL01B.C01.03_A_94

AAACTAGTTCAGCAAACCCAGTATAACCCTGTTGCATTGGT

>NL01B.C01.03_A_95

ACAATAGCAATAGCCTTACTGATTGTCGGTGTGATAATAG

>NL01B.C01.03_A_96

TCCGGAGTAACAATAAGCGCTAATTTTGTAGTAACTATCGT

>NL01B.C01.03_A_97

CCCTATATGGTAAGTAATTTTCTTCAACCCATTTTGCAG

>NL01B.C01.03_A_98

GAGCTGGGGCAATTTCGTTATATCTAAAATACTTTCCTTA

>NL01B.C01.03_A_99

AGAACATATTCTAGGGAGTATATTTCAGAGTGGTGGCATAAT

>NL01B.C01.03_A_100

TTCATCGCTCCACCAATACAGCTGTTTGTTCATGACCTCATA

>NL01B.C01.03_A_101

TATTATGCCTAACGAATGTAATTGTTTTTTAATTTCATCAA

>NL01B.C01.03_A_102

TAAATTTGTCCTCTATTTTCTAGTTCTCTAATTTTATCAAT

>NL01B.C01.03_A_103

CTTTGAAAATCATAAGCCGTAGACGGATTTGATGAGAATA

>NL01B.C01.03_A_104

GTTGTCTATCTGCGATGCCTTTAAGTTTATTAAAATAGT

>NL01B.C01.03_A_105

AATTTACAAGCGAAATTGTAATTAATAATTTCGATGGTCAAG

>NL01B.C01.03_A_106

CAATCATTACCAATAATTGTAATTGTCTTATTTTCAACTTGC

>NL01B.C01.03_A_107

CGATAAAAAAAGGTGATCATACCAATATATATTTTGCTAT

>NL01B.C01.03_A_108

TGGACGCGTTACAGAGTGTTATGAAATCACTCGTGGACTTCA

>NL01B.C01.03_A_109

AATTTACAAGCGATTAGAGGCGGTAGATTTCCTAGTTTT

>NL01B.C01.03_A_110

GGTCAAGCACTAGATGAGATGCTTGATTTACTTAACGGAAA

>NL01B.C01.03_A_111

ATAAGCTTACAGGCAACGTTGTAAGAAATGAGTACCTGAAA

>NL01B.C01.03_A_112

TTGACTCGAGAACGGGCATTCCGAATCCTTCAGTTCC

>NL01B.C01.03_A_113

AAACATTCAGATATTAGAATTGAAATAGTTCCGAAAACAAA

>NL01B.C01.03_A_114

ATACTACCAAAAAGTCAAAGAATGGAAAAGTTAATT

>NL01B.C01.03_A_115

TCTGCCAGTTTTTGGTTCCCGGCGTTCTTATATACATCATATA

>NL01B.C01.03_A_116

GTAAATATCTCTACTACCATGCCTTCCTCAATGCCTTCGA

>NL01B.C01.03_A_117

ATTGTTTTTATGTTGAAACATATTAATGGTCAAGTTATAAA

>NL01B.C01.03_A_118

AAAGAAGTTTGCACTAGTAAGCTCGACAACAACTTCTGAA

>NL01B.C01.03_A_119

AGCAAATTGTAGACATCTGTCTCATTTTCTTTGTGTGTT

>NL01B.C01.03_A_120

CTATAGCTTCAGTTAATGTTGTACCATTATTTGCACAATATACT

>NL01B.C01.03_A_121

GTCCTTAAGCCAGTTTCAACTAATAACAGGAAATAAGTCTT

>NL01B.C01.03_A_122

GACTTTCTTAATTTCTATCAGAGAGGCCCAGCGTTTTAGCC

>NL01B.C01.03_A_123

TATTCTTAAATAAATTTACGAACGAGAAGATCAAATACGC

>NL01B.C01.03_A_124

TTAAAAGTTCTAGAACTTTTCTTCCACTTTCTGTAAGTA

>NL01B.C01.03_A_125

TTTCTAAAATCACTATGCCATAGCGAAATCATTATTAGTTT

>NL01B.C01.03_A_126

AGTAGAAAAGAAGAAGATACGTCTTGGAAAAAAGGAAAAAG

>NL01B.C01.03_A_127

CAATGAAACAGAATGGGAATAAATCTTTATTTTCTTTT

>NL01B.C01.03_A_128

GTATCTCAAGACACGATTAATGCACTAGCACAAATTATAAACG

>NL01B.C01.03_A_129

AATATCCGTTCATGGTCAGAAACCTACTAACAGCTTAAGTTT

>NL01B.C01.03_A_130

GTGATACATATTTATCCACTTCAAGATATAGATGAAGCTAT

>NL01B.C01.03_A_131

AACCTAATAATAAACTTTTCGCCCAACCTCCTTTTAGGTCT

>NL01B.C01.03_A_132

TTGCAGGTTTTTACGTTGTATCAGACGCTGAATATCGTCTA

>NL01B.C01.03_A_133

GATTTAACCACAAGTTATGGTCAATATGATAATGGAAAAAA

>NL01B.C01.03_A_134

GAAGATACAATTTTTCCAAGCGAATTAGGAATTTGCTTCAGAAAAT

>NL01B.C01.03_A_135

TTTTTTACCTTAAATTGAGTTATTTTTGAAATAAATTTTA

>NL01B.C01.03_A_136

ATTTCGGGAAAGATAAGGATGAATTGAAAGAAAAATTCC

>NL01B.C01.03_A_137

CTTGTGGGGAGTCTACTATTTCTATCACGATTTTCTT

>NL01B.C01.03_A_138

AAAATTACCGTGAACTCTAGATGAGTTCAAAGGAAGAGTT

>NL01B.C01.03_A_139

TTAAATAGAATACTTACGCTTTGTTATTGGCTTTATTACGA

>NL01B.C01.03_A_140

AACTTCAACAAATCCAGCTTTCTTGTAAAGTGCATTTGA

>NL01B.C01.03_A_141

GGTAGTTGACTTCTTAGTGTCAATATATATATTCTACC

>NL01B.C01.03_A_142

CGCATGCTTTTATTTTTACTTGGTACACCTTTTCTACCTC

>NL01B.C01.03_A_143

TTATTAATTCTGTTAATTGTACACTTTCATTCTTAGTCAA

>NL01B.C01.03_A_144

GTGCCTCTGTTCTGCAGTCTACACAGAAAGCAAACTTTTAA

>NL01B.C01.03_A_145

ACTTCTTCTCATGAACATTTACAAAGGAAGAAGAAGTTGTT

>NL01B.C01.03_A_146

TAACCCAGGTCGTAGACCCGAAAGGAAACGCTGTATACGAA

>NL01B.C01.03_A_147

TGTGCAAACTGCTAAAGTCATCTTGATGAATGACATAGTA

>NL01B.C01.03_A_148

TCTACCGTGTCGATTACCGTCAATTTTTCGTTATCTAGTGGT

>NL01B.C01.03_A_149

CCATTTCTTCTTCTAAATTATCTAAATTTAAATTCTCATCTT

>NL01B.C01.03_A_150

TTTTTATTACCAGTATTTTATTAGCATAACCGTCATAGTCAA

>NL01B.C01.03_A_151

AACTTTGATAATTCTGCAACTGCCTGGTCTGTGGTAATTTGTAA

>NL01B.C01.03_A_152

TGCACCGACCCATTTTGCAATTTGTGTAAATGGAGGATC

>NL01B.C01.03_A_153

TTAATGAGTGGATTGATGAGGCAATGAGACTACATAAA

>NL01B.C01.03_A_154

CCATAAAATACTGAGATAAATCCAAACTGTATACAGCATCTTC

>NL01B.C01.03_A_155

TATCCTTACCGAGTAACTTTGATGAAATATATGCCAATAAT

>NL01B.C01.03_A_156

GTTGACATGTTCAATGATGATATGTTTGAAGCATTACAT

>NL01B.C01.03_A_157

TGATTTATAGCATATCTGTCTAGTAGCTCTAACAAATAA

>NL01B.C01.03_A_158

GTTGGTGCAATTGAACCACTCGTGAAAACAGATGATAAA

>NL01B.C01.03_A_159

AGCTCAACGGCAGGCTTACTAATAGTGTCATATCTCTCCTTCCT

>NL01B.C01.03_A_160

TGATTTTTACATTGTAACATCCAAATACGGGCCTAAGGAA

>NL01B.C01.03_A_161

GTCATTATCAGGTAAGTTGGTAATGGTTTTTTCCACAGTCTG

>NL01B.C01.03_A_162

TATAGATTGAGGGCTGAACTCATTAGGTGGGAACGT

>NL01B.C01.03_A_163

TTGGTTTCGGCTGGCTGTTATACCCCCATGTTGAATATATTT

>NL01B.C01.03_A_164

ATTGTACTTAATGAAGGCAAGTATTTTCTCCAAATTTCA

>NL01B.C01.03_A_165

TTCCCTTACCTTTCACTTTCACGTATTTTTACATAAGCTAA

>NL01B.C01.03_A_166

AATAGGGCAATATATCAAGGCTTTGGCCTAAAAGTTGCT

>NL01B.C01.03_A_167

TAAGTTTCATTTCTATTTCTGTTTCATCTACATCAAAAT

>NL01B.C01.03_A_168

TTTACATCAGCAATTGCTGTAGCATACGGATATGCACCGT

>NL01B.C01.03_A_169

AGTGAAATTCAACTAACTGCACAAACTCCAACTTTACTA

>NL01B.C01.03_A_170

TTTCAGCGTTTAACATCTTCAATGTGGAATACCGCATCACTTT

>NL01B.C01.03_A_171

TGCAACCCATGCTCTTCTGCCATGTAAGTTGTTCCTATACT

>NL01B.C01.03_A_172

TCAAACACATTAAGGAACGGATTACTTTCGGCACTCTGTATC

>NL01B.C01.03_A_173

TTCTGTGGTTGAGCTGAGGTTTTGCACGTCTTCCAAGTTAGG

>NL01B.C01.03_A_174

ATCGCTTCCAAACTAGCTAGGCTATCTGCTATTTCTTTTCT

>NL01B.C01.03_A_175

GTTTTCGGATCTTTTGCAATTAAAAATTGGAAGTAAAA

>NL01B.C01.03_A_176

GCAGTAAATAAGGATTCACCGTTTCAGGAAATTATAAG

>NL01B.C01.03_A_177

TTTAGTTGTATAGTACATTAAAATGCCAAGACCTGTACCCGA

>NL01B.C01.03_A_178

ATAAAATCCTAAATGATTATTTGTCAACTGTTGGATTTCC

>NL01B.C01.03_A_179

AAATTACAATTAATCTGTCATTTAGTGAAATTTGTAATATAAAA

>NL01B.C01.03_A_180

TTATCAAACTTGAGTATCTGTTTGTAGCAATAGGAGTAAC

>NL01B.C01.03_A_181

ACTTTAGGAGTTGGAAATATCCAAATTCCAAATGTTGAT

>NL01B.C01.03_A_182

TTTTTCGACTTTTTTGTGTTGTAGTTCAACTTCAAATTTCC

>NL01B.C01.03_A_183

ATTCTTAGTACCACAGGATTATACAAAAACACAGCACCT

>NL01B.C01.03_A_184

CATAAAGTACTACGTCATTGTTATAGAACTTCTTCCTTTCC

>NL01B.C01.03_A_185

ATTTCTGATGGAAATATTGTATCTTCAGGATATTTAACTTT

>NL01B.C01.03_A_186

CCTAGTAAGTTTAGAGTCATCGCTAAAGCAAACTCTTCAG

>NL01B.C01.03_A_187

TTGTGGATATCAAACTTACGAATATTTACAGCTTCTACAA

>NL01B.C01.03_A_188

ATTCTCAGAGACTTCATACTCCGTTTCATCATTGACAGT

>NL01B.C01.03_A_189

GTATGCTTCTTGCACTACAGGCATATATTGCTGTTGGACTTC

>NL01B.C01.03_A_190

GTTATCCATCAGCTCATGCCACCGTTTGATGAATTTACATCA

>NL01B.C01.03_A_191

AATTTGACCTCACAATAGAAAAAATAAAAAAATAGAAT

>NL01B.C01.03_A_192

CTAGGTTGTTGTTGCAGTTTTGCAACTACAAGTATAT

>NL01B.C01.03_A_193

AATTATATCCACCATATCTTAGACCTACACTGTTTGCTAGT

>NL01B.C01.03_A_194

AAGCAATATCTAAACTCAATAAGATACTATCTGCACACAGT

>NL01B.C01.03_A_195

CTTATTACTTTATGGAGTATATTTAAAATGGTGTTTTTAA

>NL01B.C01.03_A_196

TCACTTTCTTCTCCCTCTTCTCCGCTTTGACCCTCTTCGT

>NL01B.C01.03_A_197

ATTCTCATTCTCTTTCATCATTATAATATACGCTTTCGAAATATA

>NL01B.C01.03_A_198

AATACCAGGATACCAATCCTCTTCCTTTTTGTCGTGAACAA

>NL01B.C01.03_A_199

ATTGTCACTTACACGCAGTACATTACGCGGTGAGGCAGA

>NL01B.C01.04_A_1

ATATCTTGTGTATGTTACACTTAAAAAAGTGGTCAAACAG

>NL01B.C01.04_A_2

CAAAACAATCCGTTTTCTAACTGCTTAATTGCGGAAGCCA

>NL01B.C01.04_A_3

TCTCATATGGTATAATCGTAAAGTAAGCAAGATCTGCCTT

>NL01B.C01.04_A_4

CATCTTTTTCTCCCCTCCCTATAATATAACACATTCTGGTTTT

>NL01B.C01.04_A_5

TTTTCTCCCTATTCCTGGTAATTGTACTAACGCTAAAGCT

>NL01B.C01.04_A_6

CATAATGTAGTTCATAACTTGCAAAATACAAATTCTTTAT

>NL01B.C01.04_A_7

TTCAGATATCCAATTTCAATAATTCATCCATTCTTCTAT

>NL01B.C01.04_A_8

GGCTATGTACTCGTAAACGGTGTGCGAATAAGGAGGAAAAT

>NL01B.C01.04_A_9

ATGTTTACATGCAGTTGTGATGGTGGTATGATATTGACAG

>NL01B.C01.04_A_10

TAAATCTGCGTAATTTTTACACTTTCATCTTTCCCCTTGT

>NL01B.C01.04_A_11

CGTTAAGGTCTGACTTTCTCTCCACCTAGTTCCTCTCAAA

>NL01B.C01.04_A_12

TCTTCTCTTCTTTAGTACTAGAAACTTGACTCATCAATAT

>NL01B.C01.04_A_13

CTTAATGCTCCTTCGCCGAGAGAATAGAACCCCTTACGA

>NL01B.C01.04_A_14

ATTTCTCTTGCTTCTTTCAAATACTCCAAAAGTTTAGGTG

>NL01B.C01.04_A_15

ATTGATCTTATTGCAGAAGCGGTTATAGGCACATATGGTTCTTC

>NL01B.C01.04_A_16

ATAGCCTTCTTTCTCTTGTAAATTTCTTCAATCACTTCCGC

>NL01B.C01.04_A_17

TCAGCATACTTCCTAGCCTTACAGAACAACTATACTCAAG

>NL01B.C01.04_A_18

TCGCCGCCCCCGTGAATTTTTGCAAACCGAGAAAAGTGAGT

>NL01B.C01.04_A_19

AATGTACTAAATTCCGTGGCGTCCATGTACGGCATCGAGCCAG

>NL01B.C01.04_A_20

AATTTTTAGGGCTAACTGAGAAAAAAGTTAGGAAATATT

>NL01B.C01.04_A_21

AGTATACCTGAAGTTAGATTGGGACAATCGGATATAAA

>NL01B.C01.04_A_22

TGAACCTATCGAGGAATGCTCTGTCCGCTTGTGTGTTTACCCT

>NL01B.C01.04_A_23

TTATTATTATTTACGATTGCTAATGAGTAAAGTGGAATATTG

>NL01B.C01.04_A_24

CTATAGGTGTTGAAATGAAATATTCTACTAGACCGAA

>NL01B.C01.04_A_25

AACTATCCTAGGATCTCGTCCCTATGTTTGAGACAATATTCGCG

>NL01B.C01.04_A_26

AAGAAGGTACAACAGACAAATACATTGTGTTAACAAATCTAA

>NL01B.C01.04_A_27

CGAGGGGAAAGCTAAGGAGGTAGACTTCAAAGAAGAAGAGAA

>NL01B.C01.04_A_28

GTTGATATAAACGTCTGTGAAAAAGTAAAAATATATGATAG

>NL01B.C01.04_A_29

ACAGCTTGCACAAGTTTTGCCAAAAATAGTTAGTTCTCAGT

>NL01B.C01.04_A_30

CAATACGCCTATAACCACTCTTTTTCACTTTCGCTATAATACACT

>NL01B.C01.04_A_31

CTTCTTCGGACGAGTTGAAAAGATAATAGTATGTATCTGG

>NL01B.C01.04_A_32

AGGATTGAGAAACAACACATCGATTTTGCAATTGCACATAT

>NL01B.C01.04_A_33

TTATGATTGCTTTCCAAGTTTGACCATTCCAGAATAGG

>NL01B.C01.04_A_34

TTTACTTCAGCTAGCTGAATAAAAAAATTTAAATTAA

>NL01B.C01.04_A_35

TTACCCTCAGCTGTTTGGTCTCCTATGTTAGGTGAAAATA

>NL01B.C01.04_A_36

AATAATATTTCGCCTCATTTCTCCCTTCTTTTATTGACTGTTC

>NL01B.C01.04_A_37

TGATAGTTCCTTATATTCATTTTCCGACATTGACAACGCA

>NL01B.C01.04_A_38

CCGTCACCTCCACCACCTGTATACTCTTTCAATTCTATAGTAGATTAG

>NL01B.C01.04_A_39

ACCGTCGCTGAAATACGTTAGTGTCAACTCGCCCTCC

>NL01B.C01.04_A_40

TCCTTAAGCTTCACTACGACTCTACTGTCTAGGTTCTGT

>NL01B.C01.04_A_41

ATAATAAGACCGGGGTTACCGCTAATTGCAGCCATTTCG

>NL01B.C01.04_A_42

TAAAGAGTTGTCCATTTACAATATTTCCAATAATTGTATGT

>NL01B.C01.04_A_43

AATTATTTCTTTAAATGCTGAAATTGTTGAACAAATTG

>NL01B.C01.04_A_44

CCTTATATATGCGTACTTGCTCCTCCTCTTTTGGAAGATT

>NL01B.C01.04_A_45

ACATCACGTATCTCTGCTAATAAAATATGGTCTATTGAACA

>NL01B.C01.04_A_46

AAAAGTTGCTAGGGCACTTAATAGACTCGGCGGCGGGCCTGCTC

>NL01B.C01.04_A_47

TCCTTTATTAATGTTTGCAAGAAAGCACCACCATATACGG

>NL01B.C01.04_A_48

TTTCTGTCCATGCGTTTTATCGTAATATTCTTCAACTTCTG

>NL01B.C01.04_A_49

TTGCCAACATGTGTCCTGTAGTTCCTAGGTTCATTG

>NL01B.C01.04_A_50

ACCCCAGCGGAAAATTCACAGTCACGTTCAACGAGGTGGG

>NL01B.C01.04_A_51

AAAACTGTTCTTTCCTTTTTTGTTCTCTCCATCATTTCTCCT

>NL01B.C01.04_A_52

CTTTCCAATATAAGTTAATATTTCTTGAAAGAGTCCCTTAG

>NL01B.C01.04_A_53

GTTTAGGAATTAATAATTGCAATTGTTTACTAACTTTAGAT

>NL01B.C01.04_A_54

CAATTTTTGGAAGTTTGTTTAAATTACATTTATATGATTCT

>NL01B.C01.04_A_55

TAAGCAGGAACAAGTAAACACGCAACAATTATCCAACATACTG

>NL01B.C01.04_A_56

TTAATTTGTTCTAACTGAATTGAAAAAACTAACTTAATTT

>NL01B.C01.04_A_57

CCGTTTATTGGAATTTTTTTAGTCAAAATGTAATACATTAC

>NL01B.C01.04_A_58

CTTCAGGACTCAAATAGGTAAAGTTAATTTCATTTCCATCC

>NL01B.C01.04_A_59

GTATTGGCAGTGGTACAATACCGATGATTGCGTCGTA

>NL01B.C01.04_A_60

TTCATGGTCAGAAACCAACAAACAGCCTAAGTTTTCCAG

>NL01B.C01.04_A_61

AATTCGTTAAACCAAGCTTTTACCATTTGCTTTCATCCCT

>NL01B.C01.04_A_62

TTAAGTGCAAATGCATTAGTGAGTTTAAAGAAATTGAT

>NL01B.C01.04_A_63

CTTATTATTCGTTGTATTTACGCTTCGTAACAACAGTATACT

>NL01B.C01.04_A_64

ACAGTTGTGCTTGCAGTCGTTGCTTTGATTTATTCTGTCTG

>NL01B.C01.04_A_65

TAGTTATAACGGGTGGGTATTTGGTGTAAAACATGACGTT

>NL01B.C01.04_A_66

GATTAACATTTATTACAAATTTCCAAAGTTTGAATTTGC

>NL01B.C01.04_A_67

AAACCACCAACACCTATAATCATAAAAAGTTTATCTTCCC

>NL01B.C01.04_A_68

GTCTAAGTCTGCTGCGTCTAGCCATATTCGGTTGTATTGTG

>NL01B.C01.04_A_69

CCAGGTTCCTCCTTAGGACTTTAGGGTCTTTTCTCTGCT

>NL01B.C01.04_A_70

TTGTTAGATTACAAGTATTGGCAGGAGAGAACAACGCCAG

>NL01B.C01.04_A_71

AATAAACGCCAAGAATTTAGTAAATTCAGGGAATTTC

>NL01B.C01.04_A_72

TAAATAAACCCCGTAGCTCACCTCGCCTAGGAAAATAGT

>NL01B.C01.04_A_73

TATAGCGCCGATTGTTCCATATTTACCTATTTTAAGTA

>NL01B.C01.04_A_74

CTTATAGGGTCTCTAACCTCTCCATTCACTTCCTCTTCTACCTC

>NL01B.C01.04_A_75

AAGTTATACTTTTATAAATATATAAATATGTCATATTATAAGT

>NL01B.C01.04_A_76

CATATTCAAACTCTCTAGTTTCATCATTAATATCATTAGTACT

>NL01B.C01.04_A_77

CTATTATTGAAGTTTACTCTTGCTTATGTGCAGACCCATT

>NL01B.C01.04_A_78

TTTCTGGTTGCCAAATCGGGCCAAACCCGGTTCTTCTTAA

>NL01B.C01.04_A_79

AGAGAAACAGCAGGTACAAAATCAACTCAGCCAAGTACAA

>NL01B.C01.04_A_80

AATGCCATCTATCTCACCCGTGCTTGTGCTTCTTCTTGTGA

>NL01B.C01.04_A_81

ATTTTCACGCTGTTAAGTGTCGGGACGGCCAAGCCGA

>NL01B.C01.04_A_82

ATATCATAAAAATCCAATACTTTTGCATGCAATTGCGGG

>NL01B.C01.04_A_83

AATCAGGTATAACGACATAATGGTTGAATGCAGGCCTACAAG

>NL01B.C01.04_A_84

GGGGATTAGGTTATGCTCCTGAAGATGCATTGGAAGATGCAGCT

>NL01B.C01.04_A_85

AAACCAAATTCCAACAGACCCTGAAACAGACGTTTGCA

>NL01B.C01.04_A_86

TGATATAGTCTATGGTATCGGGGTCAATAGGCTCTCCACATT

>NL01B.C01.04_A_87

TCCCTACAACGAGTTTCATTTACTTTACTTTATGCCTCCAT

>NL01B.C01.04_A_88

TCTTGTTGTTTTTCTCCTTTTCCGCTTTGCTTCTTTTCGCCT

>NL01B.C01.04_A_89

AATATCGTTATGAATTTGTCGTTTGGTAATGGTCGTTTGC

>NL01B.C01.04_A_90

TGAATTACTTTCTCATTATCCACAGTTTGTGAAATTTGG

>NL01B.C01.04_A_91

CCGTCACCTCCACCACCTGTATTCTCTTCAACACAGAG

>NL01B.C01.04_A_92

AGTATGAGCTAGAAGTGGATCTTACAACTACTGTTTCTTT

>NL01B.C01.04_A_93

ATTATAACAATGATGTCATCTGGATTCATCCAGACCACTCAA

>NL01B.C01.04_A_94

TTTAATAAATGTAAAGCCTAAGCCACAGCCAACTACTTCCC

>NL01B.C01.04_A_95

TAAAGCCCAATTTGAAGCACTAGAAAAAGCAAAAAGGTGAAAA

>NL01B.C01.04_A_96

TAATTTTCTGATAAATCGGGATTAATACTTTATAATAGCTCTGAT

>NL01B.C01.04_A_97

AAGGAGTTGTGGAAACATCCAGTTGAAGGCCTTCTATAGT

>NL01B.C01.04_A_98

AAAATCACTATGCCATAGCGAAATCATTATTAGCTTAATTT

>NL01B.C01.04_A_99

GATGGCATAGTCCATGGGGATACCCAGTGTGAGGCTAT

>NL01B.C01.04_A_100

TTGTTAATCACTATAAGGAAAAACAACACAATGCCGT

>NL01B.C01.04_A_101

CACTTCTAAATAACATATTATTTATGCAAAAAAGATCTA

>NL01B.C01.04_A_102

AAAGAAAGGTGCAACACATCGACCGCAAACGCATAAATAA

>NL01B.C01.04_A_103

TTTTAAAGCTGTGCGAAAAAGAAAAATTTAAAAATTGTGA

>NL01B.C01.04_A_104

TATAGCTACTGCAGGTATCAATACGTATTACTTGCCTCTG

>NL01B.C01.04_A_105

TAATTGTTTCGAATACTTTGTAATATACATTAAGTAATAAG

>NL01B.C01.04_A_106

AACGGAAATTCTGTAAGATATTCAATGACTATACATAGCCAA

>NL01B.C01.04_A_107

TTCTTCAATAAACAATATCACTACATCACTTTTGGGAATA

>NL01B.C01.04_A_108

TTTTTTCGTAAAACTTGTGCAATATCCTCTGAAACATTTCG

>NL01B.C01.04_A_109

CACTCATTTTTATACTGAACCCCCTTAAAGATTCTGATGA

>NL01B.C01.04_A_110

CACGGTGACTTTGCGGAAGAAATGCTATCGTTCTATGCAAAT

>NL01B.C01.04_A_111

CTTCTTTTTAACCGTTAGATTTTTAAACCCCGTTCTAGT

>NL01B.C01.04_A_112

AGACTCTTTCGCTCCAGACCAAGTGTTAGACTTAAGAG

>NL01B.C01.04_A_113

GTTTGTTTTCCTGCTTGAGTAATGTAATGATATCGTCGC

>NL01B.C01.04_A_114

TACATATTATTTCTAGCATCATAGACTAAATTGGCATAAGTCGG

>NL01B.C01.04_A_115

CTGCTGGCGGTCTCGTAATTATATACGAAGCGACTTCGTCTGG

>NL01B.C01.04_A_116

GTACCTTTACTCCATGGCTAAAGCGTTTTCCATATATCACG

>NL01B.C01.04_A_117

ACAATATAGGAAGTCGCATTGGGTCTTAACTGGAGAT

>NL01B.C01.05_A_1

TTTCAATTCTATAGTAGATTAGCCCTTCAAATCCGACCTTTGTGG

>NL01B.C01.05_A_2

ACCTTCTTACCCATAATTATTATATTCATAAAATGTTAA

>NL01B.C01.05_A_3

AAAGAGTATTCCCTTCAACATATGCTTTTTTTCAGTTAAACTTT

>NL01B.C01.05_A_4

CATTATTCTCTCTACTCTTGCTTTCGGCACCGTATTTTT

>NL01B.C01.05_A_5

CGAAGTGACGGTGAACGTTATGACCTGGACGACGATTATG

>NL01B.C01.05_A_6

TCACGTTTTACCACCTTTTACAGCTTTCGTTACTTCT

>NL01B.C01.05_A_7

ATCCAAGCTCTCCCTCCAACACCGGCATAACAGGGCTGTAT

>NL01B.C01.05_A_8

TTTGATACCGTTATGATAGACTGAACCAGTTAGTATGCCA

>NL01B.C01.05_A_9

GCTGAATTCTCCGCCAGGATGACGCTGAAGGTAAATGGGA

>NL01B.C01.05_A_10

TTCTCATCGACTTTGCCCAAGAACTTTGAGAATTGTTTC

>NL01B.C01.05_A_11

ATCAGTTAGATTCAAGTTGTTGCTTGATCTTGATTACGATTA

>NL01B.C01.05_A_12

TAGAGATGCAGAAAGTGAAAGTGATGATAGGAGGGATGTA

>NL01B.C01.05_A_13

TTCTTTTTATGTGAAATTTGACATAATTATCAAGCTTTAGA

>NL01B.C01.05_A_14

GTATTGGCAGTGGTACAATACCGATGATTGCGTCGTA

>NL01B.C01.05_A_15

TAGGACTTCATCTTCAGCAGTATATTGTGTGCTTCCAGG

>NL01B.C01.05_A_16

CCATATAGTGTATATTGGGTTGATACTGCATGGAGTAAATACAAT

>NL01B.C01.05_A_17

GAGGGGAAGGTCTTGAGTACACAAGAAATCAGGGAGATGTATAA

>NL01B.C01.05_A_18

ATATTGTACGTTAAAACAGTGCTAAACTATGATGAATCTA

>NL01B.C01.05_A_19

ATCATTATCGTCACAATTATATTTCTTGTCCAACATTTTCT

>NL01B.C01.05_A_20

AATTAAAATCAGTGTCGAGATAAACCCAAAAACGGGCAGG

>NL01B.C01.05_A_21

TTTATACTGCCGTTAAATATGACGGTGCGTTTGAAGG

>NL01B.C01.05_A_22

GTTATTGATTCACCTTTTGCATAACATGTTGAAAAAACTT

>NL01B.C01.05_A_23

TTTTTGGTCAGTATATTTTTATGCCAACCCGGTATTTA

>NL01B.C01.05_A_24

GTTTCATCTATGATGACAGCACCACTTATTGATAAGC

>NL01B.C01.05_A_25

TCGCCGCCCCCGTGAATTTTTGCAAACCGAGAAAAGTGAGT

>NL01B.C01.05_A_26

TATTATAATTCCCCATACGATTTTCCAGACCTTTTCCCTTCT

>NL01B.C01.05_A_27

ACTAACATTATTAAATGCTTTCTTCTGCCTTATAAACTTTTCTTTC

>NL01B.C01.05_A_28

TGCTGTTAGGTATTGTCCTGAACACGAAGCCGATGAAGAA

>NL01B.C01.05_A_29

AGTATACCTGAAGTTAGATTGGGACAATCGGATATAAA

>NL01B.C01.05_A_30

GCTACTGTGGTATATAGCATGACAACTGGGTTACCATA

>NL01B.C01.05_A_31

TGAACTTGTAAAAAAAGGCTATTCAATAGCTCAAATTGCAAA

>NL01B.C01.05_A_32

GCAATAATAGATAAATTGGCTGAAATTCTTCCTGAGAT

>NL01B.C01.05_A_33

TTATTATTATTTACGATTGCTAATGAGTAAAGTGGAATATTG

>NL01B.C01.05_A_34

ATAATTAAAAGCCATATTGGATCTATTGATGTATTTAAATT

>NL01B.C01.05_A_35

TTGAAAAGGTGTCATTAAACTTCATATATAGGAATGAGACGA

>NL01B.C01.05_A_36

TTTGGTATATGACAGGAGGCATCCTCTATTAGAGATGGCG

>NL01B.C01.05_A_37

GATATAGATGAAATATTCAAGTGATACTATGCTAAAAGATTA

>NL01B.C01.05_A_38

TAGTATTTGCTTAGGCCCCGGCGCTGTAGAATGTAAGGCA

>NL01B.C01.05_A_39

GAGTATATAGTTCATAGCATCTTTCATTTCTTTCTCCTCAA

>NL01B.C01.05_A_40

ACAGCTTGCACAAGTTTTGCCAAAAATAGTTAGTTCTCAGT

>NL01B.C01.05_A_41

GTCCTGTTTGTTTCACCAACTTGTTGAAAACCTGCTTTTT

>NL01B.C01.05_A_42

CTTCTTCGGACGAGTTGAAAAGATAATAGTATGTATCTGG

>NL01B.C01.05_A_43

ATACATGTAGCATATCTACAACCGGGGCAAATCTATA

>NL01B.C01.05_A_44

GTTACGTTTACGTTTCTGTGCAGCAAGGTAATTCGTTTAG

>NL01B.C01.05_A_45

GGTCTTATGGAGTTGACGCAAAATACATCAAAAAGCATGAAG

>NL01B.C01.05_A_46

TATAAATCATGAGTTTTTCCACTAAAAAAATAATGAAACA

>NL01B.C01.05_A_47

ATCTCCTACAACCTATCGGTGCTTATAGGAGTAATACCTA

>NL01B.C01.05_A_48

TATTAATTTCCGTAGTAATCAATTCTGTAAACTTATTAA

>NL01B.C01.05_A_49

TTGAGTATATGGACTTTAACGGTAACTGGCATACAAATAG

>NL01B.C01.05_A_50

GAGACCTTGATAAGATTATTACTACGCGACGATTTG

>NL01B.C01.05_A_51

ATATAACATATGTAAAAACTAGTATTTAAAGTTTTCTTT

>NL01B.C01.05_A_52

ACTTTCTGAAAGATTACGGCGATTATTTTGTAATGATAAT

>NL01B.C01.05_A_53

TCTGCAAGTTCAAAGAGTTCTTTTGCAATTTCGTAATTATT

>NL01B.C01.05_A_54

ACATCACGTATCTCTGCTAATAAAATATGGTCTATTGAACA

>NL01B.C01.05_A_55

TTTCTGTCCATGCGTTTTATCGTAATATTCTTCAACTTCTG

>NL01B.C01.05_A_56

TTGTCAAGTTGTATTGGTTCAAATCCGACCTTTGTGG

>NL01B.C01.05_A_57

GCCCCTCCTCCTAAAGGCATTCCTTGACTATGATAATAA

>NL01B.C01.05_A_58

CTATTAGAAATTTCGATATAATTTATCTGTCTTTTCGTTAC

>NL01B.C01.05_A_59

AATTCTCTGGTATTATCATCAATATCATTAGCACTCATT

>NL01B.C01.05_A_60

ATAATATATTATTAAAAATATGAACATCAAAAAAAGAACGTCCA

>NL01B.C01.05_A_61

TGGAAAACTGGCAATTCAACATTTAGACCTACTTCTTGAA

>NL01B.C01.05_A_62

TCATTCATCTTAATTTCAACTCTGTTTTTTTGCAGTTGAG

>NL01B.C01.05_A_63

ATATACATTTCTCTTTCCCCCTTTATAAATTTTTCTCT

>NL01B.C01.05_A_64

CCTGCGATGTCGACCAGAAAGACCTGCGTATAGAAAAAAG

>NL01B.C01.05_A_65

CTTCAGGACTCAAATAGGTAAAGTTAATTTCATTTCCATCC

>NL01B.C01.05_A_66

TTATAGCTTCTTTTAAATACTCTATAATTTCACTATACTG

>NL01B.C01.05_A_67

AAACTGTATAGCCTTGCTTTACTAATTCATGAGCCAAATTTA

>NL01B.C01.05_A_68

AATTGCCTAATTACATAATTTCCTAATTTCTCCTCCAAT

>NL01B.C01.05_A_69

AAACAAGGGCGAAAAGCAACAAGAAGGGCAGAAGCA

>NL01B.C01.05_A_70

TGCTTATCTTGAAAATACGGATATTTCTCATCTTTGTAAT

>NL01B.C01.05_A_71

TATTTTTGAGCAAATTCATGTGCTAACTTAATTATTTGTT

>NL01B.C01.05_A_72

AGCACTGATATCATAGATATTAACATACCAAGTGCTACT

>NL01B.C01.05_A_73

TGGTCATATACACTTCCAAAAGAGATGCTTCCAATTTTAGTC

>NL01B.C01.05_A_74

AGATATAATCGAGAAGAACTAGAAAATGGATTATTATCAA

>NL01B.C01.05_A_75

GTAGGGTAGCACAGATAGCCACACTCAAAGCTACTGACGT

>NL01B.C01.05_A_76

ACAGTTGTGCTTGCAGTCGTTGCTTTGATTTATTCTGTCTG

>NL01B.C01.05_A_77

AGCTATTAATATCAATAGAAGACTTATTCGATGTATTTGTGTTG

>NL01B.C01.05_A_78

TATAAATCGTGAGTTTTTCCGTGAATAAAATAATAAAACAGC

>NL01B.C01.05_A_79

CATTTCATAATGAAGTACAAGACGTTTGACCACATCAGCGG

>NL01B.C01.05_A_80

GATTAACATTTATTACAAATTTCCAAAGTTTGAATTTGC

>NL01B.C01.05_A_81

GTAGGAAAATTAAAGACGAAAGTGTATTTCTGTGTTGCA

>NL01B.C01.05_A_82

AAACCACCAACACCTATAATCATAAAAAGTTTATCTTCCC

>NL01B.C01.05_A_83

ATATTACCTATGCGTACATGTATTCGCTGTCCTTCATTG

>NL01B.C01.05_A_84

TTCTTTTGCATTACATATAGTCTCTTCATCATTACTGCAT

>NL01B.C01.05_A_85

CCTAACCCACCGATACATAAACCAGAACCAATCGTTAA

>NL01B.C01.05_A_86

TCACCGCTTTACTCAGGTCCATCCATAGAAACTGTAAGAATAA

>NL01B.C01.05_A_87

TCTGTATTGTAGAAACTACATTCTGGTAATATTTCGCAT

>NL01B.C01.05_A_88

AATAAACGCCAAGAATTTAGTAAATTCAGGGAATTTC

>NL01B.C01.05_A_89

AACTTTTTTGCTAATTCCTCCAATTTGATACTTTCTTCCTTA

>NL01B.C01.05_A_90

AAATTCTTTATTCCATCAATTTCTTCTTTTAGTGCGTTTCGT

>NL01B.C01.05_A_91

TATTTCTAACTCTGTTGCTCACATGACTGAATTTGTCCATTTA

>NL01B.C01.05_A_92

TTTTTAGCTAGACTTTCAATTTGTGAAATTTGATATTTCACT

>NL01B.C01.05_A_93

AATTCCTTGTTCAACTGCTCTTTGTAAAAGTCTAGCAGTTT

>NL01B.C01.05_A_94

TTCTTTTGCAAATTCTGTAACCTTTTCGCCATGACAGAA

>NL01B.C01.05_A_95

AAGGGCTAGTAGTTGACTTGGGTGTACTAGTGTTACCAT

>NL01B.C01.05_A_96

GTAAATTATAAAATAATAACGCGAAAGAATTAATTTTCCCC

>NL01B.C01.05_A_97

AGGCATATCTGCTCGTTGATGATATTGGGATGGTCATGTATAT

>NL01B.C01.05_A_98

TCCCAAGGCTGGGAGGGTTTTCGTCTCGTCATCAATCAGG

>NL01B.C01.05_A_99

TATTGGATTTAATGGAGCATTTAAATTCATATTTCCACACCA

>NL01B.C01.05_A_100

ATTTCTCTTGCTTCTTTCAAATACTCCAAAAGTTTAGGTG

>NL01B.C01.05_A_101

TTTTGCTTTAATTCAGTTAAAAGATGTTTCTTAAGTTCACAAG

>NL01B.C01.05_A_102

GGAACTAACGGTATCATAGGAGCGTCTTTCGCATTTTAT

>NL01B.C01.05_A_103

TTAGCCTCCCCGCTAATAATCGTCGTCTGACCCGTAT

>NL01B.C01.05_A_104

TTTTTTCACCTCTAACTAAGAGTCTGTGTTAGACTCTA

>NL01B.C01.05_A_105

GTCAGCTGTCTAATTCTCTCAATTTCTGAAACTAGCTTTT

>NL01B.C01.05_A_106

GGGGATTAGGTTATGCTCCTGAAGATGCATTGGAAGATGCAGCT

>NL01B.C01.05_A_107

ACTTTACGCGAGGAATGAGGTGAATGAGGAACAGCTGATG

>NL01B.C01.05_A_108

TATGACTTTCCGGACCTTTTTCCTTCTTACGAAGATTATT

>NL01B.C01.05_A_109

TCCCTACAACGAGTTTCATTTACTTTACTTTATGCCTCCAT

>NL01B.C01.05_A_110

TGAGGAGGTTCTTGGTATGGTGGCGGAGCGTATGGAGAATA

>NL01B.C01.05_A_111

GACATTTTTTTATCACCGTGTGCTTAGTTCAATAATTTG

>NL01B.C01.05_A_112

TCTTGTTCTACTTTTTGACTTTTACCCATAGATTTTACACC

>NL01B.C01.05_A_113

GAAATTTTTAGACCTTCAATCTCACCTTTTATCTCAACTTCA

>NL01B.C01.05_A_114

TTATCATATTGTGCATAACTCGCGGATAAATCTGGTCTCATTC

>NL01B.C01.05_A_115

TCGTAATCATCACCGTATTCGTATAACAGTATAACGGTTCTT

>NL01B.C01.05_A_116

CGAGGGGAAAGCTAAGGAGGTAGACTTCAAAAAAGAAGAGAA

>NL01B.C01.05_A_117

CCGTCACCTCCACCACCTGTATTCTCTTCAACACAGAG

>NL01B.C01.05_A_118

CATAAGATCCGCTATCTCGTTTAATAGTAGTGCTAATTCTA

>NL01B.C01.05_A_119

TTAAATTCGCAATTTTAGCATAAATAATTATTTGCTCCTGG

>NL01B.C01.05_A_120

GGATAGACATGTTTGTATTCTTCACAAAACCACTTAAGTAAG

>NL01B.C01.05_A_121

TTTCTTGAATTTAGCATTGGGGGTATGGGGGTATCCCCCACT

>NL01B.C01.05_A_122

AGACAAAGAAGATGTAATAAGCCTAAGCCATGAGCTTGCGAAAAA

>NL01B.C01.05_A_123

TTTAATTCAAAACTTCCGCCACGGGAAGTCAGAAGATAT

>NL01B.C01.05_A_124

AGATGACGTTACTTTATATATGATCACGAGACCACCTTTGG

>NL01B.C01.05_A_125

TGCTATTTTACGCTCCAGCGCTGCTATTGTATTAAGCGCTT

>NL01B.C01.05_A_126

ATTTATATCGCTGGATTTATAGCTACTGCAAATCAGCAA

>NL01B.C01.05_A_127

GGGTTTTATAACGTAGTCGTTGTACCGTCTGAAAATAACA

>NL01B.C01.05_A_128

TTTTTTCGTAAAACTTGTGCAATATCCTCTGAAACATTTCG

>NL01B.C01.05_A_129

TTAATGTAAGATTTTAAAAAATGACAAATATAAATCATTC

>NL01B.C01.05_A_130

ATAAACGGTGGCGATTATGCATATATTCGCTCAACATTTTCTC

>NL01B.C01.05_A_131

TCAATTCTATAGTAGATTAGCAAAGTTTAACTGAAAAAAAGCATGAAG

>NL01B.C01.05_A_132

AGTTTGAAGTTGATTAAAAAAAGGAAGACGACTTATACG

>NL01B.C01.05_A_133

TGACTTAGTATGTGGTGGTGCATACATTTGTATTTCT

>NL01B.C01.05_A_134

ATATAGACTATTACCCTGAAACTTGTGCTGATAATATAAAGGGACT

>NL01B.C01.05_A_135

TCCATGTAGTATTTTCTAGATAGTTCGACAAACTGTTTCTC

>NL01B.C01.05_A_136

CAGTTAATAACCCAATTACCTATCTTAACGTACACTTCTT

>NL01B.C01.05_A_137

CCTGTCAGGAGCGTCGTAGTCATAGCAGACGGGGTTTATGT

>NL01B.C01.05_A_138

ACTTTATCACAAATATTGAAATCTTTGCAAACTTTCTCTC

>NL01B.C01.05_A_139

ATTCCTAGAGTCGGAAGATCTATAGAGATATTTGTAGTCGT

>NL01B.C01.05_A_140

ATTTATTAATTATAAAAATTATAACTTGCAAATTAATTATTTA

>NL01B.C01.05_A_141

AAAAACATAGTAGAAATGTTAATGACCAACAAAAGTGAAT

>NL01B.C01.05_A_142

ATAATGAGTATTTGAATGCGTTAGCTGAATTTGATAGAACTGG

>NL01B.C01.05_A_143

CAAACAAGAGAATATGTAAAAGAGTATGCAAATTGGTTA

>NL01B.C01.05_A_144

ATTCCTACAGCAAGGATATGACAAAAAGATTTCACATTATT

>NL01B.C01.05_A_145

ATTTCTTGCTATCTCAGTACCAACAAAATTATGGCAACGT

>NL01B.C01.05_A_146

ACATAAATTAACTTAATGATATCGTCTTCTCCGACTACGT

>NL01B.C01.05_A_147

ATAATTTCTAATCTTAAGTCGCTGATTACGTCCATCCAGACT

>NL01B.C01.05_A_148

CCAAAATAACTACATGTACTTGGTGTTATAAATCCTACAGT

>NL01B.C01.06_A_1

AATATAAACCCTATGCGAACGTTTTTTATTTTTTAACTTTA

>NL01B.C01.06_A_2

CATAGCAAATTTTCGGTGCTATTAGATTGATTGGATATGTTACT

>NL01B.C01.06_A_3

TCGTATGTATATTTCTCATAAAGTTTTCTATCATTTGCAGTATT

>NL01B.C01.06_A_4

TCTATAAGCATTCCAAAAAAATAACCAAAGAAAATAATTAA

>NL01B.C01.06_A_5

AAATCTAAGTATTTTTCTTCCTCCTTAAGCTCATATTTATCAGT

>NL01B.C01.06_A_6

GCTGAATTCTCCGCCAGGATGACGCTGAAGGTAAATGGGT

>NL01B.C01.06_A_7

ACTCTTCTAGTGAGGGGAGGAAGCCAGACCCCTAACAAA

>NL01B.C01.06_A_8

ACTTGTACTAAGATATGATCGGATGGCGATGTTATCATA

>NL01B.C01.06_A_9

TCCGTGGTGCCCAACATGTATTACGGTTACAGTGTGTTCAA

>NL01B.C01.06_A_10

TATAATAAATTTTCGTTATTTTGTGATATATTGTTTAGTGA

>NL01B.C01.06_A_11

TTAATTGCTGACGATTTAAATAAACTCATTGACGCAATTGGT

>NL01B.C01.06_A_12

AAAAAACTTGCGATTTGCCTAAGACTATAGCCCTTACG

>NL01B.C01.06_A_13

GAGGGGAAGGTCTTGAGTACACAAGAAATCAGGGAGATGTATAA

>NL01B.C01.06_A_14

TCTTCACTGTCACGTACAGCTTTGAATTCTATAGTAGATTAG

>NL01B.C01.06_A_15

AAGGGCTAGTAGTTGACTTGGGTGTACTAGTGTTACCAT

>NL01B.C01.06_A_16

ATCTATGTTTGATAATATCCATTTTATCAAATTCGTCTAT

>NL01B.C01.06_A_17

AATTTGTTATATTCTATATGCTTACCTTGGACTTGTTCTAACTG

>NL01B.C01.06_A_18

AATATATTTAAGAGTTCGATCGAGGACTTGCTCCACGCTAA

>NL01B.C01.06_A_19

TTTTCAGTTTCTATATTAATTGTATTTGCTGAGGTATAAT

>NL01B.C01.06_A_20

TTTTTCTCCTCAATATATCATTTGCAAAAACTTATATTT

>NL01B.C01.06_A_21

TATCCTCAATTTTGAAAGTTCTTTCGGTAGTTATTCTCCTGGC

>NL01B.C01.06_A_22

TCAGAAATAAAGTCAATATATGGTGGAAAATGTCCACAT

>NL01B.C01.06_A_23

AATTAAAATCAGTGTCGAGATAAACCCAAAAACGGGCAGG

>NL01B.C01.06_A_24

GCTGGCGACATTAACACACTGGTTTGCTGACATTCTCCAG

>NL01B.C01.06_A_25

TTAGGAGGATAAGCTCTAGTTCTAACCCAATTTATTGT

>NL01B.C01.06_A_26

CTCGGTTCCGCACCTACCACTGCGAAGAGGTATTCCTTTTT

>NL01B.C01.06_A_27

ATTCCTACAGCAAGGATATGACAAAAAGATTTCACATTATT

>NL01B.C01.06_A_28

AGTCTTATTGAATTTGGTGAATCTAATAACAAAGATTTTA

>NL01B.C01.06_A_29

ATTTAGTATACGATGCTCGAAACAATATGTACGGTGCAG

>NL01B.C01.06_A_30

AAGAACAAATGTAAGGATGACAAAAGCTATGTATAATAGCTAA

>NL01B.C01.06_A_31

ATTTTTCGACTTTTTGATGATATATGGCAGTTTTAGGTTTCCTA

>NL01B.C01.06_A_32

TGATTGAAATACCCAAATGCCACACCGTCAGAGTTATTAGCGA

>NL01B.C01.06_A_33

CGGGAATTACGCACCGCCCCTCCTCGAACAAATGCTG

>NL01B.C01.06_A_34

ACAATATCACCGTAAAGCGTAATACATCCCGCATCACCGG

>NL01B.C01.06_A_35

GAAGGCGTGGGCGTTCAAATAGATCACGGGGGTACCGC

>NL01B.C01.06_A_36

GATATAGATGAAATATTCAAGTGATACTATGCTAAAAGATTA

>NL01B.C01.06_A_37

TCTTCACTGTCACGTACAGCTTTGAATATTCAATATGCTTA

>NL01B.C01.06_A_38

AACTGTCTAATTTCATAATATACTAATTATATAATATGCTAA

>NL01B.C01.06_A_39

AAGTAAAACGCGAATTCAGTAGTAGTGTTTTTGCATAATGTCTTT

>NL01B.C01.06_A_40

AAACAATGAGTATTCTTTGCCTTACAAATATAGAGAGCATACCTA

>NL01B.C01.06_A_41

AACCGAGGAGCGGGAAAGTGGTCAGGAAAAGGATAGAGTACA

>NL01B.C01.06_A_42

TGAGCAGTTATCGGTATATTGTTCTTCAGTGCTATAGCAATCT

>NL01B.C01.06_A_43

AATTTGATCTTTTAGATGAAGACACGCAGCAGAAGAAGGT

>NL01B.C01.06_A_44

ATAGTATTTCAGCTATCCTAACGGTTAATAGTAAAGCGGTG

>NL01B.C01.06_A_45

TCGTAATCATCACCGTATTCGTATAACAGTATAACGGTTCTT

>NL01B.C01.06_A_46

TAACTTAGCTACATTTAAATGTAGAATATGGTCATGAA

>NL01B.C01.06_A_47

ACAACGCGATGTAGAGCGTTAAGAAAGCAACAGTATATG

>NL01B.C01.06_A_48

ACTTAATAAAAAGTAGGATTGACTCAATGAGAGAACTCA

>NL01B.C01.06_A_49

TTTCTTCTTTGGTCTTCTCTTTGTTAGTTAAGAAGTCGT

>NL01B.C01.06_A_50

GTTTTTACATAGTTATACTTGGAGGTGAAAAAGATGAG

>NL01B.C01.06_A_51

ACAGCTACACCACCCAATGCTACTACTGGAGAAGGAAATGG

>NL01B.C01.06_A_52

CCCTTTAGGGCGGGGGTTCCCCGAGGTCTCAGGCGTTACACCCCT

>NL01B.C01.06_A_53

CTTCAGCATCCTTTTGCAAATCATTAAATGTTCTCCATTC

>NL01B.C01.06_A_54

TTTGACTTTTACCCATTTTTACACCCCTTACTTAAAAGATAAA

>NL01B.C01.06_A_55

GCAAGGGAGGTGTTGCTGAGAACAAATATGAATATTCATAATAACCC

>NL01B.C01.06_A_56

TATTTTTTACAAATTTTTAATAATATGCAAAGCATGGA

>NL01B.C01.06_A_57

GATGAAAATACTATTGAGAAAAAATGCACGGCAAAATACTAT

>NL01B.C01.06_A_58

CATTTTCTAGTAGCTATAAGGAATACTATGACAATGATCAAGA

>NL01B.C01.06_A_59

AATTCTCTGGTATTATCATCAATATCATTAGCACTCATT

>NL01B.C01.06_A_60

TAGATAAAATATTAGAAGTTGTAGATAAATTAGATGATTATAA

>NL01B.C01.06_A_61

ACAAGAAGTGAAAAAGGAGTTCGGTGGACTGGTAGAAGATACT

>NL01B.C01.06_A_62

TTCTGTTGGGCTAACTGTAAGGCTTGTTGATATTTACCCT

>NL01B.C01.06_A_63

TAATGATGTCAAAATCGGAGTCGTTGTCGTTGTTGTAG

>NL01B.C01.06_A_64

TTAATCATGTTAACTAACGCCGGTCCACCGCCAAGTCTATT

>NL01B.C01.06_A_65

AATAATGTTACACAATTAGATTTAGTAACAATTTTAACAGCT

>NL01B.C01.06_A_66

TTGTCAAGTTGTATTGGTTCAAATCCGACCTTTGTGG

>NL01B.C01.06_A_67

TAATCTACCGAAGCCAGCTAACTGTGATATTTGGATTA

>NL01B.C01.06_A_68

TTAATAGTTTCAAAATGTTTACAACTAGATATCAATACCAAT

>NL01B.C01.06_A_69

GTCACTTTTCACTTTACCTTCTTTGAAATTTGTTATTTT

>NL01B.C01.06_A_70

TTTCAATTCTATAGTAGATTAGCTGCTTAACGGGCAGG

>NL01B.C01.06_A_71

TAATATACTGATGAAGATGGAGGCTTACGGCTGGATA

>NL01B.C01.06_A_72

ATACCCTTATTCGGTAACAAAGCGTAATGGCGTTGATAATG

>NL01B.C01.06_A_73

CTTCCATTGCAGAAGCTAAGGCTACAGCGGAAACGTTTT

>NL01B.C01.06_A_74

TATTTTTGAGCAAATTCATGTGCTAACTTAATTATTTGTT

>NL01B.C01.06_A_75

ATTATTGTAATTGTTTTATTCTCAACTTGCAATTTTGAATA

>NL01B.C01.06_A_76

GAGCATGGAATTGTGGTAATACCGTTGGATATGGGA

>NL01B.C01.06_A_77

AGCAAATTATAGATGTCTTGTGTTGTTCTCATCTGCTTTTCT

>NL01B.C01.06_A_78

ATAAACGGTGGCGATTATGCATATATTCGCTCAACATTTTCTT

>NL01B.C01.06_A_79

GAAATATCTCAACTGGTTCTCCATCTACTACAGTAAGT

>NL01B.C01.06_A_80

AGATATAATCGAGAAGAACTAGAAAATGGATTATTATCAA

>NL01B.C01.06_A_81

AATCAAATTGGTAATACACTTACACAAAATTTTGATAATTTTTA

>NL01B.C01.06_A_82

GAGACCGGGAGAAGTAAACAAAGAGTTTATTTAAAACAATA

>NL01B.C01.06_A_83

TGATGCAAAAACTATCTGATACGATAGCCAACCTTAGTT

>NL01B.C01.06_A_84

GGACGAAAGGGGCGAAAGAGAGAATAACTCAAACAGCTGTTT

>NL01B.C01.06_A_85

ATACGGATACGGCTGGAATTTGCTATCGAATAATGTGTCT

>NL01B.C01.06_A_86

GTAGGAAAATTAAAGACGAAAGTGTATTTCTGTGTTGCA

>NL01B.C01.06_A_87

AAATCAACACAAATGCTTCTTATCGTTCTATTATAGAA

>NL01B.C01.06_A_88

CCAAATTCGAAGTAGCTACACCTTGAGCTATTATATTCC

>NL01B.C01.06_A_89

ACCGGGTTGCTGATGTAAAGGGAGTCTTTAATGTATGTTT

>NL01B.C01.06_A_90

AAACGCATTAGACAATAATGAGTATGTTGTCACATCAATCGCA

>NL01B.C01.06_A_91

TACTTCTCAATAAATTAGTGGGTTTAAAAAACTATTTAAGTTT

>NL01B.C01.06_A_92

CTTTGTAGAACTCCTCTTCATTTTTCTTCAACCCGTCAA

>NL01B.C01.06_A_93

GTGATGATTGTACCGGGCATTATATACTCTTCTGTTACTA

>NL01B.C01.06_A_94

AACTAACTGCCATAAGGAGTTTCCATATCGCGATTCT

>NL01B.C01.06_A_95

ACTGCAGTAGTGTTTCCACTTATCGTGGTGTTTCCAGTCT

>NL01B.C01.06_A_96

ATTTATGCAGTTCTGCTAAATCAAAAGGATTATCAACATCT

>NL01B.C01.06_A_97

AGGCATATCTGCTCGTTGATGATATTGGGATGGTCATGTATAT

>NL01B.C01.06_A_98

CCATATAGTGTATATTGGGTTGATACTGCATGGAGTAAATACAAT

>NL01B.C01.06_A_99

CTTTCTGTAAGTACATATTCCCCATTTTTTTCTTCAATTAACCCGT

>NL01B.C01.06_A_100

TTGACACTTCGCCAAGTGCAACGTTTCTTGTAACAACAG

>NL01B.C01.06_A_101

CTAATGCAGGCCTTTCGAGCCTGTGACCCGGGTTCAAATCC

>NL01B.C01.06_A_102

AGTTCTATAAAATTTGGTATAGTTTCAGGTTTGACGAAA

>NL01B.C01.06_A_103

CATCTCCTATATCCGTAACATATAACGAAACGACACTAACTTT

>NL01B.C01.06_A_104

CGTTTATTAAATAATCATTAATTTTCAACACTTTTAATTCATTTTC

>NL01B.C01.06_A_105

AGTTGGACAAAGTTTCAACATAAATTTATCAAATTATCA

>NL01B.C01.06_A_106

CATTTACTATACTCTTTAACATTAACTTGAATATGGCGTCGTCT

>NL01B.C01.06_A_107

ACACACGGTGCAGTATCGTATGCTTCTAGAATTAATTT

>NL01B.C01.06_A_108

ATACTGAAGGGTAGTACTATATAATTACCAAAGAGCAGACC

>NL01B.C01.06_A_109

GGAACTAACGGTATCATAGGAGCGTCTTTCGCATTTTAT

>NL01B.C01.06_A_110

ATTGCGAAATAATAAGTATATTGTTTATAAAAATCTATAG

>NL01B.C01.06_A_111

AAATTCTTTTCACAACCTTCACAATCCATTACTAAAATATCAG

>NL01B.C01.06_A_112

TGAAAATTTTGCGTATATTCCCTAATTGAAAAAATATGGGAAT

>NL01B.C01.06_A_113

AATAGATACAAGTATTCAATAGTTAACCACGATGGGACAC

>NL01B.C01.06_A_114

ATCCTGTTCCCAAAGGCTACCCTAAGTTTTATTAGGTCGTCCC

>NL01B.C01.06_A_115

TCTTGTGGTATTGCATTTAGCTGAACTATTATTCTTTTTATT

>NL01B.C01.06_A_116

ATAGTATAGAACACATTGTGTCACCGCATTTCAGTACACC

>NL01B.C01.06_A_117

AATTGCCTTGATTATACGTTAAATTAAATTGGTATAGT

>NL01B.C01.06_A_118

GAAATTTTTAGACCTTCAATCTCACCTTTTATCTCAACTTCA

>NL01B.C01.06_A_119

TTATCATATTGTGCATAACTCGCGGATAAATCTGGTCTCATTC

>NL01B.C01.06_A_120

TCAAGTTTGTTGGCTTCACCATTCCAGCGTTTAACTTCTTCCA

>NL01B.C01.06_A_121

TCATTATTTCTAAAACAACCATTAAATGTTAAACCAGATCT

>NL01B.C01.06_A_122

ATTTAGTGATAACTGCACAAGGAAAAACTGTAAAAGAA

>NL01B.C01.06_A_123

GTTCTAATTGAATTGAAAAACTAACATTAATTTCTTGTTG

>NL01B.C01.06_A_124

ATCTATCATACCCTCATTCTCATAGTATACATAGTATGAGACAA

>NL01B.C01.06_A_125

TGTAGGGGATGAAGACGTCAAACTACTGGCGTTAATGGCGTTTA

>NL01B.C01.06_A_126

CCATAGCTCTTCTATCTCGTCGCTGTAATAGGTAAGCATCA

>NL01B.C01.06_A_127

AGATAACTTTTATTTGATTTTCTGGAATTCCTAATTTTCTAAGTT

>NL01B.C01.06_A_128

GGAGTAATAACTGTAAATAATGGAGTATCACTTATAAATAC

>NL01B.C01.06_A_129

GCTATATAAATTGGATCAAGTTCAGTTAGCACTGGCTGACCA

>NL01B.C01.06_A_130

TGACATTATAGGTTTGGGTTTGTGTGGTTTGGGATTCG

>NL01B.C01.06_A_131

TAACTTTCGGTGCGTTCATAGCAAGCCCGTTAAGCA

>NL01B.C01.06_A_132

TAGGTAATTCTGAATTTTTTAAAACTATAATTGAATGAGG

>NL01B.C01.06_A_133

GTGGGCATAATTCACATGCATGGCAATCTCAACATCAACAAA

>NL01B.C01.06_A_134

CAGTTAATAACCCAATTACCTATCTTAACGTACACTTCTT

>NL01B.C01.06_A_135

ACTTTATCACAAATATTGAAATCTTTGCAAACTTTCTCTC

>NL01B.C01.06_A_136

CCTCCTGCTGCTACGCTAGTTCCGGGCGCCGGATAAGCTGG

>NL01B.C01.06_A_137

TTTAATGATTTCTATTTTGCGACGTTCGATGATATGTCTTC

>NL01B.C01.06_A_138

TTCAAAGTGGGAAAAGTTCCAATTAATTTAGGAAATGCAGA

>NL01B.C01.06_A_139

GTGAGAATAAAACAATTACAATAATCTTTCAATTCTATAGTAGAT

>NL01B.C01.06_A_140

ATTTGTAAATTAGAACCATCATCTAATACAGCCAAAGCT

>NL01B.C01.06_A_141

GAAAGATCACTATTCCTACCCCCATCGCTTCTAACATTG

>NL01B.C01.06_A_142

CAAAGAAATATTAGATGAACTTGGAAAACAAGCTAAAGAAAA

>NL01B.C01.06_A_143

TTTCTTGGTGTGAATTTTACATATTCATTTAAAATATCAG

>NL01B.C01.06_A_144

TTTCTCTTAGTCGTCCTATGTTTGTATGTATCTGTGATGG

>NL01B.C01.06_A_145

GCACTTAGACAGCTTGGTAGACCACTTCCACTCGAATAT

>NL01B.C01.06_A_146

ATTTCTTGCTATCTCAGTACCAACAAAATTATGGCAACGT

>NL01B.C01.06_A_147

TTAAAGACGATGAAGACCTTGGTGGCTAAGTGGTATAACA

>NL01B.C01.06_A_148

ATTACTAATAATCCATTACTTGTAGTAATACCTATGAATAC

>NL01B.C01.06_A_149

CCTCATAAATTAGAATTGCTGAGGCACTTGTTAAGCTTAAGCT

>NL01B.C01.06_A_150

TAAATGCATTGAATAATTTTTCGGCCTCATCTCTCTCAA

>NL01B.C01.07_A_1

TTAACCTATCGCTTTTAATCCACGTCCATCCATCTTCC

>NL01B.C01.07_A_2

ATTCCTGATTGAATTAACACCAACAATCGTCACTAATTCAC

>NL01B.C01.07_A_3

AGCTATTAATAAGCTTAAAATGGATAAGAAGGCAGCTAAG

>NL01B.C01.07_A_4

TTCAGGTACAGGTTTAAGATAGTATGAGAAGAAATAAAG

>NL01B.C01.07_A_5

AAACATAATAAAAATGCCTACCAAAAACTCCTTTATTTATTT

>NL01B.C01.07_A_6

AACCCACACTGGTACGCCAAATACCTTTTTGTTTAGGT

>NL01B.C01.07_A_7

TGTACAATAATTTATACATATTTGATAACGTCGCTAATAAGTGGAT

>NL01B.C01.07_A_8

TTTGATACCGTTATGATAGACTGAACCAGTTAGTATGCCA

>NL01B.C01.07_A_9

GTTTAGAAGGAGAAAGATTGACAATTCAAAGAAGAAAGGGACT

>NL01B.C01.07_A_10

GATATTTAAATTTTCTGCATAACCAGTTAGGAGGAAAGCGA

>NL01B.C01.07_A_11

CAGCATCATGGATGTCTTTATCATGAATATAGTCCTTTA

>NL01B.C01.07_A_12

AAGAAAAACTGTTTTCTTCAAATGGACCTCCCAAAATAAC

>NL01B.C01.07_A_13

TTCTTTTTATGTGAAATTTGACATAATTATCAAGCTTTAGA

>NL01B.C01.07_A_14

GGTAGTCTCATGCACCATAAACTATTACTGTAATCAACCC

>NL01B.C01.07_A_15

CATTCGAGGAATGTTTAAGTCTGATAAATCCTCAAGGGTTA

>NL01B.C01.07_A_16

CAACATTGTATATTCCTTTATTATATATTATGATATTATAGCCA

>NL01B.C01.07_A_17

GCTACTGTGGTATATAGCATGACAACTGGGTTACCATA

>NL01B.C01.07_A_18

ACTGTAAATTAAACGTATGTTAGGGCCCCCAGCAGTAAAGAG

>NL01B.C01.07_A_19

TTCACAATCGACTTTAAGTACAGATCCAGAAGGAATATTG

>NL01B.C01.07_A_20

ACTATAATATTATAATTTATAATTTTGTCAAACCAAAACTCC

>NL01B.C01.07_A_21

GTCACAGTTGTATCTTTAATACTCTCAACAGCTACCACAGTCGA

>NL01B.C01.07_A_22

GTATCTGTGTAGAGGACTTCATGGTCAGCACCGATGTACTTA

>NL01B.C01.07_A_23

AAATTAGTTAAGGCACGGCTTAAAAGTGATCTTAAAAAT

>NL01B.C01.07_A_24

ACTTTAAGTAGGTGAAGGCGAGTGGCAAAAGAAAAACTAAG

>NL01B.C01.07_A_25

TATTTGAACCCGTTAAGGTTTGAAGTTGCCATTAGGTCGC

>NL01B.C01.07_A_26

GATTTATGCTAATACACCTATGGTTGAAAGCACAAGTTGG

>NL01B.C01.07_A_27

ACTAACAGCTTAAGTTTTTCAGTAAACGGATTAGATGACATA

>NL01B.C01.07_A_28

TAACGATAAAATTTTAACTTTGTGTTATTGGCTTTATTACG

>NL01B.C01.07_A_29

ACTGAATATCTTTCTAAGTAATCCGTTAGTCGCTATTAGTTC

>NL01B.C01.07_A_30

TGAAGGGGAACCTACTTTATATATGTGTGCTATTTCT

>NL01B.C01.07_A_31

ATTGTCACTTACACGCAGTACATTACGCGGTGAGGCAGA

>NL01B.C01.07_A_32

ATGCTTATAAACGTGAATTGGCAACTATAATACGGGGCG

>NL01B.C01.07_A_33

ACTACTAGTCCCATAAGTATATCCTGGAGTACCTTCTGG

>NL01B.C01.07_A_34

TATCATAAACCCAATCGAAGCAGGAATAGGTCACTTAGAA

>NL01B.C01.07_A_35

GCTATGTTGTTGGTGAAGTTGTACATCTATATAATCAAA

>NL01B.C01.07_A_36

AGCTCTGGCAAGTCCGCAGTGGTTGCTATTTCCCCACCA

>NL01B.C01.07_A_37

AAAACAGCTGTTTTAAGGAGAAAACATTAAAAAATGGGCT

>NL01B.C01.07_A_38

TTCTCAGTATCAAGCACTCCAACCTATTGCACAGCAATT

>NL01B.C01.07_A_39

TGGTTGGTTCATTATTCAGTTCCGTCTTTAAACAGCAGTG

>NL01B.C01.07_A_40

TTCTTTTAAAAACGAATTGGGAAAAGATAATGACATGGGAA

>NL01B.C01.07_A_41

AATATATCACTAAGCTTCTTAACATCTAATAACATCTCT

>NL01B.C01.07_A_42

GTTGAAAATTCCCATTAGAATGGGGATGCTAATAAACGTA

>NL01B.C01.07_A_43

TGAACTTGTAAAAAAAGGCTATTCAATAGCTCAAATTGCAAA

>NL01B.C01.07_A_44

TGTGAATAACTAAATACATATAGTTTCTGATGTTTAACAA

>NL01B.C01.07_A_45

TCTCATATGGTATAATCGTAAAGCAAGCAAGATCTGCCTT

>NL01B.C01.07_A_46

TTTTTGTAAGCGAATTCGCCCTTAAGTACTTTCTCAACAA

>NL01B.C01.07_A_47

TTGAAAAGGTGTCATTAAACTTCATATATAGGAATGAGACGA

>NL01B.C01.07_A_48

CTTAATTCCACCATTTTTTATCCCCCTGAGTATACTCCT

>NL01B.C01.07_A_49

ATAGCTTCACACCAGCAATGTTGGCAACAACACAGGCAACTG

>NL01B.C01.07_A_50

TTTTCAAATGTTGCAACTACATCGTCAATAAGTTTTGGCGT

>NL01B.C01.07_A_51

TTTTGTGCATGTAGAAGAGGAGTAAATGGATATGGTTGAAAGTTTT

>NL01B.C01.07_A_52

GCATTCTCCCTACCACCGTCTTGGCGTTAGTTACTAAAT

>NL01B.C01.07_A_53

AAGTTATACTCTGGGCGGTATCTATACAACTCTATTGCTT

>NL01B.C01.07_A_54

CTATCAATCCAAGTCTCATTTTTCCATGTCTTTTTCCTA

>NL01B.C01.07_A_55

TCTGGTATGCCCTCATACGATAATATACGATAACAAAATAT

>NL01B.C01.07_A_56

GAGCTTCGTCCTCGTTTATGTCGCAATACTCACATATACGTT

>NL01B.C01.07_A_57

TCTTGTGGTTCTCCTTCCTCTCCCTCCTCTTGATTTTCTCC

>NL01B.C01.07_A_58

TCAATTTTCCATTTTTCAGGTTCCATAATTCCATTTTTGG

>NL01B.C01.07_A_59

TCACTTTCTCAAATTGAAACTGCAATAAAGTATAACTTTCC

>NL01B.C01.07_A_60

GTTACGTTTACGTTTCTGTGCAGCAAGGTAATTCGTTTAG

>NL01B.C01.07_A_61

TTTTTAATCGCGTTAACCAAAGGTTCTGTATTAAATT

>NL01B.C01.07_A_62

TAATAATCAAACTGCTAGACTTTTGCAAAGAGCAGTTGAACA

>NL01B.C01.07_A_63

GTCACCACGTCTACTTCCCCGGCCTTGAGGCCGGCTTCCTTA

>NL01B.C01.07_A_64

TCTCACCAATACATACTATGTCCTCAAAAGTATTTAAATTTT

>NL01B.C01.07_A_65

TATTCAGATACCCCATACGATGAGTTGTATATTACAGTAAAAT

>NL01B.C01.07_A_66

ACTGTTGCCATTTCACAAAACTCTCCCTATGTAATTA

>NL01B.C01.07_A_67

GAAGACAATGTTATACCGATACTTGATACAGATAGAGGTAT

>NL01B.C01.07_A_68

ATAGTAGTGATAAATAATGCATATGTATTAAATAATAAAT

>NL01B.C01.07_A_69

TCATTTTTTCACCCGCATGTTCATTTTCTCATTCCCCCTT

>NL01B.C01.07_A_70

TATCTATCCCTGTAGACGAGAAGAAGCAAGTAAGTGTGAA

>NL01B.C01.07_A_71

TCTCTACTATTATGTCTGAAATGTAAGATGGATCCGAATTT

>NL01B.C01.07_A_72

AACATTAAAATCCACTAATCCTACAATCCGAGAAAATTTC

>NL01B.C01.07_A_73

AAATATCTTCGATTTTCTGTCCTTCTCCACCTTCTGATTCCT

>NL01B.C01.07_A_74

TAAATAAACCCGAGTTGGTTCTTTCGTCGCCGACAAACGT

>NL01B.C01.07_A_75

TTTTTCCTGTTAACAGCAAAAGCTAGTCT

>NL01B.C01.07_A_76

AACGAAAAAGGTGTGAGAGGTGCTATTGCGACAACCTTACCG

>NL01B.C01.07_A_77

CTCTGACTTCGTTAAGTGAATATGTCGACCATAGCGGGTT

>NL01B.C01.07_A_78

TATTGTGAAGCTAGAATTACATCTCTTATATTTACTGGTCTA

>NL01B.C01.07_A_79

CTTTTCCAGATAGGACCGTTTGCCCTATCCTAAAACTAGTAG

>NL01B.C01.07_A_80

CCCCTCACGGGCGTGGCATCACTGTTCAGTGCCCGCCGTCAGC

>NL01B.C01.07_A_81

TCACCACCTTTCATCCCTGTAATATGTATTACCGGGCTGA

>NL01B.C01.07_A_82

GTTGGGCAGAAAAGTTTAAATACTAGTTTTTGCATAGTTA

>NL01B.C01.07_A_83

TTTTCACAACATCAGCCTCTGAGATATCGAAATATTCCGC

>NL01B.C01.07_A_84

AAATCAGATAGTATGAAGATCTGTTTTATGTCCTTTAATCC

>NL01B.C01.07_A_85

TAAAATCTCTTATCCCCTACTTTTGCAATTACTCTTTGTCCTAC

>NL01B.C01.07_A_86

TATATTTAAATTTTTCTCTCTCTTTTTACATCTCTATCTT

>NL01B.C01.07_A_87

AACGAAGTCAAAATTTCGGAAGCTAAATTGCTAATTGGGCC

>NL01B.C01.07_A_88

TTATTTTGCGTAAAATTACGTTAAGAAAAATAAAAATAT

>NL01B.C01.07_A_89

CTGCATCACAGTACGCTACAGAAGCATCACGTTATTACAA

>NL01B.C01.07_A_90

AGACCAACTGGCGATAAGTCAATTTCTACCTCTGGAAGTA

>NL01B.C01.07_A_91

GTATTGGCAGTGGTACAATACCGATGATTGCGTCGTA

>NL01B.C01.07_A_92

TAAGCTGTAAGAGAAGAGGGTAAAAGACTCTTGAGCAT

>NL01B.C01.07_A_93

TATCTTTCGATCCTTACTACTGGTACTTTAAAGATGAATTC

>NL01B.C01.07_A_94

TTTACCCTGTATGTCCCTAGCGGCAAAAGAGGATATATA

>NL01B.C01.07_A_95

CATGTACATATCAATATAGTCTTATCATCATCAGGCAAGT

>NL01B.C01.07_A_96

ATCAGTTAGATTCAAGTTGTTGCTTGATCTTGATTACGATTA

>NL01B.C01.07_A_97

GTCAGAAGTCCTGTTATTACAGCTGTTTTGACGTCTTCATT

>NL01B.C01.07_A_98

ATAACGACCTGGGCGGTTTGTATGGCATTCAGATATCATGG

>NL01B.C01.07_A_99

AATAAACTATATTGAAATTTCAAACCGCGGAACTGGAA

>NL01B.C01.07_A_100

TCGTCACCAACGCCACGGGATTATATTGTGTCTGCTGGA

>NL01B.C01.07_A_101

ACTATTAATAGTATGAAACCTATGATGAACGAGAGTGTTGCAA

>NL01B.C01.07_A_102

TACTTATTTTTTCATCTTTGTAGCTTTCTAGTATATCGTA

>NL01B.C01.07_A_103

ATTACAGCGACGAGATAGAAGAGCTATGGAGGGAATTAGT

>NL01B.C01.07_A_104

GCAAAACTCTCCTTTCTTTATGGTAGCATTTTACACTGG

>NL01B.C01.07_A_105

TTTACTAATGGAGAACAAGCACAAGAAGCCGGGACAGCAATACAA

>NL01B.C01.07_A_106

AGGTGAAGAGAAAGCCAGCAGAATGCTTAACCAAAAAGCTCA

>NL01B.C01.07_A_107

AAAGTGTAGTACCCATGTTTTAACGTCTTTATTATGTTAGA

>NL01B.C01.07_A_108

AGCAAGTATTATGATGTTAGCAATATAATAACGTACAGAGA

>NL01B.C01.07_A_109

TTGACTCGAGAACGGGCATTCCGAATCCTTCAGTTCC

>NL01B.C01.07_A_110

AATGGTATATTGGTGCTTATTCCGTTTCCAATAAATGTT

>NL01B.C01.07_A_111

GTTATTGTATAAGTTTTCCATGTTAAACCAGTGGAATTAAG

>NL01B.C01.07_A_112

AAGAGTAATGACGTGAAAAACTTATGTGATAGTATCGATATACCA

>NL01B.C01.07_A_113

GACTTTCTTAATTTCTATCAGAGAGGCCCAGCGTTTTAGCC

>NL01B.C01.07_A_114

CCTAACCCACCGATACATAAACCAGAACCAATCGTTAA

>NL01B.C01.07_A_115

TCACCGCTTTACTCAGGTCCATCCATAGAAACTGTAAGAATAA

>NL01B.C01.07_A_116

AGGTTAACCCCACTCCCACCCTCTACATAATTCCTGAACC

>NL01B.C01.07_A_117

GGTGCACCAGCGTCACAATGGTTAGACCCACCAGTCCTTCT

>NL01B.C01.07_A_118

ACTCAGAACGTTCCGCCTCCAGGTAATATCGTTGCTAA

>NL01B.C01.07_A_119

AAAAGGACTGGTTGATCAGCGATTGTGAACATTTCAAAT

>NL01B.C01.07_A_120

TATTTTTAAATTATTCGCCCCGTTATTTCCTTTGCTTTGC

>NL01B.C01.07_A_121

CATGAACTACTAGACCTGGTTCGAATTAACGTAATCTTTTTT

>NL01B.C01.07_A_122

TATTTCTAACTCTGTTGCTCACATGACTGAATTTGTCCATTTA

>NL01B.C01.07_A_123

AAGTTATACTTTTATAAATATATAAATATGTCATATTATAAGT

>NL01B.C01.07_A_124

TTGAAGAAGGTATGAAGTTGTCGGGGAATACTACAGTTGA

>NL01B.C01.07_A_125

AGAGATATGCCACATCCCTGTGTTTTATCTATTTTACCC

>NL01B.C01.07_A_126

CCAGGTTCCTCCTTAGGACTTTAGGGTCTTTTCTCTGCT

>NL01B.C01.07_A_127

AAACCAACCGCAGTATTATTCCCAACAATACAATGTATTGT

>NL01B.C01.07_A_128

ATTATTTACGCTGGAAATCCATATGCATATACAATTAATAA

>NL01B.C01.07_A_129

GAAGTTTGTCCAAAAATTCCACTTTGGCCTAATTGTAGA

>NL01B.C01.07_A_130

GTAAATTATAAAATAATAACGCGAAAGAATTAATTTTCCCC

>NL01B.C01.07_A_131

TTCTAACGTTTTAGGAGTAAGTTCTACAGTAAAATTGAAA

>NL01B.C01.07_A_132

ATGTAAACGATATAATTACTGAGATTTTGGAGAATTATTCT

>NL01B.C01.07_A_133

GATAACTTCCCACTTAGGCTCGTCCAAGTTCATGAACCTC

>NL01B.C01.07_A_134

TAATAATTTACAACCACATAGCTAAGTTGTTAGACGAACT

>NL01B.C01.07_A_135

ATTATCGTTCATCAGCGTTGCGGTTTTGCATAACCTTGA

>NL01B.C01.07_A_136

GTTATTGGTTCACCTTTTGCATAACATGTTGAAAAAACTT

>NL01B.C01.07_A_137

TGTAGATTTATCGGAATTATACTCATATGCATCTAGTTCA

>NL01B.C01.07_A_138

GTGCCTCTGTTCTGCAGTCTACACAGAAAGCAAACTTTTAA

>NL01B.C01.07_A_139

TAACCCAGGTCGTAGACCCGAAAGGAAACGCTGTATACGAA

>NL01B.C01.07_A_140

AAATTTAACGACATGAAACAAAATGGTGATAACAATG

>NL01B.C01.07_A_141

GTTATCCATCAGCTCATGCCACCGTTTGATGAATTTACATCA

>NL01B.C01.07_A_142

AGGTGGATTCTCAAAAGGGTTCTCGTTATCATATGAAAA

>NL01B.C01.07_A_143

AATGCGATATCGCTAGCCTTCTGAAACACATTTTCCTTAG

>NL01B.C01.07_A_144

ATCATGATAAGCCTCCTAATTTTCTGTCTACCAAAGCCTTG

>NL01B.C01.07_A_145

TCTCGCTATTTCATTAGTAAATACACAAGGAGTATGATG

>NL01B.C01.07_A_146

GTAATTGTTTTCTTTTTTCCAAACCCATTTATTGAATTTACCG

>NL01B.C01.07_A_147

TTAGCCTCCCCGCTAATAATCGTCGTCTGACCCGTAT

>NL01B.C01.07_A_148

TTATTTAGACCTGTTTCAATTCGTGATGTAATTTTAG

>NL01B.C01.07_A_149

ATTTATACTTTTTGCAGAATGGCATTTTAAGTTTTCATAATA

>NL01B.C01.07_A_150

TAGTATTTGCTTAGGCCCCGGCGCTGTAGAATGTAAGGCAGT

>NL01B.C01.07_A_151

GTTGGTGCAATTGAACCACTCGTGAAAACAGATGATAAA

>NL01B.C01.07_A_152

AAAAAATGTTGCCAAATCGCCGAGATTCAGCAGGAAACTCAT

>NL01B.C01.07_A_153

ATCTATGCTATAAAATATACTAGTGAAGTCATTTGCCAAAA

>NL01B.C01.07_A_154

GGCATAATTCACATGCATGGCAATCTGAACATCAACAAACA

>NL01B.C01.07_A_155

ACAATCGTCACTAATTCACAGTCACAAACCTCAGCTTC

>NL01B.C01.07_A_156

GTCATTATCAGGTAAGTTGGTAATGGTTTTTTCCACAGTCTG

>NL01B.C01.07_A_157

TTTAATAAATGTAAAGCCTAAGCCACAGCCAACTACTTCCC

>NL01B.C01.07_A_158

ACAATTCATATTGGGAGATGACCACAAGCGTGTTTACT

>NL01B.C01.07_A_159

TATAGATTGAGGGCTGAACTCATTAGGTGGGAACGT

>NL01B.C01.07_A_160

GTATATTTTCTAGTCTGGTTATCTTGAGGTCATATAT

>NL01B.C01.07_A_161

TTAAATTCGCAATTTTAGCATAAATAATTATTTGCTCCTGG

>NL01B.C01.07_A_162

GAATCTACTGAACACTTTGCGAAATCAGCTGAAAGTTTT

>NL01B.C01.07_A_163

ACTTTCTGCTTCTTGTTGCCCTTTTTTTATGTCTTCAAG

>NL01B.C01.07_A_164

TTCCCTTACCTTTCACTTTCACGTATTTTTACATAAGCTAA

>NL01B.C01.07_A_165

TCGCATAGCATTTTTACGTTCTTATTTTTGATATCGAAA

>NL01B.C01.07_A_166

TGCAACCCATGCTCTTCTGCCATGTAAGTTGTTCCTATACT

>NL01B.C01.07_A_167

TTGCTTTGCCGTAAAACATATGTGTTCTTAAACTTTGTT

>NL01B.C01.07_A_168

GGGTTTTATAACGTAGTCGTTGTACCGTCTGAAAATAACA

>NL01B.C01.07_A_169

CGGGTTCAGGTGTCTCTAGTATTTCTTCTAATTTCTTTA

>NL01B.C01.07_A_170

GCTATACCACCAAACGCTACTACTACTGGAGGAGGAAA

>NL01B.C01.07_A_171

AGTTGTTTAACAAACTTAACGATTCGCCTCACGGTACTTACAA

>NL01B.C01.07_A_172

GTCAAAAGAGCCATAACTATACTTCCACTAATTTTCAATA

>NL01B.C01.07_A_173

TATCATAGTTCTAGAGATTCCCAATTTATCGTAAGGAA

>NL01B.C01.07_A_174

ATATAGACTATTACCCTGAAACTTGTGCTGATAATATAAAGGGACT

>NL01B.C01.07_A_175

AGTGATTTGCAAAATACTTATCTTGAATATGCAAGAAAT

>NL01B.C01.07_A_176

CGAAGTTATTCGCAAAGATATGCAAAATGTTCAGCTAAATT

>NL01B.C01.07_A_177

CTTGAAAAAACTGGAAAAGTGGTTGGAATAGACTTAGGAG

>NL01B.C01.07_A_178

ATTCTTAGTACCACAGGATTATACAAAAACACAGCACCT

>NL01B.C01.07_A_179

AGAATCTTTTGTTGTCTTTATGTTAGGATTGGTTATGTT

>NL01B.C01.07_A_180

TCTTCTCTAGTCGAGAATAATAAATAAACGCCTAATTCTATC

>NL01B.C01.07_A_181

AAATTATAGTTATTCATCTGGAAAAAGAACGCTTTCGTATAA

>NL01B.C01.07_A_182

GTATGCTTCTTGCACTACAGGCATATATTGCTGTTGGACTTC

>NL01B.C01.07_A_183

ATAAGTCCTTCGACTATCTCCTGCTGCTTTAGTAAGCTGT

>NL01B.C01.07_A_184

GAAAAGTATATACAGTGGGTTGACGTTAGAGAGGTAGTCAA

>NL01B.C01.07_A_185

CTAGGTTGTTGTTGCAGTTTTGCAACTACAAGTATAT

>NL01B.C01.07_A_186

TCAGGATATCCCTAACAGTACCACCAACAAGATATACGTTA

>NL01B.C01.07_A_187

AAGCAATATCTAAACTCAATAAGATACTATCTGCACACAGT

>NL01B.C01.07_A_188

GATATTTGTACAATATAAGTCATTATTCTTGTACCTCCTCCAA

>NL01B.C01.07_A_189

TATTCCTAGACGGAAAAAACCCGTACCTTTACTCAATGGCTAAAG

>NL01B.C01.07_A_190

ACTTTTTCCCCAGAAGGATATACATATTTTATAATAA

>NL01B.C01.07_A_191

TATCCATATCGATACTCCGAATAATTTCGGATTTTGTGGAAC

>NL01B.C01.07_A_192

CTCGTCTTCAGATTTCCTCACACATTCAACCGTATAAATGTCTA

>NL01B.C01.08_A_1

TATAGCGCCGATTGTTCCATATTTACCTATTTTAAGTA

>NL01B.C01.08_A_2

ACAATATAGGAAGTCGCATTGGGTCTTAACTGGAGAT

>NL01B.C01.08_A_3

TACGAAATAAATGACGAACAGTTAATCGAAATTATTAA

>NL01B.C01.08_A_4

CCTTATATATGCGTACTTGCTCCTCCTCTTTTGGAAGATT

>NL01B.C01.08_A_5

ATCCTCTCCTTAACAAAGATGTTGTATATTCTACCGATATATCTTA

>NL01B.C01.08_A_6

GTTGATATAAACGTCTGTGAAAAAGTAAAAATATATGATAG

>NL01B.C01.08_A_7

CTTGCTAACTTGTTTTGCAAAAAGTTTAAATACTTATTT

>NL01B.C01.08_A_8

TTTCTTCTCAGCAAGATAGTCAATAACACTGTTCAAGTACT

>NL01B.C01.08_A_9

AAACCAAATTCCAACAGACCCTGAAACAGACGTTTGCA

>NL01B.C01.08_A_10

ATATGCAGTCGATATTATTACAAGGACTGACCCCACAAA

>NL01B.C01.08_A_11

TTTTTGACCATGAGTTTTGTCATAATATTCCTCAACTTCAC

>NL01B.C01.08_A_12

TTAAGTGCAAATGCATTAGTGAGTTTAAAGAAATTGAT

>NL01B.C01.08_A_13

AATATCGTTATGAATTTGTCGTTTGGTAATGGTCGTTTGC

>NL01B.C01.08_A_14

TCTCATATGGTATAATCGTAAAGCAAGCAAGATCTGCCTT

>NL01B.C01.08_A_15

CTTATTATTCGTTGTATTTACGCTTCGTAACAACAGTATACT

>NL01B.C01.08_A_16

AATCAGGTATAACGACATAATGGTTGAATGCAGGCCTACAAG

>NL01B.C01.08_A_17

TAAAGAGTTGTCCATTTACAATATTTCCAATAATTGTATGT

>NL01B.C01.08_A_18

TACATATTATTTCTAGCATCATAGACTAAATTGGCATAAGTCGG

>NL01B.C01.08_A_19

AGTATGAGCTAGAAGTGGATCTTACAACTACTGTTTCTTT

>NL01B.C01.08_A_20

ATTATAACAATGATGTCATCTGGATTCATCCAGACCACTCAA

>NL01B.C01.08_A_21

TGGCTTAATGCTGTAGTTGATATGATAATGTTGTATTTTCC

>NL01B.C01.08_A_22

TTTAATAAATGTAAAGCCTAAGCCACAGCCAACTACTTCCC

>NL01B.C01.08_A_23

ATTACAATTGGTAAATTTTTGCCAGCAATAATAGATAAATT

>NL01B.C01.08_A_24

CTAGCATTGGGGGTTTTAGGGGGATACCCCCTAACTAGAC

>NL01B.C01.08_A_25

TAATTTTCTGATAAATCGGGATTAATACTTTATAATAGCTCTGAT

>NL01B.C01.08_A_26

AAGGAGTTGTGGAAACATCCAGTTGAAGGCCTTCTATAGT

>NL01B.C01.08_A_27

AAAATCACTATGCCATAGCGAAATCATTATTAGCTTAATTT

>NL01B.C01.08_A_28

TTCAGATATCCAATTTCAATAATTCATCCATTCTTCTAT

>NL01B.C01.08_A_29

TAATTATGGGGATACGGTTAACTTCCAACTATTATT

>NL01B.C01.08_A_30

GGCTATGTACTCGTAAACGGTGTGCGAATAAGGAGGAAAAT

>NL01B.C01.08_A_31

TAGATAAAATATTAGAAGTTGTAGATAAATTAGATGATTATAA

>NL01B.C01.08_A_32

CAAAACAATCCGTTTTCTAACTGCTTAATTGCGGAAGCCA

>NL01B.C01.08_A_33

TTTTCTCGAATTATTTCTTCAGAATATGGCACTATTTTTATCCATTC

>NL01B.C01.08_A_34

ACTGTGGATCACTTGAGCAAAAATAATCTTGACAGAA

>NL01B.C01.08_A_35

TCCTTAAGCTTCACTACGACTCTACTGTCTAGGTTCTGT

>NL01B.C01.08_A_36

CTATTATTGAAGTTTGCTCTTGCTTGTGTGCAGTCGACTGATTAT

>NL01B.C01.08_A_37

TTTTTTAGAATTGAAGTTACTGCATTTCCGCCTGTCTCATAC

>NL01B.C01.08_A_38

ACCGTCGCTGAAATACGTTAGTGTCAACTCGCCCTCC

>NL01B.C01.08_A_39

AAGTTATACTTTTATAAATATATAAATATGTCATATTATAAGT

>NL01B.C01.08_A_40

AATTTTTAGGGCTAACTGAGAAAAAAGTTAGGAAATATT

>NL01B.C01.08_A_41

TCTTCTCTTCTTTAGTACTAGAAACTTGACTCATCAATAT

>NL01B.C01.08_A_42

AAAGAAAGGTGCAACACATCGACCGCAAACGCATAAATAA

>NL01B.C01.08_A_43

TTGTTAGATTACAAGTATTGGCAGGAGAGAACAACGCCAG

>NL01B.C01.08_A_44

TAAATAAACCCCGTAGCTCACCTCGCCTAGGAAAATAGT

>NL01B.C01.08_A_45

ATGTATATATTTATCCTTATACTATATAAACTTATCTCAAAAGTG

>NL01B.C01.08_A_46

CATAAAGTACTACGTCATTGTTATAGAACTTCTTCCTTTCCAG

>NL01B.C01.08_A_47

TATAGCTACTGCAGGTATCAATACGTATTACTTGCCTCTG

>NL01B.C01.08_A_48

AAAAGTTGCTAGGGCACTTAATAGACTCGGCGGCGGGCCTGCTC

>NL01B.C01.08_A_49

CTTATAGGGTCTCTAACCTCTCCATTCACTTCCTCTTCTACCTC

>NL01B.C01.08_A_50

AACGGAAATTCTGTAAGATATTCAATGACTATACATAGCCAA

>NL01B.C01.08_A_51

AGATTGTGTATAAGTTAAATAGCGATAAGAGCGATTATAT

>NL01B.C01.08_A_52

ATAGCCTTCTTTCTCTTGTAAATTTCTTCAATCACTTCCGC

>NL01B.C01.08_A_53

TTCGGTAATCGTACTGGTGTCCCTACTGGTATTGACACTGG

>NL01B.C01.08_A_54

TTTACTTCAGCTAGCTGAATAAAAAAATTTAAATTAA

>NL01B.C01.08_A_55

GATAATGAGAAACCCTGGTGATTACAGAAAAGAGGTAGAG

>NL01B.C01.08_A_56

ACCCCAGCGGAAAATTCACAGTCACGTTCAACGAGGTGGG

>NL01B.C01.08_A_57

TTACATCTCGCTCCACATTCCAGTTGCCATCAGGACGTA

>NL01B.C01.08_A_58

CTTCTTTTTAACCGTTAGATTTTTAAACCCCGTTCTAGT

>NL01B.C01.08_A_59

AGTTGTTGTGCAAATTGTAAATCTGAATCTAAAACACCGTAG

>NL01B.C01.08_A_60

GCTTTATGCATGGATTGAAAGTTACAATTCTAATTTATCAAC

>NL01B.C01.08_A_61

ATTTGAACTGTTTTCATTTTCATATCACCAATATATAGTATGTA

>NL01B.C01.08_A_62

CATATTCAAACTCTCTAGTTTCATCATTAATATCATTAGT

>NL01B.C01.08_A_63

CGGGAATTACGCACCGCCCCTCCTCGAACAAATGCTG

>NL01B.C01.08_A_64

ATGGCTGACGACGATGACAACTATAAAGACAAGTTTAAGT

>NL01B.C01.08_A_65

AAAACTGTTCTTTCCTTTTTTGTTCTCTCCATCATTTCTCCT

>NL01B.C01.08_A_66

CACGGTGACTTTGCGGAAGAAATGCTATCGTTCTATGCAAAT

>NL01B.C01.08_A_67

TTCTTGCTCCCCAGCATAATGTATGTATAACGTAATATAT

>NL01B.C01.08_A_68

AGAGAAACAGCAGGTACAAAATCAACTCAGCCAAGTACAA

>NL01B.C01.08_A_69

CACCTTGGTGAGTATCAAGTCAAATACGAATATCACAGACA

>NL01B.C01.08_A_70

CTTTCCAATATAAGTTAATATTTCTTGAAAGAGTCCCTTAG

>NL01B.C01.08_A_71

GTATTGGCAGTGGTACAATACCGATGATTGCGTCGTA

>NL01B.C01.08_A_72

TTATGATTGCTTTCCAAGTTTGACCATTCCAGAATAGG

>NL01B.C01.08_A_73

TTAATTTGTTCTAACTGAATTGAAAAAACTAACTTAATTT

>NL01B.C01.08_A_74

AATGCCATCTATCTCACCCGTGCTTGTGCTTCTTCTTGTGA

>NL01B.C01.08_A_75

ATTTTCACGCTGTTAAGTGTCGGGACGGCCAAGCCGA

>NL01B.C01.08_A_76

AAGAAGGTACAACAGACAAATACATTGTGTTAACAAATCTAA

>NL01B.C01.08_A_77

ATATCATAAAAATCCAATACTTTTGCATGCAATTGCGGG

>NL01B.C01.08_A_78

GTTTTTACATAGTTATACTTGGAGGTGAAAAAATGAGTAAAGA

>NL01B.C01.08_A_79

TGTCGATGACCCTAGGTTTTGTCGAGTTAAGCATCTTCGG

>NL01B.C01.08_A_80

GATTATATAATATGAATTATTTGCTTAAAAAGTAGTCGAGA

>NL01B.C01.09_A_1

CAAAACAATCCGTTTTCTAACTGCTTAATTGCGGAAGCCA

>NL01B.C01.09_A_2

ACGCTTATTGCTTTTTTCTGTGCTTCTTCTTTTAGCTTAT

>NL01B.C01.09_A_3

AAAGAGTATTCCCTTCAACATATGCTTTTTTTCAGTTAAACTTT

>NL01B.C01.09_A_4

AAACAAAATTCCAACAGACCCTGAAACAGACGTTTGCAAG

>NL01B.C01.09_A_5

CGAAGTGACGGTGAACGTTATGACCTGGACGACGATTATG

>NL01B.C01.09_A_6

TTTTTCTCTAAATTCTGTAATCTCTTATTGACAACTCCAT

>NL01B.C01.09_A_7

TACTCTTTTATAAGAAAAATGGTGTAAGCTATAAAGGTAA

>NL01B.C01.09_A_8

CGTCTTTCAATTCTATAGTAGATTAG

>NL01B.C01.09_A_9

TTTTTTATCCCCCTGAGTATACTCCTTGGTCAAACAA

>NL01B.C01.09_A_10

TTAATTGCTGACGATTTAAATAAACTCATTGACGCAATTGGT

>NL01B.C01.09_A_11

TTTGTTCACGTGGGACTAATAGGAAAAAGTAATCGTCTAG

>NL01B.C01.09_A_12

GGCTATGTACTCGTAAACGGTGTGCGAATAAGGAGGAAAAT

>NL01B.C01.09_A_13

GTTACAGGCTGAGAAGCACGGCAACATAAGGTTCTTCGT

>NL01B.C01.09_A_14

AACCTTCTTTGCCATGCTAGTTGTCTTTCAGACTTGGAGT

>NL01B.C01.09_A_15

TTAATACTGCTAATCATAGTAGCCGTCCTAATTTGTAC

>NL01B.C01.09_A_16

TACGTGACGTATCAATAATCGCTGGTTTTAATATAGTCATTT

>NL01B.C01.09_A_17

ATATTGTACGTTAAAACAGTGCTAAACTATGATGAATCTA

>NL01B.C01.09_A_18

TCAACTACAGTAAGCGACCCACCACGACCGGAAGTCATTAAC

>NL01B.C01.09_A_19

TTTTCTAAATCGTTTGATATGAGATCTTCTGTTAGGGCTTT

>NL01B.C01.09_A_20

CATCTCCTATATCCGTAACATATAACGAAACGACACTAACTTT

>NL01B.C01.09_A_21

TGAATGGAATCAGTGGTTATGAAGTTAACACCTTTATTTT

>NL01B.C01.09_A_22

AGACAATGTAGAAGTCAGAATAACACCAGAGAATATAA

>NL01B.C01.09_A_23

TTCTAGGAAAATCTCATCTGGTAGAAGTAGTTGGCTCTTA

>NL01B.C01.09_A_24

AGAATTTAGTAAATTCAGGGAATTTAGTAACAGCATCGCCGA

>NL01B.C01.09_A_25

TATTATTCGAAGAAAGAGCGAGAGAAGCATGAATATATT

>NL01B.C01.09_A_26

TCGCCGCCCCCGTGAATTTTTGCAAACCGAGAAAAGTGAGT

>NL01B.C01.09_A_27

CTATCGACCCAAGTTTCATTTCTCCACTTGATTTGCTTT

>NL01B.C01.09_A_28

GTCACTTTTCACTTTACCTTCTTTGAAATTTGTTATTTT

>NL01B.C01.09_A_29

AACTTTCCAGCTCATAACTACTCACCCATATCGTGATGTAG

>NL01B.C01.09_A_30

ATTAGTCCCACGTGAACAAATGGACAGGAAGGAATGAGCTT

>NL01B.C01.09_A_31

CGATATTGCAGGGTATATCGTTTGTGCTTGATCCTGTCAA

>NL01B.C01.09_A_32

GTAGTCCTTCTCGCCCCTCAGTACGACCTCGTTCGGCTCTA

>NL01B.C01.09_A_33

AGTATACCTGAAGTTAGATTGGGACAATCGGATATAAA

>NL01B.C01.09_A_34

TTATTATTATTTACGATTGCTAATGAGTAAAGTGGAATATTG

>NL01B.C01.09_A_35

ACAATCATCACTAATTCACAGTCACAAACCTCAGCATCAGCATA

>NL01B.C01.09_A_36

CAGGTGGTATAAAGCGACGGAAAAAGATGCGAAGAAACG

>NL01B.C01.09_A_37

TAACATTAAGTCTATGCTTTTAGACACGATGTCCAGGATGAGG

>NL01B.C01.09_A_38

GTTATACTCCACAGAATCATTTCCAAGAAGATTCTACATAG

>NL01B.C01.09_A_39

AAGAAGGTACAACAGACAAATACATTGTGTTAACAAATCTAA

>NL01B.C01.09_A_40

CGAGGGGAAAGCTAAGGAGGTAGACTTCAAAGAAGAAGAGAA

>NL01B.C01.09_A_41

TTTGAAAATCGATTCTGTTGTCATTAAAAATTATTTGT

>NL01B.C01.09_A_42

GTCAGCTGTCTAATTCTCTCAATTTCTGAAACTAGCTTTT

>NL01B.C01.09_A_43

ACCCCTAACAATGCGATTTTTCCCGGCGTAGTGCTATATACT

>NL01B.C01.09_A_44

ACAGCTTGCACAAGTTTTGCCAAAAATAGTTAGTTCTCAGT

>NL01B.C01.09_A_45

TCATGTAAATCATACGGTTCACTGTACCATATTTTTATTT

>NL01B.C01.09_A_46

TGAGCAGTTATCGGTATATTGTTCTTCAGTGCTATAGCAATCT

>NL01B.C01.09_A_47

CTTCTTCGGACGAGTTGAAAAGATAATAGTATGTATCTGG

>NL01B.C01.09_A_48

GGATGTCGTAAAAGTTGCTGTAAAACTCTAGCTCCTTAGGGGT

>NL01B.C01.09_A_49

AGAATTGCCCCTTTCAAGGTGGGGAGGAAGACAGCGAA

>NL01B.C01.09_A_50

TTTTTTCTTAGAACTTGTGCAATATCTTCACTAACGTTCCT

>NL01B.C01.09_A_51

ACACTATCACAAACACTTCGATCACAGTCCAAAACACA

>NL01B.C01.09_A_52

AGACCTGGGCAGATACGATAACGAGGCACTGTGCAAAATGTTGC

>NL01B.C01.09_A_53

TAGATAAACTAGGCAAAGCAATAACGAACGCATTAAAAAGTCA

>NL01B.C01.09_A_54

CATGTACTTGGTGTTATAAATCCTACAGTTGTATTATTTTG

>NL01B.C01.09_A_55

ATGTATCTGTCATCGTCTAAGTAATTAACGACAAAGTCCTT

>NL01B.C01.09_A_56

GCTCAAACTAAAGTAGTAATAGTTCCACAAAATCCAAAACT

>NL01B.C01.09_A_57

TCCTTATATATAATGCGGGCAATTTGTTGATTTTTACATA

>NL01B.C01.09_A_58

TATTGCATACTCAAAATAATGTACCGTAGTCCTTACTAATCTG

>NL01B.C01.09_A_59

AACTAATGTTAATGAACCGTCGTAATTCCTTGAGTAATTTT

>NL01B.C01.09_A_60

CCCTTTAGGGCGGGGGTTCCCCGAGGTCTCAGGCGTTACACCCCT

>NL01B.C01.09_A_61

ACATCACGTATCTCTGCTAATAAAATATGGTCTATTGAACA

>NL01B.C01.09_A_62

TTAATTCTTTCAAACAGATGTTTACAAACCCAACGTCAATAG

>NL01B.C01.09_A_63

ACATCTCTTAACAACAACAAAGTGAATGAAATACACGAA

>NL01B.C01.09_A_64

TACACATACCCATTTTAAAGATGAAAGACACCATGATA

>NL01B.C01.09_A_65

TACTCCAAAAGCATCTTCCCTTTGCGTATCGTCGAACGTTAC

>NL01B.C01.09_A_66

TTTCTGTCCATGCGTTTTATCGTAATATTCTTCAACTTCTG

>NL01B.C01.09_A_67

GGATTGACATAGCTAGATGGGAACGATGTTACGTGGATAG

>NL01B.C01.09_A_68

TTCTGCATATTGCAACGCAAAATAAAATGTAAAATACGCTA

>NL01B.C01.09_A_69

TCTTATGGAGTTGACGCAAAATACATCAAAAAGCATGAAG

>NL01B.C01.09_A_70

ATTGTAAATCCGCCACCTTCACCGATCGTTAATCCACCA

>NL01B.C01.09_A_71

CTTGTTCTACTTTTTGACTTTTACCCATTCTGATTTACACC

>NL01B.C01.09_A_72

ATTAAAGCTAGATTGGGATGATGTCGAAAAATTAATAG

>NL01B.C01.09_A_73

TAATGATGTCAAAATCGGAGTCGTTGTCGTTGTTGTAG

>NL01B.C01.09_A_74

CTTCAGGACTCAAATAGGTAAAGTTAATTTCATTTCCATCC

>NL01B.C01.09_A_75

CGACCTCAATAAAACTAATGAAGGGCAATTGAAGGAATTAA

>NL01B.C01.09_A_76

GATTATATAATATGAATTATTTGCTTAAAAAGTAGTCGAGA

>NL01B.C01.09_A_77

ACACATTAGGCGATGCGGTATATTGCGTGTTACTGGTGAC

>NL01B.C01.09_A_78

TGACTTATACGATAACGAATCATATGTAAATTCATCGC

>NL01B.C01.09_A_79

CGTTTGTTTAGGCAAATCGTCGCGTAGTAATAATTTCAGTAA

>NL01B.C01.09_A_80

ATACCCTTATTCGGTAACAAAGCGTAATGGCGTTGATAATG

>NL01B.C01.09_A_81

ACTATCTTGCCATAGAGGTCATTAATTGTAAAATCCCT

>NL01B.C01.09_A_82

TTAAGTGCAAATGCATTAGTGAGTTTAAAGAAATTGAT

>NL01B.C01.09_A_83

CTTATTATTCGTTGTATTTACGCTTCGTAACAACAGTATACT

>NL01B.C01.09_A_84

GTGGTGTTTGCTCGGCATTGTTTATTGCAAAATCGACGTG

>NL01B.C01.09_A_85

ACAGCTACTCCACCCAATGCTACTACTGGAGAAGGAGGAAAT

>NL01B.C01.09_A_86

ACAGTTGTGCTTGCAGTCGTTGCTTTGATTTATTCTGTCTG

>NL01B.C01.09_A_87

TAGTTTGTTCCTGTCCAGCTTCGTTCTTTCCCTTAATTC

>NL01B.C01.09_A_88

AATTTTTGGGCGTTACTGAAAGAAAAGTCAAGAAAATGTT

>NL01B.C01.09_A_89

GATTAACATTTATTACAAATTTCCAAAGTTTGAATTTGC

>NL01B.C01.09_A_90

AGCGTTAATATATGTGCAACAAGCAATTAATGACGCAGAG

>NL01B.C01.09_A_91

TTCTTTTGCATTACATATAGTCTCTTCATCATTACTGCAT

>NL01B.C01.09_A_92

AATAAACGCCAAGAATTTAGTAAATTCAGGGAATTTC

>NL01B.C01.09_A_93

GATTTAAAAGCGATTAGAGGCGGTAGATTTCCTAGTTTT

>NL01B.C01.09_A_94

CACAAAAAAGCATTATTCGAAATTGTTATAAACTTTAAT

>NL01B.C01.09_A_95

TATGCTAACTGAAGAGGAAAGGAAACAAATCAAACCTG

>NL01B.C01.09_A_96

TCTAATACTCCAACATCTACACCGTTAAGTAACGTGT

>NL01B.C01.09_A_97

TTCTTTTGCAAATTCTGTAACCTTTTCGCCATGACAGAA

>NL01B.C01.09_A_98

TGCCCATATATCTAAATAGTATAAATCTGTCAGTGACAAA

>NL01B.C01.09_A_99

ATCGCCAACCGTGAAAACACTATTTGAGAACATTATCGATA

>NL01B.C01.09_A_100

ATTTATGCAGTTCTGCTAAATCAAAAGGATTATCAACATCT

>NL01B.C01.09_A_101

TGCATGGTCTGGAATTTCATTATTATTAATTCTAAATA

>NL01B.C01.09_A_102

GAGGTCGCGCTAAAAAGCGCGGCCAGGGAATGGGCCAC

>NL01B.C01.09_A_103

TTTGACTTTTACCCATTTTTACACCCCTTACTTAAAAGATA

>NL01B.C01.09_A_104

GAGGTCGCGCTAAAAAGCGCAGCCCAGATGTGGGGCGCAGA

>NL01B.C01.09_A_105

ATACATATACTTCCATTACCTATCGTAAGACCTCATGGAGAAC

>NL01B.C01.09_A_106

GTTGATATAAACGTCTGTGAAAAAGTAAAAATATATGATAG

>NL01B.C01.09_A_107

AGAAAACTTACAACATTTTCAATATCATTAACTACTGCACT

>NL01B.C01.09_A_108

ATTTCTCTTGCTTCTTTCAAATACTCCAAAAGTTTAGGTG

>NL01B.C01.09_A_109

TTTTTTCACCCCTAATTATAACTATTGTGCAAACTAATATT

>NL01B.C01.09_A_110

ACACACGGTGCAGTATCGTATGCTTCTAGAATTAATTT

>NL01B.C01.09_A_111

AATGCCATCTATCTCACCCGTGCTTGTGCTTCTTCTTGTGA

>NL01B.C01.09_A_112

ATAATATATTATTAAAAATATGAACATCAAAAAAAGAACGTCCA

>NL01B.C01.09_A_113

GGGGATTAGGTTATGCTCCTGAAGATGCATTGGAAGATGCAGCT

>NL01B.C01.09_A_114

ACTTTACGCGAGGAATGAGGTGAATGAGGAACAGCTGATG

>NL01B.C01.09_A_115

TCCCTACAACGAGTTTCATTTACTTTACTTTATGCCTCCAT

>NL01B.C01.09_A_116

AGGAGTATACTCAGTGATAAAAAATGGTGGAATTAAGTGGC

>NL01B.C01.09_A_117

TCTGATTTAGTCGAAAAACTGAAGGCGGGTAAACTGGAGACTG

>NL01B.C01.09_A_118

GTAATCATCTTTTTGACCTTCGCTAATTGTGCTAATAATT

>NL01B.C01.09_A_119

ACTGTACCACAATAACCATCGGTTGAACTATAAAGTTGGGG

>NL01B.C01.09_A_120

TTTCCAAAACTTGGTAGTTATGCTGAAGAGTTTGATATACAT

>NL01B.C01.09_A_121

TAACAAAGCCCTAATTGTTTCGAATACTTTGTAATATACAT

>NL01B.C01.09_A_122

TATAGCTACTGCAGGTATCAATACGTATTACTTGCCTCTG

>NL01B.C01.09_A_123

TCGCTAATTGTGCTAATAATTCTGGTTCCGCTGTATCATACT

>NL01B.C01.09_A_124

ACAAGAAAGTATTGCAGTAATAGAGACAAAAAAACAAACTACAT

>NL01B.C01.09_A_125

TTCCTTGAAAAACGCCTCAATCTCCTTTTCCTCTTCCTC

>NL01B.C01.09_A_126

TTTTTTCGTAAAACTTGTGCAATATCCTCTGAAACATTTCG

>NL01B.C01.09_A_127

TAACCTGTTCTAACTGAATTGAAAAACTAACATTAATTTCT

>NL01B.C01.09_A_128

TCTATTGAGCGATATCATACCACCACTATACTTACTCTTCT

>NL01B.C01.09_A_129

AAAGTCATAGCCGTATTATAGGTTATGGCTAATGGTGCAG

>NL01B.C01.09_A_130

ACAGTAAATTGAATGGAAGGAGATGTACCAGACAAACCTCTAATGA

>NL01B.C01.09_A_131

ATAATGAGTATTTGAATGCGTTAGCTGAATTTGATAGAACTGG

>NL01B.C01.09_A_132

ACAATATAACTTGCAATTAGCACACCCATTAGCGTTAAA

>NL01B.C01.09_A_133

TGATCAATTTCGTTGCAGGCATACCGATAGTGTTTTATGAAA

>NL01B.C01.09_A_134

GACGTGGTGGAGTTAGAATATGTCACAATCCCTGGTACGTT

>NL01B.C01.09_A_135

TCTGAGTTTGTTGAAGCAACCAATGGGGAAAACGTACCATTTAA

>NL01B.C01.09_A_136

AGAGTTGAAGGCGAATATCAAAACATATTAGATTCGACCCCA

>NL01B.C01.09_A_137

ATAATTTCTAATCTTAAGTCGCTGATTACGTCCATCCAGACT

>NL01B.C01.10_A_1

CATAAAGTACTACGTCATTGTTATAGAACTTCTTCCTTTCCAG

>NL01B.C01.10_A_2

TACGAAATAAATGACGAACAGTTAATCGAAATTATTAA

>NL01B.C01.10_A_3

CAAAACAATCCGTTTTCTAACTGCTTAATTGCGGAAGCCA

>NL01B.C01.10_A_4

CTATTATTGAAGTTTGCTCTTGCTTGTGTGCAGTCGACTGATTAT

>NL01B.C01.10_A_5

GTTGATATAAACGTCTGTGAAAAAGTAAAAATATATGATAG

>NL01B.C01.10_A_6

CTTGCTAACTTGTTTTGCAAAAAGTTTAAATACTTATTT

>NL01B.C01.10_A_7

TTTCTTCTCAGCAAGATAGTCAATAACACTGTTCAAGTACT

>NL01B.C01.10_A_8

ATTTGAACTGTTTTCATTTTCATATCACCAATATATAGTATGTA

>NL01B.C01.10_A_9

CACCTTGGTGAGTATCAAGTCAAATACGAATATCACAGACA

>NL01B.C01.10_A_10

TTTTTGACCATGAGTTTTGTCATAATATTCCTCAACTTCAC

>NL01B.C01.10_A_11

TTAAGTGCAAATGCATTAGTGAGTTTAAAGAAATTGAT

>NL01B.C01.10_A_12

AATATCGTTATGAATTTGTCGTTTGGTAATGGTCGTTTGC

>NL01B.C01.10_A_13

CTTATTATTCGTTGTATTTACGCTTCGTAACAACAGTATACT

>NL01B.C01.10_A_14

AATCAGGTATAACGACATAATGGTTGAATGCAGGCCTACAAG

>NL01B.C01.10_A_15

TAAAGAGTTGTCCATTTACAATATTTCCAATAATTGTATGT

>NL01B.C01.10_A_16

TTTTCTCGAATTATTTCTTCAGAATATGGCACTATTTTTATCCATTC

>NL01B.C01.10_A_17

ACCCCAGCGGAAAATTCACAGTCACGTTCAACGAGGTGGG

>NL01B.C01.10_A_18

TAATTATGGGGATACGGTTAACTTCCAACTATTATT

>NL01B.C01.10_A_19

TTTAATAAATGTAAAGCCTAAGCCACAGCCAACTACTTCCC

>NL01B.C01.10_A_20

ATTACAATTGGTAAATTTTTGCCAGCAATAATAGATAAATT

>NL01B.C01.10_A_21

CTAGCATTGGGGGTTTTAGGGGGATACCCCCTAACTAGAC

>NL01B.C01.10_A_22

TTTACTTCAGCTAGCTGAATAAAAAAATTTAAATTAA

>NL01B.C01.10_A_23

TCTTCTCTTCTTTAGTACTAGAAACTTGACTCATCAATAT

>NL01B.C01.10_A_24

AAGGAGTTGTGGAAACATCCAGTTGAAGGCCTTCTATAGT

>NL01B.C01.10_A_25

AAAATCACTATGCCATAGCGAAATCATTATTAGCTTAATTT

>NL01B.C01.10_A_26

TTCAGATATCCAATTTCAATAATTCATCCATTCTTCTAT

>NL01B.C01.10_A_27

GGCTATGTACTCGTAAACGGTGTGCGAATAAGGAGGAAAAT

>NL01B.C01.10_A_28

TATAGCTACTGCAGGTATCAATACGTATTACTTGCCTCTG

>NL01B.C01.10_A_29

AGTATGAGCTAGAAGTGGATCTTACAACTACTGTTTCTTT

>NL01B.C01.10_A_30

TATAGCGCCGATTGTTCCATATTTACCTATTTTAAGTA

>NL01B.C01.10_A_31

ATCCTCTCCTTAACAAAGATGTTGTATATTCTACCGATATATCTTA

>NL01B.C01.10_A_32

TTTTTTAGAATTGAAGTTACTGCATTTCCGCCTGTCTCATAC

>NL01B.C01.10_A_33

ACCGTCGCTGAAATACGTTAGTGTCAACTCGCCCTCC

>NL01B.C01.10_A_34

AACGGAAATTCTGTAAGATATTCAATGACTATACATAGCCAA

>NL01B.C01.10_A_35

CCAGGTTCCTCCTTAGGACTTTAGGGTCTTTTCTCTGCT

>NL01B.C01.10_A_36

TTATGATTGCTTTCCAAGTTTGACCATTCCAGAATAGG

>NL01B.C01.10_A_37

AAAGAAAGGTGCAACACATCGACCGCAAACGCATAAATAA

>NL01B.C01.10_A_38

TTGTTAGATTACAAGTATTGGCAGGAGAGAACAACGCCAG

>NL01B.C01.10_A_39

CTTCTTTTTAACCGTTAGATTTTTAAACCCCGTTCTAGT

>NL01B.C01.10_A_40

TAAATAAACCCCGTAGCTCACCTCGCCTAGGAAAATAGT

>NL01B.C01.10_A_41

TTAATTTGTTCTAACTGAATTGAAAAAACTAACTTAATTT

>NL01B.C01.10_A_42

ACTGTGGATCACTTGAGCAAAAATAATCTTGACAGAA

>NL01B.C01.10_A_43

AAACCAAATTCCAACAGACCCTGAAACAGACGTTTGCA

>NL01B.C01.10_A_44

TGGCTTAATGCTGTAGTTGATATGATAATGTTGTATTTTCC

>NL01B.C01.10_A_45

ATTATAACAATGATGTCATCTGGATTCATCCAGACCACTCAA

>NL01B.C01.10_A_46

CTTATAGGGTCTCTAACCTCTCCATTCACTTCCTCTTCTACCTC

>NL01B.C01.10_A_47

AAGTTATACTTTTATAAATATATAAATATGTCATATTATAAGT

>NL01B.C01.10_A_48

TCCTTAAGCTTCACTACGACTCTACTGTCTAGGTTCTGT

>NL01B.C01.10_A_49

CCTTATATATGCGTACTTGCTCCTCCTCTTTTGGAAGATT

>NL01B.C01.10_A_50

TCTCATATGGTATAATCGTAAAGTAAGCAAGATCTGCCTT

>NL01B.C01.10_A_51

TTCGGTAATCGTACTGGTGTCCCTACTGGTATTGACACTGG

>NL01B.C01.10_A_52

TAATTTTCTGATAAATCGGGATTAATACTTTATAATAGCTCTGAT

>NL01B.C01.10_A_53

ATAGCCTTCTTTCTCTTGTAAATTTCTTCAATCACTTCCGC

>NL01B.C01.10_A_54

AAAAGTTGCTAGGGCACTTAATAGACTCGGCGGCGGGCCTGCTC

>NL01B.C01.10_A_55

GATAATGAGAAACCCTGGTGATTACAGAAAAGAGGTAGAG

>NL01B.C01.10_A_56

CACGGTGACTTTGCGGAAGAAATGCTATCGTTCTATGCAAAT

>NL01B.C01.10_A_57

AATTTTTAGGGCTAACTGAGAAAAAAGTTAGGAAATATT

>NL01B.C01.10_A_58

AGTTGTTGTGCAAATTGTAAATCTGAATCTAAAACACCGTAG

>NL01B.C01.10_A_59

GCTTTATGCATGGATTGAAAGTTACAATTCTAATTTATCAAC

>NL01B.C01.10_A_60

CGGGAATTACGCACCGCCCCTCCTCGAACAAATGCTG

>NL01B.C01.10_A_61

ATGGCTGACGACGATGACAACTATAAAGACAAGTTTAAGT

>NL01B.C01.10_A_62

AAAACTGTTCTTTCCTTTTTTGTTCTCTCCATCATTTCTCCT

>NL01B.C01.10_A_63

TAGATAAAATATTAGAAGTTGTAGATAAATTAGATGATTATAA

>NL01B.C01.10_A_64

AGAGAAACAGCAGGTACAAAATCAACTCAGCCAAGTACAA

>NL01B.C01.10_A_65

ATATGCAGTCGATATTATTACAAGGACTGACCCCACAAA

>NL01B.C01.10_A_66

CTTTCCAATATAAGTTAATATTTCTTGAAAGAGTCCCTTAG

>NL01B.C01.10_A_67

TACATATTATTTCTAGCATCATAGACTAAATTGGCATAAGTCGG

>NL01B.C01.10_A_68

GTTTTTACATAGTTATACTTGGAGGTGAAAAAATGAGTAAAGA

>NL01B.C01.10_A_69

ACAATATAGGAAGTCGCATTGGGTCTTAACTGGAGAT

>NL01B.C01.10_A_70

AATGCCATCTATCTCACCCGTGCTTGTGCTTCTTCTTGTGA

>NL01B.C01.10_A_71

ATTTTCACGCTGTTAAGTGTCGGGACGGCCAAGCCGA

>NL01B.C01.10_A_72

ATTTAGCTAATAGCTTCAGTCCTTCAATGTTAGCATTAT

>NL01B.C01.10_A_73

AAGAAGGTACAACAGACAAATACATTGTGTTAACAAATCTAA

>NL01B.C01.10_A_74

ATATCATAAAAATCCAATACTTTTGCATGCAATTGCGGG

>NL01B.C01.10_A_75

GTATTGGCAGTGGTACAATACCGATGATTGCGTCGTA

>NL01B.C01.10_A_76

TGTCGATGACCCTAGGTTTTGTCGAGTTAAGCATCTTCGG

>NL01B.C01.10_A_77

GATTATATAATATGAATTATTTGCTTAAAAAGTAGTCGAGA

>NL01B.C01.11_A_1

GCTTTATGCATGGATTGAAAGTTACAATTCTAATTTATCAAC

>NL01B.C01.11_A_2

TACGAAATAAATGACGAACAGTTAATCGAAATTATTAA

>NL01B.C01.11_A_3

CAAAACAATCCGTTTTCTAACTGCTTAATTGCGGAAGCCA

>NL01B.C01.11_A_4

CTATTATTGAAGTTTGCTCTTGCTTGTGTGCAGTCGACTGATTAT

>NL01B.C01.11_A_5

AGAGAAACAGCAGGTACAAAATCAACTCAGCCAAGTACAA

>NL01B.C01.11_A_6

CTTGCTAACTTGTTTTGCAAAAAGTTTAAATACTTATTT

>NL01B.C01.11_A_7

TTTCTTCTCAGCAAGATAGTCAATAACACTGTTCAAGTACT

>NL01B.C01.11_A_8

AATATGGCTAGACGCAGCAGACTTAGACTGGTTAGCTGAT

>NL01B.C01.11_A_9

AAGTTATACTTTTATAAATATATAAATATGTCATATTATAAGT

>NL01B.C01.11_A_10

CACCTTGGTGAGTATCAAGTCAAATACGAATATCACAGACA

>NL01B.C01.11_A_11

TTTTTGACCATGAGTTTTGTCATAATATTCCTCAACTTCAC

>NL01B.C01.11_A_12

TTAAGTGCAAATGCATTAGTGAGTTTAAAGAAATTGAT

>NL01B.C01.11_A_13

AATATCGTTATGAATTTGTCGTTTGGTAATGGTCGTTTGC

>NL01B.C01.11_A_14

CTTATTATTCGTTGTATTTACGCTTCGTAACAACAGTATACT

>NL01B.C01.11_A_15

TAAAGAGTTGTCCATTTACAATATTTCCAATAATTGTATGT

>NL01B.C01.11_A_16

ACTGTGGATCACTTGAGCAAAAATAATCTTGACAGAA

>NL01B.C01.11_A_17

ATTATAACAATGATGTCATCTGGATTCATCCAGACCACTCAA

>NL01B.C01.11_A_18

TGGCTTAATGCTGTAGTTGATATGATAATGTTGTATTTTCC

>NL01B.C01.11_A_19

TTTAATAAATGTAAAGCCTAAGCCACAGCCAACTACTTCCC

>NL01B.C01.11_A_20

ATTACAATTGGTAAATTTTTGCCAGCAATAATAGATAAATT

>NL01B.C01.11_A_21

CTAGCATTGGGGGTTTTAGGGGGATACCCCCTAACTAGAC

>NL01B.C01.11_A_22

TAATTTTCTGATAAATCGGGATTAATACTTTATAATAGCTCTGAT

>NL01B.C01.11_A_23

AAGGAGTTGTGGAAACATCCAGTTGAAGGCCTTCTATAGT

>NL01B.C01.11_A_24

AAAATCACTATGCCATAGCGAAATCATTATTAGCTTAATTT

>NL01B.C01.11_A_25

AATTTTTAGGGCTAACTGAGAAAAAAGTTAGGAAATATT

>NL01B.C01.11_A_26

TAATTATGGGGATACGGTTAACTTCCAACTATTATT

>NL01B.C01.11_A_27

TTTTCTCGAATTATTTCTTCAGAATATGGCACTATTTTTATCCATTC

>NL01B.C01.11_A_28

TATAGCTACTGCAGGTATCAATACGTATTACTTGCCTCTG

>NL01B.C01.11_A_29

AGTATGAGCTAGAAGTGGATCTTACAACTACTGTTTCTTT

>NL01B.C01.11_A_30

TATAGCGCCGATTGTTCCATATTTACCTATTTTAAGTA

>NL01B.C01.11_A_31

ATCCTCTCCTTAACAAAGATGTTGTATATTCTACCGATATATCTTA

>NL01B.C01.11_A_32

TTTTTTAGAATTGAAGTTACTGCATTTCCGCCTGTCTCATAC

>NL01B.C01.11_A_33

ACCGTCGCTGAAATACGTTAGTGTCAACTCGCCCTCC

>NL01B.C01.11_A_34

TCCTTAAGCTTCACTACGACTCTACTGTCTAGGTTCTGT

>NL01B.C01.11_A_35

CCAGGTTCCTCCTTAGGACTTTAGGGTCTTTTCTCTGCT

>NL01B.C01.11_A_36

TCTTCTCTTCTTTAGTACTAGAAACTTGACTCATCAATAT

>NL01B.C01.11_A_37

AAAGAAAGGTGCAACACATCGACCGCAAACGCATAAATAA

>NL01B.C01.11_A_38

TTGTTAGATTACAAGTATTGGCAGGAGAGAACAACGCCAG

>NL01B.C01.11_A_39

TAAATAAACCCCGTAGCTCACCTCGCCTAGGAAAATAGT

>NL01B.C01.11_A_40

TTAATTTGTTCTAACTGAATTGAAAAAACTAACTTAATTT

>NL01B.C01.11_A_41

CATAAAGTACTACGTCATTGTTATAGAACTTCTTCCTTTCCAG

>NL01B.C01.11_A_42

ATTTGAACTGTTTTCATTTTCATATCACCAATATATAGTATGTA

>NL01B.C01.11_A_43

TAAATCGTTGCTTTTAGTAGCAATATCTTGCCCTCGCTGTA

>NL01B.C01.11_A_44

GGCTATGTACTCGTAAACGGTGTGCGAATAAGGAGGAAAAT

>NL01B.C01.11_A_45

CTTATAGGGTCTCTAACCTCTCCATTCACTTCCTCTTCTACCTC

>NL01B.C01.11_A_46

AACGGAAATTCTGTAAGATATTCAATGACTATACATAGCCAA

>NL01B.C01.11_A_47

CCTTATATATGCGTACTTGCTCCTCCTCTTTTGGAAGATT

>NL01B.C01.11_A_48

ATAGCCTTCTTTCTCTTGTAAATTTCTTCAATCACTTCCGC

>NL01B.C01.11_A_49

TCTCATATGGTATAATCGTAAAGTAAGCAAGATCTGCCTT

>NL01B.C01.11_A_50

TTCGGTAATCGTACTGGTGTCCCTACTGGTATTGACACTGG

>NL01B.C01.11_A_51

TTTACTTCAGCTAGCTGAATAAAAAAATTTAAATTAA

>NL01B.C01.11_A_52

AAAAGTTGCTAGGGCACTTAATAGACTCGGCGGCGGGCCTGCTC

>NL01B.C01.11_A_53

GATAATGAGAAACCCTGGTGATTACAGAAAAGAGGTAGAG

>NL01B.C01.11_A_54

ACCCCAGCGGAAAATTCACAGTCACGTTCAACGAGGTGGG

>NL01B.C01.11_A_55

CTTCTTTTTAACCGTTAGATTTTTAAACCCCGTTCTAGT

>NL01B.C01.11_A_56

AGTTGTTGTGCAAATTGTAAATCTGAATCTAAAACACCGTAG

>NL01B.C01.11_A_57

AAACCAAATTCCAACAGACCCTGAAACAGACGTTTGCA

>NL01B.C01.11_A_58

CGGGAATTACGCACCGCCCCTCCTCGAACAAATGCTG

>NL01B.C01.11_A_59

AACGGAAATTCTATAGTAGATTAG

>NL01B.C01.11_A_60

ATGGCTGACGACGATGACAACTATAAAGACAAGTTTAAGT

>NL01B.C01.11_A_61

AAAACTGTTCTTTCCTTTTTTGTTCTCTCCATCATTTCTCCT

>NL01B.C01.11_A_62

TAGATAAAATATTAGAAGTTGTAGATAAATTAGATGATTATAA

>NL01B.C01.11_A_63

TTCAGATATCCAATTTCAATAATTCATCCATTCTTCTAT

>NL01B.C01.11_A_64

GTTGATATAAACGTCTGTGAAAAAGTAAAAATATATGATAG

>NL01B.C01.11_A_65

ATATGCAGTCGATATTATTACAAGGACTGACCCCACAAA

>NL01B.C01.11_A_66

CTTTCCAATATAAGTTAATATTTCTTGAAAGAGTCCCTTAG

>NL01B.C01.11_A_67

AATCAGGTATAACGACATAATGGTTGAATGCAGGCCTACAAG

>NL01B.C01.11_A_68

GTTGACCAAAAACTTTAAATATTAGTTTGCACAATAGTT

>NL01B.C01.11_A_69

TTATGATTGCTTTCCAAGTTTGACCATTCCAGAATAGG

>NL01B.C01.11_A_70

GTTTTTACATAGTTATACTTGGAGGTGAAAAAATGAGTAAAGA

>NL01B.C01.11_A_71

TGTCGATGACCCTAGGTTTTGTCGAGTTAAGCATCTTCGG

>NL01B.C01.11_A_72

AATGCCATCTATCTCACCCGTGCTTGTGCTTCTTCTTGTGA

>NL01B.C01.11_A_73

ATTTTCACGCTGTTAAGTGTCGGGACGGCCAAGCCGA

>NL01B.C01.11_A_74

AAGAAGGTACAACAGACAAATACATTGTGTTAACAAATCTAA

>NL01B.C01.11_A_75

ATATCATAAAAATCCAATACTTTTGCATGCAATTGCGGG

>NL01B.C01.11_A_76

GTATTGGCAGTGGTACAATACCGATGATTGCGTCGTA

>NL01B.C01.11_A_77

ACAATATAGGAAGTCGCATTGGGTCTTAACTGGAGAT

>NL01B.C01.11_A_78

GATTATATAATATGAATTATTTGCTTAAAAAGTAGTCGAGA

>NL01B.C01.12_A_1

TACGAAATAAATGACGAACAGTTAATCGAAATTATTAA

>NL01B.C01.12_A_2

TTCTTGCTCCTCCTCTTTTGGAAGATT

>NL01B.C01.12_A_3

CTATTATTGAAGTTTGCTCTTGCTTGTGTGCAGTCGACTGATTAT

>NL01B.C01.12_A_4

GTTGATATAAACGTCTGTGAAAAAGTAAAAATATATGATAG

>NL01B.C01.12_A_5

CTTGCTAACTTGTTTTGCAAAAAGTTTAAATACTTATTT

>NL01B.C01.12_A_6

CACCTTGGTGAGTATCAAGTCAAATACGAATATCACAGACA

>NL01B.C01.12_A_7

TTTCTTCTCAGCAAGATAGTCAATAACACTGTTCAAGTACT

>NL01B.C01.12_A_8

TCCTTAAGCTTCACTACGACTCTACTGTCTAGGTTCTGT

>NL01B.C01.12_A_9

TGGCTTAATGCTGTAGTTGATATGATAATGTTGTATTTTCC

>NL01B.C01.12_A_10

TTTTTGACCATGAGTTTTGTCATAATATTCCTCAACTTCAC

>NL01B.C01.12_A_11

TTAAGTGCAAATGCATTAGTGAGTTTAAAGAAATTGAT

>NL01B.C01.12_A_12

AATATCGTTATGAATTTGTCGTTTGGTAATGGTCGTTTGC

>NL01B.C01.12_A_13

CTTTCCAATATAAGTTAATATTTCTTGAAAGAGTCCCTTAG

>NL01B.C01.12_A_14

CTTATTATTCGTTGTATTTACGCTTCGTAACAACAGTATACT

>NL01B.C01.12_A_15

AATCAGGTATAACGACATAATGGTTGAATGCAGGCCTACAAG

>NL01B.C01.12_A_16

TTTTCTCGAATTATTTCTTCAGAATATGGCACTATTTTTATCCATTC

>NL01B.C01.12_A_17

AGTATGAGCTAGAAGTGGATCTTACAACTACTGTTTCTTT

>NL01B.C01.12_A_18

ATTATAACAATGATGTCATCTGGATTCATCCAGACCACTCAA

>NL01B.C01.12_A_19

TTTAATAAATGTAAAGCCTAAGCCACAGCCAACTACTTCCC

>NL01B.C01.12_A_20

ATTACAATTGGTAAATTTTTGCCAGCAATAATAGATAAATT

>NL01B.C01.12_A_21

CTAGCATTGGGGGTTTTAGGGGGATACCCCCTAACTAGAC

>NL01B.C01.12_A_22

TTTACTTCAGCTAGCTGAATAAAAAAATTTAAATTAA

>NL01B.C01.12_A_23

AAGGAGTTGTGGAAACATCCAGTTGAAGGCCTTCTATAGT

>NL01B.C01.12_A_24

AAAATCACTATGCCATAGCGAAATCATTATTAGCTTAATTT

>NL01B.C01.12_A_25

TTCAGATATCCAATTTCAATAATTCATCCATTCTTCTAT

>NL01B.C01.12_A_26

TAATTATGGGGATACGGTTAACTTCCAACTATTATT

>NL01B.C01.12_A_27

GGCTATGTACTCGTAAACGGTGTGCGAATAAGGAGGAAAAT

>NL01B.C01.12_A_28

TAGATAAAATATTAGAAGTTGTAGATAAATTAGATGATTATAA

>NL01B.C01.12_A_29

CAAAACAATCCGTTTTCTAACTGCTTAATTGCGGAAGCCA

>NL01B.C01.12_A_30

GTTTTTACATAGTTATACTTGGAGGTGAAAAAATGAGTAAAGA

>NL01B.C01.12_A_31

TGTCGATGACCCTAGGTTTTGTCGAGTTAAGCATCTTCGG

>NL01B.C01.12_A_32

ACTGTGGATCACTTGAGCAAAAATAATCTTGACAGAA

>NL01B.C01.12_A_33

TATAGCGCCGATTGTTCCATATTTACCTATTTTAAGTA

>NL01B.C01.12_A_34

ATCCTCTCCTTAACAAAGATGTTGTATATTCTACCGATATATCTTA

>NL01B.C01.12_A_35

GCTTTATGCATGGATTGAAAGTTACAATTCTAATTTATCAAC

>NL01B.C01.12_A_36

AAGTTATACTTTTATAAATATATAAATATGTCATATTATAAGT

>NL01B.C01.12_A_37

CCAGGTTCCTCCTTAGGACTTTAGGGTCTTTTCTCTGCT

>NL01B.C01.12_A_38

TAAAGAGTTGTCCATTTACAATATTTCCAATAATTGTATGT

>NL01B.C01.12_A_39

AAAGAAAGGTGCAACACATCGACCGCAAACGCATAAATAA

>NL01B.C01.12_A_40

TTGTTAGATTACAAGTATTGGCAGGAGAGAACAACGCCAG

>NL01B.C01.12_A_41

TAAATAAACCCCGTAGCTCACCTCGCCTAGGAAAATAGT

>NL01B.C01.12_A_42

TTAATTTGTTCTAACTGAATTGAAAAAACTAACTTAATTT

>NL01B.C01.12_A_43

CATAAAGTACTACGTCATTGTTATAGAACTTCTTCCTTTCCAG

>NL01B.C01.12_A_44

AAACCAAATTCCAACAGACCCTGAAACAGACGTTTGCA

>NL01B.C01.12_A_45

CCTTATATATGCGTACTTGCTCCTCCTCTTTTGGAAGATT

>NL01B.C01.12_A_46

CTTATAGGGTCTCTAACCTCTCCATTCACTTCCTCTTCTACCTC

>NL01B.C01.12_A_47

AACGGAAATTCTGTAAGATATTCAATGACTATACATAGCCAA

>NL01B.C01.12_A_48

TTCTTGCTCAAGTGATCCACAGTCTTTCAATTCTATAGTAGATTAG

>NL01B.C01.12_A_49

AAAAGTTGCTAGGGCACTTAATAGACTCGGCGGCGGGCCTGCTC

>NL01B.C01.12_A_50

TATAGCTACTGCAGGTATCAATACGTATTACTTGCCTCTG

>NL01B.C01.12_A_51

ATAGCCTTCTTTCTCTTGTAAATTTCTTCAATCACTTCCGC

>NL01B.C01.12_A_52

TTTTTTAGAATTGAAGTTACTGCATTTCCGCCTGTCTCATAC

>NL01B.C01.12_A_53

ACCGTCGCTGAAATACGTTAGTGTCAACTCGCCCTCC

>NL01B.C01.12_A_54

TAATTTTCTGATAAATCGGGATTAATACTTTATAATAGCTCTGAT

>NL01B.C01.12_A_55

GATAATGAGAAACCCTGGTGATTACAGAAAAGAGGTAGAG

>NL01B.C01.12_A_56

CACGGTGACTTTGCGGAAGAAATGCTATCGTTCTATGCAAAT

>NL01B.C01.12_A_57

TTACATCTCGCTCCACATTCCAGTTGCCATCAGGACGTA

>NL01B.C01.12_A_58

CTTCTTTTTAACCGTTAGATTTTTAAACCCCGTTCTAGT

>NL01B.C01.12_A_59

AGTTGTTGTGCAAATTGTAAATCTGAATCTAAAACACCGTAG

>NL01B.C01.12_A_60

AATTTTTAGGGCTAACTGAGAAAAAAGTTAGGAAATATT

>NL01B.C01.12_A_61

ATTTGAACTGTTTTCATTTTCATATCACCAATATATAGTATGTA

>NL01B.C01.12_A_62

CGGGAATTACGCACCGCCCCTCCTCGAACAAATGCTG

>NL01B.C01.12_A_63

ATGGCTGACGACGATGACAACTATAAAGACAAGTTTAAGT

>NL01B.C01.12_A_64

AAAACTGTTCTTTCCTTTTTTGTTCTCTCCATCATTTCTCCT

>NL01B.C01.12_A_65

ATATCATAAAAATCCAATACTTTTGCATGCAATTGCGGG

>NL01B.C01.12_A_66

TTCTTGCTCCCCAGCATAATGTATGTATAACGTAATATAT

>NL01B.C01.12_A_67

AGAGAAACAGCAGGTACAAAATCAACTCAGCCAAGTACAA

>NL01B.C01.12_A_68

ATATGCAGTCGATATTATTACAAGGACTGACCCCACAAA

>NL01B.C01.12_A_69

TCTCATATGGTATAATCGTAAAGCAAGCAAGATCTGCCTT

>NL01B.C01.12_A_70

TACATATTATTTCTAGCATCATAGACTAAATTGGCATAAGTCGG

>NL01B.C01.12_A_71

TTATGATTGCTTTCCAAGTTTGACCATTCCAGAATAGG

>NL01B.C01.12_A_72

TCTTCTCTTCTTTAGTACTAGAAACTTGACTCATCAATAT

>NL01B.C01.12_A_73

AATGCCATCTATCTCACCCGTGCTTGTGCTTCTTCTTGTGA

>NL01B.C01.12_A_74

ATTTTCACGCTGTTAAGTGTCGGGACGGCCAAGCCGA

>NL01B.C01.12_A_75

ACCCCAGCGGAAAATTCACAGTCACGTTCAACGAGGTGGG

>NL01B.C01.12_A_76

AAGAAGGTACAACAGACAAATACATTGTGTTAACAAATCTAA

>NL01B.C01.12_A_77

TTCGGTAATCGTACTGGTGTCCCTACTGGTATTGACACTGG

>NL01B.C01.12_A_78

GTATTGGCAGTGGTACAATACCGATGATTGCGTCGTA

>NL01B.C01.12_A_79

ACAATATAGGAAGTCGCATTGGGTCTTAACTGGAGAT

>NL01B.C01.12_A_80

GATTATATAATATGAATTATTTGCTTAAAAAGTAGTCGAGA

>NL01B.C01.13_A_1

TCATTAATTTCCTTGAATTTACCATTATGATATTTGTAA

>NL01B.C01.13_A_2

AAAGAGTATTCCCTTCAACATATGCTTTTTTTCAGTTAAACTTT

>NL01B.C01.13_A_3

AACCCACACTGGTACGCCAAATACCTTTTTGTTTAGGT

>NL01B.C01.13_A_4

TTTGATACCGTTATGATAGACTGAACCAGTTAGTATGCCA

>NL01B.C01.13_A_5

GCGCTGGCGCAGCAGCGTTAGGGACAGGTATAGGCTT

>NL01B.C01.13_A_6

ATCTTTTGCAAATTCTGTAACCTTTTCGCCATGACAGAA

>NL01B.C01.13_A_7

ATTTTAGGTGTAGATGTTAAGCAATATGCCGACGCAGTAGA

>NL01B.C01.13_A_8

AGTTGTCTTAGCTTCTCCCTTCCTCAAAATCGTTAGGATG

>NL01B.C01.13_A_9

GCTACTGTGGTATATAGCATGACAACTGGGTTACCATA

>NL01B.C01.13_A_10

GTTCAGACTGTTGGTAATCGTTTGCAAGAGATGAAAAAT

>NL01B.C01.13_A_11

ATATTGTACGTTAAAACAGTGCTAAACTATGATGAATCTA

>NL01B.C01.13_A_12

TCAAAATGAGTATCACACCAAACTACTAAATTATCATCTATTA

>NL01B.C01.13_A_13

TTTTTGGTCAGTATATTTTTATGCCAACCCGGTATTTA

>NL01B.C01.13_A_14

TCGCCGCCCCCGTGAATTTTTGCAAACCGAGAAAAGTGAGT

>NL01B.C01.13_A_15

CGAATTAATTCTACATACATTATTTTTATCCTTTTCCTTAT

>NL01B.C01.13_A_16

AGTATACCTGAAGTTAGATTGGGACAATCGGATATAAA

>NL01B.C01.13_A_17

GTATCTGAGCTAACACTTGACTCTGAGATATTGTAGT

>NL01B.C01.13_A_18

TGAACTTGTAAAAAAAGGCTATTCAATAGCTCAAATTGCAAA

>NL01B.C01.13_A_19

TTATTATTATTTACGATTGCTAATGAGTAAAGTGGAATATTG

>NL01B.C01.13_A_20

TTTCTACGACCCCGACGAGAAGGAGTTCGCTTACGCAAT

>NL01B.C01.13_A_21

CGAGGGGAAAGCTAAGGAGGTAGACTTCAAAGAAGAAGAGAA

>NL01B.C01.13_A_22

AAGTCTTCTATTTCTTGCATATTCCAAGTAAGTATTTTGT

>NL01B.C01.13_A_23

TCTGGTATGCCCTCATACGATAATATACGATAACAAAATAT

>NL01B.C01.13_A_24

ACAGCTTGCACAAGTTTTGCCAAAAATAGTTAGTTCTCAGT

>NL01B.C01.13_A_25

CTTCTTCGGACGAGTTGAAAAGATAATAGTATGTATCTGG

>NL01B.C01.13_A_26

GCTAATATTAAATTGTTTGCCAGAATCCCTAACAGGAACGTTTT

>NL01B.C01.13_A_27

TTGAGTATATGGACTTTAACGGTAACTGGCATACAAATAG

>NL01B.C01.13_A_28

GTGTCTGAGTATGATACAGCAGAACCAGAATTATTAGCAC

>NL01B.C01.13_A_29

AGTATATTAGCAACTTCTAGCACATAATGCACTTTAATGAAC

>NL01B.C01.13_A_30

ATTGAAATTAACGAGGAAGTGGTAGCCCTAGCGTATGGTGCGCTCTT

>NL01B.C01.13_A_31

ACATCACGTATCTCTGCTAATAAAATATGGTCTATTGAACA

>NL01B.C01.13_A_32

TGATTTTGTCAGTTGGCACACCTCTTACAATTTTTAACT

>NL01B.C01.13_A_33

CTTATTATTCGTTGTATTTACGCTTCGTAACAACAGTATACTTA

>NL01B.C01.13_A_34

TCTTATGGAGTTGACGCAAAATACATCAAAAAGCATGAAG

>NL01B.C01.13_A_35

AACTTATCAACGCACTATTTACAATGTTCAACACTTCTCTCT

>NL01B.C01.13_A_36

TTTCTGTCCATGCGTTTTATCGTAATATTCTTCAACTTCTG

>NL01B.C01.13_A_37

ACAATCGCCACAAAAGGCGGGGCATGGGAAGAATATTTT

>NL01B.C01.13_A_38

TGGAAAACTGGCAATTCAACATTTAGACCTACTTCTTGAA

>NL01B.C01.13_A_39

ACTGTCATAAACGAATATAGAAAATACATTGACTATTTTAGA

>NL01B.C01.13_A_40

ATATACATTTCTCTTTCCCCCTTTATAAATTTTTCTCT

>NL01B.C01.13_A_41

AATATATCACTGTCAAAATACGCTTCAGCACAAGCTAAGG

>NL01B.C01.13_A_42

TGAGGTTCTTTAAATCAAGCAAATCGACCCTGCCCTTTAT

>NL01B.C01.13_A_43

CCTGCGATGTCGACCAGAAAGACCTGCGTATAGAAAAAAG

>NL01B.C01.13_A_44

ATACCTTTAAACCATTATATACACCCGTCTTCCTCACCTGTT

>NL01B.C01.13_A_45

CTTCAGGACTCAAATAGGTAAAGTTAATTTCATTTCCATCC

>NL01B.C01.13_A_46

GTATTGGCAGTGGTACAATACCGATGATTGCGTCGTA

>NL01B.C01.13_A_47

GGATAGACATGTTTGTATTCTTCACAAAACCACTTAAGTAAG

>NL01B.C01.13_A_48

CATGTACATATCAATATAGTCTTATCATCATCAGGCAAGT

>NL01B.C01.13_A_49

ATCAGTTAGATTCAAGTTGTTGCTTGATCTTGATTACGATTA

>NL01B.C01.13_A_50

TGCTTATCTTGAAAATACGGATATTTCTCATCTTTGTAAT

>NL01B.C01.13_A_51

TTCAAAATCTAAAAGGCGGAGTGGGTAAAACAACATTAACG

>NL01B.C01.13_A_52

ACTGTTACAGCTGTAGTACAAACAGTAAAAGCTGCATTATC

>NL01B.C01.13_A_53

ACAGTTGTGCTTGCAGTCGTTGCTTTGATTTATTCTGTCTG

>NL01B.C01.13_A_54

ACTCTGCGGTTTCAGAGCTATTAAGTTATTCGCTTTGAAA

>NL01B.C01.13_A_55

ATCAACTTCTCCTTGAGAAGAGTCATAAGTATAGTATAATGC

>NL01B.C01.13_A_56

GATTAACATTTATTACAAATTTCCAAAGTTTGAATTTGC

>NL01B.C01.13_A_57

TCTTTATACATCTCCCTGATTTCTTGTGTACTCAAGACCT

>NL01B.C01.13_A_58

TCAGTATTCTTTGCTATTTCACTAGCAGTCTCTAGTAGTTTTTTG

>NL01B.C01.13_A_59

AAACCACCAACACCTATAATCATAAAAAGTTTATCTTCCC

>NL01B.C01.13_A_60

GTTACGTTTACGTTTCTGTGCAGCAAGGTAATTCGTTTAG

>NL01B.C01.13_A_61

AGTTTTACTGTAATATACAACTCGTCGTACGGAATATCGGAATAC

>NL01B.C01.13_A_62

TTCTTTTGCATTACATATAGTCTCTTCATCATTACTGCAT

>NL01B.C01.13_A_63

CCTAACCCACCGATACATAAACCAGAACCAATCGTTAA

>NL01B.C01.13_A_64

TCACCGCTTTACTCAGGTCCATCCATAGAAACTGTAAGAATAA

>NL01B.C01.13_A_65

TTGTTAGATTACAAGTATTGGCAGGAGAGAACAACGCCAG

>NL01B.C01.13_A_66

AATAAACGCCAAGAATTTAGTAAATTCAGGGAATTTC

>NL01B.C01.13_A_67

AGTGTTAAGTAAGGAGTTATGCTATCCAATTCTGCCGA

>NL01B.C01.13_A_68

CTTATAGGGTCTCTAACCTCTCCATTCACTTCCTCTTCTACCTC

>NL01B.C01.13_A_69

GTGGAACTTGATGCGGTTATGTTAGTCAAGAGGGATTATACC

>NL01B.C01.13_A_70

AAAGACTTAGATTTTGTAGTCACGGATTTTGATAAAGCGTTAA

>NL01B.C01.13_A_71

TGATTTTGTCAGTGATATATTTTTCAATTCTATAGTAGATTAG

>NL01B.C01.13_A_72

TTCTAACGTTTTAGGAGTAAGTTCTACAGTAAAATTGAAA

>NL01B.C01.13_A_73

TCCCAAGGCTGGGAGGGTTTTCGTCTCGTCATCAATCAGG

>NL01B.C01.13_A_74

TCAAGAAGTTTATAATCTCTTCATTATCGAACACCTTCCTG

>NL01B.C01.13_A_75

ATTAATGACATTAATAAACAACATAAGCAAAATCGAGAACG

>NL01B.C01.13_A_76

TATTGGATTTAATGGAGCATTTAAATTCATATTTCCACACCA

>NL01B.C01.13_A_77

TATGACTTATTCAGGAATATTAGGACGATGTGTCCAAAT

>NL01B.C01.13_A_78

ATTTCTCTTGCTTCTTTCAAATACTCCAAAAGTTTAGGTG

>NL01B.C01.13_A_79

TAAAATTGCTCTTAGTTTGTCAGAGAAAGGTAATGACTTTTTA

>NL01B.C01.13_A_80

GTCAGCTGTCTAATTCTCTCAATTTCTGAAACTAGCTTTT

>NL01B.C01.13_A_81

TTAGCCTCCCCGCTAATAATCGTCGTCTGACCCGTAT

>NL01B.C01.13_A_82

TTATTTAGACCTGTTTCAATTCGTGATGTAATTTTAG

>NL01B.C01.13_A_83

ACTATTTCTAAATGTTTCGGATCTTCGTCAAATTTACTTG

>NL01B.C01.13_A_84

GGGGATTAGGTTATGCTCCTGAAGATGCATTGGAAGATGCAGCT

>NL01B.C01.13_A_85

TCCCTACAACGAGTTTCATTTACTTTACTTTATGCCTCCAT

>NL01B.C01.13_A_86

CCGTCACCTCCACCACCTGTATTCTCTTCAACACAGAG

>NL01B.C01.13_A_87

ATTGACCCCGATACCATAGACTATATCATTAGACTTATTGA

>NL01B.C01.13_A_88

CATAAGATCCGCTATCTCGTTTAATAGTAGTGCTAATTCTA

>NL01B.C01.13_A_89

TTATATTCTTTAACTATCAAATAATATCTCGCCTCATCTCT

>NL01B.C01.13_A_90

TTAAATTCGCAATTTTAGCATAAATAATTATTTGCTCCTGG

>NL01B.C01.13_A_91

TATTTCTAACTCTGTTGCTCACATGACTGAATTTGTCCATTTA

>NL01B.C01.13_A_92

TCTAAGAAAATTTTTGTCACTTGGCATTCTTCGTAACGT

>NL01B.C01.13_A_93

TATAATGAACTATCTAAGTTAGGGTATTCTATCAATGTCACTA

>NL01B.C01.13_A_94

TTTTTTCGTAAAACTTGTGCAATATCCTCTGAAACATTTCG

>NL01B.C01.13_A_95

TTAATGTAAGATTTTAAAAAATGACAAATATAAATCATTC

>NL01B.C01.13_A_96

CTTTATTCTGTTCTTTCTTTTTCTCTTCCTTTTTGGGCTTTTT

>NL01B.C01.13_A_97

ATATAGACTATTACCCTGAAACTTGTGCTGATAATATAAAGGGACT

>NL01B.C01.13_A_98

AATTGCCTAATTACATAATTTCCTAATTTCTCCTCCAAT

>NL01B.C01.13_A_99

TGAACGCTAATTTCGTCATAACAATAACATATGAGCGTG

>NL01B.C01.13_A_100

ATAATTGTAAGATATATCAATCGAATATACAACATCTTTG

>NL01B.C01.13_A_101

TGATAATTTGATAAATCTATATTGAAAGTTTGTCCAACTTG

>NL01B.C01.13_A_102

AAAAACATAGTAGAAATGTTAATGACCAACAAAAGTGAAT

>NL01B.C01.13_A_103

ATAATGAGTATTTGAATGCGTTAGCTGAATTTGATAGAACTGG

>NL01B.C01.13_A_104

ATAAAATCGAAATTATTCATGAAAACTTCTACTAGCATA

>NL01B.C01.13_A_105

TCATTATCAAAATTAGATAATGGCTCATTCCCATTAAAA

>NL01B.C01.13_A_106

ATAATTTCTAATCTTAAGTCGCTGATTACGTCCATCCAGACT

>NL01B.C01.13_A_107

CCAAAATAACTACATGTACTTGGTGTTATAAATCCTACAGT

>NL01B.C01.14_A_1

AATATAAACCCTATGCGAACGTTTTTTATTTTTTAACTTTA

>NL01B.C01.14_A_2

TCGTATGTATATTTCTCATAAAGTTTTCTATCATTTGCAGTATT

>NL01B.C01.14_A_3

TCAGAAATAAAGTCAATATATGGTGGAAAATGTCCACAT

>NL01B.C01.14_A_4

GCTGAATTCTCCGCCAGGATGACGCTGAAGGTAAATGGGT

>NL01B.C01.14_A_5

ACTTGTACTAAGATATGATCGGATGGCGATGTTATCATA

>NL01B.C01.14_A_6

TCCGTGGTGCCCAACATGTATTACGGTTACAGTGTGTTCAA

>NL01B.C01.14_A_7

TATAATAAATTTTCGTTATTTTGTGATATATTGTTTAGTGA

>NL01B.C01.14_A_8

TTAATTGCTGACGATTTAAATAAACTCATTGACGCAATTGGT

>NL01B.C01.14_A_9

TGCCAGTTTATCAAAACGAAGGAGTACGCTGTGTAA

>NL01B.C01.14_A_10

CTTCCATTGCAGAAGCTAAGGCTACAGCGGAAACGTTTT

>NL01B.C01.14_A_11

ATCTATGTTTGATAATATCCATTTTATCAAATTCGTCTAT

>NL01B.C01.14_A_12

AACTTATATCTTTACAAATACCTCATAGACAATGTAACACGC

>NL01B.C01.14_A_13

AATTTGTTATATTCTATATGCTTACCTTGGACTTGTTCTAACTG

>NL01B.C01.14_A_14

AATATATTTAAGAGTTCGATCGAGGACTTGCTCCACGCTAA

>NL01B.C01.14_A_15

TTTTCAGTTTCTATATTAATTGTATTTGCTGAGGTATAAT

>NL01B.C01.14_A_16

TTTTTCTCCTCAATATATCATTTGCAAAAACTTATATTT

>NL01B.C01.14_A_17

AGTACATTATCCAATGAATACTTTTTACCGTTAATA

>NL01B.C01.14_A_18

TGACATTATAGGTTTGGGTTTGTGTGGTTTGGGATTCG

>NL01B.C01.14_A_19

TTAGGAGGATAAGCTCTAGTTCTAACCCAATTTATTGT

>NL01B.C01.14_A_20

ATTTGTAAATTAGAACCATCATCTAATACAGCCAAAGCT

>NL01B.C01.14_A_21

TTTCTCTTAGTCGTCCTATGTTTGTATGTATCTGTGATGG

>NL01B.C01.14_A_22

AGTCTTATTGAATTTGGTGAATCTAATAACAAAGATTTTA

>NL01B.C01.14_A_23

ATTTAGTATACGATGCTCGAAACAATATGTACGGTGCAG

>NL01B.C01.14_A_24

AAGAACAAATGTAAGGATGACAAAAGCTATGTATAATAGCTAA

>NL01B.C01.14_A_25

TATCCTCAATTTTGAAAGTTCTTTCGGTAGTTATTCTCCTGGC

>NL01B.C01.14_A_26

CGGGAATTACGCACCGCCCCTCCTCGAACAAATGCTG

>NL01B.C01.14_A_27

GCTATATAAATTGGATCAAGTTCAGTTAGCACTGGCTGACCA

>NL01B.C01.14_A_28

GAAGGCGTGGGCGTTCAAATAGATCACGGGGGTACCGC

>NL01B.C01.14_A_29

TCTTCACTGTCACGTACAGCTTTGAATATTCAATATGCTTA

>NL01B.C01.14_A_30

TTATTATTTAGAAAATCTATAACTGATTGATTTGCAATA

>NL01B.C01.14_A_31

AACTGTCTAATTTCATAATATACTAATTATATAATATGCTAA

>NL01B.C01.14_A_32

AAGTAAAACGCGAATTCAGTAGTAGTGTTTTTGCATAATGTCTTT

>NL01B.C01.14_A_33

AAACAATGAGTATTCTTTGCCTTACAAATATAGAGAGCATACCTA

>NL01B.C01.14_A_34

ACTGCAGTAGTGTTTCCACTTATCGTGGTGTTTCCAGTCT

>NL01B.C01.14_A_35

TGAGCAGTTATCGGTATATTGTTCTTCAGTGCTATAGCAATCT

>NL01B.C01.14_A_36

AATTTGATCTTTTAGATGAAGACACGCAGCAGAAGAAGGT

>NL01B.C01.14_A_37

ACTGTTAAAGAGGCATGGTTCCCGTGGGCTGACTTTGACTA

>NL01B.C01.14_A_38

TCGTAATCATCACCGTATTCGTATAACAGTATAACGGTTCTT

>NL01B.C01.14_A_39

TAACTTAGCTACATTTAAATGTAGAATATGGTCATGAA

>NL01B.C01.14_A_40

ACAACGCGATGTAGAGCGTTAAGAAAGCAACAGTATATG

>NL01B.C01.14_A_41

ACTTAATAAAAAGTAGGATTGACTCAATGAGAGAACTCA

>NL01B.C01.14_A_42

TTTCTTCTTTGGTCTTCTCTTTGTTAGTTAAGAAGTCGT

>NL01B.C01.14_A_43

AGTTTTAACTAATTCTTTGTCCTCAAAATCAAGAACTAT

>NL01B.C01.14_A_44

ACAGCTACACCACCCAATGCTACTACTGGAGAAGGAAATGG

>NL01B.C01.14_A_45

CCCTTTAGGGCGGGGGTTCCCCGAGGTCTCAGGCGTTACACCCCT

>NL01B.C01.14_A_46

CTTCAGCATCCTTTTGCAAATCATTAAATGTTCTCCATTC

>NL01B.C01.14_A_47

TTTGACTTTTACCCATTTTTACACCCCTTACTTAAAAGATAAA

>NL01B.C01.14_A_48

GCAAGGGAGGTGTTGCTGAGAACAAATATGAATATTCATAATAACCC

>NL01B.C01.14_A_49

TTAATCATGTTAACTAACGCCGGTCCACCGCCAAGTCTATT

>NL01B.C01.14_A_50

GATGAAAATACTATTGAGAAAAAATGCACGGCAAAATACTAT

>NL01B.C01.14_A_51

TGTTCCACTGCTCTTTGTAATAGTCTAGCTGTCTGATTAT

>NL01B.C01.14_A_52

CTCCTGCTGCTACGCTAGTTCCGGGCGCCGGATAAGC

>NL01B.C01.14_A_53

TAGATAAAATATTAGAAGTTGTAGATAAATTAGATGATTATAA

>NL01B.C01.14_A_54

ACAAGAAGTGAAAAAGGAGTTCGGTGGACTGGTAGAAGATACT

>NL01B.C01.14_A_55

TTCTGTTGGGCTAACTGTAAGGCTTGTTGATATTTACCCT

>NL01B.C01.14_A_56

TAATGATGTCAAAATCGGAGTCGTTGTCGTTGTTGTAG

>NL01B.C01.14_A_57

TATTTTTTACAAATTTTTAATAATATGCAAAGCATGGA

>NL01B.C01.14_A_58

ACTGCTCAACGTAATAATGGCGATAAGGACAATGTTCATG

>NL01B.C01.14_A_59

TTAATAGTTTCAAAATGTTTACAACTAGATATCAATACCAAT

>NL01B.C01.14_A_60

GTCACTTTTCACTTTACCTTCTTTGAAATTTGTTATTTT

>NL01B.C01.14_A_61

TAATATACTGATGAAGATGGAGGCTTACGGCTGGATA

>NL01B.C01.14_A_62

ATACCCTTATTCGGTAACAAAGCGTAATGGCGTTGATAATG

>NL01B.C01.14_A_63

AAAAAACTTGCGATTTGCCTAAGACTATAGCCCTTACG

>NL01B.C01.14_A_64

GAGCATGGAATTGTGGTAATACCGTTGGATATGGGA

>NL01B.C01.14_A_65

AGCAAATTATAGATGTCTTGTGTTGTTCTCATCTGCTTTTCT

>NL01B.C01.14_A_66

ACAATATCACCGTAAAGCGTAATACATCCCGCATCACCGG

>NL01B.C01.14_A_67

GTTCTAATTGAATTGAAAAACTAACATTAATTTCTTGTTG

>NL01B.C01.14_A_68

AATCAAATTGGTAATACACTTACACAAAATTTTGATAATTTTTA

>NL01B.C01.14_A_69

GAGACCGGGAGAAGTAAACAAAGAGTTTATTTAAAACAATA

>NL01B.C01.14_A_70

GGACGAAAGGGGCGAAAGAGAGAATAACTCAAACAGCTGTTT

>NL01B.C01.14_A_71

GGTCTTGTGTCTTTCAGCCCCGTGTATGCGGTTAATGCTACAC

>NL01B.C01.14_A_72

AAATCAACACAAATGCTTCTTATCGTTCTATTATAGAA

>NL01B.C01.14_A_73

CCAAATTCGAAGTAGCTACACCTTGAGCTATTATATTCC

>NL01B.C01.14_A_74

AAGTTTACTAGTGTAAATGAACAAGTTAGTTCAGTTTTAAGCGAATA

>NL01B.C01.14_A_75

AAACGCATTAGACAATAATGAGTATGTTGTCACATCAATCGCA

>NL01B.C01.14_A_76

TACTTCTCAATAAATTAGTGGGTTTAAAAAACTATTTAAGTTT

>NL01B.C01.14_A_77

TCTTGTGGTATTGCATTTAGCTGAACTATTATTCTTTTTATT

>NL01B.C01.14_A_78

CTTTGTAGAACTCCTCTTCATTTTTCTTCAACCCGTCAA

>NL01B.C01.14_A_79

TTTAATGATTTCTATTTTGCGACGTTCGATGATATGTCTTC

>NL01B.C01.14_A_80

AACTAACTGCCATAAGGAGTTTCCATATCGCGATTCT

>NL01B.C01.14_A_81

TTCAAAGTGGGAAAAGTTCCAATTAATTTAGGAAATGCAGA

>NL01B.C01.14_A_82

ATTTATGCAGTTCTGCTAAATCAAAAGGATTATCAACATCT

>NL01B.C01.14_A_83

CTTTCTGTAAGTACATATTCCCCATTTTTTTCTTCAATTAACCCGT

>NL01B.C01.14_A_84

CTAATGCAGGCCTTTCGAGCCTGTGACCCGGGTTCAAATCC

>NL01B.C01.14_A_85

CATCTCCTATATCCGTAACATATAACGAAACGACACTAACTTT

>NL01B.C01.14_A_86

CGTTTATTAAATAATCATTAATTTTCAACACTTTTAATTCATTTTC

>NL01B.C01.14_A_87

AGTTGGACAAAGTTTCAACATAAATTTATCAAATTATCA

>NL01B.C01.14_A_88

CATTTACTATACTCTTTAACATTAACTTGAATATGGCGTCGTCT

>NL01B.C01.14_A_89

ACACACGGTGCAGTATCGTATGCTTCTAGAATTAATTT

>NL01B.C01.14_A_90

GATAAACAGCTAAAGTCCACGAGACCTGGGGAGAAGGTGC

>NL01B.C01.14_A_91

ATACTGAAGGGTAGTACTATATAATTACCAAAGAGCAGACC

>NL01B.C01.14_A_92

ATTGCGAAATAATAAGTATATTGTTTATAAAAATCTATAG

>NL01B.C01.14_A_93

AAATTCTTTTCACAACCTTCACAATCCATTACTAAAATATCAG

>NL01B.C01.14_A_94

TGAAAATTTTGCGTATATTCCCTAATTGAAAAAATATGGGAAT

>NL01B.C01.14_A_95

AATAGATACAAGTATTCAATAGTTAACCACGATGGGACAC

>NL01B.C01.14_A_96

ATCCTGTTCCCAAAGGCTACCCTAAGTTTTATTAGGTCGTCCC

>NL01B.C01.14_A_97

ATAAACGGTGGCGATTATGCATATATTCGCTCAACATTTTCTT

>NL01B.C01.14_A_98

AAAAATTAATGATCTAACTTCCTCCCCACCCTGGAAGGG

>NL01B.C01.14_A_99

AAAGTAGTAGATGACATAGGTGCATATGTCGGAGACACTGC

>NL01B.C01.14_A_100

AATTGCCTTGATTATACGTTAAATTAAATTGGTATAGT

>NL01B.C01.14_A_101

GAAATTTTTAGACCTTCAATCTCACCTTTTATCTCAACTTCA

>NL01B.C01.14_A_102

TGATGCAAAAACTATCTGATACGATAGCCAACCTTAGTT

>NL01B.C01.14_A_103

TCAAGTTTGTTGGCTTCACCATTCCAGCGTTTAACTTCTTCCA

>NL01B.C01.14_A_104

TGATTGAAATACCCAAATGCCACACCGTCAGAGTTATTAGCGA

>NL01B.C01.14_A_105

TCATTATTTCTAAAACAACCATTAAATGTTAAACCAGATCT

>NL01B.C01.14_A_106

ATTTAGTGATAACTGCACAAGGAAAAACTGTAAAAGAA

>NL01B.C01.14_A_107

GAAAGATCACTATTCCTACCCCCATCGCTTCTAACATTG

>NL01B.C01.14_A_108

CATAGCAAATTTTCGGTGCTATTAGATTGATTGGATATGTTACT

>NL01B.C01.14_A_109

CCATAGCTCTTCTATCTCGTCGCTGTAATAGGTAAGCATCA

>NL01B.C01.14_A_110

GGAGTAATAACTGTAAATAATGGAGTATCACTTATAAATAC

>NL01B.C01.14_A_111

ACACTGGGTATAAGTGTTGTAGTACTACCCATCCTCTG

>NL01B.C01.14_A_112

GCTGGCGACATTAACACACTGGTTTGCTGACATTCTCCAG

>NL01B.C01.14_A_113

TAGGTAATTCTGAATTTTTTAAAACTATAATTGAATGAGG

>NL01B.C01.14_A_114

GTGGGCATAATTCACATGCATGGCAATCTCAACATCAACAAA

>NL01B.C01.14_A_115

GTACAACATAAATATAACATACCGGTACAACCAATAAGACTA

>NL01B.C01.14_A_116

AACGGATGAAGAAGCAATAATATTATACGCGTCACACTTATT

>NL01B.C01.14_A_117

ACTTTATCACAAATATTGAAATCTTTGCAAACTTTCTCTC

>NL01B.C01.14_A_118

GTGATGATTGTACCGGGCATTATATACTCTTCTGTTACTA

>NL01B.C01.14_A_119

AAAAAGGAGACCTGACCTCCTCCTCACCCTGAAGGCGAG

>NL01B.C01.14_A_120

CTCGGTTCCGCACCTACCACTGCGAAGAGGTATTCCTTTTT

>NL01B.C01.14_A_121

CAAAGAAATATTAGATGAACTTGGAAAACAAGCTAAAGAAAA

>NL01B.C01.14_A_122

TAAATGCATTGAATAATTTTTCGGCCTCATCTCTCTCAA

>NL01B.C01.14_A_123

TTAAAGACGATGAAGACCTTGGTGGCTAAGTGGTATAACA

>NL01B.C01.14_A_124

CCTCATAAATTAGAATTGCTGAGGCACTTGTTAAGCTTAAGCT

>NL01B.C01.14_A_125

ATTTCTTGCTATCTCAGTACCAACAAAATTATGGCAACGT

>NL01B.C01.15_A_1

ACCTTCTTACCCATAATTATTATATTCATAAAATGTTAA

>NL01B.C01.15_A_2

CAAAACAATCCGTTTTCTAACTGCTTAATTGCGGAAGCCA

>NL01B.C01.15_A_3

CAAGACGGAAAAGTAAGGTATTCCATGACTATCCATTCGC

>NL01B.C01.15_A_4

AAACTTATTCAGACCATTAATTGGCAATTATACGGCGTTTA

>NL01B.C01.15_A_5

AAAGAGTATTCCCTTCAACATATGCTTTTTTTCAGTTAAACTTT

>NL01B.C01.15_A_6

CATATTCAAACTCTCTAGTTTCATCATTAATATCATTAGTACT

>NL01B.C01.15_A_7

AATAAAACAATGGATATAATAGTTCCAAGGGGCATAACAG

>NL01B.C01.15_A_8

ATCAATGTGTACTCTCCGAATTCTTTTTTTATCTCTTTCT

>NL01B.C01.15_A_9

CGAAGTGACGGTGAACGTTATGACCTGGACGACGATTATG

>NL01B.C01.15_A_10

TTTTTCAATATCTTCTAATGCTTTATACGCACTTTCAACG

>NL01B.C01.15_A_11

TCTTTTATTGACTGTTCTATTAATTGACTGATTTGTTCA

>NL01B.C01.15_A_12

CCGGAATTGGCGTTACACCTTGATGAGATTTTTTTATCAA

>NL01B.C01.15_A_13

AATTCATATATGTAGAAATAACGCGATGACGGTTCATTATA

>NL01B.C01.15_A_14

TTAATTGCTGACGATTTAAATAAACTCATTGACGCAATTGGT

>NL01B.C01.15_A_15

AATGCCATCTATCTCACCCGTGCTTGTGCTTCTTCTTGTGA

>NL01B.C01.15_A_16

GGCTATGTACTCGTAAACGGTGTGCGAATAAGGAGGAAAAT

>NL01B.C01.15_A_17

CTAATAAGAGAATTGTTAAAAGGGTTTAAAAGTTTTACG

>NL01B.C01.15_A_18

TCATACGTTATCTGGTCGCATACGCTTTCACATCTTACCA

>NL01B.C01.15_A_19

CCCTTAATGATTAAAAGGTTCCTGTCCTCGTCTACTATTATTCC

>NL01B.C01.15_A_20

CTCACAAATCTGGTTTAAATCGCTTTCCTGTTGCTGTAA

>NL01B.C01.15_A_21

ATTTCTCTTGCTTCTTTCAAATACTCCAAAAGTTTAGGTG

>NL01B.C01.15_A_22

TCAACTACAGTAAGCGACCCACCACGACCGGAAGTCATTAAC

>NL01B.C01.15_A_23

TCTGATTTAGTCGAAAAACTGAAGGCGGGTAAACTGGAGACTG

>NL01B.C01.15_A_24

CTTTCCAATTCTGAATTTCGTCAAAAATTAGTCCGTTAG

>NL01B.C01.15_A_25

GTTTAAAACTTTTAGTTACTAAACATATTCGTAATTTATTTAAAT

>NL01B.C01.15_A_26

ATAGCCTTCTTTCTCTTGTAAATTTCTTCAATCACTTCCGC

>NL01B.C01.15_A_27

TTTAGTATTACATCTTTGTTTGTACATTTTAATCTCCTCA

>NL01B.C01.15_A_28

TTTTTGGTCAGTATATTTTTATGCCAACCCGGTATTTA

>NL01B.C01.15_A_29

TCGCCGCCCCCGTGAATTTTTGCAAACCGAGAAAAGTGAGT

>NL01B.C01.15_A_30

CAAAACATGTATCCCATATACTATATCCAAGTTCTGAAACAGC

>NL01B.C01.15_A_31

TCACCTATGCCATAGTCTTTATAGTGCTGATTGGCTAAA

>NL01B.C01.15_A_32

ATTAAAGATGTAGACAAAGCTATAGATTTTTATAAACAATACTCT

>NL01B.C01.15_A_33

AGTATACCTGAAGTTAGATTGGGACAATCGGATATAAA

>NL01B.C01.15_A_34

ACTGTCATAAACGAATATAGAAAATACATTGACTATTTT

>NL01B.C01.15_A_35

TTGCGAGAGATAATCACATATGTACCTTGTAGAGAATGC

>NL01B.C01.15_A_36

TTATTATTATTTACGATTGCTAATGAGTAAAGTGGAATATTG

>NL01B.C01.15_A_37

TTATTTCATCAGGACTTAATAATTGATAAACTTCATCAC

>NL01B.C01.15_A_38

TGAACTAATGAATTCTGGGTACCACTTTTACCCTCTTATACT

>NL01B.C01.15_A_39

AAGAAGGTACAACAGACAAATACATTGTGTTAACAAATCTAA

>NL01B.C01.15_A_40

AATAAATAAAGCTTTAATTGGAACATATCTGCAAAAAGCAAT

>NL01B.C01.15_A_41

GTCAGCTGTCTAATTCTCTCAATTTCTGAAACTAGCTTTT

>NL01B.C01.15_A_42

AAATACACCTCTGCGAAAGCATTACCGAAAATATACTCAGGT

>NL01B.C01.15_A_43

GTGATTAACAGTCATCGTTGGTACCCAAACATAATAATCCTAA

>NL01B.C01.15_A_44

CATCTCCTATATCCGTAACATATAACGAAACGACACTAACTTT

>NL01B.C01.15_A_45

ACAGCTTGCACAAGTTTTGCCAAAAATAGTTAGTTCTCAGT

>NL01B.C01.15_A_46

TGAGCAGTTATCGGTATATTGTTCTTCAGTGCTATAGCAATCT

>NL01B.C01.15_A_47

ATAATGAGTATTTGAATGCGTTAGCTGAATTTGATAGAACTGG

>NL01B.C01.15_A_48

CTTCTTCGGACGAGTTGAAAAGATAATAGTATGTATCTGG

>NL01B.C01.15_A_49

TCATCTCCAAGCAGTCCTACCATAAGACTACCAAAAAT

>NL01B.C01.15_A_50

GACAATATGTGTTACATTATCGTTCCGTTACGACGAG

>NL01B.C01.15_A_51

GCAATAATAGATAAATTAGCTGAAATTCTTCCTGAGAT

>NL01B.C01.15_A_52

TCAGATGGTAACACTCTGATGGGTGGGGTTACCCCGCTA

>NL01B.C01.15_A_53

TAAATACATTGAATAATTTTTCGGCCTCATCTTTCTCAAA

>NL01B.C01.15_A_54

CTTATTCATGCTAGAAAAGGACTTCCTTTGACAGCTGAT

>NL01B.C01.15_A_55

ACACTATCACAAACACTTCGATCACAGTCCAAAACACA

>NL01B.C01.15_A_56

TTTAATAGTATATGTTTGTTTCCACAACTTAGTCTTCTT

>NL01B.C01.15_A_57

AGATGAAGGTAAGTAGATGTCCTTTGTAATCTCTCCA

>NL01B.C01.15_A_58

AAATAGATTTAATGACTTCCAACCGACAGGATAACTAA

>NL01B.C01.15_A_59

CGTCTTTAAAGCGGGGAGGAGATCAGCACATAAAATAAAG

>NL01B.C01.15_A_60

GTAGGTAGCACAATTACAATCAAATATGCGAACGGCTCCA

>NL01B.C01.15_A_61

TGGAAAACTGGCAATTCAACATTTAGACCTACTTCTTGAA

>NL01B.C01.15_A_62

TTATGATTTTGCAGATATTCTAAAACTTTTTCTTCTTCTT

>NL01B.C01.15_A_63

ACATCAAACCAAGCAACATACCTCATAGTGATTGGGC

>NL01B.C01.15_A_64

CCCTTTAGGGCGGGGGTTCCCCGAGGTCTCAGGCGTTACACCCCT

>NL01B.C01.15_A_65

ACATCACGTATCTCTGCTAATAAAATATGGTCTATTGAACA

>NL01B.C01.15_A_66

TCTTCAAGTTAATGTTAAAGAGTATCACAAATGTTGAGTAC

>NL01B.C01.15_A_67

TTAGATCCAATATGGCTTATAGTTATTGTCGGATTTTTT

>NL01B.C01.15_A_68

ATGTATTGGGGTCTTCCCATGCGACCCCAAAGGGGCTATA

>NL01B.C01.15_A_69

TCTTATGGAGTTGACGCAAAATACATCAAAAAGCATGAAG

>NL01B.C01.15_A_70

TTATTCAAATCTCCAGTAACGAAAAGACAGATAAATTATATCGA

>NL01B.C01.15_A_71

TTTCTGTCCATGCGTTTTATCGTAATATTCTTCAACTTCTG

>NL01B.C01.15_A_72

GTTATTATATTAGTAACTGCGTCTAATACTGTAGGATTTGCA

>NL01B.C01.15_A_73

GCGATAATATTGGGTATCATAGTTAGGAGGTTCGGTTGAT

>NL01B.C01.15_A_74

GAAGGAATTGAAGGATAGAAAAAACAGATGTCATTACGTA

>NL01B.C01.15_A_75

TCTTCTTGACTTTCTACCTTCTTACCCATATTTACACCCCTT

>NL01B.C01.15_A_76

CGAGTCCAAATACAATTTTCAATTCCAGTTGACAGAG

>NL01B.C01.15_A_77

ATATACATTTCTCTTTCCCCCTTTATAAATTTTTCTCT

>NL01B.C01.15_A_78

AATGAAATGTCCTTTTTAACAACAGCGGGATCCAGTATAACT

>NL01B.C01.15_A_79

TCTCCTATATTACCTATGCGTACATGTATTCGCTGTCCTT

>NL01B.C01.15_A_80

TCAGGAAATTGAGCTAAAAAATCTAAAACTGCTTTTTCTTTT

>NL01B.C01.15_A_81

ATGACATTTATATCACACATTGAATTAGATAGATGATATTTCT

>NL01B.C01.15_A_82

ATTCACCTTATCCAAATCAATCCCTAACATATATTTTAAAA

>NL01B.C01.15_A_83

TTAATTTGTTCTAACTGAATTGAAAAAACTAACTTAATTT

>NL01B.C01.15_A_84

GCTTATGTGCATATCCACTATTATTGAAGTTTGCTCTTGCTTGT

>NL01B.C01.15_A_85

TTAATAAAACTGCTTTTCTTCTAAATTGTAAATGATAATAT

>NL01B.C01.15_A_86

TCTAGTGCGTCTGCACTTAGTGTCATTTCAATTATATTTTTAG

>NL01B.C01.15_A_87

GATTATATAATATGAATTATTTGCTTAAAAAGTAGTCGAGA

>NL01B.C01.15_A_88

GTCACTTTTCACTTTACCTTCTTTGAAATTTGTTATTTT

>NL01B.C01.15_A_89

TTTAGTGCAAAATTTAGCATTTGATAAAATCTTACCTGA

>NL01B.C01.15_A_90

TGCTTATCTTGAAAATACGGATATTTCTCATCTTTGTAAT

>NL01B.C01.15_A_91

TTTTTTGTCTTGAGGATTAAAGTTAGCAATAGATTTAAC

>NL01B.C01.15_A_92

TTAAGTGCAAATGCATTAGTGAGTTTAAAGAAATTGAT

>NL01B.C01.15_A_93

CTTATTATTCGTTGTATTTACGCTTCGTAACAACAGTATACT

>NL01B.C01.15_A_94

GTACGTTTTTCGATGACATTCACCATCCATATACTAATA

>NL01B.C01.15_A_95

GTGGTGTTTGCTCGGCATTGTTTATTGCAAAATCGACGTG

>NL01B.C01.15_A_96

ACAGTTGTGCTTGCAGTCGTTGCTTTGATTTATTCTGTCTG

>NL01B.C01.15_A_97

TTTGCCATTTTTGGTCAATTAATATTTAAAATTGCTACAA

>NL01B.C01.15_A_98

ATTTATTTCAATACTGCAGAGATAAATTGTTTCCTAA

>NL01B.C01.15_A_99

GATTAACATTTATTACAAATTTCCAAAGTTTGAATTTGC

>NL01B.C01.15_A_100

AGCATGTTACAAAAGCCTATAGCAGATTAATGCAAATGT

>NL01B.C01.15_A_101

AAACCACCAACACCTATAATCATAAAAAGTTTATCTTCCC

>NL01B.C01.15_A_102

TAATGAGTTATGATGTATGAACATCTGACTTTCTTGAGATTTGA

>NL01B.C01.15_A_103

CTTTTTAATGCATCATCTATGATGTCAGCTATTCTGTCAA

>NL01B.C01.15_A_104

TTCTTTTGCATTACATATAGTCTCTTCATCATTACTGCAT

>NL01B.C01.15_A_105

TTATTGTTGTTATACAACCATCATTACCTGTAGCATATGC

>NL01B.C01.15_A_106

AGCATTTTATACCTAAATAATGAAAGTCGGGTATGATTGG

>NL01B.C01.15_A_107

AATAAACGCCAAGAATTTAGTAAATTCAGGGAATTTC

>NL01B.C01.15_A_108

GAATTTATCATATATGATGTCTTTGACAAAGAAGATAATAGA

>NL01B.C01.15_A_109

TATATCAAGGTTTCGGTCTTAAGGTAGCTAGGGCGCTTAAT

>NL01B.C01.15_A_110

TTGAGTATATGGACTTTAACGGTAACTGGCATACAAATAG

>NL01B.C01.15_A_111

GGATAGACATGTTTGTATTCTTCACAAAACCACTTAAGTAAG

>NL01B.C01.15_A_112

ATTGTAATTGGTGCAATAAACGGCGTTAGAAATTGAGTAA

>NL01B.C01.15_A_113

AGATAGAAGAATAATGCAATTTGCAAAGCTAAGCTAAT

>NL01B.C01.15_A_114

TAATTTTTCAAAAAATATATCATTTTTCTCTCTGTCGTA

>NL01B.C01.15_A_115

TTCTTTTGCAAATTCTGTAACCTTTTCGCCATGACAGAA

>NL01B.C01.15_A_116

CTTCTTGACTTTCTACCTTTTGACTTTTACCCATTTT

>NL01B.C01.15_A_117

AGTTTGCAAATATGTAGGATTTAAATAATTATTTACTAAA

>NL01B.C01.15_A_118

ATAATAATTGATTATTATGGAACACAATCAACTAGTCCGT

>NL01B.C01.15_A_119

TCCCAAGGCTGGGAGGGTTTTCGTCTCGTCATCAATCAGG

>NL01B.C01.15_A_120

TCAAGAAGTTTATAATCTCTTCATTATCGAACACCTTCCTG

>NL01B.C01.15_A_121

ATTGTGTATGTATGAAGGTTGTAGAAGAAGGATGGGCTG

>NL01B.C01.15_A_122

TTCTTTATTCCGAACCTAGAAAGCCCGCTGAAAAGCGG

>NL01B.C01.15_A_123

ATGTTATTTGCACGCTTTTCCAACCGACAGGATAACTAA

>NL01B.C01.15_A_124

TATTGGATTTAATGGAGCATTTAAATTCATATTTCCACACCA

>NL01B.C01.15_A_125

GTTGATATAAACGTCTGTGAAAAAGTAAAAATATATGATAG

>NL01B.C01.15_A_126

TATTCCATGCCCAAGATTCTCCAGACGGACTAGTTGATT

>NL01B.C01.15_A_127

ATATTGTACGTTAAAACAGTGCTAAACTATGATGAATCTA

>NL01B.C01.15_A_128

TTAATGAAGCTGTTTCCTTAACGTGTTCTGGTAATTCCA

>NL01B.C01.15_A_129

ACACACGGTGCAGTATCGTATGCTTCTAGAATTAATTT

>NL01B.C01.15_A_130

GGATTGACATAGCTAGATGGGAACGATGTTACGTGGATAG

>NL01B.C01.15_A_131

ATTTTCACGCTGTTAAGTGTCGGGACGGCCAAGCCGA

>NL01B.C01.15_A_132

AGCAAATTATAGATGTCTTGTGTTGTTCTCATCTGCTTTT

>NL01B.C01.15_A_133

GATTATTTCGCTGATACTAACAGATCACTGAGAGAACAGA

>NL01B.C01.15_A_134

TTATACTCTTTAATTATGAGATAGTATTTTGACTCATTTCTC

>NL01B.C01.15_A_135

GGGGATTAGGTTATGCTCCTGAAGATGCATTGGAAGATGCAGCT

>NL01B.C01.15_A_136

ACTTTACGCGAGGAATGAGGTGAATGAGGAACAGCTGATG

>NL01B.C01.15_A_137

GTGATGGGATATGGCATCGTTTCAGTAATTTCTACATGTGCCGT

>NL01B.C01.15_A_138

TCCCTACAACGAGTTTCATTTACTTTACTTTATGCCTCCAT

>NL01B.C01.15_A_139

AATATCGTTATGAATTTGTCGTTTGGTAATGGTCGTTTGC

>NL01B.C01.15_A_140

CCTGCGATGTCGACCAGAAAGACCTGCGTATAGAAAAAAG

>NL01B.C01.15_A_141

ACAGGATAACTGAAAACGTATATTATCTGGCGTTTTTGGC

>NL01B.C01.15_A_142

ACTTCGCTTTTATATGGTATAAAATCATTATTTTGTAG

>NL01B.C01.15_A_143

CCGTCACCTCCACCACCTGTATTCTCTTCAACACAGAG

>NL01B.C01.15_A_144

ACTTATGCCAATTTAGTCTATGATGCTAGAAATAATATG

>NL01B.C01.15_A_145

GTCACTTAATAATAGTTGGAAGTTAACCGTATCCCCATAAT

>NL01B.C01.15_A_146

CATAAGATCCGCTATCTCGTTTAATAGTAGTGCTAATTCTA

>NL01B.C01.15_A_147

AAGGAGTTGTGGAAACATCCAGTTGAAGGCCTTCTATAGT

>NL01B.C01.15_A_148

TCATTAATTTCCTTGAATTTACCATTATGATATTTGTAA

>NL01B.C01.15_A_149

ATAGATATATTTCTACATGACCATATTTTGCATATAAATGTCT

>NL01B.C01.15_A_150

CTATATGGTGCTACTTGATACTCAGTCTGATTTTCTGCCTG

>NL01B.C01.15_A_151

TTTTTCTCCTCATTATATCATTTACAAAAACACATATATAA

>NL01B.C01.15_A_152

GAATAAAGTGGACTTGGAGTCGTAGAAACATCTAACTGTAAG

>NL01B.C01.15_A_153

ATCTTTTCACAACTGCCTACAATCACGGTAAAAAGTATA

>NL01B.C01.15_A_154

ATTTTTCCCTTTGTACTCTTTAACTACCAAATAATATTT

>NL01B.C01.15_A_155

ATTTATATCGCTGGATTTATAGCTACTGCAAATCAGCAA

>NL01B.C01.15_A_156

TATAGCTACTGCAGGTATCAATACGTATTACTTGCCTCTG

>NL01B.C01.15_A_157

ATGGATACATATTTCTTTGAATTCTCCTTCTGCTCTCATTTACCT

>NL01B.C01.15_A_158

TAACTTCTAATCCGAAATCTTGACACATATTTATTATACGTCT

>NL01B.C01.15_A_159

ACAACATAGCTGGCAATTAATACGCCCATTAATGTTAGA

>NL01B.C01.15_A_160

AACGGAAATTCTGTAAGATATTCAATGACTATACATAGCCAA

>NL01B.C01.15_A_161

TTTTTTCGTAAAACTTGTGCAATATCCTCTGAAACATTTCG

>NL01B.C01.15_A_162

TTAATGTAAGATTTTAAAAAATGACAAATATAAATCATTC

>NL01B.C01.15_A_163

CTTCAGGACTCAAATAGGTAAAGTTAATTTCATTTCCATCC

>NL01B.C01.15_A_164

AATTGCCTAATTACATAATTTCCTAATTTCTCCTCCAAT

>NL01B.C01.15_A_165

TAACATTATCTATTACAGACACGTCCGCTATGTGTTGGCGG

>NL01B.C01.15_A_166

AAAAACATAGTAGAAATGTTAATGACCAACAAAAGTGAAT

>NL01B.C01.15_A_167

GTTATTCCATATTCCGACAAAACTGATGCTACTTGTTCATT

>NL01B.C01.15_A_168

ATAATATATTATTAAAAATATGAACATCAAAAAAAGAACGTCCA

>NL01B.C01.15_A_169

TCCCGTAATGATACGCATACATAGTCTGGTAGTTCAAAA

>NL01B.C01.15_A_170

TATATTCTTTAATTAGTAAATAATACCTTGATTCGTCCCTCCC

>NL01B.C01.15_A_171

ATAATTTCTAATCTTAAGTCGCTGATTACGTCCATCCAGACT

>NL01B.C01.15_A_172

AAAAGTTTGGAGGATAATTTTGCTGAAGGTCACATTTATCC

>NL01B.C01.15_A_173

CCAAAATAACTACATGTACTTGGTGTTATAAATCCTACAGT

>NL01B.C01.16_A_1

ATATCTTGTGTATGTTACACTTAAAAAAGTGGTCAAACAG

>NL01B.C01.16_A_2

TCTATAAGCATTCCAAAAAAATAACCAAAGAAAATAATTAA

>NL01B.C01.16_A_3

AAAGAGTATTCCCTTCAACATATGCTTTTTTTCAGTTAAACTTT

>NL01B.C01.16_A_4

GCTGAATTCTCCGCCAGGATGACGCTGAAGGTAAATGGGT

>NL01B.C01.16_A_5

CATCTTTTTCTCCCCTCCCTATAATATAACACATTCTGGTTTT

>NL01B.C01.16_A_6

CGAAGTGACGGTGAACGTTATGACCTGGACGACGATTATG

>NL01B.C01.16_A_7

TCACGTTTTACCACCTTTTACAGCTTTCGTTACTTCT

>NL01B.C01.16_A_8

TTTTCTCCCTATTCCTGGTAATTGTACTAACGCTAAAGCT

>NL01B.C01.16_A_9

TTAATTGCTGACGATTTAAATAAACTCATTGACGCAATTGGT

>NL01B.C01.16_A_10

ATGTTTACATGCAGTTGTGATGGTGGTATGATATTGACAG

>NL01B.C01.16_A_11

TAAATCTGCGTAATTTTTACACTTTCATCTTTCCCCTTGT

>NL01B.C01.16_A_12

GAGGGGAAGGTCTTGAGTACACAAGAAATCAGGGAGATGTATAA

>NL01B.C01.16_A_13

TCTCGTGTATCTCATTTACTTTATTATCGTTAAGAGATGT

>NL01B.C01.16_A_14

AACTTTTTTGCTAATTCCTCCAATTTGATACTTTCTTCCTTA

>NL01B.C01.16_A_15

TTTATACTGCCGTTAAATATGACGGTGCGTTTGAAGG

>NL01B.C01.16_A_16

TCAGCATACTTCCTAGCCTTACAGAACAACTATACTCAAG

>NL01B.C01.16_A_17

TTTTTGGTCAGTATATTTTTATGCCAACCCGGTATTTA

>NL01B.C01.16_A_18

TCGCCGCCCCCGTGAATTTTTGCAAACCGAGAAAAGTGAGT

>NL01B.C01.16_A_19

ACTAACATTATTAAATGCTTTCTTCTGCCTTATAAACTTTTCTTTC

>NL01B.C01.16_A_20

TGCTGTTAGGTATTGTCCTGAACACGAAGCCGATGAAGAA

>NL01B.C01.16_A_21

TTTTTATGCAAAAAAGAATAATAAAAGTCTGTCTGACTCT

>NL01B.C01.16_A_22

TGAACCTATCGAGGAATGCTCTGTCCGCTTGTGTGTTTACCCT

>NL01B.C01.16_A_23

AAGCTGCAGAAATGTGCAGTACAAAGGTTAAGGGTCTGCC

>NL01B.C01.16_A_24

TTATTATTATTTACGATTGCTAATGAGTAAAGTGGAATATTG

>NL01B.C01.16_A_25

CTATAGGTGTTGAAATGAAATATTCTACTAGACCGAA

>NL01B.C01.16_A_26

AGAGAGATTATAGTTCAATTGGCGACGATGTGAGGGGAG

>NL01B.C01.16_A_27

CCATATAGTGTATATTGGGTTGATACTGCATGGAGTAAATACAAT

>NL01B.C01.16_A_28

TTTGAATCTTTCTTCAATTAGTAATATTACTGTATCACTTT

>NL01B.C01.16_A_29

TTTACAATGACGCCGTTGTTATAGGAAAGTTTAGTCCCCG

>NL01B.C01.16_A_30

AAATAGAAAGTAAGTTTTATTCATAAGTAATCCCCACCCAAAT

>NL01B.C01.16_A_31

ACAGCTTGCACAAGTTTTGCCAAAAATAGTTAGTTCTCAGT

>NL01B.C01.16_A_32

AACCGAGGAGCGGGAAAGTGGTCAGGAAAAGGATAGAGTACA

>NL01B.C01.16_A_33

TGAGCAGTTATCGGTATATTGTTCTTCAGTGCTATAGCAATCT

>NL01B.C01.16_A_34

ACAATCCACCATATGCATATCCGTTTTCAATGTCAGTAA

>NL01B.C01.16_A_35

ATAGTATTTCAGCTATCCTAACGGTTAATAGTAAAGCGGTG

>NL01B.C01.16_A_36

CAATACGCCTATAACCACTCTTTTTCACTTTCGCTATAATACACT

>NL01B.C01.16_A_37

CTTCTTCGGACGAGTTGAAAAGATAATAGTATGTATCTGG

>NL01B.C01.16_A_38

AGGATTGAGAAACAACACATCGATTTTGCAATTGCACATAT

>NL01B.C01.16_A_39

ACATCACGTATCTCTGCTAATAAAATATGGTCTATTGAACA

>NL01B.C01.16_A_40

GCAATAATAGATAAATTAGCTGAAATTCTTCCTGAGAT

>NL01B.C01.16_A_41

TTACCCTCAGCTGTTTGGTCTCCTATGTTAGGTGAAAATA

>NL01B.C01.16_A_42

AATAATATTTCGCCTCATTTCTCCCTTCTTTTATTGACTGTTC

>NL01B.C01.16_A_43

TATAAATCATGAGTTTTTCCACTAAAAAAATAATGAAACA

>NL01B.C01.16_A_44

CCCTTTAGGGTGGGGGTTCCCCGAGGTCTCAGGCGTTACACCCCT

>NL01B.C01.16_A_45

ATCTCCTACAACCTATCGGTGCTTATAGGAGTAATACCTA

>NL01B.C01.16_A_46

TATTAATTTCCGTAGTAATCAATTCTGTAAACTTATTAA

>NL01B.C01.16_A_47

TTGAGTATATGGACTTTAACGGTAACTGGCATACAAATAG

>NL01B.C01.16_A_48

GAGACCTTGATAAGATTATTACTACGCGACGATTTG

>NL01B.C01.16_A_49

CCGTGAACGGCGGGACGTAAACCACGGGGTTCGTAACTTT

>NL01B.C01.16_A_50

AATTATTTCTTTAAATGCTGAAATTGTTGAACAAATTG

>NL01B.C01.16_A_51

TTTGGTATATGACAGGAGGCATCCTCTATTAGAGATGGCG

>NL01B.C01.16_A_52

TCCTTTATTAATGTTTGCAAGAAAGCACCACCATATACGG

>NL01B.C01.16_A_53

TCTTATGGAGTTGACGCAAAATACATCAAAAAGCATGAAG

>NL01B.C01.16_A_54

TTTCTGTCCATGCGTTTTATCGTAATATTCTTCAACTTCTG

>NL01B.C01.16_A_55

TTGCCAACATGTGTCCTGTAGTTCCTAGGTTCATTG

>NL01B.C01.16_A_56

GCCCCTCCTCCTAAAGGCATTCCTTGACTATGATAATAA

>NL01B.C01.16_A_57

ATTTCTCTTGCTTCTTTCAAATACTCCAAAAGTTTAGGTG

>NL01B.C01.16_A_58

TGGAAAACTGGCAATTCAACATTTAGACCTACTTCTTGAA

>NL01B.C01.16_A_59

ACTTCTGTGTATCTGTTATTGCCAATTGTTATTGTAATTTT

>NL01B.C01.16_A_60

ATACTTTTGCATACAGGTTATCTACAGCAATTACTAATTC

>NL01B.C01.16_A_61

TAATGATGTCAAAATCGGAGTCGTTGTCGTTGTTGTAG

>NL01B.C01.16_A_62

CCTGCGATGTCGACCAGAAAGACCTGCGTATAGAAAAAAG

>NL01B.C01.16_A_63

GTTTAGGAATTAATAATTGCAATTGTTTACTAACTTTAGAT

>NL01B.C01.16_A_64

CAATTTTTGGAAGTTTGTTTAAATTACATTTATATGATTCT

>NL01B.C01.16_A_65

TAAGCAGGAACAAGTAAACACGCAACAATTATCCAACATACTG

>NL01B.C01.16_A_66

CCGTTTATTGGAATTTTTTTAGTCAAAATGTAATACATTAC

>NL01B.C01.16_A_67

CTTCAGGACTCAAATAGGTAAAGTTAATTTCATTTCCATCC

>NL01B.C01.16_A_68

TAATCTACCGAAGCCAGCTAACTGTGATATTTGGATTA

>NL01B.C01.16_A_69

CATAATGTAGTTCATAACTTGCAAAATACAAATTCTTTAT

>NL01B.C01.16_A_70

TTCATGGTCAGAAACCAACAAACAGCCTAAGTTTTCCAG

>NL01B.C01.16_A_71

GTCACTTTTCACTTTACCTTCTTTGAAATTTGTTATTTT

>NL01B.C01.16_A_72

ATCGTAGAGTACGGGAGATTTATAGGATGGAAACAAATAACACC

>NL01B.C01.16_A_73

AATTGCCTAATTACATAATTTCCTAATTTCTCCTCCAAT

>NL01B.C01.16_A_74

ATACCCTTATTCGGTAACAAAGCGTAATGGCGTTGATAATG

>NL01B.C01.16_A_75

AATTCGTTAAACCAAGCTTTTACCATTTGCTTTCATCCCT

>NL01B.C01.16_A_76

TGCTTATCTTGAAAATACGGATATTTCTCATCTTTGTAAT

>NL01B.C01.16_A_77

TGATTGAAATACCCAAATGCCACACCGTCAGAGTTATTAGCGA

>NL01B.C01.16_A_78

GAGACCGGGAGAAGTAAACAAAGAGTTTATTTAAAACAATA

>NL01B.C01.16_A_79

GTGGTGTTTGCTCGGCATTGTTTATTGCAAAATCGACGTG

>NL01B.C01.16_A_80

CTATTATGTACTTAAACTTGTCTTTATAGTTGTCATCGTCGTC

>NL01B.C01.16_A_81

ACAGTTGTGCTTGCAGTCGTTGCTTTGATTTATTCTGTCTG

>NL01B.C01.16_A_82

AGCTATTAATATCAATAGAAGACTTATTCGATGTATTTGTGTTG

>NL01B.C01.16_A_83

GATTAACATTTATTACAAATTTCCAAAGTTTGAATTTGC

>NL01B.C01.16_A_84

AAACCACCAACACCTATAATCATAAAAAGTTTATCTTCCC

>NL01B.C01.16_A_85

ACCGGGTTGCTGATGTAAAGGGAGTCTTTAATGTATGTTT

>NL01B.C01.16_A_86

TTCTTTTGCATTACATATAGTCTCTTCATCATTACTGCAT

>NL01B.C01.16_A_87

AATAAACGCCAAGAATTTAGTAAATTCAGGGAATTTC

>NL01B.C01.16_A_88

TGATAGTTCCTTATATTCATTTTCCGACATTGACAACGCA

>NL01B.C01.16_A_89

GGATAGACATGTTTGTATTCTTCACAAAACCACTTAAGTAAG

>NL01B.C01.16_A_90

AATTCCTTGTTCAACTGCTCTTTGTAAAAGTCTAGCAGTTT

>NL01B.C01.16_A_91

CTTAGGACTTACAGAGAACTCGCAACTAATCTACTCAACAAG

>NL01B.C01.16_A_92

AAGGGCTAGTAGTTGACTTGGGTGTACTAGTGTTACCAT

>NL01B.C01.16_A_93

ATTTATGCAGTTCTGCTAAATCAAAAGGATTATCAACATCT

>NL01B.C01.16_A_94

CTATTATTGAAGTTTACTCTTGCTTATGTGCAGACCCATT

>NL01B.C01.16_A_95

AGGCATATCTGCTCGTTGATGATATTGGGATGGTCATGTATAT

>NL01B.C01.16_A_96

TCCCAAGGCTGGGAGGGTTTTCGTCTCGTCATCAATCAGG

>NL01B.C01.16_A_97

CATCTCCTATATCCGTAACATATAACGAAACGACACTAACTTT

>NL01B.C01.16_A_98

ATATTGTACGTTAAAACAGTGCTAAACTATGATGAATCTA

>NL01B.C01.16_A_99

ACACACGGTGCAGTATCGTATGCTTCTAGAATTAATTT

>NL01B.C01.16_A_100

GTCAGCTGTCTAATTCTCTCAATTTCTGAAACTAGCTTTT

>NL01B.C01.16_A_101

GGAACTAACGGTATCATAGGAGCGTCTTTCGCATTTTAT

>NL01B.C01.16_A_102

GGGGATTAGGTTATGCTCCTGAAGATGCATTGGAAGATGCAGCT

>NL01B.C01.16_A_103

ACTTTACGCGAGGAATGAGGTGAATGAGGAACAGCTGATG

>NL01B.C01.16_A_104

TGATATAGTCTATGGTATCGGGGTCAATAGGCTCTCCACATT

>NL01B.C01.16_A_105

TCCCTACAACGAGTTTCATTTACTTTACTTTATGCCTCCAT

>NL01B.C01.16_A_106

TCTTGTTGTTTTTCTCCTTTTCCGCTTTGCTTCTTTTCGCCT

>NL01B.C01.16_A_107

TGAATTACTTTCTCATTATCCACAGTTTGTGAAATTTGG

>NL01B.C01.16_A_108

GAAATTTTTAGACCTTCAATCTCACCTTTTATCTCAACTTCA

>NL01B.C01.16_A_109

CCGTCACCTCCACCACCTGTATTCTCTTCAACACAGAG

>NL01B.C01.16_A_110

CTAGATCAGGCAAGTATTTATTTTCTATGTCATACAACACGT

>NL01B.C01.16_A_111

TAAAGCCCAATTTGAAGCACTAGAAAAAGCAAAAAGGTGAAAA

>NL01B.C01.16_A_112

CAACAATATGACGGCAAACATTATCTCGAGGGTGGGGATTG

>NL01B.C01.16_A_113

ATCTATCATACCCTCATTCTCATAGTATACATAGTATGAGACAA

>NL01B.C01.16_A_114

TCATTAATTTCCTTGAATTTACCATTATGATATTTGTAA

>NL01B.C01.16_A_115

ACAGTGTCTTCTGCAAATTTTTCTAGCTTAGCTAATGCTA

>NL01B.C01.16_A_116

AGATGACGTTACTTTATATATGATCACGAGACCACCTTTGG

>NL01B.C01.16_A_117

TTTTAAAGCTGTGCGAAAAAGAAAAATTTAAAAATTGTGA

>NL01B.C01.16_A_118

ATTTATATCGCTGGATTTATAGCTACTGCAAATCAGCAA

>NL01B.C01.16_A_119

TGACATTATAGGTTTGGGTTTGTGTGGTTTGGGATTCG

>NL01B.C01.16_A_120

TAATTGTTTCGAATACTTTGTAATATACATTAAGTAATAAG

>NL01B.C01.16_A_121

AGTGAAATTCAACTAACTGCACAAACTCCAACTTTACTAAG

>NL01B.C01.16_A_122

TTAATCATATTTACTAGAGCAGGCCCGCCCCCAAGTCTGTTT

>NL01B.C01.16_A_123

TTCTTCAATAAACAATATCACTACATCACTTTTGGGAATA

>NL01B.C01.16_A_124

TTTTTTCGTAAAACTTGTGCAATATCCTCTGAAACATTTCG

>NL01B.C01.16_A_125

TTAATGTAAGATTTTAAAAAATGACAAATATAAATCATTC

>NL01B.C01.16_A_126

CATAAGATCCGCTATCTCGTTTAATAGTAGTGCTAATTCTA

>NL01B.C01.16_A_127

TCCATGTAGTATTTTCTAGATAGTTCGACAAACTGTTTCTC

>NL01B.C01.16_A_128

CAGTTAATAACCCAATTACCTATCTTAACGTACACTTCTT

>NL01B.C01.16_A_129

CCTGTCAGGAGCGTCGTAGTCATAGCAGACGGGGTTTATGT

>NL01B.C01.16_A_130

GATTTAGTTGTGTGGATCTCATTGTAATAAGAGTTAACGTG

>NL01B.C01.16_A_131

CAACTGGTAGCCCTGGTCTGGTGTTACCGACAGAGTTGA

>NL01B.C01.16_A_132

AAAAACATAGTAGAAATGTTAATGACCAACAAAAGTGAAT

>NL01B.C01.16_A_133

TTTCAATTCTATAGTAGATTAGCAGGGATGAAAGCAAAAAGGTGAAAA

>NL01B.C01.16_A_134

ATAATATATTATTAAAAATATGAACATCAAAAAAAGAACGTCCA

>NL01B.C01.16_A_135

GTACCTTTACTCCATGGCTAAAGCGTTTTCCATATATCACG

>NL01B.C01.16_A_136

ACAAGAAGTGAAAAAGGAGTTCGGTGGACTGGTAGAAGATACA

>NL01B.C01.16_A_137

CCAAAATAACTACATGTACTTGGTGTTATAAATCCTACAGT

>NL01B.C01.17_A_1

GCTTTATGCATGGATTGAAAGTTACAATTCTAATTTATCAAC

>NL01B.C01.17_A_2

TACGAAATAAATGACGAACAGTTAATCGAAATTATTAA

>NL01B.C01.17_A_3

CAAAACAATCCGTTTTCTAACTGCTTAATTGCGGAAGCCA

>NL01B.C01.17_A_4

CTATTATTGAAGTTTGCTCTTGCTTGTGTGCAGTCGACTGATTAT

>NL01B.C01.17_A_5

GTTGATATAAACGTCTGTGAAAAAGTAAAAATATATGATAG

>NL01B.C01.17_A_6

ATATGCAGTCGATATTATTACAAGGACTGACCCCACAAA

>NL01B.C01.17_A_7

TTTCTTCTCAGCAAGATAGTCAATAACACTGTTCAAGTACT

>NL01B.C01.17_A_8

TCTCATATGGTATAATCGTAAAGTAAGCAAGATCTGCCTT

>NL01B.C01.17_A_9

AAGTTATACTTTTATAAATATATAAATATGTCATATTATAAGT

>NL01B.C01.17_A_10

TGGCTTAATGCTGTAGTTGATATGATAATGTTGTATTTTCC

>NL01B.C01.17_A_11

TTTTTGACCATGAGTTTTGTCATAATATTCCTCAACTTCAC

>NL01B.C01.17_A_12

TTAAGTGCAAATGCATTAGTGAGTTTAAAGAAATTGAT

>NL01B.C01.17_A_13

AATATCGTTATGAATTTGTCGTTTGGTAATGGTCGTTTGC

>NL01B.C01.17_A_14

CTTATTATTCGTTGTATTTACGCTTCGTAACAACAGTATACT

>NL01B.C01.17_A_15

TTTTCTCGAATTATTTCTTCAGAATATGGCACTATTTTTATCCATTC

>NL01B.C01.17_A_16

TTACATTTTTCAGTGTGCTAATTTTTTAATGTCATACTAC

>NL01B.C01.17_A_17

AGTATGAGCTAGAAGTGGATCTTACAACTACTGTTTCTTT

>NL01B.C01.17_A_18

ATTATAACAATGATGTCATCTGGATTCATCCAGACCACTCAA

>NL01B.C01.17_A_19

TTTAATAAATGTAAAGCCTAAGCCACAGCCAACTACTTCCC

>NL01B.C01.17_A_20

ATTACAATTGGTAAATTTTTGCCAGCAATAATAGATAAATT

>NL01B.C01.17_A_21

CTAGCATTGGGGGTTTTAGGGGGATACCCCCTAACTAGAC

>NL01B.C01.17_A_22

TTTACTTCAGCTAGCTGAATAAAAAAATTTAAATTAA

>NL01B.C01.17_A_23

AAGGAGTTGTGGAAACATCCAGTTGAAGGCCTTCTATAGT

>NL01B.C01.17_A_24

AAAATCACTATGCCATAGCGAAATCATTATTAGCTTAATTT

>NL01B.C01.17_A_25

TTCAGATATCCAATTTCAATAATTCATCCATTCTTCTAT

>NL01B.C01.17_A_26

GGCTATGTACTCGTAAACGGTGTGCGAATAAGGAGGAAAAT

>NL01B.C01.17_A_27

TATAGCTACTGCAGGTATCAATACGTATTACTTGCCTCTG

>NL01B.C01.17_A_28

GTTTTTACATAGTTATACTTGGAGGTGAAAAAATGAGTAAAGA

>NL01B.C01.17_A_29

ACAATATAGGAAGTCGCATTGGGTCTTAACTGGAGAT

>NL01B.C01.17_A_30

ACTGTGGATCACTTGAGCAAAAATAATCTTGACAGAA

>NL01B.C01.17_A_31

TATAGCGCCGATTGTTCCATATTTACCTATTTTAAGTA

>NL01B.C01.17_A_32

ATCCTCTCCTTAACAAAGATGTTGTATATTCTACCGATATATCTTA

>NL01B.C01.17_A_33

TAATTATGGGGATACGGTTAACTTCCAACTATTATT

>NL01B.C01.17_A_34

AATTTTTAGGGCTAACTGAGAAAAAAGTTAGGAAATATT

>NL01B.C01.17_A_35

TCCTTAAGCTTCACTACGACTCTACTGTCTAGGTTCTGT

>NL01B.C01.17_A_36

CCAGGTTCCTCCTTAGGACTTTAGGGTCTTTTCTCTGCT

>NL01B.C01.17_A_37

TCTTCTCTTCTTTAGTACTAGAAACTTGACTCATCAATAT

>NL01B.C01.17_A_38

AAAGAAAGGTGCAACACATCGACCGCAAACGCATAAATAA

>NL01B.C01.17_A_39

TTGTTAGATTACAAGTATTGGCAGGAGAGAACAACGCCAG

>NL01B.C01.17_A_40

CTTGCTAACTTGTTTTGCAAAAAGTTTAAATACTTATTT

>NL01B.C01.17_A_41

TAAATAAACCCCGTAGCTCACCTCGCCTAGGAAAATAGT

>NL01B.C01.17_A_42

ACCGTCGCTGAAATACGTTAGTGTCAACTCGCCCTCC

>NL01B.C01.17_A_43

CATAAAGTACTACGTCATTGTTATAGAACTTCTTCCTTTCCAG

>NL01B.C01.17_A_44

TACATATTATTTCTAGCATCATAGACTAAATTGGCATAAGTCGG

>NL01B.C01.17_A_45

ATTTGAACTGTTTTCATTTTCATATCACCAATATATAGTATGTA

>NL01B.C01.17_A_46

AAAAGTTGCTAGGGCACTTAATAGACTCGGCGGCGGGCCTGCTC

>NL01B.C01.17_A_47

CTTATAGGGTCTCTAACCTCTCCATTCACTTCCTCTTCTACCTC

>NL01B.C01.17_A_48

AACGGAAATTCTGTAAGATATTCAATGACTATACATAGCCAA

>NL01B.C01.17_A_49

CCTTATATATGCGTACTTGCTCCTCCTCTTTTGGAAGATT

>NL01B.C01.17_A_50

ATAGCCTTCTTTCTCTTGTAAATTTCTTCAATCACTTCCGC

>NL01B.C01.17_A_51

TTCGGTAATCGTACTGGTGTCCCTACTGGTATTGACACTGG

>NL01B.C01.17_A_52

TAATTTTCTGATAAATCGGGATTAATACTTTATAATAGCTCTGAT

>NL01B.C01.17_A_53

TTTCAATTCTATAGTAGATTAGC

>NL01B.C01.17_A_54

TAAAGAGTTGTCCATTTACAATATTTCCAATAATTGTATGT

>NL01B.C01.17_A_55

CATATTCAAACTCTCTAGTTTCATCATTAATATCATTAGTACT

>NL01B.C01.17_A_56

CACGGTGACTTTGCGGAAGAAATGCTATCGTTCTATGCAAAT

>NL01B.C01.17_A_57

CTTCTTTTTAACCGTTAGATTTTTAAACCCCGTTCTAGT

>NL01B.C01.17_A_58

AAACCAAATTCCAACAGACCCTGAAACAGACGTTTGCA

>NL01B.C01.17_A_59

CGGGAATTACGCACCGCCCCTCCTCGAACAAATGCTG

>NL01B.C01.17_A_60

AAAACTGTTCTTTCCTTTTTTGTTCTCTCCATCATTTCTCCT

>NL01B.C01.17_A_61

TAGATAAAATATTAGAAGTTGTAGATAAATTAGATGATTATAA

>NL01B.C01.17_A_62

AGAGAAACAGCAGGTACAAAATCAACTCAGCCAAGTACAA

>NL01B.C01.17_A_63

CACCTTGGTGAGTATCAAGTCAAATACGAATATCACAGACA

>NL01B.C01.17_A_64

CTTTCCAATATAAGTTAATATTTCTTGAAAGAGTCCCTTAG

>NL01B.C01.17_A_65

AATCAGGTATAACGACATAATGGTTGAATGCAGGCCTACAAG

>NL01B.C01.17_A_66

TTATGATTGCTTTCCAAGTTTGACCATTCCAGAATAGG

>NL01B.C01.17_A_67

TTAATTTGTTCTAACTGAATTGAAAAAACTAACTTAATTT

>NL01B.C01.17_A_68

AATGCCATCTATCTCACCCGTGCTTGTGCTTCTTCTTGTGA

>NL01B.C01.17_A_69

ATTTTCACGCTGTTAAGTGTCGGGACGGCCAAGCCGA

>NL01B.C01.17_A_70

ACCCCAGCGGAAAATTCACAGTCACGTTCAACGAGGTGGG

>NL01B.C01.17_A_71

AAATATTCTATTTGTTCTATTTCCGATATGGTATATGAGCTA

>NL01B.C01.17_A_72

AAGAAGGTACAACAGACAAATACATTGTGTTAACAAATCTAA

>NL01B.C01.17_A_73

ATATCATAAAAATCCAATACTTTTGCATGCAATTGCGGG

>NL01B.C01.17_A_74

GTATTGGCAGTGGTACAATACCGATGATTGCGTCGTA

>NL01B.C01.17_A_75

TGTCGATGACCCTAGGTTTTGTCGAGTTAAGCATCTTCGG

>NL01B.C01.17_A_76

GATTATATAATATGAATTATTTGCTTAAAAAGTAGTCGAGA

>NL01B.C01.18_A_1

ACCTTCTTACCCATAATTATTATATTCATAAAATGTTAA

>NL01B.C01.18_A_2

CAAAACAATCCGTTTTCTAACTGCTTAATTGCGGAAGCCA

>NL01B.C01.18_A_3

AAAGAGTATTCCCTTCAACATATGCTTTTTTTCAGTTAAACTTT

>NL01B.C01.18_A_4

CATTATTCTCTCTACTCTTGCTTTCGGCACCGTATTTTT

>NL01B.C01.18_A_5

TTTTTGACCATGAGTTTTGTCATAATATTCCTCAACTTCAC

>NL01B.C01.18_A_6

CATATTCAAACTCTCTAGTTTCATCATTAATATCATTAGTACT

>NL01B.C01.18_A_7

CGAAGTGACGGTGAACGTTATGACCTGGACGACGATTATG

>NL01B.C01.18_A_8

TCACGTTTTACCACCTTTTACAGCTTTCGTTACTTCT

>NL01B.C01.18_A_9

ATCCAAGCTCTCCCTCCAACACCGGCATAACAGGGCTGTAT

>NL01B.C01.18_A_10

TTTGATACCGTTATGATAGACTGAACCAGTTAGTATGCCA

>NL01B.C01.18_A_11

GCTGAATTCTCCGCCAGGATGACGCTGAAGGTAAATGGGA

>NL01B.C01.18_A_12

TTCTCATCGACTTTGCCCAAGAACTTTGAGAATTGTTTC

>NL01B.C01.18_A_13

ACTTTCTGAAAGATTACGGCGATTATTTTGTAATGATAAT

>NL01B.C01.18_A_14

GTTATTGATTCACCTTTTGCATAACATGTTGAAAAAACTT

>NL01B.C01.18_A_15

TAGAGATGCAGAAAGTGAAAGTGATGATAGGAGGGATGTA

>NL01B.C01.18_A_16

TTCTTTTTATGTGAAATTTGACATAATTATCAAGCTTTAGA

>NL01B.C01.18_A_17

GGCTATGTACTCGTAAACGGTGTGCGAATAAGGAGGAAAAT

>NL01B.C01.18_A_18

TAGGACTTCATCTTCAGCAGTATATTGTGTGCTTCCAGG

>NL01B.C01.18_A_19

GCTACTGTGGTATATAGCATGACAACTGGGTTACCATA

>NL01B.C01.18_A_20

GAGGGGAAGGTCTTGAGTACACAAGAAATCAGGGAGATGTATAA

>NL01B.C01.18_A_21

ATTACAAGCGAAATTGTAATTAATAATTTCGATGGTCA

>NL01B.C01.18_A_22

ATTTCTCTTGCTTCTTTCAAATACTCCAAAAGTTTAGGTG

>NL01B.C01.18_A_23

ATCATTATCGTCACAATTATATTTCTTGTCCAACATTTTCT

>NL01B.C01.18_A_24

ATATTGTACGTTAAAACAGTGCTAAACTATGATGAATCTA

>NL01B.C01.18_A_25

AATTAAAATCAGTGTCGAGATAAACCCAAAAACGGGCAGG

>NL01B.C01.18_A_26

TTTATACTGCCGTTAAATATGACGGTGCGTTTGAAGG

>NL01B.C01.18_A_27

ATAGCCTTCTTTCTCTTGTAAATTTCTTCAATCACTTCCGC

>NL01B.C01.18_A_28

TCTCATATGGTATAATCGTAAAGTAAGCAAGATCTGCCTT

>NL01B.C01.18_A_29

ATTCCTACAGCAAGGATATGACAAAAAGATTTCACATTATT

>NL01B.C01.18_A_30

TGGTCATATACACTTCCAAAAGAGATGCTTCCAATTTTAGTC

>NL01B.C01.18_A_31

TCGCCGCCCCCGTGAATTTTTGCAAACCGAGAAAAGTGAGT

>NL01B.C01.18_A_32

TATTATAATTCCCCATACGATTTTCCAGACCTTTTCCCTTCT

>NL01B.C01.18_A_33

ACTAACATTATTAAATGCTTTCTTCTGCCTTATAAACTTTTCTTTC

>NL01B.C01.18_A_34

AATTTTTAGGGCTAACTGAGAAAAAAGTTAGGAAATATT

>NL01B.C01.18_A_35

TGCTGTTAGGTATTGTCCTGAACACGAAGCCGATGAAGAA

>NL01B.C01.18_A_36

AGTATACCTGAAGTTAGATTGGGACAATCGGATATAAA

>NL01B.C01.18_A_37

TGAACTTGTAAAAAAAGGCTATTCAATAGCTCAAATTGCAAA

>NL01B.C01.18_A_38

GCAATAATAGATAAATTGGCTGAAATTCTTCCTGAGAT

>NL01B.C01.18_A_39

ATATGCAGTCGATATTATTACAAGGACTGACCCCACAAA

>NL01B.C01.18_A_40

TTATTATTATTTACGATTGCTAATGAGTAAAGTGGAATATTG

>NL01B.C01.18_A_41

ATAATTAAAAGCCATATTGGATCTATTGATGTATTTAAATT

>NL01B.C01.18_A_42

TTGAAAAGGTGTCATTAAACTTCATATATAGGAATGAGACGA

>NL01B.C01.18_A_43

TTTGGTATATGACAGGAGGCATCCTCTATTAGAGATGGCG

>NL01B.C01.18_A_44

AATTGCCTAATTACATAATTTCCTAATTTCTCCTCCAAT

>NL01B.C01.18_A_45

CCTGCGATGTCGACCAGAAAGACCTGCGTATAGAAAAAAG

>NL01B.C01.18_A_46

GATATAGATGAAATATTCAAGTGATACTATGCTAAAAGATTA

>NL01B.C01.18_A_47

TAGTATTTGCTTAGGCCCCGGCGCTGTAGAATGTAAGGCA

>NL01B.C01.18_A_48

GAGTATATAGTTCATAGCATCTTTCATTTCTTTCTCCTCAA

>NL01B.C01.18_A_49

TTTCAATTCTATAGTAGATTAGCCCCGGCGCTGTAGAATGTAAGGCA

>NL01B.C01.18_A_50

ACAGCTTGCACAAGTTTTGCCAAAAATAGTTAGTTCTCAGT

>NL01B.C01.18_A_51

GTCCTGTTTGTTTCACCAACTTGTTGAAAACCTGCTTTTT

>NL01B.C01.18_A_52

ATACATGTAGCATATCTACAACCGGGGCAAATCTATA

>NL01B.C01.18_A_53

GTTACGTTTACGTTTCTGTGCAGCAAGGTAATTCGTTTAG

>NL01B.C01.18_A_54

ACTGTGGATCACTTGAGCAAAAATAATCTTGACAGAA

>NL01B.C01.18_A_55

GTAGGGTAGCACAGATAGCCACACTCAAAGCTACTGACGT

>NL01B.C01.18_A_56

TATAAATCATGAGTTTTTCCACTAAAAAAATAATGAAACA

>NL01B.C01.18_A_57

ATCTCCTACAACCTATCGGTGCTTATAGGAGTAATACCTA

>NL01B.C01.18_A_58

TATTAATTTCCGTAGTAATCAATTCTGTAAACTTATTAA

>NL01B.C01.18_A_59

TTGAGTATATGGACTTTAACGGTAACTGGCATACAAATAG

>NL01B.C01.18_A_60

GAGACCTTGATAAGATTATTACTACGCGACGATTTG

>NL01B.C01.18_A_61

ACCGTCGCTGAAATACGTTAGTGTCAACTCGCCCTCC

>NL01B.C01.18_A_62

ATATAACATATGTAAAAACTAGTATTTAAAGTTTTCTTT

>NL01B.C01.18_A_63

CATAAAGTACTACGTCATTGTTATAGAACTTCTTCCTTTCCAG

>NL01B.C01.18_A_64

TCTGCAAGTTCAAAGAGTTCTTTTGCAATTTCGTAATTATT

>NL01B.C01.18_A_65

ACATCACGTATCTCTGCTAATAAAATATGGTCTATTGAACA

>NL01B.C01.18_A_66

TTTTGCTTTAATTCAGTTAAAAGATGTTTCTTAAGTTCACAAG

>NL01B.C01.18_A_67

TTTCTGTCCATGCGTTTTATCGTAATATTCTTCAACTTCTG

>NL01B.C01.18_A_68

TTGTCAAGTTGTATTGGTTCAAATCCGACCTTTGTGG

>NL01B.C01.18_A_69

GCCCCTCCTCCTAAAGGCATTCCTTGACTATGATAATAA

>NL01B.C01.18_A_70

GATAATGAGAAACCCTGGTGATTACAGAAAAGAGGTAGAG

>NL01B.C01.18_A_71

CTATTAGAAATTTCGATATAATTTATCTGTCTTTTCGTTAC

>NL01B.C01.18_A_72

AATTCTCTGGTATTATCATCAATATCATTAGCACTCATT

>NL01B.C01.18_A_73

TGGAAAACTGGCAATTCAACATTTAGACCTACTTCTTGAA

>NL01B.C01.18_A_74

CATTTCATAATGAAGTACAAGACGTTTGACCACATCAGCGG

>NL01B.C01.18_A_75

ATATACATTTCTCTTTCCCCCTTTATAAATTTTTCTCT

>NL01B.C01.18_A_76

CTTTCCAATATAAGTTAATATTTCTTGAAAGAGTCCCTTAG

>NL01B.C01.18_A_77

TTAATTTGTTCTAACTGAATTGAAAAAACTAACTTAATTT

>NL01B.C01.18_A_78

CTTCAGGACTCAAATAGGTAAAGTTAATTTCATTTCCATCC

>NL01B.C01.18_A_79

GTATTGGCAGTGGTACAATACCGATGATTGCGTCGTA

>NL01B.C01.18_A_80

GGATAGACATGTTTGTATTCTTCACAAAACCACTTAAGTAAG

>NL01B.C01.18_A_81

CGAGGGGAAAGCTAAGGAGGTAGACTTCAAAAAAGAAGAGAA

>NL01B.C01.18_A_82

ATCAGTTAGATTCAAGTTGTTGCTTGATCTTGATTACGATTA

>NL01B.C01.18_A_83

TGCTTATCTTGAAAATACGGATATTTCTCATCTTTGTAAT

>NL01B.C01.18_A_84

TATTTTTGAGCAAATTCATGTGCTAACTTAATTATTTGTT

>NL01B.C01.18_A_85

AGCACTGATATCATAGATATTAACATACCAAGTGCTACT

>NL01B.C01.18_A_86

GTTTCATCTATGATGACAGCACCACTTATTGATAAGC

>NL01B.C01.18_A_87

TTAAGTGCAAATGCATTAGTGAGTTTAAAGAAATTGAT

>NL01B.C01.18_A_88

AGATATAATCGAGAAGAACTAGAAAATGGATTATTATCAA

>NL01B.C01.18_A_89

GGTCTTATGGAGTTGACGCAAAATACATCAAAAAGCATGAAG

>NL01B.C01.18_A_90

TTGTCTTACATACACATTTATGTTTGCATTTACGCGTACAAT

>NL01B.C01.18_A_91

TAATTATGGGGATACGGTTAACTTCCAACTATTATT

>NL01B.C01.18_A_92

ACAGTTGTGCTTGCAGTCGTTGCTTTGATTTATTCTGTCTG

>NL01B.C01.18_A_93

AGCTATTAATATCAATAGAAGACTTATTCGATGTATTTGTGTTG

>NL01B.C01.18_A_94

TATAAATCGTGAGTTTTTCCGTGAATAAAATAATAAAACAGC

>NL01B.C01.18_A_95

TCATTCATCTTAATTTCAACTCTGTTTTTTTGCAGTTGAG

>NL01B.C01.18_A_96

GATTAACATTTATTACAAATTTCCAAAGTTTGAATTTGC

>NL01B.C01.18_A_97

CTTCTTTTTAACCGTTAGATTTTTAAACCCCGTTCTAGT

>NL01B.C01.18_A_98

GTAGGAAAATTAAAGACGAAAGTGTATTTCTGTGTTGCA

>NL01B.C01.18_A_99

AAACCACCAACACCTATAATCATAAAAAGTTTATCTTCCC

>NL01B.C01.18_A_100

CTATTATTGAAGTTTGCTCTTGCTTGTGTGCAGTCGACTGATTAT

>NL01B.C01.18_A_101

ATATTACCTATGCGTACATGTATTCGCTGTCCTTCATTG

>NL01B.C01.18_A_102

TTCTTTTGCATTACATATAGTCTCTTCATCATTACTGCAT

>NL01B.C01.18_A_103

CCTAACCCACCGATACATAAACCAGAACCAATCGTTAA

>NL01B.C01.18_A_104

TCACCGCTTTACTCAGGTCCATCCATAGAAACTGTAAGAATAA

>NL01B.C01.18_A_105

TCTGTATTGTAGAAACTACATTCTGGTAATATTTCGCAT

>NL01B.C01.18_A_106

TTGTTAGATTACAAGTATTGGCAGGAGAGAACAACGCCAG

>NL01B.C01.18_A_107

AATAAACGCCAAGAATTTAGTAAATTCAGGGAATTTC

>NL01B.C01.18_A_108

AAATTCTTTATTCCATCAATTTCTTCTTTTAGTGCGTTTCGT

>NL01B.C01.18_A_109

TATAGCGCCGATTGTTCCATATTTACCTATTTTAAGTA

>NL01B.C01.18_A_110

CTTATAGGGTCTCTAACCTCTCCATTCACTTCCTCTTCTACCTC

>NL01B.C01.18_A_111

AATATTAATTGGGGCGGTGCAAAAAATTGTAAATTCCTAAATTC

>NL01B.C01.18_A_112

CCATATAGTGTATATTGGGTTGATACTGCATGGAGTAAATACAAT

>NL01B.C01.18_A_113

AATTCCTTGTTCAACTGCTCTTTGTAAAAGTCTAGCAGTTT

>NL01B.C01.18_A_114

GATAATGAATTAAATCTTAATTTCAATCATGTAAACAGTAT

>NL01B.C01.18_A_115

TTCTTTTGCAAATTCTGTAACCTTTTCGCCATGACAGAA

>NL01B.C01.18_A_116

AAGGGCTAGTAGTTGACTTGGGTGTACTAGTGTTACCAT

>NL01B.C01.18_A_117

GTAAATTATAAAATAATAACGCGAAAGAATTAATTTTCCCC

>NL01B.C01.18_A_118

AGGCATATCTGCTCGTTGATGATATTGGGATGGTCATGTATAT

>NL01B.C01.18_A_119

TCCCAAGGCTGGGAGGGTTTTCGTCTCGTCATCAATCAGG

>NL01B.C01.18_A_120

GCTTTATGCATGGATTGAAAGTTACAATTCTAATTTATCAAC

>NL01B.C01.18_A_121

TATTGGATTTAATGGAGCATTTAAATTCATATTTCCACACCA

>NL01B.C01.18_A_122

TTATAGCTTCTTTTAAATACTCTATAATTTCACTATACTG

>NL01B.C01.18_A_123

GTTGATATAAACGTCTGTGAAAAAGTAAAAATATATGATAG

>NL01B.C01.18_A_124

AAACCAATTTCATTGAAAATTTCTCTTGCAGAAATCTTGAA

>NL01B.C01.18_A_125

AACTTTTTTGCTAATTCCTCCAATTTGATACTTTCTTCCTTA

>NL01B.C01.18_A_126

AATGCCATCTATCTCACCCGTGCTTGTGCTTCTTCTTGTGA

>NL01B.C01.18_A_127

ATAATGAGTATTTGAATGCGTTAGCTGAATTTGATAGAACTGG

>NL01B.C01.18_A_128

GGAACTAACGGTATCATAGGAGCGTCTTTCGCATTTTAT

>NL01B.C01.18_A_129

ATATCATAAAAATCCAATACTTTTGCATGCAATTGCGGG

>NL01B.C01.18_A_130

AGTTGTTGTGCAAATTGTAAATCTGAATCTAAAACACCGTAG

>NL01B.C01.18_A_131

TTAGCCTCCCCGCTAATAATCGTCGTCTGACCCGTAT

>NL01B.C01.18_A_132

TTTTTTCACCTCTAACTAAGAGTCTGTGTTAGACTCTA

>NL01B.C01.18_A_133

ATCCTCTCCTTAACAAAGATGTTGTATATTCTACCGATATATCTTA

>NL01B.C01.18_A_134

GTCAGCTGTCTAATTCTCTCAATTTCTGAAACTAGCTTTT

>NL01B.C01.18_A_135

GGGGATTAGGTTATGCTCCTGAAGATGCATTGGAAGATGCAGCT

>NL01B.C01.18_A_136

ACTTTACGCGAGGAATGAGGTGAATGAGGAACAGCTGATG

>NL01B.C01.18_A_137

TATGACTTTCCGGACCTTTTTCCTTCTTACGAAGATTATT

>NL01B.C01.18_A_138

TCCCTACAACGAGTTTCATTTACTTTACTTTATGCCTCCAT

>NL01B.C01.18_A_139

TGAGGAGGTTCTTGGTATGGTGGCGGAGCGTATGGAGAATA

>NL01B.C01.18_A_140

GACATTTTTTTATCACCGTGTGCTTAGTTCAATAATTTG

>NL01B.C01.18_A_141

TCTTGTTCTACTTTTTGACTTTTACCCATAGATTTTACACC

>NL01B.C01.18_A_142

GAAATTTTTAGACCTTCAATCTCACCTTTTATCTCAACTTCA

>NL01B.C01.18_A_143

TTATCATATTGTGCATAACTCGCGGATAAATCTGGTCTCATTC

>NL01B.C01.18_A_144

TCGTAATCATCACCGTATTCGTATAACAGTATAACGGTTCTT

>NL01B.C01.18_A_145

CCGTCACCTCCACCACCTGTATTCTCTTCAACACAGAG

>NL01B.C01.18_A_146

AATATCGTTATGAATTTGTCGTTTGGTAATGGTCGTTTGC

>NL01B.C01.18_A_147

CATAAGATCCGCTATCTCGTTTAATAGTAGTGCTAATTCTA

>NL01B.C01.18_A_148

AAGGAGTTGTGGAAACATCCAGTTGAAGGCCTTCTATAGT

>NL01B.C01.18_A_149

TTAAATTCGCAATTTTAGCATAAATAATTATTTGCTCCTGG

>NL01B.C01.18_A_150

TATTTCTAACTCTGTTGCTCACATGACTGAATTTGTCCATTTA

>NL01B.C01.18_A_151

TTTCTTGAATTTAGCATTGGGGGTATGGGGGTATCCCCCACT

>NL01B.C01.18_A_152

AGACAAAGAAGATGTAATAAGCCTAAGCCATGAGCTTGCGAAAAA

>NL01B.C01.18_A_153

AAACAAGGGCGAAAAGCAACAAGAAGGGCAGAAGCA

>NL01B.C01.18_A_154

TTTAATTCAAAACTTCCGCCACGGGAAGTCAGAAGATAT

>NL01B.C01.18_A_155

AGATGACGTTACTTTATATATGATCACGAGACCACCTTTGG

>NL01B.C01.18_A_156

TGCTATTTTACGCTCCAGCGCTGCTATTGTATTAAGCGCTT

>NL01B.C01.18_A_157

ATTTATATCGCTGGATTTATAGCTACTGCAAATCAGCAA

>NL01B.C01.18_A_158

TATAGCTACTGCAGGTATCAATACGTATTACTTGCCTCTG

>NL01B.C01.18_A_159

TTTTTAGCTAGACTTTCAATTTGTGAAATTTGATATTTCACT

>NL01B.C01.18_A_160

GGGTTTTATAACGTAGTCGTTGTACCGTCTGAAAATAACA

>NL01B.C01.18_A_161

AACGGAAATTCTGTAAGATATTCAATGACTATACATAGCCAA

>NL01B.C01.18_A_162

TTTTTTCGTAAAACTTGTGCAATATCCTCTGAAACATTTCG

>NL01B.C01.18_A_163

TTAATGTAAGATTTTAAAAAATGACAAATATAAATCATTC

>NL01B.C01.18_A_164

ATAAACGGTGGCGATTATGCATATATTCGCTCAACATTTTCTC

>NL01B.C01.18_A_165

AGTTTGAAGTTGATTAAAAAAAGGAAGACGACTTATACG

>NL01B.C01.18_A_166

TGACTTAGTATGTGGTGGTGCATACATTTGTATTTCT

>NL01B.C01.18_A_167

ATATAGACTATTACCCTGAAACTTGTGCTGATAATATAAAGGGACT

>NL01B.C01.18_A_168

TCCATGTAGTATTTTCTAGATAGTTCGACAAACTGTTTCTC

>NL01B.C01.18_A_169

CAGTTAATAACCCAATTACCTATCTTAACGTACACTTCTT

>NL01B.C01.18_A_170

CCTGTCAGGAGCGTCGTAGTCATAGCAGACGGGGTTTATGT

>NL01B.C01.18_A_171

ACTTTATCACAAATATTGAAATCTTTGCAAACTTTCTCTC

>NL01B.C01.18_A_172

ATTCCTAGAGTCGGAAGATCTATAGAGATATTTGTAGTCGT

>NL01B.C01.18_A_173

ATTTATTAATTATAAAAATTATAACTTGCAAATTAATTATTTA

>NL01B.C01.18_A_174

AAAAACATAGTAGAAATGTTAATGACCAACAAAAGTGAAT

>NL01B.C01.18_A_175

ATGGCTGACGACGATGACAACTATAAAGACAAGTTTAAGT

>NL01B.C01.18_A_176

ATAATATATTATTAAAAATATGAACATCAAAAAAAGAACGTCCA

>NL01B.C01.18_A_177

CACCTTGGTGAGTATCAAGTCAAATACGAATATCACAGACA

>NL01B.C01.18_A_178

CAAACAAGAGAATATGTAAAAGAGTATGCAAATTGGTTA

>NL01B.C01.18_A_179

TTTTTGGTCAGTATATTTTTATGCCAACCCGGTATTTA

>NL01B.C01.18_A_180

CCAAAATAACTACATGTACTTGGTGTTATAAATCCTACAGT

>NL01B.C01.18_A_181

ACATAAATTAACTTAATGATATCGTCTTCTCCGACTACGT

>NL01B.C01.18_A_182

ATAATTTCTAATCTTAAGTCGCTGATTACGTCCATCCAGACT

>NL01B.C01.18_A_183

ACAATATAGGAAGTCGCATTGGGTCTTAACTGGAGAT

>NL01B.C01.18_A_184

ATTTCTTGCTATCTCAGTACCAACAAAATTATGGCAACGT

>NL01B.C01.19_A_1

TTGATCAAAAAGGAGGCTGTAAATGTGGTTACAGAACTGTA

>NL01B.C01.19_A_2

GCTTTATGCATGGATTGAAAGTTACAATTCTAATTTATCAAC

>NL01B.C01.19_A_3

TACGAAATAAATGACGAACAGTTAATCGAAATTATTAA

>NL01B.C01.19_A_4

CAAAACAATCCGTTTTCTAACTGCTTAATTGCGGAAGCCA

>NL01B.C01.19_A_5

CTATTATTGAAGTTTGCTCTTGCTTGTGTGCAGTCGACTGATTAT

>NL01B.C01.19_A_6

GTTGATATAAACGTCTGTGAAAAAGTAAAAATATATGATAG

>NL01B.C01.19_A_7

CTTGCTAACTTGTTTTGCAAAAAGTTTAAATACTTATTT

>NL01B.C01.19_A_8

TTTCTTCTCAGCAAGATAGTCAATAACACTGTTCAAGTACT

>NL01B.C01.19_A_9

AAACCAAATTCCAACAGACCCTGAAACAGACGTTTGCA

>NL01B.C01.19_A_10

ATATGCAGTCGATATTATTACAAGGACTGACCCCACAAA

>NL01B.C01.19_A_11

TTTTTGACCATGAGTTTTGTCATAATATTCCTCAACTTCAC

>NL01B.C01.19_A_12

TTAAGTGCAAATGCATTAGTGAGTTTAAAGAAATTGAT

>NL01B.C01.19_A_13

AATATCGTTATGAATTTGTCGTTTGGTAATGGTCGTTTGC

>NL01B.C01.19_A_14

CTTATTATTCGTTGTATTTACGCTTCGTAACAACAGTATACT

>NL01B.C01.19_A_15

AATCAGGTATAACGACATAATGGTTGAATGCAGGCCTACAAG

>NL01B.C01.19_A_16

TAAAGAGTTGTCCATTTACAATATTTCCAATAATTGTATGT

>NL01B.C01.19_A_17

AGTATGAGCTAGAAGTGGATCTTACAACTACTGTTTCTTT

>NL01B.C01.19_A_18

ATTATAACAATGATGTCATCTGGATTCATCCAGACCACTCAA

>NL01B.C01.19_A_19

TTTAATAAATGTAAAGCCTAAGCCACAGCCAACTACTTCCC

>NL01B.C01.19_A_20

ATTACAATTGGTAAATTTTTGCCAGCAATAATAGATAAATT

>NL01B.C01.19_A_21

CTAGCATTGGGGGTTTTAGGGGGATACCCCCTAACTAGAC

>NL01B.C01.19_A_22

TAATTTTCTGATAAATCGGGATTAATACTTTATAATAGCTCTGAT

>NL01B.C01.19_A_23

AAGGAGTTGTGGAAACATCCAGTTGAAGGCCTTCTATAGT

>NL01B.C01.19_A_24

AAAATCACTATGCCATAGCGAAATCATTATTAGCTTAATTT

>NL01B.C01.19_A_25

TTCAGATATCCAATTTCAATAATTCATCCATTCTTCTAT

>NL01B.C01.19_A_26

TTTTCTCGAATTATTTCTTCAGAATATGGCACTATTTTTATCCATTC

>NL01B.C01.19_A_27

CTTATAGGGTCTCTAACCTCTCCATTCACTTCCTCTTCTACCTC

>NL01B.C01.19_A_28

GTTTTTACATAGTTATACTTGGAGGTGAAAAAATGAGTAAAGA

>NL01B.C01.19_A_29

ACAATATAGGAAGTCGCATTGGGTCTTAACTGGAGAT

>NL01B.C01.19_A_30

ACTGTGGATCACTTGAGCAAAAATAATCTTGACAGAA

>NL01B.C01.19_A_31

TATAGCGCCGATTGTTCCATATTTACCTATTTTAAGTA

>NL01B.C01.19_A_32

ATCCTCTCCTTAACAAAGATGTTGTATATTCTACCGATATATCTTA

>NL01B.C01.19_A_33

AACGGAAATTCTGTAAGATATTCAATGACTATACATAGCCAA

>NL01B.C01.19_A_34

TAATTATGGGGATACGGTTAACTTCCAACTATTATT

>NL01B.C01.19_A_35

TCCTTAAGCTTCACTACGACTCTACTGTCTAGGTTCTGT

>NL01B.C01.19_A_36

CCAGGTTCCTCCTTAGGACTTTAGGGTCTTTTCTCTGCT

>NL01B.C01.19_A_37

TCTTCTCTTCTTTAGTACTAGAAACTTGACTCATCAATAT

>NL01B.C01.19_A_38

AAAGAAAGGTGCAACACATCGACCGCAAACGCATAAATAA

>NL01B.C01.19_A_39

TTGTTAGATTACAAGTATTGGCAGGAGAGAACAACGCCAG

>NL01B.C01.19_A_40

TAAATAAACCCCGTAGCTCACCTCGCCTAGGAAAATAGT

>NL01B.C01.19_A_41

TGGCTTAATGCTGTAGTTGATATGATAATGTTGTATTTTCC

>NL01B.C01.19_A_42

CATAAAGTACTACGTCATTGTTATAGAACTTCTTCCTTTCCAG

>NL01B.C01.19_A_43

TATAGCTACTGCAGGTATCAATACGTATTACTTGCCTCTG

>NL01B.C01.19_A_44

AAAAGTTGCTAGGGCACTTAATAGACTCGGCGGCGGGCCTGCTC

>NL01B.C01.19_A_45

GGCTATGTACTCGTAAACGGTGTGCGAATAAGGAGGAAAAT

>NL01B.C01.19_A_46

AAGTTATACTTTTATAAATATATAAATATGTCATATTATAAGT

>NL01B.C01.19_A_47

CCTTATATATGCGTACTTGCTCCTCCTCTTTTGGAAGATT

>NL01B.C01.19_A_48

ATAGCCTTCTTTCTCTTGTAAATTTCTTCAATCACTTCCGC

>NL01B.C01.19_A_49

TCTCATATGGTATAATCGTAAAGTAAGCAAGATCTGCCTT

>NL01B.C01.19_A_50

TTCGGTAATCGTACTGGTGTCCCTACTGGTATTGACACTGG

>NL01B.C01.19_A_51

TTTCACACAATACAACCGAATATGGCTAGACGCAGCAGACTTAG

>NL01B.C01.19_A_52

TTTACTTCAGCTAGCTGAATAAAAAAATTTAAATTAA

>NL01B.C01.19_A_53

ATACATGGTGCTGTATCATATGCTTCTAGAATTACTTTTGGT

>NL01B.C01.19_A_54

AAAGAATTGGAGATTTTTTTAAATATTAGGTTAATATGAA

>NL01B.C01.19_A_55

GATAATGAGAAACCCTGGTGATTACAGAAAAGAGGTAGAG

>NL01B.C01.19_A_56

CACGGTGACTTTGCGGAAGAAATGCTATCGTTCTATGCAAAT

>NL01B.C01.19_A_57

CTTCTTTTTAACCGTTAGATTTTTAAACCCCGTTCTAGT

>NL01B.C01.19_A_58

AGTTGTTGTGCAAATTGTAAATCTGAATCTAAAACACCGTAG

>NL01B.C01.19_A_59

AATTTTTAGGGCTAACTGAGAAAAAAGTTAGGAAATATT

>NL01B.C01.19_A_60

ATTTGAACTGTTTTCATTTTCATATCACCAATATATAGTATGTA

>NL01B.C01.19_A_61

TTTTTTAGAATTGAAGTTACTGCATTTCCGCCTGTCTCATAC

>NL01B.C01.19_A_62

CGGGAATTACGCACCGCCCCTCCTCGAACAAATGCTG

>NL01B.C01.19_A_63

TTATTGAGAAAGAGTCTTGCTATTATGCAATTTCACTAT

>NL01B.C01.19_A_64

ATGGCTGACGACGATGACAACTATAAAGACAAGTTTAAGT

>NL01B.C01.19_A_65

AAAACTGTTCTTTCCTTTTTTGTTCTCTCCATCATTTCTCCT

>NL01B.C01.19_A_66

TAGATAAAATATTAGAAGTTGTAGATAAATTAGATGATTATAA

>NL01B.C01.19_A_67

AGAGAAACAGCAGGTACAAAATCAACTCAGCCAAGTACAA

>NL01B.C01.19_A_68

CACCTTGGTGAGTATCAAGTCAAATACGAATATCACAGACA

>NL01B.C01.19_A_69

CTTTCCAATATAAGTTAATATTTCTTGAAAGAGTCCCTTAG

>NL01B.C01.19_A_70

TACATATTATTTCTAGCATCATAGACTAAATTGGCATAAGTCGG

>NL01B.C01.19_A_71

ACCGTCGCTGAAATACGTTAGTGTCAACTCGCCCTCC

>NL01B.C01.19_A_72

TTATGATTGCTTTCCAAGTTTGACCATTCCAGAATAGG

>NL01B.C01.19_A_73

TTAATTTGTTCTAACTGAATTGAAAAAACTAACTTAATTT

>NL01B.C01.19_A_74

AATGCCATCTATCTCACCCGTGCTTGTGCTTCTTCTTGTGA

>NL01B.C01.19_A_75

ATTTTCACGCTGTTAAGTGTCGGGACGGCCAAGCCGA

>NL01B.C01.19_A_76

ACCCCAGCGGAAAATTCACAGTCACGTTCAACGAGGTGGG

>NL01B.C01.19_A_77

AAGAAGGTACAACAGACAAATACATTGTGTTAACAAATCTAA

>NL01B.C01.19_A_78

ATATCATAAAAATCCAATACTTTTGCATGCAATTGCGGG

>NL01B.C01.19_A_79

GTATTGGCAGTGGTACAATACCGATGATTGCGTCGTA

>NL01B.C01.19_A_80

TGTCGATGACCCTAGGTTTTGTCGAGTTAAGCATCTTCGG

>NL01B.C01.19_A_81

GATTATATAATATGAATTATTTGCTTAAAAAGTAGTCGAGA

>NL01B.C01.20_A_1

TCATTAATTTCCTTGAATTTACCATTATGATATTTGTAA

>NL01B.C01.20_A_2

AAAGAGTATTCCCTTCAACATATGCTTTTTTTCAGTTAAACTTT

>NL01B.C01.20_A_3

AACCCACACTGGTACGCCAAATACCTTTTTGTTTAGGT

>NL01B.C01.20_A_4

TTTGATACCGTTATGATAGACTGAACCAGTTAGTATGCCA

>NL01B.C01.20_A_5

GCGCTGGCGCAGCAGCGTTAGGGACAGGTATAGGCTT

>NL01B.C01.20_A_6

ATCTTTTGCAAATTCTGTAACCTTTTCGCCATGACAGAA

>NL01B.C01.20_A_7

ATTTTAGGTGTAGATGTTAAGCAATATGCCGACGCAGTAGA

>NL01B.C01.20_A_8

TTAAATTCGCAATTTTAGCATAAATAATTATTTGCTCCTGG

>NL01B.C01.20_A_9

AGTTGTCTTAGCTTCTCCCTTCCTCAAAATCGTTAGGATG

>NL01B.C01.20_A_10

GCTACTGTGGTATATAGCATGACAACTGGGTTACCATA

>NL01B.C01.20_A_11

GTTCAGACTGTTGGTAATCGTTTGCAAGAGATGAAAAAT

>NL01B.C01.20_A_12

ATATTGTACGTTAAAACAGTGCTAAACTATGATGAATCTA

>NL01B.C01.20_A_13

TTTTTCTCTAGATTCTTTATTCTTTTATTTACAATTATTT

>NL01B.C01.20_A_14

ATAAAACGTCTGGAAATCTACAACTATCAACTAACAAATCTTTC

>NL01B.C01.20_A_15

TCAAAATGAGTATCACACCAAACTACTAAATTATCATCTATTA

>NL01B.C01.20_A_16

TTTTTGGTCAGTATATTTTTATGCCAACCCGGTATTTA

>NL01B.C01.20_A_17

TCGCCGCCCCCGTGAATTTTTGCAAACCGAGAAAAGTGAGT

>NL01B.C01.20_A_18

CGAATTAATTCTACATACATTATTTTTATCCTTTTCCTTAT

>NL01B.C01.20_A_19

AGTATACCTGAAGTTAGATTGGGACAATCGGATATAAA

>NL01B.C01.20_A_20

GTATCTGAGCTAACACTTGACTCTGAGATATTGTAGT

>NL01B.C01.20_A_21

TGAACTTGTAAAAAAAGGCTATTCAATAGCTCAAATTGCAAA

>NL01B.C01.20_A_22

TTATTATTATTTACGATTGCTAATGAGTAAAGTGGAATATTG

>NL01B.C01.20_A_23

TTTCTACGACCCCGACGAGAAGGAGTTCGCTTACGCAAT

>NL01B.C01.20_A_24

CGAGGGGAAAGCTAAGGAGGTAGACTTCAAAGAAGAAGAGAA

>NL01B.C01.20_A_25

AAGTCTTCTATTTCTTGCATATTCCAAGTAAGTATTTTGT

>NL01B.C01.20_A_26

TCTGGTATGCCCTCATACGATAATATACGATAACAAAATAT

>NL01B.C01.20_A_27

ACAGCTTGCACAAGTTTTGCCAAAAATAGTTAGTTCTCAGT

>NL01B.C01.20_A_28

CTTCTTCGGACGAGTTGAAAAGATAATAGTATGTATCTGG

>NL01B.C01.20_A_29

GCTAATATTAAATTGTTTGCCAGAATCCCTAACAGGAACGTTTT

>NL01B.C01.20_A_30

GTTACGTTTACGTTTCTGTGCAGCAAGGTAATTCGTTTAG

>NL01B.C01.20_A_31

TTGAGTATATGGACTTTAACGGTAACTGGCATACAAATAG

>NL01B.C01.20_A_32

GTGTCTGAGTATGATACAGCAGAACCAGAATTATTAGCAC

>NL01B.C01.20_A_33

AGTATATTAGCAACTTCTAGCACATAATGCACTTTAATGAAC

>NL01B.C01.20_A_34

ATTGAAATTAACGAGGAAGTGGTAGCCCTAGCGTATGGTGCGCTCTT

>NL01B.C01.20_A_35

ACATCACGTATCTCTGCTAATAAAATATGGTCTATTGAACA

>NL01B.C01.20_A_36

TGATTTTGTCAGTTGGCACACCTCTTACAATTTTTAACT

>NL01B.C01.20_A_37

CTTATTATTCGTTGTATTTACGCTTCGTAACAACAGTATACTTA

>NL01B.C01.20_A_38

TCTTATGGAGTTGACGCAAAATACATCAAAAAGCATGAAG

>NL01B.C01.20_A_39

AACTTATCAACGCACTATTTACAATGTTCAACACTTCTCTCT

>NL01B.C01.20_A_40

TTTCTGTCCATGCGTTTTATCGTAATATTCTTCAACTTCTG

>NL01B.C01.20_A_41

ACAATCGCCACAAAAGGCGGGGCATGGGAAGAATATTTT

>NL01B.C01.20_A_42

TGGAAAACTGGCAATTCAACATTTAGACCTACTTCTTGAA

>NL01B.C01.20_A_43

ACTGTCATAAACGAATATAGAAAATACATTGACTATTTTAGA

>NL01B.C01.20_A_44

ATATACATTTCTCTTTCCCCCTTTATAAATTTTTCTCT

>NL01B.C01.20_A_45

AATATATCACTGTCAAAATACGCTTCAGCACAAGCTAAGG

>NL01B.C01.20_A_46

TGAGGTTCTTTAAATCAAGCAAATCGACCCTGCCCTTTAT

>NL01B.C01.20_A_47

CCTGCGATGTCGACCAGAAAGACCTGCGTATAGAAAAAAG

>NL01B.C01.20_A_48

ATACCTTTAAACCATTATATACACCCGTCTTCCTCACCTGTT

>NL01B.C01.20_A_49

CTTCAGGACTCAAATAGGTAAAGTTAATTTCATTTCCATCC

>NL01B.C01.20_A_50

GTATTGGCAGTGGTACAATACCGATGATTGCGTCGTA

>NL01B.C01.20_A_51

GGATAGACATGTTTGTATTCTTCACAAAACCACTTAAGTAAG

>NL01B.C01.20_A_52

CATGTACATATCAATATAGTCTTATCATCATCAGGCAAGT

>NL01B.C01.20_A_53

ATCAGTTAGATTCAAGTTGTTGCTTGATCTTGATTACGATTA

>NL01B.C01.20_A_54

TGCTTATCTTGAAAATACGGATATTTCTCATCTTTGTAAT

>NL01B.C01.20_A_55

TTCAAAATCTAAAAGGCGGAGTGGGTAAAACAACATTAACG

>NL01B.C01.20_A_56

ACTGTTACAGCTGTAGTACAAACAGTAAAAGCTGCATTATC

>NL01B.C01.20_A_57

ACAGTTGTGCTTGCAGTCGTTGCTTTGATTTATTCTGTCTG

>NL01B.C01.20_A_58

ACTCTGCGGTTTCAGAGCTATTAAGTTATTCGCTTTGAAA

>NL01B.C01.20_A_59

ATCAACTTCTCCTTGAGAAGAGTCATAAGTATAGTATAATGC

>NL01B.C01.20_A_60

GATTAACATTTATTACAAATTTCCAAAGTTTGAATTTGC

>NL01B.C01.20_A_61

TCTTTATACATCTCCCTGATTTCTTGTGTACTCAAGACCT

>NL01B.C01.20_A_62

TCAGTATTCTTTGCTATTTCACTAGCAGTCTCTAGTAGTTTTTTG

>NL01B.C01.20_A_63

AAACCACCAACACCTATAATCATAAAAAGTTTATCTTCCC

>NL01B.C01.20_A_64

AGTTTTACTGTAATATACAACTCGTCGTACGGAATATCGGAATAC

>NL01B.C01.20_A_65

TTCTTTTGCATTACATATAGTCTCTTCATCATTACTGCAT

>NL01B.C01.20_A_66

CCTAACCCACCGATACATAAACCAGAACCAATCGTTAA

>NL01B.C01.20_A_67

TCACCGCTTTACTCAGGTCCATCCATAGAAACTGTAAGAATAA

>NL01B.C01.20_A_68

TTGTTAGATTACAAGTATTGGCAGGAGAGAACAACGCCAG

>NL01B.C01.20_A_69

AATAAACGCCAAGAATTTAGTAAATTCAGGGAATTTC

>NL01B.C01.20_A_70

AGTGTTAAGTAAGGAGTTATGCTATCCAATTCTGCCGA

>NL01B.C01.20_A_71

TATTTCTAACTCTGTTGCTCACATGACTGAATTTGTCCATTTA

>NL01B.C01.20_A_72

GTGGAACTTGATGCGGTTATGTTAGTCAAGAGGGATTATACC

>NL01B.C01.20_A_73

AAAGACTTAGATTTTGTAGTCACGGATTTTGATAAAGCGTTAA

>NL01B.C01.20_A_74

TTCTAACGTTTTAGGAGTAAGTTCTACAGTAAAATTGAAA

>NL01B.C01.20_A_75

TCCCAAGGCTGGGAGGGTTTTCGTCTCGTCATCAATCAGG

>NL01B.C01.20_A_76

TCAAGAAGTTTATAATCTCTTCATTATCGAACACCTTCCTG

>NL01B.C01.20_A_77

ATTAATGACATTAATAAACAACATAAGCAAAATCGAGAACG

>NL01B.C01.20_A_78

TATTGGATTTAATGGAGCATTTAAATTCATATTTCCACACCA

>NL01B.C01.20_A_79

TATGACTTATTCAGGAATATTAGGACGATGTGTCCAAAT

>NL01B.C01.20_A_80

ATTTCTCTTGCTTCTTTCAAATACTCCAAAAGTTTAGGTG

>NL01B.C01.20_A_81

GTCAGCTGTCTAATTCTCTCAATTTCTGAAACTAGCTTTT

>NL01B.C01.20_A_82

TTAGCCTCCCCGCTAATAATCGTCGTCTGACCCGTAT

>NL01B.C01.20_A_83

TTATTTAGACCTGTTTCAATTCGTGATGTAATTTTAG

>NL01B.C01.20_A_84

GGGGATTAGGTTATGCTCCTGAAGATGCATTGGAAGATGCAGCT

>NL01B.C01.20_A_85

TCCCTACAACGAGTTTCATTTACTTTACTTTATGCCTCCAT

>NL01B.C01.20_A_86

CCGTCACCTCCACCACCTGTATTCTCTTCAACACAGAG

>NL01B.C01.20_A_87

ATTGACCCCGATACCATAGACTATATCATTAGACTTATTGA

>NL01B.C01.20_A_88

CATAAGATCCGCTATCTCGTTTAATAGTAGTGCTAATTCTA

>NL01B.C01.20_A_89

TTATATTCTTTAACTATCAAATAATATCTCGCCTCATCTCT

>NL01B.C01.20_A_90

TAAAATTGCTCTTAGTTTGTCAGAGAAAGGTAATGACTTTTTA

>NL01B.C01.20_A_91

CTTATAGGGTCTCTAACCTCTCCATTCACTTCCTCTTCTACCTC

>NL01B.C01.20_A_92

TCTAAGAAAATTTTTGTCACTTGGCATTCTTCGTAACGT

>NL01B.C01.20_A_93

TATAATGAACTATCTAAGTTAGGGTATTCTATCAATGTCACTA

>NL01B.C01.20_A_94

TTTTTTCGTAAAACTTGTGCAATATCCTCTGAAACATTTCG

>NL01B.C01.20_A_95

TTAATGTAAGATTTTAAAAAATGACAAATATAAATCATTC

>NL01B.C01.20_A_96

CTTTATTCTGTTCTTTCTTTTTCTCTTCCTTTTTGGGCTTTTT

>NL01B.C01.20_A_97

ATATAGACTATTACCCTGAAACTTGTGCTGATAATATAAAGGGACT

>NL01B.C01.20_A_98

AATTGCCTAATTACATAATTTCCTAATTTCTCCTCCAAT

>NL01B.C01.20_A_99

TGAACGCTAATTTCGTCATAACAATAACATATGAGCGTG

>NL01B.C01.20_A_100

ATAATTGTAAGATATATCAATCGAATATACAACATCTTTG

>NL01B.C01.20_A_101

TGATAATTTGATAAATCTATATTGAAAGTTTGTCCAACTTG

>NL01B.C01.20_A_102

AAAAACATAGTAGAAATGTTAATGACCAACAAAAGTGAAT

>NL01B.C01.20_A_103

ATAATGAGTATTTGAATGCGTTAGCTGAATTTGATAGAACTGG

>NL01B.C01.20_A_104

ATAAAATCGAAATTATTCATGAAAACTTCTACTAGCATA

>NL01B.C01.20_A_105

TCATTATCAAAATTAGATAATGGCTCATTCCCATTAAAA

>NL01B.C01.20_A_106

ATTTTTAGGATCTGCCTTAGTTCGTCCTCAGACGTCGCGT

>NL01B.C01.20_A_107

ATAATTTCTAATCTTAAGTCGCTGATTACGTCCATCCAGACT

>NL01B.C01.20_A_108

CCAAAATAACTACATGTACTTGGTGTTATAAATCCTACAGT

>NL01B.C01.21_A_1

GAAGAGTATGAAACCCACCGCATTTTGAAAGACATCGAGA

>NL01B.C01.21_A_2

AGCTATTAATAAGCTTAAAATGGATAAGAAGGCAGCTAAG

>NL01B.C01.21_A_3

TTCAGGTACAGGTTTAAGATAGTATGAGAAGAAATAAAG

>NL01B.C01.21_A_4

AAACATAATAAAAATGCCTACCAAAAACTCCTTTATTTATTT

>NL01B.C01.21_A_5

AACCCACACTGGTACGCCAAATACCTTTTTGTTTAGGT

>NL01B.C01.21_A_6

ATCACAATCGACTTTAAGTACAGATCCAGAAGGAATATTG

>NL01B.C01.21_A_7

TTTGATACCGTTATGATAGACTGAACCAGTTAGTATGCCA

>NL01B.C01.21_A_8

GTTTAGAAGGAGAAAGATTGACAATTCAAAGAAGAAAGGGACT

>NL01B.C01.21_A_9

GATATTTAAATTTTCTGCATAACCAGTTAGGAGGAAAGCGA

>NL01B.C01.21_A_10

ACATTAGCGGAAACACTAGCAGATCCTCCAGAACCATT

>NL01B.C01.21_A_11

CGCCCCTTCCAGGGCGGGGAGGGGGTAAGGCTTCTAATACG

>NL01B.C01.21_A_12

CTAATCTGTTTAGAATGAGCAGAGTTTGTGCATGTTTATCTCT

>NL01B.C01.21_A_13

CAGCATCATGGATGTCTTTATCATGAATATAGTCCTTTA

>NL01B.C01.21_A_14

AAGAAAAACTGTTTTCTTCAAATGGACCTCCCAAAATAAC

>NL01B.C01.21_A_15

TTCTTTTTATGTGAAATTTGACATAATTATCAAGCTTTAGA

>NL01B.C01.21_A_16

GGTAGTCTCATGCACCATAAACTATTACTGTAATCAACCC

>NL01B.C01.21_A_17

CATTCGAGGAATGTTTAAGTCTGATAAATCCTCAAGGGTTA

>NL01B.C01.21_A_18

GCTATACCACCAAACGCTACTACTACTGGAGGAGGAAA

>NL01B.C01.21_A_19

TTTTCTCATGGGGCAAAGCCCCCGAGGTGAAAAAAATGGCAG

>NL01B.C01.21_A_20

GCTACTGTGGTATATAGCATGACAACTGGGTTACCATA

>NL01B.C01.21_A_21

AATATCGGTTGCAGAGAAGGAAGTTGTTGCATTAGTTTTTG

>NL01B.C01.21_A_22

ACTATAATATTATAATTTATAATTTTGTCAAACCAAAACTCC

>NL01B.C01.21_A_23

GTCACAGTTGTATCTTTAATACTCTCAACAGCTACCACAGTCGA

>NL01B.C01.21_A_24

TATTTGAACCCGTTAAGGTTTGAAGTTGCCATTAGGTCGC

>NL01B.C01.21_A_25

GTATATTTTCTAGTCTGGTTATCTTGAGGTCATATAT

>NL01B.C01.21_A_26

CTGCATCACAGTACGCTACAGAAGCATCACGTTATTACAA

>NL01B.C01.21_A_27

ACTGAATATCTTTCTAAGTAATCCGTTAGTCGCTATTAGTTC

>NL01B.C01.21_A_28

CGAAGTTATTCGCAAAGATATGCAAAATGTTCAGCTAAATT

>NL01B.C01.21_A_29

ATGCTTATAAACGTGAATTGGCAACTATAATACGGGGCG

>NL01B.C01.21_A_30

TATCATAAACCCAATCGAAGCAGGAATAGGTCACTTAGAA

>NL01B.C01.21_A_31

GCTATGTTGTTGGTGAAGTTGTACATCTATATAATCAAA

>NL01B.C01.21_A_32

TTCTTTTAAAAACGAATTGGGAAAAGATAATGACATGGGAA

>NL01B.C01.21_A_33

AGTTGTATAAGTTCTTGAATCGTTATTGGCTTAGTTAATTCCGT

>NL01B.C01.21_A_34

TGCAACCCATGCTCTTCTGCCATGTAAGTTGTTCCTATACT

>NL01B.C01.21_A_35

GTGCCTCTGTTCTGCAGTCTACACAGAAAGCAAACTTTTAA

>NL01B.C01.21_A_36

AATATATCACTAAGCTTCTTAACATCTAATAACATCTCT

>NL01B.C01.21_A_37

TGCAAAAACTCAGCTACAACGCTCTTATTAACTTCAACTA

>NL01B.C01.21_A_38

GTTGAAAATTCCCATTAGAATGGGGATGCTAATAAACGTA

>NL01B.C01.21_A_39

TTAGGCGGTGGAGCTGGAAAAAATAGCAGATATTTTATG

>NL01B.C01.21_A_40

TCTCATATGGTATAATCGTAAAGCAAGCAAGATCTGCCTT

>NL01B.C01.21_A_41

TTTTTGTAAGCGAATTCGCCCTTAAGTACTTTCTCAACAA

>NL01B.C01.21_A_42

AACATTAAAATCCACTAATCCTACAATCCGAGAAAATTTC

>NL01B.C01.21_A_43

TGTACAATAATTTATACATATTTGATAACGTCGCTAATAAGTGGAT

>NL01B.C01.21_A_44

GCATTCTCCCTACCACCGTCTTGGCGTTAGTTACTAAAT

>NL01B.C01.21_A_45

TGAACTTGTAAAAAAAGGCTATTCAATAGCTCAAATTGCAAA

>NL01B.C01.21_A_46

CTATCAATCCAAGTCTCATTTTTCCATGTCTTTTTCCTA

>NL01B.C01.21_A_47

TCTGGTATGCCCTCATACGATAATATACGATAACAAAATAT

>NL01B.C01.21_A_48

TCTTGTGGTTCTCCTTCCTCTCCCTCCTCTTGATTTTCTCC

>NL01B.C01.21_A_49

TGTGAATAACTAAATACATATAGTTTCTGATGTTTAACAA

>NL01B.C01.21_A_50

AAGTTATACTCTGGGCGGTATCTATACAACTCTATTGCTT

>NL01B.C01.21_A_51

TCAATTTTCCATTTTTCAGGTTCCATAATTCCATTTTTGG

>NL01B.C01.21_A_52

TCACTTTCTCAAATTGAAACTGCAATAAAGTATAACTTTCC

>NL01B.C01.21_A_53

GTTACGTTTACGTTTCTGTGCAGCAAGGTAATTCGTTTAG

>NL01B.C01.21_A_54

AAATTATAGTTATTCATCTGGAAAAAGAACGCTTTCGTATAA

>NL01B.C01.21_A_55

TAATAATCAAACTGCTAGACTTTTGCAAAGAGCAGTTGAACA

>NL01B.C01.21_A_56

GTCACCACGTCTACTTCCCCGGCCTTGAGGCCGGCTTCCTTA

>NL01B.C01.21_A_57

TCTCACCAATACATACTATGTCCTCAAAAGTATTTAAATTTT

>NL01B.C01.21_A_58

GGGTTTTATAACGTAGTCGTTGTACCGTCTGAAAATAACA

>NL01B.C01.21_A_59

TATTCAGATACCCCATACGATGAGTTGTATATTACAGTAAAAT

>NL01B.C01.21_A_60

GAAGACAATGTTATACCGATACTTGATACAGATAGAGGTAT

>NL01B.C01.21_A_61

ATAGTAGTGATAAATAATGCATATGTATTAAATAATAAAT

>NL01B.C01.21_A_62

AAATTAGTTAAGGCACGGCTTAAAAGTGATCTTAAAAAT

>NL01B.C01.21_A_63

TATCTATCCCTGTAGACGAGAAGAAGCAAGTAAGTGTGAA

>NL01B.C01.21_A_64

TCTCTACTATTATGTCTGAAATGTAAGATGGATCCGAATTT

>NL01B.C01.21_A_65

TTGAAAAGGTGTCATTAAACTTCATATATAGGAATGAGACGA

>NL01B.C01.21_A_66

AAATATCTTCGATTTTCTGTCCTTCTCCACCTTCTGATTCCT

>NL01B.C01.21_A_67

TAAATAAACCCGAGTTGGTTCTTTCGTCGCCGACAAACGT

>NL01B.C01.21_A_68

GGTATTCTGCTTTCGCATCTTCTAAAGCCTTCATCAAGTCT

>NL01B.C01.21_A_69

TTTTTCCTGTTAACAGCAAAAGCTAGTCT

>NL01B.C01.21_A_70

AACGAAAAAGGTGTGAGAGGTGCTATTGCGACAACCTTACCG

>NL01B.C01.21_A_71

CTCTGACTTCGTTAAGTGAATATGTCGACCATAGCGGGTT

>NL01B.C01.21_A_72

TATTGTGAAGCTAGAATTACATCTCTTATATTTACTGGTCTA

>NL01B.C01.21_A_73

AATAAACTATATTGAAATTTCAAACCGCGGAACTGGAA

>NL01B.C01.21_A_74

CCCCTCACGGGCGTGGCATCACTGTTCAGTGCCCGCCGTCAGC

>NL01B.C01.21_A_75

TCACCACCTTTCATCCCTGTAATATGTATTACCGGGCTGA

>NL01B.C01.21_A_76

TACAGAACAACTATACTCAAGCTCTTAGGGTATTAAAG

>NL01B.C01.21_A_77

ATTTATACTTTTTGCAGAATGGCATTTTAAGTTTTCATAATA

>NL01B.C01.21_A_78

TAAAATCTCTTATCCCCTACTTTTGCAATTACTCTTTGTCCTAC

>NL01B.C01.21_A_79

AACGAAGTCAAAATTTCGGAAGCTAAATTGCTAATTGGGCC

>NL01B.C01.21_A_80

TTATTTTGCGTAAAATTACGTTAAGAAAAATAAAAATAT

>NL01B.C01.21_A_81

AGACCAACTGGCGATAAGTCAATTTCTACCTCTGGAAGTA

>NL01B.C01.21_A_82

GTATTGGCAGTGGTACAATACCGATGATTGCGTCGTA

>NL01B.C01.21_A_83

TAAGCTGTAAGAGAAGAGGGTAAAAGACTCTTGAGCAT

>NL01B.C01.21_A_84

TATCTTTCGATCCTTACTACTGGTACTTTAAAGATGAATTC

>NL01B.C01.21_A_85

TTTACCCTGTATGTCCCTAGCGGCAAAAGAGGATATATA

>NL01B.C01.21_A_86

CATGTACATATCAATATAGTCTTATCATCATCAGGCAAGT

>NL01B.C01.21_A_87

ATCAGTTAGATTCAAGTTGTTGCTTGATCTTGATTACGATTA

>NL01B.C01.21_A_88

GTCAGAAGTCCTGTTATTACAGCTGTTTTGACGTCTTCATT

>NL01B.C01.21_A_89

ATAACGACCTGGGCGGTTTGTATGGCATTCAGATATCATGG

>NL01B.C01.21_A_90

CTTTTCCAGATAGGACCGTTTGCCCTATCCTAAAACTAGTAG

>NL01B.C01.21_A_91

AAAAGGACTGGTTGATCAGCGATTGTGAACATTTCAAAT

>NL01B.C01.21_A_92

AGTAAGGTTTTTAAATGATGGGGGATATTCAACAAAACTA

>NL01B.C01.21_A_93

TACTTATTTTTTCATCTTTGTAGCTTTCTAGTATATCGTA

>NL01B.C01.21_A_94

AAATTGTCGGATTTAGAGATATATTTTGGTTTGACGGAT

>NL01B.C01.21_A_95

GCAAAACTCTCCTTTCTTTATGGTAGCATTTTACACTGG

>NL01B.C01.21_A_96

CTTGAAAAAACTGGAAAAGTGGTTGGAATAGACTTAGGAG

>NL01B.C01.21_A_97

AGGTGAAGAGAAAGCCAGCAGAATGCTTAACCAAAAAGCTCA

>NL01B.C01.21_A_98

ACTTCTTAAGCTCTTCTGAAGGTGAAGATACTATCTTAT

>NL01B.C01.21_A_99

AGCAAGTATTATGATGTTAGCAATATAATAACGTACAGAGA

>NL01B.C01.21_A_100

TTGACTCGAGAACGGGCATTCCGAATCCTTCAGTTCC

>NL01B.C01.21_A_101

AAGAGTAATGACGTGAAAAACTTATGTGATAGTATCGATATACCA

>NL01B.C01.21_A_102

GACTTTCTTAATTTCTATCAGAGAGGCCCAGCGTTTTAGCC

>NL01B.C01.21_A_103

CCTAACCCACCGATACATAAACCAGAACCAATCGTTAA

>NL01B.C01.21_A_104

TCACCGCTTTACTCAGGTCCATCCATAGAAACTGTAAGAATAA

>NL01B.C01.21_A_105

ACTCAGAACGTTCCGCCTCCAGGTAATATCGTTGCTAA

>NL01B.C01.21_A_106

TATTTTTAAATTATTCGCCCCGTTATTTCCTTTGCTTTGC

>NL01B.C01.21_A_107

CATGAACTACTAGACCTGGTTCGAATTAACGTAATCTTTTTT

>NL01B.C01.21_A_108

TATTTCTAACTCTGTTGCTCACATGACTGAATTTGTCCATTTA

>NL01B.C01.21_A_109

AACGCGATATCGATAGCCTTCTGAAACACATTTTCCTTAG

>NL01B.C01.21_A_110

AAGTTATACTTTTATAAATATATAAATATGTCATATTATAAGT

>NL01B.C01.21_A_111

TTGAAGAAGGTATGAAGTTGTCGGGGAATACTACAGTTGA

>NL01B.C01.21_A_112

AGAGATATGCCACATCCCTGTGTTTTATCTATTTTACCC

>NL01B.C01.21_A_113

CCAGGTTCCTCCTTAGGACTTTAGGGTCTTTTCTCTGCT

>NL01B.C01.21_A_114

TTATTCCGTTTATCTCGACGATATACTTTGCTGCGGAAT

>NL01B.C01.21_A_115

AAACCAACCGCAGTATTATTCCCAACAATACAATGTATTGT

>NL01B.C01.21_A_116

ATTATTTACGCTGGAAATCCATATGCATATACAATTAATAA

>NL01B.C01.21_A_117

GTAAATTATAAAATAATAACGCGAAAGAATTAATTTTCCCC

>NL01B.C01.21_A_118

TTCTAACGTTTTAGGAGTAAGTTCTACAGTAAAATTGAAA

>NL01B.C01.21_A_119

ATGTAAACGATATAATTACTGAGATTTTGGAGAATTATTCT

>NL01B.C01.21_A_120

GATAACTTCCCACTTAGGCTCGTCCAAGTTCATGAACCTC

>NL01B.C01.21_A_121

ATTATCGTTCATCAGCGTTGCGGTTTTGCATAACCTTGA

>NL01B.C01.21_A_122

GTTATTGGTTCACCTTTTGCATAACATGTTGAAAAAACTT

>NL01B.C01.21_A_123

ACAATAACACTAAAGCTAGCTGGAATTGAGGCGAACGCG

>NL01B.C01.21_A_124

TGTAGATTTATCGGAATTATACTCATATGCATCTAGTTCA

>NL01B.C01.21_A_125

AATGTAAATCCAATACCCCAAAACAATCCGTTTTCTAACTGCTTAAT

>NL01B.C01.21_A_126

TAACCCAGGTCGTAGACCCGAAAGGAAACGCTGTATACGAA

>NL01B.C01.21_A_127

AAATTTAACGACATGAAACAAAATGGTGATAACAATG

>NL01B.C01.21_A_128

GGCATAATTCACATGCATGGCAATCTGAACATCAACAAACA

>NL01B.C01.21_A_129

AGGTGGATTCTCAAAAGGGTTCTCGTTATCATATGAAAA

>NL01B.C01.21_A_130

ATCATGATAAGCCTCCTAATTTTCTGTCTACCAAAGCCTTG

>NL01B.C01.21_A_131

GTAATTGTTTTCTTTTTTCCAAACCCATTTATTGAATTTACCG

>NL01B.C01.21_A_132

TTAGCCTCCCCGCTAATAATCGTCGTCTGACCCGTAT

>NL01B.C01.21_A_133

TTATTTAGACCTGTTTCAATTCGTGATGTAATTTTAG

>NL01B.C01.21_A_134

GAAAAGTATATACAGTGGGTTGACGTTAGAGAGGTAGTCAA

>NL01B.C01.21_A_135

TTTTCACAACATCAGCCTCTGAGATATCGAAATATTCCGC

>NL01B.C01.21_A_136

TAGTATTTGCTTAGGCCCCGGCGCTGTAGAATGTAAGGCAGT

>NL01B.C01.21_A_137

GTTGGTGCAATTGAACCACTCGTGAAAACAGATGATAAA

>NL01B.C01.21_A_138

AAAAAATGTTGCCAAATCGCCGAGATTCAGCAGGAAACTCAT

>NL01B.C01.21_A_139

ATCTATGCTATAAAATATACTAGTGAAGTCATTTGCCAAAA

>NL01B.C01.21_A_140

GTCATTATCAGGTAAGTTGGTAATGGTTTTTTCCACAGTCTG

>NL01B.C01.21_A_141

TTTAATAAATGTAAAGCCTAAGCCACAGCCAACTACTTCCC

>NL01B.C01.21_A_142

ACAATTCATATTGGGAGATGACCACAAGCGTGTTTACT

>NL01B.C01.21_A_143

AAATCAGATAGTATGAAGATCTGTTTTATGTCCTTTAATCC

>NL01B.C01.21_A_144

GATTTATGCTAATACACCTATGGTTGAAAGCACAAGTTGG

>NL01B.C01.21_A_145

TTAAATTCGCAATTTTAGCATAAATAATTATTTGCTCCTGG

>NL01B.C01.21_A_146

GAATCTACTGAACACTTTGCGAAATCAGCTGAAAGTTTT

>NL01B.C01.21_A_147

ACTTTCTGCTTCTTGTTGCCCTTTTTTTATGTCTTCAAG

>NL01B.C01.21_A_148

TTCCCTTACCTTTCACTTTCACGTATTTTTACATAAGCTAA

>NL01B.C01.21_A_149

TCGCATAGCATTTTTACGTTCTTATTTTTGATATCGAAA

>NL01B.C01.21_A_150

TTTACTAATGGAGAACAAGCACAAGAAGCCGGGACAGCAATACAA

>NL01B.C01.21_A_151

TTGCTTTGCCGTAAAACATATGTGTTCTTAAACTTTGTT

>NL01B.C01.21_A_152

TAACGATAAAATTTTAACTTTGTGTTATTGGCTTTATTACG

>NL01B.C01.21_A_153

CCCTCAATAACGCAGGAGATGATGTAAAACTTAAAGA

>NL01B.C01.21_A_154

TATAGATTGAGGGCTGAACTCATTAGGTGGGAACGT

>NL01B.C01.21_A_155

GTCAAAAGAGCCATAACTATACTTCCACTAATTTTCAATA

>NL01B.C01.21_A_156

TATCATAGTTCTAGAGATTCCCAATTTATCGTAAGGAA

>NL01B.C01.21_A_157

ATATAGACTATTACCCTGAAACTTGTGCTGATAATATAAAGGGACT

>NL01B.C01.21_A_158

CGTCCTCTCACCGAAAGCTTTTTAAACCCCCCTTTCCCTT

>NL01B.C01.21_A_159

AGTGATTTGCAAAATACTTATCTTGAATATGCAAGAAAT

>NL01B.C01.21_A_160

TCGTCACCAACGCCACGGGATTATATTGTGTCTGCTGGA

>NL01B.C01.21_A_161

ATTCTTAGTACCACAGGATTATACAAAAACACAGCACCT

>NL01B.C01.21_A_162

ATTACTTCAACGCTCACTACGTCTTTATTCAAACTACTTTAAA

>NL01B.C01.21_A_163

AGAATCTTTTGTTGTCTTTATGTTAGGATTGGTTATGTT

>NL01B.C01.21_A_164

TCTTCTCTAGTCGAGAATAATAAATAAACGCCTAATTCTATC

>NL01B.C01.21_A_165

ATTACAGCGACGAGATAGAAGAGCTATGGAGGGAATTAGT

>NL01B.C01.21_A_166

GTATGCTTCTTGCACTACAGGCATATATTGCTGTTGGACTTC

>NL01B.C01.21_A_167

ATAAGTCCTTCGACTATCTCCTGCTGCTTTAGTAAGCTGT

>NL01B.C01.21_A_168

GTTATCCATCAGCTCATGCCACCGTTTGATGAATTTACATCA

>NL01B.C01.21_A_169

CTAGGTTGTTGTTGCAGTTTTGCAACTACAAGTATAT

>NL01B.C01.21_A_170

TATCTTGTAATTCGAATGGCACAAGTCTCCTCGGGAAGTT

>NL01B.C01.21_A_171

CTTAATTCCACCATTTTTTATCCCCCTGAGTATACTCCT

>NL01B.C01.21_A_172

TATTCCTAGACGGAAAAAACCCGTACCTTTACTCAATGGCTAAAG

>NL01B.C01.21_A_173

ACTTTTTCCCCAGAAGGATATACATATTTTATAATAA

>NL01B.C01.21_A_174

TATCCATATCGATACTCCGAATAATTTCGGATTTTGTGGAAC

>NL01B.C01.21_A_175

TTCTCAGTATCAAGCACTCCAACCTATTGCACAGCAATT

>NL01B.C01.21_A_176

ATTGTCACTTACACGCAGTACATTACGCGGTGAGGCAGA

>NL01B.C01.22_A_1

GTTACAGGCTGAGAAGCACGGCAACATAAGGTTCTTCGT

>NL01B.C01.22_A_2

ACGCTTATTGCTTTTTTCTGTGCTTCTTCTTTTAGCTTAT

>NL01B.C01.22_A_3

ACACATTAGGCGATGCGGTATATTGCGTGTTACTGGTGAC

>NL01B.C01.22_A_4

GCTGAATTCTCCGCCAGGATGACGCTGAAGGTAAATGGGT

>NL01B.C01.22_A_5

AAACAAAATTCCAACAGACCCTGAAACAGACGTTTGCAAG

>NL01B.C01.22_A_6

CGAAGTGACGGTGAACGTTATGACCTGGACGACGATTATG

>NL01B.C01.22_A_7

TTTTTCTCTAAATTCTGTAATCTCTTATTGACAACTCCAT

>NL01B.C01.22_A_8

TACTCTTTTATAAGAAAAATGGTGTAAGCTATAAAGGTAA

>NL01B.C01.22_A_9

ACGTTTATAGTTTTAAAGTATTCATCTATCGCTTCTCTTACTTTA

>NL01B.C01.22_A_10

TTTTTTATCCCCCTGAGTATACTCCTTGGTCAAACAA

>NL01B.C01.22_A_11

TTAATTGCTGACGATTTAAATAAACTCATTGACGCAATTGGT

>NL01B.C01.22_A_12

TTTGTTCACGTGGGACTAATAGGAAAAAGTAATCGTCTAG

>NL01B.C01.22_A_13

GGCTATGTACTCGTAAACGGTGTGCGAATAAGGAGGAAAAT

>NL01B.C01.22_A_14

CAAAACAATCCGTTTTCTAACTGCTTAATTGCGGAAGCCA

>NL01B.C01.22_A_15

AACCTTCTTTGCCATGCTAGTTGTCTTTCAGACTTGGAGT

>NL01B.C01.22_A_16

TTAATACTGCTAATCATAGTAGCCGTCCTAATTTGTAC

>NL01B.C01.22_A_17

TACGTGACGTATCAATAATCGCTGGTTTTAATATAGTCATTT

>NL01B.C01.22_A_18

ATATTGTACGTTAAAACAGTGCTAAACTATGATGAATCTA

>NL01B.C01.22_A_19

TCAACTACAGTAAGCGACCCACCACGACCGGAAGTCATTAAC

>NL01B.C01.22_A_20

TTTTCTAAATCGTTTGATATGAGATCTTCTGTTAGGGCTTT

>NL01B.C01.22_A_21

TGAATGGAATCAGTGGTTATGAAGTTAACACCTTTATTTT

>NL01B.C01.22_A_22

AGACAATGTAGAAGTCAGAATAACACCAGAGAATATAA

>NL01B.C01.22_A_23

TCCCTACAACGAGTTTCATTTACTTTACTTTATGCCTCCAT

>NL01B.C01.22_A_24

ACCCCTAACAATGCGATTTTTCCCGGCGTAGTGCTATATACT

>NL01B.C01.22_A_25

TTCTAGGAAAATCTCATCTGGTAGAAGTAGTTGGCTCTTA

>NL01B.C01.22_A_26

AGAATTTAGTAAATTCAGGGAATTTAGTAACAGCATCGCCGA

>NL01B.C01.22_A_27

AAAGAGTATTCCCTTCAACATATGCTTTTTTTCAGTTAAACTTT

>NL01B.C01.22_A_28

TCAGCATACTTCCTAGCCTTACAGAACAACTATACTCAAG

>NL01B.C01.22_A_29

TATTATTCGAAGAAAGAGCGAGAGAAGCATGAATATATT

>NL01B.C01.22_A_30

TCGCCGCCCCCGTGAATTTTTGCAAACCGAGAAAAGTGAGT

>NL01B.C01.22_A_31

CTATCGACCCAAGTTTCATTTCTCCACTTGATTTGCTTT

>NL01B.C01.22_A_32

GTCACTTTTCACTTTACCTTCTTTGAAATTTGTTATTTT

>NL01B.C01.22_A_33

AACTTTCCAGCTCATAACTACTCACCCATATCGTGATGTAG

>NL01B.C01.22_A_34

ATTAGTCCCACGTGAACAAATGGACAGGAAGGAATGAGCTT

>NL01B.C01.22_A_35

CGATATTGCAGGGTATATCGTTTGTGCTTGATCCTGTCAA

>NL01B.C01.22_A_36

GTAGTCCTTCTCGCCCCTCAGTACGACCTCGTTCGGCTCTA

>NL01B.C01.22_A_37

AGTATACCTGAAGTTAGATTGGGACAATCGGATATAAA

>NL01B.C01.22_A_38

TTATTATTATTTACGATTGCTAATGAGTAAAGTGGAATATTG

>NL01B.C01.22_A_39

ACAATCATCACTAATTCACAGTCACAAACCTCAGCATCAGCATA

>NL01B.C01.22_A_40

CAGGTGGTATAAAGCGACGGAAAAAGATGCGAAGAAACG

>NL01B.C01.22_A_41

ATTCTTTTGTAACTCGTTCCAGACCTTTTCAGCAAACTCTTCA

>NL01B.C01.22_A_42

GTTATACTCCACAGAATCATTTCCAAGAAGATTCTACATAG

>NL01B.C01.22_A_43

TTTGAATCTTTCTTCAATTAGTAATATTACTGTATCACTTT

>NL01B.C01.22_A_44

AAGAAGGTACAACAGACAAATACATTGTGTTAACAAATCTAA

>NL01B.C01.22_A_45

CGAGGGGAAAGCTAAGGAGGTAGACTTCAAAGAAGAAGAGAA

>NL01B.C01.22_A_46

TTTGAAAATCGATTCTGTTGTCATTAAAAATTATTTGT

>NL01B.C01.22_A_47

GTCAGCTGTCTAATTCTCTCAATTTCTGAAACTAGCTTTT

>NL01B.C01.22_A_48

GTTGATATAAACGTCTGTGAAAAAGTAAAAATATATGATAG

>NL01B.C01.22_A_49

ACAGCTTGCACAAGTTTTGCCAAAAATAGTTAGTTCTCAGT

>NL01B.C01.22_A_50

TCATGTAAATCATACGGTTCACTGTACCATATTTTTATTT

>NL01B.C01.22_A_51

TGAGCAGTTATCGGTATATTGTTCTTCAGTGCTATAGCAATCT

>NL01B.C01.22_A_52

CTTCTTCGGACGAGTTGAAAAGATAATAGTATGTATCTGG

>NL01B.C01.22_A_53

CAACAATATGACGGCAAACATTATCTCGAGGGTGGGGATTG

>NL01B.C01.22_A_54

GGATGTCGTAAAAGTTGCTGTAAAACTCTAGCTCCTTAGGGGT

>NL01B.C01.22_A_55

GCAATAATAGATAAATTAGCTGAAATTCTTCCTGAGAT

>NL01B.C01.22_A_56

AGAATTGCCCCTTTCAAGGTGGGGAGGAAGACAGCGAA

>NL01B.C01.22_A_57

TTTTTTCTTAGAACTTGTGCAATATCTTCACTAACGTTCCT

>NL01B.C01.22_A_58

AATAATATTTCGCCTCATTTCTCCCTTCTTTTATTGACTGTTC

>NL01B.C01.22_A_59

ACACTATCACAAACACTTCGATCACAGTCCAAAACACA

>NL01B.C01.22_A_60

AGACCTGGGCAGATACGATAACGAGGCACTGTGCAAAATGTTGC

>NL01B.C01.22_A_61

TAGATAAACTAGGCAAAGCAATAACGAACGCATTAAAAAGTCA

>NL01B.C01.22_A_62

CATGTACTTGGTGTTATAAATCCTACAGTTGTATTATTTTG

>NL01B.C01.22_A_63

ATGTATCTGTCATCGTCTAAGTAATTAACGACAAAGTCCTT

>NL01B.C01.22_A_64

GCTCAAACTAAAGTAGTAATAGTTCCACAAAATCCAAAACT

>NL01B.C01.22_A_65

TCCTTATATATAATGCGGGCAATTTGTTGATTTTTACATA

>NL01B.C01.22_A_66

TAACATTAAGTCTATGCTTTTAGACACGATGTCCAGGATGAGG

>NL01B.C01.22_A_67

TATTGCATACTCAAAATAATGTACCGTAGTCCTTACTAATCTG

>NL01B.C01.22_A_68

AACTAATGTTAATGAACCGTCGTAATTCCTTGAGTAATTTT

>NL01B.C01.22_A_69

CCCTTTAGGGCGGGGGTTCCCCGAGGTCTCAGGCGTTACACCCCT

>NL01B.C01.22_A_70

AATGCCATCTATCTCACCCGTGCTTGTGCTTCTTCTTGTGA

>NL01B.C01.22_A_71

ACATCTCTTAACAACAACAAAGTGAATGAAATACACGAA

>NL01B.C01.22_A_72

TCTTATGGAGTTGACGCAAAATACATCAAAAAGCATGAAG

>NL01B.C01.22_A_73

TACTCCAAAAGCATCTTCCCTTTGCGTATCGTCGAACGTTAC

>NL01B.C01.22_A_74

TTTCTGTCCATGCGTTTTATCGTAATATTCTTCAACTTCTG

>NL01B.C01.22_A_75

TTCTGCATATTGCAACGCAAAATAAAATGTAAAATACGCTA

>NL01B.C01.22_A_76

TACACATACCCATTTTAAAGATGAAAGACACCATGATA

>NL01B.C01.22_A_77

ATTGTAAATCCGCCACCTTCACCGATCGTTAATCCACCA

>NL01B.C01.22_A_78

ATAATATATTATTAAAAATATGAACATCAAAAAAAGAACGTCCA

>NL01B.C01.22_A_79

CTTGTTCTACTTTTTGACTTTTACCCATTCTGATTTACACC

>NL01B.C01.22_A_80

ATTAAAGCTAGATTGGGATGATGTCGAAAAATTAATAG

>NL01B.C01.22_A_81

TAATGATGTCAAAATCGGAGTCGTTGTCGTTGTTGTAG

>NL01B.C01.22_A_82

CTTCAGGACTCAAATAGGTAAAGTTAATTTCATTTCCATCC

>NL01B.C01.22_A_83

GATTATATAATATGAATTATTTGCTTAAAAAGTAGTCGAGA

>NL01B.C01.22_A_84

CGACCTCAATAAAACTAATGAAGGGCAATTGAAGGAATTAA

>NL01B.C01.22_A_85

TGACTTATACGATAACGAATCATATGTAAATTCATCGC

>NL01B.C01.22_A_86

CGTTTGTTTAGGCAAATCGTCGCGTAGTAATAATTTCAGTAA

>NL01B.C01.22_A_87

ATACCCTTATTCGGTAACAAAGCGTAATGGCGTTGATAATG

>NL01B.C01.22_A_88

AATTCGTTAAACCAAGCTTTTACCATTTGCTTTCATCCCT

>NL01B.C01.22_A_89

ACTATCTTGCCATAGAGGTCATTAATTGTAAAATCCCT

>NL01B.C01.22_A_90

TTAAGTGCAAATGCATTAGTGAGTTTAAAGAAATTGAT

>NL01B.C01.22_A_91

CTTATTATTCGTTGTATTTACGCTTCGTAACAACAGTATACT

>NL01B.C01.22_A_92

GTGGTGTTTGCTCGGCATTGTTTATTGCAAAATCGACGTG

>NL01B.C01.22_A_93

ACAGCTACTCCACCCAATGCTACTACTGGAGAAGGAGGAAAT

>NL01B.C01.22_A_94

ACAGTTGTGCTTGCAGTCGTTGCTTTGATTTATTCTGTCTG

>NL01B.C01.22_A_95

TAGTTTGTTCCTGTCCAGCTTCGTTCTTTCCCTTAATTC

>NL01B.C01.22_A_96

AATTTTTGGGCGTTACTGAAAGAAAAGTCAAGAAAATGTT

>NL01B.C01.22_A_97

GATTAACATTTATTACAAATTTCCAAAGTTTGAATTTGC

>NL01B.C01.22_A_98

AGCGTTAATATATGTGCAACAAGCAATTAATGACGCAGAG

>NL01B.C01.22_A_99

AGAATTGCCCCTTTCAATTCTATAGTAGATTAG

>NL01B.C01.22_A_100

TTCTTTTGCATTACATATAGTCTCTTCATCATTACTGCAT

>NL01B.C01.22_A_101

AATAAACGCCAAGAATTTAGTAAATTCAGGGAATTTC

>NL01B.C01.22_A_102

GATTTAAAAGCGATTAGAGGCGGTAGATTTCCTAGTTTT

>NL01B.C01.22_A_103

GGATAGACATGTTTGTATTCTTCACAAAACCACTTAAGTAAG

>NL01B.C01.22_A_104

CACAAAAAAGCATTATTCGAAATTGTTATAAACTTTAAT

>NL01B.C01.22_A_105

TATGCTAACTGAAGAGGAAAGGAAACAAATCAAACCTG

>NL01B.C01.22_A_106

TCTAATACTCCAACATCTACACCGTTAAGTAACGTGT

>NL01B.C01.22_A_107

AATTCCTTGTTCAACTGCTCTTTGTAAAAGTCTAGCAGTTT

>NL01B.C01.22_A_108

GTTGATATAAACGTCTTTCAATTCTATAGTAGATTAG

>NL01B.C01.22_A_109

TTCTTTTGCAAATTCTGTAACCTTTTCGCCATGACAGAA

>NL01B.C01.22_A_110

TGCCCATATATCTAAATAGTATAAATCTGTCAGTGACAAA

>NL01B.C01.22_A_111

ATCGCCAACCGTGAAAACACTATTTGAGAACATTATCGATA

>NL01B.C01.22_A_112

ATTTATGCAGTTCTGCTAAATCAAAAGGATTATCAACATCT

>NL01B.C01.22_A_113

TAAATCTGCGTAATTTTTACACTTTCATCTTTCCCCTTGTGG

>NL01B.C01.22_A_114

CCATATAGTGTATATTGGGTTGATACTGCATGGAGTAAATACAAT

>NL01B.C01.22_A_115

GAGGTCGCGCTAAAAAGCGCGGCCAGGGAATGGGCCAC

>NL01B.C01.22_A_116

TTTGACTTTTACCCATTTTTACACCCCTTACTTAAAAGATA

>NL01B.C01.22_A_117

GAGGTCGCGCTAAAAAGCGCAGCCCAGATGTGGGGCGCAGA

>NL01B.C01.22_A_118

ATACATATACTTCCATTACCTATCGTAAGACCTCATGGAGAAC

>NL01B.C01.22_A_119

CATCTCCTATATCCGTAACATATAACGAAACGACACTAACTTT

>NL01B.C01.22_A_120

AGAAAACTTACAACATTTTCAATATCATTAACTACTGCACT

>NL01B.C01.22_A_121

ATTTCTCTTGCTTCTTTCAAATACTCCAAAAGTTTAGGTG

>NL01B.C01.22_A_122

TTTTTTCACCCCTAATTATAACTATTGTGCAAACTAATATT

>NL01B.C01.22_A_123

ACACACGGTGCAGTATCGTATGCTTCTAGAATTAATTT

>NL01B.C01.22_A_124

GGATTGACATAGCTAGATGGGAACGATGTTACGTGGATAG

>NL01B.C01.22_A_125

TTAATTCTTTCAAACAGATGTTTACAAACCCAACGTCAATAG

>NL01B.C01.22_A_126

GGAACTAACGGTATCATAGGAGCGTCTTTCGCATTTTAT

>NL01B.C01.22_A_127

ACATCACGTATCTCTGCTAATAAAATATGGTCTATTGAACA

>NL01B.C01.22_A_128

GGGGATTAGGTTATGCTCCTGAAGATGCATTGGAAGATGCAGCT

>NL01B.C01.22_A_129

ACTTTACGCGAGGAATGAGGTGAATGAGGAACAGCTGATG

>NL01B.C01.22_A_130

TGCATGGTCTGGAATTTCATTATTATTAATTCTAAATA

>NL01B.C01.22_A_131

GAAATTTTTAGACCTTCAATCTCACCTTTTATCTCAACTTCA

>NL01B.C01.22_A_132

AGGAGTATACTCAGTGATAAAAAATGGTGGAATTAAGTGGC

>NL01B.C01.22_A_133

TCTGATTTAGTCGAAAAACTGAAGGCGGGTAAACTGGAGACTG

>NL01B.C01.22_A_134

GTAATCATCTTTTTGACCTTCGCTAATTGTGCTAATAATT

>NL01B.C01.22_A_135

ACTGTACCACAATAACCATCGGTTGAACTATAAAGTTGGGG

>NL01B.C01.22_A_136

TTTCCAAAACTTGGTAGTTATGCTGAAGAGTTTGATATACAT

>NL01B.C01.22_A_137

TAACAAAGCCCTAATTGTTTCGAATACTTTGTAATATACAT

>NL01B.C01.22_A_138

ATTTATATCGCTGGATTTATAGCTACTGCAAATCAGCAA

>NL01B.C01.22_A_139

TATAGCTACTGCAGGTATCAATACGTATTACTTGCCTCTG

>NL01B.C01.22_A_140

TCGCTAATTGTGCTAATAATTCTGGTTCCGCTGTATCATACT

>NL01B.C01.22_A_141

ACAAGAAAGTATTGCAGTAATAGAGACAAAAAAACAAACTACAT

>NL01B.C01.22_A_142

TTCCTTGAAAAACGCCTCAATCTCCTTTTCCTCTTCCTC

>NL01B.C01.22_A_143

TTTTTTCGTAAAACTTGTGCAATATCCTCTGAAACATTTCG

>NL01B.C01.22_A_144

TAACCTGTTCTAACTGAATTGAAAAACTAACATTAATTTCT

>NL01B.C01.22_A_145

TCTATTGAGCGATATCATACCACCACTATACTTACTCTTCT

>NL01B.C01.22_A_146

AAAGTCATAGCCGTATTATAGGTTATGGCTAATGGTGCAG

>NL01B.C01.22_A_147

ATGATTACCTTTCAATTCTATAGTAGATTAG

>NL01B.C01.22_A_148

ACAGTAAATTGAATGGAAGGAGATGTACCAGACAAACCTCTAATGA

>NL01B.C01.22_A_149

ATAATGAGTATTTGAATGCGTTAGCTGAATTTGATAGAACTGG

>NL01B.C01.22_A_150

ACAATATAACTTGCAATTAGCACACCCATTAGCGTTAAA

>NL01B.C01.22_A_151

TGATCAATTTCGTTGCAGGCATACCGATAGTGTTTTATGAAA

>NL01B.C01.22_A_152

GACGTGGTGGAGTTAGAATATGTCACAATCCCTGGTACGTT

>NL01B.C01.22_A_153

TCTGAGTTTGTTGAAGCAACCAATGGGGAAAACGTACCATTTAA

>NL01B.C01.22_A_154

AGAGTTGAAGGCGAATATCAAAACATATTAGATTCGACCCCA

>NL01B.C01.22_A_155

AATCTATCAGTACAGAATAGCAAATCCTAACCCGCTTATTTCCA

>NL01B.C01.22_A_156

ATAATTTCTAATCTTAAGTCGCTGATTACGTCCATCCAGACT

>NL01B.C01.23_A_1

TATAGAGGGTAACTATTTGGAAATGCTTTTGCTGAGGTTTACTTTGCT

>NL01B.C01.23_A_2

ATATGAAAGTGAAAAAGATGTTGGCGGATTTAATACATTCT

>NL01B.C01.23_A_3

AAGTTTGAATTTGCAAATTTTATATTACAAATTTCACTAAAT

>NL01B.C01.23_A_4

TTATTATTTTCCCAAACTGTTCTGCATCATCTATGTCCAA

>NL01B.C01.23_A_5

GGGTATTGACGTTCCGAATCCGCTGTAAATCAGATAGTTTTCATT

>NL01B.C01.23_A_6

ATACCAGTAGGAACACCAGTACATCTACCAAATGATATA

>NL01B.C01.23_A_7

TCAGCCGTGAAAACACTATTTGAGAACATTATCGATAGG

>NL01B.C01.23_A_8

ATTCTTCTTCTTCAACTCTATTGCCATTTCTTGGTGTGAAT

>NL01B.C01.23_A_9

TTACTTTGACAAACAATATCATTCAAATATTTTATAAGGA

>NL01B.C01.23_A_10

AAAACCGCACGAATATGTTATAGTAAACGTTACGGCAC

>NL01B.C01.23_A_11

GGAGCTGTTTTTGTAACTCTTCTAATAAAATAACTTCTAT

>NL01B.C01.23_A_12

TTTTCTCTTTCTTTTTTCACACATTCTTCAAAATTTTTAACA

>NL01B.C01.23_A_13

AAAGGTATGCCGTCCTCATGGATAGGTAAGTCCATCTCTTC

>NL01B.C01.23_A_14

TTCTTTATACTGTTTATCAAGTATTCACTTATTTTTTCTA

>NL01B.C01.23_A_15

ACAGTTGTTTACAATTTATCACATTTTGTCTAATAGTGCTT

>NL01B.C01.23_A_16

TAAATCTAGGAAATGCAGAACAACAATTAAGACAATTAGG

>NL01B.C01.23_A_17

CATCTACTAGAATACCATATAGGAGGTGGATATGTCCCAAT

>NL01B.C01.23_A_18

TCTCATATGGTATAATCGTAAAGCAAGCAAGATCTGCCTT

>NL01B.C01.23_A_19

AACATTAAAATCCACTAATCCTACAATCCGAGAAAATTTC

>NL01B.C01.23_A_20

TTAGGAATGTTGTTTTAACAATAGAACATGACAAACTATA

>NL01B.C01.23_A_21

GAATACGGAATAACTGGACCATCAAGAGCTATTTATCAA

>NL01B.C01.23_A_22

GATTATCTATTTGGAAATGCTTTTGCTGAGGTTTACTTTGCT

>NL01B.C01.23_A_23

TAAATGCGGGTATTATTATTACTCTGGTGAATCAAGCAA

>NL01B.C01.23_A_24

ATTTTCTTCATTCTCTTTTAGTAGAATGACTAATTGAAC

>NL01B.C01.23_A_25

ATTTTGAATTTCATCAAATATCAATCCATTTGATAAAAATA

>NL01B.C01.23_A_26

TTTACTTCAGCTAGCTGAATAAAAAAATTTAAATTAA

>NL01B.C01.23_A_27

AATAGATTTTGATTTATCGGATTTGCTTCTAACAATTGTCCA

>NL01B.C01.23_A_28

ACTGAAAACTTTCTTACAATTGAAAGTTACAAAAAAAT

>NL01B.C01.23_A_29

AACTTAGTCTTTTAAATTGGGGTGGAAAAAATTCCCCCAA

>NL01B.C01.23_A_30

ATAGTAGTGATAAATAATGCATATGTATTAAATAATAAAT

>NL01B.C01.23_A_31

CCAATAATCATACCGGCAAATAGTAAACTTGATATACAA

>NL01B.C01.23_A_32

TCACTTCTTCCCCATTCTCTCTCTTGAATTTTAGACGGCTTATC

>NL01B.C01.23_A_33

TACAGAATTTCCGTTAGGAACTGCATTTAGCTGAACTACTAT

>NL01B.C01.23_A_34

AATTAACATTTATTACAAATTTCCAAAGTTTGAATTTGCA

>NL01B.C01.23_A_35

CTCAAAATGCCCATATAGAGAAGATTGATTGAAAAAATATC

>NL01B.C01.23_A_36

TTCTAACGTTTCAGGAGTAAGTTCTACAGTAAAATTGAAAGG

>NL01B.C01.23_A_37

TTAAAAACCAGTAGATAAACGCCAGACCAATAATCATT

>NL01B.C01.23_A_38

CTCACCATATCACAAGATGATAATGCAGGAGATGAGTGTA

>NL01B.C01.23_A_39

CGTGAGCGAGTACTATAAAAAGCTCTTCTGGTTTAATCCCAT

>NL01B.C01.23_A_40

TTCAGATATCCAATTTCAATAATTCATCCATTCTTCTAT

>NL01B.C01.23_A_41

ATAATTCAAAAAGTCATTATACGAAAGTGTTCTTTTTCC

>NL01B.C01.23_A_42

CATTGAGACGGCTTCAATAGAGTTCGTAAATCATTTATTA

>NL01B.C01.23_A_43

TCTACTGCATATTGTATTGTTGACATTTCAATTTTATTATA

>NL01B.C01.23_A_44

AGAAAGAGGTAAAGCAAGAACGTCGTGACGAGTGTAGCAACC

>NL01B.C01.23_A_45

GTATTGGCAGTGGTACAATACCGATGATTGCGTCGTA

>NL01B.C01.23_A_46

CTTATTGATAAGCTAAGATCTCTTATATCAGCATTATGTTT

>NL01B.C01.23_A_47

ACTCATTTCTTCTTCCTCCTCATCGCCATTTTATGTAACG

>NL01B.C01.23_A_48

TCCTTAAGCTTCACTACGACTCTACTGTCTAGGTTCTGT

>NL01B.C01.23_A_49

AGACCTCCAAGACCCTTAGGTGACCCGCCTGTGACACCTA

>NL01B.C01.23_A_50

TTATCTTACCGTTTTTCCTGTTAACAGCAAAAGCTAGTCT

>NL01B.C01.23_A_51

AGTATGAGCTAGAAGTGGATCTTACAACTACTGTTTCTTT

>NL01B.C01.23_A_52

TTTTATCTAGTGAGTTTCGACAACGGTATTAGTGAAGTAG

>NL01B.C01.23_A_53

ACTGTTACAGCTGTAGTACAAACAGTAAAAGCTGCATTATC

>NL01B.C01.23_A_54

ACTCTGCGGTTTCAGAGCTATTAAGTTATTCGCTTTGAAA

>NL01B.C01.23_A_55

CATTAACCCCCCTCGCTTTGTTTACTACCCAATCGAAT

>NL01B.C01.23_A_56

TATAGCGCCGATTGTTCCATATTTACCTATTTTAAGTA

>NL01B.C01.23_A_57

CCCTCCAGTCCTACTCCTCTACCGCCTATACCTCCTCCAAGT

>NL01B.C01.23_A_58

CCAGGTTCCTCCTTAGGACTTTAGGGTCTTTTCTCTGCT

>NL01B.C01.23_A_59

TTGTTAGATTACAAGTATTGGCAGGAGAGAACAACGCCAG

>NL01B.C01.23_A_60

ATTGTTATATAATAATATATACACGTTTATAAGTTCTAGAATTT

>NL01B.C01.23_A_61

TGATTACGCAAAACTTGTGCAATATCTTCACTCACATTTCT

>NL01B.C01.23_A_62

CTTATAGGGTCTCTAACCTCTCCATTCACTTCCTCTTCTACCTC

>NL01B.C01.23_A_63

AAGTTATACTTTTATAAATATATAAATATGTCATATTATAAGT

>NL01B.C01.23_A_64

ATTAATCTGTCATTTTGCGTAATTTGTGCAAAAAATGAGA

>NL01B.C01.23_A_65

GTTATTATATTAGTAACTGCGTCTAATACTGTAGGATTTG

>NL01B.C01.23_A_66

TATAGAGGGTAACTATTTCCTCACACGTAACAACAAAATTGT

>NL01B.C01.23_A_67

TATGATAAAAAAGGCTAGAGAAAAAGTGAAGGAAATGCTA

>NL01B.C01.23_A_68

TCTAATCTCATCGTAACCACCCAAACATTCGACAAGTTTA

>NL01B.C01.23_A_69

TGCAAAACTGATAAAAAGATTGATAAGAAAAAAATTTTA

>NL01B.C01.23_A_70

AAGAGCGTGATCTATTTGGGAAAGAAGGGATCGGATTACT

>NL01B.C01.23_A_71

TTATCTAATTTTAATGACGAGGGGAATTCATTAACTCAAATATC

>NL01B.C01.23_A_72

AAATATAAACAGTTGATAAATTAGAATTATATTGGGATAT

>NL01B.C01.23_A_73

ACAATATAACTTGCAATTAGCACGCCCATTAATGTTAGAAAT

>NL01B.C01.23_A_74

AGTGTTAAGTAAGGAGTTATGCTATCCAATTCTGCCGA

>NL01B.C01.23_A_75

TTACTACTATCGTAGTTACCTATCAGGTCGTTAAATTCGT

>NL01B.C01.23_A_76

GTGAGGGCTATACAGTTCAAGAAATAGCCCAATTTTTAG

>NL01B.C01.23_A_77

ATATAAAGGGTTTTTTATTTGAGAAAGGAGGATTACAGTAAGAG

>NL01B.C01.23_A_78

AAGTCTAAAGAAATTTCATTTACTGCTTTCTCAAACTCAAA

>NL01B.C01.23_A_79

ATATCGAATCCGCACTTGAAGATGCGGAAAAGAAGTGGG

>NL01B.C01.23_A_80

TACTTTCTCTGTTTATCATATTGGTAGTCCTTCCTCTCTC

>NL01B.C01.23_A_81

GCCACTTTTTACAAGAAAATAAATATCATCTCTTTCTTTTTGT

>NL01B.C01.23_A_82

TTTAATAAATGTAAAGCCTAAGCCACAGCCAACTACTTCCC

>NL01B.C01.23_A_83

ATAAAAATCCCTAGAAATAACTTCATTAGAAAAATCCGCGTTC

>NL01B.C01.23_A_84

ATTATATTTGTCATTTGACCCGACAACTAGTTGGTTTAG

>NL01B.C01.23_A_85

AGCTGATGATGTAGCCGATTCTTATGCTATCAAGTTAAGGAAAAT

>NL01B.C01.23_A_86

CTACAAGAAAAAACGGTACTGATACATCCAGAACACGG

>NL01B.C01.23_A_87

CATTATATCATTTGTAAAAACACATATATAAATCTTTCTCT

>NL01B.C01.23_A_88

AAAGTAGTTGAACAAAAACAGAATGATGAATTGTTTAAAGGT

>NL01B.C01.23_A_89

GAAGAAAATACGGTGCCGGCATTCCGAGAACGTGAAGATATGG

>NL01B.C01.23_A_90

ATTCAGAGAAATCGAAAGTAAAGATATATATGATGAAAGAG

>NL01B.C01.23_A_91

AAAGAAAGGTGCAACACATCGACCGCAAACGCATAAATAA

>NL01B.C01.23_A_92

GAAAATATTGATTTTGTCAAGACGTTACAAAAAATTGCAA

>NL01B.C01.23_A_93

TTGATTTCCATCACCATGTCCATCACCACGCTAAGTC

>NL01B.C01.23_A_94

TTCTTTGTTACTGTAACATTATCACCGAATTGTTCCCTA

>NL01B.C01.23_A_95

TCACATTCAGATATAAATATTCAATTAGTTCCGAAAACA

>NL01B.C01.23_A_96

TCTGCTATGACGCCTCCAATTTGCTGAAAGAGTAAA

>NL01B.C01.23_A_97

AATGACATAATGGCGTTTGTAAATTTGAGATCTACTTATG

>NL01B.C01.23_A_98

CACGGTGACTTTGCGGAAGAAATGCTATCGTTCTATGCAAAT

>NL01B.C01.23_A_99

ATTAAGTATGAAGCGAAATTAAAGATTGCCAGAAAAATTGT

>NL01B.C01.23_A_100

ATTTCGGCGTAAACTCAATTCCTTTAGTCTCCTCCTCAGG

>NL01B.C01.23_A_101

AAAGTAAAAGTAATTTATCAGGAAATGCCAAAAGATAGG

>NL01B.C01.23_A_102

CATAATTCATGTTAGAACTAATTAGTGCGTTAACTTTTCCT

>NL01B.C01.23_A_103

AAGCAATATCTAAACTCAATAAGATACTATCTGCACACAGT

>NL01B.C01.23_A_104

TCTTAATTTTCGAAATGGAATCCATTGGCACAACGAAGA

>NL01B.C01.23_A_105

TCCTTTTACGTCCCTTAACTCCAATCTCTTTTCCATTTAT

>NL01B.C01.23_A_106

ACAATATAGGAAGTCGCATTGGGTCTTAACTGGAGAT

>NL01B.C01.24_A_1

TACAGTAAGGAGATTTTCAAGAAGTTAGTCAAATATGTCA

>NL01B.C01.24_A_2

TCTGATATAATTTCATTAATATCTGAAGCTTTAGGGGA

>NL01B.C01.24_A_3

CATTTTTCTCACATCCCATTTATTTTTCCATTTCTCTCCT

>NL01B.C01.24_A_4

CTGGGCAAGAAGAAGATAGAACACACCTGGGATTACAGCGT

>NL01B.C01.24_A_5

TCCGTGGTGCCCAACATGTATTACGGTTACAGTGTGTTCAA

>NL01B.C01.24_A_6

TAAACCAACCGCAGTATTATTCCCAACAATACAATGTAT

>NL01B.C01.24_A_7

TTTTCTCAGGCAATAAAAGATGTAGAAAACCAGAGCGTAGC

>NL01B.C01.24_A_8

TCAGCCGTGAAAACACTATTTGAGAACATTATCGATAGG

>NL01B.C01.24_A_9

TTTTTATGACAAGTTAAAGACATGAAGCCAGAGTGTAAAGTTT

>NL01B.C01.24_A_10

ATTGTCAACCCGCCACCTTCACCGATCGTTAATCCACCACCTT

>NL01B.C01.24_A_11

ATCTATCATACCCTCATTCTCATAGTATACATAGTATGAGA

>NL01B.C01.24_A_12

TTCAGATATCCAATTTCAATAATTCATCCATTCTTCTAT

>NL01B.C01.24_A_13

CTTCCATTGCAGAAGCTAAGGCTACAGCGGAAACGTTTT

>NL01B.C01.24_A_14

AAAACCGCACGAATATGTTATAGTAAACGTTACGGCAC

>NL01B.C01.24_A_15

ACTGATGCAATTCAAGAATTGGAGAAACTAGGAATGCAAA

>NL01B.C01.24_A_16

ATAAGGGAAGACTGATCAAACCTTATTTATCATCGCTTTTCGT

>NL01B.C01.24_A_17

AACCTATAGGCGATATAGAAGACGTGCTTAATGAGAGATA

>NL01B.C01.24_A_18

CTTTCTGTAAGTACATATTCCCCATTTTTTTCTTCAATTAACCCGT

>NL01B.C01.24_A_19

AATTTGTTATATTCTATATGCTTACCTTGGACTTGTTCTAACTG

>NL01B.C01.24_A_20

TTTTTCTCCTCAATATATCATTTGCAAAAACTTATATTT

>NL01B.C01.24_A_21

GAGAATATAGATTTCATAAAGACGCTACAAAAAATTGCAA

>NL01B.C01.24_A_22

TTATACGTTATATGACATTTTAAAATCTTTTTTTATCTTTT

>NL01B.C01.24_A_23

TTTTCTCTTAGTCGTCCTATGTTTACATGCAGTTGTGAT

>NL01B.C01.24_A_24

TAAACTATGGCACTGGTAACGGTCAATAACTTCACCGTACCGT

>NL01B.C01.24_A_25

CTTCAGCATCCTTTTGCAAATCATTAAATGTTCTCCATTC

>NL01B.C01.24_A_26

GGAGCAAATAATTATTTATGCAAAAATTGTGAATTCAAAG

>NL01B.C01.24_A_27

GAAGGCGTGGGCGTTCAAATAGATCACGGGGGTACCGC

>NL01B.C01.24_A_28

TCTCATATGGTATAATCGTAAAGCAAGCAAGATCTGCCTT

>NL01B.C01.24_A_29

AACATTAAAATCCACTAATCCTACAATCCGAGAAAATTTC

>NL01B.C01.24_A_30

CCTGTGGACTCTTCAGTTAGTCCCTCTGATGCTTTTCCGCCC

>NL01B.C01.24_A_31

AAGTAAAACGCGAATTCAGTAGTAGTGTTTTTGCATAATGTCTTT

>NL01B.C01.24_A_32

AAACTAATTTCTTAATTATCATATGACAAAAATAGTTACATT

>NL01B.C01.24_A_33

CTGTTGAATTAATTCGATTAAAGCTTTTAAAATTGAA

>NL01B.C01.24_A_34

TCACAAGTTGCTAATGCCATAGTAAGGTCATCTAGAGCTA

>NL01B.C01.24_A_35

GACTATTTGAAGAGAGTGTTGAAAATAATAGCAGACAATG

>NL01B.C01.24_A_36

ATAAAGGAGACCTGACCTCCTCCTCACCCTGAAGGCGAGG

>NL01B.C01.24_A_37

GAAAATGTAATTTGTTCCTGAACAGAATGGCCTAATCTCAT

>NL01B.C01.24_A_38

CAGGCTGATAAAGAACTGAACAAAGATCTACATAATAATCG

>NL01B.C01.24_A_39

ATTGCAGAAGGGTCGTCAAAAAGTACAACGTCATACCGTTCTT

>NL01B.C01.24_A_40

AATAAATATAGTGGTGCCATTATCATTGAGTATATGGACTTT

>NL01B.C01.24_A_41

TTTACTTCAGCTAGCTGAATAAAAAAATTTAAATTAA

>NL01B.C01.24_A_42

CCTAAATTACTAAATCTCATTAAGTTTATTATTCTATTACA

>NL01B.C01.24_A_43

ATAGTAGTGATAAATAATGCATATGTATTAAATAATAAAT

>NL01B.C01.24_A_44

ACTTTTCATTGAACTTGAACAAAGTCCATGCCATG

>NL01B.C01.24_A_45

TCAATTTCCCCTTTTACTTCAATTTCGCTCTTACAACCTAA

>NL01B.C01.24_A_46

ATGCGGGGAAGCCCATGAGGGTGGGCGTGTTAAACGTGGTTTGT

>NL01B.C01.24_A_47

TTAAAAACCAGTAGATAAACGCCAGACCAATAATCATT

>NL01B.C01.24_A_48

TCGCGGTAATGCATCGACACGGCGAAATACTGCGACAG

>NL01B.C01.24_A_49

TATTTTTTACAAATTTTTAATAATATGCAAAGCATGGA

>NL01B.C01.24_A_50

TGTTCCACTGCTCTTTGTAATAGTCTAGCTGTCTGATTAT

>NL01B.C01.24_A_51

GAAAATAATTCCTAGAAATATTATAAGTACTATCGACAAT

>NL01B.C01.24_A_52

ATTTGCTAAATCTCCTTACAATTTCCTCCATAAAATACTGAGA

>NL01B.C01.24_A_53

GCACCTTACGGCGGTGTTGAACAATTCGTGAACAACGTGG

>NL01B.C01.24_A_54

CGTGAGCGAGTACTATAAAAAGCTCTTCTGGTTTAATCCCAT

>NL01B.C01.24_A_55

ATTTAGTATACGATGCTCGAAACAATATGTAAGGTGCAG

>NL01B.C01.24_A_56

TAGATAAAATATTAGAAGTTGTAGATAAATTAGATGATTATAA

>NL01B.C01.24_A_57

TTAGTGCCTGTGGAGCTCTGCCCTCTACCCGTAGCGA

>NL01B.C01.24_A_58

ATGTCAAAACAGGCACTTGCTCTTCTTGTTGTTGTGACTTT

>NL01B.C01.24_A_59

TTAACAGTATCAATGACCTTATCGCTTATTTTTGACATAT

>NL01B.C01.24_A_60

GCTCACGATCCAGATAAAAGCCGGGAGGATCCACTTCCACT

>NL01B.C01.24_A_61

TTGAGATTGTAATGAGCTGCGGTTGTCCGTTTTTGTAAGCGAA

>NL01B.C01.24_A_62

TTAATCACAAATGAAAAGAAAGAATTAGGTAAAGAGTTTTA

>NL01B.C01.24_A_63

AAAAAACTTGCGATTTGCCTAAGACTATAGCCCTTACG

>NL01B.C01.24_A_64

GCACCTTTCGCCAAGCCTGACTCTAACGCTAGACCGCCTGC

>NL01B.C01.24_A_65

TTATCTTACCGTTTTTCCTGTTAACAGCAAAAGCTAGTCT

>NL01B.C01.24_A_66

CAGATTTTCGCGTAGTTTATCCTCAGCCTTATGCGAAACA

>NL01B.C01.24_A_67

GTCTTGATATCTGCTAAACCCCTTCTTAGCATCGCGTTTAACA

>NL01B.C01.24_A_68

TAAGATAAAATGCAAACTCGGTAGCAATGTCTTTGCATAA

>NL01B.C01.24_A_69

GAAGAAATAAGAAAAGTCTATGATAGATTCAGTGGTCTC

>NL01B.C01.24_A_70

ATCATGTATTGCTATACAATATTGTTTATATTTCTGTA

>NL01B.C01.24_A_71

ATTTAACAATATTAATTGTATTTCTTCATCTGTCAGTCCATATTT

>NL01B.C01.24_A_72

TCCACGATCTTTTGGAGATTGAGAGTTTCTGTCATTTTTGGTT

>NL01B.C01.24_A_73

TATAGCGCCGATTGTTCCATATTTACCTATTTTAAGTA

>NL01B.C01.24_A_74

AGAAAAGGAGACGTAGTAGATGGAGCAAGAGACGTTTT

>NL01B.C01.24_A_75

CCAAATTCGAAGTAGCTACACCTTGAGCTATTATATTCC

>NL01B.C01.24_A_76

ATACCTACATCCAAGCTATTCACCACACTTTCTTCTTCAC

>NL01B.C01.24_A_77

ATTAATATATTCACAATATGTAGAATTAGTTGTAACTTACA

>NL01B.C01.24_A_78

CCAGGTTCCTCCTTAGGACTTTAGGGTCTTTTCTCTGCT

>NL01B.C01.24_A_79

AAGTTTACTAGTGTAAATGAACAAGTTAGTTCAGTTTTAAGCGAATA

>NL01B.C01.24_A_80

TTGTTAGATTACAAGTATTGGCAGGAGAGAACAACGCCAG

>NL01B.C01.24_A_81

AGTGTTAAGTAAGGAGTTATGCTATCCAATTCTGCCGA

>NL01B.C01.24_A_82

TGATTACGCAAAACTTGTGCAATATCTTCACTCACATTTCT

>NL01B.C01.24_A_83

TCAGGAACTGAGGGCTTTGGACTTCCAGTTTTAGAAAGCAT

>NL01B.C01.24_A_84

TTTATTTCAGATTTTAAAATTACTGCTAGAGAAATTTTC

>NL01B.C01.24_A_85

CTTATAGGGTCTCTAACCTCTCCATTCACTTCCTCTTCTACCTC

>NL01B.C01.24_A_86

ACATGGTTTAGGTCAAGTTTCAGATTGCTGGCTTGCAC

>NL01B.C01.24_A_87

AAGTTATACTTTTATAAATATATAAATATGTCATATTATAAGT

>NL01B.C01.24_A_88

AACAAGTATTATGAGGTTAGTAATGTAATAACATTTAGAGATG

>NL01B.C01.24_A_89

ATAACTATTTAACTCCTCTTTTTTGCAAATATTCTCCTA

>NL01B.C01.24_A_90

TATGATAAAAAAGGCTAGAGAAAAAGTGAAGGAAATGCTA

>NL01B.C01.24_A_91

TATCTATCCAATTAAAGATGTAGACAAAGCTATAGATTTT

>NL01B.C01.24_A_92

ACTTCAACACCTACTCCAACACCATCGCCAGTATTAAA

>NL01B.C01.24_A_93

GCTTTATGCATGGATTGAAAGTTACAATTCTAATTTATCAAC

>NL01B.C01.24_A_94

CAGGCTTATACATTTGGTGAAGGAAATCAACAGCCATTTAA

>NL01B.C01.24_A_95

AAGGGAGGACGACGACGATGACAATAAATGAGATAATTG

>NL01B.C01.24_A_96

ACGTTTAGTTCATAATTCGTATTGTAGGAGTATTCTCTCCC

>NL01B.C01.24_A_97

TATTTTCTCCAGGCATTCAATTTTGCTGAGAGCATGGACAC

>NL01B.C01.24_A_98

ATTATTTTTAAAGTGTATTTGTGCATGCTTTTTGAATTCT

>NL01B.C01.24_A_99

CATCACTTTTGCTAATTCAGCTTTATGAAACTGGAGGCAA

>NL01B.C01.24_A_100

AGTTGGACAAAGTTTCAACATAAATTTATCAAATTATCA

>NL01B.C01.24_A_101

CATTTACTATACTCTTTAACATTAACTTGAATATGGCGTCGTCT

>NL01B.C01.24_A_102

TCACTATTTGCTCCAACATTTGCTCCACCCCCTCCACCAA

>NL01B.C01.24_A_103

TTGACAAATAATCAGGTCATTTTACAATTATATTTGAATTTATTA

>NL01B.C01.24_A_104

TGAAAATTTTGCGTATATTCCCTAATTGAAAAAATATGGGAAT

>NL01B.C01.24_A_105

CTACTTTACTTTGGGGTTGAGGCTTGGGATTTGTTTGTTGTA

>NL01B.C01.24_A_106

AACGTCAATAGCAATGATAGCTACACTCATAACTGCTGTACT

>NL01B.C01.24_A_107

ATTATACGGCGTCCCTGGTACTGGTAAATCATACATTGC

>NL01B.C01.24_A_108

CCTGCTCCTGTCGCTTTTGCGGATGGAACGACTACTGAAACCGA

>NL01B.C01.24_A_109

TCTTGTGGTATTGCATTTAGCTGAACTATTATTCTTTTTATT

>NL01B.C01.24_A_110

TGGCTTAATGCTGTAGTTGATATGATAATGTTGTATTTTCC

>NL01B.C01.24_A_111

GCTACTGTAATATATAGCATGACAACTGGGTTACCATATGTAACTTT

>NL01B.C01.24_A_112

CTAACACAATTAGTTTCTACTATAAACGGTACCATAACCG

>NL01B.C01.24_A_113

TCAAGTTTGTTGGCTTCACCATTCCAGCGTTTAACTTCTTCCA

>NL01B.C01.24_A_114

AGTATGAGCTAGAAGTGGATCTTACAACTACTGTTTCTTT

>NL01B.C01.24_A_115

TTTAATAAATGTAAAGCCTAAGCCACAGCCAACTACTTCCC

>NL01B.C01.24_A_116

CTTTGTGGATTTATCTCACTTTTTATTATTATCAAGCCTTTCTT

>NL01B.C01.24_A_117

TATACTGATTGATGCTTAATAAATTCTCCTTCGTAATCAT

>NL01B.C01.24_A_118

TTTCTCAACGATTTCGTTACTTGTTGCAGGGATAACTG

>NL01B.C01.24_A_119

TTTAGCACTAATATTGTACGATATTTTTTATAACAAAA

>NL01B.C01.24_A_120

AAAGAAAGGTGCAACACATCGACCGCAAACGCATAAATAA

>NL01B.C01.24_A_121

TTAATCATGTTAACTAACGCCGGTCCACCGCCAAGTCTATT

>NL01B.C01.24_A_122

ATTTGAACTGTTTTCATTTTCATATCACCAATATATAGTATGTA

>NL01B.C01.24_A_123

TAGGTAATTCTGAATTTTTTAAAACTATAATTGAATGAGG

>NL01B.C01.24_A_124

TTGGTTCAGTACAAATATATAAGCTGGTGAGACGTTTGAG

>NL01B.C01.24_A_125

TCACATTCAGATATAAATATTCAATTAGTTCCGAAAACA

>NL01B.C01.24_A_126

AATCAAATTGGTAATACACTTACACAAAATTTTGATAATTTTTA

>NL01B.C01.24_A_127

GTCAAAAGAGCCATAACTATACTTCCACTAATTTTCAATA

>NL01B.C01.24_A_128

GTTTCTGGAACTGGAAAACAGCCAATTCCAGATATTATAGC

>NL01B.C01.24_A_129

GAATTTTAGGAATTAGTGAAAGAAAGGTTAAGCAATATA

>NL01B.C01.24_A_130

CACGGTGACTTTGCGGAAGAAATGCTATCGTTCTATGCAAAT

>NL01B.C01.24_A_131

TTTAATGATTTCTATTTTGCGACGTTCGATGATATGTCTTC

>NL01B.C01.24_A_132

ACAATAATATCGTTTTAAGGCATAATGATAAAGATTATA

>NL01B.C01.24_A_133

CTGCAACATTCTACGGATCCGATGCATCACCGTCTACATATACTA

>NL01B.C01.24_A_134

CATATCCTTCTTTTTCCAATTCTTCTTGGTCAAGTTCATCA

>NL01B.C01.24_A_135

ATTTTGCCAAACGATTCAATAGAAATTCATGTTAATAAT

>NL01B.C01.24_A_136

GTTCTAATTGAATTGAAAAACTAACATTAATTTCTTGTTG

>NL01B.C01.24_A_137

CCTCATAAATTAGAATTGCTGAGGCACTTGTTAAGCTTAAGCT

>NL01B.C01.24_A_138

ACAATATAGGAAGTCGCATTGGGTCTTAACTGGAGAT

>NL01B.C01.24_A_139

GTAATTCCATATTCGCTCAAAACTGATGCTACTTGTTCATTTA

>NL01B.C01.25_A_1

ATATGAAAGTGAAAAAGATGTTGGCGGATTTAATACATTCT

>NL01B.C01.25_A_2

AAGTTTGAATTTGCAAATTTTATATTACAAATTTCACTAAAT

>NL01B.C01.25_A_3

TTATTATTTTCCCAAACTGTTCTGCATCATCTATGTCCAA

>NL01B.C01.25_A_4

GGGTATTGACGTTCCGAATCCGCTGTAAATCAGATAGTTTTCATT

>NL01B.C01.25_A_5

AATATTTGGATATTTTTGATTTACAATTCTTTGAACTTGTAAA

>NL01B.C01.25_A_6

ACAAGGGCAGAGAATTCATTGTCGCTAAGGCGATGG

>NL01B.C01.25_A_7

ATACCAGTAGGAACACCAGTACATCTACCAAATGATATA

>NL01B.C01.25_A_8

TCAGCCGTGAAAACACTATTTGAGAACATTATCGATAGG

>NL01B.C01.25_A_9

TTCAGATATCCAATTTCAATAATTCATCCATTCTTCTAT

>NL01B.C01.25_A_10

AAAACCGCACGAATATGTTATAGTAAACGTTACGGCAC

>NL01B.C01.25_A_11

TTTTCTCTTTCTTTTTTCACACATTCTTCAAAATTTTTAACA

>NL01B.C01.25_A_12

ATTATTATATAATAATATATACGCGTTTATAAGTTCTAG

>NL01B.C01.25_A_13

AAAGGTATGCCGTCCTCATGGATAGGTAAGTCCATCTCTTC

>NL01B.C01.25_A_14

TTCTTTATACTGTTTATCAAGTATTCACTTATTTTTTCTA

>NL01B.C01.25_A_15

CCCTCCAGTCCTACTCCTCTACCGCCTATACCTCCTCCAAGT

>NL01B.C01.25_A_16

TCACTTCTTCCCCATTCTCTCTCTTGAATTTTAGACGGCTTATC

>NL01B.C01.25_A_17

GATGTAAAAAATGTTGATATTATAACTATTGGCATATTTTT

>NL01B.C01.25_A_18

TAAATCTAGGAAATGCAGAACAACAATTAAGACAATTAGG

>NL01B.C01.25_A_19

CATCTACTAGAATACCATATAGGAGGTGGATATGTCCCAAT

>NL01B.C01.25_A_20

AGCATTTTTTCTCCAATGGACAAACGAAATTTTGAGTATTT

>NL01B.C01.25_A_21

TCTCATATGGTATAATCGTAAAGCAAGCAAGATCTGCCTT

>NL01B.C01.25_A_22

AACATTAAAATCCACTAATCCTACAATCCGAGAAAATTTC

>NL01B.C01.25_A_23

TTAGGAATGTTGTTTTAACAATAGAACATGACAAACTATA

>NL01B.C01.25_A_24

GAATACGGAATAACTGGACCATCAAGAGCTATTTATCAA

>NL01B.C01.25_A_25

TAAATGCGGGTATTATTATTACTCTGGTGAATCAAGCAA

>NL01B.C01.25_A_26

ATTTTCTTCATTCTCTTTTAGTAGAATGACTAATTGAAC

>NL01B.C01.25_A_27

ATTTTGAATTTCATCAAATATCAATCCATTTGATAAAAATA

>NL01B.C01.25_A_28

TTTACTTCAGCTAGCTGAATAAAAAAATTTAAATTAA

>NL01B.C01.25_A_29

ACTGAAAACTTTCTTACAATTGAAAGTTACAAAAAAAT

>NL01B.C01.25_A_30

AACTTAGTCTTTTAAATTGGGGTGGAAAAAATTCCCCCAA

>NL01B.C01.25_A_31

GCCACTTTTTACAAGAAAATAAATATCATCTCTTTCTTTTTGT

>NL01B.C01.25_A_32

ATAGTAGTGATAAATAATGCATATGTATTAAATAATAAAT

>NL01B.C01.25_A_33

CCAATAATCATACCGGCAAATAGTAAACTTGATATACAA

>NL01B.C01.25_A_34

TCCTTAAGCTTCACTACGACTCTACTGTCTAGGTTCTGT

>NL01B.C01.25_A_35

TACAGAATTTCCGTTAGGAACTGCATTTAGCTGAACTACTAT

>NL01B.C01.25_A_36

AATTAACATTTATTACAAATTTCCAAAGTTTGAATTTGCA

>NL01B.C01.25_A_37

CTCAAAATGCCCATATAGAGAAGATTGATTGAAAAAATATC

>NL01B.C01.25_A_38

TTCTAACGTTTCAGGAGTAAGTTCTACAGTAAAATTGAAAGG

>NL01B.C01.25_A_39

TTAAAAACCAGTAGATAAACGCCAGACCAATAATCATT

>NL01B.C01.25_A_40

TTTTGTTTTCATATTTCCAATATATCTCCTAGATCCATTA

>NL01B.C01.25_A_41

CTCACCATATCACAAGATGATAATGCAGGAGATGAGTGTA

>NL01B.C01.25_A_42

CGTGAGCGAGTACTATAAAAAGCTCTTCTGGTTTAATCCCAT

>NL01B.C01.25_A_43

TTACTTTGACAAACAATATCATTCAAATATTTTATAAGGA

>NL01B.C01.25_A_44

GATGAATAACTGTAATTTTGTTGATAAGGATATCTAGCAA

>NL01B.C01.25_A_45

CATTGAGACGGCTTCAATAGAGTTCGTAAATCATTTATTA

>NL01B.C01.25_A_46

TCTACTGCATATTGTATTGTTGACATTTCAATTTTATTATA

>NL01B.C01.25_A_47

AGAAAGAGGTAAAGCAAGAACGTCGTGACGAGTGTAGCAACC

>NL01B.C01.25_A_48

GTATTGGCAGTGGTACAATACCGATGATTGCGTCGTA

>NL01B.C01.25_A_49

CTTATTGATAAGCTAAGATCTCTTATATCAGCATTATGTTT

>NL01B.C01.25_A_50

ACTCATTTCTTCTTCCTCCTCATCGCCATTTTATGTAACG

>NL01B.C01.25_A_51

AGACCTCCAAGACCCTTAGGTGACCCGCCTGTGACACCTA

>NL01B.C01.25_A_52

TTATCTTACCGTTTTTCCTGTTAACAGCAAAAGCTAGTCT

>NL01B.C01.25_A_53

AGTATGAGCTAGAAGTGGATCTTACAACTACTGTTTCTTT

>NL01B.C01.25_A_54

TTTTATCTAGTGAGTTTCGACAACGGTATTAGTGAAGTAG

>NL01B.C01.25_A_55

ACTGTTACAGCTGTAGTACAAACAGTAAAAGCTGCATTATC

>NL01B.C01.25_A_56

ACTCTGCGGTTTCAGAGCTATTAAGTTATTCGCTTTGAAA

>NL01B.C01.25_A_57

AAACTCTTTAATTGGTGTTCTGCGTCTTTTGTGCTAATAATA

>NL01B.C01.25_A_58

CATTAACCCCCCTCGCTTTGTTTACTACCCAATCGAAT

>NL01B.C01.25_A_59

AGATATTAAAACAAATTCTGGAATATGCTTGAATAAT

>NL01B.C01.25_A_60

TATAGCGCCGATTGTTCCATATTTACCTATTTTAAGTA

>NL01B.C01.25_A_61

GGAGCTGTTTTTGTAACTCTTCTAATAAAATAACTTCTAT

>NL01B.C01.25_A_62

CCAGGTTCCTCCTTAGGACTTTAGGGTCTTTTCTCTGCT

>NL01B.C01.25_A_63

TTGTTAGATTACAAGTATTGGCAGGAGAGAACAACGCCAG

>NL01B.C01.25_A_64

TGATTACGCAAAACTTGTGCAATATCTTCACTCACATTTCT

>NL01B.C01.25_A_65

CTTATAGGGTCTCTAACCTCTCCATTCACTTCCTCTTCTACCTC

>NL01B.C01.25_A_66

AAGTTATACTTTTATAAATATATAAATATGTCATATTATAAGT

>NL01B.C01.25_A_67

ATTAATCTGTCATTTTGCGTAATTTGTGCAAAAAATGAGA

>NL01B.C01.25_A_68

GTTATTATATTAGTAACTGCGTCTAATACTGTAGGATTTG

>NL01B.C01.25_A_69

TATGATAAAAAAGGCTAGAGAAAAAGTGAAGGAAATGCTA

>NL01B.C01.25_A_70

TCTAATCTCATCGTAACCACCCAAACATTCGACAAGTTTA

>NL01B.C01.25_A_71

TGCAAAACTGATAAAAAGATTGATAAGAAAAAAATTTTA

>NL01B.C01.25_A_72

AAGAGCGTGATCTATTTGGGAAAGAAGGGATCGGATTACT

>NL01B.C01.25_A_73

TTATCTAATTTTAATGACGAGGGGAATTCATTAACTCAAATATC

>NL01B.C01.25_A_74

AAGTCTAAAGAAATTTCATTTACTGCTTTCTCAAACTCAAA

>NL01B.C01.25_A_75

ACAATATAACTTGCAATTAGCACGCCCATTAATGTTAGAAAT

>NL01B.C01.25_A_76

AGTGTTAAGTAAGGAGTTATGCTATCCAATTCTGCCGA

>NL01B.C01.25_A_77

TTACTACTATCGTAGTTACCTATCAGGTCGTTAAATTCGT

>NL01B.C01.25_A_78

GTGAGGGCTATACAGTTCAAGAAATAGCCCAATTTTTAG

>NL01B.C01.25_A_79

ATATAAAGGGTTTTTTATTTGAGAAAGGAGGATTACAGTAAGAG

>NL01B.C01.25_A_80

AAATATAAACAGTTGATAAATTAGAATTATATTGGGATAT

>NL01B.C01.25_A_81

ATATCGAATCCGCACTTGAAGATGCGGAAAAGAAGTGGG

>NL01B.C01.25_A_82

TACTTTCTCTGTTTATCATATTGGTAGTCCTTCCTCTCTC

>NL01B.C01.25_A_83

AATGACATAATGGCGTTTGTAAATTTGAGATCTACTTATG

>NL01B.C01.25_A_84

TTTAATAAATGTAAAGCCTAAGCCACAGCCAACTACTTCCC

>NL01B.C01.25_A_85

ATAAAAATCCCTAGAAATAACTTCATTAGAAAAATCCGCGTTC

>NL01B.C01.25_A_86

ATTATATTTGTCATTTGACCCGACAACTAGTTGGTTTAG

>NL01B.C01.25_A_87

AGCTGATGATGTAGCCGATTCTTATGCTATCAAGTTAAGGAAAAT

>NL01B.C01.25_A_88

CTACAAGAAAAAACGGTACTGATACATCCAGAACACGG

>NL01B.C01.25_A_89

AAAGTAGTTGAACAAAAACAGAATGATGAATTGTTTAAAGGT

>NL01B.C01.25_A_90

GAAGAAAATACGGTGCCGGCATTCCGAGAACGTGAAGATATGG

>NL01B.C01.25_A_91

ATTCAGAGAAATCGAAAGTAAAGATATATATGATGAAAGAG

>NL01B.C01.25_A_92

AAAGAAAGGTGCAACACATCGACCGCAAACGCATAAATAA

>NL01B.C01.25_A_93

TATAAAACTCGTTTCAAATTACCGCTTGAATTTCTAACTC

>NL01B.C01.25_A_94

ACAGTTGTTTACAATTTATCACATTTTGTCTAATAGTGCTT

>NL01B.C01.25_A_95

GAAAATATTGATTTTGTCAAGACGTTACAAAAAATTGCAA

>NL01B.C01.25_A_96

TTGATTTCCATCACCATGTCCATCACCACGCTAAGTC

>NL01B.C01.25_A_97

TTCTTTGTTACTGTAACATTATCACCGAATTGTTCCCTA

>NL01B.C01.25_A_98

TCACATTCAGATATAAATATTCAATTAGTTCCGAAAACA

>NL01B.C01.25_A_99

TCTGCTATGACGCCTCCAATTTGCTGAAAGAGTAAA

>NL01B.C01.25_A_100

CACGGTGACTTTGCGGAAGAAATGCTATCGTTCTATGCAAAT

>NL01B.C01.25_A_101

ATTAAGTATGAAGCGAAATTAAAGATTGCCAGAAAAATTGT

>NL01B.C01.25_A_102

ATTTCGGCGTAAACTCAATTCCTTTAGTCTCCTCCTCAGG

>NL01B.C01.25_A_103

AAAGTAAAAGTAATTTATCAGGAAATGCCAAAAGATAGG

>NL01B.C01.25_A_104

CATAATTCATGTTAGAACTAATTAGTGCGTTAACTTTTCCT

>NL01B.C01.25_A_105

AAGCAATATCTAAACTCAATAAGATACTATCTGCACACAGT

>NL01B.C01.25_A_106

AATTCTGAAAACCTAGATAGAAAAATAGAAAAATCTGT

>NL01B.C01.25_A_107

TCTTAATTTTCGAAATGGAATCCATTGGCACAACGAAGA

>NL01B.C01.25_A_108

GAAACCTTCTTACTCTTGATTCTACATCGAAGACTCCAGA

>NL01B.C01.25_A_109

ACTATAACTATTGTCTTTTGTGGTTGAGAATTTGAAGG

>NL01B.C01.25_A_110

TCCTTTTACGTCCCTTAACTCCAATCTCTTTTCCATTTAT

>NL01B.C01.25_A_111

ACAATATAGGAAGTCGCATTGGGTCTTAACTGGAGAT

>NL03.C02.01_A_1

TAAGCTAGATCAAACTCGTGCCAATAACAGCATTCCAATT

>NL03.C02.01_A_2

CATACTAAGTCATGGGTAATTGTTGAATGGAGTAATAAGG

>NL03.C02.01_A_3

TCATTAATTTCCTTGAATTTACCATTATGATATTTGTAA

>NL03.C02.01_A_4

ACCTTCTTACCCATAATTATTATATTCATAAAATGTTAA

>NL03.C02.01_A_5

GATAGAATCTTTTGCATCTTCCAGATTAACTTTCGCTTCGTT

>NL03.C02.01_A_6

CAAGACGGAAAAGTAAGGTATTCCATGACTATCCATTCGC

>NL03.C02.01_A_7

AGTAGAAAAGAAGAAGATACGTCTTGGAAAAAAGGAAAAAGA

>NL03.C02.01_A_8

CAACATAAGTGACAAGGAAAGAGGTAAAGCAATAGAGAATCT

>NL03.C02.01_A_9

ATATTATACTACAAAGGGACAGAATCCAGCTTATCCGGCG

>NL03.C02.01_A_10

ATAACTTTAGAAGTTCCTCATCTGACGCTTCTATCTCATAT

>NL03.C02.01_A_11

TAAACTTCTTCAGGTAATTGATAAATTCGCGTTGTTGTCTC

>NL03.C02.01_A_12

TTTTTCAATATCTTCTAATGCTTTATACGCACTTTCAACG

>NL03.C02.01_A_13

TCTTTTATTGACTGTTCTATTAATTGACTGATTTGTTCA

>NL03.C02.01_A_14

CCGGAATTGGCGTTACACCTTGATGAGATTTTTTTATCAA

>NL03.C02.01_A_15

GCACTGTTCTCAGTGAGGATTATGGTTTCTGTAAACACTT

>NL03.C02.01_A_16

TTCAGATATCCAATTTCAATAATTCATCCATTCTTCTAT

>NL03.C02.01_A_17

CTAATAAGAGAATTGTTAAAAGGGTTTAAAAGTTTTACG

>NL03.C02.01_A_18

GTAGGTAGCACAATTACAATCAAATATGCGAACGGCTCCA

>NL03.C02.01_A_19

TATTGGCATTAACAGAAATAATATATTTAAATTTGAATAACTG

>NL03.C02.01_A_20

ATATTGTACGTTAAAACAGTGCTAAACTATGATGAATCTA

>NL03.C02.01_A_21

AGTACGCAGTTCTCATTACGAATTAGCAATATTTTAATTTGTCA

>NL03.C02.01_A_22

GCAAATCAACTTCTAACCCAACTTCTTGCAAATTTTCAGC

>NL03.C02.01_A_23

CTTTCCAATTCTGAATTTCGTCAAAAATTAGTCCGTTAG

>NL03.C02.01_A_24

TTTAGTATTACATCTTTGTTTGTACATTTTAATCTCCTCA

>NL03.C02.01_A_25

TTTTTTTATCCCTCTGAATCAATTTATCGATTATGACAAATA

>NL03.C02.01_A_26

TCGCCGCCCCCGTGAATTTTTGCAAACCGAGAAAAGTGAGT

>NL03.C02.01_A_27

ATTACTCTTGACGCTATTTCTTTGTTGCTACAAAGTTCTG

>NL03.C02.01_A_28

CGAAGTGGTTCCTCAGGCAGTCTGTCTATACCGTGTTGTAACC

>NL03.C02.01_A_29

AAAGAAAGGTGCAACACATCGACCGCAAACGCATAAATAA

>NL03.C02.01_A_30

TTATGTTCATTTTTTCACCCGCATGTACATTTTTGTTT

>NL03.C02.01_A_31

ATTAAAGATGTAGACAAAGCTATAGATTTTTATAAACAATACTCT

>NL03.C02.01_A_32

GAAAAATCTCCACGGGTTCACCGTCAACTACAGTAAGTGACC

>NL03.C02.01_A_33

CTTTTCTTTTGGCTTCAGGGTCGTTACAGTCTGCTTTGTA

>NL03.C02.01_A_34

TCTCATATGGTATAATCGTAAAGCAAGCAAGATCTGCCTT

>NL03.C02.01_A_35

TTATTATTATTTACGATTGCTAATGAGTAAAGTGGAATATTG

>NL03.C02.01_A_36

AATTTCCCGAGTTTACCAACTTCCTGGCATTTATCAAA

>NL03.C02.01_A_37

AATTCATATATGTAGAAATAACGCGATGACGGTTCATTATAA

>NL03.C02.01_A_38

ACTTCATATCCTGGCACTAGTCCGTATTTTCAATTGAA

>NL03.C02.01_A_39

GTGATTAACAGTCATCGTTGGTACCCAAACATAATAATCCTAA

>NL03.C02.01_A_40

TATGATTTACATGAAGGATTTTATATACCTTGCAAAACTGAT

>NL03.C02.01_A_41

AATTTATATAGCAGGATTTATTTCAACTGCAAATCAACAAA

>NL03.C02.01_A_42

AATGAGTTGGCATACAATCCAGAAATGGATACCTATCTT

>NL03.C02.01_A_43

AATGGTTTGGTGCGTAATCTGTCAACCAGTTGTAAGTTTCT

>NL03.C02.01_A_44

TCCCTTTTCACGTTTTCTATTTTTTTCTATCACCCGCAAT

>NL03.C02.01_A_45

ACATCTTTAGGAATTACATTTCCCATTATCGTATAACTCCTTTC

>NL03.C02.01_A_46

GCAATAATAGATAAATTAGCTGAAATTCTTCCTGAGAT

>NL03.C02.01_A_47

TTTACTTCAGCTAGCTGAATAAAAAAATTTAAATTAA

>NL03.C02.01_A_48

TCTTCAAGTTAATGTTAAAGAGTATCACAAATGTTGAGTAC

>NL03.C02.01_A_49

TAAATACATTGAATAATTTTTCGGCCTCATCTTTCTCAAA

>NL03.C02.01_A_50

TTTAATAGTATATGTTTGTTTCCACAACTTAGTCTTCTT

>NL03.C02.01_A_51

TTAATGCTACCACCAACACATGAAACTTCACCACCACCG

>NL03.C02.01_A_52

ATAGTAGTGATAAATAATGCATATGTATTAAATAATAAAT

>NL03.C02.01_A_53

AATTTATCACCTTTTGCTCTAGTCCAGACACCGTGTGACA

>NL03.C02.01_A_54

TAATTATCATGATGATGATTATAATGACCAGTACTACAATAAA

>NL03.C02.01_A_55

TCCTTAAGCTTCACTACGACTCTACTGTCTAGGTTCTGT

>NL03.C02.01_A_56

ACATCTGATGGAAATCGCGGATTACTCAGCTTTGTAACA

>NL03.C02.01_A_57

TGTTTTACGCCGTATCTGTTTAAGTATGGTCTTAGCCAATC

>NL03.C02.01_A_58

GTTATTATATTTGTCACAGCATCAAGAACAGTTGGATTAG

>NL03.C02.01_A_59

CACTCATTTTTATACTGAACCCCCTTAAAGATTCTGAT

>NL03.C02.01_A_60

TTTCACACAATACAACCGAATATGGCTAGACGCAGCAGACT

>NL03.C02.01_A_61

TCTGATGCTTTTCCGCCCTGGTTGGCTAGGTGTTCCACTAT

>NL03.C02.01_A_62

ATGTATTGGGGTCTTCCCATGCGACCCCAAAGGGGCTATA

>NL03.C02.01_A_63

GAGTATAACATAGGTTTTAAACATTTTGTTGTTGACTATG

>NL03.C02.01_A_64

ATCCCAGCACTGTTGCAATTAGCGGGACAGGTGGAA

>NL03.C02.01_A_65

TTAATGTAAGATTTTAAAAAATGACAAATATAAATCATTC

>NL03.C02.01_A_66

CCATCACTGGTGGGTAGAATACTAAGTGGTATAGAGCAT

>NL03.C02.01_A_67

GCGATAATATTGGGTATCATAGTTAGGAGGTTCGGTTGAT

>NL03.C02.01_A_68

TAAATCGTTGCTTTTAGTAGCAATATCTTGCCCTCGCTGT

>NL03.C02.01_A_69

TTTTCGATATAACCTAAGATAATACCATCGATTAAATTAAC

>NL03.C02.01_A_70

AATGAAATGTCCTTTTTAACAACAGCGGGATCCAGTATAACT

>NL03.C02.01_A_71

TCAGGAAATTGAGCTAAAAAATCTAAAACTGCTTTTTCTTTT

>NL03.C02.01_A_72

AAACAACTATTTTTCTTTATGATGCCTTCCTCTGGTAATAA

>NL03.C02.01_A_73

TTAATAAAACTGCTTTTCTTCTAAATTGTAAATGATAATAT

>NL03.C02.01_A_74

GTATTGGCAGTGGTACAATACCGATGATTGCGTCGTA

>NL03.C02.01_A_75

TCATCTCCAAGCAGTCCTACCATAAGACTACCAAAAAT

>NL03.C02.01_A_76

TTTTTTGTCTTGAGGATTAAAGTTAGCAATAGATTTAAC

>NL03.C02.01_A_77

TCAGAGACACTAAAGATGTTAGTAGGAGAGCAGATA

>NL03.C02.01_A_78

AACAAACCACGCATAAATCTCACATTAGTTGTAAATCCAT

>NL03.C02.01_A_79

ACTGTTACAGCTGTAGTACAAACAGTAAAAGCTGCATTATC

>NL03.C02.01_A_80

ATTTGCACAACTTTCATCTTTTTATCCCCAATTTACATTA

>NL03.C02.01_A_81

ACAAGAAAATTATGTTCATGTCACCTTGGAAAAATATCTGA

>NL03.C02.01_A_82

ACTCTGCGGTTTCAGAGCTATTAAGTTATTCGCTTTGAAA

>NL03.C02.01_A_83

TATTTAATTGTGCGTGTAAAAGCAGATGATGACACACTGCGA

>NL03.C02.01_A_84

GAACATGGAATCGTTGTTATTCCTTTAGAATTCGGA

>NL03.C02.01_A_85

TATAGCGCCGATTGTTCCATATTTACCTATTTTAAGTA

>NL03.C02.01_A_86

ATTCTCTCAAAACTATATTCATATCAAATCATTTTCCTTT

>NL03.C02.01_A_87

CTTTTTAATGCATCATCTATGATGTCAGCTATTCTGTCAA

>NL03.C02.01_A_88

TTATTGTTGTTATACAACCATCATTACCTGTAGCATATGC

>NL03.C02.01_A_89

TTGTTAGATTACAAGTATTGGCAGGAGAGAACAACGCCAG

>NL03.C02.01_A_90

AGTGTTAAGTAAGGAGTTATGCTATCCAATTCTGCCGA

>NL03.C02.01_A_91

TGATTACGCAAAACTTGTGCAATATCTTCACTCACATTTCT

>NL03.C02.01_A_92

CTTATAGGGTCTCTAACCTCTCCATTCACTTCCTCTTCTACCTC

>NL03.C02.01_A_93

GAATACCCGCCTGTACTTTGCGTAGTTGATGTGGTAGTAGTTGT

>NL03.C02.01_A_94

AAGTTATACTTTTATAAATATATAAATATGTCATATTATAAGT

>NL03.C02.01_A_95

ATTGTAATTGGTGCAATAAACGGCGTTAGAAATTGAGTAA

>NL03.C02.01_A_96

ATTATAACAATGATGTCATCTGGATTCATCCAGACCACTCA

>NL03.C02.01_A_97

CCAGGTTCCTCCTTAGGACTTTAGGGTCTTTTCTCTGCT

>NL03.C02.01_A_98

AGTTTGCAAATATGTAGGATTTAAATAATTATTTACTAAA

>NL03.C02.01_A_99

TCCCAAGGCTGGGAGGGTTTTCGTCTCGTCATCAATCAGG

>NL03.C02.01_A_100

TCAAGAAGTTTATAATCTCTTCATTATCGAACACCTTCCTG

>NL03.C02.01_A_101

TATTGGATTTAATGGAGCATTTAAATTCATATTTCCACACCA

>NL03.C02.01_A_102

GTTATTCCATATTCCAACAAAACTGATGCTACTTGTTCATT

>NL03.C02.01_A_103

TAACATTCTCTATTTCTAATGCCAATCCTAACGCCACTTA

>NL03.C02.01_A_104

TATAATGTGTAAGCGAGCCACAACGCTAATGAGGATTTCCCTC

>NL03.C02.01_A_105

TTAAGTTCATAGAAGAATCTTAGAACCTCTACTTCATCTT

>NL03.C02.01_A_106

CATTTTAGTACACCGAATTCATATATGTAGAAATAACG

>NL03.C02.01_A_107

GATTATTTCGCTGATACTAACAGATCACTGAGAGAACAGA

>NL03.C02.01_A_108

AACTTTACTTAACGGTTCTAATATCCTAGTGTATAACGAT

>NL03.C02.01_A_109

ATAACAAAGCGTAAGTATTCTATTTAAAGGGTCTATTTCA

>NL03.C02.01_A_110

CAAAATGCTCCCGGTATTATTTGTTGTCCTTGATAACATG

>NL03.C02.01_A_111

TTTCAAAAGAGGAGATAATAGATATGTGTAAAGAATTAGAA

>NL03.C02.01_A_112

ATATCAAATACCATCAACGTCACCATCCACGTTACGTCGT

>NL03.C02.01_A_113

AGTATGAGCTAGAAGTGGATCTTACAACTACTGTTTCTTT

>NL03.C02.01_A_114

TTTAATAAATGTAAAGCCTAAGCCACAGCCAACTACTTCCC

>NL03.C02.01_A_115

CATAAGATCCGCTATCTCGTTTAATAGTAGTGCTAATTCTA

>NL03.C02.01_A_116

ATTTCGGCGTCTCTCCAGCACCTTACTACAATGCTGAACAGTT

>NL03.C02.01_A_117

ATATGGTTCAGCAAAGCTATTACAATATTATCAAAATGGC

>NL03.C02.01_A_118

ACAAAGCGACTATTAAAATTAAGAATGTTCGTCTATCCA

>NL03.C02.01_A_119

TTCCCGATTTAGAGAAACTAGCAGAAATTACGGATGCT

>NL03.C02.01_A_120

GGATAGACATGTTTGTATTCTTCACAAAACCACTTAAGTAAG

>NL03.C02.01_A_121

ATAGATATATTTCTACATGACCATATTTTGCATATAAATGTCT

>NL03.C02.01_A_122

ATTATTGCCCCGCTCAATGGGTCTCTTACTTCCTCCTTCTC

>NL03.C02.01_A_123

GTCACTTAATAATAGTTGGAAGTTAACCGTATCCCCATAAT

>NL03.C02.01_A_124

CTAAATTATAACACATTTCCTATCTTCTCTTCTTTTTTGTTTC

>NL03.C02.01_A_125

ATTGTTTTTATGTTGAAACATATTAATGGTCAAGTTACTAAAAT

>NL03.C02.01_A_126

TCATGCACTCACCGTCATCTGGAATTGTGCAGTACATGATG

>NL03.C02.01_A_127

ATTTATATCGCTGGATTTATAGCTACTGCAAATCAGCAA

>NL03.C02.01_A_128

ATGGATACATATTTCTTTGAATTCTCCTTCTGCTCTCATTTACCT

>NL03.C02.01_A_129

GTCGGTTGGAAGTCATTAAATCTATTTTAATTTAATTCTTTTT

>NL03.C02.01_A_130

GGGTTTTATAACGTAGTCGTTGTACCGTCTGAAAATAACA

>NL03.C02.01_A_131

GTTAGGAAAGGGGGTCAGACAAATAGTTGATGCAAATAAT

>NL03.C02.01_A_132

TCACATTCAGATATAAATATTCAATTAGTTCCGAAAACA

>NL03.C02.01_A_133

TCCTTTTGTGTCCTTTGATTACTATCTCCTTTCCGTTCAC

>NL03.C02.01_A_134

ATATAGACTATTACCCTGAAACTTGTGCTGATAATATAAAGGGACT

>NL03.C02.01_A_135

CACGGTGACTTTGCGGAAGAAATGCTATCGTTCTATGCAAAT

>NL03.C02.01_A_136

TACGCTAATCCTACCAATTCAGGAGTCACTATACTTATTAG

>NL03.C02.01_A_137

TCTTTTTCTACAATTTCAGAAATCATTTGAATTATTGTA

>NL03.C02.01_A_138

AAAAACATAGTAGAAATGTTAATGACCAACAAAAGTGAAT

>NL03.C02.01_A_139

ACTTCCTGGCGTTTATCAAAGGTATAGGACCAGCTAACGC

>NL03.C02.01_A_140

GTCAGCTGTCTAATTCTCTCAATTTCTGAAACTAGCTTTT

>NL03.C02.01_A_141

ACAATTGAAACAAAAATTAGACAAAGATTTTCCAAATTCCAT

>NL03.C02.01_A_142

CTACGACCTAACATACATCATCATAGGACCTAAATTCA

>NL03.C02.01_A_143

ACAATATAGGAAGTCGCATTGGGTCTTAACTGGAGAT

>NL03.C02.02_A_1

CCCAAGATAAAAGGGGAAAGGGGGTTTAAAAAGCTTTCGCT

>NL03.C02.02_A_2

ATATCTTGTGTATGTTACACTTAAAAAAGTGGTCAAACAG

>NL03.C02.02_A_3

TACTTGGCAAGGTGTGGAGTCCCTTCCCTGTTAGTACTGA

>NL03.C02.02_A_4

TCATTTTTATTGATGAGGCAATAAACAGTATAGAGACCAG

>NL03.C02.02_A_5

AAAGAGTATTCCCTTCAACATATGCTTTTTTTCAGTTAAACTTT

>NL03.C02.02_A_6

TATTTTGTCGTCAGTCTCGAAACAAATTGTATATCCCGCAA

>NL03.C02.02_A_7

CTATAGGTGTTGAAATGAAATATTCTACTAGACCGAA

>NL03.C02.02_A_8

ACGCCACTATTCCCACAAATACAACAAACCCAACAACAACCTCAAT

>NL03.C02.02_A_9

TTGAGTATATGGACTTTAACGGTAACTGGCATACAAATAG

>NL03.C02.02_A_10

GTTAGTACTGAAATCGTGATCTCTTTCCTGTTTTTACTTGCT

>NL03.C02.02_A_11

TTTTCTCCCTATTCCTGGTAATTGTACTAACGCTAAAGCT

>NL03.C02.02_A_12

TCATCACTTACATATGTATAGATAGTTCCCTTCATTTTTCT

>NL03.C02.02_A_13

CTGATATCTATTAAAATCTTGTTATTCTTAGTAGAAAGGTCT

>NL03.C02.02_A_14

TTAATTGCTGACGATTTAAATAAACTCATTGACGCAATTGGT

>NL03.C02.02_A_15

ATGTTTACATGCAGTTGTGATGGTGGTATGATATTGACAG

>NL03.C02.02_A_16

TAAATCTGCGTAATTTTTACACTTTCATCTTTCCCCTTGT

>NL03.C02.02_A_17

AACATGGGGGTGAACATATGGTGATACTCGATTTTATTTC

>NL03.C02.02_A_18

TTCTTTTTCTAGATAGTATTTTAAATCACGGTCACTTAA

>NL03.C02.02_A_19

TAAGCTCTGGAGGTAATTTGAATGAAATCACGTCCATGTCCC

>NL03.C02.02_A_20

TCGGTCTAGATGGATTATTATTCATCTTCTGGAATTTTATAAT

>NL03.C02.02_A_21

ATATTGTACGTTAAAACAGTGCTAAACTATGATGAATCTA

>NL03.C02.02_A_22

TAAATTATCCAAATATGTTACATTGTCACCGTTTTCA

>NL03.C02.02_A_23

TAAGGTTTGCGAAGGAGGTGTGCGGTGACGCTTGCAATAA

>NL03.C02.02_A_24

CAGCAGGAATTCTGTCTAGGTATCTTATCATATTTGGG

>NL03.C02.02_A_25

AATAATTTTCCAACTATGATAAAAAATACTAATTTTCTAA

>NL03.C02.02_A_26

TCAGCATACTTCCTAGCCTTACAGAACAACTATACTCAAG

>NL03.C02.02_A_27

ATTTTTGTCCCCATTTTTGTCAGGGAAAAATGAGACAAAA

>NL03.C02.02_A_28

TTTTTGGTCAGTATATTTTTATGCCAACCCGGTATTTA

>NL03.C02.02_A_29

TCGCCGCCCCCGTGAATTTTTGCAAACCGAGAAAAGTGAGT

>NL03.C02.02_A_30

TTTCCTTCTAATCGTAGCAACTACATTAGCACCACTTAGAATG

>NL03.C02.02_A_31

CTTCCCGAGTCGGGTCAGAAAAAACAAAGCACAAAAAA

>NL03.C02.02_A_32

TTAGCTTATACTTTTGGTGAAGGAAATCAACAGCCATTTA

>NL03.C02.02_A_33

TGCTGTTAGGTATTGTCCTGAACACGAAGCCGATGAAGAA

>NL03.C02.02_A_34

TGAACCTATCGAGGAATGCTCTGTCCGCTTGTGTGTTTACCCT

>NL03.C02.02_A_35

ACAGCATTTCTCGTTGTTGCTTTAGGCATGATCTCTATATT

>NL03.C02.02_A_36

AAAAGAACAAAAGAAAGAGAAAAAAAGAGAAAAGAAA

>NL03.C02.02_A_37

CATCTTTTTCTCCCCTCCCTATAATATAACACATTCTGGTTTT

>NL03.C02.02_A_38

AATATACAATATTTAAAATATATAATAAAAAATTTTGATAGTT

>NL03.C02.02_A_39

CGAGGGGAAAGCTAAGGAGGTAGACTTCAAAGAAGAAGAGAA

>NL03.C02.02_A_40

TTGCATACAATTGCGGGTGATAGAAAAAAATAGAAAACGTGAAAA

>NL03.C02.02_A_41

TTAAACGTAATGGATGGCGGGGCTACAGTTTGCGGTGCCAAATAT

>NL03.C02.02_A_42

CAATTGACCGGCTTATTATACATAAACGCTAGTTTGAGG

>NL03.C02.02_A_43

TCGCTTTATACCATTTGTGGACTCTTCAGATAGTCCCTCTGA

>NL03.C02.02_A_44

ACAGCTTGCACAAGTTTTGCCAAAAATAGTTAGTTCTCAGT

>NL03.C02.02_A_45

TGAGCAGTTATCGGTATATTGTTCTTCAGTGCTATAGCAATCT

>NL03.C02.02_A_46

CAATACGCCTATAACCACTCTTTTTCACTTTCGCTATAATACACT

>NL03.C02.02_A_47

CTTCTTCGGACGAGTTGAAAAGATAATAGTATGTATCTGG

>NL03.C02.02_A_48

GTTATGTAGAATTTGTCCATAAGTTTTATGAACATCTG

>NL03.C02.02_A_49

AGGATTGAGAAACAACACATCGATTTTGCAATTGCACATAT

>NL03.C02.02_A_50

TCTCCTTCATGATGAACCACCTGAAGGCTTCTTTAGCCT

>NL03.C02.02_A_51

TTATTATTATTTACGATTGCTAATGAGTAAAGTGGAATATTG

>NL03.C02.02_A_52

GCAATAATAGATAAATTAGCTGAAATTCTTCCTGAGAT

>NL03.C02.02_A_53

TGCCGAGTGAGAAGTACCTAAGAGAAGTGTGGGGTCTTGA

>NL03.C02.02_A_54

AGAATTGCCCCTTTCAAGGTGGGGAGGAAGACAGCGAA

>NL03.C02.02_A_55

TTACCCTCAGCTGTTTGGTCTCCTATGTTAGGTGAAAATA

>NL03.C02.02_A_56

ACTGTAGCTAAATAGACATAGTCAAGTACATATTCTAT

>NL03.C02.02_A_57

AATAATATTTCGCCTCATTTCTCCCTTCTTTTATTGACTGTTC

>NL03.C02.02_A_58

CATCTATTCGGCGTTCGGTAAGAACGTTTTCACTGAAATCT

>NL03.C02.02_A_59

TGATAGTTCCTTATATTCATTTTCCGACATTGACAACGCA

>NL03.C02.02_A_60

TCCCCCATCGTCAATCCACCTCCATCTCCAATTGTCAACCC

>NL03.C02.02_A_61

GACCTTTTCAGCAAACTCTTCACGCTCTTTTCTGCTTAAA

>NL03.C02.02_A_62

TCGTTAATTCTATAAAACACAGTTGCCAGTGAACCTAT

>NL03.C02.02_A_63

GAGACCTTGATAAGATTATTACTACGCGACGATTTG

>NL03.C02.02_A_64

ACACACGGTGCAGTATCGTATGCTTCTAGAATTAATTT

>NL03.C02.02_A_65

TTTATTACGATAGTCACAGCCATAATAGTAACCTTCAAGA

>NL03.C02.02_A_66

CCTCAGCATTGCGTCATCCATATCGTTTATTGAAGCATTAG

>NL03.C02.02_A_67

ATTCTTTGTACCACAATTTCTCTTTGCATGAGCAATGTGT

>NL03.C02.02_A_68

GTAAAGTCATTTGCCAAAACGATGATAAGCAATAAAAACGG

>NL03.C02.02_A_69

AACTAGTCCTTTTTTCTCTAGATTCTTTATTCTTTTATT

>NL03.C02.02_A_70

AATTATTTCTTTAAATGCTGAAATTGTTGAACAAATTG

>NL03.C02.02_A_71

TTTCAATTCTATAGTAGATTAGCATGCAGTAAT

>NL03.C02.02_A_72

CCCTTTAGGGCGGGGGTTCCCCGAGGTCTCAGGCGTTACACCCCT

>NL03.C02.02_A_73

ACATCACGTATCTCTGCTAATAAAATATGGTCTATTGAACA

>NL03.C02.02_A_74

ATTTCATTTCCAGTTCTTAGAGGTGAAGAAATTGATCTAA

>NL03.C02.02_A_75

ACTATATTTATTTTTCTTTATCTTACCGTTTTTCCTGTTAA

>NL03.C02.02_A_76

GTTGACGCTACAGCGTTCGGCATAGGCGTTATGAATGTGTAA

>NL03.C02.02_A_77

TCTTATGGAGTTGACGCAAAATACATCAAAAAGCATGAAG

>NL03.C02.02_A_78

ACGTTTATAGTTTTAAAGTATTCATCTATCGCTTCTCTTACTTTA

>NL03.C02.02_A_79

TTTATAGCTTTGTGAGAGGTATCCCATTTACCCTGTATGT

>NL03.C02.02_A_80

TTGCCAACATGTGTCCTGTAGTTCCTAGGTTCATTG

>NL03.C02.02_A_81

TCCTTTATTAATGTTTGCAAGAAAGCACCACCATATACGG

>NL03.C02.02_A_82

GTGATGTTTGCTCGGCATTGTTTATTGCAAAATCGACGTG

>NL03.C02.02_A_83

TTTTTACTCTTATTATCCTTATCACCACGAGAAGGAAAAG

>NL03.C02.02_A_84

TGGAAAACTGGCAATTCAACATTTAGACCTACTTCTTGAA

>NL03.C02.02_A_85

AACTGCCTATCGATAATGTTCTCAAATAGTGTTTTTA

>NL03.C02.02_A_86

ACTTCTGTGTATCTGTTATTGCCAATTGTTATTGTAATTTT

>NL03.C02.02_A_87

TTTCTGTCCATGCGTTTTATCGTAATATTCTTCAACTTCTG

>NL03.C02.02_A_88

CCTGCGATGTCGACCAGAAAGACCTGCGTATAGAAAAAAG

>NL03.C02.02_A_89

TAAAGCCCAATTTGAAGCACTAGAAAAAGCAAAAAGGTGAAAA

>NL03.C02.02_A_90

CAATTTTTGGAAGTTTGTTTAAATTACATTTATATGATTCT

>NL03.C02.02_A_91

TAAGCAGGAACAAGTAAACACGCAACAATTATCCAACATACTG

>NL03.C02.02_A_92

CCGTTTATTGGAATTTTTTTAGTCAAAATGTAATACATTAC

>NL03.C02.02_A_93

CTTCAGGACTCAAATAGGTAAAGTTAATTTCATTTCCATCC

>NL03.C02.02_A_94

AAGAAAAAACAACACACCAGTTCAAATCAGTTATATCGATAGCTA

>NL03.C02.02_A_95

CATAATGTAGTTCATAACTTGCAAAATACAAATTCTTTAT

>NL03.C02.02_A_96

TTCATGGTCAGAAACCAACAAACAGCCTAAGTTTTCCAG

>NL03.C02.02_A_97

TTCTTAAGCGAGGTGACCAACCCATGATTTTCAATTCACA

>NL03.C02.02_A_98

GTCACTTTTCACTTTACCTTCTTTGAAATTTGTTATTTT

>NL03.C02.02_A_99

ATCGTAGAGTACGGGAGATTTATAGGATGGAAACAAATAACACC

>NL03.C02.02_A_100

CACTCATTTTTATACTGAACCCCCTTAAAGATTCTGATGA

>NL03.C02.02_A_101

TGCTTATCTTGAAAATACGGATATTTCTCATCTTTGTAAT

>NL03.C02.02_A_102

AATTCGTTAAACCAAGCTTTTACCATTTGCTTTCATCCCT

>NL03.C02.02_A_103

TTACAGCATTCATTCCTCTTATTCTAGATTCTAGCATATT

>NL03.C02.02_A_104

GTTCATAACTACTCACCCACACTGTAATGTAGAATAAGAAATAGT

>NL03.C02.02_A_105

TTATTCATCAAAAATTTGTAGATTGCATACTCAATTTTTCTGT

>NL03.C02.02_A_106

ACAGTTGTGCTTGCAGTCGTTGCTTTGATTTATTCTGTCTG

>NL03.C02.02_A_107

AGCTATTAATATCAATAGAAGACTTATTCGATGTATTTGTGTTG

>NL03.C02.02_A_108

TAATTGTATCGTGAAAAACTGCGGGTTGCAGAAAAGTTTAT

>NL03.C02.02_A_109

TTTACCCTTTAAGTGGTTATCAAGTATTCCCGCTACGGTCTT

>NL03.C02.02_A_110

GATTAACATTTATTACAAATTTCCAAAGTTTGAATTTGC

>NL03.C02.02_A_111

TCTTCAGTCTTTTCGCTCACGTCTACGCCCTTTGAGCTGAAT

>NL03.C02.02_A_112

ACTTCTTATGCCCAGTTATCTTTGCAATAATTGCTGGGTCTA

>NL03.C02.02_A_113

AAACCACCAACACCTATAATCATAAAAAGTTTATCTTCCC

>NL03.C02.02_A_114

CTAGTGTTACTTGTCCACTTGTTGCTAGGAAGTAGGA

>NL03.C02.02_A_115

TTATTTCATTAGAACTTAACAATTCATAAACTTCATCACTTAG

>NL03.C02.02_A_116

GGATATTGCATACTCACTACACCAACATTACCGTTCCCTA

>NL03.C02.02_A_117

TTCTTTTGCATTACATATAGTCTCTTCATCATTACTGCAT

>NL03.C02.02_A_118

AATAAACGCCAAGAATTTAGTAAATTCAGGGAATTTC

>NL03.C02.02_A_119

CTCATTCTTGAAAACGCTAGCCAGCGCTTTATACAAGT

>NL03.C02.02_A_120

GGATAGACATGTTTGTATTCTTCACAAAACCACTTAAGTAAG

>NL03.C02.02_A_121

TTCTTGGCAGGAGGTAAAATGACCCCTTGCCCGCCAGCTTT

>NL03.C02.02_A_122

CTTTAGATGAATTGATAAAGGCATTTCAAAATAAGCCTAA

>NL03.C02.02_A_123

CTATTATTGAAGTTTACTCTTGCTTATGTGCAGACCCATT

>NL03.C02.02_A_124

TCCCAAGGCTGGGAGGGTTTTCGTCTCGTCATCAATCAGG

>NL03.C02.02_A_125

CAATAATGCTGGGTTTAAATGAGTCTCAATATCATCCCAT

>NL03.C02.02_A_126

TTCACCACCCCCACCACCACAAGTCAAACCTTCACCCA

>NL03.C02.02_A_127

CATCTCCTATATCCGTAACATATAACGAAACGACACTAACTTT

>NL03.C02.02_A_128

ATTTCTCTTGCTTCTTTCAAATACTCCAAAAGTTTAGGTG

>NL03.C02.02_A_129

TGAATTACTTTCTCATTATCCACAGTTTGTGAAATTTGG

>NL03.C02.02_A_130

ATATCTACAAGAAGCCTCGCAAATAGCACAACAGAACGA

>NL03.C02.02_A_131

ATAATATATTATTAAAAATATGAACATCAAAAAAAGAACGTCCA

>NL03.C02.02_A_132

CGATATGGGACATGCCAGATGACGGTAAACCTAACCCAT

>NL03.C02.02_A_133

ATAGAGAATACTTGGACGCGTTAGCTGAATTTGACAGGACTG

>NL03.C02.02_A_134

GGGGATTAGGTTATGCTCCTGAAGATGCATTGGAAGATGCAGCT

>NL03.C02.02_A_135

TCCATATGTTTTTCTAGTTCTGTGCGTATGTCTGCCATACTTAT

>NL03.C02.02_A_136

ACTTTACGCGAGGAATGAGGTGAATGAGGAACAGCTGATG

>NL03.C02.02_A_137

TGATATAGTCTATGGTATCGGGGTCAATAGGCTCTCCACATT

>NL03.C02.02_A_138

TCCCTACAACGAGTTTCATTTACTTTACTTTATGCCTCCAT

>NL03.C02.02_A_139

TCTTGTTGTTTTTCTCCTTTTCCGCTTTGCTTCTTTTCGCCT

>NL03.C02.02_A_140

CGTGCTTTACATAATACCTCGTTGTCATATCTGCCCAGGTCT

>NL03.C02.02_A_141

ACACCTAGTCCCCTTTATCGTATTATAATAGATCAGCTGAA

>NL03.C02.02_A_142

CCGTCACCTCCACCACCTGTATTCTCTTCAACACAGAG

>NL03.C02.02_A_143

TATCGATGTGCACTCTCCTCCTCGTTTGAACGTCCCTTC

>NL03.C02.02_A_144

GATAAAAATGACGAGATCCAATTAACACCAGCAATATCAAGAA

>NL03.C02.02_A_145

GTTTAGGAATTAATAATTGCAATTGTTTACTAACTTTAGAT

>NL03.C02.02_A_146

CACTTCTGTGTAGTCTGTTATTAATGGCATATAGGTCTAACAC

>NL03.C02.02_A_147

CAGTGTCTGATGAAAGGTGACACGGGTTTTTGTTATATGT

>NL03.C02.02_A_148

ATTAGTGTTAGAGACGACGACGAATTTGATAGCTTAGTTT

>NL03.C02.02_A_149

TCATTAATTTCCTTGAATTTACCATTATGATATTTGTAA

>NL03.C02.02_A_150

CCTTATATATGCATACTTGCTCCTCCTCTTTTGGAAGATT

>NL03.C02.02_A_151

CTTTCATCTTTGTCTCCAGGTCTGAAGGCAAGGGAATATTA

>NL03.C02.02_A_152

CCTTTCGCCACTATTTCGCAGAACTCCCTAACATCGA

>NL03.C02.02_A_153

AATGTACTAAATTCCGTGGCGTCCATGTACGGCATCGAGCCAG

>NL03.C02.02_A_154

AGATGACGTTACTTTATATATGATCACGAGACCACCTTTGG

>NL03.C02.02_A_155

TTTTAAAGCTGTGCGAAAAAGAAAAATTTAAAAATTGTGA

>NL03.C02.02_A_156

ATTTATATCGCTGGATTTATAGCTACTGCAAATCAGCAA

>NL03.C02.02_A_157

TAATTGTTTCGAATACTTTGTAATATACATTAAGTAATAAG

>NL03.C02.02_A_158

AGTGAAATTCAACTAACTGCACAAACTCCAACTTTACTAAG

>NL03.C02.02_A_159

TTAATCATATTTACTAGAGCAGGCCCGCCCCCAAGTCTGTTT

>NL03.C02.02_A_160

TTCTTCAATAAACAATATCACTACATCACTTTTGGGAATA

>NL03.C02.02_A_161

TTTTTTCGTAAAACTTGTGCAATATCCTCTGAAACATTTCG

>NL03.C02.02_A_162

TGTGATACTGAGTATATGTAGACTTCTTTCTTATGTT

>NL03.C02.02_A_163

TTAATGTAAGATTTTAAAAAATGACAAATATAAATCATTC

>NL03.C02.02_A_164

CATAAGATCCGCTATCTCGTTTAATAGTAGTGCTAATTCTA

>NL03.C02.02_A_165

TCCATGTAGTATTTTCTAGATAGTTCGACAAACTGTTTCTC

>NL03.C02.02_A_166

AATTGCCTAATTACATAATTTCCTAATTTCTCCTCCAAT

>NL03.C02.02_A_167

ACTAGGCTCAAACATACGTTTCTTCCAATACCCTAACCTAA

>NL03.C02.02_A_168

AGACTCTTTCGCTCCAGACCAAGTGTTAGACTTAAGAG

>NL03.C02.02_A_169

TATTATTGCCCCGCTCAATGGGTCTCTTACTTCCTCCTTCTC

>NL03.C02.02_A_170

ATGGGGCCTCCTCCAACATTGCCGGCGATGGTGCCAGGT

>NL03.C02.02_A_171

AAAAACATAGTAGAAATGTTAATGACCAACAAAAGTGAAT

>NL03.C02.02_A_172

GTCAGCTGTCTAATTCTCTCAATTTCTGAAACTAGCTTTT

>NL03.C02.02_A_173

ACCCCTTAAAACCGCACTCTACTGTGCAGGAAGAAACCCA

>NL03.C02.02_A_174

CAACTCATATGAGCTATTCCAGCCAAAAAAATTTAAATTTGCA

>NL03.C02.02_A_175

GTACCTTTACTCCATGGCTAAAGCGTTTTCCATATATCACG

>NL03.C02.02_A_176

CCAAAATAACTACATGTACTTGGTGTTATAAATCCTACAGT

>NL03.C02.03_A_1

CTTATTATTCGTTGTATTTACGCTTCGTAACAACAGTATACT

>NL03.C02.03_A_2

TACGAAATAAATGACGAACAGTTAATCGAAATTATTAA

>NL03.C02.03_A_3

CAAAACAATCCGTTTTCTAACTGCTTAATTGCGGAAGCCA

>NL03.C02.03_A_4

ATCCTCTCCTTAACAAAGATGTTGTATATTCTACCGATATATCTTA

>NL03.C02.03_A_5

GTTGATATAAACGTCTGTGAAAAAGTAAAAATATATGATAG

>NL03.C02.03_A_6

CTTGCTAACTTGTTTTGCAAAAAGTTTAAATACTTATTT

>NL03.C02.03_A_7

TTTCTTCTCAGCAAGATAGTCAATAACACTGTTCAAGTACT

>NL03.C02.03_A_8

ATTTGAACTGTTTTCATTTTCATATCACCAATATATAGTATGTA

>NL03.C02.03_A_9

AAGTTATACTTTTATAAATATATAAATATGTCATATTATAAGT

>NL03.C02.03_A_10

CACCTTGGTGAGTATCAAGTCAAATACGAATATCACAGACA

>NL03.C02.03_A_11

TTTTTGACCATGAGTTTTGTCATAATATTCCTCAACTTCAC

>NL03.C02.03_A_12

TTAAGTGCAAATGCATTAGTGAGTTTAAAGAAATTGAT

>NL03.C02.03_A_13

AATATCGTTATGAATTTGTCGTTTGGTAATGGTCGTTTGC

>NL03.C02.03_A_14

TCTCATATGGTATAATCGTAAAGCAAGCAAGATCTGCCTT

>NL03.C02.03_A_15

AGTTGTTGTGCAAATTGTAAATCTGAATCTAAAACACCGTAG

>NL03.C02.03_A_16

AGTATGAGCTAGAGGAAGTCGCATTGGGTCTTAACTGGAGAT

>NL03.C02.03_A_17

ACTGTGGATCACTTGAGCAAAAATAATCTTGACAGAA

>NL03.C02.03_A_18

TAATTATGGGGATACGGTTAACTTCCAACTATTATT

>NL03.C02.03_A_19

TTTAATAAATGTAAAGCCTAAGCCACAGCCAACTACTTCCC

>NL03.C02.03_A_20

ATTACAATTGGTAAATTTTTGCCAGCAATAATAGATAAATT

>NL03.C02.03_A_21

CTAGCATTGGGGGTTTTAGGGGGATACCCCCTAACTAGAC

>NL03.C02.03_A_22

TAATTTTCTGATAAATCGGGATTAATACTTTATAATAGCTCTGAT

>NL03.C02.03_A_23

TTCACTAGCCACGTGCCACTCATATATGTACTTGAATTGTA

>NL03.C02.03_A_24

AAAATCACTATGCCATAGCGAAATCATTATTAGCTTAATTT

>NL03.C02.03_A_25

AATTTTTAGGGCTAACTGAGAAAAAAGTTAGGAAATATT

>NL03.C02.03_A_26

GGCTATGTACTCGTAAACGGTGTGCGAATAAGGAGGAAAAT

>NL03.C02.03_A_27

GTTTTTACATAGTTATACTTGGAGGTGAAAAAATGAGTAAAGA

>NL03.C02.03_A_28

TGTCGATGACCCTAGGTTTTGTCGAGTTAAGCATCTTCGG

>NL03.C02.03_A_29

AGTATGAGCTAGAAGTGGATCTTACAACTACTGTTTCTTT

>NL03.C02.03_A_30

TCCTTAAGCTTCACTACGACTCTACTGTCTAGGTTCTGT

>NL03.C02.03_A_31

CTATTATTGAAGTTTGCTCTTGCTTGTGTGCAGTCGACTGATTAT

>NL03.C02.03_A_32

ACCGTCGCTGAAATACGTTAGTGTCAACTCGCCCTCC

>NL03.C02.03_A_33

TTTTCTCGAATTATTTCTTCAGAATATGGCACTATTTTTATCCATTC

>NL03.C02.03_A_34

TATAGCGCCGATTGTTCCATATTTACCTATTTTAAGTA

>NL03.C02.03_A_35

TAAAGAGTTGTCCATTTACAATATTTCCAATAATTGTATGT

>NL03.C02.03_A_36

AAAGAAAGGTGCAACACATCGACCGCAAACGCATAAATAA

>NL03.C02.03_A_37

TTGTTAGATTACAAGTATTGGCAGGAGAGAACAACGCCAG

>NL03.C02.03_A_38

GGCTGTTGACTCAACACTATCCACGGGCTGGTTTTGAATGC

>NL03.C02.03_A_39

TAAATAAACCCCGTAGCTCACCTCGCCTAGGAAAATAGT

>NL03.C02.03_A_40

TTAATTTGTTCTAACTGAATTGAAAAAACTAACTTAATTT

>NL03.C02.03_A_41

CATAAAGTACTACGTCATTGTTATAGAACTTCTTCCTTTCCAG

>NL03.C02.03_A_42

TACATATTATTTCTAGCATCATAGACTAAATTGGCATAAGTCGG

>NL03.C02.03_A_43

TATAGCTACTGCAGGTATCAATACGTATTACTTGCCTCTG

>NL03.C02.03_A_44

TGGCTTAATGCTGTAGTTGATATGATAATGTTGTATTTTCC

>NL03.C02.03_A_45

CACGGTGACTTTGCGGAAGAAATGCTATCGTTCTATGCAAAT

>NL03.C02.03_A_46

ATTATAACAATGATGTCATCTGGATTCATCCAGACCACTCAA

>NL03.C02.03_A_47

CTTATAGGGTCTCTAACCTCTCCATTCACTTCCTCTTCTACCTC

>NL03.C02.03_A_48

AACGGAAATTCTGTAAGATATTCAATGACTATACATAGCCAA

>NL03.C02.03_A_49

AAAAGTTGCTAGGGCACTTAATAGACTCGGCGGCGGGCCTGCTC

>NL03.C02.03_A_50

CCTTATATATGCGTACTTGCTCCTCCTCTTTTGGAAGATT

>NL03.C02.03_A_51

TTTTTTAGAATTGAAGTTACTGCATTTCCGCCTGTCTCATAC

>NL03.C02.03_A_52

CCAGGTTCCTCCTTAGGACTTTAGGGTCTTTTCTCTGCT

>NL03.C02.03_A_53

TTTACTTCAGCTAGCTGAATAAAAAAATTTAAATTAA

>NL03.C02.03_A_54

ATAGCCTTCTTTCTCTTGTAAATTTCTTCAATCACTTCCGC

>NL03.C02.03_A_55

GATAATGAGAAACCCTGGTGATTACAGAAAAGAGGTAGAG

>NL03.C02.03_A_56

ACCCCAGCGGAAAATTCACAGTCACGTTCAACGAGGTGGG

>NL03.C02.03_A_57

AAACCAAATTCCAACAGACCCTGAAACAGACGTTTGCA

>NL03.C02.03_A_58

CTTCTTTTTAACCGTTAGATTTTTAAACCCCGTTCTAGT

>NL03.C02.03_A_59

ATTAGGAAACAAGTAAAAGTATTAAAAGCCTTGGTGATCAAT

>NL03.C02.03_A_60

GCTTTATGCATGGATTGAAAGTTACAATTCTAATTTATCAAC

>NL03.C02.03_A_61

AAGGAGTTGTGGAAACATCCAGTTGAAGGCCTTCTATAGT

>NL03.C02.03_A_62

TTCGGTAATCGTACTGGTGTCCCTACTGGTATTGACACTGG

>NL03.C02.03_A_63

CGGGAATTACGCACCGCCCCTCCTCGAACAAATGCTG

>NL03.C02.03_A_64

ATGGCTGACGACGATGACAACTATAAAGACAAGTTTAAGT

>NL03.C02.03_A_65

AAAACTGTTCTTTCCTTTTTTGTTCTCTCCATCATTTCTCCT

>NL03.C02.03_A_66

TAGATAAAATATTAGAAGTTGTAGATAAATTAGATGATTATAA

>NL03.C02.03_A_67

TTCAGATATCCAATTTCAATAATTCATCCATTCTTCTAT

>NL03.C02.03_A_68

AGAGAAACAGCAGGTACAAAATCAACTCAGCCAAGTACAA

>NL03.C02.03_A_69

ATATGCAGTCGATATTATTACAAGGACTGACCCCACAAA

>NL03.C02.03_A_70

CTTTCCAATATAAGTTAATATTTCTTGAAAGAGTCCCTTAG

>NL03.C02.03_A_71

GTATTGGCAGTGGTACAATACCGATGATTGCGTCGTA

>NL03.C02.03_A_72

TTATGATTGCTTTCCAAGTTTGACCATTCCAGAATAGG

>NL03.C02.03_A_73

TCTTCTCTTCTTTAGTACTAGAAACTTGACTCATCAATAT

>NL03.C02.03_A_74

AATGCCATCTATCTCACCCGTGCTTGTGCTTCTTCTTGTGA

>NL03.C02.03_A_75

ATTTTCACGCTGTTAAGTGTCGGGACGGCCAAGCCGA

>NL03.C02.03_A_76

TTAGCATACCACTATTTTTAATACCCTATATGATACTATCA

>NL03.C02.03_A_77

AAGAAGGTACAACAGACAAATACATTGTGTTAACAAATCTAA

>NL03.C02.03_A_78

ATATCATAAAAATCCAATACTTTTGCATGCAATTGCGGG

>NL03.C02.03_A_79

AATCAGGTATAACGACATAATGGTTGAATGCAGGCCTACAAG

>NL03.C02.03_A_80

ACAATATAGGAAGTCGCATTGGGTCTTAACTGGAGAT

>NL03.C02.03_A_81

GATTATATAATATGAATTATTTGCTTAAAAAGTAGTCGAGA

>NL03.C02.04_A_1

ATTATTTACGCTGGAAATCCATATGCATATACAATTAATAA

>NL03.C02.04_A_2

GAAGAGTATGAAACCCACCGCATTTTGAAAGACATCGAGA

>NL03.C02.04_A_3

ATGGTGTCAAACGTATCATTGCCGTTCATCGCTCCACCAA

>NL03.C02.04_A_4

AGCTATTAATAAGCTTAAAATGGATAAGAAGGCAGCTAAG

>NL03.C02.04_A_5

TTCAGGTACAGGTTTAAGATAGTATGAGAAGAAATAAAG

>NL03.C02.04_A_6

AAACATAATAAAAATGCCTACCAAAAACTCCTTTATTTATTT

>NL03.C02.04_A_7

AACCCACACTGGTACGCCAAATACCTTTTTGTTTAGGT

>NL03.C02.04_A_8

TTTTGTGCATGTAGAAGAGGAGTAAATGGATATGGTTGAAAGTTTT

>NL03.C02.04_A_9

TTACAAATGGGGCACCAAGTGATGTCGTATACTGCAAG

>NL03.C02.04_A_10

TTTGATACCGTTATGATAGACTGAACCAGTTAGTATGCCA

>NL03.C02.04_A_11

GTTTAGAAGGAGAAAGATTGACAATTCAAAGAAGAAAGGGACT

>NL03.C02.04_A_12

GATATTTAAATTTTCTGCATAACCAGTTAGGAGGAAAGCGA

>NL03.C02.04_A_13

CGCCCCTTCCAGGGCGGGGAGGGGGTAAGGCTTCTAATACG

>NL03.C02.04_A_14

AAGAAAAACTGTTTTCTTCAAATGGACCTCCCAAAATAAC

>NL03.C02.04_A_15

TTCTTTTTATGTGAAATTTGACATAATTATCAAGCTTTAGA

>NL03.C02.04_A_16

GGTAGTCTCATGCACCATAAACTATTACTGTAATCAACCC

>NL03.C02.04_A_17

GCTATACCACCAAACGCTACTACTACTGGAGGAGGAAA

>NL03.C02.04_A_18

TTTTCTCATGGGGCAAAGCCCCCGAGGTGAAAAAAATGGCAG

>NL03.C02.04_A_19

GCTACTGTGGTATATAGCATGACAACTGGGTTACCATA

>NL03.C02.04_A_20

TTCACAATCGACTTTAAGTACAGATCCAGAAGGAATATTG

>NL03.C02.04_A_21

ACTATAATATTATAATTTATAATTTTGTCAAACCAAAACTCC

>NL03.C02.04_A_22

GTCACAGTTGTATCTTTAATACTCTCAACAGCTACCACAGTCGA

>NL03.C02.04_A_23

TTTTCAGTTTCTATATTAATTGTATTTGCTGAGGTATAAT

>NL03.C02.04_A_24

TATTTGAACCCGTTAAGGTTTGAAGTTGCCATTAGGTCGC

>NL03.C02.04_A_25

GATTTATGCTAATACACCTATGGTTGAAAGCACAAGTTGG

>NL03.C02.04_A_26

CATTCGAGGAATGTTTAAGTCTGATAAATCCTCAAGGGTTA

>NL03.C02.04_A_27

ACTGAATATCTTTCTAAGTAATCCGTTAGTCGCTATTAGTTC

>NL03.C02.04_A_28

CGAAGTTATTCGCAAAGATATGCAAAATGTTCAGCTAAATT

>NL03.C02.04_A_29

ATGCTTATAAACGTGAATTGGCAACTATAATACGGGGCG

>NL03.C02.04_A_30

ACTACTAGTCCCATAAGTATATCCTGGAGTACCTTCTGG

>NL03.C02.04_A_31

TATCATAAACCCAATCGAAGCAGGAATAGGTCACTTAGAA

>NL03.C02.04_A_32

GCTATGTTGTTGGTGAAGTTGTACATCTATATAATCAAA

>NL03.C02.04_A_33

AGTTGTATAAGTTCTTGAATCGTTATTGGCTTAGTTAATTCCGT

>NL03.C02.04_A_34

GCATTCCTGACAAATACATGGAAGAATTACAGAAACTGGG

>NL03.C02.04_A_35

TTCTTTTAAAAACGAATTGGGAAAAGATAATGACATGGGAA

>NL03.C02.04_A_36

AATATATCACTAAGCTTCTTAACATCTAATAACATCTCT

>NL03.C02.04_A_37

TGCAAAAACTCAGCTACAACGCTCTTATTAACTTCAACTA

>NL03.C02.04_A_38

TGAACTTGTAAAAAAAGGCTATTCAATAGCTCAAATTGCAAA

>NL03.C02.04_A_39

TGTGAATAACTAAATACATATAGTTTCTGATGTTTAACAA

>NL03.C02.04_A_40

TCTCATATGGTATAATCGTAAAGCAAGCAAGATCTGCCTT

>NL03.C02.04_A_41

AGTTCAAATCCGCAACCAAATGAAAATAAAGCGAGTCAA

>NL03.C02.04_A_42

TTTTTGTAAGCGAATTCGCCCTTAAGTACTTTCTCAACAA

>NL03.C02.04_A_43

TTGAAAAGGTGTCATTAAACTTCATATATAGGAATGAGACGA

>NL03.C02.04_A_44

GCATTCTCCCTACCACCGTCTTGGCGTTAGTTACTAAAT

>NL03.C02.04_A_45

AAGTTATACTCTGGGCGGTATCTATACAACTCTATTGCTT

>NL03.C02.04_A_46

TCTGGTATGCCCTCATACGATAATATACGATAACAAAATAT

>NL03.C02.04_A_47

TCTTGTGGTTCTCCTTCCTCTCCCTCCTCTTGATTTTCTCC

>NL03.C02.04_A_48

TTAGGCGGTGGAGCTGGAAAAAATAGCAGATATTTTATG

>NL03.C02.04_A_49

TCAATTTTCCATTTTTCAGGTTCCATAATTCCATTTTTGG

>NL03.C02.04_A_50

TCACTTTCTCAAATTGAAACTGCAATAAAGTATAACTTTCC

>NL03.C02.04_A_51

GTTACGTTTACGTTTCTGTGCAGCAAGGTAATTCGTTTAG

>NL03.C02.04_A_52

ATTACAGCGACGAGATAGAAGAGCTATGGAGGGAATTAGT

>NL03.C02.04_A_53

GTCACCACGTCTACTTCCCCGGCCTTGAGGCCGGCTTCCTTA

>NL03.C02.04_A_54

TCTCACCAATACATACTATGTCCTCAAAAGTATTTAAATTTT

>NL03.C02.04_A_55

TATTCAGATACCCCATACGATGAGTTGTATATTACAGTAAAAT

>NL03.C02.04_A_56

ACTGTTGCCATTTCACAAAACTCTCCCTATGTAATTA

>NL03.C02.04_A_57

GAAGACAATGTTATACCGATACTTGATACAGATAGAGGTAT

>NL03.C02.04_A_58

ATAGTAGTGATAAATAATGCATATGTATTAAATAATAAAT

>NL03.C02.04_A_59

AAATTAGTTAAGGCACGGCTTAAAAGTGATCTTAAAAAT

>NL03.C02.04_A_60

TATCTATCCCTGTAGACGAGAAGAAGCAAGTAAGTGTGAA

>NL03.C02.04_A_61

AACATTAAAATCCACTAATCCTACAATCCGAGAAAATTTC

>NL03.C02.04_A_62

AAATATCTTCGATTTTCTGTCCTTCTCCACCTTCTGATTCCT

>NL03.C02.04_A_63

TAAATAAACCCGAGTTGGTTCTTTCGTCGCCGACAAACGT

>NL03.C02.04_A_64

GGTATTCTGCTTTCGCATCTTCTAAAGCCTTCATCAAGTCT

>NL03.C02.04_A_65

TTTTTCCTGTTAACAGCAAAAGCTAGTCT

>NL03.C02.04_A_66

TTTACTAATGGAGAACAAGCACAAGAAGCCGGGACAGCAATACAA

>NL03.C02.04_A_67

TATTGTGAAGCTAGAATTACATCTCTTATATTTACTGGTCTA

>NL03.C02.04_A_68

AATAAACTATATTGAAATTTCAAACCGCGGAACTGGAA

>NL03.C02.04_A_69

CCCCTCACGGGCGTGGCATCACTGTTCAGTGCCCGCCGTCAGC

>NL03.C02.04_A_70

TCACCACCTTTCATCCCTGTAATATGTATTACCGGGCTGA

>NL03.C02.04_A_71

GTTGGGCAGAAAAGTTTAAATACTAGTTTTTGCATAGTTA

>NL03.C02.04_A_72

TACAGAACAACTATACTCAAGCTCTTAGGGTATTAAAG

>NL03.C02.04_A_73

TTTTCACAACATCAGCCTCTGAGATATCGAAATATTCCGC

>NL03.C02.04_A_74

TAAAATCTCTTATCCCCTACTTTTGCAATTACTCTTTGTCCTAC

>NL03.C02.04_A_75

AACGAAGTCAAAATTTCGGAAGCTAAATTGCTAATTGGGCC

>NL03.C02.04_A_76

TTATTTTGCGTAAAATTACGTTAAGAAAAATAAAAATAT

>NL03.C02.04_A_77

AGACCAACTGGCGATAAGTCAATTTCTACCTCTGGAAGTA

>NL03.C02.04_A_78

TATCTTGTAATTCGAATGGCACAAGTCTCCTCGGGAAGTT

>NL03.C02.04_A_79

TAAGCTGTAAGAGAAGAGGGTAAAAGACTCTTGAGCAT

>NL03.C02.04_A_80

TATCTTTCGATCCTTACTACTGGTACTTTAAAGATGAATTC

>NL03.C02.04_A_81

TTTACCCTGTATGTCCCTAGCGGCAAAAGAGGATATATA

>NL03.C02.04_A_82

CATGTACATATCAATATAGTCTTATCATCATCAGGCAAGT

>NL03.C02.04_A_83

ATCAGTTAGATTCAAGTTGTTGCTTGATCTTGATTACGATTA

>NL03.C02.04_A_84

GTCAGAAGTCCTGTTATTACAGCTGTTTTGACGTCTTCATT

>NL03.C02.04_A_85

ATAACGACCTGGGCGGTTTGTATGGCATTCAGATATCATGG

>NL03.C02.04_A_86

CTTTTCCAGATAGGACCGTTTGCCCTATCCTAAAACTAGTAG

>NL03.C02.04_A_87

AAAAGGACTGGTTGATCAGCGATTGTGAACATTTCAAAT

>NL03.C02.04_A_88

TTGACTCGAGAACGGGCATTCCGAATCCTTCAGTTCC

>NL03.C02.04_A_89

AGTAAGGTTTTTAAATGATGGGGGATATTCAACAAAACTA

>NL03.C02.04_A_90

TACTTATTTTTTCATCTTTGTAGCTTTCTAGTATATCGTA

>NL03.C02.04_A_91

AAATTGTCGGATTTAGAGATATATTTTGGTTTGACGGAT

>NL03.C02.04_A_92

AACGAAAAAGGTGTGAGAGGTGCTATTGCGACAACCTTACCG

>NL03.C02.04_A_93

AGGTGAAGAGAAAGCCAGCAGAATGCTTAACCAAAAAGCTCA

>NL03.C02.04_A_94

AGCAAGTATTATGATGTTAGCAATATAATAACGTACAGAGA

>NL03.C02.04_A_95

GTCAGCAAACACAATTATAAATAGAAATCAAGCATGTGAAAA

>NL03.C02.04_A_96

GTGGTAGAACTAGCACAGTTTGCAGATAAGTTAAAGCAC

>NL03.C02.04_A_97

AAGAGTAATGACGTGAAAAACTTATGTGATAGTATCGATATACCA

>NL03.C02.04_A_98

GACTTTCTTAATTTCTATCAGAGAGGCCCAGCGTTTTAGCC

>NL03.C02.04_A_99

CCAGGTTCCTCCTTAGGACTTTAGGGTCTTTTCTCTGCT

>NL03.C02.04_A_100

TCACCGCTTTACTCAGGTCCATCCATAGAAACTGTAAGAATAA

>NL03.C02.04_A_101

ACTCAGAACGTTCCGCCTCCAGGTAATATCGTTGCTAA

>NL03.C02.04_A_102

TCGTCACCAACGCCACGGGATTATATTGTGTCTGCTGGA

>NL03.C02.04_A_103

TATTTTTAAATTATTCGCCCCGTTATTTCCTTTGCTTTGC

>NL03.C02.04_A_104

CATGAACTACTAGACCTGGTTCGAATTAACGTAATCTTTTTT

>NL03.C02.04_A_105

TATTTCTAACTCTGTTGCTCACATGACTGAATTTGTCCATTTA

>NL03.C02.04_A_106

AAGTTATACTTTTATAAATATATAAATATGTCATATTATAAGT

>NL03.C02.04_A_107

TTGAAGAAGGTATGAAGTTGTCGGGGAATACTACAGTTGA

>NL03.C02.04_A_108

CTTGAAAAAACTGGAAAAGTGGTTGGAATAGACTTAGGAG

>NL03.C02.04_A_109

CCTAACCCACCGATACATAAACCAGAACCAATCGTTAA

>NL03.C02.04_A_110

TTATTCCGTTTATCTCGACGATATACTTTGCTGCGGAAT

>NL03.C02.04_A_111

AAACCAACCGCAGTATTATTCCCAACAATACAATGTATTGT

>NL03.C02.04_A_112

ATTCCTGATTGAATTAACACCAACAATCGTCACTAATTCAC

>NL03.C02.04_A_113

GTAAATTATAAAATAATAACGCGAAAGAATTAATTTTCCCC

>NL03.C02.04_A_114

TTCTAACGTTTTAGGAGTAAGTTCTACAGTAAAATTGAAA

>NL03.C02.04_A_115

GTTGAAAATTCCCATTAGAATGGGGATGCTAATAAACGTA

>NL03.C02.04_A_116

ATGTAAACGATATAATTACTGAGATTTTGGAGAATTATTCT

>NL03.C02.04_A_117

GATAACTTCCCACTTAGGCTCGTCCAAGTTCATGAACCTC

>NL03.C02.04_A_118

ATTATCGTTCATCAGCGTTGCGGTTTTGCATAACCTTGA

>NL03.C02.04_A_119

GTTATTGGTTCACCTTTTGCATAACATGTTGAAAAAACTT

>NL03.C02.04_A_120

ACAATAACACTAAAGCTAGCTGGAATTGAGGCGAACGCG

>NL03.C02.04_A_121

TGTAGATTTATCGGAATTATACTCATATGCATCTAGTTCA

>NL03.C02.04_A_122

GTGCCTCTGTTCTGCAGTCTACACAGAAAGCAAACTTTTAA

>NL03.C02.04_A_123

TAACCCAGGTCGTAGACCCGAAAGGAAACGCTGTATACGAA

>NL03.C02.04_A_124

TGACCTTGGACGCGAGTCTGATAGACTTTCGTAGGGACTGA

>NL03.C02.04_A_125

TAATAATCAAACTGCTAGACTTTTGCAAAGAGCAGTTGAACA

>NL03.C02.04_A_126

AGGTGGATTCTCAAAAGGGTTCTCGTTATCATATGAAAA

>NL03.C02.04_A_127

AATGCGATATCGCTAGCCTTCTGAAACACATTTTCCTTAG

>NL03.C02.04_A_128

ATCATGATAAGCCTCCTAATTTTCTGTCTACCAAAGCCTTG

>NL03.C02.04_A_129

TTAGCCTCCCCGCTAATAATCGTCGTCTGACCCGTAT

>NL03.C02.04_A_130

TTATTTAGACCTGTTTCAATTCGTGATGTAATTTTAG

>NL03.C02.04_A_131

GAAAAGTATATACAGTGGGTTGACGTTAGAGAGGTAGTCAA

>NL03.C02.04_A_132

ATTTATACTTTTTGCAGAATGGCATTTTAAGTTTTCATAATA

>NL03.C02.04_A_133

TAGTATTTGCTTAGGCCCCGGCGCTGTAGAATGTAAGGCAGT

>NL03.C02.04_A_134

GTTGGTGCAATTGAACCACTCGTGAAAACAGATGATAAA

>NL03.C02.04_A_135

AAAAAATGTTGCCAAATCGCCGAGATTCAGCAGGAAACTCAT

>NL03.C02.04_A_136

ATCTATGCTATAAAATATACTAGTGAAGTCATTTGCCAAAA

>NL03.C02.04_A_137

GGCATAATTCACATGCATGGCAATCTGAACATCAACAAACA

>NL03.C02.04_A_138

GTCATTATCAGGTAAGTTGGTAATGGTTTTTTCCACAGTCTG

>NL03.C02.04_A_139

TTTAATAAATGTAAAGCCTAAGCCACAGCCAACTACTTCCC

>NL03.C02.04_A_140

CCCTCAATAACGCAGGAGATGATGTAAAACTTAAAGA

>NL03.C02.04_A_141

TATAGATTGAGGGCTGAACTCATTAGGTGGGAACGT

>NL03.C02.04_A_142

GTATATTTTCTAGTCTGGTTATCTTGAGGTCATATAT

>NL03.C02.04_A_143

TATTCCTAGACGGAAAAAACCCGTACCTTTACTCAATGGCTAAAG

>NL03.C02.04_A_144

GAATCTACTGAACACTTTGCGAAATCAGCTGAAAGTTTT

>NL03.C02.04_A_145

ACTTTCTGCTTCTTGTTGCCCTTTTTTTATGTCTTCAAG

>NL03.C02.04_A_146

TTCCCTTACCTTTCACTTTCACGTATTTTTACATAAGCTAA

>NL03.C02.04_A_147

TATCCATATCGATACTCCGAATAATTTCGGATTTTGTGGAAC

>NL03.C02.04_A_148

TCGCATAGCATTTTTACGTTCTTATTTTTGATATCGAAA

>NL03.C02.04_A_149

TGCAACCCATGCTCTTCTGCCATGTAAGTTGTTCCTATACT

>NL03.C02.04_A_150

TTGCTTTGCCGTAAAACATATGTGTTCTTAAACTTTGTT

>NL03.C02.04_A_151

TAACGATAAAATTTTAACTTTGTGTTATTGGCTTTATTACG

>NL03.C02.04_A_152

GGGTTTTATAACGTAGTCGTTGTACCGTCTGAAAATAACA

>NL03.C02.04_A_153

AAATCAGATAGTATGAAGATCTGTTTTATGTCCTTTAATCC

>NL03.C02.04_A_154

TTAACACGTTACTTAACGGTGTAGATGTTGGAGTATTAGA

>NL03.C02.04_A_155

GTCAAAAGAGCCATAACTATACTTCCACTAATTTTCAATA

>NL03.C02.04_A_156

ATATAGACTATTACCCTGAAACTTGTGCTGATAATATAAAGGGACT

>NL03.C02.04_A_157

CGTCCTCTCACCGAAAGCTTTTTAAACCCCCCTTTCCCTT

>NL03.C02.04_A_158

AGTGATTTGCAAAATACTTATCTTGAATATGCAAGAAAT

>NL03.C02.04_A_159

AGAGATATGCCACATCCCTGTGTTTTATCTATTTTACCC

>NL03.C02.04_A_160

ATTCTTAGTACCACAGGATTATACAAAAACACAGCACCT

>NL03.C02.04_A_161

ATTACTTCAACGCTCACTACGTCTTTATTCAAACTACTTTAAA

>NL03.C02.04_A_162

AGAATCTTTTGTTGTCTTTATGTTAGGATTGGTTATGTT

>NL03.C02.04_A_163

GGTGTTACCAGAGAAGGTGGTTGTAAAATTGGCGAAAGAA

>NL03.C02.04_A_164

TCTTCTCTAGTCGAGAATAATAAATAAACGCCTAATTCTATC

>NL03.C02.04_A_165

AAATTATAGTTATTCATCTGGAAAAAGAACGCTTTCGTATAA

>NL03.C02.04_A_166

GTATGCTTCTTGCACTACAGGCATATATTGCTGTTGGACTTC

>NL03.C02.04_A_167

ATAAGTCCTTCGACTATCTCCTGCTGCTTTAGTAAGCTGT

>NL03.C02.04_A_168

GTTATCCATCAGCTCATGCCACCGTTTGATGAATTTACATCA

>NL03.C02.04_A_169

CTAGGTTGTTGTTGCAGTTTTGCAACTACAAGTATAT

>NL03.C02.04_A_170

GTATTGGCAGTGGTACAATACCGATGATTGCGTCGTA

>NL03.C02.04_A_171

AAGCAATATCTAAACTCAATAAGATACTATCTGCACACAGT

>NL03.C02.04_A_172

TAACAAATTCGAAATAGTTGTGGTTGAGGTGACTCACAAA

>NL03.C02.04_A_173

CTTAATTCCACCATTTTTTATCCCCCTGAGTATACTCCT

>NL03.C02.04_A_174

TTAAATTCGCAATTTTAGCATAAATAATTATTTGCTCCTGG

>NL03.C02.04_A_175

ACTTTTTCCCCAGAAGGATATACATATTTTATAATAA

>NL03.C02.04_A_176

TTCCTGTTTTTCGCCTGCCGTATCTGCCGAGTTACTACCCA

>NL03.C02.04_A_177

TTCTCAGTATCAAGCACTCCAACCTATTGCACAGCAATT

>NL03.C02.04_A_178

ATTGTCACTTACACGCAGTACATTACGCGGTGAGGCAGA

>NL03.C02.05_A_1

TAAGCTAGATCAAACTCGTGCCAATAACAGCATTCCAATT

>NL03.C02.05_A_2

CATACTAAGTCATGGGTAATTGTTGAATGGAGTAATAAGG

>NL03.C02.05_A_3

CAGCTTATTCATACATCCGGAAATGGAGATATAAGGTTCCGAT

>NL03.C02.05_A_4

ACCTTCTTACCCATAATTATTATATTCATAAAATGTTAA

>NL03.C02.05_A_5

ATAGATATATTTCTACATGACCATATTTTGCATATAAATGTCT

>NL03.C02.05_A_6

ACGGCGTAAAGTTGTAAATGCCAGGCTGGAAGTACATGTTG

>NL03.C02.05_A_7

AGTAGAAAAGAAGAAGATACGTCTTGGAAAAAAGGAAAAAGA

>NL03.C02.05_A_8

TGTGATAACTAAGCTTAATGTGTAAGGCGTTTTTTGTGC

>NL03.C02.05_A_9

CATACTAGAAGTTAATCTCTGGAAAGGCGAAGCCAACA

>NL03.C02.05_A_10

AAACATAATAAAAATGCCTACCAAAAACTCCTTTATTTATTT

>NL03.C02.05_A_11

CAACATAAGTGACAAGGAAAGAGGTAAAGCAATAGAGAATCT

>NL03.C02.05_A_12

AAAACTACACCAACCATAGCTGTAACATATGTCACTTTCTA

>NL03.C02.05_A_13

TTCTGTAATTGTTGTTTTTTAATATCATACGTTTTATTAGG

>NL03.C02.05_A_14

ATATTATACTACAAAGGGACAGAATCCAGCTTATCCGGCG

>NL03.C02.05_A_15

CAAATAACAAATTTCAAAGAAGGAAAAGTAAAAAACGATG

>NL03.C02.05_A_16

CATCTATCTCTTAATTTTTCTTCTTTGTCTGGCTATGTAT

>NL03.C02.05_A_17

GTTTAGAAGGAGAAAGATTGACAATTCAAAGAAGAAAGGGACT

>NL03.C02.05_A_18

TAAACTTCTTCAGGTAATTGATAAATTCGCGTTGTTGTCTC

>NL03.C02.05_A_19

TTTTTCAATATCTTCTAATGCTTTATACGCACTTTCAACG

>NL03.C02.05_A_20

CGAAGTGGTTCCTCAGGCAGTCTGTCTATACCGTGTTGTAACC

>NL03.C02.05_A_21

CCGGAATTGGCGTTACACCTTGATGAGATTTTTTTATCAA

>NL03.C02.05_A_22

GCACTGTTCTCAGTGAGGATTATGGTTTCTGTAAACACTT

>NL03.C02.05_A_23

AACTCTGATGCCGTCAGTGCGTTTATAATTTGTGCTAGTG

>NL03.C02.05_A_24

TTCAGATATCCAATTTCAATAATTCATCCATTCTTCTAT

>NL03.C02.05_A_25

TTAGCAACATCTTCAAGAAATTGACGCGCAGGAGTCGAATGAAT

>NL03.C02.05_A_26

ACACTAAACGCAAAAAACTAACAACAAATAACATGATGAAT

>NL03.C02.05_A_27

CTAATAAGAGAATTGTTAAAAGGGTTTAAAAGTTTTACG

>NL03.C02.05_A_28

CAAGACGGAAAAGTAAGGTATTCCATGACTATCCATTCGC

>NL03.C02.05_A_29

ACTATAATATTATAATTTATAATTTTGTCAAACCAAAACTCC

>NL03.C02.05_A_30

TATTGGCATTAACAGAAATAATATATTTAAATTTGAATAACTG

>NL03.C02.05_A_31

TATAATGTGTAAGCGAGCCACAACGCTAATGAGGATTTCCCTC

>NL03.C02.05_A_32

AAATTCGAATTAAGGGCTCTGATTATTAGAAAGGACAGTAT

>NL03.C02.05_A_33

AGTACGCAGTTCTCATTACGAATTAGCAATATTTTAATTTGTCA

>NL03.C02.05_A_34

GCAAATCAACTTCTAACCCAACTTCTTGCAAATTTTCAGC

>NL03.C02.05_A_35

GAGAATATAGATTTCATAAAGACGCTACAAAAAATTGCAA

>NL03.C02.05_A_36

GTATATTTTCTAGTCTGGTTATCTTGAGGTCATATAT

>NL03.C02.05_A_37

GATTTCGTAGCGTTTCCTTACGACCGCGAGACATTGAGGCC

>NL03.C02.05_A_38

GAGATAGAAAAATAGAAAAATCTGTAAAATTAGACTAGTTAA

>NL03.C02.05_A_39

AATAAATGACACATGGACTACATATCTAGGTGTAAATACAGG

>NL03.C02.05_A_40

TTTAGTATTACATCTTTGTTTGTACATTTTAATCTCCTCA

>NL03.C02.05_A_41

ACCCATTGTTTGTGCACGCCTACTCTCATTCTGCAACTTC

>NL03.C02.05_A_42

ACTGAATATCTTTCTAAGTAATCCGTTAGTCGCTATTAGTTC

>NL03.C02.05_A_43

TTTTTTTATCCCTCTGAATCAATTTATCGATTATGACAAATA

>NL03.C02.05_A_44

ATTGTCACTTACACGCAGTACATTACGCGGTGAGGCAGA

>NL03.C02.05_A_45

ATGCTTATAAACGTGAATTGGCAACTATAATACGGGGCG

>NL03.C02.05_A_46

TCGCCGCCCCCGTGAATTTTTGCAAACCGAGAAAAGTGAGT

>NL03.C02.05_A_47

ATTACTCTTGACGCTATTTCTTTGTTGCTACAAAGTTCTG

>NL03.C02.05_A_48

TTCCTCTATTGCTTCCTGTATCAATTTCTTTATTTGCTCA

>NL03.C02.05_A_49

TTGTCATGCGGAATATCTTTTCTAGTGTTTATCTTTAGCT

>NL03.C02.05_A_50

TCATGCACTCACCGTCATCTGGAATTGTGCAGTACATGATG

>NL03.C02.05_A_51

GTTTGCACAATAGTTTAATTAGGGGTGAAAAAGATGGT

>NL03.C02.05_A_52

GTTAGAAGGTTAAGTTCTTTTATCTTTCCACCACCAGAGAC

>NL03.C02.05_A_53

TGCAACCCATGCTCTTCTGCCATGTAAGTTGTTCCTATACT

>NL03.C02.05_A_54

ATTAAAGATGTAGACAAAGCTATAGATTTTTATAAACAATACTCT

>NL03.C02.05_A_55

GAAAAATCTCCACGGGTTCACCGTCAACTACAGTAAGTGACC

>NL03.C02.05_A_56

GGATAGACATGTTTGTATTCTTCACAAAACCACTTAAGTAAG

>NL03.C02.05_A_57

CTTTTCTTTTGGCTTCAGGGTCGTTACAGTCTGCTTTGTA

>NL03.C02.05_A_58

TCTCATATGGTATAATCGTAAAGCAAGCAAGATCTGCCTT

>NL03.C02.05_A_59

TTATTATTATTTACGATTGCTAATGAGTAAAGTGGAATATTG

>NL03.C02.05_A_60

AATTTCCCGAGTTTACCAACTTCCTGGCATTTATCAAA

>NL03.C02.05_A_61

AATTCATATATGTAGAAATAACGCGATGACGGTTCATTATAA

>NL03.C02.05_A_62

TTAATAAGGATAGTAATAATGAAATGGAAGAAAAAATTAG

>NL03.C02.05_A_63

ACAAGAAAATTATGTTCATGTCACCTTGGAAAAATATCTGA

>NL03.C02.05_A_64

AAGTTATACTCTGGGCGGTATCTATACAACTCTATTGCTT

>NL03.C02.05_A_65

ACTTCATATCCTGGCACTAGTCCGTATTTTCAATTGAA

>NL03.C02.05_A_66

AGCTCAGATAATGGCTTTTTCTCTCTATCGGTAAACTTCA

>NL03.C02.05_A_67

GGTGAGGAACTAGTATTCAAACCAATTGATGCACCGAAAAGA

>NL03.C02.05_A_68

GTGATTAACAGTCATCGTTGGTACCCAAACATAATAATCCTAA

>NL03.C02.05_A_69

TCAGAGACACTAAAGATGTTAGTAGGAGAGCAGATA

>NL03.C02.05_A_70

ATATTGTACGTTAAAACAGTGCTAAACTATGATGAATCTA

>NL03.C02.05_A_71

CGAAGTTATTCGCAAAGATATGCAAAATGGTCAGCTAAATT

>NL03.C02.05_A_72

TATGATTTACATGAAGGATTTTATATACCTTGCAAAACTGAT

>NL03.C02.05_A_73

TCTTCTGAACTGCCTCTTTATTCCATTAAACTCACTAATCTTTTC

>NL03.C02.05_A_74

TCAATTTTCCATTTTTCAGGTTCCATAATTCCATTTTTGG

>NL03.C02.05_A_75

AATTTATATAGCAGGATTTATTTCAACTGCAAATCAACAAA

>NL03.C02.05_A_76

TTTAGACCATCTCCAACCCCATCTTCTCCATCACGTTTTTG

>NL03.C02.05_A_77

ATTGGAAATTCGGAATGGACAATAAATGCCTTTAAAAATTC

>NL03.C02.05_A_78

ATAACTTTAGAAGTTCCTCATCTGACGCTTCTATCTCATAT

>NL03.C02.05_A_79

AATGGTTTGGTGCGTAATCTGTCAACCAGTTGTAAGTTTCT

>NL03.C02.05_A_80

TCCCTTTTCACGTTTTCTATTTTTTTCTATCACCCGCAAT

>NL03.C02.05_A_81

ACATCTTTAGGAATTACATTTCCCATTATCGTATAACTCCTTTC

>NL03.C02.05_A_82

GCAATAATAGATAAATTAGCTGAAATTCTTCCTGAGAT

>NL03.C02.05_A_83

TTTACTTCAGCTAGCTGAATAAAAAAATTTAAATTAA

>NL03.C02.05_A_84

TCTTCAAGTTAATGTTAAAGAGTATCACAAATGTTGAGTAC

>NL03.C02.05_A_85

TAAATACATTGAATAATTTTTCGGCCTCATCTTTCTCAAA

>NL03.C02.05_A_86

ACAAAAAGGAGAAAAAACAGTGTACGTCACGGATTTATTA

>NL03.C02.05_A_87

TTTAATAGTATATGTTTGTTTCCACAACTTAGTCTTCTT

>NL03.C02.05_A_88

AGGTATGCCCCATGGGGGCGGGGCAGGGAAAAACTCACGT

>NL03.C02.05_A_89

TTAATGCTACCACCAACACATGAAACTTCACCACCACCG

>NL03.C02.05_A_90

TCTGATGCTTTTCCGCCCTGGTTGGCTAGGTGTTCCACTAT

>NL03.C02.05_A_91

GATAGAATCTTTTGCATCTTCCAGATTAACTTTCGCTTCGTT

>NL03.C02.05_A_92

AATTTATCACCTTTTGCTCTAGTCCAGACACCGTGTGACA

>NL03.C02.05_A_93

TAATTATCATGATGATGATTATAATGACCAGTACTACAATAAA

>NL03.C02.05_A_94

TCCTTAAGCTTCACTACGACTCTACTGTCTAGGTTCTGT

>NL03.C02.05_A_95

ACATCTGATGGAAATCGCGGATTACTCAGCTTTGTAACA

>NL03.C02.05_A_96

TCTTTTATTGACTGTTCTATTAATTGACTGATTTGTTCA

>NL03.C02.05_A_97

GTTATTATATTTGTCACAGCATCAAGAACAGTTGGATTAG

>NL03.C02.05_A_98

ATTTGCACAACTTTCATCTTTTTATCCCCAATTTACATTA

>NL03.C02.05_A_99

TTTCACACAATACAACCGAATATGGCTAGACGCAGCAGACT

>NL03.C02.05_A_100

TATCATAAACCCAATCGAAGCAGGAATAGGTCACTTAGAA

>NL03.C02.05_A_101

ATGTATTGGGGTCTTCCCATGCGACCCCAAAGGGGCTATA

>NL03.C02.05_A_102

TGCGGGAAATCCTGTGAGTAGTATTGTTTCTCTTAAATGA

>NL03.C02.05_A_103

GAGTATAACATAGGTTTTAAACATTTTGTTGTTGACTATG

>NL03.C02.05_A_104

ATCCCAGCACTGTTGCAATTAGCGGGACAGGTGGAA

>NL03.C02.05_A_105

AAGTTATTTCTAAAAAGACACTTCATGATAATACCAAAGAACC

>NL03.C02.05_A_106

TTAATGTAAGATTTTAAAAAATGACAAATATAAATCATTC

>NL03.C02.05_A_107

CTTTTCCAGATAGGACCGTTTGCCCTATCCTAAAACTAGTAG

>NL03.C02.05_A_108

CCATCACTGGTGGGTAGAATACTAAGTGGTATAGAGCAT

>NL03.C02.05_A_109

GCGATAATATTGGGTATCATAGTTAGGAGGTTCGGTTGAT

>NL03.C02.05_A_110

GACGCGTTCATCACGATGAACATTATATCGCAACCGATTTTT

>NL03.C02.05_A_111

TAAATCGTTGCTTTTAGTAGCAATATCTTGCCCTCGCTGT

>NL03.C02.05_A_112

CTTATAGGGTCTCTAACCTCTCCATTCACTTCCTCTTCTACCTC

>NL03.C02.05_A_113

TTTTCGATATAACCTAAGATAATACCATCGATTAAATTAAC

>NL03.C02.05_A_114

TGGTAAAGCCTGTACCGGCGATATAACACGGCTTACAATC

>NL03.C02.05_A_115

AATGAAATGTCCTTTTTAACAACAGCGGGATCCAGTATAACT

>NL03.C02.05_A_116

TTTATACCTTTACATTTCGCTCCACATTCCAGTTGCCATCAA

>NL03.C02.05_A_117

ATACTATACTTCTTCGGATTATTTTTGGTACCATCATG

>NL03.C02.05_A_118

TTTTCACAACATCAGCCTCTGAGATATCGAAATATTCCGC

>NL03.C02.05_A_119

ACACGTTATATTCTTCTCCAGTTCCCCTCAACTTAGCACCAGG

>NL03.C02.05_A_120

TCAGGAAATTGAGCTAAAAAATCTAAAACTGCTTTTTCTTTT

>NL03.C02.05_A_121

TGTAATTTGAATAAAAAATTATGGTATGGTGAACCAGTTTAT

>NL03.C02.05_A_122

AAACAACTATTTTTCTTTATGATGCCTTCCTCTGGTAATAA

>NL03.C02.05_A_123

TTTCAATTCTATAGTAGATTAGCAGATTCTCTATT

>NL03.C02.05_A_124

ATAGTAGTGATAAATAATGCATATGTATTAAATAATAAAT

>NL03.C02.05_A_125

TTTATTATATATTATGATATTATAGCCAGCGTCCTTCAGT

>NL03.C02.05_A_126

TTAATAAAACTGCTTTTCTTCTAAATTGTAAATGATAATAT

>NL03.C02.05_A_127

GTATTGGCAGTGGTACAATACCGATGATTGCGTCGTA

>NL03.C02.05_A_128

TAAGCTGTAAGAGAAGAGGGTAAAAGACTCTTGAGCAT

>NL03.C02.05_A_129

TCATCTCCAAGCAGTCCTACCATAAGACTACCAAAAAT

>NL03.C02.05_A_130

TTTTTTGTCTTGAGGATTAAAGTTAGCAATAGATTTAAC

>NL03.C02.05_A_131

CTATGATTTAAAATATAGTCTAAGAGTATATATTGGTATCCGT

>NL03.C02.05_A_132

TTTTTGTTCTCTCCATCATTTCTCCTCATTTCACAAATGCA

>NL03.C02.05_A_133

TTGCATTCCAGACGTTAAAAAGCGGGATTGTATAATCGTTAA

>NL03.C02.05_A_134

TTTACACGGGGCTAAGAGAATTATTGGTTTTGAAAAAAG

>NL03.C02.05_A_135

ATTTATCACGTCGTTCTAGATAACAACTTGCTTACTCTTT

>NL03.C02.05_A_136

TGTTTTACGCCGTATCTGTTTAAGTATGGTCTTAGCCAATC

>NL03.C02.05_A_137

GAACATGGAATCGTTGTTATTCCTTTAGAATTCGGA

>NL03.C02.05_A_138

AACAAACCACGCATAAATCTCACATTAGTTGTAAATCCAT

>NL03.C02.05_A_139

ACTGTTACAGCTGTAGTACAAACAGTAAAAGCTGCATTATC

>NL03.C02.05_A_140

CACTCATTTTTATACTGAACCCCCTTAAAGATTCTGAT

>NL03.C02.05_A_141

TTTACTAATGGAGAACAAGCACAAGAAGCCGGGACAGCAATACAA

>NL03.C02.05_A_142

ACTCTGCGGTTTCAGAGCTATTAAGTTATTCGCTTTGAAA

>NL03.C02.05_A_143

TTTCAATTCTATAGTAGATTAGCTTCCACCTGTCCC

>NL03.C02.05_A_144

TATTTAATTGTGCGTGTAAAAGCAGATGATGACACACTGCGA

>NL03.C02.05_A_145

TTGACTCGAGAACGGGCATTCCGAATCCTTCAGTTCC

>NL03.C02.05_A_146

TCAGGACGTATATTATGCTCATCACTTCTCCGAAGGATGCTA

>NL03.C02.05_A_147

TATAGCGCCGATTGTTCCATATTTACCTATTTTAAGTA

>NL03.C02.05_A_148

ATTCTCTCAAAACTATATTCATATCAAATCATTTTCCTTT

>NL03.C02.05_A_149

CTTTTTAATGCATCATCTATGATGTCAGCTATTCTGTCAA

>NL03.C02.05_A_150

CGAGGAAGCCTGACGATAAAATAATTCCTATAAGAACTAA

>NL03.C02.05_A_151

TCCTTTTGTGTCCTTTGATTACTATCTCCTTTCCGTTCAC

>NL03.C02.05_A_152

TTAAACGGCGGTGATTATGCATATATTCGCTCAACATTTTCTCC

>NL03.C02.05_A_153

TTGTTAGATTACAAGTATTGGCAGGAGAGAACAACGCCAG

>NL03.C02.05_A_154

AGTCTGTATGCAATAATATAGCGTCTATCTTTAAAGTTAAA

>NL03.C02.05_A_155

TTAGCTCAACAGTTGGCGAAAGACCCCCAGAAAGTAA

>NL03.C02.05_A_156

ATTTAGTAAGATTAATTGAATTTCTTCATCTGTTAAACCA

>NL03.C02.05_A_157

AGTGTTAAGTAAGGAGTTATGCTATCCAATTCTGCCGA

>NL03.C02.05_A_158

TGATTACGCAAAACTTGTGCAATATCTTCACTCACATTTCT

>NL03.C02.05_A_159

GTAGGTAGCACAATTACAATCAAATATGCGAACGGCTCCA

>NL03.C02.05_A_160

GAATACCCGCCTGTACTTTGCGTAGTTGATGTGGTAGTAGTTGT

>NL03.C02.05_A_161

AAGTTATACTTTTATAAATATATAAATATGTCATATTATAAGT

>NL03.C02.05_A_162

ATTGTAATTGGTGCAATAAACGGCGTTAGAAATTGAGTAA

>NL03.C02.05_A_163

AATAGGTGTAGACAGATCAACACTAAACCGTTACGTTAA

>NL03.C02.05_A_164

CCCGCACGTCACGCTTACGTTATTCCCGCCCAACTTGTTA

>NL03.C02.05_A_165

ATTATAACAATGATGTCATCTGGATTCATCCAGACCACTCA

>NL03.C02.05_A_166

CCAGGTTCCTCCTTAGGACTTTAGGGTCTTTTCTCTGCT

>NL03.C02.05_A_167

AGTTTGCAAATATGTAGGATTTAAATAATTATTTACTAAA

>NL03.C02.05_A_168

TTTACTCTCACCTCATAACCCTTTTTCAATATCCTCTAATGCCT

>NL03.C02.05_A_169

TCCCAAGGCTGGGAGGGTTTTCGTCTCGTCATCAATCAGG

>NL03.C02.05_A_170

TCAAGAAGTTTATAATCTCTTCATTATCGAACACCTTCCTG

>NL03.C02.05_A_171

ATTACTATGATTTTTTTATGGAAAGATTATGGAATTTAG

>NL03.C02.05_A_172

ATTTTTATTTGGGTTTAGTATGAGGAAGTTACGTGCTT

>NL03.C02.05_A_173

TCAAATTCCCAACCACAAAAAACAATAATACTAATCACGT

>NL03.C02.05_A_174

TATTGGATTTAATGGAGCATTTAAATTCATATTTCCACACCA

>NL03.C02.05_A_175

ATATTATTGGCATGGCAAAATAATTATGGATTCTCTACTT

>NL03.C02.05_A_176

GTTATTCCATATTCCAACAAAACTGATGCTACTTGTTCATT

>NL03.C02.05_A_177

TAACATTCTCTATTTCTAATGCCAATCCTAACGCCACTTA

>NL03.C02.05_A_178

AATGAGTTGGCATACAATCCAGAAATGGATACCTATCTT

>NL03.C02.05_A_179

TTAAGTTCATAGAAGAATCTTAGAACCTCTACTTCATCTT

>NL03.C02.05_A_180

CATTTTAGTACACCGAATTCATATATGTAGAAATAACG

>NL03.C02.05_A_181

AGGTGGATTCTCAAAAGGGTTCTCGTTATCATATGAAAA

>NL03.C02.05_A_182

TGAGCATCAGCCGAACCATCGTAATATTCTACACTTTCAAA

>NL03.C02.05_A_183

TTTAAGCTATTTTATTTTTTTATTTTTTCTTTTTCAGA

>NL03.C02.05_A_184

GTACACCGTAGTAGTTGTAAAACCCGCTCTTCTTATCGCTA

>NL03.C02.05_A_185

GATTATTTCGCTGATACTAACAGATCACTGAGAGAACAGA

>NL03.C02.05_A_186

AACTTTACTTAACGGTTCTAATATCCTAGTGTATAACGAT

>NL03.C02.05_A_187

TTTCCGTTATCTATTTTTCTGAATGTCATTAAAAAGAAGT

>NL03.C02.05_A_188

AACTCATCTATTATTTCCTTCAGGCTTGCCATTTTTTTCGCCT

>NL03.C02.05_A_189

TCTTGTTCTTTTGCTATATCTAAAATTTTTAGCAATTGTTTAG

>NL03.C02.05_A_190

ATAACAAAGCGTAAGTATTCTATTTAAAGGGTCTATTTCA

>NL03.C02.05_A_191

GTTGGTGCAATTGAACCACTCGTGAAAACAGATGATAAA

>NL03.C02.05_A_192

AGTTACAAAGTTCAGTTACAAAACAATATGATTTTGCA

>NL03.C02.05_A_193

CAAAATGCTCCCGGTATTATTTGTTGTCCTTGATAACATG

>NL03.C02.05_A_194

TATAATGTGTTGGTGGTAGCATTAACTTTCAATTCTATAGTAGATTAG

>NL03.C02.05_A_195

TTATGTTCATTTTTTCACCCGCATGTACATTTTTGTTT

>NL03.C02.05_A_196

TTTCAATTCTATAGTAGATTAGCAATAGAGAATCT

>NL03.C02.05_A_197

AATACTATTTGAGCAGTTATAAATACGCCAAATTCATCA

>NL03.C02.05_A_198

TTATTGTTGTTATACAACCATCATTACCTGTAGCATATGC

>NL03.C02.05_A_199

TTTCAATTCTATAGTAGATTAGCAATATTTTAATTTGTCA

>NL03.C02.05_A_200

TTTCAAAAGAGGAGATAATAGATATGTGTAAAGAATTAGAA

>NL03.C02.05_A_201

TTCCCGATTTAGAGAAACTAGCAGAAATTACGGATGCT

>NL03.C02.05_A_202

GTCACTTAATAATAGTTGGAAGTTAACCGTATCCCCATAAT

>NL03.C02.05_A_203

CTTTCCAATTCTGAATTTCGTCAAAAATTAGTCCGTTAG

>NL03.C02.05_A_204

TTTAATAAATGTAAAGCCTAAGCCACAGCCAACTACTTCCC

>NL03.C02.05_A_205

TATAGATTGAGGGCTGAACTCATTAGGTGGGAACGT

>NL03.C02.05_A_206

CATAAGATCCGCTATCTCGTTTAATAGTAGTGCTAATTCTA

>NL03.C02.05_A_207

CGAATGAGTTAGTGTTGGATTGGCATCATGTATCCGCCAA

>NL03.C02.05_A_208

ATTTCGGCGTCTCTCCAGCACCTTACTACAATGCTGAACAGTT

>NL03.C02.05_A_209

ATATGGTTCAGCAAAGCTATTACAATATTATCAAAATGGC

>NL03.C02.05_A_210

ACAAAGCGACTATTAAAATTAAGAATGTTCGTCTATCCA

>NL03.C02.05_A_211

ATATCAAATACCATCAACGTCACCATCCACGTTACGTCGT

>NL03.C02.05_A_212

TCATTAATTTCCTTGAATTTACCATTATGATATTTGTAA

>NL03.C02.05_A_213

TTCCCTTACCTTTCACTTTCACGTATTTTTACATAAGCTAA

>NL03.C02.05_A_214

ATTATTGCCCCGCTCAATGGGTCTCTTACTTCCTCCTTCTC

>NL03.C02.05_A_215

AGTATGAGCTAGAAGTGGATCTTACAACTACTGTTTCTTT

>NL03.C02.05_A_216

ACCCCATGTCCTCATATGGTCGTTTAGCATCTCTTCGAT

>NL03.C02.05_A_217

TATCTCCACACATATATAACGCTATACCATTTATTTAGC

>NL03.C02.05_A_218

CTAAATTATAACACATTTCCTATCTTCTCTTCTTTTTTGTTTC

>NL03.C02.05_A_219

ATTGCATACTTTATTGCATCAGTTGCATCTTTTTCATCA

>NL03.C02.05_A_220

TAAGCCGATTTAATACTGATATTTTCTTCTCTTAGTTTTG

>NL03.C02.05_A_221

ATTGTTTTTATGTTGAAACATATTAATGGTCAAGTTACTAAAAT

>NL03.C02.05_A_222

CGTCTGGTTGGACACGTAAATGTAAGAACCCTGTGCTACTTCAA

>NL03.C02.05_A_223

AAAGAAAGGTGCAACACATCGACCGCAAACGCATAAATAA

>NL03.C02.05_A_224

GTCATTATCAGGTAAGTTGGTAATGGTTTTTTCCACAGTCTG

>NL03.C02.05_A_225

TTTCTCATACCTGGAGACAGAAGCCCAGGTCTTTTTTCTAA

>NL03.C02.05_A_226

ATTTATATCGCTGGATTTATAGCTACTGCAAATCAGCAA

>NL03.C02.05_A_227

ATGGATACATATTTCTTTGAATTCTCCTTCTGCTCTCATTTACCT

>NL03.C02.05_A_228

TAAATAAATCCCGTAACTCACCTCGCCTAGGAAGACTGT

>NL03.C02.05_A_229

GTCGGTTGGAAGTCATTAAATCTATTTTAATTTAATTCTTTTT

>NL03.C02.05_A_230

GGGTTTTATAACGTAGTCGTTGTACCGTCTGAAAATAACA

>NL03.C02.05_A_231

GTTAGGAAAGGGGGTCAGACAAATAGTTGATGCAAATAAT

>NL03.C02.05_A_232

GTTTGTAAAACAATAACTAAACAAAGAAAATCTATGATTA

>NL03.C02.05_A_233

CAATCTCCCGGAATTCCATCATCTCCAAGCTCTCCCTCCA

>NL03.C02.05_A_234

TCACATTCAGATATAAATATTCAATTAGTTCCGAAAACA

>NL03.C02.05_A_235

TCTGAAACTCTCTTTAGCTTTGCCTTCAAGTCCTTTTTTCTT

>NL03.C02.05_A_236

TATCATAGTTCTAGAGATTCCCAATTTATCGTAAGGAA

>NL03.C02.05_A_237

ATATAGACTATTACCCTGAAACTTGTGCTGATAATATAAAGGGACT

>NL03.C02.05_A_238

AGGATGGCGTAAGAGCCGTTGGAGAGCTGAGAAATCAAGA

>NL03.C02.05_A_239

AAAATTCCACTTTGGCCTAATTGTAGAAAACTAAATGAAT

>NL03.C02.05_A_240

CACGGTGACTTTGCGGAAGAAATGCTATCGTTCTATGCAAAT

>NL03.C02.05_A_241

TACGCTAATCCTACCAATTCAGGAGTCACTATACTTATTAG

>NL03.C02.05_A_242

ATTCTTAGTACCACAGGATTATACAAAAACACAGCACCT

>NL03.C02.05_A_243

ACTTCCTGGCGTTTATCAAAGGTATAGGACCAGCTAACGC

>NL03.C02.05_A_244

TCTTTTTCTACAATTTCAGAAATCATTTGAATTATTGTA

>NL03.C02.05_A_245

AAAAACATAGTAGAAATGTTAATGACCAACAAAAGTGAAT

>NL03.C02.05_A_246

TACTTTCAGCTTTACTATTTGATTTGTCCATGTTTCTAT

>NL03.C02.05_A_247

AAAAATTATATGGGCATCTTTTTGTTCGAATGTCCAGT

>NL03.C02.05_A_248

GTTATCCATCAGCTCATGCCACCGTTTGATGAATTTACATCA

>NL03.C02.05_A_249

GTCAGCTGTCTAATTCTCTCAATTTCTGAAACTAGCTTTT

>NL03.C02.05_A_250

ACTGTCTTAAAGAATGCTTAACGTTAGCGTGGAGCAAGTCC

>NL03.C02.05_A_251

TCACCTTTCCATTGCTTCTTTTCTTCCTTCCCTTCTTTCTC

>NL03.C02.05_A_252

AAGCAATATCTAAACTCAATAAGATACTATCTGCACACAGT

>NL03.C02.05_A_253

ACAATTGAAACAAAAATTAGACAAAGATTTTCCAAATTCCAT

>NL03.C02.05_A_254

ATTACAATTTATATGTTTGTAAGAAATTCGATTCAATATCCT

>NL03.C02.05_A_255

CTACGACCTAACATACATCATCATAGGACCTAAATTCA

>NL03.C02.05_A_256

ATGTAATACTAAACTTTCAATTCTATAGTAGATTAG

>NL03.C02.05_A_257

TATCCATATCGATACTCCGAATAATTTCGGATTTTGTGGAAC

>NL03.C02.05_A_258

CTTTCTTTTAGTTAAACCACTTACAATTCCAAATTTTATGG

>NL03.C02.05_A_259

ACAATATAGGAAGTCGCATTGGGTCTTAACTGGAGAT

>NL03.C02.05_A_260

TTTCAAGCCACGCTCTGGTTATTTGAGTGGACAAGAA

>NL03.C02.06_A_1

CCATGCTATCTTAACGGGAAAAGAGCTATTGCAATCATG

>NL03.C02.06_A_2

CATCTATCTCTTAATTTTTCTTCTTTGTCTGGCTATGTAT

>NL03.C02.06_A_3

CAAAACAATCCGTTTTCTAACTGCTTAATTGCGGAAGCCA

>NL03.C02.06_A_4

TTTTGATAGTATTGTAATAGCTTTGCTGAGCCATATTGATTTA

>NL03.C02.06_A_5

TCGATATTATATTTTATAGAAACAACATAATATCTATCATAT

>NL03.C02.06_A_6

TGTGATAACTAAGCTTAATGTGTAAGGCGTTTTTTGTGC

>NL03.C02.06_A_7

AAAGAGTATTCCCTTCAACATATGCTTTTTTTCAGTTAAACTTT

>NL03.C02.06_A_8

ATGATACCGTTAGTTCCTGGAATTGTGAGGTAGTTTTCAC

>NL03.C02.06_A_9

TCAGATGGTAACACTCTGATGGGTGGGGTTACCCCGCTA

>NL03.C02.06_A_10

AATAAAACAATGGATATAATAGTTCCAAGGGGCATAACAG

>NL03.C02.06_A_11

CAATTTCTGTACCGTTTAAACTTATTCTCATTTATCCCA

>NL03.C02.06_A_12

CGAAGTGACGGTGAACGTTATGACCTGGACGACGATTATG

>NL03.C02.06_A_13

CAAAACATGTATCCCATATACTATATCCAAGTTCTGAAACAGC

>NL03.C02.06_A_14

CAAATAACAAATTTCAAAGAAGGAAAAGTAAAAAACGATG

>NL03.C02.06_A_15

CTGATATCTATTAAAATCTTGTTATTCTTAGTAGAAAGGTCT

>NL03.C02.06_A_16

TTAATTGCTGACGATTTAAATAAACTCATTGACGCAATTGGT

>NL03.C02.06_A_17

AATGCCATCTATCTCACCCGTGCTTGTGCTTCTTCTTGTGA

>NL03.C02.06_A_18

GGCTATGTACTCGTAAACGGTGTGCGAATAAGGAGGAAAAT

>NL03.C02.06_A_19

TCATACGTTATCTGGTCGCATACGCTTTCACATCTTACCA

>NL03.C02.06_A_20

TAACGTATCGTCAAGAACGACTTTAAACTTTCCTAAAACG

>NL03.C02.06_A_21

AATAGGTGTAGACAGATCAACACTAAACCGTTACGTTAA

>NL03.C02.06_A_22

ATTTCTCTTGCTTCTTTCAAATACTCCAAAAGTTTAGGTG

>NL03.C02.06_A_23

TCAACTACAGTAAGCGACCCACCACGACCGGAAGTCATTAAC

>NL03.C02.06_A_24

TAAGGTTTGCGAAGGAGGTGTGCGGTGACGCTTGCAATAA

>NL03.C02.06_A_25

TCTGATTTAGTCGAAAAACTGAAGGCGGGTAAACTGGAGACTG

>NL03.C02.06_A_26

GAGAATATAGATTTCATAAAGACGCTACAAAAAATTGCAA

>NL03.C02.06_A_27

GATTTCGTAGCGTTTCCTTACGACCGCGAGACATTGAGGCC

>NL03.C02.06_A_28

GAGATAGAAAAATAGAAAAATCTGTAAAATTAGACTAGTTAA

>NL03.C02.06_A_29

ATCTTTTCACAACTGCCTACAATCACGGTAAAAAGTATA

>NL03.C02.06_A_30

TTTTTCTCTAGATTCTTTATTCTTTTATTTACAATTATTT

>NL03.C02.06_A_31

TTTATTCTCTTCATTCTCGACGAAACGACCGCTAAGAA

>NL03.C02.06_A_32

AATAAATGACACATGGACTACATATCTAGGTGTAAATACAGG

>NL03.C02.06_A_33

TTTAGTATTACATCTTTGTTTGTACATTTTAATCTCCTCA

>NL03.C02.06_A_34

ACTGAATATCTTTCTAAGTAATCCGTTAGTCGCTATTAGTTC

>NL03.C02.06_A_35

TCGCCGCCCCCGTGAATTTTTGCAAACCGAGAAAAGTGAGT

>NL03.C02.06_A_36

AATAATAAAAAAAGGTTTGACTATAATTACACTCTTTATTG

>NL03.C02.06_A_37

TATCATAAACCCAATCGAAGCAGGAATAGGTCACTTAGAA

>NL03.C02.06_A_38

TTCCTCTATTGCTTCCTGTATCAATTTCTTTATTTGCTCA

>NL03.C02.06_A_39

TTGTCATGCGGAATATCTTTTCTAGTGTTTATCTTTAGCT

>NL03.C02.06_A_40

TTAGCTTATACTTTTGGTGAAGGAAATCAACAGCCATTTA

>NL03.C02.06_A_41

AGTATACCTGAAGTTAGATTGGGACAATCGGATATAAA

>NL03.C02.06_A_42

ACAGCATTTCTCGTTGTTGCTTTAGGCATGATCTCTATATT

>NL03.C02.06_A_43

ACTGTCATAAACGAATATAGAAAATACATTGACTATTTT

>NL03.C02.06_A_44

TTATTATTATTTACGATTGCTAATGAGTAAAGTGGAATATTG

>NL03.C02.06_A_45

TTAATAAGGATAGTAATAATGAAATGGAAGAAAAAATTAG

>NL03.C02.06_A_46

ATTCTTTTGTAACTCGTTCCAGACCTTTTCAGCAAACTCTTCA

>NL03.C02.06_A_47

AAGAAGGTACAACAGACAAATACATTGTGTTAACAAATCTAA

>NL03.C02.06_A_48

CGAGGGGAAAGCTAAGGAGGTAGACTTCAAAGAAGAAGAGAA

>NL03.C02.06_A_49

GTCAGCTGTCTAATTCTCTCAATTTCTGAAACTAGCTTTT

>NL03.C02.06_A_50

GTTTCAGTCGGGCTTTACAGAGGAGTACCTAACTATTCAC

>NL03.C02.06_A_51

ACACTATCACAAACACTTCGATCACAGTCCAAAACACA

>NL03.C02.06_A_52

TCGCTTTATACCATTTGTGGACTCTTCAGATAGTCCCTCTGA

>NL03.C02.06_A_53

ACAGCTTGCACAAGTTTTGCCAAAAATAGTTAGTTCTCAGT

>NL03.C02.06_A_54

TGAGCAGTTATCGGTATATTGTTCTTCAGTGCTATAGCAATCT

>NL03.C02.06_A_55

TCTTCTGAACTGCCTCTTTATTCCATTAAACTCACTAATCTTTTC

>NL03.C02.06_A_56

CTTCTTCGGACGAGTTGAAAAGATAATAGTATGTATCTGG

>NL03.C02.06_A_57

TTTTCACACACTCCACCAGTTTCGCTTCTTCCTTTAACCAC

>NL03.C02.06_A_58

TCATCTCCAAGCAGTCCTACCATAAGACTACCAAAAAT

>NL03.C02.06_A_59

TCACCTTTCCATTGCTTCTTTTCTTCCTTCCCTTCTTTCTC

>NL03.C02.06_A_60

TATCTGTAAAGAAGTTCAAAACGCAATTGCTGTATAAGC

>NL03.C02.06_A_61

GCAATAATAGATAAATTAGCTGAAATTCTTCCTGAGAT

>NL03.C02.06_A_62

CGACGGGCGGACATGCGGGTTGGTGTGCCAAACCAACAAACCA

>NL03.C02.06_A_63

AATAATATTTCGCCTCATTTCTCCCTTCTTTTATTGACTGTTC

>NL03.C02.06_A_64

ATCAATGTGTACTCTCCGAATTCTTTTTTTATCTCTTTCT

>NL03.C02.06_A_65

TTTAATAGTATATGTTTGTTTCCACAACTTAGTCTTCTT

>NL03.C02.06_A_66

TAGATAAACTAGGCAAAGCAATAACGAACGCATTAAAAAGTCA

>NL03.C02.06_A_67

GGTGAGGAACTAGTATTCAAACCAATTGATGCACCGAAAAGA

>NL03.C02.06_A_68

TGTAAATAACGCAGAAATAGTAGCAAGTCCTGCTATTGCTCC

>NL03.C02.06_A_69

TACTTTCAGCTTTACTATTTGATTTGTCCATGTTTCTAT

>NL03.C02.06_A_70

GGATGTCGTAAAAGTTGCTGTAAAACTCTAGCTCCTTAGGGGT

>NL03.C02.06_A_71

ACAGAACCTTCCTTAGTTCTTAGAACAATAGAACTGAA

>NL03.C02.06_A_72

GTTTGTAAAACAATAACTAAACAAAGAAAATCTATGATTA

>NL03.C02.06_A_73

CCCTTTAGGGCGGGGGTTCCCCGAGGTCTCAGGCGTTACACCCCT

>NL03.C02.06_A_74

ACATCACGTATCTCTGCTAATAAAATATGGTCTATTGAACA

>NL03.C02.06_A_75

TAAGACTTATTAGCTTTAAACGTTTTAGATTTAAATAGA

>NL03.C02.06_A_76

ACATTGAATTCGAATTCGGCAAATAAAAACTTTACGGCG

>NL03.C02.06_A_77

ATTTTCACGCTGTTAAGTGTCGGGACGGCCAAGCCGA

>NL03.C02.06_A_78

TTTTTCTCTAAATTATATAGCCTTTTATTCATAACTG

>NL03.C02.06_A_79

TGCGGGAAATCCTGTGAGTAGTATTGTTTCTCTTAAATGA

>NL03.C02.06_A_80

CGATTTAGGCAATGTCAGCAAGATAATCAATGGATATGG

>NL03.C02.06_A_81

TTTCTGTCCATGCGTTTTATCGTAATATTCTTCAACTTCTG

>NL03.C02.06_A_82

GTTATTATATTAGTAACTGCGTCTAATACTGTAGGATTTGCA

>NL03.C02.06_A_83

CTATGATTTAAAATATAGTCTAAGAGTATATATTGGTATCCGT

>NL03.C02.06_A_84

TTTTTACTCTTATTATCCTTATCACCACGAGAAGGAAAAG

>NL03.C02.06_A_85

TCTTATGGAGTTGACGCAAAATACATCAAAAAGCATGAAG

>NL03.C02.06_A_86

TCAATAACGCAGGAGATGATGTAAAACTCAAAGATGTTAT

>NL03.C02.06_A_87

TATATCAAGGTTTCGGTCTTAAGGTAGCTAGGGCGCTTAAT

>NL03.C02.06_A_88

CAGTATCTGATGAAAGGTGACACGGGTTTTTGTTATATGT

>NL03.C02.06_A_89

CGAGTCCAAATACAATTTTCAATTCCAGTTGACAGAG

>NL03.C02.06_A_90

TGGTAAAGCCTGTACCGGCGATATAACACGGCTTACAATC

>NL03.C02.06_A_91

ATATACATTTCTCTTTCCCCCTTTATAAATTTTTCTCT

>NL03.C02.06_A_92

AAAACTGTTCTTTCCTTTTTTGTTCTCTCCATCATTTCTCCT

>NL03.C02.06_A_93

ATTTGAGATCTATCAAAACACTACATCTACTTTTACCAGACCATT

>NL03.C02.06_A_94

TTTATACCTTTACATTTCGCTCCACATTCCAGTTGCCATCAA

>NL03.C02.06_A_95

GTACACCGTAGTAGTTGTAAAACCCGCTCTTCTTATCGCTA

>NL03.C02.06_A_96

AACTCATCTATTATTTCCTTCAGGCTTGCCATTTTTTTCGCCT

>NL03.C02.06_A_97

ACACGTTATATTCTTCTCCAGTTCCCCTCAACTTAGCACCAGG

>NL03.C02.06_A_98

ATGACATTTATATCACACATTGAATTAGATAGATGATATTTCT

>NL03.C02.06_A_99

TGTAATTTGAATAAAAAATTATGGTATGGTGAACCAGTTTAT

>NL03.C02.06_A_100

ATTTCTGTTCACGTCGTTAATTTTTGCCCTTACCCTAA

>NL03.C02.06_A_101

TTAATTTGTTCTAACTGAATTGAAAAAACTAACTTAATTT

>NL03.C02.06_A_102

CTTCAGGACTCAAATAGGTAAAGTTAATTTCATTTCCATCC

>NL03.C02.06_A_103

ACACCCCCACTGCTAAATTCAAGAAAAGGTATATGTATAG

>NL03.C02.06_A_104

GATTATATAATATGAATTATTTGCTTAAAAAGTAGTCGAGA

>NL03.C02.06_A_105

TTCTTAAGCGAGGTGACCAACCCATGATTTTCAATTCACA

>NL03.C02.06_A_106

GTCACTTTTCACTTTACCTTCTTTGAAATTTGTTATTTT

>NL03.C02.06_A_107

ATGGACAGATAACATTTCTAAACGCCACGGTAAGTCTGCGTT

>NL03.C02.06_A_108

TCCTCAGCAGGCTGGGGTTGAACGCATTAGCCCTCAAACAGC

>NL03.C02.06_A_109

ATACCCTTATTCGGTAACAAAGCGTAATGGCGTTGATAATG

>NL03.C02.06_A_110

ACCACTATATGCATACCCGCAGTCGCGGAACCGTATGAA

>NL03.C02.06_A_111

CTTTTCCAGATAGGACCGTTTGCCCTATCCTAAAACTAGTAG

>NL03.C02.06_A_112

CATATTCAAACTCTCTAGTTTCATCATTAATATCATTAGTACT

>NL03.C02.06_A_113

TTAAGTGCAAATGCATTAGTGAGTTTAAAGAAATTGAT

>NL03.C02.06_A_114

TTTACACGGGGCTAAGAGAATTATTGGTTTTGAAAAAAG

>NL03.C02.06_A_115

CTTATTATTCGTTGTATTTACGCTTCGTAACAACAGTATACT

>NL03.C02.06_A_116

ATTTATCACGTCGTTCTAGATAACAACTTGCTTACTCTTT

>NL03.C02.06_A_117

GTACGTTTTTCGATGACATTCACCATCCATATACTAATA

>NL03.C02.06_A_118

GTGGTGTTTGCTCGGCATTGTTTATTGCAAAATCGACGTG

>NL03.C02.06_A_119

GCGATAATATTGGGTATCATAGTTAGGAGGTTCGGTTGAT

>NL03.C02.06_A_120

ACAGTTGTGCTTGCAGTCGTTGCTTTGATTTATTCTGTCTG

>NL03.C02.06_A_121

TTTACTAATGGAGAACAAGCACAAGAAGCCGGGACAGCAATACAA

>NL03.C02.06_A_122

TTTGCCATTTTTGGTCAATTAATATTTAAAATTGCTACAA

>NL03.C02.06_A_123

TTTACCCTTTAAGTGGTTATCAAGTATTCCCGCTACGGTCTT

>NL03.C02.06_A_124

GATTAACATTTATTACAAATTTCCAAAGTTTGAATTTGC

>NL03.C02.06_A_125

TCTTTCAGGAGGAGTTTCCATATCCCGATTCTTAGGAGTT

>NL03.C02.06_A_126

AAACCACCAACACCTATAATCATAAAAAGTTTATCTTCCC

>NL03.C02.06_A_127

TAATGAGTTATGATGTATGAACATCTGACTTTCTTGAGATTTGA

>NL03.C02.06_A_128

TGAGCATCAGCCGAACCATCGTAATATTCTACACTTTCAAA

>NL03.C02.06_A_129

CGAGGAAGCCTGACGATAAAATAATTCCTATAAGAACTAA

>NL03.C02.06_A_130

TTCTTTTGCATTACATATAGTCTCTTCATCATTACTGCAT

>NL03.C02.06_A_131

AGCATTTTATACCTAAATAATGAAAGTCGGGTATGATTGG

>NL03.C02.06_A_132

AATAAACGCCAAGAATTTAGTAAATTCAGGGAATTTC

>NL03.C02.06_A_133

GAATATAAAGGCGAAGGAGTAGTAGAAACATCTAATTGTAAACCT

>NL03.C02.06_A_134

ATTTAGTAAGATTAATTGAATTTCTTCATCTGTTAAACCA

>NL03.C02.06_A_135

CTTAGCGGAACCTTACTACCAGGGGCAAAACTTACAACAAGT

>NL03.C02.06_A_136

GGATAGACATGTTTGTATTCTTCACAAAACCACTTAAGTAAG

>NL03.C02.06_A_137

CACAAAAAAGCATTATTCGAAATTGTTATAAACTTTAAT

>NL03.C02.06_A_138

CCCGCACGTCACGCTTACGTTATTCCCGCCCAACTTGTTA

>NL03.C02.06_A_139

AGGATGGCGTAAGAGCCGTTGGAGAGCTGAGAAATCAAGA

>NL03.C02.06_A_140

TAATTTTTCAAAAAATATATCATTTTTCTCTCTGTCGTA

>NL03.C02.06_A_141

AGTTACAAAGTTCAGTTACAAAACAATATGATTTTGCA

>NL03.C02.06_A_142

CTTCTTGACTTTCTACCTTTTGACTTTTACCCATTTT

>NL03.C02.06_A_143

CTTTAGATGAATTGATAAAGGCATTTCAAAATAAGCCTAA

>NL03.C02.06_A_144

TCCCAAGGCTGGGAGGGTTTTCGTCTCGTCATCAATCAGG

>NL03.C02.06_A_145

TCAAGAAGTTTATAATCTCTTCATTATCGAACACCTTCCTG

>NL03.C02.06_A_146

TTCTTTATTCCGAACCTAGAAAGCCCGCTGAAAAGCGG

>NL03.C02.06_A_147

TATTGGATTTAATGGAGCATTTAAATTCATATTTCCACACCA

>NL03.C02.06_A_148

CAATAATGCTGGGTTTAAATGAGTCTCAATATCATCCCAT

>NL03.C02.06_A_149

ATATTATTGGCATGGCAAAATAATTATGGATTCTCTACTT

>NL03.C02.06_A_150

GTTGATATAAACGTCTGTGAAAAAGTAAAAATATATGATAG

>NL03.C02.06_A_151

ATATTGTACGTTAAAACAGTGCTAAACTATGATGAATCTA

>NL03.C02.06_A_152

TTAATGAAGCTGTTTCCTTAACGTGTTCTGGTAATTCCA

>NL03.C02.06_A_153

ACACACGGTGCAGTATCGTATGCTTCTAGAATTAATTT

>NL03.C02.06_A_154

GGATTGACATAGCTAGATGGGAACGATGTTACGTGGATAG

>NL03.C02.06_A_155

ATAATATATTATTAAAAATATGAACATCAAAAAAAGAACGTCCA

>NL03.C02.06_A_156

CGATATGGGACATGCCAGATGACGGTAAACCTAACCCAT

>NL03.C02.06_A_157

GCTTATGTGCATATCCACTATTATTGAAGTTTGCTCTTGCTTGT

>NL03.C02.06_A_158

TTTTCACGTTGATAGAATTTTTTTCTTACTCACAGTACTCTCT

>NL03.C02.06_A_159

ATTTCTTTCGTTTTTTTCGTTTTTTTCGCAAATTTCCCAA

>NL03.C02.06_A_160

ATACTATACTTCTTCGGATTATTTTTGGTACCATCATG

>NL03.C02.06_A_161

CTTTCTTTTAGTTAAACCACTTACAATTCCAAATTTTATGG

>NL03.C02.06_A_162

ATCACTTTTCTCTTTTTTCGCGCCTATACGGTTTCCGA

>NL03.C02.06_A_163

TAATGATGTCAAAATCGGAGTCGTTGTCGTTGTTGTAG

>NL03.C02.06_A_164

GGGGATTAGGTTATGCTCCTGAAGATGCATTGGAAGATGCAGCT

>NL03.C02.06_A_165

CAAAACAGCTGGTACAGCTGGTATAATTCCAGAAGTTTT

>NL03.C02.06_A_166

ACTTTACGCGAGGAATGAGGTGAATGAGGAACAGCTGATG

>NL03.C02.06_A_167

CATACTGCTTCACTAGTCAAGTCGTCGAACGTTGCAATATAGA

>NL03.C02.06_A_168

TCCCTACAACGAGTTTCATTTACTTTACTTTATGCCTCCAT

>NL03.C02.06_A_169

AATATCGTTATGAATTTGTCGTTTGGTAATGGTCGTTTGC

>NL03.C02.06_A_170

CGTGCTTTACATAATACCTCGTTGTCATATCTGCCCAGGTCT

>NL03.C02.06_A_171

ACACCTAGTCCCCTTTATCGTATTATAATAGATCAGCTGAA

>NL03.C02.06_A_172

TTTAGACCATCTCCAACCCCATCTTCTCCATCACGTTTTTG

>NL03.C02.06_A_173

CCGTCACCTCCACCACCTGTATTCTCTTCAACACAGAG

>NL03.C02.06_A_174

GTCATTATCAGGTAAGTTGGTAATGGTTTTTTCCACAGTCTG

>NL03.C02.06_A_175

CTGAATATCCATGAATTCGCTGTATCATTCTTTTTGCCTCC

>NL03.C02.06_A_176

CATAAGATCCGCTATCTCGTTTAATAGTAGTGCTAATTCTA

>NL03.C02.06_A_177

CGAATGAGTTAGTGTTGGATTGGCATCATGTATCCGCCAA

>NL03.C02.06_A_178

AAGGAGTTGTGGAAACATCCAGTTGAAGGCCTTCTATAGT

>NL03.C02.06_A_179

ATTAGTGTTAGAGACGACGACGAATTTGATAGCTTAGTTT

>NL03.C02.06_A_180

TCATTAATTTCCTTGAATTTACCATTATGATATTTGTAA

>NL03.C02.06_A_181

TTCCCTTACCTTTCACTTTCACGTATTTTTACATAAGCTAA

>NL03.C02.06_A_182

CCTTATATATGCATACTTGCTCCTCCTCTTTTGGAAGATT

>NL03.C02.06_A_183

ACCCCATGTCCTCATATGGTCGTTTAGCATCTCTTCGAT

>NL03.C02.06_A_184

AACGGAAATTCTGTAAGATATTCAATGACTATACATAGCCAA

>NL03.C02.06_A_185

AAATTCGAATTAAGGGCTCTGATTATTAGAAAGGACAGTAT

>NL03.C02.06_A_186

CCTTTCGCCACTATTTCGCAGAACTCCCTAACATCGA

>NL03.C02.06_A_187

CGTCTGGTTGGACACGTAAATGTAAGAACCCTGTGCTACTTCAA

>NL03.C02.06_A_188

TGCAACCCATGCTCTTCTGCCATGTAAGTTGTTCCTATACT

>NL03.C02.06_A_189

TTTCTCATACCTGGAGACAGAAGCCCAGGTCTTTTTTCTAA

>NL03.C02.06_A_190

ATTTATATCGCTGGATTTATAGCTACTGCAAATCAGCAA

>NL03.C02.06_A_191

TATAGCTACTGCAGGTATCAATACGTATTACTTGCCTCTG

>NL03.C02.06_A_192

ATGGATACATATTTCTTTGAATTCTCCTTCTGCTCTCATTTACCT

>NL03.C02.06_A_193

TAAATAAATCCCGTAACTCACCTCGCCTAGGAAGACTGT

>NL03.C02.06_A_194

CATCTCCTATATCCGTAACATATAACGAAACGACACTAACTTT

>NL03.C02.06_A_195

TTTTTTCGTAAAACTTGTGCAATATCCTCTGAAACATTTCG

>NL03.C02.06_A_196

AACTCCTTCCGGATGTTCTTTTAAAAAATCCAATATTGC

>NL03.C02.06_A_197

TTAATGTAAGATTTTAAAAAATGACAAATATAAATCATTC

>NL03.C02.06_A_198

TCTGAAACTCTCTTTAGCTTTGCCTTCAAGTCCTTTTTTCTT

>NL03.C02.06_A_199

TTTGAATATGAAGCCCAATATTACGGCGCCGATACTGAAGGG

>NL03.C02.06_A_200

TATCATAGTTCTAGAGATTCCCAATTTATCGTAAGGAA

>NL03.C02.06_A_201

TAATATTAAAGTACACAAAAAATGCATAAAAAATTTGG

>NL03.C02.06_A_202

ATAGCCTTCTTTCTCTTGTAAATTTCTTCAATCACTTCCGC

>NL03.C02.06_A_203

AAAATTCCACTTTGGCCTAATTGTAGAAAACTAAATGAAT

>NL03.C02.06_A_204

TCAGGACGTATATTATGCTCATCACTTCTCCGAAGGATGCTA

>NL03.C02.06_A_205

AAAAACATAGTAGAAATGTTAATGACCAACAAAAGTGAAT

>NL03.C02.06_A_206

TATTATATTTTGATGCAAATGAAAGAAAATGGAAAGA

>NL03.C02.06_A_207

ATAATGAGTATTTGAATGCGTTAGCTGAATTTGATAGAACTGG

>NL03.C02.06_A_208

ACTGTCTTAAAGAATGCTTAACGTTAGCGTGGAGCAAGTCC

>NL03.C02.06_A_209

TATCGATGTGCACTCTCCTCCTCGTTTGAACGTCCCTTC

>NL03.C02.06_A_210

ATTTTTCCCTTTGTACTCTTTAACTACCAAATAATATTT

>NL03.C02.06_A_211

ACATTAAGATTGAAGCGACTCGTTTCACGGAAAAGGCGTT

>NL03.C02.06_A_212

ATTACAATTTATATGTTTGTAAGAAATTCGATTCAATATCCT

>NL03.C02.06_A_213

CTTTCCTTCATAGTAATTGTCAAAATAATCATAGTATA

>NL03.C02.06_A_214

AACCTTCTTTGCCATGCTAGTTGTCTTTCAGACTTGGAGTG

>NL03.C02.06_A_215

ATAATTTCTAATCTTAAGTCGCTGATTACGTCCATCCAGACT

>NL03.C02.07_A_1

TACAGTAAGGAGATTTTCAAGAAGTTAGTCAAATATGTCA

>NL03.C02.07_A_2

AACGTCAATAGCAATGATAGCTACACTCATAACTGCTGTACT

>NL03.C02.07_A_3

GTTAATTTCCGAGGGTAAATATCAACAAGCCTTACAGTTA

>NL03.C02.07_A_4

CATGTACATATCAATATAGTCTTATCATCATCAGGCAAGT

>NL03.C02.07_A_5

GTTATTGATTCACCTTTTGCATAACATGTTGAAAAAACTT

>NL03.C02.07_A_6

AACAAGTATTATGAGGTTAGTAATGTAATAACATTTAGAGATG

>NL03.C02.07_A_7

AAAAAACTTGCGATTTGCCTAAGACTATAGCCCTTACG

>NL03.C02.07_A_8

TCTGGTATGCCCTCATACGATAATATACGATAACAAAATAT

>NL03.C02.07_A_9

CTGGGCAAGAAGAAGATAGAACACACCTGGGATTACAGCGT

>NL03.C02.07_A_10

GCTACTGTAATATATAGCATGACAACTGGGTTACCATATGTAACTTT

>NL03.C02.07_A_11

GTTTAGAAGGAGAAAGATTGACAATTCAAAGAAGAAAGGGACT

>NL03.C02.07_A_12

GAAAATGTAATTTGTTCCTGAACAGAATGGCCTAATCTCAT

>NL03.C02.07_A_13

TTCTAACGTTTTAGGAGTAAGTTCTACAGTAAAATTGAAA

>NL03.C02.07_A_14

AGTATGAGCTAGAAGTGGATCTTACAACTACTGTTTCTTT

>NL03.C02.07_A_15

GTCTTGATATCTGCTAAACCCCTTCTTAGCATCGCGTTTAACA

>NL03.C02.07_A_16

TTTAATAAATGTAAAGCCTAAGCCACAGCCAACTACTTCCC

>NL03.C02.07_A_17

ATGCGGGGAAGCCCATGAGGGTGGGCGTGTTAAACGTGGTTTGT

>NL03.C02.07_A_18

ACAAGAAAATTATGTTCATGTCACCTTGGAAAAATATCTGA

>NL03.C02.07_A_19

ACTTCAACACCTACTCCAACACCATCGCCAGTATTAAA

>NL03.C02.07_A_20

ATCATGTATTGCTATACAATATTGTTTATATTTCTGTA

>NL03.C02.07_A_21

CTTCCATTGCAGAAGCTAAGGCTACAGCGGAAACGTTTT

>NL03.C02.07_A_22

TCTCCAACAAGCACTTCAGTATATTCCGTCGAATTCACAG

>NL03.C02.07_A_23

TATAGCGCCGATTGTTCCATATTTACCTATTTTAAGTA

>NL03.C02.07_A_24

AGAAAAGGAGACGTAGTAGATGGAGCAAGAGACGTTTT

>NL03.C02.07_A_25

ATAGTAGTGATAAATAATGCATATGTATTAAATAATAAAT

>NL03.C02.07_A_26

CCAAATTCGAAGTAGCTACACCTTGAGCTATTATATTCC

>NL03.C02.07_A_27

TTTTTATGACAAGTTAAAGACATGAAGCCAGAGTGTAAAGTTC

>NL03.C02.07_A_28

CTGTTGAATTAATTCGATTAAAGCTTTTAAAATTGAA

>NL03.C02.07_A_29

ACTTTTCATTGAACTTGAACAAAGTCCATGCCATG

>NL03.C02.07_A_30

AAAGAAAGGTGCAACACATCGACCGCAAACGCATAAATAA

>NL03.C02.07_A_31

TTGTTAGATTACAAGTATTGGCAGGAGAGAACAACGCCAG

>NL03.C02.07_A_32

AATTTGTTATATTCTATATGCTTACCTTGGACTTGTTCTAACTG

>NL03.C02.07_A_33

CCTGTGGACTCTTCAGTTAGTCCCTCTGATGCTTTTCCGCCC

>NL03.C02.07_A_34

AGTGTTAAGTAAGGAGTTATGCTATCCAATTCTGCCGA

>NL03.C02.07_A_35

TGATTACGCAAAACTTGTGCAATATCTTCACTCACATTTCT

>NL03.C02.07_A_36

AACTTGAACGATATAATAAACAGCGGTTTTGCGTTAGAT

>NL03.C02.07_A_37

TTTATTTCAGATTTTAAAATTACTGCTAGAGAAATTTTC

>NL03.C02.07_A_38

CTTATAGGGTCTCTAACCTCTCCATTCACTTCCTCTTCTACCTC

>NL03.C02.07_A_39

CTTCAGCATCCTTTTGCAAATCATTAAATGTTCTCCATTC

>NL03.C02.07_A_40

TTTTCTCTTAGTCGTCCTATGTTTACATGCAGTTGTGAT

>NL03.C02.07_A_41

TCACATTCAGATATAAATATTCAATTAGTTCCGAAAACA

>NL03.C02.07_A_42

TTGGTTCAGTACAAATATATAAGCTGGTGAGACGTTTGAG

>NL03.C02.07_A_43

GTCAAAAGAGCCATAACTATACTTCCACTAATTTTCAATA

>NL03.C02.07_A_44

ACAATAATATCGTTTTAAGGCATAATGATAAAGATTATA

>NL03.C02.07_A_45

ATATAGACTATTACCCTGAAACTTGTGCTGATAATATAAAGGGACT

>NL03.C02.07_A_46

TAAACTATGGCACTGGTAACGGTCAATAACTTCACCGTACCGT

>NL03.C02.07_A_47

TGTTCCACTGCTCTTTGTAATAGTCTAGCTGTCTGATTAT

>NL03.C02.07_A_48

TATGATAAAAAAGGCTAGAGAAAAAGTGAAGGAAATGCTA

>NL03.C02.07_A_49

CACGGTGACTTTGCGGAAGAAATGCTATCGTTCTATGCAAAT

>NL03.C02.07_A_50

TTCAGGTACAGGTTTAAGATAGTATGAGAAGAAATAAA

>NL03.C02.07_A_51

GAAAATAATTCCTAGAAATATTATAAGTACTATCGACAAT

>NL03.C02.07_A_52

CAGGCTTATACATTTGGTGAAGGAAATCAACAGCCATTTAA

>NL03.C02.07_A_53

GCACCTTACGGCGGTGTTGAACAATTCGTGAACAACGTGG

>NL03.C02.07_A_54

TATTTTCTCCAGGCATTCAATTTTGCTGAGAGCATGGACAC

>NL03.C02.07_A_55

TATCCATATCGATACTCCGAATAATTTCGGATTTTGTGGAAC

>NL03.C02.07_A_56

ACATGGTTTAGGTCAAGTTTCAGATTGCTGGCTTGCAC

>NL03.C02.07_A_57

ACAGTATTAGACGCAGTTACTAATATAATAACTGGATCTCC

>NL03.C02.07_A_58

GAAGGCGTGGGCGTTCAAATAGATCACGGGGGTACCGC

>NL03.C02.07_A_59

CATCACTTTTGCTAATTCAGCTTTATGAAACTGGAGGCAA

>NL03.C02.07_A_60

CATATCCTTCTTTTTCCAATTCTTCTTGGTCAAGTTCATCA

>NL03.C02.07_A_61

TCTCATATGGTATAATCGTAAAGCAAGCAAGATCTGCCTT

>NL03.C02.07_A_62

GTTCTAATTGAATTGAAAAACTAACATTAATTTCTTGTTG

>NL03.C02.07_A_63

TATCATAAACCCAATCGAAGCAGGAATAGGTCACTTAGAA

>NL03.C02.07_A_64

ACAATATAGGAAGTCGCATTGGGTCTTAACTGGAGAT

>NL03.C02.07_A_65

TCAGCTGGTTTTATATACGGATGTGATGTAACAGATCTATT

>NL03.C02.07_A_66

TTATATTGAAGGTGCGATATGATGGGAAAGACAAAGG

>NL03.C02.07_A_67

TAAGCTGTAAGAGAAGAGGGTAAAAGACTCTTGAGCAT

>NL03.C02.07_A_68

TGAAAATTTTGCGTATATTCCCTAATTGAAAAAATATGGGAAT

>NL03.C02.08_A_1

TTAGCCTCCCCGCTAATAATCGTCGTCTGACCCGTAT

>NL03.C02.08_A_2

AACCTGGTCAGGCAAGCAATCAGGGAAAAAGCTATCATAC

>NL03.C02.08_A_3

ATCAGTTAGATTCAAGTTGTTGCTTGATCTTGATTACGATTA

>NL03.C02.08_A_4

TATTTCTAACTCTGTTGCTCACATGACTGAATTTGTCCATTTA

>NL03.C02.08_A_5

TTTGATACCGTTATGATAGACTGAACCAGTTAGTATGCCA

>NL03.C02.08_A_6

GTTTAGAAGGAGAAAGATTGACAATTCAAAGAAGAAAGGGACT

>NL03.C02.08_A_7

AGGTGAAGAGAAAGCCAGCAGAATGCTTAACCAAAAAGCTCA

>NL03.C02.08_A_8

TTCTTTTTATGTGAAATTTGACATAATTATCAAGCTTTAGA

>NL03.C02.08_A_9

TTAAATTCGCAATTTTAGCATAAATAATTATTTGCTCCTGG

>NL03.C02.08_A_10

ATATACACTGACACTTGTCATAACTAAAAATAATGCCATCTT

>NL03.C02.08_A_11

TTAGGAAATACAATAAATGCAATTTATTTAAGCGGTTAT

>NL03.C02.08_A_12

GCTACTGTGGTATATAGCATGACAACTGGGTTACCATA

>NL03.C02.08_A_13

AACACTCTAGTAGGATTTTACGCGTCACCTACCGCTAGGT

>NL03.C02.08_A_14

TCGCATAGCATTTTTACGTTCTTATTTTTGATATCGAAA

>NL03.C02.08_A_15

ACTATAATATTATAATTTATAATTTTGTCAAACCAAAACTCC

>NL03.C02.08_A_16

TTGTTAGATTACAAGTATTGGCAGGAGAGAACAACGCCAG

>NL03.C02.08_A_17

TGACGGGATACCTTATAGTTATATATGAGGTGGGATAAA

>NL03.C02.08_A_18

GGGTTTTATAACGTAGTCGTTGTACCGTCTGAAAATAACA

>NL03.C02.08_A_19

ATGCTTATAAACGTGAATTGGCAACTATAATACGGGGCG

>NL03.C02.08_A_20

TATCATAAACCCAATCGAAGCAGGAATAGGTCACTTAGAA

>NL03.C02.08_A_21

CTTATAGGGTCTCTAACCTCTCCATTCACTTCCTCTTCTACCTC

>NL03.C02.08_A_22

ATTCTTAGTACCACAGGATTATACAAAAACACAGCACCT

>NL03.C02.08_A_23

AAATAGTTTGCAACTGTTTTAGGTGAAATGGGCGTAAATGGCT

>NL03.C02.08_A_24

TTGAAGGTATAGCGTTATATGTAGATGAAAGAATAACGGAGCC

>NL03.C02.08_A_25

TAACCCAGGTCGTAGACCCGAAAGGAAACGCTGTATACGAA

>NL03.C02.08_A_26

TGAACTTGTAAAAAAAGGCTATTCAATAGCTCAAATTGCAAA

>NL03.C02.08_A_27

TTTTCACAACATCAGCCTCTGAGATATCGAAATATTCCGC

>NL03.C02.08_A_28

ACAACCTATCCGTGCTTATAGGAGTAATACCCAGTGTTTGT

>NL03.C02.08_A_29

ACAATATAGGAAGTCGCATTGGGTCTTAACTGGAGAT

>NL03.C02.08_A_30

TATCCATATCGATACTCCGAATAATTTCGGATTTTGTGGAAC

>NL03.C02.08_A_31

TAAGCTGTAAGAGAAGAGGGTAAAAGACTCTTGAGCAT

>NL03.C02.09_A_1

CCATGCTATCTTAACGGGAAAAGAGCTATTGCAATCATG

>NL03.C02.09_A_2

CAAAACAATCCGTTTTCTAACTGCTTAATTGCGGAAGCCA

>NL03.C02.09_A_3

TTTTGATAGTATTGTAATAGCTTTGCTGAGCCATATTGATTTA

>NL03.C02.09_A_4

TCGATATTATATTTTATAGAAACAACATAATATCTATCATAT

>NL03.C02.09_A_5

AAAGAGTATTCCCTTCAACATATGCTTTTTTTCAGTTAAACTTT

>NL03.C02.09_A_6

ATGATACCGTTAGTTCCTGGAATTGTGAGGTAGTTTTCAC

>NL03.C02.09_A_7

CTTTAGATGAATTGATAAAGGCATTTCAAAATAAGCCTAA

>NL03.C02.09_A_8

AATAAAACAATGGATATAATAGTTCCAAGGGGCATAACAG

>NL03.C02.09_A_9

ATCAATGTGTACTCTCCGAATTCTTTTTTTATCTCTTTCT

>NL03.C02.09_A_10

CGAAGTGACGGTGAACGTTATGACCTGGACGACGATTATG

>NL03.C02.09_A_11

AATAATAAAAAAAGGTTTGACTATAATTACACTCTTTATTG

>NL03.C02.09_A_12

CTGATATCTATTAAAATCTTGTTATTCTTAGTAGAAAGGTCT

>NL03.C02.09_A_13

TTAATTGCTGACGATTTAAATAAACTCATTGACGCAATTGGT

>NL03.C02.09_A_14

AATGCCATCTATCTCACCCGTGCTTGTGCTTCTTCTTGTGA

>NL03.C02.09_A_15

GGCTATGTACTCGTAAACGGTGTGCGAATAAGGAGGAAAAT

>NL03.C02.09_A_16

TCATACGTTATCTGGTCGCATACGCTTTCACATCTTACCA

>NL03.C02.09_A_17

TAACGTATCGTCAAGAACGACTTTAAACTTTCCTAAAACG

>NL03.C02.09_A_18

ATTTCTCTTGCTTCTTTCAAATACTCCAAAAGTTTAGGTG

>NL03.C02.09_A_19

TCAACTACAGTAAGCGACCCACCACGACCGGAAGTCATTAAC

>NL03.C02.09_A_20

TAAGGTTTGCGAAGGAGGTGTGCGGTGACGCTTGCAATAA

>NL03.C02.09_A_21

TCTGATTTAGTCGAAAAACTGAAGGCGGGTAAACTGGAGACTG

>NL03.C02.09_A_22

ATCTTTTCACAACTGCCTACAATCACGGTAAAAAGTATA

>NL03.C02.09_A_23

TTTTTCTCTAGATTCTTTATTCTTTTATTTACAATTATTT

>NL03.C02.09_A_24

TTTATTCTCTTCATTCTCGACGAAACGACCGCTAAGAA

>NL03.C02.09_A_25

ATAGCCTTCTTTCTCTTGTAAATTTCTTCAATCACTTCCGC

>NL03.C02.09_A_26

TTTAGTATTACATCTTTGTTTGTACATTTTAATCTCCTCA

>NL03.C02.09_A_27

GTTGATATAAACGTCTGTGAAAAAGTAAAAATATATGATAG

>NL03.C02.09_A_28

TCGCCGCCCCCGTGAATTTTTGCAAACCGAGAAAAGTGAGT

>NL03.C02.09_A_29

CAAAACATGTATCCCATATACTATATCCAAGTTCTGAAACAGC

>NL03.C02.09_A_30

TTAGCTTATACTTTTGGTGAAGGAAATCAACAGCCATTTA

>NL03.C02.09_A_31

AGTATACCTGAAGTTAGATTGGGACAATCGGATATAAA

>NL03.C02.09_A_32

ACAGCATTTCTCGTTGTTGCTTTAGGCATGATCTCTATATT

>NL03.C02.09_A_33

ACTGTCATAAACGAATATAGAAAATACATTGACTATTTT

>NL03.C02.09_A_34

TTATTATTATTTACGATTGCTAATGAGTAAAGTGGAATATTG

>NL03.C02.09_A_35

ATTCTTTTGTAACTCGTTCCAGACCTTTTCAGCAAACTCTTCA

>NL03.C02.09_A_36

AAGAAGGTACAACAGACAAATACATTGTGTTAACAAATCTAA

>NL03.C02.09_A_37

CGAGGGGAAAGCTAAGGAGGTAGACTTCAAAGAAGAAGAGAA

>NL03.C02.09_A_38

GTCAGCTGTCTAATTCTCTCAATTTCTGAAACTAGCTTTT

>NL03.C02.09_A_39

GTTTCAGTCGGGCTTTACAGAGGAGTACCTAACTATTCAC

>NL03.C02.09_A_40

CATCTCCTATATCCGTAACATATAACGAAACGACACTAACTTT

>NL03.C02.09_A_41

TCGCTTTATACCATTTGTGGACTCTTCAGATAGTCCCTCTGA

>NL03.C02.09_A_42

ACAGCTTGCACAAGTTTTGCCAAAAATAGTTAGTTCTCAGT

>NL03.C02.09_A_43

TGAGCAGTTATCGGTATATTGTTCTTCAGTGCTATAGCAATCT

>NL03.C02.09_A_44

CTTCTTCGGACGAGTTGAAAAGATAATAGTATGTATCTGG

>NL03.C02.09_A_45

TTTTCACACACTCCACCAGTTTCGCTTCTTCCTTTAACCAC

>NL03.C02.09_A_46

TCATCTCCAAGCAGTCCTACCATAAGACTACCAAAAAT

>NL03.C02.09_A_47

TATCTGTAAAGAAGTTCAAAACGCAATTGCTGTATAAGC

>NL03.C02.09_A_48

AACTCCTTCCGGATGTTCTTTTAAAAAATCCAATATTGC

>NL03.C02.09_A_49

CGACGGGCGGACATGCGGGTTGGTGTGCCAAACCAACAAACCA

>NL03.C02.09_A_50

AATAATATTTCGCCTCATTTCTCCCTTCTTTTATTGACTGTTC

>NL03.C02.09_A_51

TTTAATAGTATATGTTTGTTTCCACAACTTAGTCTTCTT

>NL03.C02.09_A_52

TAGATAAACTAGGCAAAGCAATAACGAACGCATTAAAAAGTCA

>NL03.C02.09_A_53

TGTAAATAACGCAGAAATAGTAGCAAGTCCTGCTATTGCTCC

>NL03.C02.09_A_54

ACAGAACCTTCCTTAGTTCTTAGAACAATAGAACTGAA

>NL03.C02.09_A_55

CCCTTTAGGGCGGGGGTTCCCCGAGGTCTCAGGCGTTACACCCCT

>NL03.C02.09_A_56

ACATCACGTATCTCTGCTAATAAAATATGGTCTATTGAACA

>NL03.C02.09_A_57

TAAGACTTATTAGCTTTAAACGTTTTAGATTTAAATAGA

>NL03.C02.09_A_58

ACATTGAATTCGAATTCGGCAAATAAAAACTTTACGGCG

>NL03.C02.09_A_59

ATTTTCACGCTGTTAAGTGTCGGGACGGCCAAGCCGA

>NL03.C02.09_A_60

TTTTTCTCTAAATTATATAGCCTTTTATTCATAACTG

>NL03.C02.09_A_61

TCTTATGGAGTTGACGCAAAATACATCAAAAAGCATGAAG

>NL03.C02.09_A_62

CGATTTAGGCAATGTCAGCAAGATAATCAATGGATATGG

>NL03.C02.09_A_63

TTTCTGTCCATGCGTTTTATCGTAATATTCTTCAACTTCTG

>NL03.C02.09_A_64

ATTTCTGTTCACGTCGTTAATTTTTGCCCTTACCCTAA

>NL03.C02.09_A_65

GCGATAATATTGGGTATCATAGTTAGGAGGTTCGGTTGAT

>NL03.C02.09_A_66

TCAATAACGCAGGAGATGATGTAAAACTCAAAGATGTTAT

>NL03.C02.09_A_67

TATATCAAGGTTTCGGTCTTAAGGTAGCTAGGGCGCTTAAT

>NL03.C02.09_A_68

CAGTATCTGATGAAAGGTGACACGGGTTTTTGTTATATGT

>NL03.C02.09_A_69

CGAGTCCAAATACAATTTTCAATTCCAGTTGACAGAG

>NL03.C02.09_A_70

ATATACATTTCTCTTTCCCCCTTTATAAATTTTTCTCT

>NL03.C02.09_A_71

AAAACTGTTCTTTCCTTTTTTGTTCTCTCCATCATTTCTCCT

>NL03.C02.09_A_72

ATTTGAGATCTATCAAAACACTACATCTACTTTTACCAGACCATT

>NL03.C02.09_A_73

TAATGATGTCAAAATCGGAGTCGTTGTCGTTGTTGTAG

>NL03.C02.09_A_74

ATGACATTTATATCACACATTGAATTAGATAGATGATATTTCT

>NL03.C02.09_A_75

GTTATTATATTAGTAACTGCGTCTAATACTGTAGGATTTGCA

>NL03.C02.09_A_76

TTAATTTGTTCTAACTGAATTGAAAAAACTAACTTAATTT

>NL03.C02.09_A_77

TTTGAATATGAAGCCCAATATTACGGCGCCGATACTGAAGGG

>NL03.C02.09_A_78

ACACCCCCACTGCTAAATTCAAGAAAAGGTATATGTATAG

>NL03.C02.09_A_79

GATTATATAATATGAATTATTTGCTTAAAAAGTAGTCGAGA

>NL03.C02.09_A_80

TTCTTAAGCGAGGTGACCAACCCATGATTTTCAATTCACA

>NL03.C02.09_A_81

GTCACTTTTCACTTTACCTTCTTTGAAATTTGTTATTTT

>NL03.C02.09_A_82

ATGGACAGATAACATTTCTAAACGCCACGGTAAGTCTGCGTT

>NL03.C02.09_A_83

TCCTCAGCAGGCTGGGGTTGAACGCATTAGCCCTCAAACAGC

>NL03.C02.09_A_84

ATACCCTTATTCGGTAACAAAGCGTAATGGCGTTGATAATG

>NL03.C02.09_A_85

ACCACTATATGCATACCCGCAGTCGCGGAACCGTATGAA

>NL03.C02.09_A_86

CATATTCAAACTCTCTAGTTTCATCATTAATATCATTAGTACT

>NL03.C02.09_A_87

TTAAGTGCAAATGCATTAGTGAGTTTAAAGAAATTGAT

>NL03.C02.09_A_88

CTTATTATTCGTTGTATTTACGCTTCGTAACAACAGTATACT

>NL03.C02.09_A_89

GTACGTTTTTCGATGACATTCACCATCCATATACTAATA

>NL03.C02.09_A_90

GTGGTGTTTGCTCGGCATTGTTTATTGCAAAATCGACGTG

>NL03.C02.09_A_91

TTTTTACTCTTATTATCCTTATCACCACGAGAAGGAAAAG

>NL03.C02.09_A_92

ACAGTTGTGCTTGCAGTCGTTGCTTTGATTTATTCTGTCTG

>NL03.C02.09_A_93

TTTGCCATTTTTGGTCAATTAATATTTAAAATTGCTACAA

>NL03.C02.09_A_94

TTTACCCTTTAAGTGGTTATCAAGTATTCCCGCTACGGTCTT

>NL03.C02.09_A_95

GATTAACATTTATTACAAATTTCCAAAGTTTGAATTTGC

>NL03.C02.09_A_96

TCTTTCAGGAGGAGTTTCCATATCCCGATTCTTAGGAGTT

>NL03.C02.09_A_97

AAACCACCAACACCTATAATCATAAAAAGTTTATCTTCCC

>NL03.C02.09_A_98

TAATGAGTTATGATGTATGAACATCTGACTTTCTTGAGATTTGA

>NL03.C02.09_A_99

TTCTTTTGCATTACATATAGTCTCTTCATCATTACTGCAT

>NL03.C02.09_A_100

AGCATTTTATACCTAAATAATGAAAGTCGGGTATGATTGG

>NL03.C02.09_A_101

AATAAACGCCAAGAATTTAGTAAATTCAGGGAATTTC

>NL03.C02.09_A_102

GAATATAAAGGCGAAGGAGTAGTAGAAACATCTAATTGTAAACCT

>NL03.C02.09_A_103

CTTAGCGGAACCTTACTACCAGGGGCAAAACTTACAACAAGT

>NL03.C02.09_A_104

GGATAGACATGTTTGTATTCTTCACAAAACCACTTAAGTAAG

>NL03.C02.09_A_105

CACAAAAAAGCATTATTCGAAATTGTTATAAACTTTAAT

>NL03.C02.09_A_106

TAATTTTTCAAAAAATATATCATTTTTCTCTCTGTCGTA

>NL03.C02.09_A_107

CTTCTTGACTTTCTACCTTTTGACTTTTACCCATTTT

>NL03.C02.09_A_108

TCAGATGGTAACACTCTGATGGGTGGGGTTACCCCGCTA

>NL03.C02.09_A_109

TCCCAAGGCTGGGAGGGTTTTCGTCTCGTCATCAATCAGG

>NL03.C02.09_A_110

TCAAGAAGTTTATAATCTCTTCATTATCGAACACCTTCCTG

>NL03.C02.09_A_111

TTCTTTATTCCGAACCTAGAAAGCCCGCTGAAAAGCGG

>NL03.C02.09_A_112

TATTGGATTTAATGGAGCATTTAAATTCATATTTCCACACCA

>NL03.C02.09_A_113

CAATAATGCTGGGTTTAAATGAGTCTCAATATCATCCCAT

>NL03.C02.09_A_114

ACACTATCACAAACACTTCGATCACAGTCCAAAACACA

>NL03.C02.09_A_115

ATATTGTACGTTAAAACAGTGCTAAACTATGATGAATCTA

>NL03.C02.09_A_116

TTAATGAAGCTGTTTCCTTAACGTGTTCTGGTAATTCCA

>NL03.C02.09_A_117

ACACACGGTGCAGTATCGTATGCTTCTAGAATTAATTT

>NL03.C02.09_A_118

GGATTGACATAGCTAGATGGGAACGATGTTACGTGGATAG

>NL03.C02.09_A_119

ATAATATATTATTAAAAATATGAACATCAAAAAAAGAACGTCCA

>NL03.C02.09_A_120

CAATTTCTGTACCGTTTAAACTTATTCTCATTTATCCCA

>NL03.C02.09_A_121

CGATATGGGACATGCCAGATGACGGTAAACCTAACCCAT

>NL03.C02.09_A_122

GCTTATGTGCATATCCACTATTATTGAAGTTTGCTCTTGCTTGT

>NL03.C02.09_A_123

TTTTCACGTTGATAGAATTTTTTTCTTACTCACAGTACTCTCT

>NL03.C02.09_A_124

ATCACTTTTCTCTTTTTTCGCGCCTATACGGTTTCCGA

>NL03.C02.09_A_125

GGGGATTAGGTTATGCTCCTGAAGATGCATTGGAAGATGCAGCT

>NL03.C02.09_A_126

CAAAACAGCTGGTACAGCTGGTATAATTCCAGAAGTTTT

>NL03.C02.09_A_127

ACTTTACGCGAGGAATGAGGTGAATGAGGAACAGCTGATG

>NL03.C02.09_A_128

CATACTGCTTCACTAGTCAAGTCGTCGAACGTTGCAATATAGA

>NL03.C02.09_A_129

TCCCTACAACGAGTTTCATTTACTTTACTTTATGCCTCCAT

>NL03.C02.09_A_130

AATATCGTTATGAATTTGTCGTTTGGTAATGGTCGTTTGC

>NL03.C02.09_A_131

CGTGCTTTACATAATACCTCGTTGTCATATCTGCCCAGGTCT

>NL03.C02.09_A_132

ACACCTAGTCCCCTTTATCGTATTATAATAGATCAGCTGAA

>NL03.C02.09_A_133

CCGTCACCTCCACCACCTGTATTCTCTTCAACACAGAG

>NL03.C02.09_A_134

CTGAATATCCATGAATTCGCTGTATCATTCTTTTTGCCTCC

>NL03.C02.09_A_135

CATAAGATCCGCTATCTCGTTTAATAGTAGTGCTAATTCTA

>NL03.C02.09_A_136

AAGGAGTTGTGGAAACATCCAGTTGAAGGCCTTCTATAGT

>NL03.C02.09_A_137

ACATTAAGATTGAAGCGACTCGTTTCACGGAAAAGGCGTT

>NL03.C02.09_A_138

TCATTAATTTCCTTGAATTTACCATTATGATATTTGTAA

>NL03.C02.09_A_139

CCTTATATATGCATACTTGCTCCTCCTCTTTTGGAAGATT

>NL03.C02.09_A_140

CCTTTCGCCACTATTTCGCAGAACTCCCTAACATCGA

>NL03.C02.09_A_141

GGATGTCGTAAAAGTTGCTGTAAAACTCTAGCTCCTTAGGGGT

>NL03.C02.09_A_142

ATTTATATCGCTGGATTTATAGCTACTGCAAATCAGCAA

>NL03.C02.09_A_143

TATAGCTACTGCAGGTATCAATACGTATTACTTGCCTCTG

>NL03.C02.09_A_144

ATGGATACATATTTCTTTGAATTCTCCTTCTGCTCTCATTTACCT

>NL03.C02.09_A_145

AACGGAAATTCTGTAAGATATTCAATGACTATACATAGCCAA

>NL03.C02.09_A_146

TTTTTTCGTAAAACTTGTGCAATATCCTCTGAAACATTTCG

>NL03.C02.09_A_147

GCAATAATAGATAAATTAGCTGAAATTCTTCCTGAGAT

>NL03.C02.09_A_148

TTAATGTAAGATTTTAAAAAATGACAAATATAAATCATTC

>NL03.C02.09_A_149

CTTCAGGACTCAAATAGGTAAAGTTAATTTCATTTCCATCC

>NL03.C02.09_A_150

TAATATTAAAGTACACAAAAAATGCATAAAAAATTTGG

>NL03.C02.09_A_151

AAAAACATAGTAGAAATGTTAATGACCAACAAAAGTGAAT

>NL03.C02.09_A_152

TATTATATTTTGATGCAAATGAAAGAAAATGGAAAGA

>NL03.C02.09_A_153

ATAATGAGTATTTGAATGCGTTAGCTGAATTTGATAGAACTGG

>NL03.C02.09_A_154

TATCGATGTGCACTCTCCTCCTCGTTTGAACGTCCCTTC

>NL03.C02.09_A_155

ATAATTTCTAATCTTAAGTCGCTGATTACGTCCATCCAGACT

>NL03.C02.09_A_156

ATTAGTGTTAGAGACGACGACGAATTTGATAGCTTAGTTT

>NL03.C02.09_A_157

CTTTCCTTCATAGTAATTGTCAAAATAATCATAGTATA

>NL03.C02.09_A_158

ATTTTTCCCTTTGTACTCTTTAACTACCAAATAATATTT

>NL03.C02.10_A_1

TACGAAATAAATGACGAACAGTTAATCGAAATTATTAA

>NL03.C02.10_A_2

CAAAACAATCCGTTTTCTAACTGCTTAATTGCGGAAGCCA

>NL03.C02.10_A_3

CTATTATTGAAGTTTGCTCTTGCTTGTGTGCAGTCGACTGATTAT

>NL03.C02.10_A_4

CTTGCTAACTTGTTTTGCAAAAAGTTTAAATACTTATTT

>NL03.C02.10_A_5

AAACCAAATTCCAACAGACCCTGAAACAGACGTTTGCA

>NL03.C02.10_A_6

TTCAGGTACAGGTTTAAGATAGTATGAGAAGAAATAAAG

>NL03.C02.10_A_7

TTAAGTGCAAATGCATTAGTGAGTTTAAAGAAATTGAT

>NL03.C02.10_A_8

AATATCGTTATGAATTTGTCGTTTGGTAATGGTCGTTTGC

>NL03.C02.10_A_9

CTTATTATTCGTTGTATTTACGCTTCGTAACAACAGTATACT

>NL03.C02.10_A_10

ATTATAACAATGATGTCATCTGGATTCATCCAGACCACTCAA

>NL03.C02.10_A_11

TTTAATAAATGTAAAGCCTAAGCCACAGCCAACTACTTCCC

>NL03.C02.10_A_12

TAATTTTCTGATAAATCGGGATTAATACTTTATAATAGCTCTGAT

>NL03.C02.10_A_13

AAGGAGTTGTGGAAACATCCAGTTGAAGGCCTTCTATAGT

>NL03.C02.10_A_14

AAAATCACTATGCCATAGCGAAATCATTATTAGCTTAATTT

>NL03.C02.10_A_15

TATAGCGCCGATTGTTCCATATTTACCTATTTTAAGTA

>NL03.C02.10_A_16

TAATTATGGGGATACGGTTAACTTCCAACTATTATT

>NL03.C02.10_A_17

TCCTTAAGCTTCACTACGACTCTACTGTCTAGGTTCTGT

>NL03.C02.10_A_18

TCTTCTCTTCTTTAGTACTAGAAACTTGACTCATCAATAT

>NL03.C02.10_A_19

TTGTTAGATTACAAGTATTGGCAGGAGAGAACAACGCCAG

>NL03.C02.10_A_20

TAAATAAACCCCGTAGCTCACCTCGCCTAGGAAAATAGT

>NL03.C02.10_A_21

TATAGCTACTGCAGGTATCAATACGTATTACTTGCCTCTG

>NL03.C02.10_A_22

ACTTCCTCTTCGAGTATCAGTCAGTAGCTAAGATCATTGC

>NL03.C02.10_A_23

CTTATAGGGTCTCTAACCTCTCCATTCACTTCCTCTTCTACCTC

>NL03.C02.10_A_24

AACGGAAATTCTGTAAGATATTCAATGACTATACATAGCCAA

>NL03.C02.10_A_25

CCTTATATATGCGTACTTGCTCCTCCTCTTTTGGAAGATT

>NL03.C02.10_A_26

ATAGCCTTCTTTCTCTTGTAAATTTCTTCAATCACTTCCGC

>NL03.C02.10_A_27

TTCGGTAATCGTACTGGTGTCCCTACTGGTATTGACACTGG

>NL03.C02.10_A_28

ACCCCAGCGGAAAATTCACAGTCACGTTCAACGAGGTGGG

>NL03.C02.10_A_29

CTTCTTTTTAACCGTTAGATTTTTAAACCCCGTTCTAGT

>NL03.C02.10_A_30

AATTTTTAGGGCTAACTGAGAAAAAAGTTAGGAAATATT

>NL03.C02.10_A_31

CATATTCAAACTCTCTAGTTTCATCATTAATATCATTAGT

>NL03.C02.10_A_32

CGGGAATTACGCACCGCCCCTCCTCGAACAAATGCTG

>NL03.C02.10_A_33

AAAACTGTTCTTTCCTTTTTTGTTCTCTCCATCATTTCTCCT

>NL03.C02.10_A_34

GTTGATATAAACGTCTGTGAAAAAGTAAAAATATATGATAG

>NL03.C02.10_A_35

TGGCTTAATGCTGTAGTTGATATGATAATGTTGTATTTTCC

>NL03.C02.10_A_36

TCTCATATGGTATAATCGTAAAGCAAGCAAGATCTGCCTT

>NL03.C02.10_A_37

TACATATTATTTCTAGCATCATAGACTAAATTGGCATAAGTCGG

>NL03.C02.10_A_38

ATAGCTTCACACCAGCAATGTTGATGTCAATAGGATCGAC

>NL03.C02.10_A_39

GTTTTTACATAGTTATACTTGGAGGTGAAAAAATGAGTAAAGA

>NL03.C02.10_A_40

TTAATTTGTTCTAACTGAATTGAAAAAACTAACTTAATTT

>NL03.C02.10_A_41

AATGCCATCTATCTCACCCGTGCTTGTGCTTCTTCTTGTGA

>NL03.C02.10_A_42

ATTTTCACGCTGTTAAGTGTCGGGACGGCCAAGCCGA

>NL03.C02.10_A_43

AAGAAGGTACAACAGACAAATACATTGTGTTAACAAATCTAA

>NL03.C02.10_A_44

GTATTGGCAGTGGTACAATACCGATGATTGCGTCGTA

>NL03.C02.10_A_45

TGTCGATGACCCTAGGTTTTGTCGAGTTAAGCATCTTCGG

>NL03.C02.11_A_1

TTGGTCTATTCCTATCCGGTACCATCTCTCATCGCCGT

>NL03.C02.11_A_2

TAAATCCAGCGATATATATCGGATCTAACTCTGTAAGTAC

>NL03.C02.11_A_3

TTTTCTCTTACTATTTCTTCTGAATATGGCACTATTTTT

>NL03.C02.11_A_4

GTCACTTTTCACTTTACCTTCTTTGAAATTTGTTATTTT

>NL03.C02.11_A_5

CCGTTAAACTCCCCAGTTTAACGAAATCGTAGATTGTTGAT

>NL03.C02.11_A_6

TTTTTCTCTAAATTCTGTAATCTCTTATTGACAACTCCAT

>NL03.C02.11_A_7

TACTCTTTTATAAGAAAAATGGTGTAAGCTATAAAGGTAA

>NL03.C02.11_A_8

TCAGATTATACAACATACGATTTCTCTATATTTAGTAATATTA

>NL03.C02.11_A_9

ACACCCCATACTGCTTCACTAGTCAAGTCGTCGAACGT

>NL03.C02.11_A_10

TATGTTGTACAAACCTATACTCTATCTCAAGC

>NL03.C02.11_A_11

TTTTTTATCCCCCTGAGTATACTCCTTGGTCAAACAA

>NL03.C02.11_A_12

TTAATTGCTGACGATTTAAATAAACTCATTGACGCAATTGGT

>NL03.C02.11_A_13

GGCTATGTACTCGTAAACGGTGTGCGAATAAGGAGGAAAAT

>NL03.C02.11_A_14

TTATCATGTGGAATGTCTTTCCTGGTGTTAATTTTTAACT

>NL03.C02.11_A_15

TTTTTTCGGGGTGTAAGGTATGAAGGTACCGGACGTAA

>NL03.C02.11_A_16

TATATGTAAAAAATAATGTTGCTGGAATATATTTATATGAT

>NL03.C02.11_A_17

TACGTGACGTATCAATAATCGCTGGTTTTAATATAGTCATTT

>NL03.C02.11_A_18

ATTTCTCTTGCTTCTTTCAAATACTCCAAAAGTTTAGGTG

>NL03.C02.11_A_19

TTTTCTAAATCGTTTGATATGAGATCTTCTGTTAGGGCTTT

>NL03.C02.11_A_20

TATTTTTATTTTAGGATAAACTGGTTCACCATACCATAATTT

>NL03.C02.11_A_21

CATCTCCTATATCCGTAACATATAACGAAACGACACTAACTTT

>NL03.C02.11_A_22

TCTGATTTAGTCGAAAAACTGAAGGCGGGTAAACTGGAGACTG

>NL03.C02.11_A_23

ATTTAGAAATAATATATCATAGAAAAAGAAAACAAAAAACAG

>NL03.C02.11_A_24

AATTAGTTAATAGAGCCATAAAGGAAGAGAATTTTGAAGCACA

>NL03.C02.11_A_25

CTATCGACCCAAGTTTCATTTCTCCACTTGATTTGCTTT

>NL03.C02.11_A_26

ATTAGTCCCACGTGAACAAATGGACAGGAAGGAATGAGCTT

>NL03.C02.11_A_27

ATTTCGCTTGGAAAAATTGTATCTTCAGGATATTTAACTT

>NL03.C02.11_A_28

AGTATACCTGAAGTTAGATTGGGACAATCGGATATAAA

>NL03.C02.11_A_29

ATATTTTTGTAATGGATTGTGAGGGATGTGAAGCCAAGTTA

>NL03.C02.11_A_30

CTTTTCTTTTGGCTTCAGGGTCGTTACAGTCTGCTTTGTA

>NL03.C02.11_A_31

GCTCAAACTAAAGTAGTAATAGTTCCACAAAATCCAAAACT

>NL03.C02.11_A_32

TTATTATTATTTACGATTGCTAATGAGTAAAGTGGAATATTG

>NL03.C02.11_A_33

TTATCATATTGTGCATATGTAGAAGTTAAAGTTGGAGATAT

>NL03.C02.11_A_34

AGACCTGGGCAGATACGATAACGAGGCACTGTGCAAAATGTTGC

>NL03.C02.11_A_35

ACCAGCACAAGCGGGTGCCTAAAGACATGCTATATGAGAAGA

>NL03.C02.11_A_36

AAGAAGGTACAACAGACAAATACATTGTGTTAACAAATCTAA

>NL03.C02.11_A_37

CGAGGGGAAAGCTAAGGAGGTAGACTTCAAAGAAGAAGAGAA

>NL03.C02.11_A_38

TTTCAAGTAGGAGATTTTGTAGAAGTCCTAGCACAGGGAAG

>NL03.C02.11_A_39

TATTATCGAAGTCAATAAGTCTAATGATATAGTCTATGG

>NL03.C02.11_A_40

ACCCCTAACAATGCGATTTTTCCCGGCGTAGTGCTATATACT

>NL03.C02.11_A_41

ATATAACGCCTATTTTACGCCTATACCACGCTCATTACA

>NL03.C02.11_A_42

ACAGCTTGCACAAGTTTTGCCAAAAATAGTTAGTTCTCAGT

>NL03.C02.11_A_43

TGAGCAGTTATCGGTATATTGTTCTTCAGTGCTATAGCAATCT

>NL03.C02.11_A_44

TTTTCCATAGCATAATACATAACAATCCTCACCATTGCATTT

>NL03.C02.11_A_45

ATTGCTGTTAGTGGTAACAATACAATACTTAGTAACTATGT

>NL03.C02.11_A_46

AATTTATATAGCAGGATTTATTTCAACTGCAAATCAACAAA

>NL03.C02.11_A_47

ACATCTAGAGCAAAACCACTATTAATTATATCATTCAAA

>NL03.C02.11_A_48

AAATAATATAGTTCTTGCTAAATGTGACTGTTGTTCACCCTC

>NL03.C02.11_A_49

TTTTTTCTTAGAACTTGTGCAATATCTTCACTAACGTTCCT

>NL03.C02.11_A_50

ACAATCATCACTAATTCACAGTCACAAACCTCAGCATCAGCATA

>NL03.C02.11_A_51

ACACTATCACAAACACTTCGATCACAGTCCAAAACACA

>NL03.C02.11_A_52

GAGACCTTGATAAGATTATTACTACGCGACGATTTG

>NL03.C02.11_A_53

GTAGGTAGCACAATTACAATCAAATATGCGAACGGCTCCA

>NL03.C02.11_A_54

ACTTTCCTACCCATATTTCTCATGATTATTATGCATTCT

>NL03.C02.11_A_55

TCGCTTATAACTGCAACTAAATAGCAATTGGCACTGAAG

>NL03.C02.11_A_56

ATGTATCTGTCATCGTCTAAGTAATTAACGACAAAGTCCTT

>NL03.C02.11_A_57

TCAATTTCCCCTTTTACTTCAATTTCGCTCTTACAACCTAA

>NL03.C02.11_A_58

TCCTTATATATAATGCGGGCAATTTGTTGATTTTTACATA

>NL03.C02.11_A_59

CCCTTTAGGGCGGGGGTTCCCCGAGGTCTCAGGCGTTACACCCCT

>NL03.C02.11_A_60

CAGGCTTATACATTTGGTGAAGGAAAACAACAGCCGTTTG

>NL03.C02.11_A_61

TACACATACCCATTTTAAAGATGAAAGACACCATGATA

>NL03.C02.11_A_62

CAAAATAGAATTCTCACGCTTTGTTATTGGCTTTATTATGAT

>NL03.C02.11_A_63

AGATATATTGTAAGTACCAGATATTTGTACAATATAAGTCAT

>NL03.C02.11_A_64

CTTGTTCTACTTTTTGACTTTTACCCATTCTGATTTACACC

>NL03.C02.11_A_65

ATTAAAGCTAGATTGGGATGATGTCGAAAAATTAATAG

>NL03.C02.11_A_66

ATATACATTTCTCTTTCCCCCTTTATAAATTTTTCTCT

>NL03.C02.11_A_67

TGACATTATAGGTTTGGGTTTGTGTGGTTTGGGATTCGG

>NL03.C02.11_A_68

TAATGATGTCAAAATCGGAGTCGTTGTCGTTGTTGTAG

>NL03.C02.11_A_69

TTTCTGTCCATGCGTTTTATCGTAATATTCTTCAACTTCTG

>NL03.C02.11_A_70

GATTATATAATATGAATTATTTGCTTAAAAAGTAGTCGAGA

>NL03.C02.11_A_71

CGACCTCAATAAAACTAATGAAGGGCAATTGAAGGAATTAA

>NL03.C02.11_A_72

CGTTTGTTTAGGCAAATCGTCGCGTAGTAATAATTTCAGTAA

>NL03.C02.11_A_73

ATACCCTTATTCGGTAACAAAGCGTAATGGCGTTGATAATG

>NL03.C02.11_A_74

AAATCGTCAAGTAGTTCAAACCCCTCCGCAGGGTTAACCTT

>NL03.C02.11_A_75

GAAGAAAGCGTGTTGAATAACCTGGATACAGGCATAGGCGAAC

>NL03.C02.11_A_76

TTAAGTGCAAATGCATTAGTGAGTTTAAAGAAATTGAT

>NL03.C02.11_A_77

TTCAATGTGACGTATAGGATCACGCCTTACAGTACCGA

>NL03.C02.11_A_78

CTTATTATTCGTTGTATTTACGCTTCGTAACAACAGTATACT

>NL03.C02.11_A_79

GGGGATTAGGTTATGCTCCTGAAGATGCATTGGAAGATGCAGCT

>NL03.C02.11_A_80

GTGGTGTTTGCTCGGCATTGTTTATTGCAAAATCGACGTG

>NL03.C02.11_A_81

AAGAGGTATTTTTATGTGATTATACTTTCTCTAGATTTC

>NL03.C02.11_A_82

AGAATTCTGTTGTTCCTCAAGTATTCTAATGTCTCTTTGA

>NL03.C02.11_A_83

ATAGCAGTTATAGTCAACACATGCAGATAAACTATGTTGG

>NL03.C02.11_A_84

GATTAACATTTATTACAAATTTCCAAAGTTTGAATTTGC

>NL03.C02.11_A_85

AGCACTACTTTGTAAGCTTCAGTTAATGGCACTCCTTCG

>NL03.C02.11_A_86

TTTAAGTAGGGTGTTATTGTCACAGAGCCTGAATTTGTGT

>NL03.C02.11_A_87

AGCGTTAATATATGTGCAACAAGCAATTAATGACGCAGAG

>NL03.C02.11_A_88

TATTGTCTTACAAATTCATGAGCTATTTCAATTATTTGT

>NL03.C02.11_A_89

GATTGCTACCTTCATTTGTGAGTCACCTCAACCACAACTA

>NL03.C02.11_A_90

AATAAACGCCAAGAATTTAGTAAATTCAGGGAATTTC

>NL03.C02.11_A_91

TCATTATGTATGACATAGTATACCTTGACATTATAGGTTTCTTT

>NL03.C02.11_A_92

TAAATTGAAAGGTTGGTTGAGAGGTTATGGCCGAGGTCGG

>NL03.C02.11_A_93

CGATTAAATCCATTATCTTACCCCTTAAACTCCCCAGTTT

>NL03.C02.11_A_94

TAAACTCTTTAATTGATGTTCTGCGTCTTTTGTGCTAAT

>NL03.C02.11_A_95

GACGATACTAACGCGTGTAATAATATACCTGCCAGCTCCG

>NL03.C02.11_A_96

ATTGAGTTAGAAGAAGTTGATTAGTGTAAACTATTTGAGAT

>NL03.C02.11_A_97

GTATAAGTGAGGGTGACGGTGCCTGACTGAGTAGTGAGAAC

>NL03.C02.11_A_98

TATATTTGCTAATTATCTGTATCAAGGTATTAATGATAT

>NL03.C02.11_A_99

ACTTATAAATTATTACCATGTACTTTACCATGTAAATCGT

>NL03.C02.11_A_100

ATTTATGCAGTTCTGCTAAATCAAAAGGATTATCAACATCT

>NL03.C02.11_A_101

TCCCTACAACGAGTTTCATTTACTTTACTTTATGCCTCCAT

>NL03.C02.11_A_102

ATTATAGGCCAGATATTATACAATTCCTTTAAGGTACCT

>NL03.C02.11_A_103

TTTGACTTTTACCCATTTTTACACCCCTTACTTAAAAGATA

>NL03.C02.11_A_104

GTTGATATAAACGTCTGTGAAAAAGTAAAAATATATGATAG

>NL03.C02.11_A_105

AGAAAACTTACAACATTTTCAATATCATTAACTACTGCACT

>NL03.C02.11_A_106

TATGCAAAACCCCTCAGACAAATGTCTCAGAGGTTTAACA

>NL03.C02.11_A_107

ACACACGGTGCAGTATCGTATGCTTCTAGAATTAATTT

>NL03.C02.11_A_108

AATGCCATCTATCTCACCCGTGCTTGTGCTTCTTCTTGTGA

>NL03.C02.11_A_109

TTAATTCTTTCAAACAGATGTTTACAAACCCAACGTCAATAG

>NL03.C02.11_A_110

AATCTATCAGTACAGGAATAGCAAATCCTAACCCGCTTATTTCCA

>NL03.C02.11_A_111

TTTTCTAAATCGTTCTCTGTCAGATCTTCTGTTAGATCTCTC

>NL03.C02.11_A_112

AAAGTGATACAGTAATATTACTAATTGAAGAAAGAATTAAA

>NL03.C02.11_A_113

AAAAGTGATGTAGTGATAGTTTTTGTTGAAGAAAGAATTAAA

>NL03.C02.11_A_114

TGCATGGTCTGGAATTTCATTATTATTAATTCTAAATA

>NL03.C02.11_A_115

AAAGATAGAGTCCAAATTCAATGAAGGATATGATGTAATT

>NL03.C02.11_A_116

AATAATGTTACACAATTAGATTTAGTTTCTATCTTAACAGCT

>NL03.C02.11_A_117

CATGTCATGCTCATATATGATGTTTTCATCAACTTGGATAA

>NL03.C02.11_A_118

GTCACTTAATAATAGTTGGAAGTTAACCGTATCCCCATAAT

>NL03.C02.11_A_119

TTTAGTGTCTTGATCTTTATCTTTTTGGGTTCGAAT

>NL03.C02.11_A_120

AAAAATTGCTTAAAAATAACTGCAAACAGTCCATCAGAAATTCA

>NL03.C02.11_A_121

TCCTCTTGGTTGCCTTCTTCTCCACTTTGCTGTTCTC

>NL03.C02.11_A_122

AGCAGGAATAGAGGCATATATTCATAAAGTCGAAAAACCA

>NL03.C02.11_A_123

TTGTAGTCAAGTATATAATTCGCAATCACCTTTTCAGCAAATGC

>NL03.C02.11_A_124

AATAATATCTCGCCTCATCTTTTCCTTCCTCTATTGCTTCCTG

>NL03.C02.11_A_125

ATACTGTCATCAATATACCATATCCATTCAACAGTATAGG

>NL03.C02.11_A_126

ATAGATTTTACACCCCTAATAAGAGAATTGTTAAAAGGGTT

>NL03.C02.11_A_127

ATCTTTTTATCAGTTTTGCAAGGTATATAAAATCCTTCA

>NL03.C02.11_A_128

TATAGCTACTGCAGGTATCAATACGTATTACTTGCCTCTG

>NL03.C02.11_A_129

ACAAGAAAGTATTGCAGTAATAGAGACAAAAAAACAAACTACAT

>NL03.C02.11_A_130

TTCCTTGAAAAACGCCTCAATCTCCTTTTCCTCTTCCTC

>NL03.C02.11_A_131

TTTTTTCGTAAAACTTGTGCAATATCCTCTGAAACATTTCG

>NL03.C02.11_A_132

GTAGCCGATATAAGGAGTAATTATAACATGAAGGACGAGGA

>NL03.C02.11_A_133

TCTTAATACTGTGGCGTGATAGGCAACCCTAGAACCGTGGG

>NL03.C02.11_A_134

AAAGTCATAGCCGTATTATAGGTTATGGCTAATGGTGCAG

>NL03.C02.11_A_135

ATAATGAGTATTTGAATGCGTTAGCTGAATTTGATAGAACTGG

>NL03.C02.11_A_136

ATATCATTAGCACTCATTAATATTAATTCACATTCGCCGTA

>NL03.C02.11_A_137

TGACAAGAATTCAGGAAATATTAACTGAGCTGACACTGAAAAA

>NL03.C02.11_A_138

AGAGTTGAAGGCGAATATCAAAACATATTAGATTCGACCCCA

>NL03.C02.11_A_139

ATCCTTCTTCTACAACCTTCATACATACACAATCGGAA

>NL03.C02.11_A_140

ATAATTTCTAATCTTAAGTCGCTGATTACGTCCATCCAGACT

>NL03.C02.12_A_1

TTGGTCTATTCCTATCCGGTACCATCTCTCATCGCCGT

>NL03.C02.12_A_2

TAAATCCAGCGATATATATCGGATCTAACTCTGTAAGTAC

>NL03.C02.12_A_3

TTTTCTCTTACTATTTCTTCTGAATATGGCACTATTTTT

>NL03.C02.12_A_4

CGACCTCAATAAAACTAATGAAGGGCAATTGAAGGAATTAA

>NL03.C02.12_A_5

CCGTTAAACTCCCCAGTTTAACGAAATCGTAGATTGTTGAT

>NL03.C02.12_A_6

TTTTTCTCTAAATTCTGTAATCTCTTATTGACAACTCCAT

>NL03.C02.12_A_7

CAAAATAGAATTCTCACGCTTTGTTATTGGCTTTATTATGAT

>NL03.C02.12_A_8

TACTCTTTTATAAGAAAAATGGTGTAAGCTATAAAGGTAA

>NL03.C02.12_A_9

TCAGATTATACAACATACGATTTCTCTATATTTAGTAATATTA

>NL03.C02.12_A_10

ACACCCCATACTGCTTCACTAGTCAAGTCGTCGAACGT

>NL03.C02.12_A_11

TATGTTGTACAAACCTATACTCTATCTCAAGC

>NL03.C02.12_A_12

TTTTTTATCCCCCTGAGTATACTCCTTGGTCAAACAA

>NL03.C02.12_A_13

TTAATTGCTGACGATTTAAATAAACTCATTGACGCAATTGGT

>NL03.C02.12_A_14

GGCTATGTACTCGTAAACGGTGTGCGAATAAGGAGGAAAAT

>NL03.C02.12_A_15

TTATCATGTGGAATGTCTTTCCTGGTGTTAATTTTTAACT

>NL03.C02.12_A_16

TTTTTTCGGGGTGTAAGGTATGAAGGTACCGGACGTAA

>NL03.C02.12_A_17

ATTAGTCCCACGTGAACAAATGGACAGGAAGGAATGAGCTT

>NL03.C02.12_A_18

TATATGTAAAAAATAATGTTGCTGGAATATATTTATATGAT

>NL03.C02.12_A_19

TACGTGACGTATCAATAATCGCTGGTTTTAATATAGTCATTT

>NL03.C02.12_A_20

ATTTCTCTTGCTTCTTTCAAATACTCCAAAAGTTTAGGTG

>NL03.C02.12_A_21

TATTTTTATTTTAGGATAAACTGGTTCACCATACCATAATTT

>NL03.C02.12_A_22

TCTGATTTAGTCGAAAAACTGAAGGCGGGTAAACTGGAGACTG

>NL03.C02.12_A_23

ATTTAGAAATAATATATCATAGAAAAAGAAAACAAAAAACAG

>NL03.C02.12_A_24

AATTAGTTAATAGAGCCATAAAGGAAGAGAATTTTGAAGCACA

>NL03.C02.12_A_25

TATTGTCTTACAAATTCATGAGCTATTTCAATTATTTGT

>NL03.C02.12_A_26

GGGGATTAGGTTATGCTCCTGAAGATGCATTGGAAGATGCAGCT

>NL03.C02.12_A_27

ATTTCGCTTGGAAAAATTGTATCTTCAGGATATTTAACTT

>NL03.C02.12_A_28

TTATCATATTGTGCATATGTAGAAGTTAAAGTTGGAGATAT

>NL03.C02.12_A_29

ATATTTTTGTAATGGATTGTGAGGGATGTGAAGCCAAGTTA

>NL03.C02.12_A_30

CTTTTCTTTTGGCTTCAGGGTCGTTACAGTCTGCTTTGTA

>NL03.C02.12_A_31

TCAATTTCCCCTTTTACTTCAATTTCGCTCTTACAACCTAA

>NL03.C02.12_A_32

TCGCTTATAAATAACTGCAAACAGTCCATCAGAAATTCA

>NL03.C02.12_A_33

ACAATCATCACTAATTCACAGTCACAAACCTCAGCATCAGCATA

>NL03.C02.12_A_34

AGACCTGGGCAGATACGATAACGAGGCACTGTGCAAAATGTTGC

>NL03.C02.12_A_35

ACCAGCACAAGCGGGTGCCTAAAGACATGCTATATGAGAAGA

>NL03.C02.12_A_36

AAGAAGGTACAACAGACAAATACATTGTGTTAACAAATCTAA

>NL03.C02.12_A_37

CGAGGGGAAAGCTAAGGAGGTAGACTTCAAAGAAGAAGAGAA

>NL03.C02.12_A_38

TTTCAAGTAGGAGATTTTGTAGAAGTCCTAGCACAGGGAAG

>NL03.C02.12_A_39

TATTATCGAAGTCAATAAGTCTAATGATATAGTCTATGG

>NL03.C02.12_A_40

CGAGGGGAAAGAGAAATGTATATCTTTCAATTCTATAGTAGATTAG

>NL03.C02.12_A_41

ACACTATCACAAACACTTCGATCACAGTCCAAAACACA

>NL03.C02.12_A_42

ATATAACGCCTATTTTACGCCTATACCACGCTCATTACA

>NL03.C02.12_A_43

ACAGCTTGCACAAGTTTTGCCAAAAATAGTTAGTTCTCAGT

>NL03.C02.12_A_44

TGAGCAGTTATCGGTATATTGTTCTTCAGTGCTATAGCAATCT

>NL03.C02.12_A_45

TTTTCCATAGCATAATACATAACAATCCTCACCATTGCATTT

>NL03.C02.12_A_46

ATTGCTGTTAGTGGTAACAATACAATACTTAGTAACTATGT

>NL03.C02.12_A_47

AATTTATATAGCAGGATTTATTTCAACTGCAAATCAACAAA

>NL03.C02.12_A_48

ACATCTAGAGCAAAACCACTATTAATTATATCATTCAAA

>NL03.C02.12_A_49

TTATTATTATTTACGATTGCTAATGAGTAAAGTGGAATATTG

>NL03.C02.12_A_50

AAATAATATAGTTCTTGCTAAATGTGACTGTTGTTCACCCTC

>NL03.C02.12_A_51

TTTTTTCTTAGAACTTGTGCAATATCTTCACTAACGTTCCT

>NL03.C02.12_A_52

CTATCGACCCAAGTTTCATTTCTCCACTTGATTTGCTTT

>NL03.C02.12_A_53

GAGACCTTGATAAGATTATTACTACGCGACGATTTG

>NL03.C02.12_A_54

GTAGGTAGCACAATTACAATCAAATATGCGAACGGCTCCA

>NL03.C02.12_A_55

ACTTTCCTACCCATATTTCTCATGATTATTATGCATTCT

>NL03.C02.12_A_56

TCGCTTATAACTGCAACTAAATAGCAATTGGCACTGAAG

>NL03.C02.12_A_57

ATGTATCTGTCATCGTCTAAGTAATTAACGACAAAGTCCTT

>NL03.C02.12_A_58

GCTCAAACTAAAGTAGTAATAGTTCCACAAAATCCAAAACT

>NL03.C02.12_A_59

TCCTTATATATAATGCGGGCAATTTGTTGATTTTTACATA

>NL03.C02.12_A_60

CCCTTTAGGGCGGGGGTTCCCCGAGGTCTCAGGCGTTACACCCCT

>NL03.C02.12_A_61

CAGGCTTATACATTTGGTGAAGGAAAACAACAGCCGTTTG

>NL03.C02.12_A_62

TACACATACCCATTTTAAAGATGAAAGACACCATGATA

>NL03.C02.12_A_63

GATTGCTACCTTCATTTGTGAGTCACCTCAACCACAACTA

>NL03.C02.12_A_64

AGATATATTGTAAGTACCAGATATTTGTACAATATAAGTCAT

>NL03.C02.12_A_65

CTTGTTCTACTTTTTGACTTTTACCCATTCTGATTTACACC

>NL03.C02.12_A_66

ATTAAAGCTAGATTGGGATGATGTCGAAAAATTAATAG

>NL03.C02.12_A_67

ATATACATTTCTCTTTCCCCCTTTATAAATTTTTCTCT

>NL03.C02.12_A_68

TGACATTATAGGTTTGGGTTTGTGTGGTTTGGGATTCGG

>NL03.C02.12_A_69

TAATGATGTCAAAATCGGAGTCGTTGTCGTTGTTGTAG

>NL03.C02.12_A_70

TTTCTGTCCATGCGTTTTATCGTAATATTCTTCAACTTCTG

>NL03.C02.12_A_71

AGTATACCTGAAGTTAGATTGGGACAATCGGATATAAA

>NL03.C02.12_A_72

GATTATATAATATGAATTATTTGCTTAAAAAGTAGTCGAGA

>NL03.C02.12_A_73

GTCACTTTTCACTTTACCTTCTTTGAAATTTGTTATTTT

>NL03.C02.12_A_74

CGTTTGTTTAGGCAAATCGTCGCGTAGTAATAATTTCAGTAA

>NL03.C02.12_A_75

ATACCCTTATTCGGTAACAAAGCGTAATGGCGTTGATAATG

>NL03.C02.12_A_76

GTTGATATAAACGTCTGTGAAAAAGTAAAAATATATGATAG

>NL03.C02.12_A_77

AAATCGTCAAGTAGTTCAAACCCCTCCGCAGGGTTAACCTT

>NL03.C02.12_A_78

GAAGAAAGCGTGTTGAATAACCTGGATACAGGCATAGGCGAAC

>NL03.C02.12_A_79

TTAAGTGCAAATGCATTAGTGAGTTTAAAGAAATTGAT

>NL03.C02.12_A_80

TTCAATGTGACGTATAGGATCACGCCTTACAGTACCGA

>NL03.C02.12_A_81

CTTATTATTCGTTGTATTTACGCTTCGTAACAACAGTATACT

>NL03.C02.12_A_82

GTGGTGTTTGCTCGGCATTGTTTATTGCAAAATCGACGTG

>NL03.C02.12_A_83

AAGAGGTATTTTTATGTGATTATACTTTCTCTAGATTTC

>NL03.C02.12_A_84

AGAATTCTGTTGTTCCTCAAGTATTCTAATGTCTCTTTGA

>NL03.C02.12_A_85

ATAGCAGTTATAGTCAACACATGCAGATAAACTATGTTGG

>NL03.C02.12_A_86

GATTAACATTTATTACAAATTTCCAAAGTTTGAATTTGC

>NL03.C02.12_A_87

AGCACTACTTTGTAAGCTTCAGTTAATGGCACTCCTTCG

>NL03.C02.12_A_88

TTTAAGTAGGGTGTTATTGTCACAGAGCCTGAATTTGTGT

>NL03.C02.12_A_89

AGCGTTAATATATGTGCAACAAGCAATTAATGACGCAGAG

>NL03.C02.12_A_90

ACCCCTAACAATGCGATTTTTCCCGGCGTAGTGCTATATACT

>NL03.C02.12_A_91

AATAAACGCCAAGAATTTAGTAAATTCAGGGAATTTC

>NL03.C02.12_A_92

TCATTATGTATGACATAGTATACCTTGACATTATAGGTTTCTTT

>NL03.C02.12_A_93

TAAATTGAAAGGTTGGTTGAGAGGTTATGGCCGAGGTCGG

>NL03.C02.12_A_94

CGATTAAATCCATTATCTTACCCCTTAAACTCCCCAGTTT

>NL03.C02.12_A_95

TAAACTCTTTAATTGATGTTCTGCGTCTTTTGTGCTAAT

>NL03.C02.12_A_96

GACGATACTAACGCGTGTAATAATATACCTGCCAGCTCCG

>NL03.C02.12_A_97

TCGCTTATAACTGCAACAGTCCATCAGAAATTCA

>NL03.C02.12_A_98

ATTGAGTTAGAAGAAGTTGATTAGTGTAAACTATTTGAGAT

>NL03.C02.12_A_99

GTATAAGTGAGGGTGACGGTGCCTGACTGAGTAGTGAGAAC

>NL03.C02.12_A_100

TATATTTGCTAATTATCTGTATCAAGGTATTAATGATAT

>NL03.C02.12_A_101

ACTTATAAATTATTACCATGTACTTTACCATGTAAATCGT

>NL03.C02.12_A_102

ATTTATGCAGTTCTGCTAAATCAAAAGGATTATCAACATCT

>NL03.C02.12_A_103

TGCATGGTCTGGAATTTCATTATTATTAATTCTAAATA

>NL03.C02.12_A_104

ATTATAGGCCAGATATTATACAATTCCTTTAAGGTACCT

>NL03.C02.12_A_105

TTTGACTTTTACCCATTTTTACACCCCTTACTTAAAAGATA

>NL03.C02.12_A_106

CATCTCCTATATCCGTAACATATAACGAAACGACACTAACTTT

>NL03.C02.12_A_107

AGAAAACTTACAACATTTTCAATATCATTAACTACTGCACT

>NL03.C02.12_A_108

TATGCAAAACCCCTCAGACAAATGTCTCAGAGGTTTAACA

>NL03.C02.12_A_109

ACACACGGTGCAGTATCGTATGCTTCTAGAATTAATTT

>NL03.C02.12_A_110

AATGCCATCTATCTCACCCGTGCTTGTGCTTCTTCTTGTGA

>NL03.C02.12_A_111

TTAATTCTTTCAAACAGATGTTTACAAACCCAACGTCAATAG

>NL03.C02.12_A_112

AATCTATCAGTACAGGAATAGCAAATCCTAACCCGCTTATTTCCA

>NL03.C02.12_A_113

TTTTCTAAATCGTTCTCTGTCAGATCTTCTGTTAGATCTCTC

>NL03.C02.12_A_114

AAAGTGATACAGTAATATTACTAATTGAAGAAAGAATTAAA

>NL03.C02.12_A_115

AAAAGTGATGTAGTGATAGTTTTTGTTGAAGAAAGAATTAAA

>NL03.C02.12_A_116

TCCCTACAACGAGTTTCATTTACTTTACTTTATGCCTCCAT

>NL03.C02.12_A_117

AAAGATAGAGTCCAAATTCAATGAAGGATATGATGTAATT

>NL03.C02.12_A_118

AATAATGTTACACAATTAGATTTAGTTTCTATCTTAACAGCT

>NL03.C02.12_A_119

CATGTCATGCTCATATATGATGTTTTCATCAACTTGGATAA

>NL03.C02.12_A_120

GTCACTTAATAATAGTTGGAAGTTAACCGTATCCCCATAAT

>NL03.C02.12_A_121

TTTAGTGTCTTGATCTTTATCTTTTTGGGTTCGAAT

>NL03.C02.12_A_122

TCCTCTTGGTTGCCTTCTTCTCCACTTTGCTGTTCTC

>NL03.C02.12_A_123

AGCAGGAATAGAGGCATATATTCATAAAGTCGAAAAACCA

>NL03.C02.12_A_124

TTGTAGTCAAGTATATAATTCGCAATCACCTTTTCAGCAAATGC

>NL03.C02.12_A_125

AATAATATCTCGCCTCATCTTTTCCTTCCTCTATTGCTTCCTG

>NL03.C02.12_A_126

ATACTGTCATCAATATACCATATCCATTCAACAGTATAGG

>NL03.C02.12_A_127

ATAGATTTTACACCCCTAATAAGAGAATTGTTAAAAGGGTT

>NL03.C02.12_A_128

ATCTTTTTATCAGTTTTGCAAGGTATATAAAATCCTTCA

>NL03.C02.12_A_129

TATAGCTACTGCAGGTATCAATACGTATTACTTGCCTCTG

>NL03.C02.12_A_130

ACAAGAAAGTATTGCAGTAATAGAGACAAAAAAACAAACTACAT

>NL03.C02.12_A_131

TTCCTTGAAAAACGCCTCAATCTCCTTTTCCTCTTCCTC

>NL03.C02.12_A_132

TTTTTTCGTAAAACTTGTGCAATATCCTCTGAAACATTTCG

>NL03.C02.12_A_133

GTAGCCGATATAAGGAGTAATTATAACATGAAGGACGAGGA

>NL03.C02.12_A_134

AAAGTCATAGCCGTATTATAGGTTATGGCTAATGGTGCAG

>NL03.C02.12_A_135

ATAATGAGTATTTGAATGCGTTAGCTGAATTTGATAGAACTGG

>NL03.C02.12_A_136

ATATCATTAGCACTCATTAATATTAATTCACATTCGCCGTA

>NL03.C02.12_A_137

TGACAAGAATTCAGGAAATATTAACTGAGCTGACACTGAAAAA

>NL03.C02.12_A_138

AGAGTTGAAGGCGAATATCAAAACATATTAGATTCGACCCCA

>NL03.C02.12_A_139

ATCCTTCTTCTACAACCTTCATACATACACAATCGGAA

>NL03.C02.12_A_140

ATAATTTCTAATCTTAAGTCGCTGATTACGTCCATCCAGACT

>NL13.C01.01_A_1

TACGAAATAAATGACGAACAGTTAATCGAAATTATTAA

>NL13.C01.01_A_2

CAAAACAATCCGTTTTCTAACTGCTTAATTGCGGAAGCCA

>NL13.C01.01_A_3

CTATTATTGAAGTTTGCTCTTGCTTGTGTGCAGTCGACTGATTAT

>NL13.C01.01_A_4

GTTGATATAAACGTCTGTGAAAAAGTAAAAATATATGATAG

>NL13.C01.01_A_5

CTTGCTAACTTGTTTTGCAAAAAGTTTAAATACTTATTT

>NL13.C01.01_A_6

CACCTTGGTGAGTATCAAGTCAAATACGAATATCACAGACA

>NL13.C01.01_A_7

TTTCTTCTCAGCAAGATAGTCAATAACACTGTTCAAGTACT

>NL13.C01.01_A_8

TCCTTAAGCTTCACTACGACTCTACTGTCTAGGTTCTGT

>NL13.C01.01_A_9

AAGTTATACTTTTATAAATATATAAATATGTCATATTATAAGT

>NL13.C01.01_A_10

ATATGCAGTCGATATTATTACAAGGACTGACCCCACAAA

>NL13.C01.01_A_11

CACGGTGACTTTGCGGAAGAAATGCTATCGTTCTATGCAAAT

>NL13.C01.01_A_12

TTAAGTGCAAATGCATTAGTGAGTTTAAAGAAATTGAT

>NL13.C01.01_A_13

AATATCGTTATGAATTTGTCGTTTGGTAATGGTCGTTTGC

>NL13.C01.01_A_14

TAGTTGTTCCCCTGGCTGCAGCTTAGTCAGAAAGCTGC

>NL13.C01.01_A_15

TCTCATATGGTATAATCGTAAAGCAAGCAAGATCTGCCTT

>NL13.C01.01_A_16

CTTATTATTCGTTGTATTTACGCTTCGTAACAACAGTATACT

>NL13.C01.01_A_17

TTTTCTCGAATTATTTCTTCAGAATATGGCACTATTTTTATCCATTC

>NL13.C01.01_A_18

TTATGATTGCTTTCCAAGTTTGACCATTCCAGAATAGG

>NL13.C01.01_A_19

TAATTATGGGGATACGGTTAACTTCCAACTATTATT

>NL13.C01.01_A_20

TTTAATAAATGTAAAGCCTAAGCCACAGCCAACTACTTCCC

>NL13.C01.01_A_21

ATTACAATTGGTAAATTTTTGCCAGCAATAATAGATAAATT

>NL13.C01.01_A_22

CTAGCATTGGGGGTTTTAGGGGGATACCCCCTAACTAGAC

>NL13.C01.01_A_23

TAATTTTCTGATAAATCGGGATTAATACTTTATAATAGCTCTGAT

>NL13.C01.01_A_24

AAGGAGTTGTGGAAACATCCAGTTGAAGGCCTTCTATAGT

>NL13.C01.01_A_25

AAAATCACTATGCCATAGCGAAATCATTATTAGCTTAATTT

>NL13.C01.01_A_26

ACATAAAACCCTACAAGATTTGCCCATGTTATCTTCCTTTTTTA

>NL13.C01.01_A_27

GGCTATGTACTCGTAAACGGTGTGCGAATAAGGAGGAAAAT

>NL13.C01.01_A_28

GTATTGGCAGTGGTACAATACCGATGATTGCGTCGTA

>NL13.C01.01_A_29

ACTGTGGATCACTTGAGCAAAAATAATCTTGACAGAA

>NL13.C01.01_A_30

TATAGCGCCGATTGTTCCATATTTACCTATTTTAAGTA

>NL13.C01.01_A_31

ATCCTCTCCTTAACAAAGATGTTGTATATTCTACCGATATATCTTA

>NL13.C01.01_A_32

AAACCAAATTCCAACAGACCCTGAAACAGACGTTTGCA

>NL13.C01.01_A_33

AATTTTTAGGGCTAACTGAGAAAAAAGTTAGGAAATATT

>NL13.C01.01_A_34

TTTTTGACCATGAGTTTTGTCATAATATTCCTCAACTTCAC

>NL13.C01.01_A_35

TTCCTGTTTTTCGCCTGCCGTATCTGCCGAGTTACTACCCACAA

>NL13.C01.01_A_36

CCAGGTTCCTCCTTAGGACTTTAGGGTCTTTTCTCTGCT

>NL13.C01.01_A_37

TAAAGAGTTGTCCATTTACAATATTTCCAATAATTGTATGT

>NL13.C01.01_A_38

TTGTTAGATTACAAGTATTGGCAGGAGAGAACAACGCCAG

>NL13.C01.01_A_39

GATAATGAGAAACCCTGGTGATTACAGAAAAGAGGTAGAG

>NL13.C01.01_A_40

TAAATAAACCCCGTAGCTCACCTCGCCTAGGAAAATAGT

>NL13.C01.01_A_41

ACCGTCGCTGAAATACGTTAGTGTCAACTCGCCCTCC

>NL13.C01.01_A_42

CATAAAGTACTACGTCATTGTTATAGAACTTCTTCCTTTCCAG

>NL13.C01.01_A_43

ATTTGAACTGTTTTCATTTTCATATCACCAATATATAGTATGTA

>NL13.C01.01_A_44

CCTTATATATGCGTACTTGCTCCTCCTCTTTTGGAAGATT

>NL13.C01.01_A_45

ATTATAACAATGATGTCATCTGGATTCATCCAGACCACTCAA

>NL13.C01.01_A_46

CTTATAGGGTCTCTAACCTCTCCATTCACTTCCTCTTCTACCTC

>NL13.C01.01_A_47

AACGGAAATTCTGTAAGATATTCAATGACTATACATAGCCAA

>NL13.C01.01_A_48

AAAAGTTGCTAGGGCACTTAATAGACTCGGCGGCGGGCCTGCTC

>NL13.C01.01_A_49

TATAGCTACTGCAGGTATCAATACGTATTACTTGCCTCTG

>NL13.C01.01_A_50

ATAGCCTTCTTTCTCTTGTAAATTTCTTCAATCACTTCCGC

>NL13.C01.01_A_51

AAGAAGGTACTTTCAATTCTATAGTAGATTAG

>NL13.C01.01_A_52

TTCGGTAATCGTACTGGTGTCCCTACTGGTATTGACACTGG

>NL13.C01.01_A_53

TTTACTTCAGCTAGCTGAATAAAAAAATTTAAATTAA

>NL13.C01.01_A_54

AAAGGGAGAGCTGCTTGACCTCCATCTGAGGTACGGCAAAGA

>NL13.C01.01_A_55

GAGAAAGTTGTAAACGAGGACCTAAGTAAGGATACAGTTCCT

>NL13.C01.01_A_56

GCTATCCCCTCTGAACAAGGGTGGCTGTGTCCTAATGAT

>NL13.C01.01_A_57

CTTCTTTTTAACCGTTAGATTTTTAAACCCCGTTCTAGT

>NL13.C01.01_A_58

AGTTGTTGTGCAAATTGTAAATCTGAATCTAAAACACCGTAG

>NL13.C01.01_A_59

GCTTTATGCATGGATTGAAAGTTACAATTCTAATTTATCAAC

>NL13.C01.01_A_60

TTTTTTAGAATTGAAGTTACTGCATTTCCGCCTGTCTCATAC

>NL13.C01.01_A_61

CGGGAATTACGCACCGCCCCTCCTCGAACAAATGCTG

>NL13.C01.01_A_62

ATGGCTGACGACGATGACAACTATAAAGACAAGTTTAAGT

>NL13.C01.01_A_63

AAAACTGTTCTTTCCTTTTTTGTTCTCTCCATCATTTCTCCT

>NL13.C01.01_A_64

TAGATAAAATATTAGAAGTTGTAGATAAATTAGATGATTATAA

>NL13.C01.01_A_65

TTCAGATATCCAATTTCAATAATTCATCCATTCTTCTAT

>NL13.C01.01_A_66

AGAGAAACAGCAGGTACAAAATCAACTCAGCCAAGTACAA

>NL13.C01.01_A_67

TGGCTTAATGCTGTAGTTGATATGATAATGTTGTATTTTCC

>NL13.C01.01_A_68

CTTTCCAATATAAGTTAATATTTCTTGAAAGAGTCCCTTAG

>NL13.C01.01_A_69

TACATATTATTTCTAGCATCATAGACTAAATTGGCATAAGTCGG

>NL13.C01.01_A_70

TTAATTTGTTCTAACTGAATTGAAAAAACTAACTTAATTT

>NL13.C01.01_A_71

GTTTTTACATAGTTATACTTGGAGGTGAAAAAATGAGTAAAGA

>NL13.C01.01_A_72

TCTTCTCTTCTTTAGTACTAGAAACTTGACTCATCAATAT

>NL13.C01.01_A_73

AATGCCATCTATCTCACCCGTGCTTGTGCTTCTTCTTGTGA

>NL13.C01.01_A_74

ATTTTCACGCTGTTAAGTGTCGGGACGGCCAAGCCGA

>NL13.C01.01_A_75

ACCCCAGCGGAAAATTCACAGTCACGTTCAACGAGGTGGG

>NL13.C01.01_A_76

AAGAAGGTACAACAGACAAATACATTGTGTTAACAAATCTAA

>NL13.C01.01_A_77

ATATCATAAAAATCCAATACTTTTGCATGCAATTGCGGG

>NL13.C01.01_A_78

AATCAGGTATAACGACATAATGGTTGAATGCAGGCCTACAAG

>NL13.C01.01_A_79

TGTCGATGACCCTAGGTTTTGTCGAGTTAAGCATCTTCGG

>NL13.C01.01_A_80

GATTATATAATATGAATTATTTGCTTAAAAAGTAGTCGAGA

>NL13.C01.02_A_1

CAGCTTATTCATACATCCGGAAATGGAGATATAAGGTTCCGAT

>NL13.C01.02_A_2

CATCTATCTCTTAATTTTTCTTCTTTGTCTGGCTATGTAT

>NL13.C01.02_A_3

ACGGCGTAAAGTTGTAAATGCCAGGCTGGAAGTACATGTTG

>NL13.C01.02_A_4

TGTGATAACTAAGCTTAATGTGTAAGGCGTTTTTTGTGC

>NL13.C01.02_A_5

CATACTAGAAGTTAATCTCTGGAAAGGCGAAGCCAACA

>NL13.C01.02_A_6

AAACATAATAAAAATGCCTACCAAAAACTCCTTTATTTATTT

>NL13.C01.02_A_7

AAAACTACACCAACCATAGCTGTAACATATGTCACTTTCTA

>NL13.C01.02_A_8

CAAATAACAAATTTCAAAGAAGGAAAAGTAAAAAACGATG

>NL13.C01.02_A_9

TTAGCTCAACAGTTGGCGAAAGACCCCCAGAAAGTAA

>NL13.C01.02_A_10

GTTTAGAAGGAGAAAGATTGACAATTCAAAGAAGAAAGGGACT

>NL13.C01.02_A_11

AACTCTGATGCCGTCAGTGCGTTTATAATTTGTGCTAGTG

>NL13.C01.02_A_12

TTAGCAACATCTTCAAGAAATTGACGCGCAGGAGTCGAATGAAT

>NL13.C01.02_A_13

AATAGGTGTAGACAGATCAACACTAAACCGTTACGTTAA

>NL13.C01.02_A_14

ACTATAATATTATAATTTATAATTTTGTCAAACCAAAACTCC

>NL13.C01.02_A_15

GAGAATATAGATTTCATAAAGACGCTACAAAAAATTGCAA

>NL13.C01.02_A_16

TTTACTCTCACCTCATAACCCTTTTTCAATATCCTCTAATGCCT

>NL13.C01.02_A_17

GATTTCGTAGCGTTTCCTTACGACCGCGAGACATTGAGGCC

>NL13.C01.02_A_18

TATCTCCACACATATATAACGCTATACCATTTATTTAGC

>NL13.C01.02_A_19

AATAAATGACACATGGACTACATATCTAGGTGTAAATACAGG

>NL13.C01.02_A_20

ACCCATTGTTTGTGCACGCCTACTCTCATTCTGCAACTTC

>NL13.C01.02_A_21

AAAAATTATATGGGCATCTTTTTGTTCGAATGTCCAGT

>NL13.C01.02_A_22

ATTGTCACTTACACGCAGTACATTACGCGGTGAGGCAGA

>NL13.C01.02_A_23

ATGCTTATAAACGTGAATTGGCAACTATAATACGGGGCG

>NL13.C01.02_A_24

TATCATAAACCCAATCGAAGCAGGAATAGGTCACTTAGAA

>NL13.C01.02_A_25

TTCCTCTATTGCTTCCTGTATCAATTTCTTTATTTGCTCA

>NL13.C01.02_A_26

TTGTCATGCGGAATATCTTTTCTAGTGTTTATCTTTAGCT

>NL13.C01.02_A_27

GTTTGCACAATAGTTTAATTAGGGGTGAAAAAGATGGT

>NL13.C01.02_A_28

GTTAGAAGGTTAAGTTCTTTTATCTTTCCACCACCAGAGAC

>NL13.C01.02_A_29

AAGTTATACTCTGGGCGGTATCTATACAACTCTATTGCTT

>NL13.C01.02_A_30

TTAATAAGGATAGTAATAATGAAATGGAAGAAAAAATTAG

>NL13.C01.02_A_31

CGAAGTTATTCGCAAAGATATGCAAAATGGTCAGCTAAATT

>NL13.C01.02_A_32

ATGAAACTTACAACATTTTCAATATCATTAACTACTGCACTA

>NL13.C01.02_A_33

TCTTCTGAACTGCCTCTTTATTCCATTAAACTCACTAATCTTTTC

>NL13.C01.02_A_34

TCAATTTTCCATTTTTCAGGTTCCATAATTCCATTTTTGG

>NL13.C01.02_A_35

TTTAGACCATCTCCAACCCCATCTTCTCCATCACGTTTTTG

>NL13.C01.02_A_36

ATTGGAAATTCGGAATGGACAATAAATGCCTTTAAAAATTC

>NL13.C01.02_A_37

ATTACTATGATTTTTTTATGGAAAGATTATGGAATTTAG

>NL13.C01.02_A_38

ATTGCATACTTTATTGCATCAGTTGCATCTTTTTCATCA

>NL13.C01.02_A_39

ACAAAAAGGAGAAAAAACAGTGTACGTCACGGATTTATTA

>NL13.C01.02_A_40

GGTGAGGAACTAGTATTCAAACCAATTGATGCACCGAAAAGA

>NL13.C01.02_A_41

TACTTTCAGCTTTACTATTTGATTTGTCCATGTTTCTAT

>NL13.C01.02_A_42

TGCGGGAAATCCTGTGAGTAGTATTGTTTCTCTTAAATGA

>NL13.C01.02_A_43

CTTTTCCAGATAGGACCGTTTGCCCTATCCTAAAACTAGTAG

>NL13.C01.02_A_44

CTCTTTTTTCTTCTTAATGATCATGTTTATTATCTCTTTAT

>NL13.C01.02_A_45

GACGCGTTCATCACGATGAACATTATATCGCAACCGATTTTT

>NL13.C01.02_A_46

TGGTAAAGCCTGTACCGGCGATATAACACGGCTTACAATC

>NL13.C01.02_A_47

TTTATACCTTTACATTTCGCTCCACATTCCAGTTGCCATCAA

>NL13.C01.02_A_48

GTACACCGTAGTAGTTGTAAAACCCGCTCTTCTTATCGCTA

>NL13.C01.02_A_49

TTTTCACAACATCAGCCTCTGAGATATCGAAATATTCCGC

>NL13.C01.02_A_50

ACACGTTATATTCTTCTCCAGTTCCCCTCAACTTAGCACCAGG

>NL13.C01.02_A_51

TGTAATTTGAATAAAAAATTATGGTATGGTGAACCAGTTTAT

>NL13.C01.02_A_52

TTTATTATATATTATGATATTATAGCCAGCGTCCTTCAGT

>NL13.C01.02_A_53

TAAGCTGTAAGAGAAGAGGGTAAAAGACTCTTGAGCAT

>NL13.C01.02_A_54

CTATGATTTAAAATATAGTCTAAGAGTATATATTGGTATCCGT

>NL13.C01.02_A_55

TTTTTGTTCTCTCCATCATTTCTCCTCATTTCACAAATGCA

>NL13.C01.02_A_56

TTGCATTCCAGACGTTAAAAAGCGGGATTGTATAATCGTTAA

>NL13.C01.02_A_57

TTTACACGGGGCTAAGAGAATTATTGGTTTTGAAAAAAG

>NL13.C01.02_A_58

ATTTATCACGTCGTTCTAGATAACAACTTGCTTACTCTTT

>NL13.C01.02_A_59

TTTACTAATGGAGAACAAGCACAAGAAGCCGGGACAGCAATACAA

>NL13.C01.02_A_60

AAGTTATTTCTAAAAAGACACTTCATGATAATACCAAAGAACC

>NL13.C01.02_A_61

TTGACTCGAGAACGGGCATTCCGAATCCTTCAGTTCC

>NL13.C01.02_A_62

TCAGGACGTATATTATGCTCATCACTTCTCCGAAGGATGCTA

>NL13.C01.02_A_63

CGAGGAAGCCTGACGATAAAATAATTCCTATAAGAACTAA

>NL13.C01.02_A_64

TTAAACGGCGGTGATTATGCATATATTCGCTCAACATTTTCTCC

>NL13.C01.02_A_65

AGTCTGTATGCAATAATATAGCGTCTATCTTTAAAGTTAAA

>NL13.C01.02_A_66

ATTTAGTAAGATTAATTGAATTTCTTCATCTGTTAAACCA

>NL13.C01.02_A_67

CCCGCACGTCACGCTTACGTTATTCCCGCCCAACTTGTTA

>NL13.C01.02_A_68

AGGATGGCGTAAGAGCCGTTGGAGAGCTGAGAAATCAAGA

>NL13.C01.02_A_69

ACACTAAACGCAAAAAACTAACAACAAATAACATGATGAAT

>NL13.C01.02_A_70

ATTTTTATTTGGGTTTAGTATGAGGAAGTTACGTGCTT

>NL13.C01.02_A_71

ATATTATTGGCATGGCAAAATAATTATGGATTCTCTACTT

>NL13.C01.02_A_72

GTTATCCATCAGCTCATGCCACCGTTTGATGAATTTACATCA

>NL13.C01.02_A_73

AGGTGGATTCTCAAAAGGGTTCTCGTTATCATATGAAAA

>NL13.C01.02_A_74

TGAGCATCAGCCGAACCATCGTAATATTCTACACTTTCAAA

>NL13.C01.02_A_75

TTTAAGCTATTTTATTTTTTTATTTTTTCTTTTTCAGA

>NL13.C01.02_A_76

ATTTCTTTCGTTTTTTTCGTTTTTTTCGCAAATTTCCCAA

>NL13.C01.02_A_77

ATACTATACTTCTTCGGATTATTTTTGGTACCATCATG

>NL13.C01.02_A_78

CTTTCTTTTAGTTAAACCACTTACAATTCCAAATTTTATGG

>NL13.C01.02_A_79

TTTCCGTTATCTATTTTTCTGAATGTCATTAAAAAGAAGT

>NL13.C01.02_A_80

AACTCATCTATTATTTCCTTCAGGCTTGCCATTTTTTTCGCCT

>NL13.C01.02_A_81

TCTTGTTCTTTTGCTATATCTAAAATTTTTAGCAATTGTTTAG

>NL13.C01.02_A_82

GTTGGTGCAATTGAACCACTCGTGAAAACAGATGATAAA

>NL13.C01.02_A_83

AGTTACAAAGTTCAGTTACAAAACAATATGATTTTGCA

>NL13.C01.02_A_84

AATACTATTTGAGCAGTTATAAATACGCCAAATTCATCA

>NL13.C01.02_A_85

GTCATTATCAGGTAAGTTGGTAATGGTTTTTTCCACAGTCTG

>NL13.C01.02_A_86

TATAGATTGAGGGCTGAACTCATTAGGTGGGAACGT

>NL13.C01.02_A_87

CGAATGAGTTAGTGTTGGATTGGCATCATGTATCCGCCAA

>NL13.C01.02_A_88

GTATATTTTCTAGTCTGGTTATCTTGAGGTCATATAT

>NL13.C01.02_A_89

TTCCCTTACCTTTCACTTTCACGTATTTTTACATAAGCTAA

>NL13.C01.02_A_90

TTCTGTAATTGTTGTTTTTTAATATCATACGTTTTATTAGG

>NL13.C01.02_A_91

ACCCCATGTCCTCATATGGTCGTTTAGCATCTCTTCGAT

>NL13.C01.02_A_92

GAGATAGAAAAATAGAAAAATCTGTAAAATTAGACTAGTTAA

>NL13.C01.02_A_93

AAATTCGAATTAAGGGCTCTGATTATTAGAAAGGACAGTAT

>NL13.C01.02_A_94

TAAGCCGATTTAATACTGATATTTTCTTCTCTTAGTTTTG

>NL13.C01.02_A_95

CGTCTGGTTGGACACGTAAATGTAAGAACCCTGTGCTACTTCAA

>NL13.C01.02_A_96

TGCAACCCATGCTCTTCTGCCATGTAAGTTGTTCCTATACT

>NL13.C01.02_A_97

TTTCTCATACCTGGAGACAGAAGCCCAGGTCTTTTTTCTAA

>NL13.C01.02_A_98

TAAATAAATCCCGTAACTCACCTCGCCTAGGAAGACTGT

>NL13.C01.02_A_99

AGGTATGCCCCATGGGGGCGGGGCAGGGAAAAACTCACGT

>NL13.C01.02_A_100

GTTTGTAAAACAATAACTAAACAAAGAAAATCTATGATTA

>NL13.C01.02_A_101

CAATCTCCCGGAATTCCATCATCTCCAAGCTCTCCCTCCA

>NL13.C01.02_A_102

TCTGAAACTCTCTTTAGCTTTGCCTTCAAGTCCTTTTTTCTT

>NL13.C01.02_A_103

TATCATAGTTCTAGAGATTCCCAATTTATCGTAAGGAA

>NL13.C01.02_A_104

AAAATTCCACTTTGGCCTAATTGTAGAAAACTAAATGAAT

>NL13.C01.02_A_105

ATTCTTAGTACCACAGGATTATACAAAAACACAGCACCT

>NL13.C01.02_A_106

AGCTCAGATAATGGCTTTTTCTCTCTATCGGTAAACTTCA

>NL13.C01.02_A_107

ACTGAATATCTTTCTAAGTAATCCGTTAGTCGCTATTAGTTC

>NL13.C01.02_A_108

TCAAATTCCCAACCACAAAAAACAATAATACTAATCACGT

>NL13.C01.02_A_109

ACTGTCTTAAAGAATGCTTAACGTTAGCGTGGAGCAAGTCC

>NL13.C01.02_A_110

TCACCTTTCCATTGCTTCTTTTCTTCCTTCCCTTCTTTCTC

>NL13.C01.02_A_111

AAGCAATATCTAAACTCAATAAGATACTATCTGCACACAGT

>NL13.C01.02_A_112

ATTACAATTTATATGTTTGTAAGAAATTCGATTCAATATCCT

>NL13.C01.02_A_113

TATCCATATCGATACTCCGAATAATTTCGGATTTTGTGGAAC

>NL13.C01.02_A_114

TTTCAAGCCACGCTCTGGTTATTTGAGTGGACAAGAA

>NL13.C01.03_A_1

TTAGCCTCCCCGCTAATAATCGTCGTCTGACCCGTAT

>NL13.C01.03_A_2

ATTATACGGCGTCCCTGGTACTGGTAAATCATACATTGC

>NL13.C01.03_A_3

ACAACCTATCCGTGCTTATAGGAGTAATACCCAGTGTTTGT

>NL13.C01.03_A_4

TTATTTAGACCTGTTTCAATTCGTGATGTAATTTTAG

>NL13.C01.03_A_5

AAATTCACTTCTGTGAATGAGCAAGTAGCATCAGTTTTAAG

>NL13.C01.03_A_6

ATCAGTTAGATTCAAGTTGTTGCTTGATCTTGATTACGATTA

>NL13.C01.03_A_7

AACGTCAATAGCAATGATAGCTACACTCATAACTGCTGTACT

>NL13.C01.03_A_8

AACCTATAGGCGATATAGAAGACGTGCTTAATGAGAGATA

>NL13.C01.03_A_9

TCTGGTATGCCCTCATACGATAATATACGATAACAAAATAT

>NL13.C01.03_A_10

ATATTATGGCAAATAGTGAGTTTAAAATGTATAACGAAGA

>NL13.C01.03_A_11

CTGGGCAAGAAGAAGATAGAACACACCTGGGATTACAGCGT

>NL13.C01.03_A_12

CATATCCTTCTTTTTCCAATTCTTCTTGGTCAAGTTCATCA

>NL13.C01.03_A_13

GTCAAAAGAGCCATAACTATACTTCCACTAATTTTCAATA

>NL13.C01.03_A_14

GACTATTTGAAGAGAGTGTTGAAAATAATAGCAGACAATG

>NL13.C01.03_A_15

GCACCTTTCGCCAAGCCTGACTCTAACGCTAGACCGCCTGC

>NL13.C01.03_A_16

TAAACCAACCGCAGTATTATTCCCAACAATACAATGTAT

>NL13.C01.03_A_17

TTTGATACCGTTATGATAGACTGAACCAGTTAGTATGCCA

>NL13.C01.03_A_18

TCTCCAACAAGCACTTCAGTATATTCCGTCGAATTCACAG

>NL13.C01.03_A_19

CAGGCTGATAAAGAACTGAACAAAGATCTACATAATAATCG

>NL13.C01.03_A_20

TATCTATCCAATTAAAGATGTAGACAAAGCTATAGATTTT

>NL13.C01.03_A_21

AGTATGAGCTAGAAGTGGATCTTACAACTACTGTTTCTTT

>NL13.C01.03_A_22

TAGAAGATTTGCATTTTGTGGTACAGATGTATCAACTCTAA

>NL13.C01.03_A_23

ATGCGGGGAAGCCCATGAGGGTGGGCGTGTTAAACGTGGTTTGT

>NL13.C01.03_A_24

TTTTTATGACAAGTTAAAGACATGAAGCCAGAGTGTAAAGTTT

>NL13.C01.03_A_25

GAAAATGTAATTTGTTCCTGAACAGAATGGCCTAATCTCAT

>NL13.C01.03_A_26

TAAGATAAAATGCAAACTCGGTAGCAATGTCTTTGCATAA

>NL13.C01.03_A_27

TTCTTTTTATGTGAAATTTGACATAATTATCAAGCTTTAGA

>NL13.C01.03_A_28

GAAGAAATAAGAAAAGTCTATGATAGATTCAGTGGTCTC

>NL13.C01.03_A_29

TTAAATTCGCAATTTTAGCATAAATAATTATTTGCTCCTGG

>NL13.C01.03_A_30

CTTATAGGGTCTCTAACCTCTCCATTCACTTCCTCTTCTACCTC

>NL13.C01.03_A_31

TTTCTCAACGATTTCGTTACTTGTTGCAGGGATAACTG

>NL13.C01.03_A_32

TCCACGATCTTTTGGAGATTGAGAGTTTCTGTCATTTTTGGTT

>NL13.C01.03_A_33

GCTACTGTGGTATATAGCATGACAACTGGGTTACCATA

>NL13.C01.03_A_34

ATTGTCAACCCGCCACCTTCACCGATCGTTAATCCACCACCTT

>NL13.C01.03_A_35

ACTGATGCAATTCAAGAATTGGAGAAACTAGGAATGCAAA

>NL13.C01.03_A_36

CAAAATAGAATTCTCACGCTTTGCTATTGGCTTTATTACGAC

>NL13.C01.03_A_37

CCTAACCCACCGATACATAAACCAGAACCAATCGTTAA

>NL13.C01.03_A_38

AAAGAAAGGTGCAACACATCGACCGCAAACGCATAAATAA

>NL13.C01.03_A_39

TTGTTAGATTACAAGTATTGGCAGGAGAGAACAACGCCAG

>NL13.C01.03_A_40

TGTAGAAAACATCCTTGAAGCACCCAACATAAAGAACAAA

>NL13.C01.03_A_41

AGTGTTAAGTAAGGAGTTATGCTATCCAATTCTGCCGA

>NL13.C01.03_A_42

TGATTACGCAAAACTTGTGCAATATCTTCACTCACATTTCT

>NL13.C01.03_A_43

GTATATTTTCTAGTCTGGTTATCTTGAGGTCATATAT

>NL13.C01.03_A_44

GGGTTTTATAACGTAGTCGTTGTACCGTCTGAAAATAACA

>NL13.C01.03_A_45

ATTCCCAAAGTTGGAAGATCAATACTAATATTTGAATAAC

>NL13.C01.03_A_46

ACCAGTGAAGACGGAAGTTACGGAGAAGCAGTAATCGAGTT

>NL13.C01.03_A_47

TTTACTAATGGAGAACAAGCACAAGAAGCCGGGACAGCAATACAA

>NL13.C01.03_A_48

TCACATTCAGATATAAATATTCAATTAGTTCCGAAAACA

>NL13.C01.03_A_49

TATAGATTGAGGGCTGAACTCATTAGGTGGGAACGT

>NL13.C01.03_A_50

TCGCGGTAATGCATCGACACGGCGAAATACTGCGACAG

>NL13.C01.03_A_51

ATTGCAGAAGGGTCGTCAAAAAGTACAACGTCATACCGTTCTT

>NL13.C01.03_A_52

TTAATCATGTTAACTAACGCCGGTCCACCGCCAAGTCTATT

>NL13.C01.03_A_53

TATCATAGTTCTAGAGATTCCCAATTTATCGTAAGGAA

>NL13.C01.03_A_54

GTAATTCCATATTCGCTCAAAACTGATGCTACTTGTTCATTTA

>NL13.C01.03_A_55

CTTTTCCAGATAGGACCGTTTGCCCTATCCTAAAACTAGTAG

>NL13.C01.03_A_56

TATGATAAAAAAGGCTAGAGAAAAAGTGAAGGAAATGCTA

>NL13.C01.03_A_57

TTCTAACGTTTTAGGAGTAAGTTCTACAGTAAAATTGAAA

>NL13.C01.03_A_58

GCACCTTACGGCGGTGTTGAACAATTCGTGAACAACGTGG

>NL13.C01.03_A_59

ACTTCAACACCTACTCCAACACCATCGCCAGTATTAAA

>NL13.C01.03_A_60

CCTGTACTTTGCGTAGTGGTTGATGCGGTAGTAGTTGTGGA

>NL13.C01.03_A_61

GAAAATAATTCCTAGAAATATTATAAGTACTATCGACAAT

>NL13.C01.03_A_62

CAGGCTTATACATTTGGTGAAGGAAATCAACAGCCATTTAA

>NL13.C01.03_A_63

ACGTTTAGTTCATAATTCGTATTGTAGGAGTATTCTCTCCC

>NL13.C01.03_A_64

ATTTAGTATACGATGCTCGAAACAATATGTAAGGTGCAG

>NL13.C01.03_A_65

TAACGTGTTCTGGTAATTCCAATTTAGCACTCTCATCGTTT

>NL13.C01.03_A_66

TGAACTTGTAAAAAAAGGCTATTCAATAGCTCAAATTGCAAA

>NL13.C01.03_A_67

CAAATAAATTTTTAAACTTATCCATCCCCGCCCTGAAGGGT

>NL13.C01.03_A_68

TACGGATCGTGATTAGGGGGACACCCCTGTGGGGGATAT

>NL13.C01.03_A_69

TATTTCTAACTCTGTTGCTCACATGACTGAATTTGTCCATTTA

>NL13.C01.03_A_70

CTGTAAAAGCCCAGCGACCATAATGGTTTTGTTTATAACCTCGT

>NL13.C01.03_A_71

AACATTAAAATCCACTAATCCTACAATCCGAGAAAATTTC

>NL13.C01.03_A_72

CCTCATAAATTAGAATTGCTGAGGCACTTGTTAAGCTTAAGCT

>NL13.C01.03_A_73

ATAGTAGTGATAAATAATGCATATGTATTAAATAATAAAT

>NL13.C01.03_A_74

ACAATATAGGAAGTCGCATTGGGTCTTAACTGGAGAT

>NL13.C01.03_A_75

TACAAGATATACCTCCTCCCGTTTTTGATCATCTTCACTG

>NL13.C01.04_A_1

ATATACACTGACACTTGTCATAACTAAAAATAATGCCATCTT

>NL13.C01.04_A_2

CGAAGTTATTCGCAAAGATATGCAAAATGTTCAGCTAAATT

>NL13.C01.04_A_3

AAACATAATAAAAATGCCTACCAAAAACTCCTTTATTTATTT

>NL13.C01.04_A_4

AACCCACACTGGTACGCCAAATACCTTTTTGTTTAGGT

>NL13.C01.04_A_5

GTTATTCCATATTCCGACAAAACTGAACTAACTTGTTCATTCA

>NL13.C01.04_A_6

TTTGATACCGTTATGATAGACTGAACCAGTTAGTATGCCA

>NL13.C01.04_A_7

GTTTAGAAGGAGAAAGATTGACAATTCAAAGAAGAAAGGGACT

>NL13.C01.04_A_8

CATGTACATATCAATATAGTCTTATCATCATCAGGCAAGT

>NL13.C01.04_A_9

ACACTGGTACTCCAAACACTTTTTTATTCAGGTCTGCTTCT

>NL13.C01.04_A_10

ATCAGTTAGATTCAAGTTGTTGCTTGATCTTGATTACGATTA

>NL13.C01.04_A_11

TTCTTTTTATGTGAAATTTGACATAATTATCAAGCTTTAGA

>NL13.C01.04_A_12

GCTACTGTGGTATATAGCATGACAACTGGGTTACCATA

>NL13.C01.04_A_13

TTTTTATGACAAGTTAAAGACATGAAGCCAGAGTGTAAAGTTC

>NL13.C01.04_A_14

ACTATAATATTATAATTTATAATTTTGTCAAACCAAAACTCC

>NL13.C01.04_A_15

TCTTTACAATTTGCAATTTTTGAATTGTAAGTTTTAGCTACTAA

>NL13.C01.04_A_16

TAAATGCATTGAATAATTTTTCTAACTCATCTTTCTCG

>NL13.C01.04_A_17

GTATATTTTCTAGTCTGGTTATCTTGAGGTCATATAT

>NL13.C01.04_A_18

TGTAGAAAACATCCTTGAAGCACCCAACATAAAGAACAAA

>NL13.C01.04_A_19

ACCAGTGAAGACGGAAGTTACGGAGAAGCAGTAATCGAGTT

>NL13.C01.04_A_20

TCAGCTGGTTTTATATACGGATGTGATGTAACAGATCTATT

>NL13.C01.04_A_21

TCTAAACTTTTCAATTTCCTTCGGTCTAGATGGATTATTATT

>NL13.C01.04_A_22

ATGCTTATAAACGTGAATTGGCAACTATAATACGGGGCG

>NL13.C01.04_A_23

TATCATAAACCCAATCGAAGCAGGAATAGGTCACTTAGAA

>NL13.C01.04_A_24

GGAGCAAATAATTATTTATGCAAAAATTGTGAATTCAAAG

>NL13.C01.04_A_25

TTGAAGGTATAGCGTTATATGTAGATGAAAGAATAACGGAGCC

>NL13.C01.04_A_26

AAAATATGAAGATATGGAACTTTTTTTCTTATATAATAAA

>NL13.C01.04_A_27

TGAACTTGTAAAAAAAGGCTATTCAATAGCTCAAATTGCAAA

>NL13.C01.04_A_28

TCTCATATGGTATAATCGTAAAGCAAGCAAGATCTGCCTT

>NL13.C01.04_A_29

ACATCTTTAGGAATTACATTTCCCATTATCGTATAACTCCTTTC

>NL13.C01.04_A_30

ACAGTATTAGACGCAGTTACTAATATAATAACTGGATCTCC

>NL13.C01.04_A_31

CTGTAAAAGCCCAGCGACCATAATGGTTTTGTTTATAACCTCGT

>NL13.C01.04_A_32

TTGAAAAGGTGTCATTAAACTTCATATATAGGAATGAGACGA

>NL13.C01.04_A_33

AAGTTATACTCTGGGCGGTATCTATACAACTCTATTGCTT

>NL13.C01.04_A_34

TCTGGTATGCCCTCATACGATAATATACGATAACAAAATAT

>NL13.C01.04_A_35

ACTCGGTATCATTTTTTATCTCTAACTTCATTTTTTAG

>NL13.C01.04_A_36

TCAATTTTCCATTTTTCAGGTTCCATAATTCCATTTTTGG

>NL13.C01.04_A_37

GAAAATGTAATTTGTTCCTGAACAGAATGGCCTAATCTCAT

>NL13.C01.04_A_38

GTTACGTTTACGTTTCTGTGCAGCAAGGTAATTCGTTTAG

>NL13.C01.04_A_39

TGAGCGTTTTGCAACGCTGTGCCTGCTTCTTGTGCTTGA

>NL13.C01.04_A_40

ACTTGTGCAAATCCAAATTCATCTTGATTTATACAAAAATTATTGT

>NL13.C01.04_A_41

TAGTATTTGCTTAGGCCCCGGCGCTGTAGAATGTAAGGCA

>NL13.C01.04_A_42

AACGAATGTAATTGCTTTTTAATCTCATCAGTTGATAAATATCC

>NL13.C01.04_A_43

ATTCTGATTTACACCCCTTACTTAAAAAATAAGACAGA

>NL13.C01.04_A_44

TCTCCAACAAGCACTTCAGTATATTCCGTCGAATTCACAG

>NL13.C01.04_A_45

GAAGACAATGTTATACCGATACTTGATACAGATAGAGGTAT

>NL13.C01.04_A_46

ATAGTAGTGATAAATAATGCATATGTATTAAATAATAAAT

>NL13.C01.04_A_47

GAGGGAGGACGACGACGATGACAATAAATGAGATAATTGAAGAA

>NL13.C01.04_A_48

TAATTTTCTTAGTCTTACTAACTTCTAAATCTGGAATTACTAT

>NL13.C01.04_A_49

TCCTTAAGCTTCACTACGACTCTACTGTCTAGGTTCTGT

>NL13.C01.04_A_50

ACTGTTACAGCTGTAGTACAAACAGTAAAAGCTGCATTATC

>NL13.C01.04_A_51

TGACGGGATACCTTATAGTTATATATGAGGTGGGATAAA

>NL13.C01.04_A_52

ATTCCCAAAGTTGGAAGATCAATACTAATATTTGAATAAC

>NL13.C01.04_A_53

TTTACTAATGGAGAACAAGCACAAGAAGCCGGGACAGCAATACAA

>NL13.C01.04_A_54

TGATTTCGCAAAACTTGAGCTATATCTTCACTTACGTTA

>NL13.C01.04_A_55

AAACTTTTGGAATATTTGAAAGAAGCAAGGGAAAGAGGA

>NL13.C01.04_A_56

TTTGCCACTAATTGGACGGCATTTATTTCCCTTATGTCCCAGCT

>NL13.C01.04_A_57

AGGTGAAGAGAAAGCCAGCAGAATGCTTAACCAAAAAGCTCA

>NL13.C01.04_A_58

CCTTACAATTTCCTCCATAAAATACTGAGATAAATCCAAACTG

>NL13.C01.04_A_59

TAACGTGTTCTGGTAATTCCAATTTAGCACTCTCATCGTTT

>NL13.C01.04_A_60

CTTTTCCAGATAGGACCGTTTGCCCTATCCTAAAACTAGTAG

>NL13.C01.04_A_61

CCTGTACTTTGCGTAGTGGTTGATGCGGTAGTAGTTGTGGA

>NL13.C01.04_A_62

GAAAATAATTCCTAGAAATATTATAAGTACTATCGACAAT

>NL13.C01.04_A_63

AAATAGTTTGCAACTGTTTTAGGTGAAATGGGCGTAAATGGCT

>NL13.C01.04_A_64

GTCTCGGTTTTCATGGGTATCATTGTCGTTATCCTCCCCT

>NL13.C01.04_A_65

CCATCATCCCCAGAGGTTCCCCTGTTTCCGCGTCCATCAT

>NL13.C01.04_A_66

TTTTCACAACATCAGCCTCTGAGATATCGAAATATTCCGC

>NL13.C01.04_A_67

TACGGATCGTGATTAGGGGGACACCCCTGTGGGGGATAT

>NL13.C01.04_A_68

TATCATAGTTCTAGAGATTCCCAATTTATCGTAAGGAA

>NL13.C01.04_A_69

AGAAATCCGCTTTTTAGACCAGTCGCTATAAGAGATGTAAT

>NL13.C01.04_A_70

TTATATTGAAGGTGCGATATGATGGGAAAGACAAAGG

>NL13.C01.04_A_71

GTATTGGCAGTGGTACAATACCGATGATTGCGTCGTA

>NL13.C01.04_A_72

CAGGCTTATACATTTGGTGAAGGAAATCAACAGCCATCTAA

>NL13.C01.04_A_73

AATGATGTATGCAGAATCGAAATCTCTTAGAGCATTAAGTC

>NL13.C01.04_A_74

AACCTGGTCAGGCAAGCAATCAGGGAAAAAGCTATCATAC

>NL13.C01.04_A_75

AATGCTTTACTATTAATCTTTGTAGCTGACACAATTGCAAA

>NL13.C01.04_A_76

AAATTCACTTCTGTGAATGAGCAAGTAGCATCAGTTTTAAG

>NL13.C01.04_A_77

GTTATTGATTCACCTTTTGCATAACATGTTGAAAAAACTT

>NL13.C01.04_A_78

ATATTATGGCAAATAGTGAGTTTAAAATGTATAACGAAGA

>NL13.C01.04_A_79

GTTAATTTCCGAGGGTAAATATCAACAAGCCTTACAGTTA

>NL13.C01.04_A_80

TAGAAGATTTGCATTTTGTGGTACAGATGTATCAACTCTAA

>NL13.C01.04_A_81

ACAAGAAAATTATGTTCATGTCACCTTGGAAAAATATCTGA

>NL13.C01.04_A_82

ACTCTGCGGTTTCAGAGCTATTAAGTTATTCGCTTTGAAA

>NL13.C01.04_A_83

TTGACTCGAGAACGGGCATTCCGAATCCTTCAGTTCC

>NL13.C01.04_A_84

TATAGCGCCGATTGTTCCATATTTACCTATTTTAAGTA

>NL13.C01.04_A_85

ATTATAGTATGTAAACTATAAATTGTAGGAATATAAGTT

>NL13.C01.04_A_86

GACTTTCTTAATTTCTATCAGAGAGGCCCAGCGTTTTAGCC

>NL13.C01.04_A_87

CCTAACCCACCGATACATAAACCAGAACCAATCGTTAA

>NL13.C01.04_A_88

TCACCGCTTTACTCAGGTCCATCCATAGAAACTGTAAGAATAA

>NL13.C01.04_A_89

TTGTTAGATTACAAGTATTGGCAGGAGAGAACAACGCCAG

>NL13.C01.04_A_90

AGTGTTAAGTAAGGAGTTATGCTATCCAATTCTGCCGA

>NL13.C01.04_A_91

TGATTACGCAAAACTTGTGCAATATCTTCACTCACATTTCT

>NL13.C01.04_A_92

CTTATAGGGTCTCTAACCTCTCCATTCACTTCCTCTTCTACCTC

>NL13.C01.04_A_93

AAGTTATACTTTTATAAATATATAAATATGTCATATTATAAGT

>NL13.C01.04_A_94

CCAGGTTCCTCCTTAGGACTTTAGGGTCTTTTCTCTGCT

>NL13.C01.04_A_95

ATTCTTATGCTATCAAGTTAAGGAAAATTGCCAAGTTTAA

>NL13.C01.04_A_96

GTAAATTATAAAATAATAACGCGAAAGAATTAATTTTCCCC

>NL13.C01.04_A_97

TATGATAAAAAAGGCTAGAGAAAAAGTGAAGGAAATGCTA

>NL13.C01.04_A_98

TTCTAACGTTTTAGGAGTAAGTTCTACAGTAAAATTGAAA

>NL13.C01.04_A_99

ACTTCAACACCTACTCCAACACCATCGCCAGTATTAAA

>NL13.C01.04_A_100

GAAACTATACCAAATTTTATAGAACTAGGAAAATCTTTGTCT

>NL13.C01.04_A_101

ATATGGTCCTGCTAAACTTCTCCAGTATTATCAAAATGG

>NL13.C01.04_A_102

TCCTTTTGCGTCCTTTGATTACTATCTCCTTTCCGTTCAC

>NL13.C01.04_A_103

GTGCCTCTGTTCTGCAGTCTACACAGAAAGCAAACTTTTAA

>NL13.C01.04_A_104

TAACCCAGGTCGTAGACCCGAAAGGAAACGCTGTATACGAA

>NL13.C01.04_A_105

CATATTGCATAGCTATTCATGATTGGACAGAAAATCGTTT

>NL13.C01.04_A_106

TTAGGAACTGAAATGTTCTCAAGTGGAGTTGGCAAAACTGA

>NL13.C01.04_A_107

ACAACCTATCCGTGCTTATAGGAGTAATACCCAGTGTTTGT

>NL13.C01.04_A_108

ATTAATGTTAGAAATCCTTCTAGTGCGTCTGCACTTAGCGT

>NL13.C01.04_A_109

AGGTGGATTCTCAAAAGGGTTCTCGTTATCATATGAAAA

>NL13.C01.04_A_110

TACAAGATATACCTCCTCCCGTTTTTGATCATCTTCACTG

>NL13.C01.04_A_111

TTAGCCTCCCCGCTAATAATCGTCGTCTGACCCGTAT

>NL13.C01.04_A_112

TTTTCTGCATGCAGAAGCAGAACAAACGGATAAGGCTGAA

>NL13.C01.04_A_113

TTATTTAGACCTGTTTCAATTCGTGATGTAATTTTAG

>NL13.C01.04_A_114

GTTGGTGCAATTGAACCACTCGTGAAAACAGATGATAAA

>NL13.C01.04_A_115

TTCACTTCAGCTAGCTGAATAAAAAAATTTAAATTAA

>NL13.C01.04_A_116

AGTATGAGCTAGAAGTGGATCTTACAACTACTGTTTCTTT

>NL13.C01.04_A_117

TTTAATAAATGTAAAGCCTAAGCCACAGCCAACTACTTCCC

>NL13.C01.04_A_118

TATAGATTGAGGGCTGAACTCATTAGGTGGGAACGT

>NL13.C01.04_A_119

TTAAATTCGCAATTTTAGCATAAATAATTATTTGCTCCTGG

>NL13.C01.04_A_120

TATTTCTAACTCTGTTGCTCACATGACTGAATTTGTCCATTTA

>NL13.C01.04_A_121

TTAGGAAATACAATAAATGCAATTTATTTAAGCGGTTAT

>NL13.C01.04_A_122

AACACTCTAGTAGGATTTTACGCGTCACCTACCGCTAGGT

>NL13.C01.04_A_123

TCGCATAGCATTTTTACGTTCTTATTTTTGATATCGAAA

>NL13.C01.04_A_124

AAAGAAAGGTGCAACACATCGACCGCAAACGCATAAATAA

>NL13.C01.04_A_125

AACTTGAACGATATAATAAACAGCGGTTTTGCGTTAGAT

>NL13.C01.04_A_126

GGGTTTTATAACGTAGTCGTTGTACCGTCTGAAAATAACA

>NL13.C01.04_A_127

TCACATTCAGATATAAATATTCAATTAGTTCCGAAAACA

>NL13.C01.04_A_128

ATTTATGAGTTTTAGAATACTCCTATTTCTTTTGAATCGTAT

>NL13.C01.04_A_129

GTCAAAAGAGCCATAACTATACTTCCACTAATTTTCAATA

>NL13.C01.04_A_130

AGCAATTAGCGATATTCCAACCTTTATGCAAAAAATTG

>NL13.C01.04_A_131

ATATAGACTATTACCCTGAAACTTGTGCTGATAATATAAAGGGACT

>NL13.C01.04_A_132

ACTAACACATAACGAAATGAATAAATATAATAATTTTGGAATA

>NL13.C01.04_A_133

CACGGTGACTTTGCGGAAGAAATGCTATCGTTCTATGCAAAT

>NL13.C01.04_A_134

TTCAGGTACAGGTTTAAGATAGTATGAGAAGAAATAAA

>NL13.C01.04_A_135

ATTCTTAGTACCACAGGATTATACAAAAACACAGCACCT

>NL13.C01.04_A_136

CAAAATAGAATTCTCACGCTTTGCTATTGGCTTTATTACGAC

>NL13.C01.04_A_137

TATCAAGAAATCATAATACCAAAGCAAAGAACAACAACGG

>NL13.C01.04_A_138

GTTATCCATCAGCTCATGCCACCGTTTGATGAATTTACATCA

>NL13.C01.04_A_139

CTAGGTTGTTGTTGCAGTTTTGCAACTACAAGTATAT

>NL13.C01.04_A_140

CAAATAAATTTTTAAACTTATCCATCCCCGCCCTGAAGGGT

>NL13.C01.04_A_141

ATTTTGCCAAACGATTCAATAGAAATTCATGTTAATAAT

>NL13.C01.04_A_142

TTTATAACTTTTCTTCTTATTAAATACATAATTCCTCCAATGAT

>NL13.C01.04_A_143

AAGCAATATCTAAACTCAATAAGATACTATCTGCACACAGT

>NL13.C01.04_A_144

GAGATTAACTTCTAGCATGTGGAATACCGCATCACTTTT

>NL13.C01.04_A_145

TAAGCTGTAAGAGAAGAGGGTAAAAGACTCTTGAGCAT

>NL13.C01.04_A_146

TATCCATATCGATACTCCGAATAATTTCGGATTTTGTGGAAC

>NL13.C01.04_A_147

ACAATATAGGAAGTCGCATTGGGTCTTAACTGGAGAT

>NL01B.C01.01_NL_0

TACAGTATACTCAAACGAAGGACGCCGTCGATATT

>NL01B.C01.01_NL_1

TAAACGATGCCTATACATTTTTCCAAGATGTCGTTAAT

>NL01B.C01.01_NL_2

TTTTATAAAAACCGTGAAAGAAGACCTAGAATCTCAAAA

>NL01B.C01.01_NL_3

TTGGATCTAATTCAGTTAGCACTGGCTGACCAGAAACGGT

>NL01B.C01.01_NL_4

TACTGCTGTGGCATAGCGTTCAAACTTCAGGTTAGTGTA

>NL01B.C01.01_NL_5

TGATGAATTGAAAGTACATGCTTGATTTCAAATTTCAA

>NL01B.C01.01_NL_6

TGCGATATTAATAGCTAGCATGATAGCTGCACTAAT

>NL01B.C01.01_NL_7

TATGAAGACCGAATAGTACTGCTAATAACATCATAAC

>NL01B.C01.01_NL_8

CTTGTTAGCTTAGGAATTAATAGCTGTAATTGTTTA

>NL01B.C01.01_NL_9

ATATCAAACAAACTAGTATTTGATAGTCGGTGGGAAA

>NL01B.C01.01_NL_10

GGAATACTATGACAATGATCAAGATGATGATTATCG

>NL01B.C01.01_NL_11

ATATTATTATCTCGATCCTTGTAGGCGGGTTCATGT

>NL01B.C01.01_NL_12

TGGATTTAGTAATGAAGGGGAATATGTTTCACGTGAT

>NL01B.C01.01_NL_13

ATAGGCTCGATGCAAATCCCTCACTCACTCATTAC

>NL01B.C01.01_NL_14

TACTTCTATTTTCTATTTATGTTATGGACGCAGTAA

>NL01B.C01.01_NL_15

TGGAATGAAGCTTTGCGTTGTTGAAACGCTATACTG

>NL01B.C01.01_NL_16

CCCTCGGAAATTAACTGGTTTATCTGTTGTGCGATAG

>NL01B.C01.01_NL_17

TCAGTAGTTTCACTTCCTAAATCTTTAGCGAGAGGGTT

>NL01B.C01.01_NL_18

TGATGATACTATATGAAATTAGGGAAAACGAGAAAG

>NL01B.C01.01_NL_19

TTTTGCGGAATAGTTGTAAATCAAGTACGCCGTCACACT

>NL01B.C01.01_NL_20

TAGAGAATTGGAAAAAGAAAAGTTTAATCAAAATGT

>NL01B.C01.01_NL_21

CTCCACACAATTTAGCAGCTTTAAATTCACAGAAAGGG

>NL01B.C01.01_NL_22

TTTTCTTGCTCAAACTGTTGTTCGTTCACATCCTC

>NL01B.C01.01_NL_23

ACTTTTGTTTTGTGTTTTTTGATTTCTCATTCTTTT

>NL01B.C01.01_NL_24

TAATTTTTGATATACTGAATCAACTTGTTTCACATAAGGCTGAGGG

>NL01B.C01.01_NL_25

AGACTTGAAATGATTGCATTAAAATCAGCAATTATTGATA

>NL01B.C01.01_NL_26

TAATTAAAAAGTTCTAAATTTGACCTCATAATGTAGTTC

>NL01B.C01.01_NL_27

TCTTTTGAGAAAAAAGAAAAAGAAGCCTGTTTATACT

>NL01B.C01.01_NL_28

TAACATGTCAGCACTTCATGACGTAAACAGTCTGGCA

>NL01B.C01.01_NL_29

TTTTTCGAGATCGTGACGGGCTGCGGTTGTCCGTTCT

>NL01B.C01.01_NL_30

TTCTATTTCCTTCTTTGAAAACTCTCGCTAGAGAA

>NL01B.C01.01_NL_31

TTGAGTAATCCCGAGAAAGCATTCCGCTCGCCTAGTTC

>NL01B.C01.01_NL_32

TTGAGAACGGACAACCCATGGTAACAAACGACCCGATAACG

>NL01B.C01.01_NL_33

CATTGTAACATCCAAATACGGGCCTAAGGAATATG

>NL01B.C01.01_NL_34

TATTAAGGAGAAAAAGCCAGAAGTGATAGCAGTAGT

>NL01B.C01.01_NL_35

TAACCACCAAAAAGTGTATTTTAGAGATATCCAGA

>NL01B.C01.01_NL_36

TCATCGTCTCTAATAAAAAACATGATTAGTAGTTTG

>NL01B.C01.01_NL_37

AATACATCAAGTATAGCATTTTCTAAATAACTAA

>NL01B.C01.01_NL_38

TTACCTGGGGGAACTGAAATATTTTCAAGTGGAGTTGG

>NL01B.C01.01_NL_39

TAAAGGTGCAAAAAAAGTAATTGGATATGAGAAGAGTGG

>NL01B.C01.01_NL_40

TACTTCTTGACTACCTCTCTAACGTCAACCCACTGTAT

>NL01B.C01.01_NL_41

TCTTATAAAAATCCAGATAATGAATTTGAGCCTATT

>NL01B.C01.01_NL_42

ACCAGGCAGTCTCGCAGTTCAACAAGGCGGGAGGGC

>NL01B.C01.01_NL_43

TAAATCTAAATAAGGTCTCCCAAAATTACTTAAGGCACGTGAAA

>NL01B.C01.01_NL_44

TTCAAGTCCTCTAAGGATTTCAAATTTCAA

>NL01B.C01.01_NL_45

TGATGAATTGAAAGTACATGCTTGATTTCTCATTCTTTT

>NL01B.C01.01_NL_46

TAAAGGTGCAAAAAAGTAATTGGATATGAGAAGAGTGG

>NL01B.C01.01_NL_47

TTTCTACAGATTTCAAGTCCTCTAAGGATTTCTACAAA

>NL01B.C01.01_NL_48

TTTTGCGGAATAATTGTAACATCCAAATACGGGCCTAAGGAATATG

>NL01B.C01.01_NL_49

GTTTCAAGTCCTCTAAGGATT

>NL01B.C01.01_NL_50

TACAGTATACTCAAACTGTTGTTCGTTCACATCCTC

>NL01B.C01.01_NL_51

TTCTATTTCCTTCTTTGAAAACTCTCGCTGAGGG

>NL01B.C01.01_NL_52

TACAGTATACTCAAACGAAGGACGCCGTCACACT

>NL01B.C01.01_NL_53

TTACCTGGTTTATCTGTTGTGCGATAG

>NL01B.C01.01_NL_54

GATGATGAATTGAAAGTACATGCTTGATTTCAAATTTCAA

>NL01B.C01.01_NL_55

GACTACTTCTTGACTACCTCTCTAACGTAAACAGTCTGGCA

>NL01B.C01.01_NL_56

CCCTCCACACAATTTAGCAGCTTTAAATTCACAGAAAGGG

>NL01B.C01.01_NL_57

TACTGAGTTTCAAGTCCTCTAAGGATTTCTACAAA

>NL01B.C01.01_NL_58

CTTGTTAGCTTAGGAATTAATAGCTAGCATGATAGCTGCACTAAT

>NL01B.C01.01_NL_59

TATTAAGGAGAAAAGCCAGAAGTGATAGCAGTAGT

>NL01B.C01.01_NL_60

TCCGCACCTACGACCAGAACTACGATTGCCACTATTATG

>NL01B.C01.04_NL_0

TTTTGAGATTCTAGGTCTTCTTTCACGGTTTTTATAAA

>NL01B.C01.04_NL_1

TCTCAAAATTAAGAATGCGTTAAAAGATGCAGTAAAA

>NL01B.C01.04_NL_2

TATTACCAGCACTGTTCTCAGTGAGGATTACGGTTTCTG

>NL01B.C01.04_NL_3

ATTGATGGATAACTGGAGTTCCCATAGCCATGCTTT

>NL01B.C01.04_NL_4

TGGGCACTATACTGTCATACCTAGAGGTCATGTATAACG

>NL01B.C01.04_NL_5

TGCGAGGAAATTCAGTACAGTGATGGAAGCATGGAAAG

>NL01B.C01.04_NL_6

CATCCGAGAATATTCTGGAAAGTGCCATTTGAAATGC

>NL01B.C01.04_NL_7

TGTCTTCCCTCAACCTCTACGGAACGAATAAAAAAA

>NL01B.C01.04_NL_8

TTTCATTAGCCCAGTCGTAATAAAGCCAATAGCAAA

>NL01B.C01.04_NL_9

CGATGCGATTAGAAGACTATCAGAGCTATTATAAAGTA

>NL01B.C01.04_NL_10

TTATAATATCTATACCAGAAGCATAGGAAGGAGACA

>NL01B.C01.04_NL_11

CTTGAAGTTAAGGCTGAACGAAGTGAATCGCGAAGCGAT

>NL01B.C01.04_NL_12

CATGAACTCATCTTCCTTCAGGGACTTCGCTGACAAGG

>NL01B.C01.04_NL_13

CCTAATTTTGTTGGTGTCCCACCCATAGGGACATGTGACA

>NL01B.C01.04_NL_14

TAGTCACTTGCGGTGATTTCAGACCCATCATTCAG

>NL01B.C01.04_NL_15

CTATTACGGTGGCGAGTCAAGTAACGGAAAACCTGCC

>NL01B.C01.04_NL_16

TTAAACCCAGTCCAACGTTTCTCAGACCTGCCCTAAGG

>NL01B.C01.04_NL_17

TCTGATACTGTTAAGAGCGATGTAACTGAATCCCCAG

>NL01B.C01.04_NL_18

CACTAACTTATATCAATATGAATTAGTGGGACAATAT

>NL01B.C01.04_NL_19

TACGACATAAACGAAAAATTAGACGATATTCAGCGTC

>NL01B.C01.04_NL_20

GCTTGATCCTCTCTAAGTCTTCAATTTCACCCCTCTC

>NL01B.C01.04_NL_21

TACTTCTATTTTCTATTTATGTTATGGACGCAGTAA

>NL01B.C01.04_NL_22

ATTTTTGAGAAGTATAGCCTCTCGTTTTTGAAAATGT

>NL01B.C01.04_NL_23

TCAGTAGTTTCACTTCCTAAATCTTTAGCGAGAGGGTT

>NL01B.C01.04_NL_24

GCGGCAGCTTCCCTGGCTTGCTTCTGTTCCGGCGTAG

>NL01B.C01.04_NL_25

TACCTCCTCCACTTTGACTTCTCCTACCGTTTTCACGT

>NL01B.C01.04_NL_26

TTTTGCATAATCCAATCAACTTCTCCTTGAGAAGGA

>NL01B.C01.04_NL_27

CACAAATTGCAAAATGGGTCGGTGCAAGGTCATTTA

>NL01B.C01.04_NL_28

ATTGATTCGACTGCTGAAATTGTAGATTTTGTCATT

>NL01B.C01.04_NL_29

TCAGTTAGATGTTTCTACGACTCCAAGTCCACTTTATT

>NL01B.C01.04_NL_30

TTATGAATGTCACGCGGTAGCCGTACTGGCGTTCCAAC

>NL01B.C01.04_NL_31

TAAATCGTTCTTCGCCACTGCATCACAGTACGCTAC

>NL01B.C01.04_NL_32

TCGTCCCATGAAGCAGGAGTCGAAGTCCCTCCCGCT

>NL01B.C01.04_NL_33

TACCTCCACCTAAGGTATGGTAAAGACGTTGATGAT

>NL01B.C01.04_NL_34

CTTCTGGTTCAATGCGGTACCTATATTGCGTAGATA

>NL01B.C01.04_NL_35

GTTTCTCTTCTTTTTCATAGCCTACGACATGCTTTG

>NL01B.C01.04_NL_36

ATTTCAGAAAATGACCTAACAGATACCCATTTCGCAA

>NL01B.C01.04_NL_37

GTTTGTTTTCTATATCTTTCACTTCGTTGCCTAAAGCGTCA

>NL01B.C01.04_NL_38

TCTTTTTCACTTCTTCTAATATTCCGGAACTCATGTT

>NL01B.C01.04_NL_39

TTTGAGACCCCACACAGTTCTCAAGTAATCAGGGGAAGG

>NL01B.C01.04_NL_40

TTTTTCGAGATCGTGACGGGCTGCGGTTGTCCGTTCT

>NL01B.C01.04_NL_41

AATATTCTATTAAAGTATTTTTGATGTTTTTCGTAGAAAG

>NL01B.C01.04_NL_42

TATTTGATGAGGCTCATAACCTCGACGACTTCAACA

>NL01B.C01.04_NL_43

TTCTGAATATTTCTCGTTTAACTGGGGAGTTAAATCAT

>NL01B.C01.04_NL_44

TCGAGTTCTTGGGATGATGGTGGTGGCGGTGGAGGTACG

>NL01B.C01.04_NL_45

TAATGCAGATAATGGATTTGTCTCAATTGTTGATAA

>NL01B.C01.04_NL_46

CCTCTAACTTTTCGTCGTCGTTTTTGAACCTTAAGA

>NL01B.C01.04_NL_47

TGAAAAGGCATAAATGGCTGGTATATTAGCGATATT

>NL01B.C01.04_NL_48

CTGATCCTTGTTGGTTATTGATAGTCCAAGATAAAGA

>NL01B.C01.04_NL_49

AAAAACTTTTCGGATGCATTTCTAAGCATTTGGCACGT

>NL01B.C01.04_NL_50

ATAATTTAGTAGCTTCACTTAATATTCCTTCAAACGTT

>NL01B.C01.04_NL_51

TTACCTGGGGGAACTGAAATATTTTCAAGTGGAGTTGG

>NL01B.C01.04_NL_52

TCTTATAAAAATCCAGATAATGAATTTGAGCCTATT

>NL01B.C01.04_NL_53

ACATTTAATGGTTGTTTTAGGAGTAACGAGGGCCAACT

>NL01B.C01.04_NL_54

TCCGCACCTACGACCAGAACTACGATTGCCACTATTATG

>NL01B.C01.04_NL_55

TTTATGACAGCTCCCTTAGACCTCCTGCTAAAATAC

>NL01B.C01.04_NL_56

TTTCAAGTCCTCTAAGGATTTCTACAAATGCATGC

>NL01B.C01.04_NL_57

GATATTATAAGTTTCAAGTCCTCTAAGGATTTCTACA

>NL01B.C01.04_NL_58

CAAGTCCTCTAAGGATTTCTCCAAATCGTACC

>NL01B.C01.04_NL_59

TTTCAAGTCCTCTAAGGATTTAGCGAGAGGGTT

>NL01B.C01.04_NL_60

CCTAATTTTGTTGGTGGCGGTGGAGGTACG

>NL01B.C01.04_NL_61

TACTGAGTTTCAAGTCCTCTAAGGATTTCTACAAA

>NL01B.C01.04_NL_62

TCGAGTTCTTGGGACGATTTCAAGTCCTCTAAGGATTTCTACAAA

>NL01B.C01.04_NL_63

TTCAAGTCCTCTAAGGATTTCTACAAATCGTACC

>NL01B.C01.04_NL_64

TCTGATACTGTTAAGGCTGAACGAAGTGAATCGCGAAGCGAT

>NL01B.C01.04_NL_65

AGTCCTCTAAGGATTTCTACAAATATGAT

>NL01B.C01.04_NL_66

TTTCAAGTCCTCTAAGGATTTCTACCAACGTT

>NL01B.C01.04_NL_67

TATTTGATGTTTCAAGTCCTCTAAGGATTTCTACAAA

>NL01B.C01.04_NL_68

TCCTCTAAGGATTTCTACAAATCAAAGCATG

>NL01B.C01.04_NL_69

AACAGTATCAGATTTCAAGTCCTCTAAGGATTTC

>NL01B.C01.05_NL_0

TACAGTATACTCAAACGAAGGACGCCGTCGATATT

>NL01B.C01.05_NL_1

TAAACGATGCCTATACATTTTTCCAAGATGTCGTTAAT

>NL01B.C01.05_NL_2

TTGGATCTAATTCAGTTAGCACTGGCTGACCAGAAACGGT

>NL01B.C01.05_NL_3

TTTTATAAAAACCGTGAAAGAAGACCTAGAATCTCAAAA

>NL01B.C01.05_NL_4

TACTGCTGTGGCATAGCGTTCAAACTTCAGGTTAGTGTA

>NL01B.C01.05_NL_5

TGATGAATTGAAAGTACATGCTTGATTTCAAATTTCAA

>NL01B.C01.05_NL_6

TATGAAGACCGAATAGTACTGCTAATAACATCATAAC

>NL01B.C01.05_NL_7

TGCGATATTAATAGCTAGCATGATAGCTGCACTAAT

>NL01B.C01.05_NL_8

CTTGTTAGCTTAGGAATTAATAGCTGTAATTGTTTA

>NL01B.C01.05_NL_9

ATATCAAACAAACTAGTATTTGATAGTCGGTGGGAAA

>NL01B.C01.05_NL_10

GGAATACTATGACAATGATCAAGATGATGATTATCG

>NL01B.C01.05_NL_11

ATATTATTATCTCGATCCTTGTAGGCGGGTTCATGT

>NL01B.C01.05_NL_12

TGGATTTAGTAATGAAGGGGAATATGTTTCACGTGAT

>NL01B.C01.05_NL_13

ATAGGCTCGATGCAAATCCCTCACTCACTCATTAC

>NL01B.C01.05_NL_14

TACTTCTATTTTCTATTTATGTTATGGACGCAGTAA

>NL01B.C01.05_NL_15

TGGAATGAAGCTTTGCGTTGTTGAAACGCTATACTG

>NL01B.C01.05_NL_16

CCCTCGGAAATTAACTGGTTTATCTGTTGTGCGATAG

>NL01B.C01.05_NL_17

TCAGTAGTTTCACTTCCTAAATCTTTAGCGAGAGGGTT

>NL01B.C01.05_NL_18

TGATGATACTATATGAAATTAGGGAAAACGAGAAAG

>NL01B.C01.05_NL_19

TTTTGCGGAATAGTTGTAAATCAAGTACGCCGTCACACT

>NL01B.C01.05_NL_20

TAGAGAATTGGAAAAAGAAAAGTTTAATCAAAATGT

>NL01B.C01.05_NL_21

CTCCACACAATTTAGCAGCTTTAAATTCACAGAAAGGG

>NL01B.C01.05_NL_22

TTTTCTTGCTCAAACTGTTGTTCGTTCACATCCTC

>NL01B.C01.05_NL_23

ACTTTTGTTTTGTGTTTTTTGATTTCTCATTCTTTT

>NL01B.C01.05_NL_24

TAATTTTTGATATACTGAATCAACTTGTTTCACATAAGGCTGAGGG

>NL01B.C01.05_NL_25

AGACTTGAAATGATTGCATTAAAATCAGCAATTATTGATA

>NL01B.C01.05_NL_26

TAATTAAAAAGTTCTAAATTTGACCTCATAATGTAGTTC

>NL01B.C01.05_NL_27

TCTTTTGAGAAAAAAGAAAAAGAAGCCTGTTTATACT

>NL01B.C01.05_NL_28

TAACATGTCAGCACTTCATGACGTAAACAGTCTGGCA

>NL01B.C01.05_NL_29

TTTTTCGAGATCGTGACGGGCTGCGGTTGTCCGTTCT

>NL01B.C01.05_NL_30

TTCTATTTCCTTCTTTGAAAACTCTCGCTAGAGAA

>NL01B.C01.05_NL_31

TACTGAGTTTCAAGTCCTCTAAGGATTTCTACAAA

>NL01B.C01.05_NL_32

TTGAGTAATCCCGAGAAAGCATTCCGCTCGCCTAGTTC

>NL01B.C01.05_NL_33

TTGAGAACGGACAACCCATGGTAACAAACGACCCGATAACG

>NL01B.C01.05_NL_34

CATTGTAACATCCAAATACGGGCCTAAGGAATATG

>NL01B.C01.05_NL_35

TATTAAGGAGAAAAAGCCAGAAGTGATAGCAGTAGT

>NL01B.C01.05_NL_36

TAACCACCAAAAAGTGTATTTTAGAGATATCCAGA

>NL01B.C01.05_NL_37

TCATCGTCTCTAATAAAAAACATGATTAGTAGTTTG

>NL01B.C01.05_NL_38

AATACATCAAGTATAGCATTTTCTAAATAACTAA

>NL01B.C01.05_NL_39

TTACCTGGGGGAACTGAAATATTTTCAAGTGGAGTTGG

>NL01B.C01.05_NL_40

TAAAGGTGCAAAAAAAGTAATTGGATATGAGAAGAGTGG

>NL01B.C01.05_NL_41

TACTTCTTGACTACCTCTCTAACGTCAACCCACTGTAT

>NL01B.C01.05_NL_42

TCTTATAAAAATCCAGATAATGAATTTGAGCCTATT

>NL01B.C01.05_NL_43

ACCAGGCAGTCTCGCAGTTCAACAAGGCGGGAGGGC

>NL01B.C01.05_NL_44

TAAATCTAAATAAGGTCTCCCAAAATTACTTAAGGCACGTGAAA

>NL01B.C01.05_NL_45

TACAGTATACTCAAACGAAGCCTGTTTATACT

>NL01B.C01.05_NL_46

TTGGATCTAATTCAGTTAGCACTGGCTGACCCGATAACG

>NL01B.C01.05_NL_47

ACTTTTGTTCGTTCACATCCTC

>NL01B.C01.05_NL_48

GAATCTTTTGAGAAAAAAGAAAAAGAAGCCTGTTTATACT

>NL01B.C01.05_NL_49

TTTTGCGGAATAGCTGTAATTGTTTA

>NL01B.C01.05_NL_50

TCTTATAAAAATCCAGATACGGGCCTAAGGAATATG

>NL01B.C01.05_NL_51

CCTCTAAGGATTTCTACAAATATTAGTGC

>NL01B.C01.05_NL_52

ACCAGGCAGTCTCGCAGTTCAACAAGGCACGTGAAA

>NL01B.C01.05_NL_53

TACAGTATACTCAAACGAAGTACGCCGTCACACT

>NL01B.C01.05_NL_54

GAACAATACATCAAGTATAGCATTTTCTAAATAACTAA

>NL01B.C01.05_NL_55

GAACACCAGGCAGTCTCGCAGTTCAACAAGGCGGGAGGGC

>NL01B.C01.05_NL_56

CATTGTAACATCCAAATACGGGCCGATAACG

>NL01B.C01.05_NL_57

TATGTTTCAAGTCCTCTAAGGATTTCTA

>NL01B.C01.05_NL_58

CATTGTAACATCCAAATACGGGCTGCGGTTGTCCGTTCT

>NL01B.C01.05_NL_59

CCCCCTCGGAAATTAACTGGTTTATCTGTTGTGCGATAG

>NL01B.C01.05_NL_60

ACTTTTGTTTTGTGTTTATACT

>NL01B.C01.05_NL_61

TCAAGTCCTCTAAGGATTTCTACAAA

>NL01B.C01.08_NL_0

TTTTGAGATTCTAGGTCTTCTTTCACGGTTTTTATAAA

>NL01B.C01.08_NL_1

TCTCAAAATTAAGAATGCGTTAAAAGATGCAGTAAAA

>NL01B.C01.08_NL_2

TATTACCAGCACTGTTCTCAGTGAGGATTACGGTTTCTG

>NL01B.C01.08_NL_3

ATTGATGGATAACTGGAGTTCCCATAGCCATGCTTT

>NL01B.C01.08_NL_4

TGGGCACTATACTGTCATACCTAGAGGTCATGTATAACG

>NL01B.C01.08_NL_5

TGCGAGGAAATTCAGTACAGTGATGGAAGCATGGAAAG

>NL01B.C01.08_NL_6

CATCCGAGAATATTCTGGAAAGTGCCATTTGAAATGC

>NL01B.C01.08_NL_7

TGTCTTCCCTCAACCTCTACGGAACGAATAAAAAAA

>NL01B.C01.08_NL_8

TTTCATTAGCCCAGTCGTAATAAAGCCAATAGCAAA

>NL01B.C01.08_NL_9

CGATGCGATTAGAAGACTATCAGAGCTATTATAAAGTA

>NL01B.C01.08_NL_10

CTTGAAGTTAAGGCTGAACGAAGTGAATCGCGAAGCGAT

>NL01B.C01.08_NL_11

TTATAATATCTATACCAGAAGCATAGGAAGGAGACA

>NL01B.C01.08_NL_12

CATGAACTCATCTTCCTTCAGGGACTTCGCTGACAAGG

>NL01B.C01.08_NL_13

CCTAATTTTGTTGGTGTCCCACCCATAGGGACATGTGACA

>NL01B.C01.08_NL_14

TAGTCACTTGCGGTGATTTCAGACCCATCATTCAG

>NL01B.C01.08_NL_15

CTATTACGGTGGCGAGTCAAGTAACGGAAAACCTGCC

>NL01B.C01.08_NL_16

TTAAACCCAGTCCAACGTTTCTCAGACCTGCCCTAAGG

>NL01B.C01.08_NL_17

CACTAACTTATATCAATATGAATTAGTGGGACAATAT

>NL01B.C01.08_NL_18

TACGACATAAACGAAAAATTAGACGATATTCAGCGTC

>NL01B.C01.08_NL_19

GCTTGATCCTCTCTAAGTCTTCAATTTCACCCCTCTC

>NL01B.C01.08_NL_20

TCTGATACTGTTAAGAGCGATGTAACTGAATCCCCAG

>NL01B.C01.08_NL_21

TACTTCTATTTTCTATTTATGTTATGGACGCAGTAA

>NL01B.C01.08_NL_22

ATTTTTGAGAAGTATAGCCTCTCGTTTTTGAAAATGT

>NL01B.C01.08_NL_23

TCAGTAGTTTCACTTCCTAAATCTTTAGCGAGAGGGTT

>NL01B.C01.08_NL_24

GCGGCAGCTTCCCTGGCTTGCTTCTGTTCCGGCGTAG

>NL01B.C01.08_NL_25

TACCTCCTCCACTTTGACTTCTCCTACCGTTTTCACGT

>NL01B.C01.08_NL_26

TTTTGCATAATCCAATCAACTTCTCCTTGAGAAGGA

>NL01B.C01.08_NL_27

CACAAATTGCAAAATGGGTCGGTGCAAGGTCATTTA

>NL01B.C01.08_NL_28

ATTGATTCGACTGCTGAAATTGTAGATTTTGTCATT

>NL01B.C01.08_NL_29

TTATGAATGTCACGCGGTAGCCGTACTGGCGTTCCAAC

>NL01B.C01.08_NL_30

TCAGTTAGATGTTTCTACGACTCCAAGTCCACTTTATT

>NL01B.C01.08_NL_31

TAAATCGTTCTTCGCCACTGCATCACAGTACGCTAC

>NL01B.C01.08_NL_32

TCGTCCCATGAAGCAGGAGTCGAAGTCCCTCCCGCT

>NL01B.C01.08_NL_33

CTTCTGGTTCAATGCGGTACCTATATTGCGTAGATA

>NL01B.C01.08_NL_34

TACCTCCACCTAAGGTATGGTAAAGACGTTGATGAT

>NL01B.C01.08_NL_35

GTTTCTCTTCTTTTTCATAGCCTACGACATGCTTTG

>NL01B.C01.08_NL_36

ATTTCAGAAAATGACCTAACAGATACCCATTTCGCAA

>NL01B.C01.08_NL_37

GTTTGTTTTCTATATCTTTCACTTCGTTGCCTAAAGCGTCA

>NL01B.C01.08_NL_38

TCTTTTTCACTTCTTCTAATATTCCGGAACTCATGTT

>NL01B.C01.08_NL_39

TTTGAGACCCCACACAGTTCTCAAGTAATCAGGGGAAGG

>NL01B.C01.08_NL_40

TTTTTCGAGATCGTGACGGGCTGCGGTTGTCCGTTCT

>NL01B.C01.08_NL_41

AATATTCTATTAAAGTATTTTTGATGTTTTTCGTAGAAAG

>NL01B.C01.08_NL_42

TATTTGATGAGGCTCATAACCTCGACGACTTCAACA

>NL01B.C01.08_NL_43

TTCTGAATATTTCTCGTTTAACTGGGGAGTTAAATCAT

>NL01B.C01.08_NL_44

TCGAGTTCTTGGGATGATGGTGGTGGCGGTGGAGGTACG

>NL01B.C01.08_NL_45

TAATGCAGATAATGGATTTGTCTCAATTGTTGATAA

>NL01B.C01.08_NL_46

CCTCTAACTTTTCGTCGTCGTTTTTGAACCTTAAGA

>NL01B.C01.08_NL_47

TGAAAAGGCATAAATGGCTGGTATATTAGCGATATT

>NL01B.C01.08_NL_48

CTGATCCTTGTTGGTTATTGATAGTCCAAGATAAAGA

>NL01B.C01.08_NL_49

AAAAACTTTTCGGATGCATTTCTAAGCATTTGGCACGT

>NL01B.C01.08_NL_50

ATAATTTAGTAGCTTCACTTAATATTCCTTCAAACGTT

>NL01B.C01.08_NL_51

TTACCTGGGGGAACTGAAATATTTTCAAGTGGAGTTGG

>NL01B.C01.08_NL_52

TCTTATAAAAATCCAGATAATGAATTTGAGCCTATT

>NL01B.C01.08_NL_53

ACATTTAATGGTTGTTTTAGGAGTAACGAGGGCCAACT

>NL01B.C01.08_NL_54

TCCGCACCTACGACCAGAACTACGATTGCCACTATTATG

>NL01B.C01.08_NL_55

TTTATGACAGCTCCCTTAGACCTCCTGCTAAAATAC

>NL01B.C01.08_NL_56

TTACCTGGGGGAACTGAAATTGTAGATTTTGTCATT

>NL01B.C01.08_NL_57

TTGAAATAAATCGTTCTTCGCCACTGCATCACAGTACGCTAC

>NL01B.C01.08_NL_58

GAAACTTTCATTAGCCCAGTCGTAATAAAGCCAATAGCAAA

>NL01B.C01.08_NL_59

ATTGATGGATAACTGGGGTTGTCTCAATTGTTGATAA

>NL01B.C01.08_NL_60

TTTCAAGTCCTCTAAGGATTTCTACAAA

>NL01B.C01.08_NL_61

GATCGAGTTCTTGGGATGATGGTGGTGGCGGTGGAGGTACG

>NL01B.C01.08_NL_62

TCTAAGGATTTCTACAAATTTGC

>NL01B.C01.08_NL_63

TACCTCCTCCACTTTGACTTCTCCTTGAGAAGGA

>NL01B.C01.08_NL_64

GGATGATGGTGGTGGCGGTGGAGGTACG

>NL01B.C01.08_NL_65

TTTCAAGTCCTCTAAGGATTTCTACAAATATATTGTCCC

>NL01B.C01.08_NL_66

TTAAACCCCACACAGTTCTCAAGTAATCAGGGGAAGG

>NL01B.C01.08_NL_67

TTTCATTAGCCCAGTCGAAGTCCCTCCCGCT

>NL01B.C01.08_NL_68

GTTGGACTGGGTTTAAGTTTCAAGTCCTCTAAGGATTTCTAC

>NL01B.C01.08_NL_69

TGTCTTCCCTCAACCTCTAAGGATTTCTACAAA

>NL01B.C01.08_NL_70

TTTCAAGTCCTCTAAGGATTTGGCACGT

>NL01B.C01.08_NL_71

AGTGAAACATTGATGGATAACTGGAGTTCCCATAGCCATGCTTT

>NL01B.C01.08_NL_72

TACGACATAAACGAAAAATTTCAAGTCCTCTAAGGATTTCTACAAA

>NL01B.C01.10_NL_0

TTTTGAGATTCTAGGTCTTCTTTCACGGTTTTTATAAA

>NL01B.C01.10_NL_1

TCTCAAAATTAAGAATGCGTTAAAAGATGCAGTAAAA

>NL01B.C01.10_NL_2

TATTACCAGCACTGTTCTCAGTGAGGATTACGGTTTCTG

>NL01B.C01.10_NL_3

ATTGATGGATAACTGGAGTTCCCATAGCCATGCTTT

>NL01B.C01.10_NL_4

TGGGCACTATACTGTCATACCTAGAGGTCATGTATAACG

>NL01B.C01.10_NL_5

TGCGAGGAAATTCAGTACAGTGATGGAAGCATGGAAAG

>NL01B.C01.10_NL_6

CATCCGAGAATATTCTGGAAAGTGCCATTTGAAATGC

>NL01B.C01.10_NL_7

TGTCTTCCCTCAACCTCTACGGAACGAATAAAAAAA

>NL01B.C01.10_NL_8

TTTCATTAGCCCAGTCGTAATAAAGCCAATAGCAAA

>NL01B.C01.10_NL_9

CGATGCGATTAGAAGACTATCAGAGCTATTATAAAGTA

>NL01B.C01.10_NL_10

TTATAATATCTATACCAGAAGCATAGGAAGGAGACA

>NL01B.C01.10_NL_11

CTTGAAGTTAAGGCTGAACGAAGTGAATCGCGAAGCGAT

>NL01B.C01.10_NL_12

CATGAACTCATCTTCCTTCAGGGACTTCGCTGACAAGG

>NL01B.C01.10_NL_13

CCTAATTTTGTTGGTGTCCCACCCATAGGGACATGTGACA

>NL01B.C01.10_NL_14

TAGTCACTTGCGGTGATTTCAGACCCATCATTCAG

>NL01B.C01.10_NL_15

CTATTACGGTGGCGAGTCAAGTAACGGAAAACCTGCC

>NL01B.C01.10_NL_16

TTAAACCCAGTCCAACGTTTCTCAGACCTGCCCTAAGG

>NL01B.C01.10_NL_17

TCTGATACTGTTAAGAGCGATGTAACTGAATCCCCAG

>NL01B.C01.10_NL_18

CACTAACTTATATCAATATGAATTAGTGGGACAATAT

>NL01B.C01.10_NL_19

TACGACATAAACGAAAAATTAGACGATATTCAGCGTC

>NL01B.C01.10_NL_20

GCTTGATCCTCTCTAAGTCTTCAATTTCACCCCTCTC

>NL01B.C01.10_NL_21

TACTTCTATTTTCTATTTATGTTATGGACGCAGTAA

>NL01B.C01.10_NL_22

ATTTTTGAGAAGTATAGCCTCTCGTTTTTGAAAATGT

>NL01B.C01.10_NL_23

TCAGTAGTTTCACTTCCTAAATCTTTAGCGAGAGGGTT

>NL01B.C01.10_NL_24

GCGGCAGCTTCCCTGGCTTGCTTCTGTTCCGGCGTAG

>NL01B.C01.10_NL_25

TACCTCCTCCACTTTGACTTCTCCTACCGTTTTCACGT

>NL01B.C01.10_NL_26

TTTTGCATAATCCAATCAACTTCTCCTTGAGAAGGA

>NL01B.C01.10_NL_27

CACAAATTGCAAAATGGGTCGGTGCAAGGTCATTTA

>NL01B.C01.10_NL_28

ATTGATTCGACTGCTGAAATTGTAGATTTTGTCATT

>NL01B.C01.10_NL_29

TTATGAATGTCACGCGGTAGCCGTACTGGCGTTCCAAC

>NL01B.C01.10_NL_30

TCAGTTAGATGTTTCTACGACTCCAAGTCCACTTTATT

>NL01B.C01.10_NL_31

TAAATCGTTCTTCGCCACTGCATCACAGTACGCTAC

>NL01B.C01.10_NL_32

TCGTCCCATGAAGCAGGAGTCGAAGTCCCTCCCGCT

>NL01B.C01.10_NL_33

TACCTCCACCTAAGGTATGGTAAAGACGTTGATGAT

>NL01B.C01.10_NL_34

CTTCTGGTTCAATGCGGTACCTATATTGCGTAGATA

>NL01B.C01.10_NL_35

GTTTCTCTTCTTTTTCATAGCCTACGACATGCTTTG

>NL01B.C01.10_NL_36

ATTTCAGAAAATGACCTAACAGATACCCATTTCGCAA

>NL01B.C01.10_NL_37

GTTTGTTTTCTATATCTTTCACTTCGTTGCCTAAAGCGTCA

>NL01B.C01.10_NL_38

TCTTTTTCACTTCTTCTAATATTCCGGAACTCATGTT

>NL01B.C01.10_NL_39

TTTGAGACCCCACACAGTTCTCAAGTAATCAGGGGAAGG

>NL01B.C01.10_NL_40

TTTTTCGAGATCGTGACGGGCTGCGGTTGTCCGTTCT

>NL01B.C01.10_NL_41

AATATTCTATTAAAGTATTTTTGATGTTTTTCGTAGAAAG

>NL01B.C01.10_NL_42

TATTTGATGAGGCTCATAACCTCGACGACTTCAACA

>NL01B.C01.10_NL_43

TTCTGAATATTTCTCGTTTAACTGGGGAGTTAAATCAT

>NL01B.C01.10_NL_44

TCGAGTTCTTGGGATGATGGTGGTGGCGGTGGAGGTACG

>NL01B.C01.10_NL_45

TAATGCAGATAATGGATTTGTCTCAATTGTTGATAA

>NL01B.C01.10_NL_46

CCTCTAACTTTTCGTCGTCGTTTTTGAACCTTAAGA

>NL01B.C01.10_NL_47

TGAAAAGGCATAAATGGCTGGTATATTAGCGATATT

>NL01B.C01.10_NL_48

CTGATCCTTGTTGGTTATTGATAGTCCAAGATAAAGA

>NL01B.C01.10_NL_49

AAAAACTTTTCGGATGCATTTCTAAGCATTTGGCACGT

>NL01B.C01.10_NL_50

ATAATTTAGTAGCTTCACTTAATATTCCTTCAAACGTT

>NL01B.C01.10_NL_51

TTACCTGGGGGAACTGAAATATTTTCAAGTGGAGTTGG

>NL01B.C01.10_NL_52

TCTTATAAAAATCCAGATAATGAATTTGAGCCTATT

>NL01B.C01.10_NL_53

ACATTTAATGGTTGTTTTAGGAGTAACGAGGGCCAACT

>NL01B.C01.10_NL_54

TCCGCACCTACGACCAGAACTACGATTGCCACTATTATG

>NL01B.C01.10_NL_55

TTTATGACAGCTCCCTTAGACCTCCTGCTAAAATAC

>NL01B.C01.10_NL_56

TTTCAAGTCCTCTAAGGATTTCTACAAA

>NL01B.C01.10_NL_57

TTTCAAGTCCTCTAAGGATTTGGCACGT

>NL01B.C01.11_NL_0

TTTTGAGATTCTAGGTCTTCTTTCACGGTTTTTATAAA

>NL01B.C01.11_NL_1

TCTCAAAATTAAGAATGCGTTAAAAGATGCAGTAAAA

>NL01B.C01.11_NL_2

TATTACCAGCACTGTTCTCAGTGAGGATTACGGTTTCTG

>NL01B.C01.11_NL_3

ATTGATGGATAACTGGAGTTCCCATAGCCATGCTTT

>NL01B.C01.11_NL_4

TGGGCACTATACTGTCATACCTAGAGGTCATGTATAACG

>NL01B.C01.11_NL_5

TGCGAGGAAATTCAGTACAGTGATGGAAGCATGGAAAG

>NL01B.C01.11_NL_6

CATCCGAGAATATTCTGGAAAGTGCCATTTGAAATGC

>NL01B.C01.11_NL_7

TGTCTTCCCTCAACCTCTACGGAACGAATAAAAAAA

>NL01B.C01.11_NL_8

TTTCATTAGCCCAGTCGTAATAAAGCCAATAGCAAA

>NL01B.C01.11_NL_9

CGATGCGATTAGAAGACTATCAGAGCTATTATAAAGTA

>NL01B.C01.11_NL_10

TTATAATATCTATACCAGAAGCATAGGAAGGAGACA

>NL01B.C01.11_NL_11

CTTGAAGTTAAGGCTGAACGAAGTGAATCGCGAAGCGAT

>NL01B.C01.11_NL_12

CATGAACTCATCTTCCTTCAGGGACTTCGCTGACAAGG

>NL01B.C01.11_NL_13

CAACCCCCTCGTCTGGTTCATAAGTAACGCTGTCAA

>NL01B.C01.11_NL_14

CCTAATTTTGTTGGTGTCCCACCCATAGGGACATGTGACA

>NL01B.C01.11_NL_15

TAGTCACTTGCGGTGATTTCAGACCCATCATTCAG

>NL01B.C01.11_NL_16

CTATTACGGTGGCGAGTCAAGTAACGGAAAACCTGCC

>NL01B.C01.11_NL_17

TTAAACCCAGTCCAACGTTTCTCAGACCTGCCCTAAGG

>NL01B.C01.11_NL_18

TCTGATACTGTTAAGAGCGATGTAACTGAATCCCCAG

>NL01B.C01.11_NL_19

CACTAACTTATATCAATATGAATTAGTGGGACAATAT

>NL01B.C01.11_NL_20

TACGACATAAACGAAAAATTAGACGATATTCAGCGTC

>NL01B.C01.11_NL_21

GCTTGATCCTCTCTAAGTCTTCAATTTCACCCCTCTC

>NL01B.C01.11_NL_22

TACTTCTATTTTCTATTTATGTTATGGACGCAGTAA

>NL01B.C01.11_NL_23

ATTTTTGAGAAGTATAGCCTCTCGTTTTTGAAAATGT

>NL01B.C01.11_NL_24

TCAGTAGTTTCACTTCCTAAATCTTTAGCGAGAGGGTT

>NL01B.C01.11_NL_25

GCGGCAGCTTCCCTGGCTTGCTTCTGTTCCGGCGTAG

>NL01B.C01.11_NL_26

TACCTCCTCCACTTTGACTTCTCCTACCGTTTTCACGT

>NL01B.C01.11_NL_27

TTTTGCATAATCCAATCAACTTCTCCTTGAGAAGGA

>NL01B.C01.11_NL_28

CACAAATTGCAAAATGGGTCGGTGCAAGGTCATTTA

>NL01B.C01.11_NL_29

ATTGATTCGACTGCTGAAATTGTAGATTTTGTCATT

>NL01B.C01.11_NL_30

TCAGTTAGATGTTTCTACGACTCCAAGTCCACTTTATT

>NL01B.C01.11_NL_31

TTATGAATGTCACGCGGTAGCCGTACTGGCGTTCCAAC

>NL01B.C01.11_NL_32

TAAATCGTTCTTCGCCACTGCATCACAGTACGCTAC

>NL01B.C01.11_NL_33

TCGTCCCATGAAGCAGGAGTCGAAGTCCCTCCCGCT

>NL01B.C01.11_NL_34

TACCTCCACCTAAGGTATGGTAAAGACGTTGATGAT

>NL01B.C01.11_NL_35

CTTCTGGTTCAATGCGGTACCTATATTGCGTAGATA

>NL01B.C01.11_NL_36

GTTTCTCTTCTTTTTCATAGCCTACGACATGCTTTG

>NL01B.C01.11_NL_37

ATTTCAGAAAATGACCTAACAGATACCCATTTCGCAA

>NL01B.C01.11_NL_38

GTTTGTTTTCTATATCTTTCACTTCGTTGCCTAAAGCGTCA

>NL01B.C01.11_NL_39

TCTTTTTCACTTCTTCTAATATTCCGGAACTCATGTT

>NL01B.C01.11_NL_40

TTTGAGACCCCACACAGTTCTCAAGTAATCAGGGGAAGG

>NL01B.C01.11_NL_41

TTTTTCGAGATCGTGACGGGCTGCGGTTGTCCGTTCT

>NL01B.C01.11_NL_42

AATATTCTATTAAAGTATTTTTGATGTTTTTCGTAGAAAG

>NL01B.C01.11_NL_43

TATTTGATGAGGCTCATAACCTCGACGACTTCAACA

>NL01B.C01.11_NL_44

TTGTAGTAAAACATCACCGGAGTATCTATTTCCTTCAA

>NL01B.C01.11_NL_45

TTCTGAATATTTCTCGTTTAACTGGGGAGTTAAATCAT

>NL01B.C01.11_NL_46

TCGAGTTCTTGGGATGATGGTGGTGGCGGTGGAGGTACG

>NL01B.C01.11_NL_47

TAATGCAGATAATGGATTTGTCTCAATTGTTGATAA

>NL01B.C01.11_NL_48

CCTCTAACTTTTCGTCGTCGTTTTTGAACCTTAAGA

>NL01B.C01.11_NL_49

TGAAAAGGCATAAATGGCTGGTATATTAGCGATATT

>NL01B.C01.11_NL_50

CTGATCCTTGTTGGTTATTGATAGTCCAAGATAAAGA

>NL01B.C01.11_NL_51

AAAAACTTTTCGGATGCATTTCTAAGCATTTGGCACGT

>NL01B.C01.11_NL_52

ATAATTTAGTAGCTTCACTTAATATTCCTTCAAACGTT

>NL01B.C01.11_NL_53

TTACCTGGGGGAACTGAAATATTTTCAAGTGGAGTTGG

>NL01B.C01.11_NL_54

TCTTATAAAAATCCAGATAATGAATTTGAGCCTATT

>NL01B.C01.11_NL_55

ACATTTAATGGTTGTTTTAGGAGTAACGAGGGCCAACT

>NL01B.C01.11_NL_56

TCCGCACCTACGACCAGAACTACGATTGCCACTATTATG

>NL01B.C01.11_NL_57

TTTATGACAGCTCCCTTAGACCTCCTGCTAAAATAC

>NL01B.C01.11_NL_58

CCCTTGAAGTTAAGGCTGAACGAAGTGAATCGCGAAGCGAT

>NL01B.C01.11_NL_59

TAACTTAAACCCAGTCCAACGTTTCTCAGACCTGCCCTAAGG

>NL01B.C01.11_NL_60

TCTTATAAAAATCCAGATAATGATTTCAGACCCATCATTCAG

>NL01B.C01.11_NL_61

TTTCAAGTCCTCTAAGGATTTCTACAAATAACGTTTGAAGG

>NL01B.C01.11_NL_62

GAATTGTAGTAAAACATCACCGGAGTATCTATTTCCTTCAA

>NL01B.C01.11_NL_63

TTTCAAGTCCTCTAAGGATTTGGCACGT

>NL01B.C01.11_NL_64

GGTTGGTTTCAAGTCCTCTAAGGATTTCTAC

>NL01B.C01.11_NL_65

TTGAAATTCTGAATATTTCTCGTTTAACTGGGGAGTTAAATCAT

>NL01B.C01.12_NL_0

TTTTGAGATTCTAGGTCTTCTTTCACGGTTTTTATAAA

>NL01B.C01.12_NL_1

TCTCAAAATTAAGAATGCGTTAAAAGATGCAGTAAAA

>NL01B.C01.12_NL_2

TATTACCAGCACTGTTCTCAGTGAGGATTACGGTTTCTG

>NL01B.C01.12_NL_3

ATTGATGGATAACTGGAGTTCCCATAGCCATGCTTT

>NL01B.C01.12_NL_4

TGGGCACTATACTGTCATACCTAGAGGTCATGTATAACG

>NL01B.C01.12_NL_5

CATCCGAGAATATTCTGGAAAGTGCCATTTGAAATGC

>NL01B.C01.12_NL_6

TGTCTTCCCTCAACCTCTACGGAACGAATAAAAAAA

>NL01B.C01.12_NL_7

TTTCATTAGCCCAGTCGTAATAAAGCCAATAGCAAA

>NL01B.C01.12_NL_8

CGATGCGATTAGAAGACTATCAGAGCTATTATAAAGTA

>NL01B.C01.12_NL_9

TTATAATATCTATACCAGAAGCATAGGAAGGAGACA

>NL01B.C01.12_NL_10

CTTGAAGTTAAGGCTGAACGAAGTGAATCGCGAAGCGAT

>NL01B.C01.12_NL_11

CATGAACTCATCTTCCTTCAGGGACTTCGCTGACAAGG

>NL01B.C01.12_NL_12

CCTAATTTTGTTGGTGTCCCACCCATAGGGACATGTGACA

>NL01B.C01.12_NL_13

TAGTCACTTGCGGTGATTTCAGACCCATCATTCAG

>NL01B.C01.12_NL_14

CTATTACGGTGGCGAGTCAAGTAACGGAAAACCTGCC

>NL01B.C01.12_NL_15

TTAAACCCAGTCCAACGTTTCTCAGACCTGCCCTAAGG

>NL01B.C01.12_NL_16

CACTAACTTATATCAATATGAATTAGTGGGACAATAT

>NL01B.C01.12_NL_17

GCTTGATCCTCTCTAAGTCTTCAATTTCACCCCTCTC

>NL01B.C01.12_NL_18

TACTTCTATTTTCTATTTATGTTATGGACGCAGTAA

>NL01B.C01.12_NL_19

ATTTTTGAGAAGTATAGCCTCTCGTTTTTGAAAATGT

>NL01B.C01.12_NL_20

TCAGTAGTTTCACTTCCTAAATCTTTAGCGAGAGGGTT

>NL01B.C01.12_NL_21

GCGGCAGCTTCCCTGGCTTGCTTCTGTTCCGGCGTAG

>NL01B.C01.12_NL_22

TACCTCCTCCACTTTGACTTCTCCTACCGTTTTCACGT

>NL01B.C01.12_NL_23

TTTTGCATAATCCAATCAACTTCTCCTTGAGAAGGA

>NL01B.C01.12_NL_24

CACAAATTGCAAAATGGGTCGGTGCAAGGTCATTTA

>NL01B.C01.12_NL_25

ATTGATTCGACTGCTGAAATTGTAGATTTTGTCATT

>NL01B.C01.12_NL_26

TCGTCCCATGAAGCAGGAGTCGAAGTCCCTCCCGCT

>NL01B.C01.12_NL_27

CTTCTGGTTCAATGCGGTACCTATATTGCGTAGATA

>NL01B.C01.12_NL_28

ATTTCAGAAAATGACCTAACAGATACCCATTTCGCAA

>NL01B.C01.12_NL_29

GTTTGTTTTCTATATCTTTCACTTCGTTGCCTAAAGCGTCA

>NL01B.C01.12_NL_30

TCTTTTTCACTTCTTCTAATATTCCGGAACTCATGTT

>NL01B.C01.12_NL_31

TTTTTCGAGATCGTGACGGGCTGCGGTTGTCCGTTCT

>NL01B.C01.12_NL_32

AATATTCTATTAAAGTATTTTTGATGTTTTTCGTAGAAAG

>NL01B.C01.12_NL_33

TATTTGATGAGGCTCATAACCTCGACGACTTCAACA

>NL01B.C01.12_NL_34

TTCTGAATATTTCTCGTTTAACTGGGGAGTTAAATCAT

>NL01B.C01.12_NL_35

TAATGCAGATAATGGATTTGTCTCAATTGTTGATAA

>NL01B.C01.12_NL_36

CCTCTAACTTTTCGTCGTCGTTTTTGAACCTTAAGA

>NL01B.C01.12_NL_37

GCGGCAGCTTCCCTGGGGGAACTGAAATATTTTCAAGTGGAGTTGG

>NL01B.C01.12_NL_38

CTGATCCTTGTTGGTTATTGATAGTCCAAGATAAAGA

>NL01B.C01.12_NL_39

AAAAACTTTTCGGATGCATTTCTAAGCATTTGGCACGT

>NL01B.C01.12_NL_40

ATAATTTAGTAGCTTCACTTAATATTCCTTCAAACGTT

>NL01B.C01.12_NL_41

TTACCTGGGGGAACTGAAATATTTTCAAGTGGAGTTGG

>NL01B.C01.12_NL_42

TCTTATAAAAATCCAGATAATGAATTTGAGCCTATT

>NL01B.C01.12_NL_43

ACATTTAATGGTTGTTTTAGGAGTAACGAGGGCCAACT

>NL01B.C01.12_NL_44

TCCGCACCTACGACCAGAACTACGATTGCCACTATTATG

>NL01B.C01.12_NL_45

TTTATGACAGCTCCCTTAGACCTCCTGCTAAAATAC

>NL01B.C01.12_NL_46

TTTATGACAGCTCCCTTAGACCTGCCCTAAGG

>NL01B.C01.12_NL_47

TTTCATTAGCCCAGTCGTAATAAAGCAGGAGTCGAAGTCCCTCCCGCT

>NL01B.C01.12_NL_48

TTTCAAGTCCTCTAAGGATTTCTACAAA

>NL01B.C01.12_NL_49

GCCATCCGAGAATATTCTGGAAAGTGCCATTTGAAATGC

>NL01B.C01.12_NL_50

GTTTCTCTTCTTTTTCATAGCCTACGACATGCTTTG

>NL01B.C01.12_NL_51

TTATAATATCTATACCAGATAATGAATTTGAGCCTATT

>NL01B.C01.12_NL_52

TTTCAAGTCCTCTAAGGATTTGGCACGT

>NL01B.C01.13_NL_0

TACAGTATACTCAAACGAAGGACGCCGTCGATATT

>NL01B.C01.13_NL_1

TAAACGATGCCTATACATTTTTCCAAGATGTCGTTAAT

>NL01B.C01.13_NL_2

TTTTATAAAAACCGTGAAAGAAGACCTAGAATCTCAAAA

>NL01B.C01.13_NL_3

ATTGATGGATAACTGGAGTTCCCATAGCCATGCTTT

>NL01B.C01.13_NL_4

TTGGATCTAATTCAGTTAGCACTGGCTGACCAGAAACGGT

>NL01B.C01.13_NL_5

TACTGCTGTGGCATAGCGTTCAAACTTCAGGTTAGTGTA

>NL01B.C01.13_NL_6

TGATGAATTGAAAGTACATGCTTGATTTCAAATTTCAA

>NL01B.C01.13_NL_7

TGCGATATTAATAGCTAGCATGATAGCTGCACTAAT

>NL01B.C01.13_NL_8

TATGAAGACCGAATAGTACTGCTAATAACATCATAAC

>NL01B.C01.13_NL_9

CTTGTTAGCTTAGGAATTAATAGCTGTAATTGTTTA

>NL01B.C01.13_NL_10

ATATCAAACAAACTAGTATTTGATAGTCGGTGGGAAA

>NL01B.C01.13_NL_11

GGAATACTATGACAATGATCAAGATGATGATTATCG

>NL01B.C01.13_NL_12

ATATTATTATCTCGATCCTTGTAGGCGGGTTCATGT

>NL01B.C01.13_NL_13

TGGATTTAGTAATGAAGGGGAATATGTTTCACGTGAT

>NL01B.C01.13_NL_14

ATAGGCTCGATGCAAATCCCTCACTCACTCATTAC

>NL01B.C01.13_NL_15

TACTTCTATTTTCTATTTATGTTATGGACGCAGTAA

>NL01B.C01.13_NL_16

TGGAATGAAGCTTTGCGTTGTTGAAACGCTATACTG

>NL01B.C01.13_NL_17

CCCTCGGAAATTAACTGGTTTATCTGTTGTGCGATAG

>NL01B.C01.13_NL_18

TCAGTAGTTTCACTTCCTAAATCTTTAGCGAGAGGGTT

>NL01B.C01.13_NL_19

TGATGATACTATATGAAATTAGGGAAAACGAGAAAG

>NL01B.C01.13_NL_20

TTTTGCGGAATAGTTGTAAATCAAGTACGCCGTCACACT

>NL01B.C01.13_NL_21

TAGAGAATTGGAAAAAGAAAAGTTTAATCAAAATGT

>NL01B.C01.13_NL_22

CTCCACACAATTTAGCAGCTTTAAATTCACAGAAAGGG

>NL01B.C01.13_NL_23

TTTTCTTGCTCAAACTGTTGTTCGTTCACATCCTC

>NL01B.C01.13_NL_24

TAATTTTTGATATACTGAATCAACTTGTTTCACATAAGGCTGAGGG

>NL01B.C01.13_NL_25

AGACTTGAAATGATTGCATTAAAATCAGCAATTATTGATA

>NL01B.C01.13_NL_26

TAATTAAAAAGTTCTAAATTTGACCTCATAATGTAGTTC

>NL01B.C01.13_NL_27

TCTTTTGAGAAAAAAGAAAAAGAAGCCTGTTTATACT

>NL01B.C01.13_NL_28

TAACATGTCAGCACTTCATGACGTAAACAGTCTGGCA

>NL01B.C01.13_NL_29

TTTTTCGAGATCGTGACGGGCTGCGGTTGTCCGTTCT

>NL01B.C01.13_NL_30

TTCTATTTCCTTCTTTGAAAACTCTCGCTAGAGAA

>NL01B.C01.13_NL_31

TTGAGTAATCCCGAGAAAGCATTCCGCTCGCCTAGTTC

>NL01B.C01.13_NL_32

TTGAGAACGGACAACCCATGGTAACAAACGACCCGATAACG

>NL01B.C01.13_NL_33

TAAAGGTGCAAAAAAAGTAATTGGATATGAGAAGAGTGA

>NL01B.C01.13_NL_34

CATTGTAACATCCAAATACGGGCCTAAGGAATATG

>NL01B.C01.13_NL_35

CATCATGGCAATGGAACCTTTTGATTAAATCTAGT

>NL01B.C01.13_NL_36

TATTAAGGAGAAAAAGCCAGAAGTGATAGCAGTAGT

>NL01B.C01.13_NL_37

TAACCACCAAAAAGTGTATTTTAGAGATATCCAGA

>NL01B.C01.13_NL_38

TCATCGTCTCTAATAAAAAACATGATTAGTAGTTTG

>NL01B.C01.13_NL_39

AATACATCAAGTATAGCATTTTCTAAATAACTAA

>NL01B.C01.13_NL_40

TTACCTGGGGGAACTGAAATATTTTCAAGTGGAGTTGG

>NL01B.C01.13_NL_41

TACTTCTTGACTACCTCTCTAACGTCAACCCACTGTAT

>NL01B.C01.13_NL_42

TCTTATAAAAATCCAGATAATGAATTTGAGCCTATT

>NL01B.C01.13_NL_43

ACCAGGCAGTCTCGCAGTTCAACAAGGCGGGAGGGC

>NL01B.C01.13_NL_44

TAAATCTAAATAAGGTCTCCCAAAATTACTTAAGGCACGTGAAA

>NL01B.C01.13_NL_45

TACTTCTTGACGTAAACAGTCTGGCA

>NL01B.C01.13_NL_46

CTCTAAGGATTTCTACAAATTTCTCTAGCGAGAGGGTT

>NL01B.C01.13_NL_47

TGGAATGAAGCTTTGCGTTCACAGAAAGGG

>NL01B.C01.13_NL_48

TAAAGGTGCAAAAAAGTAATTGGATATGAGAAGAGTGA

>NL01B.C01.13_NL_49

TTACTTTTTTTGCACCTTTATTTCAAGTCCTCTAAGGATTTCTACAAA

>NL01B.C01.13_NL_50

TTTCAAGTCCTCTAAGGATTTCTACAAATAACCCTCTCGCTAGAGAA

>NL01B.C01.13_NL_51

ACCAGGCAGTCTCGCAGTTCATGACGTAAACAGTCTGGCA

>NL01B.C01.13_NL_52

TACTTCTTGACTACCTCTCTAACGTAAACAGTCTGGCA

>NL01B.C01.13_NL_53

CCCTCCACACAATTTAGCAGCTTTAAATTCACAGAAAGGG

>NL01B.C01.13_NL_54

TTTTTCGAGATCGTGACGTAAACAGTCTGGCA

>NL01B.C01.13_NL_55

TTAACTGGTTTATCTGTTGTGCGATAG

>NL01B.C01.13_NL_56

CAATCTTATAAAAATCCAGATAATGAATTTGAGCCTATT

>NL01B.C01.13_NL_57

TACTGAGTTTCAAGTCCTCTAAGGATTTCTACAAA

>NL01B.C01.13_NL_58

TACACCAGGCAGTCTCGCAGTTCAACAAGGCGGGAGGGC

>NL01B.C01.13_NL_59

TAAAGGTGCAAAAAAAGTAATTTCAAGTCCTCTAAGGATTTCTA

>NL01B.C01.13_NL_60

CAACTTGGATCTAATTCAGTTAGCACTGGCTGACCAGAAACGGT

>NL01B.C01.13_NL_61

TGTAGTTTCAAGTCCTCTAAGGATTTCTACAAA

>NL01B.C01.14_NL_0

TAATTTTTGATATACTGAATCAACTTGTTTCACATAAGGCTGAGGG

>NL01B.C01.18_NL_0

TACAGTATACTCAAACGAAGGACGCCGTCGATATT

>NL01B.C01.18_NL_1

TAAACGATGCCTATACATTTTTCCAAGATGTCGTTAAT

>NL01B.C01.18_NL_2

TTGGATCTAATTCAGTTAGCACTGGCTGACCAGAAACGGT

>NL01B.C01.18_NL_3

TACTGCTGTGGCATAGCGTTCAAACTTCAGGTTAGTGTA

>NL01B.C01.18_NL_4

TGTCTTCCCTCAACCTCTACGGAACGAATAAAAAAA

>NL01B.C01.18_NL_5

TATGAAGACCGAATAGTACTGCTAATAACATCATAAC

>NL01B.C01.18_NL_6

CTTGTTAGCTTAGGAATTAATAGCTGTAATTGTTTA

>NL01B.C01.18_NL_7

ATATCAAACAAACTAGTATTTGATAGTCGGTGGGAAA

>NL01B.C01.18_NL_8

GGAATACTATGACAATGATCAAGATGATGATTATCG

>NL01B.C01.18_NL_9

CACTAACTTATATCAATATGAATTAGTGGGACAATAT

>NL01B.C01.18_NL_10

ATAGGCTCGATGCAAATCCCTCACTCACTCATTAC

>NL01B.C01.18_NL_11

TACTTCTATTTTCTATTTATGTTATGGACGCAGTAA

>NL01B.C01.18_NL_12

ATTTTTGAGAAGTATAGCCTCTCGTTTTTGAAAATGT

>NL01B.C01.18_NL_13

CCCTCGGAAATTAACTGGTTTATCTGTTGTGCGATAG

>NL01B.C01.18_NL_14

TCAGTAGTTTCACTTCCTAAATCTTTAGCGAGAGGGTT

>NL01B.C01.18_NL_15

TGATGATACTATATGAAATTAGGGAAAACGAGAAAG

>NL01B.C01.18_NL_16

CTCCACACAATTTAGCAGCTTTAAATTCACAGAAAGGG

>NL01B.C01.18_NL_17

ACTTTTGTTTTGTGTTTTTTGATTTCTCATTCTTTT

>NL01B.C01.18_NL_18

TAATTTTTGATATACTGAATCAACTTGTTTCACATAAGGCTGAGGG

>NL01B.C01.18_NL_19

GTTTGTTTTCTATATCTTTCACTTCGTTGCCTAAAGCGTCA

>NL01B.C01.18_NL_20

TTTTTCGAGATCGTGACGGGCTGCGGTTGTCCGTTCT

>NL01B.C01.18_NL_21

TTCTATTTCCTTCTTTGAAAACTCTCGCTAGAGAA

>NL01B.C01.18_NL_22

CATTGTAACATCCAAATACGGGCCTAAGGAATATG

>NL01B.C01.18_NL_23

TAACCACCAAAAAGTGTATTTTAGAGATATCCAGA

>NL01B.C01.18_NL_24

AATACATCAAGTATAGCATTTTCTAAATAACTAA

>NL01B.C01.18_NL_25

TTACCTGGGGGAACTGAAATATTTTCAAGTGGAGTTGG

>NL01B.C01.18_NL_26

TAAAGGTGCAAAAAAAGTAATTGGATATGAGAAGAGTGG

>NL01B.C01.18_NL_27

TACTTCTTGACTACCTCTCTAACGTCAACCCACTGTAT

>NL01B.C01.18_NL_28

TCTTATAAAAATCCAGATAATGAATTTGAGCCTATT

>NL01B.C01.18_NL_29

ACCAGGCAGTCTCGCAGTTCAACAAGGCGGGAGGGC

>NL01B.C01.18_NL_30

TTTTGAGATTCTAGGTCTTCTTTCACGGTTTTTATAAA

>NL01B.C01.18_NL_31

TCTCAAAATTAAGAATGCGTTAAAAGATGCAGTAAAA

>NL01B.C01.18_NL_32

TCCTCTAAGGATTTCTACAAA

>NL01B.C01.18_NL_33

CATCCGAGAATATTCTGGAAAGTGCCATTTGAAATGC

>NL01B.C01.18_NL_34

TTTCAAGTCCTCTAAGGATTTCTACAAATTCACAGAAAGGG

>NL01B.C01.18_NL_35

CGATGCGATTAGAAGACTATCAGAGCTATTATAAAGTA

>NL01B.C01.18_NL_36

TTATAATATCTATACCAGAAGCATAGGAAGGAGACA

>NL01B.C01.18_NL_37

CATGAACTCATCTTCCTTCAGGGACTTCGCTGACAAGG

>NL01B.C01.18_NL_38

ATATTATTATCTCGATCCTTGTAGGCGGGTTCATGT

>NL01B.C01.18_NL_39

CACAAATTGCAAAATGGGTCGGTGCAAGGTCATTTA

>NL01B.C01.18_NL_40

AGACTTGAAATGATTGCATTAAAATCAGCAATTATTGATA

>NL01B.C01.18_NL_41

CTTCTGGTTCAATGCGGTACCTATATTGCGTAGATA

>NL01B.C01.18_NL_42

TCTTTTTCACTTCTTCTAATATTCCGGAACTCATGTT

>NL01B.C01.18_NL_43

CATTGTAACATCCAGATAATGACTTTGAGCCTATT

>NL01B.C01.18_NL_44

AAAATAAAGGTGCAAAAAAAGTAATTGGATATGAGAAGAGTGG

>NL01B.C01.18_NL_45

AATATTCTATTAAAGTATTTTTGATGTTTTTCGTAGAAAG

>NL01B.C01.18_NL_46

TACTGAGTTTCAAGTCCTCTAAGGATTTCTACAAA

>NL01B.C01.18_NL_47

TATTTGATGAGGCTCATAACCTCGACGACTTCAACA

>NL01B.C01.18_NL_48

CATTGTAACATCCAAATACGGGCTGCGGTTGTCCGTTCT

>NL01B.C01.18_NL_49

CCTCTAACTTTTCGTCGTCGTTTTTGAACCTTAAGA

>NL01B.C01.18_NL_50

TACTGAGTTTCAAGTCCTCTAAGGATTTCTACAA

>NL01B.C01.18_NL_51

CTGATCCTTGTTGGTTATTGATAGTCCAAGATAAAGA

>NL01B.C01.18_NL_52

ATAATTTAGTAGCTTCACTTAATATTCCTTCAAACGTT

>NL01B.C01.18_NL_53

TCCGCACCTACGACCAGAACTACGATTGCCACTATTATG

>NL01B.C01.18_NL_54

TTTATGACAGCTCCCTTAGACCTCCTGCTAAAATAC

>NL01B.C01.19_NL_0

TTTTGAGATTCTAGGTCTTCTTTCACGGTTTTTATAAA

>NL01B.C01.19_NL_1

TCTCAAAATTAAGAATGCGTTAAAAGATGCAGTAAAA

>NL01B.C01.19_NL_2

TATTACCAGCACTGTTCTCAGTGAGGATTACGGTTTCTG

>NL01B.C01.19_NL_3

ATTGATGGATAACTGGAGTTCCCATAGCCATGCTTT

>NL01B.C01.19_NL_4

TGGGCACTATACTGTCATACCTAGAGGTCATGTATAACG

>NL01B.C01.19_NL_5

TGCGAGGAAATTCAGTACAGTGATGGAAGCATGGAAAG

>NL01B.C01.19_NL_6

CATCCGAGAATATTCTGGAAAGTGCCATTTGAAATGC

>NL01B.C01.19_NL_7

TGTCTTCCCTCAACCTCTACGGAACGAATAAAAAAA

>NL01B.C01.19_NL_8

TTTCATTAGCCCAGTCGTAATAAAGCCAATAGCAAA

>NL01B.C01.19_NL_9

CGATGCGATTAGAAGACTATCAGAGCTATTATAAAGTA

>NL01B.C01.19_NL_10

CTTGAAGTTAAGGCTGAACGAAGTGAATCGCGAAGCGAT

>NL01B.C01.19_NL_11

TTATAATATCTATACCAGAAGCATAGGAAGGAGACA

>NL01B.C01.19_NL_12

CATGAACTCATCTTCCTTCAGGGACTTCGCTGACAAGG

>NL01B.C01.19_NL_13

CCTAATTTTGTTGGTGTCCCACCCATAGGGACATGTGACA

>NL01B.C01.19_NL_14

TAGTCACTTGCGGTGATTTCAGACCCATCATTCAG

>NL01B.C01.19_NL_15

CTATTACGGTGGCGAGTCAAGTAACGGAAAACCTGCC

>NL01B.C01.19_NL_16

TTAAACCCAGTCCAACGTTTCTCAGACCTGCCCTAAGG

>NL01B.C01.19_NL_17

CACTAACTTATATCAATATGAATTAGTGGGACAATAT

>NL01B.C01.19_NL_18

TACGACATAAACGAAAAATTAGACGATATTCAGCGTC

>NL01B.C01.19_NL_19

GCTTGATCCTCTCTAAGTCTTCAATTTCACCCCTCTC

>NL01B.C01.19_NL_20

TCTGATACTGTTAAGAGCGATGTAACTGAATCCCCAG

>NL01B.C01.19_NL_21

TACTTCTATTTTCTATTTATGTTATGGACGCAGTAA

>NL01B.C01.19_NL_22

ATTTTTGAGAAGTATAGCCTCTCGTTTTTGAAAATGT

>NL01B.C01.19_NL_23

TCAGTAGTTTCACTTCCTAAATCTTTAGCGAGAGGGTT

>NL01B.C01.19_NL_24

GCGGCAGCTTCCCTGGCTTGCTTCTGTTCCGGCGTAG

>NL01B.C01.19_NL_25

TACCTCCTCCACTTTGACTTCTCCTACCGTTTTCACGT

>NL01B.C01.19_NL_26

TTTTGCATAATCCAATCAACTTCTCCTTGAGAAGGA

>NL01B.C01.19_NL_27

CACAAATTGCAAAATGGGTCGGTGCAAGGTCATTTA

>NL01B.C01.19_NL_28

ATTGATTCGACTGCTGAAATTGTAGATTTTGTCATT

>NL01B.C01.19_NL_29

TTATGAATGTCACGCGGTAGCCGTACTGGCGTTCCAAC

>NL01B.C01.19_NL_30

TCAGTTAGATGTTTCTACGACTCCAAGTCCACTTTATT

>NL01B.C01.19_NL_31

TAAATCGTTCTTCGCCACTGCATCACAGTACGCTAC

>NL01B.C01.19_NL_32

TCGTCCCATGAAGCAGGAGTCGAAGTCCCTCCCGCT

>NL01B.C01.19_NL_33

TACCTCCACCTAAGGTATGGTAAAGACGTTGATGAT

>NL01B.C01.19_NL_34

GTTTCTCTTCTTTTTCATAGCCTACGACATGCTTTG

>NL01B.C01.19_NL_35

ATTTCAGAAAATGACCTAACAGATACCCATTTCGCAA

>NL01B.C01.19_NL_36

GTTTGTTTTCTATATCTTTCACTTCGTTGCCTAAAGCGTCA

>NL01B.C01.19_NL_37

TCTTTTTCACTTCTTCTAATATTCCGGAACTCATGTT

>NL01B.C01.19_NL_38

TTTGAGACCCCACACAGTTCTCAAGTAATCAGGGGAAGG

>NL01B.C01.19_NL_39

TTTTTCGAGATCGTGACGGGCTGCGGTTGTCCGTTCT

>NL01B.C01.19_NL_40

TATTTGATGAGGCTCATAACCTCGACGACTTCAACA

>NL01B.C01.19_NL_41

AATATTCTATTAAAGTATTTTTGATGTTTTTCGTAGAAAG

>NL01B.C01.19_NL_42

TTCTGAATATTTCTCGTTTAACTGGGGAGTTAAATCAT

>NL01B.C01.19_NL_43

TCGAGTTCTTGGGATGATGGTGGTGGCGGTGGAGGTACG

>NL01B.C01.19_NL_44

TAATGCAGATAATGGATTTGTCTCAATTGTTGATAA

>NL01B.C01.19_NL_45

TGAAAAGGCATAAATGGCTGGTATATTAGCGATATT

>NL01B.C01.19_NL_46

CTGATCCTTGTTGGTTATTGATAGTCCAAGATAAAGA

>NL01B.C01.19_NL_47

AAAAACTTTTCGGATGCATTTCTAAGCATTTGGCACGT

>NL01B.C01.19_NL_48

ATAATTTAGTAGCTTCACTTAATATTCCTTCAAACGTT

>NL01B.C01.19_NL_49

TTACCTGGGGGAACTGAAATATTTTCAAGTGGAGTTGG

>NL01B.C01.19_NL_50

TCTTATAAAAATCCAGATAATGAATTTGAGCCTATT

>NL01B.C01.19_NL_51

ACATTTAATGGTTGTTTTAGGAGTAACGAGGGCCAACT

>NL01B.C01.19_NL_52

TCCGCACCTACGACCAGAACTACGATTGCCACTATTATG

>NL01B.C01.19_NL_53

TCAGTTAGATGTTTCTAGGTCTTCTTTCACGGTTTTTATAAA

>NL01B.C01.19_NL_54

TACCATTAAATGTGTTTCAAGTCCTCTAAGGAT

>NL01B.C01.19_NL_55

AAGTCCTCTAAGGATTTCTACAAATCTACGCCGGAACGAATAAAAAAA

>NL01B.C01.19_NL_56

TCAGTTAGATGTTTCTCAGACCTGCCCTAAGG

>NL01B.C01.19_NL_57

TACGACATAAACGAAAAATTAGACGACTTCAACA

>NL01B.C01.19_NL_58

TTTCAAGTCCTCTAAGGATTTCTACAAA

>NL01B.C01.19_NL_59

TACAACCATTAAATGTGTTTCAAGTCCTCTAAGGATTTCTACAAA

>NL01B.C01.19_NL_60

TTTGTCCTCTAAGGATTTCTACAAA

>NL01B.C01.19_NL_61

TTTGAGACCCCACACAGTTCTCAGTGAGGATTACGGTTTCTG

>NL01B.C01.19_NL_62

TTAAACCCAGTCCAACGTTTCTCAGGTAATCAGGGGAAGG

>NL01B.C01.19_NL_63

TATTACCAGCACTGTTCTCAAGTAATCAGGGGAAGG

>NL01B.C01.19_NL_64

TCAGTTAGATGTTTCTACGACTCCAAGTCCTCTAAGGATTTCTACAAA

>NL01B.C01.19_NL_65

TTCAAGTCCTCTAAGGATTTCTACAAATCTGGGGAGTTAAATCAT

>NL01B.C01.19_NL_66

TCGTCCCATGAAGCAGGAGTCCAAGATAAAGA

>NL01B.C01.19_NL_67

TCGAGTTCTTCTTTCACGGTTTTTATAAA

>NL01B.C01.19_NL_68

GTAATTTCAAGTCCTCTAAGGATTTCTACAAA

>NL01B.C01.19_NL_69

TCGTCCCATGAAGCAGGAGTCCTCTAAGGATTTCTACAAA

>NL01B.C01.19_NL_70

TTCTGAATATTTCTCGTTTAACTGGGGACATGTGACA

>NL01B.C01.19_NL_71

TTTCAAGTCCTCTAAGGATTTGGCACGT

>NL01B.C01.19_NL_72

CATTTTTCGAGATCGTGACGGGCTGCGGTTGTCCGTTCT

>NL01B.C01.19_NL_73

GAATAATGCAGATAATGGATTTGTCTCAATTGTTGATAA

>NL01B.C01.19_NL_74

CATCGAGTTCTTGGGATGATGGTGGTGGCGGTGGAGGTACG

>NL01B.C01.19_NL_75

TTTTGAGATCGTGACGGGCTGCGGTTGTCCGTTCT

>NL01B.C01.20_NL_0

TACAGTATACTCAAACGAAGGACGCCGTCGATATT

>NL01B.C01.20_NL_1

TAAACGATGCCTATACATTTTTCCAAGATGTCGTTAAT

>NL01B.C01.20_NL_2

TTTTATAAAAACCGTGAAAGAAGACCTAGAATCTCAAAA

>NL01B.C01.20_NL_3

ATTGATGGATAACTGGAGTTCCCATAGCCATGCTTT

>NL01B.C01.20_NL_4

TTGGATCTAATTCAGTTAGCACTGGCTGACCAGAAACGGT

>NL01B.C01.20_NL_5

TACTGCTGTGGCATAGCGTTCAAACTTCAGGTTAGTGTA

>NL01B.C01.20_NL_6

TGATGAATTGAAAGTACATGCTTGATTTCAAATTTCAA

>NL01B.C01.20_NL_7

TGCGATATTAATAGCTAGCATGATAGCTGCACTAAT

>NL01B.C01.20_NL_8

TATGAAGACCGAATAGTACTGCTAATAACATCATAAC

>NL01B.C01.20_NL_9

CTTGTTAGCTTAGGAATTAATAGCTGTAATTGTTTA

>NL01B.C01.20_NL_10

ATATCAAACAAACTAGTATTTGATAGTCGGTGGGAAA

>NL01B.C01.20_NL_11

GGAATACTATGACAATGATCAAGATGATGATTATCG

>NL01B.C01.20_NL_12

ATATTATTATCTCGATCCTTGTAGGCGGGTTCATGT

>NL01B.C01.20_NL_13

TGGATTTAGTAATGAAGGGGAATATGTTTCACGTGAT

>NL01B.C01.20_NL_14

ATAGGCTCGATGCAAATCCCTCACTCACTCATTAC

>NL01B.C01.20_NL_15

TACTTCTATTTTCTATTTATGTTATGGACGCAGTAA

>NL01B.C01.20_NL_16

TGGAATGAAGCTTTGCGTTGTTGAAACGCTATACTG

>NL01B.C01.20_NL_17

CCCTCGGAAATTAACTGGTTTATCTGTTGTGCGATAG

>NL01B.C01.20_NL_18

TCAGTAGTTTCACTTCCTAAATCTTTAGCGAGAGGGTT

>NL01B.C01.20_NL_19

TGATGATACTATATGAAATTAGGGAAAACGAGAAAG

>NL01B.C01.20_NL_20

TTTTGCGGAATAGTTGTAAATCAAGTACGCCGTCACACT

>NL01B.C01.20_NL_21

TAGAGAATTGGAAAAAGAAAAGTTTAATCAAAATGT

>NL01B.C01.20_NL_22

CTCCACACAATTTAGCAGCTTTAAATTCACAGAAAGGG

>NL01B.C01.20_NL_23

TTTTCTTGCTCAAACTGTTGTTCGTTCACATCCTC

>NL01B.C01.20_NL_24

TAATTTTTGATATACTGAATCAACTTGTTTCACATAAGGCTGAGGG

>NL01B.C01.20_NL_25

AGACTTGAAATGATTGCATTAAAATCAGCAATTATTGATA

>NL01B.C01.20_NL_26

TAATTAAAAAGTTCTAAATTTGACCTCATAATGTAGTTC

>NL01B.C01.20_NL_27

TCTTTTGAGAAAAAAGAAAAAGAAGCCTGTTTATACT

>NL01B.C01.20_NL_28

TAACATGTCAGCACTTCATGACGTAAACAGTCTGGCA

>NL01B.C01.20_NL_29

TTTTTCGAGATCGTGACGGGCTGCGGTTGTCCGTTCT

>NL01B.C01.20_NL_30

TTCTATTTCCTTCTTTGAAAACTCTCGCTAGAGAA

>NL01B.C01.20_NL_31

TTGAGTAATCCCGAGAAAGCATTCCGCTCGCCTAGTTC

>NL01B.C01.20_NL_32

TTGAGAACGGACAACCCATGGTAACAAACGACCCGATAACG

>NL01B.C01.20_NL_33

TAAAGGTGCAAAAAAAGTAATTGGATATGAGAAGAGTGA

>NL01B.C01.20_NL_34

CATTGTAACATCCAAATACGGGCCTAAGGAATATG

>NL01B.C01.20_NL_35

CATCATGGCAATGGAACCTTTTGATTAAATCTAGT

>NL01B.C01.20_NL_36

TATTAAGGAGAAAAAGCCAGAAGTGATAGCAGTAGT

>NL01B.C01.20_NL_37

TAACCACCAAAAAGTGTATTTTAGAGATATCCAGA

>NL01B.C01.20_NL_38

TCATCGTCTCTAATAAAAAACATGATTAGTAGTTTG

>NL01B.C01.20_NL_39

AATACATCAAGTATAGCATTTTCTAAATAACTAA

>NL01B.C01.20_NL_40

TTACCTGGGGGAACTGAAATATTTTCAAGTGGAGTTGG

>NL01B.C01.20_NL_41

TACTTCTTGACTACCTCTCTAACGTCAACCCACTGTAT

>NL01B.C01.20_NL_42

TCTTATAAAAATCCAGATAATGAATTTGAGCCTATT

>NL01B.C01.20_NL_43

ACCAGGCAGTCTCGCAGTTCAACAAGGCGGGAGGGC

>NL01B.C01.20_NL_44

TAAATCTAAATAAGGTCTCCCAAAATTACTTAAGGCACGTGAAA

>NL01B.C01.20_NL_45

GAACTTGGATCTAATTCAGTTAGCACTGGCTGACCAGAAACGGT

>NL01B.C01.20_NL_46

TCAGTAGTTCATGACGTAAACAGTCTGGCA

>NL01B.C01.20_NL_47

AATGGATTTAGTAATGAAGGGGAATATGTTTCACGTGAT

>NL01B.C01.20_NL_48

TACTGAGTTTCAAGTCCTCTAAGGATTTCTACAAA

>NL01B.C01.20_NL_49

TGCGATATTAATAGCTGTAATTGTTTA

>NL01B.C01.23_NL_0

TAAACGATGCCTATACATTTTTCCAAGATGTCGTTAAT

>NL01B.C01.23_NL_1

TTTTATAAAAACCGTGAAAGAAGACCTAGAATCTCAAAA

>NL01B.C01.23_NL_2

ATTGATGGATAACTGGAGTTCCCATAGCCATGCTTT

>NL01B.C01.23_NL_3

TTGGATCTAATTCAGTTAGCACTGGCTGACCAGAAACGGT

>NL01B.C01.23_NL_4

ATAAATGAAATAGTACAGAATAAAGAATTTGATGAT

>NL01B.C01.23_NL_5

TGATGAATTGAAAGTACATGCTTGATTTCAAATTTCAA

>NL01B.C01.23_NL_6

TGCGATATTAATAGCTAGCATGATAGCTGCACTAAT

>NL01B.C01.23_NL_7

TATGAAGACCGAATAGTACTGCTAATAACATCATAAC

>NL01B.C01.23_NL_8

GGAATACTATGACAATGATCAAGATGATGATTATCG

>NL01B.C01.23_NL_9

ATATTATTATCTCGATCCTTGTAGGCGGGTTCATGT

>NL01B.C01.23_NL_10

TCATTCTCAATATCAATTTGGGGTCTAATAATACATC

>NL01B.C01.23_NL_11

TGGATTTAGTAATGAAGGGGAATATGTTTCACGTGAT

>NL01B.C01.23_NL_12

TAAACAAAATTGTTATTGCCATTTGCTTGTGTCATT

>NL01B.C01.23_NL_13

ATAGGCTCGATGCAAATCCCTCACTCACTCATTAC

>NL01B.C01.23_NL_14

TACTTCTATTTTCTATTTATGTTATGGACGCAGTAA

>NL01B.C01.23_NL_15

TGGAATGAAGCTTTGCGTTGTTGAAACGCTATACTG

>NL01B.C01.23_NL_16

CCCTCGGAAATTAACTGGTTTATCTGTTGTGCGATAG

>NL01B.C01.23_NL_17

TCCTGAAGAAGTTCAAAGCGTTTTTAACGTTATGAAA

>NL01B.C01.23_NL_18

TCAGTAGTTTCACTTCCTAAATCTTTAGCGAGAGGGTT

>NL01B.C01.23_NL_19

TGATGATACTATATGAAATTAGGGAAAACGAGAAAG

>NL01B.C01.23_NL_20

TTTTGCGGAATAGTTGTAAATCAAGTACGCCGTCACACT

>NL01B.C01.23_NL_21

TAGAGAATTGGAAAAAGAAAAGTTTAATCAAAATGT

>NL01B.C01.23_NL_22

CTCCACACAATTTAGCAGCTTTAAATTCACAGAAAGGG

>NL01B.C01.23_NL_23

TTTTCTTGCTCAAACTGTTGTTCGTTCACATCCTC

>NL01B.C01.23_NL_24

ATTGATTCGACTGCTGAAATTGTAGATTTTGTCATT

>NL01B.C01.23_NL_25

CTGGAGTACCCATGGCCATAGATTCTAGAACTGGTAA

>NL01B.C01.23_NL_26

TATGCTATATAATGTATATAAATTGATGAAATTGGCG

>NL01B.C01.23_NL_27

TAATTAAAAAGTTCTAAATTTGACCTCATAATGTAGTTC

>NL01B.C01.23_NL_28

TCTTTTGAGAAAAAAGAAAAAGAAGCCTGTTTATACT

>NL01B.C01.23_NL_29

TTTTTCGAGATCGTGACGGGCTGCGGTTGTCCGTTCT

>NL01B.C01.23_NL_30

TTGAGTAATCCCGAGAAAGCATTCCGCTCGCCTAGTTC

>NL01B.C01.23_NL_31

TTGAGAACGGACAACCCATGGTAACAAACGACCCGATAACG

>NL01B.C01.23_NL_32

TAAAGGTGCAAAAAAAGTAATTGGATATGAGAAGAGTGA

>NL01B.C01.23_NL_33

CATTGTAACATCCAAATACGGGCCTAAGGAATATG

>NL01B.C01.23_NL_34

CATCATGGCAATGGAACCTTTTGATTAAATCTAGT

>NL01B.C01.23_NL_35

GGATTCAAGGAAATAGTTTATCTAATGCTTACATTTGGA

>NL01B.C01.23_NL_36

TAACCACCAAAAAGTGTATTTTAGAGATATCCAGA

>NL01B.C01.23_NL_37

TCATCGTCTCTAATAAAAAACATGATTAGTAGTTTG

>NL01B.C01.23_NL_38

TTACCTGGGGGAACTGAAATATTTTCAAGTGGAGTTGG

>NL01B.C01.23_NL_39

TCTTATAAAAATCCAGATAATGAATTTGAGCCTATT

>NL01B.C01.23_NL_40

AAGAAGATCCATTTTCAAACATTTTTGGGCCTGTTAGAA

>NL01B.C01.23_NL_41

TTTATGACAGCTCCCTTAGACCTCCTGCTAAAATAC

>NL01B.C01.23_NL_42

GATCTATCCTTCCTGATATTTTCATTCCTTCAATTTCGT

>NL01B.C01.23_NL_43

TAAATCTAAATAAGGTCTCCCAAAATTACTTAAGGCACGTGAAA

>NL01B.C01.23_NL_44

ATAAATGAAATAGTACAAAATGAAGATTTTGATAAT

>NL01B.C01.23_NL_45

TGCGATATTAATAGCTAGCACTGGCTGACCAGAAACGGT

>NL01B.C01.23_NL_46

GATCCAAGTTTCAAGTCCTCTAAGGATTTCTACAAA

>NL01B.C01.23_NL_47

TTCAAGTCCTCTAAGGATTTCTACAAATCAAACTACCCGATAACG

>NL01B.C01.23_NL_48

TTTCAAGTCCTCTAAGGATTTCTACAAATCCCTCACTCACTCATTAC

>NL01B.C01.23_NL_49

CTCTAAGGATTTCTACAAATAGTGTGACGGGCTGCGGTTGTCCGTTCT

>NL01B.C01.23_NL_50

TTTACAACTATTCCGCAAAATTTCAAGTCCTCTAAGGATTTCTACAAA

>NL01B.C01.23_NL_51

TTGGATCTAATTCAGTTAGCATGATAGCTGCACTAAT

>NL01B.C01.23_NL_52

TAAAGGTGCAAAAAAGTAATTGGATATGAGAAGAGTGA

>NL01B.C01.23_NL_53

TGCGATATTAATATCGCATTTCAAGTCCTCTAAGGATTTCTACAAA

>NL01B.C01.23_NL_54

TCTTGGATCTAATTCAGTTAGCACTGGCTGACCAGAAACGGT

>NL01B.C01.23_NL_55

TGCGATATTAATAGCTAGCATGATAGCTGACCAGAAACGGT

>NL01B.C01.24_NL_0

TTTTGAGATTCTAGGTCTTCTTTCACGGTTTTTATAAA

>NL01B.C01.24_NL_1

TTTCATTAGCCCAGTCCAACGTTTCTCAGACCTGCCCTAAGG

>NL01B.C01.24_NL_2

TCTCAAAATTAAGAATGCGTTAAAAGATGCAGTAAAA

>NL01B.C01.24_NL_3

TAAGTCAACGTCTGGCATAACCGTCATTGCGTAACC

>NL01B.C01.24_NL_4

TATTACCAGCACTGTTCTCAGTGAGGATTACGGTTTCTG

>NL01B.C01.24_NL_5

GTCCAGAATTTTTAAAAGCGTTTACAGTCCATTCTGAAT

>NL01B.C01.24_NL_6

TGGGCACTATACTGTCATACCTAGAGGTCATGTATAACG

>NL01B.C01.24_NL_7

TAAAAATTAGAAAGAAAGACAAAGAAAAATAAAAAA

>NL01B.C01.24_NL_8

TGCGAGGAAATTCAGTACAGTGATGGAAGCATGGAAAG

>NL01B.C01.24_NL_9

TGTCTTCCCTCAACCTCTACGGAACGAATAAAAAAA

>NL01B.C01.24_NL_10

TTTCATTAGCCCAGTCGTAATAAAGCCAATAGCAAA

>NL01B.C01.24_NL_11

TAACTATTGAAGAAAACGGAGTACAGGCCAACGGACAC

>NL01B.C01.24_NL_12

CGATGCGATTAGAAGACTATCAGAGCTATTATAAAGTA

>NL01B.C01.24_NL_13

TTACATAGAGGGTCAAAAAATGGTTGAACCCCAATAC

>NL01B.C01.24_NL_14

CTTGAAGTTAAGGCTGAACGAAGTGAATCGCGAAGCGAT

>NL01B.C01.24_NL_15

TTATAATATCTATACCAGAAGCATAGGAAGGAGACA

>NL01B.C01.24_NL_16

TATAATTTCGATGGTCAAGAAGAAACCGGGTTTTGTTTAT

>NL01B.C01.24_NL_17

CATGAACTCATCTTCCTTCAGGGACTTCGCTGACAAGG

>NL01B.C01.24_NL_18

TGCAATTGCACATAAGCAGTAAGGCGATTCAATAATA

>NL01B.C01.24_NL_19

CCTAATTTTGTTGGTGTCCCACCCATAGGGACATGTGACA

>NL01B.C01.24_NL_20

TAGTCACTTGCGGTGATTTCAGACCCATCATTCAG

>NL01B.C01.24_NL_21

CTATTACGGTGGCGAGTCAAGTAACGGAAAACCTGCC

>NL01B.C01.24_NL_22

TACGACATAAACGAAAAATTAGACGATATTCAGCGTC

>NL01B.C01.24_NL_23

CACTAACTTATATCAATATGAATTAGTGGGACAATAT

>NL01B.C01.24_NL_24

GCTTGATCCTCTCTAAGTCTTCAATTTCACCCCTCTC

>NL01B.C01.24_NL_25

TTAGAAAAAATGTCCAATGAACTAAAGCTTTTTATA

>NL01B.C01.24_NL_26

TCTGATACTGTTAAGAGCGATGTAACTGAATCCCCAG

>NL01B.C01.24_NL_27

TACTTCTATTTTCTATTTATGTTATGGACGCAGTAA

>NL01B.C01.24_NL_28

ATTTTTGAGAAGTATAGCCTCTCGTTTTTGAAAATGT

>NL01B.C01.24_NL_29

TCAGTAGTTTCACTTCCTAAATCTTTAGCGAGAGGGTT

>NL01B.C01.24_NL_30

GCGGCAGCTTCCCTGGCTTGCTTCTGTTCCGGCGTAG

>NL01B.C01.24_NL_31

TTTTGCATAATCCAATCAACTTCTCCTTGAGAAGGA

>NL01B.C01.24_NL_32

CACAAATTGCAAAATGGGTCGGTGCAAGGTCATTTA

>NL01B.C01.24_NL_33

CTGGAGTACCCATGGCCATAGATTCTAGAACTGGTAA

>NL01B.C01.24_NL_34

TTATGAATGTCACGCGGTAGCCGTACTGGCGTTCCAAC

>NL01B.C01.24_NL_35

TAAATCGTTCTTCGCCACTGCATCACAGTACGCTAC

>NL01B.C01.24_NL_36

TCGTCCCATGAAGCAGGAGTCGAAGTCCCTCCCGCT

>NL01B.C01.24_NL_37

CTTCTGGTTCAATGCGGTACCTATATTGCGTAGATA

>NL01B.C01.24_NL_38

TACCTCCACCTAAGGTATGGTAAAGACGTTGATGAT

>NL01B.C01.24_NL_39

TACTCAAACTCGGCATCCTTATACTTCTGCTTGTATT

>NL01B.C01.24_NL_40

GTTTCTCTTCTTTTTCATAGCCTACGACATGCTTTG

>NL01B.C01.24_NL_41

ATTTCAGAAAATGACCTAACAGATACCCATTTCGCAA

>NL01B.C01.24_NL_42

GTTTGTTTTCTATATCTTTCACTTCGTTGCCTAAAGCGTCA

>NL01B.C01.24_NL_43

TTTGAGACCCCACACAGTTCTCAAGTAATCAGGGGAAGG

>NL01B.C01.24_NL_44

TTTTTCGAGATCGTGACGGGCTGCGGTTGTCCGTTCT

>NL01B.C01.24_NL_45

TATTGTTGCAGAGTAATAAGTGAAGTTCGTGGTACT

>NL01B.C01.24_NL_46

AATATTCTATTAAAGTATTTTTGATGTTTTTCGTAGAAAG

>NL01B.C01.24_NL_47

TATTTGATGAGGCTCATAACCTCGACGACTTCAACA

>NL01B.C01.24_NL_48

TCACAGCGTCCTACCCAATCGTGGTGTGAAAAGTATT

>NL01B.C01.24_NL_49

TAAATAAGTTAGTGCTTGCACATAAGCAACAATAGGAA

>NL01B.C01.24_NL_50

TCAATGGGAGAGATCGCTTAGAGAATCTGGTATACCAGCT

>NL01B.C01.24_NL_51

TTCTGAATATTTCTCGTTTAACTGGGGAGTTAAATCAT

>NL01B.C01.24_NL_52

TGAAGGGATGTTTACTACATTCTTGGAGTTGTTACAA

>NL01B.C01.24_NL_53

TCGAGTTCTTGGGATGATGGTGGTGGCGGTGGAGGTACG

>NL01B.C01.24_NL_54

TAATGCAGATAATGGATTTGTCTCAATTGTTGATAA

>NL01B.C01.24_NL_55

CCTCTAACTTTTCGTCGTCGTTTTTGAACCTTAAGA

>NL01B.C01.24_NL_56

GGATTCAAGGAAATAGTTTATCTAATGCTTACATTTGGA

>NL01B.C01.24_NL_57

TGAAAAGGCATAAATGGCTGGTATATTAGCGATATT

>NL01B.C01.24_NL_58

CTGATCCTTGTTGGTTATTGATAGTCCAAGATAAAGA

>NL01B.C01.24_NL_59

TATTTCTAATCCGAAATCTTTGCACATATTTATTATACG

>NL01B.C01.24_NL_60

AAAAACTTTTCGGATGCATTTCTAAGCATTTGGCACGT

>NL01B.C01.24_NL_61

ATAATTTAGTAGCTTCACTTAATATTCCTTCAAACGTT

>NL01B.C01.24_NL_62

TTACCTGGGGGAACTGAAATATTTTCAAGTGGAGTTGG

>NL01B.C01.24_NL_63

TAGTTCTGTGAGATATTCAATTTTTGTAATTGTAAAC

>NL01B.C01.24_NL_64

TTTGGTAGACCTTCACCACCCCCACCACCACAAGTCAAA

>NL01B.C01.24_NL_65

TCTTATAAAAATCCAGATAATGAATTTGAGCCTATT

>NL01B.C01.24_NL_66

ACATTTAATGGTTGTTTTAGGAGTAACGAGGGCCAACT

>NL01B.C01.24_NL_67

TCCGCACCTACGACCAGAACTACGATTGCCACTATTATG

>NL01B.C01.24_NL_68

TTTATGACAGCTCCCTTAGACCTCCTGCTAAAATAC

>NL01B.C01.24_NL_69

TATAATTTTTTCCTTCATTTTTTCACCCCGAAAATTA

>NL01B.C01.24_NL_70

CTATTACGGTGGCGATTCAATAATA

>NL01B.C01.24_NL_71

TTCAAGTCCTCTAAGGATTTCTACAAATGTATTGGGGTTCAGCC

>NL01B.C01.24_NL_72

CCGCGGCAGCTTCCCTGGCTTGCTTCTGTTCCGGCGTAG

>NL01B.C01.24_NL_73

TATTTCTAATCCGAAATCTTTGAACCTTAAGA

>NL01B.C01.24_NL_74

TTGGTATCGTAACTGCAATTGCTACAGAGTTCCCATTG

>NL01B.C01.24_NL_75

GGGTACTCCAGGTTTCAAGTCCTCTAAGGATT

>NL01B.C01.24_NL_76

CAAATATTGTTGCAGAGTAATAAGTGAAGTTCGTGGTACT

>NL01B.C01.24_NL_77

GACTTTTGCATAATCCAATCAACTTCTCCTTGAGAAGGA

>NL01B.C01.24_NL_78

TTTCATTAGCCCAGTCCAACGTTTCTCAAGTAATCAGGGGAAGG

>NL01B.C01.25_NL_0

TAAACGATGCCTATACATTTTTCCAAGATGTCGTTAAT

>NL01B.C01.25_NL_1

TTTTATAAAAACCGTGAAAGAAGACCTAGAATCTCAAAA

>NL01B.C01.25_NL_2

ATTGATGGATAACTGGAGTTCCCATAGCCATGCTTT

>NL01B.C01.25_NL_3

TTGGATCTAATTCAGTTAGCACTGGCTGACCAGAAACGGT

>NL01B.C01.25_NL_4

ATAAATGAAATAGTACAGAATAAAGAATTTGATGAT

>NL01B.C01.25_NL_5

TGATGAATTGAAAGTACATGCTTGATTTCAAATTTCAA

>NL01B.C01.25_NL_6

TGCGATATTAATAGCTAGCATGATAGCTGCACTAAT

>NL01B.C01.25_NL_7

TATGAAGACCGAATAGTACTGCTAATAACATCATAAC

>NL01B.C01.25_NL_8

GGAATACTATGACAATGATCAAGATGATGATTATCG

>NL01B.C01.25_NL_9

ATATTATTATCTCGATCCTTGTAGGCGGGTTCATGT

>NL01B.C01.25_NL_10

TCATTCTCAATATCAATTTGGGGTCTAATAATACATC

>NL01B.C01.25_NL_11

TGGATTTAGTAATGAAGGGGAATATGTTTCACGTGAT

>NL01B.C01.25_NL_12

TAAACAAAATTGTTATTGCCATTTGCTTGTGTCATT

>NL01B.C01.25_NL_13

ATAGGCTCGATGCAAATCCCTCACTCACTCATTAC

>NL01B.C01.25_NL_14

TACTTCTATTTTCTATTTATGTTATGGACGCAGTAA

>NL01B.C01.25_NL_15

TTGGATCTAATTCAGTTAGCATGATAGCTGCACTAAT

>NL01B.C01.25_NL_16

TGGAATGAAGCTTTGCGTTGTTGAAACGCTATACTG

>NL01B.C01.25_NL_17

CCCTCGGAAATTAACTGGTTTATCTGTTGTGCGATAG

>NL01B.C01.25_NL_18

TCCTGAAGAAGTTCAAAGCGTTTTTAACGTTATGAAA

>NL01B.C01.25_NL_19

TCAGTAGTTTCACTTCCTAAATCTTTAGCGAGAGGGTT

>NL01B.C01.25_NL_20

TGATGATACTATATGAAATTAGGGAAAACGAGAAAG

>NL01B.C01.25_NL_21

TTTTGCGGAATAGTTGTAAATCAAGTACGCCGTCACACT

>NL01B.C01.25_NL_22

TAGAGAATTGGAAAAAGAAAAGTTTAATCAAAATGT

>NL01B.C01.25_NL_23

CTCCACACAATTTAGCAGCTTTAAATTCACAGAAAGGG

>NL01B.C01.25_NL_24

TTTTCTTGCTCAAACTGTTGTTCGTTCACATCCTC

>NL01B.C01.25_NL_25

ATTGATTCGACTGCTGAAATTGTAGATTTTGTCATT

>NL01B.C01.25_NL_26

CTGGAGTACCCATGGCCATAGATTCTAGAACTGGTAA

>NL01B.C01.25_NL_27

TATGCTATATAATGTATATAAATTGATGAAATTGGCG

>NL01B.C01.25_NL_28

TAATTAAAAAGTTCTAAATTTGACCTCATAATGTAGTTC

>NL01B.C01.25_NL_29

TCTTTTGAGAAAAAAGAAAAAGAAGCCTGTTTATACT

>NL01B.C01.25_NL_30

TTTTTCGAGATCGTGACGGGCTGCGGTTGTCCGTTCT

>NL01B.C01.25_NL_31

TTGAGTAATCCCGAGAAAGCATTCCGCTCGCCTAGTTC

>NL01B.C01.25_NL_32

TTGAGAACGGACAACCCATGGTAACAAACGACCCGATAACG

>NL01B.C01.25_NL_33

TAAAGGTGCAAAAAAAGTAATTGGATATGAGAAGAGTGA

>NL01B.C01.25_NL_34

CATTGTAACATCCAAATACGGGCCTAAGGAATATG

>NL01B.C01.25_NL_35

CATCATGGCAATGGAACCTTTTGATTAAATCTAGT

>NL01B.C01.25_NL_36

GGATTCAAGGAAATAGTTTATCTAATGCTTACATTTGGA

>NL01B.C01.25_NL_37

TAACCACCAAAAAGTGTATTTTAGAGATATCCAGA

>NL01B.C01.25_NL_38

TCATCGTCTCTAATAAAAAACATGATTAGTAGTTTG

>NL01B.C01.25_NL_39

TTACCTGGGGGAACTGAAATATTTTCAAGTGGAGTTGG

>NL01B.C01.25_NL_40

TCTTATAAAAATCCAGATAATGAATTTGAGCCTATT

>NL01B.C01.25_NL_41

AAGAAGATCCATTTTCAAACATTTTTGGGCCTGTTAGAA

>NL01B.C01.25_NL_42

TTTATGACAGCTCCCTTAGACCTCCTGCTAAAATAC

>NL01B.C01.25_NL_43

GATCTATCCTTCCTGATATTTTCATTCCTTCAATTTCGT

>NL01B.C01.25_NL_44

TAAATCTAAATAAGGTCTCCCAAAATTACTTAAGGCACGTGAAA

>NL01B.C01.25_NL_45

TTTCAAGTCCTCTAAGGATTTCTACAAATCTATCGCGAGAGGGTT

>NL01B.C01.25_NL_46

TTCAAGTCCTCTAAGGATTTCTACAAATAAAGC

>NL01B.C01.25_NL_47

TCATCGTCTCTAATAACATCATAAC

>NL01B.C01.25_NL_48

TCCTGAAGAAGTTCAAAGCATTCCGCTCGCCTAGTTC

>NL01B.C01.25_NL_49

TATAATTTCGATGGTCAAGAAGAAACCGGGTTTTGTTTAT

>NL01B.C01.25_NL_50

TTACCTGGTTTATCTGTTGTGCGATAG

>NL01B.C01.25_NL_51

TTTACAACTATTCCGCAAAATTTCAAGTCCTCTAAGGATTTCTACAAA

>NL01B.C01.25_NL_52

TTTAATTATTTCAAGTCCTCTAAGGATTTCTACAAA

>NL01B.C01.25_NL_53

CAAATTTTTCGAGATCGTGACGGGCTGCGGTTGTCCGTTCT

>NL01B.C01.25_NL_54

CCCCCTCGGAAATTAACTGGTTTATCTGTTGTGCGATAG

>NL01B.C01.25_NL_55

TCCGCACCTACGACCAGAACTACGATTGCCACTATTATG

>NL03.C02.01_NL_0

CACAAATTGCAAAATGGGTCGGTGCAAGGTCATTTA

>NL03.C02.01_NL_1

TTCTGAATATTTCTCGTTTAACTGGGGAGTTAAATCAT

>NL03.C02.03_NL_0

TTTTGAGATTCTAGGTCTTCTTTCACGGTTTTTATAAA

>NL03.C02.03_NL_1

TATTACCAGCACTGTTCTCAGTGAGGATTACGGTTTCTG

>NL03.C02.03_NL_2

ATTGATGGATAACTGGAGTTCCCATAGCCATGCTTT

>NL03.C02.03_NL_3

TGGGCACTATACTGTCATACCTAGAGGTCATGTATAACG

>NL03.C02.03_NL_4

TGCGAGGAAATTCAGTACAGTGATGGAAGCATGGAAAG

>NL03.C02.03_NL_5

CATCCGAGAATATTCTGGAAAGTGCCATTTGAAATGC

>NL03.C02.03_NL_6

TGTCTTCCCTCAACCTCTACGGAACGAATAAAAAAA

>NL03.C02.03_NL_7

TTTCATTAGCCCAGTCGTAATAAAGCCAATAGCAAA

>NL03.C02.03_NL_8

CGATGCGATTAGAAGACTATCAGAGCTATTATAAAGTA

>NL03.C02.03_NL_9

CTTGAAGTTAAGGCTGAACGAAGTGAATCGCGAAGCGAT

>NL03.C02.03_NL_10

CATGAACTCATCTTCCTTCAGGGACTTCGCTGACAAGG

>NL03.C02.03_NL_11

CCTAATTTTGTTGGTGTCCCACCCATAGGGACATGTGACA

>NL03.C02.03_NL_12

TAGTCACTTGCGGTGATTTCAGACCCATCATTCAG

>NL03.C02.03_NL_13

CTATTACGGTGGCGAGTCAAGTAACGGAAAACCTGCC

>NL03.C02.03_NL_14

TTAAACCCAGTCCAACGTTTCTCAGACCTGCCCTAAGG

>NL03.C02.03_NL_15

TCTGATACTGTTAAGAGCGATGTAACTGAATCCCCAG

>NL03.C02.03_NL_16

CACTAACTTATATCAATATGAATTAGTGGGACAATAT

>NL03.C02.03_NL_17

TACGACATAAACGAAAAATTAGACGATATTCAGCGTC

>NL03.C02.03_NL_18

GCTTGATCCTCTCTAAGTCTTCAATTTCACCCCTCTC

>NL03.C02.03_NL_19

ATTTTTGAGAAGTATAGCCTCTCGTTTTTGAAAATGT

>NL03.C02.03_NL_20

TCAGTAGTTTCACTTCCTAAATCTTTAGCGAGAGGGTT

>NL03.C02.03_NL_21

GCGGCAGCTTCCCTGGCTTGCTTCTGTTCCGGCGTAG

>NL03.C02.03_NL_22

TACCTCCTCCACTTTGACTTCTCCTACCGTTTTCACGT

>NL03.C02.03_NL_23

TTTTGCATAATCCAATCAACTTCTCCTTGAGAAGGA

>NL03.C02.03_NL_24

ATTGATTCGACTGCTGAAATTGTAGATTTTGTCATT

>NL03.C02.03_NL_25

TTATGAATGTCACGCGGTAGCCGTACTGGCGTTCCAAC

>NL03.C02.03_NL_26

TCAGTTAGATGTTTCTACGACTCCAAGTCCACTTTATT

>NL03.C02.03_NL_27

TAAATCGTTCTTCGCCACTGCATCACAGTACGCTAC

>NL03.C02.03_NL_28

TCGTCCCATGAAGCAGGAGTCGAAGTCCCTCCCGCT

>NL03.C02.03_NL_29

TACCTCCACCTAAGGTATGGTAAAGACGTTGATGAT

>NL03.C02.03_NL_30

CTTCTGGTTCAATGCGGTACCTATATTGCGTAGATA

>NL03.C02.03_NL_31

ATTTCAGAAAATGACCTAACAGATACCCATTTCGCAA

>NL03.C02.03_NL_32

GTTTGTTTTCTATATCTTTCACTTCGTTGCCTAAAGCGTCA

>NL03.C02.03_NL_33

TCTTTTTCACTTCTTCTAATATTCCGGAACTCATGTT

>NL03.C02.03_NL_34

TTTGAGACCCCACACAGTTCTCAAGTAATCAGGGGAAGG

>NL03.C02.03_NL_35

TTTTTCGAGATCGTGACGGGCTGCGGTTGTCCGTTCT

>NL03.C02.03_NL_36

AATATTCTATTAAAGTATTTTTGATGTTTTTCGTAGAAAG

>NL03.C02.03_NL_37

TATTTGATGAGGCTCATAACCTCGACGACTTCAACA

>NL03.C02.03_NL_38

TCGAGTTCTTGGGATGATGGTGGTGGCGGTGGAGGTACG

>NL03.C02.03_NL_39

TAATGCAGATAATGGATTTGTCTCAATTGTTGATAA

>NL03.C02.03_NL_40

TGAAAAGGCATAAATGGCTGGTATATTAGCGATATT

>NL03.C02.03_NL_41

CTGATCCTTGTTGGTTATTGATAGTCCAAGATAAAGA

>NL03.C02.03_NL_42

AAAAACTTTTCGGATGCATTTCTAAGCATTTGGCACGT

>NL03.C02.03_NL_43

TTACCTGGGGGAACTGAAATATTTTCAAGTGGAGTTGG

>NL03.C02.03_NL_44

TCTTATAAAAATCCAGATAATGAATTTGAGCCTATT

>NL03.C02.03_NL_45

ACATTTAATGGTTGTTTTAGGAGTAACGAGGGCCAACT

>NL03.C02.03_NL_46

TCCGCACCTACGACCAGAACTACGATTGCCACTATTATG

>NL03.C02.03_NL_47

TTTATGACAGCTCCCTTAGACCTCCTGCTAAAATAC

>NL03.C02.03_NL_48

TACCTCTAAGGATTTCTACAAA

>NL03.C02.03_NL_49

ACATTTAATGGTTGTTTTCACGT

>NL03.C02.03_NL_50

TTATAATATCTATACCAGAAGCATAGGAAGGAGACA

>NL03.C02.03_NL_51

CATCCGAGAATATTCTGGAAAGTGCCACTATTATG

>NL03.C02.03_NL_52

TACTTCTATTTTCTATTTATGTTATGGACGCAGTAA

>NL03.C02.03_NL_53

TCCACTAACTTATATCAATATGAATTAGTGGGACAATAT

>NL03.C02.03_NL_54

CCCCTAATTTTGTTGGTGTCCCACCCATAGGGACATGTGACA

>NL03.C02.03_NL_55

CTCTAAGGATTTCTACAAATCTACGCCGGAAC

>NL03.C02.03_NL_56

CATGAACTCATCAAATAGTTTCAAGTCCTCTAAGGATTTCTACAA

>NL03.C02.03_NL_57

TTCTGAATATTTCTCGTTTAACTGGGGAGTTAAATCAT

>NL03.C02.03_NL_58

CCTCTAACTTTTCGTCGTCGTTTTTGAACCTTAAGA

>NL03.C02.03_NL_59

TCGTCCTCTAAGGATTTCTACAAA

>NL03.C02.03_NL_60

TTATAATATCTATACCGGAAGCATAGGAAGGAGACA

>NL03.C02.03_NL_61

TTCAAGTCCTCTAAGGATTTCTACAAATCGTAC

>NL03.C02.03_NL_62

GATTTCAAGTCCTCTAAGGATTTCTACAAA

>NL03.C02.03_NL_63

CAACCGATGCGATTAGAAGACTATCAGAGCTATTATAAAGTA

>NL03.C02.05_NL_0

AAAAGTGCATAAGTTGAATATTCTTGATTTGCTAAAT

>NL03.C02.05_NL_1

TGTCCCTAACGGAATGACAGTTTATTGGCTAAGAG

>NL03.C02.05_NL_2

TCTAGTAACATTTGATGATGGTACGTCTGAGGAAGCGA

>NL03.C02.05_NL_3

ATTTTTCAGAAGAGGAGAAAAAGAAAATTGCAGAACTCT

>NL03.C02.05_NL_4

ACATTTCTTGCAGCAGCACCGACTAGTTTGGTACCGT

>NL03.C02.05_NL_5

TCTACAGAAAGTGGAGGCATTTTTCAGGACGTAAG

>NL03.C02.05_NL_6

ATTTGTCTTTTTGTAATTGGAGATTTGAATAGTGGA

>NL03.C02.05_NL_7

TATATTATGACATTTTTATATTTTTTATAATGAACT

>NL03.C02.05_NL_8

TAAACTATAATTGAATGAGGCAAATATTTGGATAAT

>NL03.C02.05_NL_9

TTGAGACCTGATGAAATAAAAGCAAAATTTAGAGATAAATT

>NL03.C02.05_NL_10

TCCTAAACCTCCAGAGTTAGGTGGTCCGCCATGGTA

>NL03.C02.05_NL_11

AAATATCAACTATGTCTTTATCATCAAATTCTTTAT

>NL03.C02.05_NL_12

TAATCTATATCTTTTTCTTCCAAACTTTCCAAAATAC

>NL03.C02.05_NL_13

TTTCACAAAACAAGAATAAATAAACTCAAAGTAAA

>NL03.C02.05_NL_14

ACTCCATTTATCGCTCCAGTTACTTTAGGAAAATTT

>NL03.C02.05_NL_15

TCCAGATTTAGCTAAATTCATAGTTTCATTTATACCA

>NL03.C02.06_NL_0

TCTTATGACGTTTTAAAATTCATCTTCTACGTTTTTC

>NL03.C02.06_NL_1

TCAGTTTCTTCTCCATTTGCAACACTATCAAGAAATTGA

>NL03.C02.06_NL_2

CTGATCCTTGTTGGTTATTGATAGTCCAAGATAAAGA

>NL03.C02.06_NL_3

TCTACAGAAAGTGGAGGCATTTTTCAGGACGTAAG

>NL03.C02.06_NL_4

TGCGATATTTATAGCTAGCATGATAGCTGCACTAAT

>NL03.C02.06_NL_5

GCGGCAGCTTCCCTGGCTTGCTTCTGTTCCGGCGTAG

>NL03.C02.06_NL_6

TCATTCTCAATATCAATTTGGGGTCTAATAATACATC

>NL03.C02.06_NL_7

TGAAGGGATGTTTACTACATTCTTGGAGTTGTTACAA

>NL03.C02.06_NL_8

GTCCAGAATTTTTAAAAGCGTTTACAGTCCATTCTGAAT

>NL03.C02.06_NL_9

TTGAGACCTGATGAAATAAAAGCAAAATTTAGAGATAAATT

>NL03.C02.06_NL_10

TACAATACAAGGTTTGATAAAACTGGAGCTGAAAATAC

>NL03.C02.06_NL_11

CAAAAAGTATTTTCAGAATTTTCAGTTCCTAGTGAT

>NL03.C02.06_NL_12

TAACTATTGAAGAAAACGGAGTACAGGCCAACGGACAC

>NL03.C02.06_NL_13

TGGAATGAAGCTTTGCGTTGTTGAAACGCTATACTG

>NL03.C02.06_NL_14

CTCTTTGATCTCATCATATTCAATTCATAAAATAT

>NL03.C02.06_NL_15

TAAGTCAACGTCTGGCATAACCGTCATTGCGTAACC

>NL03.C02.06_NL_16

AAGAAAAAGTGATATACATAATATAATGTATATGC

>NL03.C02.06_NL_17

TCATCTCTTATAGCGACTGGTCTAAAAAGCGGATTTCT

>NL03.C02.06_NL_18

TCCTGAAGAAGTTCAAAGCGTTTTTAACGTTATGAAA

>NL03.C02.06_NL_19

TAAAGGTGCAAAAAAAGTAATTTCAAGTCCTCTAAGGATTTCTACAAA

>NL03.C02.06_NL_20

TCCAGATTTAGCTAAATTCATAGTTTCATTTATACCA

>NL03.C02.06_NL_21

ATTTTTCAGAAGAGGAGAAAAAGAAAATTGCAGAACTCT

>NL03.C02.07_NL_0

TCTTATAAAAATCCAGATAATGAATTTGAGCCTATT

>NL03.C02.07_NL_1

TGGAATGAAGCTTTGCGTTGTTGAAACGCTATACTG

>NL03.C02.07_NL_2

TGGATTTAGTAATGAAGGGGAATATGTTTCACGTGAT

>NL03.C02.07_NL_3

TTTTATAAAAACCGTGAAAGAAGACCTAGAATCTCAAAA

>NL03.C02.07_NL_4

TTTTGCATAATCCAATCAACTTCTCCTTGAGAAGGA

>NL03.C02.07_NL_5

TACGACATAAACGAAAAATTAGACGATATTCAGCGTC

>NL03.C02.07_NL_6

CTATTACGGTGGCGAGTCAAGTAACGGAAAACCTGCC

>NL03.C02.07_NL_7

ACATTTAATGGTTGTTTTAGGAGTAACGAGGGCCAACT

>NL03.C02.07_NL_8

ATTGATTCGACTGCTGAAATTGTAGAGTTTGTCATT

>NL03.C02.07_NL_9

TTGAGTAATCCCGAGAAAGCATTCCGCTCGCCTAGTTC

>NL03.C02.07_NL_10

GTTTGTTTTCTATATCTTTCACTTCGTTGCCTAAAGCGTCA

>NL03.C02.07_NL_11

TTACCTGGGGGAACTGAAATATTTTCAAGTGGAGTTGG

>NL03.C02.07_NL_12

CTGGAGTACCCATGGCCATAGATTCTAGAACTGGTAA

>NL03.C02.07_NL_13

TTCTGAATATTTCTCGTTTAACTGGGGAGTTAAATCAT

>NL03.C02.07_NL_14

TTTACGCTCTCGATAGACTTTATAGCATTAGCGTTCCA

>NL03.C02.07_NL_15

CTGATCCTTGTTGGTTATTGATAGTCCAAGATAAAGA

>NL03.C02.07_NL_16

AAAAACTTTTCGGATGCATTTCTAAGCATTTGGCACGT

>NL03.C02.07_NL_17

CGATGCGATTAGAAGACTATCAGAGCTATTATAAAGTA

>NL03.C02.07_NL_18

TTTATGACAGCTCCCTTAGACCTCCTGCTAAAATAC

>NL03.C02.07_NL_19

TTTTGCGGAATAGTTGTAAATCAAGTACGCCGTCACACT

>NL03.C02.07_NL_20

TTTCATTAGCCCAGTCGTAATAAAGCCAATAGCAAA

>NL03.C02.07_NL_21

TCGTCCCATGAAGCAGGAGTCGAAGTCCCTCCCGCT

>NL03.C02.07_NL_22

CTGCAATTGCACATAAGCAGTAAGGCGATTCAATAATA

>NL03.C02.07_NL_23

CTTCTTTCTTCTCTATCTTTTAATTCACTTGCTATC

>NL03.C02.07_NL_24

TCTTTTTCACTTCTTCTAATATTCCGGAACTCATGTT

>NL03.C02.07_NL_25

TTTTTCGAGATCGTGACGGGCTGCGGTTGTCCGTTCT

>NL03.C02.07_NL_26

TCAAGTCCTCTAAGGATTTCTACAAATGAGGATG

>NL03.C02.07_NL_27

TGATGATACTATATGAAATTAGGGAAAACGAGAAAG

>NL03.C02.07_NL_28

TTACAATTTCTCGATGTCTTTCAAAATGCGGTGGGTTT

>NL03.C02.07_NL_29

TGAAAAGGCATAAATGGCTGGTATATTAGCGATATT

>NL03.C02.07_NL_30

TTGGTATCGTAACTGCAATTGCTACAGAGTTCCCATTG

>NL03.C02.07_NL_31

CTTCTGGTTCAATGCGGTACCTATATTGCGTAGATA

>NL03.C02.07_NL_32

CCCTCGGAAATTAACTGGTTTATCTGTTGTGCGATAG

>NL03.C02.07_NL_33

TTGAGAACGGACAACCCATGGTAACAAACGACCCGATAACG

>NL03.C02.07_NL_34

TACTTCTATTTTCTATTTATGTTATGGACGCAGTAA

>NL03.C02.07_NL_35

TCTGATACTGTTAAGAGCGATGTAACTGAATCCCCAG

>NL03.C02.07_NL_36

TAAAGGTGCAAAAAAAGTAATTGGATATGAGAAGAGTGA

>NL03.C02.07_NL_37

TTGAAGTCTCTCGGCTTCCGCTATCCATTCTTCAACGC

>NL03.C02.07_NL_38

TTTTCTTGCTCAAACTGTTGTTCGTTCACATCCTC

>NL03.C02.08_NL_0

TCATTCTCAATATCAATTTGGGGTCTAATAATACATC

>NL03.C02.08_NL_1

TGATGATACTATATGAAATTAGGGAAAACGAGAAAG

>NL03.C02.10_NL_0

CTATTACGGTGGCGAGTCAAGTAACGGAAAACCTGCC

>NL03.C02.10_NL_1

CTTGAAGTTAAGGCTGAACGAAGTGAATCGCGAAGCGAT

>NL03.C02.10_NL_2

TTGTCAAATTTTTCTCAGTTTACAGATAAAATTTCAA

>NL03.C02.10_NL_3

CGATGCGATTAGAAGACTATCAGAGCTATTATAAAGTA

>NL03.C02.10_NL_4

ATTTTTGAGAAGTATAGCCTCTCGTTTTTGAAAATGT

>NL03.C02.10_NL_5

TTATGAATGTCACGCGGTAGCCGTACTGGCGTTCCAAC

>NL03.C02.10_NL_6

TGGGCACTATACTGTCATACCTAGAGGTCATGTATAACG

>NL03.C02.10_NL_7

GCTTGATCCTCTCTAAGTCTTCAATTTCACCCCTCTC

>NL03.C02.10_NL_8

CCTCTAACTTTTCGTCGTCGTTTTTGAACCTTAAGA

>NL03.C02.10_NL_9

TCCGCACCTACGACCAGAACTACGATTGCCACTATTATG

>NL03.C02.10_NL_10

TCAGTAGTTTCACTTCCTAAATCTTTAGCGAGAGGGTT

>NL03.C02.10_NL_11

GTTTCTCTTCTTTTTCATAGCCTACGACATGCTTTG

>NL03.C02.10_NL_12

TTTGAGACCCCACACAGTTCTCAAGTAATCAGGGGAAGG

>NL03.C02.10_NL_13

TTACCTGGGGGAACTGAAATATTTTCAAGTGGAGTTGG

>NL03.C02.10_NL_14

CTGATCCTTGTTGGTTATTGATAGTCCAAGATAAAGA

>NL03.C02.10_NL_15

AAAAACTTTTCGGATGCATTTCTAAGCATTTGGCACGT

>NL03.C02.10_NL_16

ATAATTTAGTAGCTTCACTTAATATTCCTTCAAACGTT

>NL03.C02.10_NL_17

TCTCAAAATTAAGAATGCGTTAAAAGATGCAGTAAAA

>NL03.C02.10_NL_18

TCCGCACCTACGATCAGAACTACGATTGCCACTATTATG

>NL03.C02.10_NL_19

TCGTCCCATGAAGCAGGAGTCGAAGTCCCTCCCGCT

>NL03.C02.10_NL_20

TACCTCCTCCACTTTGACTTCTCCTACCGTTTTCACGT

>NL03.C02.10_NL_21

TCTTTTTCACTTCTTCTAATATTCCGGAACTCATGTT

>NL03.C02.10_NL_22

CATCCGAGAATATTCTGGAAAGTGCCATTTGAAATGC

>NL03.C02.10_NL_23

ATTGATGGATAACTGGAGTTCCCATAGCCATGCTTT

>NL03.C02.10_NL_24

CCTAATTTTGTTGGTGTCCCACCCATAGGGACATGTGACA

>NL03.C02.10_NL_25

CTTCTGGTTCAATGCGGTACCTATATTGCGTAGATA

>NL03.C02.10_NL_26

CACAAATTGCAAAATGGGTCGGTGCAAGGTCATTTA

>NL03.C02.10_NL_27

TCGAGTTCTTGGGATGATGGTGGTGGCGGTGGAGGTACG

>NL03.C02.10_NL_28

TGTCTTCCCTCAACCTCTACGGAACGAATAAAAAAA

>NL13.C01.01_NL_0

TTTTGAGATTCTAGGTCTTCTTTCACGGTTTTTATAAA

>NL13.C01.01_NL_1

TCTCAAAATTAAGAATGCGTTAAAAGATGCAGTAAAA

>NL13.C01.01_NL_2

TATTACCAGCACTGTTCTCAGTGAGGATTACGGTTTCTG

>NL13.C01.01_NL_3

ATTGATGGATAACTGGAGTTCCCATAGCCATGCTTT

>NL13.C01.01_NL_4

TGGGCACTATACTGTCATACCTAGAGGTCATGTATAACG

>NL13.C01.01_NL_5

TGCGAGGAAATTCAGTACAGTGATGGAAGCATGGAAAG

>NL13.C01.01_NL_6

CATCCGAGAATATTCTGGAAAGTGCCATTTGAAATGC

>NL13.C01.01_NL_7

TGTCTTCCCTCAACCTCTACGGAACGAATAAAAAAA

>NL13.C01.01_NL_8

TTTCATTAGCCCAGTCGTAATAAAGCCAATAGCAAA

>NL13.C01.01_NL_9

CGATGCGATTAGAAGACTATCAGAGCTATTATAAAGTA

>NL13.C01.01_NL_10

TTATAATATCTATACCAGAAGCATAGGAAGGAGACA

>NL13.C01.01_NL_11

CTTGAAGTTAAGGCTGAACGAAGTGAATCGCGAAGCGAT

>NL13.C01.01_NL_12

CATGAACTCATCTTCCTTCAGGGACTTCGCTGACAAGG

>NL13.C01.01_NL_13

CCTAATTTTGTTGGTGTCCCACCCATAGGGACATGTGACA

>NL13.C01.01_NL_14

TAGTCACTTGCGGTGATTTCAGACCCATCATTCAG

>NL13.C01.01_NL_15

CTATTACGGTGGCGAGTCAAGTAACGGAAAACCTGCC

>NL13.C01.01_NL_16

TTAAACCCAGTCCAACGTTTCTCAGACCTGCCCTAAGG

>NL13.C01.01_NL_17

TCTGATACTGTTAAGAGCGATGTAACTGAATCCCCAG

>NL13.C01.01_NL_18

CACTAACTTATATCAATATGAATTAGTGGGACAATAT

>NL13.C01.01_NL_19

TACGACATAAACGAAAAATTAGACGATATTCAGCGTC

>NL13.C01.01_NL_20

GCTTGATCCTCTCTAAGTCTTCAATTTCACCCCTCTC

>NL13.C01.01_NL_21

TACTTCTATTTTCTATTTATGTTATGGACGCAGTAA

>NL13.C01.01_NL_22

ATTTTTGAGAAGTATAGCCTCTCGTTTTTGAAAATGT

>NL13.C01.01_NL_23

TCAGTAGTTTCACTTCCTAAATCTTTAGCGAGAGGGTT

>NL13.C01.01_NL_24

GCGGCAGCTTCCCTGGCTTGCTTCTGTTCCGGCGTAG

>NL13.C01.01_NL_25

TACCTCCTCCACTTTGACTTCTCCTACCGTTTTCACGT

>NL13.C01.01_NL_26

TTTTGCATAATCCAATCAACTTCTCCTTGAGAAGGA

>NL13.C01.01_NL_27

CACAAATTGCAAAATGGGTCGGTGCAAGGTCATTTA

>NL13.C01.01_NL_28

ATTGATTCGACTGCTGAAATTGTAGATTTTGTCATT

>NL13.C01.01_NL_29

TCAGTTAGATGTTTCTACGACTCCAAGTCCACTTTATT

>NL13.C01.01_NL_30

TTATGAATGTCACGCGGTAGCCGTACTGGCGTTCCAAC

>NL13.C01.01_NL_31

TAAATCGTTCTTCGCCACTGCATCACAGTACGCTAC

>NL13.C01.01_NL_32

TCGTCCCATGAAGCAGGAGTCGAAGTCCCTCCCGCT

>NL13.C01.01_NL_33

TACCTCCACCTAAGGTATGGTAAAGACGTTGATGAT

>NL13.C01.01_NL_34

CTTCTGGTTCAATGCGGTACCTATATTGCGTAGATA

>NL13.C01.01_NL_35

GTTTCTCTTCTTTTTCATAGCCTACGACATGCTTTG

>NL13.C01.01_NL_36

ATTTCAGAAAATGACCTAACAGATACCCATTTCGCAA

>NL13.C01.01_NL_37

GTTTGTTTTCTATATCTTTCACTTCGTTGCCTAAAGCGTCA

>NL13.C01.01_NL_38

TCTTTTTCACTTCTTCTAATATTCCGGAACTCATGTT

>NL13.C01.01_NL_39

TTTGAGACCCCACACAGTTCTCAAGTAATCAGGGGAAGG

>NL13.C01.01_NL_40

TTTTTCGAGATCGTGACGGGCTGCGGTTGTCCGTTCT

>NL13.C01.01_NL_41

AATATTCTATTAAAGTATTTTTGATGTTTTTCGTAGAAAG

>NL13.C01.01_NL_42

TATTTGATGAGGCTCATAACCTCGACGACTTCAACA

>NL13.C01.01_NL_43

TTCTGAATATTTCTCGTTTAACTGGGGAGTTAAATCAT

>NL13.C01.01_NL_44

TCGAGTTCTTGGGATGATGGTGGTGGCGGTGGAGGTACG

>NL13.C01.01_NL_45

TAATGCAGATAATGGATTTGTCTCAATTGTTGATAA

>NL13.C01.01_NL_46

CCTCTAACTTTTCGTCGTCGTTTTTGAACCTTAAGA

>NL13.C01.01_NL_47

TGAAAAGGCATAAATGGCTGGTATATTAGCGATATT

>NL13.C01.01_NL_48

CTGATCCTTGTTGGTTATTGATAGTCCAAGATAAAGA

>NL13.C01.01_NL_49

AAAAACTTTTCGGATGCATTTCTAAGCATTTGGCACGT

>NL13.C01.01_NL_50

ATAATTTAGTAGCTTCACTTAATATTCCTTCAAACGTT

>NL13.C01.01_NL_51

TTACCTGGGGGAACTGAAATATTTTCAAGTGGAGTTGG

>NL13.C01.01_NL_52

TCTTATAAAAATCCAGATAATGAATTTGAGCCTATT

>NL13.C01.01_NL_53

ACATTTAATGGTTGTTTTAGGAGTAACGAGGGCCAACT

>NL13.C01.01_NL_54

TCCGCACCTACGACCAGAACTACGATTGCCACTATTATG

>NL13.C01.01_NL_55

TTTATGACAGCTCCCTTAGACCTCCTGCTAAAATAC

>NL13.C01.01_NL_56

CAACACATTTAATGGTTGTTTTAGGAGTAACGAGGGCCAACT

>NL13.C01.01_NL_57

TCTTATAAAAATCCAGATAATGAATTAGTGGGACAATAT

>NL13.C01.01_NL_58

TAACTCTTTTTCACTTCTTCTAATATTCCGGAACTCATGTT

>NL13.C01.01_NL_59

ACATTTAATGTTGTTTTAGGAGTAACGAGGGCCAACT

>NL13.C01.01_NL_60

ATTGATGGAAGCATGGAAAG

>NL13.C01.01_NL_61

GAACGCGGCAGCTTCCCTGGCTTGCTTCTGTTCCGGCGTAG

>NL13.C01.01_NL_62

AAGTGACTAGTTTCAAGTCCTCTAAGGATTT

>NL13.C01.02_NL_0

TAAACTATAATTGAATGAGGCAAATATTTGGATAAT

>NL13.C01.02_NL_1

TACAATACAAGGTTTGATAAAACTGGAGCTGAAAATAC

>NL13.C01.02_NL_2

CAAAAAGTATTTTCAGAATTTTCAGTTCCTAGTGAT

>NL13.C01.02_NL_3

ACATTTCTTGCAGCAGCACCGACTAGTTTGGTACCGT

>NL13.C01.02_NL_4

TCTTATGACGTTTTAAAATTCATCTTCTACGTTTTTC

>NL13.C01.02_NL_5

TCAGTTTCTTCTCCATTTGCAACACTATCAAGAAATTGA

>NL13.C01.02_NL_6

TCTAGTAACATTTGATGATGGTACGTCTGAGGAAGCGA

>NL13.C01.02_NL_7

TTTGTGCAAAAGCATTAGCGACATTTTTGAAAAATCCA

>NL13.C01.02_NL_8

TATATTATGACATTTTTATATTTTTTATAATGAACT

>NL13.C01.02_NL_9

ATTTTTACGTTTATATCTTACGCTATTTTTATCAT

>NL13.C01.02_NL_10

TCTACAGAAAGTGGAGGCATTTTTCAGGACGTAAG

>NL13.C01.02_NL_11

AAATATCAACTATGTCTTTATCATCAAATTCTTTAT

>NL13.C01.02_NL_12

ATTTGTCTTTTTGTAATTGGAGATTTGAATAGTGGA

>NL13.C01.02_NL_13

TATAGGAACTGAAAACGGCGGTGTTTTTTTATATGA

>NL13.C01.02_NL_14

TTTCACAAAACAAGAATAAATAAACTCAAAGTAAA

>NL13.C01.02_NL_15

ATTTTTCAGAAGAGGAGAAAAAGAAAATTGCAGAACTCT

>NL13.C01.02_NL_16

TTTATATCTTGAATATCAAATTTATGAATTCTCCAT

>NL13.C01.02_NL_17

TCCAGATTTAGCTAAATTCATAGTTTCATTTATACCA

>NL13.C01.02_NL_18

TTCGTCGTAGTTTTTTTGCAGTTCCATCTTCAACTTCATC

>NL13.C01.02_NL_19

AAGAAAAAGTGATATACATAATATAATGTATATGC

>NL13.C01.02_NL_20

TGTCCCTAACGGAATGACAGTTTATTGGCTAAGAG

>NL13.C01.02_NL_21

CTCTGACCCATTGCCAAAACATAGTTCCCGTAAATGCT

>NL13.C01.02_NL_22

CATTTTAATACTTTAAATCACGGTCACTTAAGATCAT

>NL13.C01.02_NL_23

TTAATAATTTACAATATGATTCTGAATCAAAAACAGT

>NL13.C01.02_NL_24

TTGAGACCTGATGAAATAAAAGCAAAATTTAGAGATAAATT

>NL13.C01.02_NL_25

AAGATTTTGAAGTTCCTGAAAATGTACATTTTGTTTCA

>NL13.C01.02_NL_26

TTTCATTCAAATTTTCATAATTGTTTATATTCTCTATTT

>NL13.C01.02_NL_27

TAATAATAGATATTTGTCGAATAAAAAGCGTTTAGC

>NL13.C01.02_NL_28

AGTGGAATTTTTGGACAAACTTCTGGTCTTGCATTTAT

>NL13.C01.02_NL_29

AAAAGTGCATAAGTTGAATATTCTTGATTTGCTAAAT

>NL13.C01.02_NL_30

TAATCTATATCTTTTTCTTCCAAACTTTCCAAAATAC

>NL13.C01.02_NL_31

TCATCTCTTATAGCGACTGGTCTAAAAAGCGGATTTCT

>NL13.C01.02_NL_32

TCCTAAACCTCCAGAGTTAGGTGGTCCGCCATGGTA

>NL13.C01.02_NL_33

ACTCCATTTATCGCTCCAGTTACTTTAGGAAAATTT

>NL13.C01.02_NL_34

CAATTTAGAAGAAAAGCGATATTCCTTAGAGACTTAG

>NL13.C01.02_NL_35

ACAGTAGTGATTTGGACTGGTTGGCTGATGAAGT

>NL13.C01.02_NL_36

CTCTTTGATCTCATCATATTCAATTCATAAAATAT

>NL13.C01.02_NL_37

CAAATACAATACAAGGTTTGATAAAACTGGAGCTGAAAATAC

>NL13.C01.03_NL_0

ATTGATGGATAACTGGAGTTCCCATAGCCATGCTTT

>NL13.C01.03_NL_1

TAAAAATTAGAAAGAAAGACAAAGAAAAATAAAAAA

>NL13.C01.03_NL_2

TCTATGTAAAGAACCTAAGGATGCCGGACGAAATAT

>NL13.C01.03_NL_3

TTGAAGTCTCTCGGCTTCCGCTATCCATTCTTCAACGC

>NL13.C01.03_NL_4

TTACAATTTCTCGATGTCTTTCAAAATGCGGTGGGTTT

>NL13.C01.03_NL_5

TGGATTTAGTAATGAAGGGGAATATGTTTCACGTGAT

>NL13.C01.03_NL_6

TTGACCAATTTAATGCTTACTCAAAATTTAAAGAAATATAA

>NL13.C01.03_NL_7

TGGAATGAAGCTTTGCGTTGTTGAAACGCTATACTG

>NL13.C01.03_NL_8

TTTTGCGGAATAGTTGTAAATCAAGTACGCCGTCACACT

>NL13.C01.03_NL_9

TTTTCTTGCTCAAACTGTTGTTCGTTCACATCCTC

>NL13.C01.03_NL_10

ATTGATTCGACTGCTGAAATTGTAGATTTTGTCATT

>NL13.C01.03_NL_11

TATTCCAGAATTTAATCCACAAAATGTGACAGAATTAGA

>NL13.C01.03_NL_12

TCTGGTCCCAATAGGGCAATATATCAAGGCTTTGGATT

>NL13.C01.03_NL_13

TCTTTTTCACTTCTTCTAATATTCCGGAACTCATGTT

>NL13.C01.03_NL_14

TTTACGCTCTCGATAGACTTTATAGCATTAGCGTTCCA

>NL13.C01.03_NL_15

TCTTTTGAGAAAAAAGAAAAAGAAGCCTGTTTATACT

>NL13.C01.03_NL_16

TTGGTATCGTAACTGCAATTGCTACAGAGTTCCCATTG

>NL13.C01.03_NL_17

TTTTTCGAGATCGTGACGGGCTGCGGTTGTCCGTTCT

>NL13.C01.03_NL_18

TTGAGAACGGACAACCCATGGTAACAAACGACCCGATAACG

>NL13.C01.03_NL_19

TAAAGGTGCAAAAAAAGTAATTGGATATGAGAAGAGTGA

>NL13.C01.03_NL_20

TGGAATTTCTATACCTCCAACCATTGTAGATGCTGGAT

>NL13.C01.03_NL_21

AAAAAATATTAGAAATTATTCAGTCTGGTCGATATAA

>NL13.C01.03_NL_22

TCATCGTCTCTAATAAAAAACATGATTAGTAGTTTG

>NL13.C01.03_NL_23

CTTCTTTCTTCTCTATCTTTTAATTCACTTGCTATC

>NL13.C01.03_NL_24

TTACCTGGGGGAACTGAAATATTTTCAAGTGGAGTTGG

>NL13.C01.03_NL_25

TCTTATAAAAATCCAGATAATGAATTTGAGCCTATT

>NL13.C01.03_NL_26

ATTTAGATATTTTCATGTTGTATTTATTTGCTAATT

>NL13.C01.03_NL_27

TTTATGACAGCTCCCTTAGACCTCCTGCTAAAATAC

>NL13.C01.03_NL_28

TGGGCACTATACTGTCATACCTAGAGGTCATGTATAACG

>NL13.C01.03_NL_29

TGCGAGGAAATTCAGTACAGTGATGGAAGCATGGAAAG

>NL13.C01.03_NL_30

TGTCTTCCCTCAACCTCTACGGAACGAATAAAAAAA

>NL13.C01.03_NL_31

TTTCATTAGCCCAGTCGTAATAAAGCCAATAGCAAA

>NL13.C01.03_NL_32

TGCAATTGCACATAAGCAGTAAGGCGATTCAATAATA

>NL13.C01.03_NL_33

GGAATACTATGACAATGATCAAGATGATGATTATCG

>NL13.C01.03_NL_34

CTATTACGGTGGCGAGTCAAGTAACGGAAAACCTGCC

>NL13.C01.03_NL_35

CACTAACTTATATCAATATGAATTAGTGGGACAATAT

>NL13.C01.03_NL_36

TACGACATAAACGAAAAATTAGACGATATTCAGCGTC

>NL13.C01.03_NL_37

GCTTGATCCTCTCTAAGTCTTCAATTTCACCCCTCTC

>NL13.C01.03_NL_38

ATTTTTGAGAAGTATAGCCTCTCGTTTTTGAAAATGT

>NL13.C01.03_NL_39

TTATGAATGTCACGCGGTAGCCGTACTGGCGTTCCAAC

>NL13.C01.03_NL_40

TCGTCCCATGAAGCAGGAGTCGAAGTCCCTCCCGCT

>NL13.C01.03_NL_41

TACCTCCACCTAAGGTATGGTAAAGACGTTGATGAT

>NL13.C01.03_NL_42

TTTGAGACCCCACACAGTTCTCAAGTAATCAGGGGAAGG

>NL13.C01.03_NL_43

TATTGTTGCAGAGTAATAAGTGAAGTTCGTGGTACT

>NL13.C01.03_NL_44

TAAATAAGTTAGTGCTTGCACATAAGCAACAATAGGAA

>NL13.C01.03_NL_45

TCGAGTTCTTGGGATGATGGTGGTGGCGGTGGAGGTACG

>NL13.C01.03_NL_46

CTGGAGTACCCCTGGCCATAGATTCTAGAACTGGTAA

>NL13.C01.03_NL_47

CCTCTAACTTTTCGTCGTCGTTTTTGAACCTTAAGA

>NL13.C01.03_NL_48

TATTTCTAATCCGAAATCTTTGCACATATTTATTATACG

>NL13.C01.03_NL_49

CTTGAAGTTAAGGCTGAACGAAGTGAATCGCGAAGCGATT

>NL13.C01.04_NL_0

TATTGTTGCAGAGTAATAAGTGAAGTTCGTGGTACT

>NL13.C01.04_NL_1

CTTCTGGTTCAATGCGGTACCTATATTGCGTAGATA

>NL13.C01.04_NL_2

CCTCTAACTTTTCGTCGTCGTTTTTGAACCTTAAGA

>NL13.C01.04_NL_3

TCGTCCCATGAAGCAGGAGTCGAAGTCCCTCCCGCT

>NL13.C01.04_NL_4

CTGATCCTTGTTGGTTATTGATAGTCCAAGATAAAGA

>NL13.C01.04_NL_5

CCCTCGGAAATTAACTGGTTTATCTGTTGTGCGATAG

>NL13.C01.04_NL_6

CTCCACACAATTTAGCAGCTTTAAATTCACAGAAAGGG

>NL13.C01.04_NL_7

TACTTCTATTTTCTATTTATGTTATGGACGCAGTAA

>NL13.C01.04_NL_8

AAAAACTTTTCGGATGCATTTCTAAGCATTTGGCACGT
